# Divergent and Enantioselective Synthesis of Three Types of Chiral Polycyclic N‑Heterocycles via Copper-Catalyzed Dearomative Cyclization

**DOI:** 10.1021/acscentsci.5c00248

**Published:** 2025-05-02

**Authors:** Wen-Feng Luo, Li-Gao Liu, Yan-Xin Zheng, Miao Sun, Xin Lu, Bo Zhou, Long-Wu Ye, Long Li

**Affiliations:** † State Key Laboratory of Physical Chemistry of Solid Surfaces, Key Laboratory of Chemical Biology of Fujian Province, and College of Chemistry and Chemical Engineering, 12466 Xiamen University, Xiamen 361005, China; ‡ College of Chemistry & Materials Engineering, 26495 Wenzhou University, Wenzhou 325035, China; § China State Key Laboratory of Organometallic Chemistry, ShanghaiInstitute of Organic Chemistry, Chinese Academy of Sciences, Shanghai 200032, China

## Abstract

Significant advancements have been made in the catalytic asymmetric dearomatization of indoles for constructing valuable chiral polycyclic N-heterocycles. However, the asymmetric dearomative cyclopropanation of indoles continues to pose a formidable challenge. Furthermore, the diverse transformations of indoline-fused cyclopropanes via strain release remain largely unexplored, potentially unveiling new chemistry. Here, we disclose a Cu-catalyzed asymmetric dearomative cyclopropanation of indole-diynes and subsequent [3 + 2] cycloaddition with oxygen, facilitating the divergent and atom-economical synthesis of enantioenriched cyclopropane- and 1,2-dioxolane-fused indolines with moderate to excellent yields and generally outstanding diastereo- and enantioselectivities with broad substrate scope. Importantly, this protocol not only represents the first asymmetric dearomative cyclopropanation of indoles utilizing alkynes as carbene precursors but also constitutes the first catalytic asymmetric construction of chiral 1,2-dioxolanes with high stereoselectivity. Interestingly, Brønsted acid-promoted ring-opening and rearrangement of cyclopropane-fused indolines display distinctive chemoselectivity to afford enantioenriched cyclohepta­[*b*]­indoles in good to excellent efficiency and enantiocontrol. In addition, both potential reaction pathways and the origins of chiral control within this Cu-catalyzed asymmetric tandem sequence are robustly supported by control experiments and theoretical calculations.

## Graphical abstract

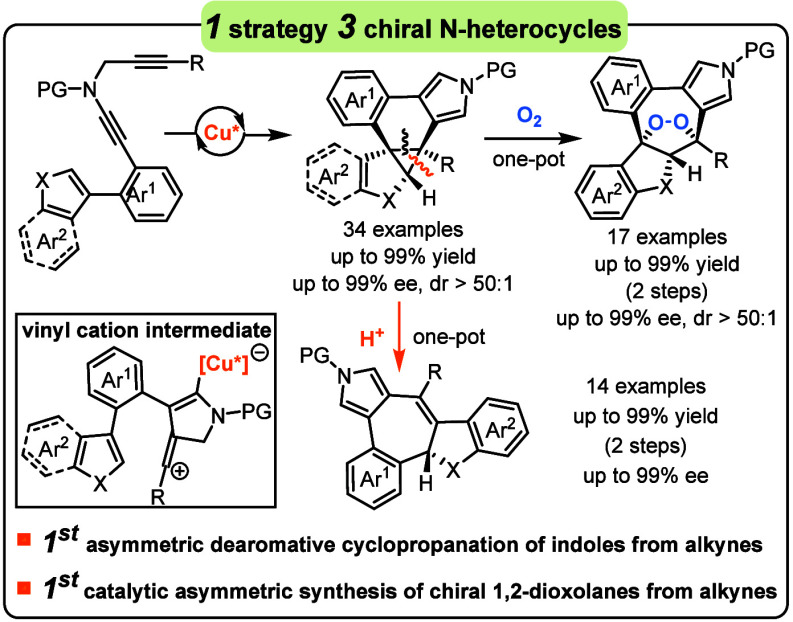

## Introduction

In the past decade, catalytic asymmetric dearomatization (CADA, coined by You and co-workers) reactions^1–7^ have undergone remarkable growth and demonstrated significant potential for the rapid assembly of valuable and complex heterocycles from a variety of (hetero)­aromatic compounds, particularly the CADA reactions of indoles.^8–22^ Among them, asymmetric dearomative cyclopropanation of indoles has garnered much attention due to its highly efficient construction of the cyclopropane-fused indolines, which serve as core frameworks found in many natural products (Figure 1).^23–26^ However, successful examples of such CADA reactions are still very scarce.^27–30^ For example, Zhou and co-workers disclosed the first intramolecular enantioselective cyclopropanation of indoles, leading to chiral polycyclic N-heterocycles bearing cyclopropane moieties in good to high yields with excellent enantioselectivities by using copper or iron catalysts.^28^ Recently, Bi and co-workers developed an elegant intermolecular protocol by employing trifluoromethyl *N*-triftosylhydrazones as carbene precursors to achieve rhodium-catalyzed asymmetric dearomative cyclopropanation of indoles, yielding diverse cyclopropane-fused indolines in high enantioselectivities (Scheme 1A, left top).^30^ Despite these encouraging results, most have been constrained by reliance on high-energy, potentially explosive diazo compounds.^27–29^ Trifluoromethyl *N*-triftosylhydrazones as relatively safe carbene precursors, originated by Bi’s group,^31–34^ may not be a priority option in atom-economical synthesis. Furthermore, competitive ring opening of the cyclopropane probably resulted in certain reports and remains a formidable challenge.^30,34^ Consequently, integrating safety with atom economy in catalytic asymmetric dearomative cyclopropanation of indoles with high efficacy and enantiopurity, especially based on the alkynes as precursors (Scheme 1A, right top), is still underexplored and extremely challenging but highly appealing. More importantly, complex and uncontrollable chemoselectivity in C–C bond cleavage via strain release probably hinders further explorations of versatile transformations of cyclopropanes but potentially unveils new chemistry (Scheme 1A, bottom).

**Figure 1:**
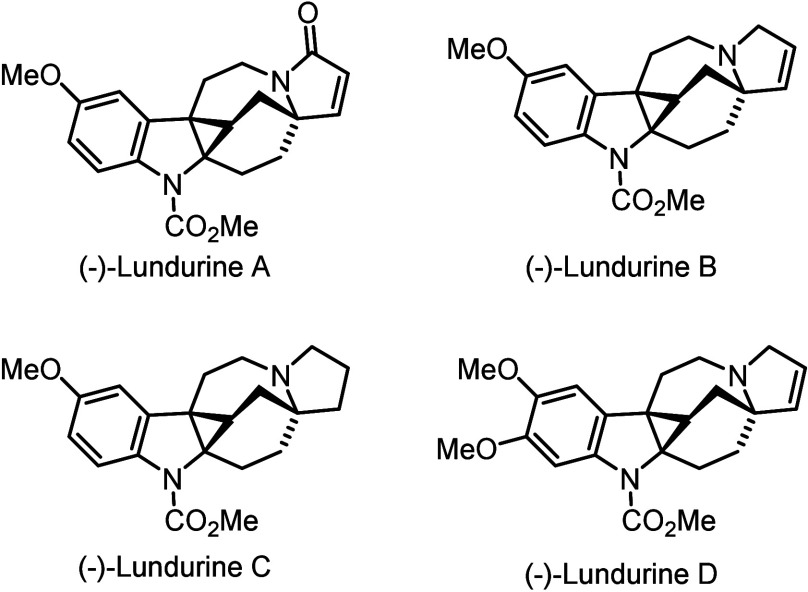
Representative natural products featuring cyclopropane-fused indoline frameworks.

**Scheme 1:**
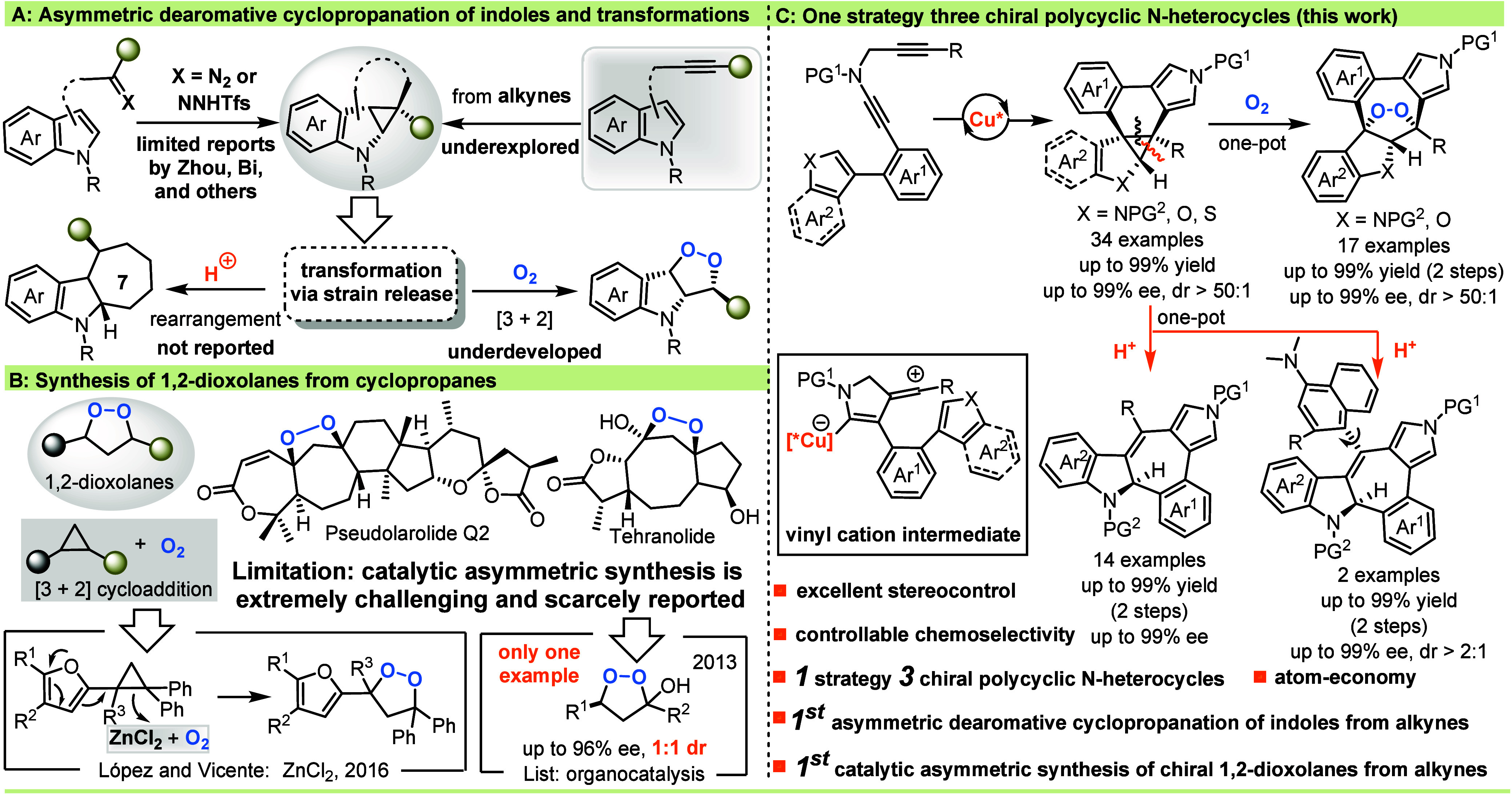
Asymmetric Dearomative Cyclopropanation of Indoles and Transformations

1,2-Dioxolanes are distinctive 5-membered heterocycles characterized by a unique peroxy bond and are prevalent in numerous natural products (Scheme 1B).^35,36^ Because of their unique antimalarial properties, numerous methods have been developed for the preparation of these oxygenated heterocycles, specially the [3 + 2] cycloaddition of cyclopropanes with oxygen.^37,38^ Despite remarkable advancements in racemic synthesis, in sharp contrast, the catalytic asymmetric version remains an extreme challenge, with scarce reports. In 2013, List and co-workers achieved a pioneering progress in constructing chiral 1,2-dioxolanes with high enantiopurity via organocatalytic asymmetric hydroperoxidation of enones but with poor diastereo- and chemoselectivity.^39^ Thus, exploring the efficient preparation of enantioenriched 1,2-dioxolanes with high stereocontrol, particularly via new approaches such as catalytic asymmetric [3 + 2] cycloaddition of cyclopropanes derived from alkyne cyclization, is still underdeveloped but highly desirable.

Vinyl cations have been regarded as versatile intermediates for rapid access to various useful scaffolds in the past decade, on account of their unique carbene-like reactivity.^40,41^ However, the development of a catalytic asymmetric version involving vinyl cation intermediates remains elusive but great in demand.^42^ In the past several years, our group focused on the asymmetric transformations of vinyl cations, which were generated by copper-catalyzed diyne cyclization, for efficient synthesis of versatile valuable chiral N-heterocycles via a remote control of enantioselectivity,^43–47^ including intramolecular C–H functionalization,^43^ one-carbon ring expansion,^44^ the Büchner reaction,^45^ [1,2]-Stevens-type rearrangement,^46^ and atroposelective cyclization.^47^ Inspired by our recent studies on the chiral N-heterocycle synthesis from ynamides^48–51^ and the above results, we envisaged that intramolecular indole moieties might capture the vinyl cations generated from Cu-catalyzed cyclization of propargyl ynamides^52,53^ and finally deliver the enantioenriched cyclopropane-fused indolines via a remote control of enantioselectivity (Scheme 1C). Notably, realizing such an asymmetric cascade cyclization in an orderly manner is highly challenging due to the following considerations: (1) how to avoid the background reaction of indole attacking ynamide^54,55^ and the C­(sp^2^)–H functionalization of indole via vinyl cation;^43^ (2) how to control the chemo-, diastereo-, and enantioselectivity; and (3) how to provide other related chiral N-heterocycles via further transformations of cyclopropanes with controllable chemoselectivity. Herein, we present the realization of a practical and atom-economical synthesis of enantioenriched cyclopropane-fused indolines in generally good to excellent yields and stereocontrols with a wide substrate scope via chiral Cu-catalyzed tandem cyclization/dearomative cyclopropanation of indolyl diynes. Of note, this work marks the first instance of dearomative cyclopropanation of indoles utilizing alkynes as carbene precursors. Importantly, this protocol is successfully extended to the construction of valuable enantioenriched 1,2-dioxolanes via further [3 + 2] cycloaddition of cyclopropanes with oxygen, which constitutes the first highly stereocontrolled construction of chiral 1,2-dioxolanes from alkynes. Interestingly, unexpected chiral cyclohepta­[*b*]­indoles are obtained in mostly excellent yields and excellent enantioselectivities with distinctive chemoselectivities through Brønsted acid-promoted ring-opening and rearrangement of cyclopropanes. Additionally, control experiments and theoretical calculations reveal both potential reaction pathways and the origins of chiral control within this Cu-catalyzed asymmetric tandem sequence.

## Results and Discussion

### Optimization of Reaction Conditions

At the outset, indolyl diyne **A1** was selected as the model substrate to validate our design, as summarized in Table 1. First, a typical Tang’s side-armed bisoxazoline (SaBOX)^56^ ligand **L1** (12 mol %) was employed as the chiral ligand for conducting asymmetric dearomative cyclopropanation in the presence of 10 mol % of Cu­(MeCN)_4_PF_6_ as catalyst and 12 mol % of NaBAr^F^_4_ as additive under N_2_ atmosphere. Encouragingly, the expected chiral cyclopropane-fused indoline **B1** was formed in 78% yield with 81% ee (Table 1, entry 1), thereby strongly supporting the feasibility of our presupposition. Further screening of a variety of other chiral SaBOX ligands **L2**–**L3** and phosphine ligands **L4**–**L6** yielded significantly enhanced results (Table 1, entries 2–6), particularly with **L3** providing **B1** in 95% yield and 96% ee (Table 1, entry 3). Then, other conventional copper catalysts, such as Cu­(MeCN)_4_BF_4_, Cu­(MeCN)_4_OTf, and CuI, were also investigated but could not elevate either efficiency or enantioselectivity (Table 1, entries 7–9). Subsequently, solvent examinations, including DCE, toluene, CHCl_3_, Et_2_O, and PhCl led to negative outcomes (Table 1, entries 10–14). Gratifyingly, lowering the reaction temperature further enhanced both the yield and enantiopurity of **B1** to 99% yield and 98% ee at 0 °C (Table 1, entry 15). Notably, neither background indole addition to ynamide^54,55^ nor C­(sp^2^)–H insertion into vinyl cation^43^ was observed in this reaction.

**Table 1:**
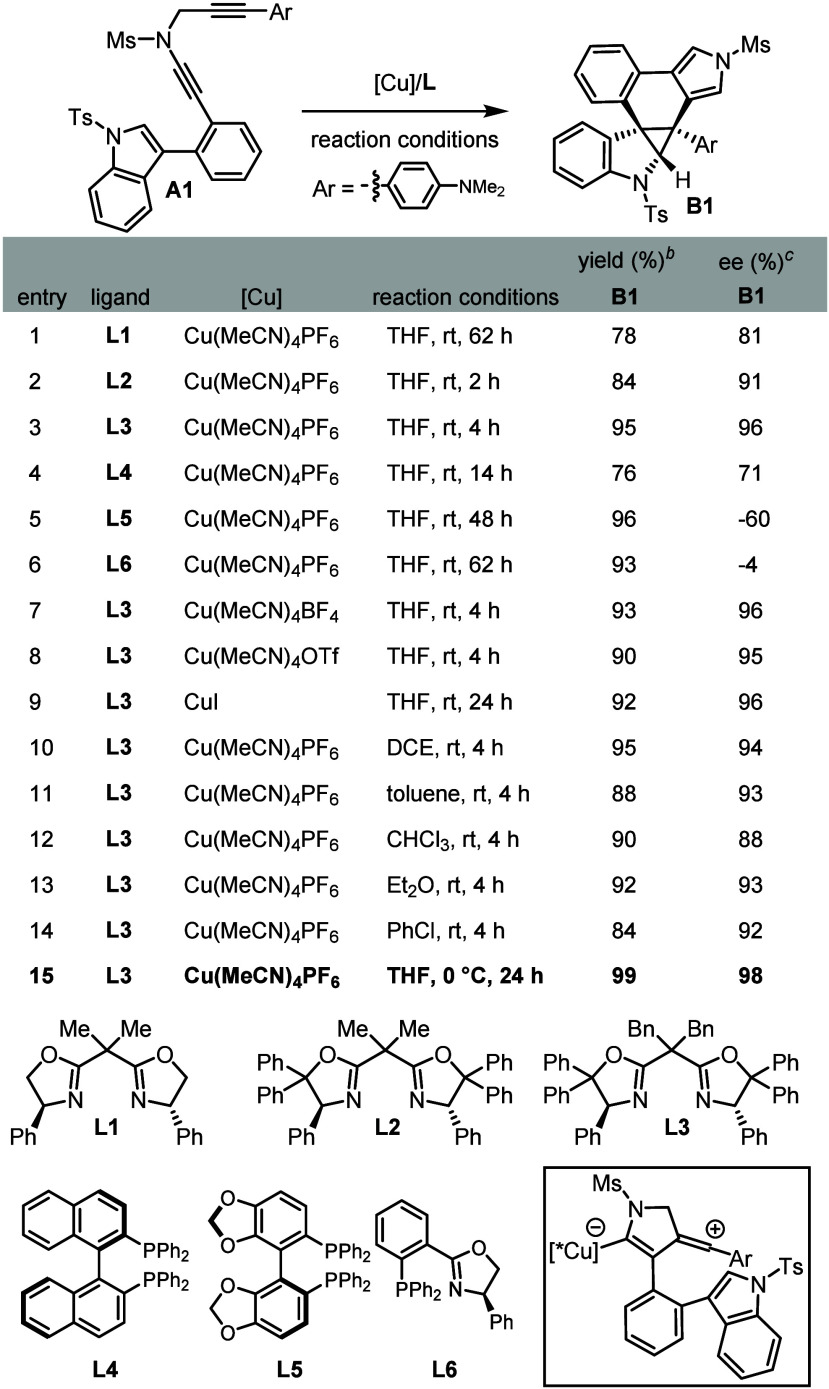
Optimization of Reaction Conditions^a^

a Reaction conditions: **A1** (0.05 mmol), [Cu] (0.005 mmol), **L** (0.006 mmol), NaBAr^F^_4_ (0.006 mmol), solvent (1 mL), in Schlenk tubes.

b Measured by ^1^H NMR using 1,3,5-trimethoxybenzene as internal standard.

c Determined by HPLC analysis. Ms = methanesulfonyl, Ts = *p*-toluenesulfonyl, NaBAr^F^_4_ = sodium tetrakis­[3,5-bis­(trifluoromethyl)­phenyl]­borate.

### Substrate Scope

With the optimal reaction conditions established, the evaluation of a wide range of indole-diynes **A** was conducted to assess the tolerance of functional groups in this asymmetric dearomative cyclopropanation for constructing various enantioenriched cyclopropane-fused indolines **B**, as shown in Table 2. Initially, different protecting groups such as Ts-, SO_2_Ph-, Mbs-, and Bs- on the N atom of propargyl ynamides exhibited good compatibility and were transformed into products **B2**–**B5** in almost quantitative yields and 91–93% ee’s. Ynamides containing a variety of electron-rich aryl rings were converted into the expected chiral products **B6**–**B12** in 76–99% yields with 80–98% ee’s. It is notable that low efficiency (<30%) was observed in case of substrates with the non-electron-rich groups, which is similar to the previous protocols.^43–47^ Furthermore, substituents including 1-F, 2-Cl, 2-OMe, 3-Cl, and 3-Me on the Ar^1^ ring were compatible with this dearomative process to furnish **B13**–**B17** in excellent yields and enantiocontrols. Subsequently, ynamides **A18**–**A25** substituted with a series of functional groups, whatever their electronic properties, on the different positions of the indole ring underwent this Cu-catalyzed tandem reaction smoothly, forging **B18**–**B25** in almost quantitative yields coupled with outstanding enantioselectivities. Afterward, different protecting groups on the N atom of indole covering SO_2_Ph-, Ms-, and Boc- were investigated to deliver the corresponding dearomative products **B26**–**B28** in 94–98% yields and 97–99% ee’s. In particular, this Cu-catalyzed dearomative cyclopropanation was successfully extended to other heteroarenes for the divergent synthesis of a variety of chiral heteroarene-fused cyclopropanes **B29**–**B34** with moderate to excellent yields and mostly excellent enantiopurities. Thus, this protocol not only represents the first catalytic asymmetric dearomative cyclopropanation of heteroarenes by using alkynes as precursors but also features the first enantioselective dearomative cyclopropanation of thiophenes and benzothiophenes.^57^ Notably, this reaction displayed remarkable diastereoselectivity (>50:1 dr) in all examples, and importantly, neither background indole addition to ynamide^54,55^ nor C­(sp^2^)–H insertion into vinyl cation^43^ was detected in all cases. The absolute configurations of products **B1** and **B32** were confirmed by X-ray crystallographic analysis.

**Table 2:**
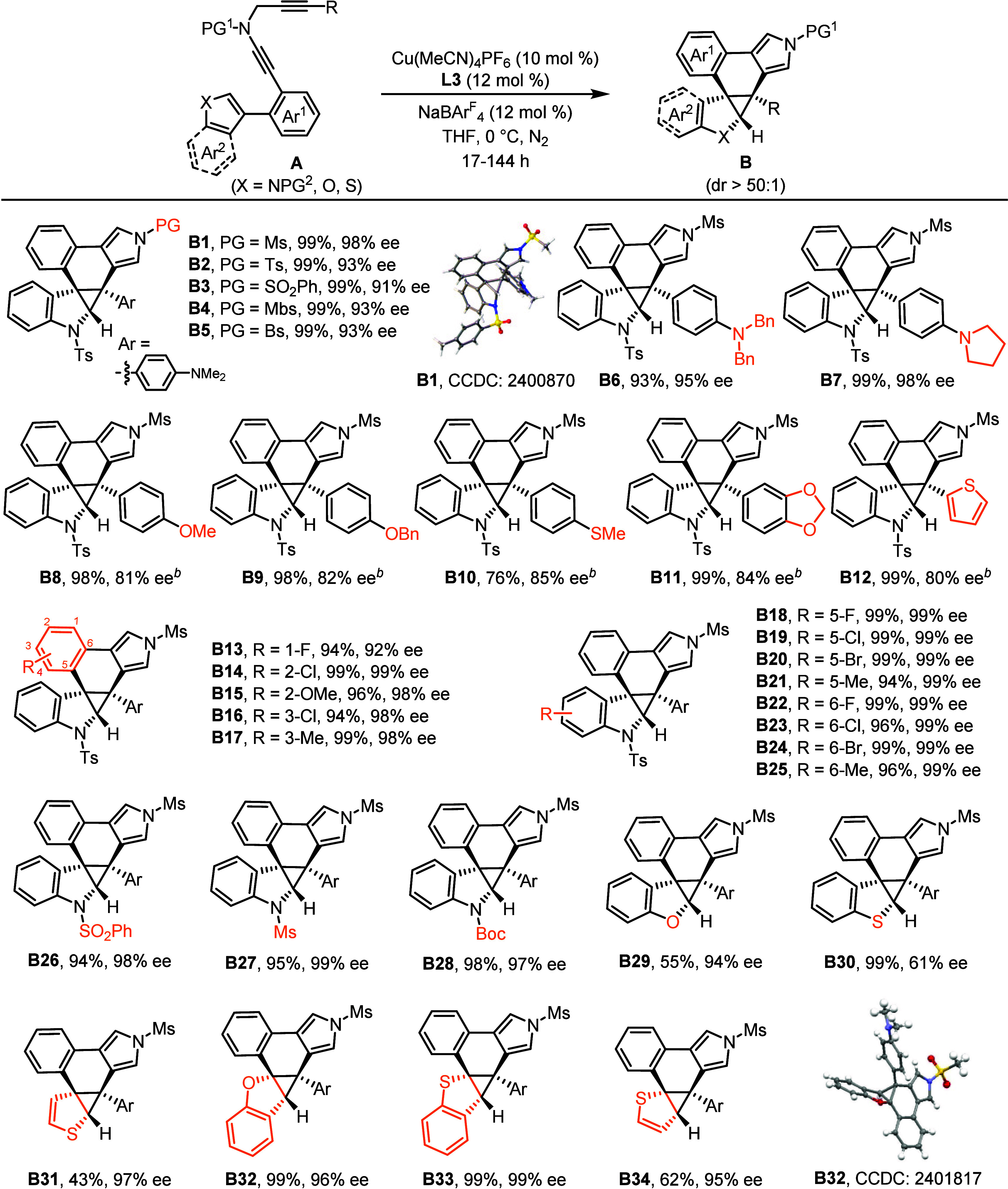
Substrate Scope for the Synthesis of Chiral Cyclopropane-Fused Polycyclic N-Heterocycles **B** ^a^

a Reaction conditions: **A** (0.15 mmol), Cu­(MeCN)_4_PF_6_ (0.015 mmol), **L3** (0.018 mmol), NaBAr^F^_4_ (0.018 mmol) in THF (3 mL), 0 °C, in Schlenk tubes; yields are those for the isolated products; ee’s are determined by HPLC analysis.

b At 30 °C. Mbs = 4-methoxybenzenesulfonyl, Bs = 4-bromobenzenesulfonyl.

Then, we turned our attention to deeply exploring the versatile transformation of indoline-cyclopropanes generated from dearomative cyclopropanations of indoles, which was scarcely reported but potentially unveiled new chemistry. Given their significance in antimalarial applications and the challenge associated with catalytic asymmetric synthesis, we took the initiative to achieve the catalytic asymmetric synthesis of 1,2-dioxolanes through the strain release of chiral cyclopropanes. Encouraged by López and Vicente’s findings (Scheme 1B, left bottom),^38^ oxygen was directly introduced to perform [3 + 2] cycloaddition with **B1** by replacing nitrogen into oxygen atmosphere for the one-pot synthesis of chiral 1,2-dioxolane **C1** by employing Cu catalyst (see the Supporting Information). Pleasingly, the expected chiral 1,2-dioxolane **C1** was obtained in moderate yield with retained enantiocontrol and excellent diastereoselectivity. Further investigation of various solvents in the [3 + 2] cycloaddition process revealed that DCE provided the best efficiency to deliver **C1** in 86% yield and 98% ee with >50:1 dr. The subsequent evaluation of substrate scope demonstrated that this Cu-catalyzed tandem sequence involving cyclization/dearomative cyclopropanation/[3 + 2] cycloaddition of indole-diynes **A** exhibited good compatibility with functionalized groups, resulting in diverse enantioenriched 1,2-dioxolane-fused indolines **C** in good to excellent yields and diastereo- and enantioselectivities under a one-pot operation, as summarized in Table 3. Initially, Ts- and Bs- were suitable protecting groups on the N atom of ynamide to produce chiral 1,2-dioxolanes **C2**–**C3** in 77–80% yields with 93% ee. Next, substituents including 2-Cl, 2-OMe, 3-Cl, and 3-Me on the aryl ring (Ar^1^) were compatible with this one-pot reaction, providing **C4**–**C7** in 66–99% yields and 97–99% ee’s. A range of chiral 1,2-dioxolanes **C8**–**C13** containing various functional groups such as Cl, Br, and Me on the different sites of indole were successfully obtained with good efficacy and excellent stereocontrol. Following, ynamides bearing SO_2_Ph- and Ms- as protecting groups on the N atom of indole could be smoothly converted into the desired **C14**–**C15** in good yields with outstanding enantioselectivities. Interestingly, chiral 1,2-dioxolane **C16** was gained in 56% yield with 95% ee by the replacement of indole with benzofuran. When R was changed into the 4-*N*,*N*-(Bn)_2_-C_6_H_4_ group, the reaction also underwent a smooth tandem sequence, affording **C17** in 87% yield with 95% ee. Significantly, this reaction was also directly conducted in DCE with an O_2_ atmosphere to produce chiral **C1** in 65% yield and 97% ee without a complex one-pot operation. Of note, attempts to synthesize other heterocycle-fused 1,2-dioxolanes **C18**–**C21** failed probably because these substrates were not reactive enough. In addition, the absolute configuration of product **C1** was confirmed by an X-ray crystallographic analysis. Again, excellent diastereoselectivities (>50:1 dr) were achieved in all cases. Thus, this work constitutes the first catalytic asymmetric synthesis of chiral 1,2-dioxolanes with high diastereoselectivity and enantiopurity.

**Table 3:**
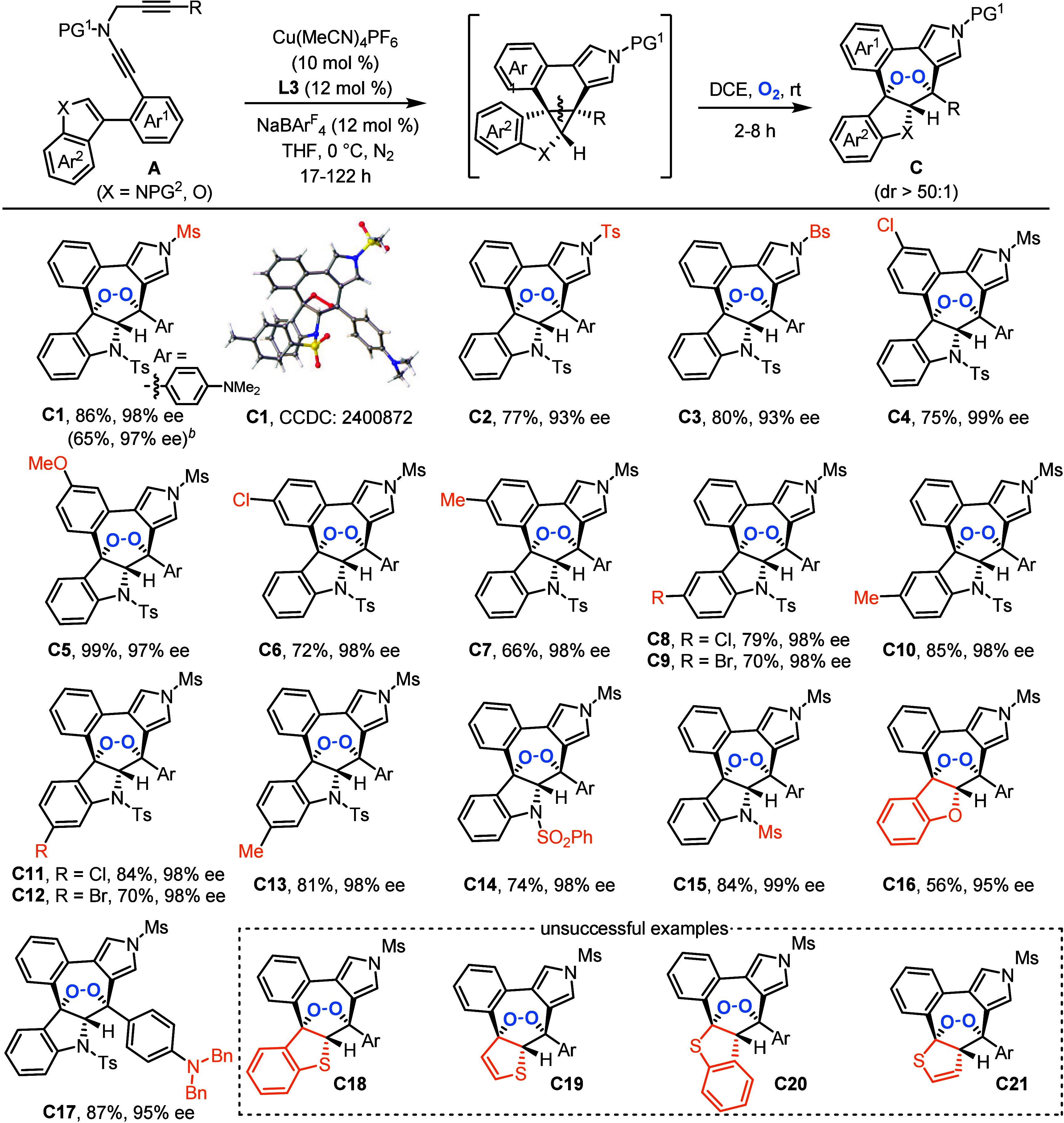
Substrate Scope for the Synthesis of Chiral 1,2-Dioxolane-Fused Indolines **C** ^a^

a Reaction conditions: **A** (0.15 mmol), Cu­(MeCN)_4_PF_6_ (0.015 mmol), **L3** (0.018 mmol), NaBAr^F^_4_ (0.018 mmol) in THF (3 mL), 0 °C, in Schlenk tubes; after the first step, add DCE (1 mL) and change the reaction atmosphere from nitrogen to oxygen, rt; yields are those for the isolated products; ee’s are determined by HPLC analysis.

b Reaction was conducted in DCE with O_2_.

Interestingly, enantioenriched cyclopropane **B1** was successfully converted into the unexpected chiral 7-membered ring **D1** with excellent yield and invariable enantioselectivity in DCE as solvent under acidic conditions (see the Supporting Information). Especially, this ring expansion promoted by acid could be followed in the Cu-catalyzed asymmetric dearomative cyclopropanation seamlessly without isolating enantioenriched cyclopropanes, thus allowing the efficient synthesis of chiral cyclohepta­[*b*]­indoles **D** in almost quantitative yields and remarkable enantiocontrols with distinctive chemoselectivities under a one-pot operation (Table 4). For example, various protecting groups on the different N atoms of ynamides were compatible with the one-pot process to furnish enantioenriched **D1**–**D5** in 77–99% yield with 93–99% ee’s. Chiral cyclohepta­[*b*]­indoles **D6**–**D14** featuring a diverse array of substituents, whatever electron-donating or -withdrawing groups, were obtained in 94–99% yields and 98–99% ee’s. Moreover, ynamides bearing steric groups also performed well in this tandem sequence, yielding axially chiral cyclohepta­[*b*]­indoles **D15**–**D16** in excellent efficiency and enantiocontrol with up to 2.4:1 dr. Notably, our attempts to synthesize **D17**–**D20** also failed in this acid-promoted ring expansion reaction. The absolute configuration of product **D9** was further confirmed by X-ray crystallographic analysis. Importantly, this acid-promoted ring expansion not only exhibited significantly different chemoselectivity in C–C bond cleavage compared with previous quinoline synthesis^30,34^ but also provided an efficient and practical way for the construction of valuable enantioenriched cyclohepta­[*b*]­indoles, which could be found in a variety of natural products and pharmaceuticals.^58–61^

**Table 4:**
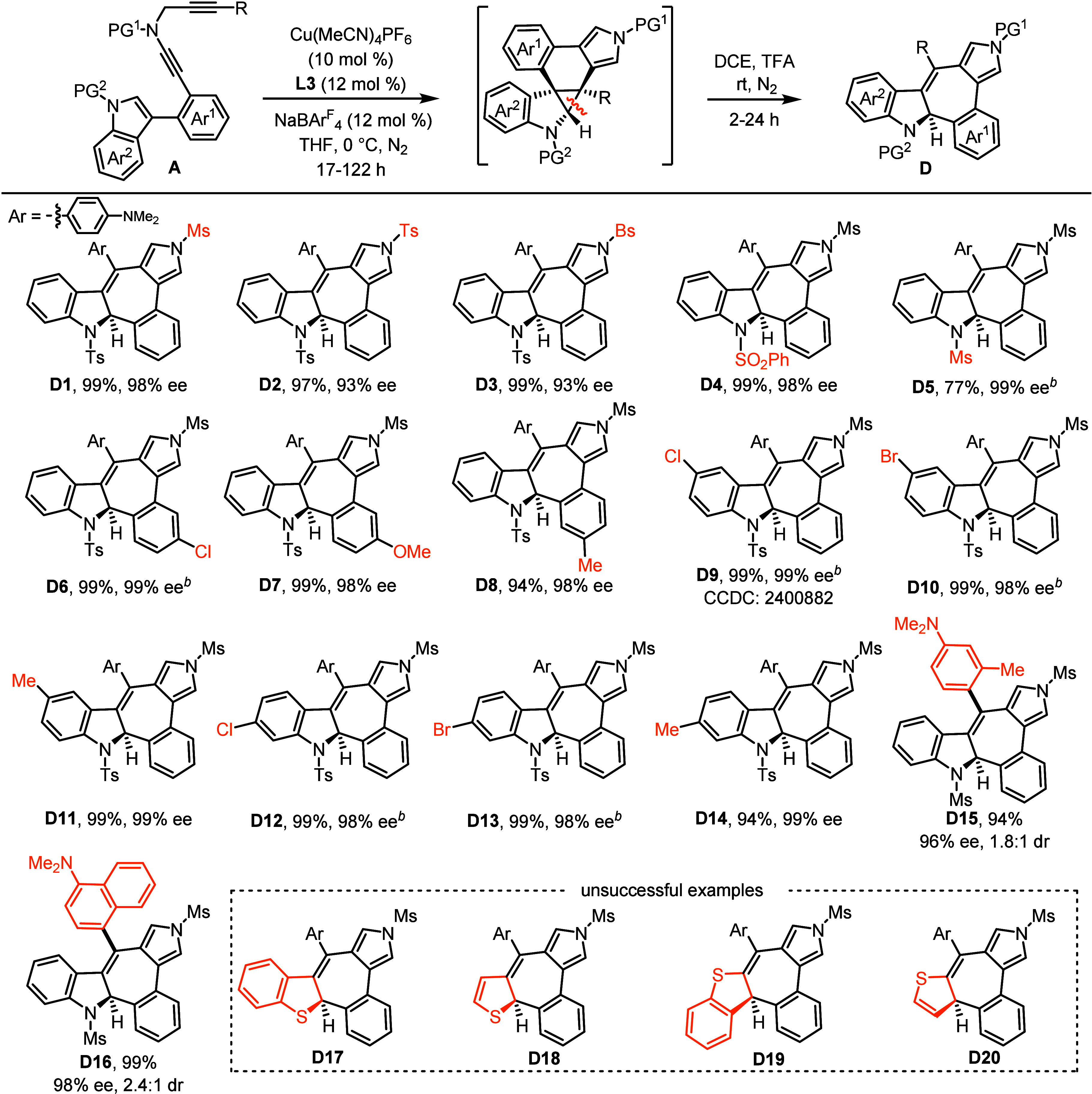
Substrate Scope for the Synthesis of Chiral Cyclohepta­[*b*]­indoles **D** ^a^

a Reaction conditions: **A** (0.15 mmol), Cu­(MeCN)_4_PF_6_ (0.015 mmol), **L3** (0.018 mmol), NaBAr^F^_4_ (0.018 mmol) in THF (3 mL), 0 °C, in Schlenk tubes; after the first step, remove THF and add DCE (3 mL), TFA (1.5 mL), rt; yields are those for the isolated products; ee’s are determined by HPLC analysis.

b The second step’s temperature is 40 °C.

### Gram-Scale Reaction and Product Elaborations

To explore the synthetic utility of this method, a preparative-scale reaction of indole-diyne **A1** was carried out, resulting in the formation of chiral cyclopropane-fused indoline **B1** in 97% yield with 98% ee (Scheme 2A). We also investigated the divergent transformation to generate the chiral 1,2-dioxolane-fused indoline **C1** (87% yield, 98% ee) and cyclohepta­[*b*]­indole **D1** (99% yield, 98% ee) in 1.0 mmol scale reactions (Scheme 2B). Then, further synthetic elaborations of the products described above were explored (Scheme 2C). For example, facile deprotection of the pyrrole ring of **B1** delivered **E1** in 99% yield. **B1** could be reduced by NaBH_3_CN/TFA to obtain **E2** in 82% yield. Meanwhile, **B1** underwent tandem [4 + 2] cycloaddition/rearomative ring expansion to afford **E3** in 99% yield with 6:1 dr (see the Supporting Information). Similarly, deprotection of the pyrrole ring of **C1** and protection again by the Boc group could furnish **E4** in 95% yield. Following, the O–O bond of the chiral 1,2-dioxolane moiety of **C1** was readily cleaved to provide **E5** in 82% yield via Pd/C-catalyzed hydrogenation. Interestingly, selective deprotection of **D1** could deliver **E6** (98%) and **E7** (81%) by treatment with KOH and TfOH, respectively. In addition, **D1** went through a [4 + 2] cycloaddition/1,5-H shift process to gain **E8** in 75% yield with 10:1 dr (see the Supporting Information). Of note, the enantiopurity was well maintained in all these transformations. The absolute configurations of compounds **E3** and **E8** were confirmed by an X-ray crystallographic analysis.

**Scheme 2:**
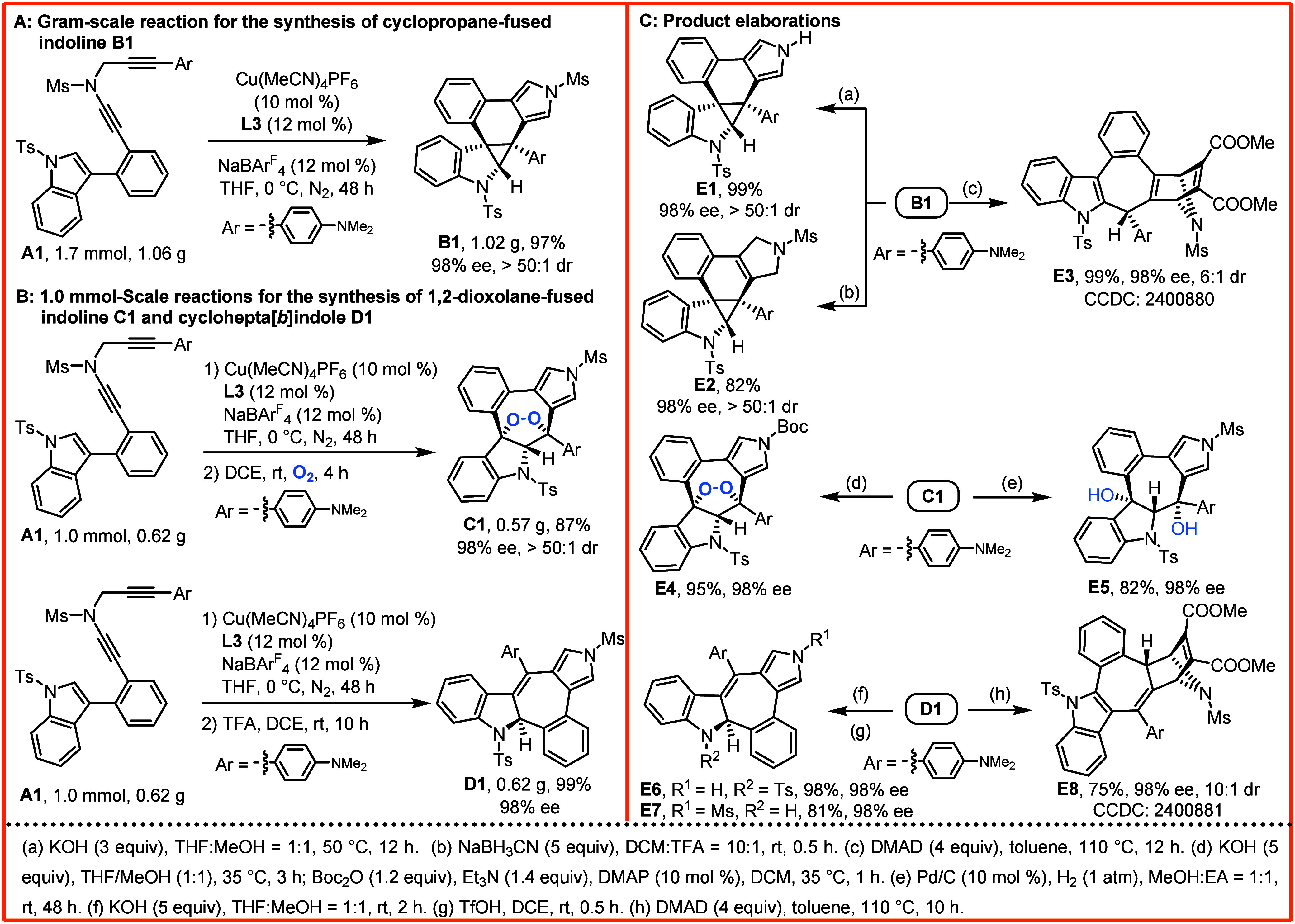
Preparative-Scale Reactions and Product Elaborations

### Mechanistic Studies

To clarify the reasonable pathway for the formation of chiral cyclopropanes **B** via Cu-catalyzed cascade cyclization of diynes **A**, detailed density functional theory (DFT) calculations were conducted using the substrate **A1** as a model based on the above experimental results and our previous studies (Scheme 3A).^43–47^ Initially, **Int**-**B1**, generated from the coordination of copper species to diyne **A1**, underwent intramolecular cyclization to form the vinyl cation intermediate containing the alkenyl copper species **Int**-**B2** with a free energy barrier of 7.6 kcal/mol and was strongly exergonic by 20.8 kcal/mol. Then, this vinyl cation was trapped by the indole moiety to afford the dearomatized carbon cation intermediate **Int**-**B3**, which experienced a free energy barrier of 5.4 kcal/mol and was exergonic by 18.6 kcal/mol. In fact, we also computed another possible process from **Int**-**B2** in which the vinyl cation was trapped by the C3 position of the indole ring, but the computational results exhibited that it was thermodynamically unfavored compared to the pathway displayed above (see Figure S1). Subsequent cyclopropanation of **Int**-**B3** resulted in the carbene intermediate **Int**-**B4** with a free energy barrier of 2.0 kcal/mol and was slightly exergonic by 3.6 kcal/mol. Afterward, **Int**-**B4** went through formal [1,4]-H shift, assisted by another molecule of **A1** as Lewis base,^43–47^ and final demetalation to afford dearomative **B1** with regenerating the copper catalyst. Additionally, after the formation of **Int-B3**, besides the cyclopropanation process mentioned above, we also evaluated the possibility of an **A1**-assisted direct deprotonation process to afford intermediate **Int-B5** via a transition state **TS**_**C2**_. The DFT-computed free energy barrier of the direct deprotonation is 36.7 kcal/mol and cannot possibly occur under the experimental conditions, which support the mechanism above to afford the cyclopropane product **B1** but not the indole C–H functionalization product **Int-B5**.

**Scheme 3:**
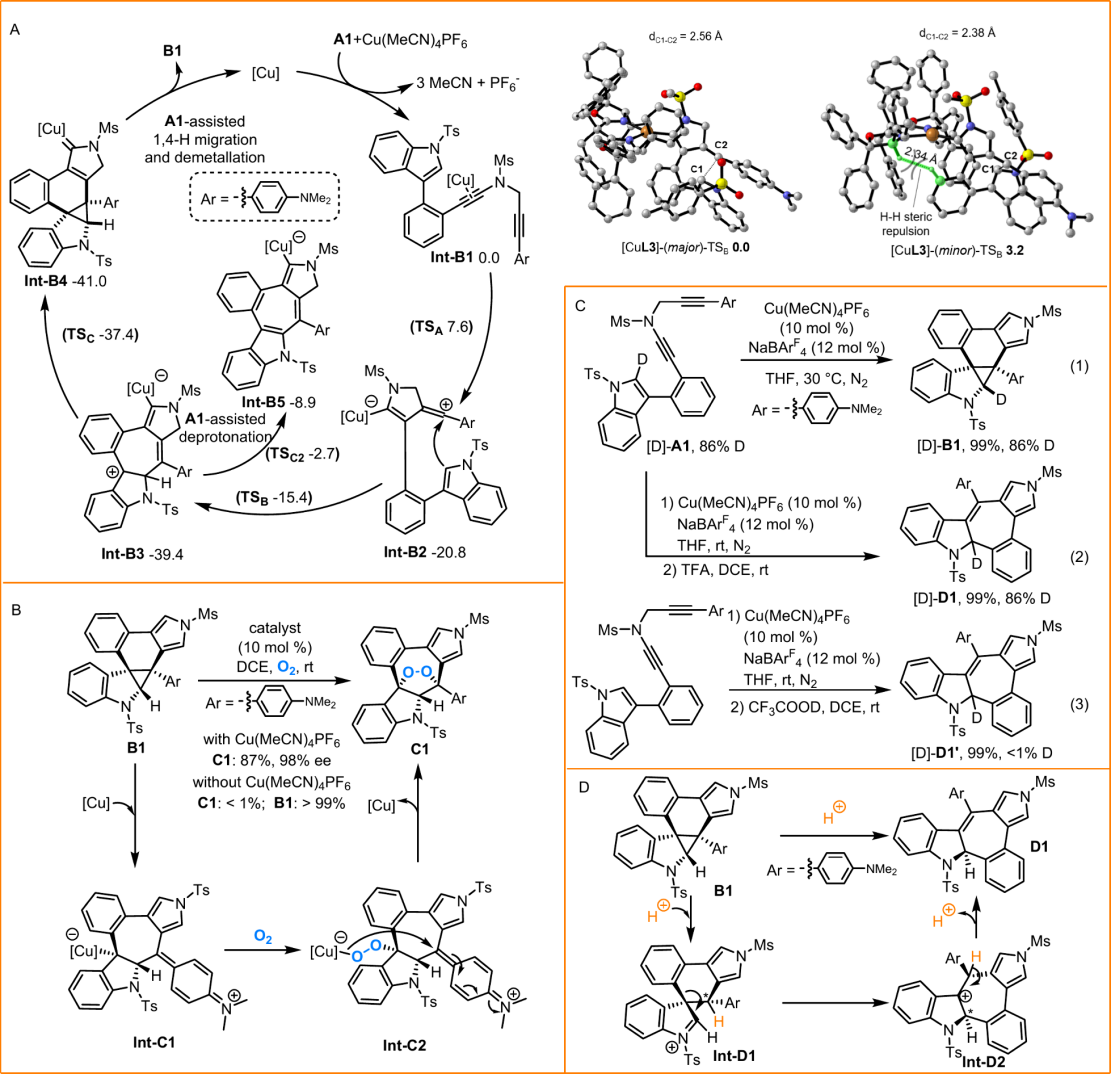
Mechanistic Studies

The enantio-determining step in the synthesis of chiral product **B1** was also computationally investigated, by employing the chiral ligand **L3** coordinated to the Cu^I^ center in the irreversible enantio-determining nucleophilic addition step (Scheme 3A). Upon further observation of structures of these enantio-determining transition states, it was found that in [Cu**L3**]-(*minor*)-**TS**_**B**_, there was a significant H–H repulsion interaction between the substrate and the bulky chiral ligand, which resulted in the free energy difference of 3.2 kcal/mol between the two enantio-determining transition states, ultimately leading to the enantioselectivity (98% ee value) of the product.

Next, two control experiments were carried out to elucidate the potential pathways for the synthesis of enantioenriched 1,2-dioxolanes (Scheme 3B). As a result, cyclopropane **B1** did not undergo [3 + 2] cycloaddition with O_2_ without Cu catalysis, thus indicating that Cu catalysis played the key role in constructing chiral 1,2-dioxolane **C1**. Combining the above truth and observations made by López and Vicente^38^ led us to propose a favorable mechanism. First, **B1** went through a Cu-catalyzed ring opening to generate **Int**-**C1**, which reacted with O_2_ to furnish **Int**-**C2**. Ultimately, intramolecular cyclization provided the 1,2-dioxolane **C1** while simultaneously regenerating the Cu catalyst. Notably, chiral cyclopropane **B1** played a predominant role in inducing chirality in chiral 1,2-dioxolane **C1**.

To elucidate the detailed process in the formation of cyclohepta­[*b*]­indole **D1**, several deuterium labeling experiments were performed (Scheme 3C). First, substrate [D]-**A1** was tested under standard conditions, leading to retention-deuterated cyclopropane [D]-**B1** (Scheme 3C, eq (1)). Subsequently, [D]-**A1** proceeded smoothly for the one-pot synthesis of cyclohepta­[*b*]­indole [D]-**D1** with no decrease in deuterium (Scheme 3C, eq (2)). In addition, **A1** was introduced in the presence of CF_3_COOD to perform this reaction to obtain cyclohepta­[*b*]­indole [D]-**D1**′ with <1% deuterium (Scheme 3C, eq (3)). According to the aforementioned observations, a rational process for the formation of cyclohepta­[*b*]­indole **D1** was described in Scheme 3D. The initial acid-promoted ring opening of **B1**, involving chemoselective C–C bond cleavage, rapidly generated iminium **Int**-**D1**, which underwent a carbocation rearrangement to gain the carbocation **Int**-**D2**. Finally, deprotonation of **Int**-**D2** formed the desired cyclohepta­[*b*]­indole **D1**, accompanied by regeneration of the acid. It is notable that this reaction pathway also well explained the stereocontrol in this rearrangement process.

## Conclusion

In conclusion, we have developed a chiral copper-catalyzed asymmetric dearomative cyclopropanation of indole-diynes, leading to the highly efficient and atom-economic synthesis of a range of chiral cyclopropane-fused polycyclic N-heterocycles in good to excellent yields with generally excellent enantio- and diastereoselectivities via a remote stereocontrol strategy under mild reaction conditions. Interestingly, subsequent [3 + 2] cycloaddition of enantioenriched cyclopropanes with oxygen via controllable C–C bond cleavage provides various chiral 1,2-dioxolanes in moderate to excellent yields with generally excellent stereocontrols under one-pot operation. Thus, this protocol not only represents the first asymmetric dearomative cyclopropanation of indoles utilizing alkynes as carbene precursors but also constitutes the first highly stereocontrolled construction of chiral 1,2-dioxolanes from alkynes. Moreover, further acid-promoted one-pot construction of chiral cyclohepta­[*b*]­indoles exhibits significantly different chemoselectivity in C–C bond cleavage and intriguing indole rearrangement. In addition, control experiments and theoretical calculations probably reveal both potential reaction pathways and the origins of chiral control within this Cu-catalyzed asymmetric tandem sequence for the divergent preparation of three chiral N-heterocycles. Further investigation of constructing other types of chiral N-heterocycles via chiral Cu-catalyzed alkyne transformations is ongoing in our laboratory.

## Supplementary Material

















## Notes

Experimental procedures, characterizations and analytical data of products, and HPLC and NMR spectra (PDF)
Transparent Peer Review report available (PDF)
Crystallographic structure of **B1** (CIF)
Crystallographic structure of **B32** (CIF)
Crystallographic structure of **C1** (CIF)
Crystallographic structure of **D9** (CIF)
Crystallographic structure of **E3** (CIF)
Crystallographic structure of **E8** (CIF)
