# Supplementary material for: Divergent and Enantioselective Synthesis of Three Types of Chiral Polycyclic N‑Heterocycles via Copper-Catalyzed Dearomative Cyclization
Source: ACS Cent Sci. 2025 May 2;11(5):805–15. doi: 10.1021/acscentsci.5c00248 (PMC12123464; doi:10.1021/acscentsci.5c00248)

Supporting Information

Divergent and Enantioselective Synthesis of Three Types of Chiral Polycyclic N-Heterocycles via Copper-Catalyzed Dearomative Cyclization

Wen-Feng Luo,[a,b,#] Li-Gao Liu,[a,#] Yan-Xin Zheng,[a,#] Miao Sun,[a] Xin Lu,[a] Bo Zhou,[a] Long-Wu Ye,[a,c] and Long Li*[b]

[a]State Key Laboratory of Physical Chemistry of Solid Surfaces, Key Laboratory of Chemical Biology of Fujian Province, and College of Chemistry and Chemical Engineering, Xiamen University, Xiamen 361005, China

[b]College of Chemistry & Materials Engineering, Wenzhou University, Wenzhou 325035, China

[c]China State Key Laboratory of Organometallic Chemistry, ShanghaiInstitute of Organic Chemistry, Chinese Academy of Sciences, Shanghai 200032, China

*E-mail: longwzu1990@wzu.edu.cn

Content	Page Number
1. General Information	2
2. More Reaction Condition and Mechanism Studies	3
3. Preparation of Starting Materials	8
4. General Procedure for the Synthesis of Chiral Cyclopropane-Fused Indolines, 1,2-Dioxolanes and Cyclohepta[*b*]indoles	35
5. Crystal Data	100
6. Theoretical Calculations	106
7. References	171
8. HPLC Chromatograms	173
9. NMR Spectra	248

1. General Information

Ethyl acetate (ACS grade), hexanes (ACS grade) and anhydrous 1,2-dichloroethane (ACS grade) were obtained commercially and used without further purification. Methylene chloride, tetrahydrofuran, toluene and diethyl ether were purified according to standard methods unless otherwise noted. Commercially available reagents were used without further purification. Reactions were monitored by thin layer chromatography (TLC) using silicycle pre-coated silica gel plates. Flash column chromatography was performed over silica gel (300-400 mesh). Infrared spectra were recorded on a Nicolet AVATER FTIR330 spectrometer as thin film and are reported in reciprocal centimeter (cm^{-1}). Mass spectra were recorded with Micromass QTOF2 Quadrupole/Time-of-Flight Tandem mass spectrometer using electron spray ionization.

^{1}H NMR spectra, ^{19}F NMR spectra and ^{13}C NMR spectra were recorded on a Bruker AV-400 spectrometer or a Bruker AV-500 in chloroform-d_3 and dimethyl sulfoxide-d_6. For ^{1}H NMR spectra, chemical shifts are reported in ppm with the internal TMS signal at 0.0 ppm as a standard. For ^{13}C NMR spectra, chemical shifts are reported in ppm with the internal chloroform signal at 77.0 ppm as a standard. The data is being reported as (s = singlet, d = doublet, t = triplet, q = quartet, m = multiplet or unresolved, coupling constant(s) in Hz, integration).

Enantiomeric excesses (ee) were determined by an UltiMate 3000 chiral HPLC. The chiral columns used for the determination of enantiomeric excesses by chiral HPLC were Chiralpak columns (IA, IB, IC, IE, INA). The particle size is 5 μm and dimensions is 4.6 mm I.D * 250 mm L.

2. More Reaction Condition and Mechanism Studies

2.1 More Reaction Condition Studies

Table S1. Screening of reaction conditions for the synthesis of chiral 1,2-dioxolane **C1**[a]

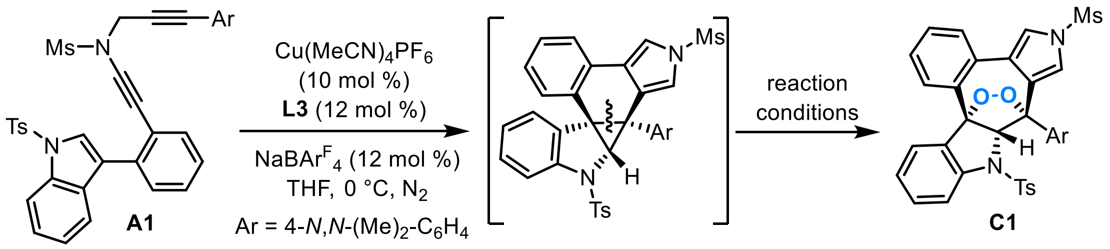

entry	reaction conditions	yield (%)[b]	ee (%)[c]
1	THF, O_2, rt, 12 h	31	98
**2**	**DCE, O_2, rt, 2.5 h**	**87**	**98**
3	toluene, O_2, rt, 4 h	75	98
4	Et_2O, O_2, rt, 12 h	<5	98
5	PhCl, O_2, rt, 4 h	62	98
6	1,4-dioxane, O_2, rt, 12 h	35	98

[a]Reaction conditions: **A1** (0.05 mmol), $Cu(MeCN)_4PF_6$ (10 mol %), **L3** (12 mol %), $NaBAr^F{}_4$ (12 mol %) in THF (1 mL), 0 °C, in Schlenk tubes; after the first step, add solvent (0.3 mL) and change the reaction atmosphere from nitrogen to oxygen, rt; [b]Measured by ^1H NMR using 1,3,5-trimethoxybenzene as the internal standard; [c]Determined by HPLC analysis

Table S2. Screening of reaction conditions for the synthesis of chiral cyclohepta[*b*]indole **D1**[a]

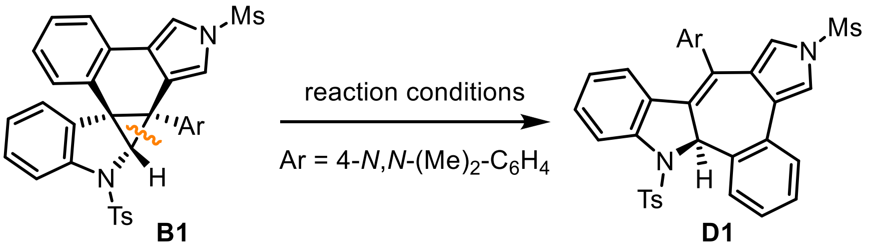

entry	reaction conditions	yield (%)[b]	ee (%)[c]
1	TFA (0.5 mL), THF, rt, 24 h	<1	-
**2**	**TFA (0.5 mL), DCE, rt, 4 h**	**99**	**98**
3	HNTf$_2$ (130 eq), DCE, rt, 2 h	45	98
4	TsOH (130 eq), DCE, rt, 10 h	<1	-
5	MsOH (0.5 mL), DCE, rt, 4 h	82	98
6	TfOH (0.5 mL), DCE, rt, 4 h	<1	-

[a]Reaction conditions: **B1** (0.05 mmol), acid, in solvent (1 mL), rt, in Schlenk tubes; [b]Measured by ^{1}H NMR using 1,3,5-trimethoxybenzene as the internal standard; [c]Determined by HPLC analysis.

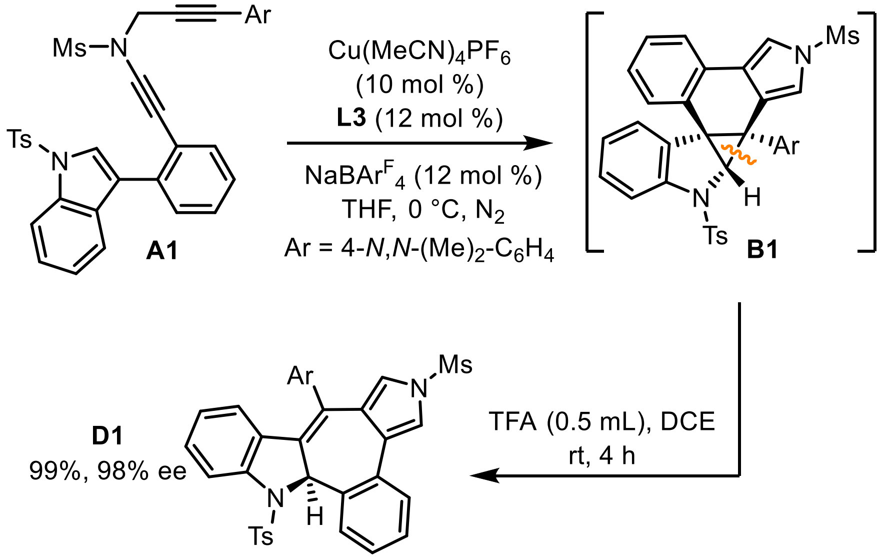

Reaction conditions: **A1** (0.05 mmol), Cu(MeCN)$_4$PF$_6$ (10 mol %), **L3** (12 mol %), NaBAr$^F{}_4$ (12 mol %) in THF (1 mL), 0 °C, in Schlenk tubes; after the first step, remove the THF and add DCE (1 mL), TFA (0.5 mL), rt.

2.2 Mechanism Studies

The reaction of [D]-**A1** under the standard conditions:

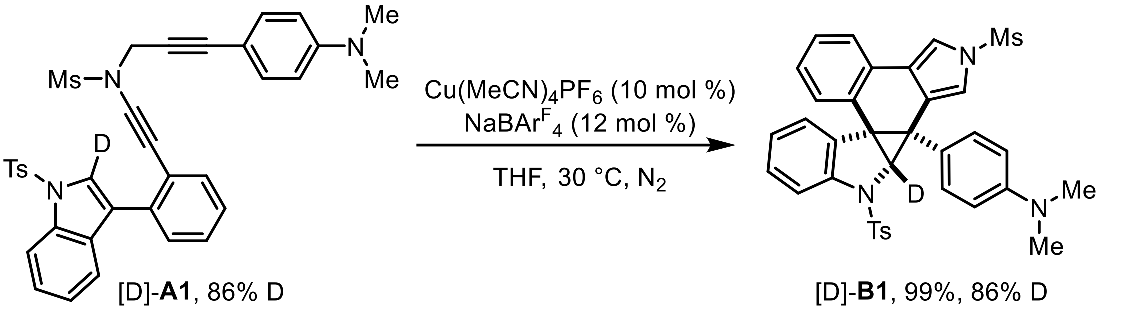

[D]-**A1**, 86% D   [D]-**B1**, 99%, 86% D

The reaction of [D]-**A1** under the standard reaction conditions resulted in [D]-**B1** in 99% yield. The deuterium atom was completely retained in product [D]-**B1**.

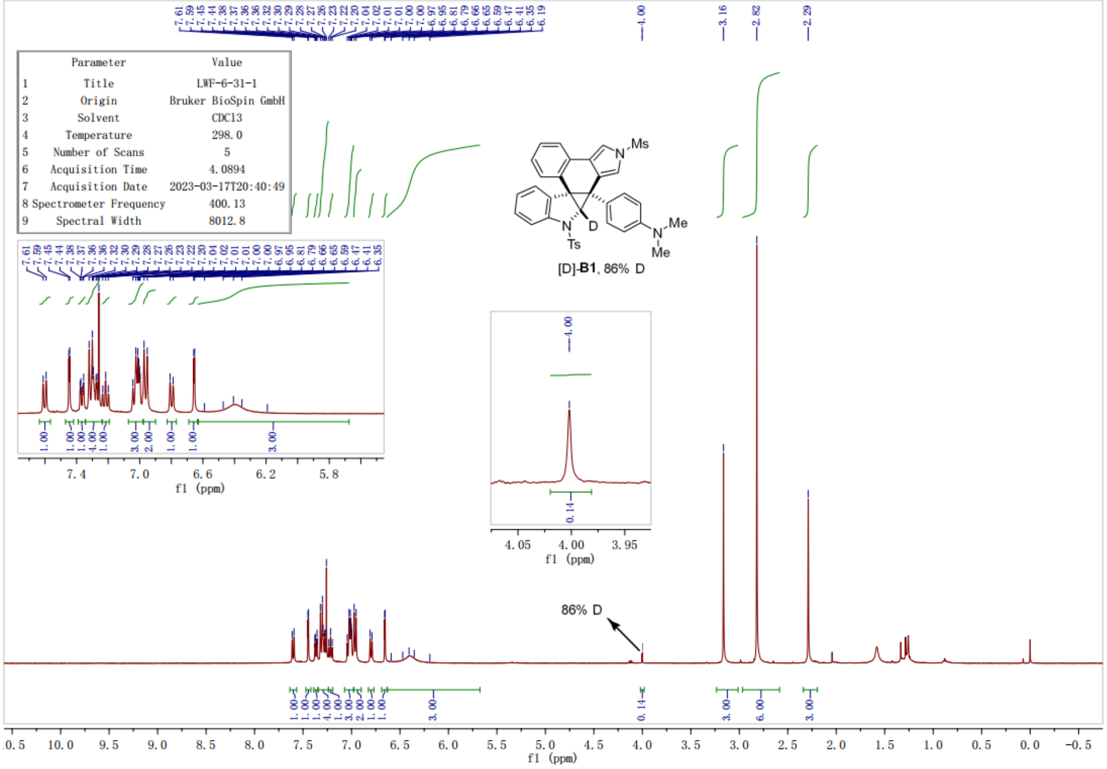

The reaction of [D]-**A1** under the standard conditions:

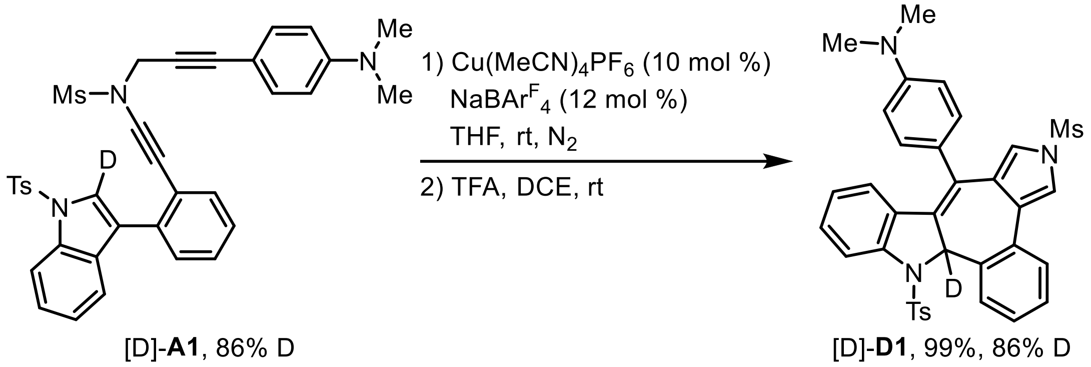

The reaction of [D]-**A1** under the standard reaction conditions resulted in [D]-**D1** in 99% yield. The deuterium atom was completely retained in product [D]-**D1**.

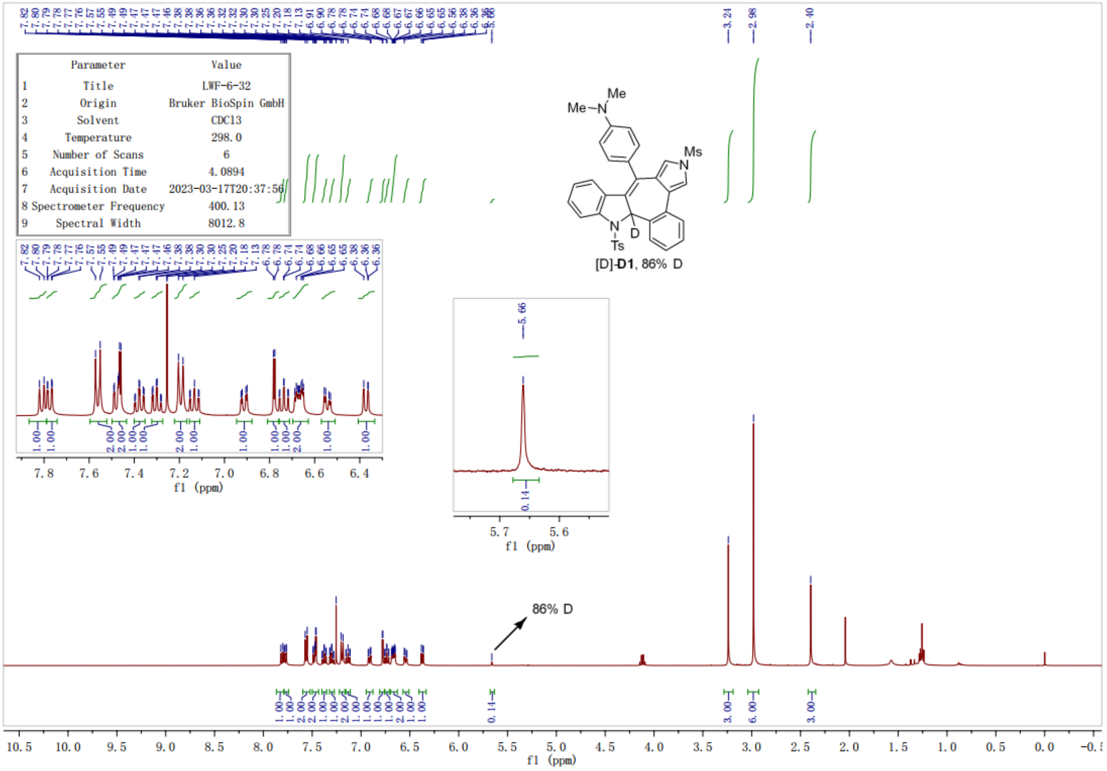

Hydrogen/deuterium exchange experiments of **A1** and (±)-[D]-**D1**:

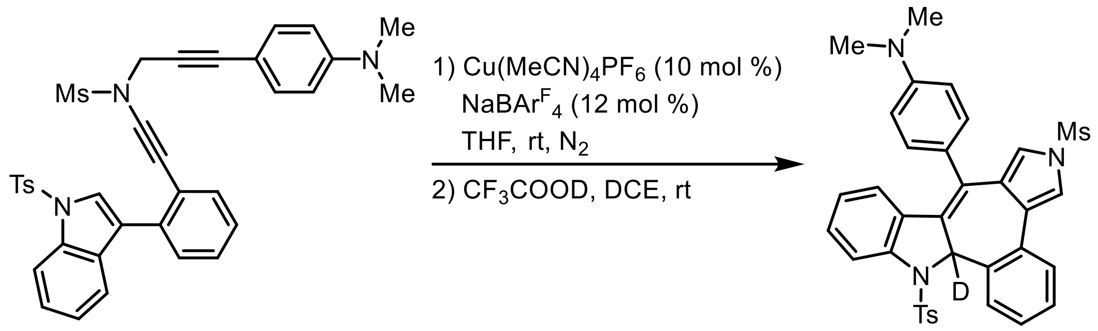

A1 was introduced in the presence of CF_3COOD to perform this reaction to obtain cyclohepta[*b*]indole [D]-**D1’** in 99% yield with <1% deuterium.

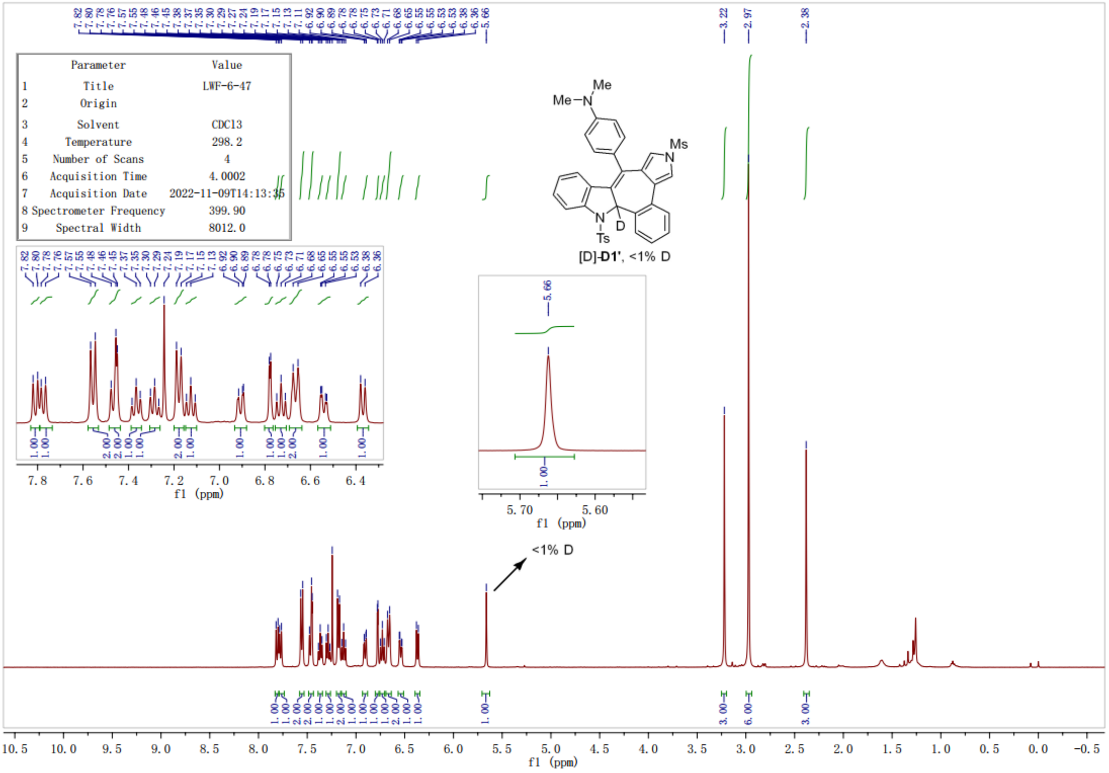

3. Preparation of Starting Materials

General synthetic procedures for the preparation of *N*-propargyl ynamides A:

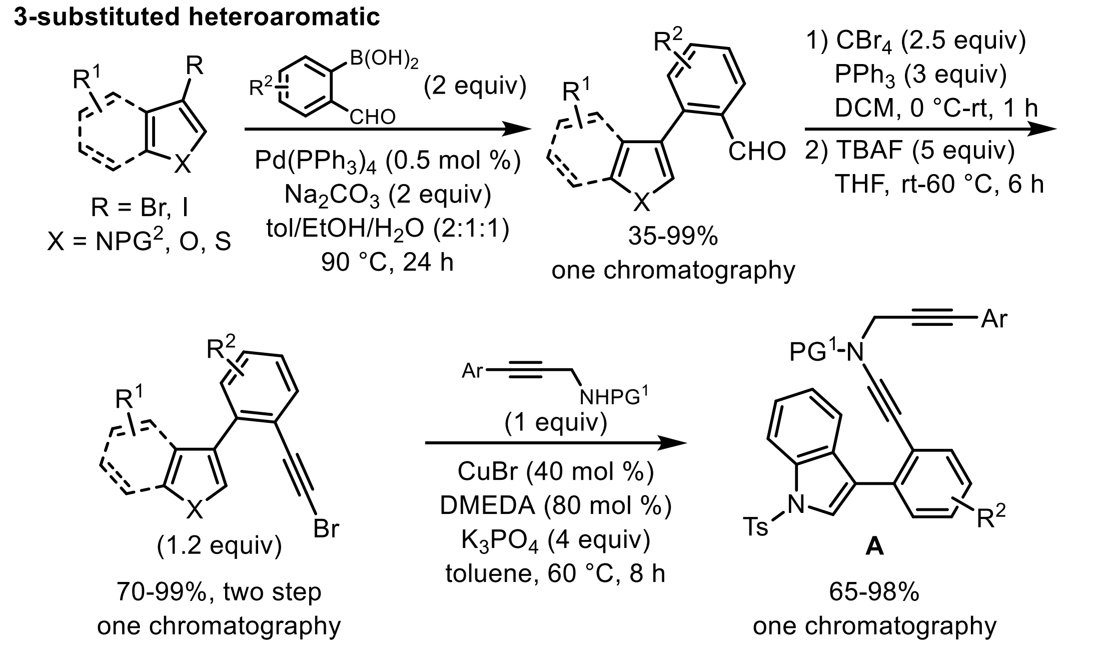

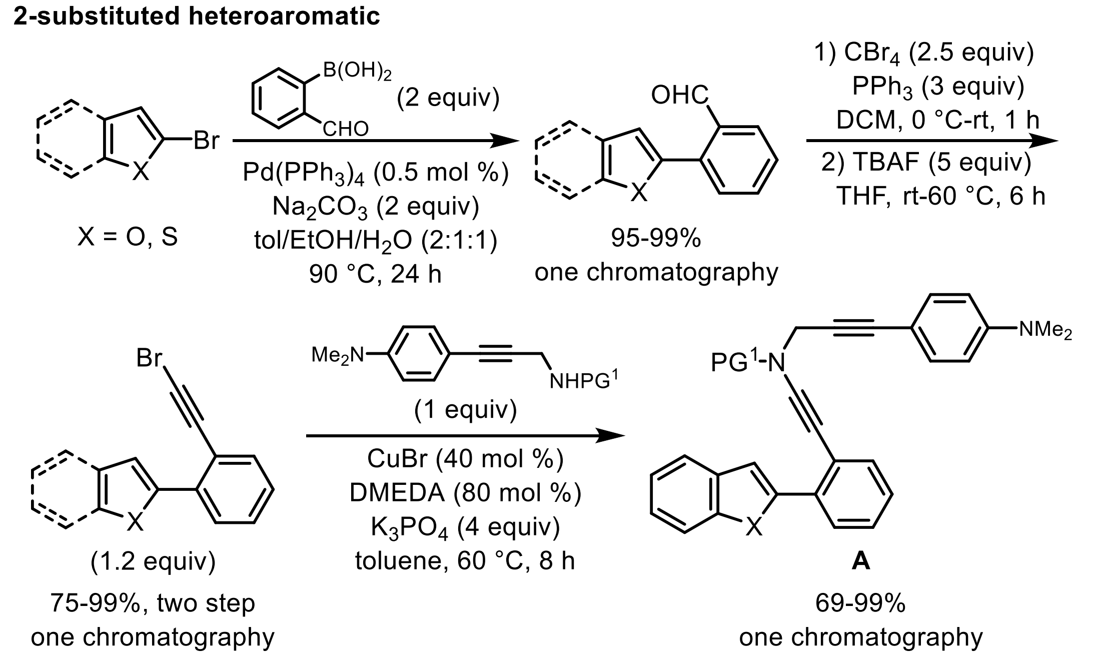

To a solution of substituted bromo-heteroaromatic and iodide-heteroaromatic (5 mmol) in toluene (20 mL), EtOH (10 mL) and H_2O (10 mL) were added substituted 2-formylbenzeneboronic acid (10 mmol), Na_2CO_3 (10 mmol, 2.12 g) and $Pd(PPh_3)_4$ (0.025 mmol, 57.8 mg) at room temperature. The reaction mixture was stirred at 90 °C for 24 h.[1] Upon completion (monitored by TLC), the reaction was filtered, extracted

with ethyl acetate for three times, dried over $MgSO_4$ and filtered. The filtrate was concentrated under reduced pressure and purified by column chromatography on silica gel (eluent: PE/EtOAc) to afford the desired heteroaromatic substituted benzaldehyde (35–99% yield).

To a solution of PPh_3 (4.4 mmol, 1.15 g) in DCM (5 mL) was added CBr_4 (5.3 mmol, 1.76 g) carefully at 0 °C, and the reaction was stirred at this temperature for 0.5 h. Then the solution of the above heteroaromatic substituted benzaldehyde (1.75 mmol) in DCM (2 mL) was added slowly at 0 °C. The reaction was warmed to room temperature and stirred for 1 h.[2] Upon completion (monitored by TLC), the reaction was concentrated under reduced pressure, the coarse product through a Celite pad, and washed with PE/EtOAc = 10:1 for three times. The filtrate was concentrated under reduced pressure and directly used in the next step without further purification. To a solution of dibromide derivative (1.75 mmol) in THF (5 mL) was added TBAF (8.75 mmol, 2.29 g) and the reaction was stirred at 60 °C for 6 h.[2] Upon completion (monitored by TLC), the reaction mixture was diluted with water, extracted with ethyl acetate for three times, dried over $MgSO_4$, and filtered. The filtrate was concentrated under reduced pressure and purified by column chromatography on silica gel (eluent: PE/EtOAc) to afford the alkynyl bromide (70%–99% yield, two steps).

To a solution of the above alkynyl bromide (1.2 mmol) in toluene (5 mL) were added copper bromide (0.4 mmol, 57.4 mg), DMEDA (0.8 mmol, 70.5 mg), K_3PO_4 (4 mmol, 0.85 g), and protected propargylamide derivative (1 mmol).[3] The reaction was stirred at 60 °C for 8 h. Upon completion (monitored by TLC), the solution was filtered and concentrated under reduced pressure. The residue was purified by chromatography on silica gel (eluent: PE/EtOAc) to give the desired ynamides **A** (65–98% yield).

***N*-(3-(4-(dimethylamino)phenyl)prop-2-yn-1-yl)-*N*-((2-(1-tosyl-1*H*-indol-3-yl)phenyl)ethynyl)methanesulfonamide (A1)**

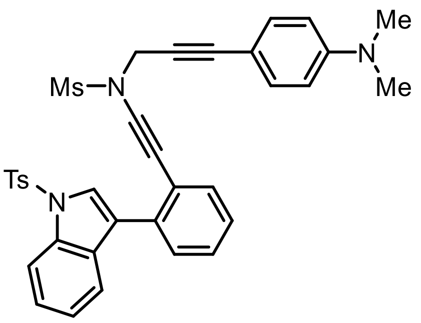

A1

Compound **A1** was prepared in 58% yield (576.5 mg) with four steps according to the general procedure as a white solid (mp 161–162 °C). ^{1}H NMR (400 MHz, $CDCl_3$) δ 7.99 (d, *J* = 8.4 Hz, 1H), 7.86 (s, 1H), 7.79 (d, *J* = 8.4 Hz, 2H), 7.59 (d, *J* = 7.6 Hz, 2H), 7.46 (d, *J* = 7.2 Hz, 1H), 7.39 – 7.34 (m, 1H), 7.34 – 7.29 (m, 1H), 7.27 (d, *J* = 7.6 Hz, 1H), 7.24 – 7.19 (m, 4H), 7.17 (d, *J* = 7.6 Hz, 1H), 6.56 (d, *J* = 8.8 Hz, 2H), 4.28 (s, 2H), 2.95 (s, 6H), 2.72 (s, 3H), 2.31 (s, 3H); ^{13}C NMR (100 MHz, $CDCl_3$) δ 150.4, 145.0, 135.1, 134.8, 134.4, 133.2, 132.9, 129.9, 129.8, 129.7, 128.1, 127.4, 126.9, 124.8, 124.6, 123.3, 122.3, 121.8, 121.2, 113.6, 111.6, 108.2, 88.1, 85.1, 79.1, 70.5, 43.0, 40.1, 38.4, 21.5; IR (neat): 2922(bs), 2227(s), 1606, 1523, 1445, 1361, 1169, 1024, 755, 575 cm^{-1}; HRESIMS Calcd for $[C_{35}H_{31}N_3NaO_4S_2]^+$ (M + Na$^+$) 644.1648, found 644.1642.

***N*-(3-(4-(dimethylamino)phenyl)prop-2-yn-1-yl)-4-methyl-*N*-((2-(1-tosyl-1*H*-indol-3-yl)phenyl)ethynyl)benzenesulfonamide (A2)**

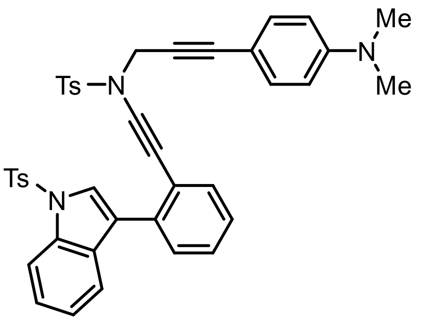

A2

Compound **A2** was prepared in 49% yield (548.4 mg) with four steps according to the general procedure as a white solid (mp 94–95 °C). ^{1}H NMR (400 MHz, $CDCl_3$) δ 8.02 (d, *J* = 7.6 Hz, 1H), 7.89 (s, 1H), 7.81 (d, *J* = 7.2 Hz, 2H), 7.70 (d, *J* = 7.2 Hz, 2H), 7.60

– 7.50 (m, 2H), 7.44 (d, *J* = 6.4 Hz, 1H), 7.37 – 7.08 (m, 8H), 6.95 (d, *J* = 7.6 Hz, 2H), 6.46 (d, *J* = 7.6 Hz, 2H), 4.26 (s, 2H), 2.89 (s, 6H), 2.30 (s, 3H), 2.25 (s, 3H); ^{13}C NMR (100 MHz, $CDCl_3$) δ 150.0, 144.9, 144.6, 135.1, 134.8, 134.1, 133.3, 132.7, 129.82, 129.75, 129.6, 129.4, 127.88, 127.85, 127.2, 126.8, 125.0, 124.5, 123.2, 121.8, 121.7, 120.8, 113.5, 111.3, 108.6, 87.6, 85.7, 78.6, 70.4, 42.9, 40.0, 21.5, 21.4; IR (neat): 3064(bs), 2229(s), 1608, 1520, 1446, 1366, 1170, 1131, 665, 582 cm^{-1}; HRESIMS Calcd for $[C_{41}H_{35}N_3NaO_4S_2]^+$ (M + Na^+) 720.1961, found 720.1955.

N-(3-(4-(dimethylamino)phenyl)prop-2-yn-1-yl)-N-((2-(1-tosyl-1H-indol-3-yl)phenyl)ethynyl)benzenesulfonamide (A3)

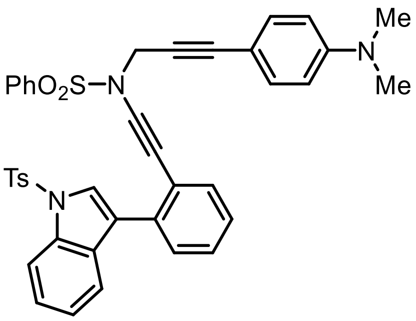

A3

Compound **A3** was prepared in 58% yield (625.1 mg) with four steps according to the general procedure as a white solid (mp 91–93 °C). ^{1}H NMR (400 MHz, $CDCl_3$) δ 8.02 (d, *J* = 8.4 Hz, 1H), 7.87 (s, 1H), 7.83 – 7.77 (m, 4H), 7.56 (d, *J* = 8.0 Hz, 1H), 7.52 (d, *J* = 7.2 Hz, 1H), 7.49 (d, *J* = 7.2 Hz, 1H), 7.45 (d, *J* = 7.6 Hz, 1H), 7.37 – 7.29 (m, 4H), 7.28 (d, *J* = 7.6 Hz, 1H), 7.21 (d, *J* = 9.6 Hz, 1H), 7.17 (d, *J* = 8.0 Hz, 2H), 6.97 (d, *J* = 8.8 Hz, 2H), 6.47 (d, *J* = 8.8 Hz, 2H), 4.28 (s, 2H), 2.91 (s, 6H), 2.27 (s, 3H); ^{13}C NMR (100 MHz, $CDCl_3$) δ 150.1, 144.9, 137.0, 135.1, 134.8, 134.2, 133.6, 133.3, 132.7, 129.9, 129.8, 129.7, 128.8, 127.93, 127.89, 127.2, 126.8, 125.0, 124.6, 123.2, 121.8, 120.9, 113.5, 111.4, 108.5, 87.7, 85.5, 78.6, 70.5, 43.0, 40.0, 21.5; IR (neat): 2922(bs), 2230(s), 1608, 1522, 1446, 1367, 1174, 1131, 1088, 748, 581 cm^{-1}; HRESIMS Calcd for $[C_{40}H_{33}N_3NaO_4S_2]^+$ (M + Na^+) 706.1805, found 706.1801.

N-(3-(4-(dimethylamino)phenyl)prop-2-yn-1-yl)-4-methoxy-N-((2-(1-tosyl-1H-indol-3-yl)phenyl)ethynyl)benzenesulfonamide (A4)

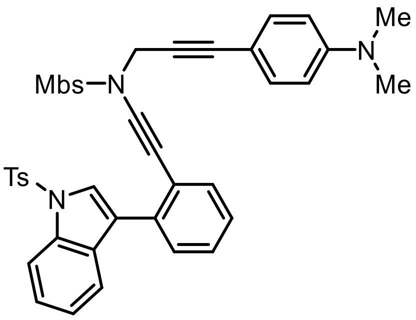

A4

Compound **A4** was prepared in 62% yield (709.7 mg) with four steps according to the general procedure as a white solid (mp 88–91 °C). ^{1}H NMR (400 MHz, $CDCl_3$) δ 8.03 (d, *J* = 8.4 Hz, 1H), 7.89 (s, 1H), 7.81 (d, *J* = 8.0 Hz, 2H), 7.71 (d, *J* = 8.8 Hz, 2H), 7.57 (d, *J* = 7.6 Hz, 1H), 7.53 (d, *J* = 7.6 Hz, 1H), 7.44 (d, *J* = 7.2 Hz, 1H), 7.35 – 7.16 (m, 6H), 6.97 (d, *J* = 8.4 Hz, 2H), 6.77 (d, *J* = 8.8 Hz, 2H), 6.47 (d, *J* = 8.4 Hz, 2H), 4.27 (s, 2H), 3.72 (s, 3H), 2.90 (s, 6H), 2.26 (s, 3H); ^{13}C NMR (100 MHz, $CDCl_3$) δ 163.6, 150.1, 144.9, 135.1, 134.8, 134.0, 133.3, 132.7, 130.2, 129.9, 129.6, 128.6, 127.8, 127.2, 126.8, 125.0, 124.5, 123.3, 122.0, 121.9, 120.9, 114.0, 113.5, 111.4, 108.7, 87.6, 86.0, 78.8, 70.5, 55.5, 42.9, 40.0, 21.4; IR (neat): 2924(bs), 2229(s), 1608, 1596, 1521, 1446, 1366, 1176, 1131, 582 cm^{-1}; HRESIMS Calcd for $[C_{41}H_{35}N_3NaO_5S_2]^+$ (M + Na^+) 736.1910, found 736.1905.

***4*-bromo-N-(3-(4-(dimethylamino)phenyl)prop-2-yn-1-yl)-N-((2-(1-tosyl-1H-indol-3-yl)phenyl)ethynyl)benzenesulfonamide (A5)**

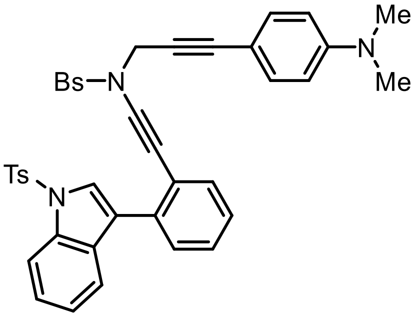

A5

Compound **A5** was prepared in 44% yield (533.9 mg) with four steps according to the general procedure as a white solid (mp 97–100 °C). ^{1}H NMR (400 MHz, $CDCl_3$) δ 8.03 (d, *J* = 8.0 Hz, 1H), 7.87 (s, 1H), 7.82 (d, *J* = 7.2 Hz, 2H), 7.63 (d, *J* = 6.8 Hz, 2H), 7.56 (d, *J* = 8.0 Hz, 1H), 7.52 (d, *J* = 7.6 Hz, 1H), 7.48 – 7.40 (m, 3H), 7.37 – 7.28 (m, 3H), 7.24 – 7.18 (m, 3H), 6.93 (d, *J* = 7.2 Hz, 2H), 6.51 (d, *J* = 7.2 Hz, 2H), 4.27 (s, 2H), 2.94 (s, 6H), 2.29 (s, 3H); ^{13}C NMR (100 MHz, $CDCl_3$) δ 150.2, 144.9, 136.2, 135.1, 134.8, 134.1, 133.2, 132.7, 132.0, 129.9, 129.8, 129.7, 129.4, 128.9, 128.0, 127.3, 126.8, 125.0, 124.6, 123.3, 121.8, 121.7, 120.8, 113.6, 111.5, 108.2, 88.1, 85.2, 78.4, 70.7, 43.2, 40.0, 21.4; IR (neat): 2921(bs), 2230(s), 1607, 1521, 1446, 1370, 1175, 1131, 1087, 745, 581, cm^{-1}; HRESIMS Calcd for $[C_{40}H_{32}BrN_3NaO_4S_2]^+$ (M + Na$^+$) 784.0910 (isotopic ion: 786.0896), found 784.0906 (isotopic ion: 786.0890).

***N*-(3-(4-(dibenzylamino)phenyl)prop-2-yn-1-yl)-*N*-((2-(1-tosyl-1*H*-indol-3-yl)phenyl)ethynyl)methanesulfonamide (A6)**

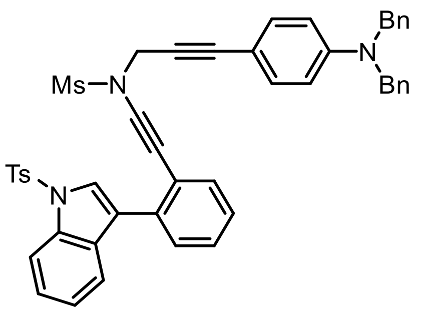

A6

Compound **A6** was prepared in 61% yield (375.3 mg) with four steps according to the general procedure as a white solid (mp 88–90 °C). ^{1}H NMR (400 MHz, $CDCl_3$) δ 7.96 (d, *J* = 8.4 Hz, 1H), 7.85 (s, 1H), 7.79 (d, *J* = 8.4 Hz, 2H), 7.61 – 7.53 (m, 2H), 7.48 – 7.43 (m, 1H), 7.39 – 7.36 (m, 1H), 7.35 – 7.34 (m, 1H), 7.33 – 7.32 (m, 2H), 7.31 – 7.29 (m, 2H), 7.29 – 7.27 (m, 1H), 7.27 – 7.25 (m, 1H), 7.24 (s, 1H), 7.23 – 7.17 (m, 6H), 7.15 – 7.07 (m, 3H), 6.58 (d, *J* = 9.2 Hz, 2H), 4.65 (s, 4H), 4.26 (s, 2H), 2.73 (s, 3H), 2.31 (s, 3H); ^{13}C NMR (100 MHz, $CDCl_3$) δ 149.3, 145.0, 137.7, 135.1, 134.8, 134.4, 133.2, 133.1, 129.9, 129.8, 129.7, 128.7, 128.1, 127.4, 127.1, 126.9, 126.4, 124.9, 124.6, 123.3, 122.2, 121.8, 121.1, 113.6, 112.0, 108.9, 87.9, 85.1, 79.2, 70.5, 54.1, 43.0,

38.4, 21.5; IR (neat): 3028(bs), 2232(s), 1604, 1519, 1361, 1249, 1166, 1130, 748, 581 cm^{-1}; HRESIMS Calcd for $[C_{47}H_{39}N_3NaO_4S_2]^+$ (M + Na^+) 796.2274, found 796.2256.

***N*-(3-(4-(pyrrolidin-1-yl)phenyl)prop-2-yn-1-yl)-*N*-((2-(1-tosyl-1*H*-indol-3-yl)phenyl)ethynyl)methanesulfonamide (A7)**

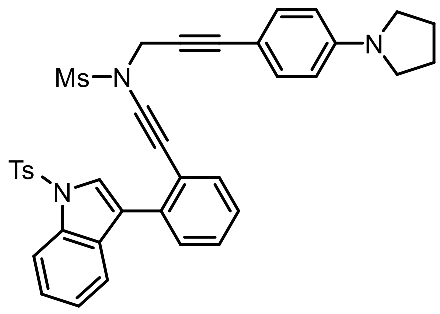

A7

Compound **A7** was prepared in 46% yield (235.6 mg) with four steps according to the general procedure as a white solid (mp 107–110 °C). ^{1}H NMR (400 MHz, $CDCl_3$) δ 7.99 (d, *J* = 8.4 Hz, 1H), 7.85 (s, 1H), 7.80 (d, *J* = 8.4 Hz, 2H), 7.60 (d, *J* = 8.0 Hz, 2H), 7.50 – 7.43 (m, 1H), 7.40 – 7.35 (m, 1H), 7.35 – 7.31 (m, 1H), 7.31 – 7.27 (m, 1H), 7.24 – 7.08 (m, 5H), 6.42 (d, *J* = 8.8 Hz, 2H), 4.27 (s, 2H), 3.38 – 3.19 (m, 4H), 2.71 (s, 3H), 2.33 (s, 3H), 2.07 – 1.96 (m, 4H); ^{13}C NMR (100 MHz, $CDCl_3$) δ 148.0, 145.0, 135.2, 134.8, 134.5, 133.2, 133.1, 133.0, 129.9, 129.8, 128.1, 127.4, 126.9, 124.9, 124.6, 123.4, 122.4, 121.9, 121.2, 113.6, 111.3, 107.3, 88.5, 85.2, 78.9, 70.5, 47.4, 43.1, 38.4, 25.4, 21.5; IR (neat): 2968(bs), 2232(s), 1606, 1521, 1446, 1366, 1187, 1175, 1131, 749, 581 cm^{-1}; HRESIMS Calcd for $[C_{37}H_{33}N_3NaO_4S_2]^+$ (M + Na^+) 670.1805, found 670.1792.

***N*-(3-(4-methoxyphenyl)prop-2-yn-1-yl)-*N*-((2-(1-tosyl-1*H*-indol-3-yl)phenyl)ethynyl)methanesulfonamide (A8)**

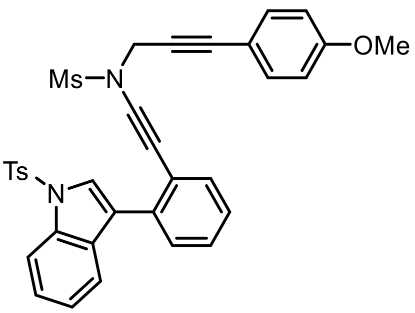

A8

Compound **A8** was prepared in 59% yield (569.1 mg) with four steps according to the general procedure as a white solid (mp 92–94 °C). ^{1}H NMR (400 MHz, $CDCl_3$) δ 7.98 (d, *J* = 8.4 Hz, 1H), 7.86 (s, 1H), 7.80 (d, *J* = 8.4 Hz, 2H), 7.62 – 7.60 (m, 1H), 7.60 – 7.57 (m, 1H), 7.49 – 7.45 (m, 1H), 7.41 – 7.36 (m, 1H), 7.35 – 7.32 (m, 1H), 7.31 – 7.27 (m, 1H), 7.27 – 7.24 (m, 2H), 7.23 (s, 1H), 7.22 – 7.16 (m, 2H), 6.81 – 6.79 (m, 1H), 6.78 – 6.76 (m, 1H), 4.30 (s, 2H), 3.80 (s, 3H), 2.76 (s, 3H), 2.33 (s, 3H); ^{13}C NMR (100 MHz, $CDCl_3$) δ 160.0, 145.1, 135.2, 134.8, 134.4, 133.3, 133.2, 130.0, 129.84, 129.77, 128.2, 127.5, 126.9, 124.9, 124.6, 123.3, 122.2, 121.8, 121.1, 114.0, 113.73, 113.65, 86.9, 84.9, 80.0, 70.7, 55.3, 42.8, 38.5, 21.6; IR (neat): 2929(bs), 2234(s), 1606, 1509, 1447, 1363, 1250, 1166, 1131, 581 cm^{-1}; HRESIMS Calcd for $[C_{34}H_{28}N_2NaO_5S_2]^+$ (M + Na$^+$) 631.1332, found 631.1322.

***N*-(3-(4-(benzyloxy)phenyl)prop-2-yn-1-yl)-*N*-((2-(1-tosyl-1*H*-indol-3-yl)phenyl)ethynyl)methanesulfonamide (A9)**

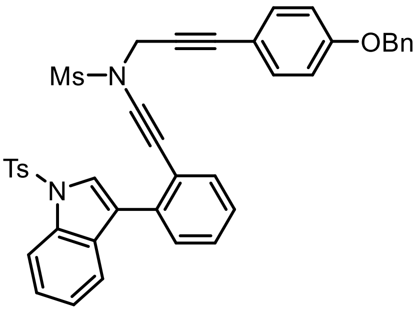

A9

Compound **A9** was prepared in 60% yield (520.8 mg) with four steps according to the general procedure as a white solid (mp 84–86 °C). ^{1}H NMR (400 MHz, $CDCl_3$) δ 7.98

(d, J = 8.4 Hz, 1H), 7.86 (s, 1H), 7.79 (d, J = 8.4 Hz, 2H), 7.63 – 7.55 (m, 2H), 7.46 (d, J = 7.6 Hz, 1H), 7.43 – 7.35 (m, 5H), 7.35 – 7.29 (m, 2H), 7.29 – 7.23 (m, 2H), 7.23 – 7.20 (m, 3H), 7.20 – 7.14 (m, 1H), 6.87 – 6.86 (m, 1H), 6.85 – 6.82 (m, 1H), 5.05 (s, 2H), 4.30 (s, 2H), 2.76 (s, 3H), 2.32 (s, 3H); ^{13}C NMR (100 MHz, $CDCl_3$) δ 159.2, 145.1, 136.5, 135.2, 134.8, 134.4, 133.3, 133.2, 130.0, 129.83, 129.77, 128.6, 128.2, 128.1, 127.5, 127.4, 126.9, 124.9, 124.6, 123.3, 122.2, 121.8, 121.1, 114.9, 114.0, 113.6, 86.9, 84.9, 80.1, 70.7, 70.0, 42.7, 38.4, 21.5; IR (neat): 2928(bs), 2234(s), 1604, 1508, 1447, 1363, 1244, 1166, 748, 582 cm^{-1}; HRESIMS Calcd for $[C_{40}H_{32}N_2NaO_5S_2]^+$ (M + Na$^+$) 707.1645, found 707.1640.

***N*-(3-(4-(methylthio)phenyl)prop-2-yn-1-yl)-*N*-((2-(1-tosyl-1*H*-indol-3-yl)phenyl)ethynyl)methanesulfonamide (A10)**

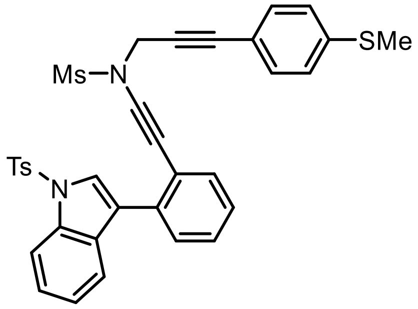

A10

Compound **A10** was prepared in 54% yield (428.6 mg) with four steps according to the general procedure as a white solid (mp 84–85 °C). ^{1}H NMR (400 MHz, $CDCl_3$) δ 7.98 (d, J = 8.0 Hz, 1H), 7.86 (s, 1H), 7.79 (d, J = 8.0 Hz, 2H), 7.64 – 7.56 (m, 2H), 7.47 (d, J = 7.6 Hz, 1H), 7.42 – 7.35 (m, 1H), 7.33 (d, J = 7.6 Hz, 1H), 7.29 (d, J = 8.0 Hz, 1H), 7.26 (d, J = 4.8 Hz, 1H), 7.24 – 7.15 (m, 4H), 7.10 (d, J = 8.0 Hz, 2H), 4.32 (s, 2H), 2.78 (s, 3H), 2.46 (s, 3H), 2.33 (s, 3H); ^{13}C NMR (100 MHz, $CDCl_3$) δ 145.1, 140.3, 135.2, 134.8, 134.4, 133.2, 132.0, 130.0, 129.82, 129.76, 128.3, 127.5, 126.9, 125.7, 124.9, 124.7, 123.3, 122.1, 121.7, 121.1, 117.8, 113.7, 86.7, 84.8, 81.4, 70.8, 42.7, 38.5, 21.5, 15.2; IR (neat): 2926(bs), 2233(s), 1603, 1492, 1446, 1363, 1166, 1131, 749, 582 cm^{-1}; HRESIMS Calcd for $[C_{34}H_{28}N_2NaO_4S_3]^+$ (M + Na$^+$) 647.1103, found 647.1097.

_N_-(3-(benzo[_d_][1,3]dioxol-5-yl)prop-2-yn-1-yl)-_N_-((2-(1-tosyl-1_H_-indol-3-yl)phenyl)ethynyl)methanesulfonamide (A11)

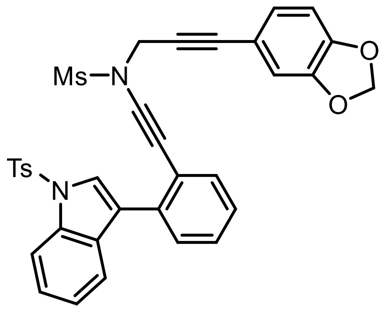

A11

Compound **A11** was prepared in 57% yield (558.2 mg) with four steps according to the general procedure as a white solid (mp 84–86 °C). ^{1}H NMR (400 MHz, $CDCl_3$) δ 7.98 (d, _J_ = 8.4 Hz, 1H), 7.85 (s, 1H), 7.80 (d, _J_ = 8.4 Hz, 2H), 7.63 – 7.52 (m, 2H), 7.50 – 7.44 (m, 1H), 7.41 – 7.35 (m, 1H), 7.35 – 7.31 (m, 1H), 7.31 – 7.26 (m, 1H), 7.22 (d, _J_ = 8.4 Hz, 2H), 7.20 – 7.15 (m, 1H), 6.84 – 6.80 (m, 1H), 6.75 (d, _J_ = 1.6 Hz, 1H), 6.69 (d, _J_ = 8.0 Hz, 1H), 5.96 (s, 2H), 4.29 (s, 2H), 2.76 (s, 3H), 2.33 (s, 3H); ^{13}C NMR (100 MHz, $CDCl_3$) δ 148.4, 147.4, 145.1, 135.1, 134.8, 134.4, 133.1, 130.0, 129.8, 129.7, 128.2, 127.5, 126.9, 126.7, 124.9, 124.6, 123.3, 122.1, 121.7, 121.0, 114.8, 113.6, 111.6, 108.4, 101.4, 86.8, 84.8, 79.7, 70.7, 42.6, 38.4, 21.5; IR (neat): 3064(bs), 2232(s), 1503, 1489, 1445, 1363, 1167, 1131, 1037, 749, 582 cm^{-1}; HRESIMS Calcd for $[C_{34}H_{26}N_2NaO_6S_2]^+$ (M + Na$^+$) 645.1124, found 645.1117.

_N_-(3-(thiophen-2-yl)prop-2-yn-1-yl)-_N_-((2-(1-tosyl-1_H_-indol-3-yl)phenyl)ethynyl) methanesulfonamide (A12)

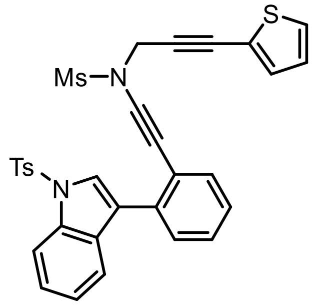

A12

Compound **A12** was prepared in 50% yield (432.7 mg) with four steps according to the general procedure as a white solid (mp 86–88 °C). ^{1}H NMR (400 MHz, $CDCl_3$) δ 7.99 (d, *J* = 8.4 Hz, 1H), 7.84 (s, 1H), 7.80 (d, *J* = 8.4 Hz, 2H), 7.62 – 7.54 (m, 2H), 7.50 – 7.43 (m, 1H), 7.42 – 7.35 (m, 1H), 7.35 – 7.30 (m, 1H), 7.30 – 7.21 (m, 4H), 7.21 – 7.16 (m, 1H), 7.14 (d, *J* = 3.6 Hz, 1H), 7.00 – 6.88 (m, 1H) 4.30 (s, 2H), 2.71 (s, 3H), 2.33 (s, 3H); ^{13}C NMR (100 MHz, $CDCl_3$) δ 145.1, 135.2, 134.8, 134.5, 133.14, 133.05, 130.0, 129.9, 129.8, 128.3, 128.0, 127.5, 127.1, 126.9, 124.8, 124.7, 123.3, 122.2, 121.7, 121.5, 121.1, 113.6, 85.4, 84.7, 80.2, 70.9, 42.7, 38.4, 21.5; IR (neat): 2928 (bs), 2232(s), 1640, 1446, 1363, 1174, 1166, 1130, 1108, 749, 581 cm^{-1}; HRESIMS Calcd for $[C_{31}H_{24}N_2NaO_4S_3]^+$ (M + Na$^+$) 607.0790, found 607.0785.

***N*-(3-(4-(dimethylamino)phenyl)prop-2-yn-1-yl)-*N*-((2-fluoro-6-(1-tosyl-1*H*-indol-3-yl)phenyl)ethynyl)methanesulfonamide (A13)**

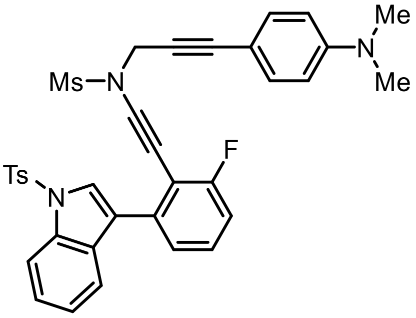

A13

Compound **A13** was prepared in 40% yield (385.9 mg) with four steps according to the general procedure as a white solid (mp 82–84 °C). ^{1}H NMR (400 MHz, $CDCl_3$) δ 8.00 (d, *J* = 8.4 Hz, 1H), 7.90 (s, 1H), 7.81 (d, *J* = 8.4 Hz, 2H), 7.60 (d, *J* = 7.6 Hz, 1H), 7.38 – 7.32 (m, 1H), 7.32 – 7.27 (m, 2H), 7.26 – 7.23 (m, 3H), 7.22 – 7.17 (m, 2H), 7.13 – 7.05 (m, 1H), 6.58 (d, *J* = 8.8 Hz, 2H), 4.33 (s, 2H), 2.96 (s, 6H), 2.84 (s, 3H), 2.33 (s, 3H); ^{13}C NMR (100 MHz, $CDCl_3$) δ 163.6 (d, *J* = 250.9 Hz), 150.5, 145.2, 136.7, 135.1, 134.8, 133.0, 130.0, 129.4, 129.2 (d, *J* = 9.0 Hz), 126.9, 125.33, 125.30, 124.8, 123.5, 121.1 (d, *J* = 3.0 Hz), 121.0, 114.3 (d, *J* = 21.6 Hz), 113.7, 111.7, 110.8 (d, *J* = 16.3 Hz), 108.3, 90.3 (d, *J* = 3.7 Hz), 88.3, 79.0, 64.1, 43.2, 40.1, 38.5, 21.5; ^{19}F NMR (376 MHz, $CDCl_3$) δ -107.73; IR (neat): 2925(bs), 2232(s), 1605, 1519, 1448, 1361, 1175,

1166, 1130, 963, 748 cm^{-1}; HRESIMS Calcd for $[C_{35}H_{30}FN_3NaO_4S_2]^+$ (M + Na$^+$) 662.1554, found 662.1543.

_N_-((5-chloro-2-(1-tosyl-1_H_-indol-3-yl)phenyl)ethynyl)-_N_-(3-(4-(dimethylamino)phenyl)prop-2-yn-1-yl)methanesulfonamide (A14)

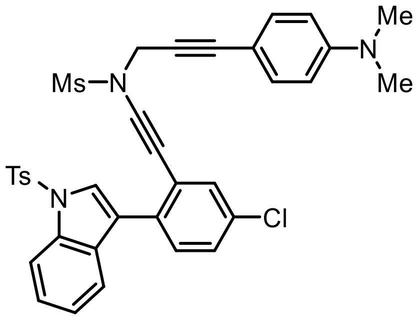

A14

Compound **A14** was prepared in 42% yield (476.8 mg) with four steps according to the general procedure as a white solid (mp 103–105 °C). ^{1}H NMR (400 MHz, $CDCl_3$) δ 7.99 (d, *J* = 8.0 Hz, 1H), 7.85 (s, 1H), 7.80 (d, *J* = 7.6 Hz, 2H), 7.64 – 7.50 (m, 2H), 7.39 (d, *J* = 8.0 Hz, 1H), 7.36 – 7.15 (m, 7H), 6.57 (d, *J* = 8.4 Hz, 2H), 4.28 (s, 2H), 2.96 (s, 6H), 2.76 (s, 3H), 2.33 (s, 3H); ^{13}C NMR (100 MHz, $CDCl_3$) δ 150.5, 145.1, 135.1, 134.8, 133.1, 133.0, 132.8, 132.6, 131.0, 130.0, 129.5, 128.3, 126.9, 125.0, 124.8, 123.5, 123.4, 121.1, 120.9, 113.7, 111.7, 108.1, 88.4, 86.3, 78.9, 69.7, 43.0, 40.1, 38.6, 21.5; IR (neat): 2926(bs), 2233(s), 1607, 1522, 1446, 1363, 1167, 1133, 1091, 815, 589 cm^{-1}; HRESIMS Calcd for $[C_{35}H_{30}ClN_3NaO_4S_2]^+$ (M + Na$^+$) 678.1258 (isotopic ion: 680.1243), found 678.1258 (isotopic ion: 680.1248).

_N_-(3-(4-(dimethylamino)phenyl)prop-2-yn-1-yl)-_N_-((5-methoxy-2-(1-tosyl-1_H_-indol-3-yl)phenyl)ethynyl)methanesulfonamide (A15)

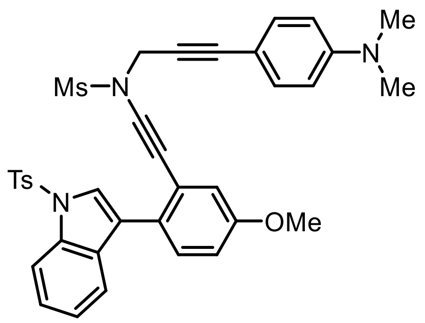

A15

Compound **A15** was prepared in 50% yield (521.3 mg) with four steps according to the general procedure as a white solid (mp 100–103 °C). ^{1}H NMR (400 MHz, $CDCl_3$) δ 7.98 (d, *J* = 8.4 Hz, 1H), 7.87 – 7.71 (m, 3H), 7.57 (d, *J* = 7.6 Hz, 1H), 7.35 (d, *J* = 8.4 Hz, 1H), 7.27 – 7.15 (m, 6H), 7.10 (d, *J* = 2.8 Hz, 1H), 6.98 – 6.87 (m, 1H), 6.56 (d, *J* = 8.8 Hz, 2H), 4.26 (s, 2H), 3.83 (s, 3H), 2.96 (s, 6H), 2.70 (s, 3H), 2.32 (s, 3H); ^{13}C NMR (100 MHz, $CDCl_3$) δ 158.7, 150.5, 145.0, 135.2, 134.8, 133.0, 131.0, 130.1, 129.9, 127.0, 126.9, 124.52, 124.48, 123.3, 122.9, 122.1, 121.2, 117.4, 115.1, 113.6, 111.7, 108.3, 88.2, 85.0, 79.1, 70.6, 55.5, 43.0, 40.1, 38.4, 21.5; IR (neat): 2929(bs), 2237(s), 1607, 1522, 1446, 1362, 1166, 1131, 1090, 665, 571 cm^{-1}; HRESIMS Calcd for $[C_{36}H_{33}N_3NaO_5S_2]^+$ (M + Na$^+$) 674.1754, found 674.1741.

***N*-((4-chloro-2-(1-tosyl-1*H*-indol-3-yl)phenyl)ethynyl)-*N*-(3-(4-(dimethylamino)phenyl)prop-2-yn-1-yl)methanesulfonamide (A16)**

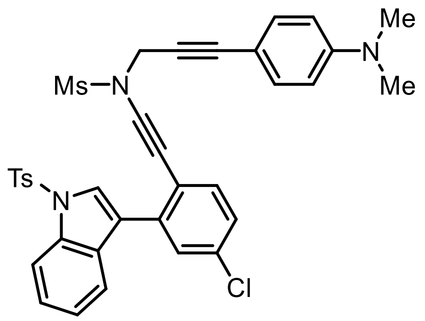

A16

Compound **A16** was prepared in 60% yield (538.9 mg) with four steps according to the general procedure as a white solid (mp 105–106 °C). ^{1}H NMR (400 MHz, $CDCl_3$) δ 8.00 (d, *J* = 8.0 Hz, 1H), 7.88 (s, 1H), 7.80 (d, *J* = 7.6 Hz, 2H), 7.58 (d, *J* = 7.6 Hz, 1H),

7.51 (d, *J* = 8.0 Hz, 1H), 7.46 (s, 1H), δ 7.33 – 7.27 (m, 2H), 7.26 – 7.16 (m, 5H), 6.56 (d, *J* = 8.0 Hz, 2H), 4.29 (s, 2H), 2.96 (s, 6H), 2.76 (s, 3H), 2.32 (s, 3H); ^{13}C NMR (100 MHz, $CDCl_3$) δ 150.5, 145.2, 136.0, 135.1 134.8, 134.3, 133.9, 132.9, 130.0, 129.7, 129.3, 127.6, 126.9, 125.2, 124.8, 123.5, 121.0, 120.9, 120.4, 113.7, 111.7, 108.1, 88.3, 86.0, 79.0, 69.7, 43.0, 40.1, 38.6, 21.5; IR (neat): 2926 (bs), 2233(s), 1608, 1522, 1446, 1363, 1166, 1131, 1104, 946, 581 cm^{-1}; HRESIMS Calcd for $[C_{35}H_{30}ClN_3NaO_4S_2]^+$ (M + Na^+) 678.1258 (isotopic ion: 680.1243), found 678.1262 (isotopic ion: 680.1245).

***N*-(3-(4-(dimethylamino)phenyl)prop-2-yn-1-yl)-*N*-((4-methyl-2-(1-tosyl-1*H*-indol -3-yl)phenyl)ethynyl)methanesulfonamide (A17)**

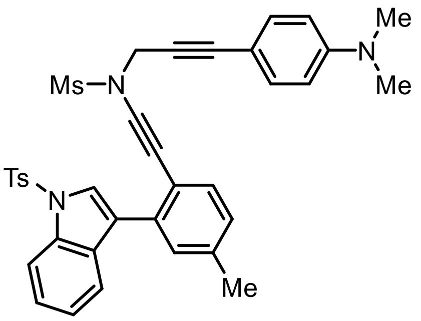

A17

Compound **A17** was prepared in 33% yield (547.6 mg) with four steps according to the general procedure as a white solid (mp 96–98 °C). ^{1}H NMR (400 MHz, $CDCl_3$) δ 7.98 (d, *J* = 8.0 Hz, 1H), 7.83 (s, 1H), 7.79 (d, *J* = 8.0 Hz, 2H), 7.60 (d, *J* = 7.6 Hz, 1H), 7.49 (d, *J* = 7.6 Hz, 1H), 7.30 – 7.12 (m, 8H), 6.57 (d, *J* = 8.4 Hz, 2H), 4.27 (s, 2H), 2.96 (s, 6H), 2.69 (s, 3H), 2.39 (s, 3H), 2.32 (s, 3H); ^{13}C NMR (100 MHz, $CDCl_3$) δ 150.5, 145.0, 138.4, 135.2, 134.9, 134.5, 133.3, 133.0, 130.6, 130.0, 129.8, 128.3, 126.9, 124.8, 124.6, 123.3, 122.5, 121.3, 118.8, 113.6, 111.7, 108.4, 88.1, 84.4, 79.2, 70.5, 43.1, 40.1, 38.3, 21.4; IR (neat): 2924(bs), 2214(s), 1607, 1522, 1446, 1164, 1130, 1093, 671, 583 cm^{-1}; HRESIMS Calcd for $[C_{36}H_{33}N_3NaO_4S_2]^+$ (M + Na^+) 658.1805, found 658.1794.

***N*-(3-(4-(dimethylamino)phenyl)prop-2-yn-1-yl)-*N*-((2-(5-fluoro-1-tosyl-1*H*-indol-3-yl)phenyl)ethynyl)methanesulfonamide (A18)**

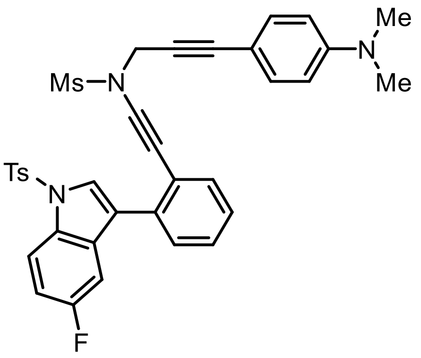

A18

Compound **A18** was prepared in 50% yield (503.3 mg) with four steps according to the general procedure as a white solid (mp 96–98 °C). ^{1}H NMR (400 MHz, $CDCl_3$) δ 7.99 – 7.92 (m, 1H), 7.91 (s, 1H), 7.78 (d, *J* = 8.4Hz, 2H), 7.60 (d, *J* = 6.8 Hz, 1H), 7.43 (d, *J* = 7.2 Hz, 1H), 7.40 – 7.36 (m, 1H), 7.35 – 7.30 (m, 1H), 7.26 – 7.21 (m, 3H), 7.18 (d, *J* = 8.8 Hz, 2H), 7.06 – 6.98 (m, 1H), 6.55 (d, *J* = 8.8 Hz, 2H), 4.31 (s, 2H), 2.96 (s, 6H), 2.96 (s, 3H), 2.34 (s, 3H); ^{13}C NMR (100 MHz, $CDCl_3$) δ 159.7 (d, *J* = 240.5 Hz), 150.4, 145.2, 135.0, 133.8, 133.3, 132.9, 131.2, 130.9 (d, *J* = 9.8 Hz), 130.0, 129.5, 128.2, 127.6, 126.9, 126.6, 121.9 (d, *J* = 4.1 Hz), 121.7, 114.7 (d, *J* = 9.3 Hz), 112.7 (d, *J* = 25.7 Hz), 111.6, 108.2, 106.5 (d, *J* = 24.5 Hz), 88.3, 85.3, 78.9, 70.4, 43.1, 40.1, 38.7, 21.6; ^{19}F NMR (376 MHz, $CDCl_3$) δ -117.10. IR (neat): 2926(bs), 2232(s), 1608, 1522, 1463, 1363, 1192, 1167, 1128, 1092, 589 cm^{-1}; HRESIMS Calcd for $[C_{35}H_{30}FN_3NaO_4S_2]^+$ (M + Na^+) 662.1554, found 662.1545.

***N*-((2-(5-chloro-1-tosyl-1*H*-indol-3-yl)phenyl)ethynyl)-*N*-(3-(4-(dimethylamino)phenyl)prop-2-yn-1-yl)methanesulfonamide (A19)**

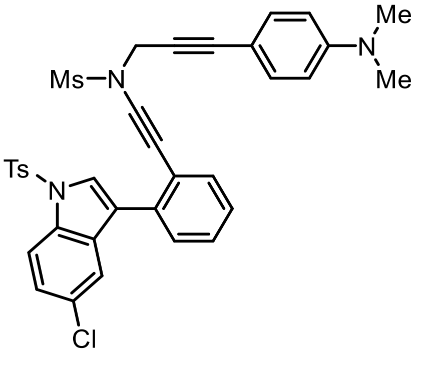

A19

Compound **A19** was prepared in 67% yield (573.1 mg) with four steps according to the general procedure as a white solid (mp 99–101 °C). ^{1}H NMR (400 MHz, $CDCl_3$) δ 8.00 – 7.87 (m, 2H), 7.79 (d, *J* = 8.0 Hz, 2H), 7.61 (d, *J* = 7.2 Hz, 1H), 7.56 (s, 1H), 7.44 (d, *J* = 7.2 Hz, 1H), 7.41 – 7.35 (m, 1H), 7.35 – 7.30 (m, 1H), 7.26 – 7.20 (m, 3H), 7.14 (d, *J* = 8.8 Hz, 2H), 6.54 (d, *J* = 8.8 Hz, 2H), 4.36 (s, 2H), 2.99 (s, 3H), 2.96 (s, 6H), 2.34 (s, 3H); ^{13}C NMR (100 MHz, $CDCl_3$) δ 150.4, 145.3, 135.0, 133.6, 133.4, 133.3, 132.9, 131.1, 130.0, 129.6, 129.3, 128.2, 127.6, 126.9, 126.2, 124.9, 121.8, 121.4, 120.4, 114.8, 111.6, 108.2, 88.3, 85.4, 78.8, 70.4, 43.2, 40.1, 38.7, 21.5; IR (neat): 2926(bs), 2233(s), 1608, 1522, 1442, 1363, 1166, 1116, 1091, 803, 586 cm^{-1}; HRESIMS Calcd for $[C_{35}H_{30}ClN_3NaO_4S_2]^+$ (M + Na$^+$) 678.1257 (isotopic ion: 680.1243), found 678.1251 (isotopic ion: 680.1234).

***N*-((2-(5-bromo-1-tosyl-1*H*-indol-3-yl)phenyl)ethynyl)-*N*-(3-(4-(dimethylamino)phenyl)prop-2-yn-1-yl)methanesulfonamide (A20)**

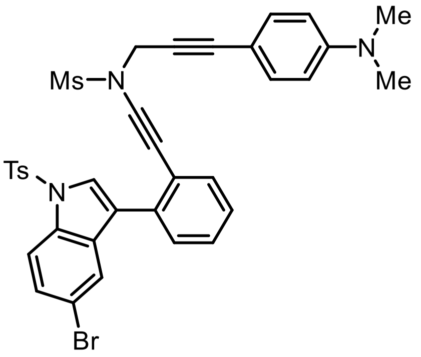

A20

Compound **A20** was prepared in 40% yield (701.0 mg) with four steps according to the general procedure as a white solid (mp 142–145 °C). ^{1}H NMR (400 MHz, $CDCl_3$) δ 7.95 – 7.84 (m, 2H), 7.78 (d, *J* = 8.4 Hz, 2H), 7.71 (d, *J* = 0.9 Hz, 1H), 7.61 (d, *J* = 7.6 Hz, 1H), 7.43 (d, *J* = 7.2 Hz, 1H), 7.41 – 7.36 (m, 2H), 7.36 – 7.30 (m, 1H), 7.23 (d, *J* = 8.0 Hz, 2H), 7.13 (d, *J* = 8.8 Hz, 2H), 6.54 (d, *J* = 8.8 Hz, 2H), 4.38 (s, 2H), 3.00 (s, 3H), 2.97 (s, 6H), 2.34 (s, 3H); ^{13}C NMR (100 MHz, $CDCl_3$) δ 150.4, 145.3, 135.0, 133.63, 133.55, 133.4, 132.9, 131.5, 130.0, 129.6, 128.2, 127.62, 127.56, 126.9, 126.1, 123.5, 121.8, 121.2, 117.0, 115.2, 111.6, 108.2, 88.3, 85.4, 78.8, 70.4, 43.2, 40.1, 38.7,

21.5; IR (neat): 2925(bs), 2232(s), 1608, 1522, 1445, 1362, 1167, 1134, 677, 589 cm^{-1}; HRESIMS Calcd for $[C_{35}H_{30}BrN_3NaO_4S_2]^+$ (M + Na$^+$) 722.0753 (isotopic ion: 724.0737), found 722.0749 (isotopic ion: 724.0731).

***N*-(3-(4-(dimethylamino)phenyl)prop-2-yn-1-yl)-*N*-((2-(5-methyl-1-tosyl-1*H*-indol-3-yl)phenyl)ethynyl)methanesulfonamide (A21)**

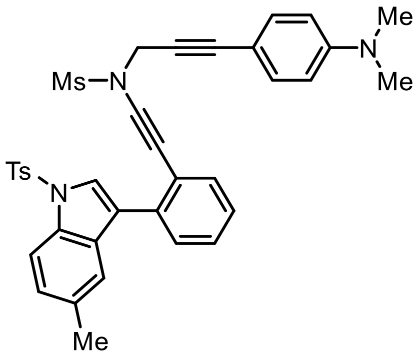

A21

Compound **A21** was prepared in 44% yield (516.6 mg) with four steps according to the general procedure as a white solid (mp 139–141 °C). ^{1}H NMR (400 MHz, $CDCl_3$) δ 7.88 (d, *J* = 8.4 Hz, 1H), 7.81 (s, 1H), 7.78 (d, *J* = 8.4 Hz, 2H), 7.60 (d, *J* = 7.6 Hz, 1H), 7.46 (d, *J* = 7.2 Hz, 1H), 7.39 (d, *J* = 6.4 Hz, 1H), 7.36 (s, 1H), 7.34 – 7.26 (m, 1H), 7.23 – 7.15 (m, 4H), 7.12 (d, *J* = 8.4 Hz, 1H), 6.56 (d, *J* = 8.8 Hz, 2H), 4.27 (s, 2H), 2.96 (s, 6H), 2.83 (s, 3H), 2.37 (s, 3H), 2.32 (s, 3H); ^{13}C NMR (100 MHz, $CDCl_3$) δ 150.4, 144.9, 135.2, 134.6, 133.3, 133.1, 133.0, 130.1, 129.9, 129.8, 128.1, 127.4, 126.9, 126.1, 125.0, 122.1, 121.9, 120.8, 113.4, 111.6, 108.3, 88.1, 85.1, 79.0, 70.5, 43.1, 40.1, 38.5, 21.5, 21.4; IR (neat): 2926(bs), 2232(s), 1635, 1608, 1522, 1361, 1134, 766, 676, 589 cm^{-1}; HRESIMS Calcd for $[C_{36}H_{33}N_3NaO_4S_2]^+$ (M + Na$^+$) 658.1805, found 658.1798.

***N*-(3-(4-(dimethylamino)phenyl)prop-2-yn-1-yl)-*N*-((2-(6-fluoro-1-tosyl-1*H*-indol-3-yl)phenyl)ethynyl)methanesulfonamide (A22)**

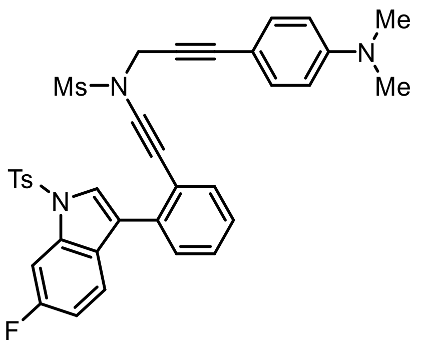

A22

Compound **A22** was prepared in 40% yield (523.0 mg) with four steps according to the general procedure as a white solid (mp 91–93 °C). ^{1}H NMR (400 MHz, $CDCl_3$) δ 7.83 (s, 1H), 7.80 (d, *J* = 8.4 Hz, 2H), 7.74 – 7.68 (m, 1H), 7.62 – 7.57 (m, 1H), 7.53 – 7.48 (m, 1H), 7.45 – 7.41 (m, 1H), 7.40 – 7.35 (m, 1H), 7.35 – 7.29 (m, 1H), 7.24 (d, *J* = 9.6 Hz, 2H), 7.18 (d, *J* = 8.8 Hz, 2H), 6.97 – 6.89 (m, 1H), 6.56 (d, *J* = 8.8 Hz, 2H), 4.32 (s, 2H), 2.96 (s, 6H), 2.83 (s, 3H), 2.34 (s, 3H); ^{13}C NMR (100 MHz, $CDCl_3$) δ 160.8 (d, *J* = 242.5 Hz), 150.5, 145.3, 135.0, 134.9, 134.0, 133.3, 132.9, 130.1, 129.7, 128.2, 127.6, 126.9, 126.1, 125.0 (d, *J* = 3.5 Hz), 122.0 (d, *J* = 5.5 Hz), 121.9 (d, *J* = 15.0 Hz), 111.9, 111.67, 111.65, 108.1, 100.9 (d, *J* = 28.5 Hz), 88.2, 85.2, 79.0, 70.4, 43.1, 40.1, 38.5, 21.6; ^{19}F NMR (376 MHz, $CDCl_3$) δ -116.21; IR (neat): 2926 (bs), 2232(s), 1608, 1522, 1362, 1166, 1105, 965, 675, 595 cm^{-1}; HRESIMS Calcd for $[C_{35}H_{30}FN_3NaO_4S_2]^+$ (M + Na^+) 662.1554, found 662.1547.

***N*-((2-(6-chloro-1-tosyl-1*H*-indol-3-yl)phenyl)ethynyl)-*N*-(3-(4-(dimethylamino)phenyl)prop-2-yn-1-yl)methanesulfonamide (A23)**

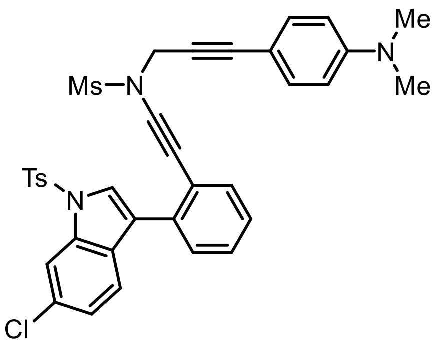

A23

Compound **A23** was prepared in 32% yield (468.5 mg) with four steps according to the general procedure as a white solid (mp 90–92 °C). ^{1}H NMR (400 MHz, $CDCl_3$) δ 8.01 (d, *J* = 1.2 Hz, 1H), 7.85 (s, 1H), 7.80 (d, *J* = 8.0 Hz, 2H), 7.60 (d, *J* = 7.2 Hz, 1H), 7.50 (d, *J* = 8.4 Hz, 1H), 7.42 (d, *J* = 7.2 Hz, 1H), 7.40 – 7.35 (m, 1H), 7.35 – 7.30 (m, 1H), 7.25 (d, *J* = 7.2 Hz, 2H), 7.15 (d, *J* = 8.8 Hz, 3H), 6.55 (d, *J* = 8.8 Hz, 2H), 4.34 (s, 2H), 2.97 (s, 6H), 2.84 (s, 3H), 2.35 (s, 3H); ^{13}C NMR (100 MHz, $CDCl_3$) δ 150.5, 145.4, 135.2, 135.0, 133.8, 133.3, 132.9, 130.6, 130.1, 129.7, 128.3, 128.2, 127.6, 126.9, 125.4, 124.0, 121.9, 121.84, 121.81, 113.8, 111.7, 108.1, 88.3, 85.3, 78.9, 70.4, 43.1, 40.1, 38.5, 21.6; IR (neat): 2928(bs), 2233(s), 1636, 1608, 1522, 1362, 1165, 1139, 671, 593 cm^{-1}; HRESIMS Calcd for $[C_{35}H_{30}ClN_3NaO_4S_2]^+$ (M + Na^+) 678.1258 (isotopic ion: 680.1243), found 678.1258 (isotopic ion: 680.1242).

***N*-((2-(6-bromo-1-tosyl-1*H*-indol-3-yl)phenyl)ethynyl)-*N*-(3-(4-(dimethylamino)phenyl)prop-2-yn-1-yl)methanesulfonamide (A24)**

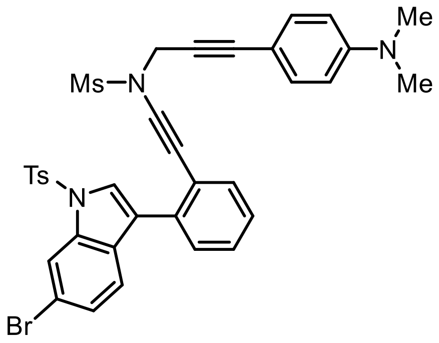

A24

Compound **A24** was prepared in 30% yield (510.0 mg) with four steps according to the general procedure as a white solid (mp 117–119 °C). ^{1}H NMR (400 MHz, $CDCl_3$) δ 8.17 (d, *J* = 1.6 Hz, 1H), 7.83 (s, 1H), 7.80 (d, *J* = 8.4 Hz, 2H), 7.65 – 7.56 (m, 1H), 7.45 (d, *J* = 8.4 Hz, 1H), 7.43 – 7.39 (m, 1H), 7.39 – 7.35 (m, 1H), 7.35 – 7.31 (m, 1H), 7.30 – 7.27 (m, 1H), 7.26 (d, *J* = 2.4 Hz, 1H), 7.24 (s, 1H), 7.15 (d, *J* = 8.8 Hz, 2H), 6.55 (d, *J* = 8.8 Hz, 2H), 4.35 (s, 2H), 2.97 (s, 6H), 2.83 (s, 3H), 2.35 (s, 3H); ^{13}C NMR (100 MHz, $CDCl_3$) δ 150.5, 145.4, 135.5, 134.9, 133.7, 133.3, 132.9, 130.1, 129.7, 128.6, 128.2, 127.6, 126.9, 126.7, 125.3, 122.2, 121.9, 121.8, 118.3, 116.6, 111.7, 108.1,

88.3, 85.3, 78.9, 70.4, 43.1, 40.1, 38.5, 21.6; IR (neat): 2926(bs), 2233(s), 1608, 1522, 1363, 1166, 1135, 936, 814, 591 cm^{-1}; HRESIMS Calcd for $[C_{35}H_{30}BrN_3NaO_4S_2]^+$ (M + Na^+) 722.0753 (isotopic ion: 724.0737), found 722.0746 (isotopic ion: 724.0724).

***N*-(3-(4-(dimethylamino)phenyl)prop-2-yn-1-yl)-*N*-((2-(6-methyl-1-tosyl-1*H*-indol-3-yl)phenyl)ethynyl)methanesulfonamide (A25)**

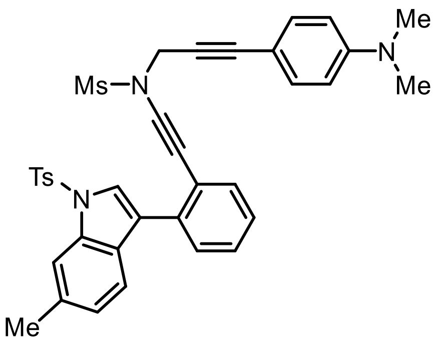

A25

Compound **A25** was prepared in 52% yield (613.5 mg) with four steps according to the general procedure as a white solid (mp 96–98 °C). ^{1}H NMR (400 MHz, $CDCl_3$) δ 7.96 – 7.71 (m, 4H), 7.59 (d, *J* = 7.2 Hz, 1H), 7.52 – 7.41 (m, 2H), 7.39 – 7.34 (m, 1H), 7.33 – 7.28 (m, 1H), 7.27 – 7.16 (m, 4H), 7.01 (d, *J* = 8.0 Hz, 1H), 6.56 (d, *J* = 8.4 Hz, 2H), 4.30 (s, 2H), 2.96 (s, 6H), 2.75 (s, 3H), 2.42 (s, 3H), 2.33 (s, 3H); ^{13}C NMR (100 MHz, $CDCl_3$) δ 150.4, 144.9, 135.3, 135.3, 134.8, 134.6, 133.2, 133.0, 130.0, 129.8, 128.1, 127.5, 127.3, 126.9, 124.9, 124.3, 122.2, 121.8, 120.7, 113.6, 111.7, 108.3, 88.1, 85.1, 79.1, 70.6, 43.1, 40.1, 38.4, 21.8, 21.5; IR (neat): 2924(bs), 2233(s), 1607, 1522, 1362, 1165, 1110, 814, 768, 581 cm^{-1}; HRESIMS Calcd for $[C_{36}H_{33}N_3NaO_4S_2]^+$ (M + Na^+) 658.1805, found 658.1797.

***N*-(3-(4-(dimethylamino)phenyl)prop-2-yn-1-yl)-*N*-((2-(1-(phenylsulfonyl)-1*H*-indol-3-yl)phenyl)ethynyl)methanesulfonamide (A26)**

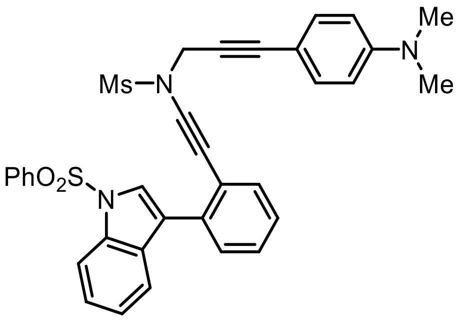

A26

Compound **A26** was prepared in 50% yield (589.3 mg) with four steps according to the general procedure as a white solid (mp 87–189 °C). ^{1}H NMR (400 MHz, $CDCl_3$) δ 8.00 (d, *J* = 8.4 Hz, 1H), 7.96 – 7.88 (m, 2H), 7.86 (s, 1H), 7.60 (d, *J* = 8.0 Hz, 2H), 7.54 – 7.49 (m, 1H), 7.49 – 7.40 (m, 3H), 7.40 – 7.35 (m, 1H), 7.35 – 7.30 (m, 1H), 7.29 – 7.26 (m, 1H), 7.24 – 7.21 (m, 1H), 7.21 – 7.16 (m, 2H), 6.57 (d, *J* = 8.8 Hz, 2H), 4.27 (s, 2H), 2.96 (s, 6H), 2.70 (s, 3H).; ^{13}C NMR (100 MHz, $CDCl_3$) δ 150.5, 138.1, 134.9, 134.4, 133.9, 133.2, 133.0, 129.9, 129.8, 129.4, 128.2, 127.5, 126.9, 124.8, 124.7, 123.5, 122.5, 121.9, 121.3, 113.6, 111.7, 108.3, 88.2, 85.2, 79.1, 70.5, 43.1, 40.1, 38.4; IR (neat): 2927(bs), 2233(s), 1607, 1522, 1447, 1166, 1130, 1013, 749, 591 cm^{-1}; HRESIMS Calcd for $[C_{34}H_{29}N_3NaO_4S_2]^+$ (M + Na^+) 630.1492, found 630.1482.

***N*-(3-(4-(dimethylamino)phenyl)prop-2-yn-1-yl)-*N*-((2-(1-(methylsulfonyl)-1*H*-indol-3-yl)phenyl)ethynyl)methanesulfonamide (A27)**

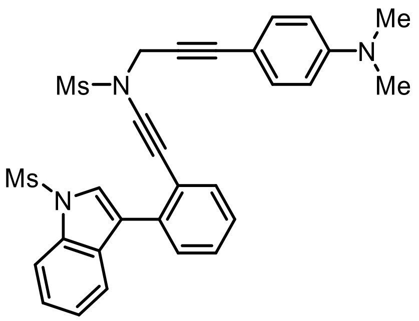

A27

Compound **A27** was prepared in 18% yield (217.8 mg) with four steps according to the general procedure as a white solid (mp 88–91 °C). ^{1}H NMR (400 MHz, $CDCl_3$) δ 7.98 (d, *J* = 8.4 Hz, 1H), 7.77 (s, 1H), 7.71 (d, *J* = 7.6 Hz, 1H), 7.67 – 7.62 (m, 1H), 7.56 (d,

J = 7.6 Hz, 1H), 7.46 – 7.43 (m, 1H), 7.43 – 7.40 (m, 1H), 7.40 – 7.37 (m, 1H), 7.36 – 7.32 (m, 1H), 7.31 – 7.27 (m, 1H), 7.23 (d, *J* = 8.8 Hz, 2H), 6.59 (d, *J* = 8.8 Hz, 2H), 4.42 (s, 2H), 3.20 (s, 3H), 3.00 (s, 6H), 2.95 (s, 3H); ^{13}C NMR (100 MHz, $CDCl_3$) δ 150.4, 134.9, 133.9, 132.9, 132.7, 129.8, 129.7, 128.0, 127.4, 124.8, 124.7, 123.4, 121.8, 121.6, 121.1, 113.2, 111.6, 107.9, 88.2, 85.0, 79.0, 70.7, 43.1, 40.7, 40.0, 38.7; IR (neat): 2927(bs), 2233(s), 1607, 1522, 1361, 1165, 1131, 962, 767, 748 cm^{-1}; HRESIMS Calcd for $[C_{29}H_{27}N_3NaO_4S_2]^+$ (M + Na$^+$) 568.1335, found 568.1327.

tert-butyl 3-(2-((*N*-(3-(4-(dimethylamino)phenyl)prop-2-yn-1-yl)methylsulfonamido)ethynyl)phenyl)-1*H*-indole-1-carboxylate (A28)

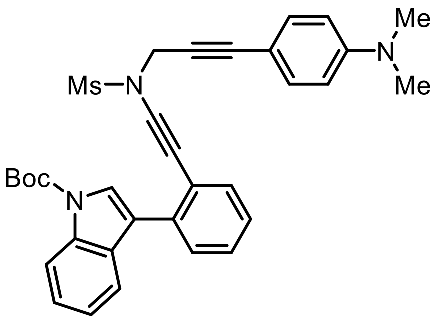

A28

Compound **A28** was prepared in 28% yield (513.8 mg) with four steps according to the general procedure as a white solid (mp 90–92 °C). ^{1}H NMR (400 MHz, $CDCl_3$) δ 8.18 (d, *J* = 8.0 Hz, 1H), 7.85 (s, 1H), 7.64 (d, *J* = 7.6 Hz, 1H), 7.59 (d, *J* = 7.6 Hz, 1H), 7.52 (d, *J* = 7.2 Hz, 1H), 7.40 – 7.35 (m, 1H), 7.34 – 7.27 (m, 2H), 7.24 – 7.20 (m, 1H), 7.11 (d, *J* = 8.8 Hz, 2H), 6.52 (d, *J* = 8.8 Hz, 2H), 4.32 (s, 2H), 2.94 (s, 6H), 2.85 (s, 3H), 1.65 (s, 9H); ^{13}C NMR (100 MHz, $CDCl_3$) δ 150.4, 149.7, 135.3, 135.1, 132.9, 132.6, 129.9, 129.5, 127.9, 127.1, 124.8, 124.3, 122.6, 121.9, 120.5, 120.4, 115.3, 111.6, 108.2, 88.0, 84.9, 83.8, 79.0, 70.9, 43.1, 40.1, 38.5, 28.1; IR (neat): 2930(bs), 2233(s), 1732, 1607, 1522, 1451, 1368, 1237, 1166, 766 cm^{-1}; HRESIMS Calcd for $[C_{33}H_{33}N_3NaO_4S]^+$ (M + Na$^+$) 590.2084, found 590.2076.

***N*-((2-(benzofuran-3-yl)phenyl)ethynyl)-*N*-(3-(4-(dimethylamino)phenyl)prop-2-yn-1-yl)methanesulfonamide (A29)**

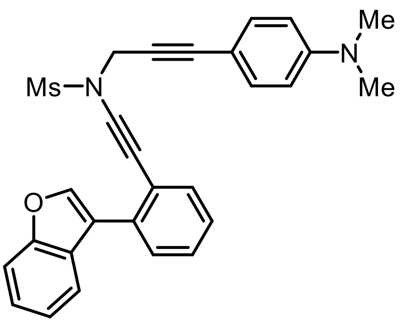

A29

Compound **A29** was prepared in 30% yield (413.2 mg) with four steps according to the general procedure as a white solid (mp 86–89 °C). ^{1}H NMR (400 MHz, $CDCl_3$) δ 8.14 (s, 1H), 7.76 (d, *J* = 7.6 Hz, 1H), 7.66 (d, *J* = 7.6 Hz, 1H), 7.63 (d, *J* = 7.6 Hz, 1H), 7.57 (d, *J* = 8.0 Hz, 1H), 7.47 – 7.42 (m, 1H), 7.40 – 7.33 (m, 2H), 7.33 – 7.27 (m, 3H), 6.62 (d, *J* = 8.8 Hz, 2H), 4.44 (s, 2H), 3.00 (s, 3H), 2.99 (s, 6H); ^{13}C NMR (100 MHz, $CDCl_3$) δ 155.0, 150.4, 143.6, 133.1, 132.9, 132.8, 129.2, 128.1, 127.2, 126.9, 124.2, 122.7, 121.4, 120.7, 120.1, 111.6, 111.5, 107.9, 88.2, 85.0, 79.1, 70.7, 43.0, 40.0, 38.5; IR (neat): 2926(bs), 2233(s), 1607, 1522, 1452, 1363, 1166, 1130, 966, 750 cm^{-1}; HRESIMS Calcd for $[C_{28}H_{24}N_2NaO_3S]^+$ (M + Na$^+$) 491.1400, found 491.1397.

***N*-((2-(benzo[*b*]thiophen-3-yl)phenyl)ethynyl)-*N*-(3-(4-(dimethylamino)phenyl)prop-2-yn-1-yl)methanesulfonamide (A30)**

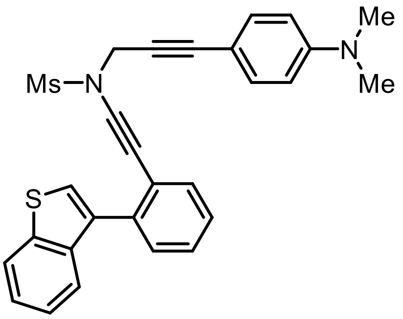

A30

Compound **A30** was prepared in 60% yield (339.4 mg) with four steps according to the general procedure as a white solid (mp 88–90 °C). ^{1}H NMR (400 MHz, $CDCl_3$) δ 7.91 – 7.82 (m, 1H), 7.74 – 7.67 (m, 1H), 7.64 – 7.54 (m, 2H), 7.46 (d, *J* = 7.2 Hz, 1H), 7.42 – 7.38 (m, 1H), 7.38 – 7.35 (m, 1H), 7.34 – 7.29 (m, 2H), 7.21 (d, *J* = 8.8 Hz, 2H), 6.58

(d, J = 8.8 Hz, 2H), 4.25 (s, 2H), 2.97 (s, 6H), 2.74 (s, 3H); ^{13}C NMR (100 MHz, $CDCl_3$) δ 150.5, 139.9, 138.4, 137.5, 136.0, 133.0, 132.4, 130.0, 127.9, 127.5, 125.5, 124.18, 124.15, 123.4, 122.6, 122.3, 111.6, 108.2, 88.0, 85.0, 79.2, 70.6, 43.1, 40.1, 38.3; IR (neat): 2926(bs), 2234(s), 1607, 1522, 1362, 1165, 1128, 965, 765, 734 cm^{-1}; HRESIMS Calcd for $[C_{28}H_{24}N_2NaO_2S_2]^+$ $(M + Na^+)$ 507.1171, found 507.1160.

***N*-(3-(4-(dimethylamino)phenyl)prop-2-yn-1-yl)-*N*-((2-(thiophen-3-yl)phenyl)ethynyl)methanesulfonamide (A31)**

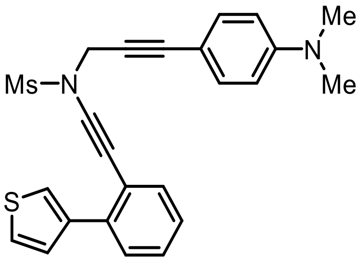

A31

Compound **A31** was prepared in 70% yield (313.9 mg) with four steps according to the general procedure as a white solid (mp 84–86 °C). ^{1}H NMR (400 MHz, $CDCl_3$) δ 7.68 (d, J = 2.0 Hz, 1H), 7.54 (d, J = 7.6 Hz, 1H), 7.47 (d, J = 5.2 Hz, 1H), 7.45 (d, J = 7.6 Hz, 1H), 7.38 – 7.32 (m, 1H), 7.30 (d, J = 8.8 Hz, 2H), 7.27 – 7.22 (m, 2H), 6.60 (d, J = 8.8 Hz, 2H), 4.54 (s, 2H), 3.09 (s, 3H), 2.97 (s, 6H); ^{13}C NMR (100 MHz, $CDCl_3$) δ 150.6, 140.8, 138.0, 133.10, 133.07, 128.9, 128.6, 128.3, 126.9, 124.8, 123.7, 120.4, 111.7, 108.1, 88.3, 84.7, 79.5, 71.2, 43.3, 40.1, 38.7; IR (neat): 2925 (bs), 2232(s), 1606, 1521, 1360, 1164, 1128, 1075, 965, 796, 759 cm^{-1}; HRESIMS Calcd for $[C_{24}H_{22}N_2NaO_2S_2]^+$ $(M + Na^+)$ 457.1015, found 457.1015.

***N*-((2-(benzofuran-2-yl)phenyl)ethynyl)-*N*-(3-(4-(dimethylamino)phenyl)prop-2-yn-1-yl)methanesulfonamide (A32)**

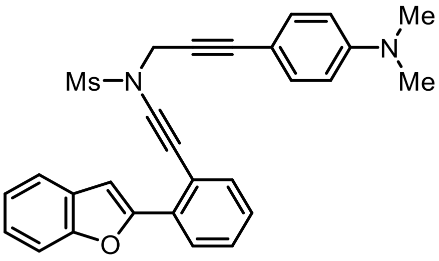

A32

Compound **A32** was prepared in 60% yield (331.6 mg) with four steps according to the general procedure as a white solid (mp 138–141 °C). ^{1}H NMR (400 MHz, $CDCl_3$) δ 8.00 (d, *J* = 7.6 Hz, 1H), 7.82 (s, 1H), 7.61 – 7.55 (m, 1H), 7.50 (d, *J* = 8.4 Hz, 1H), 7.47 (d, *J* = 7.6 Hz, 1H), 7.42 – 7.37 (m, 1H), 7.30 – 7.23 (m, 4H), 7.19 – 7.11 (m, 1H), 6.55 (d, *J* = 8.8 Hz, 2H), 4.66 (s, 2H), 3.24 (s, 3H), 2.93 (s, 6H); ^{13}C NMR (100 MHz, $CDCl_3$) δ 154.3, 153.7, 150.6, 133.5, 133.0, 130.9, 129.3, 128.1, 127.7, 126.6, 124.6, 122.8, 121.5, 119.1, 111.7, 110.9, 107.9, 105.9, 88.7, 86.5, 79.3, 71.4, 43.4, 40.0, 39.0; IR (neat): 2926(bs), 2228(s), 1634, 1607, 1522, 1362, 1165, 1125, 846, 751 cm^{-1}; HRESIMS Calcd for $[C_{28}H_{24}N_2NaO_3S]^+$ (M + Na$^+$) 491.1400, found 491.1396.

***N*-((2-(benzo[*b*]thiophen-2-yl)phenyl)ethynyl)-*N*-(3-(4-(dimethylamino)phenyl)prop-2-yn-1-yl)methanesulfonamide (A33)**

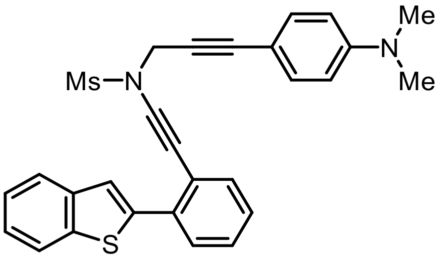

A33

Compound **A33** was prepared in 60% yield (433.9 mg) with four steps according to the general procedure as a white solid (mp 92–94 °C). ^{1}H NMR (400 MHz, $CDCl_3$) δ 7.98 (s, 1H), 7.79 (d, *J* = 6.0 Hz, 1H), 7.74 (d, *J* = 5.2 Hz, 1H), 7.61 (d, *J* = 6.8 Hz, 1H), 7.57 (d, *J* = 5.6 Hz, 1H), 7.39 – 7.09 (m, 6H), 6.55 (d, *J* = 6.8 Hz, 2H), 4.57 (s, 2H), 3.07 (s, 3H), 2.94 (s, 6H); ^{13}C NMR (100 MHz, $CDCl_3$) δ 150.5, 141.9, 140.4, 139.8, 135.2, 133.3, 133.0, 129.5, 128.2, 127.6, 124.4, 124.1, 123.7, 121.9, 120.6, 111.7, 108.1, 88.5,

86.0, 79.4, 71.3, 43.3, 40.1, 38.8; IR (neat): 2926(bs), 2229(s), 1605, 1520, 1364, 1164, 1130, 964, 795, 758 cm^{-1}; HRESIMS Calcd for $[C_{28}H_{24}N_2NaO_2S_2]^+$ (M + Na^+) 507.1171, found 507.1165.

_N_-(3-(4-(dimethylamino)phenyl)prop-2-yn-1-yl)-_N_-((2-(thiophen-2-yl)phenyl)ethynyl)methanesulfonamide (A34)

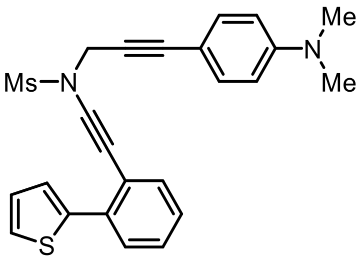

A34

Compound **A34** was prepared in 80% yield (354.9 mg) with four steps according to the general procedure as a white solid (mp 92–94 °C). ^{1}H NMR (400 MHz, $CDCl_3$) δ 7.62 (d, J = 3.2 Hz, 1H), 7.58 – 7.47 (m, 2H), 7.34 – 7.29 (m, 2H), 7.26 (d, J = 10.0 Hz, 2H), 7.23 (d, J = 7.6 Hz, 1H), 7.05 – 6.93 (m, 1H), 6.60 (d, J = 8.8 Hz, 2H), 4.57 (s, 2H), 3.15 (s, 3H), 2.97 (s, 6H); ^{13}C NMR (100 MHz, $CDCl_3$) δ 150.6, 142.0, 135.8, 133.6, 133.1, 129.0, 128.3, 127.4, 127.1, 126.9, 125.7, 120.1, 111.7, 108.2, 88.3, 85.6, 79.6, 71.1, 43.3, 40.1, 39.0; IR (neat): 2926(bs), 2229(s), 1604, 1519, 1366, 1164, 1130, 964, 795, 760 cm^{-1}; HRESIMS Calcd for $[C_{24}H_{22}N_2NaO_2S_2]^+$ (M + Na^+) 457.1015, found 457.1009.

_N_-(3-(4-(dimethylamino)-2-methylphenyl)prop-2-yn-1-yl)-_N_-((2-(1-tosyl-1_H_-indol-3-yl)phenyl)ethynyl)methanesulfonamide (A35)

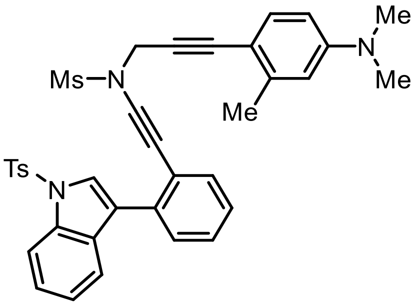

A35

Compound **A35** was prepared in 42% yield (419.8 mg) with four steps according to the general procedure as a white solid (mp 96–98 °C). ^{1}H NMR (400 MHz, $CDCl_3$) δ 7.98 (d, *J* = 8.4 Hz, 1H), 7.86 (s, 1H), 7.79 (d, *J* = 8.4 Hz, 2H), 7.62 – 7.55 (m, 2H), 7.49 – 7.44 (m, 1H), 7.39 – 7.34 (m, 1H), 7.33 – 7.25 (m, 2H), 7.22 – 7.14 (m, 4H), 6.51 – 6.34 (m, 2H), 4.34 (s, 2H), 2.94 (s, 6H), 2.73 (s, 3H), 2.31 (s, 3H), 2.26 (s, 3H); ^{13}C NMR (100 MHz, $CDCl_3$) δ 150.6, 145.0, 141.5, 135.2, 134.8, 134.4, 133.3, 133.2, 129.9, 129.8, 128.1, 127.4, 126.9, 124.8, 124.6, 123.3, 122.2, 121.8, 121.1, 113.6, 112.7, 109.5, 108.6, 87.1, 85.2, 82.7, 70.6, 43.1, 40.1, 38.4, 21.5, 21.1; IR (neat): 2924(bs), 2233(s), 1607, 1519, 1447 13362, 1167, 1131 767, 749 cm^{-1}; HRESIMS Calcd for $[C_{36}H_{33}N_3NaO_4S_2]^+$ (M + Na$^+$) 658.1805, found 658.1805..

***N*-(3-(4-(dimethylamino)naphthalen-1-yl)prop-2-yn-1-yl)-*N*-((2-(1-tosyl-1*H*-indol -3-yl)phenyl)ethynyl)methanesulfonamide (A36)**

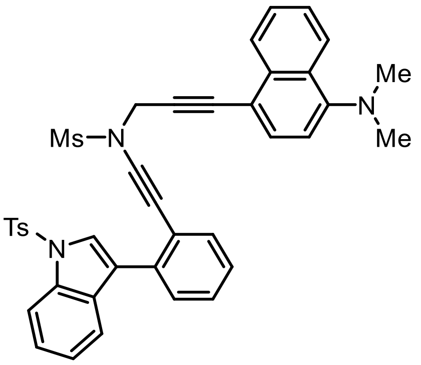

A36

Compound **A36** was prepared in 59% yield (631.3 mg) with four steps according to the general procedure as a white solid (mp 107–109 °C). ^{1}H NMR (400 MHz, $CDCl_3$) δ 8.24 (d, *J* = 8.0 Hz, 1H), 8.16 (d, *J* = 8.4 Hz, 1H), 7.96 (d, *J* = 8.0 Hz, 1H), 7.88 (s, 1H), 7.70 (d, *J* = 8.0 Hz, 2H), 7.64 (d, *J* = 7.6 Hz, 1H), 7.55 (d, *J* = 8.0 Hz, 1H), 7.51 – 7.39 (m, 3H), 7.35 (d, *J* = 7.6 Hz, 1H), 7.31 (d, *J* = 7.6 Hz, 1H), 7.26 – 7.19 (m, 2H), 7.14 – 6.99 (m, 3H), 6.89 (d, *J* =8.0 Hz, 1H), 4.47 (s, 2H), 2.86 (s, 6H), 2.74 (s, 3H), 2.20 (s, 3H); ^{13}C NMR (100 MHz, $CDCl_3$) δ 152.2, 144.9, 134.9, 134.7, 134.5, 134.4, 133.2, 131.3, 129.8, 129.6, 128.2, 128.0, 127.4, 126.7, 126.6, 126.2, 125.4, 124.8, 124.6, 124.5,

123.3, 122.0, 121.6, 121.0, 113.5, 113.00, 112.95, 85.7, 85.1, 85.0, 70.9, 44.7, 43.0, 38.2, 21.3; IR (neat): 2928(bs), 2231(s),1574, 1447, 1390, 1363, 1189, 1167, 1132, 935, 768, 749 cm^{-1}; HRESIMS Calcd for $[C_{39}H_{33}N_3NaO_4S_2]^+$ (M + Na^+) 694.1805, found 694.1802.

4. General Procedure for the Synthesis of Chiral Cyclopropane-Fused Indolines, 1,2-Dioxolanes and Cyclohepta[*b*]indoles

General procedure for the synthesis of Chiral Cyclopropane-Fused Indolines B:

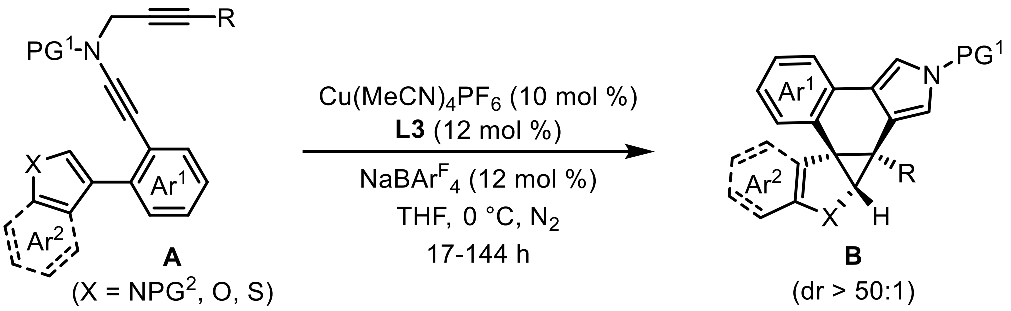

To the mixture of $Cu(MeCN)_4PF_6$ (0.015 mmol, 5.6 mg), **L3** (0.018 mmol, 14.2 mg), and $NaBAr^F{}_4$ (0.018 mmol, 15.9 mg) were introduced into an oven-dried Schlenk tube under nitrogen atmosphere. After THF (1 mL) was injected into the Schlenk tube, the solution was stirred at rt under the argon atmosphere for 2 h. Then the reaction was cooled to 0 °C, and ynamide **A** (0.15 mmol) in THF (2 mL) was introduced into the system dropwise. The resulting mixture was stirred at indicating temperature and the progress of the reaction was monitored by TLC. After concentration in vacuo, the residue was purified by flash chromatography on silica gel (eluent: hexanes/EA or hexanes/DCM) to give the final product **B**.

***N,N*-dimethyl-4-((3b*R*,3c*S*,8b*S*)-2-(methylsulfonyl)-4-tosyl-3c,4-dihydrobenzo[6',7']isoindolo[5',4':2,3]cyclopropa[1,2-*b*]indol-3b(2*H*)-yl)aniline (B1)**

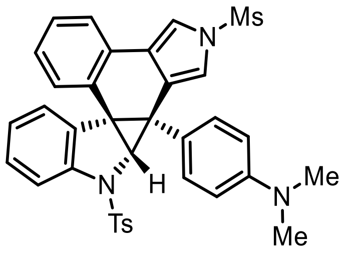

B1

Compound **B1** was prepared according to the general procedure (0 °C, 26 h) in 99% yield (93.0 mg) as a yellow solid (mp 154–156 °C). $[\alpha]_D^{20}$ = +327.1 (c = 1.0, $CHCl_3$). 98% ee. (determined by HPLC: Chiralpak IA Column, 50/50 *i*-PrOH/hexane, 1.0 mL/min, 254 nm; TR = 6.70 min (major), 8.88 min (minor)). ^{1}H NMR (400 MHz, $CDCl_3$) δ 7.59 (d, *J* = 7.6 Hz, 1H), 7.45 (d, *J* = 2.0 Hz, 1H), 7.38 – 7.34 (m, 1H), 7.33 – 7.24 (m, 4H), 7.23 – 7.18 (m, 1H), 7.05 – 6.98 (m, 3H), 6.95 (d, *J* = 8.0 Hz, 2H), 6.80 (d, *J* = 7.6 Hz, 1H), 6.66 (d, *J* = 2.0 Hz, 1H), 6.66 – 5.65 (m, 3H), 4.01 (s, 1H), 3.14 (s, 3H), 2.80 (s, 6H), 2.27 (s, 3H); ^{13}C NMR (100 MHz, $CDCl_3$) δ 149.0, 143.9, 141.6, 133.7, 132.8, 131.1, 131.0, 129.3, 127.7, 127.3, 127.0, 126.8, 126.5, 126.0, 124.4, 123.1, 122.7, 121.9, 119.2, 117.5, 113.7, 111.7, 55.3, 42.8, 40.3, 40.2, 31.0, 21.3; IR (neat): 2925(bs), 1613, 1525, 1362, 1171, 1074, 983, 770, 661 cm^{-1}; HRESIMS Calcd for $[C_{35}H_{31}N_3NaO_4S_2]^+$ (M + Na$^+$) 644.1648, found 644.1643.

4-((3b*R*,3c*S*,8b*S*)-2,4-ditosyl-3c,4-dihydrobenzo[6',7']isoindolo[5',4':2,3]cyclopropa[1,2-*b*]indol-3b(2*H*)-yl)-*N*,*N*-dimethylaniline (B2)

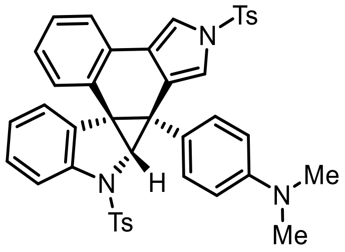

B2

Compound **B2** was prepared according to the general procedure (0 °C, 96 h) in 99% yield (104.1 mg) as a white solid (mp 164–166 °C). $[\alpha]_D^{20}$ = +263.2 (c = 1.0, $CHCl_3$). 93% ee. (determined by HPLC: Chiralpak IC Column, 50/50 *i*-PrOH/hexane, 1.0

mL/min, 254 nm; TR = 11.75 min (major), 14.49 min (minor)). ^{1}H NMR (400 MHz, $CDCl_3$) δ 7.74 (d, *J* = 8.4 Hz, 2H), 7.56 (d, *J* = 7.2 Hz, 1H), 7.48 (d, *J* = 2.0 Hz, 1H), 7.36 – 7.32 (m, 1H), 7.29 (d, *J* = 8.4 Hz, 2H), 7.26 – 7.20 (m, 4H), 7.18 – 7.14 (m, 1H), 7.00 – 6.94 (m, 3H), 6.90 (d, *J* = 8.0 Hz, 2H), 6.70 (d, *J* = 7.6 Hz, 1H), 6.65 (d, *J* = 1.6 Hz, 1H), 6.65 – 5.97 (m, 3H), 3.91 (s, 1H), 2.78 (s, 6H), 2.39 (s, 3H), 2.24 (s, 3H); ^{13}C NMR (100 MHz, $CDCl_3$) δ 148.9, 145.1, 143.8, 141.6, 135.6, 133.6, 133.0, 131.1, 130.8, 130.1, 129.2, 127.6, 127.1, 126.99, 126.95, 126.6, 126.4, 126.3, 126.1, 125.9, 124.5, 123.0, 122.8, 121.9, 119.4, 117.7, 113.8, 111.7, 55.2, 40.32, 40.25, 31.2, 21.6, 21.3; IR (neat): 2923(bs), 1612, 1525, 1484, 1361, 1170, 1046, 811, 670, 579 cm^{-1}; HRESIMS Calcd for $[C_{41}H_{35}N_3NaO_4S_2]^+$ (M + Na$^+$) 720.1961, found 720.1953.

***N*,*N*-dimethyl-4-((3b*R*,3c*S*,8b*S*)-2-(phenylsulfonyl)-4-tosyl-3c,4-dihydrobenzo[6',7']isoindolo[5',4':2,3]cyclopropa[1,2-*b*]indol-3b(2*H*)-yl)aniline (B3)**

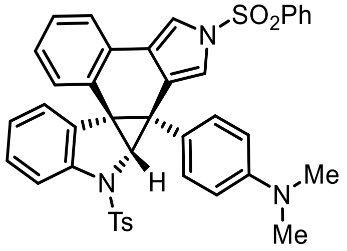

B3

Compound **B3** was prepared according to the general procedure (0 °C, 110 h) in 99% yield (101.6 mg) as a white solid (mp 151–153 °C). $[\alpha]_D^{20}$ = +286.0 (c = 1.0, $CHCl_3$). 91% ee. (determined by HPLC: Chiralpak IC Column, 50/50 *i*-PrOH/hexane, 1.0 mL/min, 254 nm; TR = 9.78 min (major), 12.04 min (minor)). ^{1}H NMR (400 MHz, $CDCl_3$) δ 7.86 (d, *J* = 7.6 Hz, 2H), 7.62 – 7.58 (m, 1H), 7.57 (d, *J* = 7.6 Hz, 1H), 7.52 (d, *J* = 8.0 Hz, 2H), 7.48 (d, *J* = 2.4 Hz, 1H), 7.36 – 7.33 (m, 1H), 7.26 (s, 1H), 7.25 – 7.21 (m, 3H), 7.19 – 7.15 (m, 1H), 7.00 – 6.95 (m, 3H), 6.91 (d, *J* = 8.0 Hz, 2H), 6.71 (d, *J* = 7.6 Hz, 1H), 6.68 (d, *J* = 2.4 Hz, 1H), 6.67 – 6.07 (m, 3H), 3.92 (s, 1H), 2.79 (s, 6H), 2.25 (s, 3H); ^{13}C NMR (100 MHz, $CDCl_3$) δ 148.9, 143.9, 141.6, 138.6, 134.0, 133.6, 133.0, 131.1, 130.8, 129.5, 129.2, 127.6, 127.2, 127.0, 126.9, 126.8, 126.4, 126.0,

125.9, 124.5, 123.0, 122.8, 122.1, 119.5, 117.7, 113.8, 111.8, 55.2, 40.3, 31.2, 21.3; IR (neat): 2923(bs), 1612, 1525, 1484, 1361, 1170, 1071, 811, 768, 670 cm^{-1}; HRESIMS Calcd for $[C_{40}H_{33}N_3NaO_4S_2]^+$ (M + Na^+) 706.1805, found 706.1804.

4-((3b*R*,3c*S*,8b*S*)-2-((4-methoxyphenyl)sulfonyl)-4-tosyl-3c,4-dihydrobenzo[6',7'] isoindolo[5',4':2,3]cyclopropa[1,2-*b*]indol-3b(2*H*)-yl)-*N*,*N*-dimethylaniline (B4)

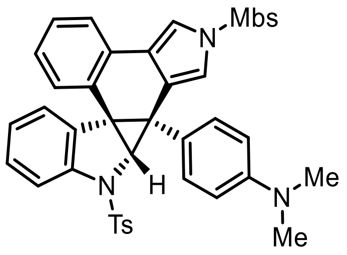

B4

Compound **B4** was prepared according to the general procedure (0 °C, 96 h) in 99% yield (106.1 mg) as a white solid (mp 149–151 °C). $[\alpha]_D^{20}$ = +267.5 (c = 1.0, $CHCl_3$). 93% ee. (determined by HPLC: Chiralpak IC Column, 50/50 *i*-PrOH/hexane, 1.0 mL/min, 254 nm; TR = 13.85 min (major), 17.50 min (minor)). ^{1}H NMR (400 MHz, $CDCl_3$) δ 7.80 (d, *J* = 8.8 Hz, 2H), 7.56 (d, *J* = 7.6 Hz, 1H), 7.47 (d, *J* = 2.0 Hz, 1H), 7.37 – 7.32 (m, 1H), 7.27 – 7.20 (m, 4H), 7.19 – 7.14 (m, 1H), 7.01 – 6.94 (m, 5H), 6.91 (d, *J* = 8.0 Hz, 2H), 6.70 (d, *J* = 7.6 Hz, 1H), 6.64 (d, *J* = 2.0 Hz, 1H), 6.63 – 5.76 (m, 3H), 3.91 (s, 1H), 3.84 (s, 3H), 2.79 (s, 6H), 2.25 (s, 3H); ^{13}C NMR (100 MHz, $CDCl_3$) δ 163.9, 148.9, 143.8, 141.6, 133.5, 133.0, 131.1, 130.7, 129.9, 129.3, 129.2, 127.6, 127.1, 127.0, 126.5, 126.4, 126.3, 126.2, 125.9, 124.5, 123.0, 122.9, 121.7, 119.3, 117.7, 114.7, 113.7, 111.8, 55.6, 55.1, 40.3, 31.2, 21.3; IR (neat): 2926(bs), 1595, 1525, 1360, 1265, 1165, 1072, 1045, 811, 768, 735, 681 cm^{-1}; HRESIMS Calcd for $[C_{41}H_{35}N_3NaO_5S_2]^+$ (M + Na^+) 736.1910, found 736.1896.

4-((3b*R*,3c*S*,8b*S*)-2-((4-bromophenyl)sulfonyl)-4-tosyl-3c,4-dihydrobenzo[6',7']isoindolo[5',4':2,3]cyclopropa[1,2-*b*]indol-3b(2*H*)-yl)-*N*,*N*-dimethylaniline (B5)

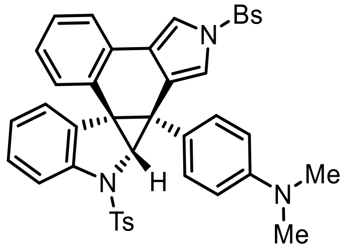

B5

Compound **B5** was prepared according to the general procedure (0 °C, 121 h) in 99% yield (116.2 mg) as a white solid (mp 139–140 °C). $[\alpha]_D^{20}$ = +258.1 (c = 1.0, $CHCl_3$). 93% ee. (determined by HPLC: Chiralpak IC Column, 50/50 *i*-PrOH/hexane, 1.0 mL/min, 254 nm; TR = 10.61 min (major), 12.90 min (minor)). ^{1}H NMR (400 MHz, $CDCl_3$) δ 7.91 – 7.83 (m, 2H), 7.64 – 7.60 (m, 1H), 7.57 (d, *J* = 7.2 Hz, 1H), 7.55 – 7.50 (m, 2H), 7.48 (d, *J* = 2.0 Hz, 1H), 7.37 – 7.33 (m, 1H), 7.25 – 7.23 (m, 2H), 7.22 (d, *J* = 2.4 Hz, 1H), 7.20 – 7.15 (m, 1H), 7.02 – 6.95 (m, 3H), 6.91 (d, *J* = 8.0 Hz, 2H), 6.71 (d, *J* = 7.2 Hz, 1H), 6.67 (d, *J* = 2.0 Hz, 1H), 6.67 – 5.98 (m, 3H), 3.91 (s, 1H), 2.81 (s, 6H), 2.26 (s, 3H); ^{13}C NMR (100 MHz, $CDCl_3$) δ 149.0, 143.9, 141.6, 138.6, 134.0, 133.6, 133.0, 131.1, 130.9, 129.5, 129.3, 127.7, 127.2, 127.1, 126.9, 126.8, 126.4, 126.1, 125.9, 124.5, 123.1, 122.8, 122.1, 119.5, 117.8, 113.8, 111.9, 55.2, 40.3, 31.2, 21.4; IR (neat): 2926(bs), 1595, 1525, 1360, 1265, 1166, 1072, 1026, 811, 735, 680 cm^{-1}; HRESIMS Calcd for $[C_{40}H_{32}BrN_3NaO_4S_2]^+$ (M + Na^+) 784.0910 (isotopic ion: 786.0896), found 784.0909 (isotopic ion: 786.0893).

***N*,*N*-dibenzyl-4-((3b*R*,3c*S*,8b*S*)-2-(methylsulfonyl)-4-tosyl-3c,4-dihydrobenzo[6',7']isoindolo[5',4':2,3]cyclopropa[1,2-*b*]indol-3b(2*H*)-yl)aniline (B6)**

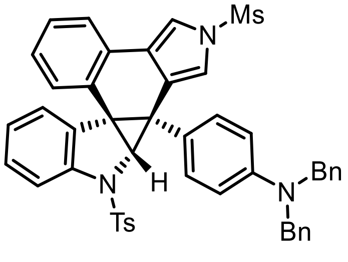

B6

Compound **B6** was prepared according to the general procedure (0 °C, 50 h) in 93% yield (108.2 mg) as a white solid (mp 119–121 °C). $[\alpha]_D^{20}$ = +126.1 (c = 1.0, $CHCl_3$). 95% ee. (determined by HPLC: Chiralpak IA Column, 50/50 *i*-PrOH/hexane, 1.0 mL/min, 254 nm; TR = 8.09 min (major), 10.47 min (minor)). ^{1}H NMR (400 MHz, $CDCl_3$) δ 7.57 (d, *J* = 7.6 Hz, 1H), 7.44 (d, *J* = 2.4 Hz, 1H), 7.37 (d, *J* = 8.0 Hz, 1H), 7.29 (d, *J* = 1.6 Hz, 2H), 7.27 (s, 2H), 7.25 (d, *J* = 1.2 Hz, 2H), 7.24 – 7.15 (m, 5H), 7.10 – 7.08 (m, 2H), 7.07 (d, *J* = 5.2 Hz, 2H), 7.05 – 7.01 (m, 1H), 6.99 (d, *J* = 6.4 Hz, 1H), 6.96 (d, *J* = 7.6 Hz, 1H), 6.92 (d, *J* = 8.0 Hz, 2H), 6.76 (d, *J* = 7.6 Hz, 1H), 6.73 (d, *J* = 2.0 Hz, 1H), 6.61 – 6.08 (m, 3H), 4.45 (q, *J* = 16.0 Hz, 4H), 3.98 (s, 1H), 3.13 (s, 3H), 2.25 (s, 3H); ^{13}C NMR (100 MHz, $CDCl_3$) δ 147.8, 143.9, 141.6, 138.4, 133.7, 133.0, 131.4, 130.9, 129.3, 128.44, 128.38, 127.7, 127.01, 126.95, 126.74, 126.71, 126.5, 126.01, 125.96, 124.5, 123.1, 122.9, 122.0, 119.3, 117.7, 113.8, 112.0, 55.1, 53.9, 42.8, 40.3, 31.0, 21.3; IR (neat): 2926(bs), 1611, 1521, 1453, 1263, 1170, 1075, 810, 768, 732 cm^{-1}; HRESIMS Calcd for $[C_{47}H_{39}N_3NaO_4S_2]^+$ (M + Na^+) 796.2274, found 796.2256.

(3b*R*,3c*S*,8b*S*)-2-(methylsulfonyl)-3b-(4-(pyrrolidin-1-yl)phenyl)-4-tosyl-2,3b,3c,4-tetrahydrobenzo[6',7']isoindolo[5',4':2,3]cyclopropa[1,2-*b*]indole (B7)

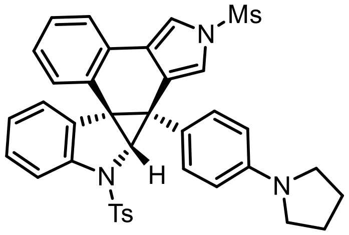

B7

Compound **B7** was prepared according to the general procedure (0 °C, 24 h) in 99% yield (96.7 mg) as a white solid (mp 185–187 °C). $[\alpha]_D^{20}$ = +294.7 (c = 1.0, $CHCl_3$). 98% ee. (determined by HPLC: Chiralpak IA Column, 50/50 *i*-PrOH/hexane, 1.0 mL/min, 254 nm; TR = 6.85 min (major), 9.40 min (minor)). ^{1}H NMR (400 MHz, $CDCl_3$) δ 7.59 (d, *J* = 7.6 Hz, 1H), 7.45 (d, *J* = 1.2 Hz, 1H), 7.39 – 7.36 (m, 1H), 7.32 (s, 1H), 7.31 – 7.27 (m, 2H), 7.24 (s, 1H), 7.22 – 7.18 (m, 1H), 7.04 – 6.98 (m, 3H),

6.95 (d, *J* = 8.0 Hz, 2H), 6.81 (d, *J* = 8.0 Hz, 1H), 6.66 (d, *J* = 1.2 Hz, 1H), 6.57 – 5.87 (m, 3H), 4.00 (s, 1H), 3.16 – 3.08 (m, 7H), 2.27 (s, 3H), 1.94 – 1.87 (m, 4H); ^{13}C NMR (100 MHz, $CDCl_3$) δ 146.4, 143.9, 141.7, 133.8, 132.8, 131.2, 131.1, 129.3, 127.7, 127.2, 127.00, 126.96, 126.49, 126.47, 126.0, 124.3, 123.1, 121.9, 121.6, 119.2, 117.4, 113.7, 111.1, 55.4, 47.4, 42.8, 40.4, 31.1, 25.3, 21.3; IR (neat): 2926(bs), 1611, 1522, 1484, 1364, 1364, 1264, 1171, 1075, 983, 811, 770 cm^{-1}; HRESIMS Calcd for $[C_{37}H_{33}N_3NaO_4S_2]^+$ (M + Na$^+$) 670.1805, found 670.1788.

(3b*R*,3c*S*,8b*S*)-3b-(4-methoxyphenyl)-2-(methylsulfonyl)-4-tosyl-2,3b,3c,4-tetrahydrobenzo[6',7']isoindolo[5',4':2,3]cyclopropa[1,2-*b*]indole (B8)

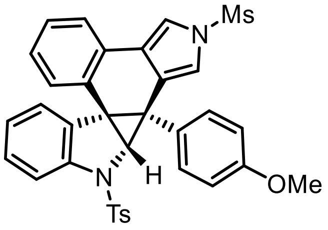

B8

Compound **B8** was prepared according to the general procedure (30 °C, 144 h) in 98% yield (89.9 mg) as a white solid (mp 142–145 °C). $[\alpha]_D^{20}$ = +296.6 (c = 1.0, $CHCl_3$). 81% ee. (determined by HPLC: Chiralpak IC Column, 50/50 *i*-PrOH/hexane, 1.0 mL/min, 254 nm; TR = 10.63 min (minor), 15.09 min (major)). ^{1}H NMR (400 MHz, $CDCl_3$) δ 7.60 (d, *J* = 7.2 Hz, 1H), 7.46 (d, *J* = 2.0 Hz, 1H), 7.36 – 7.34 (m, 1H), 7.32 (s, 1H), 7.30 (s, 1H), 7.30 – 7.24 (m, 2H), 7.24 – 7.20 (m, 1H), 7.06 – 7.03 (m, 1H), 7.02 – 6.99 (m, 2H), 6.98 (s, 1H), 6.96 (s, 1H), 6.80 (d, *J* = 7.6 Hz, 1H), 6.77 – 6.36 (m, 4H), 4.03 (s, 1H), 3.66 (s, 3H), 3.16 (s, 3H), 2.28 (s, 3H); ^{13}C NMR (100 MHz, $CDCl_3$) δ 158.1, 144.0, 141.5, 133.7, 132.7, 131.6, 130.7, 129.4, 127.7, 127.4, 127.0, 126.7, 126.6, 126.3, 126.02, 125.96, 124.5, 123.2, 121.9, 119.2, 117.6, 113.9, 113.0, 55.1, 54.9, 42.9, 40.3, 30.8, 21.4; IR (neat): 2928(bs), 1609, 1513, 1462, 1247, 1172, 1075, 1046, 983, 798, 769 cm^{-1}; HRESIMS Calcd for $[C_{34}H_{28}N_2NaO_5S_2]^+$ (M + Na$^+$) 631.1332, found 631.1316.

(3b*R*,3c*S*,8b*S*)-3b-(4-(benzyloxy)phenyl)-2-(methylsulfonyl)-4-tosyl-2,3b,3c,4-tetrahydrobenzo[6',7']isoindolo[5',4':2,3]cyclopropa[1,2-*b*]indole (B9)

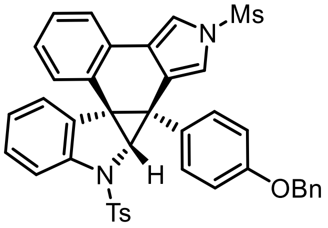

B9

Compound **B9** was prepared according to the general procedure (30 °C, 144 h) in 98% yield (100.5 mg) as a white solid (mp 119–121 °C). $[\alpha]_D^{20}$ = +247.0 (c = 1.0, $CHCl_3$). 82% ee. (determined by HPLC: Chiralpak IC Column, 50/50 *i*-PrOH/hexane, 1.0 mL/min, 254 nm; TR = 11.15 min (minor), 13.39 min (major)). ^{1}H NMR (400 MHz, $CDCl_3$) δ 7.60 (d, *J* = 7.2 Hz, 1H), 7.46 (d, *J* = 2.0 Hz, 1H), 7.36 (d, *J* = 2.0 Hz, 1H), 7.34 (s, 2H), 7.33 (s, 2H), 7.32 (s, 1H), 7.32 – 7.28 (m, 3H), 7.27 (d, *J* = 2.4 Hz, 1H), 7.23 – 7.16 (m, 1H), 7.05 – 6.99 (m, 3H), 6.96 (d, *J* = 8.0 Hz, 2H), 6.80 (d, *J* = 7.6 Hz, 1H), 6.79 – 6.37 (m, 4H), 4.89 (s, 2H), 4.04 (s, 1H), 3.14 (s, 3H), 2.27 (s, 3H); ^{13}C NMR (100 MHz, $CDCl_3$) δ 157.4, 144.0, 141.5, 136.8, 133.7, 132.7, 131.7, 130.7, 129.4, 128.4, 127.8, 127.7, 127.5, 127.4, 127.3, 127.0, 126.7, 126.6, 126.2, 126.03, 125.96, 124.6, 123.2, 121.9, 119.2, 117.6, 114.3, 113.9, 69.8, 55.1, 42.9, 40.3, 30.8, 21.4; IR (neat): 2927(bs), 1732, 1606, 1511, 1461, 1361, 1239, 1172, 1074, 983, 769 cm^{-1}; HRESIMS Calcd for $[C_{40}H_{32}N_2NaO_5S_2]^+$ (M + Na^+) 707.1645, found 707.1630.

(3b*R*,3c*S*,8b*S*)-2-(methylsulfonyl)-3b-(4-(methylthio)phenyl)-4-tosyl-2,3b,3c,4-tetrahydrobenzo[6',7']isoindolo[5',4':2,3]cyclopropa[1,2-*b*]indole (B10)

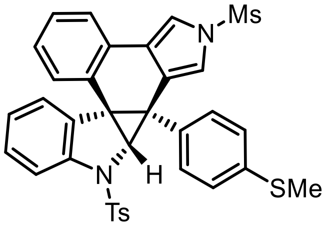

B10

Compound **B10** was prepared according to the general procedure (30 °C, 144 h) in 76% yield (71.3 mg) as a white solid (mp 124–125 °C). $[\alpha]_D^{20}$ = +280.2 (c = 1.0, $CHCl_3$). 85% ee. (determined by HPLC: Chiralpak IC Column, 50/50 *i*-PrOH/hexane, 1.0 mL/min, 254 nm; TR = 10.77 min (minor), 13.91 min (major)). ^{1}H NMR (400 MHz, $CDCl_3$) δ 7.60 (d, *J* = 7.2 Hz, 1H), 7.47 (d, *J* = 2.0 Hz, 1H), 7.37 – 7.34 (m, 1H), 7.34 – 7.29 (m, 3H), 7.29 – 7.26 (m, 1H), 7.24 – 7.20 (m, 1H), 7.06 – 6.98 (m, 5H), 6.97 – 6.81 (m, 3H), 6.80 (d, *J* = 7.6 Hz, 1H), 6.63 (d, *J* = 2.0 Hz, 1H), 4.05 (s, 1H), 3.16 (s, 3H), 2.36 (s, 3H), 2.29 (s, 3H); ^{13}C NMR (100 MHz, $CDCl_3$) δ 144.1, 141.5, 136.9, 133.7, 132.5, 131.7, 130.9, 130.5, 129.4, 127.7, 127.6, 127.1, 126.8, 126.7, 126.03, 125.97, 125.8, 125.7, 124.6, 123.2, 121.9, 119.2, 117.7, 114.0, 55.0, 42.9, 40.3, 31.0, 21.4, 15.4; IR (neat): 2925(bs), 1598, 1527, 1485, 1364, 1264, 1171, 1074, 984, 812, 770 cm^{-1}; HRESIMS Calcd for $[C_{34}H_{28}N_2NaO_4S_3]^+$ (M + Na$^+$) 647.1103, found 647.1098.

(3b*R*,3c*S*,8b*S*)-3b-(benzo[d][1,3]dioxol-5-yl)-2-(methylsulfonyl)-4-tosyl-2,3b,3c,4-tetrahydrobenzo[6',7']isoindolo[5',4':2,3]cyclopropa[1,2-*b*]indole (B11)

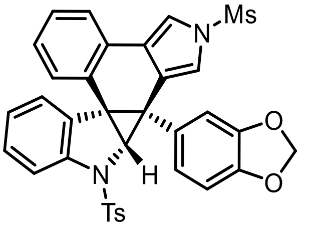

B11

Compound **B11** was prepared according to the general procedure (30 °C, 144 h) in 99% yield (92.9 mg) as a white solid (mp 156–158 °C). $[\alpha]_D^{20}$ = +297.0 (c = 1.0, $CHCl_3$). 84% ee. (determined by HPLC: Chiralpak IC Column, 50/50 *i*-PrOH/hexane, 1.0 mL/min, 254 nm; TR = 12.59 min (minor), 15.53 min (major)). ^{1}H NMR (400 MHz, $CDCl_3$) δ 7.60 (d, *J* = 7.6 Hz, 1H), 7.46 (d, *J* = 2.0 Hz, 1H), 7.41 (d, *J* = 7.2 Hz, 1H), 7.31 (d, *J* = 8.0 Hz, 2H), 7.29 – 7.26 (m, 1H), 7.24 – 7.20 (m, 1H), 7.14 – 6.99 (m, 4H), 6.97 (d, *J* = 8.0 Hz, 2H), 6.77 (d, *J* = 8.0 Hz, 1H), 6.68 (s, 1H), 6.63 – 6.33 (m, 1H), 6.15 – 5.89 (m, 1H), 5.78 (s, 2H), 4.02 (s, 1H), 3.18 (s, 3H), 2.29 (s, 3H); ^{13}C NMR

(100 MHz, $CDCl_3$) δ 146.9, 146.3, 144.1, 141.5, 133.6, 132.6, 130.5, 129.4, 128.5, 127.7, 127.5, 127.1, 126.74, 126.65, 126.1, 126.0, 125.9, 124.7, 124.2, 123.2, 121.9, 119.2, 117.8, 113.9, 110.7, 107.5, 100.7, 55.1, 43.0, 40.4, 31.2, 21.4; IR (neat): 2926(bs), 1731, 1599, 1485, 1440, 1364, 1232, 1171, 1070, 983, 809, 768 cm^{-1}; HRESIMS Calcd for $[C_{34}H_{26}N_2NaO_6S_2]^+$ (M + Na^+) 645.1124, found 645.1123.

(3b*S*,3c*R*,8b*R*)-2-(methylsulfonyl)-3b-(thiophen-2-yl)-4-tosyl-2,3b,3c,4-tetrahydrobenzo[6',7']isoindolo[5',4':2,3]cyclopropa[1,2-*b*]indole (B12)

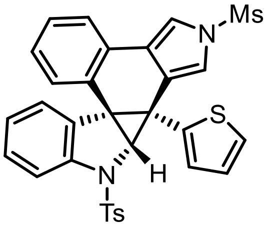

B12

Compound **B12** was prepared according to the general procedure (30 °C, 144 h) in 99% yield (87.1 mg) as a white solid (mp 134–137 °C). $[\alpha]_D^{20}$ = +278.8 (c = 1.0, $CHCl_3$). 80% ee. (determined by HPLC: Chiralpak IC Column, 50/50 *i*-PrOH/hexane, 1.0 mL/min, 254 nm; TR = 9.19 min (minor), 12.33 min (major)). ^1H NMR (400 MHz, $CDCl_3$) δ 7.61 (d, *J* = 7.6 Hz, 1H), 7.49 (d, *J* = 2.0 Hz, 1H), 7.46 (d, *J* = 8.0 Hz, 1H), 7.32 (d, *J* = 8.0 Hz, 2H), 7.27 (d, *J* = 7.6 Hz, 1H), 7.22 (d, *J* = 7.2 Hz, 1H), 7.12 – 7.07 (m, 1H), 7.06 – 7.01 (m, 2H), 6.98 (d, *J* = 3.2 Hz, 2H), 6.95 (d, *J* = 5.2 Hz, 2H), 6.82 (d, *J* = 2.0 Hz, 1H), 6.78 (d, *J* = 7.6 Hz, 1H), 6.72 – 6.62 (m, 1H), 4.13 (s, 1H), 3.18 (s, 3H), 2.28 (s, 3H); ^{13}C NMR (100 MHz, $CDCl_3$) δ 144.1, 141.9, 136.8, 133.6, 131.9, 130.1, 129.43, 129.37, 127.73, 127.70, 127.0, 126.9, 126.7, 126.4, 125.9, 125.7, 125.6, 125.4, 124.7, 123.2, 121.7, 119.5, 117.5, 114.0, 55.9, 42.9, 42.1, 26.4, 21.4; IR (neat): 2927(bs), 1598, 1461, 1365, 1264, 1171, 1077, 1047, 983, 768, 733 cm^{-1}; HRESIMS Calcd for $[C_{31}H_{24}N_2NaO_4S_3]^+$ (M + Na^+) 607.0790, found 607.0786.

4-((3b*R*,3c*S*,8b*S*)-12-fluoro-2-(methylsulfonyl)-4-tosyl-3c,4-dihydrobenzo[6',7']isoindolo[5',4':2,3]cyclopropa[1,2-*b*]indol-3b(2*H*)-yl)-*N*,*N*-dimethylaniline (B13)

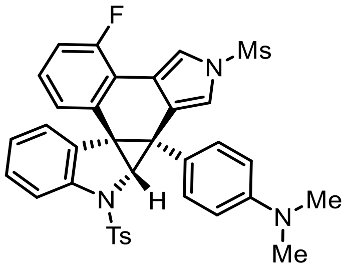

B13

Compound **B13** was prepared according to the general procedure (0 °C, 116 h) in 94% yield (90.1 mg) as a white solid (mp 266–269 °C). $[\alpha]_D^{20}$ = +290.6 (c = 1.0, $CHCl_3$). 92% ee. (determined by HPLC: Chiralpak IA Column, 10/90 *i*-PrOH/hexane, 1.0 mL/min, 254 nm; TR = 19.80 min (major), 23.40 min (minor)). ^{1}H NMR (400 MHz, $CDCl_3$) δ 7.66 – 7.63 (m, 1H), 7.39 – 7.36 (m, 1H), 7.34 – 7.26 (m, 3H), 7.26 (d, *J* = 2.0 Hz, 1H), 7.03 – 6.95 (m, 6H), 6.68 (d, *J* = 2.0 Hz, 1H), 6.62 – 6.59 (m, 1H), 6.59 – 6.01 (m, 3H), 4.01 (s, 1H), 3.16 (s, 3H), 2.80 (s, 6H), 2.28 (s, 3H); ^{13}C NMR (100 MHz, $CDCl_3$) δ 159.8 (d, *J* = 249.0 Hz), 149.1, 143.9, 141.7, 133.7, 133.6, 132.6, 131.1, 129.3, 127.4, 127.0, 126.7 (d, *J* = 9.2 Hz), 126.4, 126.0, 124.5, 123.2 (d, *J* = 2.5 Hz), 122.4, 118.7, 118.0 (d, *J* = 17.5 Hz), 117.7, 116.3 (d, *J* = 1.0 Hz), 114.9 (d, *J* = 15.4 Hz), 113.4 (d, *J* = 21.8 Hz), 111.9, 55.3, 42.9, 40.3, 40.2, 31.3, 21.3; ^{19}F NMR (376 MHz, $CDCl_3$) δ -111.37; IR (neat): 2926(bs), 1614, 1566, 1525, 1479, 1364, 1264, 1171, 1071, 983, 800 cm^{-1}; HRESIMS Calcd for $[C_{35}H_{30}FN_3NaO_4S_2]^+$ (M + Na$^+$) 662.1554, found 662.1543.

4-((3b*R*,3c*S*,8b*S*)-11-chloro-2-(methylsulfonyl)-4-tosyl-3c,4-dihydrobenzo[6',7']isoindolo[5',4':2,3]cyclopropa[1,2-*b*]indol-3b(2*H*)-yl)-*N,N*-dimethylaniline (B14)

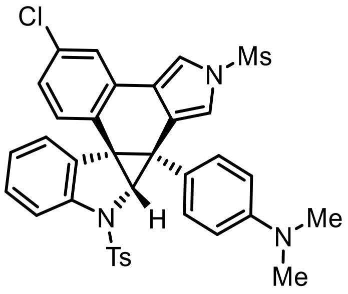

B14

Compound **B14** was prepared according to the general procedure (0 °C, 27 h) in 99% yield (97.2 mg) as a white solid (mp 295–297 °C). $[\alpha]_D^{20}$ = +358.4 (c = 1.0, $CHCl_3$). 99% ee. (determined by HPLC: Chiralpak IC Column, 50/50 *i*-PrOH/hexane, 1.0 mL/min, 254 nm; TR = 10.94 min (minor), 13.29 min (major)). ^{1}H NMR (400 MHz, $CDCl_3$) δ 7.57 (d, *J* = 2.0 Hz, 1H), 7.45 (d, *J* = 2.0Hz, 1H), 7.38 – 7.35 (m, 1H), 7.34 – 7.31 (m, 1H), 7.31 – 7.29 (m, 1H), 7.25 – 7.22 (m, 1H), 7.15 – 6.85 (m, 6H), 6.74 (d, *J* = 8.4 Hz, 1H), 6.66 (d, *J* = 2.0 Hz, 1H), 6.66 – 6.04 (m, 3H), 3.99 (s, 1H), 3.16 (s, 3H), 2.80 (s, 6H), 2.30 (s, 3H); ^{13}C NMR (100 MHz, $CDCl_3$) δ 149.1, 144.0, 141.7, 133.8, 132.3, 131.1, 129.6, 129.4, 129.0, 127.9, 127.5, 127.0, 126.7, 126.4, 125.8, 124.5, 123.1, 122.3, 120.8, 119.4, 117.6, 114.3, 111.8, 55.3, 43.0, 40.3, 40.0, 31.0, 21.4; IR (neat): 2928(bs), 1612, 1524, 1482, 1364, 1261, 1171, 1074, 983, 811, 767 cm^{-1}; HRESIMS Calcd for $[C_{35}H_{30}ClN_3NaO_4S_2]^+$ (M + Na^+) 678.1258 (isotopic ion: 680.1243), found 678.1251 (isotopic ion: 680.1236).

4-((3b*R*,3c*S*,8b*S*)-11-methoxy-2-(methylsulfonyl)-4-tosyl-3c,4-dihydrobenzo[6',7'] isoindolo[5',4':2,3]cyclopropa[1,2-*b*]indol-3b(2*H*)-yl)-*N*,*N*-dimethylaniline (B15)

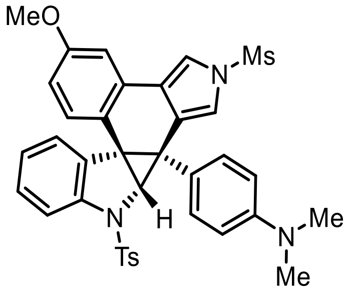

B15

Compound **B15** was prepared according to the general procedure (0 °C, 32 h) in 96% yield (94.0 mg) as a white solid (mp 273–276 °C). $[\alpha]_D^{20}$ = +315.6 (c = 1.0, $CHCl_3$). 98% ee. (determined by HPLC: Chiralpak IC Column, 50/50 *i*-PrOH/hexane, 1.0 mL/min, 254 nm; TR = 13.09 min (minor), 17.24 min (major)). ^{1}H NMR (400 MHz, $CDCl_3$) δ 7.45 (d, *J* = 2.0 Hz, 1H), 7.35 – 7.32 (m, 3H), 7.29 – 7.24 (m, 2H), 7.11 (d, *J* = 2.4 Hz, 1H), 7.00 – 6.97 (m, 4H), 6.74 (d, *J* = 8.8 Hz, 1H), 6.66 (d, *J* = 2.0 Hz, 1H), 6.64 – 6.60 (m, 1H), 6.60 – 6.17 (m, 3H), 3.97 (s, 1H), 3.83 (s, 3H), 3.14 (s, 3H), 2.80

(s, 6H), 2.28 (s, 3H); ^{13}C NMR (100 MHz, $CDCl_3$) δ 158.3, 149.0, 143.8, 141.5, 134.0, 132.9, 131.1, 129.3, 128.9, 127.2, 127.1, 127.0, 125.9, 124.3, 123.5, 122.8, 122.0, 119.3, 117.3, 113.8, 113.3, 111.8, 107.4, 55.3, 55.0, 42.8, 40.2, 39.9, 30.5, 21.3; IR (neat): 2929(bs), 1612, 1522, 1698(s), 1460, 1363, 1264, 1171, 1073, 1040, 982, 807, 771 cm^{-1}; HRESIMS Calcd for $[C_{36}H_{33}N_3NaO_5S_2]^+$ (M + Na^+) 674.1754, found 674.1744.

4-((3b*R*,3c*S*,8b*S*)-10-chloro-2-(methylsulfonyl)-4-tosyl-3c,4-dihydrobenzo[6',7']isoindolo[5',4':2,3]cyclopropa[1,2-*b*]indol-3b(2*H*)-yl)-*N*,*N*-dimethylaniline (B16)

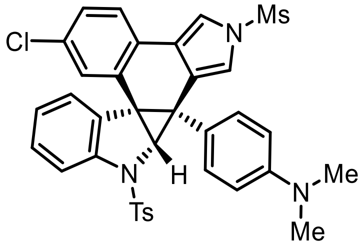

B16

Compound **B16** was prepared according to the general procedure (0 °C, 26 h) in 94% yield (92.7 mg) as a white solid (mp 266–269 °C). $[\alpha]_D^{20}$ = +287.1 (c = 1.0, $CHCl_3$). 98% ee. (determined by HPLC: Chiralpak IA Column, 50/50 *i*-PrOH/hexane, 1.0 mL/min, 254 nm; TR = 6.44 min (major), 9.77 min (minor)). ^{1}H NMR (400 MHz, $CDCl_3$) δ 7.50 (d, *J* = 8.4 Hz, 1H), 7.43 (d, *J* = 2.0 Hz, 1H), 7.40 – 7.37 (m, 1H), 7.32 – 7.20 (m, 4H), 7.18 – 7.15 (m, 1H), 7.06 – 7.02 (m, 2H), 6.99 (d, *J* = 8.0 Hz, 2H), 6.65 (d, *J* = 1.6 Hz, 2H), 6.63 – 6.04 (m, 3H), 4.01 (s, 1H), 3.15 (s, 3H), 2.81 (s, 6H), 2.33 (s, 3H); ^{13}C NMR (100 MHz, $CDCl_3$) δ 149.1, 144.5, 141.7, 133.3, 133.1, 132.5, 132.3, 131.0, 129.3, 127.50, 127.46, 127.1, 126.63, 126.59, 125.7, 125.0, 124.6, 124.4, 122.3, 121.0, 119.4, 118.3, 113.9, 111.8, 55.4, 42.9, 40.3, 40.2, 31.5, 21.5; IR (neat): 2926(bs), 1613, 1526, 1461, 1365, 1263, 1171, 1071, 982, 812, 768 cm^{-1}; HRESIMS Calcd for $[C_{35}H_{30}ClN_3NaO_4S_2]^+$ (M + Na^+) 678.1258 (isotopic ion: 680.1243), found 678.1250 (isotopic ion: 680.1240).

***N*,*N*-dimethyl-4-((3b*R*,3c*S*,8b*S*)-10-methyl-2-(methylsulfonyl)-4-tosyl-3c,4-dihydrobenzo[6',7']isoindolo[5',4':2,3]cyclopropa[1,2-*b*]indol-3b(2*H*)-yl)aniline (B17)**

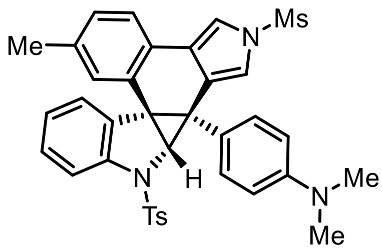

B17

Compound **B17** was prepared according to the general procedure (0 °C, 17 h) in 99% yield (95.4 mg) as a white solid (mp 274–276 °C). $[\alpha]_D^{20}$ = +341.0 (c = 1.0, $CHCl_3$). 98% ee. (determined by HPLC: Chiralpak IC Column, 50/50 *i*-PrOH/hexane, 1.0 mL/min, 254 nm; TR = 12.15 min (minor), 16.59 min (major)). ^{1}H NMR (400 MHz, $CDCl_3$) δ 7.49 (d, *J* = 8.0 Hz, 1H), 7.40 (d, *J* = 2.0 Hz, 1H), 7.37 – 7.32 (m, 3H), 7.30 – 7.27 (m, 1H), 7.24 (s, 1H), 7.04 – 6.97 (m, 5H), 6.66 – 6.62 (m, 2H), 6.62 – 6.12 (m, 3H), 4.02 (s, 1H), 3.12 (s, 3H), 2.79 (s, 6H), 2.29 (s, 3H), 2.20 (s, 3H); ^{13}C NMR (100 MHz, $CDCl_3$) δ 149.0, 143.7, 141.6, 136.4, 134.1, 132.9, 131.1, 131.0, 129.2, 128.1, 127.4, 127.19, 127.15, 126.8, 126.0, 124.4, 123.2, 123.1, 122.8, 122.0, 119.2, 117.5, 113.2, 111.7, 55.3, 42.8, 40.3, 31.0, 21.4, 21.3; IR (neat): 2925(bs), 1613, 1524, 1460, 1362, 1265, 1170, 1071, 983, 812 cm^{-1}; HRESIMS Calcd for $[C_{36}H_{33}N_3NaO_4S_2]^+$ (M + Na^+) 658.1805, found 658.1795.

4-((3b*R*,3c*S*,8b*S*)-7-fluoro-2-(methylsulfonyl)-4-tosyl-3c,4-dihydrobenzo[6',7']isoindolo[5',4':2,3]cyclopropa[1,2-*b*]indol-3b(2*H*)-yl)-*N*,*N*-dimethylaniline (B18)

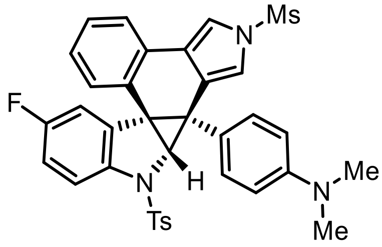

B18

Compound **B18** was prepared according to the general procedure (0 °C, 27 h) in 99% yield (95.1 mg) as a white solid (mp 268–271 °C). $[\alpha]_D^{20}$ = +288.2 (c = 1.0, $CHCl_3$). 99% ee. (determined by HPLC: Chiralpak IA Column, 50/50 *i*-PrOH/hexane, 1.0 mL/min, 254 nm; TR = 7.90 min (major), 9.88min (minor)). ^{1}H NMR (400 MHz, $CDCl_3$) δ 7.58 (d, *J* = 7.6 Hz, 1H), 7.45 (d, *J* = 2.0 Hz, 1H), 7.34 – 7.31 (m, 1H), 7.27 – 7.24 (m, 2H), 7.23 – 7.18 (m, 1H), 7.04 – 6.99 (m, 1H), 6.95 (d, *J* = 8.0 Hz, 3H), 6.93 – 5.75 (m, 7H), 4.02 (s, 1H), 3.15 (s, 3H), 2.82 (s, 6H), 2.29 (s, 3H); ^{13}C NMR (100 MHz, $CDCl_3$) δ 160.1 (d, *J* = 243.2 Hz), 149.1, 144.1, 137.6 (d, *J* = 1.7 Hz), 135.1 (d, *J* = 8.9 Hz), 133.1, 131.0, 130.2, 129.4, 127.3, 127.1, 126.7, 126.6, 126.5, 125.9, 123.2, 122.3, 121.8, 119.4, 119.2 (d, *J* = 8.8 Hz), 114.4 (d, *J* = 23.9 Hz), 113.8, 112.7 (d, *J* = 24.5 Hz), 112.0, 55.8, 42.9, 40.5 (d, *J* = 2.4 Hz), 40.2, 31.4, 21.3; ^{19}F NMR (376 MHz, $CDCl_3$) δ -117.10; IR (neat): 2926(bs), 1613, 1524, 1475, 1364, 1264, 1170, 1074, 984, 948, 809 cm^{-1}; HRESIMS Calcd for $[C_{35}H_{30}FN_3NaO_4S_2]^+$ (M + Na^+) 662.1554, found 662.1552.

4-((3b*R*,3c*S*,8b*S*)-7-chloro-2-(methylsulfonyl)-4-tosyl-3c,4-dihydrobenzo[6',7']isoindolo[5',4':2,3]cyclopropa[1,2-*b*]indol-3b(2*H*)-yl)-*N*,*N*-dimethylaniline (B19)

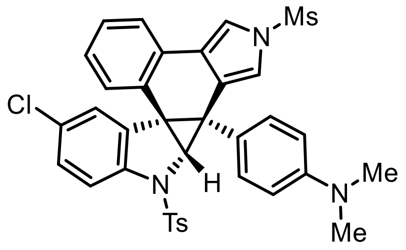

B19

Compound **B19** was prepared according to the general procedure (0 °C, 24 h) in 99% yield (97.3 mg) as a white solid (mp 270–272 °C). $[\alpha]_D^{20}$ = +170.1 (c = 1.0, $CHCl_3$). 99% ee. (determined by HPLC: Chiralpak IB Column, 15/85 *i*-PrOH/hexane, 1.0 mL/min, 254 nm; TR = 14.98 min (major), 17.52 min (minor)). ^{1}H NMR (400 MHz, $CDCl_3$) δ 7.59 (d, *J* = 7.6 Hz, 1H), 7.45 (s, 1H), 7.32 – 7.27 (m, 3H), 7.26 – 7.23 (m, 2H), 7.21 (d, *J* = 7.6 Hz, 1H), 7.06 – 7.02 (m, 1H), 6.98 (d, *J* = 7.2 Hz, 3H), 6.73 (d, *J*

= 7.6 Hz, 1H), 6.66 (s, 1H), 6.60 – 6.18 (m, 3H), 4.02 (s, 1H), 3.15 (s, 3H), 2.83 (s, 6H), 2.30 (s, 3H); ^{13}C NMR (100 MHz, $CDCl_3$) δ 149.1, 144.2, 140.3, 134.8, 133.5, 131.1, 130.2, 129.7, 129.5, 127.5, 127.4, 127.0, 126.8, 126.7, 126.4, 126.0, 125.9, 123.2, 122.2, 121.7, 119.4, 118.5, 113.8, 111.8, 55.7, 42.9, 40.2, 31.1, 21.4; IR (neat): 2925(bs), 1613, 524, 1466, 1365, 1263, 1171, 1077, 1047, 983, 948, 812, 769 cm^{-1}; HRESIMS Calcd for $[C_{35}H_{30}ClN_3NaO_4S_2]^+$ (M + Na^+) 678.1258 (isotopic ion: 680.1243), found 678.1254 (isotopic ion: 680.1235).

4-((3b*R*,3c*S*,8b*S*)-7-bromo-2-(methylsulfonyl)-4-tosyl-3c,4-dihydrobenzo[6',7']isoindolo[5',4':2,3]cyclopropa[1,2-*b*]indol-3b(2*H*)-yl)-*N*,*N*-dimethylaniline (B20)

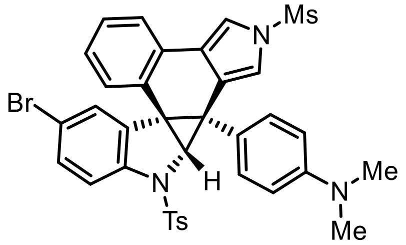

B20

Compound **B20** was prepared according to the general procedure (0 °C, 24 h) in 99% yield (104.3 mg) as a white solid (mp 164–167 °C). $[\alpha]_D^{20}$ = +115.5 (c = 1.0, $CHCl_3$). 99% ee. (determined by HPLC: Chiralpak IA Column, 50/50 *i*-PrOH/hexane, 1.0 mL/min, 254 nm; TR = 8.99 min (major), 11.15 min (minor)). ^{1}H NMR (400 MHz, $CDCl_3$) δ 7.59 (d, *J* = 7.6 Hz, 1H), 7.45 (d, *J* = 2.0 Hz, 1H), 7.40 (d, *J* = 2.0 Hz, 1H), 7.32 – 7.29 (m, 2H), 7.25 (d, *J* = 1.2 Hz, 1H), 7.24 – 7.18 (m, 2H), 7.13 – 7.10 (m, 1H), 7.07 – 7.02 (m, 1H), 6.99 (d, *J* = 8.0 Hz, 2H), 6.75 (d, *J* = 7.2 Hz, 1H), 6.66 (d, *J* = 2.0 Hz, 1H), 6.59 – 6.27 (m, 3H), 4.01 (s, 1H), 3.15 (s, 3H), 2.82 (s, 6H), 2.29 (s, 3H); ^{13}C NMR (100 MHz, $CDCl_3$) δ 149.1, 144.2, 140.8, 135.2, 133.5, 131.1, 130.4, 130.2, 129.5, 128.8, 127.4, 127.0, 126.8, 126.7, 126.3, 126.0, 123.2, 122.1, 121.7, 119.4, 118.8, 117.2, 113.8, 111.8, 55.6, 42.9, 40.22, 40.17, 31.1, 21.4; IR (neat): 2925(bs), 1613, 1524, 1464, 1364, 1263, 1171, 1076, 1047, 983, 811 cm^{-1}; HRESIMS Calcd for

$[C_{35}H_{30}BrN_3NaO_4S_2]^+$ (M + Na^+) 722.0753 (isotopic ion: 724.0737), found 722.0748 (isotopic ion: 724.0721).

_N_,_N_-dimethyl-4-((3b_R_,3c_S_,8b_S_)-7-methyl-2-(methylsulfonyl)-4-tosyl-3c,4-dihydrobenzo[6',7']isoindolo[5',4':2,3]cyclopropa[1,2-_b_]indol-3b(2_H_)-yl)aniline (B21)

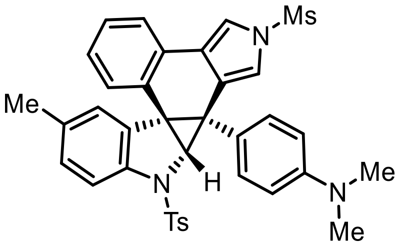

B21

Compound **B21** was prepared according to the general procedure (0 °C, 24 h) in 94% yield (89.7 mg) as a white solid (mp 138–141 °C). $[\alpha]_D^{20}$ = +213.6 (c = 1.0, $CHCl_3$). 99% ee. (determined by HPLC: Chiralpak IA Column, 50/50 *i*-PrOH/hexane, 1.0 mL/min, 254 nm; TR = 5.98 min (major), 8.88 min (minor)). ^1H NMR (400 MHz, $CDCl_3$) δ 7.58 (d, *J* = 7.2 Hz, 1H), 7.43 (d, *J* = 2.4 Hz, 1H), 7.30 – 7.27 (m, 2H), 7.25 – 7.22 (m, 2H), 7.22 – 7.17 (m, 1H), 7.06 (s, 1H), 7.05 – 6.98 (m, 1H), 6.94 (d, *J* = 8.0 Hz, 2H), 6.82 – 6.76 (m, 2H), 6.63 (d, *J* = 2.4 Hz, 1H), 6.62 – 5.97 (m, 3H), 3.96 (s, 1H), 3.14 (s, 3H), 2.81 (s, 6H), 2.27 (s, 3H), 2.23 (s, 3H); ^{13}C NMR (100 MHz, $CDCl_3$) δ 149.0, 143.7, 139.4, 134.1, 133.7, 132.9, 131.2, 131.1, 129.3, 128.1, 127.7, 127.1, 127.0, 126.5, 126.4, 126.3, 126.0, 123.1, 122.9, 122.0, 119.2, 117.4, 113.7, 111.8, 55.5, 42.9, 40.34, 40.30, 31.2, 21.3, 21.1; IR (neat): 2926(bs), 1612, 1526, 1482, 1362, 1265 1169, 1074, 982, 815, 771 cm^{-1}; HRESIMS Calcd for $[C_{36}H_{33}N_3NaO_4S_2]^+$ (M + Na^+) 658.1805, found 658.1793.

4-((3b_R_,3c_S_,8b_S_)-6-fluoro-2-(methylsulfonyl)-4-tosyl-3c,4-dihydrobenzo[6',7']isoindolo[5',4':2,3]cyclopropa[1,2-_b_]indol-3b(2_H_)-yl)-_N_,_N_-dimethylaniline (B22)

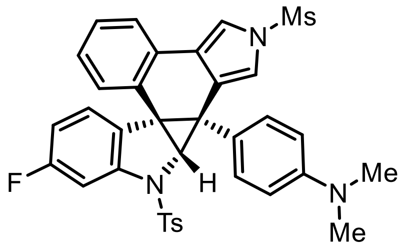

B22

Compound **B22** was prepared according to the general procedure (0 °C, 48 h) in 99% yield (95.6 mg) as a white solid (mp 182–183 °C). $[\alpha]_D^{20}$ = +366.7 (c = 1.0, $CHCl_3$). 99% ee. (determined by HPLC: Chiralpak IA Column, 30/70 *i*-PrOH/hexane, 1.0 mL/min, 254 nm; TR = 9.98 min (major), 11.73 min (minor)). ^{1}H NMR (400 MHz, $CDCl_3$) δ 7.60 (d, *J* = 7.6 Hz, 1H), 7.45 (d, *J* = 2.0 Hz, 1H), 7.38 (d, *J* = 8.4 Hz, 2H), 7.27 – 7.19 (m, 3H), 7.11 – 7.08 (m, 1H), 7.06 (d, *J* = 8.0 Hz, 1H), 7.02 (d, *J* = 8.4 Hz, 2H), 6.82 (d, *J* = 7.6 Hz, 1H), 6.73 – 6.68 (m, 1H), 6.67 (d, *J* = 2.0 Hz, 1H), 6.66 – 5.94 (m, 3H), 4.02 (s, 1H), 3.15 (s, 3H), 2.83 (s, 6H), 2.30 (s, 3H); ^{13}C NMR (100 MHz, $CDCl_3$) δ 161.9 (d, *J* = 244.3 Hz), 149.1, 144.2, 142.9 (d, *J* = 11.8 Hz), 133.9, 131.1, 130.8, 129.5, 128.4 (d, *J* = 2.4 Hz), 127.5, 127.0, 126.8, 126.68, 126.66, 126.6, 126.1, 123.2, 122.4, 121.8, 119.3, 113.8, 111.9, 111.5 (d, *J* = 23.1 Hz), 104.9 (d, *J* = 27.5 Hz), 56.0, 42.9, 40.2, 39.7, 30.8, 21.4; ^{19}F NMR (376 MHz, $CDCl_3$) δ -112.97; IR (neat): 2926(bs), 1612, 1525, 1491, 1364, 1264, 1171, 1075, 1048, 983, 811 cm^{-1}; HRESIMS Calcd for $[C_{35}H_{30}FN_3NaO_4S_2]^+$ (M + Na^+) 662.1554, found 662.1548.

4-((3b*R*,3c*S*,8b*S*)-6-chloro-2-(methylsulfonyl)-4-tosyl-3c,4-dihydrobenzo[6',7']isoindolo[5',4':2,3]cyclopropa[1,2-*b*]indol-3b(2*H*)-yl)-*N*,*N*-dimethylaniline (B23)

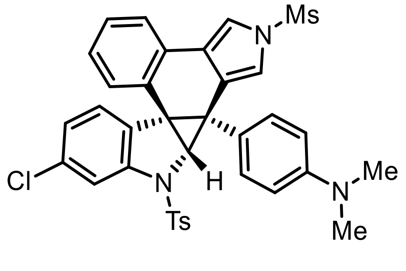

B23

Compound **B23** was prepared according to the general procedure (0 °C, 24 h) in 96% yield (94.7 mg) as a white solid (mp 159–160 °C). $[\alpha]_D^{20}$ = +281.3 (c = 1.0, $CHCl_3$). 99% ee. (determined by HPLC: Chiralpak IB Column, 15/85 *i*-PrOH/hexane, 1.0 mL/min, 254 nm; TR = 15.08 min (major), 17.46 min (minor)). ^{1}H NMR (400 MHz, $CDCl_3$) δ 7.60 (d, *J* = 7.6 Hz, 1H), 7.45 (d, *J* = 2.0 Hz, 1H), 7.39 – 7.36 (m, 3H), 7.24 (d, *J* = 6.4 Hz, 1H), 7.21 (d, *J* = 8.0 Hz, 2H), 7.05 (d, *J* = 7.6 Hz, 1H), 7.02 (d, *J* = 8.0 Hz, 2H), 6.98 – 6.96 (m, 1H), 6.80 (d, *J* = 7.6 Hz, 1H), 6.66 (d, *J* = 2.0 Hz, 1H), 6.63 – 6.15 (m, 3H), 4.01 (s, 1H), 3.14 (s, 3H), 2.84 (s, 6H), 2.30 (s, 3H); ^{13}C NMR (100 MHz, $CDCl_3$) δ 149.2, 144.3, 142.8, 133.8, 132.8, 131.3, 131.1, 130.5, 129.5, 127.5, 127.0, 126.8, 126.74, 126.67, 126.5, 126.1, 124.5, 123.2, 122.2, 121.8, 119.3, 117.1, 113.8, 112.0, 55.8, 42.9, 40.2, 40.0, 30.9, 21.4; IR (neat): 2926(bs), 1613, 1525, 1482, 1364, 1263, 1171, 1074, 983, 814, 770 cm^{-1}; HRESIMS Calcd for $[C_{35}H_{30}ClN_3NaO_4S_2]^+$ (M + Na$^+$) 678.1258 (isotopic ion: 680.1243), found 678.1252 (isotopic ion: 680.1234).

4-((3b*R*,3c*S*,8b*S*)-6-bromo-2-(methylsulfonyl)-4-tosyl-3c,4-dihydrobenzo[6',7']isoindolo[5',4':2,3]cyclopropa[1,2-*b*]indol-3b(2*H*)-yl)-*N*,*N*-dimethylaniline (B24)

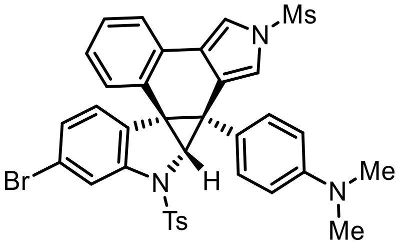

B24

Compound **B24** was prepared according to the general procedure (0 °C, 48 h) in 99% yield (104.6 mg) as a white solid (mp 162–164 °C). $[\alpha]_D^{20}$ = +238.2 (c = 1.0, $CHCl_3$). 99% ee. (determined by HPLC: Chiralpak IC Column, 20/80 *i*-PrOH/hexane, 1.0 mL/min, 254 nm; TR = 21.72 min (minor), 23.85 min (major)). ^{1}H NMR (400 MHz, $CDCl_3$) δ 7.59 (d, *J* = 7.6 Hz, 1H), 7.54 (d, *J* = 1.6 Hz, 1H), 7.45 (d, *J* = 2.4 Hz, 1H), 7.39 – 7.36 (m, 2H), 7.25 (s, 1H), 7.24 – 7.18 (m, 1H), 7.16 (d, *J* = 8.0 Hz, 1H), 7.13 – 7.10 (m, 1H), 7.06 – 7.03 (m, 1H), 7.03 – 7.00 (m, 2H), 6.80 (d, *J* = 7.6 Hz, 1H), 6.66

(d, *J* = 2.4 Hz, 1H), 6.64 – 6.11 (m, 3H), 4.00 (s, 1H), 3.14 (s, 3H), 2.83 (s, 6H), 2.29 (s, 3H); ^{13}C NMR (100 MHz, $CDCl_3$) δ 149.2, 144.3, 143.0, 133.8, 131.8, 131.0, 130.4, 129.5, 127.5, 127.3, 127.2, 126.9, 126.74, 126.65, 126.4, 126.0, 123.2, 122.2, 121.7, 120.5, 119.9, 119.3, 113.8, 111.9, 55.7, 42.8, 40.2, 40.0, 30.8, 21.4; IR (neat): 2925(bs), 1613, 1524, 1482, 1364, 1171, 1075, 1048, 984, 813 cm^{-1}; HRESIMS Calcd for $[C_{35}H_{30}BrN_3NaO_4S_2]^+$ (M + Na$^+$) 722.0753 (isotopic ion: 724.0737), found 722.0748 (isotopic ion: 724.0720).

N**,*****N*****-dimethyl-4-((3b*****R*****,3c*****S*****,8b*****S*****)-6-methyl-2-(methylsulfonyl)-4-tosyl-3c,4-dihydrobenzo[6',7']isoindolo[5',4':2,3]cyclopropa[1,2-*****b*****]indol-3b(2*****H*****)-yl)aniline (B25)**

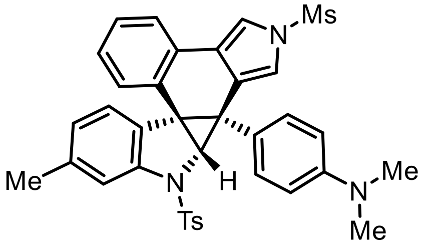

B25

Compound **B25** was prepared according to the general procedure (0 °C, 24 h) in 96% yield (91.5 mg) as a white solid (mp 159–160 °C). $[\alpha]_D^{20}$ = +303.1 (c = 1.0, $CHCl_3$). 99% ee. (determined by HPLC: Chiralpak IA Column, 30/70 *i*-PrOH/hexane, 1.0 mL/min, 254 nm; TR = 11.14 min (major), 12.72 min (minor)). ^{1}H NMR (400 MHz, $CDCl_3$) δ 7.58 (d, *J* = 7.2 Hz, 1H), 7.43 (d, *J* = 2.4 Hz, 1H), 7.29 (d, *J* = 8.4 Hz, 2H), 7.25 – 7.22 (m, 2H), 7.22 – 7.16 (m, 1H), 7.06 (s, 1H), 7.04 – 6.99 (m, 1H), 6.94 (d, *J* = 8.0 Hz, 2H), 6.82 – 6.79 (m, 1H), 6.78 (d, *J* = 8.0 Hz, 1H), 6.63 (d, *J* = 2.4 Hz, 1H), 6.62 – 6.10 (m, 3H), 3.96 (s, 1H), 3.14 (s, 3H), 2.81 (s, 6H), 2.27 (s, 3H), 2.23 (s, 3H); ^{13}C NMR (100 MHz, $CDCl_3$) δ 149.0, 143.7, 139.4, 134.1, 133.7, 132.9, 131.2, 131.1, 129.3, 128.1, 127.7, 127.1, 127.0, 126.5, 126.4, 126.3, 126.0, 123.1, 122.9, 122.0, 119.2, 117.4, 113.7, 111.9, 55.5, 42.9, 40.34, 40.30, 31.2, 21.3, 21.1; IR (neat): 2925(bs), 1613,

1525, 1494, 1362, 1329, 1264, 1171, 1076, 984, 809, 771 cm^{-1}; HRESIMS Calcd for $[C_{36}H_{33}N_3NaO_4S_2]^+$ (M + Na$^+$) 658.1805, found 658.1793.

***N*,*N*-dimethyl-4-((3b*R*,3c*S*,8b*S*)-2-(methylsulfonyl)-4-(phenylsulfonyl)-3c,4-dihydrobenzo[6',7']isoindolo[5',4':2,3]cyclopropa[1,2-*b*]indol-3b(2*H*)-yl)aniline (B26)**

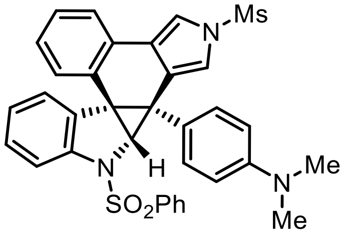

B26

Compound **B26** was prepared according to the general procedure (0 °C, 36 h) in 94% yield (85.9 mg) as a white solid (mp 168–170 °C). $[\alpha]_D^{20}$ = +315.6 (c = 1.0, $CHCl_3$). 98% ee. (determined by HPLC: Chiralpak IC Column, 50/50 *i*-PrOH/hexane, 1.0 mL/min, 254 nm; TR = 9.77 min (minor), 14.01 min (major)). ^{1}H NMR (400 MHz, $CDCl_3$) δ 7.63 (d, *J* = 7.6 Hz, 1H), 7.51 (s, 1H), 7.49 (d, *J* = 1.6 Hz, 2H), 7.46 – 7.37 (m, 2H), 7.34 – 7.31 (m, 1H), 7.28 (s, 1H), 7.26 – 7.20 (m, 3H), 7.10 – 7.00 (m, 3H), 6.87 (d, *J* = 7.6 Hz, 1H), 6.70 (d, *J* = 2.4 Hz, 1H), 6.69 – 6.08 (m, 3H), 4.08 (s, 1H), 3.18 (s, 3H), 2.84 (s, 6H); ^{13}C NMR (100 MHz, $CDCl_3$) δ 149.0, 141.6, 137.0, 133.0, 132.7, 131.1, 131.0, 128.7, 127.7, 127.4, 127.0, 126.84, 126.76, 126.6, 126.1, 126.0, 124.4, 123.1, 122.7, 122.0, 119.3, 117.3, 113.8, 111.9, 55.3, 42.9, 40.3, 31.0; IR (neat): 2926(bs), 1612, 1525, 1482, 1363, 1265, 1172, 1075, 1047, 984, 813, 769 cm^{-1}; HRESIMS Calcd for $[C_{34}H_{29}N_3NaO_4S_2]^+$ (M + Na$^+$) 630.1492, found 630.1482.

4-((3b*R*,3c*S*,8b*S*)-2,4-bis(methylsulfonyl)-3c,4-dihydrobenzo[6',7']isoindolo[5',4':2,3]cyclopropa[1,2-*b*]indol-3b(2*H*)-yl)-*N*,*N*-dimethylaniline (B27)

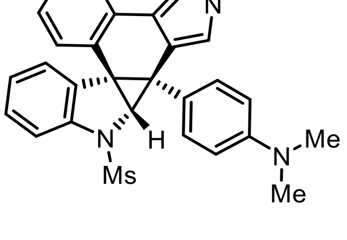

B27

Compound **B27** was prepared according to the general procedure (0 °C, 24 h) in 95% yield (78.8 mg) as a white solid (mp 173–176 °C). $[\alpha]_D^{20}$ = +531.8 (c = 1.0, $CHCl_3$). 99% ee. (determined by HPLC: Chiralpak IA Column, 50/50 *i*-PrOH/hexane, 1.0 mL/min, 254 nm; TR = 8.05 min (minor), 10.12 min (major)). ^{1}H NMR (400 MHz, $CDCl_3$) δ 7.68 (d, *J* = 7.6 Hz, 1H), 7.60 (d, *J* = 7.6 Hz, 1H), 7.53 (d, *J* = 7.2 Hz, 1H), 7.48 (d, *J* = 2.0 Hz, 1H), 7.34 – 7.28 (m, 1H), 7.27 – 7.22 (m, 2H), 7.20 (d, *J* = 7.6 Hz, 1H), 7.15 – 6.88 (m, 3H), 6.68 (d, *J* = 2.0 Hz, 1H), 6.54 – 6.29 (m, 2H), 4.05 (s, 1H), 3.14 (s, 3H), 2.81 (s, 6H), 2.77 (s, 3H); ^{13}C NMR (100 MHz, $CDCl_3$) δ 149.1, 141.7, 131.8, 131.2, 131.0, 127.8, 127.7, 127.2, 127.0, 126.7, 126.5, 124.1, 123.5, 122.3, 121.8, 119.2, 115.6, 113.9, 111.8, 55.2, 42.8, 40.4, 40.2, 37.1, 30.6; IR (neat): 2927(bs), 1612, 1525, 1482, 1365, 1265, 1165, 1075, 1047, 983, 771 cm^{-1}; HRESIMS Calcd for $[C_{29}H_{27}N_3NaO_4S_2]^+$ (M + Na$^+$) 568.1335, found 568.1329.

tert-butyl (3bR,3cS,8bS)-3b-(4-(dimethylamino)phenyl)-2-(methylsulfonyl)-3b,3c-dihydrobenzo[6',7']isoindolo[5',4':2,3]cyclopropa[1,2-*b*]indole-4(2H)-carboxylate (B28)

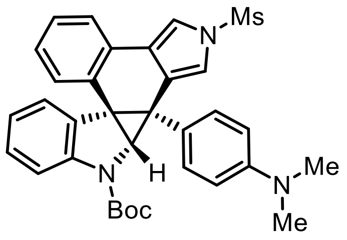

B28

Compound **B28** was prepared according to the general procedure (0 °C, 13 h) in 98% yield (83.7 mg) as a white solid (mp 207–210 °C). $[\alpha]_D^{20}$ = +633.6 (c = 1.0, $CHCl_3$). 97% ee. (determined by HPLC: Chiralpak IC Column, 30/70 *i*-PrOH/hexane, 1.0 mL/min, 254 nm; TR = 7.60 min (minor), 8.74 min (major)). ^{1}H NMR (400 MHz, $CDCl_3$) δ 7.72 – 7.63 (m, 2H), 7.53 – 7.45 (m, 2H), 7.39 – 7.18 (m, 4H), 7.08 – 7.03 (m, 1H), 7.02 – 6.98 (m, 1H), 6.77 – 6.71 (m, 1H), 6.70 – 5.92 (m, 3H), 4.07 (s, 0.4H), 3.93 (s, 0.6H), 3.23 – 3.09 (m, 3H), 2.95 – 2.79 (m, 6H), 1.71 – 1.60 (m, 9H); ^{13}C NMR

(125 MHz, $CDCl_3$) δ 152.9, 151.9, 149.1, 142.9, 141.8, 131.9, 131.8, 131.0, 130.9, 130.1, 128.4, 128.2, 127.6, 127.5, 127.4, 127.2, 127.1, 126.9, 126.73, 126.70, 126.5, 126.0, 123.4, 123.32, 123.29, 122.4, 122.2, 121.9, 121.8, 118.7, 118.5, 114.89, 114.85, 113.8, 113.7, 111.8, 82.2, 81.2, 52.8, 52.6, 42.8, 42.7, 40.4, 40.3, 39.6, 31.5, 31.2, 28.5, 28.3; IR (neat): 2927(bs), 1702, 1612, 1524, 1476, 1368, 1262, 1172, 1076, 983, 812 cm^{-1}; HRESIMS Calcd for $[C_{33}H_{33}N_3NaO_4S]^+$ (M + Na^+) 590.2084, found 590.2073.

***N,N*-dimethyl-4-((3b*R*,3c*S*,8b*S*)-2-(methylsulfonyl)-2*H*-benzo[*e*]benzofuro[3',2':1,3]cyclopropa[1,2-*g*]isoindol-3b(3c*H*)-yl)aniline (B29)**

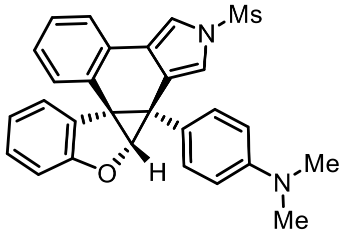

B29

Compound **B29** was prepared according to the general procedure (0 °C, 26 h) in 55% yield 40.0 mg) as a white solid (mp 109–110 °C). $[\alpha]_D^{20}$ = +432.8 (c = 1.0, $CHCl_3$). 94% ee. (determined by HPLC: Chiralpak IA Column, 50/50 *i*-PrOH/hexane, 1.0 mL/min, 254 nm; TR = 6.48 min (minor), 11.65 min (major)). ^{1}H NMR (400 MHz, $CDCl_3$) δ 7.70 (d, *J* = 7.2 Hz, 1H), 7.65 (d, *J* = 7.2 Hz, 1H), 7.46 (d, *J* = 2.0 Hz, 1H), 7.43 (d, *J* = 7.2 Hz, 1H), 7.31 – 7.24 (m, 3H), 6.97 – 6.93 (m, 1H), 6.88 – 6.84 (m, 1H), 6.69 (d, *J* = 2.0 Hz, 1H), 6.58 (d, *J* = 8.0 Hz, 1H), 6.56 – 6.21 (m, 3H), 4.49 (s, 1H), 3.11 (s, 3H), 2.82 (s, 6H); ^{13}C NMR (100 MHz, $CDCl_3$) δ 160.0, 149.1, 131.5, 131.2, 127.88, 127.87, 127.7, 127.2, 126.7, 126.5, 126.4, 125.8, 123.3, 122.8, 122.5, 120.5, 118.7, 113.8, 111.9, 109.9, 73.1, 42.8, 42.4, 40.4, 30.3; IR (neat): 2925(bs), 1612, 1524, 1483, 1493, 1365, 1263, 1172, 1076, 983, 811, 770, 750 cm^{-1}; HRESIMS Calcd for $[C_{28}H_{24}N_2NaO_3S]^+$ (M + Na^+) 491.1400, found 491.1399.

***N,N*-dimethyl-4-((3b*R*,3c*S*,8b*S*)-2-(methylsulfonyl)-2*H*-benzo[*e*]benzo[4',5']thieno[3',2':1,3]cyclopropa[1,2-*g*]isoindol-3b(3c*H*)-yl)aniline (B30)**

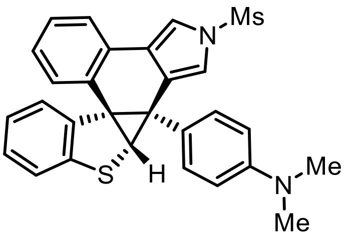

B30

Compound **B30** was prepared according to the general procedure (0 °C, 44 h) in 99% yield (72.5 mg) as a white solid (mp 138–140 °C). $[\alpha]_D^{20}$ = +331.6 (c = 1.0, $CHCl_3$). 61% ee. (determined by HPLC: Chiralpak IC Column, 20/80 *i*-PrOH/hexane, 1.0 mL/min, 254 nm; TR = 10.13 min (minor), 12.09 min (major)). ^{1}H NMR (400 MHz, $CDCl_3$) δ 7.66 (d, *J* = 7.6 Hz, 1H), 7.62 – 7.35 (m, 4H), 7.30 – 7.26 (m, 1H), 7.23 – 7.18 (m, 1H), 7.07 – 7.02 (m, 1H), 6.99 – 6.94 (m, 1H), 6.85 (d, *J* = 8.0 Hz, 1H), 6.66 (d, *J* = 2.0 Hz, 1H), 6.65 – 6.05 (m, 3H), 3.08 (s, 3H), 2.94 (s, 1H), 2.83 (s, 6H); ^{13}C NMR (100 MHz, $CDCl_3$) δ 148.9, 143.7, 138.8, 133.5, 131.7, 129.1, 128.8, 128.1, 127.3, 127.0, 126.8, 126.3, 123.7, 123.6, 123.3, 121.9, 121.8, 118.7, 113.9, 111.2, 49.2, 42.7, 42.4, 40.3, 30.4; IR (neat): 2925(bs), 1612, 1523, 1479, 1365, 1264, 1172, 1077, 983, 767 cm^{-1}; HRESIMS Calcd for $[C_{28}H_{24}N_2NaO_2S_2]^+$ (M + Na^+) 507.1171, found 507.1166.

***N*,*N*-dimethyl-4-((3b*R*,3c*R*,6a*S*)-2-(methylsulfonyl)-2*H*-benzo[*e*]thieno[3',2':1,3]cyclopropa[1,2-*g*]isoindol-3b(3c*H*)-yl)aniline (B31)**

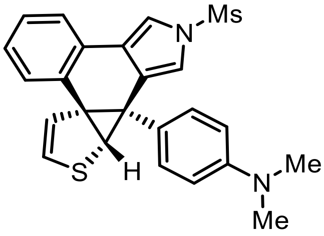

B31

Compound **B31** was prepared according to the general procedure (0 °C, 21 h) in 43% yield (22.3 mg) as a white solid (mp 135–137 °C). $[\alpha]_D^{20}$ = +384.3 (c = 1.0, $CHCl_3$). 97% ee. (determined by HPLC: Chiralpak IB Column, 50/50 *i*-PrOH/hexane, 1.0 mL/min, 254 nm; TR = 6.48 min (major), 7.89 min (minor)). ^{1}H NMR (400 MHz,

CDCl3) δ 7.69 – 7.50 (m, 2H), 7.47 – 7.42 (m, 2H), 7.30 – 7.26 (m, 2H), 7.00 – 6.85 (m, 1H), 6.72 – 6.58 (m, 3H), 5.96 – 5.86 (m, 2H), 3.11 (s, 3H), 2.94 (s, 6H), 2.87 (d, *J* = 1.6 Hz, 1H); ^{13}C NMR (100 MHz, CDCl3) δ 149.2, 134.1, 131.6, 129.2, 129.0, 128.0, 127.3, 126.8, 125.7, 124.6, 123.8, 123.2, 122.0, 118.6, 113.9, 111.1, 51.2, 42.83, 42.76, 40.4, 27.5; IR (neat): 2925(bs), 1612, 1522, 1478, 1363, 1264, 1172, 1074, 983, 947, 766 cm^{-1}; HRESIMS Calcd for $[C_{24}H_{22}N_2NaO_2S_2]^+$ (M + Na$^+$) 457.1015, found 457.1015.

***N*,*N*-dimethyl-4-((7b*S*,7c*S*,12a*R*)-6-(methylsulfonyl)-6*H*-benzo[*e*]benzofuro[2',3': 1,3]cyclopropa[1,2-*g*]isoindol-7b(7c*H*)-yl)aniline (B32)**

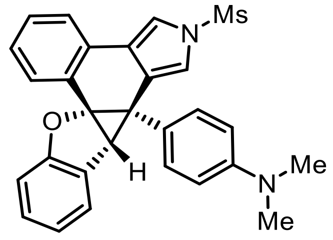

B32

Compound **B32** was prepared according to the general procedure (0 °C, 17 h) in 99% yield (72.3 mg) as a white solid (mp 127–129 °C). $[\alpha]_D^{20}$ = +303.7 (c = 1.0, CHCl3). 96% ee. (determined by HPLC: Chiralpak IC Column, 50/50 *i*-PrOH/hexane, 1.0 mL/min, 254 nm; TR = 5.47 min (minor), 6.55 min (major)). ^{1}H NMR (400 MHz, CDCl3) δ 7.93 – 7.86 (m, 1H), 7.70 – 7.65 (m, 1H), 7.53 (d, *J* = 2.0 Hz, 1H), 7.40 – 7.32 (m, 3H), 7.07 (d, *J* = 8.8 Hz, 2H), 7.01 – 6.96 (m, 1H), 6.86 (d, *J* = 7.2 Hz, 1H), 6.84 (d, *J* = 2.0 Hz, 1H), 6.70 (d, *J* = 8.0 Hz, 1H), 6.49 (d, *J* = 8.8 Hz, 2H), 3.12 (s, 3H), 2.85 (s, 6H), 2.70 (s, 1H); ^{13}C NMR (100 MHz, CDCl3) δ 159.3, 149.1, 131.8, 131.6, 129.4, 128.2, 127.3, 127.2, 127.1, 126.1, 125.0, 124.9, 123.0, 122.5, 121.9, 120.8, 117.9, 113.8, 111.9, 109.5, 75.4, 42.8, 40.3, 38.6, 29.8, 29.6; IR (neat): 2926(bs), 1613, 1525, 1476, 1365, 1234, 1171, 1077, 982, 815 cm^{-1}; HRESIMS Calcd for $[C_{28}H_{24}N_2NaO_3S]^+$ (M + Na$^+$) 491.1400, found 491.1399.

***N*,*N*-dimethyl-4-((7b*S*,7c*S*,12a*R*)-6-(methylsulfonyl)-6*H*-benzo[*e*]benzo[4',5']thieno[2',3':1,3]cyclopropa[1,2-*g*]isoindol-7b(7c*H*)-yl)aniline (B33)**

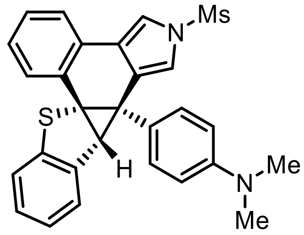

B33

Compound **B33** was prepared according to the general procedure (0 °C, 27 h) in 99% yield (71.8 mg) as a white solid (mp 141–143 °C). $[\alpha]_D^{20}$ = +428.4 (c = 1.0, $CHCl_3$). 99% ee. (determined by HPLC: Chiralpak IB Column, 15/85 *i*-PrOH/hexane, 1.0 mL/min, 254 nm; TR = 10.42 min (minor), 11.70 min (major)). ^{1}H NMR (400 MHz, $CDCl_3$) δ 8.07 – 7.96 (m, 1H), 7.68 – 7.60 (m, 1H), 7.46 (d, *J* = 2.0 Hz, 1H), 7.36 (d, *J* = 7.2 Hz, 1H), 7.30 – 7.23 (m, 2H), 7.12 (d, *J* = 8.0 Hz, 1H), 7.06 – 7.01 (m, 1H), 7.01 – 6.96 (m, 1H), 6.90 (d, *J* = 7.6 Hz, 1H), 6.75 (d, *J* = 8.0 Hz, 1H), 6.69 – 6.54 (m, 2H), 6.30 (d, *J* = 7.6 Hz, 1H), 3.09 (s, 3H), 2.86 (s, 1H), 2.84 (s, 6H); ^{13}C NMR (100 MHz, $CDCl_3$) δ 149.0, 141.6, 138.1, 133.12, 133.09, 130.8, 130.0, 129.4, 127.4, 126.81, 126.75, 126.0, 125.8, 124.6, 124.0, 123.4, 121.8, 121.3, 118.4, 113.8, 112.5, 110.5, 49.9, 47.1, 42.8, 40.3, 32.0; IR (neat): 2925(bs), 1612, 1522, 1466, 1364, 1263, 1171, 1075, 982, 805, 768 cm^{-1}; HRESIMS Calcd for $[C_{28}H_{24}N_2NaO_2S_2]^+$ (M + Na^+) 507.1171, found 507.1170.

***N*,*N*-dimethyl-4-((3b*S*,3c*S*,6a*R*)-2-(methylsulfonyl)-2*H*-benzo[*e*]thieno[2',3':1,3]cyclopropa[1,2-*g*]isoindol-3b(3c*H*)-yl)aniline (B34)**

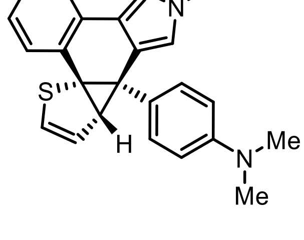

B34

Compound **B34** was prepared according to the general procedure (0 °C, 26 h) in 62% yield (33.4 mg) as a white solid (mp 126–128 °C). $[\alpha]_D^{20}$ = +276.1 (c = 1.0, $CHCl_3$). 95% ee. (determined by HPLC: Chiralpak IA Column, 50/50 *i*-PrOH/hexane, 1.0 mL/min, 254 nm; TR = 6.76 min (minor), 13.59 min (major)). ^{1}H NMR (400 MHz, $CDCl_3$) δ 8.02 – 7.82 (m, 1H), 7.64 – 7.59 (m, 1H), 7.43 (d, *J* = 2.4 Hz, 1H), 7.33 – 7.28 (m, 1H), 7.27 – 7.25 (m, 1H), 7.22 – 7.08 (m, 2H), 6.73 – 6.56 (m, 3H), 5.88 – 5.77 (m, 2H), 3.10 (s, 3H), 2.94 (s, 6H), 2.41 (d, *J* = 2.4 Hz, 1H); ^{13}C NMR (100 MHz, $CDCl_3$) δ 149.2, 133.4, 132.8, 131.5, 130.2, 129.2, 127.4, 127.2, 126.6, 125.8, 125.0, 123.3, 121.9, 121.2, 118.3, 113.7, 112.4, 110.6, 51.6, 48.0, 42.7, 40.4, 29.5; IR (neat): 2925(bs), 1612, 1523, 1474, 1364, 1264, 1171, 1052, 983, 768 cm^{-1}; HRESIMS Calcd for $[C_{24}H_{22}N_2NaO_2S_2]^+$ (M + Na^+) 457.1015, found 457.1014.

General procedure for the synthesis of chiral 1,2-dioxolanes C:

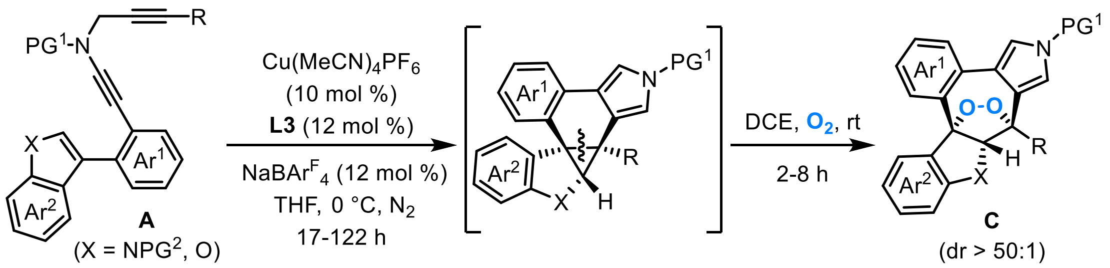

To the mixture of $Cu(MeCN)_4PF_6$ (0.015 mmol, 5.6 mg), **L3** (0.018 mmol, 14.2 mg), and $NaBAr^F{}_4$ (0.018 mmol, 15.9 mg) were introduced into an oven-dried Schlenk tube under nitrogen atmosphere. After THF (1 mL) was injected into the Schlenk tube, the solution was stirred at rt under the argon atmosphere for 2 h. Then the reaction was cooled to 0 °C, and *N*-propargyl ynamide **A** (0.15 mmol) in THF (2 mL) was introduced into the system dropwise. The resulting mixture was stirred at indicating temperature. The reaction was monitored by TLC and once it confirmscomplete consumption of **A**, DCE (1 mL) was added and the reaction atmosphere was changed from nitrogen to oxygen and the reaction was heated to rt. Once the TLC confirms complete consumption of the product from the previous reaction and concentrated under reduced

pressure. The residue was purified by flash chromatography on silica gel (eluent: hexanes/EA or hexanes/DCM) to give the final product **C**.

***N*,*N*-dimethyl-4-((8*S*,8a*S*,13b*S*)-6-(methylsulfonyl)-9-tosyl-8a,9-dihydro-8,13b-epidioxybenzo[3,4]pyrrolo[3',4':5,6]cyclohepta[1,2-*b*]indol-8(6*H*)-yl)aniline (C1)**

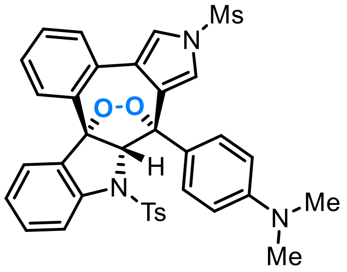

C1

Compound **C1** was prepared according to the general procedure (29 h) in 86% yield (84.7 mg) as a white solid (mp 169–171 °C). $[\alpha]_D^{20}$ = +178.0 (c = 1.0, $CHCl_3$). 98% ee. (determined by HPLC: Chiralpak IA Column, 50/50 *i*-PrOH/hexane, 1.0 mL/min, 254 nm; TR = 14.56 min (major), 29.07 min (minor)). ^{1}H NMR (400 MHz, $CDCl_3$) δ 7.81 (d, *J* = 8.0 Hz, 1H), 7.63 (d, *J* = 2.4 Hz, 1H), 7.55 (d, *J* = 8.0 Hz, 1H), 7.45 (d, *J* = 7.6 Hz, 1H), 7.42 – 7.38 (m, 1H), 7.34 – 7.13 (m, 4H), 6.99 – 6.93 (m, 3H), 6.85 (d, *J* = 2.4 Hz, 1H), 6.77 (d, *J* = 8.0 Hz, 2H), 6.74 – 6.56 (m, 3H), 5.48 (s, 1H), 3.10 (s, 3H), 2.96 (s, 6H), 2.21 (s, 3H); ^{13}C NMR (100 MHz, $CDCl_3$) δ 150.1, 145.0, 143.9, 132.5, 132.1, 131.7, 130.51, 130.47, 129.4, 129.0, 128.7, 127.4, 127.3, 127.2, 127.1, 126.6, 126.21, 126.15, 121.8, 120.5, 118.9, 117.8, 111.2, 94.8, 91.4, 85.0, 42.9, 40.2, 21.3; IR (neat): 2926(bs), 1613, 1524, 1461, 1364, 1262, 1171, 1084, 1015, 980, 809 cm^{-1}; HRESIMS Calcd for $[C_{35}H_{31}N_3NaO_6S_2]^+$ (M + Na^+) 676.1546, found 676.1545.

4-((8*S*,8a*S*,13b*S*)-6,9-ditosyl-8a,9-dihydro-8,13b-epidioxybenzo[3,4]pyrrolo[3',4':5,6]cyclohepta[1,2-*b*]indol-8(6*H*)-yl)-*N*,*N*-dimethylaniline (C2)

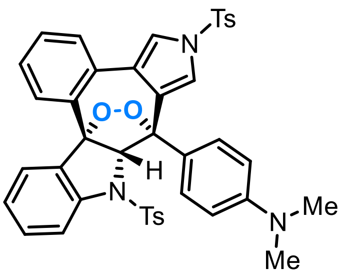

C2

Compound **C2** was prepared according to the general procedure (125 h) in 77% yield (83.9 mg) as a white solid (mp 155–157 °C). $[\alpha]_D^{20}$ = +179.7 (c = 1.0, $CHCl_3$). 93% ee. (determined by HPLC: Chiralpak IA Column, 50/50 *i*-PrOH/hexane, 1.0 mL/min, 254 nm; TR = 11.05 min (major), 15.94 min (minor)). ^{1}H NMR (400 MHz, $CDCl_3$) δ 7.80 (d, *J* = 8.0 Hz, 1H), 7.75 (d, *J* = 8.4 Hz, 2H), 7.64 (d, *J* = 2.0 Hz, 1H), 7.52 (d, *J* = 8.0 Hz, 1H), 7.42 (d, *J* = 7.6 Hz, 1H), 7.41 – 7.33 (m, 2H), 7.32 – 7.23 (m, 5H), 6.97 (d, *J* = 8.0 Hz, 2H), 6.95 – 6.89 (m, 2H), 6.84 – 6.65 (m, 4H), 6.63 (d, *J* = 7.6 Hz, 1H), 5.43 (s, 1H), 3.01 (s, 6H), 2.39 (s, 3H), 2.21 (s, 3H); ^{13}C NMR (100 MHz, $CDCl_3$) δ 150.2, 145.5, 145.0, 143.9, 135.4, 132.7, 132.3, 131.7, 130.5, 130.4, 130.2, 129.2, 129.0, 128.6, 127.40, 127.37, 127.12, 127.06, 126.6, 126.10, 126.07, 122.1, 120.6, 118.8, 117.8, 111.1, 94.7, 91.5, 85.1, 40.3, 21.6, 21.3; IR (neat): 2924(bs), 1614, 1461, 1362, 1264, 1172, 1081, 1014, 810, 760, 673 cm^{-1}; HRESIMS Calcd for $[C_{41}H_{35}N_3NaO_6S_2]^+$ (M + Na^+) 752.1859, found 752.1855.

4-((8*S*,8a*S*,13b*S*)-6-((4-bromophenyl)sulfonyl)-9-tosyl-8a,9-dihydro-8,13b-epidioxybenzo[3,4]pyrrolo[3',4':5,6]cyclohepta[1,2-*b*]indol-8(6*H*)-yl)-*N*,*N*-dimethylaniline (C3)

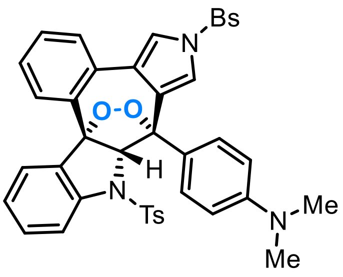

C3

Compound **C3** was prepared according to the general procedure (125 h) in 80% yield (95.2 mg) as a white solid (mp 192–194 °C). $[\alpha]_D^{20}$ = +122.2 (c = 1.0, $CHCl_3$). 93% ee. (determined by HPLC: Chiralpak IB Column, 50/50 *i*-PrOH/hexane, 1.0 mL/min, 254 nm; TR = 9.29 min (major), 13.58 min (minor)). ^{1}H NMR (400 MHz, $CDCl_3$) δ 7.79 (d, *J* = 7.6 Hz, 1H), 7.73 (d, *J* = 8.8 Hz, 2H), 7.69 – 7.63 (m, 2H), 7.62 (d, *J* = 2.4 Hz, 1H), 7.53 (d, *J* = 8.0 Hz, 1H), 7.47 – 7.34 (m, 3H), 7.29 – 7.23 (m, 3H), 7.01 – 6.92 (m, 3H), 6.91 (d, *J* = 2.4 Hz, 1H), 6.86 – 6.66 (m, 4H), 6.64 (dd, *J* = 7.6, 0.8 Hz, 1H), 5.43 (s, 1H), 3.02 (s, 6H), 2.22 (s, 3H); ^{13}C NMR (100 MHz, $CDCl_3$) δ 150.2, 145.0, 143.9, 137.4, 133.0, 132.8, 132.1, 131.9, 130.49, 130.45, 129.9, 129.7, 129.1, 128.7, 128.5, 128.0, 127.5, 127.1, 126.6, 126.3, 126.1, 122.1, 120.4, 118.9, 117.8, 111.2, 94.7, 91.5, 85.1, 40.3, 21.3; IR (neat): 2961(bs), 1732, 1614, 1525, 1463, 1393, 1263, 1081, 867, 808, 701 cm^{-1}; HRESIMS Calcd for $[C_{40}H_{32}BrN_3NaO_6S_2]^+$ (M + Na$^+$) 816.0808 (isotopic ion: 818.0794), found 816.0805 (isotopic ion: 818.0786).

4-((8*S*,8a*S*,13b*S*)-3-chloro-6-(methylsulfonyl)-9-tosyl-8a,9-dihydro-8,13b-epidioxybenzo[3,4]pyrrolo[3',4':5,6]cyclohepta[1,2-*b*]indol-8(6*H*)-yl)-*N,N*-dimethylaniline (C4)

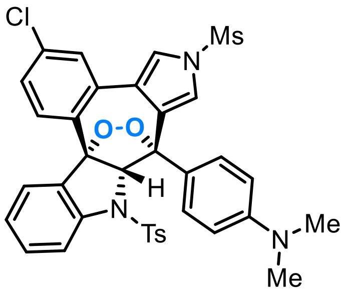

C4

Compound **C4** was prepared according to the general procedure (29 h) in 75% yield (77.3 mg) as a white solid (mp 177–178 °C). $[\alpha]_D^{20}$ = +232.1 (c = 1.0, $CHCl_3$). 99% ee. (determined by HPLC: Chiralpak IC Column, 50/50 *i*-PrOH/hexane, 1.0 mL/min, 254 nm; TR = 12.80 min (major), 19.86 min (minor)). ^{1}H NMR (400 MHz, $CDCl_3$) δ 7.80 (d, *J* = 2.0 Hz, 1H), 7.62 (d, *J* = 2.4 Hz, 1H), 7.56 (d, *J* = 8.0 Hz, 1H), 7.44 (s, 1H), 7.43 – 7.36 (m, 2H), 7.35 – 7.26 (m, 2H), 7.00 (d, *J* = 8.0 Hz, 2H), 6.94 – 6.90 (m, 1H), 6.87

(d, J = 2.4 Hz, 1H), 6.83 (d, J = 8.0 Hz, 2H), 6.77 – 6.63 (m, 2H), 6.58 (d, J = 8.4 Hz, 1H), 5.42 (s, 1H), 3.16 (s, 3H), 2.99 (s, 6H), 2.26 (s, 3H); ^{13}C NMR (100 MHz, $CDCl_3$) δ 150.2, 145.1, 144.2, 134.7, 134.1, 132.5, 130.7, 130.4, 130.1, 129.4, 129.1, 127.9, 127.5, 127.3, 126.9, 126.3, 126.1, 126.0, 122.1, 120.1, 119.1, 118.3, 111.1, 94.3, 91.4, 85.0, 43.1, 40.2, 21.4; IR (neat): 2926(bs), 1613, 1525, 1461, 1366, 1263, 1172, 1083, 1023, 982, 811 cm^{-1}; HRESIMS Calcd for $[C_{35}H_{30}ClN_3NaO_6S_2]^+$ (M + Na$^+$) 710.1157 (isotopic ion: 712.1142), found 710.1153 (isotopic ion: 712.1129).

4-((8*S*,8a*S*,13b*S*)-3-methoxy-6-(methylsulfonyl)-9-tosyl-8a,9-dihydro-8,13b-epidioxybenzo[3,4]pyrrolo[3',4':5,6]cyclohepta[1,2-*b*]indol-8(6*H*)-yl)-*N*,*N*-dimethylaniline (C5)

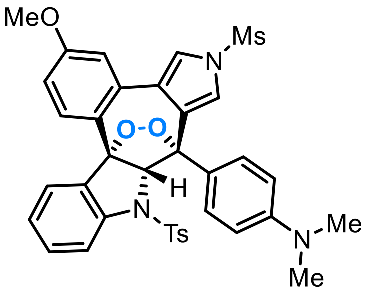

C5

Compound **C5** was prepared according to the general procedure (53 h) in 99% yield (102.5 mg) as a white solid (mp 182–183 °C). $[\alpha]_D^{20}$ = +185.1 (c = 1.0, $CHCl_3$). 97% ee. (determined by HPLC: Chiralpak IC Column, 50/50 *i*-PrOH/hexane, 1.0 mL/min, 254 nm; TR = 20.65 min (major), 33.38 min (minor)). ^{1}H NMR (400 MHz, $CDCl_3$) δ 7.62 (d, J = 2.4 Hz, 1H), 7.52 (d, J = 8.0 Hz, 1H), 7.44 (d, J = 7.6 Hz, 1H), 7.42 – 7.38 (m, 1H), 7.38 – 7.32 (m, 2H), 7.28 – 7.24 (m, 2H), 7.00 (d, J = 8.0 Hz, 2H), 6.86 (d, J = 2.4 Hz, 1H), 6.81 (d, J = 8.0 Hz, 2H), 6.75 – 6.63 (m, 2H), 6.59 (d, J = 8.8 Hz, 1H), 6.52 – 6.48 (m, 1H), 5.47 (s, 1H), 3.84 (s, 3H), 3.13 (s, 3H), 2.97 (s, 6H), 2.24 (s, 3H); ^{13}C NMR (100 MHz, $CDCl_3$) δ 159.7, 150.1, 144.9, 143.8, 133.6, 132.8, 130.8, 130.4, 129.6, 129.0, 127.9, 127.5, 127.2, 127.0, 126.1, 124.4, 121.9, 120.6, 118.8, 117.8, 113.4, 111.1, 111.0, 94.6, 91.3, 85.0, 55.5, 43.0, 40.2, 21.4; IR (neat): 2926(bs), 1612, 1524,

1461, 1363, 1262, 1171, 1084, 1021, 805, 662 cm^{-1}; HRESIMS Calcd for $[C_{36}H_{33}N_3NaO_7S_2]^+$ (M + Na^+) 706.1652, found 706.1651.

4-((8*S*,8a*S*,13b*S*)-2-chloro-6-(methylsulfonyl)-9-tosyl-8a,9-dihydro-8,13b-epidioxybenzo[3,4]pyrrolo[3',4':5,6]cyclohepta[1,2-*b*]indol-8(6*H*)-yl)-*N,N*-dimethylaniline (C6)

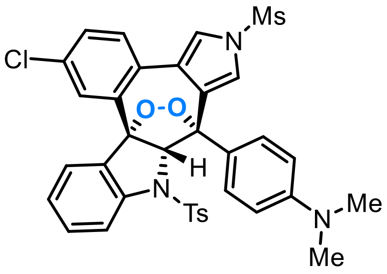

C6

Compound **C6** was prepared according to the general procedure (40 h) in 72% yield (74.3 mg) as a white solid (mp 184–185 °C). $[\alpha]_D^{20}$ = +101.7 (c = 1.0, $CHCl_3$). 98% ee. (determined by HPLC: Chiralpak IC Column, 50/50 *i*-PrOH/hexane, 1.0 mL/min, 254 nm; TR = 13.19 min (major), 22.74 min (minor)). ^1H NMR (400 MHz, $CDCl_3$) δ 7.74 (d, J = 8.4 Hz, 1H), 7.63 (d, J = 8.0 Hz, 1H), 7.60 (d, J = 2.4 Hz, 1H), 7.53 – 7.38 (m, 3H), 7.35 – 7.32 (m, 1H), 7.28 – 7.24 (m, 2H), 7.01 (d, J = 8.4 Hz, 2H), 6.89 – 6.83 (m, 3H), 6.83 – 6.62 (m, 2H), 6.55 (d, J = 2.4 Hz, 1H), 5.44 (s, 1H), 3.19 (s, 3H), 3.01 (s, 6H), 2.30 (s, 3H); ^{13}C NMR (100 MHz, $CDCl_3$) δ 150.3, 145.1, 144.7, 133.8, 132.28, 132.27, 130.84, 130.78, 130.0, 129.5, 129.1, 128.7, 128.6, 127.6, 126.8, 126.7, 126.3, 122.0, 120.1, 119.5, 117.9, 111.3, 94.3, 91.5, 85.1, 43.7, 40.3, 21.6; IR (neat): 2926(bs), 1613, 1524, 1462, 1366, 1263, 1172, 1081, 1014, 981, 810, 768 cm^{-1}; HRESIMS Calcd for $[C_{35}H_{30}ClN_3NaO_6S_2]^+$ (M + Na^+) 710.1157 (isotopic ion: 712.1142), found 710.1153 (isotopic ion: 712.1142).

***N,N*-dimethyl-4-((8*S*,8a*S*,13b*S*)-2-methyl-6-(methylsulfonyl)-9-tosyl-8a,9-dihydro-8,13b-epidioxybenzo[3,4]pyrrolo[3',4':5,6]cyclohepta[1,2-*b*]indol-8(6*H*)-yl)aniline (C7)**

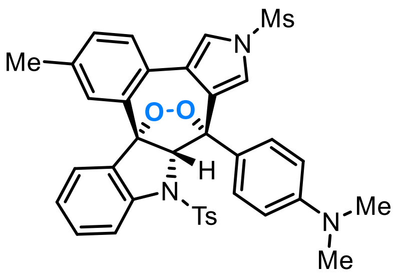

C7

Compound **C7** was prepared according to the general procedure (29 h) in 66% yield (66.4 mg) as a white solid (mp 181–182 °C). $[\alpha]_D^{20}$ = +170.4 (c = 1.0, $CHCl_3$). 98% ee. (determined by HPLC: Chiralpak IA Column, 50/50 *i*-PrOH/hexane, 1.0 mL/min, 254 nm; TR = 15.22 min (major), 24.89 min (minor)). ^{1}H NMR (400 MHz, $CDCl_3$) δ 7.71 (d, *J* = 8.0 Hz, 1H), 7.59 (d, *J* = 2.4 Hz, 1H), 7.53 (d, *J* = 8.0 Hz, 1H), 7.47 (d, *J* = 7.6 Hz, 1H), 7.44 – 7.32 (m, 2H), 7.32 – 7.25 (m, 2H), 7.08 (d, *J* = 8.0 Hz, 1H), 7.02 (d, *J* = 8.4 Hz, 2H), 6.84 (d, *J* = 2.8 Hz, 2H), 6.82 (s, 1H), 6.77 – 6.58 (m, 2H), 6.48 (s, 1H), 5.53 (s, 1H), 3.14 (s, 3H), 2.98 (s, 6H), 2.24 (s, 3H), 2.18 (s, 3H); ^{13}C NMR (100 MHz, $CDCl_3$) δ 150.1, 145.0, 143.7, 136.3, 133.1, 131.7, 130.6, 130.4, 129.4, 129.3, 129.2, 129.0, 127.6, 127.4, 127.3, 127.1, 126.2, 121.7, 120.6, 118.8, 117.3, 111.2, 94.9, 91.5, 85.0, 43.0, 40.3, 21.4, 21.0; IR (neat): 2926(bs), 1613, 1523, 1478, 1364, 1263, 1171, 1081, 1014, 982, 805, 768 cm^{-1}; HRESIMS Calcd for $[C_{36}H_{33}N_3NaO_6S_2]^+$ (M + Na^+) 690.1703, found 690.1695.

4-((8*S*,8a*S*,13b*S*)-12-chloro-6-(methylsulfonyl)-9-tosyl-8a,9-dihydro-8,13b-epidioxybenzo[3,4]pyrrolo[3',4':5,6]cyclohepta[1,2-*b*]indol-8(6*H*)-yl)-*N*,*N*-dimethylaniline (C8)

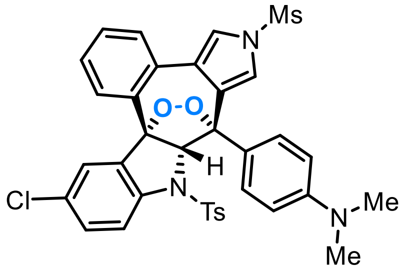

C8

Compound **C8** was prepared according to the general procedure (39 h) in 79% yield (81.4 mg) as a white solid (mp 180–182 °C). $[\alpha]_D^{20}$ = +108.9 (c = 1.0, $CHCl_3$). 98% ee. (determined by HPLC: Chiralpak IC Column, 50/50 *i*-PrOH/hexane, 1.0 mL/min, 254 nm; TR = 12.49 min (major), 29.19 min (minor)). ^{1}H NMR (500 MHz, $CDCl_3$) δ 7.82 (dd, *J* = 8.0, 1.0 Hz, 1H), 7.64 (d, *J* = 2.5 Hz, 1H), 7.50 (d, *J* = 9.0 Hz, 1H), 7.43 (d, *J* = 2.0 Hz, 1H), 7.38 (dd, *J* = 9.0, 2.0 Hz, 1H), 7.35 – 7.27 (m, 2H), 7.01 – 6.97 (m, 3H), 6.86 (d, *J* = 2.5 Hz, 1H), 6.82 (d, *J* = 8.0 Hz, 2H), 6.81 – 6.60 (m, 3H), 6.59 (dd, *J* = 7.6, 1.0 Hz, 1H), 5.48 (s, 1H), 3.17 (s, 3H), 2.99 (s, 6H), 2.26 (s, 3H); ^{13}C NMR (125 MHz, $CDCl_3$) δ 150.2, 144.3, 143.6, 132.34, 132.31, 132.0, 131.5, 131.1, 130.8, 129.3, 128.9, 127.52, 127.46, 127.1, 127.0, 126.4, 126.3, 121.9, 120.07, 120.06, 117.9, 111.2, 94.6, 91.5, 85.5, 43.1, 40.2, 21.4; IR (neat): 2924(bs), 1728, 1614, 1525, 1469, 1262, 1168, 1084, 1014, 981, 810 cm^{-1}; HRESIMS Calcd for $[C_{35}H_{30}ClN_3NaO_6S_2]^+$ (M + Na^+) 710.1157 (isotopic ion: 712.1142), found 710.1157 (isotopic ion: 712.1135).

4-((8*S*,8a*S*,13b*S*)-12-bromo-6-(methylsulfonyl)-9-tosyl-8a,9-dihydro-8,13b-epidioxybenzo[3,4]pyrrolo[3',4':5,6]cyclohepta[1,2-*b*]indol-8(6*H*)-yl)-*N*,*N*-dimethylaniline (C9)

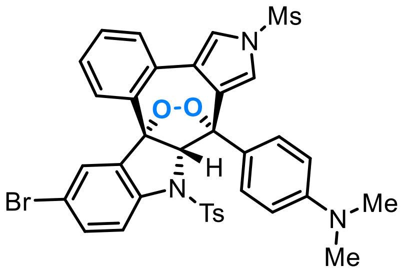

C9

Compound **C9** was prepared according to the general procedure (40 h) in 70% yield (76.4 mg) as a white solid (mp 190–192 °C). $[\alpha]_D^{20}$ = +68.0 (c = 1.0, $CHCl_3$). 98% ee. (determined by HPLC: Chiralpak IC Column, 50/50 *i*-PrOH/hexane, 1.0 mL/min, 254 nm; TR = 13.37 min (major), 33.73 min (minor)). ^{1}H NMR (400 MHz, $CDCl_3$) δ 7.83 (d, *J* = 7.6 Hz, 1H), 7.64 (d, *J* = 2.4 Hz, 1H), 7.58 (d, *J* = 2.0 Hz, 1H), 7.54 – 7.51 (m, 1H), 7.49 – 7.36 (m, 2H), 7.36 – 7.25 (m, 2H), 7.03 – 6.96 (m, 3H), 6.87 (d, *J* = 2.4 Hz,

1H), 6.83 (d, *J* = 8.0 Hz, 2H), 6.79 – 6.64 (m, 2H), 6.62 – 6.58 (m, 1H), 5.48 (s, 1H), 3.18 (s, 3H), 3.00 (s, 6H), 2.26 (s, 3H); ^{13}C NMR (100 MHz, $CDCl_3$) δ 150.2, 144.3, 144.1, 133.6, 132.7, 132.4, 132.0, 131.1, 130.0, 129.3, 129.2, 129.0, 127.53, 127.46, 127.1, 126.4, 126.3, 121.9, 120.4, 120.1, 119.0, 117.9, 111.2, 94.5, 91.5, 85.4, 43.1, 40.3, 21.4; IR (neat): 2925(bs), 1614, 1525, 1468, 1367, 1262, 1173, 1085, 1014, 981, 811 cm^{-1}; HRESIMS Calcd for $[C_{35}H_{30}BrN_3NaO_6S_2]^+$ (M + Na^+) 754.0652 (isotopic ion: 756.0636), found 754.0651 (isotopic ion: 756.0625).

***N*,*N*-dimethyl-4-((8*S*,8a*S*,13b*S*)-12-methyl-6-(methylsulfonyl)-9-tosyl-8a,9-dihydro-8,13b-epidioxybenzo[3,4]pyrrolo[3',4':5,6]cyclohepta[1,2-*b*]indol-8(6*H*)-yl)aniline (C10)**

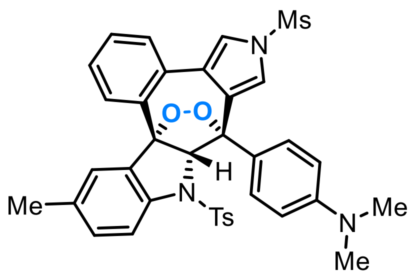

C10

Compound **C10** was prepared according to the general procedure (37 h) in 85% yield (84.8 mg) as a white solid (mp 176–178 °C). $[\alpha]_D^{20}$ = +141.6 (c = 1.0, $CHCl_3$). 98% ee. (determined by HPLC: Chiralpak IA Column, 50/50 *i*-PrOH/hexane, 1.0 mL/min, 254 nm; TR = 10.86 min (major), 21.22 min (minor)). ^{1}H NMR (400 MHz, $CDCl_3$) δ 7.81 (d, *J* = 7.6 Hz, 1H), 7.63 (d, *J* = 2.4 Hz, 1H), 7.48 – 7.36 (m, 2H), 7.30 – 7.25 (m, 2H), 7.24 – 7.19 (m, 2H), 7.00 – 6.97 (m, 2H), 6.97 – 6.92 (m, 1H), 6.84 (d, *J* = 2.4 Hz, 1H), 6.78 (d, *J* = 8.0 Hz, 2H), 6.75 – 6.60 (m, 3H), 5.46 (s, 1H), 3.14 (s, 3H), 2.98 (s, 6H), 2.39 (s, 3H), 2.23 (s, 3H); ^{13}C NMR (100 MHz, $CDCl_3$) δ 150.1, 143.8, 142.7, 136.1, 132.50, 132.48, 132.1, 131.8, 131.5, 130.5, 129.6, 129.0, 128.6, 127.5, 127.31, 127.26, 126.6, 126.2, 121.8, 120.5, 118.7, 117.8, 111.2, 94.9, 91.4, 85.2, 43.0, 40.2, 21.3, 21.2; IR (neat): 2926(bs), 1614, 1524, 1486, 1365, 1171, 1085, 1015, 981, 811, 769 cm^{-1}; HRESIMS Calcd for $[C_{36}H_{33}N_3NaO_6S_2]^+$ (M + Na^+) 690.1703, found 690.1697.

4-((8*S*,8a*S*,13b*S*)-11-chloro-6-(methylsulfonyl)-9-tosyl-8a,9-dihydro-8,13b-epidioxybenzo[3,4]pyrrolo[3',4':5,6]cyclohepta[1,2-*b*]indol-8(6*H*)-yl)-*N*,*N*-dimethylaniline (C11)

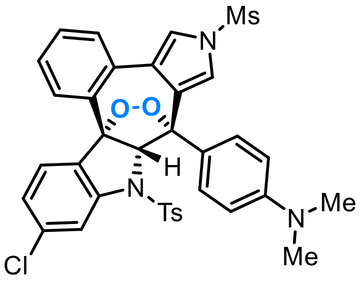

C11

Compound **C11** was prepared according to the general procedure (42 h) in 84% yield (86.7 mg) as a white solid (mp 184–185 °C). $[\alpha]_D^{20}$ = +110.1 (c = 1.0, $CHCl_3$). 98% ee. (determined by HPLC: Chiralpak IE Column, 50/50 *i*-PrOH/hexane, 1.0 mL/min, 254 nm; TR = 26.22 min (minor), 32.40 min (major)). ^{1}H NMR (400 MHz, $CDCl_3$) δ 7.83 (d, *J* = 8.0 Hz, 1H), 7.63 (d, *J* = 2.4 Hz, 1H), 7.56 (d, *J* = 1.6 Hz, 1H), 7.45 – 7.35 (m, 2H), 7.31 – 7.24 (m, 3H), 7.03 (d, *J* = 8.0 Hz, 2H), 7.00 – 6.96 (m, 1H), 6.88 (d, *J* = 2.4 Hz, 1H), 6.84 (d, *J* = 8.0 Hz, 2H), 6.79 – 6.65 (m, 2H), 6.60 (d, *J* = 7.6 Hz, 1H), 5.50 (s, 1H), 3.17 (s, 3H), 3.00 (s, 6H), 2.25 (s, 3H); ^{13}C NMR (100 MHz, $CDCl_3$) δ 150.3, 146.1, 144.3, 136.5, 132.6, 132.1, 131.4, 129.3, 129.1, 128.9, 127.9, 127.5, 127.4, 127.2 126.5, 126.4, 121.9, 120.1, 119.1, 117.9, 111.2, 94.4, 91.5, 85.6, 43.1, 40.2, 21.4; IR (neat): 2924 (bs), 1614, 1599, 1524, 1468, 1367, 1262, 1172, 1084, 1014, 985, 812 cm^{-1}; HRESIMS Calcd for $[C_{35}H_{30}ClN_3NaO_6S_2]^+$ (M + Na^+) 710.1157 (isotopic ion: 712.1142), found 710.1156 (isotopic ion: 712.1132).

4-((8*S*,8a*S*,13b*S*)-11-bromo-6-(methylsulfonyl)-9-tosyl-8a,9-dihydro-8,13b-epidioxybenzo[3,4]pyrrolo[3',4':5,6]cyclohepta[1,2-*b*]indol-8(6*H*)-yl)-*N*,*N*-dimethylaniline (C12)

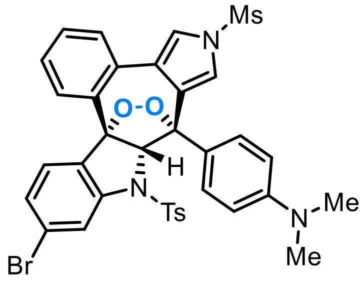

C12

Compound **C12** was prepared according to the general procedure (53 h) in 70% yield (77.1 mg) as a white solid (mp 178–180 °C). $[\alpha]_D^{20}$ = +50.2 (c = 1.0, $CHCl_3$). 98% ee. (determined by HPLC: Chiralpak IC Column, 50/50 *i*-PrOH/hexane, 1.0 mL/min, 254 nm; TR = 9.86 min (minor), 11.73 min (major)). ^{1}H NMR (400 MHz, $CDCl_3$) δ 7.82 (d, *J* = 7.6 Hz, 1H), 7.73 (d, *J* = 1.6 Hz, 1H), 7.63 (d, *J* = 2.4 Hz, 1H), 7.50 – 7.36 (m, 2H), 7.32 (d, *J* = 8.4 Hz, 1H), 7.31 – 7.25 (m, 2H), 7.03 (d, *J* = 8.0 Hz, 2H), 6.99 – 6.94 (m, 1H), 6.88 (d, *J* = 2.4 Hz, 1H), 6.84 (d, *J* = 8.0 Hz, 2H), 6.79 – 6.64 (m, 2H), 6.59 (d, *J* = 7.2 Hz, 1H), 5.48 (s, 1H), 3.16 (s, 3H), 2.99 (s, 6H), 2.25 (s, 3H); ^{13}C NMR (100 MHz, $CDCl_3$) δ 150.2, 146.2, 144.3, 132.47, 132.46, 132.1, 131.6, 129.6, 129.4, 129.3, 129.2, 128.9, 128.2, 127.5, 127.4, 127.1, 126.4, 126.3, 124.4, 122.0, 121.9, 120.0, 117.9, 111.2, 94.4, 91.5, 85.4, 43.0, 40.2, 21.4; IR (neat): 2926 (bs), 1613, 1594, 1524, 1475, 1367, 1263, 1172, 1084, 1014, 980, 811 cm^{-1}; HRESIMS Calcd for $[C_{35}H_{30}BrN_3NaO_6S_2]^+$ (M + Na$^+$) 754.0652 (isotopic ion: 756.0636), found 754.0650 (isotopic ion: 756.0631).

***N*,*N*-dimethyl-4-((8*S*,8a*S*,13b*S*)-11-methyl-6-(methylsulfonyl)-9-tosyl-8a,9-dihydro-8,13b-epidioxybenzo[3,4]pyrrolo[3',4':5,6]cyclohepta[1,2-*b*]indol-8(6*H*)-yl)aniline (C13)**

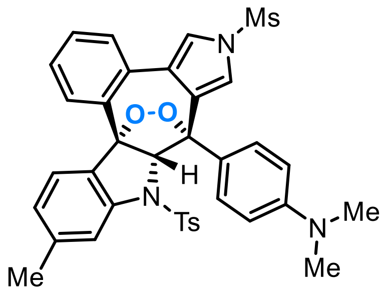

C13

Compound **C13** was prepared according to the general procedure (36 h) in 81% yield (81.0 mg) as a white solid (mp 189–190 °C). $[\alpha]_D^{20}$ = +123.6 (c = 1.0, $CHCl_3$). 98% ee. (determined by HPLC: Chiralpak IA Column, 50/50 *i*-PrOH/hexane, 1.0 mL/min, 254 nm; TR = 18.23 min (major), 38.53 min (minor)). ^{1}H NMR(400 MHz, $CDCl_3$) δ 7.81 (d, *J* = 7.6 Hz, 1H), 7.62 (d, *J* = 2.4 Hz, 1H), 7.44 – 7.32 (m, 3H), 7.28 – 7.23 (m, 2H), 7.10 (d, *J* = 8.0 Hz, 1H), 6.99 (d, *J* = 8.0 Hz, 2H), 6.97 – 6.93 (m, 1H), 6.85 (d, *J* = 2.0 Hz, 1H), 6.79 (d, *J* = 8.0 Hz, 2H), 6.76 – 6.61 (m, 3H), 5.47 (s, 1H), 3.14 (s, 3H), 2.98 (s, 6H), 2.41 (s, 3H), 2.23 (s, 3H); ^{13}C NMR (100 MHz, $CDCl_3$) δ 150.2, 145.2, 143.8, 141.0, 132.64, 132.61, 132.1, 131.9, 129.6, 129.0, 128.6, 127.7, 127.4, 127.33, 127.30, 127.28, 126.64, 126.62, 126.2, 121.8, 120.7, 119.4, 117.7, 111.2, 94.7, 91.4, 85.3, 43.0, 40.3, 21.7, 21.3; IR (neat): 2926(bs), 1614, 1525, 1365, 1262, 1172, 1084, 1015, 981, 811 cm^{-1}; HRESIMS Calcd for $[C_{36}H_{33}N_3NaO_6S_2]^+$ (M + Na^+) 690.1703, found 690.1698.

***N*,*N*-dimethyl-4-((8*S*,8a*S*,13b*S*)-6-(methylsulfonyl)-9-(phenylsulfonyl)-8a,9-dihydro-8,13b-epidioxybenzo[3,4]pyrrolo[3',4':5,6]cyclohepta[1,2-*b*]indol-8(6*H*)-yl)aniline (C14)**

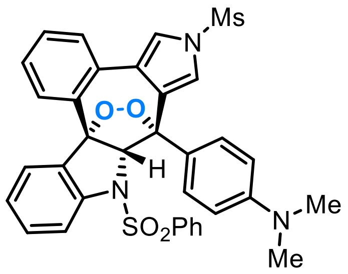

C14

Compound **C14** was prepared according to the general procedure (31 h) in 74% yield (70.7 mg) as a white solid (mp 243–245 °C). $[\alpha]_D^{20}$ = +113.3 (c = 1.0, $CHCl_3$). 98% ee. (determined by HPLC: Chiralpak IC Column, 50/50 *i*-PrOH/hexane, 1.0 mL/min, 254 nm; TR = 13.11 min (major), 25.06 min (minor)). ^{1}H NMR (400 MHz, $CDCl_3$) δ 7.87 (d, *J* = 7.6 Hz, 1H), 7.68 (d, *J* = 2.4 Hz, 1H), 7.57 (d, *J* = 8.0 Hz, 1H), 7.50 (d, *J* = 7.2 Hz, 1H), 7.46 – 7.42 (m, 1H), 7.39 – 7.25 (m, 5H), 7.17 (d, *J* = 7.6 Hz, 2H), 7.07 – 6.98 (m, 3H), 6.91 (d, *J* = 2.4 Hz, 1H), 6.80 – 6.67 (m, 3H), 5.59 (s, 1H), 3.18 (s, 3H), 3.02 (s, 6H); ^{13}C NMR (100 MHz, $CDCl_3$) δ 150.2, 144.9, 135.9, 133.0, 132.1, 131.8, 130.5, 130.4, 129.5, 128.8, 128.5, 127.5, 127.4, 127.3, 127.2, 126.7, 126.2, 121.9, 120.5, 118.7, 117.8, 111.2, 94.8, 91.5, 85.0, 43.0, 40.3; IR (neat): 2925(bs), 1613, 1525 1473, 1446, 1362, 1264, 1084, 1015, 810, 773 cm^{-1}; HRESIMS Calcd for $[C_{34}H_{29}N_3NaO_6S_2]^+$ (M + Na^+) 662.1390, found 662.1388.

4-((8*S*,8a*S*,13b*S*)-6,9-bis(methylsulfonyl)-8a,9-dihydro-8,13b-epidioxybenzo[3,4]pyrrolo[3',4':5,6]cyclohepta[1,2-*b*]indol-8(6*H*)-yl)-*N*,*N*-dimethylaniline (C15)

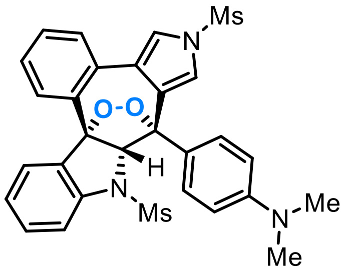

C15

Compound **C15** was prepared according to the general procedure (51 h) in 84% yield (72.7 mg) as a white solid (mp 185–188 °C). $[\alpha]_D^{20}$ = +353.8 (c = 1.0, $CHCl_3$). 99% ee. (determined by HPLC: Chiralpak IB Column, 50/50 *i*-PrOH/hexane, 1.0 mL/min, 254 nm; TR = 9.92 min (minor), 12.59 min (major)). ^{1}H NMR (400 MHz, $CDCl_3$) δ 7.89 (d, *J* = 8.0 Hz, 1H), 7.70 – 7.62 (m, 2H), 7.46 – 7.41 (m, 2H), 7.41 – 7.33 (m, 2H), 7.33 – 7.26 (m, 2H), 7.24 – 7.17 (m, 2H), 6.83 (d, *J* = 2.4 Hz, 1H), 6.81 – 6.70 (m, 1H), 6.68 – 6.51 (m, 1H), 5.87 (s, 1H), 3.16 (s, 3H), 2.97 (s, 6H), 2.16 (s, 3H); ^{13}C NMR (100 MHz, $CDCl_3$) δ 150.4, 144.8, 132.3, 131.7, 131.1, 130.6, 129.3, 128.8, 128.5, 127.6,

127.0, 124.5, 122.0, 121.1, 117.7, 116.1, 111.3, 110.7, 95.2, 91.2, 84.2, 43.0, 41.1, 40.3; IR (neat): 2927(bs), 1612, 1524, 1478, 1365, 1171, 1085, 1017, 983, 760 cm^{-1}; HRESIMS Calcd for $[C_{29}H_{27}N_3NaO_6S_2]^+$ (M + Na^+) 600.1233, found 600.1229.

***N*,*N*-dimethyl-4-((8*S*,8a*S*,13b*S*)-6-(methylsulfonyl)-6*H*-8,13b-epidioxybenzo[3,4]benzofuro[3',2':5,6]cyclohepta[1,2-*c*]pyrrol-8(8a*H*)-yl)aniline (C16)**

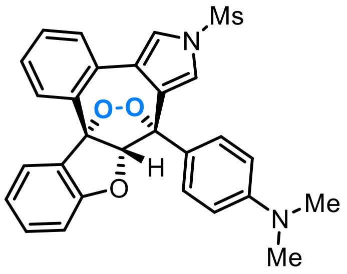

C16

Compound **C16** was prepared according to the general procedure (72 h) in 56% yield (41.9 mg) as a white solid (mp 186–188 °C). $[\alpha]_D^{20}$ = +300.5 (c = 1.0, $CHCl_3$). 95% ee. (determined by HPLC: Chiralpak IB Column, 50/50 *i*-PrOH/hexane, 1.0 mL/min, 254 nm; TR = 9.51 min (minor), 11.19 min (major)). ^1H NMR (400 MHz, DMSO-d_6) δ 8.14 (d, *J* = 8.0 Hz, 1H), 8.12 (d, *J* = 2.4 Hz, 1H), 7.71 (d, *J* = 7.6 Hz, 1H), 7.52 (d, *J* = 7.6 Hz, 1H), 7.48 – 7.33 (m, 3H), 7.32 – 7.28 (m, 1H), 7.21 – 7.10 (m, 2H), 6.91 (d, *J* = 8.4 Hz, 1H), 6.83 – 6.71 (m, 2H), 6.48 (d, *J* = 2.4 Hz, 1H), 6.27 (s, 1H), 3.59 (s, 3H), 2.95 (s, 6H); ^{13}C NMR (100 MHz, DMSO-d_6) δ 161.2, 149.8, 132.5, 131.4, 130.5, 129.4, 128.8, 127.8, 127.61, 127.59, 127.0, 126.9, 126.6, 125.7, 123.6, 121.8, 121.43, 121.37, 118.8, 112.2, 111.2, 110.4, 98.4, 95.9, 90.4, 42.2, 39.9; IR (neat): 2925(bs), 1594, 1517, 1474, 1374, 1208, 1173, 1081, 1013, 988, 809 cm^{-1}; HRESIMS Calcd for $[C_{28}H_{24}N_2NaO_5S]^+$ (M + Na^+) 523.1298, found 523.1298.

***N*,*N*-dibenzyl-4-((8*S*,8a*S*,13b*S*)-6-(methylsulfonyl)-9-tosyl-8a,9-dihydro-8,13b-epidioxybenzo[3,4]pyrrolo[3',4':5,6]cyclohepta[1,2-*b*]indol-8(6*H*)-yl)aniline (C17)**

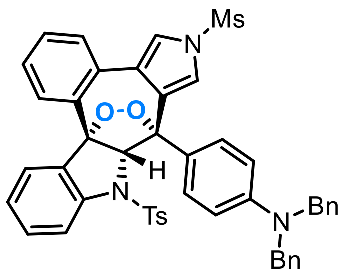

C17

Compound **C17** was prepared according to the general procedure (42 h) in 87% yield (105.5 mg) as a white solid (mp 145–147 °C). $[\alpha]_D^{20}$ = +100.6 (c = 1.0, $CHCl_3$). 95% ee. (determined by HPLC: Chiralpak IB Column, 50/50 *i*-PrOH/hexane, 1.0 mL/min, 254 nm; TR = 12.02 min (minor), 13.42 min (major)). ^{1}H NMR (400 MHz, $CDCl_3$) δ 7.80 (d, *J* = 7.6 Hz, 1H), 7.61 (d, *J* = 2.4 Hz, 1H), 7.52 (d, *J* = 8.0 Hz, 1H), 7.44 (d, *J* = 7.6 Hz, 1H), 7.41 – 7.38 (m, 1H), 7.37 – 7.35 (m, 1H), 7.35 – 7.31 (m, 4H), 7.29 (d, *J* = 1.6 Hz, 3H), 7.28 – 7.23 (m, 6H), 7.00 (d, *J* = 8.4 Hz, 2H), 6.96 – 6.93 (m, 1H), 6.90 (d, *J* = 2.4 Hz, 1H), 6.79 (d, *J* = 8.0 Hz, 4H), 6.64 (d, *J* = 6.8 Hz, 1H), 5.47 (s, 1H), 4.67 (s, 4H), 3.15 (s, 3H), 2.22 (s, 3H); ^{13}C NMR (100 MHz, $CDCl_3$) δ 149.4, 145.0, 143.9, 138.4, 132.7, 132.1, 131.8, 130.5, 129.4, 129.1, 128.7, 128.6, 127.5, 127.4, 127.1, 126.9, 126.6, 126.3, 126.2, 121.8, 121.1, 118.9, 117.8, 111.6, 94.8, 91.4, 85.0, 53.9, 43.0, 21.3; IR (neat): 2926(bs), 1613, 1521, 1454, 1366, 1264, 1172, 1084, 1014, 810, 765 cm^{-1}; HRESIMS Calcd for $[C_{47}H_{39}N_3NaO_6S_2]^+$ (M + Na^+) 828.2172, found 828.2167.

General procedure for the synthesis of chiral cyclohepta[*b*]indols D:

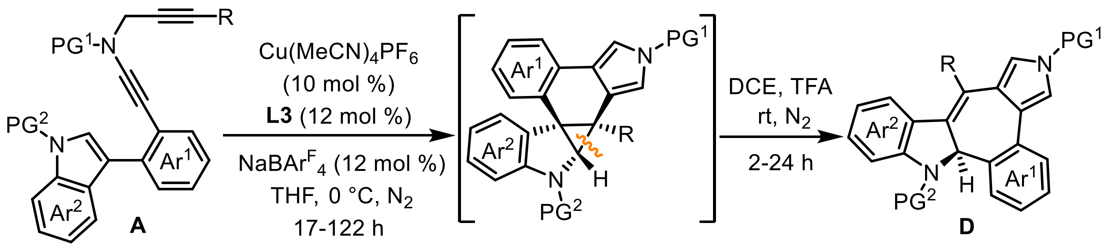

To the mixture of $Cu(MeCN)_4PF_6$ (0.015 mmol, 5.6 mg), **L3** (0.018 mmol, 14.2 mg), and $NaBAr^F{}_4$ (0.018 mmol, 15.9 mg) were introduced into an oven-dried Schlenk tube under nitrogen atmosphere. After THF (1 mL) was injected into the Schlenk tube, the solution was stirred at rt under the argon atmosphere for 2 h. Then the reaction was

cooled to 0 °C, and *N*-propargyl ynamide **A** (0.15 mmol) in THF (2 mL) was introduced into the system dropwise. The resulting mixture was stirred at indicating temperature. The reaction was monitored by TLC and once it confirmscomplete consumption of **A**, the reaction solvent was removed and added DCE (3 mL), TFA (1.5 mL), and the reaction was heated to rt. Upon completion, appropriate saturated sodium carbonate solution was added to quench the reaction. The mixed solution was then extracted by DCM/H_2O and washed with brine 3 times. The organic phase was dried over $MgSO_4$ and concentrated. The resulting mixture was purified by flash chromatography on silica gel (eluent: hexanes/EA or hexanes/DCM) to give the final product **D**.

(*R*)-*N*,*N*-dimethyl-4-(6-(methylsulfonyl)-13-tosyl-13,13a-dihydro-6*H*-benzo[6,7]pyrrolo[3',4':4,5]cyclohepta[1,2-*b*]indol-8-yl)aniline (D1)

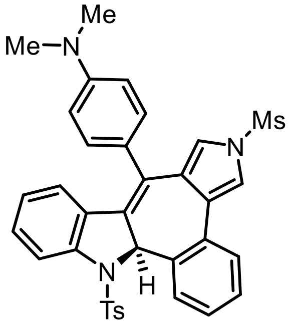

D1

Compound **D1** was prepared according to the general procedure (31 h) in 99% yield (92.9 mg) as a white solid (mp 145–147 °C). $[\alpha]_D^{20}$ = -201.4 (c = 1.0, $CHCl_3$). 98% ee. (determined by HPLC: Chiralpak IE Column, 30/70 *i*-PrOH/hexane, 1.0 mL/min, 254 nm; TR = 16.84 min (major), 20.21 min (minor)). ^{1}H NMR (400 MHz, $CDCl_3$) δ 7.81 (d, *J* = 8.0 Hz, 1H), 7.77 (d, *J* = 7.6 Hz, 1H), 7.56 (d, *J* = 8.4 Hz, 2H), 7.51 – 7.42 (m, 2H), 7.39 – 7.33 (m, 1H), 7.31 – 7.26 (m, 1H), 7.17 (d, *J* = 8.0 Hz, 2H), 7.15 – 7.10 (m, 1H), 6.94 – 6.85 (m, 1H), 6.78 (d, *J* = 2.4 Hz, 1H), 6.75 – 6.70 (m, 1H), 6.67 (d, *J* = 8.4 Hz, 2H), 6.58 – 6.49 (m, 1H), 6.37 (d, *J* = 8.0 Hz, 1H), 5.66 (s, 1H), 3.21 (s, 3H), 2.97 (s, 6H), 2.38 (s, 3H); ^{13}C NMR (100 MHz, $CDCl_3$) δ 150.2, 144.5, 144.0, 137.8, 134.4, 131.6, 130.8, 130.3, 130.2, 130.0, 129.5, 128.5, 128.2, 128.1, 128.0, 127.4, 127.3, 126.9,

126.2, 124.8, 124.4, 124.1, 119.3, 117.8, 116.5, 112.7, 111.7, 67.6, 43.0, 40.3, 21.5; IR (neat): 2926(bs), 1607, 1522, 1458, 1363, 1263, 1171, 1086, 1063, 981, 812, 757 cm^{-1}; HRESIMS Calcd for $[C_{35}H_{31}N_3NaO_4S_2]^+$ (M + Na$^+$) 644.1648, found 644.1640.

(*R*)-4-(6,13-ditosyl-13,13a-dihydro-6*H*-benzo[6,7]pyrrolo[3',4':4,5]cyclohepta[1,2-*b*]indol-8-yl)-*N*,*N*-dimethylaniline (D2)

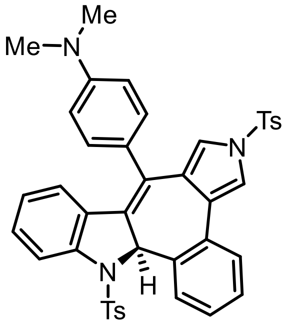

D2

Compound **D2** was prepared according to the general procedure (98 h) in 97% yield (102.2 mg) as a white solid (mp 238–240 °C). $[\alpha]_D^{20}$ = -188.3 (c = 1.0, $CHCl_3$). 93% ee. (determined by HPLC: Chiralpak IE Column, 50/50 *i*-PrOH/hexane, 1.0 mL/min, 254 nm; TR = 13.67 min (major), 16.79 min (minor)). ^{1}H NMR (400 MHz, $CDCl_3$) δ 7.78 (d, *J* = 8.0 Hz, 3H), 7.71 (d, *J* = 7.2 Hz, 1H), 7.55 – 7.39 (m, 4H), 7.34 (d, *J* = 7.6 Hz, 2H), 7.30 (d, *J* = 7.6 Hz, 1H), 7.25 (d, *J* = 7.6 Hz, 1H), 7.11 (d, *J* = 7.2 Hz, 3H), 6.85 (d, *J* = 8.4 Hz, 1H), 6.82 (s, 1H), 6.75 – 6.68 (m, 1H), 6.65 (d, *J* = 8.0 Hz, 1H), 6.59 (d, *J* = 8.0 Hz, 1H), 6.52 (d, *J* = 7.6 Hz, 1H), 6.37 (d, *J* = 7.6 Hz, 1H), 5.53 (s, 1H), 2.97 (s, 6H), 2.42 (s, 3H), 2.37 (s, 3H); ^{13}C NMR (100 MHz, $CDCl_3$) δ 150.2, 145.4, 144.5, 143.9, 137.6, 135.5, 134.1, 131.1, 130.9, 130.4, 130.19, 130.17, 130.1, 129.5, 129.3, 128.34, 128.29, 128.0, 127.9, 127.3, 127.2, 127.03, 127.00, 126.1, 124.8, 124.2, 123.8, 119.6, 118.1, 116.8, 112.5, 111.5, 67.6, 40.2, 21.6, 21.5; IR (neat): 2924(bs), 1607, 1522, 1458, 1359, 1262, 1171, 1088, 1061, 812 cm^{-1}; HRESIMS Calcd for $[C_{41}H_{35}N_3NaO_4S_2]^+$ (M + Na$^+$) 720.1961, found 720.1952.

(*R*)-4-(6-((4-bromophenyl)sulfonyl)-13-tosyl-13,13a-dihydro-6*H*-benzo[6,7]pyrrolo[3',4':4,5]cyclohepta[1,2-*b*]indol-8-yl)-*N*,*N*,3-trimethylaniline (D3)

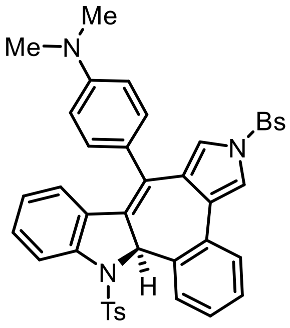

D3

Compound **D3** was prepared according to the general procedure (125 h) in 99% yield (114.0 mg) as a white solid (mp 265–267 °C). $[\alpha]_D^{20}$ = -111.3 (c = 1.0, $CHCl_3$). 93% ee. (determined by HPLC: Chiralpak IE Column, 50/50 *i*-PrOH/hexane, 1.0 mL/min, 254 nm; TR = 12.02 min (major), 14.28 min (minor)). ^{1}H NMR (500 MHz, $CDCl_3$) δ 7.80 (d, *J* = 8.0 Hz, 1H), 7.75 – 7.72 (m, 3H), 7.69 – 7.67 (m, 2H), 7.50 – 7.46 (m, 3H), 7.43 (dd, *J* = 7.5, 1.5 Hz, 1H), 7.34 – 7.31 (m, 1H), 7.26 – 7.23 (m, 1H), 7.14 – 7.10 (m, 3H), 6.84 (dd, *J* = 8.5, 2.0 Hz, 1H), 6.79 (d, *J* = 2.5 Hz, 1H), 6.73 – 6.70 (m, 1H), 6.66 – 6.62 (m, 2H), 6.53 (dd, *J* = 8.5, 2.5 Hz, 1H), 6.37 (d, *J* = 7.5 Hz, 1H), 5.53 (s, 1H), 2.97 (s, 6H), 2.37 (s, 3H); ^{13}C NMR (125 MHz, $CDCl_3$) δ 150.2, 144.5, 144.0, 137.6, 137.3, 134.2, 132.8, 131.5, 130.9, 130.8, 130.4, 129.9, 129.5, 129.33, 129.27, 128.8, 128.4, 128.2, 128.0, 127.4, 127.2, 126.7, 126.0, 124.8, 124.3, 123.9, 119.4, 117.9, 116.8, 112.5, 111.5, 67.5, 40.2, 21.5; IR (neat): 2924(bs), 1608, 1522, 1456, 1359, 1262, 1167, 1089, 1010, 799, 745 cm^{-1}; HRESIMS Calcd for $[C_{40}H_{32}BrN_3NaO_4S_2]^+$ (M + Na$^+$) 784.0910 (isotopic ion: 786.0896), found 784.0910 (isotopic ion: 786.0880).

(*R*)-*N*,*N*-dimethyl-4-(6-(methylsulfonyl)-13-(phenylsulfonyl)-13,13a-dihydro-6*H*-benzo[6,7]pyrrolo[3',4':4,5]cyclohepta[1,2-*b*]indol-8-yl)aniline (D4)

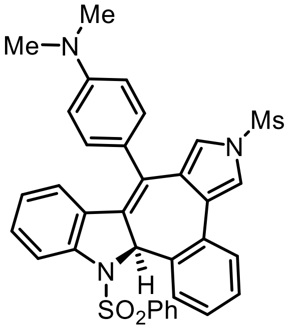

D4

Compound **D4** was prepared according to the general procedure (28 h) in 99% yield (90.5 mg) as a white solid (mp 148–149 °C). $[\alpha]_D^{20}$ = -109.0 (c = 1.0, $CHCl_3$). 98% ee. (determined by HPLC: Chiralpak IC Column, 50/50 *i*-PrOH/hexane, 1.0 mL/min, 254 nm; TR = 9.94 min (major), 13.17 min (minor)). ^{1}H NMR (400 MHz, $CDCl_3$) δ 7.83 (d, *J* = 8.0 Hz, 1H), 7.77 (d, *J* = 7.6 Hz, 1H), 7.68 (d, *J* = 7.6 Hz, 2H), 7.57 – 7.52 (m, 1H), 7.47 (d, *J* = 7.6 Hz, 2H), 7.41 – 7.33 (m, 3H), 7.30 – 7.25 (m, 1H), 7.16 – 7.10 (m, 1H), 6.89 (d, *J* = 8.0 Hz, 1H), 6.78 – 6.70 (m, 2H), 6.66 (d, *J* = 7.6 Hz, 2H), 6.54 (d, *J* = 8.4 Hz, 1H), 6.37 (d, *J* = 7.6 Hz, 1H), 5.66 (s, 1H), 3.20 (s, 3H), 2.95 (s, 6H); ^{13}C NMR (100 MHz, $CDCl_3$) δ 150.2, 144.4, 137.6, 137.3, 133.1, 131.4, 130.7, 130.3, 130.1, 130.0, 129.4, 128.8, 128.5, 128.2, 128.02, 127.99, 127.4, 127.2, 127.1, 126.1, 124.9, 124.4, 124.0, 119.3, 117.8, 116.5, 112.6, 111.7, 67.6, 42.9, 40.2; IR (neat): 2962(bs), 1609, 1522, 1446, 1360, 1261, 1062, 948, 795, 719 cm^{-1}; HRESIMS Calcd for $[C_{34}H_{29}N_3NaO_4S_2]^+$ (M + Na$^+$) 630.1492, found 630.1491.

(*R*)-4-(6,13-bis(methylsulfonyl)-13,13a-dihydro-6*H*-benzo[6,7]pyrrolo[3',4':4,5]cyclohepta[1,2-*b*]indol-8-yl)-*N*,*N*-dimethylaniline (D5)

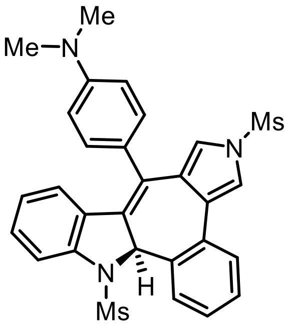

D5

Compound **D5** was prepared according to the general procedure (48 h) in 77% yield (62.9 mg) as a white solid (mp 155–157 °C). $[\alpha]_D^{20}$ = -21.7 (c = 1.0, $CHCl_3$). 99% ee. (determined by HPLC: Chiralpak IE Column, 50/50 *i*-PrOH/hexane, 1.0 mL/min, 254 nm; TR = 8.64 min (major), 10.76 min (minor)). ^{1}H NMR (400 MHz, $CDCl_3$) δ 7.70 (d, *J* = 7.6 Hz, 1H), 7.68 (d, *J* = 8.0 Hz, 1H), 7.50 – 7.45 (m, 2H), 7.39 – 7.34 (m, 1H), 7.33 – 7.29 (m, 1H), 7.29 – 7.26 (m, 1H), 7.19 – 7.13 (m, 1H), 7.01 – 6.95 (m, 1H), 6.86 (d, *J* = 2.4 Hz, 1H), 6.84 – 6.79 (m, 1H), 6.77 – 6.71 (m, 1H), 6.71 – 6.66 (m, 1H), 6.57 (d, *J* = 7.6 Hz, 1H), 5.72 (s, 1H), 3.21 (s, 3H), 3.01 (s, 6H), 2.94 (s, 3H); ^{13}C NMR (100 MHz, $CDCl_3$) δ 150.4, 144.2, 137.7, 131.6, 131.0, 130.4, 130.2, 129.4, 129.2, 128.9, 128.3, 128.12, 128.06, 127.6, 127.5, 126.2, 124.8, 124.7, 123.9, 119.5, 116.6, 116.5, 112.9, 111.9, 67.4, 43.1, 40.3, 36.1; IR (neat): 2926(bs), 1608, 1522, 1459, 1352, 1262, 1163, 1085, 1063, 981, 788 cm^{-1}; HRESIMS Calcd for $[C_{29}H_{27}N_3NaO_4S_2]^+$ (M + Na^+) 568.1335, found 568.1335.

(*R*)-4-(3-chloro-6-(methylsulfonyl)-13-tosyl-13,13a-dihydro-6*H*-benzo[6,7]pyrrolo [3',4':4,5]cyclohepta[1,2-*b*]indol-8-yl)-*N*,*N*-dimethylaniline (D6)

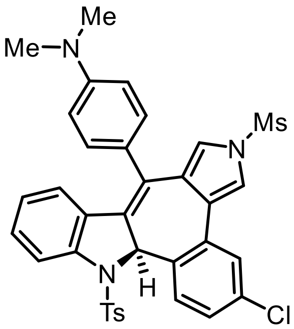

D6

Compound **D6** was prepared according to the general procedure (48 h) in 99% yield (98.2 mg) as a white solid (mp 157–160 °C). $[\alpha]_D^{20}$ = -208.9 (c = 1.0, $CHCl_3$). 99% ee. (determined by HPLC: Chiralpak IE Column, 50/50 *i*-PrOH/hexane, 1.0 mL/min, 254 nm; TR = 8.87 min (major), 10.55 min (minor)). ^{1}H NMR (400 MHz, $CDCl_3$) δ 7.80 (d, *J* = 8.0 Hz, 1H), 7.71 (d, *J* = 8.4 Hz, 1H), 7.56 (d, *J* = 8.4 Hz, 2H), 7.48 (d, *J* = 2.4 Hz,

2H), 7.35 – 7.29 (m, 1H), 7.20 (d, *J* = 8.0 Hz, 2H), 7.17 – 7.11 (m, 1H), 6.95 – 6.87 (m, 1H), 6.79 (d, *J* = 2.4 Hz, 1H), 6.77 – 6.72 (m, 1H), 6.71 – 6.61 (m, 2H), 6.58 – 6.51 (m, 1H), 6.38 (d, *J* = 7.6 Hz, 1H), 5.60 (s, 1H), 3.25 (s, 3H), 2.97 (s, 6H), 2.39 (s, 3H); ^{13}C NMR (100 MHz, $CDCl_3$) δ 150.3, 144.3, 144.2, 136.3, 134.2, 133.0, 131.4, 131.0, 130.7, 130.3, 130.0, 129.7, 129.5, 128.6, 128.0, 127.8, 127.2, 127.0, 126.8, 125.9, 125.6, 124.9, 124.4, 119.5, 117.7, 116.9, 112.7, 111.7, 67.1, 43.1, 40.3, 21.5; IR (neat): 2926(bs), 1608, 1522, 1459, 1364, 1265, 1171, 1083, 1062, 983, 813, 769 cm^{-1}; HRESIMS Calcd for $[C_{35}H_{30}ClN_3NaO_4S_2]^+$ (M + Na^+) 678.1258 (isotopic ion: 680.1243), found 678.1258 (isotopic ion: 680.1242).

(*R*)-4-(3-methoxy-6-(methylsulfonyl)-13-tosyl-13,13a-dihydro-6*H*-benzo[6,7]pyrrolo[3',4':4,5]cyclohepta[1,2-*b*]indol-8-yl)-*N*,*N*-dimethylaniline (D7)

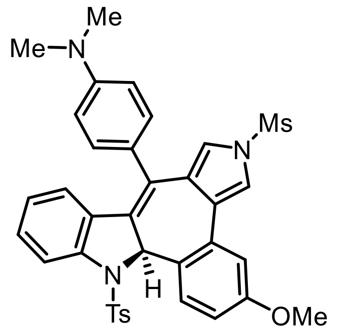

D7

Compound **D7** was prepared according to the general procedure (26 h) in 99% yield (96.9 mg) as a white solid (mp 168–169 °C). $[\alpha]_D^{20}$ = -268.4 (c = 1.0, $CHCl_3$). 98% ee. (determined by HPLC: Chiralpak IE Column, 50/50 *i*-PrOH/hexane, 1.0 mL/min, 254 nm; TR = 13.42 min (major), 16.58 min (minor)). ^{1}H NMR (400 MHz, $CDCl_3$) δ 7.80 (d, *J* = 8.0 Hz, 1H), 7.69 (d, *J* = 8.8 Hz, 1H), 7.56 (d, *J* = 8.0 Hz, 2H), 7.46 (d, *J* = 2.4 Hz, 1H), 7.18 (d, *J* = 8.0 Hz, 2H), 7.14 – 7.09 (m, 1H), 7.02 (d, *J* = 2.4 Hz, 1H), 6.94 – 6.88 (m, 2H), 6.77 (d, *J* = 2.4 Hz, 1H), 6.75 – 6.70 (m, 1H), 6.70 – 6.61 (m, 2H), 6.58 – 6.49 (m, 1H), 6.38 (d, *J* = 7.6 Hz, 1H), 5.62 (s, 1H), 3.79 (s, 3H), 3.21 (s, 3H), 2.96 (s, 6H), 2.37 (s, 3H); ^{13}C NMR (100 MHz, $CDCl_3$) δ 158.8, 150.2, 144.5, 144.0, 134.3, 132.0, 130.8, 130.4, 130.3, 130.2, 129.9, 129.4, 128.4, 128.0, 127.2, 126.6, 126.2, 125.2,

124.7, 124.3, 119.3, 117.7, 116.6, 113.44, 113.39, 112.6, 111.7, 67.2, 55.4, 42.9, 40.2, 21.5; IR (neat): 2927(bs), 1607, 1521, 1458, 1362, 1262, 1170, 1082, 1063, 983, 802 cm^{-1}; HRESIMS Calcd for $[C_{36}H_{33}N_3NaO_5S_2]^+$ (M + Na$^+$) 674.1754, found 674.1753.

(*R*)-*N,N*-dimethyl-4-(2-methyl-6-(methylsulfonyl)-13-tosyl-13,13a-dihydro-6*H*-benzo[6,7]pyrrolo[3',4':4,5]cyclohepta[1,2-*b*]indol-8-yl)aniline (D8)

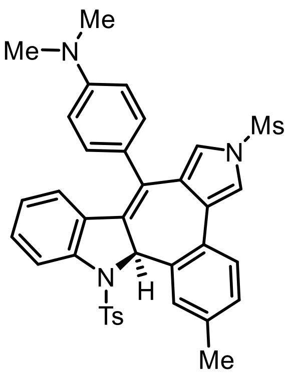

D8

Compound **D8** was prepared according to the general procedure (26 h) in 94% yield (89.4 mg) as a white solid (mp 172–174 °C). $[\alpha]_D^{20}$ = -201.9 (c = 1.0, $CHCl_3$). 98% ee. (determined by HPLC: Chiralpak IC Column, 30/70 *i*-PrOH/hexane, 1.0 mL/min, 254 nm; TR = 18.93 min (major), 25.20 min (minor)). ^{1}H NMR (400 MHz, $CDCl_3$) δ 7.83 (d, *J* = 8.0 Hz, 1H), 7.55 (d, *J* = 8.4 Hz, 3H), 7.42 (d, *J* = 2.4 Hz, 1H), 7.36 (d, *J* = 7.6 Hz, 1H), 7.18 (d, *J* = 8.0 Hz, 2H), 7.15 – 7.11 (m, 1H), 7.09 (d, *J* = 7.6 Hz, 1H), 6.93 – 6.86 (m, 1H), 6.76 (d, *J* = 2.4 Hz, 1H), 6.75 – 6.71 (m, 1H), 6.68 – 6.57 (m, 2H), 6.57 – 6.47 (m, 1H), 6.37 (d, *J* = 7.6 Hz, 1H), 5.64 (s, 1H), 3.20 (s, 3H), 2.96 (s, 6H), 2.38 (s, 3H), 2.35 (s, 3H); ^{13}C NMR (100 MHz, $CDCl_3$) δ 150.1, 144.5, 144.0, 138.1, 137.4, 134.3, 131.5, 130.8, 130.3, 130.1, 129.4, 128.3, 128.2, 128.1, 128.0, 127.2, 127.0, 126.6, 126.2, 124.8, 124.5, 124.3, 119.2, 118.0, 116.1, 112.6, 111.7, 67.5, 42.9, 40.2, 21.5, 21.4; IR (neat): 2925(bs), 1677, 1608, 1521, 1457, 1364, 1263, 1170, 1084, 1063, 982, 812 cm^{-1}; HRESIMS Calcd for $[C_{36}H_{33}N_3NaO_4S_2]^+$ (M + Na$^+$) 658.1805, found 658.1801.

(*R*)-4-(10-chloro-6-(methylsulfonyl)-13-tosyl-13,13a-dihydro-6*H*-benzo[6,7]pyrrolo[3',4':4,5]cyclohepta[1,2-*b*]indol-8-yl)-*N*,*N*-dimethylaniline (D9)

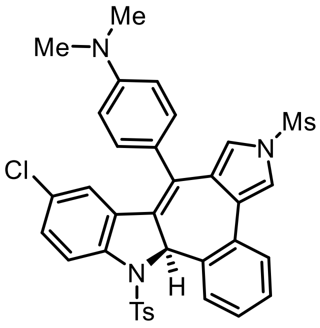

D9

Compound **D9** was prepared according to the general procedure (48 h) in 99% yield (98.0 mg) as a white solid (mp 180–182 °C). $[\alpha]_D^{20}$ = -233.7 (c = 1.0, $CHCl_3$). 99% ee. (determined by HPLC: Chiralpak IE Column, 30/70 *i*-PrOH/hexane, 1.0 mL/min, 254 nm; TR = 12.43 min (major), 13.94 min (minor)). ^{1}H NMR (400 MHz, $CDCl_3$) δ 7.74 (d, *J* = 8.4 Hz, 2H), 7.56 (d, *J* = 8.4 Hz, 2H), 7.50 – 7.43 (m, 2H), 7.40 – 7.34 (m, 1H), 7.32 – 7.27 (m, 1H), 7.22 (d, *J* = 8.0 Hz, 2H), 7.12 – 7.04 (m, 1H), 6.93 – 6.84 (m, 1H), 6.80 (d, *J* = 2.4 Hz, 1H), 6.71 – 6.64 (m, 1H), 6.64 – 6.59 (m, 1H), 6.57 – 6.50 (m, 1H), 6.31 (d, *J* = 2.0 Hz, 1H), 5.65 (s, 1H), 3.23 (s, 3H), 2.97 (s, 6H), 2.40 (s, 3H); ^{13}C NMR (100 MHz, $CDCl_3$) δ 150.6, 144.3, 143.0, 137.5, 134.1, 131.8, 130.7, 130.2, 130.1, 130.0, 129.8, 129.6, 129.3, 128.6, 128.3, 128.2, 128.1, 128.0, 127.6, 127.3, 125.3, 124.1, 123.9, 119.7, 118.8, 116.6, 112.7, 111.7, 68.0, 43.0, 40.3, 21.5; IR (neat): 2926(bs), 1608, 1522, 1453, 1363, 1263, 1170, 1089, 982, 815, 771 cm^{-1}; HRESIMS Calcd for $[C_{35}H_{30}ClN_3NaO_4S_2]^+$ (M + Na^+) 678.1258 (isotopic ion: 680.1243), found 678.1251 (isotopic ion: 680.1244).

(*R*)-4-(10-bromo-6-(methylsulfonyl)-13-tosyl-13,13a-dihydro-6*H*-benzo[6,7]pyrrolo[3',4':4,5]cyclohepta[1,2-*b*]indol-8-yl)-*N*,*N*-dimethylaniline (D10)

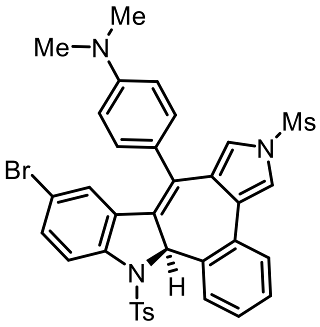

D10

Compound **D10** was prepared according to the general procedure (32 h) in 99% yield (104.4 mg) as a white solid (mp 207–208 °C). $[\alpha]_D^{20}$ = -188.2 (c = 1.0, $CHCl_3$). 98% ee. (determined by HPLC: Chiralpak IE Column, 20/80 *i*-PrOH/hexane, 1.0 mL/min, 254 nm; TR = 20.96 min (major), 24.21 min (minor)). ^{1}H NMR (400 MHz, $CDCl_3$) δ 7.73 (d, *J* = 7.6 Hz, 1H), 7.68 (d, *J* = 8.8 Hz, 1H), 7.56 (d, *J* = 8.0 Hz, 2H), 7.50 – 7.44 (m, 2H), 7.40 – 7.35 (m, 1H), 7.32 – 7.27 (m, 1H), 7.24 – 7.20 (m, 3H), 6.92 – 6.86 (m, 1H), 6.81 (d, *J* = 2.4 Hz, 1H), 6.70 – 6.62 (m, 2H), 6.59 – 6.53 (m, 1H), 6.45 (d, *J* = 2.0 Hz, 1H), 5.65 (s, 1H), 3.23 (s, 3H), 2.97 (s, 6H), 2.40 (s, 3H); ^{13}C NMR (100 MHz, $CDCl_3$) δ 150.6, 144.3, 143.5, 137.5, 134.2, 132.2, 131.1, 130.7, 130.1, 129.9, 129.8, 129.6, 129.3, 128.6, 128.3, 128.09, 128.05, 127.6, 127.2, 127.1, 125.3, 123.9, 119.7, 119.1, 118.0, 116.6, 112.7, 111.8, 67.9, 43.0, 40.3, 21.5; IR (neat): 2924(bs), 1607, 1526, 1488, 1370, 1171, 1088, 1048, 984, 814 cm^{-1}; HRESIMS Calcd for $[C_{35}H_{30}BrN_3NaO_4S_2]^+$ (M + Na^+) 722.0753 (isotopic ion: 724.0737), found 722.0752 (isotopic ion: 724.0735).

(*R*)-*N*,*N*-dimethyl-4-(10-methyl-6-(methylsulfonyl)-13-tosyl-13,13a-dihydro-6*H*-benzo[6,7]pyrrolo[3',4':4,5]cyclohepta[1,2-*b*]indol-8-yl)aniline (D11)

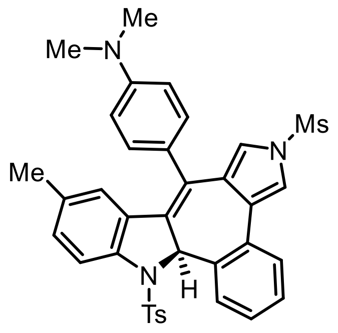

D11

Compound **D11** was prepared according to the general procedure (27 h) in 99% yield (95.3 mg) as a white solid (mp 160–162 °C). $[\alpha]_D^{20}$ = -204.6 (c = 1.0, $CHCl_3$). 99% ee. (determined by HPLC: Chiralpak IE Column, 30/70 *i*-PrOH/hexane, 1.0 mL/min, 254 nm; TR = 17.10 min (major), 19.27 min (minor)). ^{1}H NMR (400 MHz, $CDCl_3$) δ 7.76 (d, *J* = 7.6 Hz, 1H), 7.70 (d, *J* = 8.4 Hz, 1H), 7.55 (d, *J* = 8.0 Hz, 2H), 7.46 (d, *J* = 7.2 Hz, 2H), 7.36 – 7.31 (m, 1H), 7.29 – 7.24 (m, 1H), 7.18 (d, *J* = 8.0 Hz, 2H), 6.94 (d, *J* = 8.0 Hz, 1H), 6.90 (d, *J* = 7.6 Hz, 1H), 6.77 (d, *J* = 2.4 Hz, 1H), 6.70 – 6.60 (m, 2H), 6.58 – 6.50 (m, 1H), 6.17 (s, 1H), 5.63 (s, 1H), 3.20 (s, 3H), 2.96 (s, 6H), 2.37 (s, 3H), 1.99 (s, 3H); ^{13}C NMR (100 MHz, $CDCl_3$) δ 150.3, 143.9, 142.4, 137.8, 134.30, 134.28, 131.7, 130.7, 130.4, 130.2, 130.1, 129.42, 129.39, 129.3, 128.14, 128.11, 127.9, 127.32, 127.26, 126.6, 126.3, 124.7, 123.9, 119.2, 117.6, 116.5, 112.6, 111.6, 67.8, 42.9, 40.3, 21.5, 21.1; IR (neat): 2925(bs), 1607, 1521, 1364, 1263, 1170, 1084, 1061, 982, 801, 773 cm^{-1}; HRESIMS Calcd for $[C_{36}H_{33}N_3NaO_4S_2]^+$ (M + Na^+) 658.1805, found 658.1801.

(*R*)-4-(11-chloro-6-(methylsulfonyl)-13-tosyl-13,13a-dihydro-6*H*-benzo[6,7]pyrrolo[3',4':4,5]cyclohepta[1,2-*b*]indol-8-yl)-*N*,*N*-dimethylaniline (D12)

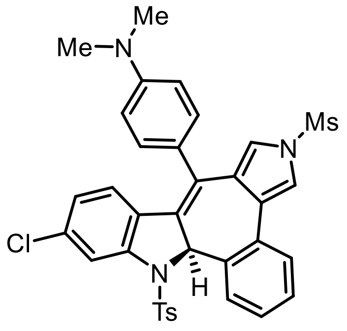

D12

Compound **D12** was prepared according to the general procedure (32 h) in 99% yield (97.5 mg) as a white solid (mp 160–162 °C). $[\alpha]_D^{20}$ = -89.4 (c = 1.0, $CHCl_3$). 98% ee. (determined by HPLC: Chiralpak IE Column, 50/50 *i*-PrOH/hexane, 1.0 mL/min, 254 nm; TR = 8.35 min (major), 9.60 min (minor)). ^{1}H NMR (400 MHz, $CDCl_3$) δ 7.82 (d, *J* = 2.0 Hz, 1H), 7.73 (d, *J* = 7.6 Hz, 1H), 7.60 (d, *J* = 8.4 Hz, 2H), 7.51 – 7.44 (m, 2H), 7.40 – 7.35 (m, 1H), 7.32 – 7.27 (m, 1H), 7.23 (d, *J* = 7.2 Hz, 2H), 6.92 – 6.87 (m, 1H), 6.79 (d, *J* = 2.4 Hz, 1H), 6.72 – 6.68 (m, 1H), 6.68 – 6.60 (m, 2H), 6.57 – 6.50 (m, 1H), 6.28 (d, *J* = 8.4 Hz, 1H), 5.67 (s, 1H), 3.22 (s, 3H), 2.96 (s, 6H), 2.40 (s, 3H); ^{13}C NMR (100 MHz, $CDCl_3$) δ 150.3, 145.4, 144.4, 137.5, 134.3, 133.8, 130.8, 130.22, 130.17, 129.9, 129.6, 129.3, 128.5, 128.2, 128.1, 128.0, 127.6, 127.5, 127.2, 125.7, 124.9, 123.9, 119.4, 117.7, 116.6, 112.6, 111.7, 68.0, 43.0, 40.2, 21.5; IR (neat): 2926(bs), 1608, 1522, 1468, 1365, 1263, 1171, 1072, 981, 813, 773 cm^{-1}; HRESIMS Calcd for $[C_{35}H_{30}ClN_3NaO_4S_2]^+$ (M + Na^+) 678.1258 (isotopic ion: 680.1243), found 678.1248 (isotopic ion: 680.1240).

(*R*)-4-(11-bromo-6-(methylsulfonyl)-13-tosyl-13,13a-dihydro-6*H*-benzo[6,7]pyrrolo[3',4':4,5]cyclohepta[1,2-*b*]indol-8-yl)-*N*,*N*-dimethylaniline (D13)

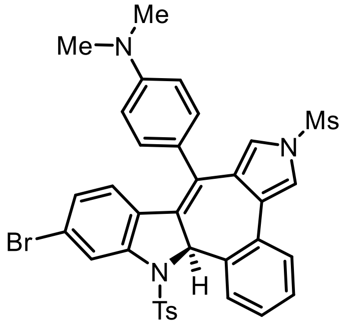

D13

Compound **D13** was prepared according to the general procedure (32 h) in 99% yield (104.7 mg) as a white solid (mp 194–1195 °C). $[\alpha]_D^{20}$ = -105.8 (c = 1.0, $CHCl_3$). 98% ee. (determined by HPLC: Chiralpak IE Column, 50/50 *i*-PrOH/hexane, 1.0 mL/min, 220 nm; TR = 8.76 min (major), 9.85 min (minor)). ^{1}H NMR (400 MHz, $CDCl_3$) δ 7.73 (d, *J* = 7.6 Hz, 1H), 7.68 (d, *J* = 8.4 Hz, 1H), 7.56 (d, *J* = 8.0 Hz, 2H), 7.48 (d, *J* = 6.8 Hz, 2H), 7.40 – 7.35 (m, 1H), 7.32 – 7.28 (m, 1H), 7.22 (d, *J* = 8.0 Hz, 3H), 6.89 (d, *J* = 8.0 Hz, 1H), 6.81 (s, 1H), 6.69 – 6.61 (m, 2H), 6.56 (d, *J* = 8.0 Hz, 1H), 6.45 (s, 1H), 5.65 (s, 1H), 3.23 (s, 3H), 2.97 (s, 6H), 2.40 (s, 3H); ^{13}C NMR (100 MHz, $CDCl_3$) δ 150.3, 145.6, 144.4, 137.5, 134.3, 130.8, 130.3, 130.2, 129.9, 129.7, 129.3, 129.0, 128.3, 128.1, 128.0, 127.8, 127.7, 127.6, 127.2, 125.7, 125.3, 124.0, 121.7, 120.6, 119.5, 116.6, 112.6, 111.7, 67.9, 43.0, 40.2, 21.5; IR (neat): 2925(bs), 1607, 1523, 1468, 1364, 1171, 1075, 985, 813, 799 cm^{-1}; HRESIMS Calcd for $[C_{35}H_{30}BrN_3NaO_4S_2]^+$ (M + Na$^+$) 722.0753 (isotopic ion: 724.0737), found 722.0750 (isotopic ion: 724.0736).

(*R*)-*N*,*N*-dimethyl-4-(11-methyl-6-(methylsulfonyl)-13-tosyl-13,13a-dihydro-6*H*-benzo[6,7]pyrrolo[3',4':4,5]cyclohepta[1,2-*b*]indol-8-yl)aniline (D14)

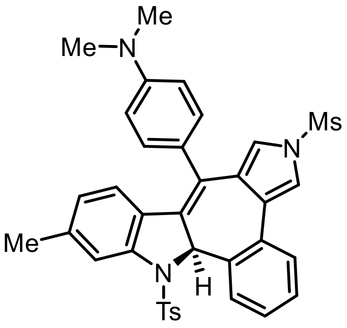

D14

Compound **D14** was prepared according to the general procedure (32 h) in 94% yield (89.4 mg) as a white solid (mp 165–166 °C). $[\alpha]_D^{20}$ = -212.5 (c = 1.0, $CHCl_3$). 99% ee. (determined by HPLC: Chiralpak IE Column, 50/50 *i*-PrOH/hexane, 1.0 mL/min, 254 nm; TR = 8.61 min (major), 10.28 min (minor)). ^{1}H NMR (400 MHz, $CDCl_3$) δ 7.76 (d, *J* = 7.6 Hz, 1H), 7.65 (s, 1H), 7.57 (d, *J* = 8.4 Hz, 2H), 7.50 – 7.43 (m, 2H), 7.38 – 7.33 (m, 1H), 7.30 – 7.25 (m, 1H), 7.19 (d, *J* = 8.0 Hz, 2H), 6.94 – 6.87 (m, 1H), 6.76 (d, *J* = 2.4 Hz, 1H), 6.71 – 6.62 (m, 2H), 6.60 – 6.49 (m, 2H), 6.26 (d, *J* = 8.0 Hz, 1H), 5.64 (s, 1H), 3.20 (s, 3H), 2.96 (s, 6H), 2.38 (s, 3H), 2.32 (s, 3H); ^{13}C NMR (100 MHz, $CDCl_3$) δ 150.1, 144.8, 144.0, 139.0, 137.8, 134.5, 131.7, 130.8, 130.4, 130.3, 129.4, 128.14, 128.08, 128.0, 127.4, 127.3, 127.2, 126.4, 125.74, 125.70, 123.99, 123.96, 119.0, 118.2, 116.5, 112.6, 111.7, 67.9, 42.9, 40.3, 21.7, 21.5; IR (neat): 2925 (bs), 1607, 1522, 1474, 1361, 1263, 1168, 1085, 1064, 981, 802 cm^{-1}; HRESIMS Calcd for $[C_{36}H_{33}N_3NaO_4S_2]^+$ (M + Na^+) 658.1805, found 658.1805.

(*R*)-*N*,*N*,3-trimethyl-4-(6-(methylsulfonyl)-13-tosyl-13,13a-dihydro-6*H*-bsenzo[6,7]pyrrolo[3',4':4,5]cyclohepta[1,2-*b*]indol-8-yl)aniline (D15)

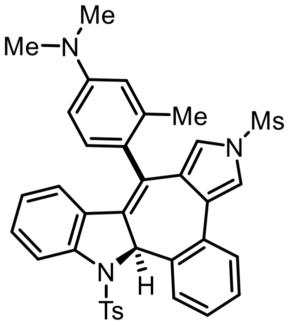

D15

Compound **D15** was prepared according to the general procedure (29 h) in 94% yield (89.6 mg) as a white solid (mp 183–184 °C). $[\alpha]_D^{20}$ = -113.3 (c = 1.0, $CHCl_3$). 96% ee. (determined by HPLC: Chiralpak IC Column, 20/80 *i*-PrOH/hexane, 1.0 mL/min, 254 nm; TR = 30.95 min (major), 66.29 min (minor)); d.r. = 1:1.8 (determined by ^{1}H NMR). ^{1}H NMR (400 MHz, $CDCl_3$) δ 7.91 – 7.80 (m, 2H), 7.57 – 7.45 (m, 4H), 7.42 – 7.35

(m, 1H), 7.32 – 7.26 (m, 1H), 7.20 – 7.09 (m, 3H), 6.79 – 6.69 (m, 2H), 6.66 – 6.44 (m, 3H), 6.14 – 6.01 (m, 1H), 5.69 (s, 0.64H), 5.60 (s, 0.36H), 3.28 – 3.14 (m, 3H), 3.02 – 2.88 (m, 6H), 2.41 – 2.31 (m, 3H), 1.66 – 1.39 (m, 3H); ^{13}C NMR (100 MHz, $CDCl_3$) δ 150.5, 150.1, 144.5, 144.4, 144.1, 144.0, 137.9, 137.7, 137.5, 135.9, 134.4, 133.8, 133.6, 130.7, 130.3, 130.0, 129.8, 129.6, 129.51, 129.47, 129.4, 128.8, 128.6, 128.2, 128.12, 128.05, 127.9, 127.8, 127.7, 127.5, 127.4, 127.24, 127.15, 126.5, 126.1, 125.5, 125.4, 124.9, 124.3, 124.22, 124.17, 123.3, 118.7, 118.4, 117.8, 117.3, 116.6, 114.6, 113.7, 110.8, 110.1, 67.2, 66.9, 43.0, 42.9, 40.4, 40.3, 21.5, 21.4, 19.8, 18.8; IR (neat): 2924(bs), 1608, 1514, 1467, 1361, 1263, 1170, 1085, 1062, 982, 800, 738 cm^{-1}; HRESIMS Calcd for $[C_{36}H_{33}N_3NaO_4S_2]^+$ (M + Na^+) 658.1805, found 658.1802.

(*R*)-*N*,*N*-dimethyl-4-(6-(methylsulfonyl)-13-tosyl-13,13a-dihydro-6*H*-benzo[6,7]pyrrolo[3',4':4,5]cyclohepta[1,2-*b*]indol-8-yl)naphthalen-1-amine (D16)

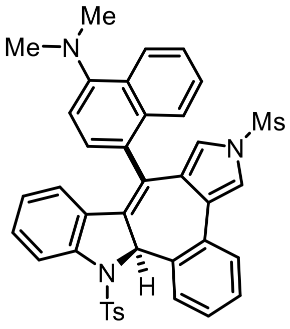

D16

Compound **D16** was prepared according to the general procedure (205 h) in 99% yield (100.3 mg) as a white solid (mp 171–1173 °C). $[\alpha]_D^{20}$ = -109.1 (c = 1.0, $CHCl_3$). 98% ee (determined by HPLC: Chiralpak IC Column, 50/50 *i*-PrOH/hexane, 1.0 mL/min, 254 nm; TR = 11.53 min (major), 17.01 min (minor)); d.r. = 1:2.4 (determined by ^{1}H NMR). ^{1}H NMR (500 MHz, $CDCl_3$) δ 8.24 – 8.20 (m, 1H), 7.93 – 7.86 (m, 1H), 7.86 – 7.80 (m, 1H), 7.60 – 7.51 (m, 3H), 7.50 – 7.47 (m, 1H), 7.44 – 7.40 (m, 1H), 7.39 – 7.31 (m, 2H), 7.26 – 7.20 (m, 2H), 7.09 – 7.00 (m, 3H), 6.98 – 6.93 (m, 1H), 6.91 – 6.82 (m, 1H), 6.68 – 6.53 (m, 1H), 6.53 – 6.41 (m, 1H), 5.84 – 5.79 (m, 1H), 5.72 (d, *J* = 7.5 Hz, 0.71H), 5.59 (d, *J* = 7.5 Hz, 0.29H), 3.15 – 3.08 (m, 3H), 2.94 – 2.90 (m, 6H),

2.47 – 2.40 (m, 3H); ^{13}C NMR (125 MHz, $CDCl_3$) δ 151.4, 151.0, 144.7, 144.5, 144.1, 144.0, 137.8, 137.51, 135.47, 134.9, 134.3, 134.1, 132.6, 131.7, 131.1, 130.2, 130.1, 129.7, 129.5, 129.34, 129.27, 129.2, 129.12, 129.08, 128.92, 128.88, 128.7, 128.3, 128.12, 128.10, 127.8, 127.7, 127.6, 127.5, 127.3, 126.2, 126.1, 125.9, 125.8, 125.2, 125.1, 124.97, 124.96, 124.8, 124.7, 124.51, 124.48, 124.4, 124.31, 124.28, 124.1, 123.9, 118.8, 118.5, 117.4, 117.3, 116.7, 116.4, 113.9, 113.5, 67.4, 67.0, 45.14, 45.09, 42.90, 42.87, 21.7, 21.5; IR (neat): 2924 (bs), 1577, 1507, 1456, 1369, 1263, 1170, 1086, 982, 799, 705 cm^{-1}; HRESIMS Calcd for $[C_{39}H_{33}N_3NaO_4S_2]^+$ (M + Na^+) 694.1805, found 694.1802.

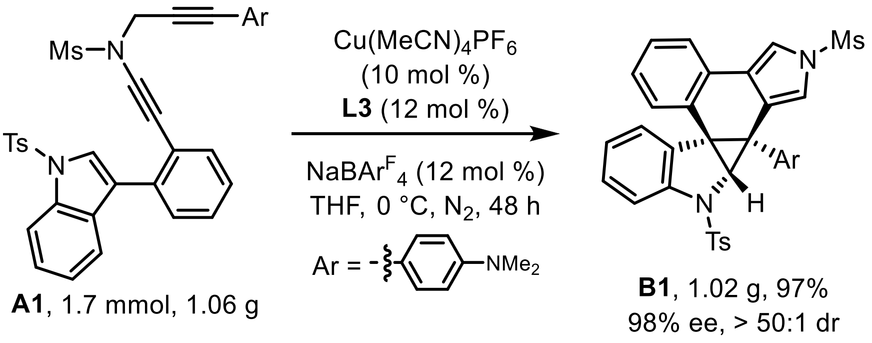

To the mixture of $Cu(MeCN)_4PF_6$ (0.17 mmol, 63.4 mg), **L3** (0.20 mmol, 158.2 mg), and $NaBAr^F{}_4$ (0.20 mmol, 177.2 mg) were introduced into an oven-dried Schlenk tube under argon atmosphere. After THF (5 mL) was injected into the Schlenk tube, the solution was stirred at rt under the argon atmosphere for 2 h. Then the reaction was cooled to 0 °C, and *N*-propargyl ynamide **A1** (1.7 mmol, 1.06 g) in THF (25 mL) was introduced into the system dropwise. The resulting mixture was stirred at indicating temperature and the progress of the reaction was monitored by TLC. After concentration in vacuo, the residue was purified by flash chromatography on silica gel (eluent: hexanes/EA = 3:1) to give the final product **B1** in 97% yield (1.02 g) with 98% ee.

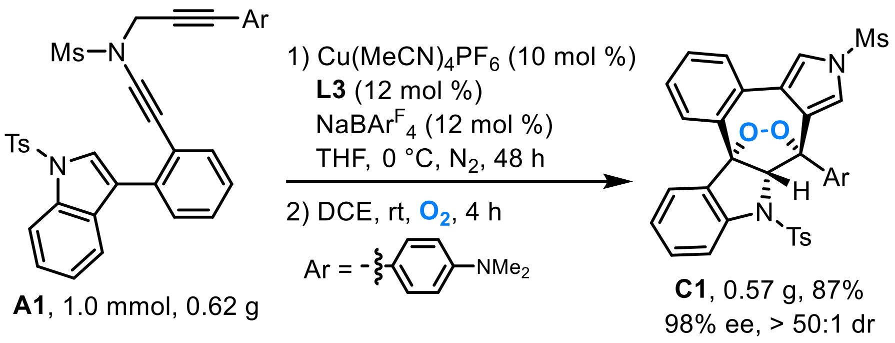

To the mixture of Cu(MeCN)$_4$PF$_6$ (0.1 mmol, 37.3 mg), **L3** (0.12 mmol, 94.9 mg), and NaBAr$^F_4$ (0.12 mmol, 106.3 mg) were introduced into an oven-dried Schlenk tube under nitrogen atmosphere. After THF (5 mL) was injected into the Schlenk tube, the solution was stirred at rt under the argon atmosphere for 2 h. Then the reaction was cooled to 0 °C, and *N*-propargyl ynamide **A1** (1 mmol, 0.62 g) in THF (15 mL) was introduced into the system dropwise. The resulting mixture was stirred at indicating temperature. The reaction was monitored by TLC and once it confirmscomplete consumption of **A1**, DCE (5 mL) was added and the reaction atmosphere was changed from nitrogen to oxygen and the reaction was heated to rt. Once the TLC confirms complete consumption of the product from the previous reaction, and concentrated under reduced pressure. The residue was purified by flash chromatography on silica gel (eluent: hexanes/EA = 3:1) to give the final product **C1** in 87% yield (0.57 g) with 98% ee.

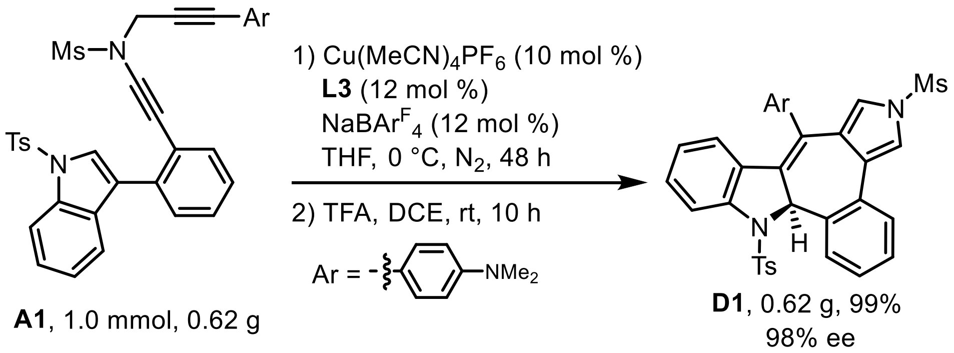

To the mixture of Cu(MeCN)$_4$PF$_6$ (0.1 mmol, 37.3 mg), **L3** (0.12 mmol, 94.9 mg), and NaBAr$^F_4$ (0.12 mmol, 106.3 mg) were introduced into an oven-dried Schlenk tube under nitrogen atmosphere. After THF (5 mL) was injected into the Schlenk tube, the

solution was stirred at rt under the argon atmosphere for 2 h. Then the reaction was cooled to 0 °C, and N-propargyl ynamide **A1** (1 mmol, 0.62 g) in THF (15 mL) was introduced into the system dropwise. The resulting mixture was stirred at indicating temperature. The reaction was monitored by TLC and once it confirmscomplete consumption of **A1**, the reaction solvent was removed and added DCE (20 mL), TFA (5 mL), and the reaction was heated to rt. Upon completion, appropriate saturated sodium carbonate solution was added to quench the reaction. The mixed solution was then extracted by DCE/H_2O and washed by brine 3 times. The organic phase was dried over $MgSO_4$ and concentrated. The resulting mixture was purified by flash chromatography on silica gel (eluent: hexanes/EA = 3:1) to give the final product **D1** in 99% yield (0.62 g) with 98% ee.

***N*,*N*-dimethyl-4-((3b*R*,3c*S*,8b*S*)-4-tosyl-3c,4-dihydrobenzo[6',7']isoindolo[5',4':2,3]cyclopropa[1,2-*b*]indol-3b(2*H*)-yl)aniline (E1)**

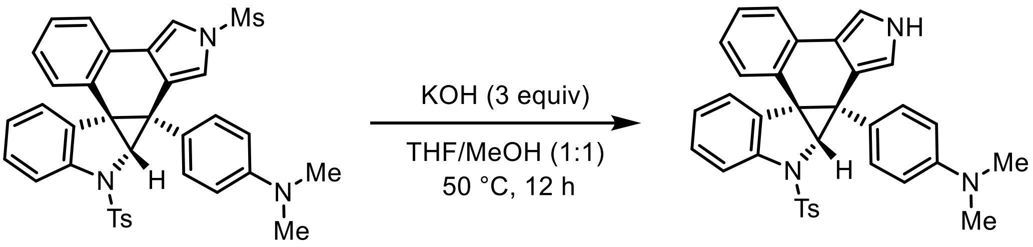

B1, 98% ee
E1, 99%, 98% ee, > 50:1 dr

Compound **E1** was prepared in 99% yield (85.6 mg) according to the known procedure (0.15 mmol scale).[4] White solid (mp 154–156 °C). $[\alpha]_D^{20}$ = +326.5 (c = 1.0, $CHCl_3$). 98% ee (determined by HPLC: Chiralpak IC Column, 50/50 *i*-PrOH/hexane, 1.0 mL/min, 254 nm; TR = 4.29 min (major), 7.00 min (minor)). ^{1}H NMR (400 MHz, $CDCl_3$) δ 8.25 (s, 1H), 7.56 – 7.53 (m, 1H), 7.40 – 7.24 (m, 5H), 7.16 – 7.12 (m, 1H), 7.05 – 7.03 (m, 1H), 7.00 – 6.96 (m, 2H), 6.96 – 6.92 (m, 3H), 6.91 – 6.87 (m, 1H), 6.78 – 6.76 (m, 1H), 6.47 – 6.33 (m, 2H), 6.23 – 6.20 (m, 1H), 3.95 (s, 1H), 2.76 (s, 6H), 2.26 (s, 3H); ^{13}C NMR (100 MHz, $CDCl_3$) δ 148.7, 143.7, 141.6, 134.0, 133.8, 131.2, 130.0, 129.3, 128.7, 127.6, 127.1, 126.8, 126.1, 124.6, 124.5, 124.2, 122.1, 121.3, 117.4, 117.2, 117.1, 111.9, 111.8, 54.6, 40.4, 40.3, 31.7, 21.4; IR (neat): 3403(bs),

2924(bs), 1613, 1523, 1481, 1461, 1356, 1168, 1089, 811, 764, 735 cm^{-1}; HRESIMS Calcd for $[C_{34}H_{29}N_3NaO_2S]^+$ (M + Na$^+$) 566.1873, found 566.1873.

_N_,_N_-dimethyl-4-((3b_R_,3c_S_,8b_S_)-2-(methylsulfonyl)-4-tosyl-2,3,3c,4-tetrahydrobenzo[6',7']isoindolo[5',4':2,3]cyclopropa[1,2-_b_]indol-3b(1_H_)-yl)aniline (E2)

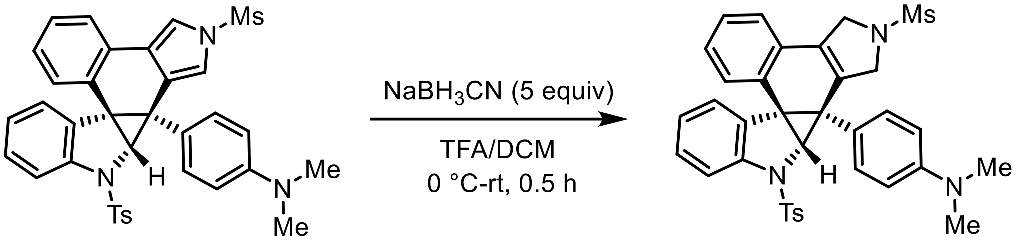

B1, 98% ee　　**E2**, 82%, 98% ee, > 50:1 dr

Compound **E2** was prepared in 82% yield (76.4 mg) according to the known procedure (0.15 mmol scale).[5] White solid (mp 129–131 °C). $[\alpha]_D^{20}$ = +421.4 (c = 1.0, $CHCl_3$). 98% ee (determined by HPLC: Chiralpak IB Column, 30/70 *i*-PrOH/hexane, 1.0 mL/min, 254 nm; TR = 11.69 min (major), 14.38 min (minor)). ^{1}H NMR (400 MHz, $CDCl_3$) δ 7.40 – 7.35 (m, 3H), 7.29 – 7.24 (m, 3H), 7.17 – 7.13 (m, 1H), 7.04 (d, *J* = 6.8 Hz, 2H), 7.03 – 7.00 (m, 3H), 6.97 (d, *J* = 7.2 Hz, 1H), 6.43 (d, *J* = 38.4 Hz, 2H), 6.24 (d, *J* = 14.8 Hz, 1H), 4.62 (t, *J* = 4.0 Hz, 2H), 4.35 (dt, *J* = 14.8, 4.0 Hz, 1H), 3.86 (s, 1H), 3.72 (dt, *J* = 14.8, 4.0 Hz, 1H), 2.84 (s, 3H), 2.81 (s, 6H), 2.29 (s, 3H); ^{13}C NMR (100 MHz, $CDCl_3$) δ 149.3, 144.1, 141.5, 134.6, 134.3, 132.0, 131.4, 130.9, 130.6, 129.4, 127.6, 127.5, 127.1, 127.0, 126.8, 126.7, 126.02, 125.96, 124.2, 123.7, 120.4, 116.9, 112.1, 111.5, 57.0, 54.6, 50.4, 42.1, 40.2, 35.0, 33.7, 21.4; IR (neat): 2924(bs), 1613, 1524, 1461, 1338, 1167, 1090, 810, 759, 660 cm^{-1}; HRESIMS Calcd for $[C_{35}H_{33}N_3NaO_4S_2]^+$ (M + Na$^+$) 646.1805, found 646.1804.

dimethyl (5*S*,8*R*,9*S*)-9-(4-(dimethylamino)phenyl)-15-(methylsulfonyl)-10-tosyl-5,8,9,10-tetrahydro-5,8-epiminodibenzo[3,4:5,6]cyclohepta[1,2-b]indole-6,7-dicarboxylate (E3)

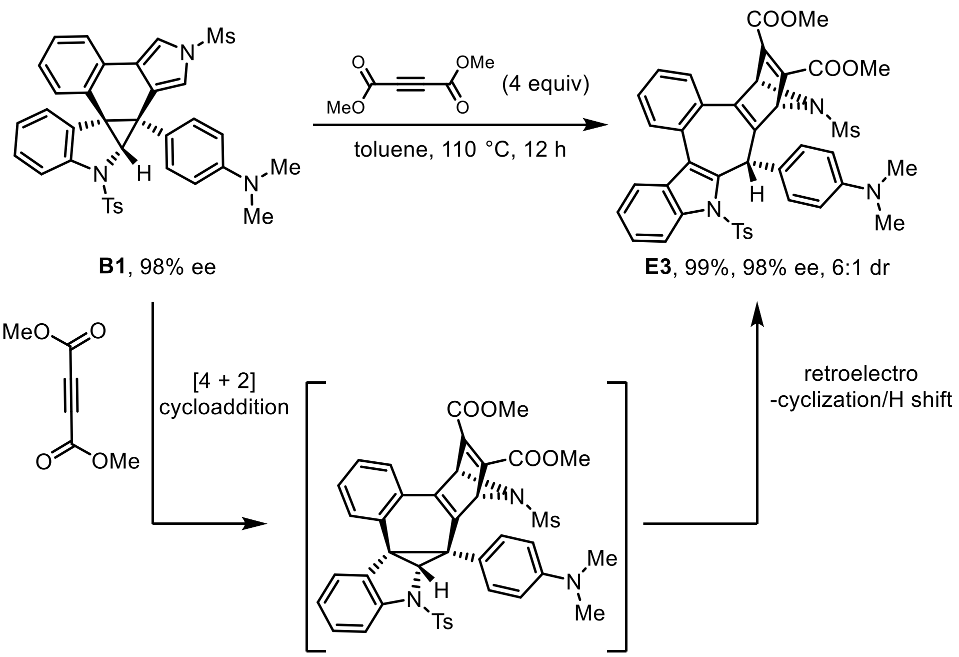

Compound **E3** was prepared in 99% yield (113.9 mg) according to the known procedure (0.15 mmol scale).[6] Yellow solid (mp 162–164 °C). $[\alpha]_D^{20}$ = -471.4 (c = 1.0, $CHCl_3$). 98% ee (determined by HPLC: Chiralpak IA Column, 50/50 *i*-PrOH/hexane, 1.0 mL/min, 254 nm; TR = 6.67 min (major), 9.11 min (minor)); dr = 6:1 (determined by ^{1}H NMR). ^{1}H NMR (400 MHz, $CDCl_3$) δ 8.30 (d, *J* = 8.4 Hz, 1H), 7.89 (d, *J* = 7.2 Hz, 1H), 7.77 (d, *J* = 7.6 Hz, 1H), 7.61 (d, *J* = 8.0 Hz, 1H), 7.53 – 7.47 (m, 1H), 7.46 – 7.32 (m, 3H), 7.26 (d, *J* = 9.2 Hz, 2H), 6.91 (d, *J* = 8.8 Hz, 2H), 6.83 (d, *J* = 8.0 Hz, 2H), 6.60 (d, *J* = 8.8 Hz, 2H), 5.81 (s, 1H), 5.71 (d, *J* = 1.2 Hz, 1H), 4.07 (s, 3H), 3.93 (s, 3H), 3.10 – 2.91 (m, 7H), 2.91 (s, 3H), 2.20 (s, 3H); ^{13}C NMR (100 MHz, $CDCl_3$) δ 163.2, 162.8, 149.7, 147.6, 144.3, 139.2, 138.5, 138.4, 138.2, 134.2, 133.2, 130.8, 130.1, 129.4, 129.2, 129.1, 128.90, 128.85, 127.7, 127.4, 126.7, 126.6, 125.9, 125.0, 124.9, 120.8, 117.7, 111.6, 67.2, 66.2, 53.3, 52.6, 46.7, 40.7, 40.3, 21.5; IR (neat): 2923(bs), 1729, 1611, 1522, 1346, 1219, 1175, 1157, 1015, 813, 773 cm^{-1}; HRESIMS Calcd for $[C_{41}H_{37}N_3NaO_8S_2]^+$ (M + Na$^+$) 786.1914, found 786.1914.

***tert*-butyl (8*S*,8a*S*,13b*S*)-8-(4-(dimethylamino)phenyl)-9-tosyl-8a,9-dihydro-8,13b-epidioxybenzo[3,4]pyrrolo[3',4':5,6]cyclohepta[1,2-*b*]indole-6(8*H*)-carboxylate (E4)**

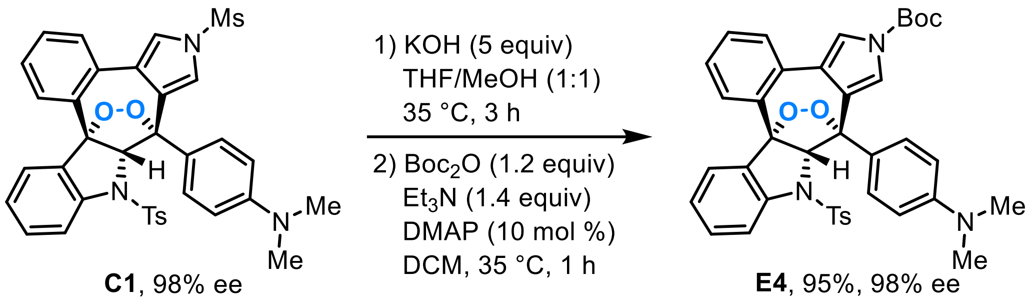

To an oven-dried 10-mL Schlenk tube with a stir bar were added **C1** (0.15 mmol, 98.1 mg) and KOH (42.0 mg, 0.75 mmol) were added. Subsequently, 3 mL (1:1 ratio) of THF and methanol was added sequentially and stirred at 35 °C for 3 h. The reaction was monitored by TLC and once it confirmscomplete consumption of **C1**, the reaction solvent was removed and the crude product was dissolved in DCM (3 mL) and followed by addition of Boc_2O (0.18 mmol, 39.3 mg), DMAP (0.015 mmol, 1.8 mg) and Et_3N (0.21 mmol, 29 μL). The reaction was stirred at 35 °C for about 1 h until the reaction was complete monitored by TLC. The mixed solution was then extracted by DCM/H_2O. The organic phase was dried over $MgSO_4$ and concentrated. The resulting mixture was purified by chromatography on silica gel (eluent: PE/EA = 5:1) to afford the desired Boc-protected compound **E4** in 95% yield (96.1 mg) as a white solid (mp 158–160 °C). $[\alpha]_D^{20}$ = +75.9 (c = 1.0, $CHCl_3$). 98% ee (determined by HPLC: Chiralpak IA Column, 50/50 *i*-PrOH/hexane, 1.0 mL/min, 254 nm; TR = 6.08 min (major), 10.32 min (minor)). ^{1}H NMR (400 MHz, $CDCl_3$) δ 7.84 (d, *J* = 7.6 Hz, 1H), 7.73 (d, *J* = 2.4 Hz, 1H), 7.55 (d, *J* = 8.0 Hz, 1H), 7.47 (d, *J* = 7.6 Hz, 1H), 7.44 – 7.33 (m, 2H), 7.31 – 7.21 (m, 3H), 7.05 – 6.96 (m, 3H), 6.95 – 6.89 (m, 1H), 6.86 – 6.66 (m, 4H), 6.64 (d, *J* = 6.8 Hz, 1H), 5.48 (s, 1H), 3.00 (s, 6H), 2.22 (s, 3H), 1.57 (s, 9H); ^{13}C NMR (100 MHz, $CDCl_3$) δ 150.2, 148.3, 145.0, 143.8, 133.1, 132.8, 131.7, 130.8, 130.4, 129.0, 128.5, 127.8, 127.5, 127.3, 127.1, 126.5, 126.1, 126.0, 125.7, 121.7, 121.0, 118.9, 117.7, 111.2, 94.9, 91.7, 85.4, 84.5, 40.3, 27.9, 21.3; IR (neat): 2923(bs), 1741, 1654, 1528, 1492, 1460, 1382, 1252, 1151, 1084, 990 cm^{-1}; HRESIMS Calcd for $[C_{39}H_{37}N_3NaO_6S]^+$ (M + Na^+) 698.2295, found 698.2289.

(8*S*,8a*S*,13b*S*)-8-(4-(dimethylamino)phenyl)-6-(methylsulfonyl)-9-tosyl-6,8,8a,9-tetrahydro-13b*H*-benzo[3,4]pyrrolo[3',4':5,6]cyclohepta[1,2-*b*]indole-8,13b-diol (E5)

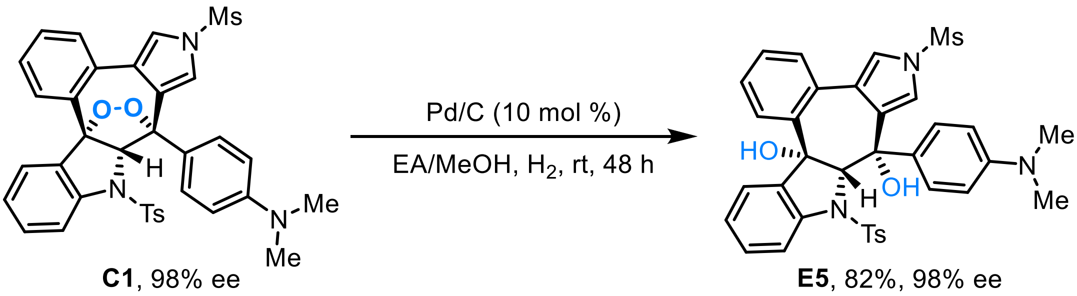

Compound **E5** was prepared in 82% yield (81.1 mg) according to the known procedure (0.20 mmol scale).[7] White solid (mp 136–137 °C). $[\alpha]_D^{20}$ = +288.1 (c = 1.0, $CHCl_3$). 98% ee (determined by HPLC: Chiralpak IC Column, 50/50 *i*-PrOH/hexane, 1.0 mL/min, 254 nm; TR = 9.53 min (major), 14.83 min (minor)). ^1H NMR (400 MHz, $CDCl_3$) δ 7.73 (d, *J* = 8.0 Hz, 1H), 7.58 (d, *J* = 7.6 Hz, 1H), 7.36 – 7.31 (m, 1H), 7.24 (s, 2H), 7.23 – 7.00 (m, 4H), 6.94 (s, 1H), 6.85 – 6.80 (m, 1H), 6.72 (d, *J* = 8.0 Hz, 2H), 6.67 (d, *J* = 8.0 Hz, 2H), 6.62 (d, *J* = 7.2 Hz, 2H), 6.22 (d, *J* = 7.2 Hz, 1H), 4.98 (s, 1H), 4.71 (s, 1H), 4.58 (s, 1H), 3.10 (s, 3H), 2.89 (s, 6H), 2.21 (s, 3H); ^{13}C NMR (100 MHz, $CDCl_3$) δ 149.4, 144.2, 143.5, 141.1, 135.9, 135.2, 133.5, 131.6, 131.4, 129.5, 128.7, 128.6, 128.3, 127.8, 127.6, 126.7, 126.1, 123.6, 121.5, 120.2, 117.0, 111.3, 82.9, 78.9, 77.2, 42.7, 40.4, 21.3; IR (neat): 3421(bs), 2925(bs), 1593, 1520, 1366, 1263, 1172, 1079, 982, 810 cm^{-1}; HRESIMS Calcd for $[C_{35}H_{33}N_3NaO_6S_2]^+$ (M + Na^+) 678.1703, found 678.1702.

(*R*)-*N*,*N*-dimethyl-4-(13-tosyl-13,13a-dihydro-6*H*-benzo[6,7]pyrrolo[3',4':4,5]cyclohepta[1,2-*b*]indol-8-yl)aniline (E6)

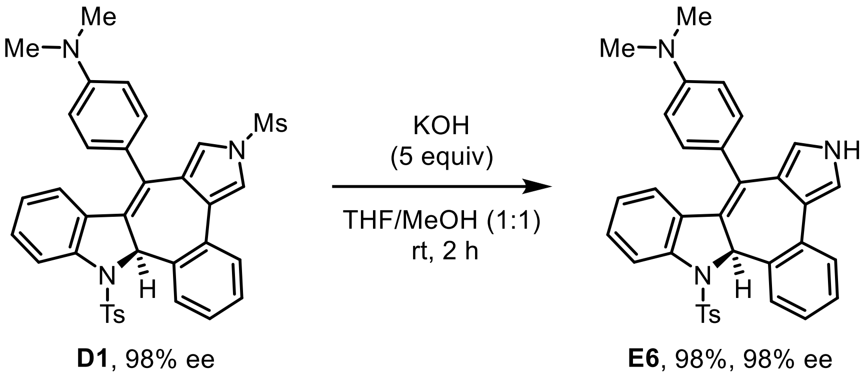

Compound **E6** was prepared in 98% yield (80.0 mg) according to the known procedure (0.15 mmol scale).[4] White solid (mp 143–145 °C). $[\alpha]_D^{20}$ = -124.3 (c = 1.0, $CHCl_3$). 98% ee (determined by HPLC: Chiralpak INA Column, 20/80 *i*-PrOH/hexane, 1.0 mL/min, 254 nm; TR = 6.88 min (major), 9.35 min (minor)). ^{1}H NMR (400 MHz, $CDCl_3$) δ 8.60 (s, 1H), 7.79 (d, *J* = 8.0 Hz, 1H), 7.76 (d, *J* = 7.6 Hz, 1H), 7.51 (d, *J* = 8.0 Hz, 2H), 7.39 (d, *J* = 7.6 Hz, 1H), 7.30 – 7.26 (m, 1H), 7.24 (d, *J* = 7.2 Hz, 1H), 7.12 (d, *J* = 8.0 Hz, 2H), 7.08 – 6.99 (m, 1H), 6.95 – 6.82 (m, 2H), 6.72 – 6.67 (m, 1H), 6.67 – 6.57 (m, 2H), 6.52 (dd, *J* = 8.4, 2.4 Hz, 1H), 6.33 (d, *J* = 7.2 Hz, 2H), 5.62 (s, 1H), 2.92 (s, 6H), 2.32 (s, 3H); ^{13}C NMR (100 MHz, $CDCl_3$) δ 150.0, 144.1, 143.8, 137.0, 134.4, 131.5, 131.2, 130.9, 130.4, 129.4, 129.1, 127.8, 127.6, 127.4, 127.3, 126.9, 126.7, 125.6, 124.7, 123.8, 123.6, 118.3, 117.8, 115.3, 112.4, 111.6, 68.3, 40.3, 21.4; IR (neat) 3401(bs), 2924(bs), 1608, 1522, 1456, 1354, 1263, 1166, 1089, 811, 756 cm^{-1}; HRESIMS Calcd for $[C_{34}H_{29}N_3NaO_2S]^+$ (M + Na$^+$) 566.1873, found 566.1865.

(*R*)-*N,N*-dimethyl-4-(6-(methylsulfonyl)-13,13a-dihydro-6*H*-benzo[6,7]pyrrolo[3',4':4,5]cyclohepta[1,2-*b*]indol-8-yl)aniline (E7)

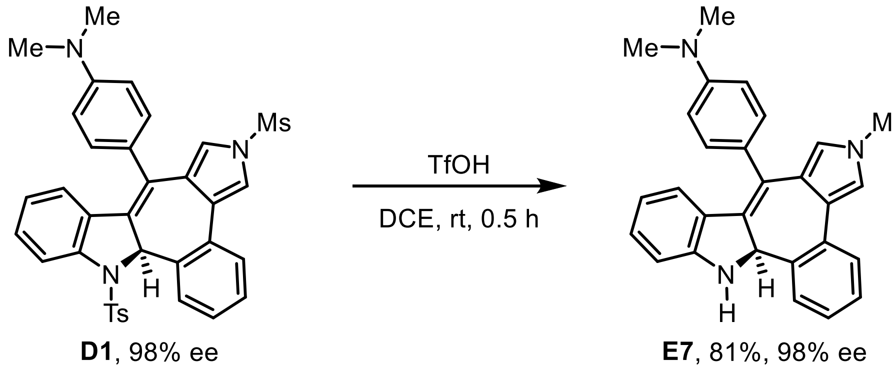

To an oven-dried 10-mL Schlenk tube with a stir bar were added **D1** (0.15 mmol, 93.3 mg), DCE (3 mL) and TfOH (1.5 mL) were added sequentially and stirred at rt for 0.5 h. Upon completion, appropriate saturated sodium carbonate solution was added to quench the reaction. The mixed solution was then extracted by DCE/H_2O and washed with brine 3 times. The organic phase was dried over Na_2SO_4 and concentrated. The resulting mixture was purified by chromatography on silica gel (eluent: PE/EtOAc = 3:1) to obtain compound **E7** in 81% yield (56.9 mg) as a white solid (mp 154–155 °C). $[\alpha]_D^{20}$ = -159.8 (c = 1.0, $CHCl_3$). 98% ee (determined by HPLC: Chiralpak IB Column, 50/50 *i*-PrOH/hexane, 1.0 mL/min, 220 nm; TR = 8.22 min (minor), 16.29 min (major)). ^{1}H NMR (400 MHz, $CDCl_3$) δ 7.49 (d, *J* = 8.4 Hz, 2H), 7.44 (d, *J* = 2.4 Hz, 1H), 7.36 – 7.25 (m, 3H), 7.05 – 6.93 (m, 2H), 6.78 (d, *J* = 2.4 Hz, 1H), 6.76 (d, *J* = 8.0 Hz, 1H), 6.72 (d, *J* = 8.0 Hz, 2H), 6.48 – 6.32 (m, 2H), 5.30 (s, 1H), 4.74 – 4.32 (m, 1H), 3.17 (s, 3H), 3.00 (s, 6H).; ^{13}C NMR (100 MHz, $CDCl_3$) δ 153.7, 149.9, 139.5, 137.5, 131.7, 130.8, 130.3, 130.2, 129.0, 128.3, 128.2, 127.9, 127.8, 127.0, 125.1, 124.6, 124.5, 122.4, 118.3, 118.1, 115.9, 112.9, 112.3, 109.3, 62.7, 42.9, 40.5; IR (neat): 3421(bs), 2923(bs), 1959, 1734, 1607, 1522, 1467, 1366, 1173, 1084, 980, 765 cm^{-1}; HRESIMS Calcd for $[C_{28}H_{25}N_3NaO_2S]^+$ (M + Na^+) 490.1560, found 490.1560.

dimethyl (5*S*,8*R*,9*S*)-9-(4-(dimethylamino)phenyl)-15-(methylsulfonyl)-14-tosyl-5,8,9,14-tetrahydro-5,8-epiminodibenzo[4,5:6,7]cyclohepta[1,2-*b*]indole-6,7-dicarboxylate (E8)

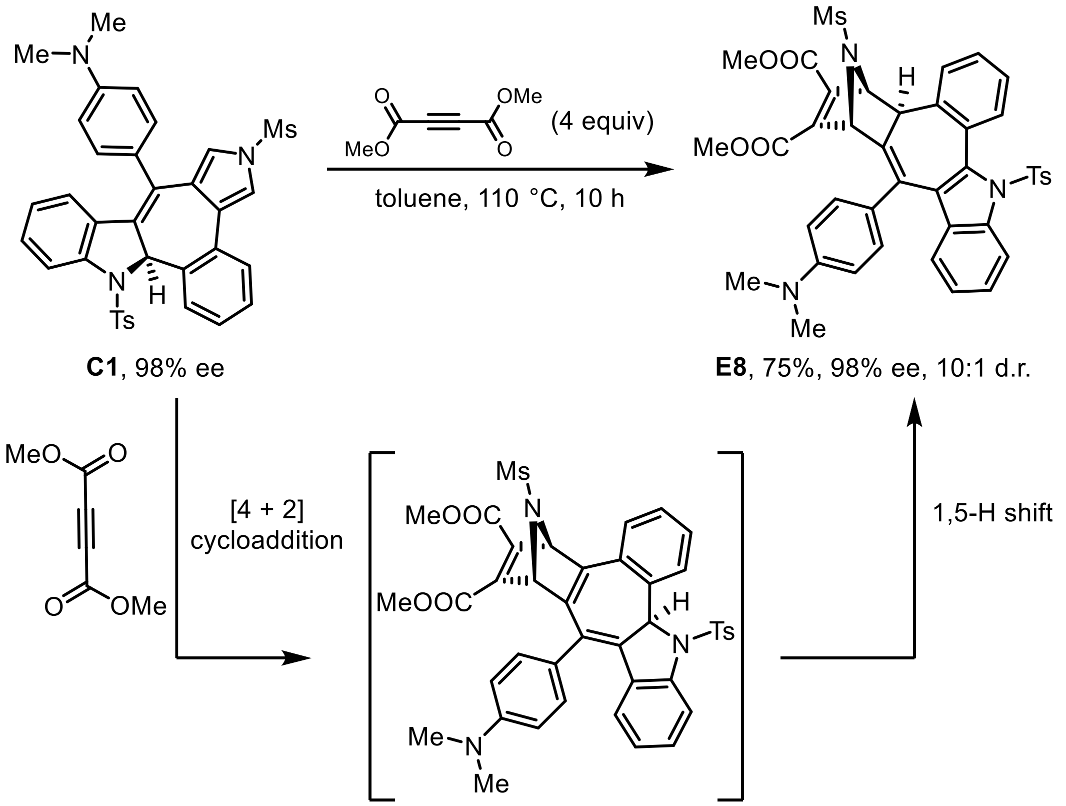

Compound **E8** was prepared in 75% yield (86.4 mg) according to the known procedure (0.15 mmol scale).[6] White solid (mp 178–181 °C). $[\alpha]_D^{20}$ = -168.4 (c = 1.0, $CHCl_3$). 98% ee (determined by HPLC: Chiralpak IA Column, 30/70 *i*-PrOH/hexane, 1.0 mL/min, 254 nm; TR = 8.82 min (minor), 11.64 min (major)) dr = 10:1 (determined by ^1H NMR). ^1H NMR (400 MHz, $CDCl_3$) δ 8.33 (d, *J* = 8.4 Hz, 1H), 7.98 (d, *J* = 7.6 Hz, 1H), 7.58 (d, *J* = 4.0 Hz, 2H), 7.46 – 7.38 (m, 1H), 7.31 – 7.25 (m, 1H), 7.18 (d, *J* = 8.4 Hz, 2H), 7.03 – 6.90 (m, 3H), 6.85 (d, *J* = 8.4 Hz, 2H), 6.57 (d, *J* = 8.8 Hz, 2H), 6.36 (d, *J* = 8.0 Hz, 1H), 5.79 (d, *J* = 1.6 Hz, 1H), 5.35 (d, *J* = 1.6 Hz, 1H), 3.99 (s, 3H), 3.86 (s, 3H), 2.93 (s, 6H), 2.86 (s, 3H), 2.76 (s, 1H), 2.24 (s, 3H); ^{13}C NMR (100 MHz, $CDCl_3$) δ 162.7, 162.4, 150.1, 146.1, 144.8, 141.2, 138.2, 138.1, 135.5, 135.1, 133.4, 133.1, 130.1, 129.9, 129.7, 129.6, 129.2, 129.1, 126.7, 126.0, 125.2, 125.1, 125.0, 124.2, 123.2, 122.0, 116.9, 111.7, 66.9, 66.8, 52.9, 52.6, 45.6, 40.5, 40.2, 21.5; IR (neat): 2958(bs), 1720, 1611, 1572, 1436, 1345, 1262, 1177, 1157, 1019, 764 cm^{-1}; HRESIMS Calcd for $[C_{41}H_{37}N_3NaO_8S_2]^+$ (M + Na^+) 786.1914, found 786.1909.

5. Crystal Data

Crystal data and structure refinement for B1. CCDC number: 2400870.

ORTEP drawing of **B1** (thermal ellipsoids set at 50% probability). Recrystallization from n-hexane/CH_2Cl_2 afforded single crystals suitable for X-ray diffraction analysis.

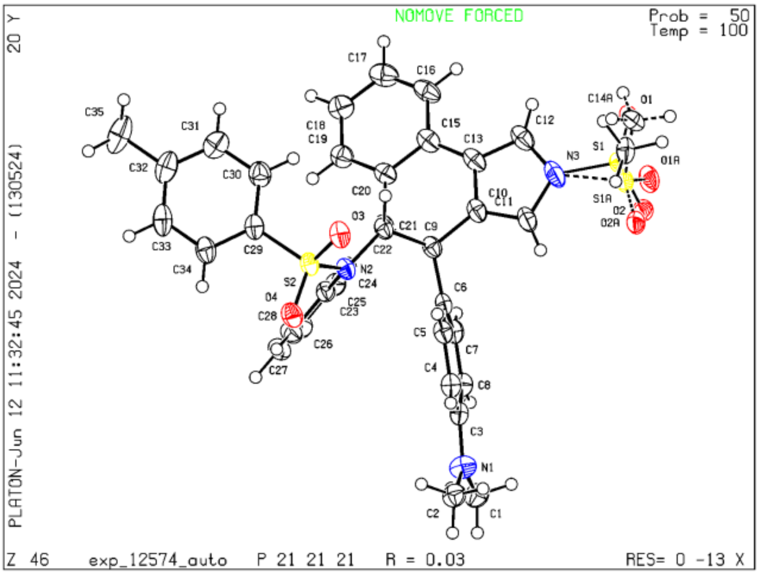

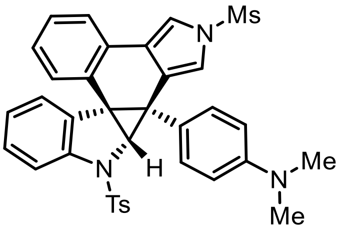

Bond precision: C-C = 0.0035 A Wavelength=1.54184

Cell: a=9.9441(1) b=11.7291(1) c=25.7758(2)
alpha=90 beta=90 gamma=90

Temperature: 100 K

	Calculated	Reported
Volume	3006.37(5)	3006.37(5)
Space group	P 21 21 21	P 21 21 21
Hall group	P 2ac 2ab	P 2ac 2ab
Moiety formula	C35 H31 N3 O4 S2	C35 H31 N3 O4 S2
Sum formula	C35 H31 N3 O4 S2	C35 H31 N3 O4 S2
Mr	621.75	621.75
Dx,g cm-3	1.374	1.374
Z	4	4
Mu (mm-1)	1.975	1.975
F000	1304.0	1304.0
F000’	1310.18	
h,k,lmax	12,14,32	12,14,32
Nref	6241[3524]	5994
Tmin,Tmax	0.977,0.980	0.599,1.000
Tmin’	0.674	

Correction method= # Reported T Limits: Tmin=0.599 Tmax=1.000
AbsCorr = MULTI-SCAN

Data completeness= 1.70/0.96 Theta(max)= 75.468

R(reflections)= 0.0318(5873) wR2(reflections)= 0.0786(5994)

S = 1.062 Npar= 442

Crystal data and structure refinement for B32. CCDC number: 2401817.

ORTEP drawing of **B32** (thermal ellipsoids set at 50% probability). Recrystallization from n-hexane/CH_2Cl_2 afforded single crystals suitable for X-ray diffraction analysis.

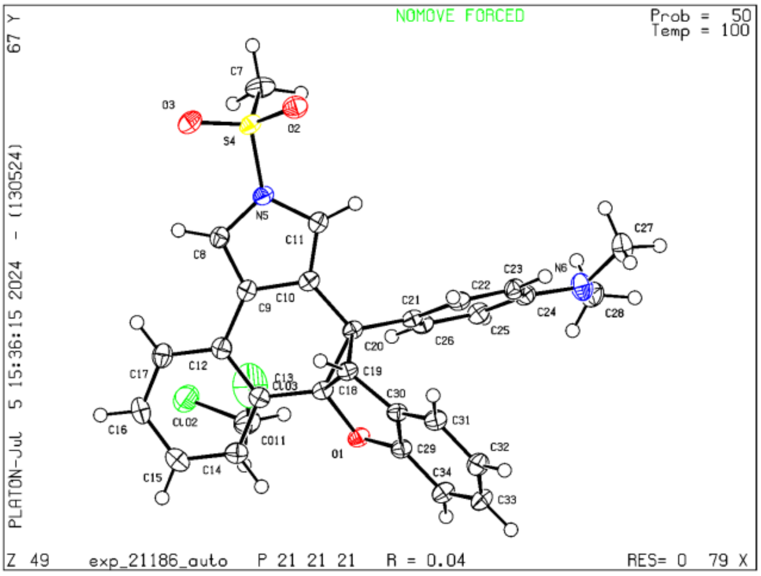

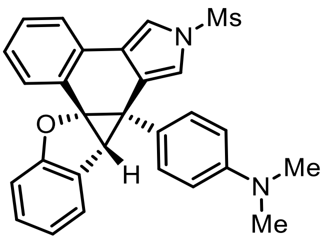

Bond precision: C-C = 0.0045 A Wavelength=1.54184

Cell:	a=9.19204(17)	b=13.1056(2)	c=21.5561(4)
	alpha=90	beta=90	gamma=90
Temperature:	100 K		

	Calculated	Reported
Volume	2596.80(8)	2596.80(8)
Space group	P 21 21 21	P 21 21 21
Hall group	P 2ac 2ab	P 2ac 2ab
Moiety formula	C28 H24 N2 O3 S, C H2 Cl2	0.364(C H2 Cl2), 0.364(C28 H24 N2 O3 S)
Sum formula	C29 H26 Cl2 N2 O3 S	C10.55 H9.45 Cl0.73 N0.73 O1.09 S0.36
Mr	553.48	201.26
Dx,g cm-3	1.416	1.416
Z	4	11
Mu (mm-1)	3.286	3.286
F000	1152.0	1152.0
F000’	1159.08	
h,k,lmax	11,16,26	11,16,26
Nref	5309[3009]	5172
Tmin,Tmax	0.924,0.936	0.599,1.000
Tmin’	0.848	

Correction method= # Reported T Limits: Tmin=0.599 Tmax=1.000
AbsCorr = MULTI-SCAN

Data completeness= 1.72/0.97 Theta(max)= 74.355

R(reflections)= 0.0387(4870) wR2(reflections)= 0.1010(5172)

S = 1.051 Npar= 337

Crystal data and structure refinement for C1. CCDC number:2400872.

ORTEP drawing of **C1** (thermal ellipsoids set at 50% probability). Recrystallization from n-hexane/CH_2Cl_2/THF afforded single crystals suitable for X-ray diffraction analysis.

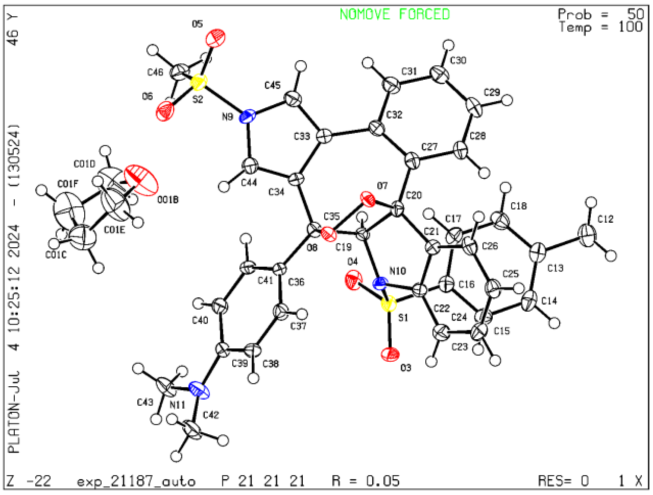

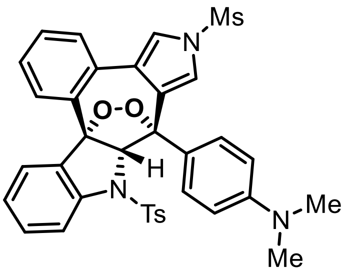

Bond precision: C-C = 0.0072 A Wavelength=1.54184

Cell:	a=8.8353(3)	b=11.1372(4)	c=35.3642(13)
	alpha=90	beta=90	gamma=90
Temperature:	100 K		

	Calculated	Reported
Volume	3479.9(2)	3479.9(2)
Space group	P 21 21 21	P 21 21 21
Hall group	P 2ac 2ab	P 2ac 2ab
Moiety formula	C35 H31 N3 O6 S2, C4 H8 O	0.267(C35 H31 N3 O6 S2), 0.267(C4 H8 O)
Sum formula	C39 H39 N3 O7 S2	C10.40 H10.40 N0.80 O1.87 S0.53
Mr	725.85	193.56
Dx,g cm-3	1.385	1.385
Z	4	15
Mu (mm-1)	1.853	1.853
F000	1528.0	1528.0
F000’	1535.04	
h,k,lmax	11,13,44	11,13,43
Nref	7070[4009]	6930
Tmin,Tmax	0.957,0.964	0.796,1.000
Tmin’	0.912	

Correction method= # Reported T Limits: Tmin=0.796 Tmax=1.000
AbsCorr = MULTI-SCAN

Data completeness= 1.73/0.98 Theta(max)= 74.268

R(reflections)= 0.0535(6300) wR2(reflections)= 0.1427(6930)

S = 1.041 Npar= 464

Crystal data and structure refinement for D9. CCDC number: 2400882.

ORTEP drawing of **D9** (thermal ellipsoids set at 50% probability). Recrystallization from n-hexane/CH_2Cl_2 afforded single crystals suitable for X-ray diffraction analysis.

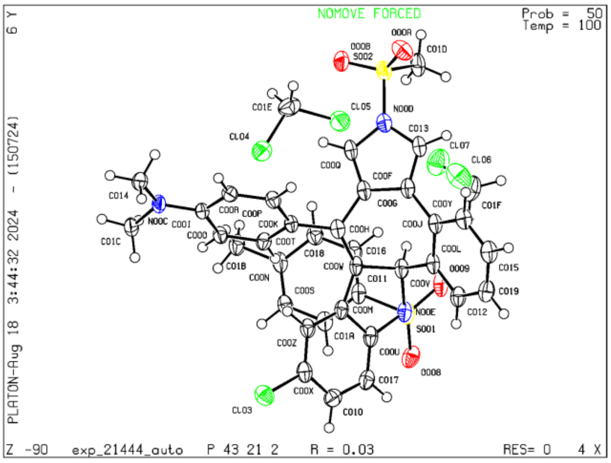

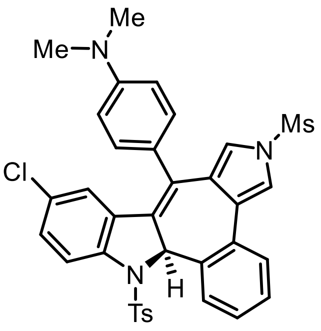

Bond precision:	C-C = 0.0045 A		Wavelength=1.54184
Cell:	a=14.4285(1)	b=14.4285(1)	c=36.1670(3)
	alpha=90	beta=90	gamma=90
Temperature:	100 K		

	Calculated	Reported
Volume	7529.31(12)	7529.30(12)
Space group	P 43 21 2	P 43 21 2
Hall group	P 4nw 2abw	P 4nw 2abw
Moiety formula	C35 H30 Cl N3 O4 S2, 2(C H2 Cl2) [+ solvent]	0.5(C35 H30 Cl N3 O4 S2), C H2 Cl2
Sum formula	C37 H34 Cl5 N3 O4 S2 [+ solvent]	C18.50 H17 Cl2.50 N1.50 O2 S
Mr	826.04	413.02
Dx,g cm-3	1.457	1.457
Z	8	16
Mu (mm-1)	4.911	4.911
F000	3408.0	3408.0
F000'	3435.43	
h,k,lmax	17,17,42	17,17,42
Nref	6594[3827]	6549
Tmin,Tmax	0.889,0.906	0.474,1.000
Tmin'	0.782	

Correction method= # Reported T Limits: Tmin=0.474 Tmax=1.000
AbsCorr = MULTI-SCAN

Data completeness= 1.71/0.99 Theta(max)= 66.142

R(reflections)= 0.0311(6290) wR2(reflections)= 0.0873(6549)

S = 1.019 Npar= 464

Crystal data and structure refinement for E3. CCDC number: 2400880.

ORTEP drawing of **E3** (thermal ellipsoids set at 50% probability). Recrystallization from n-hexane/CH_2Cl_2 afforded single crystals suitable for X-ray diffraction analysis.

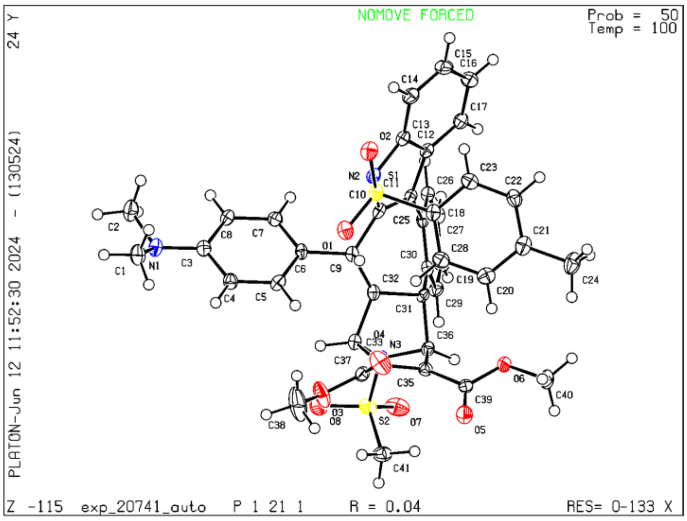

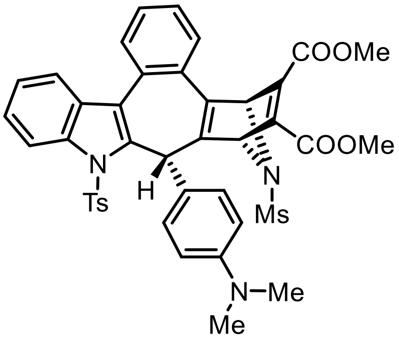

Bond precision:	C-C = 0.0041 A		Wavelength=0.71073
Cell:	a=10.4738(2)	b=10.2663(1)	c=16.8723(2)
	alpha=90	beta=91.911(1)	gamma=90
Temperature:	100 K		

	Calculated	Reported
Volume	1813.22(4)	1813.22(4)
Space group	P 21	P 1 21 1
Hall group	P 2yb	P 2yb
Moiety formula	C41 H37 N3 O8 S2	0.25(C41 H37 N3 O8 S2)
Sum formula	C41 H37 N3 O8 S2	C10.25 H9.25 N0.75 O2 S0.50
Mr	763.86	190.96
Dx,g cm-3	1.399	1.399
Z	2	8
Mu (mm-1)	0.207	0.207
F000	800.0	800.0
F000'	800.86	
h,k,lmax	14,14,23	14,14,22
Nref	10258[5397]	9339
Tmin,Tmax	0.959,0.959	0.665,1.000
Tmin'	0.959	

Correction method= # Reported T Limits: Tmin=0.665 Tmax=1.000
AbsCorr = MULTI-SCAN

Data completeness= 1.73/0.91 Theta(max)= 29.645

R(reflections)= 0.0437(8057) wR2(reflections)= 0.1008(9339)

S = 1.067 Npar= 493

Crystal data and structure refinement for E8. CCDC number: 2400881.

ORTEP drawing of **E8** (thermal ellipsoids set at 50% probability). Recrystallization from n-hexane/CH_2Cl_2 afforded single crystals suitable for X-ray diffraction analysis.

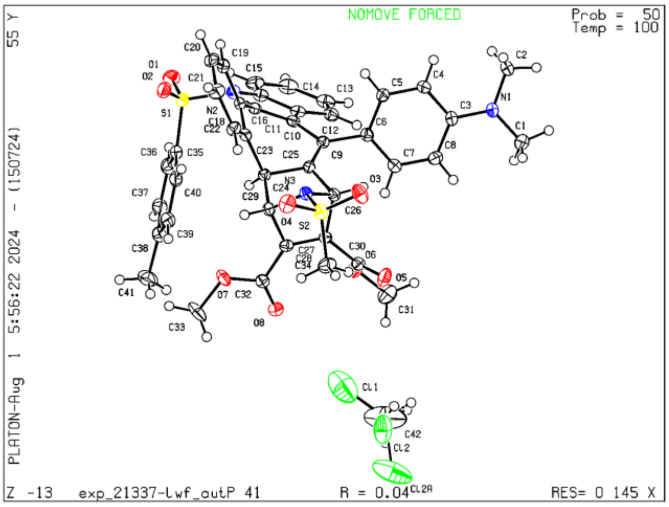

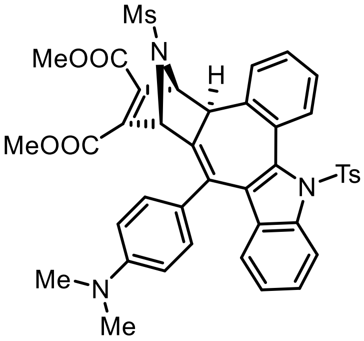

Bond precision: C-C = 0.0057 A Wavelength=1.54184

Cell:	a=13.74447(4)	b=13.74447(4)	c=21.32100(13)
	alpha=90	beta=90	gamma=90
Temperature:	100 K		

	Calculated	Reported
Volume	4027.76(3)	4027.76(4)
Space group	P 41	P 41
Hall group	P 4w	P 4w
Moiety formula	C41 H37 N3 O8 S2, C H2 Cl2	C41 H37 N3 O8 S2, C H2 Cl2
Sum formula	C42 H39 Cl2 N3 O8 S2	C42 H39 Cl2 N3 O8 S2
Mr	848.78	848.78
Dx,g cm-3	1.400	1.400
Z	4	4
Mu (mm-1)	2.897	2.897
F000	1768.0	1768.0
F000’	1778.39	
h,k,lmax	16,16,25	16,16,25
Nref	7015[3613]	6971
Tmin,Tmax	0.786,0.748	0.363,1.000
Tmin’	0.713	

Correction method= # Reported T Limits: Tmin=0.363 Tmax=1.000
AbsCorr = CYLINDER

Data completeness= 1.93/0.99 Theta(max)= 65.957

R(reflections)= 0.0385(6807) wR2(reflections)= 0.0997(6971)

S = 1.032 Npar= 530

6. Theoretical Calculations

6.1 Computational Details

All calculations were performed using Gaussian16 package.[8] Geometry optimizations and vibrational analysis were conducted under the B3LYP-D3(BJ) level of theory[9,10] with the LANL2DZ basis set[11,12] for Cu atom, and the 6-31G(d) basis set[13] for C, H, O, N, and S atom. Electronic energy of all the intermediates and transition states were recomputed at the B3LYP-D3(BJ)/def2TZVP[14,15] level of theory. All local minimums were confirmed with no imaginary frequency and all transition states had only one imaginary frequency. And every transition state was checked by intrinsic reaction coordinate (IRC) analysis. The SMD solvation model[16] with THF was used for single point calculations. The ball stick models of molecules were drawn by CYLview 1.0.[17]

To correct the Gibbs free energies under 1 atm to the standard state in solution (1 mol/L), a correction of RTln(cs/cg) is added to energies of all species. cs stands for the standard molar concentration in solution (1 mol/L), cg stands for the standard molar concentration in gas phase (0.040876 mol/L), and R is the gas constant. For calculated intermediates at the standard state of 1 mol/L at 298.15 K, the correction value equaling to 1.89 kcal/mol was used.

6.2 Alternative Reaction Pathways from Int-B2

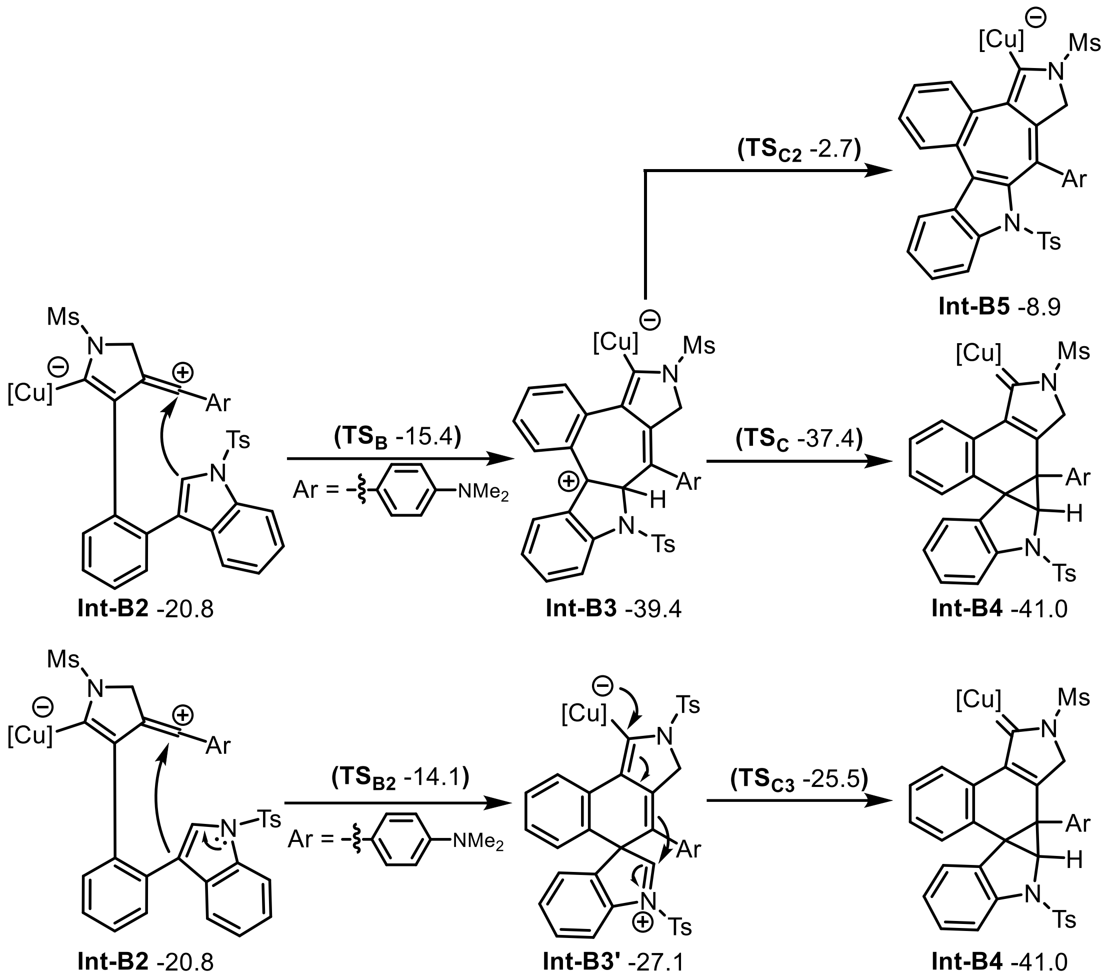

Figure S1: Alternative reaction pathways starting from **Int-B2**.

XYZ Coordinates

A1

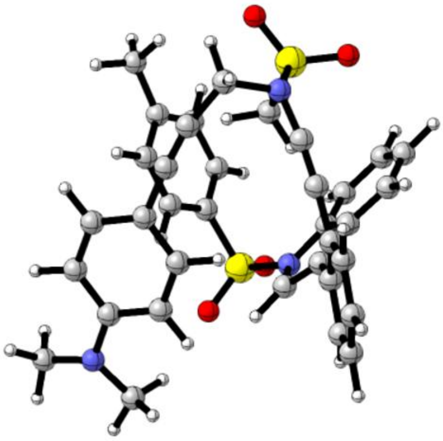

N	3.19035000	0.38330900	-2.18852500
C	2.54133100	0.12339600	-3.51216500
H	2.97824000	-0.80023600	-3.89678000
H	2.85243900	0.94308400	-4.16922700
C	1.08697900	0.06587100	-3.39455900
C	-0.08497800	0.16049700	-3.09543900
C	-1.42429500	0.31270100	-2.64123000
C	-1.70779400	1.26166900	-1.63975200
C	-2.47896600	-0.50167400	-3.08829400
C	-2.97038000	1.35708800	-1.07816700
H	-0.91235000	1.90832600	-1.28643200
C	-3.74377000	-0.42030100	-2.52260500
H	-2.29345200	-1.22690200	-3.87521700
C	-4.02321300	0.50042900	-1.48305800
H	-3.13987400	2.10112300	-0.31044500
H	-4.51402200	-1.09141100	-2.88015100
N	-5.26664100	0.56548900	-0.88500000
C	-5.38969300	1.25119100	0.39662000

H	-6.41737600	1.15441800	0.75039600
H	-4.71471700	0.83067200	1.15489200
H	-5.18015800	2.32138200	0.28877900
C	-6.24837500	-0.46033700	-1.19370800
H	-7.17507400	-0.23585900	-0.66314200
H	-6.47480700	-0.47013800	-2.26536500
H	-5.91531400	-1.46887100	-0.90125400
C	2.67920600	1.40640000	-1.46934100
C	2.11367000	2.30240100	-0.87771500
C	1.40292700	3.37685900	-0.26768500
C	0.52547900	3.16984400	0.82998000
C	1.49559900	4.66010400	-0.84047900
C	-0.24310500	4.24913900	1.28793400
C	0.71973500	5.71400100	-0.37024400
H	2.17261900	4.80455300	-1.67606900
C	-0.16005400	5.50666300	0.69375200
H	-0.90051700	4.08965800	2.13734700
H	0.80194400	6.69336500	-0.83217100
H	-0.76842100	6.32389800	1.06989700
C	0.39219100	1.86085200	1.49068600
C	1.44800300	1.04894700	2.07534100
C	-0.78925300	1.22024100	1.74331700
C	0.83350300	-0.07409700	2.68572700
C	2.84340900	1.17540700	2.12169500
H	-1.79758700	1.46987500	1.45964900
C	1.56727200	-1.06865200	3.33177500
C	3.58499700	0.17047400	2.73245500
H	3.33463000	2.02259700	1.65704700
C	2.95422800	-0.93434600	3.33222500

H	1.07610200	-1.89676400	3.82682600
H	4.66766600	0.23530200	2.73776600
H	3.55904700	-1.69653800	3.81415800
N	-0.55483800	0.05851400	2.49035400
S	-1.67294900	-1.24217500	2.53753500
O	-2.95400600	-0.64428400	2.16366700
O	-1.46802500	-1.91146500	3.81858600
C	-1.10875200	-2.31865700	1.23846200
C	-1.44448600	-2.02926800	-0.08505000
C	-0.29710100	-3.40806700	1.56339500
C	-0.92588700	-2.83570800	-1.09441700
H	-2.09360200	-1.19534200	-0.32176700
C	0.21353500	-4.19714000	0.53628800
H	-0.08315300	-3.63287400	2.60105100
C	-0.07167500	-3.90943900	-0.80566900
H	-1.17045000	-2.60768400	-2.12559800
H	0.85431900	-5.04040200	0.77908100
C	0.58450000	-4.68563400	-1.91798200
H	-0.04678300	-4.71992100	-2.81129100
H	1.53158900	-4.20647000	-2.20274300
H	0.81359000	-5.71192000	-1.61478400
S	3.66039400	-1.00861600	-1.25204500
O	4.71762300	-0.56385400	-0.35128600
O	3.88527700	-2.07640600	-2.23170900
C	2.18941100	-1.38382300	-0.30696300
H	1.38628000	-1.61113800	-1.00563000
H	2.41665700	-2.23973700	0.32867700
H	1.93917800	-0.51869100	0.30200200

Int-B1

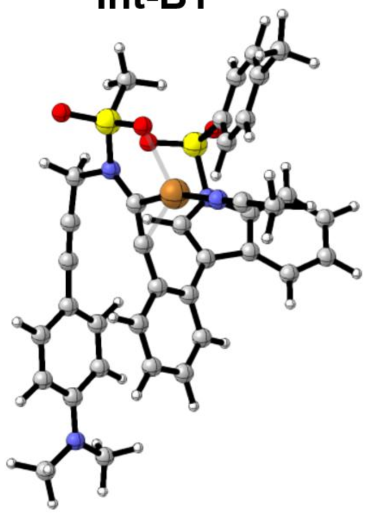

N	-0.91549400	-1.73080700	-1.64710200
C	-0.08031400	-1.49836300	-2.89639200
H	-0.52985900	-0.65159700	-3.42413100
H	-0.16686000	-2.40059800	-3.51023200
C	1.29702400	-1.22100500	-2.56340700
C	2.43561500	-0.97436500	-2.21330100
C	3.73217000	-0.69275400	-1.72184600
C	4.67946200	-1.71983900	-1.52983400
C	4.08800200	0.61514800	-1.33577100
C	5.91578800	-1.45953600	-0.96838100
H	4.42838400	-2.73282600	-1.82847900
C	5.31885000	0.88467500	-0.76742700
H	3.37554300	1.42160600	-1.47661200
C	6.26881000	-0.14792500	-0.55639200
H	6.61496200	-2.27670700	-0.84601600
H	5.54817400	1.90365800	-0.48418800
N	7.48224600	0.11155300	0.02825500
C	7.83993300	1.47437600	0.38762000
H	8.82726800	1.47535200	0.84979200

H	7.87036800	2.13663200	-0.48835200
H	7.12824300	1.89508200	1.10991400
C	8.47261700	-0.94666900	0.15332200
H	9.36516700	-0.54611700	0.63429300
H	8.09844500	-1.77360400	0.77048400
H	8.76337200	-1.35283100	-0.82487200
C	-0.40498700	-1.60980400	-0.38837300
C	0.64599200	-1.21127400	0.18349300
C	1.69932800	-0.64952700	0.92694800
C	1.75259800	0.75767500	1.18387200
C	2.75013900	-1.48248200	1.37527200
C	2.81965100	1.25140700	1.94220900
C	3.80842000	-0.96029500	2.10172100
H	2.71038300	-2.53862600	1.13219300
C	3.83390000	0.40821800	2.39199400
H	2.87875000	2.31787800	2.13226000
H	4.61640400	-1.60546800	2.42921700
H	4.66409100	0.82495300	2.95456100
C	0.74080100	1.66305200	0.64157600
C	0.08878200	2.78138100	1.31057400
C	0.22110900	1.59061000	-0.62362900
C	-0.83011400	3.34365900	0.39091500
C	0.16911100	3.31737400	2.60359700
H	0.46981900	0.93715000	-1.44209300
C	-1.66364800	4.41259700	0.71184500
C	-0.65774300	4.38582900	2.93586500
H	0.85694200	2.90166000	3.33297800
C	-1.56083700	4.92610700	2.00261100
H	-2.35089700	4.82822900	-0.01546300

H	-0.60438400	4.81350500	3.93225100
H	-2.18805100	5.76389900	2.29017700
N	-0.71558800	2.61197900	-0.81241500
S	-2.00223000	2.41107400	-1.92374400
O	-1.52022300	1.41661200	-2.88669700
O	-2.44281000	3.73074400	-2.35428400
C	-3.27564400	1.68152400	-0.91007400
C	-2.94228700	0.79952900	0.12159800
C	-4.60445100	1.93341100	-1.24823700
C	-3.96495600	0.15564600	0.80859300
H	-1.90652200	0.61601000	0.38088300
C	-5.61369500	1.26523900	-0.55455700
H	-4.84180400	2.63802400	-2.03797100
C	-5.31299300	0.36600300	0.47645800
H	-3.71678800	-0.52934000	1.61493800
H	-6.65109000	1.44994200	-0.81741000
C	-6.40362700	-0.34942100	1.23128300
H	-6.17657300	-1.41647900	1.33426300
H	-6.51266700	0.06241500	2.24235800
H	-7.36977800	-0.25323800	0.72884100
S	-2.35312500	-2.63010500	-1.84121800
O	-2.84421000	-2.88844700	-0.46337300
O	-2.10429700	-3.74358800	-2.75040300
C	-3.47273800	-1.47061300	-2.62259300
H	-2.97365800	-1.01946000	-3.48178400
H	-4.34267200	-2.04820000	-2.94244600
H	-3.74458700	-0.71112700	-1.89305900
Cu	-1.44879600	-2.26034600	1.14362400
N	-2.09245100	-2.73097200	2.88583000

C	-2.48923200	-3.08828400	3.91248300
C	-2.98716200	-3.54108300	5.20284600
H	-3.32409400	-2.68353400	5.79359700
H	-3.82572500	-4.22894900	5.05585300
H	-2.19021900	-4.05890700	5.74574600

TS$_A$

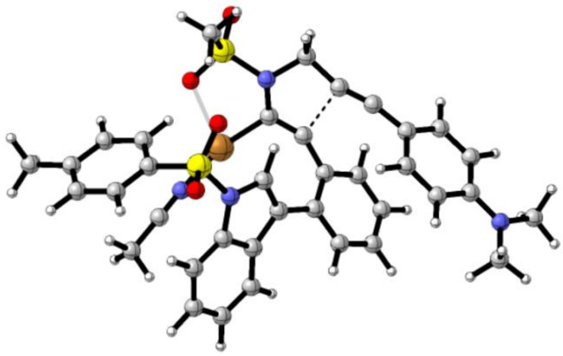

N	-0.16586400	2.95306900	0.04048300
C	1.02588700	3.70104000	0.55133500
H	0.83420900	4.01086600	1.58345500
H	1.19245000	4.59721200	-0.05945400
C	2.16675500	2.79233900	0.47086200
C	3.24773200	2.26426900	0.74387400
C	4.37971000	1.45278300	0.80724700
C	5.58481600	1.80739100	0.14964600
C	4.31015700	0.18449100	1.43570900
C	6.65213900	0.94046900	0.10417800
H	5.65669700	2.77760900	-0.33108700
C	5.36405600	-0.69824200	1.37356000
H	3.39452700	-0.10268300	1.94148400
C	6.57053500	-0.35160600	0.70056400
H	7.55564900	1.24856200	-0.40525100

H	5.26267200	-1.66642300	1.84534600
N	7.61168600	-1.22614900	0.62837500
C	7.50183400	-2.55045100	1.22836500
H	8.42263100	-3.10304800	1.04464100
H	7.34977300	-2.48883500	2.31283000
H	6.66944600	-3.11647300	0.79173300
C	8.84710400	-0.84836600	-0.04883300
H	9.55597800	-1.67310100	0.01659300
H	8.67192600	-0.63040900	-1.10977200
H	9.30435100	0.03261100	0.41710000
C	0.10467300	1.94825800	-0.87311600
C	1.28819800	1.44354800	-1.03212100
C	2.09937400	0.30940800	-1.29445800
C	1.75726700	-0.99249800	-0.80432100
C	3.31319700	0.49002500	-1.99055300
C	2.67044300	-2.03427500	-1.03019500
C	4.19493000	-0.56069100	-2.19714900
H	3.54857000	1.48634400	-2.34851500
C	3.87328600	-1.82569400	-1.70138100
H	2.44678600	-3.01836300	-0.63392900
H	5.12919400	-0.39388300	-2.72251000
H	4.56010900	-2.65596100	-1.83726000
C	0.49473300	-1.30423700	-0.12143800
C	-0.25575100	-2.54677800	-0.25957900
C	-0.17358800	-0.54608800	0.81510600
C	-1.33718800	-2.50044400	0.65824000
C	-0.13399500	-3.66709400	-1.09738300
H	0.05617800	0.41492800	1.24237700
C	-2.23760100	-3.55409400	0.81787400

C	-1.04050200	-4.71448300	-0.96307600
H	0.65502900	-3.71689500	-1.83960600
C	-2.06831700	-4.66740400	-0.00210800
H	-3.01634800	-3.52406900	1.56836900
H	-0.94299200	-5.59034300	-1.59713600
H	-2.73812300	-5.51467200	0.10885000
N	-1.26919300	-1.25703500	1.30003800
S	-2.42872500	-0.60724700	2.39532400
O	-1.94018800	0.74902700	2.65040100
O	-2.60884300	-1.59193800	3.45313700
C	-3.91850000	-0.53071000	1.42984900
C	-4.06484100	0.48990900	0.49036200
C	-4.92437400	-1.47558500	1.63975600
C	-5.22856400	0.54678900	-0.26856600
H	-3.29297600	1.23372000	0.34526200
C	-6.08485500	-1.40059500	0.87146000
H	-4.80764500	-2.23439800	2.40484000
C	-6.25574100	-0.39431900	-0.09243400
H	-5.33862200	1.34054100	-1.00192600
H	-6.87743800	-2.12561200	1.03368400
C	-7.52917700	-0.29927000	-0.89315900
H	-8.22705000	0.40393100	-0.42147500
H	-7.33881100	0.06641800	-1.90715400
H	-8.03644100	-1.26612800	-0.96122500
S	-1.62559500	3.82140800	-0.14008000
O	-2.52814000	2.91127700	-0.87950800
O	-1.35540500	5.16633100	-0.64200600
C	-2.19910800	3.91415000	1.55618300
H	-1.50662000	4.53586200	2.12690200

H	-3.18175800	4.39062800	1.52622000
H	-2.25484600	2.90409300	1.96663900
Cu	-1.34342200	0.95815500	-1.70035200
N	-2.48353600	-0.45755700	-2.25433300
C	-3.13880200	-1.41103900	-2.25632300
C	-3.97511600	-2.59736600	-2.23145400
H	-3.36590100	-3.46120700	-1.94836500
H	-4.76497100	-2.45601400	-1.48683100
H	-4.42314700	-2.76299500	-3.21577900

Int-B2

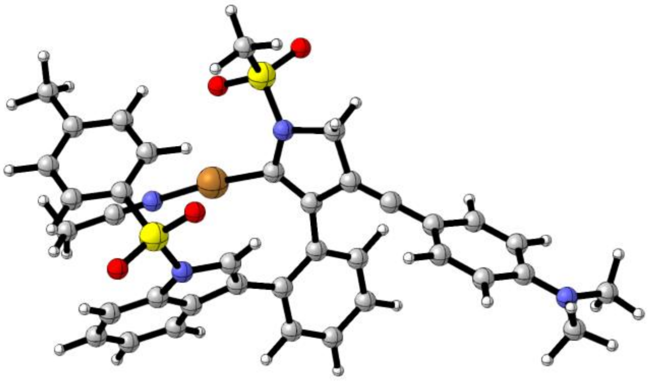

N	0.24043500	2.48320300	-0.17556900
C	1.66473200	2.74734200	0.15883800
H	1.77535500	2.99078300	1.21840500
H	2.05669600	3.56957900	-0.44760800
C	2.31905400	1.42245000	-0.19673600
C	3.54291500	1.08379100	0.11209600
C	4.82245200	0.70350200	0.31682000
C	5.84381800	0.93069400	-0.67540400
C	5.22162400	0.03559700	1.53008200
C	7.13322400	0.53444700	-0.47666300
H	5.56178900	1.42966300	-1.59652700

C	6.50758100	-0.37157400	1.73182200
H	4.46553600	-0.14655300	2.28639700
C	7.51655000	-0.13575100	0.73613200
H	7.87019600	0.72344100	-1.24553600
H	6.76512300	-0.87601200	2.65328300
N	8.78513900	-0.53245200	0.93255100
C	9.17247900	-1.22320400	2.16788600
H	8.61154900	-2.15584800	2.28557300
H	10.23305400	-1.46320000	2.12102500
H	8.99968100	-0.58687300	3.04181400
C	9.81476600	-0.28665900	-0.08355700
H	10.76610400	-0.67392500	0.27643800
H	9.56712600	-0.79392100	-1.02164100
H	9.92763600	0.78548800	-0.27335700
C	0.09278900	1.25262900	-0.85205900
C	1.29288900	0.59473100	-0.85422200
C	1.60540400	-0.73737600	-1.41352800
C	0.86902600	-1.91340600	-1.12495200
C	2.70744600	-0.82931900	-2.27933500
C	1.28102600	-3.12369200	-1.71202000
C	3.09442400	-2.03396000	-2.85827700
H	3.25087400	0.07985100	-2.51978400
C	2.37569300	-3.19311900	-2.56795700
H	0.74604400	-4.03222000	-1.45761500
H	3.94392500	-2.06523400	-3.53424600
H	2.66683300	-4.14658200	-2.99828100
C	-0.29110700	-1.95513600	-0.21571100
C	-1.47606600	-2.78675600	-0.38601300
C	-0.43821200	-1.30537700	0.98221400

C	-2.28499700	-2.62188900	0.76661000
C	-1.94022800	-3.60973700	-1.42385700
H	0.20109000	-0.59830900	1.48312500
C	-3.49890100	-3.28784100	0.93826700
C	-3.16143200	-4.25988000	-1.27493900
H	-1.35822100	-3.73193000	-2.33088400
C	-3.92377200	-4.11418500	-0.10052400
H	-4.06495000	-3.19334400	1.85641000
H	-3.52206100	-4.90858600	-2.06777600
H	-4.85239500	-4.66717300	0.00749100
N	-1.61955600	-1.71596200	1.60769200
S	-2.38206900	-0.79035800	2.83899400
O	-1.33519300	0.10378100	3.32662600
O	-3.07738300	-1.74261900	3.69672500
C	-3.59425400	0.14836300	1.93635800
C	-3.19113600	1.28061800	1.22268900
C	-4.91963900	-0.28226100	1.92303400
C	-4.13656000	1.98260900	0.48259400
H	-2.15395200	1.59600900	1.24836000
C	-5.85202100	0.43176200	1.16962500
H	-5.21547200	-1.14347000	2.50987600
C	-5.47643700	1.56561600	0.43890300
H	-3.81987700	2.84365900	-0.09501700
H	-6.88932400	0.10796500	1.16152800
C	-6.48161300	2.33632200	-0.37865000
H	-6.68634500	3.31445800	0.07311800
H	-6.10560000	2.52464400	-1.39073100
H	-7.43384300	1.80310300	-0.45556800
S	-0.68249500	3.83092300	-0.68707200

O	-1.91709100	3.30625500	-1.28260200
O	0.17543200	4.75707600	-1.42882200
C	-1.09355400	4.58662800	0.89017300
H	-0.17140800	4.83872200	1.41728400
H	-1.65373000	5.49736900	0.66648000
H	-1.69894200	3.89163500	1.47287000
Cu	-1.61537000	0.59713800	-1.42996400
N	-3.26356000	-0.25829400	-1.81881900
C	-4.26764500	-0.83151200	-1.82731100
C	-5.53586100	-1.54118800	-1.82770600
H	-5.63388800	-2.08310200	-0.88391300
H	-6.35914600	-0.82885200	-1.93446300
H	-5.56076700	-2.25836000	-2.65306000

TS_B

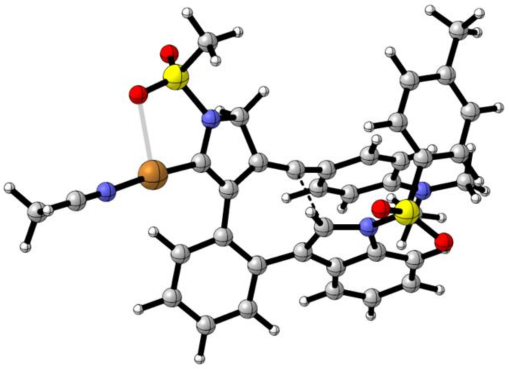

N	-2.91207000	1.38929000	-1.19540100
C	-1.62935400	1.27336100	-1.92089100
H	-1.05237800	2.19905600	-1.88069500
H	-1.80709400	1.00586300	-2.96838400
C	-0.98471100	0.12376700	-1.14695900
C	0.32299400	-0.00144500	-1.11012400
C	1.61965500	-0.07794500	-1.53654900

C	2.00894600	-1.09270100	-2.47342100
C	2.65754800	0.73807300	-0.98837100
C	3.32396700	-1.32976500	-2.76306200
H	1.23361300	-1.71028000	-2.91419800
C	3.97269000	0.52978500	-1.30436500
H	2.38315500	1.54684200	-0.32704100
C	4.35829200	-0.55658900	-2.14813900
H	3.57724600	-2.13578600	-3.43823300
H	4.72128300	1.17673700	-0.86937500
N	5.66311900	-0.84573300	-2.35978500
C	6.70199300	-0.11490400	-1.63942900
H	7.67725800	-0.50772100	-1.92356300
H	6.67903900	0.95151600	-1.88797800
H	6.58119700	-0.22858200	-0.55473800
C	6.04257900	-2.01540000	-3.15047100
H	7.12908300	-2.07982400	-3.19111700
H	5.65321500	-2.93626400	-2.69980800
H	5.66841700	-1.93542900	-4.17621400
C	-3.21800600	0.20582000	-0.50693500
C	-2.08528600	-0.58323600	-0.47445600
C	-2.05732400	-2.00355900	-0.09543900
C	-0.89806700	-2.67849600	0.35940000
C	-3.22015500	-2.77421200	-0.30484800
C	-0.94406100	-4.06800800	0.58517500
C	-3.25957200	-4.13836700	-0.05120200
H	-4.09466400	-2.28314000	-0.71870800
C	-2.10936200	-4.79682400	0.39227500
H	-0.05784200	-4.56337000	0.96722500
H	-4.17678700	-4.69294400	-0.22657400

H	-2.12287800	-5.86440800	0.58833600
C	0.34339600	-1.97729300	0.69607300
C	1.70147700	-2.47195300	0.55155400
C	0.43375100	-0.79562400	1.41173300
C	2.56763500	-1.56534500	1.21399700
C	2.25145700	-3.56464700	-0.13435400
H	-0.35546600	-0.17299100	1.80184100
C	3.94983000	-1.73175900	1.23041700
C	3.63046700	-3.73985300	-0.12478100
H	1.60678400	-4.25432300	-0.66858000
C	4.46715400	-2.83463400	0.55181200
H	4.59457100	-1.04771500	1.76631700
H	4.06809400	-4.58849300	-0.64170100
H	5.54011200	-3.00249100	0.56060500
N	1.75820400	-0.53416500	1.73850100
S	2.18100600	0.57604100	3.00682900
O	3.32031800	-0.02180200	3.68948400
O	0.91372500	0.88167100	3.65971000
C	2.72666800	2.02289900	2.13655400
C	4.08457400	2.19030600	1.86747000
C	1.77793500	2.95766600	1.71540700
C	4.49123500	3.30670700	1.14018700
H	4.80378800	1.46733600	2.23510900
C	2.20534200	4.06365600	0.98726800
H	0.73120100	2.81685700	1.96083700
C	3.56316200	4.25433700	0.68431000
H	5.54768900	3.45322300	0.93263100
H	1.47758900	4.79847600	0.65464600
C	4.01519900	5.47338100	-0.07724700

H	4.15669500	6.32193300	0.60401200
H	4.96795000	5.29932200	-0.58573200
H	3.27517400	5.77599000	-0.82460200
S	-4.11650900	2.41448700	-1.80450100
O	-5.41170000	1.90445300	-1.33180900
O	-3.84848000	2.62329300	-3.22666300
C	-3.78774400	3.94768300	-0.92854200
H	-2.77630800	4.28062400	-1.16893700
H	-4.51857900	4.68182700	-1.27540700
H	-3.90006600	3.76467100	0.14114400
Cu	-5.00258000	-0.04814700	0.16430500
N	-6.71009900	-0.41107800	0.90967500
C	-7.77439300	-0.60059100	1.32222500
C	-9.11410700	-0.83810800	1.83959800
H	-9.85662700	-0.43489300	1.14397700
H	-9.28176100	-1.91286200	1.95999400
H	-9.22964100	-0.34715400	2.81089500

Int-B3

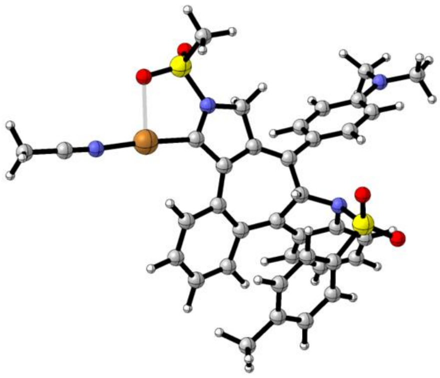

N	2.50639900	2.17113700	-0.33497300
C	1.08116300	2.49551800	-0.13749100
H	0.67549700	3.06964900	-0.97605900

H	0.97646500	3.09950400	0.76824700
C	0.48577800	1.11039100	-0.04710000
C	-0.86237800	0.88681500	-0.19949600
C	-1.86789500	1.92928200	-0.05395600
C	-1.84997900	2.80385500	1.05167000
C	-2.88453200	2.12164300	-1.01557600
C	-2.77861300	3.81756600	1.19846100
H	-1.10894900	2.65393600	1.83206000
C	-3.80857900	3.14152000	-0.89295100
H	-2.92820700	1.47209000	-1.88137300
C	-3.78953000	4.02119200	0.22228700
H	-2.73296000	4.44616300	2.07840200
H	-4.55544200	3.25972800	-1.66738500
N	-4.71292100	5.02260100	0.35334500
C	-5.75976600	5.18793300	-0.64453700
H	-6.39358400	6.02821800	-0.36127800
H	-5.33987900	5.39760900	-1.63656500
H	-6.39123100	4.29316000	-0.71985100
C	-4.68096300	5.89976200	1.51386700
H	-5.47972000	6.63634600	1.42740100
H	-4.82988900	5.34213500	2.44814300
H	-3.72759000	6.43895100	1.58273200
C	2.80615500	0.85909600	-0.15145200
C	1.57398800	0.16593500	0.03888500
C	1.50822200	-1.19197300	0.52815900
C	0.30481900	-1.70484400	1.13309200
C	2.66344600	-2.01272300	0.60380000
C	0.37989300	-2.86793500	1.95627800
C	2.68749100	-3.17406500	1.34479800

H	3.55603600	-1.70707600	0.06914900
C	1.54443100	-3.59044400	2.06197000
H	-0.53169700	-3.24151100	2.40608500
H	3.59468700	-3.77029600	1.37956500
H	1.56932300	-4.50158600	2.65102600
C	-0.96796700	-1.18931100	0.80792100
C	-2.23303800	-1.31032500	1.48595200
C	-1.25585500	-0.55378100	-0.51684200
C	-3.25978700	-1.04266400	0.54628600
C	-2.56695100	-1.59545100	2.81899700
H	-0.67493100	-0.94825700	-1.34847300
C	-4.60340400	-1.08501600	0.89438000
C	-3.91178300	-1.65137000	3.17151600
H	-1.79206400	-1.75154700	3.56183800
C	-4.91475900	-1.40723300	2.21840300
H	-5.37095000	-0.88873800	0.15705600
H	-4.19076200	-1.86897200	4.19743500
H	-5.95670400	-1.45141800	2.51986800
N	-2.70601500	-0.73884500	-0.72409300
S	-3.07508800	-1.81335600	-2.03394100
O	-2.43547200	-1.20665800	-3.19928700
O	-4.51238200	-2.05070200	-1.95148700
C	-2.17901500	-3.27765200	-1.56476700
C	-0.89957700	-3.49031500	-2.08730600
C	-2.68822800	-4.08398200	-0.54455300
C	-0.11947000	-4.51423800	-1.56287100
H	-0.53573100	-2.86130700	-2.89230200
C	-1.88952900	-5.10551200	-0.03506000
H	-3.68696700	-3.91331800	-0.15933900

C	-0.59519000	-5.32768300	-0.52307500
H	0.87710200	-4.68408600	-1.96083200
H	-2.27745400	-5.73904800	0.75744400
C	0.27784000	-6.41457300	0.04764800
H	0.52457900	-7.16340800	-0.71408600
H	1.22296900	-5.99715700	0.41353500
H	-0.21191100	-6.92946800	0.87886800
S	3.68206900	3.42457400	-0.39686300
O	4.98739600	2.78366300	-0.18892500
O	3.20231200	4.49243400	0.47141500
C	3.57651600	3.94707200	-2.11026200
H	2.56262200	4.29815200	-2.31056500
H	4.29055500	4.76426600	-2.23719200
H	3.83848500	3.10086900	-2.74730600
Cu	4.67687300	0.38226900	-0.02479300
N	6.47472900	-0.21668400	0.14233700
C	7.59444300	-0.47856000	0.27059700
C	9.00386000	-0.80311900	0.43275300
H	9.58878000	0.11914400	0.50525800
H	9.14727100	-1.39103200	1.34468500
H	9.35348700	-1.38325000	-0.42691800

TS$_C$

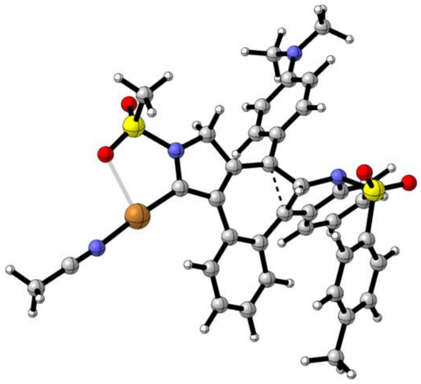

N	-2.75894400	1.87508200	0.60260300
C	-1.34444000	2.28273200	0.56011100
H	-1.02121600	2.72282200	1.51015700
H	-1.19330400	3.02937900	-0.22480700
C	-0.67784000	0.96337100	0.29676800
C	0.72536500	0.81364700	0.29069000
C	1.59211700	1.97634100	0.03453600
C	1.48601700	2.68397600	-1.17482700
C	2.52249300	2.43466000	0.98216000
C	2.26298200	3.79970300	-1.43544400
H	0.79410200	2.33114800	-1.93593800
C	3.30256800	3.55410800	0.74100400
H	2.63655100	1.90302500	1.91996000
C	3.19866600	4.27095200	-0.47795900
H	2.15645900	4.30086000	-2.38890700
H	4.00229300	3.87232200	1.50300700
N	3.97417400	5.37516300	-0.72432600
C	4.96264500	5.80331800	0.25261100
H	5.47602400	6.68881600	-0.12265800
H	4.49413900	6.06409000	1.21039000
H	5.71494000	5.02496300	0.43945100
C	3.87552200	6.06593100	-1.99966200

H	4.55678000	6.91704200	-1.99900400
H	4.14582100	5.41265500	-2.84084400
H	2.86041300	6.44599300	-2.17289300
C	-2.96086400	0.57849800	0.27069000
C	-1.66769200	-0.01268000	0.09423800
C	-1.41099800	-1.31025400	-0.51637600
C	-0.11019500	-1.58258400	-1.02297900
C	-2.43693300	-2.24007500	-0.77535000
C	0.08479900	-2.69611900	-1.86400600
C	-2.21594300	-3.35732900	-1.56446600
H	-3.42111200	-2.07203700	-0.34919700
C	-0.95299500	-3.57457000	-2.13290200
H	1.08086800	-2.90469700	-2.23572800
H	-3.02421800	-4.05951200	-1.74556800
H	-0.77458700	-4.44549800	-2.75566400
C	1.04904700	-0.82395800	-0.58804200
C	2.35889300	-0.75332600	-1.22749100
C	1.28319800	-0.48686800	0.83698800
C	3.33276700	-0.55554100	-0.22730300
C	2.73523100	-0.79404300	-2.57189700
H	0.70226700	-0.96157000	1.61921100
C	4.68039500	-0.40750400	-0.53448200
C	4.08761500	-0.67233200	-2.88894300
H	1.98856800	-0.91289900	-3.35048500
C	5.04513400	-0.48530100	-1.88100000
H	5.41182300	-0.25505300	0.24872000
H	4.40170300	-0.70526500	-3.92732200
H	6.09150100	-0.38345800	-2.15160600
N	2.72656900	-0.50125900	1.06217200

S	3.20836100	-1.71820200	2.21134600
O	2.41235600	-1.43599400	3.40344500
O	4.66652200	-1.69789700	2.20389000
C	2.61198300	-3.21118200	1.44842400
C	1.35902600	-3.70918900	1.81739400
C	3.33145900	-3.76969700	0.39001300
C	0.82116100	-4.77423700	1.10426900
H	0.82885200	-3.26931200	2.65476000
C	2.77359600	-4.83737400	-0.31007000
H	4.30597300	-3.37749400	0.12385000
C	1.51354400	-5.34838200	0.02771600
H	-0.15396300	-5.16466900	1.38154000
H	3.32652600	-5.27988500	-1.13379900
C	0.90999900	-6.49864600	-0.73584300
H	0.93685100	-7.42060000	-0.14210600
H	-0.13927700	-6.29848600	-0.97864100
H	1.44890400	-6.68921400	-1.66841900
S	-4.02318400	3.04844400	0.70330900
O	-5.25648900	2.35264600	0.31908100
O	-3.54451900	4.22750300	-0.00545000
C	-4.08134700	3.37980700	2.46558000
H	-3.11573200	3.77777400	2.78272300
H	-4.86520100	4.12512700	2.62065000
H	-4.32209600	2.45134200	2.98578700
Cu	-4.76685100	-0.03195200	-0.03137600
N	-6.48957200	-0.75601100	-0.37856600
C	-7.56619900	-1.11078700	-0.61030800
C	-8.92135700	-1.55332000	-0.90236700
H	-9.59209300	-0.68914900	-0.93831200

H	-8.94416300	-2.06553700	-1.86932400
H	-9.26364700	-2.24165300	-0.12332100

Int-B4

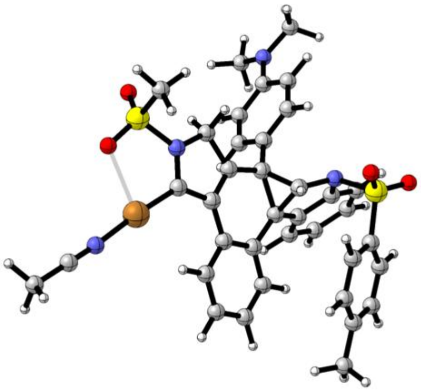

N	-2.86956600	1.62868500	0.69885100
C	-1.47126400	2.09168700	0.71050700
H	-1.15363600	2.40428500	1.71231200
H	-1.34959700	2.94589100	0.03717400
C	-0.76593500	0.86006100	0.24984300
C	0.67046900	0.75372400	0.11463800
C	1.41191500	2.02108800	-0.14281900
C	1.35979000	2.62373600	-1.40648500
C	2.12710300	2.67021700	0.87043700
C	1.99658700	3.82912800	-1.65914500
H	0.82524100	2.12710500	-2.21268900
C	2.76951000	3.87823000	0.63592500
H	2.20198000	2.21150000	1.85112300
C	2.72089300	4.49547000	-0.63808700
H	1.94270300	4.25003600	-2.65494000
H	3.32112700	4.33883500	1.44534000
N	3.35225600	5.69357100	-0.87698800
C	4.17601300	6.29648700	0.15764000

H	4.58786200	7.23484900	-0.21493200
H	3.58659000	6.52169900	1.05536800
H	5.01254200	5.64639200	0.45145500
C	3.36920600	6.24926200	-2.21948100
H	3.90661500	7.19793700	-2.20684900
H	3.86734900	5.58216600	-2.93787300
H	2.35236900	6.44496300	-2.58268900
C	-3.02135000	0.38166600	0.21138100
C	-1.69430700	-0.11900100	-0.05112300
C	-1.32501800	-1.37927000	-0.69917500
C	0.03340500	-1.57389700	-1.04852200
C	-2.26736300	-2.35601700	-1.05092300
C	0.39676700	-2.71763400	-1.76810600
C	-1.89014500	-3.49550700	-1.75362200
H	-3.30703400	-2.22074200	-0.76735400
C	-0.55428200	-3.67212500	-2.12024400
H	1.44054500	-2.87901000	-2.01049800
H	-2.63386500	-4.24260900	-2.01351900
H	-0.24801800	-4.56083600	-2.66329500
C	1.08484000	-0.63996600	-0.57755900
C	2.44535700	-0.56499300	-1.16517900
C	1.31109200	-0.39580900	0.88923700
C	3.37799000	-0.33322000	-0.14026200
C	2.86383800	-0.60660500	-2.49256900
H	0.72692600	-0.87814100	1.66031200
C	4.72776400	-0.12708900	-0.40824400
C	4.22150300	-0.43701000	-2.77301100
H	2.14653000	-0.76505100	-3.29192000
C	5.13888100	-0.19973100	-1.74125300

H	5.42725000	0.06154000	0.39614200
H	4.56611700	-0.47092800	-3.80177100
H	6.18839400	-0.05791900	-1.97984100
N	2.73696500	-0.30262700	1.13759400
S	3.27565800	-1.48051400	2.30468400
O	2.39189300	-1.29211900	3.45388000
O	4.72291100	-1.31958400	2.38045900
C	2.87493000	-3.02083400	1.50690400
C	1.65586200	-3.63904500	1.79727200
C	3.71420200	-3.50917000	0.50327500
C	1.27447000	-4.75579800	1.06207800
H	1.03042000	-3.25152500	2.59362100
C	3.31233100	-4.62944200	-0.22100100
H	4.66009400	-3.02292400	0.29689700
C	2.09019300	-5.26324000	0.04019300
H	0.32647900	-5.24001700	1.27898700
H	3.95942800	-5.01699400	-1.00281800
C	1.66076500	-6.47573500	-0.74527700
H	1.82823200	-7.39460200	-0.16960300
H	0.59260600	-6.43239500	-0.98362700
H	2.22000200	-6.56715100	-1.68108400
S	-4.19032100	2.71746700	0.99295400
O	-5.39404200	2.01460800	0.53878400
O	-3.78189300	4.00175500	0.44342200
C	-4.21064400	2.79427900	2.78518100
H	-3.25877100	3.19901300	3.13367900
H	-5.02796100	3.46540100	3.06050800
H	-4.38739000	1.79039900	3.17461700
Cu	-4.79413000	-0.29188200	-0.13487700

N	-6.47647300	-1.07081500	-0.54923900
C	-7.53058000	-1.46673600	-0.81527300
C	-8.85724600	-1.96149300	-1.15036000
H	-9.58527400	-1.14799300	-1.07162300
H	-8.86079600	-2.34855600	-2.17414400
H	-9.13916600	-2.76426200	-0.46187800

TS_{C2}

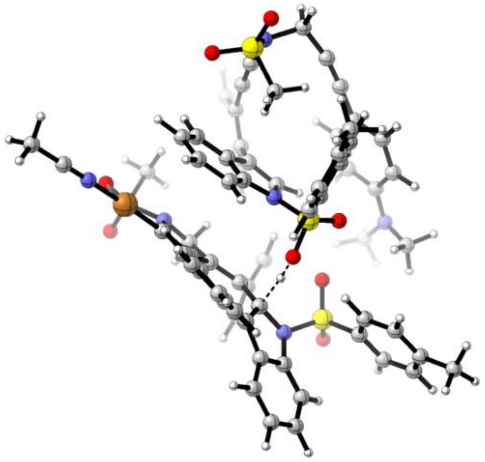

N	1.64031000	-3.12258600	1.13761600
C	0.33011300	-2.73765600	1.67517300
H	0.42166900	-1.87448700	2.33395200
H	-0.12149800	-3.56006800	2.24448700
C	-0.45697100	-2.44131800	0.41411200
C	-1.69536600	-1.88934600	0.52419800
C	-2.28561500	-1.72675200	1.88664500
C	-3.19335800	-2.67571600	2.37803900
C	-1.93418000	-0.68123600	2.74536100
C	-3.72451500	-2.58768100	3.65844800
H	-3.49921900	-3.49863400	1.73799800

C	-2.43994200	-0.57924300	4.03402400
H	-1.27223400	0.09100600	2.37773300
C	-3.35495000	-1.53741100	4.52982600
H	-4.43342100	-3.34039000	3.97859300
H	-2.13856500	0.26201500	4.64619500
N	-3.86279100	-1.44867200	5.81172100
C	-3.61762200	-0.24864100	6.58997800
H	-4.09492900	-0.35049800	7.56558000
H	-2.54369400	-0.10191900	6.76008500
H	-4.01219600	0.65803300	6.10436700
C	-4.94549200	-2.33157200	6.21216300
H	-5.20983600	-2.12404200	7.25009000
H	-5.84507400	-2.20217600	5.59137500
H	-4.63677700	-3.38157100	6.15195100
C	1.59452100	-3.34109800	-0.24731100
C	0.35692600	-2.90860000	-0.70768100
C	-0.00099200	-2.88136800	-2.14264300
C	-1.30723200	-2.66899400	-2.67252200
C	1.02810600	-3.05134000	-3.09019700
C	-1.47862600	-2.54281600	-4.06744800
C	0.83361800	-2.97838000	-4.46400100
H	2.03014300	-3.22184200	-2.71984900
C	-0.43300900	-2.69589300	-4.96987500
H	-2.45898400	-2.28423100	-4.44634600
H	1.67962300	-3.11782700	-5.13178500
H	-0.60921500	-2.60033100	-6.03670300
C	-2.52096000	-2.41172700	-1.88003600
C	-3.87415500	-2.63919100	-2.32219500
C	-2.58361600	-1.71221500	-0.64685300

C	-4.74793500	-2.03067700	-1.38262900
C	-4.43024500	-3.38910400	-3.38015100
H	-1.91793000	-0.52474300	-1.36362500
C	-6.13734900	-2.09305600	-1.50334000
C	-5.80932800	-3.46317300	-3.49752000
H	-3.79094800	-3.93284400	-4.06545200
C	-6.65309600	-2.81095700	-2.57577900
H	-6.78573400	-1.64020800	-0.76407200
H	-6.24834300	-4.04973900	-4.29806000
H	-7.72955000	-2.89979900	-2.68398500
N	-3.97663800	-1.43341800	-0.39093400
S	-4.48625900	0.04344500	0.38432700
O	-3.25079800	0.78016000	0.64735900
O	-5.43360400	-0.29969200	1.43814100
C	-5.36390000	0.90837600	-0.90315200
C	-4.67327100	1.33041800	-2.04153500
C	-6.71143100	1.20832000	-0.71771200
C	-5.36002600	2.04779000	-3.01323500
H	-3.62163700	1.10370800	-2.16530600
C	-7.37927600	1.93776700	-1.70004900
H	-7.21874200	0.87842300	0.18194100
C	-6.72087600	2.36106800	-2.86117800
H	-4.83347800	2.37773900	-3.90460700
H	-8.42886000	2.18066000	-1.56168200
C	-7.45609600	3.11888300	-3.93670300
H	-6.83141900	3.90837200	-4.36711600
H	-7.74292000	2.44792700	-4.75650800
H	-8.37087300	3.57721500	-3.55057300
S	2.70774700	-3.98721200	2.12378300

O	3.90299300	-4.26356300	1.30279200
O	2.01398500	-5.08416400	2.80166800
C	3.15414200	-2.76067800	3.35374200
H	2.26245200	-2.44247700	3.89428100
H	3.85108000	-3.25064000	4.03696900
H	3.62435600	-1.91361600	2.85744500
Cu	3.26391300	-3.99283500	-0.97561900
N	4.84253400	-4.64283400	-1.82207800
C	5.84305700	-5.09697600	-2.18597700
C	7.10303900	-5.67328800	-2.63370000
H	7.52808700	-6.29188200	-1.83706600
H	6.93710900	-6.29513300	-3.51877200
H	7.81110200	-4.87679200	-2.88276600
N	5.59871000	3.42445900	0.73777800
C	5.23764100	4.87743100	0.87675500
H	5.69816100	5.39496100	0.03219300
H	5.73456200	5.22596900	1.78810800
C	3.79282600	5.07114600	0.96614500
C	2.59295200	4.98979100	1.13774500
C	1.20343000	4.82336300	1.39457300
C	0.77891500	4.08231300	2.51436800
C	0.20677600	5.33097200	0.53644700
C	-0.56336400	3.81249800	2.73820100
H	1.52379600	3.68661400	3.19696500
C	-1.13508800	5.06435500	0.74866100
H	0.50027500	5.94558800	-0.31051100
C	-1.56399800	4.26845200	1.84223000
H	-0.83266300	3.21706900	3.60193000
H	-1.86059100	5.45669400	0.04799200

N	-2.88973000	3.94976900	2.01705900
C	-3.27218900	3.02588900	3.07471700
H	-4.36004300	2.94984000	3.10206300
H	-2.87137800	2.01750800	2.91586400
H	-2.94007500	3.39764700	4.05055000
C	-3.82580800	4.16433600	0.92210600
H	-4.80513700	3.78537200	1.21651100
H	-3.93546900	5.23226900	0.69829700
H	-3.51458500	3.63837200	0.01115500
C	4.96974800	2.58731500	1.59011000
C	4.32003000	1.94954700	2.39079400
C	3.48248800	1.28531100	3.33134800
C	2.22765000	0.74499400	2.94405400
C	3.84727600	1.26813400	4.69122000
C	1.36699100	0.26097800	3.93825800
C	2.98282700	0.76742100	5.66002700
H	4.81130300	1.67871500	4.97226700
C	1.73157300	0.27585900	5.28389100
H	0.40469400	-0.14693800	3.65359200
H	3.28128100	0.77002800	6.70379200
H	1.04269700	-0.10762000	6.03078400
C	1.82655000	0.67061900	1.52668800
C	2.57451400	0.05099600	0.43773700
C	0.64837200	1.11836700	1.01259100
C	1.78653600	0.12510600	-0.73632800
C	3.84788900	-0.52499200	0.36426500
H	-0.14407800	1.69767600	1.45553100
C	2.22659000	-0.34813300	-1.96735600
C	4.30533500	-0.99286800	-0.86275200

H	4.48809300	-0.56278800	1.23684600
C	3.50275700	-0.90549500	-2.01214200
H	1.60943100	-0.32391900	-2.85250800
H	5.30575100	-1.40345600	-0.93606400
H	3.87836200	-1.28195300	-2.95827300
N	0.58241500	0.78107400	-0.36247400
S	-0.58494900	1.41806400	-1.36290200
O	-1.35872900	2.38032900	-0.60664600
O	-1.33648200	0.24073200	-2.00610500
C	0.24652000	2.15044800	-2.73114300
C	0.87509300	3.37470800	-2.47848700
C	0.27221400	1.54008800	-3.98623600
C	1.55773900	3.98865800	-3.52029600
H	0.82591200	3.83000500	-1.49408200
C	0.96658100	2.17997200	-5.00992900
H	-0.22729200	0.59209900	-4.15362000
C	1.61985300	3.40258000	-4.79641800
H	2.05413300	4.93851700	-3.34310200
H	1.00284700	1.71953800	-5.99251900
C	2.36761400	4.08894600	-5.90886400
H	1.85747800	5.01335700	-6.20576400
H	3.37824500	4.36706200	-5.59028300
H	2.44953300	3.45143000	-6.79290600
S	5.79743700	2.82346000	-0.87520600
O	6.35944900	1.48347000	-0.74877500
O	6.47610300	3.88673700	-1.61334200
C	4.10758200	2.69945200	-1.47561500
H	3.68033600	3.70130300	-1.48597000
H	4.14130800	2.27021100	-2.47791700

H	3.55043400	2.05067200	-0.80223800

Int-B5

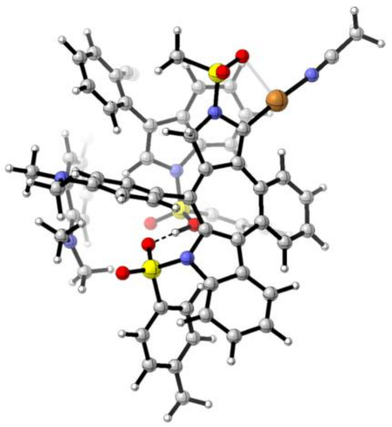

N	1.57836600	1.03906000	-2.99565800
C	0.36542000	0.21409200	-3.01841100
H	0.58500200	-0.79135800	-2.66252800
H	-0.04995800	0.13676000	-4.03143100
C	-0.57347300	0.98524900	-2.11753700
C	-1.82653600	0.49181700	-1.86544900
C	-2.20934900	-0.81569900	-2.48078000
C	-3.06135900	-0.87399500	-3.59399900
C	-1.68849100	-2.02665700	-2.01600100
C	-3.37181300	-2.07585700	-4.21605300
H	-3.49578900	0.04458000	-3.97708700
C	-1.97026100	-3.23943400	-2.63329500
H	-1.06303800	-2.01957400	-1.12992200
C	-2.82218100	-3.29705500	-3.75991600
H	-4.04397800	-2.06235900	-5.06456800
H	-1.53858200	-4.14451400	-2.22449300
N	-3.10157300	-4.49587400	-4.38673700

C	-2.67091100	-5.73652500	-3.77014600
H	-2.98084400	-6.57468800	-4.39561900
H	-1.57754900	-5.77515800	-3.68126200
H	-3.09808300	-5.87915200	-2.76473100
C	-4.12408500	-4.54223500	-5.41847400
H	-4.19922600	-5.56110600	-5.80103600
H	-5.11317400	-4.23823200	-5.04381000
H	-3.86221100	-3.88985800	-6.25958900
C	1.35406600	2.26916700	-2.35245400
C	0.09113900	2.22514700	-1.77135100
C	-0.42206100	3.29164600	-0.87354800
C	-1.79302500	3.51122500	-0.54190400
C	0.52206600	4.16352400	-0.30342000
C	-2.10601800	4.56802700	0.33580700
C	0.18361500	5.24645200	0.50604600
H	1.56672900	3.98095600	-0.51760700
C	-1.14958600	5.45240500	0.83555700
H	-3.13219000	4.69132500	0.65282600
H	0.96377700	5.89967600	0.88730000
H	-1.45368700	6.27283000	1.47941600
C	-2.92342100	2.64110300	-0.92984500
C	-4.34523400	2.99143300	-0.84558800
C	-2.85800400	1.30081800	-1.27206600
C	-5.09692400	1.80422900	-0.99860000
C	-5.05472100	4.20107300	-0.76391000
H	-2.07624700	0.09851200	0.82264600
C	-6.48166400	1.76364300	-1.01078000
C	-6.45006700	4.17727800	-0.76653700
H	-4.53644400	5.15203600	-0.73838000

C	-7.16211400	2.97588200	-0.87233000
H	-7.01422500	0.82854700	-1.14592100
H	-6.99354200	5.11537000	-0.70634600
H	-8.24709700	2.98664800	-0.88427300
N	-4.18612600	0.72628800	-1.18244200
S	-4.35165900	-0.63358100	-0.12225400
O	-2.98445400	-0.91332900	0.43776800
O	-5.03295800	-1.73065700	-0.79006500
C	-5.32735700	-0.06176500	1.24611800
C	-4.83283900	0.96410700	2.05825200
C	-6.56368600	-0.65467100	1.48227600
C	-5.60870200	1.39707000	3.12363700
H	-3.87619600	1.43012200	1.84923100
C	-7.32048600	-0.21012500	2.56616100
H	-6.92094700	-1.44291000	0.82920300
C	-6.86138000	0.81872700	3.39611400
H	-5.24345700	2.20068200	3.75697100
H	-8.28576700	-0.66657700	2.76372400
C	-7.68344000	1.30519900	4.56142600
H	-7.16757400	1.11746900	5.51078600
H	-7.85654900	2.38548500	4.49589300
H	-8.65580100	0.80749500	4.60271900
S	2.64217700	0.95859100	-4.31046200
O	3.78348100	1.82889100	-3.96894700
O	1.93370700	1.15134500	-5.57839400
C	3.18478000	-0.75019600	-4.23505500
H	2.32898900	-1.41075700	-4.37752900
H	3.89555200	-0.87876600	-5.05403500
H	3.65823900	-0.93900300	-3.27391200

Cu	2.86025200	3.47292300	-2.40934400
N	4.26297900	4.76017500	-2.46167200
C	5.15199400	5.47816500	-2.64547800
C	6.27102100	6.37755300	-2.88836900
H	6.73620500	6.13438700	-3.84880800
H	5.91934700	7.41354100	-2.91422500
H	7.01648800	6.27181500	-2.09412000
N	5.83330600	-2.54384400	2.29989200
C	5.58243200	-3.72441100	3.20235700
H	5.97483100	-3.45330800	4.18533300
H	6.20183600	-4.53666200	2.80939200
C	4.16890000	-4.09273400	3.22433900
C	2.97794500	-4.21426300	3.01571500
C	1.57991900	-4.30308800	2.76775800
C	1.08078200	-4.70740200	1.51466300
C	0.64343600	-3.90727000	3.74383100
C	-0.27978600	-4.67372800	1.23278500
H	1.77762100	-5.01301500	0.74079600
C	-0.71315300	-3.88361100	3.47783200
H	0.99987500	-3.60501200	4.72408000
C	-1.22099100	-4.24335700	2.20382900
H	-0.61054800	-4.96716500	0.24392700
H	-1.38876200	-3.55401900	4.25605000
N	-2.56901700	-4.16928800	1.93367200
C	-3.05828400	-4.44905400	0.59266200
H	-4.14896900	-4.46553500	0.60775000
H	-2.74598000	-3.69497200	-0.13844600
H	-2.71569300	-5.43381500	0.25625600
C	-3.43470600	-3.41507200	2.82918500

H	-4.43915000	-3.38497900	2.40472900
H	-3.50281500	-3.89127600	3.81475400
H	-3.08605300	-2.38239300	2.96053200
C	5.27743700	-2.65289300	1.07473000
C	4.69516800	-2.88227300	0.03670700
C	3.83706400	-3.21942300	-1.04620200
C	2.53480300	-2.66023100	-1.13124100
C	4.21866500	-4.21988600	-1.95926800
C	1.64493500	-3.17449500	-2.08046500
C	3.32323600	-4.69819000	-2.91188400
H	5.21885800	-4.63354300	-1.88612200
C	2.02548300	-4.18682200	-2.96066500
H	0.64223900	-2.77462200	-2.14001100
H	3.63439100	-5.47574900	-3.60269700
H	1.30950000	-4.56159700	-3.68635300
C	2.13123200	-1.53718000	-0.26016700
C	2.81050300	-0.24973200	-0.16212900
C	1.05242000	-1.49874400	0.56667000
C	2.08531300	0.57862100	0.72597500
C	3.99364200	0.22817100	-0.73483500
H	0.34252700	-2.26578300	0.83582500
C	2.50967500	1.86424200	1.05322300
C	4.42056200	1.51269500	-0.42790800
H	4.58732400	-0.40934500	-1.37814500
C	3.68338800	2.31830400	0.45321400
H	1.96447200	2.50783900	1.72489800
H	5.34295800	1.88771900	-0.85669100
H	4.03828500	3.31613400	0.69110900
N	1.00846300	-0.22822600	1.21454400

S	-0.31423300	0.18123800	2.12569100
O	-0.76844700	-1.00630700	2.82977700
O	-1.39663300	0.82954300	1.21915200
C	0.18695700	1.49742800	3.15716800
C	1.03470400	1.16604000	4.22195400
C	-0.23010700	2.80469000	2.90310300
C	1.48831700	2.18987000	5.04077500
H	1.32209200	0.13568900	4.40148100
C	0.23932700	3.80793100	3.74654900
H	-0.87237600	3.03952100	2.06357800
C	1.10198700	3.52408400	4.81453600
H	2.14991500	1.95645100	5.86969300
H	-0.06436500	4.83104900	3.55281500
C	1.59583200	4.61889100	5.72212900
H	1.07305000	4.58362900	6.68616600
H	2.66505000	4.50865200	5.93066300
H	1.42736600	5.60759200	5.28744900
S	5.66042500	-0.97485100	3.02010100
O	6.07773600	-0.01455800	2.00485100
O	6.30466200	-1.06267300	4.32794300
C	3.88153000	-0.80632800	3.25972400
H	3.57473200	-1.46750500	4.06996400
H	3.69786200	0.24371700	3.49331800
H	3.38465100	-1.08470800	2.33185700

TS$_{B2}$

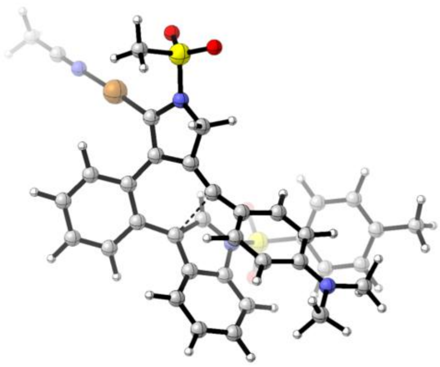

N	-2.98826900	-1.79760300	-0.92153400
C	-1.70591800	-1.64901600	-1.64841800
H	-1.87693700	-1.53221800	-2.72721700
H	-1.05165800	-2.50227600	-1.47687000
C	-1.20272400	-0.35189400	-1.03183700
C	0.09240900	-0.09232200	-0.97059600
C	1.38085600	-0.28126000	-1.45084600
C	2.31219100	-1.09664900	-0.74749400
C	1.86615900	0.43298300	-2.58862000
C	3.62492100	-1.19754800	-1.13324000
H	1.95703300	-1.66842000	0.09990100
C	3.18451500	0.36585100	-2.96720600
H	1.17768800	1.06430200	-3.14141800
C	4.12028800	-0.43486100	-2.23568800
H	4.28478200	-1.84235800	-0.56884200
H	3.51253800	0.94255700	-3.82257700
N	5.43283300	-0.46894500	-2.57146200
C	5.94041600	0.35549200	-3.66673800
H	7.00796400	0.17246400	-3.78708000
H	5.44273800	0.10717700	-4.61098700
H	5.78998100	1.42297300	-3.46047800

C	6.37683700	-1.25200100	-1.77476800
H	7.37803900	-1.13220100	-2.18764300
H	6.38791800	-0.91530600	-0.73088700
H	6.12120600	-2.31802600	-1.79459200
C	-3.42939100	-0.57607800	-0.39198300
C	-2.38157000	0.33042200	-0.48920700
C	-2.46641700	1.78653400	-0.31881000
C	-1.32084200	2.61061100	-0.14865000
C	-3.71155500	2.43834900	-0.48248800
C	-1.46133100	4.01512000	-0.15160300
C	-3.83817700	3.82021500	-0.44726800
H	-4.59282700	1.83721000	-0.68593100
C	-2.70219800	4.62225400	-0.28649100
H	-0.58016100	4.62908400	0.00329200
H	-4.81787300	4.27358800	-0.57408400
H	-2.78464300	5.70506800	-0.26486000
C	0.01827700	2.07538500	0.12375500
C	1.29419400	2.65701500	-0.25921800
C	0.31504900	1.06568900	1.04653300
C	2.30705500	1.99532900	0.47373200
C	1.65569800	3.60135100	-1.23182400
H	-0.36735400	0.50283700	1.66418900
C	3.66038800	2.25618000	0.27756800
C	3.00492500	3.87841800	-1.42775100
H	0.89369600	4.09204400	-1.82850600
C	3.99374100	3.21242500	-0.68117800
H	4.42818400	1.73023400	0.82856900
H	3.30114100	4.60637000	-2.17709700
H	5.04130600	3.43897400	-0.85759600

N	1.67951500	1.03429300	1.29941400
S	2.30098200	0.48679500	2.84160800
O	1.13410900	-0.09299600	3.50721400
O	3.02376000	1.62963100	3.39714100
C	3.46718300	-0.80387200	2.47492700
C	3.00724900	-2.11764700	2.34922800
C	4.82493000	-0.49212400	2.36268600
C	3.92543200	-3.12395200	2.06029700
H	1.95413500	-2.34829000	2.47097700
C	5.72581400	-1.51447300	2.07636600
H	5.17522500	0.52297300	2.51170100
C	5.29288900	-2.84013600	1.91261500
H	3.57501800	-4.14645500	1.95135400
H	6.78309500	-1.28092300	1.98619500
C	6.28225900	-3.93619100	1.61355800
H	6.73876400	-4.30510400	2.54172700
H	5.80119300	-4.78747200	1.12166300
H	7.09451900	-3.57543000	0.97331400
S	-4.05473400	-3.02635000	-1.38648200
O	-4.91273700	-3.34778800	-0.23977400
O	-3.21292000	-4.06337500	-1.99422900
C	-5.07627100	-2.27010900	-2.65818500
H	-4.43158700	-1.93892800	-3.47620100
H	-5.78335000	-3.02631800	-3.00978900
H	-5.60404500	-1.42413500	-2.21094100
Cu	-5.14516600	-0.33044300	0.46638800
N	-6.83399900	-0.00549500	1.30243900
C	-7.85231300	0.16808100	1.82665000
C	-9.12844900	0.38481900	2.48454600

H	-9.35565000	1.45575400	2.50343600
H	-9.91749200	-0.14424500	1.94016500
H	-9.08010900	0.00635400	3.51087900

Int-B3'

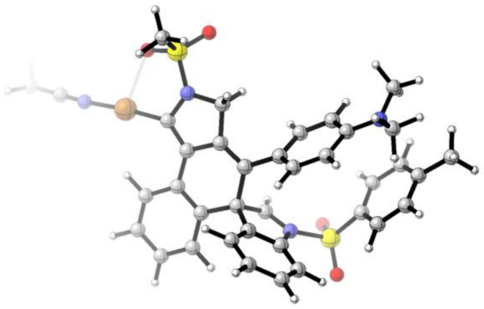

N	-3.16042000	1.71886300	0.29186800
C	-1.68871400	1.78599000	0.36524600
H	-1.34727600	2.26027100	1.29267600
H	-1.30360600	2.37259600	-0.47128100
C	-1.32522600	0.32026900	0.29628600
C	-0.07412100	-0.20271500	0.44307000
C	1.13031200	0.65768900	0.56034400
C	1.39070600	1.62229400	-0.42910300
C	2.03000900	0.60021000	1.63738700
C	2.46033800	2.50002100	-0.34513400
H	0.73555900	1.68261200	-1.29401300
C	3.12509800	1.45234900	1.72211000
H	1.86386000	-0.10399500	2.44396100
C	3.36691600	2.43509800	0.73720800
H	2.59685100	3.23023900	-1.13223800
H	3.77435200	1.37062800	2.58445700
N	4.44843800	3.29657900	0.81632500

C	5.26845100	3.28384100	2.01554800
H	6.08793400	3.99395000	1.89501400
H	4.70617200	3.55470000	2.92215900
H	5.70513700	2.29022500	2.16794800
C	4.43370000	4.51056800	0.01406000
H	5.31754400	5.10466100	0.24953700
H	4.46748100	4.27538400	-1.05576400
H	3.54097900	5.12518300	0.20006600
C	-3.64671100	0.42365600	0.10919100
C	-2.54692800	-0.41986200	0.13720400
C	-2.49684100	-1.87493800	0.09629700
C	-1.24883500	-2.51791600	0.21974000
C	-3.64419800	-2.67718000	-0.03074200
C	-1.17160000	-3.91503500	0.18348600
C	-3.55968300	-4.06349100	-0.06520200
H	-4.61323800	-2.19298100	-0.10127300
C	-2.31614600	-4.69306800	0.03562600
H	-0.20318400	-4.39876200	0.27873800
H	-4.46368300	-4.65594000	-0.17183300
H	-2.23861800	-5.77500200	0.00066900
C	0.06497500	-1.74533200	0.34638900
C	1.02704500	-2.31851200	1.37246600
C	0.90926200	-1.81633100	-0.89785800
C	2.27239400	-2.52892800	0.77402000
C	0.84230000	-2.62547700	2.71139400
H	0.61461200	-1.53020900	-1.89832200
C	3.36046800	-3.05698000	1.45513800
C	1.93160000	-3.12963000	3.43203600
H	-0.12535800	-2.47919200	3.18035300

C	3.16763200	-3.34448500	2.81068300
H	4.29452500	-3.28392500	0.95866900
H	1.81308800	-3.37263700	4.48310400
H	3.98978300	-3.76243300	3.38234500
N	2.14968900	-2.13926400	-0.60770500
S	3.49279500	-2.09732800	-1.82816700
O	2.76010000	-1.90392100	-3.07106500
O	4.24440900	-3.29980800	-1.50552500
C	4.43156700	-0.65856300	-1.44117300
C	4.32270700	0.45079300	-2.28504800
C	5.37094800	-0.69897000	-0.40335400
C	5.19961300	1.51436900	-2.10288400
H	3.59335000	0.45737900	-3.08613900
C	6.21939100	0.38516000	-0.23030600
H	5.45840500	-1.56916000	0.23400500
C	6.16609500	1.49625000	-1.08787900
H	5.14356100	2.36644400	-2.77357900
H	6.96050100	0.35747700	0.56299400
C	7.17750900	2.60306100	-0.96225700
H	6.80268900	3.54175900	-1.37760700
H	7.45993900	2.77066800	0.08077400
H	8.09219300	2.34352500	-1.51051300
S	-4.09388800	3.11031000	0.14954000
O	-5.41127400	2.69279700	-0.35529900
O	-3.28786900	4.11137000	-0.54540100
C	-4.30991300	3.63255700	1.85577500
H	-3.32825800	3.82888000	2.29130900
H	-4.90640200	4.54763800	1.84391400
H	-4.82930800	2.83895000	2.39557500

Cu	-5.52434500	0.22744800	-0.25737600
N	-7.35088700	-0.10360900	-0.65898000
C	-8.46743000	-0.21405400	-0.94199600
C	-9.87199000	-0.34879200	-1.29976400
H	-9.98013300	-1.06603400	-2.11929000
H	-10.44408000	-0.70262400	-0.43647200
H	-10.26673900	0.62103900	-1.61828000

TS_{C3}

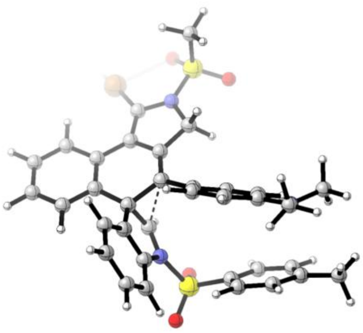

N	-2.86060900	-1.72718200	-0.34608000
C	-1.39991000	-1.68411700	-0.52876800
H	-1.09181300	-2.14513800	-1.47488800
H	-0.91176700	-2.22905300	0.28091900
C	-1.14550000	-0.19784800	-0.49786400
C	0.07308000	0.42681200	-0.68026000
C	1.30996300	-0.33852200	-0.95665600
C	1.69453400	-1.38409600	-0.09763100
C	2.10350200	-0.13408700	-2.09534200
C	2.77018900	-2.20982600	-0.37538300
H	1.14160100	-1.54755700	0.82301700
C	3.21223300	-0.92870300	-2.36635500
H	1.84400000	0.64748900	-2.79953000

C	3.57358400	-1.99766200	-1.51764700
H	3.01060300	-2.99673000	0.32592500
H	3.77569900	-0.73018700	-3.26898300
N	4.68384100	-2.79738600	-1.76965700
C	5.41405300	-2.59111500	-3.01206100
H	6.30163800	-3.22685900	-3.00974600
H	4.81916100	-2.82885100	-3.90852300
H	5.74907200	-1.55231700	-3.08647500
C	4.66770000	-4.16893600	-1.27055600
H	5.60944800	-4.65264500	-1.53692600
H	4.58804300	-4.19073300	-0.18196800
H	3.83928300	-4.75951000	-1.69077500
C	-3.43585200	-0.47839500	-0.20772600
C	-2.40111000	0.45493400	-0.32936100
C	-2.49367600	1.90762600	-0.45702900
C	-1.30534600	2.65175300	-0.61697100
C	-3.72070100	2.58686800	-0.50433500
C	-1.37019500	4.03748900	-0.78813200
C	-3.77597400	3.96597900	-0.67489500
H	-4.64066600	2.01726500	-0.41722200
C	-2.59690300	4.69899700	-0.81504700
H	-0.45353600	4.60843000	-0.88765000
H	-4.73847400	4.46827800	-0.70076300
H	-2.62788900	5.77692600	-0.93783600
C	0.05285600	1.98310200	-0.49590800
C	1.23152500	2.67911500	-1.13206500
C	0.55036600	1.74766800	0.89174800
C	2.30338000	2.67839800	-0.23210200
C	1.38018300	3.25071800	-2.38820000

H	-0.01184900	1.39449700	1.74235000
C	3.53256100	3.25333600	-0.53267900
C	2.61718400	3.81028600	-2.72522100
H	0.54973300	3.26371900	-3.08692600
C	3.67252200	3.81357200	-1.80624500
H	4.32892900	3.31192000	0.19775800
H	2.75536800	4.25990800	-3.70313800
H	4.61587900	4.27822200	-2.07442500
N	1.85720000	2.03558900	0.97036600
S	2.83087200	1.70969900	2.41713100
O	1.82547800	1.37338100	3.41730100
O	3.69295000	2.87899200	2.50784200
C	3.79765000	0.27486700	2.03971800
C	3.63901500	-0.84766100	2.85473000
C	4.78207500	0.32709700	1.04851200
C	4.50429900	-1.92501100	2.68093700
H	2.87156000	-0.86069400	3.61983600
C	5.61972300	-0.76726900	0.88294100
H	4.89245200	1.19673700	0.41416700
C	5.51289900	-1.89659000	1.70856100
H	4.40272700	-2.79547600	3.32277600
H	6.37643900	-0.74516100	0.10540600
C	6.49080100	-3.03360000	1.56967600
H	6.06059300	-3.98178100	1.90589000
H	6.81943700	-3.14624800	0.53272100
H	7.38335700	-2.84643700	2.18015400
S	-3.69301400	-3.18945600	-0.16860000
O	-4.99757300	-2.86007100	0.42158100
O	-2.77114000	-4.12725200	0.46456100

C	-3.97357000	-3.70337800	-1.86753700
H	-3.00868200	-3.83402700	-2.36116600
H	-4.51110300	-4.65383600	-1.83373700
H	-4.57224500	-2.93806700	-2.36450900
Cu	-5.30150900	-0.38855200	0.25098900
N	-7.12081300	-0.16055200	0.74551100
C	-8.22258200	-0.09900900	1.09353700
C	-9.60801600	-0.02485200	1.53332800
H	-9.70102900	0.70112200	2.34704100
H	-10.24678300	0.28610400	0.70081800
H	-9.93544700	-1.00652900	1.88966600

2a

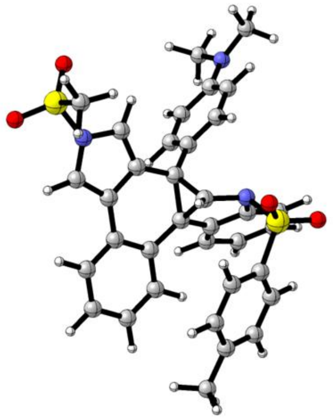

N	-2.83357400	-3.00779400	-0.01071600
C	-2.65478200	-1.65690200	0.29160600
H	-3.45357100	-1.07462000	0.72225200
C	-1.39187200	-1.31768800	-0.11496600
C	-0.72620400	0.00451800	-0.04459300
C	-1.61705900	1.19729700	-0.17888200
C	-2.09963300	1.59025800	-1.43102300

C	-2.03861600	1.91626000	0.94187900
C	-2.96363700	2.66775200	-1.56883600
H	-1.79107400	1.03932700	-2.31540600
C	-2.90685700	2.99699300	0.82299100
H	-1.67132500	1.63545800	1.92380900
C	-3.38180000	3.41112200	-0.44040600
H	-3.31210600	2.93193700	-2.55937500
H	-3.20776300	3.52091300	1.72145300
N	-4.21919400	4.51197400	-0.57315200
C	-4.81049100	5.09202600	0.61859300
H	-5.42742700	5.94564900	0.33130500
H	-5.44098300	4.37908800	1.17444400
H	-4.03433500	5.45938100	1.29965300
C	-4.88196900	4.74308800	-1.84353300
H	-5.49443100	5.64356000	-1.76718000
H	-4.14960800	4.90898000	-2.64204700
H	-5.53173300	3.90602200	-2.14636300
C	-1.67636800	-3.52490700	-0.58450700
C	-0.77199100	-2.48999200	-0.68521900
C	0.53908400	-2.44763500	-1.32770100
C	1.23834800	-1.21820500	-1.36479500
C	1.10876400	-3.57703300	-1.93445700
C	2.48071300	-1.16185500	-2.00745900
C	2.34268900	-3.50512500	-2.57257100
H	0.57040400	-4.52030200	-1.91047200
C	3.03064100	-2.29022300	-2.61142700
H	3.03014700	-0.22756200	-2.01017200
H	2.76366400	-4.39105800	-3.03927600
H	3.99420400	-2.22017500	-3.10776100

C	0.70231000	-0.01149600	-0.67825900
C	1.18484000	1.35947600	-0.99904000
C	0.49379400	0.04238600	0.83867300
C	1.28183300	2.12140600	0.17635400
C	1.44686300	1.95441500	-2.23000500
H	0.69162400	-0.80287200	1.48279300
C	1.61338700	3.47259000	0.15294800
C	1.81542100	3.30191600	-2.26692800
H	1.36120100	1.37639000	-3.14503600
C	1.89497500	4.05083600	-1.08732400
H	1.66799400	4.04002900	1.07318500
H	2.02221700	3.77643100	-3.22169300
H	2.16866000	5.10076000	-1.13365600
N	0.96427500	1.33483900	1.33155300
S	2.23264200	1.17316200	2.49638500
O	1.71428200	0.25348900	3.50965700
O	2.65897400	2.53271500	2.82241400
C	3.52746500	0.35098200	1.58810900
C	3.61920100	-1.04204800	1.64486600
C	4.34259100	1.09363200	0.73255900
C	4.53931500	-1.69269300	0.83142700
H	2.98031500	-1.59578600	2.32325300
C	5.26065600	0.42191200	-0.07288100
H	4.25805900	2.17329300	0.70099300
C	5.36888500	-0.97377400	-0.04108200
H	4.60803100	-2.77654500	0.86256700
H	5.89905000	0.99245000	-0.74194300
C	6.35166800	-1.70091500	-0.92300900
H	7.16107000	-2.14597900	-0.33104100

H	5.85809600	-2.51598300	-1.46388200
H	6.80596700	-1.02881200	-1.65711500
S	-4.11117900	-3.96436800	0.59779800
O	-4.11152800	-5.19130500	-0.19178800
O	-5.27262900	-3.08819300	0.69396900
C	-3.53273500	-4.34320600	2.26012200
H	-3.40340100	-3.40592400	2.80362400
H	-4.29743200	-4.96269600	2.73419200
H	-2.58927700	-4.88585400	2.18254000
H	-1.64956400	-4.55400000	-0.90573300

[CuSaBOX2]-(major)-TS$_B$

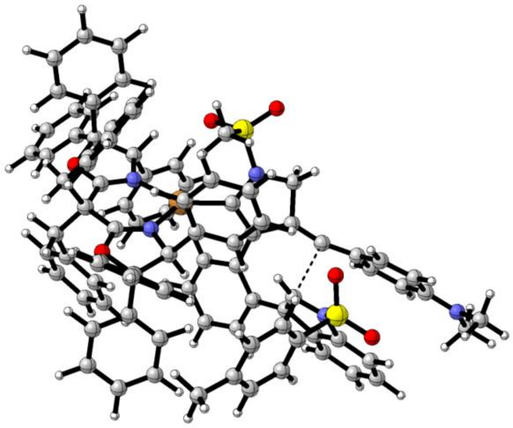

C	3.71057900	-2.46880300	-1.21595900
C	3.85490000	-1.27663300	1.26643200
C	3.96570700	-0.36851600	0.23214000
Cu	-1.32631100	-0.69043000	-0.44946500
N	-3.29946000	-0.89505700	-0.12418900
N	-1.56259600	1.27741200	0.12170800
N	0.41658600	-2.07504100	-2.36452900
C	1.66234600	-2.84948700	-2.53077600

H	2.18314400	-2.58304700	-3.44969600
H	1.45548700	-3.92604500	-2.53307700
C	2.39837100	-2.43434900	-1.25233700
C	5.02967100	-2.78119600	-1.38476400
C	5.91211400	-1.91505400	-2.11006600
C	5.61584300	-3.90630300	-0.71787800
C	7.26314800	-2.13478600	-2.14252300
H	5.48678300	-1.07434300	-2.64456400
C	6.97025900	-4.10079300	-0.70512900
H	4.96337300	-4.58000400	-0.17277300
C	7.84568400	-3.20027000	-1.39052200
H	7.89011500	-1.45223000	-2.69995500
H	7.37713800	-4.93530000	-0.15050500
N	9.18992600	-3.35212000	-1.32488400
C	9.77455400	-4.37766900	-0.46515400
H	9.48474600	-5.38243400	-0.79153200
H	10.86042600	-4.30557600	-0.51284400
H	9.45990900	-4.23917000	0.57641100
C	10.07337800	-2.39519800	-1.98735600
H	11.10789700	-2.69499000	-1.82480200
H	9.89029900	-2.37366800	-3.06678800
H	9.93494000	-1.38303800	-1.58777100
C	0.20851800	-1.65872700	-1.05710600
C	1.36874000	-1.92371700	-0.33595600
C	1.42775100	-1.92922500	1.12782700
C	2.60997000	-1.72331800	1.88413300
C	0.25208300	-2.26803200	1.83601000
C	2.57920100	-1.86391700	3.28614100
C	0.23475700	-2.38550300	3.21779200

H	-0.64673600	-2.49583400	1.27448700
C	1.40706100	-2.18592000	3.95495700
H	3.48481900	-1.66814800	3.85089300
H	-0.69061900	-2.66014300	3.70953700
H	1.40554800	-2.28241300	5.03682000
C	5.21333900	-1.58953200	1.68369100
C	6.10459600	-0.80409500	0.90847700
C	5.73897800	-2.53000800	2.58139600
H	3.18883700	0.12655600	-0.32580500
C	7.48924300	-0.90443700	1.02914900
C	7.11937400	-2.63969400	2.71053800
H	5.07479300	-3.16982300	3.15208000
C	7.98020500	-1.82972500	1.95022900
H	8.14806900	-0.29307800	0.42863800
H	7.53796800	-3.36124300	3.40540000
H	9.05471500	-1.92427800	2.07727700
N	5.30660400	-0.05347200	0.01632100
S	5.75696400	1.30881100	-0.91273200
O	5.00520300	1.22471200	-2.16602400
O	7.21666300	1.32799100	-0.91655300
C	5.06913300	2.64726200	0.03831200
C	5.13650700	2.63044200	1.43248200
C	4.47578500	3.70765600	-0.64971300
C	4.57519200	3.68898700	2.14284900
H	5.60109200	1.80105200	1.95577600
C	3.93784200	4.76167900	0.08126700
H	4.41981200	3.69463300	-1.73158500
C	3.96252300	4.76555800	1.48436000
H	4.61417500	3.68085300	3.22793500

H	3.48284500	5.59382300	-0.44673400
C	3.32820100	5.89658500	2.25066400
H	2.27087200	6.00011400	1.98219300
H	3.38654200	5.73445300	3.32967900
H	3.81948400	6.84899700	2.01952200
S	-0.79239100	-2.11102400	-3.54051100
O	-2.04755800	-2.62906500	-2.97660100
O	-0.17924600	-2.77197300	-4.69401800
C	-1.02998400	-0.36779500	-3.88553600
H	-1.35297700	0.13723500	-2.97481900
H	-0.08641200	0.05078000	-4.23500400
H	-1.80136400	-0.29456700	-4.65445600
C	-4.01041700	1.37213700	0.71718700
C	-4.49929300	1.56265700	2.19527100
H	-5.58622000	1.44727100	2.18265900
H	-4.28963400	2.60353700	2.46013400
C	-4.95355200	2.25803900	-0.17968100
H	-5.93306200	1.77067000	-0.16612500
H	-5.06175200	3.21802700	0.33222100
C	-4.51292000	2.50384700	-1.60401000
C	-4.24350700	3.81015200	-2.03026600
C	-4.42027400	1.46274400	-2.53792200
C	-3.89180500	4.07694000	-3.35484500
C	-4.07061500	1.72546500	-3.86094900
C	-3.80556800	3.03252600	-4.27610600
H	-3.68693800	5.09822900	-3.66190400
H	-4.03430600	0.90480600	-4.57121600
C	-3.91150300	0.62664100	3.22529700
C	-4.75862400	-0.21636000	3.95497200

C	-2.53910400	0.60434600	3.50922900
C	-4.25334800	-1.06520400	4.94113400
C	-2.03049200	-0.23982600	4.49407900
C	-2.88468500	-1.07983500	5.21157600
H	-4.92876200	-1.71112800	5.49432400
H	-0.96490300	-0.23668600	4.69746800
C	-2.59433200	1.88008500	0.57043800
C	-0.42245900	2.20638000	0.11529200
C	-0.98569500	3.46744900	0.88599200
C	-4.17196500	-0.08121900	0.33136900
C	-3.93666700	-2.20773800	-0.33817000
C	-5.46161200	-1.85884100	-0.25315800
O	-5.42427400	-0.56176900	0.47190600
O	-2.42393500	3.16371400	0.95517100
H	-3.66549000	-2.52899400	-1.33791400
H	0.36631200	1.74157200	0.70654700
H	-1.85832700	1.26147700	2.98314800
H	-5.82629900	-0.19953800	3.75551800
H	-4.31765400	4.62881200	-1.32155300
H	-4.64875000	0.44596100	-2.24896000
H	-2.48715000	-1.73423800	5.98218000
H	-3.54657000	3.23623200	-5.31120000
C	-6.30327900	-2.84011000	0.52866500
C	-6.89814600	-2.50648900	1.74557700
C	-6.46869900	-4.13006600	0.01115900
C	-7.64222000	-3.45788500	2.44651200
H	-6.77224300	-1.50843100	2.14527900
C	-7.20472400	-5.07936500	0.71419000
H	-6.01801300	-4.39075000	-0.94211600

C	-7.79413800	-4.74605100	1.93659400
H	-8.10227700	-3.18818400	3.39292900
H	-7.32082000	-6.07929100	0.30705800
H	-8.37051600	-5.48614700	2.48354000
C	-6.08573400	-1.54253300	-1.61397400
C	-7.25341900	-0.76405500	-1.62075200
C	-5.55164100	-1.96813800	-2.83447200
C	-7.85021400	-0.38501700	-2.81853200
H	-7.68500900	-0.44872900	-0.67636200
C	-6.15985300	-1.59538700	-4.03771300
H	-4.65394800	-2.57237000	-2.88384000
C	-7.30032600	-0.79642000	-4.03588800
H	-8.74628900	0.22827100	-2.80405100
H	-5.72938600	-1.93312200	-4.97596600
H	-7.76348300	-0.50060000	-4.97237000
C	-3.37769400	-3.22131900	0.63191300
C	-2.70024300	-4.33553000	0.12880100
C	-3.48008600	-3.04487600	2.01587400
C	-2.14716400	-5.27684800	0.99920500
H	-2.59075200	-4.45132600	-0.94585900
C	-2.94031200	-3.99051200	2.88373300
H	-3.98051200	-2.17358300	2.41975700
C	-2.27187900	-5.10895100	2.37833400
H	-1.62054200	-6.13841700	0.59908800
H	-3.04021000	-3.84760600	3.95550000
H	-1.84595300	-5.84191700	3.05737600
C	0.09198000	2.39361500	-1.29194100
C	-0.74627300	2.84679800	-2.31509200
C	1.42224500	2.08753400	-1.58532500

C	-0.25113900	3.00501200	-3.60724300
H	-1.78240300	3.08018200	-2.10519900
C	1.92102300	2.22798000	-2.88185800
H	2.07631500	1.74888900	-0.78800400
C	1.08115400	2.69402100	-3.89504600
H	-0.91289300	3.36496400	-4.38843500
H	2.95391500	1.96874100	-3.08835100
H	1.46258500	2.81348700	-4.90521500
C	-0.45923200	3.53259700	2.31935800
C	-1.30524300	3.94689400	3.35475500
C	0.87500400	3.23119900	2.61580600
C	-0.83807800	4.02004800	4.66571700
H	-2.33628400	4.19730500	3.13342700
C	1.34293600	3.30838100	3.92796800
H	1.56706800	2.93815700	1.83309200
C	0.48752000	3.69544800	4.95895800
H	-1.51263000	4.32756500	5.45902100
H	2.37678200	3.05360100	4.13963700
H	0.84940200	3.74528400	5.98144000
C	-0.82230100	4.81331200	0.20422300
C	0.44963600	5.23286500	-0.20158200
C	-1.91129800	5.66775000	0.02126400
C	0.62198000	6.47502000	-0.80602700
H	1.30331500	4.57894700	-0.06833900
C	-1.73704300	6.91418600	-0.58370600
H	-2.89442500	5.35816000	0.35155000
C	-0.47184100	7.32099700	-1.00346800
H	1.61055500	6.78366500	-1.13429000
H	-2.59392100	7.56704500	-0.72233500

H	-0.33642900	8.28828300	-1.47763000

[CuSaBOX2]-(minor)-TS_B

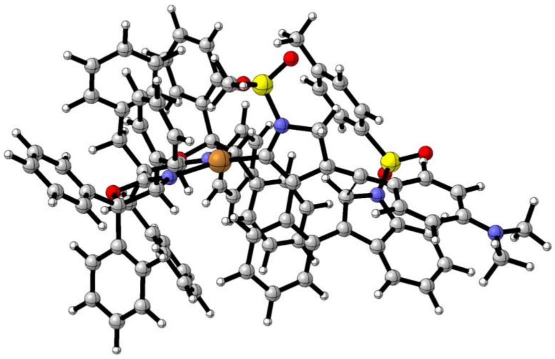

C	-3.99485300	-1.51504500	-1.30909600
C	-3.86896700	0.07062300	0.45469800
Cu	1.22047000	-0.46415000	-0.59730200
N	1.47803200	1.34653500	0.32187100
N	3.03430300	-1.02293200	0.08284000
N	-0.77020800	-1.03639000	-2.70890200
C	-2.13515500	-1.53786200	-2.96669800
H	-2.09374000	-2.54127200	-3.40911800
H	-2.68441900	-0.88052100	-3.63687800
C	-2.68585300	-1.57625700	-1.54639300
C	-5.23860300	-2.11027800	-1.42687900
C	-6.45393000	-1.37252400	-1.52970000
C	-5.35159500	-3.52900600	-1.26990600
C	-7.67692400	-1.98641700	-1.42759300
H	-6.42555500	-0.30203400	-1.68178700
C	-6.56897700	-4.14900200	-1.16050900
H	-4.43817100	-4.11039800	-1.19322000
C	-7.77812500	-3.38743400	-1.19354000
H	-8.56869100	-1.37912100	-1.50105200

H	-6.60428800	-5.22009900	-1.00930200
N	-8.98443200	-3.97785800	-0.99980600
C	-9.08064700	-5.41620500	-0.77284700
H	-10.13172200	-5.69722500	-0.71462800
H	-8.62629500	-5.97472100	-1.59805100
H	-8.58978600	-5.71093500	0.16354600
C	-10.18807700	-3.15923400	-0.88432600
H	-11.03203200	-3.80009300	-0.63118300
H	-10.07165400	-2.40695300	-0.09491800
H	-10.41615400	-2.64730100	-1.82639900
C	-0.41571400	-1.07458900	-1.36821600
C	-1.54584200	-1.47110100	-0.64885100
C	-1.52894300	-1.89616000	0.75128200
C	-2.66350200	-1.83387400	1.60180200
C	-0.35200000	-2.47883500	1.26863300
C	-2.59011900	-2.38996300	2.89499200
C	-0.28237400	-2.98309100	2.55847700
H	0.50876600	-2.55280300	0.61225600
C	-1.41816300	-2.95523800	3.37713200
H	-3.45232100	-2.30719600	3.54754500
H	0.64679300	-3.41707600	2.91749600
H	-1.38285500	-3.35640500	4.38573500
C	-3.86288900	-1.09840500	1.22140700
C	-5.22696400	-1.29025300	1.67412100
C	-5.99720200	-0.19044200	1.21416900
C	-5.87971300	-2.33643400	2.34505100
H	-3.02265500	0.60731300	0.05855900
C	-7.36389000	-0.07111500	1.45710400
C	-7.24610100	-2.23847600	2.57809200

H	-5.32549300	-3.21304800	2.66216400
C	-7.97500600	-1.11258600	2.15087500
H	-7.93089900	0.77755000	1.09561500
H	-7.76019500	-3.03980300	3.09996800
H	-9.03817000	-1.05189100	2.36408500
N	-5.12772100	0.64255100	0.47858100
S	-5.61332300	2.14193100	-0.25382000
O	-5.65767900	3.15819900	0.79359100
O	-6.82717600	1.81103500	-1.00450700
C	-4.27754000	2.49304700	-1.36764800
C	-3.56052300	3.67281800	-1.17250800
C	-4.03371900	1.66782400	-2.47198200
C	-2.56830300	4.01835800	-2.08895300
H	-3.79280500	4.30983500	-0.32751300
C	-3.03429100	2.02496600	-3.36829400
H	-4.61082100	0.76411200	-2.62316900
C	-2.29287100	3.20825300	-3.19766600
H	-2.00926400	4.93863000	-1.94654700
H	-2.82343700	1.39478200	-4.22679500
C	-1.25309000	3.59805200	-4.21334100
H	-0.47873600	2.82808400	-4.28335200
H	-1.70504500	3.69749100	-5.20694700
H	-0.77975300	4.54841300	-3.95486900
S	0.12538000	-0.20639500	-3.86656600
O	0.72517500	0.98568600	-3.24718900
O	-0.75740300	-0.06066700	-5.02925800
C	1.45569400	-1.34415500	-4.24364400
H	1.02734200	-2.24955100	-4.67467600
H	2.11934500	-0.85105000	-4.95591300

H	1.99005900	-1.56359300	-3.31923200
C	3.86581100	1.13200900	1.11052600
C	4.24204100	1.28673600	2.62615200
H	4.49252000	2.33915800	2.77963100
H	5.15534000	0.70701500	2.78482600
C	4.96901900	1.88921900	0.28159000
H	4.77784400	2.95839900	0.42137400
H	5.92798600	1.66957500	0.75955300
C	5.06821300	1.56231000	-1.19264100
C	3.96194500	1.62027300	-2.05073100
C	6.31698300	1.23466200	-1.73737000
C	4.10351000	1.37276300	-3.41469700
C	6.46380500	0.98661600	-3.10360300
C	5.35524900	1.06138900	-3.94844000
H	3.22480800	1.43351900	-4.04838600
H	7.44253400	0.73962900	-3.50431500
C	3.17367500	0.88130500	3.61725300
C	2.71055900	-0.43771600	3.71480100
C	2.64755800	1.83930100	4.49253000
C	1.75071000	-0.78701200	4.66442800
C	1.68258900	1.49508000	5.43920800
C	1.23275100	0.17772700	5.52924500
H	1.41295600	-1.81610000	4.72558900
H	1.28591600	2.25474000	6.10636900
C	3.89970600	-0.32836800	0.72085900
C	3.54183600	-2.39832300	-0.06610400
C	4.83663600	-2.40738600	0.83897800
C	2.52775000	1.80270900	0.89411400
C	0.42920600	2.37341900	0.40852500

C	1.27188600	3.67294500	0.66949100
O	2.46568600	3.07199900	1.34071300
O	5.02913100	-0.96422800	1.08496200
H	-0.07595000	2.44278000	-0.55353100
H	2.78937900	-3.05825600	0.36139200
H	3.00385400	2.86387200	4.43246400
H	3.10720100	-1.20826200	3.06773200
H	2.98190100	1.86735200	-1.67081700
H	7.18751200	1.19046700	-1.08751500
H	0.48533800	-0.09452900	6.26855900
H	5.46804100	0.87978800	-5.01361700
C	0.62869700	4.67037500	1.59914000
C	-0.04363200	5.77849800	1.07258300
C	0.58419000	4.43286500	2.97708800
C	-0.75941100	6.63288700	1.91225900
H	-0.00766900	5.97256400	0.00543600
C	-0.12082400	5.29280200	3.81499200
H	1.07749200	3.56027200	3.38439200
C	-0.79900900	6.39289300	3.28580300
H	-1.27904100	7.48908600	1.49221500
H	-0.14992600	5.09674300	4.88287000
H	-1.35359900	7.05901200	3.93999700
C	1.83319400	4.30363200	-0.60201800
C	2.85176300	5.25751000	-0.44221900
C	1.44033000	3.94954900	-1.89480600
C	3.47092000	5.83144700	-1.54758800
H	3.15598000	5.54185000	0.56025500
C	2.06188800	4.52857300	-3.00583100
H	0.68599600	3.19450000	-2.07131800

C	3.07709800	5.46560300	-2.83836800
H	4.25960300	6.56406800	-1.40428800
H	1.75608100	4.22358300	-4.00150900
H	3.56286100	5.90731600	-3.70322600
C	-0.61295000	2.02572300	1.45483700
C	-1.80276700	2.76443900	1.48313200
C	-0.42034000	1.01593000	2.39849300
C	-2.78621900	2.50373900	2.43289900
H	-1.93845500	3.57045800	0.77390200
C	-1.40718400	0.74828000	3.34983300
H	0.49107400	0.43336600	2.39998800
C	-2.59046600	1.48303700	3.36772900
H	-3.70459800	3.08110400	2.43220500
H	-1.24425600	-0.04865600	4.06506400
H	-3.35992600	1.26405400	4.10245300
C	3.72304000	-2.78558100	-1.51307600
C	4.49453400	-2.00875100	-2.38400900
C	3.13317300	-3.96197600	-1.98464300
C	4.69201800	-2.42079400	-3.69990000
H	4.94478400	-1.08766000	-2.03769800
C	3.32240000	-4.37171200	-3.30547900
H	2.52731400	-4.56504300	-1.31221100
C	4.10992200	-3.60319000	-4.16416000
H	5.30115900	-1.81201100	-4.35987800
H	2.86242900	-5.28989900	-3.65893400
H	4.26950700	-3.92353100	-5.18967100
C	4.61718100	-3.04103500	2.21208900
C	3.64473400	-4.01204300	2.46870000
C	5.44798700	-2.62581100	3.26305800

C	3.47731100	-4.52678900	3.75813500
H	3.00806000	-4.39091900	1.67652800
C	5.27592900	-3.13014700	4.54809000
H	6.21941400	-1.88857600	3.06889300
C	4.28186100	-4.07845300	4.80318800
H	2.71610400	-5.28051200	3.93742500
H	5.91727600	-2.78308000	5.35240400
H	4.14462700	-4.47034900	5.80630200
C	6.07858500	-2.95521000	0.16652600
C	6.19391200	-4.33807900	-0.01331900
C	7.07928900	-2.11364800	-0.32155200
C	7.28776400	-4.86955700	-0.69118300
H	5.42628400	-5.00000300	0.37604900
C	8.17971100	-2.64861000	-0.99301400
H	6.99045700	-1.04336500	-0.18899500
C	8.28553300	-4.02531100	-1.18439500
H	7.36393800	-5.94376500	-0.83033000
H	8.95291700	-1.98438800	-1.36831000
H	9.14039200	-4.44046800	-1.70963900

7. Reference:

1. Zhao, H.-B.; Liu, Z.-J.; Song, J.; Xu, H.-C. Reagent-Free C-H/N-H Cross-Coupling: Regioselective Synthesis of N-Heteroaromatics from Biaryl Aldehydes and NH_3. *Angew. Chem., Int. Ed.* **2017**, *56*, 12732−12735.

2. Huang, R. Y.; Franke, P. T.; Nicolaus, N.; Lautens, M. Domino C−H Functionalization Reactions of Gem-Dibromoolefins: Synthesis of N-Fused Benzo[*c*]carbazoles. *Tetrahedron* **2013**, *69*, 4395−4402.

3. Hong, F.-L.; Wang, Z.-S.; Wei, D.-D.; Zhai, T.-Y.; Deng, G.-C.; Lu, X.; Liu, R.-S.; Ye, L.-W. Generation of Donor/Donor Copper Carbenes through Coppercatalyzed Diyne Cyclization: Enantioselective and Divergent Synthesis of Chiral Polycyclic Pyrroles. *J. Am. Chem. Soc.* **2019**, *141*, 16961–16970.

4. Rajasekar, S.; Anbarasan, P. Rhodium-catalyzed transannulation of 1,2,3triazoles to polysubstituted pyrroles. *J. Org. Chem.* **2014**, *79*, 8428–8434.

5. Zhu, X.-Q.; Hong, P.; Zheng, Y.-X.; Zhen, Y.-Y.; Hong, F.-L.; Lu, X.; Ye, L.-W. Copper-catalyzed asymmetric cyclization of alkenyl diynes: method development and new mechanistic insights. *Chem. Sci.* **2021**, *12*, 9466–9474.

6. Pascalis, L. D.; Tekkam, S.; Finn, M. G. Azanorbornadienes as Thiol-Reactive Cleavable Linkers. *Org. Lett.* **2020**, *22*, 6248–6251.

7. Signaigo, F. K.; Adkins, H. The Reactions of Hydrogen with Derivatives of Pyrrole. *J. Am. Chem. Soc.* **1936**, *58*, 709–716.

8. Frisch, M. J.; Trucks, G. W.; Schlegel, H. B.; Scuseria, G. E.; Robb, M. A.; Cheeseman, J. R.; Scalmani, G.; Barone, V.; Petersson, G. A.; Nakatsuji, H.; Li, X.; Caricato, M.; Marenich, A. V.; Bloino, J.; Janesko, B. G.; Gomperts, R.; Mennucci, B.; Hratchian, H. P.; Ortiz, J. V.; Izmaylov, A. F.; Sonnenberg, J. L.; Williams-Young, D.; Ding, F.; Lipparini, F.; Egidi, F.; Goings, J.; Peng, B. Petrone, A.; Henderson, T.; Ranasinghe, D.; Zakrzewski, V. G.; Gao, J.; Rega, N.; Zheng, G.; Liang, W.; Hada, M.; Ehara, M.; Toyota, K.; Fukuda, R.; Hasegawa, J.; Ishida, M.; Nakajima, T.; Honda, Y.; Kitao, O.; Nakai, H.; Vreven, T.; Throssell, K.; Montgomery, J. A.; Jr., Peralta, J. E.; Ogliaro, F.; Bearpark, M. J.; Heyd, J. J.; Brothers, E. N.; Kudin, K. N.; Staroverov, V.

N.; Keith, T. A.; Kobayashi, R.; Normand, J.; Raghavachari, K.; Rendell, A. P.; Burant, J. C.; Iyengar, S. S.; Tomasi, J.; Cossi, M.; Millam, J. M.; Klene, M.; Adamo, C.; Cammi, R.; Ochterski, J. W.; Martin, R. L.; Morokuma, K.; Farkas, O.; Foresman, J. B.; Fox, D. J. *Gaussian*, *Inc*., *Wallingford CT*, **2016**.

9. Becke, A. D. Density-Functional Thermochemistry. III. The Role of Exact Exchange. *J. Chem. Phys*. **1993**, *98*, 5648–5652.

10. Lee, C.; Yang, W.; Parr, R. G. Development of the Colle-Salvetti Correlation-Energy Formula into a Functional of the Electron Density. *Phys. Rev. B*: *Condens. Matter Mater. Phys*. **1988**, *37*, 785–789.

11. Wadt, W. R. & Hay, P. J. Ab initio Effective Core Potentials for Molecular Calculations. Potentials for Main Group Elements Na to Bi. *J. Chem. Phys*. 1985, **82**, 284–298.

12. Hay, P. J.; Wadt, W. R. Ab initio Effective Core Potentials for Molecular Calculations. Potentials for the Transition Metal Atoms Sc to Hg. *J. Chem. Phys*. **1985**, *82*, 270–283.

13. Woon, D. E.; Dunning, T. H. Gaussian Basis Sets for use in Correlated Molecular Calculations. V. Core-Valence Basis Sets for Boron Through Neon. *J. Chem. Phys*. **1995**, *103*, 4572–4585.

14. Weigend, F.; Ahlrichs, R. Balanced Basis Sets of Split Valence, Triple Zeta Valence and Quadruple Zeta Valence Quality for H to Rn: Design and Assessment of Accuracy. *Phy. Chem. Chem. Phy*. **2005**, *7,* 3297-3305.

15. Weigend, F. Accurate Coulomb-Fitting Basis Sets for H to Rn. *Phy. Chem. Chem. Phy*. **2006**, *8,* 1057-1065.

16. Marenich, A. V.; Cramer, C. J.; Truhlar, D. G. Universal Solvation Model based on Solute Electron Density and on a Continuum Model of the Solvent Defined by the Bulk Dielectric Constant and Atomic Surface Tensions. *J. Phys. Chem. B*. **2009**, *113*, 6378–6396.

17. CYLview 1.0b; Legault, C. Y. Université de Sherbrooke, **2009**, (http://www.cylview.org).

8. HPLC Chromatograms

Compound B1: IA, *i*-PrOH/hexane = 50/50, v = 1.0 mL/min, λ = 254 nm

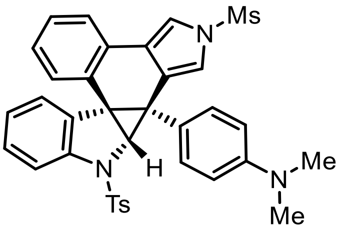

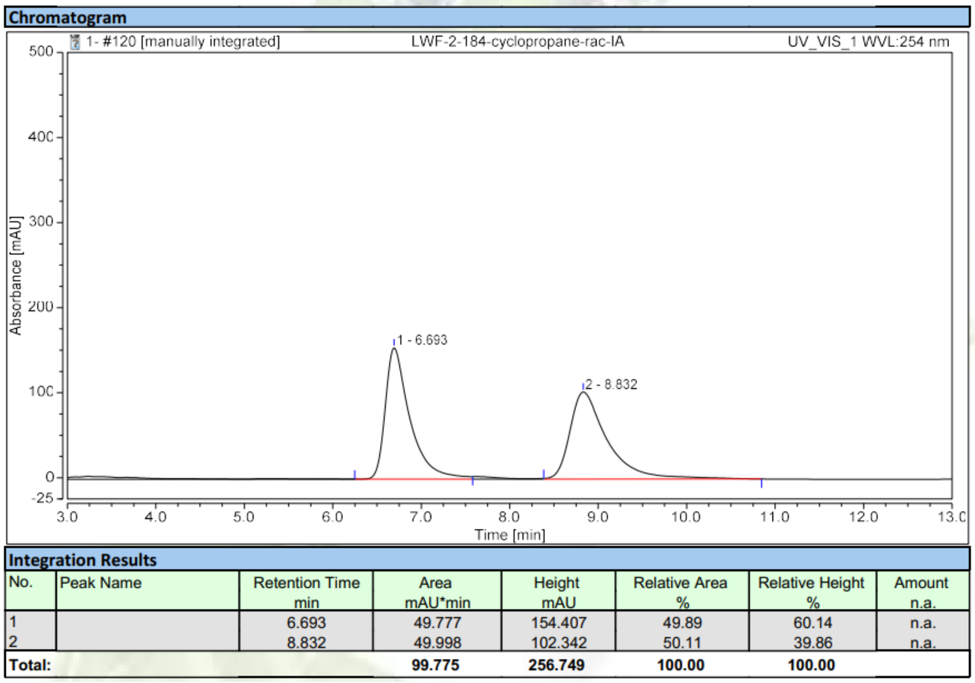

Integration Results

No.	Peak Name	Retention Time min	Area mAU*min	Height mAU	Relative Area %	Relative Height %	Amount n.a.
1		6.693	49.777	154.407	49.89	60.14	n.a.
2		8.832	49.998	102.342	50.11	39.86	n.a.
**Total:**			**99.775**	**256.749**	**100.00**	**100.00**	

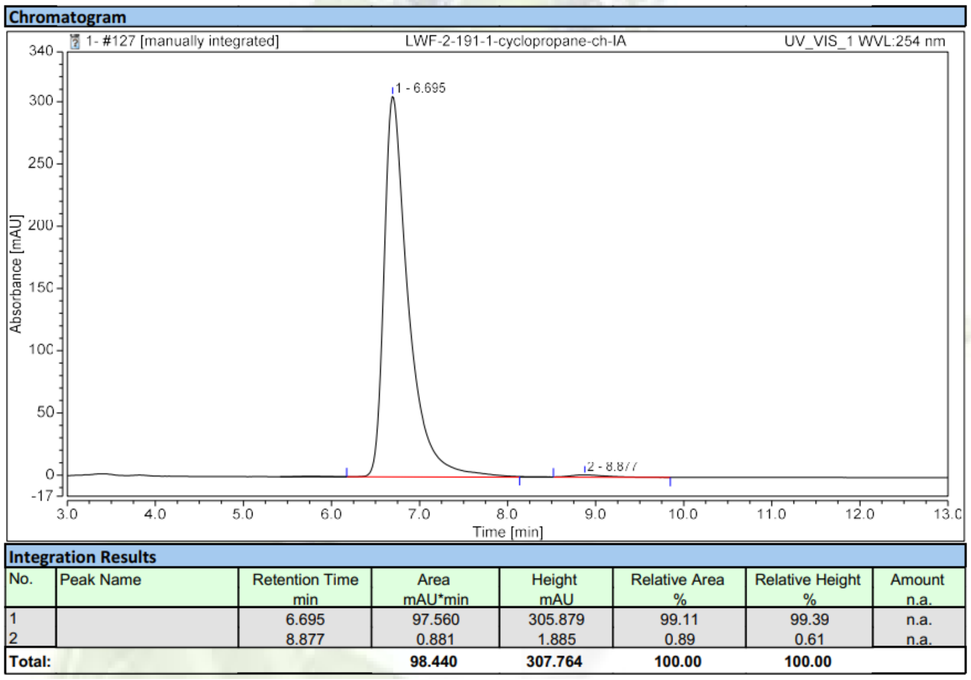

Integration Results

No.	Peak Name	Retention Time min	Area mAU*min	Height mAU	Relative Area %	Relative Height %	Amount n.a.
1		6.695	97.560	305.879	99.11	99.39	n.a.
2		8.877	0.881	1.885	0.89	0.61	n.a.
**Total:**			**98.440**	**307.764**	**100.00**	**100.00**	

Compound B2: IC, *i*-PrOH/hexane = 50/50, v = 1.0 mL/min, λ = 254 nm

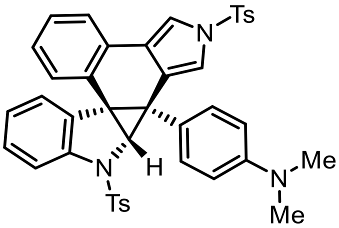

Chromatogram

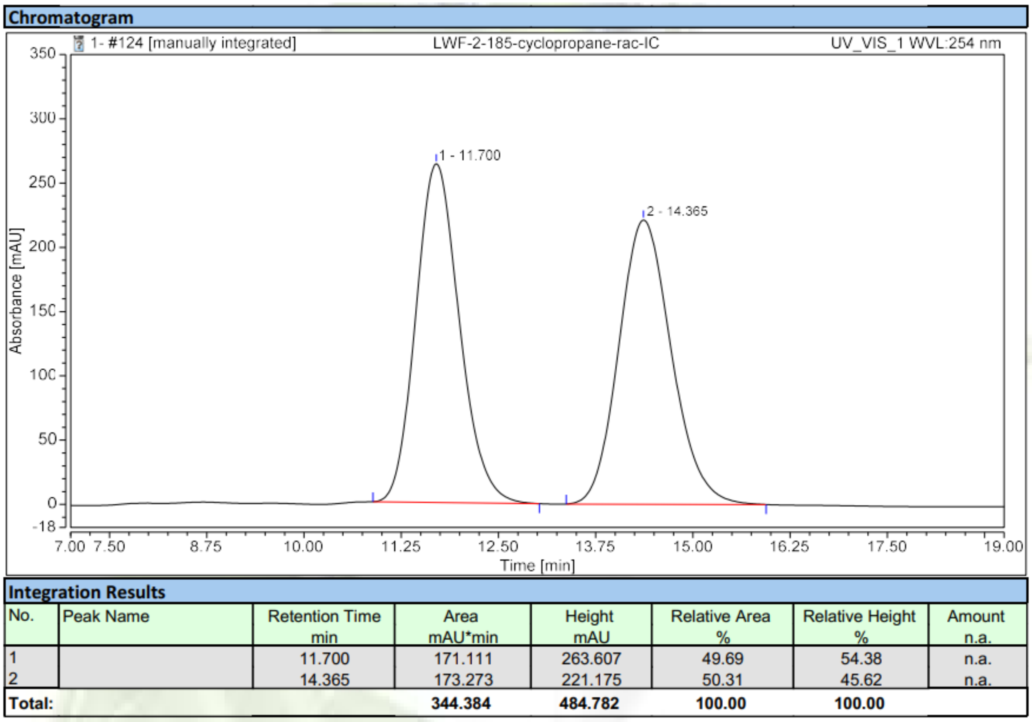

Integration Results

No.	Peak Name	Retention Time min	Area mAU*min	Height mAU	Relative Area %	Relative Height %	Amount n.a.
1		11.700	171.111	263.607	49.69	54.38	n.a.
2		14.365	173.273	221.175	50.31	45.62	n.a.
**Total:**			**344.384**	**484.782**	**100.00**	**100.00**	

Chromatogram

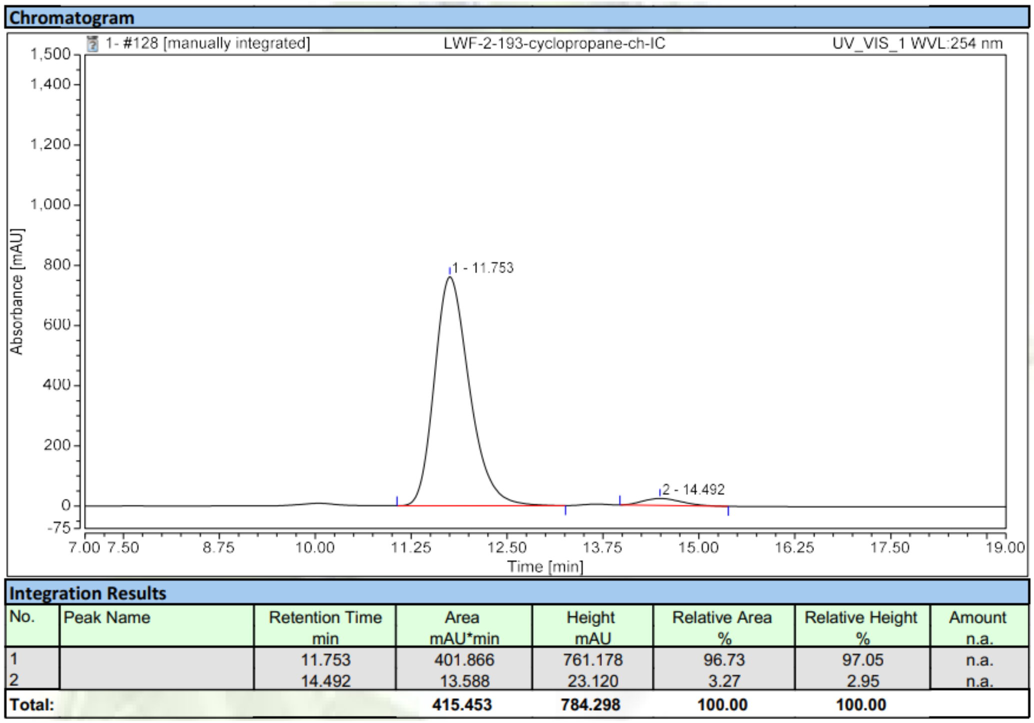

Integration Results

No.	Peak Name	Retention Time min	Area mAU*min	Height mAU	Relative Area %	Relative Height %	Amount n.a.
1		11.753	401.866	761.178	96.73	97.05	n.a.
2		14.492	13.588	23.120	3.27	2.95	n.a.
**Total:**			**415.453**	**784.298**	**100.00**	**100.00**	

Compound B3: IC, *i*-PrOH/hexane = 50/50, v = 1.0 mL/min, λ = 254 nm

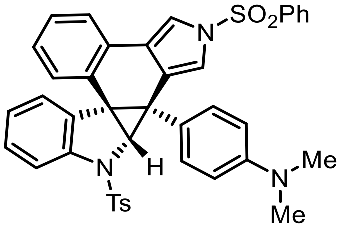

Chromatogram

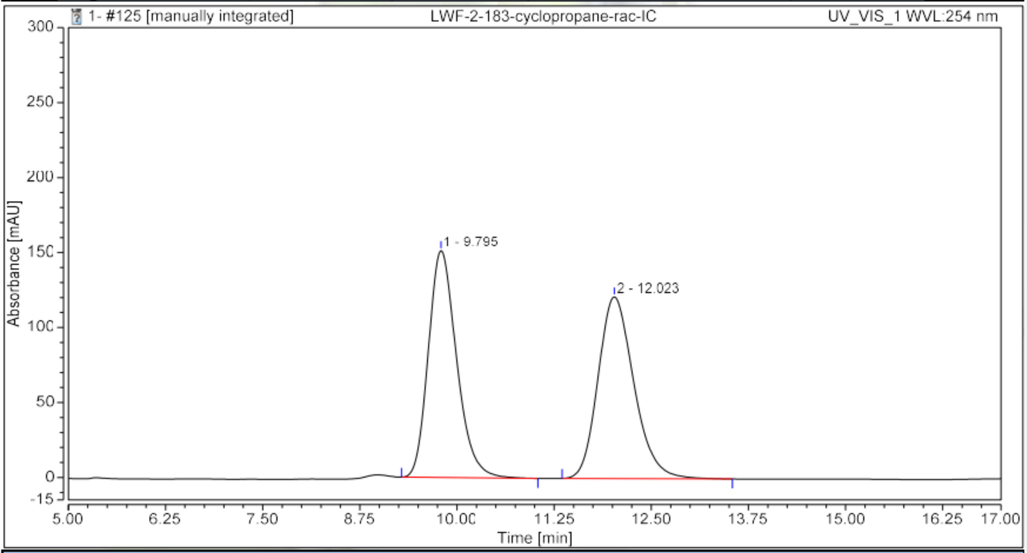

Integration Results

No.	Peak Name	Retention Time min	Area mAU*min	Height mAU	Relative Area %	Relative Height %	Amount n.a.
1		9.795	63.777	151.408	49.63	55.53	n.a.
2		12.023	64.716	121.238	50.37	44.47	n.a.
**Total:**			**128.493**	**272.646**	**100.00**	**100.00**	

Chromatogram

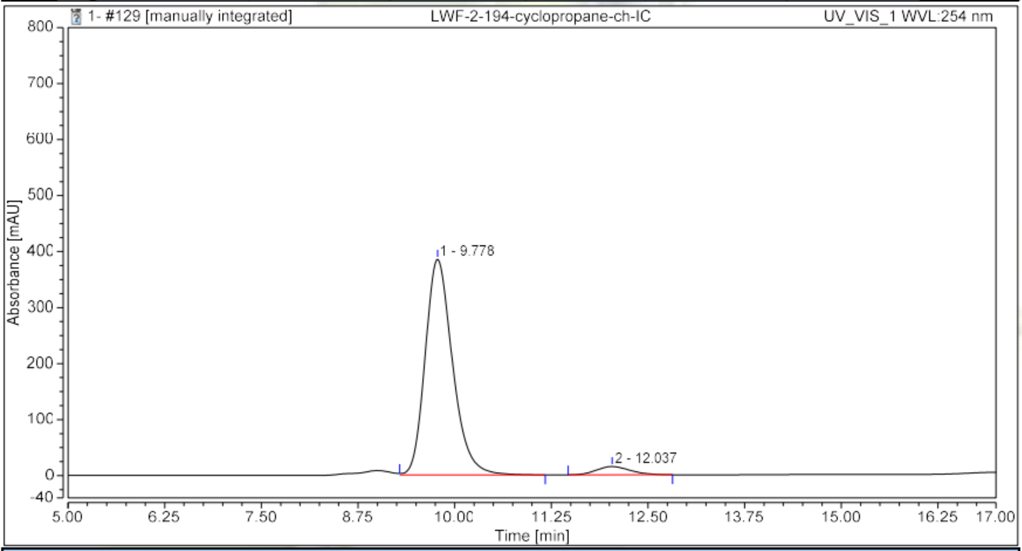

Integration Results

No.	Peak Name	Retention Time min	Area mAU*min	Height mAU	Relative Area %	Relative Height %	Amount n.a.
1		9.778	158.163	385.308	95.44	96.31	n.a.
2		12.037	7.556	14.746	4.56	3.69	n.a.
**Total:**			**165.720**	**400.053**	**100.00**	**100.00**	

Compound B4: IC, *i*-PrOH/hexane = 50/50, v = 1.0 mL/min, λ = 254 nm

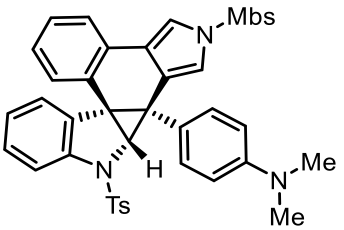

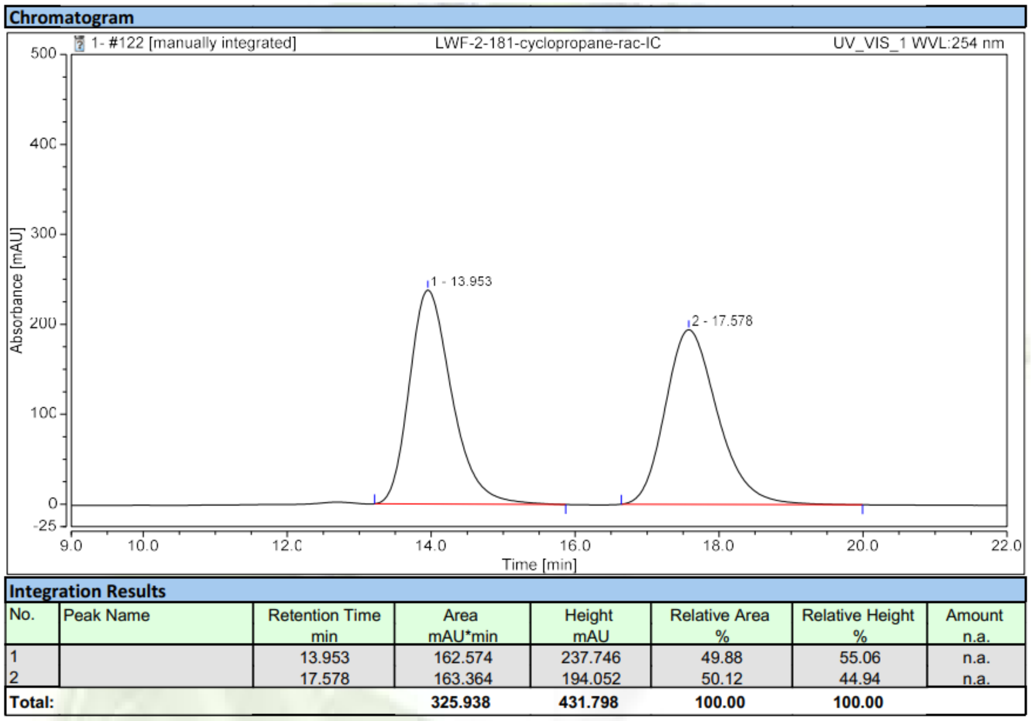

Integration Results

No.	Peak Name	Retention Time min	Area mAU*min	Height mAU	Relative Area %	Relative Height %	Amount n.a.
1		13.953	162.574	237.746	49.88	55.06	n.a.
2		17.578	163.364	194.052	50.12	44.94	n.a.
**Total:**			**325.938**	**431.798**	**100.00**	**100.00**	

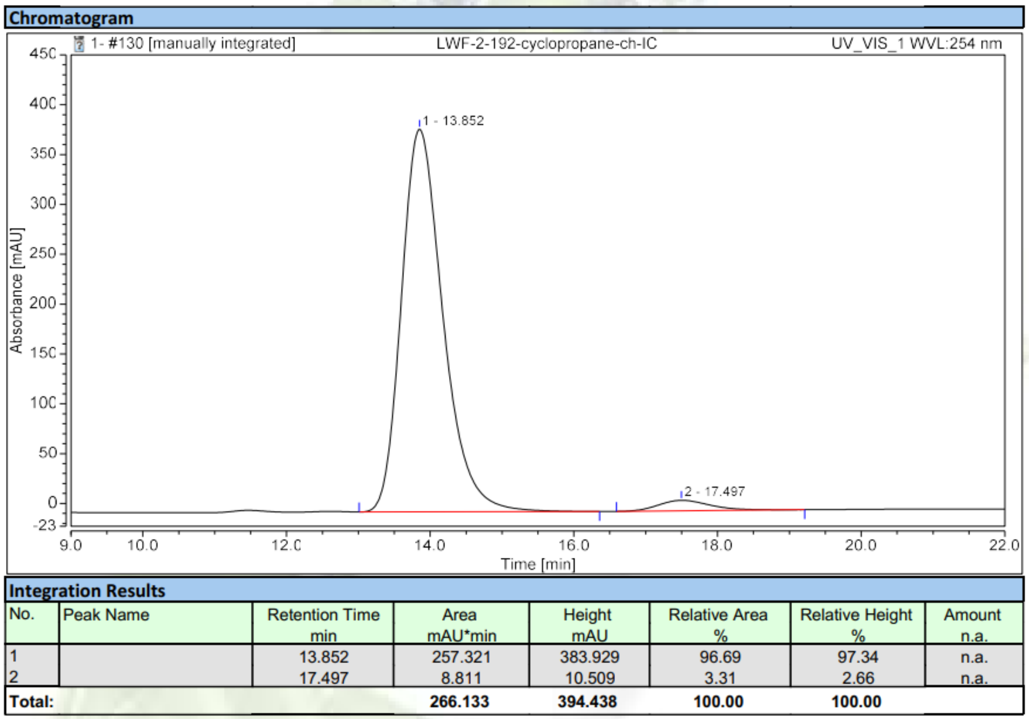

Integration Results

No.	Peak Name	Retention Time min	Area mAU*min	Height mAU	Relative Area %	Relative Height %	Amount n.a.
1		13.852	257.321	383.929	96.69	97.34	n.a.
2		17.497	8.811	10.509	3.31	2.66	n.a.
**Total:**			**266.133**	**394.438**	**100.00**	**100.00**	

Compound B5: IC, *i*-PrOH/hexane = 50/50, v = 1.0 mL/min, λ = 254 nm

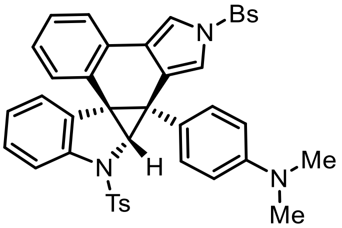

Chromatogram

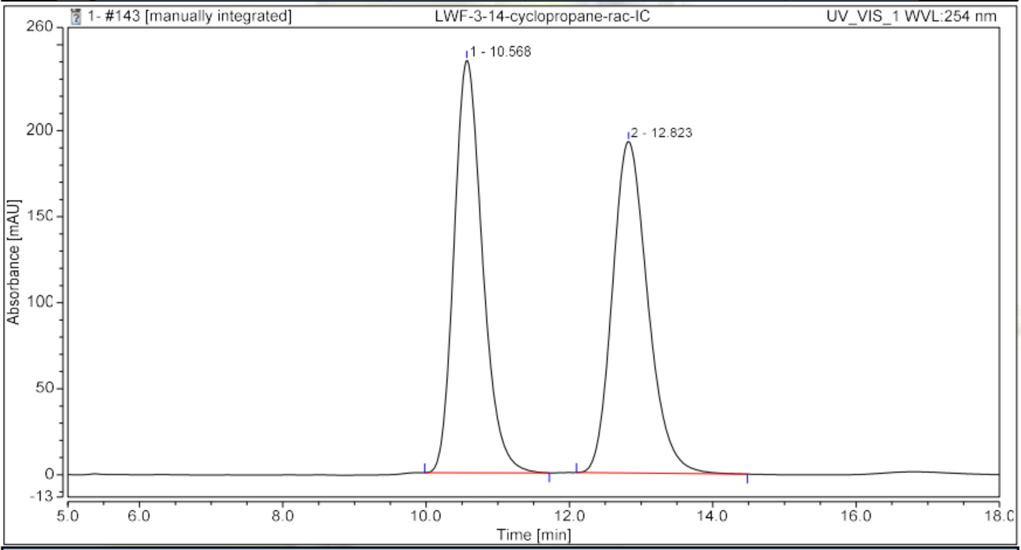

Integration Results

No.	Peak Name	Retention Time min	Area mAU*min	Height mAU	Relative Area %	Relative Height %	Amount n.a.
1		10.568	110.102	239.805	49.97	55.44	n.a.
2		12.823	110.241	192.768	50.03	44.56	n.a.
**Total:**			**220.343**	**432.573**	**100.00**	**100.00**	

Chromatogram

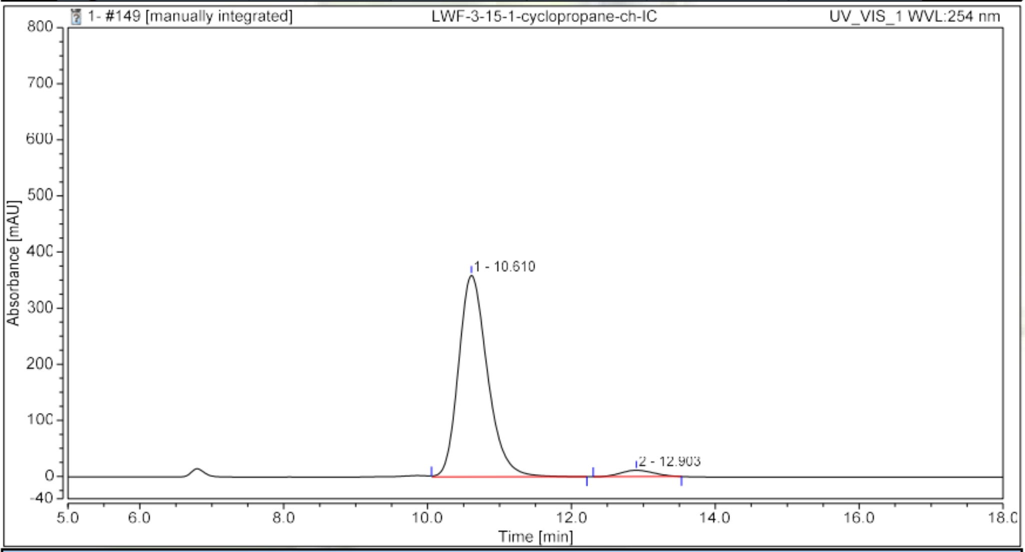

Integration Results

No.	Peak Name	Retention Time min	Area mAU*min	Height mAU	Relative Area %	Relative Height %	Amount n.a.
1		10.610	167.495	359.239	96.49	96.94	n.a.
2		12.903	6.092	11.331	3.51	3.06	n.a.
**Total:**			**173.587**	**370.570**	**100.00**	**100.00**	

Compound B6: IA, *i*-PrOH/hexane = 50/50, v = 1.0 mL/min, λ = 254 nm

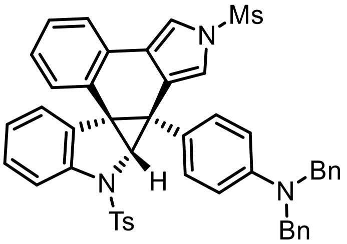

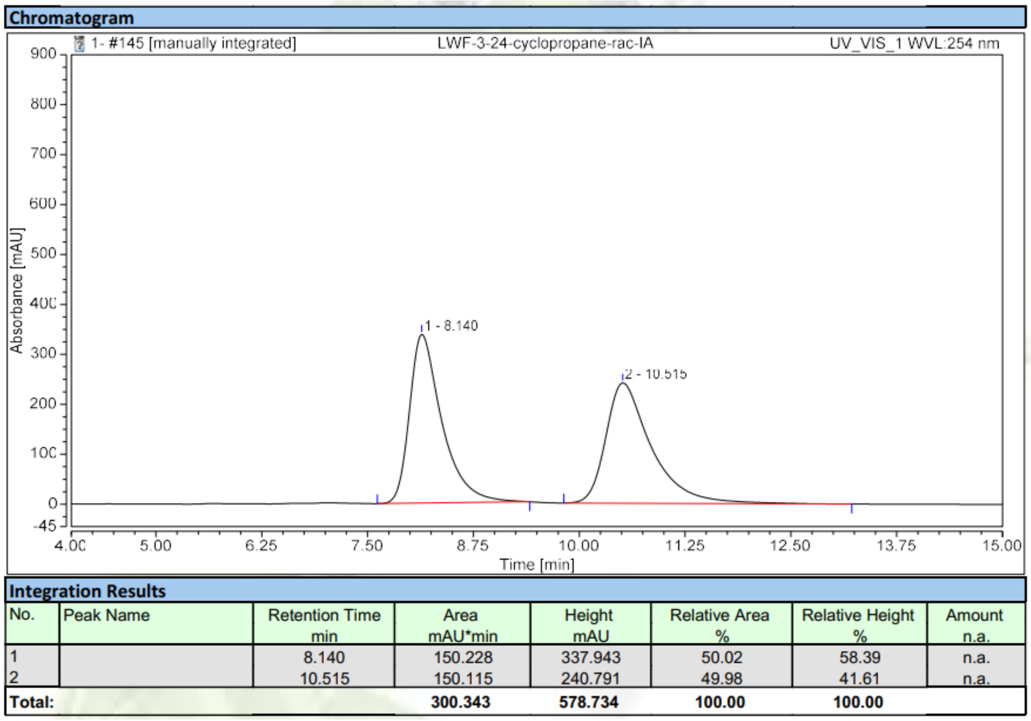

Integration Results

No.	Peak Name	Retention Time min	Area mAU*min	Height mAU	Relative Area %	Relative Height %	Amount n.a.
1		8.140	150.228	337.943	50.02	58.39	n.a.
2		10.515	150.115	240.791	49.98	41.61	n.a.
**Total:**			**300.343**	**578.734**	**100.00**	**100.00**	

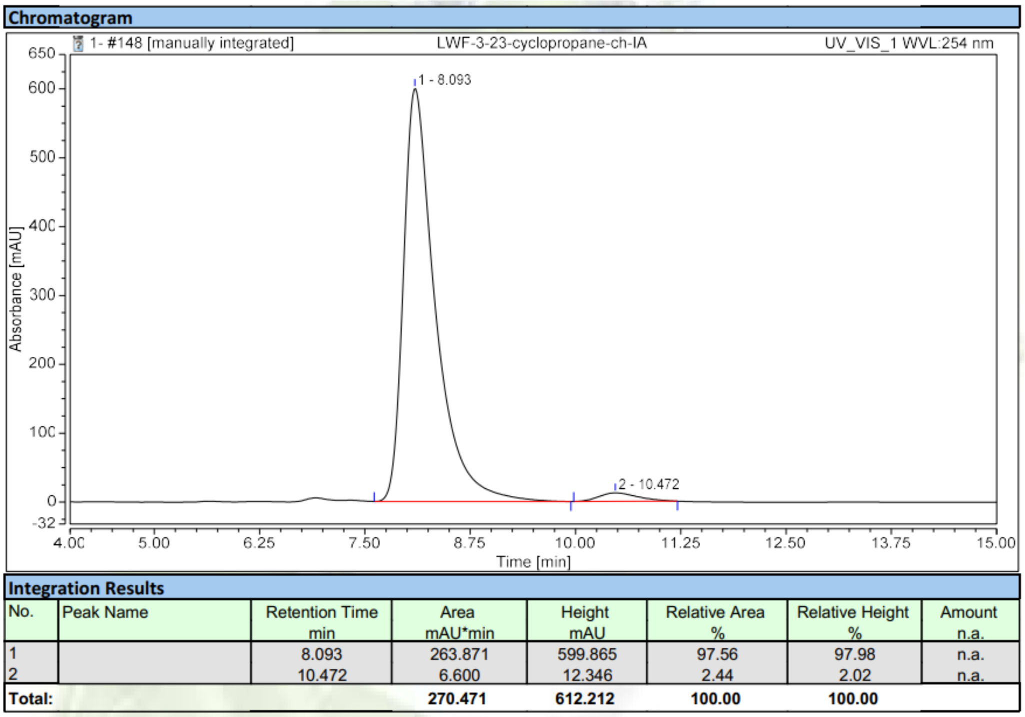

Integration Results

No.	Peak Name	Retention Time min	Area mAU*min	Height mAU	Relative Area %	Relative Height %	Amount n.a.
1		8.093	263.871	599.865	97.56	97.98	n.a.
2		10.472	6.600	12.346	2.44	2.02	n.a.
**Total:**			**270.471**	**612.212**	**100.00**	**100.00**	

Compound B7: IA, *i*-PrOH/hexane = 50/50, v = 1.0 mL/min, λ = 254 nm

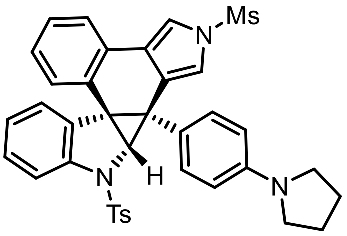

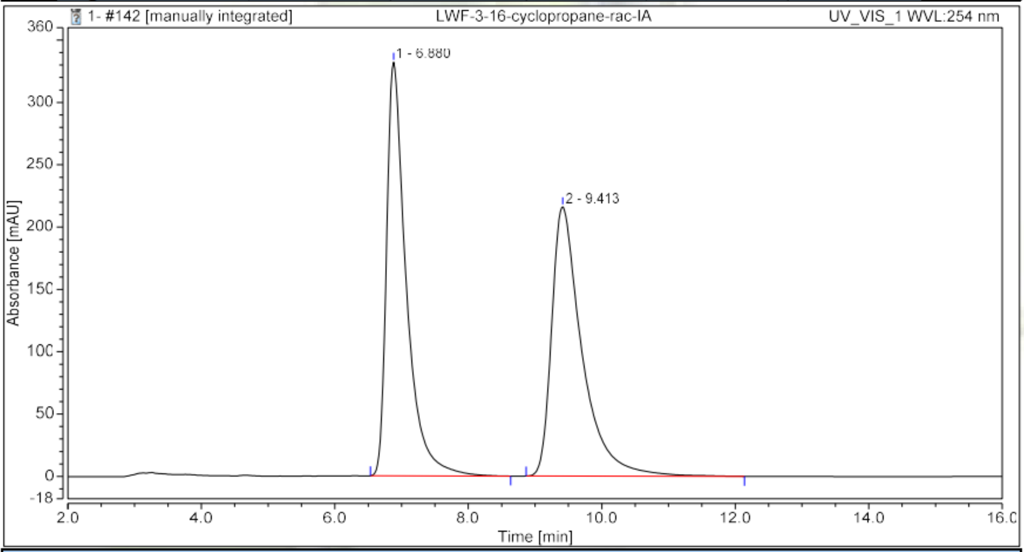

Integration Results

No.	Peak Name	Retention Time min	Area mAU*min	Height mAU	Relative Area %	Relative Height %	Amount n.a.
1		6.880	113.604	331.934	49.98	60.57	n.a.
2		9.413	113.713	216.108	50.02	39.43	n.a.
Total:			227.317	548.041	100.00	100.00	

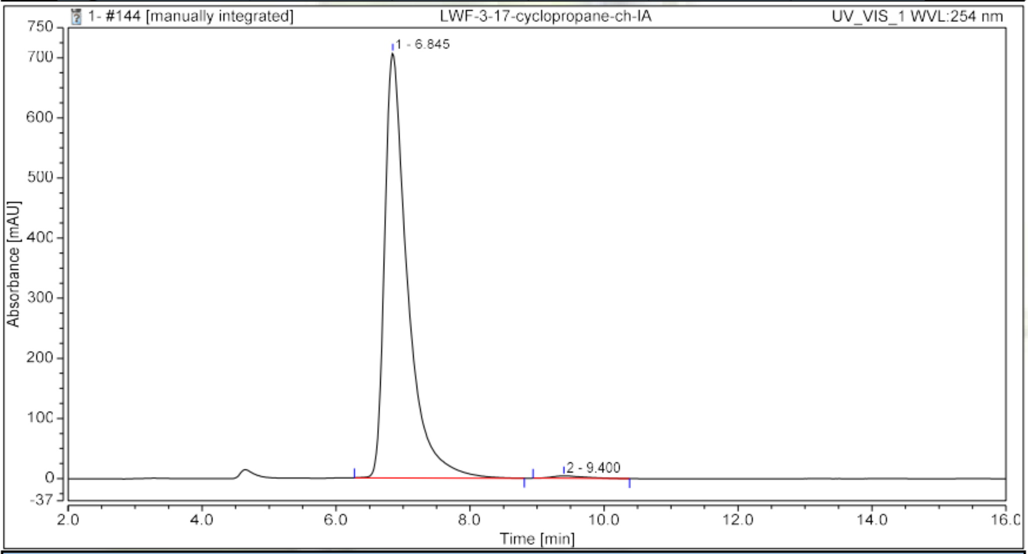

Integration Results

No.	Peak Name	Retention Time min	Area mAU*min	Height mAU	Relative Area %	Relative Height %	Amount n.a.
1		6.845	279.248	706.710	99.18	99.43	n.a.
2		9.400	2.307	4.027	0.82	0.57	n.a.
Total:			281.556	710.737	100.00	100.00	

Compound B8: IC, *i*-PrOH/hexane = 50/50, v = 1.0 mL/min, λ = 254 nm

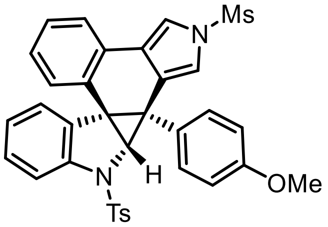

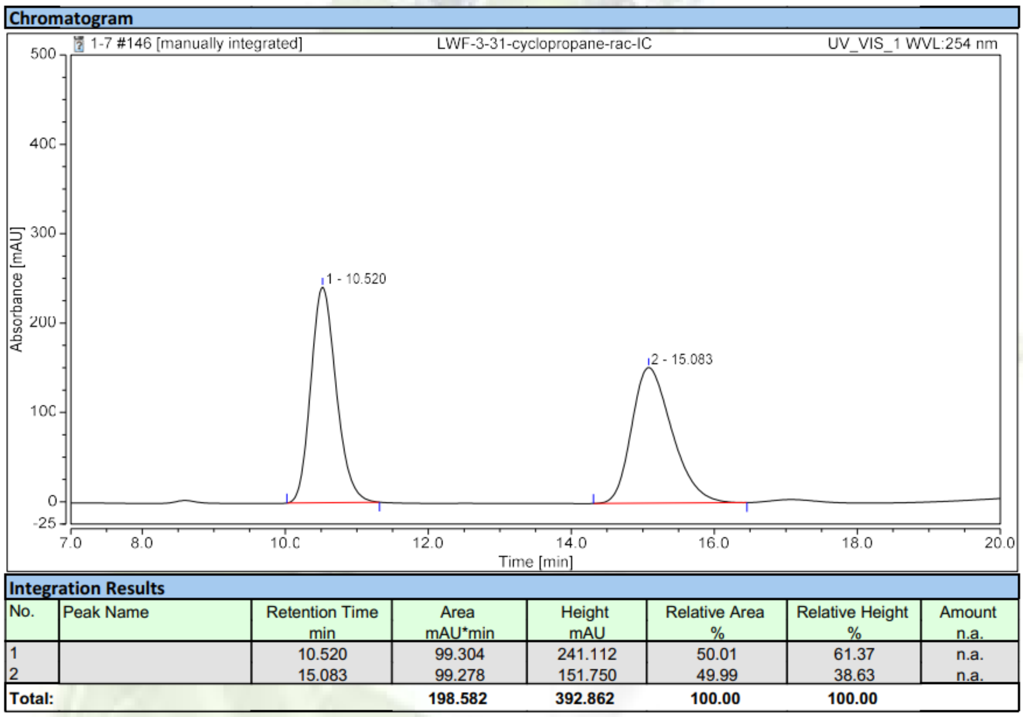

Integration Results

No.	Peak Name	Retention Time min	Area mAU*min	Height mAU	Relative Area %	Relative Height %	Amount n.a.
1		10.520	99.304	241.112	50.01	61.37	n.a.
2		15.083	99.278	151.750	49.99	38.63	n.a.
**Total:**			**198.582**	**392.862**	**100.00**	**100.00**	

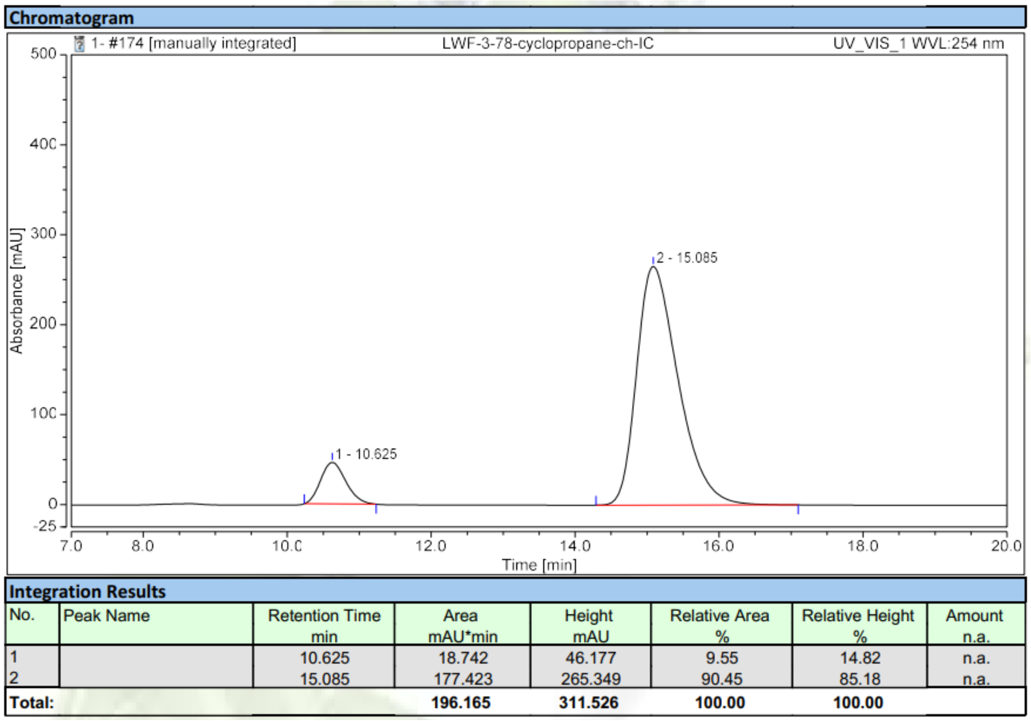

Integration Results

No.	Peak Name	Retention Time min	Area mAU*min	Height mAU	Relative Area %	Relative Height %	Amount n.a.
1		10.625	18.742	46.177	9.55	14.82	n.a.
2		15.085	177.423	265.349	90.45	85.18	n.a.
**Total:**			**196.165**	**311.526**	**100.00**	**100.00**	

Compound B9: IC, *i*-PrOH/hexane = 50/50, v = 1.0 mL/min, λ = 254 nm

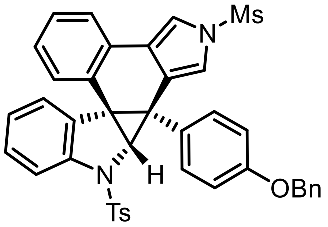

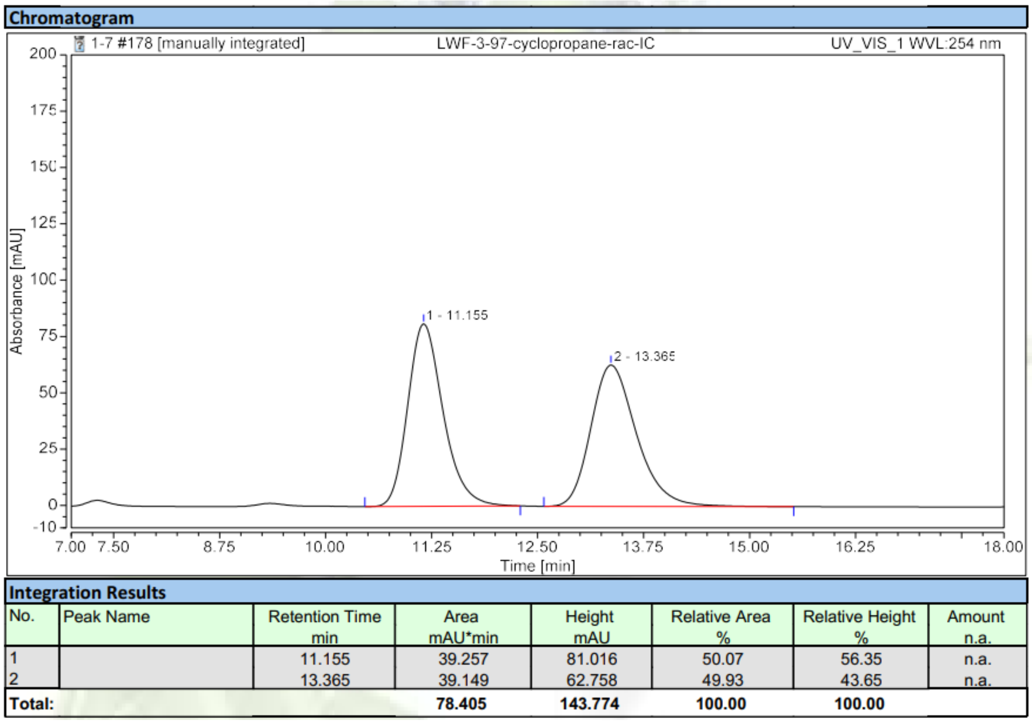

Integration Results

No.	Peak Name	Retention Time min	Area mAU*min	Height mAU	Relative Area %	Relative Height %	Amount n.a.
1		11.155	39.257	81.016	50.07	56.35	n.a.
2		13.365	39.149	62.758	49.93	43.65	n.a.
**Total:**			**78.405**	**143.774**	**100.00**	**100.00**	

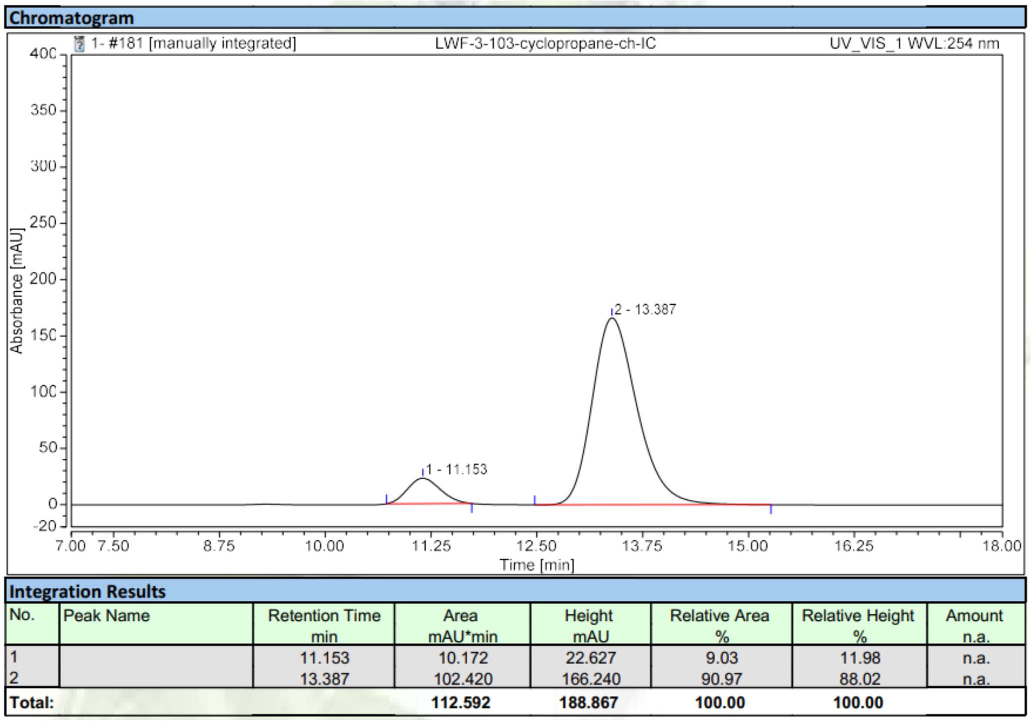

Integration Results

No.	Peak Name	Retention Time min	Area mAU*min	Height mAU	Relative Area %	Relative Height %	Amount n.a.
1		11.153	10.172	22.627	9.03	11.98	n.a.
2		13.387	102.420	166.240	90.97	88.02	n.a.
**Total:**			**112.592**	**188.867**	**100.00**	**100.00**	

Compound B10: IC, *i*-PrOH/hexane = 50/50, v = 1.0 mL/min, λ = 254 nm

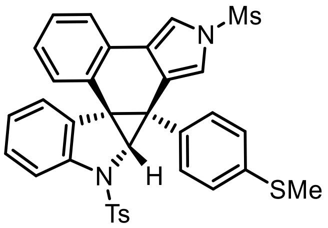

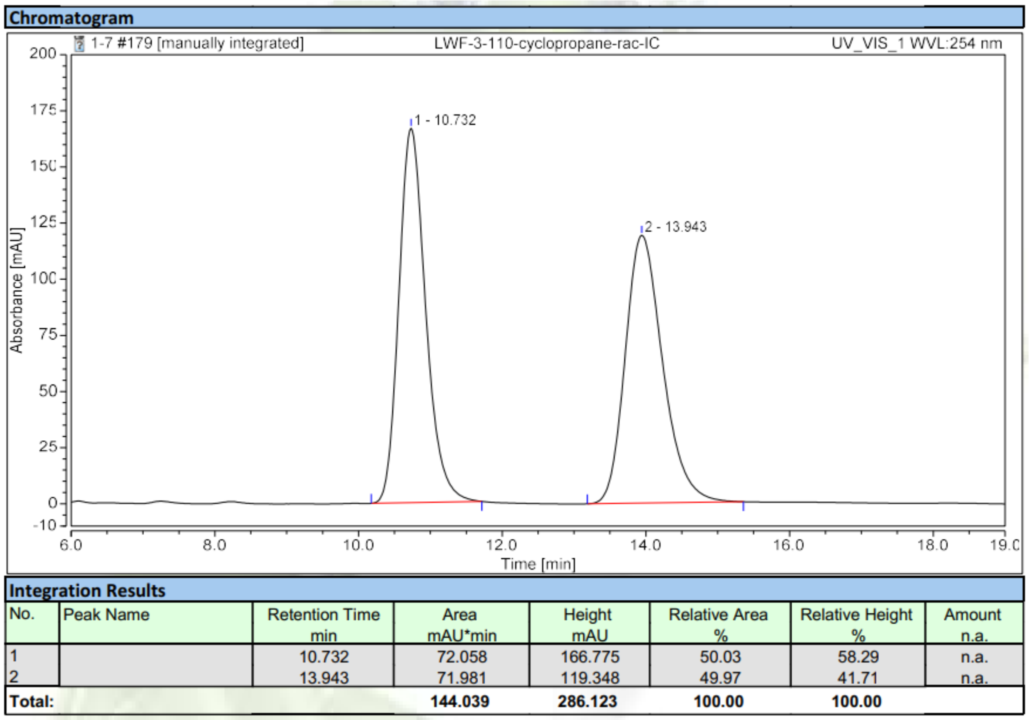

Integration Results

No.	Peak Name	Retention Time min	Area mAU*min	Height mAU	Relative Area %	Relative Height %	Amount n.a.
1		10.732	72.058	166.775	50.03	58.29	n.a.
2		13.943	71.981	119.348	49.97	41.71	n.a.
Total:			144.039	286.123	100.00	100.00	

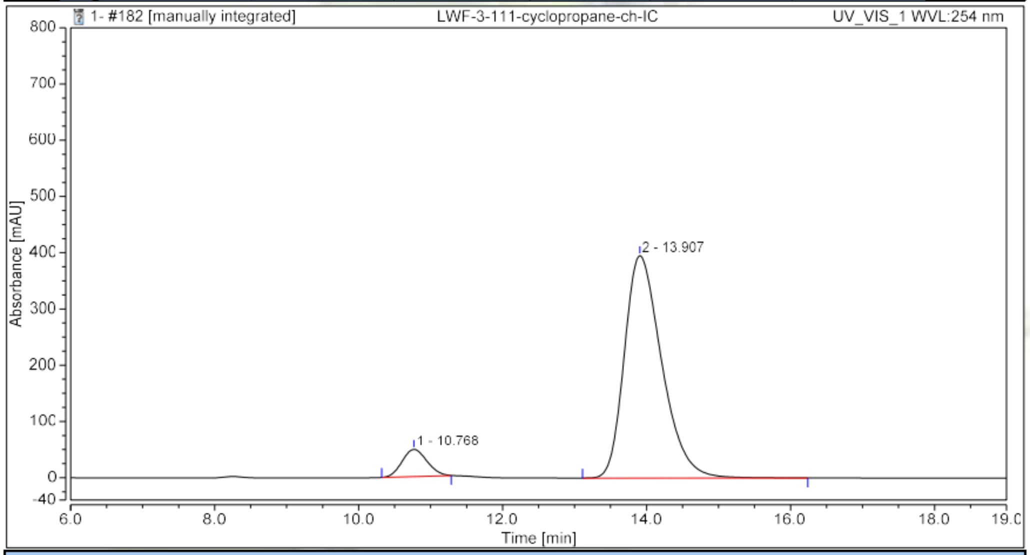

Integration Results

No.	Peak Name	Retention Time min	Area mAU*min	Height mAU	Relative Area %	Relative Height %	Amount n.a.
1		10.768	19.294	48.254	7.51	10.88	n.a.
2		13.907	237.448	395.407	92.49	89.12	n.a.
Total:			256.742	443.662	100.00	100.00	

Compound B11: IC, *i*-PrOH/hexane = 50/50, v = 1.0 mL/min, λ = 254 nm

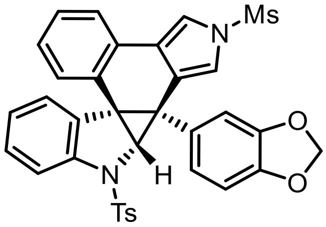

Chromatogram

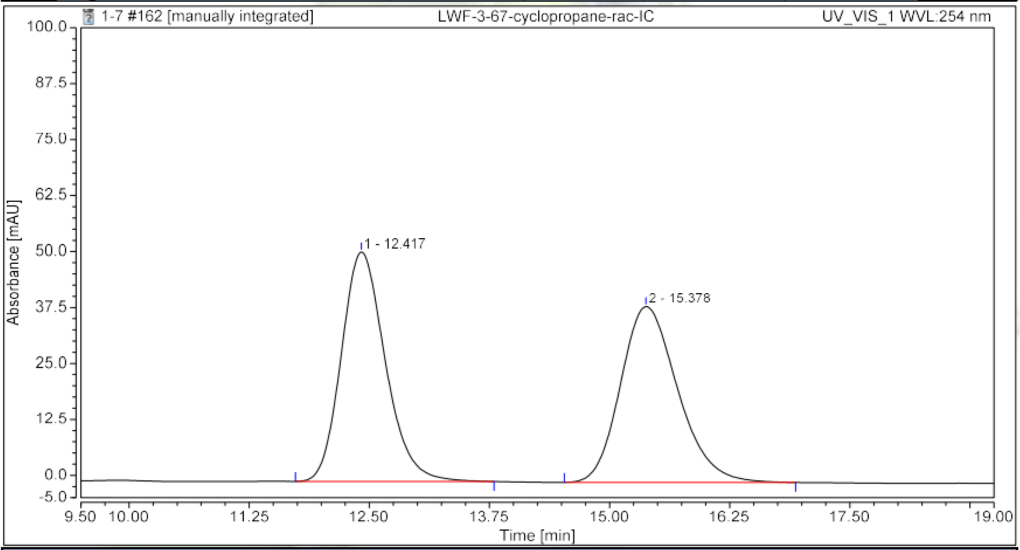

Integration Results

No.	Peak Name	Retention Time min	Area mAU*min	Height mAU	Relative Area %	Relative Height %	Amount n.a.
1		12.417	27.185	51.263	50.05	56.61	n.a.
2		15.378	27.136	39.292	49.95	43.39	n.a.
**Total:**			**54.321**	**90.555**	**100.00**	**100.00**	

Chromatogram

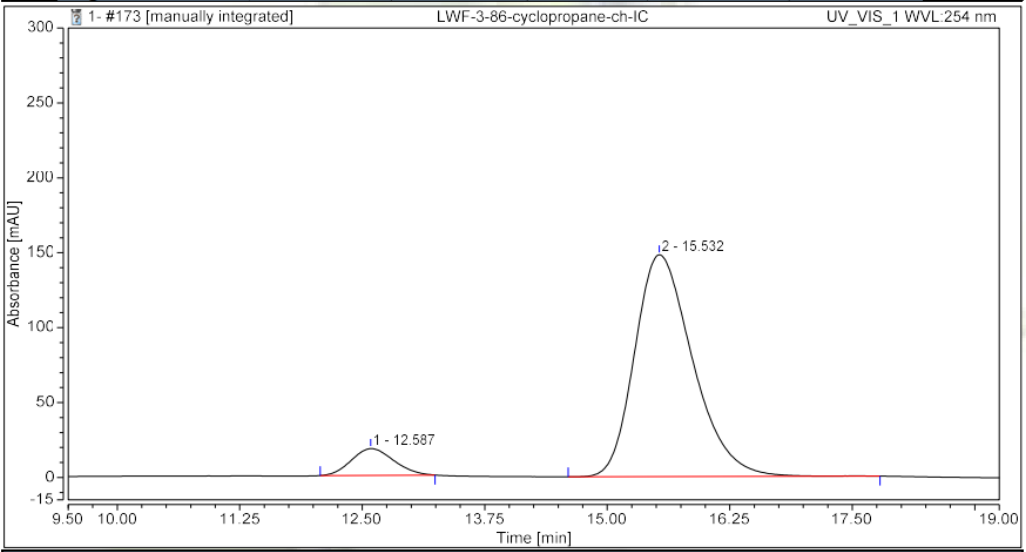

Integration Results

No.	Peak Name	Retention Time min	Area mAU*min	Height mAU	Relative Area %	Relative Height %	Amount n.a.
1		12.587	8.922	18.002	8.08	10.84	n.a.
2		15.532	101.531	148.091	91.92	89.16	n.a.
**Total:**			**110.453**	**166.092**	**100.00**	**100.00**	

Compound B12: IC, *i*-PrOH/hexane = 50/50, v = 1.0 mL/min, λ = 254 nm

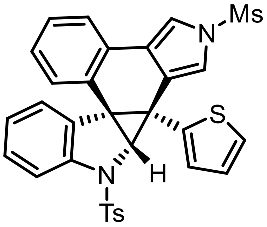

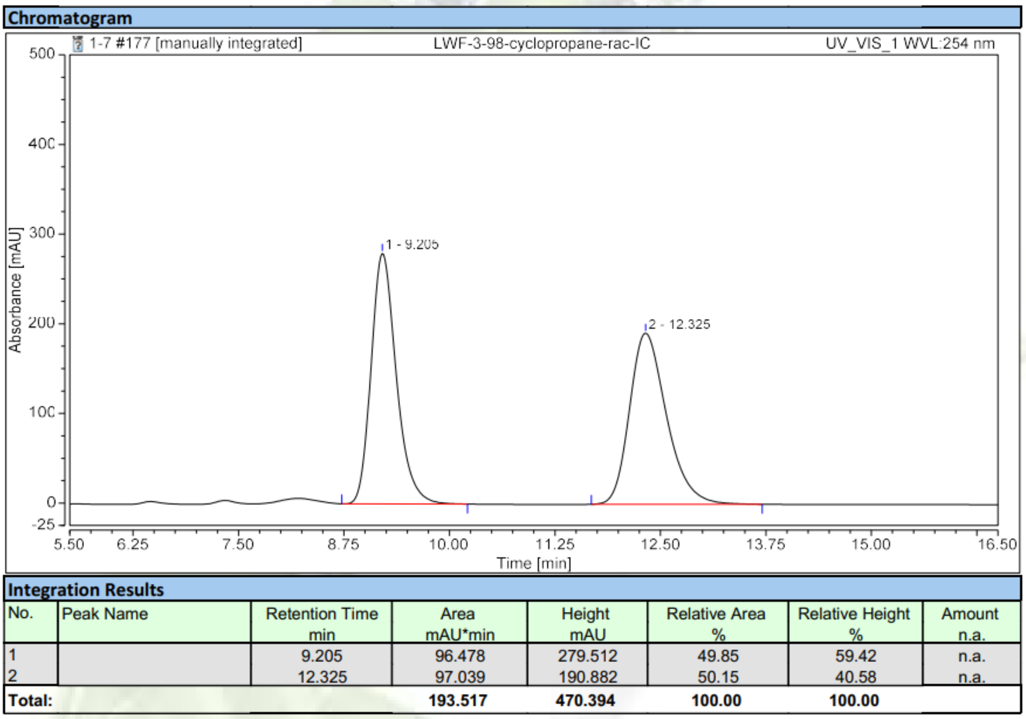

Integration Results

No.	Peak Name	Retention Time min	Area mAU*min	Height mAU	Relative Area %	Relative Height %	Amount n.a.
1		9.205	96.478	279.512	49.85	59.42	n.a.
2		12.325	97.039	190.882	50.15	40.58	n.a.
**Total:**			**193.517**	**470.394**	**100.00**	**100.00**	

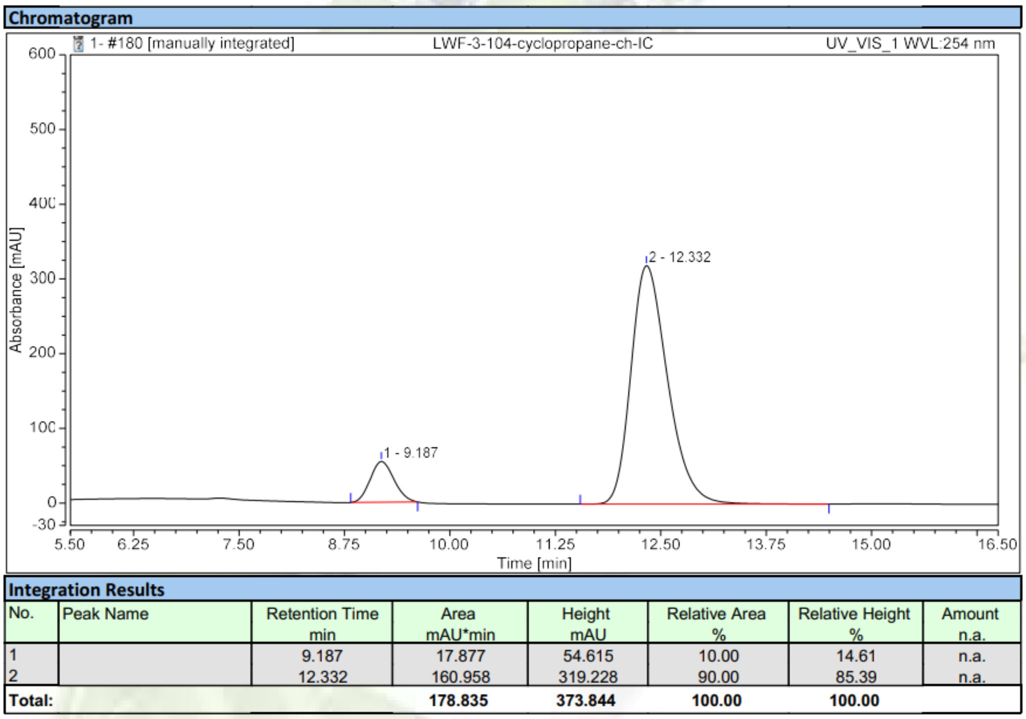

Integration Results

No.	Peak Name	Retention Time min	Area mAU*min	Height mAU	Relative Area %	Relative Height %	Amount n.a.
1		9.187	17.877	54.615	10.00	14.61	n.a.
2		12.332	160.958	319.228	90.00	85.39	n.a.
**Total:**			**178.835**	**373.844**	**100.00**	**100.00**	

Compound B13: IA, *i*-PrOH/hexane = 10/90, v = 1.0 mL/min, λ = 254 nm

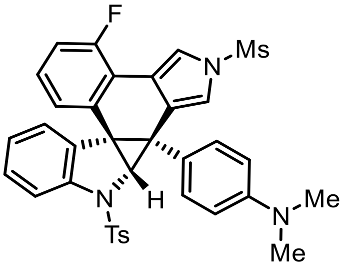

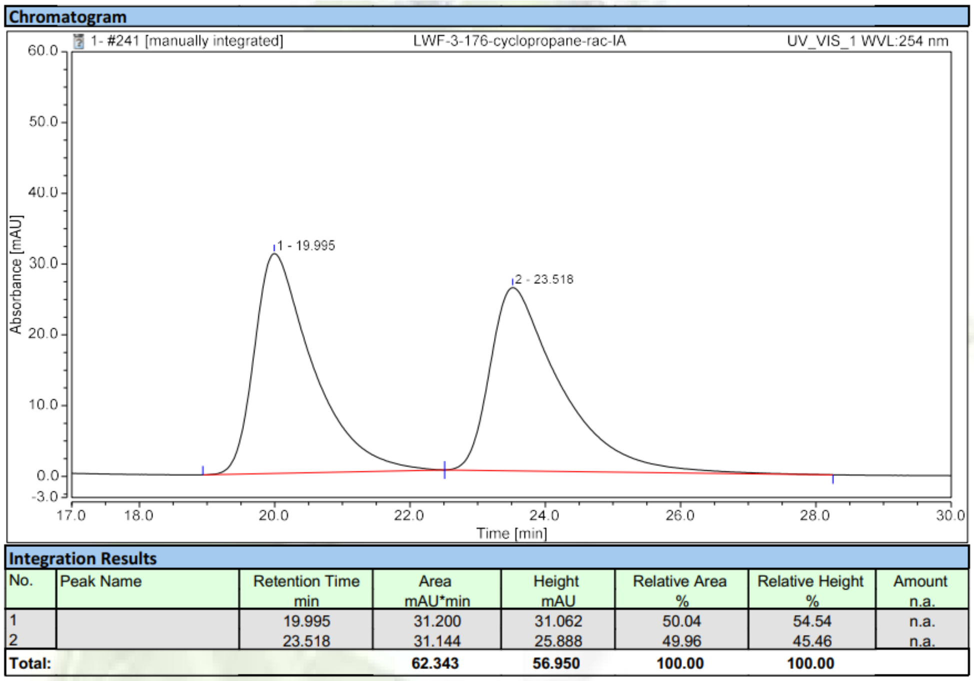

Integration Results

No.	Peak Name	Retention Time min	Area mAU*min	Height mAU	Relative Area %	Relative Height %	Amount n.a.
1		19.995	31.200	31.062	50.04	54.54	n.a.
2		23.518	31.144	25.888	49.96	45.46	n.a.
**Total:**			**62.343**	**56.950**	**100.00**	**100.00**	

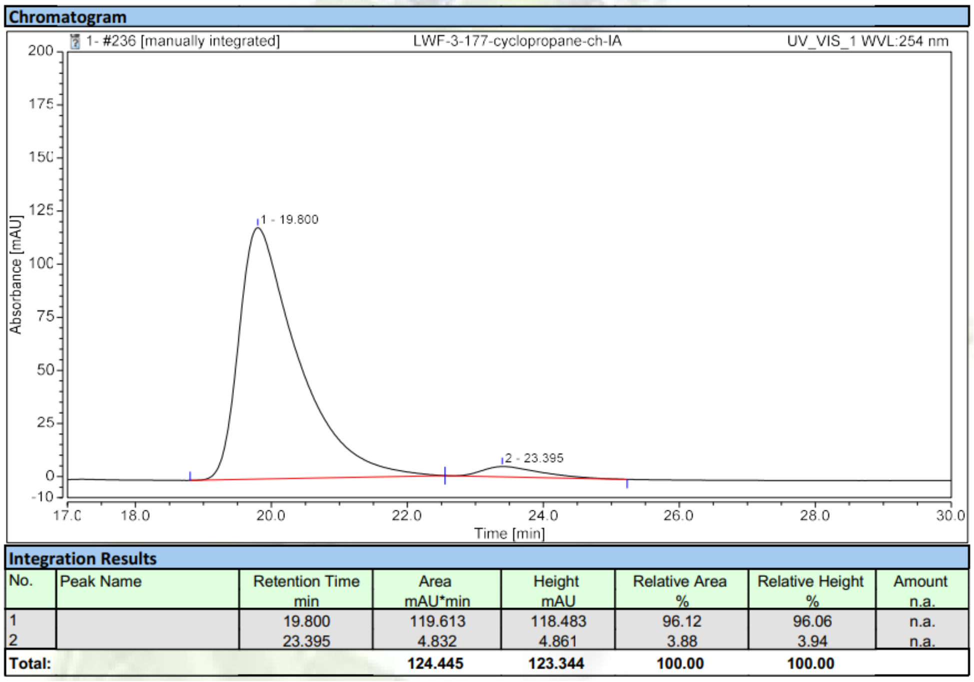

Integration Results

No.	Peak Name	Retention Time min	Area mAU*min	Height mAU	Relative Area %	Relative Height %	Amount n.a.
1		19.800	119.613	118.483	96.12	96.06	n.a.
2		23.395	4.832	4.861	3.88	3.94	n.a.
**Total:**			**124.445**	**123.344**	**100.00**	**100.00**	

Compound B14: IC, *i*-PrOH/hexane = 50/50, v = 1.0 mL/min, λ = 254 nm

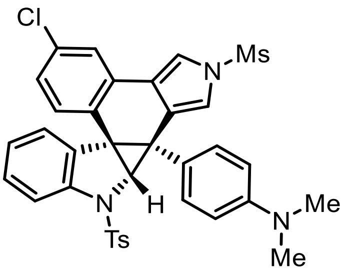

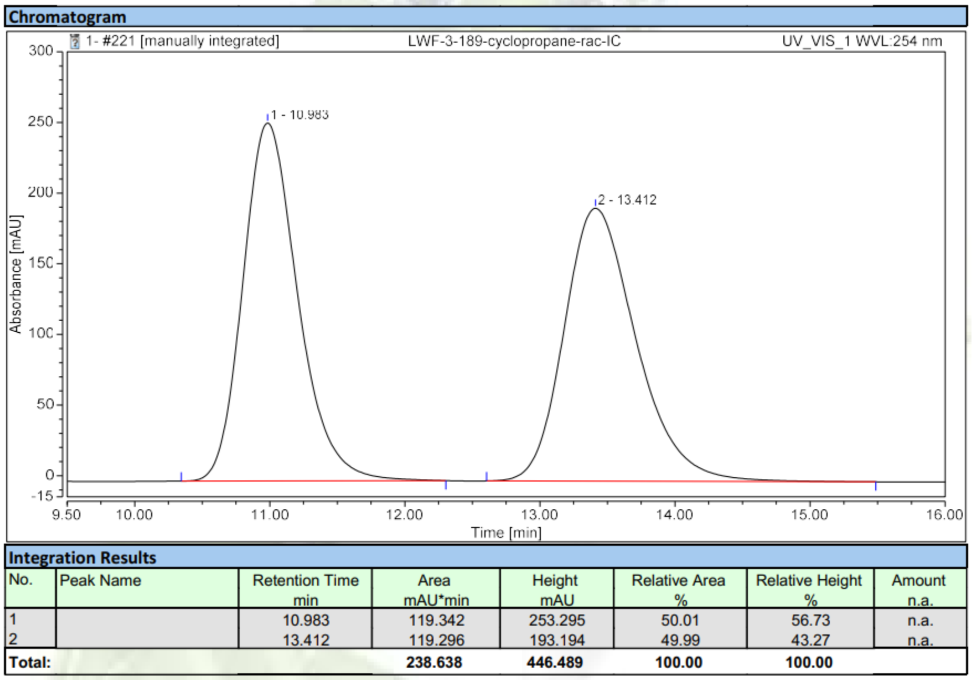

Integration Results

No.	Peak Name	Retention Time<br>min	Area<br>mAU*min	Height<br>mAU	Relative Area<br>%	Relative Height<br>%	Amount<br>n.a.
1		10.983	119.342	253.295	50.01	56.73	n.a.
2		13.412	119.296	193.194	49.99	43.27	n.a.
**Total:**			**238.638**	**446.489**	**100.00**	**100.00**	

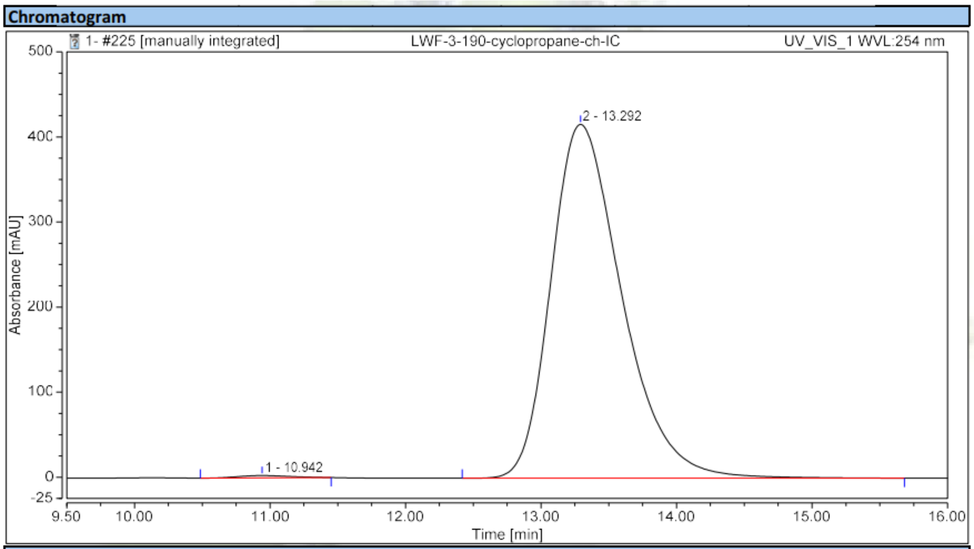

Integration Results

No.	Peak Name	Retention Time<br>min	Area<br>mAU*min	Height<br>mAU	Relative Area<br>%	Relative Height<br>%	Amount<br>n.a.
1		10.942	1.213	2.754	0.48	0.66	n.a.
2		13.292	252.492	415.965	99.52	99.34	n.a.
**Total:**			**253.705**	**418.718**	**100.00**	**100.00**	

Compound B15: IC, *i*-PrOH/hexane = 50/50, v = 1.0 mL/min, λ = 254 nm

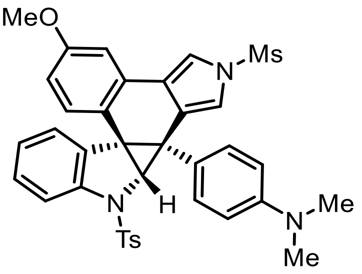

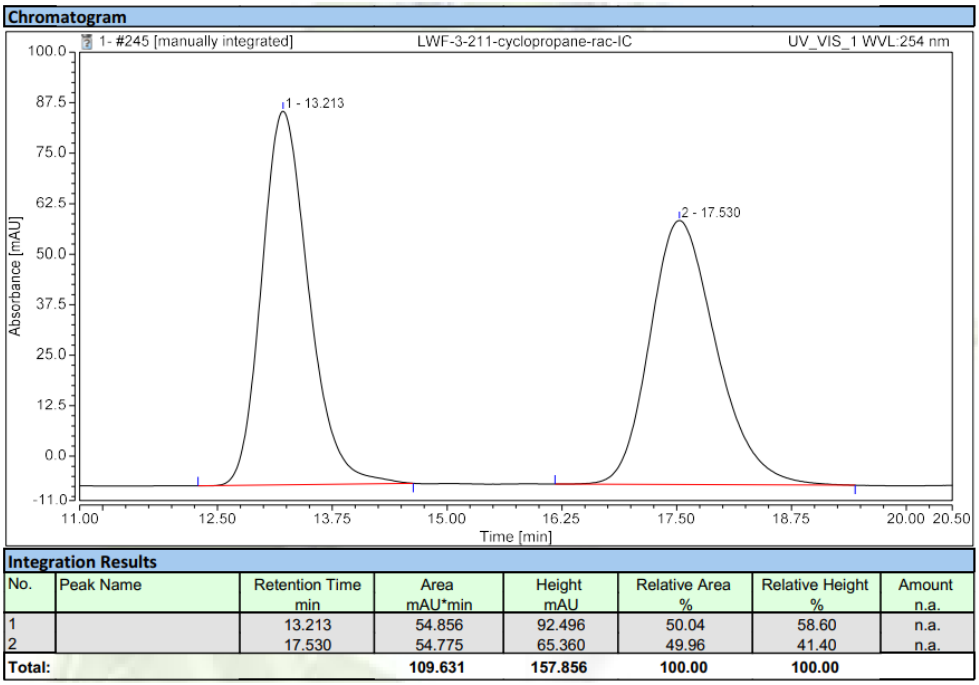

Integration Results

No.	Peak Name	Retention Time min	Area mAU*min	Height mAU	Relative Area %	Relative Height %	Amount n.a.
1		13.213	54.856	92.496	50.04	58.60	n.a.
2		17.530	54.775	65.360	49.96	41.40	n.a.
**Total:**			**109.631**	**157.856**	**100.00**	**100.00**	

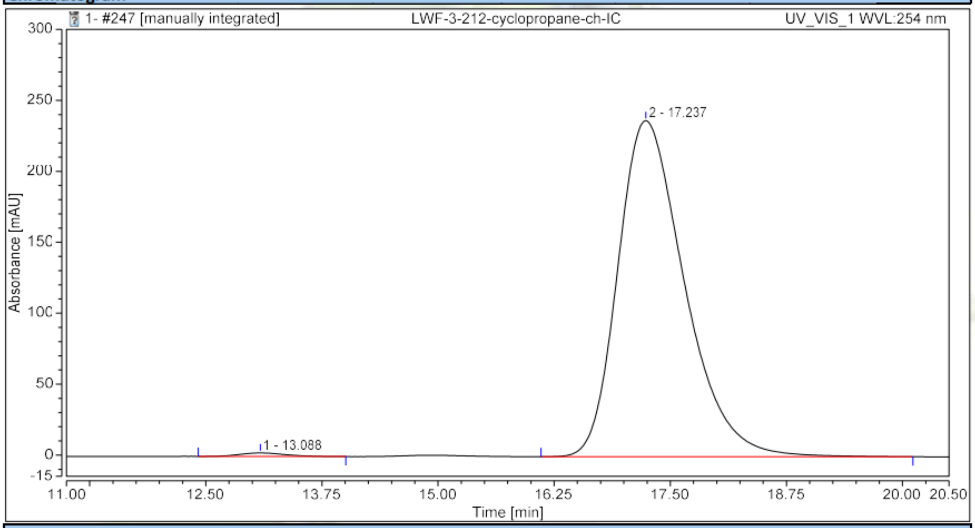

Integration Results

No.	Peak Name	Retention Time min	Area mAU*min	Height mAU	Relative Area %	Relative Height %	Amount n.a.
1		13.088	1.392	2.443	0.70	1.02	n.a.
2		17.237	196.611	236.848	99.30	98.98	n.a.
**Total:**			**198.003**	**239.292**	**100.00**	**100.00**	

Compound B16: IA, *i*-PrOH/hexane = 50/50, v = 1.0 mL/min, λ = 254 nm

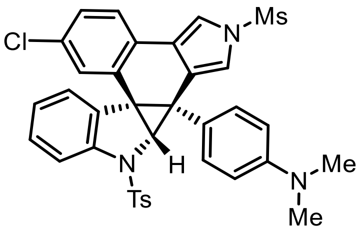

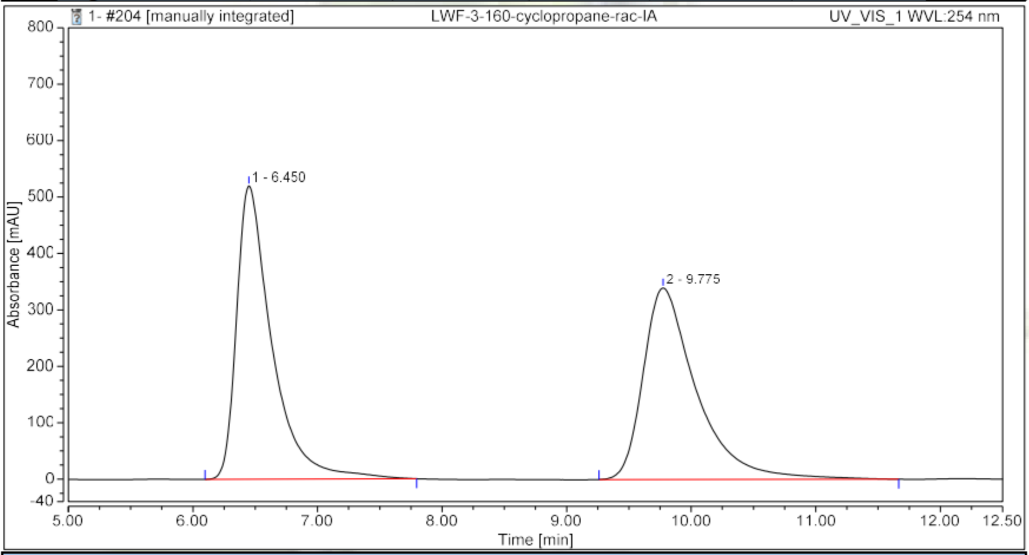

Integration Results

No.	Peak Name	Retention Time min	Area mAU*min	Height mAU	Relative Area %	Relative Height %	Amount n.a.
1		6.450	170.693	520.117	50.38	60.52	n.a.
2		9.775	168.091	339.308	49.62	39.48	n.a.
**Total:**			**338.784**	**859.426**	**100.00**	**100.00**	

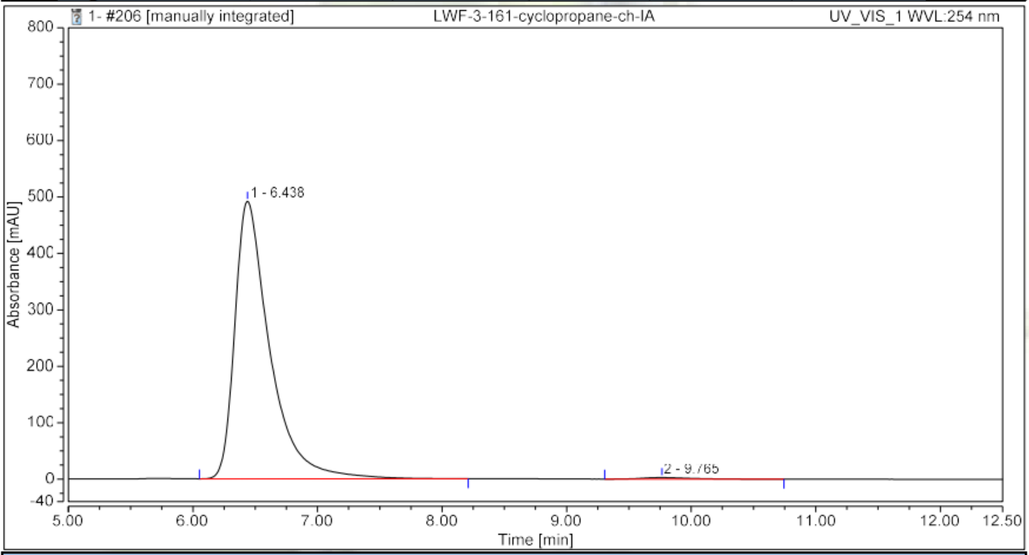

Integration Results

No.	Peak Name	Retention Time min	Area mAU*min	Height mAU	Relative Area %	Relative Height %	Amount n.a.
1		6.438	160.058	492.140	99.22	99.46	n.a.
2		9.765	1.251	2.690	0.78	0.54	n.a.
**Total:**			**161.309**	**494.830**	**100.00**	**100.00**	

Compound B17: IC, *i*-PrOH/hexane = 50/50, v = 1.0 mL/min, λ = 254 nm

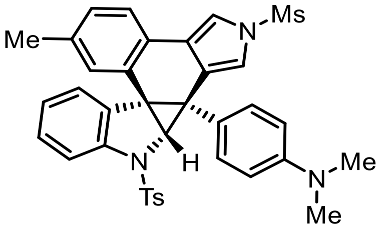

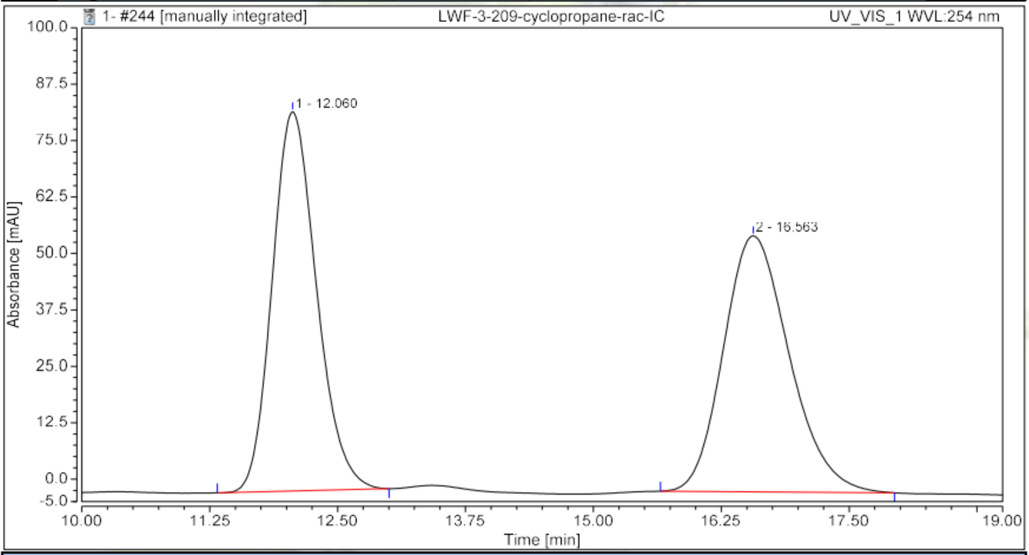

Integration Results

No.	Peak Name	Retention Time min	Area mAU*min	Height mAU	Relative Area %	Relative Height %	Amount n.a.
1		12.060	42.210	84.015	49.94	59.70	n.a.
2		16.563	42.307	56.714	50.06	40.30	n.a.
Total:			84.517	140.729	100.00	100.00	

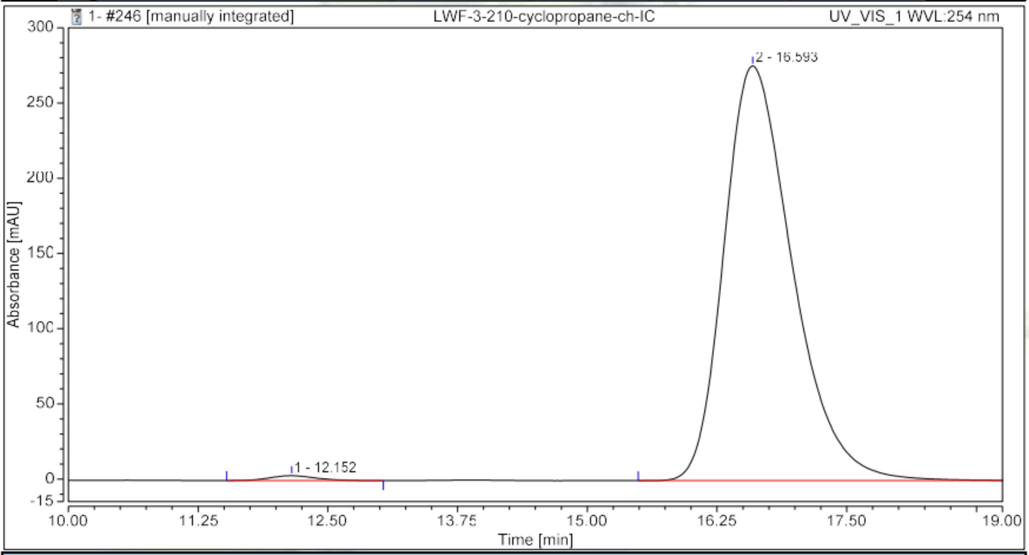

Integration Results

No.	Peak Name	Retention Time min	Area mAU*min	Height mAU	Relative Area %	Relative Height %	Amount n.a.
1		12.152	1.670	3.233	0.79	1.16	n.a.
2		16.593	210.761	275.644	99.21	98.84	n.a.
Total:			212.431	278.876	100.00	100.00	

Compound B18: IA, *i*-PrOH/hexane = 50/50, v = 1.0 mL/min, λ = 254 nm

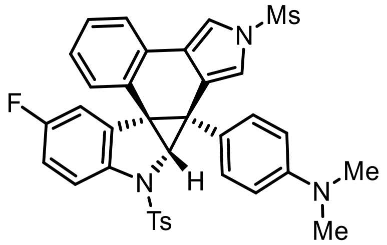

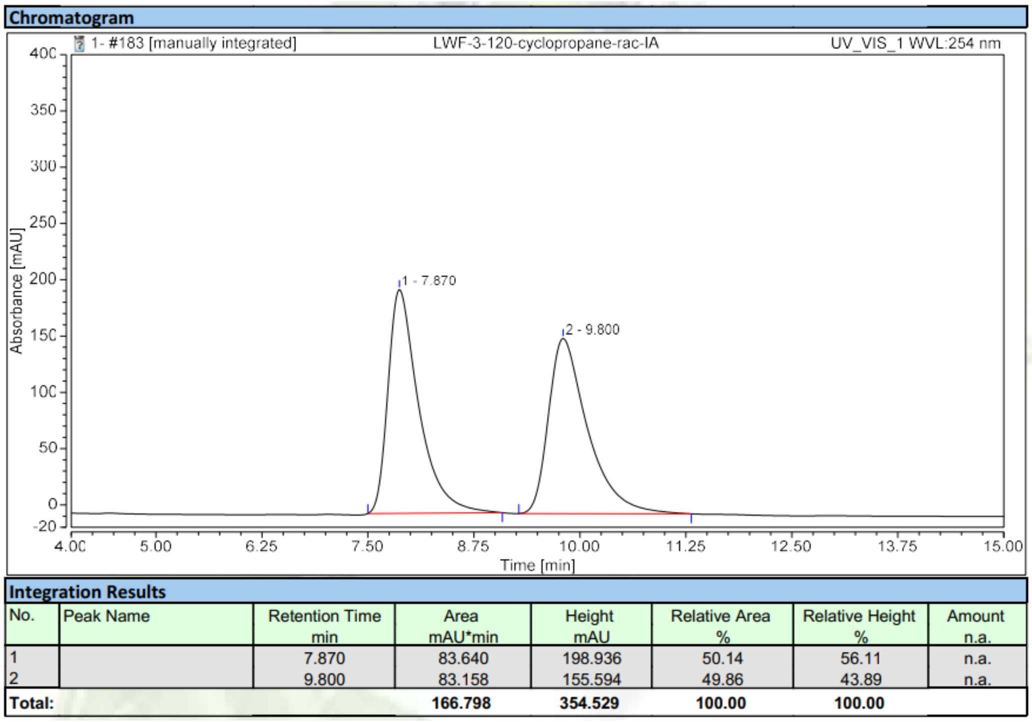

Integration Results

No.	Peak Name	Retention Time min	Area mAU*min	Height mAU	Relative Area %	Relative Height %	Amount n.a.
1		7.870	83.640	198.936	50.14	56.11	n.a.
2		9.800	83.158	155.594	49.86	43.89	n.a.
**Total:**			**166.798**	**354.529**	**100.00**	**100.00**	

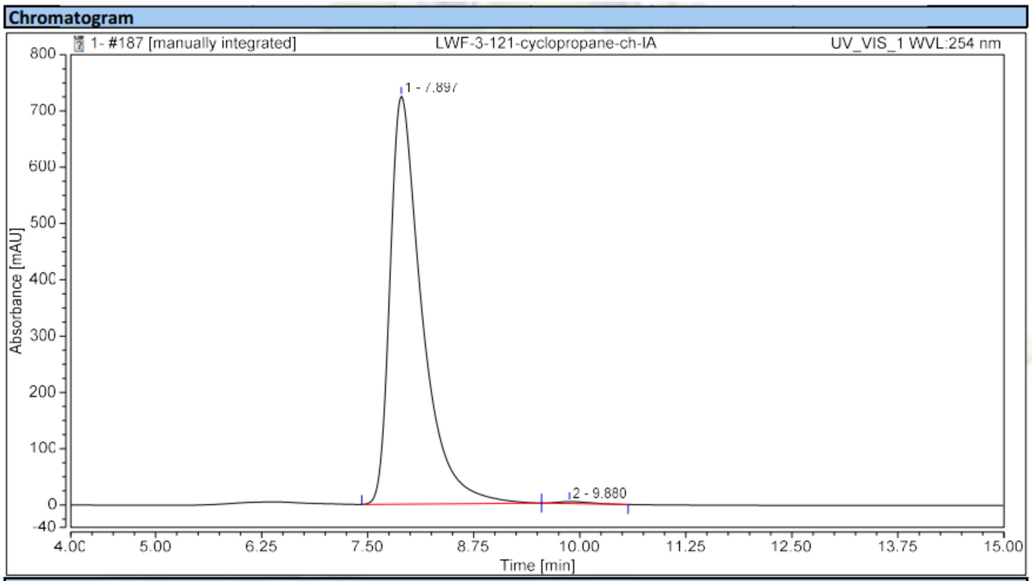

Integration Results

No.	Peak Name	Retention Time min	Area mAU*min	Height mAU	Relative Area %	Relative Height %	Amount n.a.
1		7.897	315.774	724.689	99.50	99.51	n.a.
2		9.880	1.598	3.543	0.50	0.49	n.a.
**Total:**			**317.372**	**728.232**	**100.00**	**100.00**	

Compound B19: IB, *i*-PrOH/hexane = 15/85, v = 1.0 mL/min, λ = 254 nm

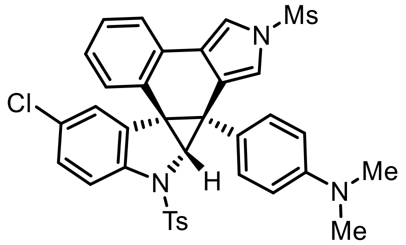

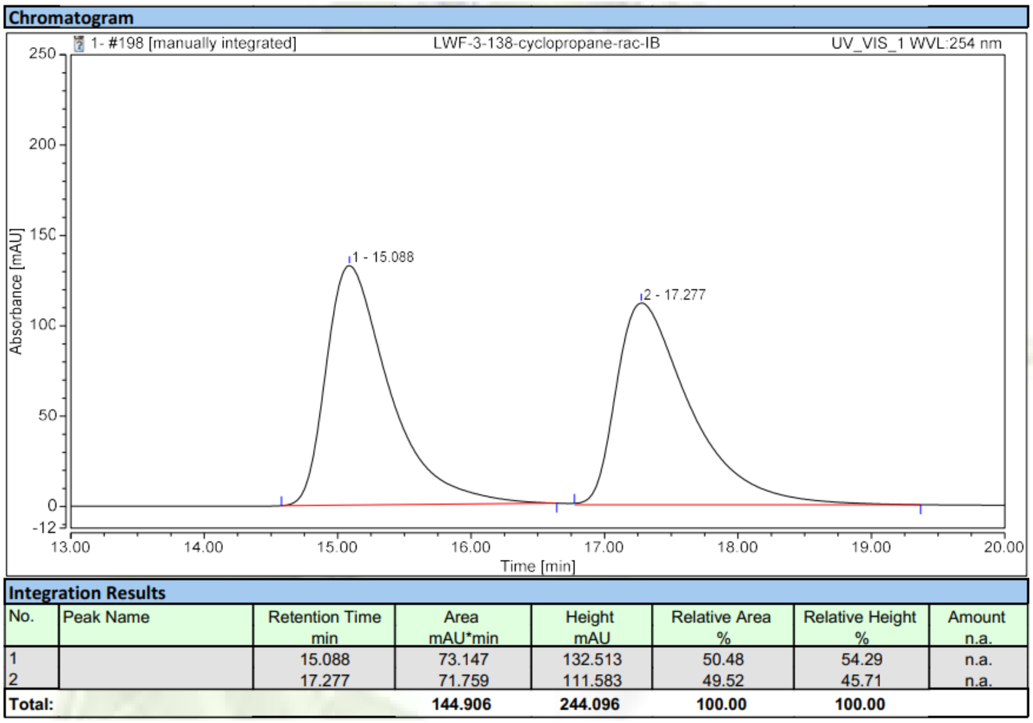

Integration Results

No.	Peak Name	Retention Time min	Area mAU*min	Height mAU	Relative Area %	Relative Height %	Amount n.a.
1		15.088	73.147	132.513	50.48	54.29	n.a.
2		17.277	71.759	111.583	49.52	45.71	n.a.
Total:			144.906	244.096	100.00	100.00	

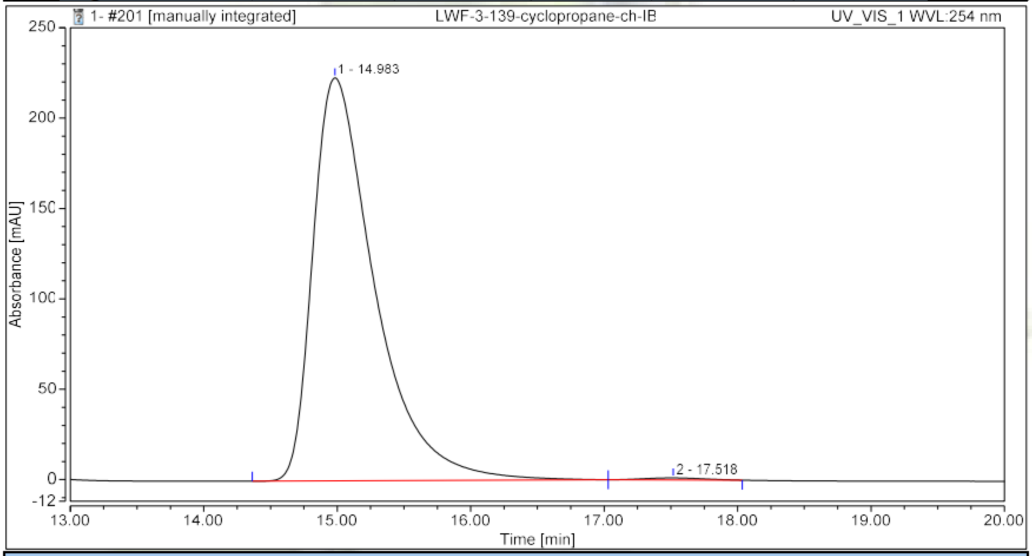

Integration Results

No.	Peak Name	Retention Time min	Area mAU*min	Height mAU	Relative Area %	Relative Height %	Amount n.a.
1		14.983	119.613	222.981	99.54	99.50	n.a.
2		17.518	0.554	1.121	0.46	0.50	n.a.
Total:			120.167	224.102	100.00	100.00	

Compound B20: IA, *i*-PrOH/hexane = 50/50, v = 1.0 mL/min, λ = 254 nm

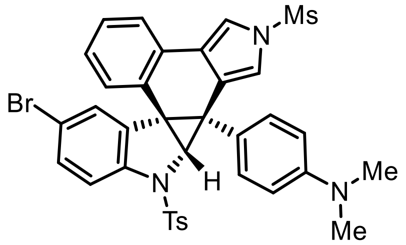

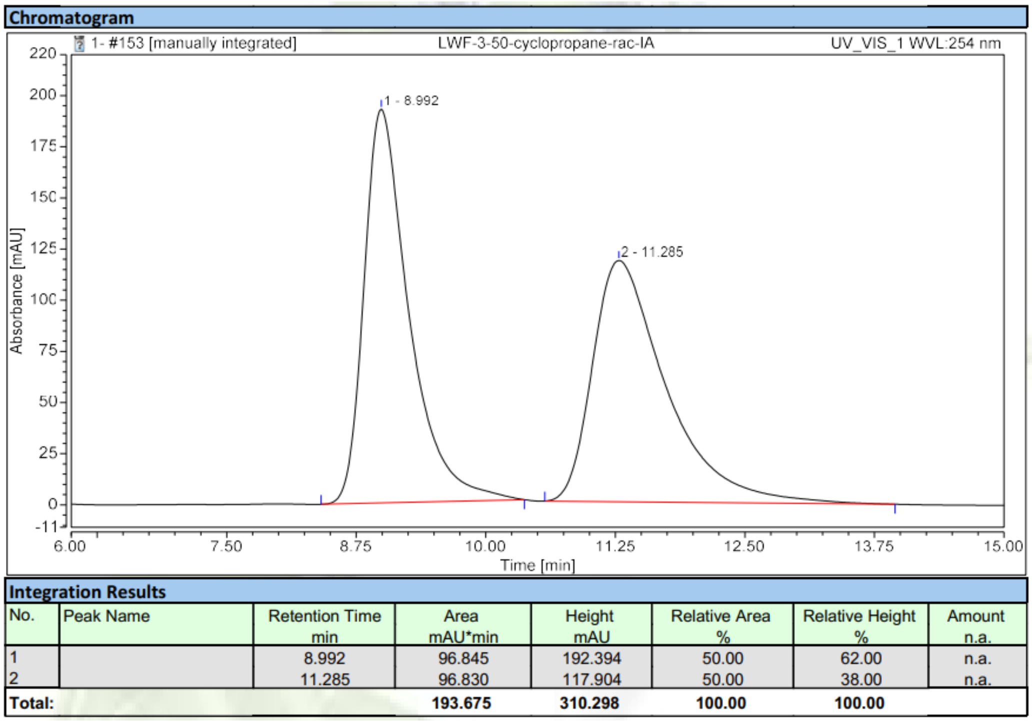

Integration Results

No.	Peak Name	Retention Time min	Area mAU*min	Height mAU	Relative Area %	Relative Height %	Amount n.a.
1		8.992	96.845	192.394	50.00	62.00	n.a.
2		11.285	96.830	117.904	50.00	38.00	n.a.
Total:			193.675	310.298	100.00	100.00	

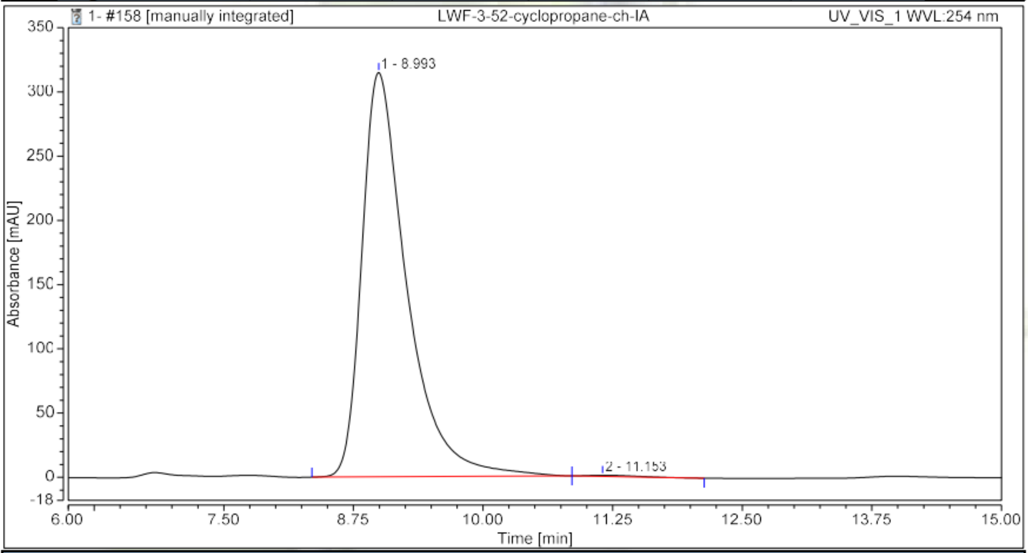

Integration Results

No.	Peak Name	Retention Time min	Area mAU*min	Height mAU	Relative Area %	Relative Height %	Amount n.a.
1		8.993	157.745	314.752	99.69	99.72	n.a.
2		11.153	0.489	0.871	0.31	0.28	n.a.
Total:			158.234	315.623	100.00	100.00	

Compound B21: IA, *i*-PrOH/hexane = 50/50, v = 1.0 mL/min, λ = 254 nm

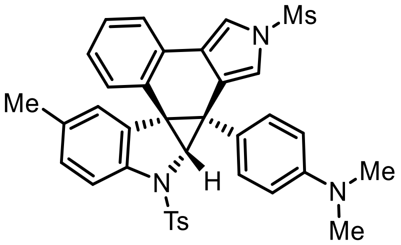

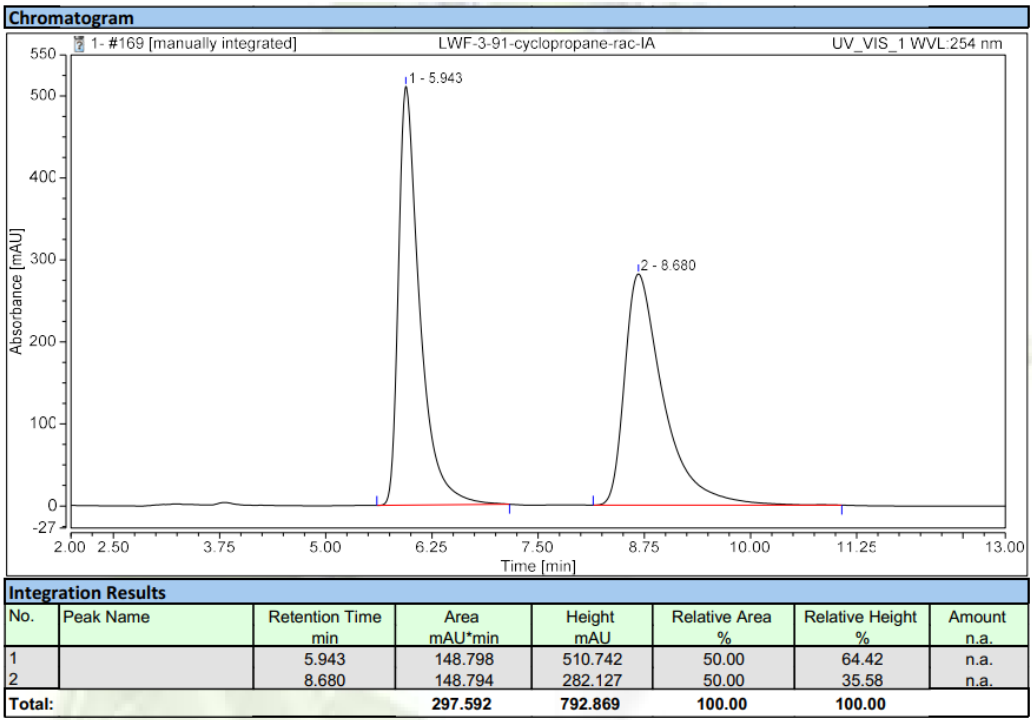

Integration Results

No.	Peak Name	Retention Time min	Area mAU*min	Height mAU	Relative Area %	Relative Height %	Amount n.a.
1		5.943	148.798	510.742	50.00	64.42	n.a.
2		8.680	148.794	282.127	50.00	35.58	n.a.
Total:			297.592	792.869	100.00	100.00	

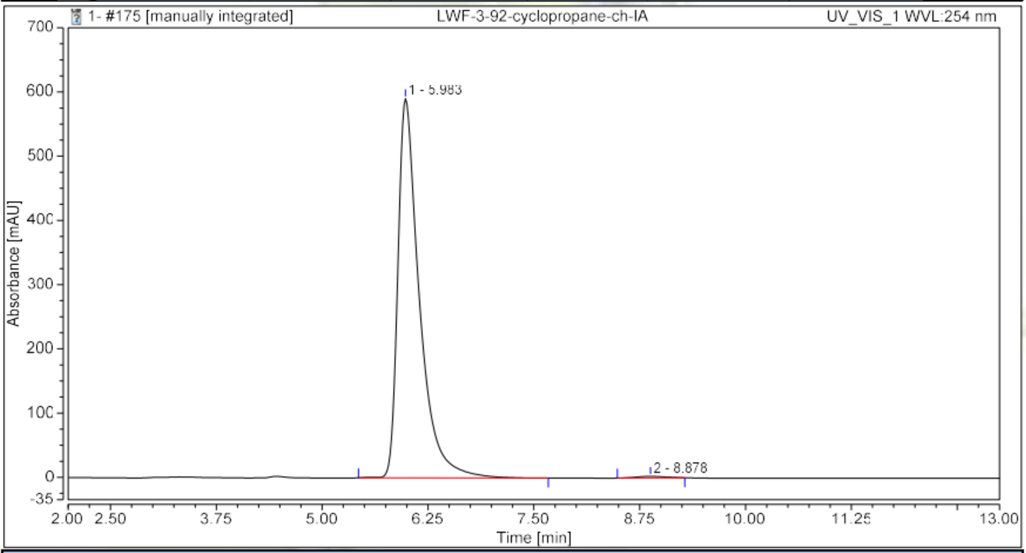

Integration Results

No.	Peak Name	Retention Time min	Area mAU*min	Height mAU	Relative Area %	Relative Height %	Amount n.a.
1		5.983	174.179	590.391	99.44	99.60	n.a.
2		8.878	0.977	2.379	0.56	0.40	n.a.
Total:			175.157	592.769	100.00	100.00	

Compound B22: IA, *i*-PrOH/hexane = 30/70, v = 1.0 mL/min, λ = 254 nm

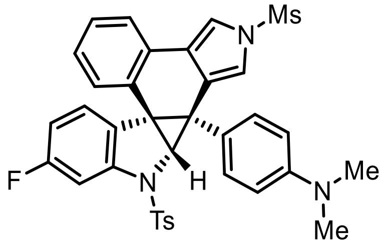

Chromatogram

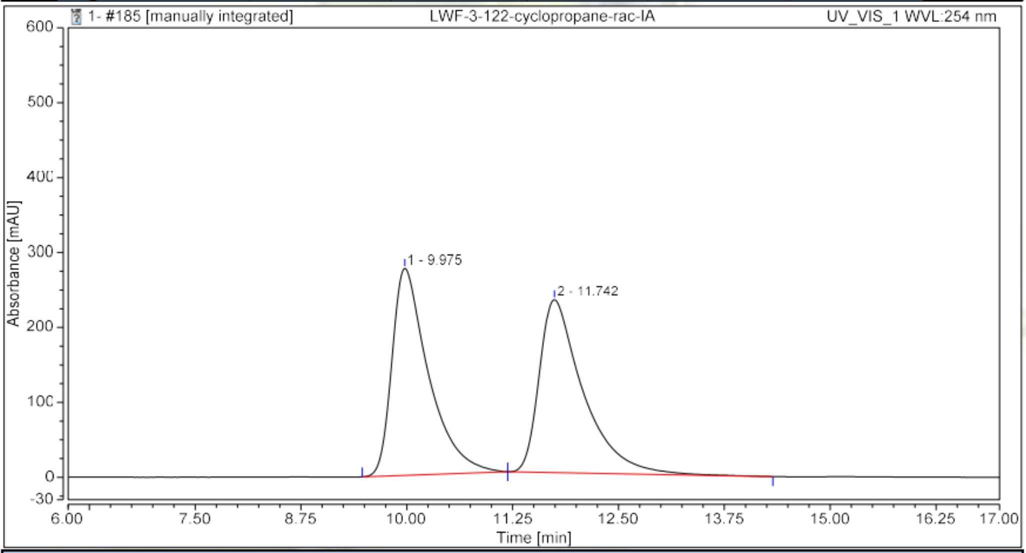

Integration Results

No.	Peak Name	Retention Time min	Area mAU*min	Height mAU	Relative Area %	Relative Height %	Amount n.a.
1		9.975	138.402	276.381	50.00	54.49	n.a.
2		11.742	138.406	230.797	50.00	45.51	n.a.
**Total:**			**276.808**	**507.178**	**100.00**	**100.00**	

Chromatogram

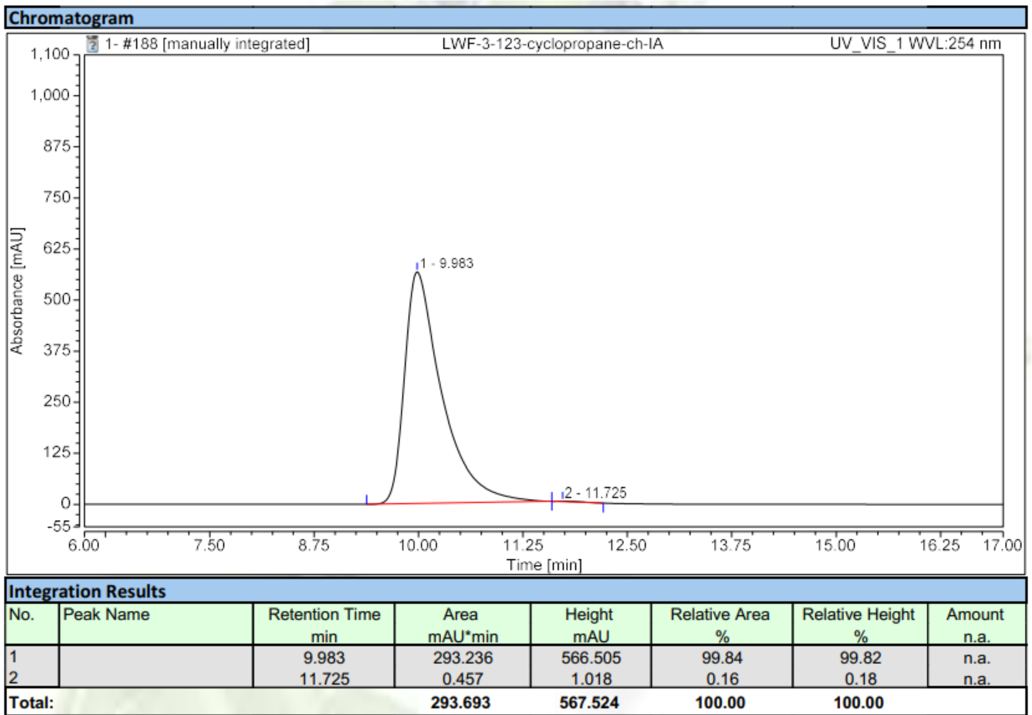

Integration Results

No.	Peak Name	Retention Time min	Area mAU*min	Height mAU	Relative Area %	Relative Height %	Amount n.a.
1		9.983	293.236	566.505	99.84	99.82	n.a.
2		11.725	0.457	1.018	0.16	0.18	n.a.
**Total:**			**293.693**	**567.524**	**100.00**	**100.00**	

Compound B23: IB, *i*-PrOH/hexane = 15/85, v = 1.0 mL/min, λ = 254 nm

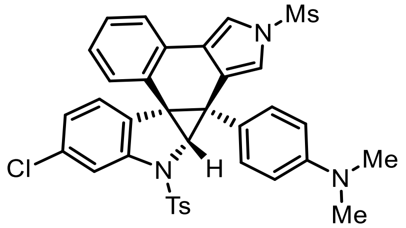

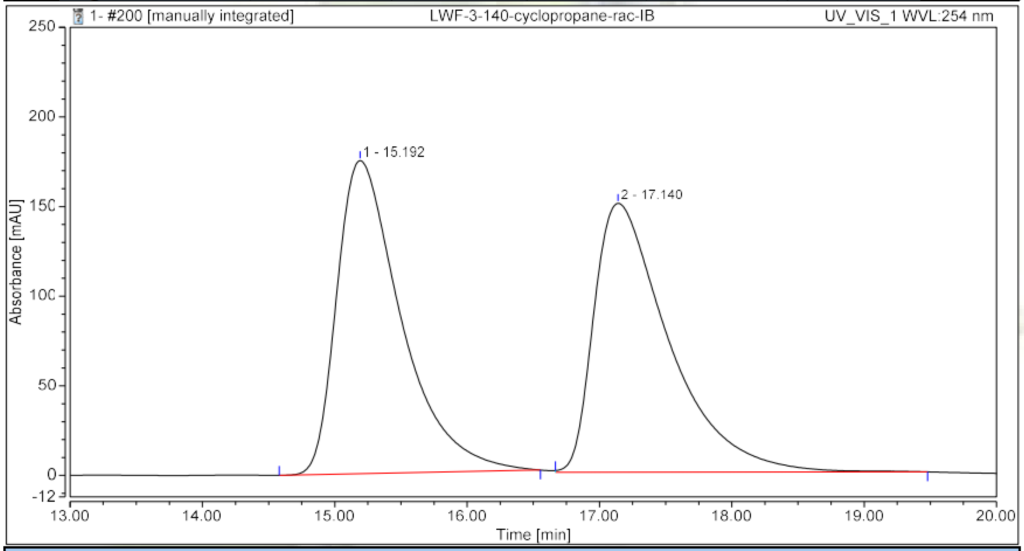

Integration Results

No.	Peak Name	Retention Time min	Area mAU*min	Height mAU	Relative Area %	Relative Height %	Amount n.a.
1		15.192	98.356	174.703	49.97	53.80	n.a.
2		17.140	98.481	150.015	50.03	46.20	n.a.
**Total:**			**196.837**	**324.718**	**100.00**	**100.00**	

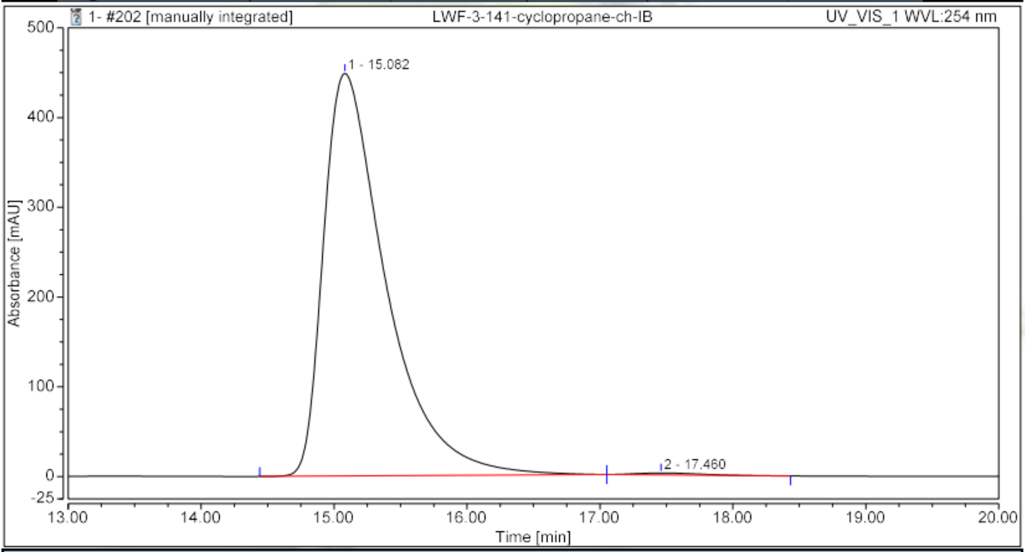

Integration Results

No.	Peak Name	Retention Time min	Area mAU*min	Height mAU	Relative Area %	Relative Height %	Amount n.a.
1		15.082	243.961	448.415	99.51	99.52	n.a.
2		17.460	1.198	2.161	0.49	0.48	n.a.
**Total:**			**245.159**	**450.576**	**100.00**	**100.00**	

Compound B24: IC, *i*-PrOH/hexane = 20/80, v = 1.0 mL/min, λ = 254 nm

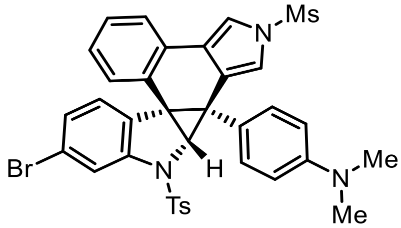

Chromatogram

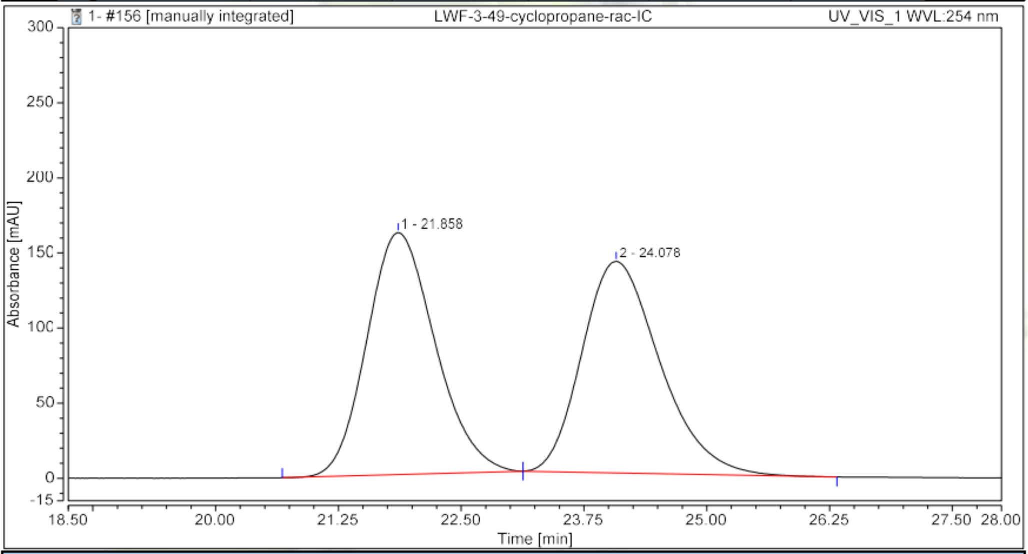

Integration Results

No.	Peak Name	Retention Time min	Area mAU*min	Height mAU	Relative Area %	Relative Height %	Amount n.a.
1		21.858	129.769	161.030	50.07	53.35	n.a.
2		24.078	129.424	140.781	49.93	46.65	n.a.
**Total:**			**259.193**	**301.811**	**100.00**	**100.00**	

Chromatogram

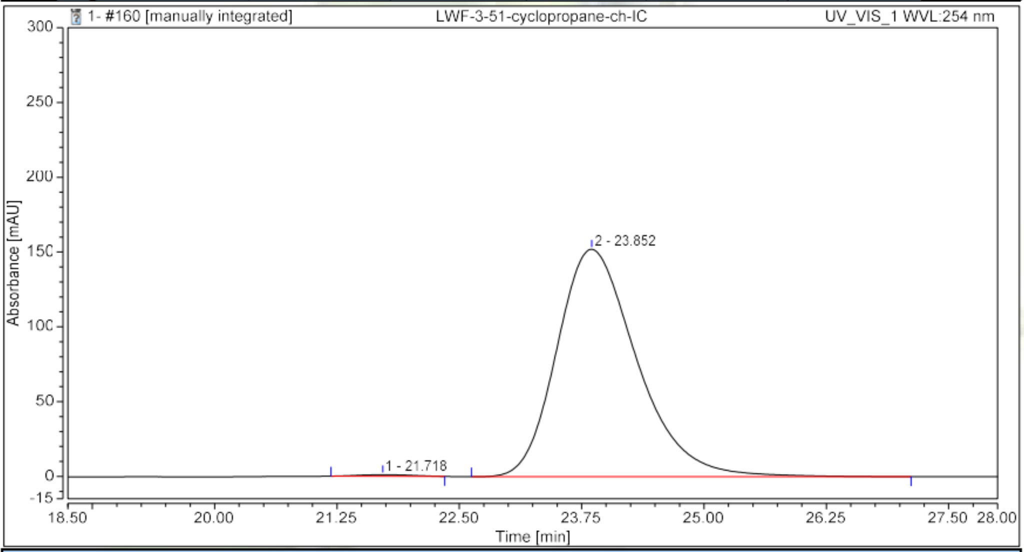

Integration Results

No.	Peak Name	Retention Time min	Area mAU*min	Height mAU	Relative Area %	Relative Height %	Amount n.a.
1		21.718	0.678	1.065	0.47	0.70	n.a.
2		23.852	144.185	152.037	99.53	99.30	n.a.
**Total:**			**144.863**	**153.103**	**100.00**	**100.00**	

Compound B25: IA, *i*-PrOH/hexane = 30/70, v = 1.0 mL/min, λ = 254 nm

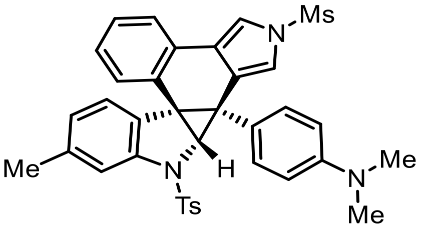

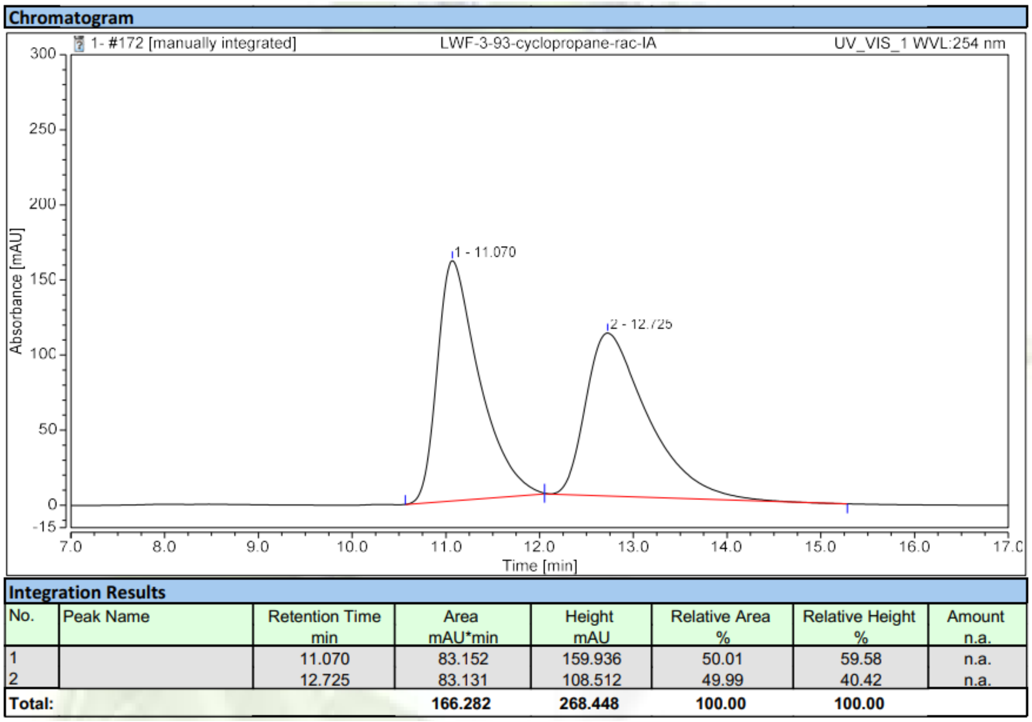

Integration Results

No.	Peak Name	Retention Time<br>min	Area<br>mAU*min	Height<br>mAU	Relative Area<br>%	Relative Height<br>%	Amount<br>n.a.
1		11.070	83.152	159.936	50.01	59.58	n.a.
2		12.725	83.131	108.512	49.99	40.42	n.a.
**Total:**			**166.282**	**268.448**	**100.00**	**100.00**	

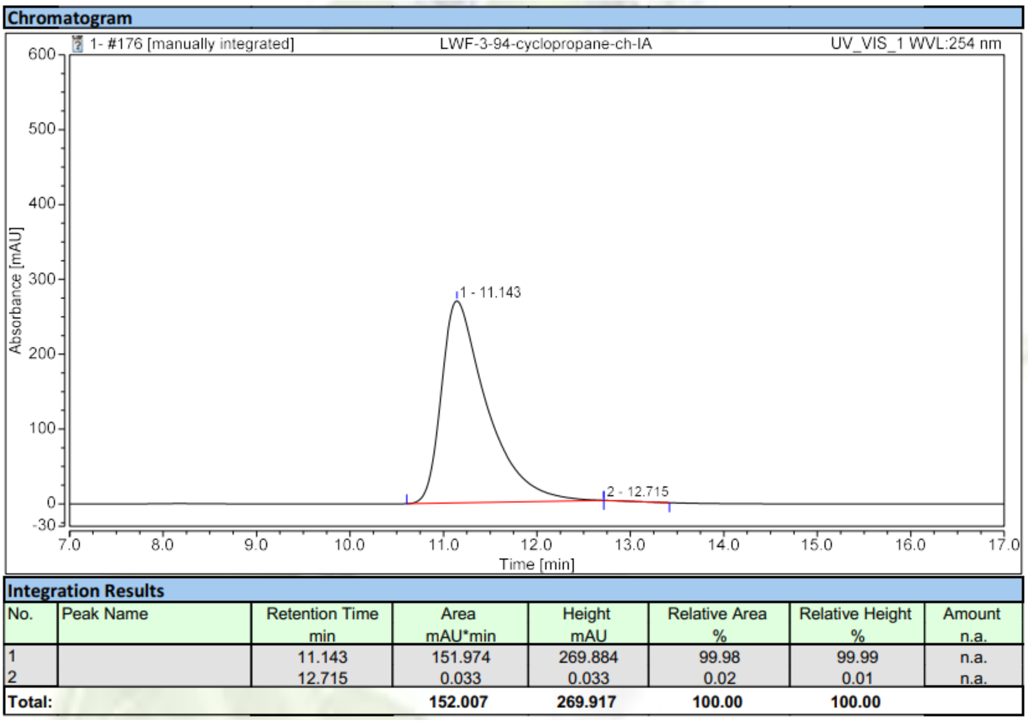

Integration Results

No.	Peak Name	Retention Time<br>min	Area<br>mAU*min	Height<br>mAU	Relative Area<br>%	Relative Height<br>%	Amount<br>n.a.
1		11.143	151.974	269.884	99.98	99.99	n.a.
2		12.715	0.033	0.033	0.02	0.01	n.a.
**Total:**			**152.007**	**269.917**	**100.00**	**100.00**	

Compound B26: IC, *i*-PrOH/hexane = 50/50, v = 1.0 mL/min, λ = 254 nm

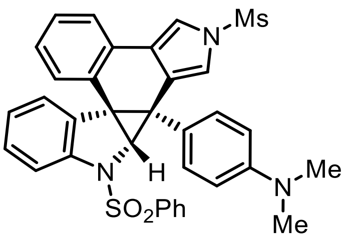

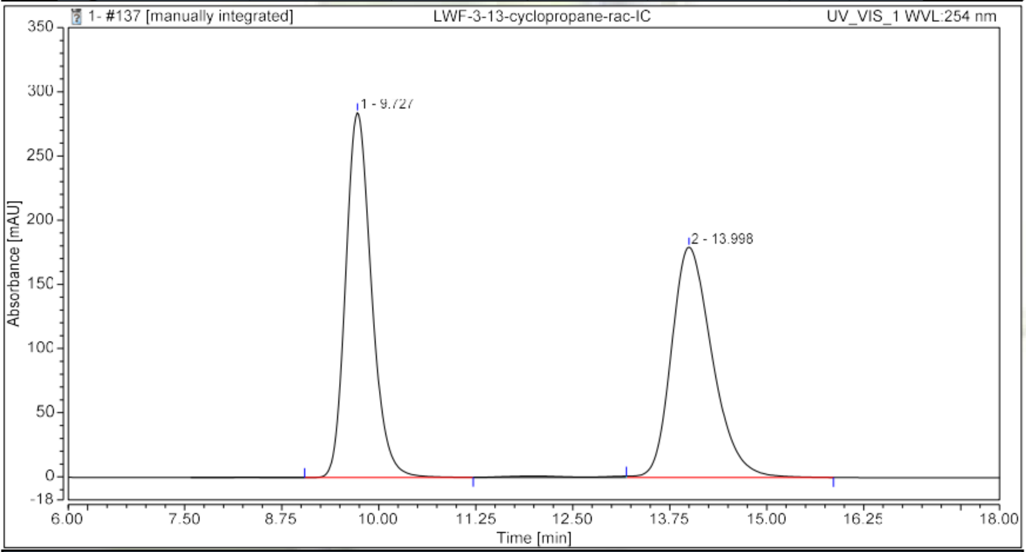

Integration Results

No.	Peak Name	Retention Time min	Area mAU*min	Height mAU	Relative Area %	Relative Height %	Amount n.a.
1		9.727	109.520	284.284	50.00	61.30	n.a.
2		13.998	109.541	179.487	50.00	38.70	n.a.
**Total:**			**219.060**	**463.771**	**100.00**	**100.00**	

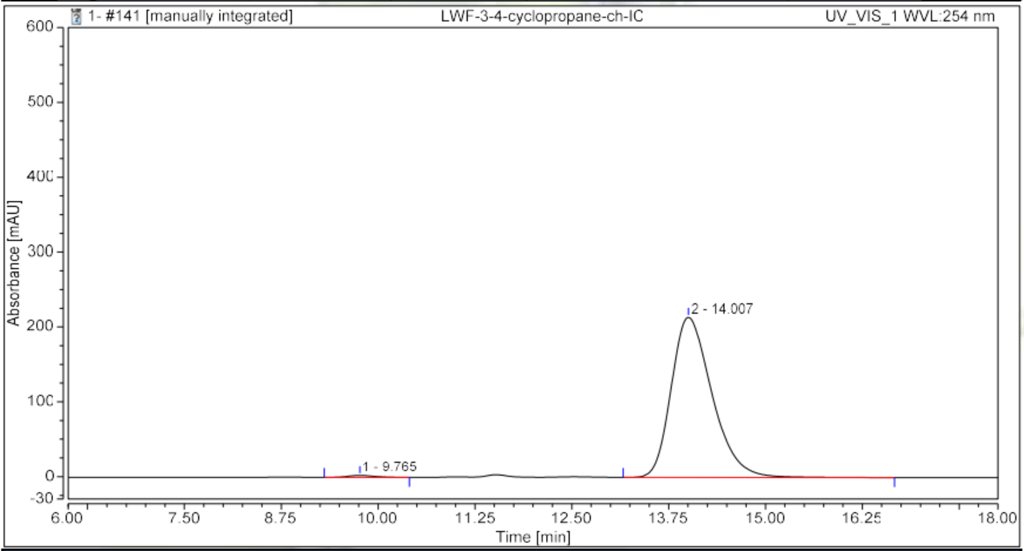

Integration Results

No.	Peak Name	Retention Time min	Area mAU*min	Height mAU	Relative Area %	Relative Height %	Amount n.a.
1		9.765	1.071	2.665	0.81	1.23	n.a.
2		14.007	130.515	213.891	99.19	98.77	n.a.
**Total:**			**131.586**	**216.556**	**100.00**	**100.00**	

Compound B27: IA, *i*-PrOH/hexane = 50/50, v = 1.0 mL/min, λ = 254 nm

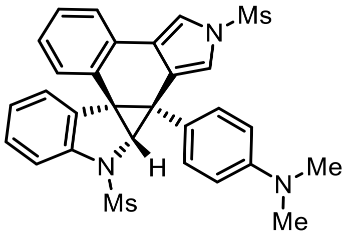

Chromatogram

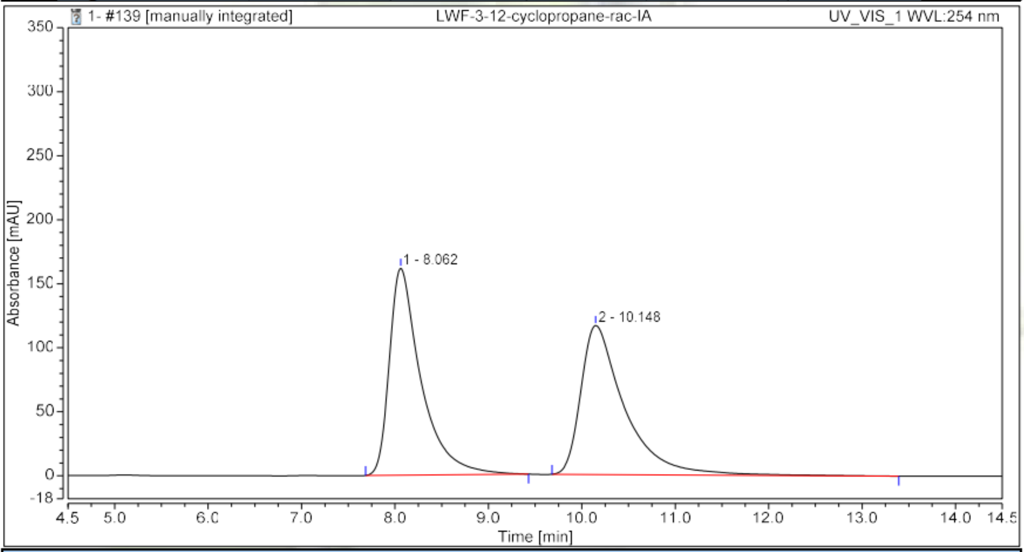

Integration Results

No.	Peak Name	Retention Time min	Area mAU*min	Height mAU	Relative Area %	Relative Height %	Amount n.a.
1		8.062	63.245	161.637	50.06	58.13	n.a.
2		10.148	63.099	116.420	49.94	41.87	n.a.
**Total:**			**126.344**	**278.057**	**100.00**	**100.00**	

Chromatogram

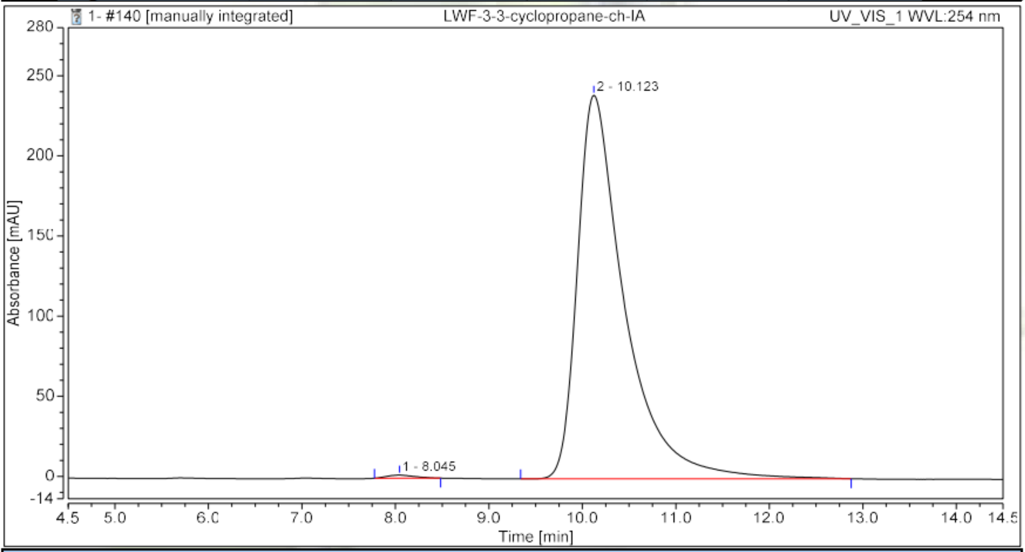

Integration Results

No.	Peak Name	Retention Time min	Area mAU*min	Height mAU	Relative Area %	Relative Height %	Amount n.a.
1		8.045	0.660	1.938	0.47	0.80	n.a.
2		10.123	139.869	239.414	99.53	99.20	n.a.
**Total:**			**140.530**	**241.352**	**100.00**	**100.00**	

Compound B28: IC, *i*-PrOH/hexane = 30/70, v = 1.0 mL/min, λ = 254 nm

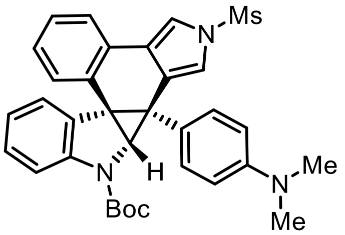

Chromatogram

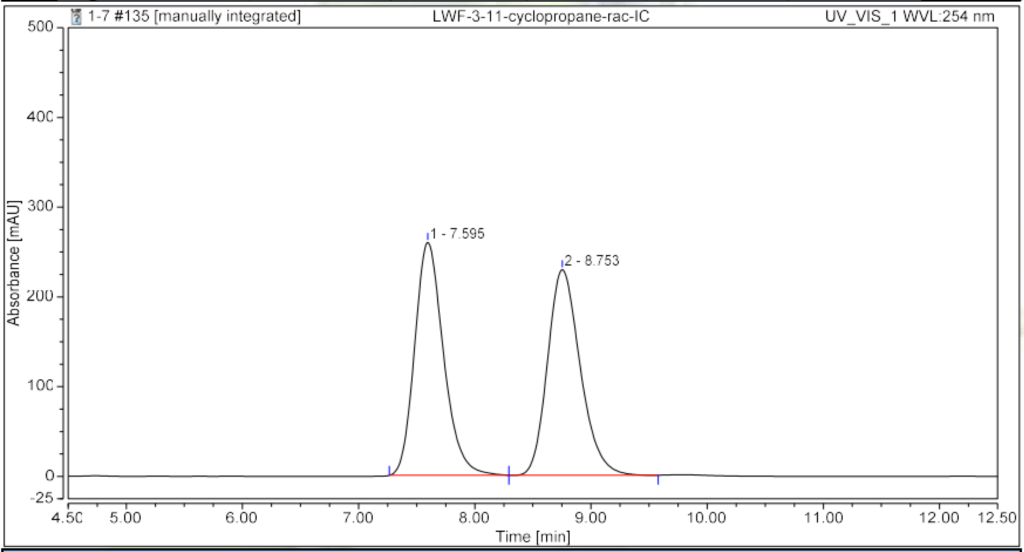

Integration Results

No.	Peak Name	Retention Time min	Area mAU*min	Height mAU	Relative Area %	Relative Height %	Amount n.a.
1		7.595	74.841	259.566	50.02	53.10	n.a.
2		8.753	74.779	229.255	49.98	46.90	n.a.
**Total:**			**149.620**	**488.821**	**100.00**	**100.00**	

Chromatogram

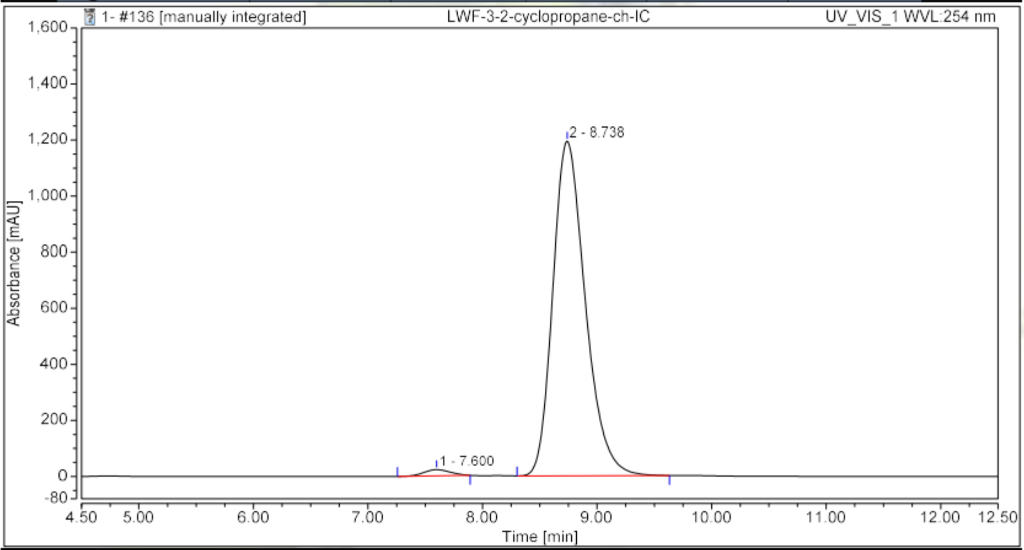

Integration Results

No.	Peak Name	Retention Time min	Area mAU*min	Height mAU	Relative Area %	Relative Height %	Amount n.a.
1		7.600	5.856	21.925	1.47	1.80	n.a.
2		8.738	392.005	1193.587	98.53	98.20	n.a.
**Total:**			**397.860**	**1215.512**	**100.00**	**100.00**	

Compound B29: IA, *i*-PrOH/hexane = 50/50, v = 1.0 mL/min, λ = 254 nm

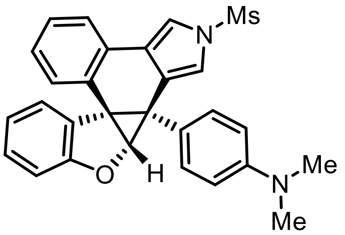

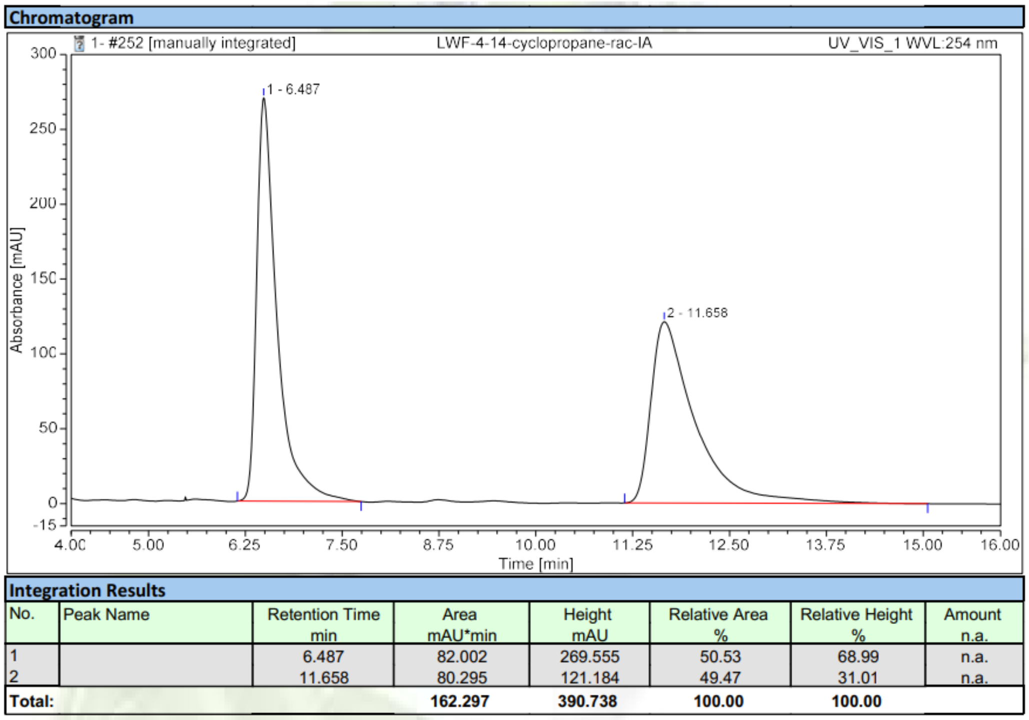

Integration Results

No.	Peak Name	Retention Time min	Area mAU*min	Height mAU	Relative Area %	Relative Height %	Amount n.a.
1		6.487	82.002	269.555	50.53	68.99	n.a.
2		11.658	80.295	121.184	49.47	31.01	n.a.
Total:			162.297	390.738	100.00	100.00	

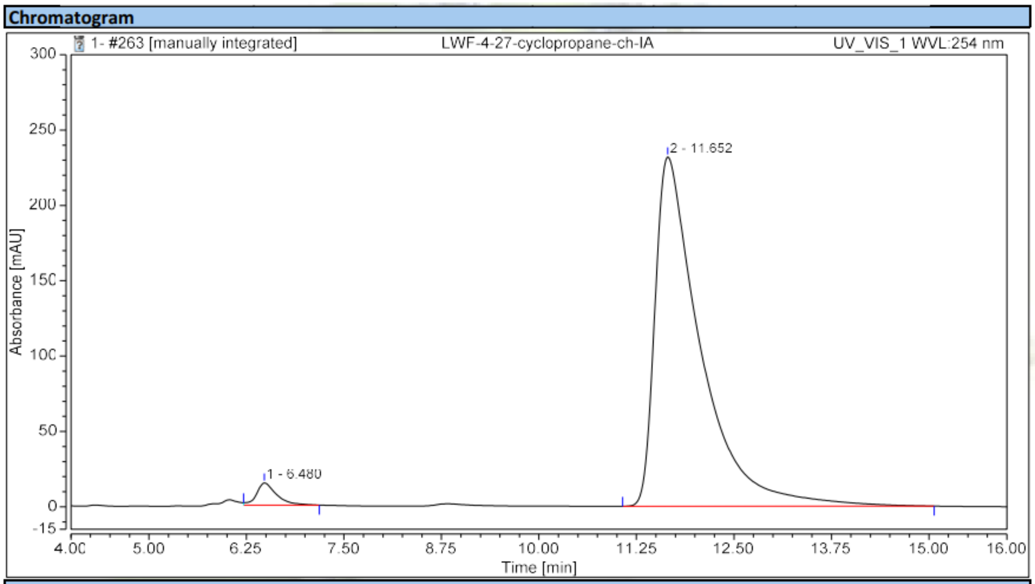

Integration Results

No.	Peak Name	Retention Time min	Area mAU*min	Height mAU	Relative Area %	Relative Height %	Amount n.a.
1		6.480	4.379	14.749	2.72	5.98	n.a.
2		11.652	156.913	231.862	97.28	94.02	n.a.
Total:			161.292	246.611	100.00	100.00	

Compound B30: IC, *i*-PrOH/hexane = 20/80, v = 1.0 mL/min, λ = 254 nm

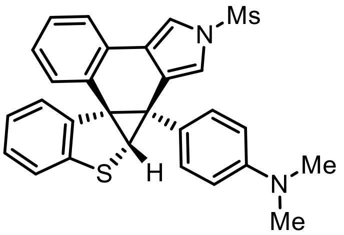

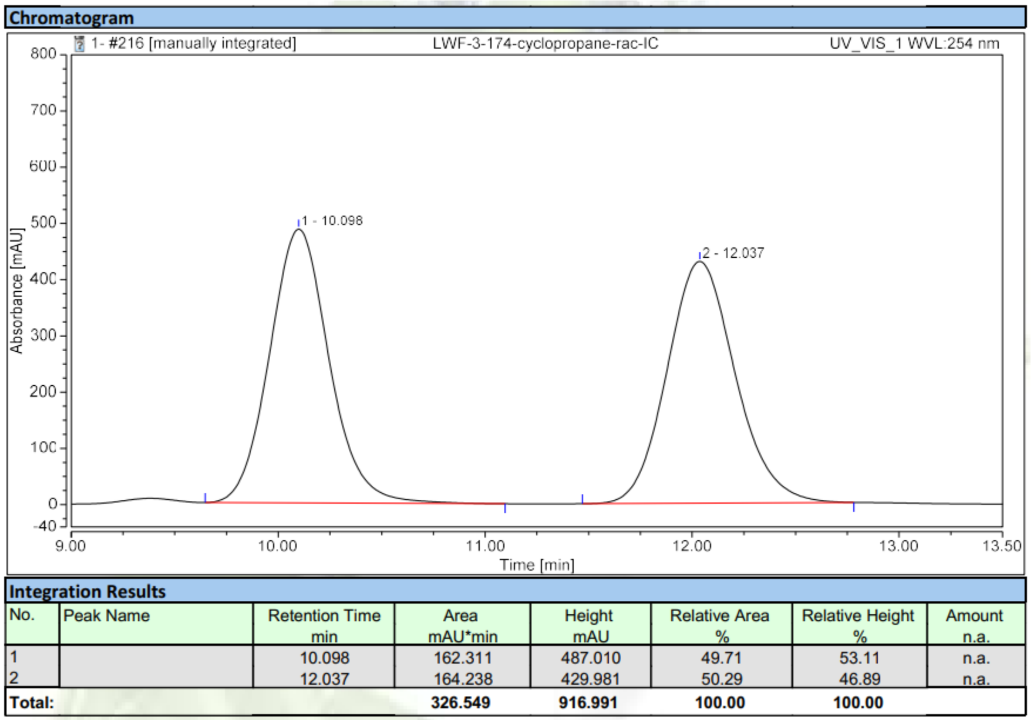

Integration Results

No.	Peak Name	Retention Time min	Area mAU*min	Height mAU	Relative Area %	Relative Height %	Amount n.a.
1		10.098	162.311	487.010	49.71	53.11	n.a.
2		12.037	164.238	429.981	50.29	46.89	n.a.
**Total:**			**326.549**	**916.991**	**100.00**	**100.00**	

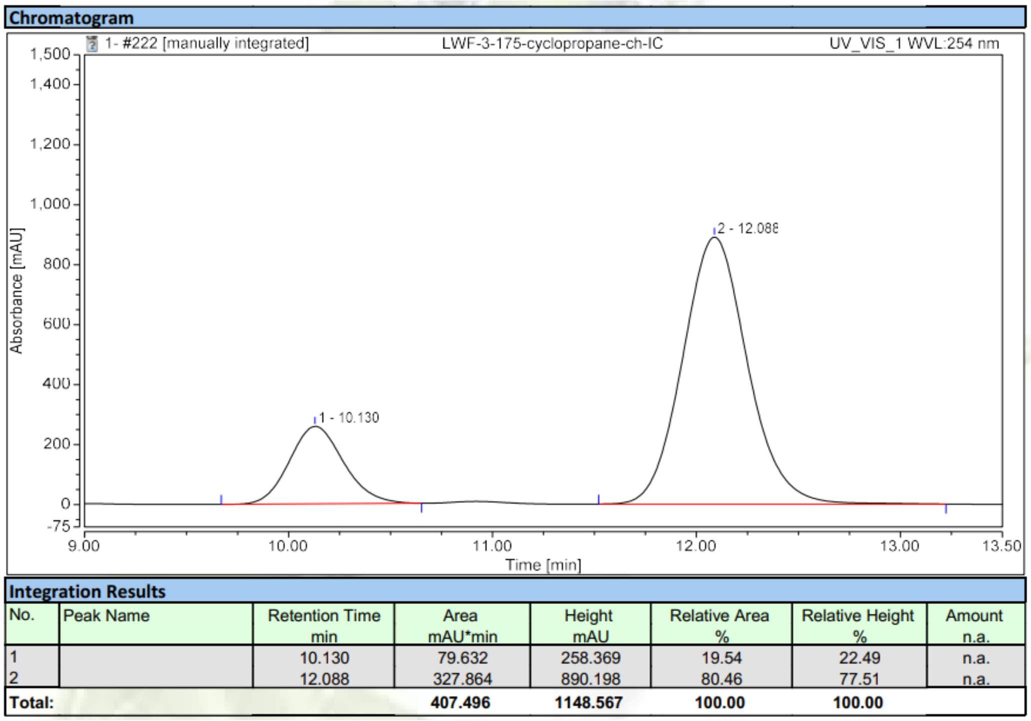

Integration Results

No.	Peak Name	Retention Time min	Area mAU*min	Height mAU	Relative Area %	Relative Height %	Amount n.a.
1		10.130	79.632	258.369	19.54	22.49	n.a.
2		12.088	327.864	890.198	80.46	77.51	n.a.
**Total:**			**407.496**	**1148.567**	**100.00**	**100.00**	

Compound B31: IB, *i*-PrOH/hexane = 50/50, v = 1.0 mL/min, λ = 254 nm

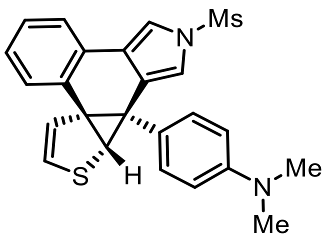

Chromatogram

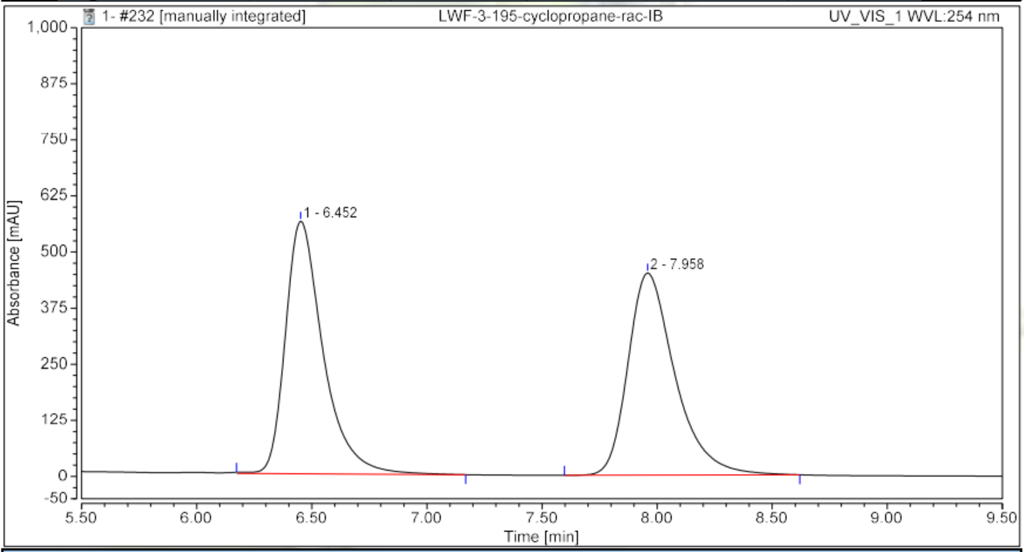

Integration Results

No.	Peak Name	Retention Time min	Area mAU*min	Height mAU	Relative Area %	Relative Height %	Amount n.a.
1		6.452	107.098	562.836	50.12	55.55	n.a.
2		7.958	106.593	450.391	49.88	44.45	n.a.
**Total:**			**213.691**	**1013.227**	**100.00**	**100.00**	

Chromatogram

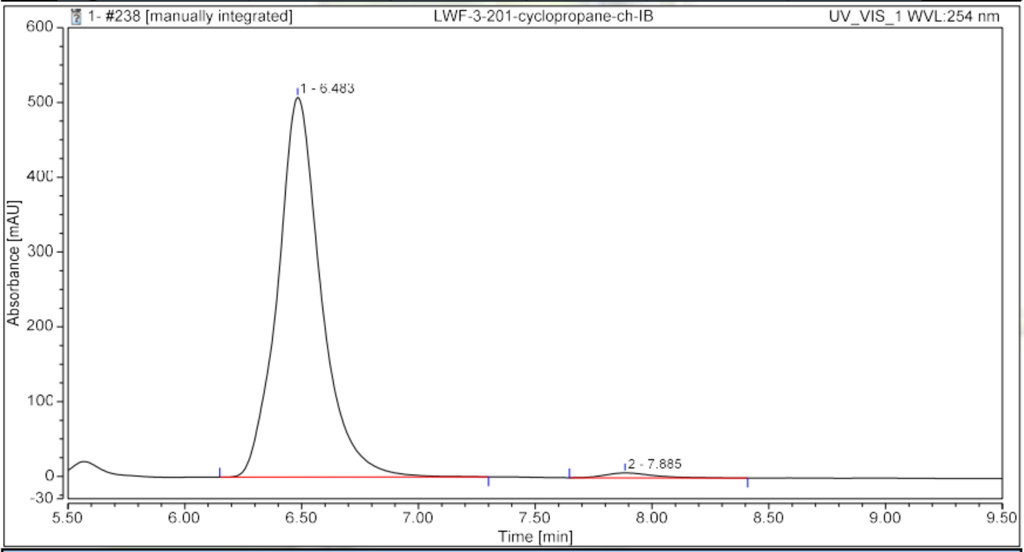

Integration Results

No.	Peak Name	Retention Time min	Area mAU*min	Height mAU	Relative Area %	Relative Height %	Amount n.a.
1		6.483	109.154	507.890	98.56	98.72	n.a.
2		7.885	1.594	6.610	1.44	1.28	n.a.
**Total:**			**110.748**	**514.500**	**100.00**	**100.00**	

Compound B32: IC, *i*-PrOH/hexane = 50/50, v = 1.0 mL/min, λ = 254 nm

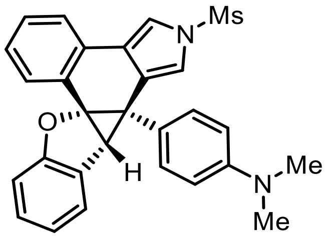

Chromatogram

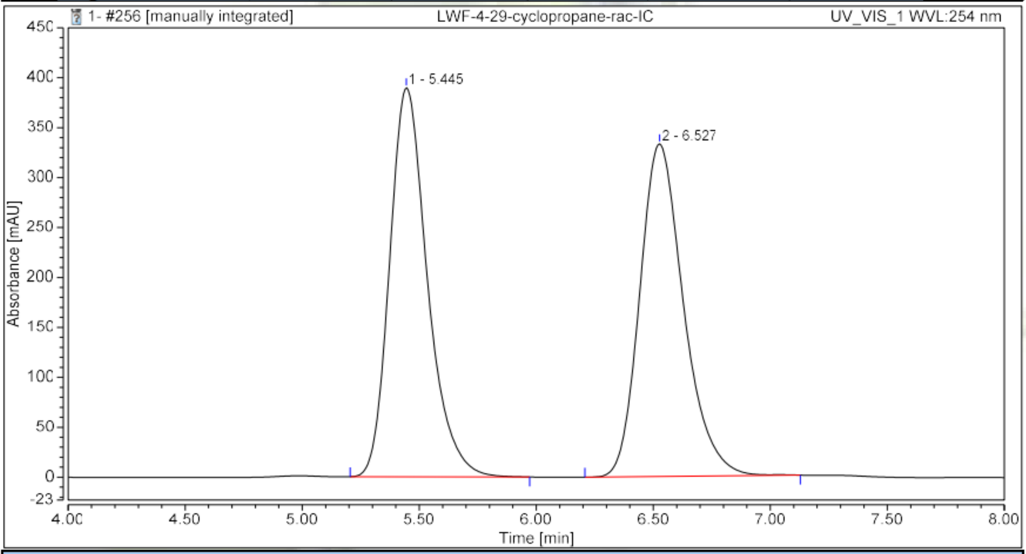

Integration Results

No.	Peak Name	Retention Time min	Area mAU*min	Height mAU	Relative Area %	Relative Height %	Amount n.a.
1		5.445	72.843	389.497	50.23	53.90	n.a.
2		6.527	72.176	333.103	49.77	46.10	n.a.
**Total:**			**145.019**	**722.600**	**100.00**	**100.00**	

Chromatogram

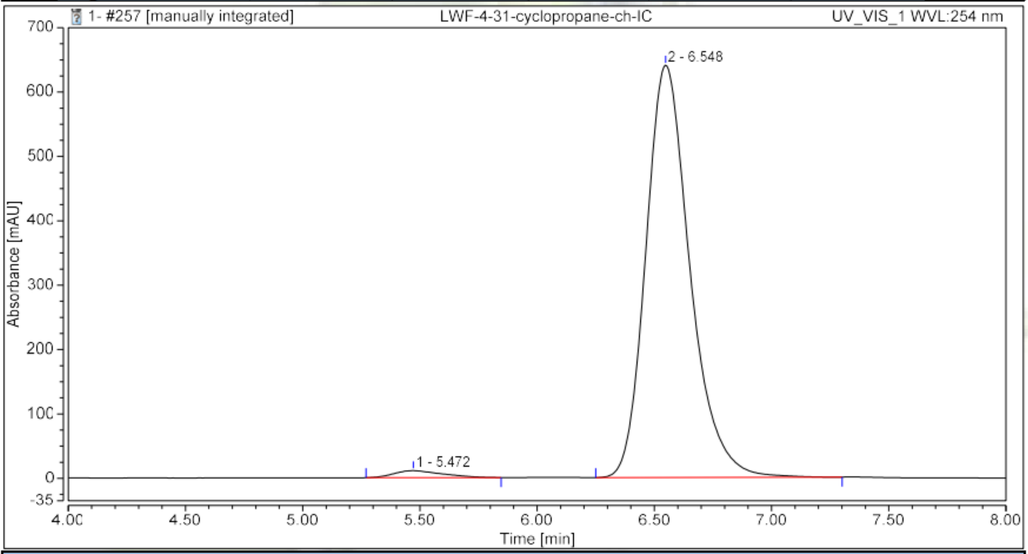

Integration Results

No.	Peak Name	Retention Time min	Area mAU*min	Height mAU	Relative Area %	Relative Height %	Amount n.a.
1		5.472	2.599	10.876	1.83	1.67	n.a.
2		6.548	139.530	640.588	98.17	98.33	n.a.
**Total:**			**142.129**	**651.464**	**100.00**	**100.00**	

Compound B33: IB, *i*-PrOH/hexane = 15/85, v = 1.0 mL/min, λ = 254 nm

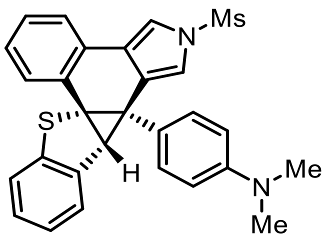

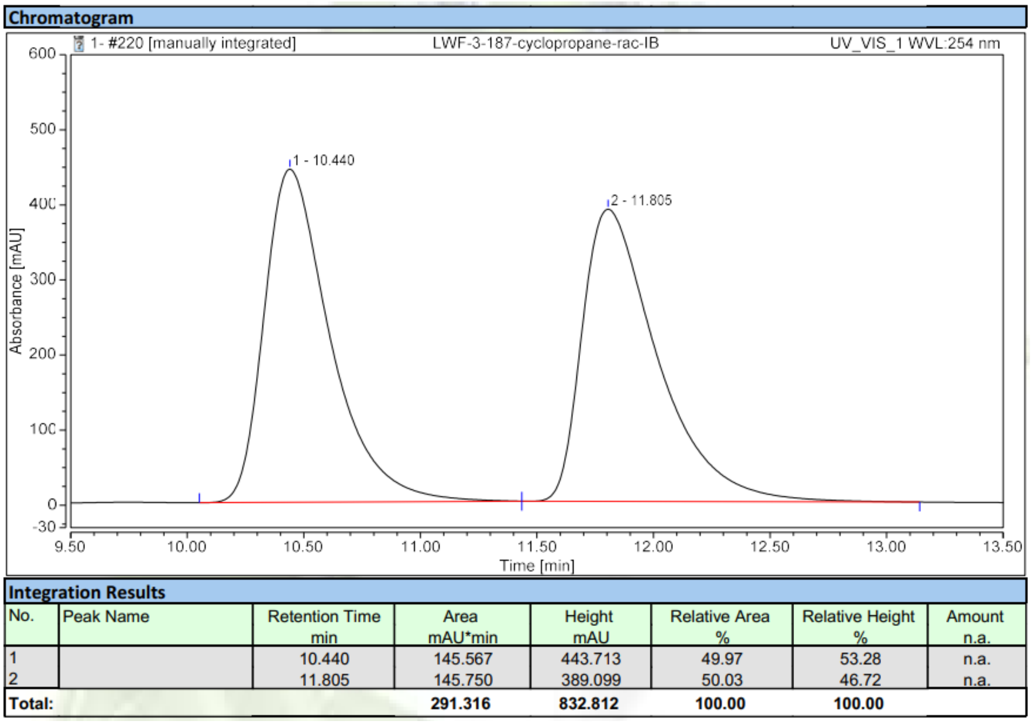

Integration Results

No.	Peak Name	Retention Time min	Area mAU*min	Height mAU	Relative Area %	Relative Height %	Amount n.a.
1		10.440	145.567	443.713	49.97	53.28	n.a.
2		11.805	145.750	389.099	50.03	46.72	n.a.
**Total:**			**291.316**	**832.812**	**100.00**	**100.00**	

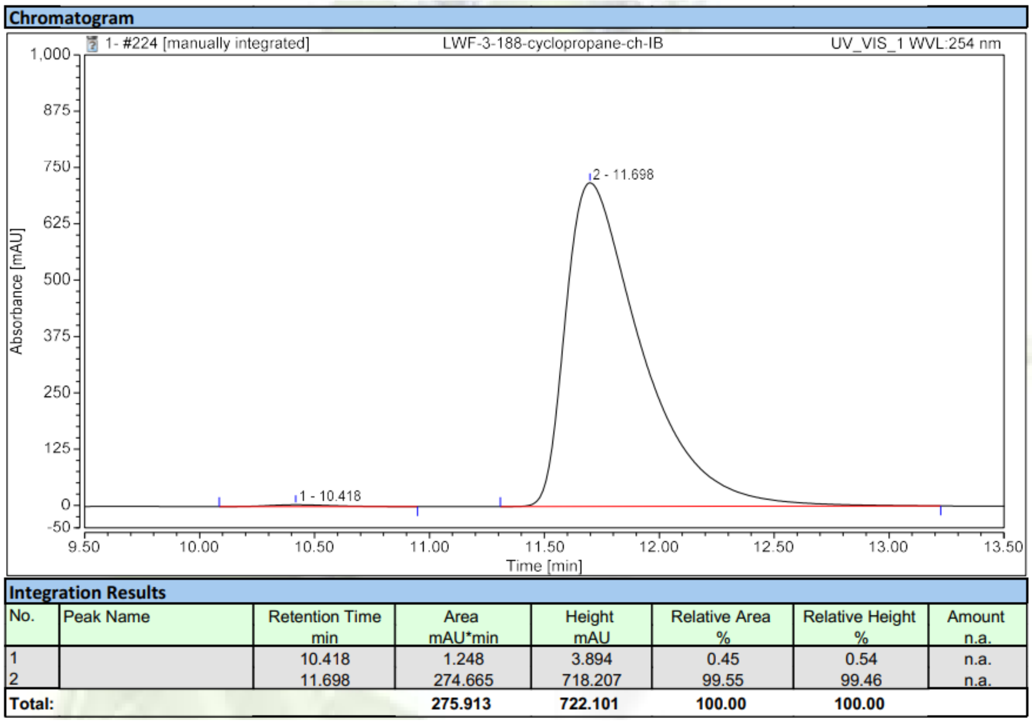

Integration Results

No.	Peak Name	Retention Time min	Area mAU*min	Height mAU	Relative Area %	Relative Height %	Amount n.a.
1		10.418	1.248	3.894	0.45	0.54	n.a.
2		11.698	274.665	718.207	99.55	99.46	n.a.
**Total:**			**275.913**	**722.101**	**100.00**	**100.00**	

Compound B34: IA, *i*-PrOH/hexane = 50/50, v = 1.0 mL/min, λ = 254 nm

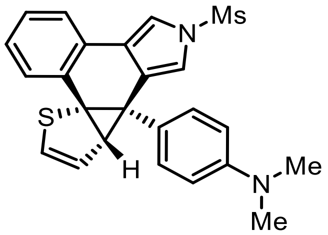

Chromatogram

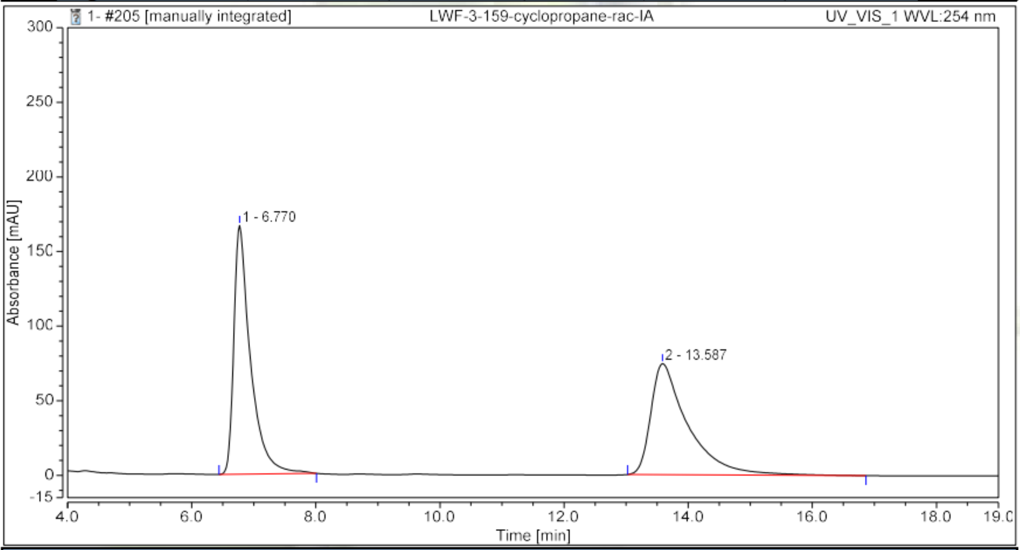

Integration Results

No.	Peak Name	Retention Time min	Area mAU*min	Height mAU	Relative Area %	Relative Height %	Amount n.a.
1		6.770	51.684	166.881	50.08	69.15	n.a.
2		13.587	51.518	74.467	49.92	30.85	n.a.
**Total:**			**103.202**	**241.349**	**100.00**	**100.00**	

Chromatogram

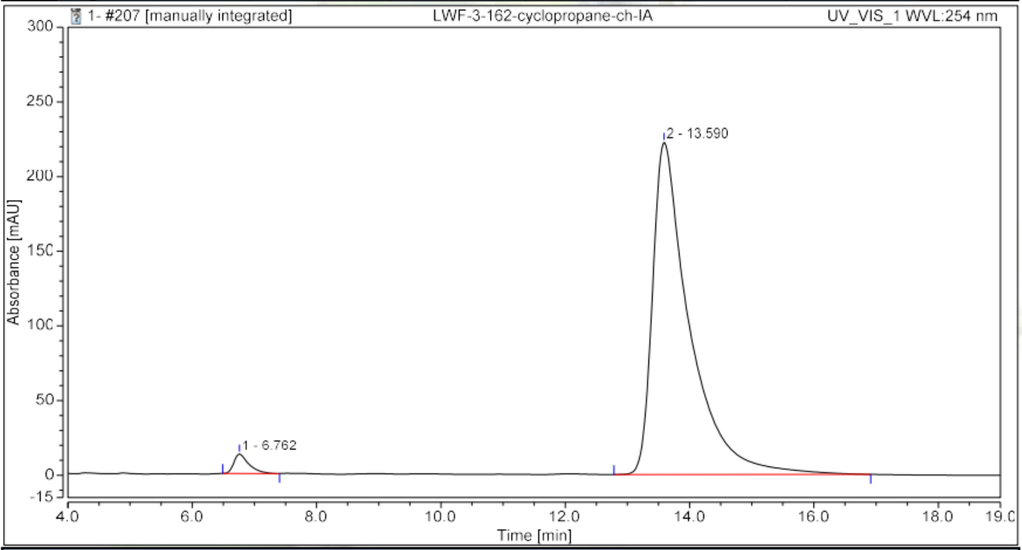

Integration Results

No.	Peak Name	Retention Time min	Area mAU*min	Height mAU	Relative Area %	Relative Height %	Amount n.a.
1		6.762	3.632	13.053	2.31	5.54	n.a.
2		13.590	153.500	222.504	97.69	94.46	n.a.
**Total:**			**157.132**	**235.557**	**100.00**	**100.00**	

Compound C1: IA, *i*-PrOH/hexane = 50/50, v = 1.0 mL/min, λ = 254 nm

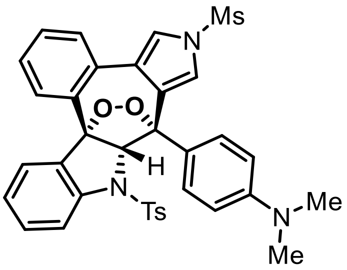

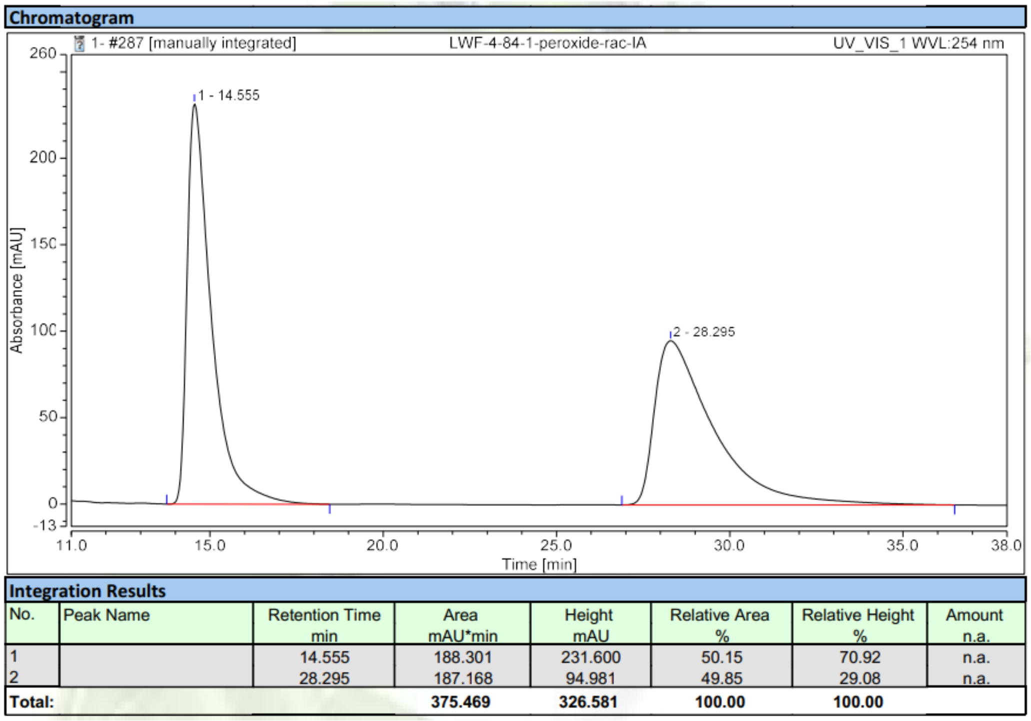

Integration Results

No.	Peak Name	Retention Time min	Area mAU*min	Height mAU	Relative Area %	Relative Height %	Amount n.a.
1		14.555	188.301	231.600	50.15	70.92	n.a.
2		28.295	187.168	94.981	49.85	29.08	n.a.
**Total:**			**375.469**	**326.581**	**100.00**	**100.00**	

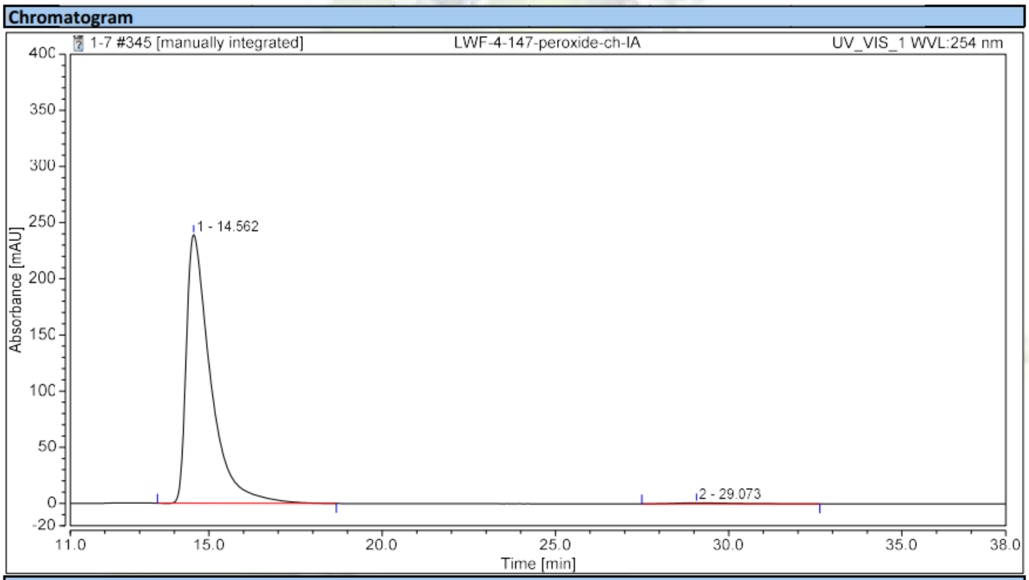

Integration Results

No.	Peak Name	Retention Time min	Area mAU*min	Height mAU	Relative Area %	Relative Height %	Amount n.a.
1		14.562	193.510	239.200	99.22	99.64	n.a.
2		29.073	1.530	0.862	0.78	0.36	n.a.
**Total:**			**195.040**	**240.063**	**100.00**	**100.00**	

Compound C2: IA, *i*-PrOH/hexane = 50/50, v = 1.0 mL/min, λ = 254 nm

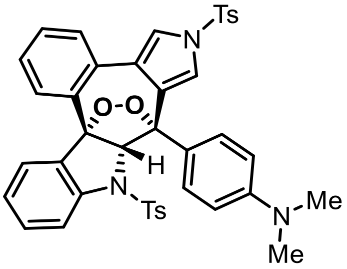

Chromatogram

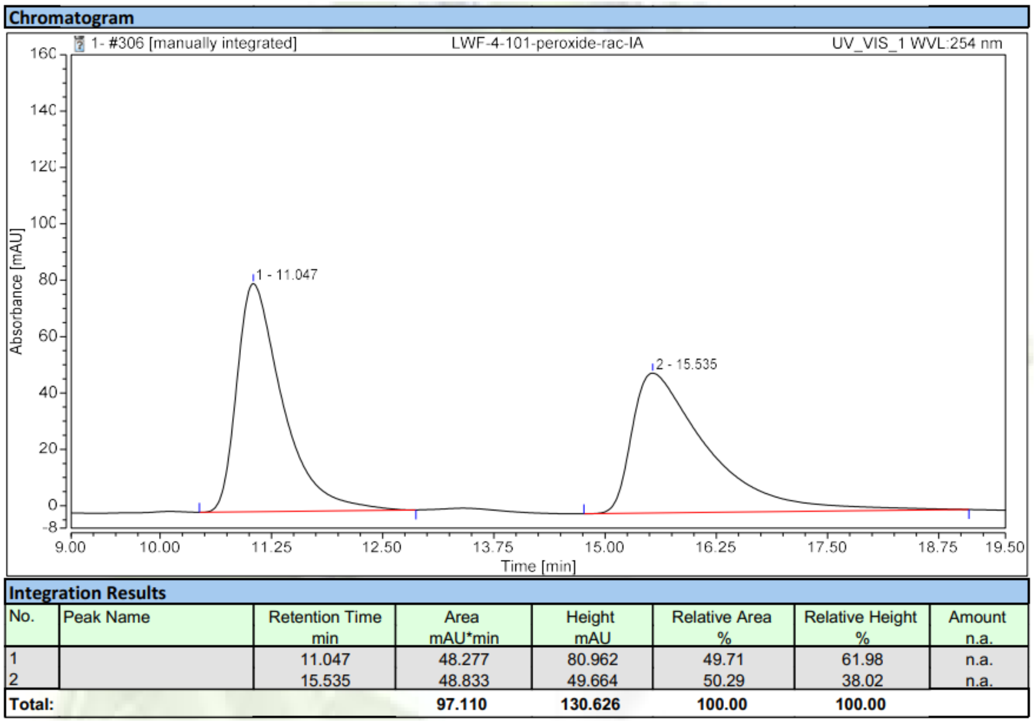

Integration Results

No.	Peak Name	Retention Time min	Area mAU*min	Height mAU	Relative Area %	Relative Height %	Amount n.a.
1		11.047	48.277	80.962	49.71	61.98	n.a.
2		15.535	48.833	49.664	50.29	38.02	n.a.
**Total:**			**97.110**	**130.626**	**100.00**	**100.00**	

Chromatogram

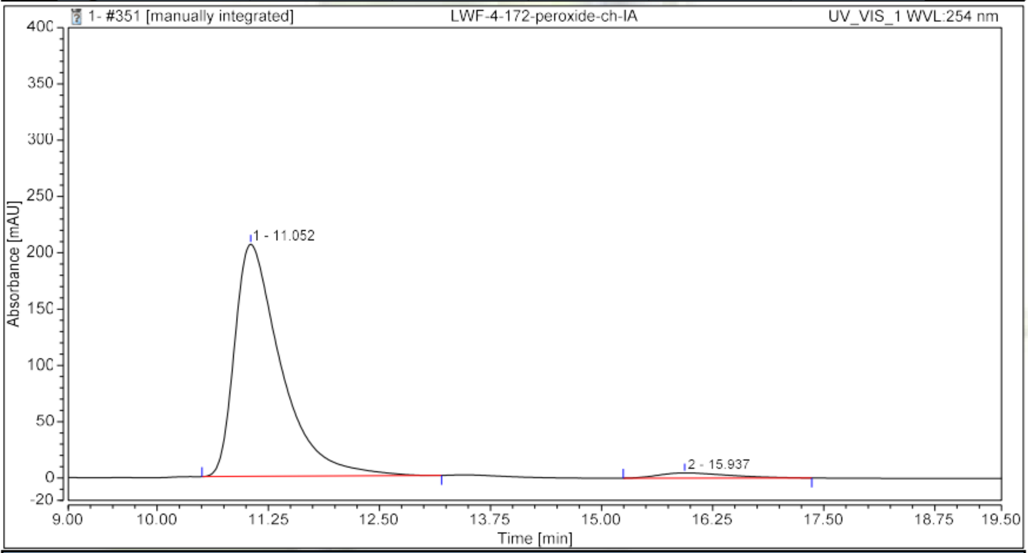

Integration Results

No.	Peak Name	Retention Time min	Area mAU*min	Height mAU	Relative Area %	Relative Height %	Amount n.a.
1		11.052	124.687	206.170	96.75	97.83	n.a.
2		15.937	4.184	4.584	3.25	2.17	n.a.
**Total:**			**128.871**	**210.753**	**100.00**	**100.00**	

Compound C3: IB, *i*-PrOH/hexane = 50/50, v = 1.0 mL/min, λ = 254 nm

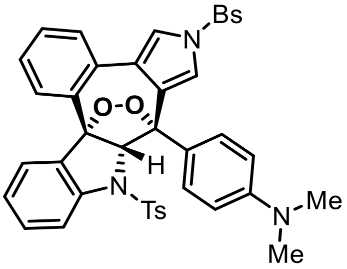

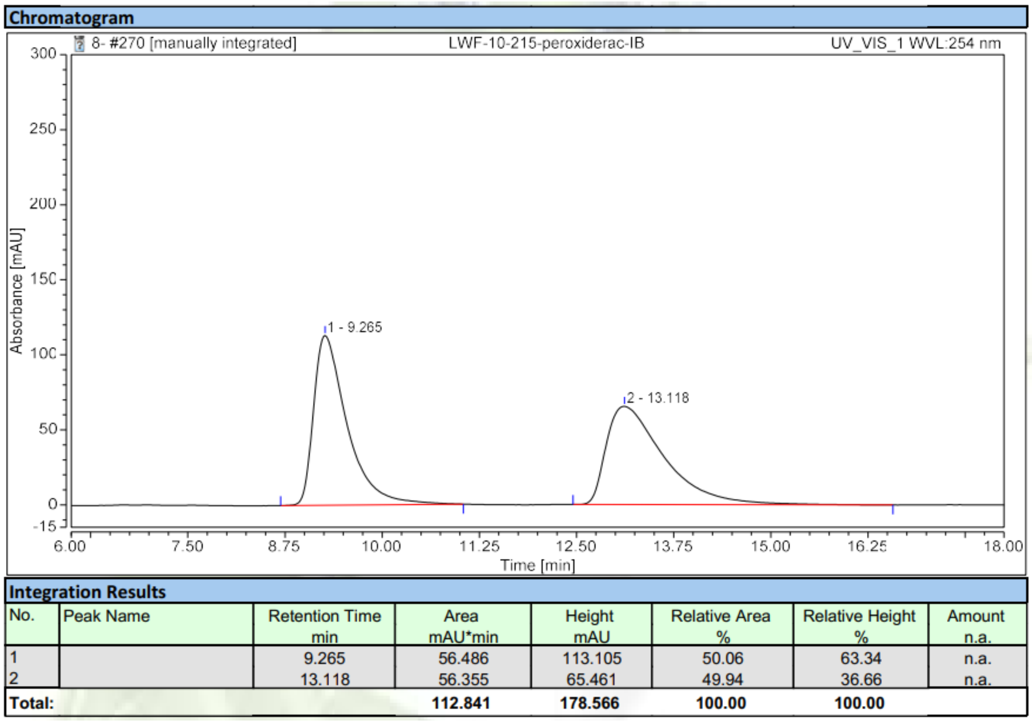

Integration Results

No.	Peak Name	Retention Time min	Area mAU*min	Height mAU	Relative Area %	Relative Height %	Amount n.a.
1		9.265	56.486	113.105	50.06	63.34	n.a.
2		13.118	56.355	65.461	49.94	36.66	n.a.
**Total:**			**112.841**	**178.566**	**100.00**	**100.00**	

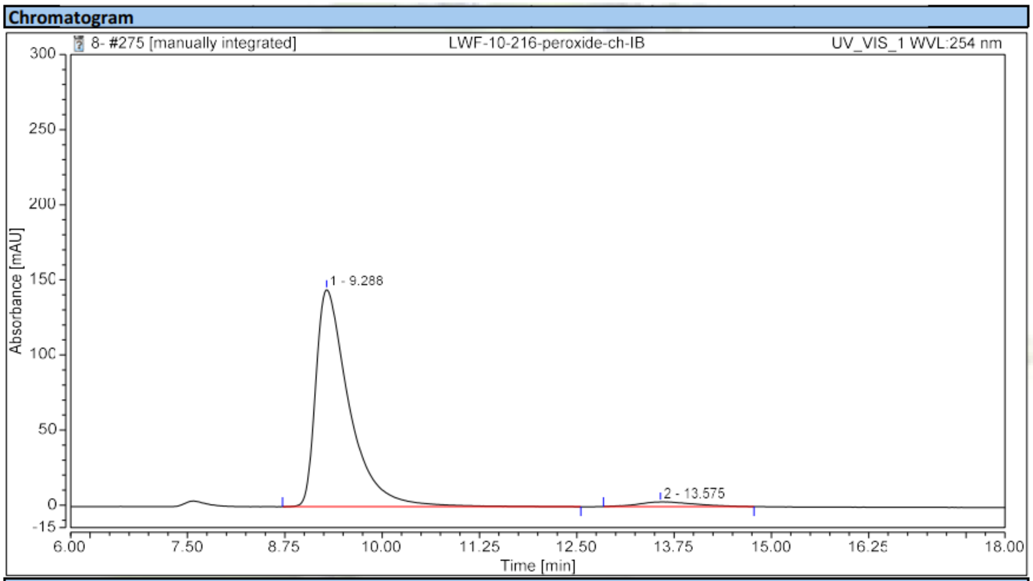

Integration Results

No.	Peak Name	Retention Time min	Area mAU*min	Height mAU	Relative Area %	Relative Height %	Amount n.a.
1		9.288	71.983	144.577	96.56	97.92	n.a.
2		13.575	2.563	3.078	3.44	2.08	n.a.
**Total:**			**74.546**	**147.655**	**100.00**	**100.00**	

Compound C4: IC, *i*-PrOH/hexane = 50/50, v = 1.0 mL/min, λ = 254 nm

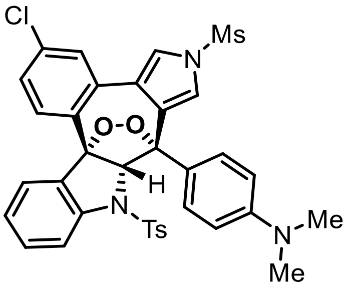

Chromatogram

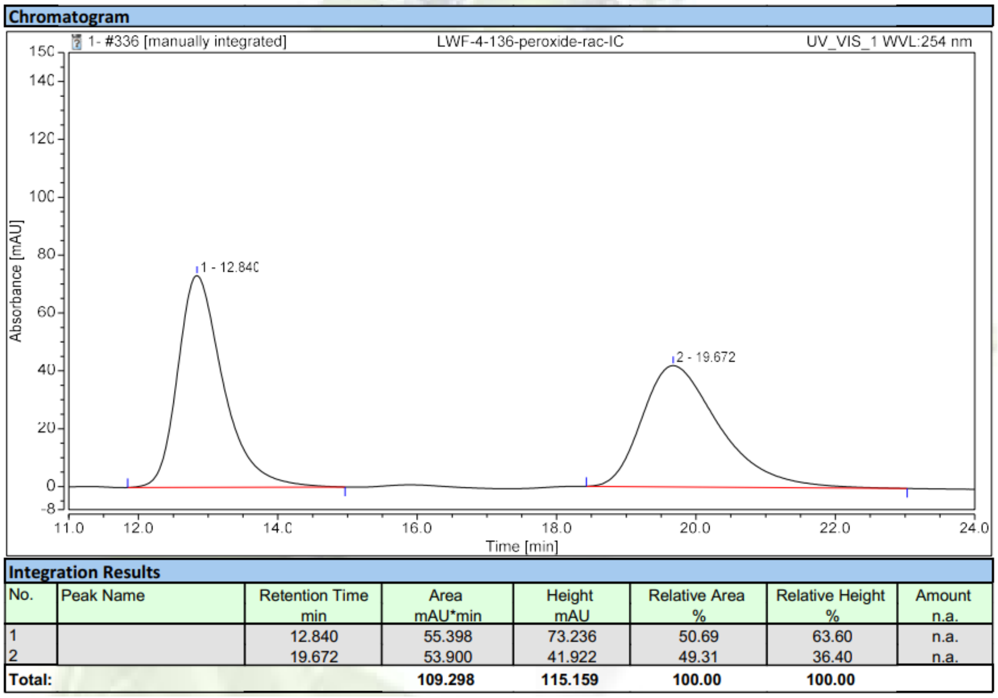

Integration Results

No.	Peak Name	Retention Time min	Area mAU*min	Height mAU	Relative Area %	Relative Height %	Amount n.a.
1		12.840	55.398	73.236	50.69	63.60	n.a.
2		19.672	53.900	41.922	49.31	36.40	n.a.
**Total:**			**109.298**	**115.159**	**100.00**	**100.00**	

Chromatogram

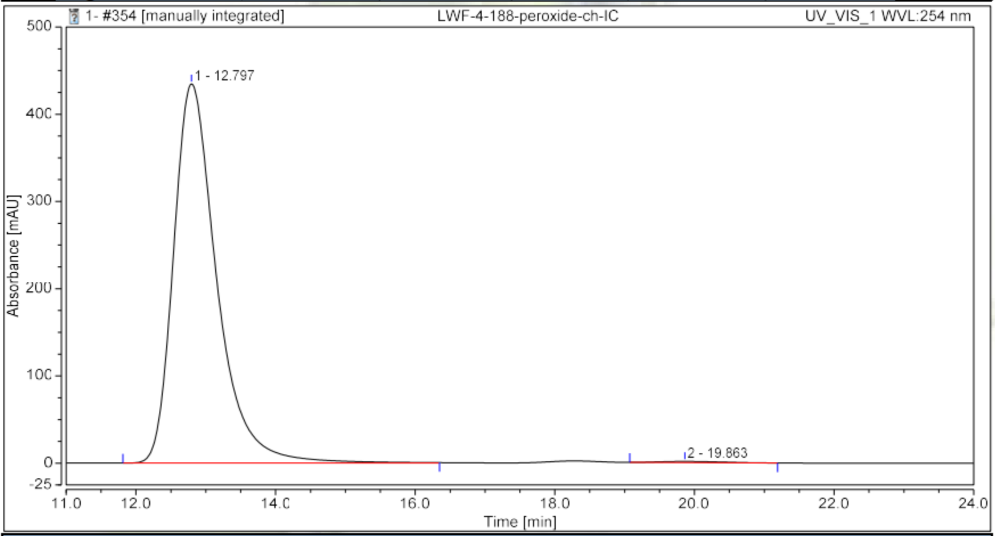

Integration Results

No.	Peak Name	Retention Time min	Area mAU*min	Height mAU	Relative Area %	Relative Height %	Amount n.a.
1		12.797	311.844	434.845	99.56	99.69	n.a.
2		19.863	1.376	1.353	0.44	0.31	n.a.
**Total:**			**313.220**	**436.197**	**100.00**	**100.00**	

Compound C5: IC, *i*-PrOH/hexane = 50/50, v = 1.0 mL/min, λ = 254 nm

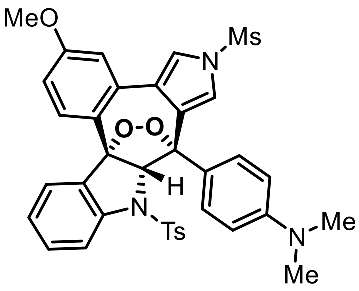

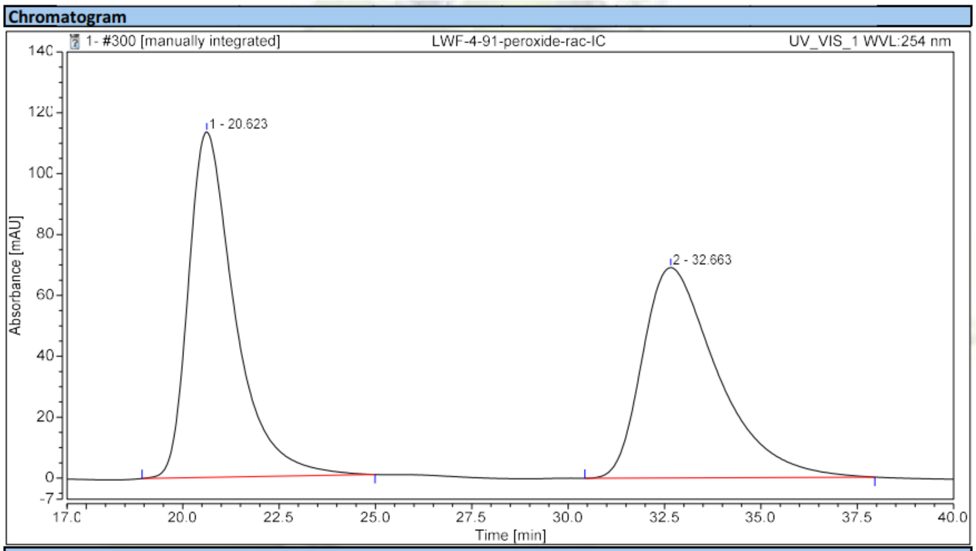

Integration Results

No.	Peak Name	Retention Time min	Area mAU*min	Height mAU	Relative Area %	Relative Height %	Amount n.a.
1		20.623	158.813	113.515	50.37	62.16	n.a.
2		32.663	156.476	69.095	49.63	37.84	n.a.
Total:			315.288	182.610	100.00	100.00	

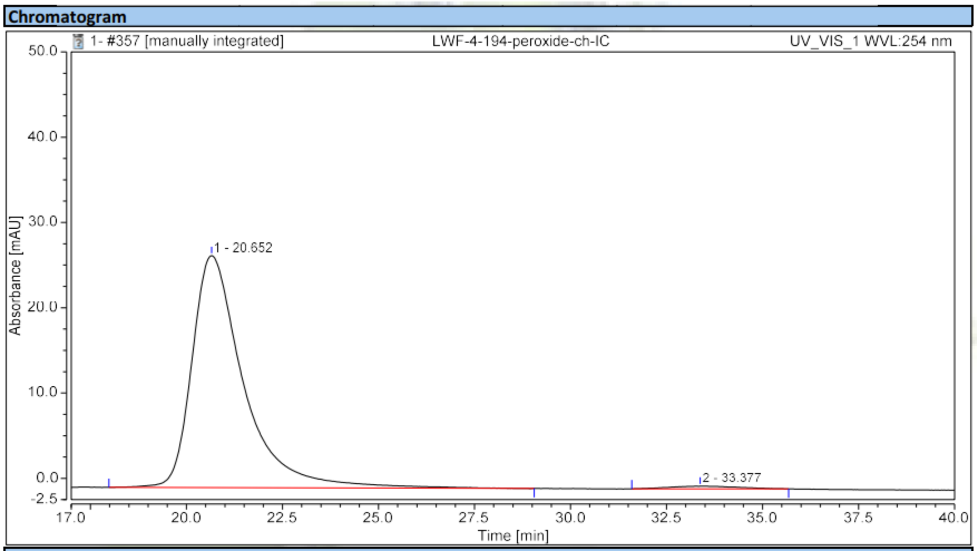

Integration Results

No.	Peak Name	Retention Time min	Area mAU*min	Height mAU	Relative Area %	Relative Height %	Amount n.a.
1		20.652	42.505	27.215	98.53	98.87	n.a.
2		33.377	0.633	0.312	1.47	1.13	n.a.
Total:			43.138	27.527	100.00	100.00	

Compound C6: IC, *i*-PrOH/hexane = 50/50, v = 1.0 mL/min, λ = 254 nm

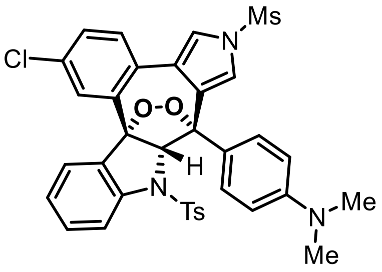

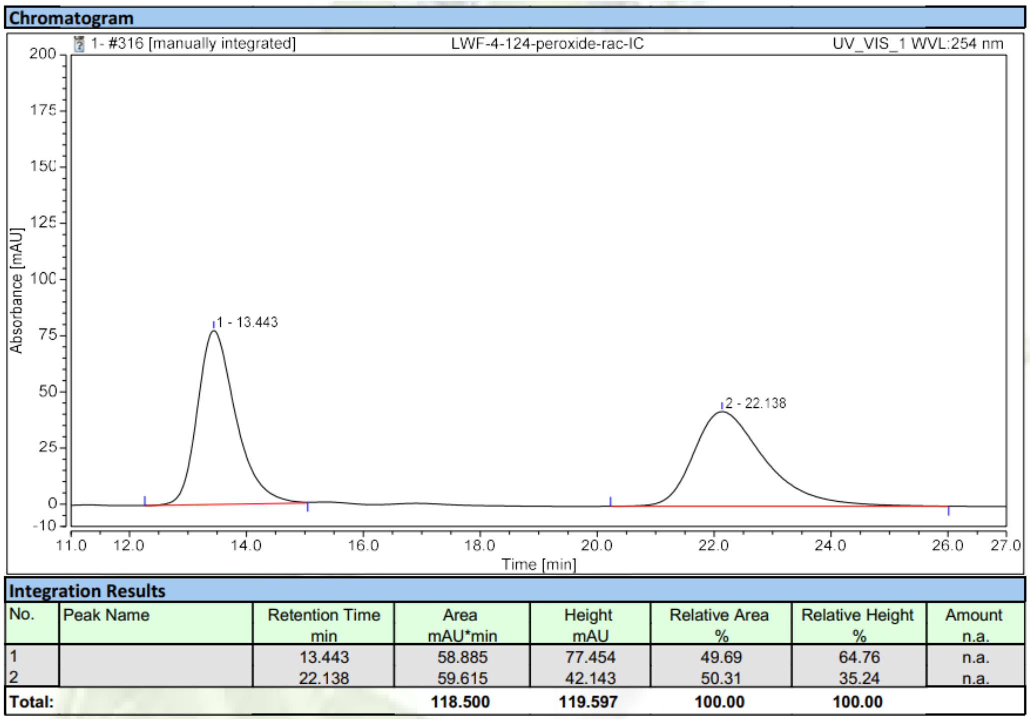

Integration Results

No.	Peak Name	Retention Time min	Area mAU*min	Height mAU	Relative Area %	Relative Height %	Amount n.a.
1		13.443	58.885	77.454	49.69	64.76	n.a.
2		22.138	59.615	42.143	50.31	35.24	n.a.
**Total:**			**118.500**	**119.597**	**100.00**	**100.00**	

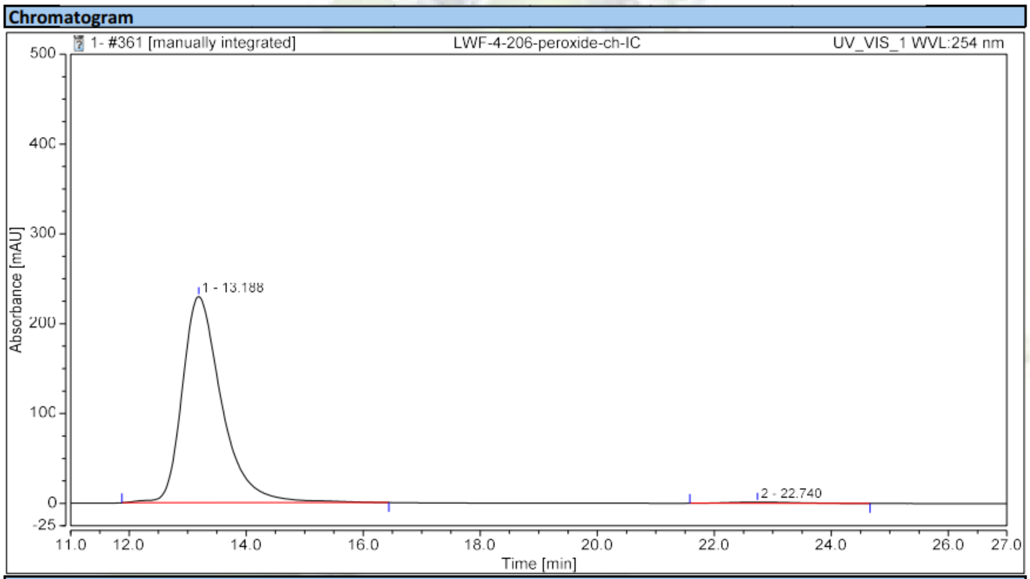

Integration Results

No.	Peak Name	Retention Time min	Area mAU*min	Height mAU	Relative Area %	Relative Height %	Amount n.a.
1		13.188	179.545	229.502	99.02	99.43	n.a.
2		22.740	1.775	1.315	0.98	0.57	n.a.
**Total:**			**181.319**	**230.817**	**100.00**	**100.00**	

Compound C7: IA, *i*-PrOH/hexane = 50/50, v = 1.0 mL/min, λ = 254 nm

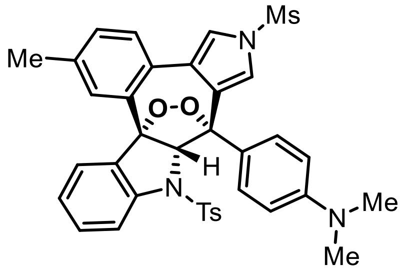

Chromatogram

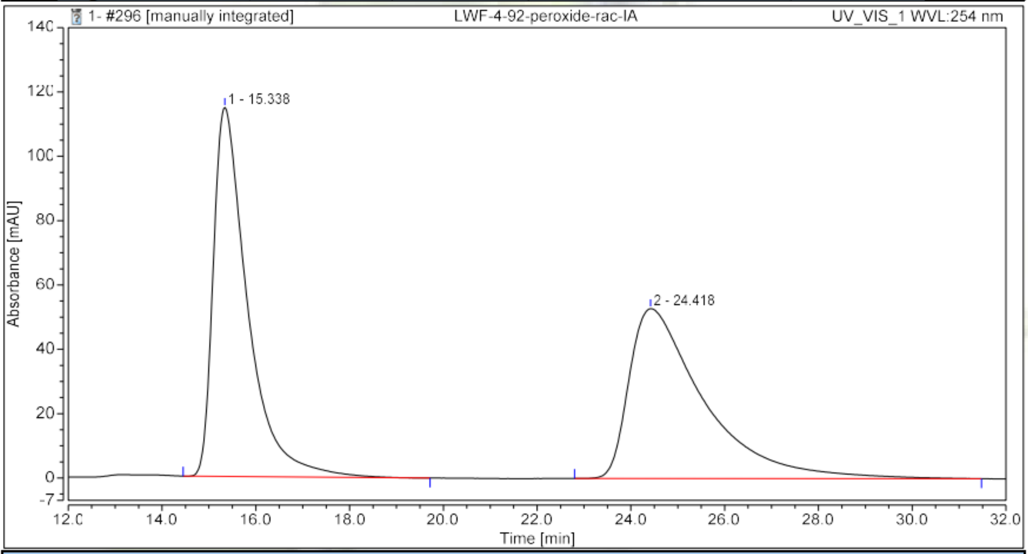

Integration Results

No.	Peak Name	Retention Time min	Area mAU*min	Height mAU	Relative Area %	Relative Height %	Amount n.a.
1		15.338	97.221	114.750	49.92	68.49	n.a.
2		24.418	97.521	52.804	50.08	31.51	n.a.
**Total:**			**194.742**	**167.554**	**100.00**	**100.00**	

Chromatogram

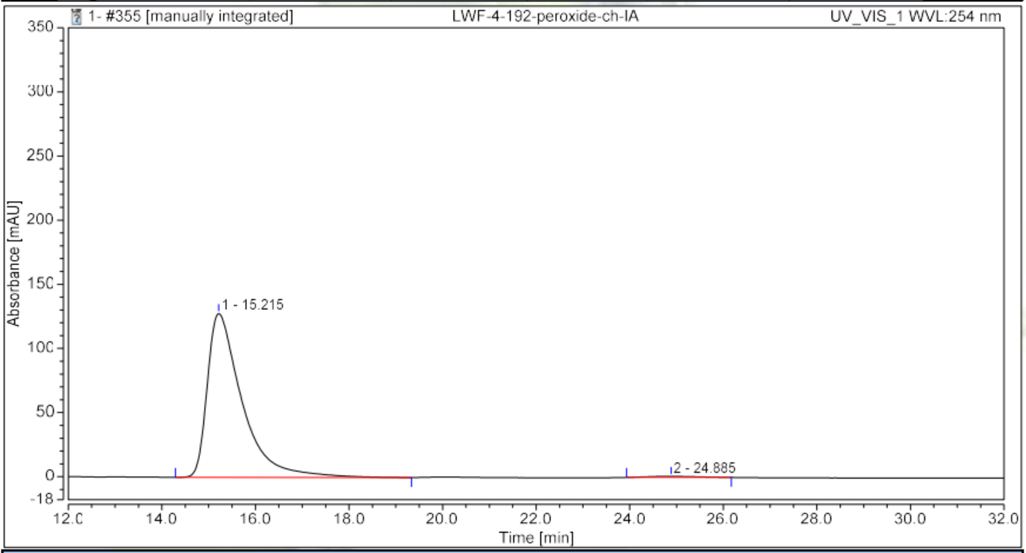

Integration Results

No.	Peak Name	Retention Time min	Area mAU*min	Height mAU	Relative Area %	Relative Height %	Amount n.a.
1		15.215	109.819	127.733	99.09	99.37	n.a.
2		24.885	1.006	0.806	0.91	0.63	n.a.
**Total:**			**110.825**	**128.539**	**100.00**	**100.00**	

Compound C8: IC, *i*-PrOH/hexane = 50/50, v = 1.0 mL/min, λ = 254 nm

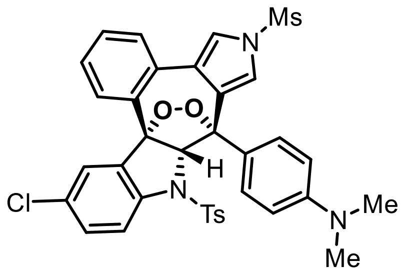

Chromatogram

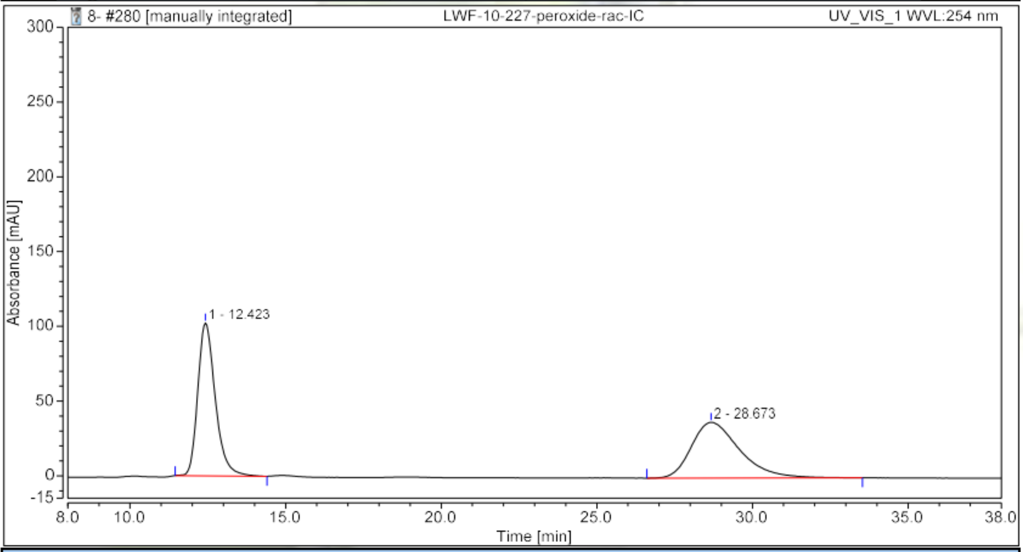

Integration Results

No.	Peak Name	Retention Time min	Area mAU*min	Height mAU	Relative Area %	Relative Height %	Amount n.a.
1		12.423	66.735	102.227	49.91	73.29	n.a.
2		28.673	66.968	37.248	50.09	26.71	n.a.
**Total:**			**133.703**	**139.475**	**100.00**	**100.00**	

Chromatogram

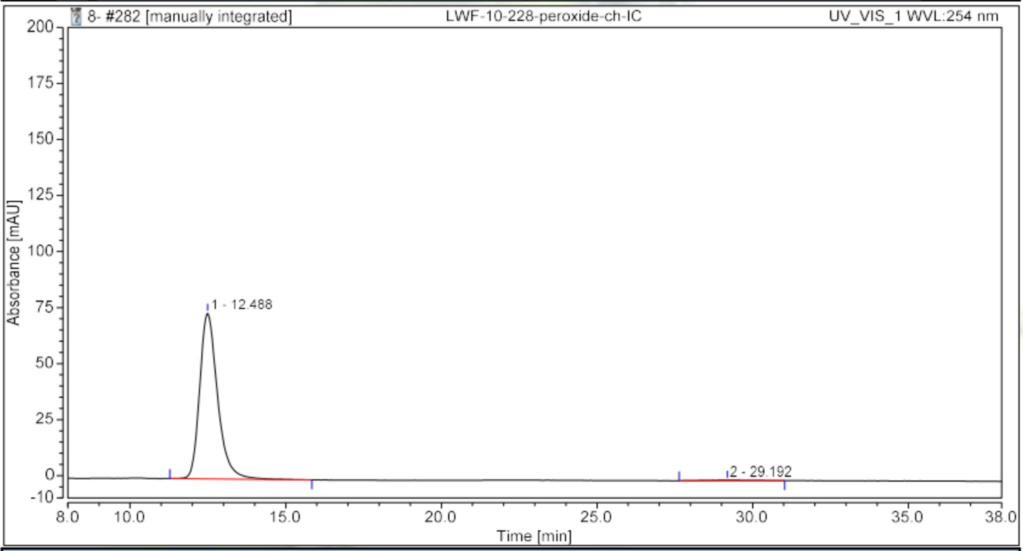

Integration Results

No.	Peak Name	Retention Time min	Area mAU*min	Height mAU	Relative Area %	Relative Height %	Amount n.a.
1		12.488	49.421	73.960	99.27	99.66	n.a.
2		29.192	0.365	0.256	0.73	0.34	n.a.
**Total:**			**49.786**	**74.216**	**100.00**	**100.00**	

Compound C9: IC, *i*-PrOH/hexane = 50/50, v = 1.0 mL/min, λ = 254 nm

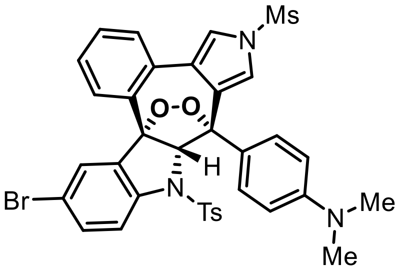

Chromatogram

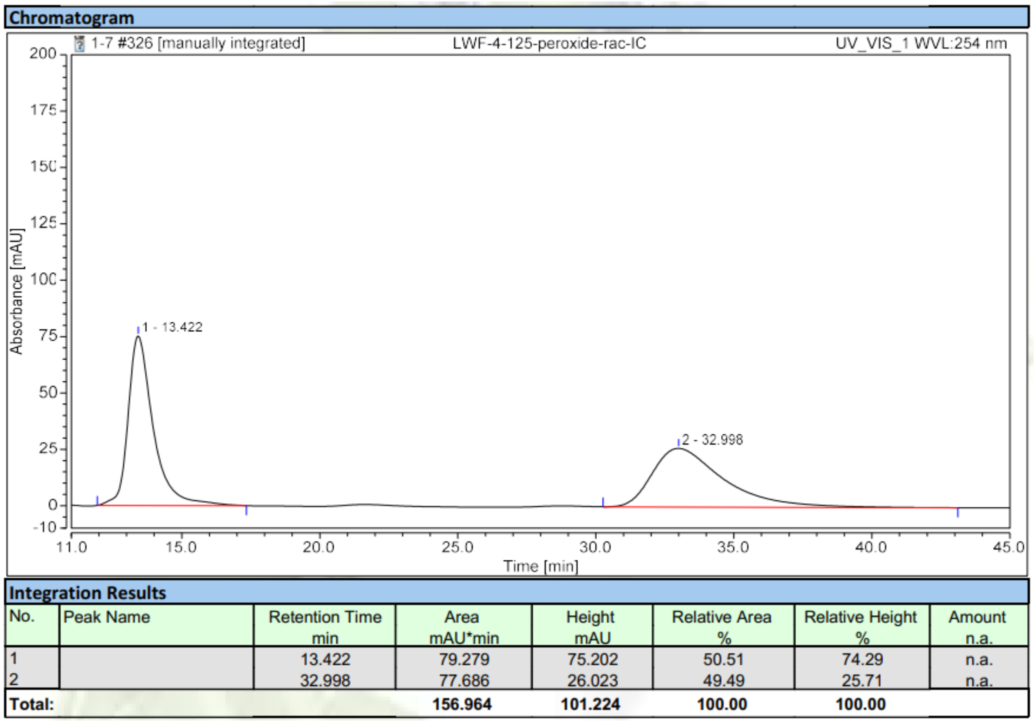

Integration Results

No.	Peak Name	Retention Time min	Area mAU*min	Height mAU	Relative Area %	Relative Height %	Amount n.a.
1		13.422	79.279	75.202	50.51	74.29	n.a.
2		32.998	77.686	26.023	49.49	25.71	n.a.
**Total:**			**156.964**	**101.224**	**100.00**	**100.00**	

Chromatogram

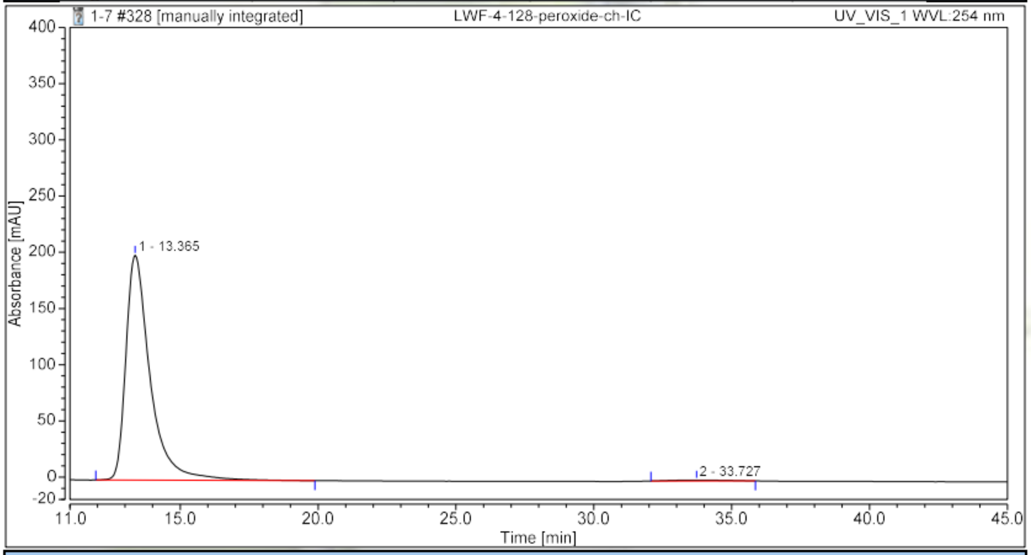

Integration Results

No.	Peak Name	Retention Time min	Area mAU*min	Height mAU	Relative Area %	Relative Height %	Amount n.a.
1		13.365	204.213	200.147	99.11	99.60	n.a.
2		33.727	1.836	0.799	0.89	0.40	n.a.
**Total:**			**206.049**	**200.946**	**100.00**	**100.00**	

Compound C10: IA, *i*-PrOH/hexane = 50/50, v = 1.0 mL/min, λ = 254 nm

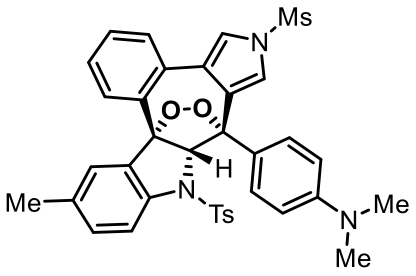

Chromatogram

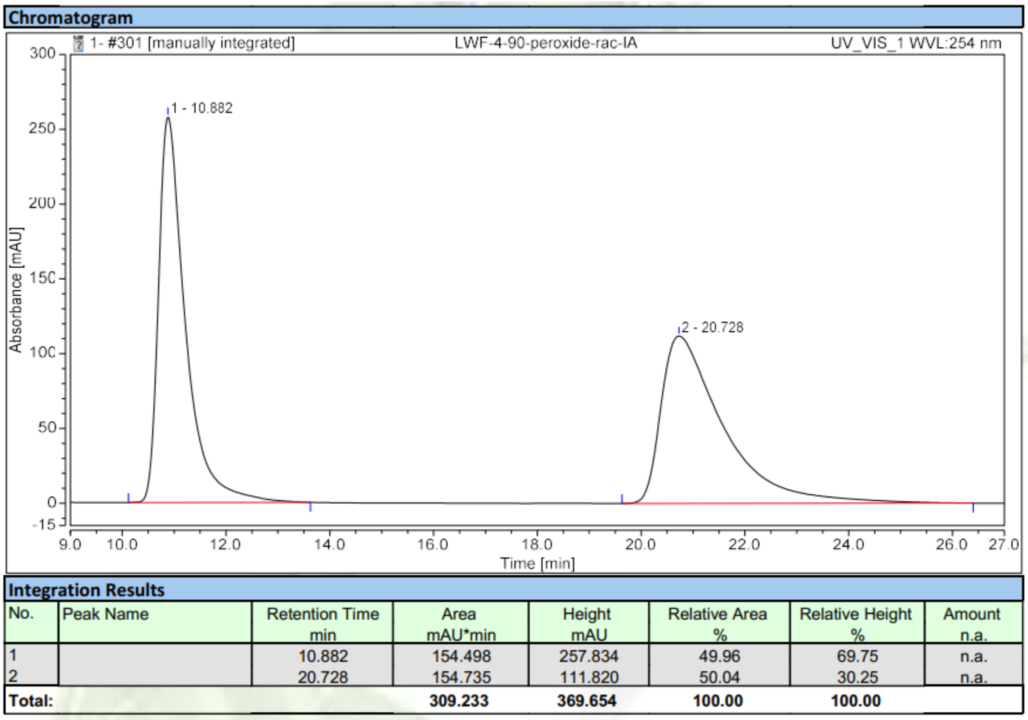

Integration Results

No.	Peak Name	Retention Time min	Area mAU*min	Height mAU	Relative Area %	Relative Height %	Amount n.a.
1		10.882	154.498	257.834	49.96	69.75	n.a.
2		20.728	154.735	111.820	50.04	30.25	n.a.
**Total:**			**309.233**	**369.654**	**100.00**	**100.00**	

Chromatogram

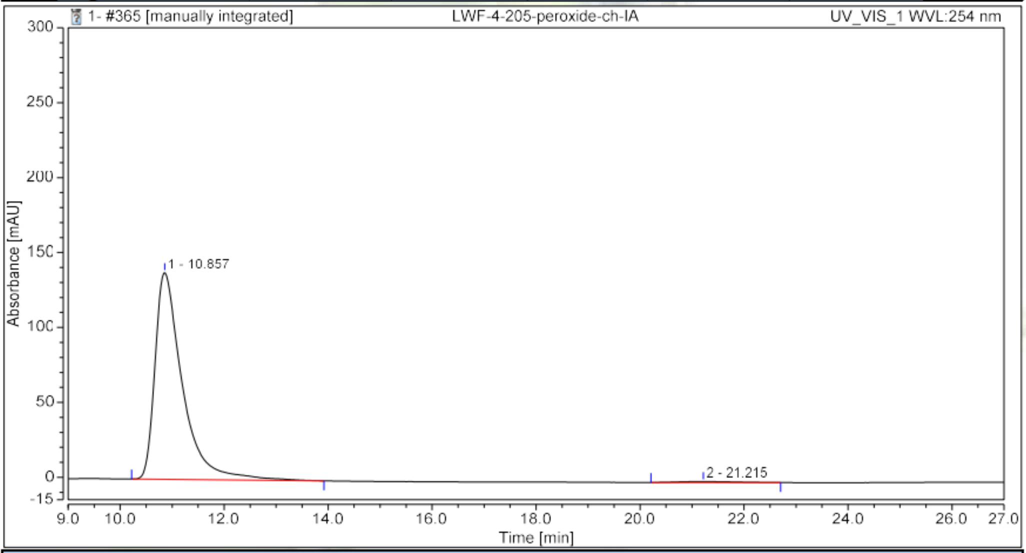

Integration Results

No.	Peak Name	Retention Time min	Area mAU*min	Height mAU	Relative Area %	Relative Height %	Amount n.a.
1		10.857	84.315	138.045	99.10	99.54	n.a.
2		21.215	0.763	0.644	0.90	0.46	n.a.
**Total:**			**85.077**	**138.689**	**100.00**	**100.00**	

Compound C11: IE, *i*-PrOH/hexane = 50/50, v = 1.0 mL/min, λ = 254 nm

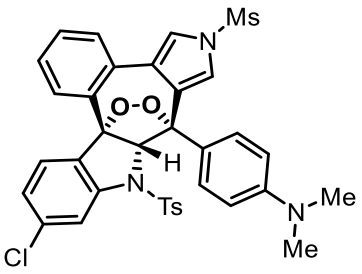

Chromatogram

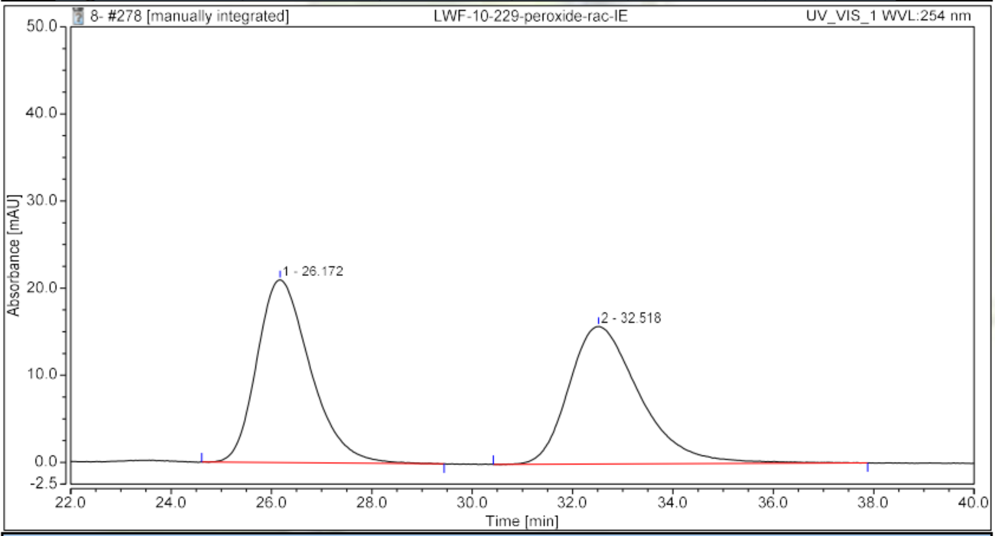

Integration Results

No.	Peak Name	Retention Time min	Area mAU*min	Height mAU	Relative Area %	Relative Height %	Amount n.a.
1		26.172	25.773	20.986	50.02	57.05	n.a.
2		32.518	25.756	15.798	49.98	42.95	n.a.
Total:			51.528	36.784	100.00	100.00	

Chromatogram

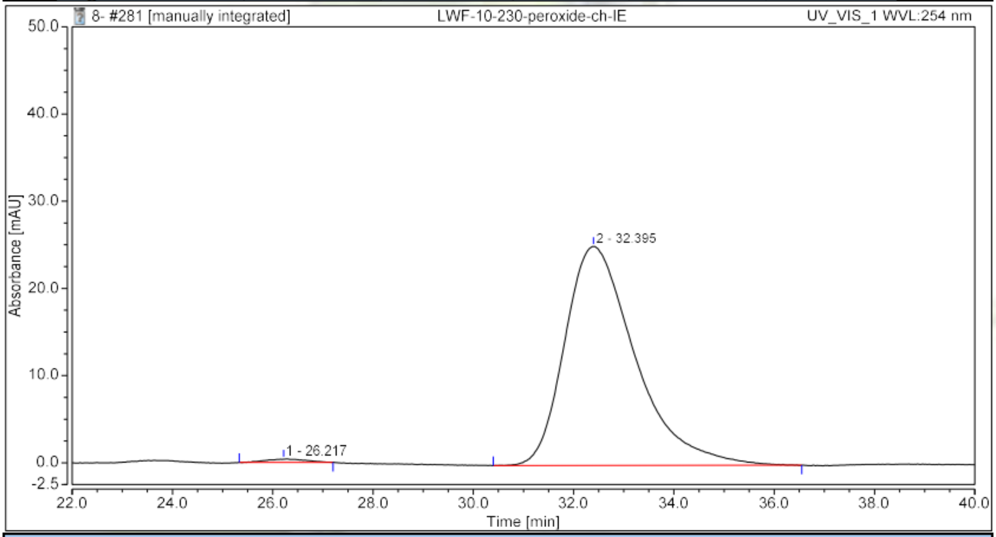

Integration Results

No.	Peak Name	Retention Time min	Area mAU*min	Height mAU	Relative Area %	Relative Height %	Amount n.a.
1		26.217	0.368	0.394	0.89	1.54	n.a.
2		32.395	40.801	25.114	99.11	98.46	n.a.
Total:			41.169	25.508	100.00	100.00	

Compound C12: IC, *i*-PrOH/hexane = 50/50, v = 1.0 mL/min, λ = 254 nm

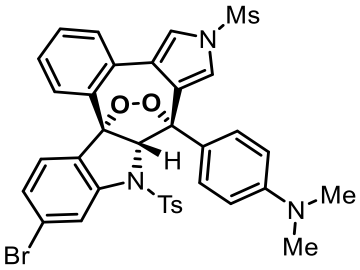

Chromatogram

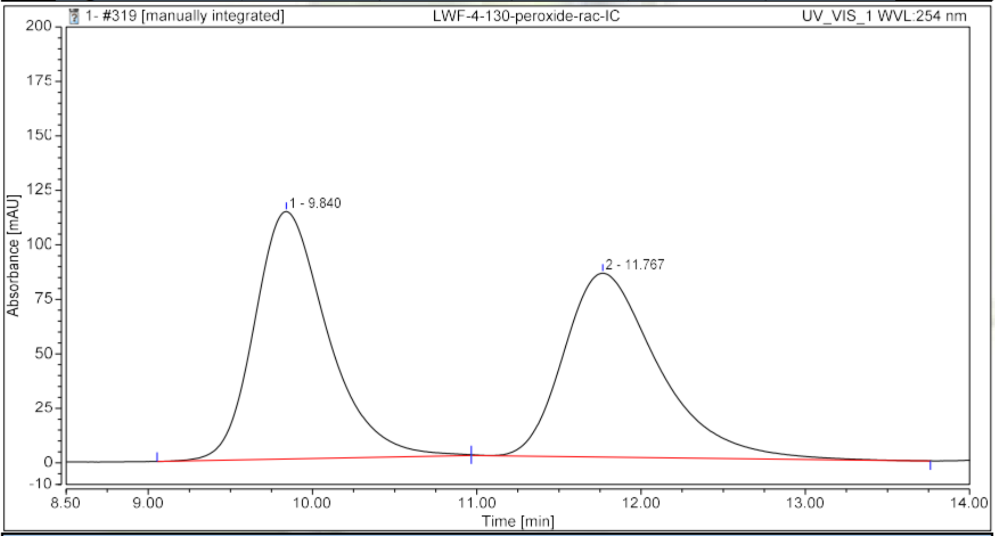

Integration Results

No.	Peak Name	Retention Time min	Area mAU*min	Height mAU	Relative Area %	Relative Height %	Amount n.a.
1		9.840	58.641	113.530	50.94	57.38	n.a.
2		11.767	56.484	84.341	49.06	42.62	n.a.
**Total:**			**115.124**	**197.872**	**100.00**	**100.00**	

Chromatogram

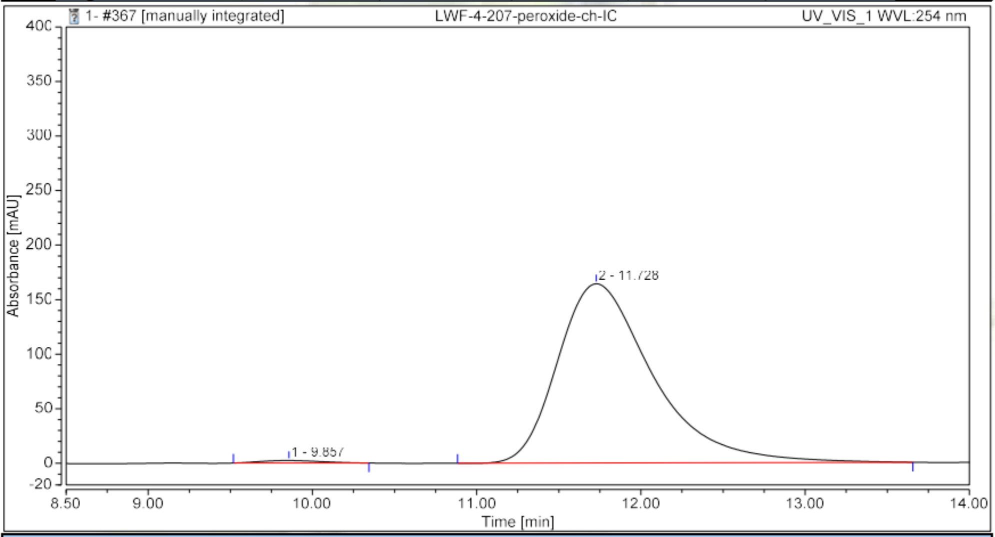

Integration Results

No.	Peak Name	Retention Time min	Area mAU*min	Height mAU	Relative Area %	Relative Height %	Amount n.a.
1		9.857	0.973	2.290	0.87	1.37	n.a.
2		11.728	110.645	164.278	99.13	98.63	n.a.
**Total:**			**111.617**	**166.568**	**100.00**	**100.00**	

Compound C13: IA, *i*-PrOH/hexane = 50/50, v = 1.0 mL/min, λ = 254 nm

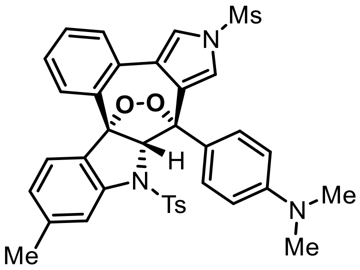

Chromatogram

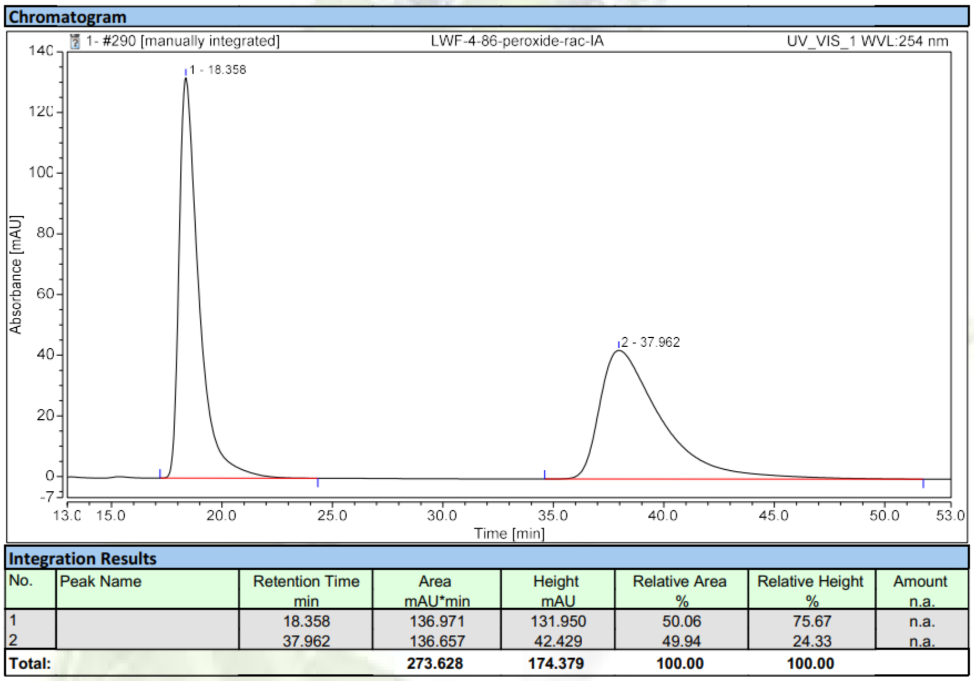

Integration Results

No.	Peak Name	Retention Time min	Area mAU*min	Height mAU	Relative Area %	Relative Height %	Amount n.a.
1		18.358	136.971	131.950	50.06	75.67	n.a.
2		37.962	136.657	42.429	49.94	24.33	n.a.
**Total:**			**273.628**	**174.379**	**100.00**	**100.00**	

Chromatogram

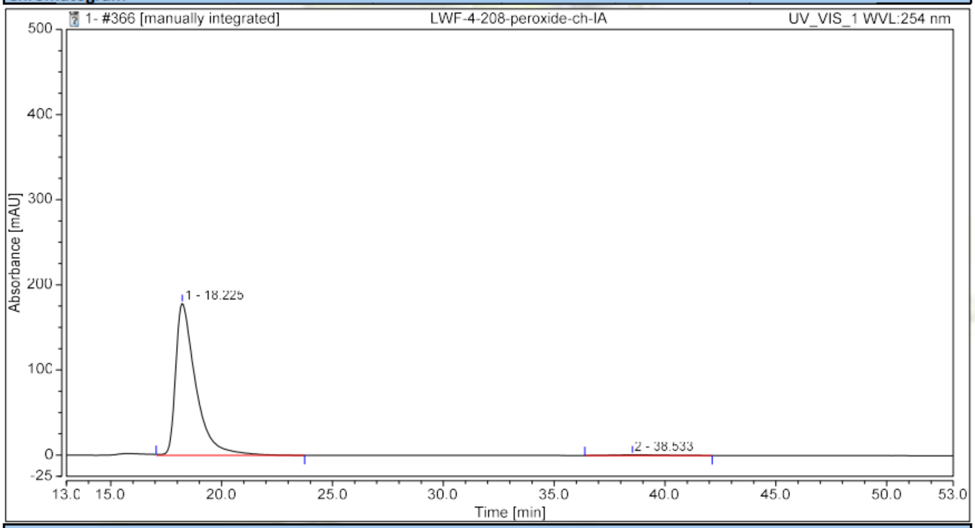

Integration Results

No.	Peak Name	Retention Time min	Area mAU*min	Height mAU	Relative Area %	Relative Height %	Amount n.a.
1		18.225	187.212	178.178	99.05	99.63	n.a.
2		38.533	1.789	0.657	0.95	0.37	n.a.
**Total:**			**189.001**	**178.835**	**100.00**	**100.00**	

Compound C14: IC, *i*-PrOH/hexane = 50/50, v = 1.0 mL/min, λ = 254 nm

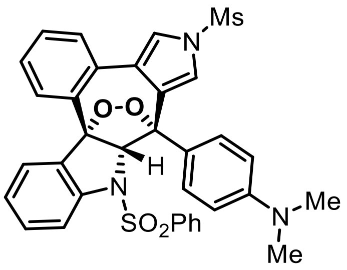

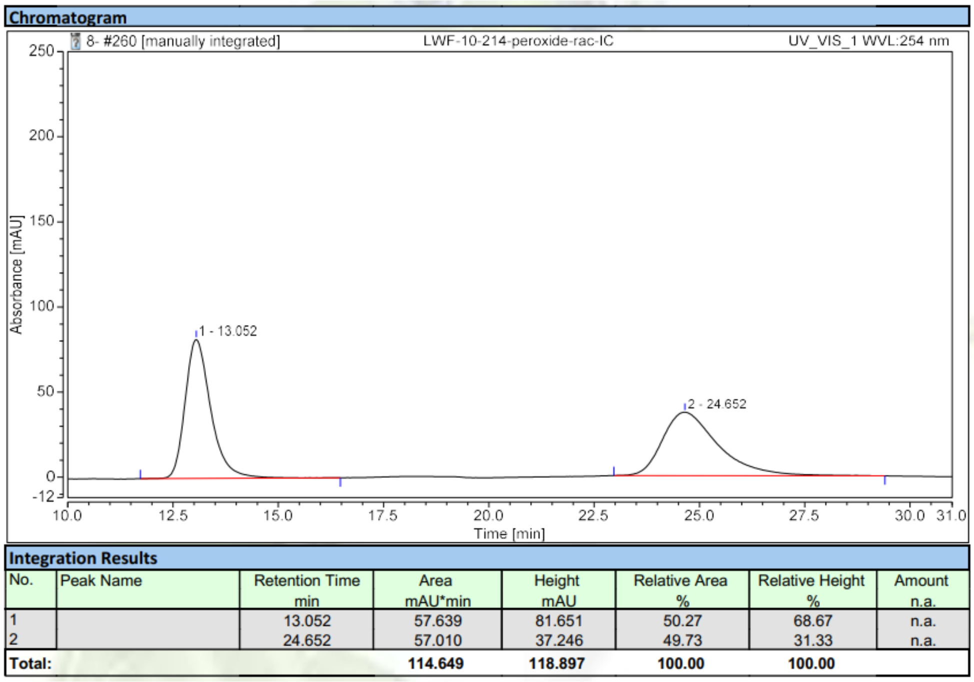

Integration Results

No.	Peak Name	Retention Time min	Area mAU*min	Height mAU	Relative Area %	Relative Height %	Amount n.a.
1		13.052	57.639	81.651	50.27	68.67	n.a.
2		24.652	57.010	37.246	49.73	31.33	n.a.
**Total:**			**114.649**	**118.897**	**100.00**	**100.00**	

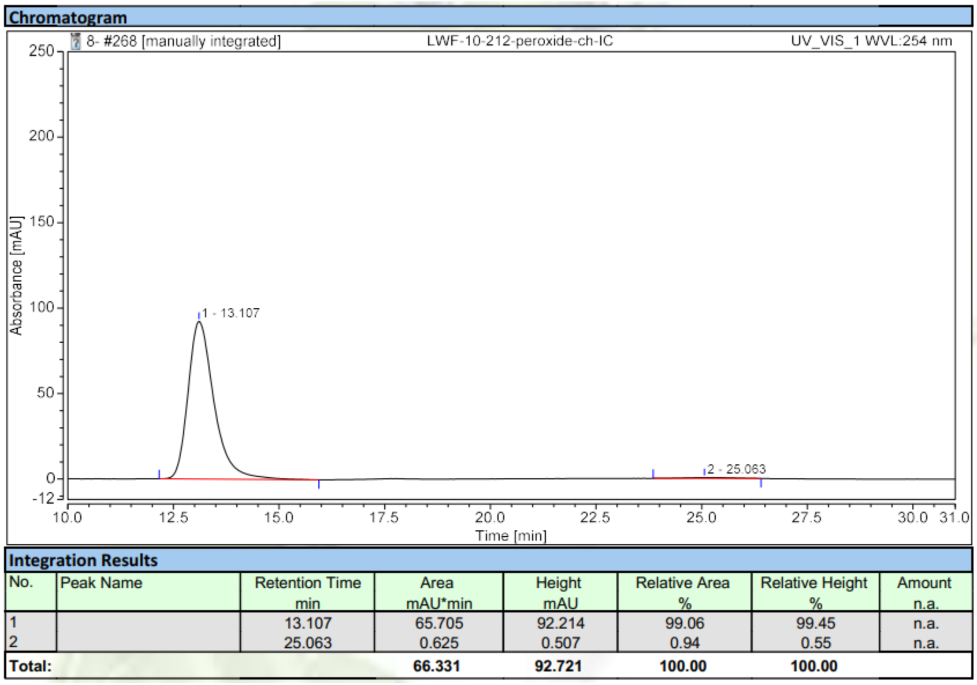

Integration Results

No.	Peak Name	Retention Time min	Area mAU*min	Height mAU	Relative Area %	Relative Height %	Amount n.a.
1		13.107	65.705	92.214	99.06	99.45	n.a.
2		25.063	0.625	0.507	0.94	0.55	n.a.
**Total:**			**66.331**	**92.721**	**100.00**	**100.00**	

Compound C15: IB, *i*-PrOH/hexane = 50/50, v = 1.0 mL/min, λ = 254 nm

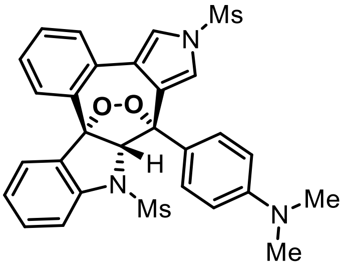

Chromatogram

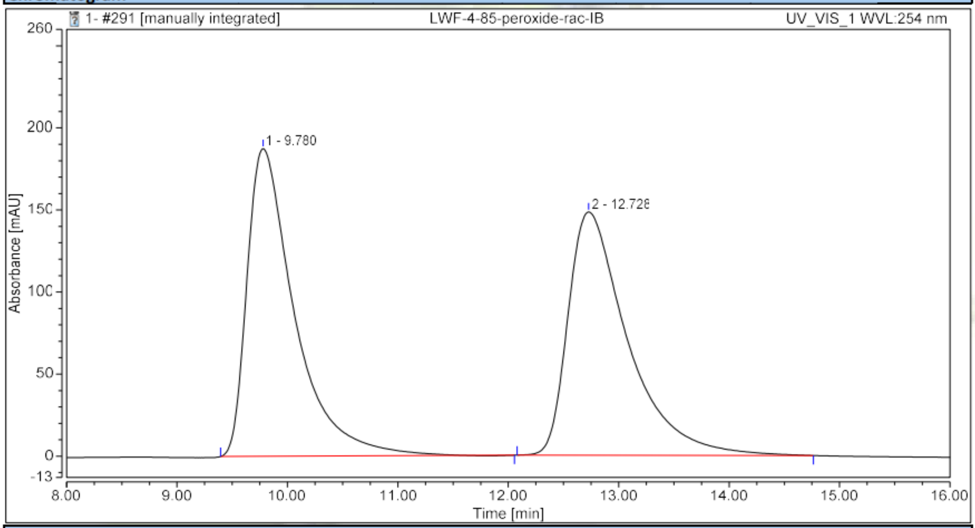

Integration Results

No.	Peak Name	Retention Time<br>min	Area<br>mAU*min	Height<br>mAU	Relative Area<br>%	Relative Height<br>%	Amount<br>n.a.
1		9.780	93.090	187.462	50.17	55.85	n.a.
2		12.728	92.465	148.162	49.83	44.15	n.a.
**Total:**			**185.555**	**335.624**	**100.00**	**100.00**	

Chromatogram

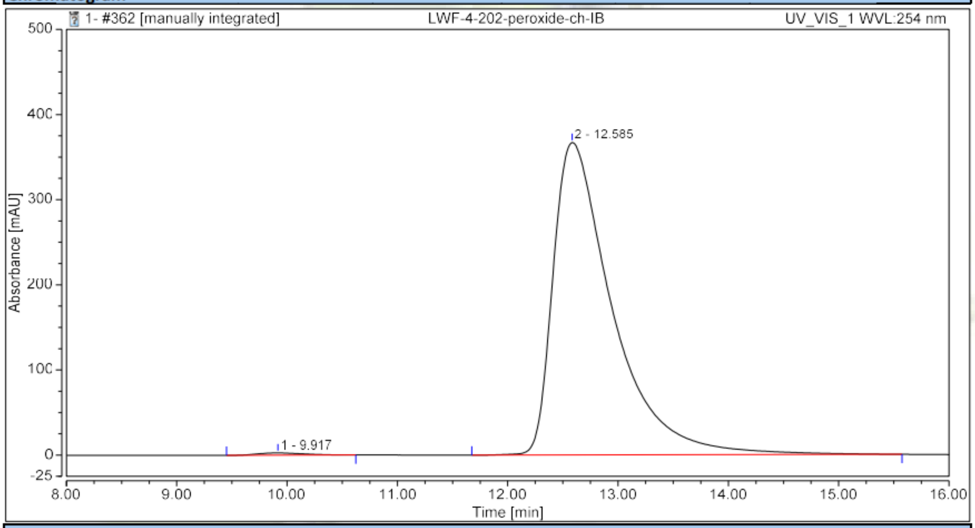

Integration Results

No.	Peak Name	Retention Time<br>min	Area<br>mAU*min	Height<br>mAU	Relative Area<br>%	Relative Height<br>%	Amount<br>n.a.
1		9.917	1.265	2.666	0.55	0.72	n.a.
2		12.585	228.723	367.081	99.45	99.28	n.a.
**Total:**			**229.988**	**369.746**	**100.00**	**100.00**	

Compound C16: IB, *i*-PrOH/hexane = 50/50, v = 1.0 mL/min, λ = 254 nm

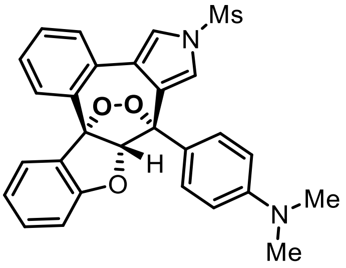

Chromatogram

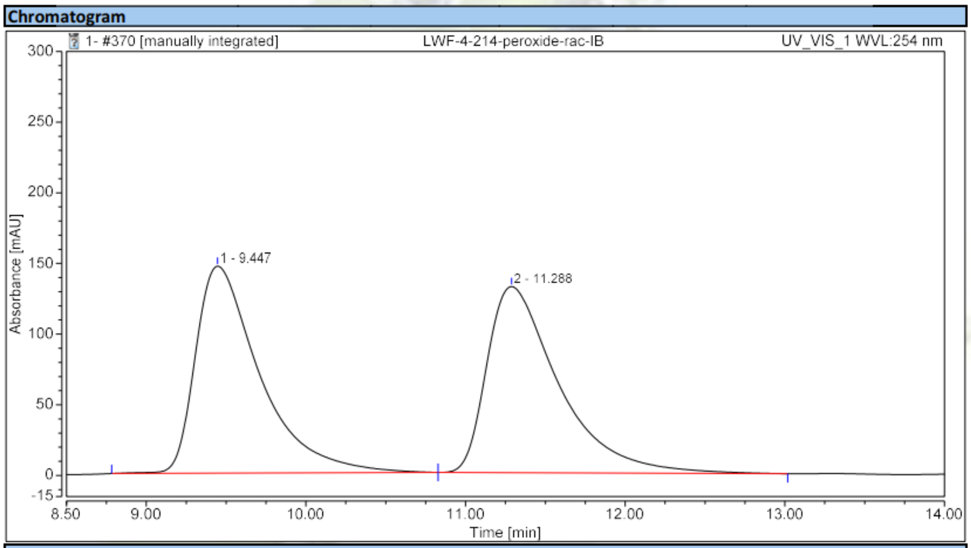

Integration Results

No.	Peak Name	Retention Time min	Area mAU*min	Height mAU	Relative Area %	Relative Height %	Amount n.a.
1		9.447	68.232	146.423	49.94	52.66	n.a.
2		11.288	68.405	131.615	50.06	47.34	n.a.
**Total:**			**136.638**	**278.038**	**100.00**	**100.00**	

Chromatogram

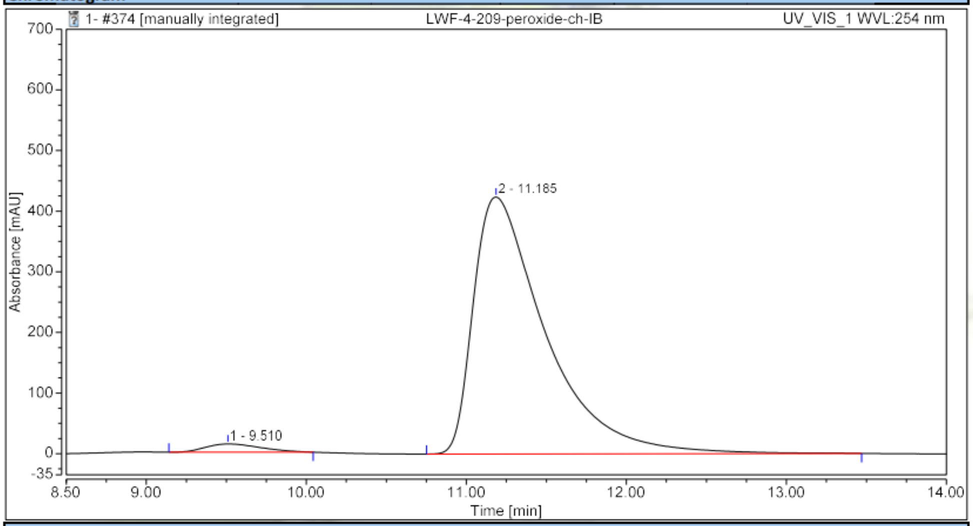

Integration Results

No.	Peak Name	Retention Time min	Area mAU*min	Height mAU	Relative Area %	Relative Height %	Amount n.a.
1		9.510	5.474	13.663	2.42	3.12	n.a.
2		11.185	220.919	423.898	97.58	96.88	n.a.
**Total:**			**226.394**	**437.561**	**100.00**	**100.00**	

Compound C17: IB, *i*-PrOH/hexane = 50/50, v = 1.0 mL/min, λ = 254 nm

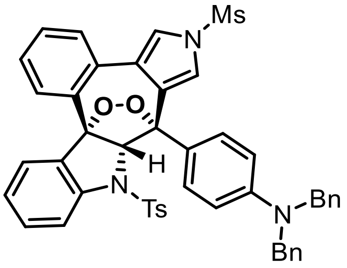

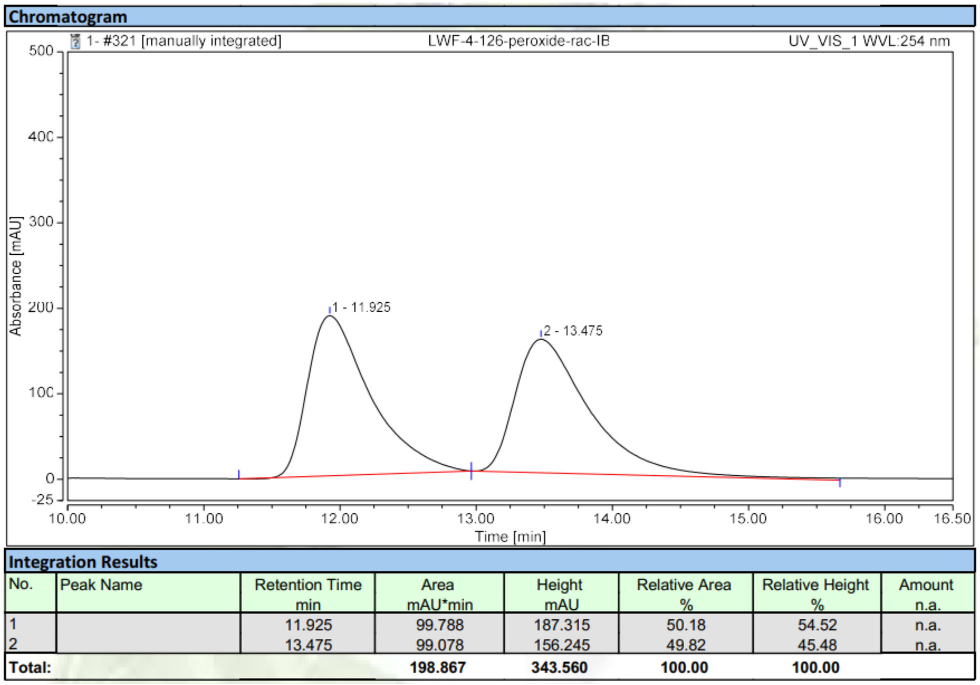

Integration Results

No.	Peak Name	Retention Time min	Area mAU*min	Height mAU	Relative Area %	Relative Height %	Amount n.a.
1		11.925	99.788	187.315	50.18	54.52	n.a.
2		13.475	99.078	156.245	49.82	45.48	n.a.
**Total:**			**198.867**	**343.560**	**100.00**	**100.00**	

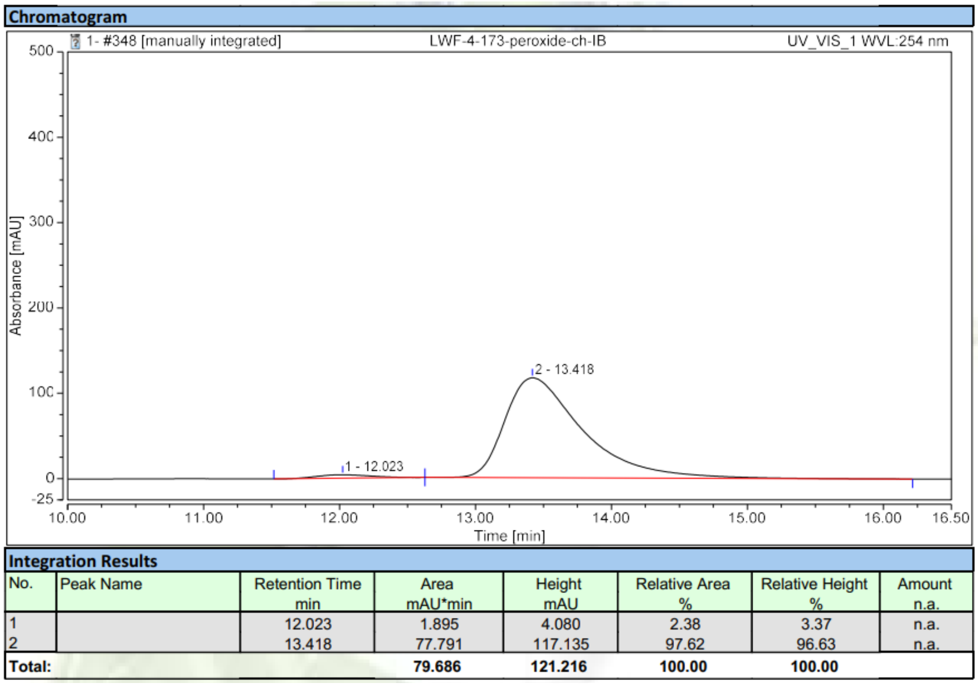

Integration Results

No.	Peak Name	Retention Time min	Area mAU*min	Height mAU	Relative Area %	Relative Height %	Amount n.a.
1		12.023	1.895	4.080	2.38	3.37	n.a.
2		13.418	77.791	117.135	97.62	96.63	n.a.
**Total:**			**79.686**	**121.216**	**100.00**	**100.00**	

Compound D1: IE, *i*-PrOH/hexane = 30/70, v = 1.0 mL/min, λ = 254 nm

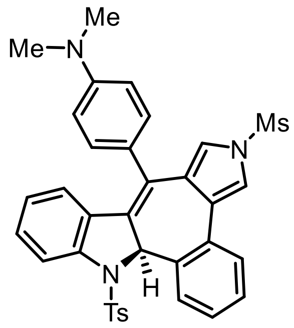

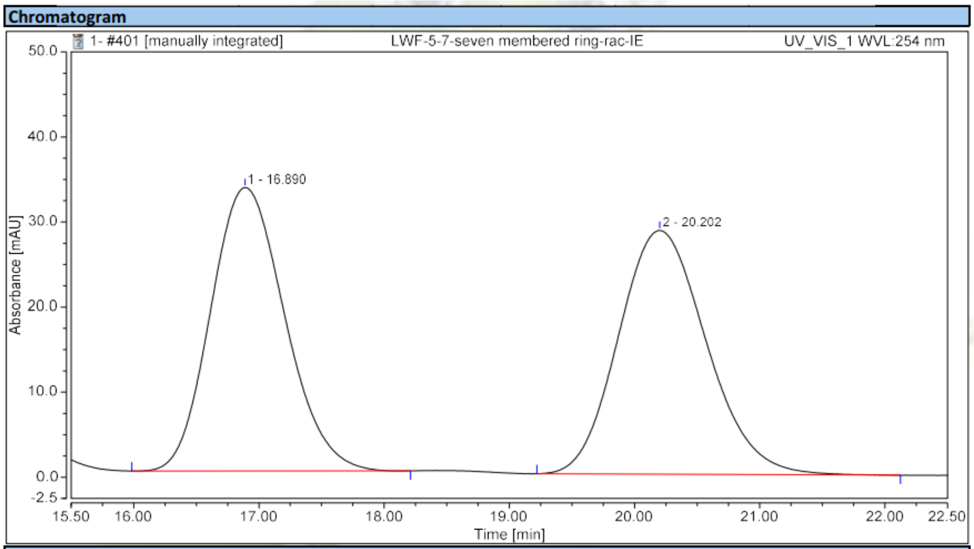

Integration Results

No.	Peak Name	Retention Time min	Area mAU*min	Height mAU	Relative Area %	Relative Height %	Amount n.a.
1		16.890	22.817	33.309	49.50	53.76	n.a.
2		20.202	23.279	28.654	50.50	46.24	n.a.
**Total:**			**46.096**	**61.962**	**100.00**	**100.00**	

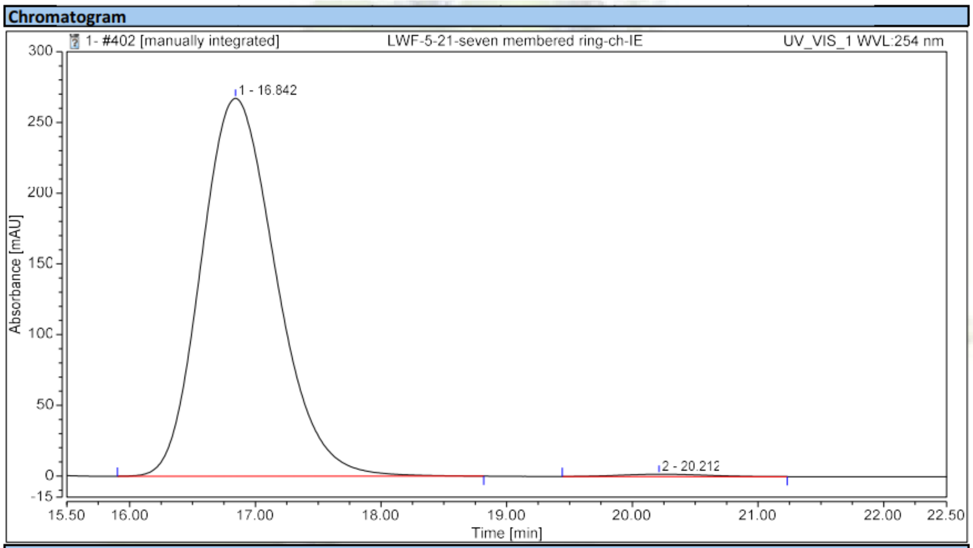

Integration Results

No.	Peak Name	Retention Time min	Area mAU*min	Height mAU	Relative Area %	Relative Height %	Amount n.a.
1		16.842	184.257	267.129	99.32	99.40	n.a.
2		20.212	1.269	1.623	0.68	0.60	n.a.
**Total:**			**185.526**	**268.752**	**100.00**	**100.00**	

Compound D2: IE, *i*-PrOH/hexane = 50/50, v = 1.0 mL/min, λ = 254 nm

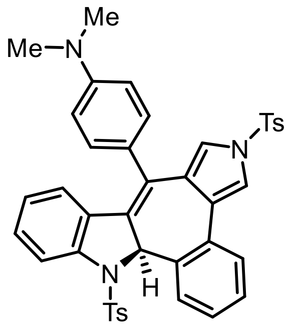

Chromatogram

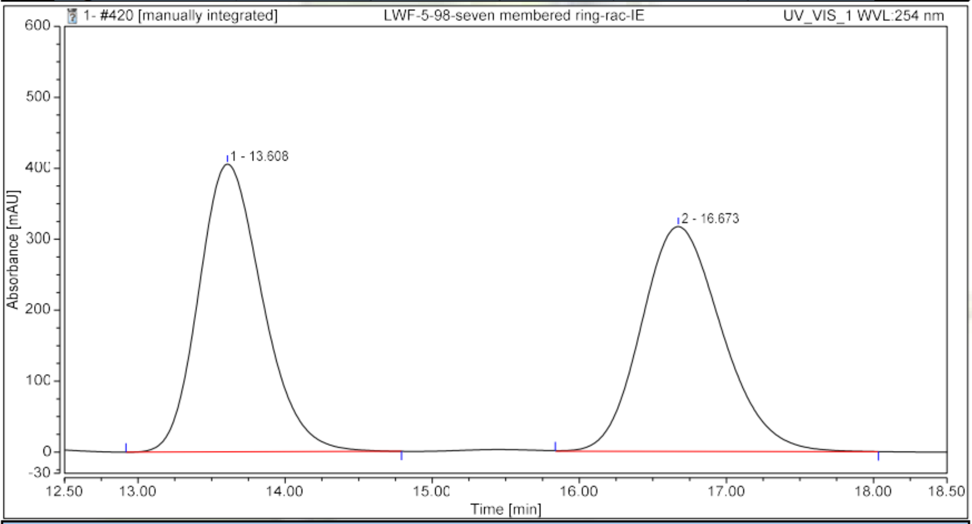

Integration Results

No.	Peak Name	Retention Time min	Area mAU*min	Height mAU	Relative Area %	Relative Height %	Amount n.a.
1		13.608	203.694	405.599	50.22	56.13	n.a.
2		16.673	201.921	316.971	49.78	43.87	n.a.
**Total:**			**405.615**	**722.570**	**100.00**	**100.00**	

Chromatogram

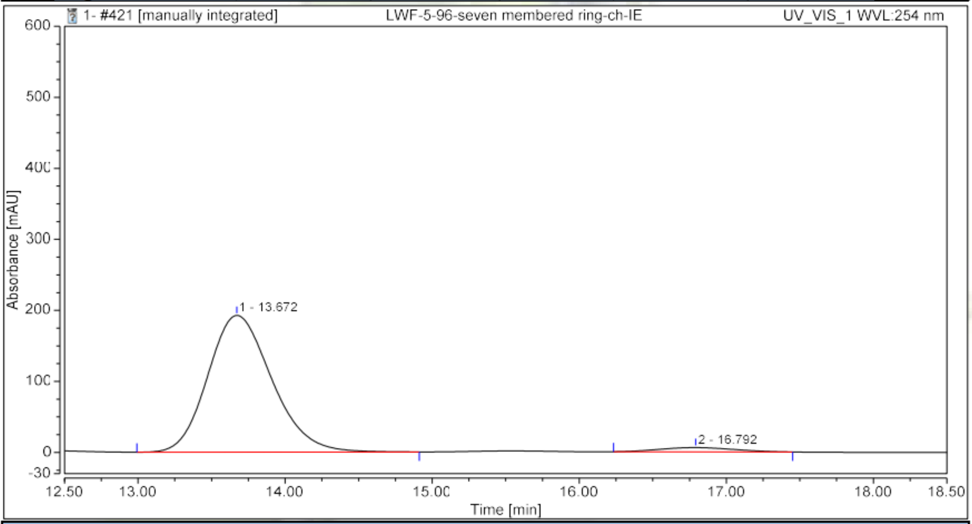

Integration Results

No.	Peak Name	Retention Time min	Area mAU*min	Height mAU	Relative Area %	Relative Height %	Amount n.a.
1		13.672	97.126	192.449	96.50	96.98	n.a.
2		16.792	3.523	6.002	3.50	3.02	n.a.
**Total:**			**100.649**	**198.451**	**100.00**	**100.00**	

Compound D3: IE, *i*-PrOH/hexane = 50/50, v = 1.0 mL/min, λ = 254 nm

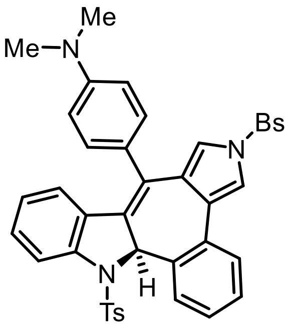

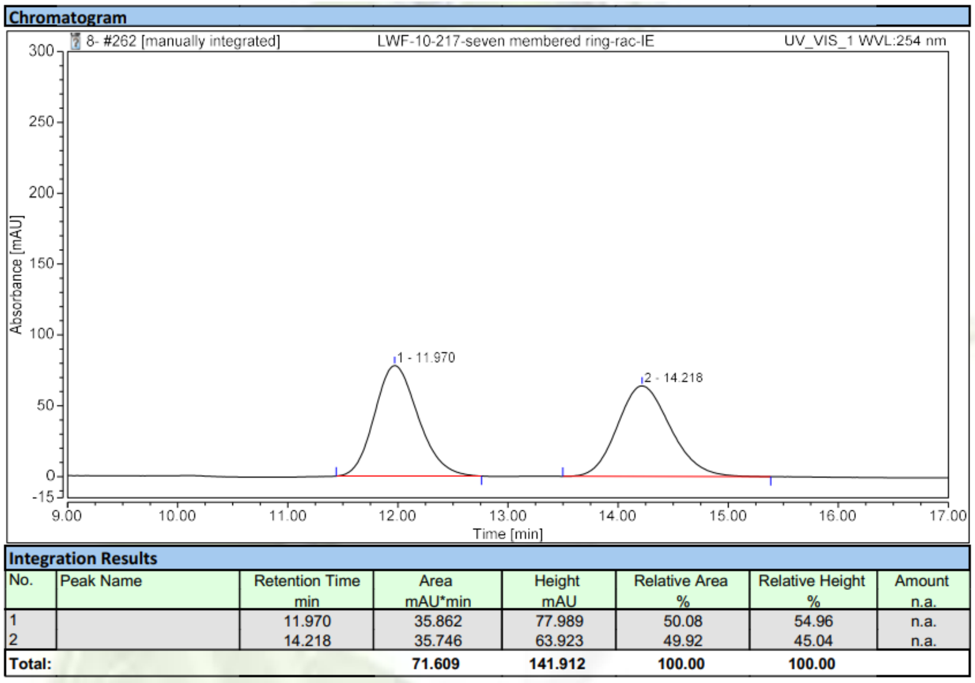

Integration Results

No.	Peak Name	Retention Time min	Area mAU*min	Height mAU	Relative Area %	Relative Height %	Amount n.a.
1		11.970	35.862	77.989	50.08	54.96	n.a.
2		14.218	35.746	63.923	49.92	45.04	n.a.
**Total:**			**71.609**	**141.912**	**100.00**	**100.00**	

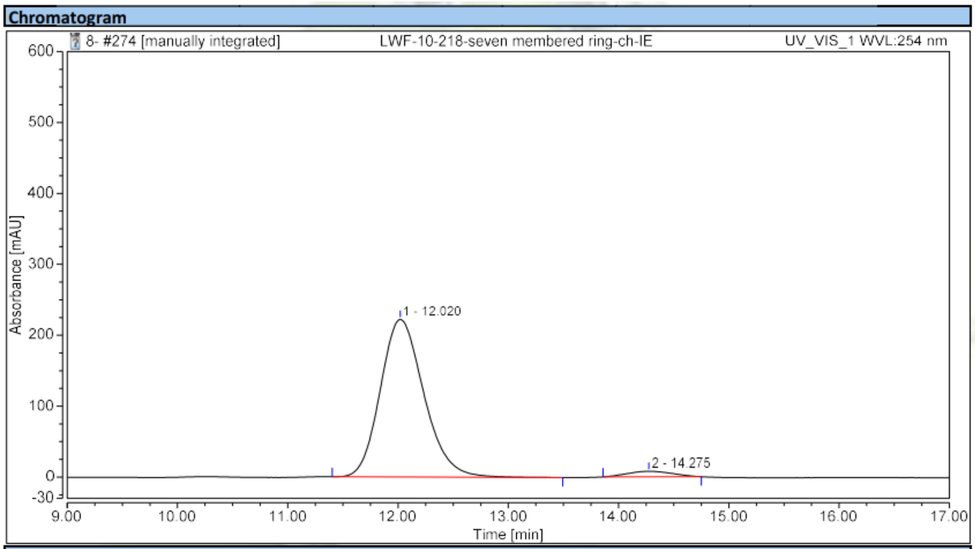

Integration Results

No.	Peak Name	Retention Time min	Area mAU*min	Height mAU	Relative Area %	Relative Height %	Amount n.a.
1		12.020	101.610	222.112	96.53	96.62	n.a.
2		14.275	3.649	7.767	3.47	3.38	n.a.
**Total:**			**105.259**	**229.879**	**100.00**	**100.00**	

Compound D4: IC, *i*-PrOH/hexane = 50/50, v = 1.0 mL/min, λ = 254 nm

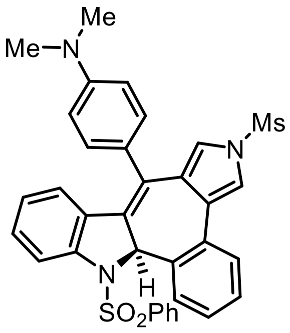

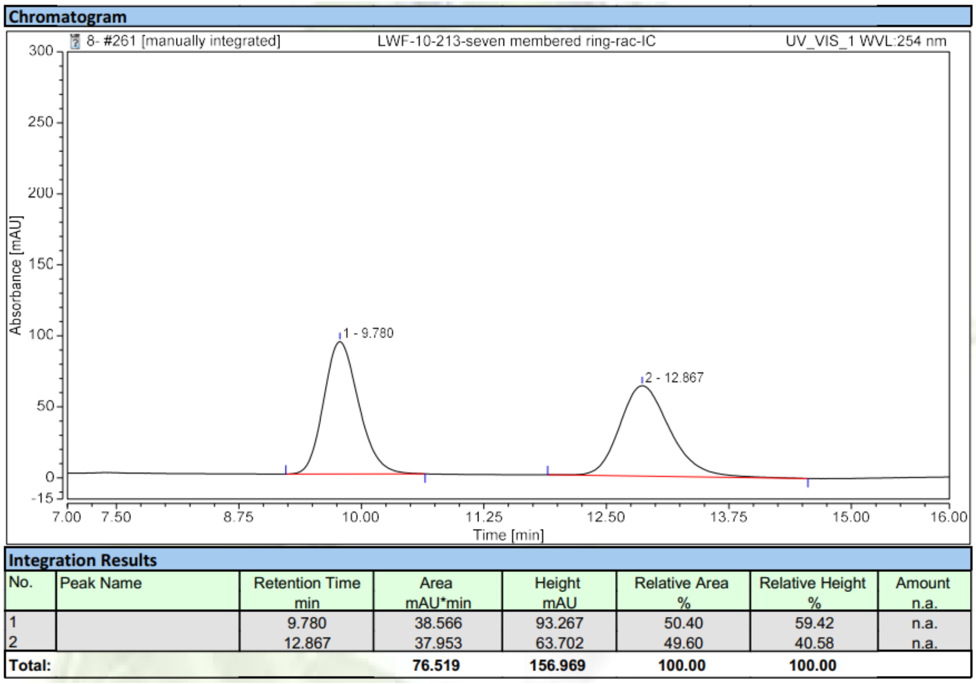

Integration Results

No.	Peak Name	Retention Time min	Area mAU*min	Height mAU	Relative Area %	Relative Height %	Amount n.a.
1		9.780	38.566	93.267	50.40	59.42	n.a.
2		12.867	37.953	63.702	49.60	40.58	n.a.
**Total:**			**76.519**	**156.969**	**100.00**	**100.00**	

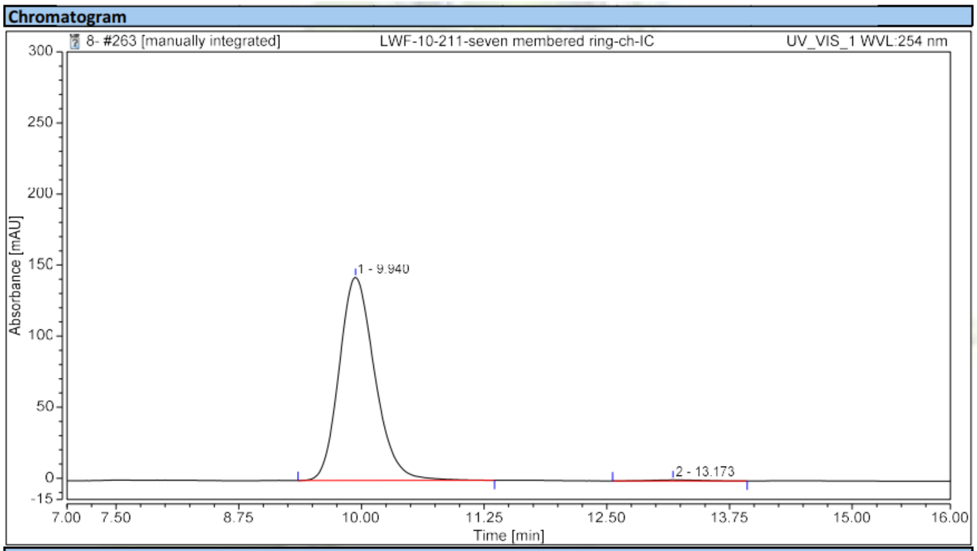

Integration Results

No.	Peak Name	Retention Time min	Area mAU*min	Height mAU	Relative Area %	Relative Height %	Amount n.a.
1		9.940	60.152	142.861	99.39	99.49	n.a.
2		13.173	0.368	0.729	0.61	0.51	n.a.
**Total:**			**60.520**	**143.590**	**100.00**	**100.00**	

Compound D5: IE, *i*-PrOH/hexane = 50/50, v = 1.0 mL/min, λ = 254 nm

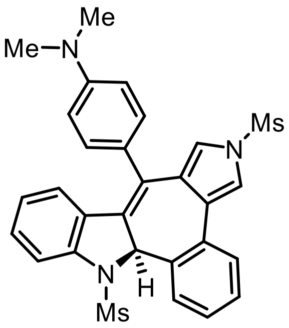

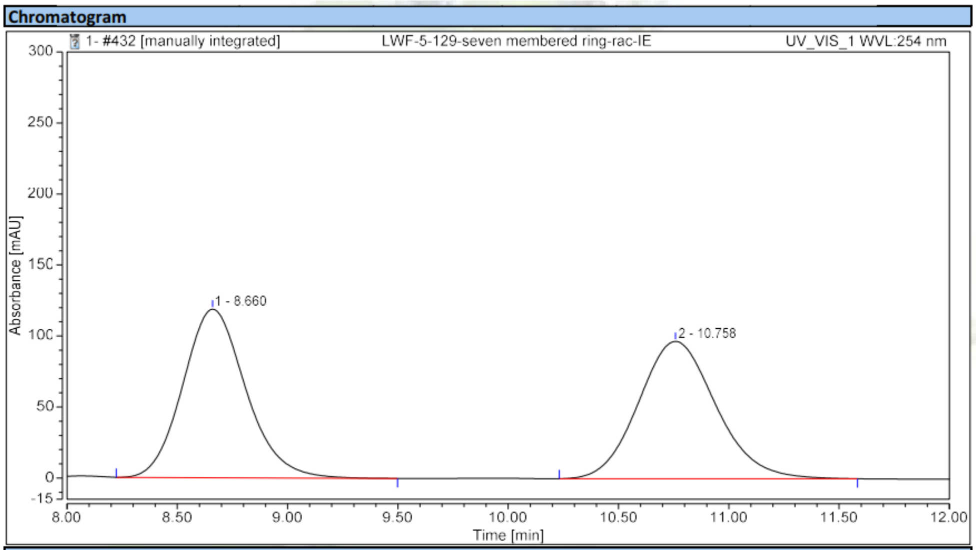

Integration Results

No.	Peak Name	Retention Time min	Area mAU*min	Height mAU	Relative Area %	Relative Height %	Amount n.a.
1		8.660	39.305	118.611	49.93	55.09	n.a.
2		10.758	39.419	96.696	50.07	44.91	n.a.
**Total:**			**78.724**	**215.307**	**100.00**	**100.00**	

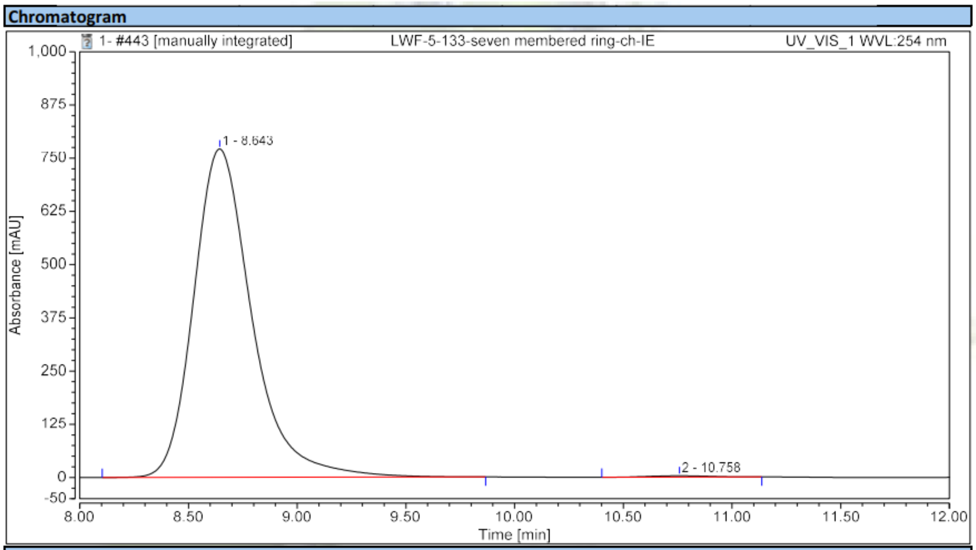

Integration Results

No.	Peak Name	Retention Time min	Area mAU*min	Height mAU	Relative Area %	Relative Height %	Amount n.a.
1		8.643	239.396	771.386	99.58	99.59	n.a.
2		10.758	1.019	3.165	0.42	0.41	n.a.
**Total:**			**240.415**	**774.551**	**100.00**	**100.00**	

Compound D6: IE, *i*-PrOH/hexane = 50/50, v = 1.0 mL/min, λ = 254 nm

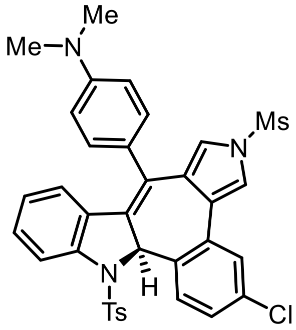

Chromatogram

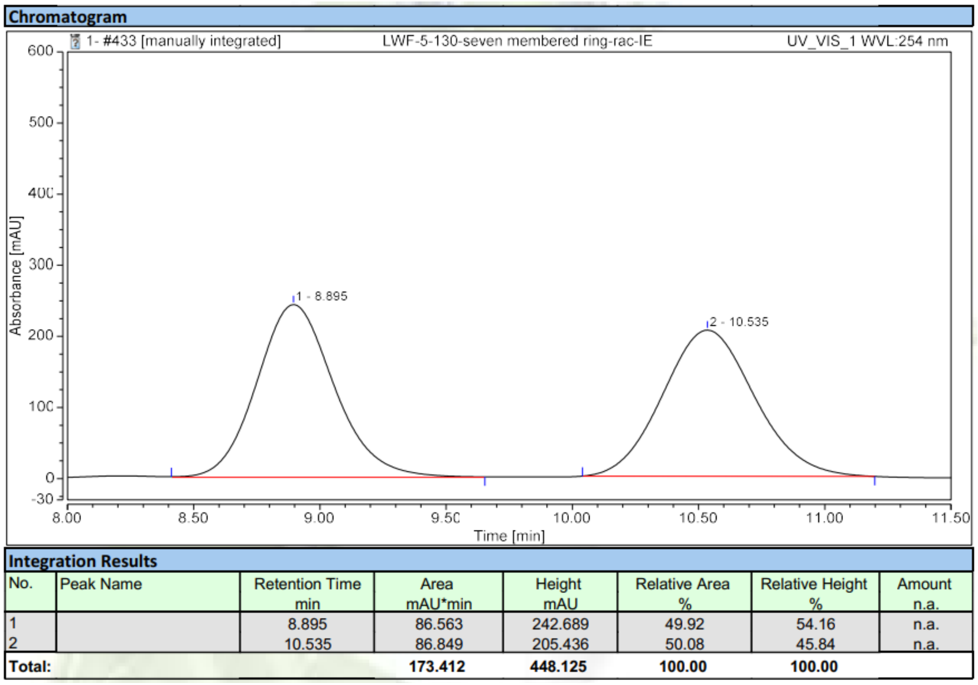

Integration Results

No.	Peak Name	Retention Time min	Area mAU*min	Height mAU	Relative Area %	Relative Height %	Amount n.a.
1		8.895	86.563	242.689	49.92	54.16	n.a.
2		10.535	86.849	205.436	50.08	45.84	n.a.
**Total:**			**173.412**	**448.125**	**100.00**	**100.00**	

Chromatogram

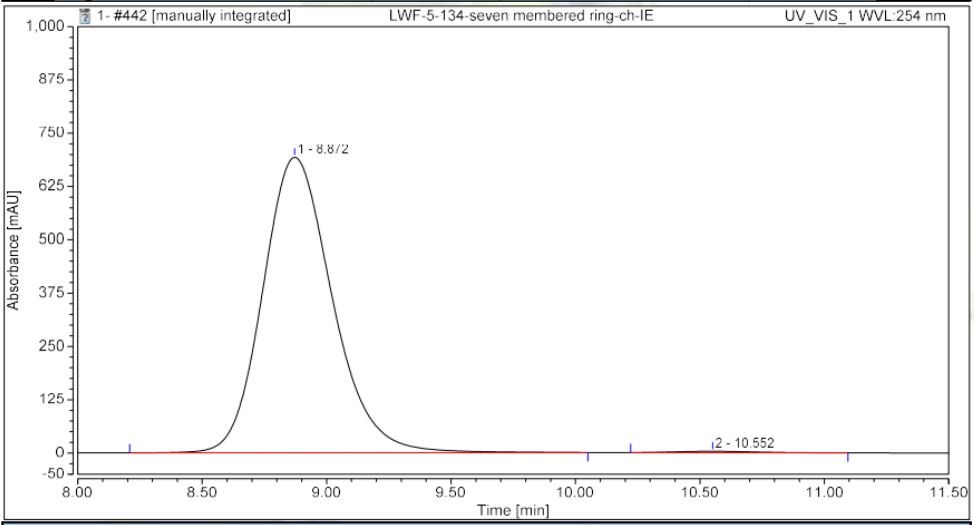

Integration Results

No.	Peak Name	Retention Time min	Area mAU*min	Height mAU	Relative Area %	Relative Height %	Amount n.a.
1		8.872	224.819	692.857	99.46	99.52	n.a.
2		10.552	1.230	3.315	0.54	0.48	n.a.
**Total:**			**226.049**	**696.172**	**100.00**	**100.00**	

Compound D7: IE, *i*-PrOH/hexane = 50/50, v = 1.0 mL/min, λ = 254 nm

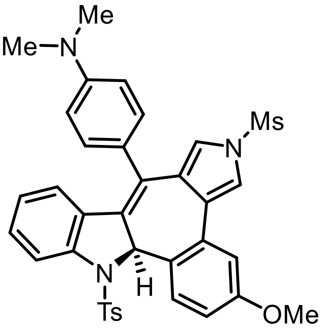

Chromatogram

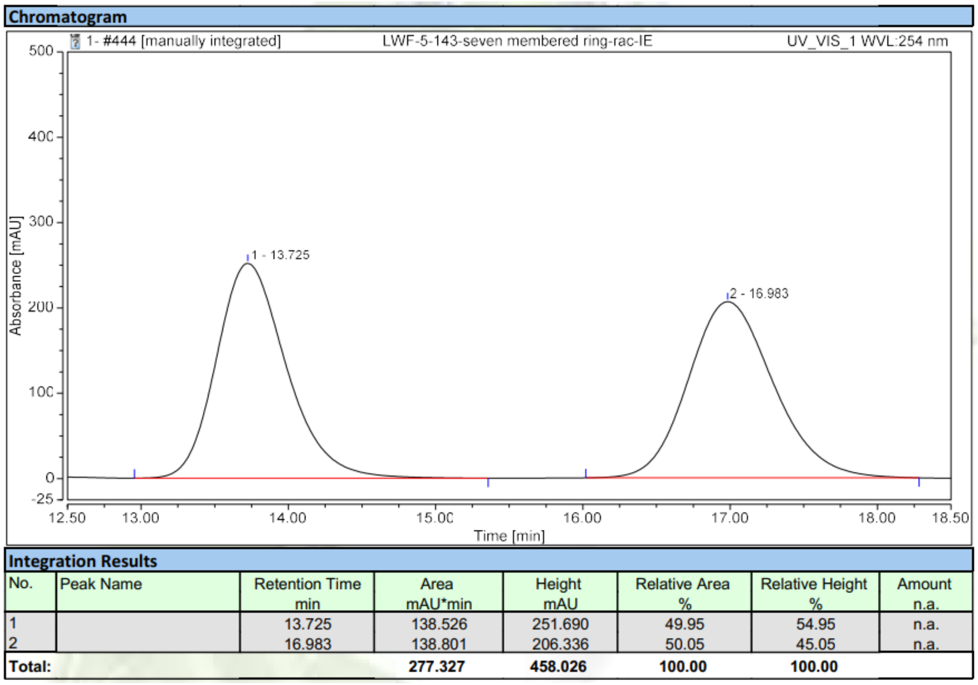

Integration Results

No.	Peak Name	Retention Time min	Area mAU*min	Height mAU	Relative Area %	Relative Height %	Amount n.a.
1		13.725	138.526	251.690	49.95	54.95	n.a.
2		16.983	138.801	206.336	50.05	45.05	n.a.
**Total:**			**277.327**	**458.026**	**100.00**	**100.00**	

Chromatogram

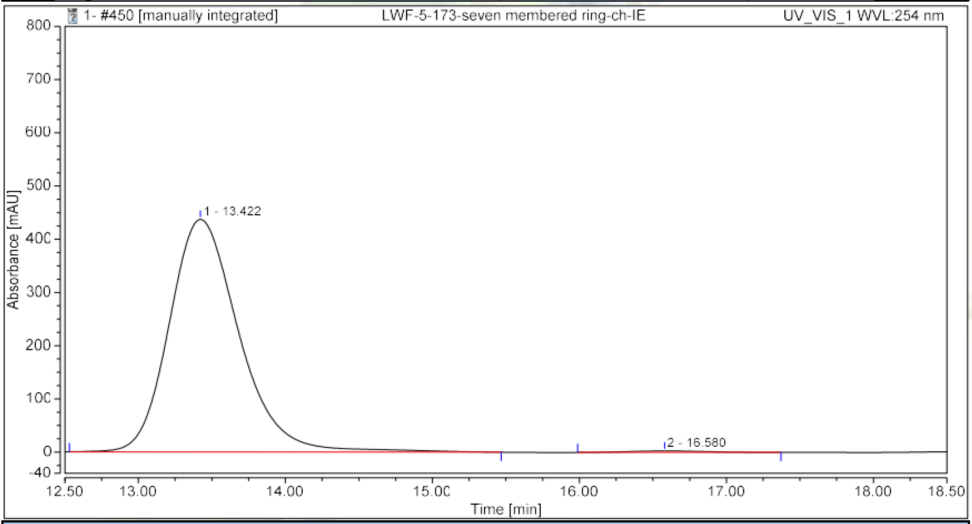

Integration Results

No.	Peak Name	Retention Time min	Area mAU*min	Height mAU	Relative Area %	Relative Height %	Amount n.a.
1		13.422	240.365	437.089	99.35	99.40	n.a.
2		16.580	1.577	2.632	0.65	0.60	n.a.
**Total:**			**241.942**	**439.721**	**100.00**	**100.00**	

Compound D8: IC, *i*-PrOH/hexane = 30/70, v = 1.0 mL/min, λ = 254 nm

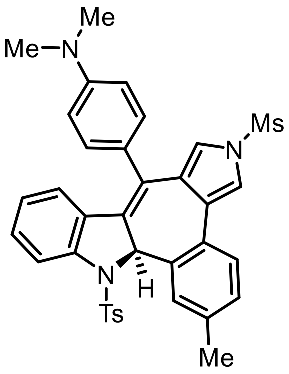

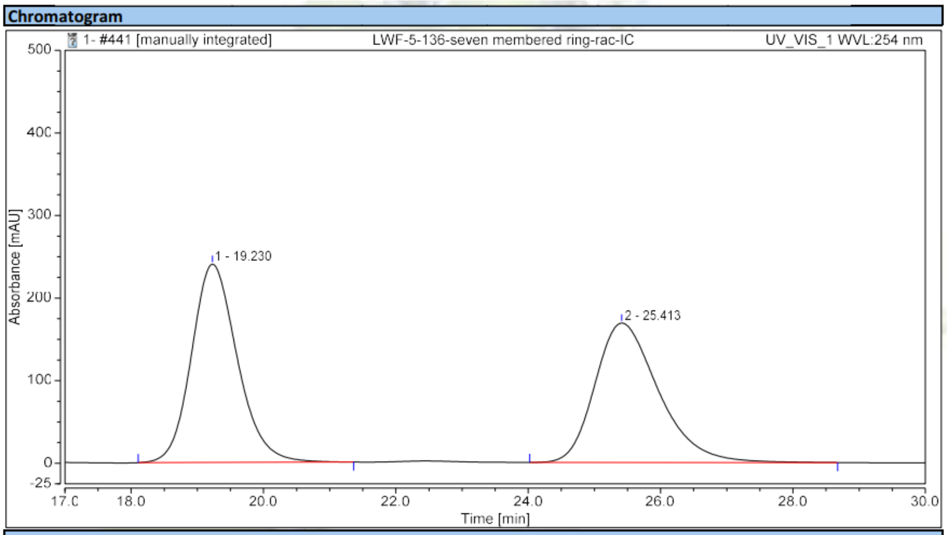

Integration Results

No.	Peak Name	Retention Time min	Area mAU*min	Height mAU	Relative Area %	Relative Height %	Amount n.a.
1		19.230	192.663	240.133	50.65	58.73	n.a.
2		25.413	187.735	168.754	49.35	41.27	n.a.
**Total:**			**380.397**	**408.887**	**100.00**	**100.00**	

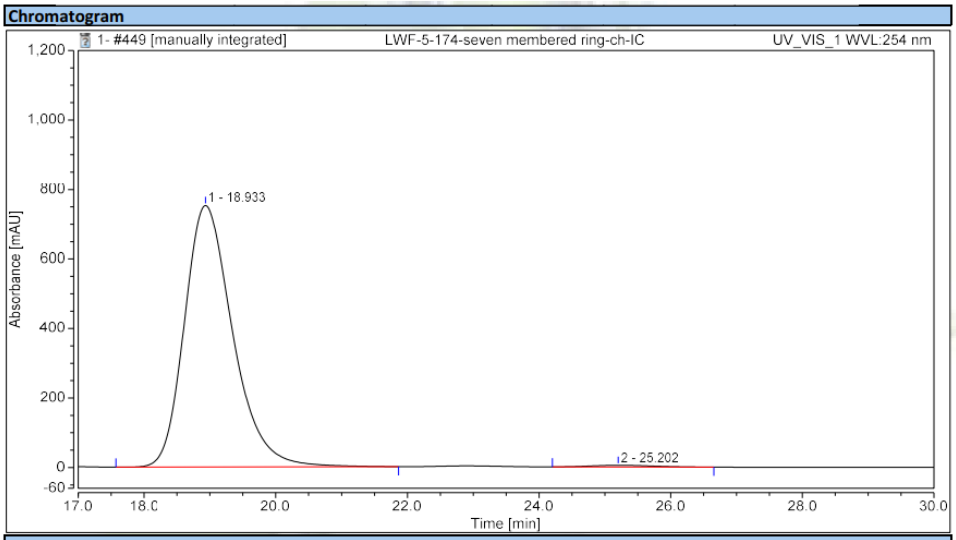

Integration Results

No.	Peak Name	Retention Time min	Area mAU*min	Height mAU	Relative Area %	Relative Height %	Amount n.a.
1		18.933	640.343	752.584	99.17	99.35	n.a.
2		25.202	5.350	4.941	0.83	0.65	n.a.
**Total:**			**645.694**	**757.525**	**100.00**	**100.00**	

Compound D9: IE, *i*-PrOH/hexane = 30/70, v = 1.0 mL/min, λ = 254 nm

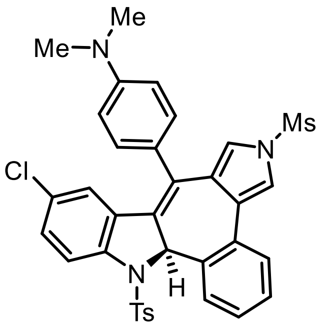

Chromatogram

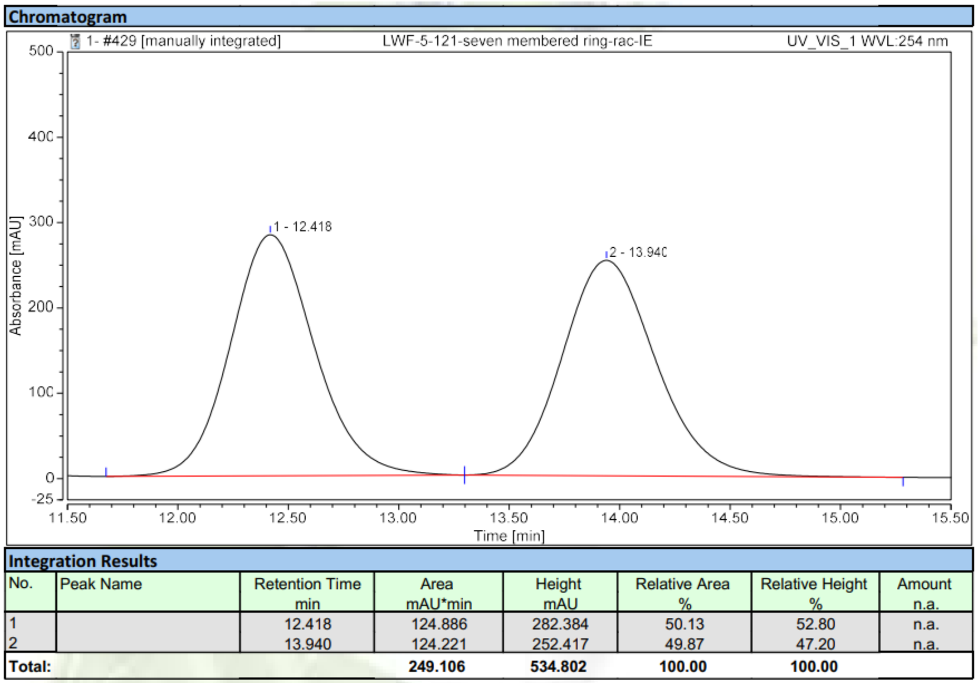

Integration Results

No.	Peak Name	Retention Time min	Area mAU*min	Height mAU	Relative Area %	Relative Height %	Amount n.a.
1		12.418	124.886	282.384	50.13	52.80	n.a.
2		13.940	124.221	252.417	49.87	47.20	n.a.
**Total:**			**249.106**	**534.802**	**100.00**	**100.00**	

Chromatogram

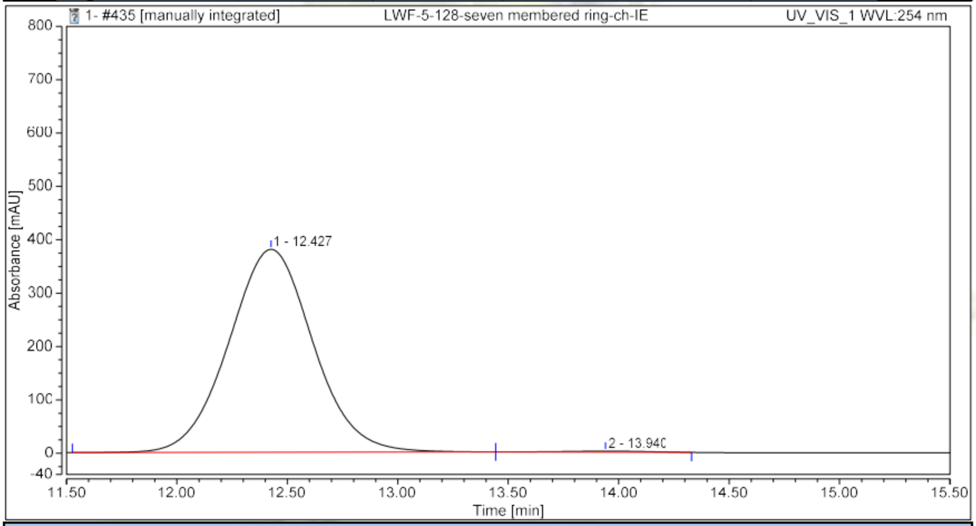

Integration Results

No.	Peak Name	Retention Time min	Area mAU*min	Height mAU	Relative Area %	Relative Height %	Amount n.a.
1		12.427	168.522	380.288	99.51	99.51	n.a.
2		13.940	0.833	1.864	0.49	0.49	n.a.
**Total:**			**169.355**	**382.152**	**100.00**	**100.00**	

Compound D10: IE, *i*-PrOH/hexane = 20/80, v = 1.0 mL/min, λ = 254 nm

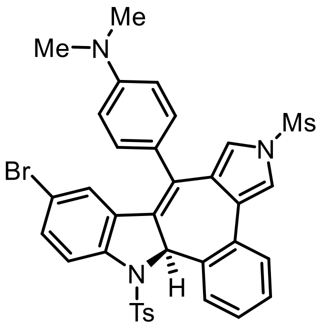

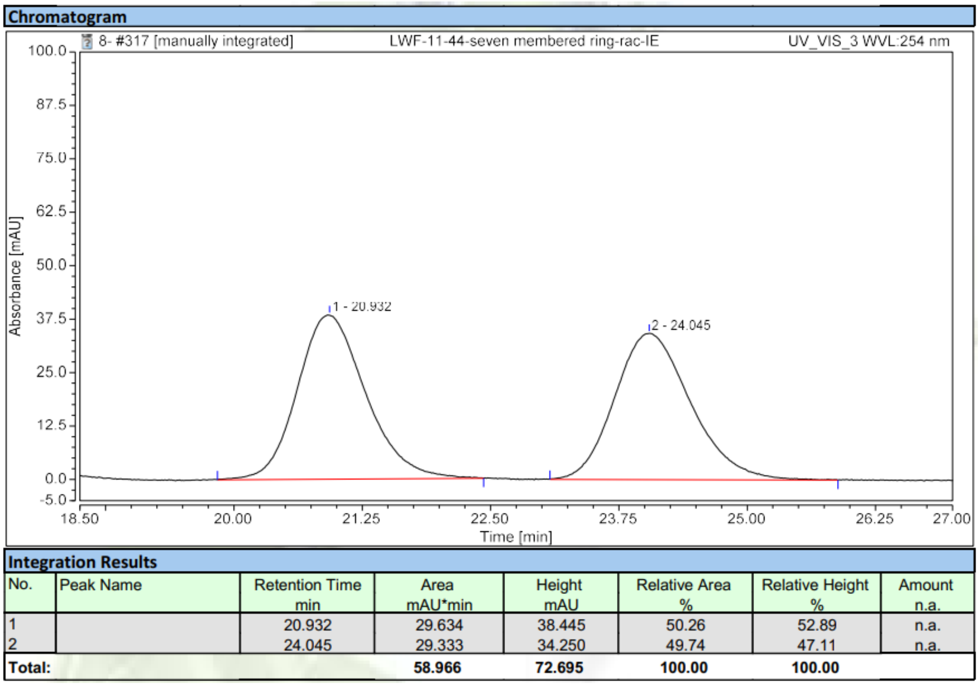

Integration Results

No.	Peak Name	Retention Time min	Area mAU*min	Height mAU	Relative Area %	Relative Height %	Amount n.a.
1		20.932	29.634	38.445	50.26	52.89	n.a.
2		24.045	29.333	34.250	49.74	47.11	n.a.
Total:			58.966	72.695	100.00	100.00	

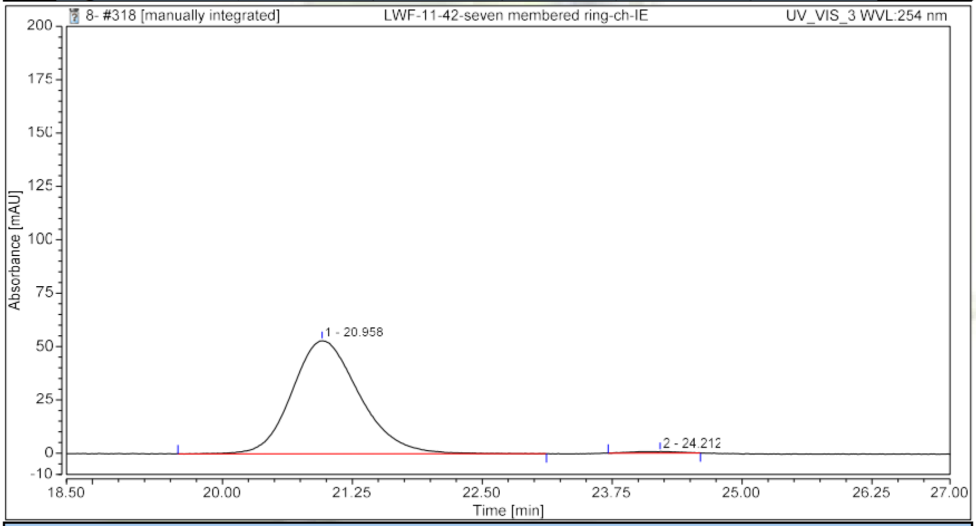

Integration Results

No.	Peak Name	Retention Time min	Area mAU*min	Height mAU	Relative Area %	Relative Height %	Amount n.a.
1		20.958	39.779	53.096	99.07	98.55	n.a.
2		24.212	0.373	0.783	0.93	1.45	n.a.
Total:			40.153	53.879	100.00	100.00	

Compound D11: IE, *i*-PrOH/hexane = 30/70, v = 1.0 mL/min, λ = 254 nm

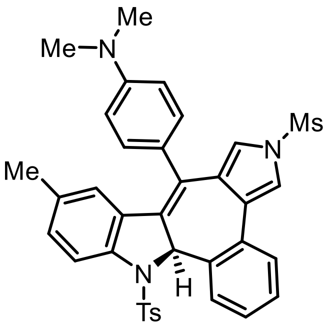

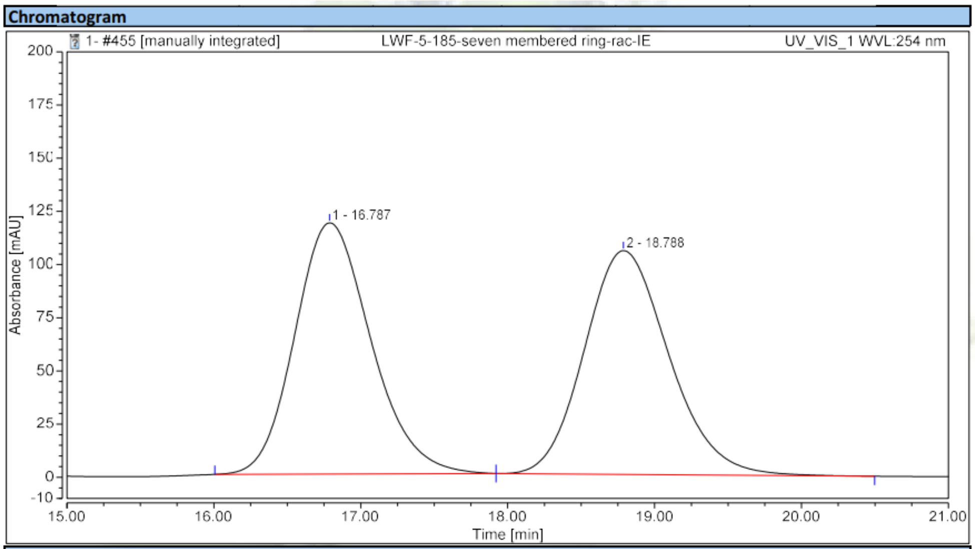

Integration Results

No.	Peak Name	Retention Time min	Area mAU*min	Height mAU	Relative Area %	Relative Height %	Amount n.a.
1		16.787	70.942	118.098	50.27	52.89	n.a.
2		18.788	70.192	105.200	49.73	47.11	n.a.
**Total:**			**141.134**	**223.298**	**100.00**	**100.00**	

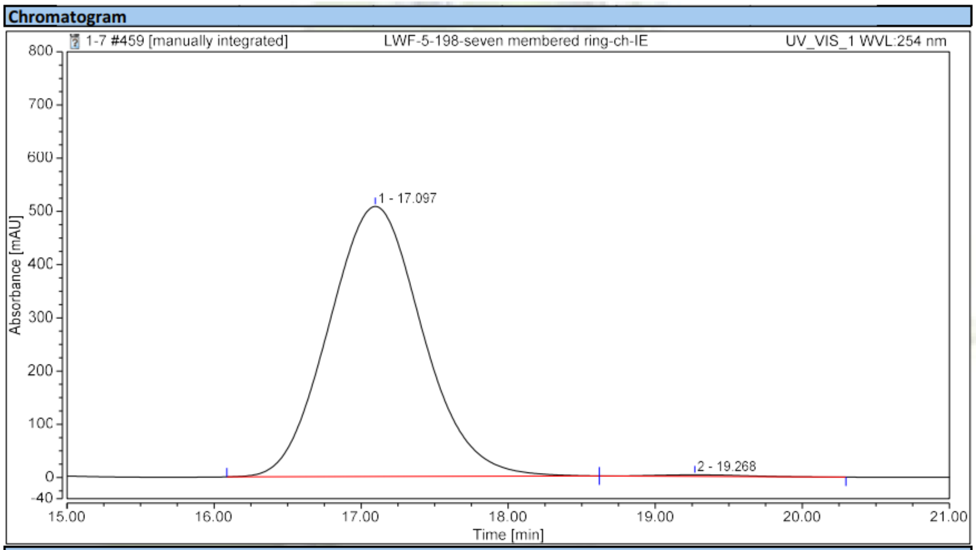

Integration Results

No.	Peak Name	Retention Time min	Area mAU*min	Height mAU	Relative Area %	Relative Height %	Amount n.a.
1		17.097	374.004	507.497	99.47	99.43	n.a.
2		19.268	2.003	2.920	0.53	0.57	n.a.
**Total:**			**376.007**	**510.417**	**100.00**	**100.00**	

Compound D12: IE, *i*-PrOH/hexane = 50/50, v = 1.0 mL/min, λ = 254 nm

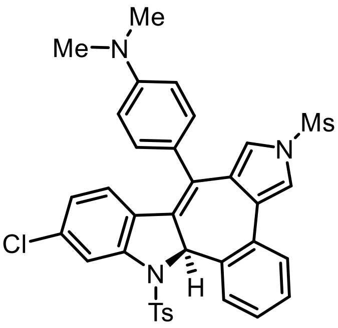

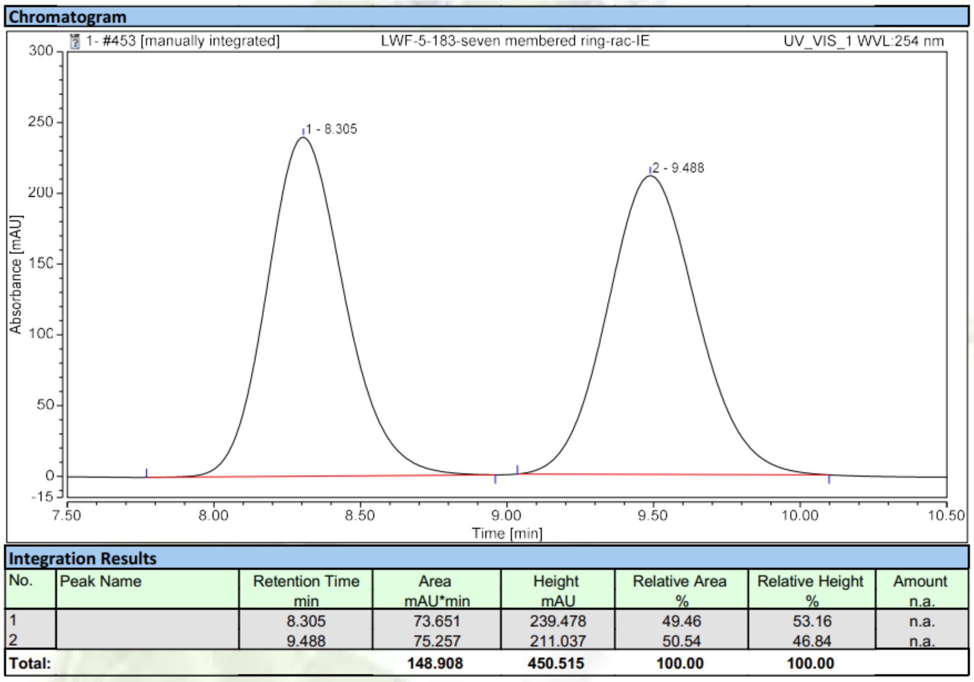

Integration Results

No.	Peak Name	Retention Time min	Area mAU*min	Height mAU	Relative Area %	Relative Height %	Amount n.a.
1		8.305	73.651	239.478	49.46	53.16	n.a.
2		9.488	75.257	211.037	50.54	46.84	n.a.
**Total:**			**148.908**	**450.515**	**100.00**	**100.00**	

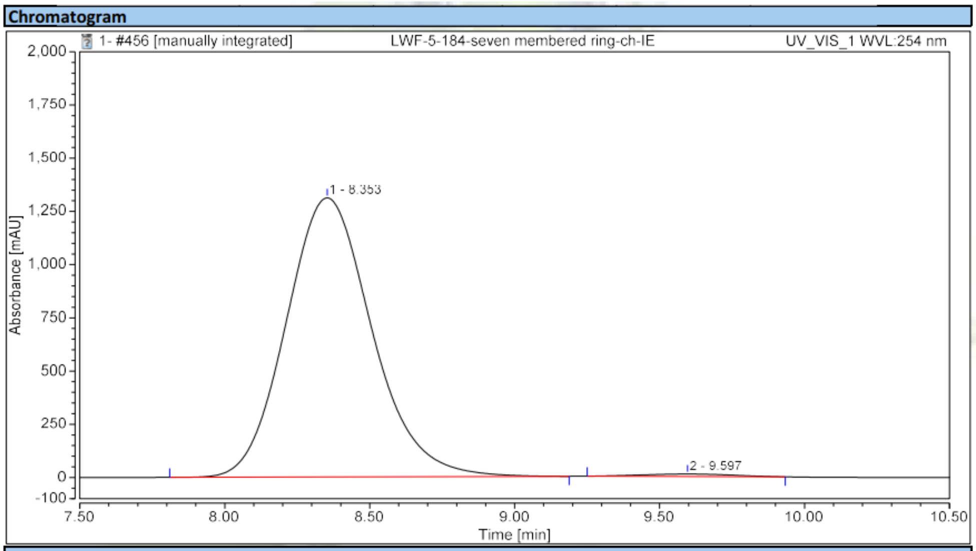

Integration Results

No.	Peak Name	Retention Time min	Area mAU*min	Height mAU	Relative Area %	Relative Height %	Amount n.a.
1		8.353	449.569	1311.212	99.06	99.11	n.a.
2		9.597	4.259	11.767	0.94	0.89	n.a.
**Total:**			**453.828**	**1322.978**	**100.00**	**100.00**	

Compound D13: IE, *i*-PrOH/hexane = 50/50, v = 1.0 mL/min, λ = 220 nm

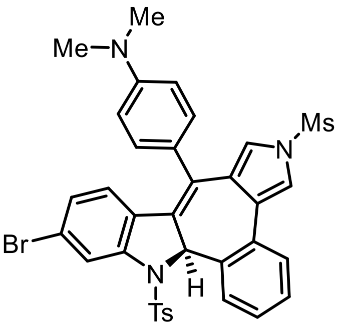

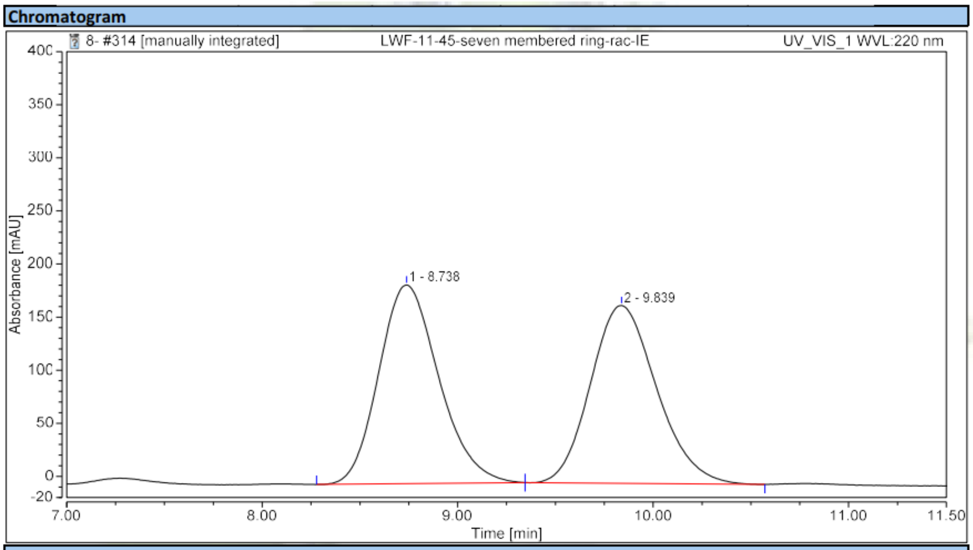

Integration Results

No.	Peak Name	Retention Time min	Area mAU*min	Height mAU	Relative Area %	Relative Height %	Amount n.a.
1		8.738	66.614	187.141	50.35	52.77	n.a.
2		9.839	65.701	167.518	49.65	47.23	n.a.
**Total:**			**132.315**	**354.659**	**100.00**	**100.00**	

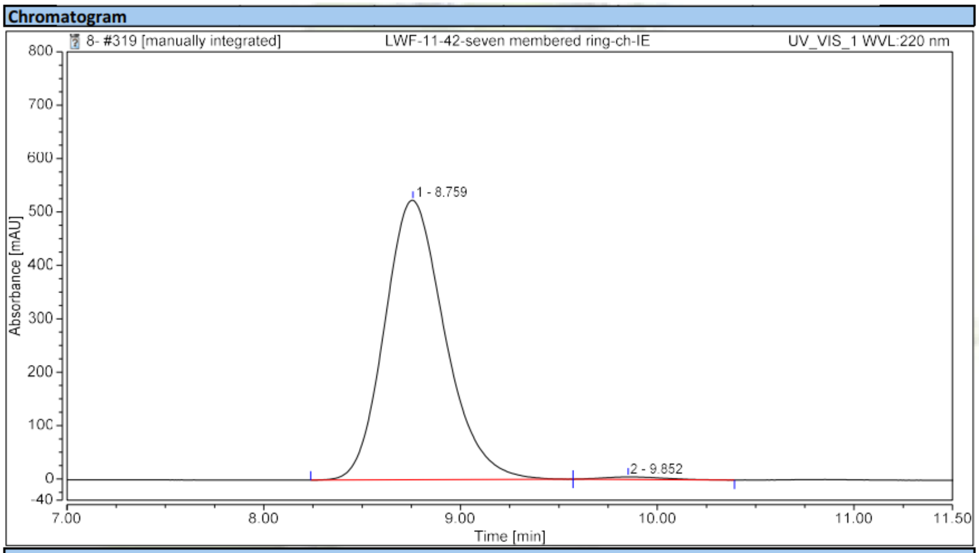

Integration Results

No.	Peak Name	Retention Time min	Area mAU*min	Height mAU	Relative Area %	Relative Height %	Amount n.a.
1		8.759	185.523	523.343	99.14	99.13	n.a.
2		9.852	1.610	4.617	0.86	0.87	n.a.
**Total:**			**187.133**	**527.960**	**100.00**	**100.00**	

Compound D14: IE, *i*-PrOH/hexane = 50/50, v = 1.0 mL/min, λ = 254 nm

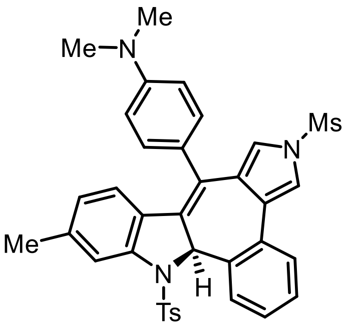

Chromatogram

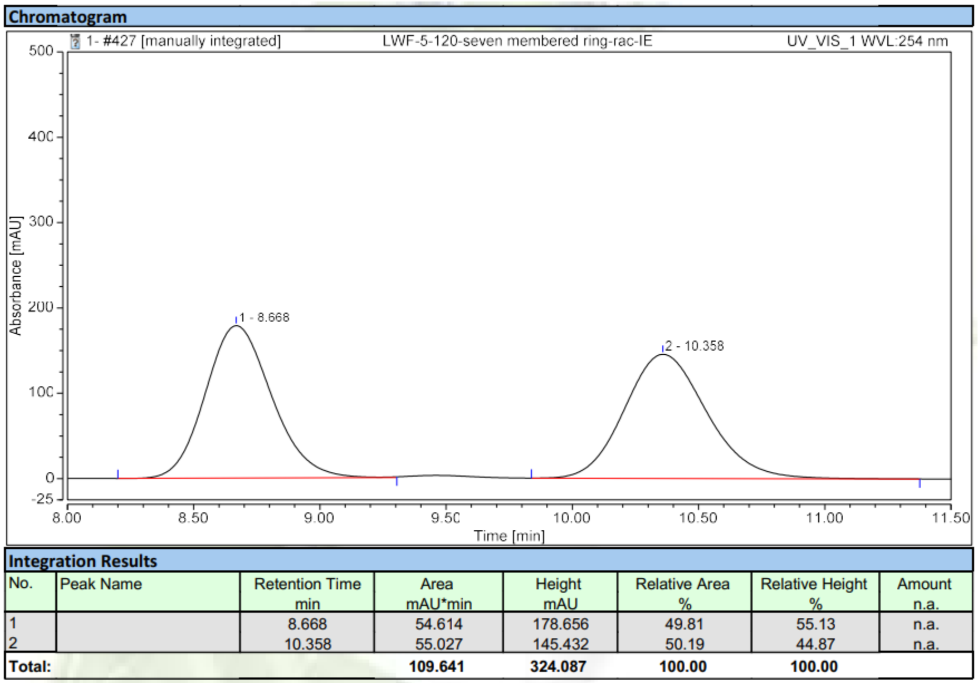

Integration Results

No.	Peak Name	Retention Time min	Area mAU*min	Height mAU	Relative Area %	Relative Height %	Amount n.a.
1		8.668	54.614	178.656	49.81	55.13	n.a.
2		10.358	55.027	145.432	50.19	44.87	n.a.
**Total:**			**109.641**	**324.087**	**100.00**	**100.00**	

Chromatogram

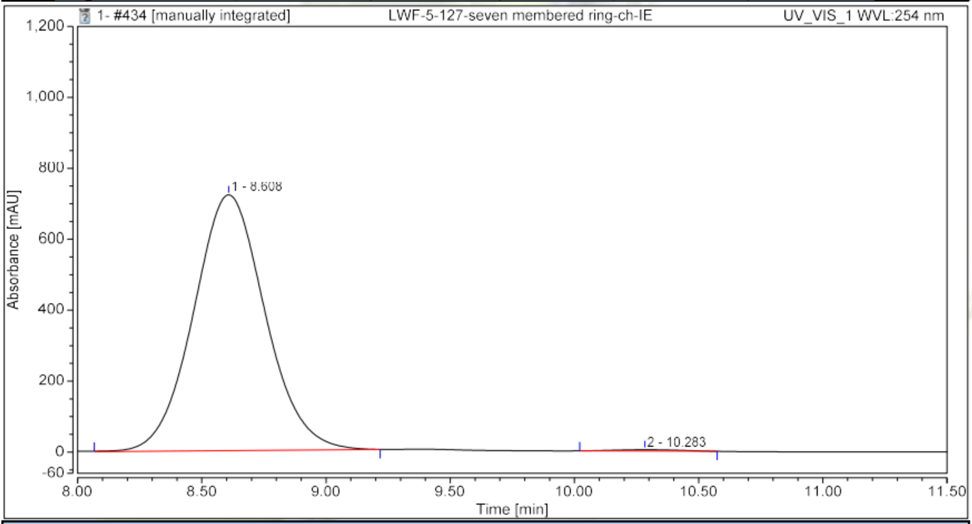

Integration Results

No.	Peak Name	Retention Time min	Area mAU*min	Height mAU	Relative Area %	Relative Height %	Amount n.a.
1		8.608	241.183	721.146	99.52	99.48	n.a.
2		10.283	1.170	3.800	0.48	0.52	n.a.
**Total:**			**242.352**	**724.945**	**100.00**	**100.00**	

Compound D15: IC, *i*-PrOH/hexane = 20/80, v = 1.0 mL/min, λ = 254 nm

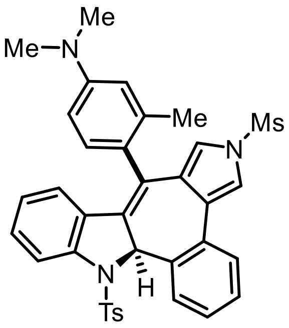

Chromatogram

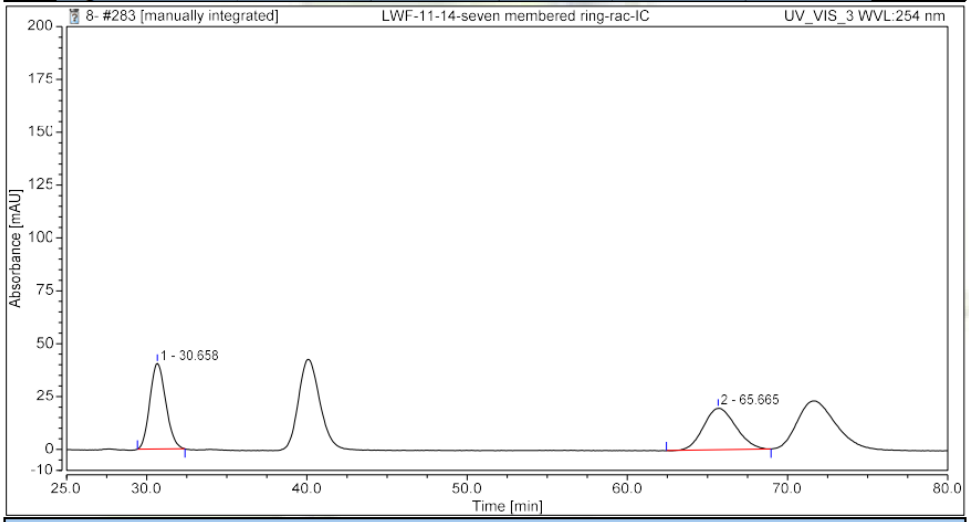

Integration Results

No.	Peak Name	Retention Time min	Area mAU*min	Height mAU	Relative Area %	Relative Height %	Amount n.a.
1		30.658	46.586	40.555	50.44	67.26	n.a.
2		65.665	45.776	19.742	49.56	32.74	n.a.
**Total:**			**92.362**	**60.297**	**100.00**	**100.00**	

Chromatogram

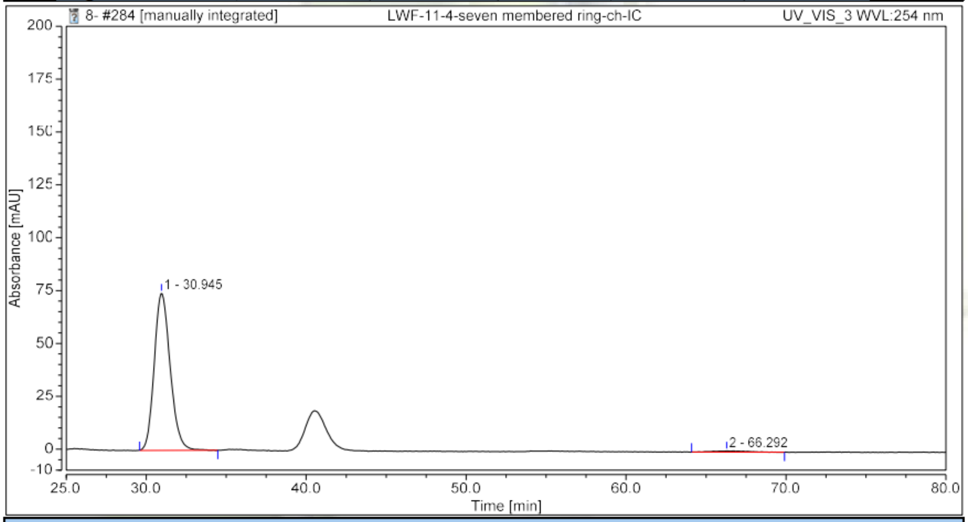

Integration Results

No.	Peak Name	Retention Time min	Area mAU*min	Height mAU	Relative Area %	Relative Height %	Amount n.a.
1		30.945	85.812	74.481	98.28	99.09	n.a.
2		66.292	1.504	0.682	1.72	0.91	n.a.
**Total:**			**87.316**	**75.163**	**100.00**	**100.00**	

Compound D16: IC, *i*-PrOH/hexane = 50/50, v = 1.0 mL/min, λ = 254 nm

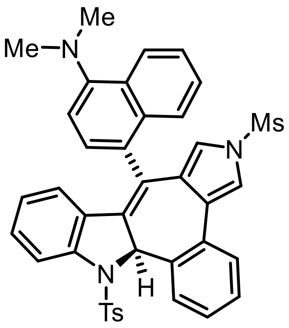

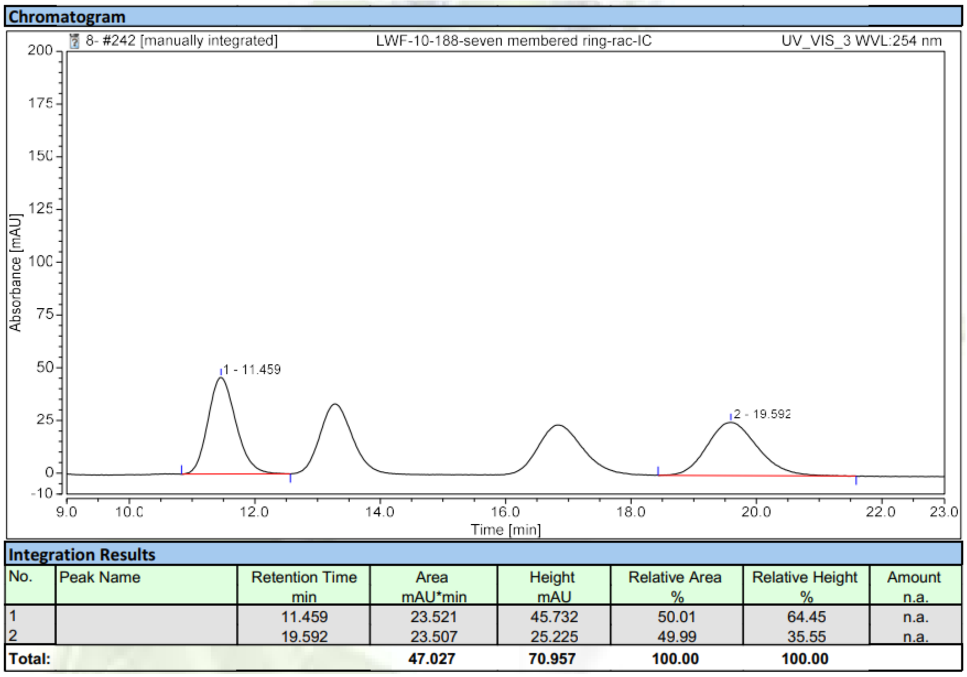

Integration Results

No.	Peak Name	Retention Time min	Area mAU*min	Height mAU	Relative Area %	Relative Height %	Amount n.a.
1		11.459	23.521	45.732	50.01	64.45	n.a.
2		19.592	23.507	25.225	49.99	35.55	n.a.
**Total:**			**47.027**	**70.957**	**100.00**	**100.00**	

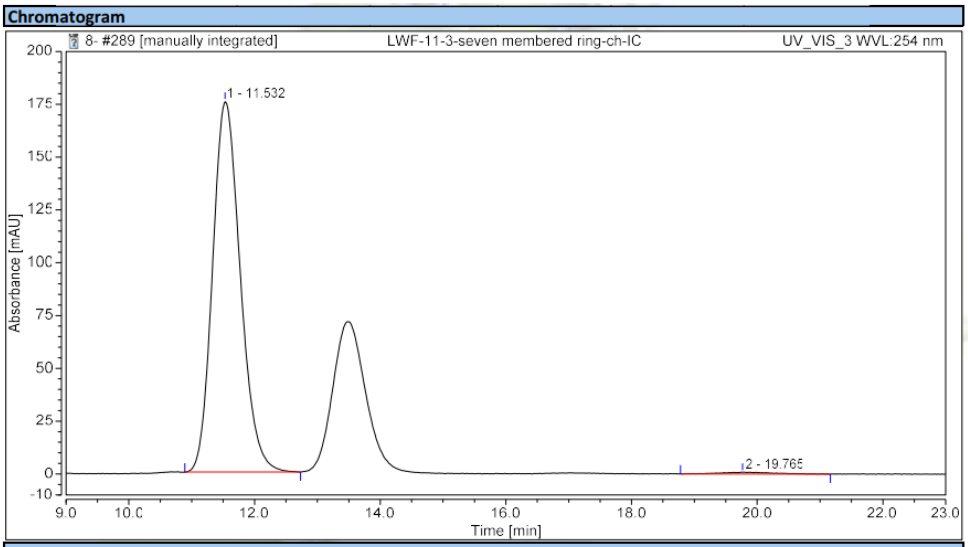

Integration Results

No.	Peak Name	Retention Time min	Area mAU*min	Height mAU	Relative Area %	Relative Height %	Amount n.a.
1		11.532	89.574	175.509	99.03	99.49	n.a.
2		19.765	0.875	0.900	0.97	0.51	n.a.
**Total:**			**90.450**	**176.408**	**100.00**	**100.00**	

Compound E1: IC, *i*-PrOH/hexane = 50/50, v = 1.0 mL/min, λ = 254 nm

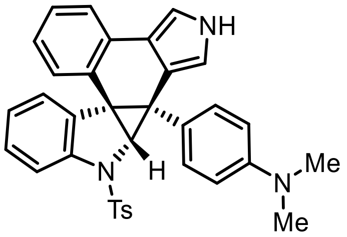

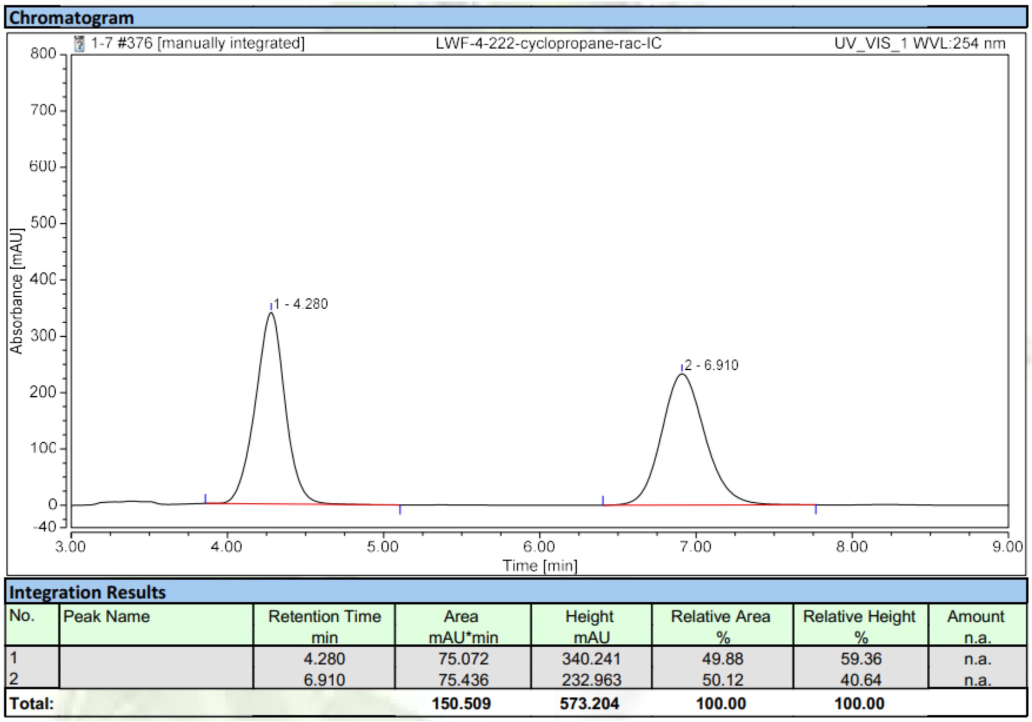

Integration Results

No.	Peak Name	Retention Time min	Area mAU*min	Height mAU	Relative Area %	Relative Height %	Amount n.a.
1		4.280	75.072	340.241	49.88	59.36	n.a.
2		6.910	75.436	232.963	50.12	40.64	n.a.
Total:			150.509	573.204	100.00	100.00	

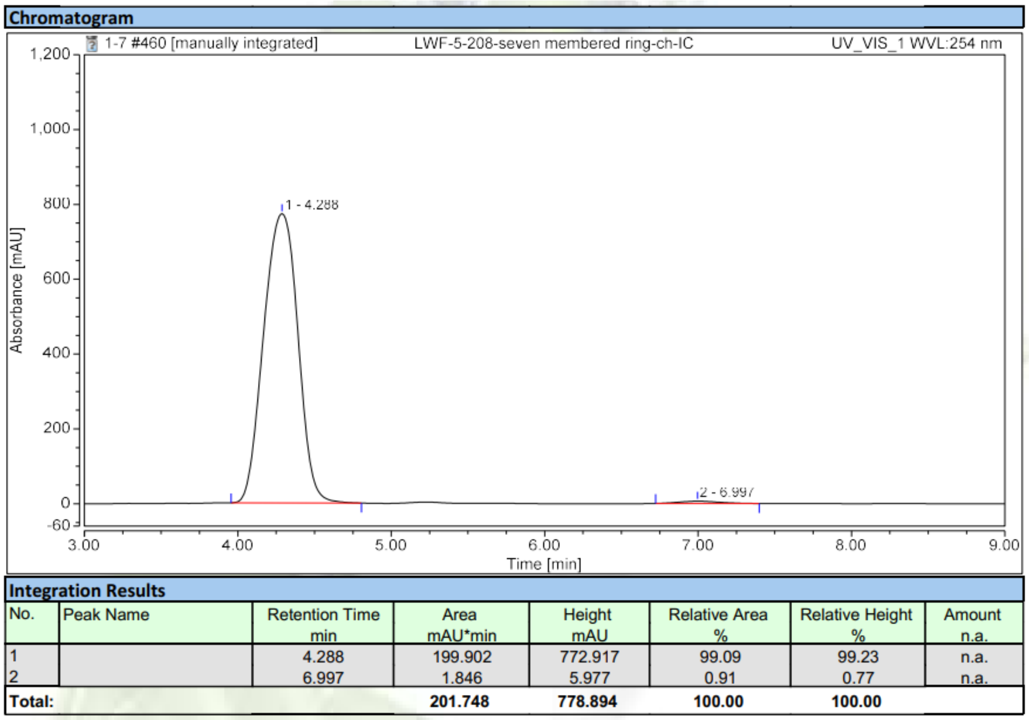

Integration Results

No.	Peak Name	Retention Time min	Area mAU*min	Height mAU	Relative Area %	Relative Height %	Amount n.a.
1		4.288	199.902	772.917	99.09	99.23	n.a.
2		6.997	1.846	5.977	0.91	0.77	n.a.
Total:			201.748	778.894	100.00	100.00	

Compound E2: IB, *i*-PrOH/hexane = 30/70, v = 1.0 mL/min, λ = 254 nm

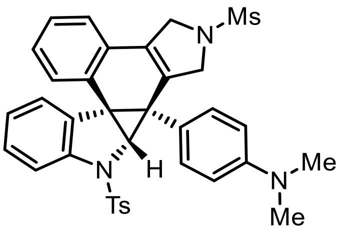

Chromatogram

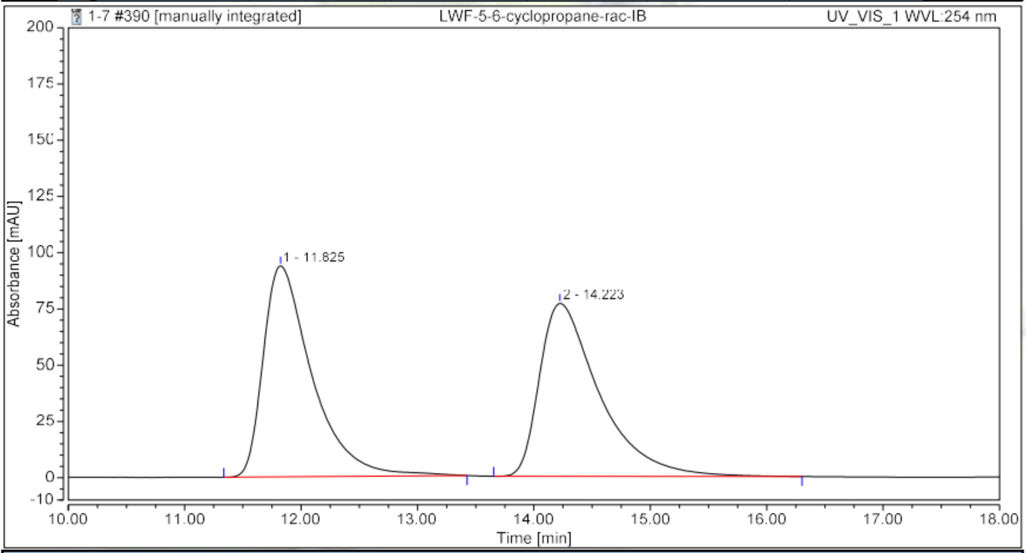

Integration Results

No.	Peak Name	Retention Time min	Area mAU*min	Height mAU	Relative Area %	Relative Height %	Amount n.a.
1		11.825	45.568	93.802	49.87	54.96	n.a.
2		14.223	45.809	76.865	50.13	45.04	n.a.
**Total:**			**91.377**	**170.667**	**100.00**	**100.00**	

Chromatogram

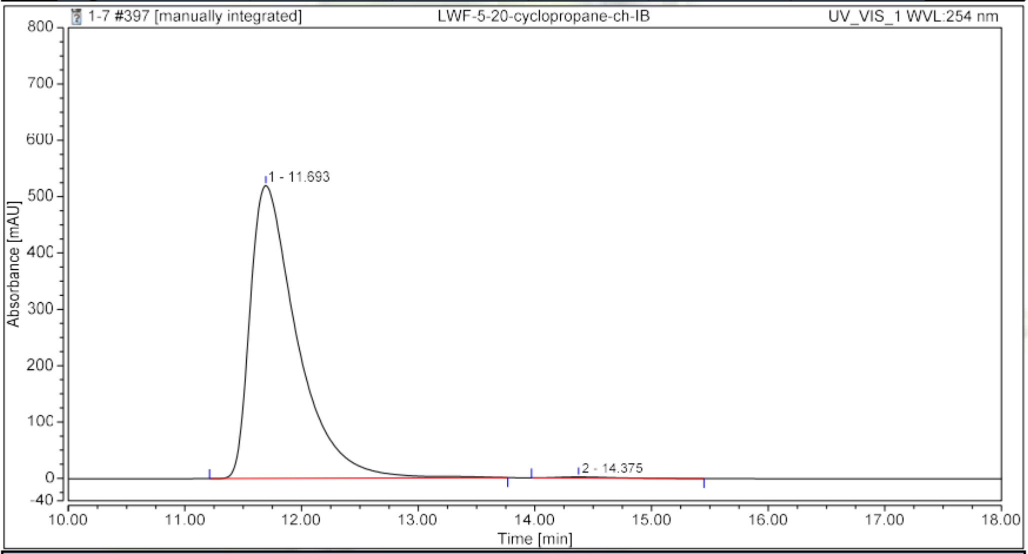

Integration Results

No.	Peak Name	Retention Time min	Area mAU*min	Height mAU	Relative Area %	Relative Height %	Amount n.a.
1		11.693	242.249	519.887	99.59	99.63	n.a.
2		14.375	0.987	1.951	0.41	0.37	n.a.
**Total:**			**243.236**	**521.837**	**100.00**	**100.00**	

Compound E3: IA, *i*-PrOH/hexane = 50/50, v = 1.0 mL/min, λ = 254 nm

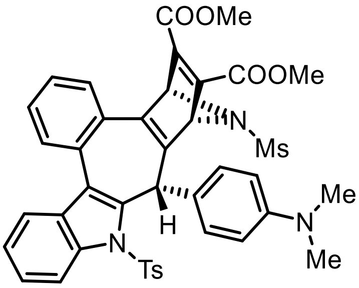

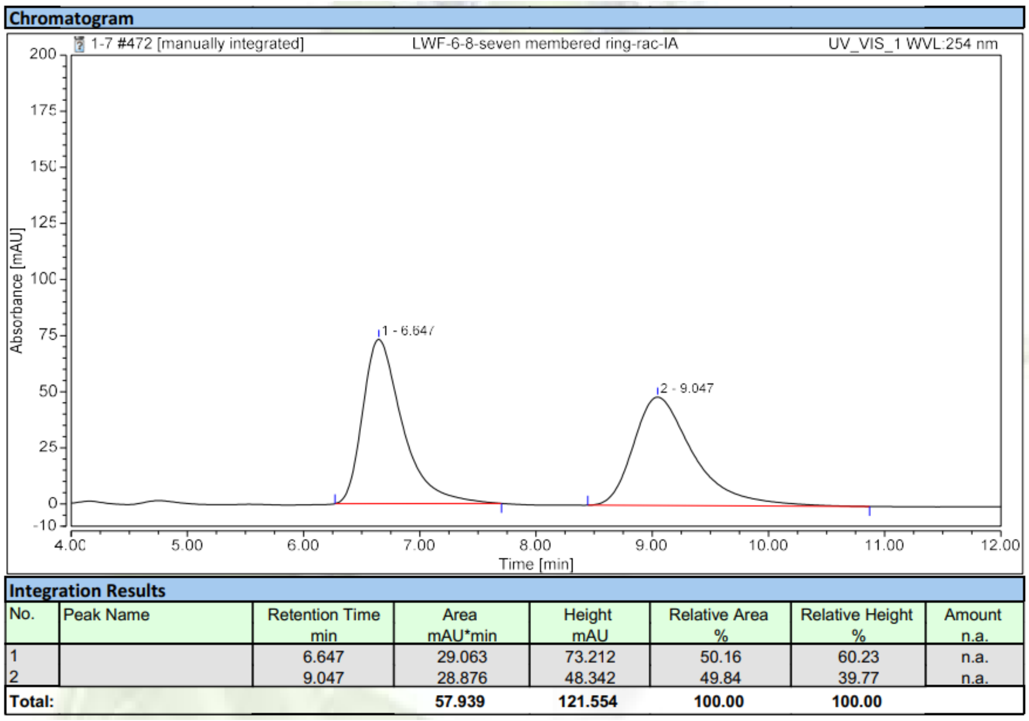

Integration Results

No.	Peak Name	Retention Time<br>min	Area<br>mAU*min	Height<br>mAU	Relative Area<br>%	Relative Height<br>%	Amount<br>n.a.
1		6.647	29.063	73.212	50.16	60.23	n.a.
2		9.047	28.876	48.342	49.84	39.77	n.a.
**Total:**			**57.939**	**121.554**	**100.00**	**100.00**	

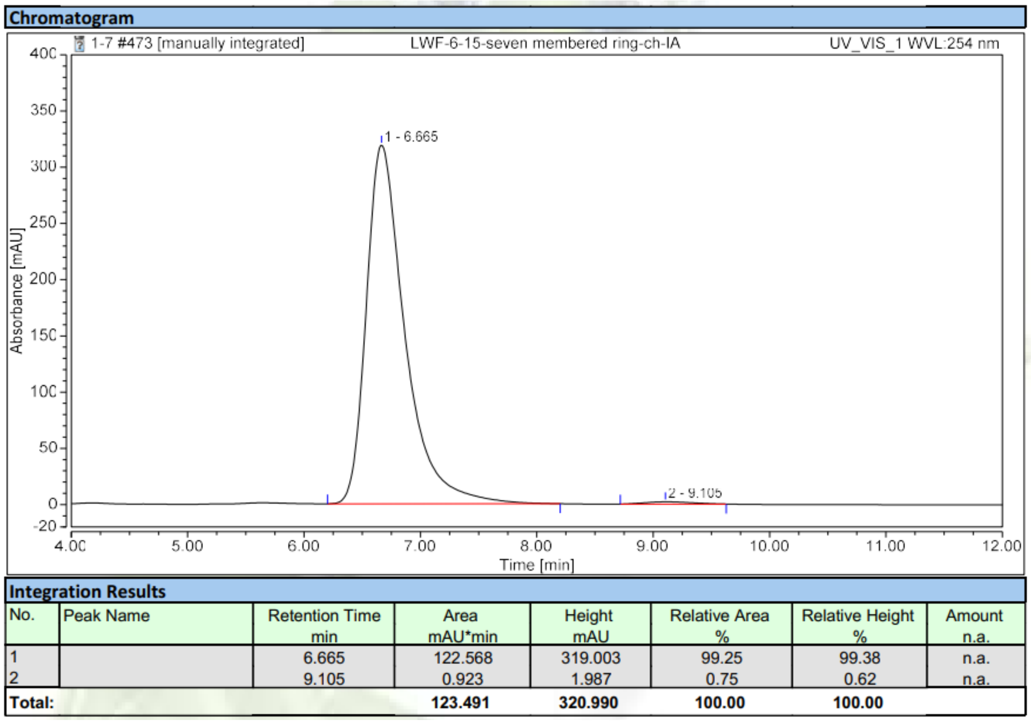

Integration Results

No.	Peak Name	Retention Time min	Area mAU*min	Height mAU	Relative Area %	Relative Height %	Amount n.a.
1		6.665	122.568	319.003	99.25	99.38	n.a.
2		9.105	0.923	1.987	0.75	0.62	n.a.
**Total:**			**123.491**	**320.990**	**100.00**	**100.00**	

Compound E4: IA, *i*-PrOH/hexane = 50/50, v = 1.0 mL/min, λ = 254 nm

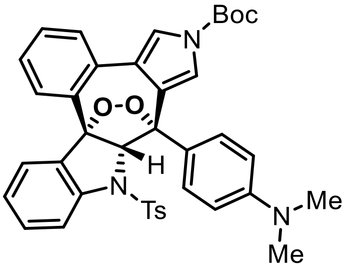

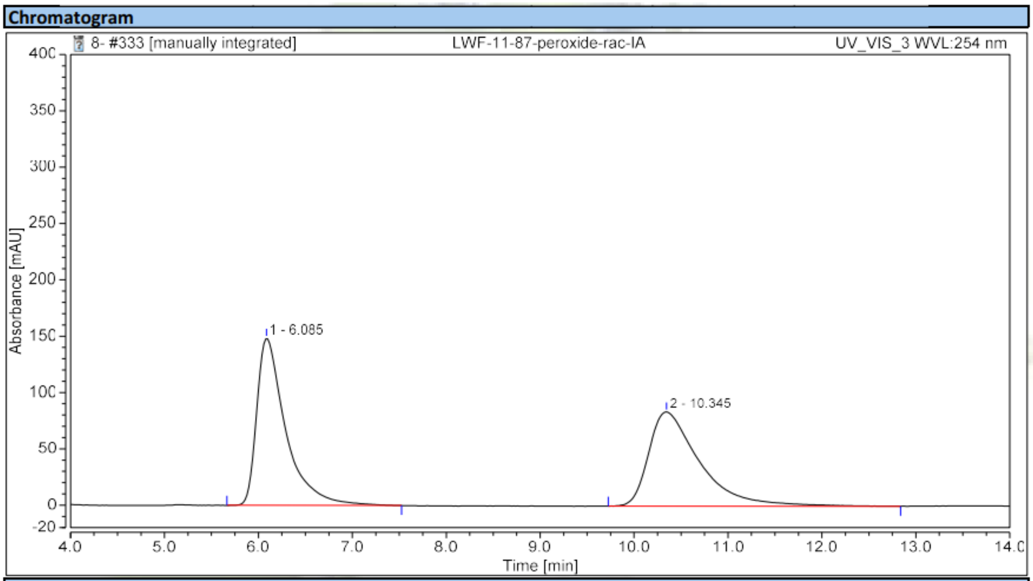

Integration Results

No.	Peak Name	Retention Time min	Area mAU*min	Height mAU	Relative Area %	Relative Height %	Amount n.a.
1		6.085	52.908	148.462	50.15	63.95	n.a.
2		10.345	52.584	83.683	49.85	36.05	n.a.
**Total:**			**105.492**	**232.145**	**100.00**	**100.00**	

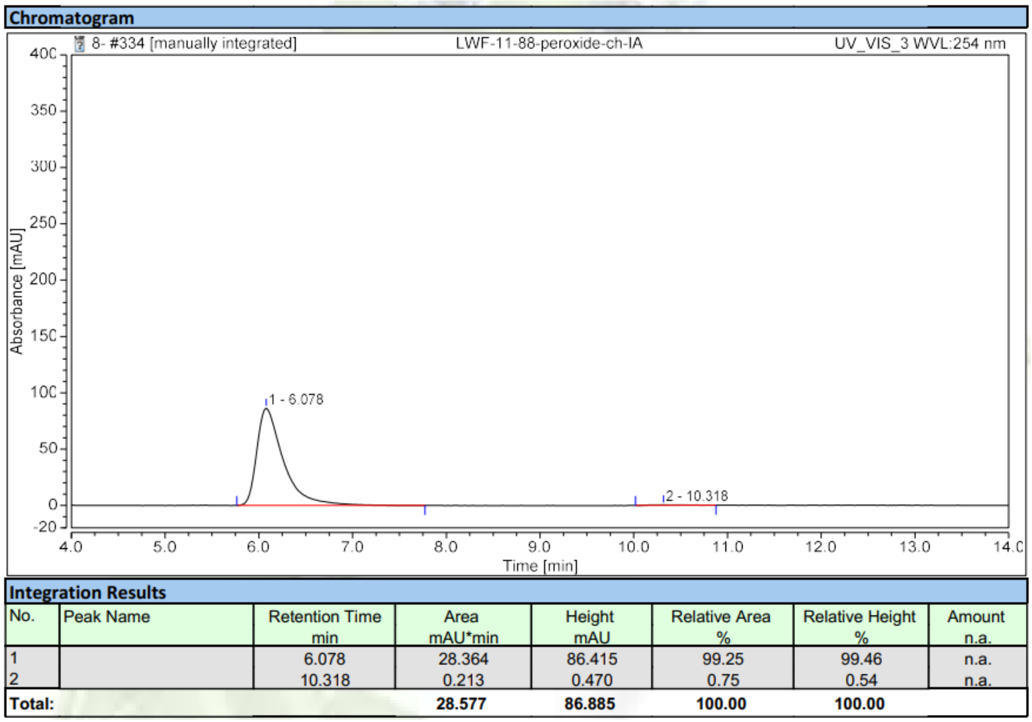

Integration Results

No.	Peak Name	Retention Time min	Area mAU*min	Height mAU	Relative Area %	Relative Height %	Amount n.a.
1		6.078	28.364	86.415	99.25	99.46	n.a.
2		10.318	0.213	0.470	0.75	0.54	n.a.
Total:			28.577	86.885	100.00	100.00	

Compound E5: IC, *i*-PrOH/hexane = 50/50, v = 1.0 mL/min, λ = 254 nm

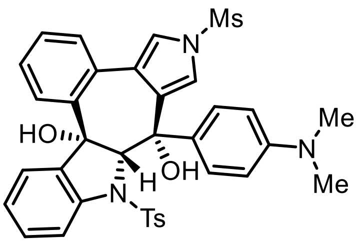

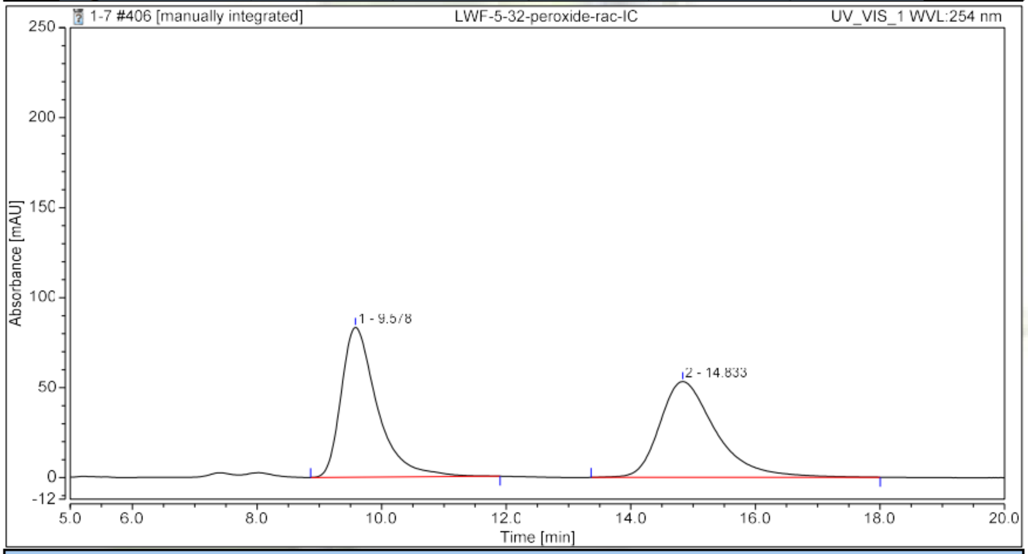

Integration Results

No.	Peak Name	Retention Time min	Area mAU*min	Height mAU	Relative Area %	Relative Height %	Amount n.a.
1		9.578	55.937	83.227	50.24	60.99	n.a.
2		14.833	55.411	53.222	49.76	39.01	n.a.
Total:			111.347	136.449	100.00	100.00	

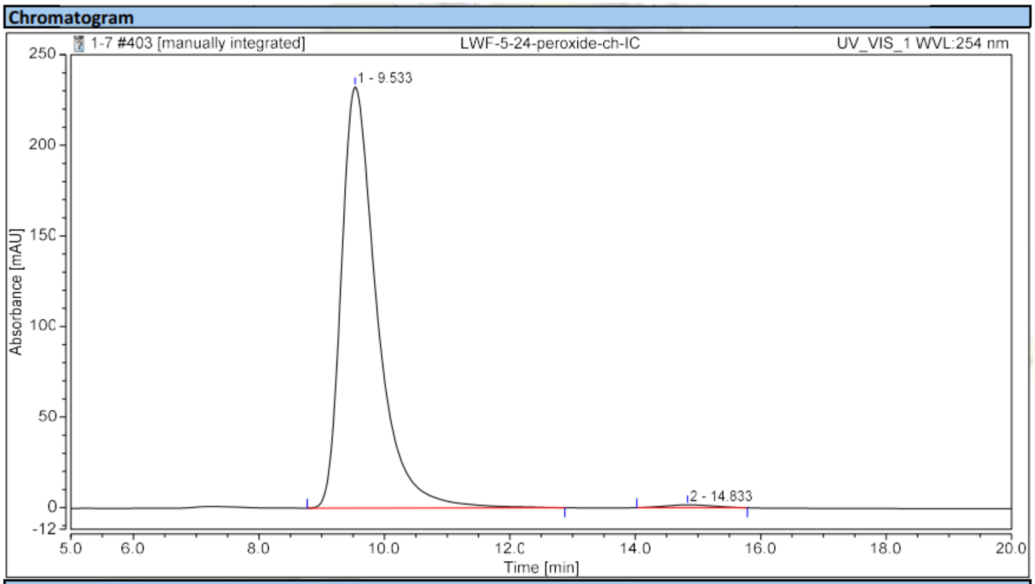

Integration Results

No.	Peak Name	Retention Time min	Area mAU*min	Height mAU	Relative Area %	Relative Height %	Amount n.a.
1		9.533	153.064	232.481	99.08	99.32	n.a.
2		14.833	1.421	1.600	0.92	0.68	n.a.
**Total:**			**154.485**	**234.081**	**100.00**	**100.00**	

Compound E6: INA, *i*-PrOH/hexane = 20/80, v = 1.0 mL/min, λ = 254 nm

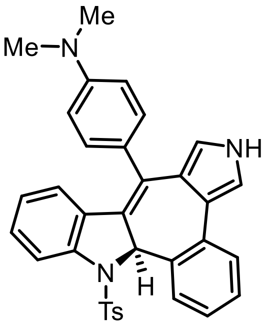

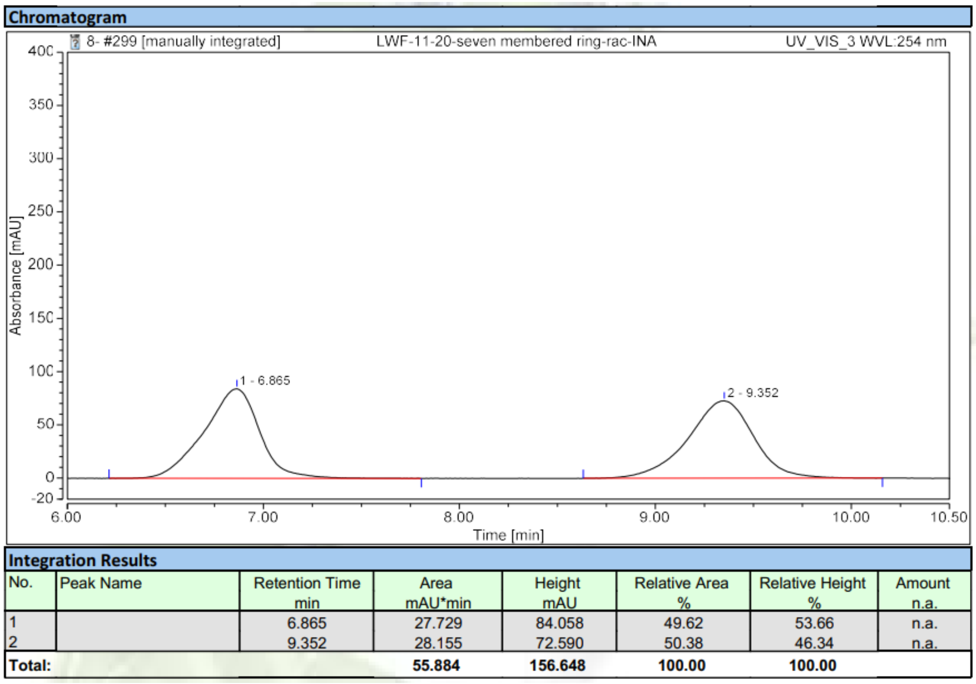

Integration Results

No.	Peak Name	Retention Time min	Area mAU*min	Height mAU	Relative Area %	Relative Height %	Amount n.a.
1		6.865	27.729	84.058	49.62	53.66	n.a.
2		9.352	28.155	72.590	50.38	46.34	n.a.
Total:			55.884	156.648	100.00	100.00	

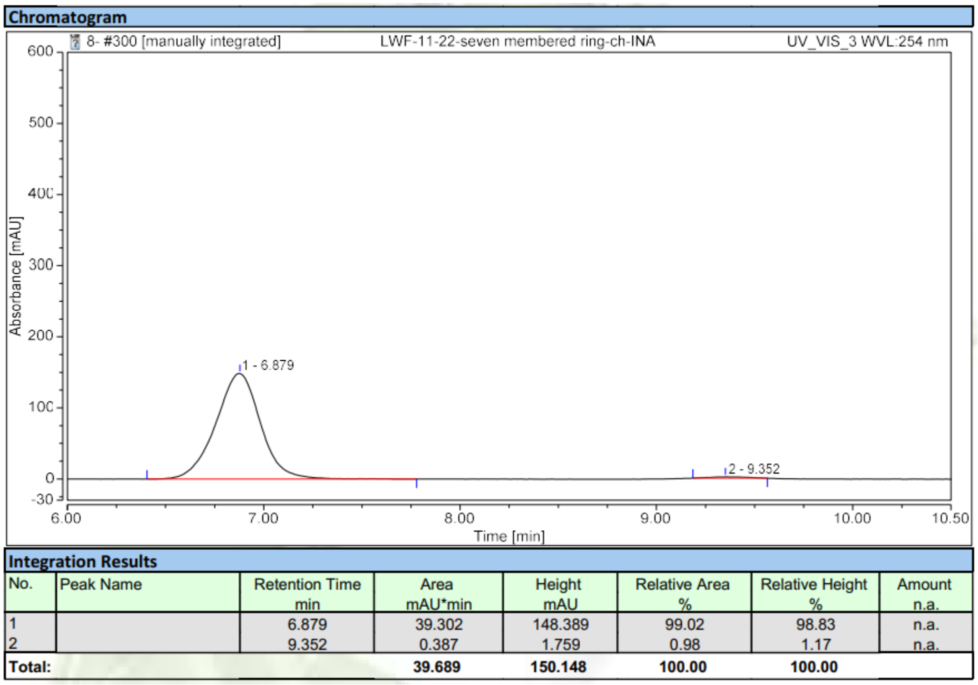

Integration Results

No.	Peak Name	Retention Time min	Area mAU*min	Height mAU	Relative Area %	Relative Height %	Amount n.a.
1		6.879	39.302	148.389	99.02	98.83	n.a.
2		9.352	0.387	1.759	0.98	1.17	n.a.
Total:			39.689	150.148	100.00	100.00	

Compound E7: IB, *i*-PrOH/hexane = 50/50, v = 1.0 mL/min, λ = 220 nm

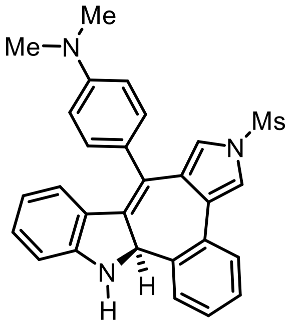

Chromatogram

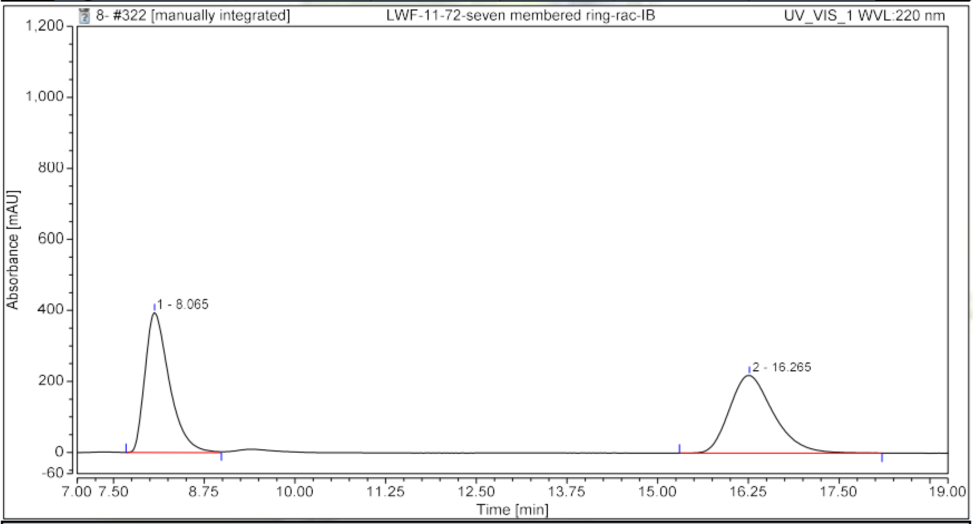

Integration Results

No.	Peak Name	Retention Time min	Area mAU*min	Height mAU	Relative Area %	Relative Height %	Amount n.a.
1		8.065	153.353	393.633	50.01	64.30	n.a.
2		16.265	153.308	218.579	49.99	35.70	n.a.
**Total:**			**306.661**	**612.212**	**100.00**	**100.00**	

Chromatogram

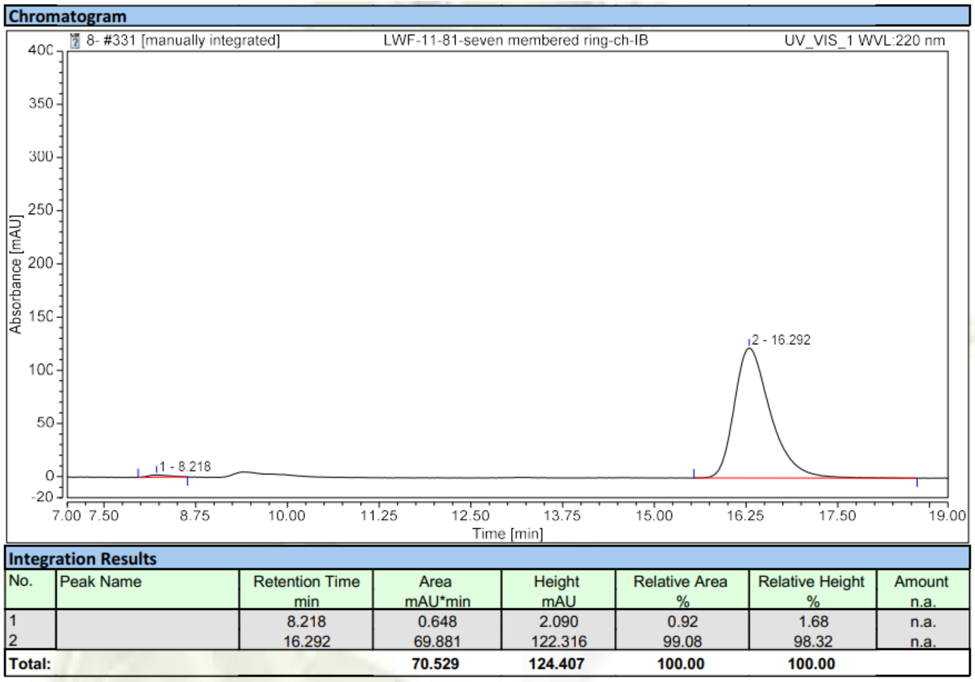

Integration Results

No.	Peak Name	Retention Time min	Area mAU*min	Height mAU	Relative Area %	Relative Height %	Amount n.a.
1		8.218	0.648	2.090	0.92	1.68	n.a.
2		16.292	69.881	122.316	99.08	98.32	n.a.
**Total:**			**70.529**	**124.407**	**100.00**	**100.00**	

Compound E8: IA, *i*-PrOH/hexane = 30/70, v = 1.0 mL/min, λ = 254 nm

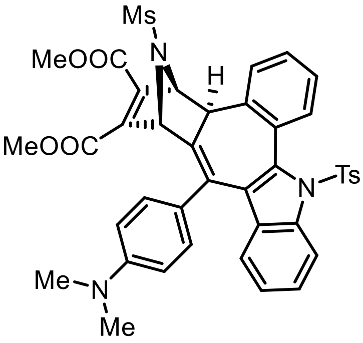

Chromatogram

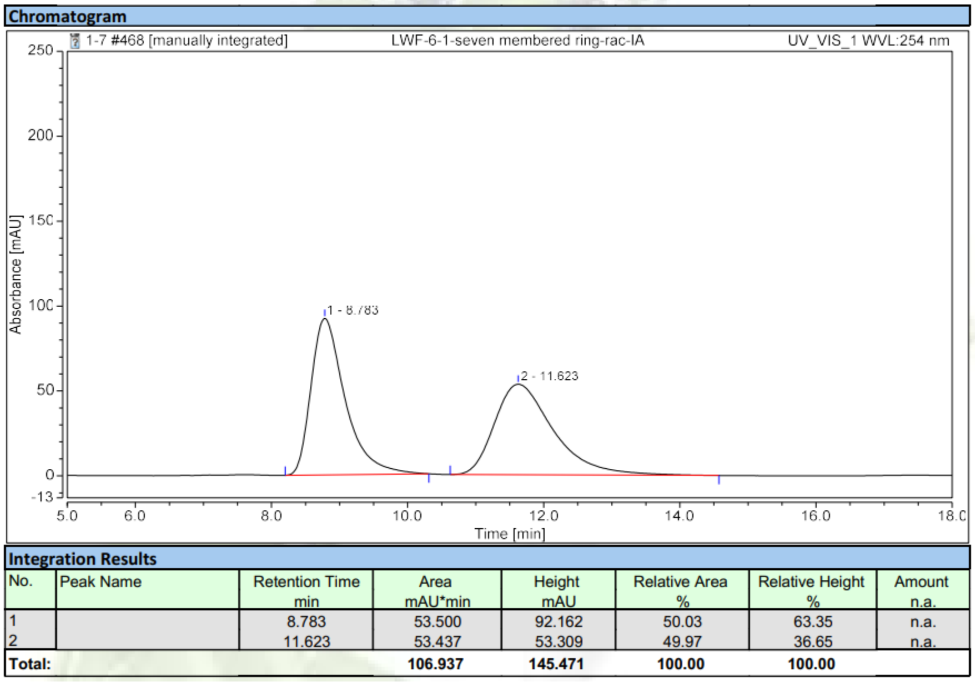

Integration Results

No.	Peak Name	Retention Time min	Area mAU*min	Height mAU	Relative Area %	Relative Height %	Amount n.a.
1		8.783	53.500	92.162	50.03	63.35	n.a.
2		11.623	53.437	53.309	49.97	36.65	n.a.
**Total:**			**106.937**	**145.471**	**100.00**	**100.00**	

Chromatogram

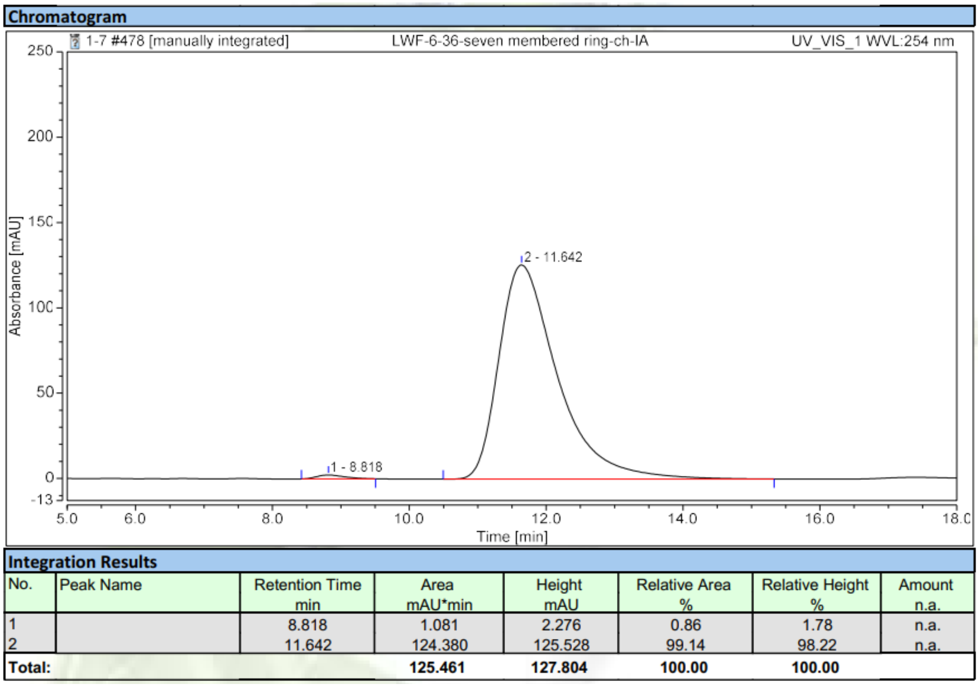

Integration Results

No.	Peak Name	Retention Time min	Area mAU*min	Height mAU	Relative Area %	Relative Height %	Amount n.a.
1		8.818	1.081	2.276	0.86	1.78	n.a.
2		11.642	124.380	125.528	99.14	98.22	n.a.
**Total:**			**125.461**	**127.804**	**100.00**	**100.00**	

9. NMR Spectra

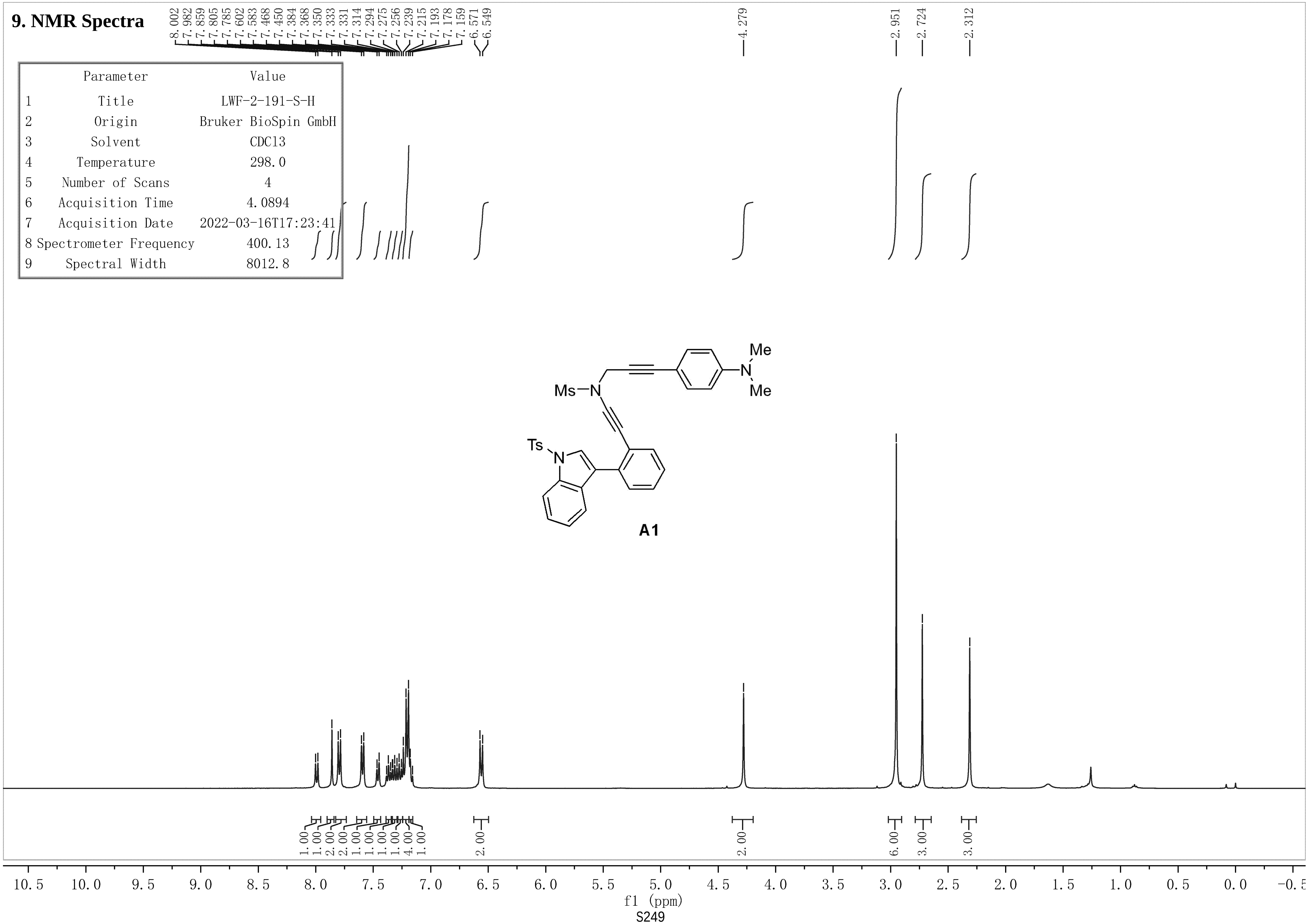

	Parameter	Value
1	Title	LWF-2-191-S-H
2	Origin	Bruker BioSpin GmbH
3	Solvent	CDCl3
4	Temperature	298.0
5	Number of Scans	4
6	Acquisition Time	4.0894
7	Acquisition Date	2022-03-16T17:23:41
8	Spectrometer Frequency	400.13
9	Spectral Width	8012.8

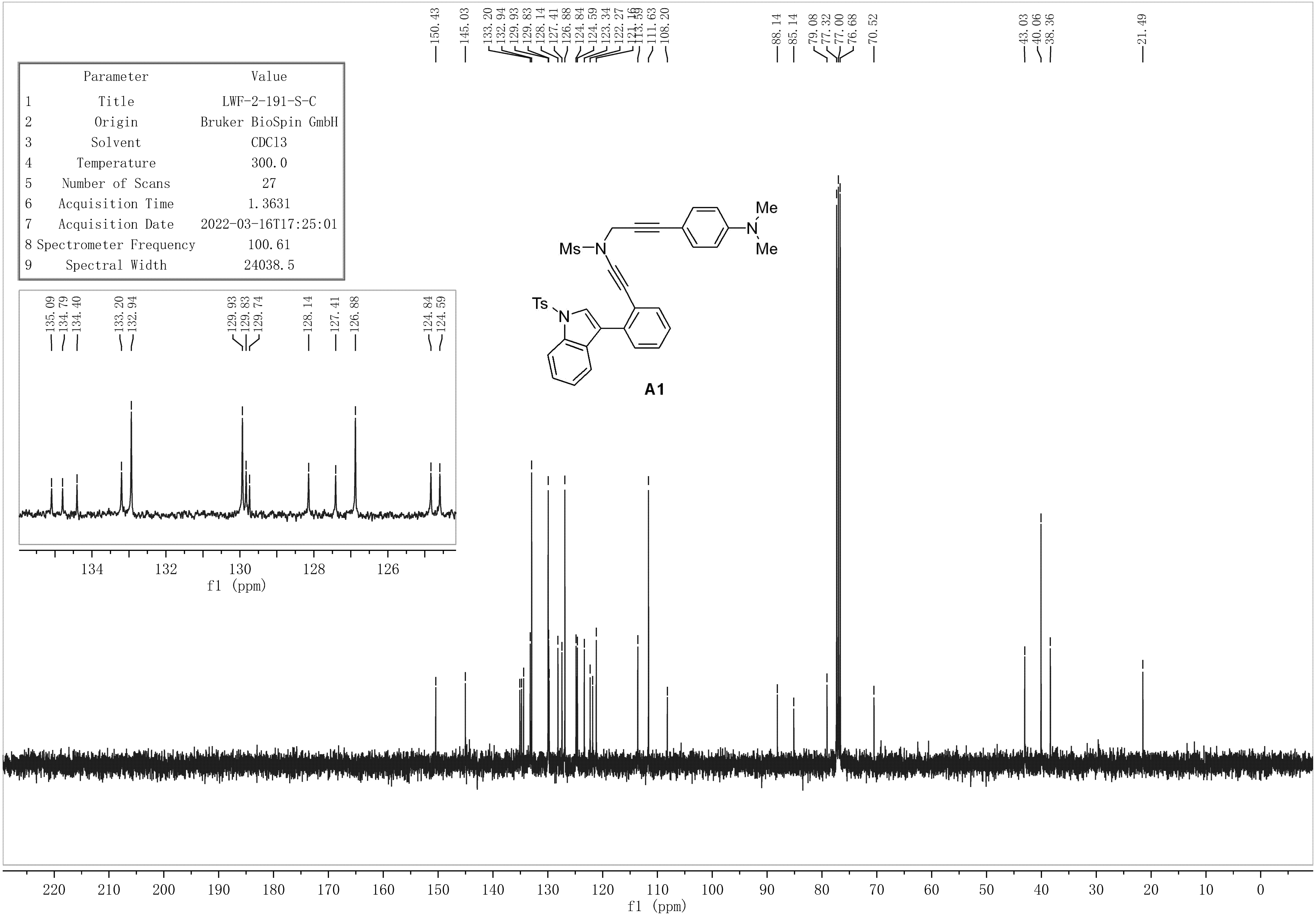
150.43
145.03
133.20
132.94
129.93
129.83
128.14
127.41
126.88
124.84
124.59
123.34
122.27
121.16
113.59
111.63
108.20
88.14
85.14
79.08
77.32
77.00
76.68
70.52
43.03
40.06
38.36
21.49
Parameter Value
1 Title LWF-2-191-S-C
2 Origin Bruker BioSpin GmbH
3 Solvent CDCl3
4 Temperature 300.0
5 Number of Scans 27
6 Acquisition Time 1.3631
7 Acquisition Date 2022-03-16T17:25:01
8 Spectrometer Frequency 100.61
9 Spectral Width 24038.5
135.09
134.79
134.40
133.20
132.94
129.93
129.83
129.74
128.14
127.41
126.88
124.84
124.59
134
132
130
128
126
f1 (ppm)
Ms
N
Me
Me
Ts
A1
220
210
200
190
180
170
160
150
140
130
120
110
100
90
80
70
60
50
40
30
20
10
0
f1 (ppm)

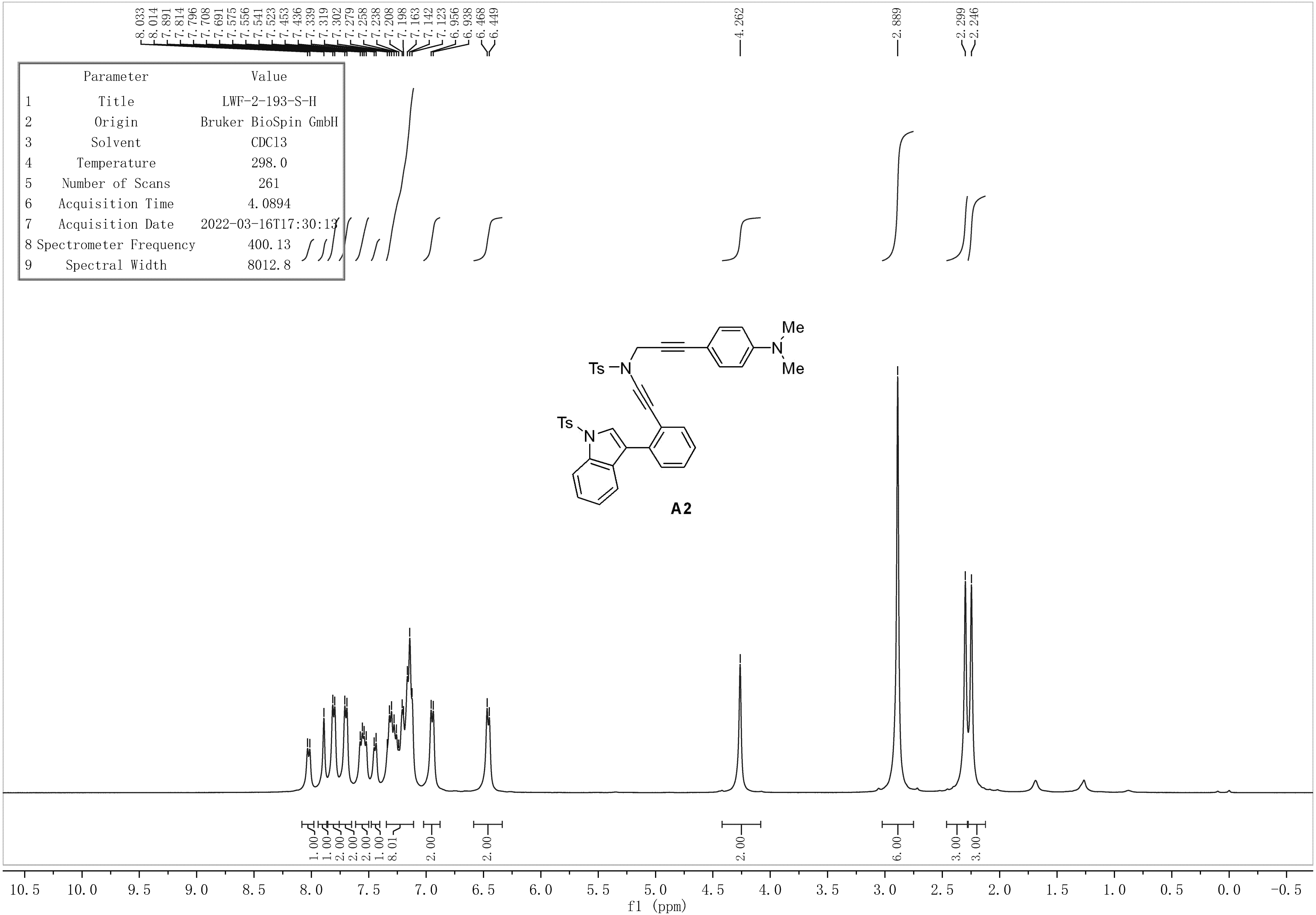
8.033
8.014
7.891
7.814
7.796
7.708
7.691
7.575
7.556
7.541
7.523
7.453
7.436
7.339
7.319
7.302
7.279
7.258
7.238
7.208
7.198
7.163
7.142
7.123
6.956
6.938
6.468
6.449
4.262
2.889
2.299
2.246
Parameter Value
1 Title LWF-2-193-S-H
2 Origin Bruker BioSpin GmbH
3 Solvent CDCl3
4 Temperature 298.0
5 Number of Scans 261
6 Acquisition Time 4.0894
7 Acquisition Date 2022-03-16T17:30:13
8 Spectrometer Frequency 400.13
9 Spectral Width 8012.8
Me
N
Me
Ts
N
Ts
N
A2
1.00
1.00
2.00
2.00
2.00
1.00
8.01
2.00
2.00
2.00
6.00
3.00
3.00
10.5 10.0 9.5 9.0 8.5 8.0 7.5 7.0 6.5 6.0 5.5 5.0 4.5 4.0 3.5 3.0 2.5 2.0 1.5 1.0 0.5 0.0 -0.5
f1 (ppm)

	Parameter	Value
1	Title	LWF-2-193-S
2	Origin	
3	Solvent	CDCl3
4	Temperature	297.3
5	Number of Scans	500
6	Acquisition Time	1.0000
7	Acquisition Date	2022-03-17T01:08:05
8	Spectrometer Frequency	100.56
9	Spectral Width	26041.0

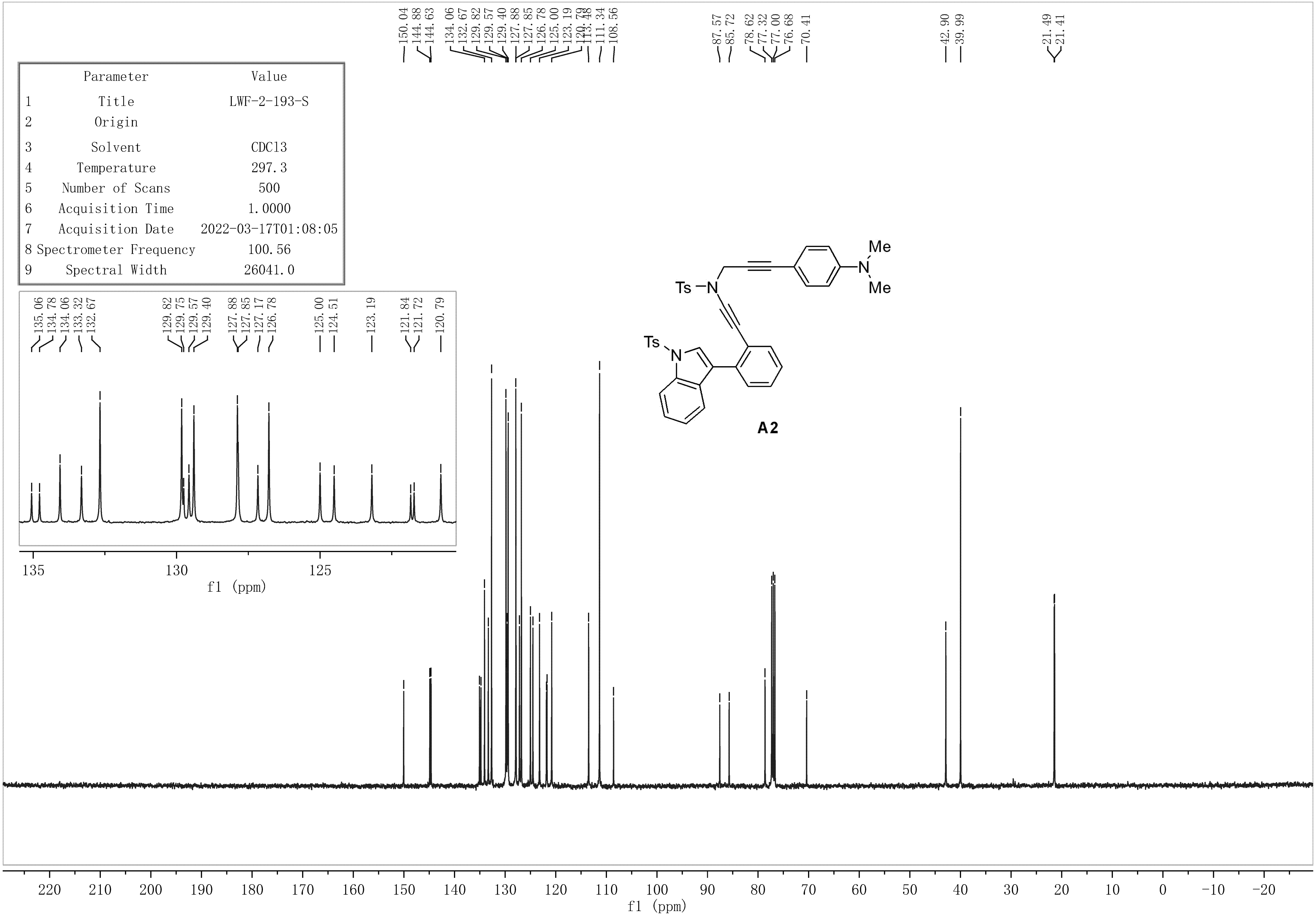

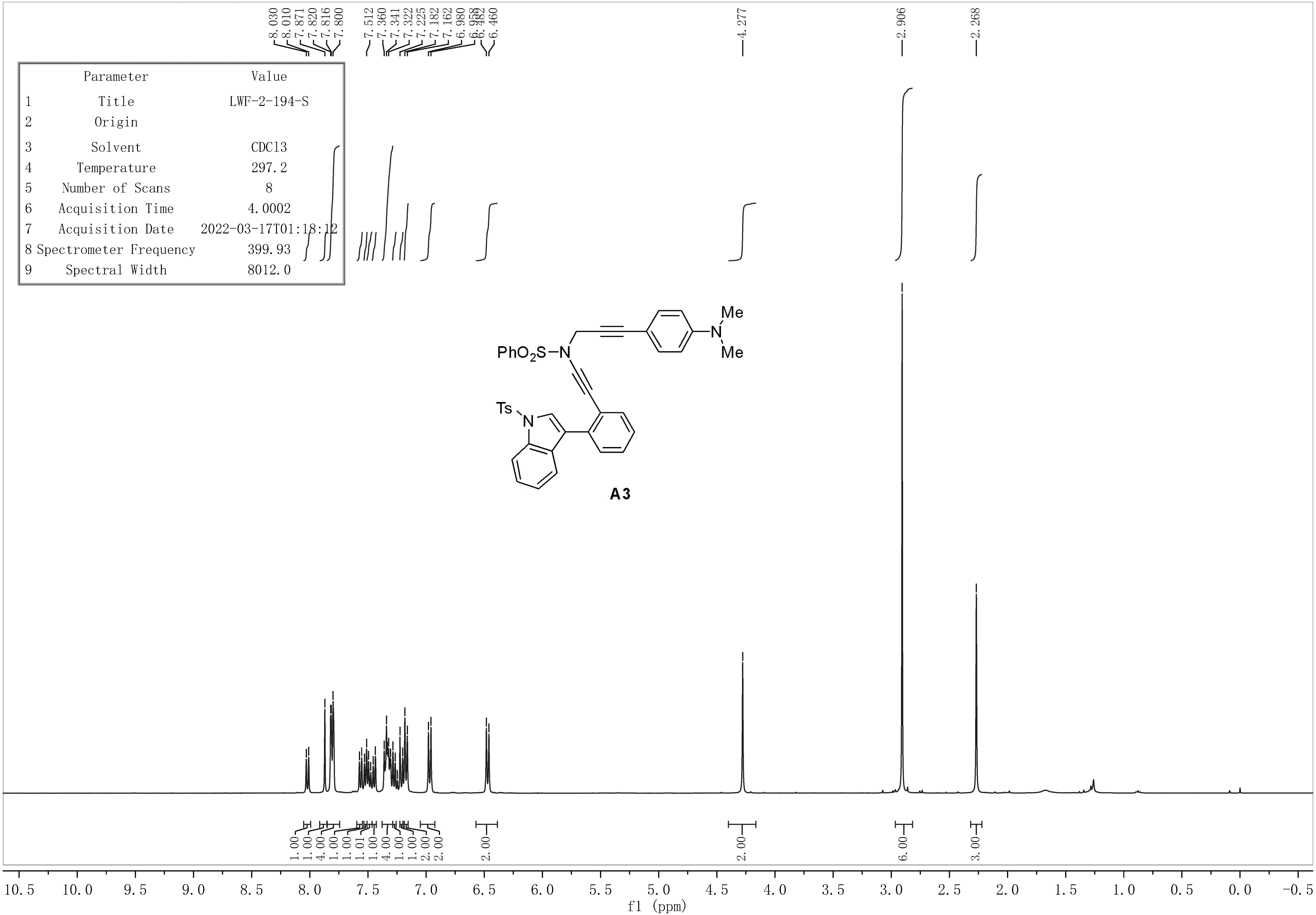

8.030
8.010
7.871
7.820
7.816
7.800
7.512
7.360
7.341
7.322
7.225
7.182
7.162
6.980
6.958
6.482
6.460
4.277
2.906
2.268
Parameter | Value
1 Title | LWF-2-194-S
2 Origin
3 Solvent | CDCl3
4 Temperature | 297.2
5 Number of Scans | 8
6 Acquisition Time | 4.0002
7 Acquisition Date | 2022-03-17T01:18:12
8 Spectrometer Frequency | 399.93
9 Spectral Width | 8012.0
PhO2S
N
Me
Me
Ts
A3
1.00
1.00
4.00
1.00
1.00
1.01
1.00
4.00
1.00
1.00
2.00
2.00
2.00
2.00
6.00
3.00
10.5 10.0 9.5 9.0 8.5 8.0 7.5 7.0 6.5 6.0 5.5 5.0 4.5 4.0 3.5 3.0 2.5 2.0 1.5 1.0 0.5 0.0 -0.5
f1 (ppm)

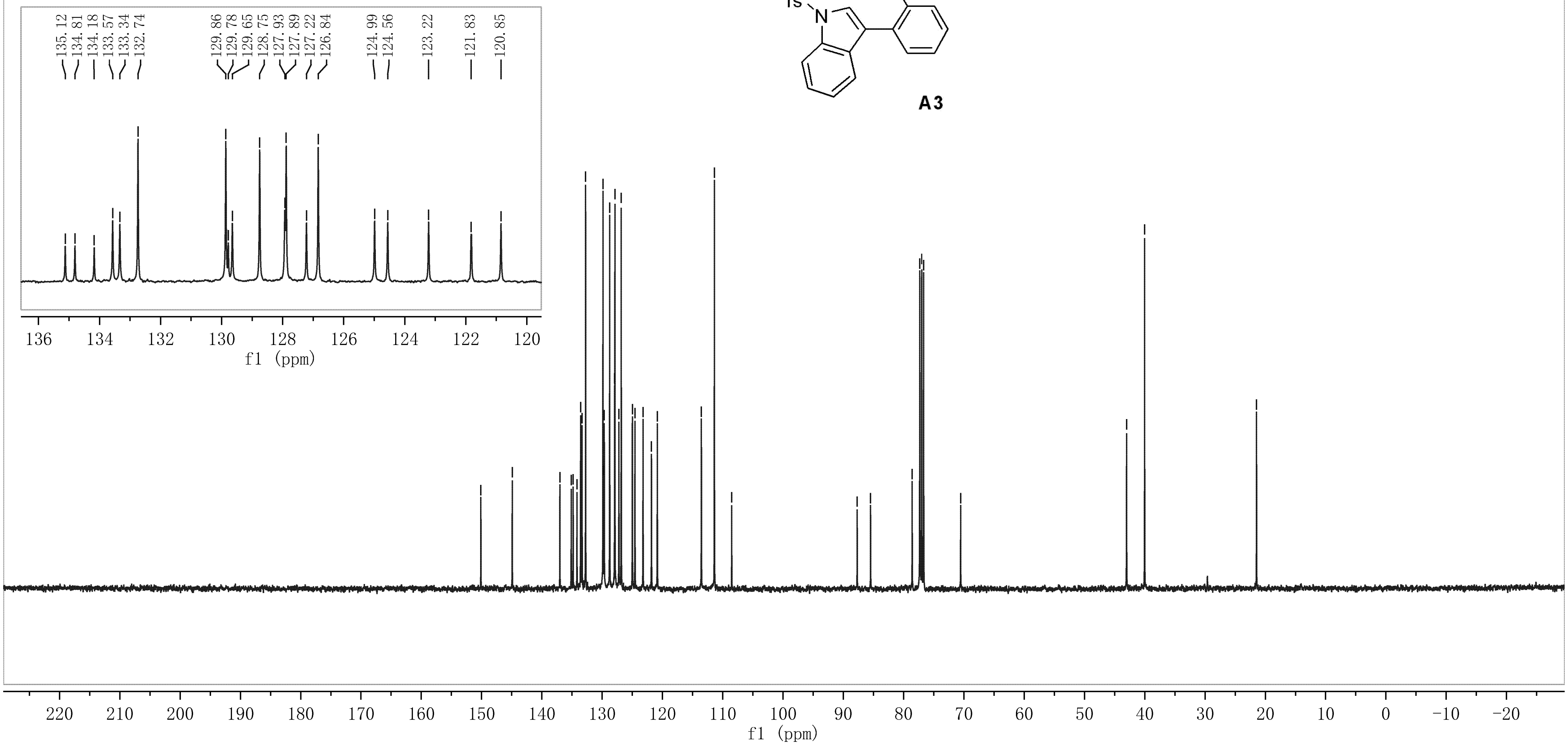

	Parameter	Value
1	Title	LWF-2-194-S
2	Origin	
3	Solvent	CDCl3
4	Temperature	297.2
5	Number of Scans	500
6	Acquisition Time	1.0000
7	Acquisition Date	2022-03-17T01:35:26
8	Spectrometer Frequency	100.56
9	Spectral Width	26041.0

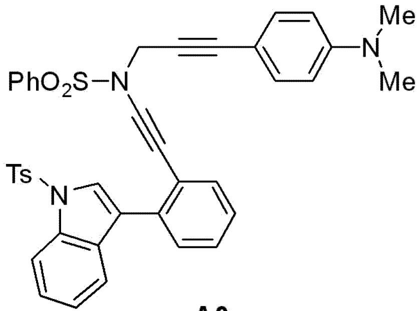

A3

	Parameter	Value
1	Title	LWF-2-192-S-H
2	Origin	Bruker BioSpin GmbH
3	Solvent	CDCl3
4	Temperature	298.0
5	Number of Scans	9
6	Acquisition Time	4.0894
7	Acquisition Date	2022-03-18T17:04:46
8	Spectrometer Frequency	400.13
9	Spectral Width	8012.8

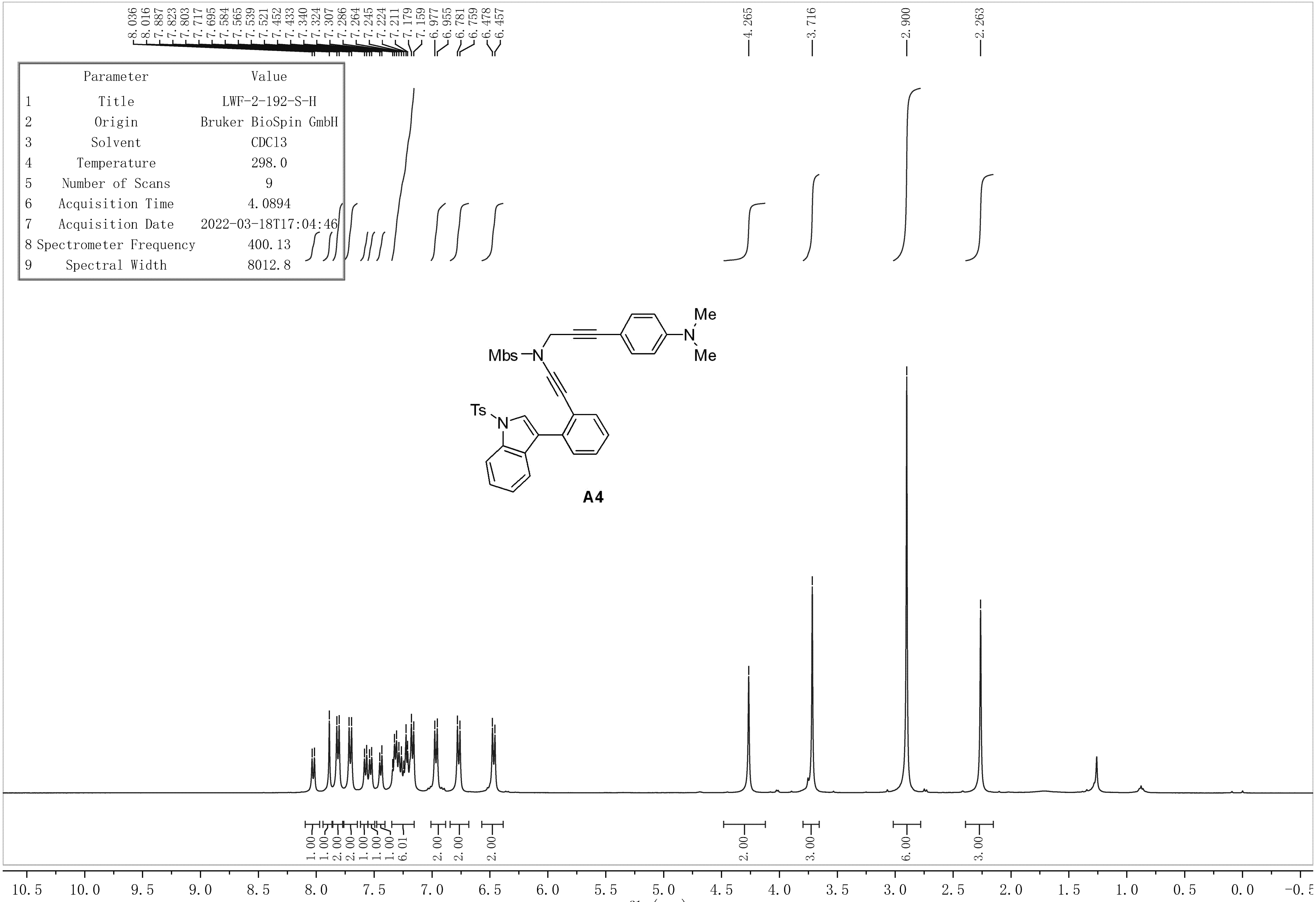

	Parameter	Value
1	Title	LWF-2-192-S-C
2	Origin	Bruker BioSpin GmbH
3	Solvent	CDCl3
4	Temperature	300.0
5	Number of Scans	60
6	Acquisition Time	1.3631
7	Acquisition Date	2022-03-18T17:06:54
8	Spectrometer Frequency	100.61
9	Spectral Width	24038.5

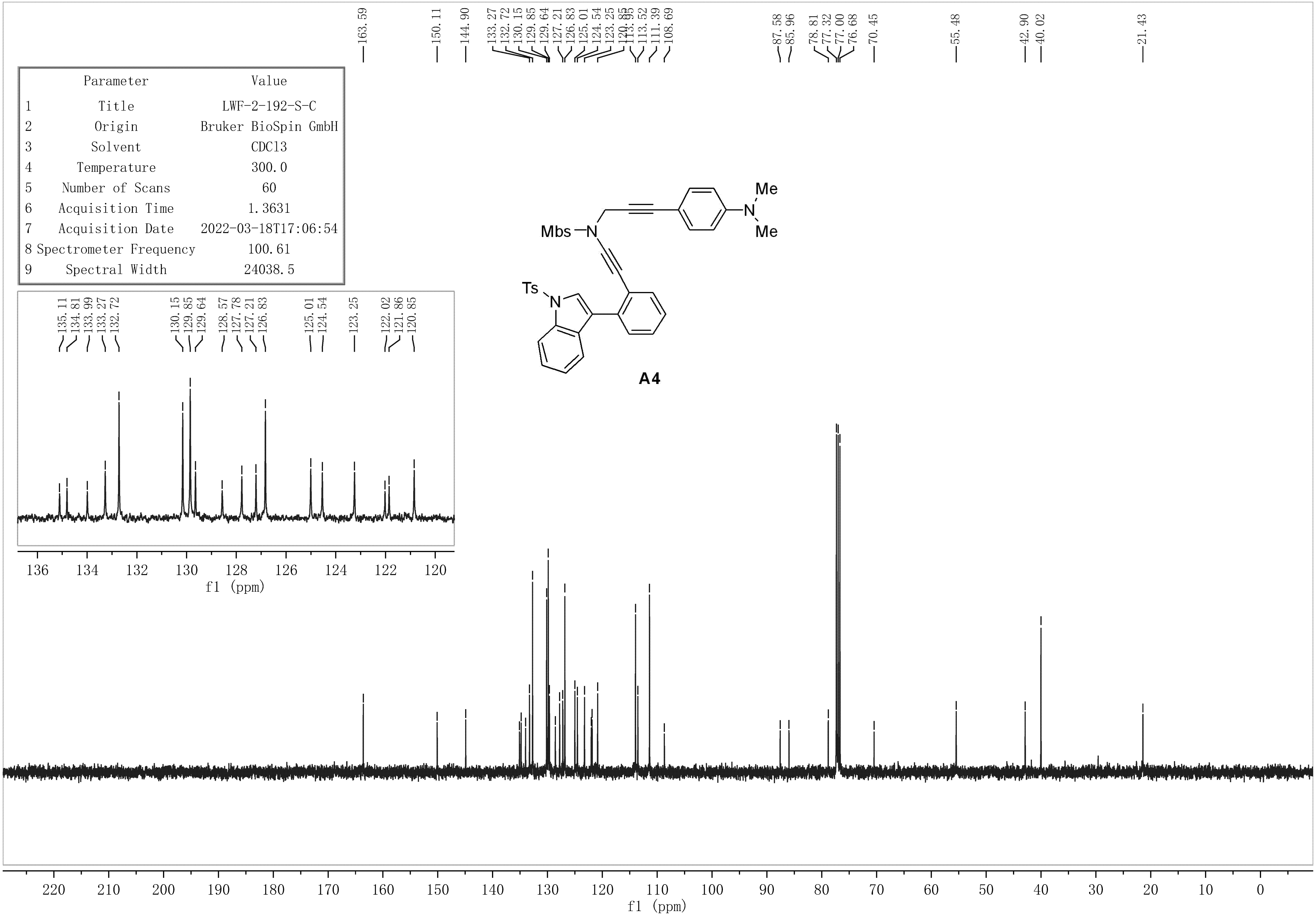

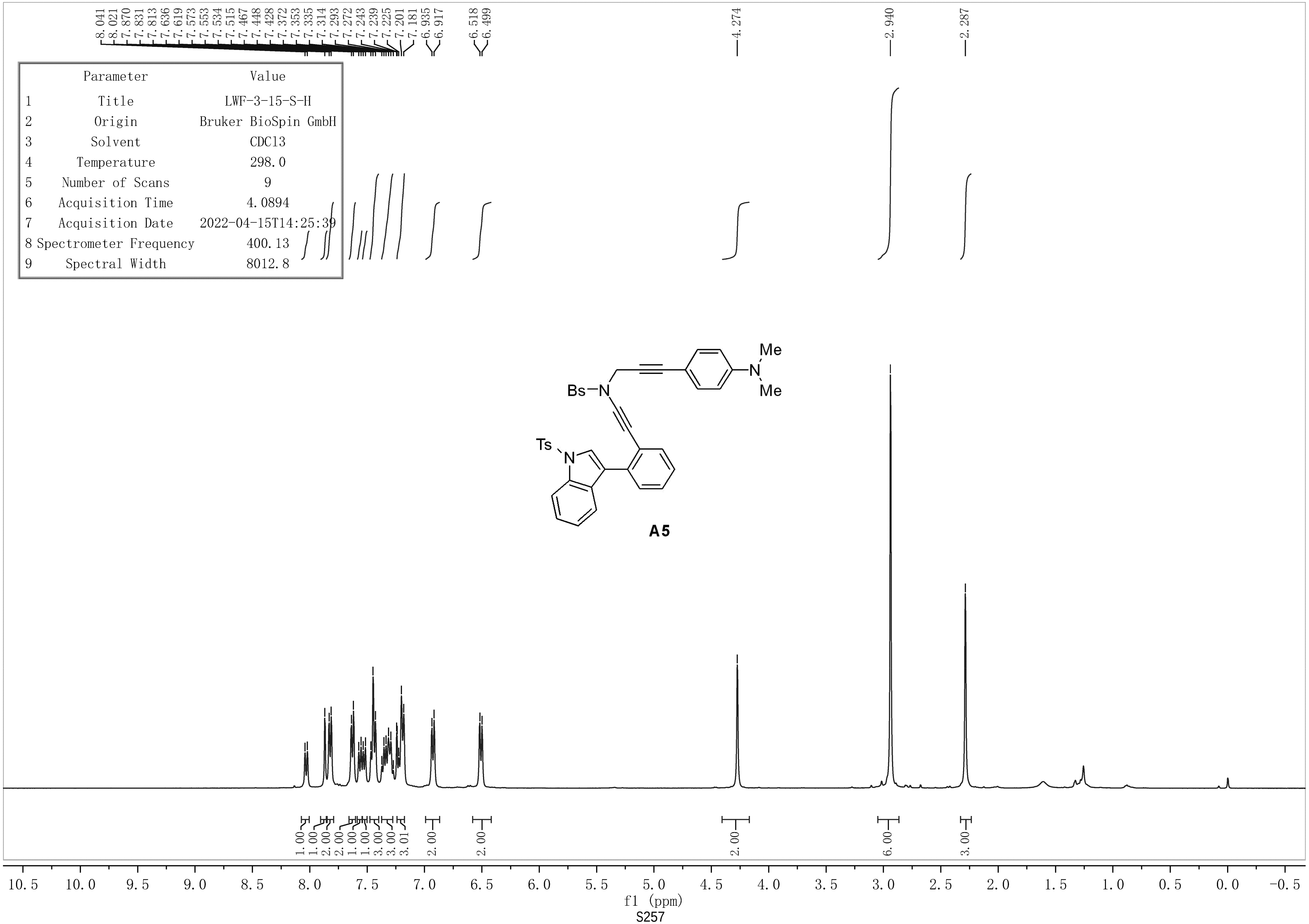
Parameter Value
1 Title LWF-3-15-S-H
2 Origin Bruker BioSpin GmbH
3 Solvent CDCl3
4 Temperature 298.0
5 Number of Scans 9
6 Acquisition Time 4.0894
7 Acquisition Date 2022-04-15T14:25:39
8 Spectrometer Frequency 400.13
9 Spectral Width 8012.8
8.041 8.021 7.870 7.831 7.813 7.636 7.619 7.573 7.553 7.534 7.515 7.467 7.448 7.428 7.372 7.353 7.335 7.314 7.293 7.272 7.243 7.239 7.225 7.201 7.181 6.935 6.917 6.518 6.499
4.274
2.940
2.287
Me
N
Me
Bs
N
Ts
N
A5
1.00 1.00 2.00 2.00 1.00 1.00 3.00 3.00 3.01 2.00 2.00 2.00 6.00 3.00
10.5 10.0 9.5 9.0 8.5 8.0 7.5 7.0 6.5 6.0 5.5 5.0 4.5 4.0 3.5 3.0 2.5 2.0 1.5 1.0 0.5 0.0 -0.5
f1 (ppm)

	Parameter	Value
1	Title	LWF-3-15-S-C
2	Origin	Bruker BioSpin GmbH
3	Solvent	CDCl3
4	Temperature	300.0
5	Number of Scans	56
6	Acquisition Time	1.3631
7	Acquisition Date	2022-04-11T14:32:01
8	Spectrometer Frequency	100.61
9	Spectral Width	24038.5

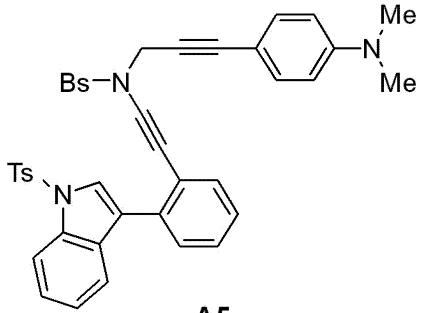

A5

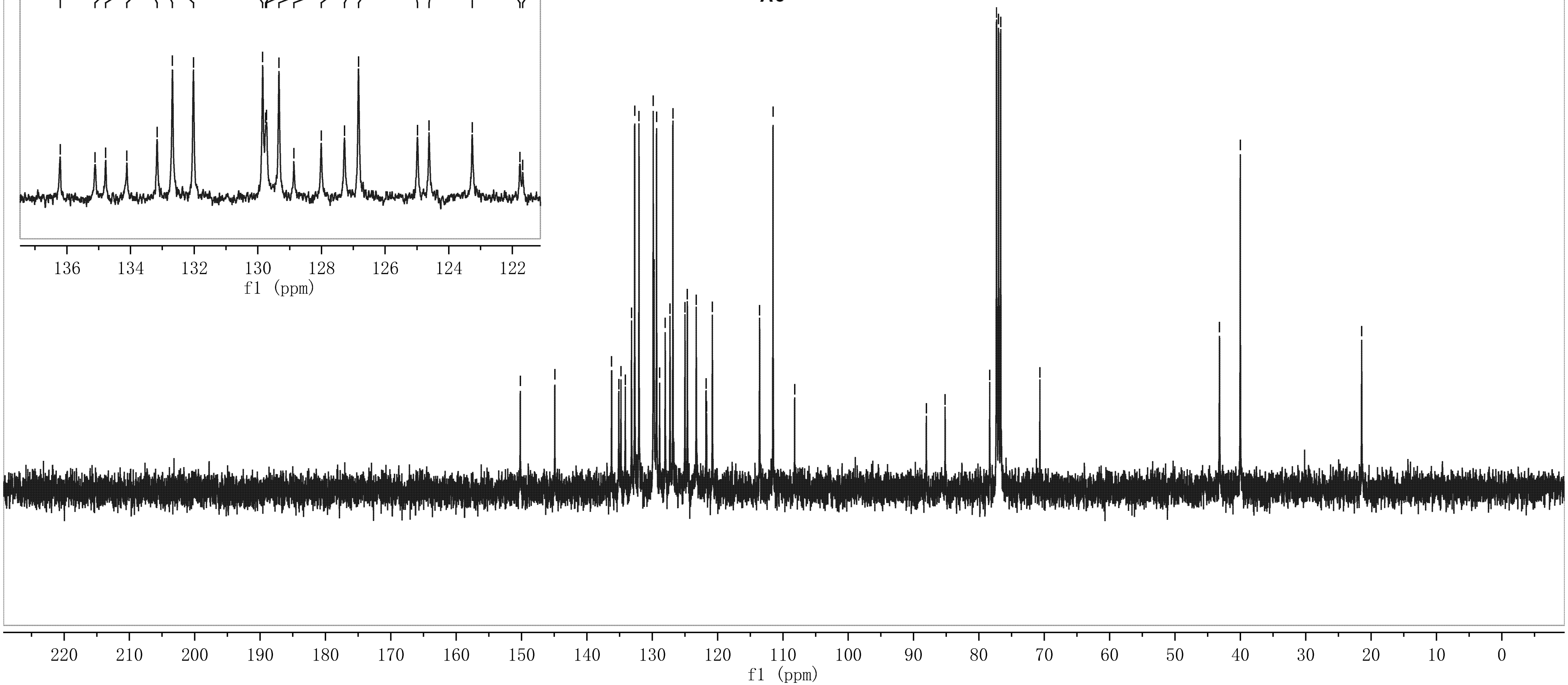

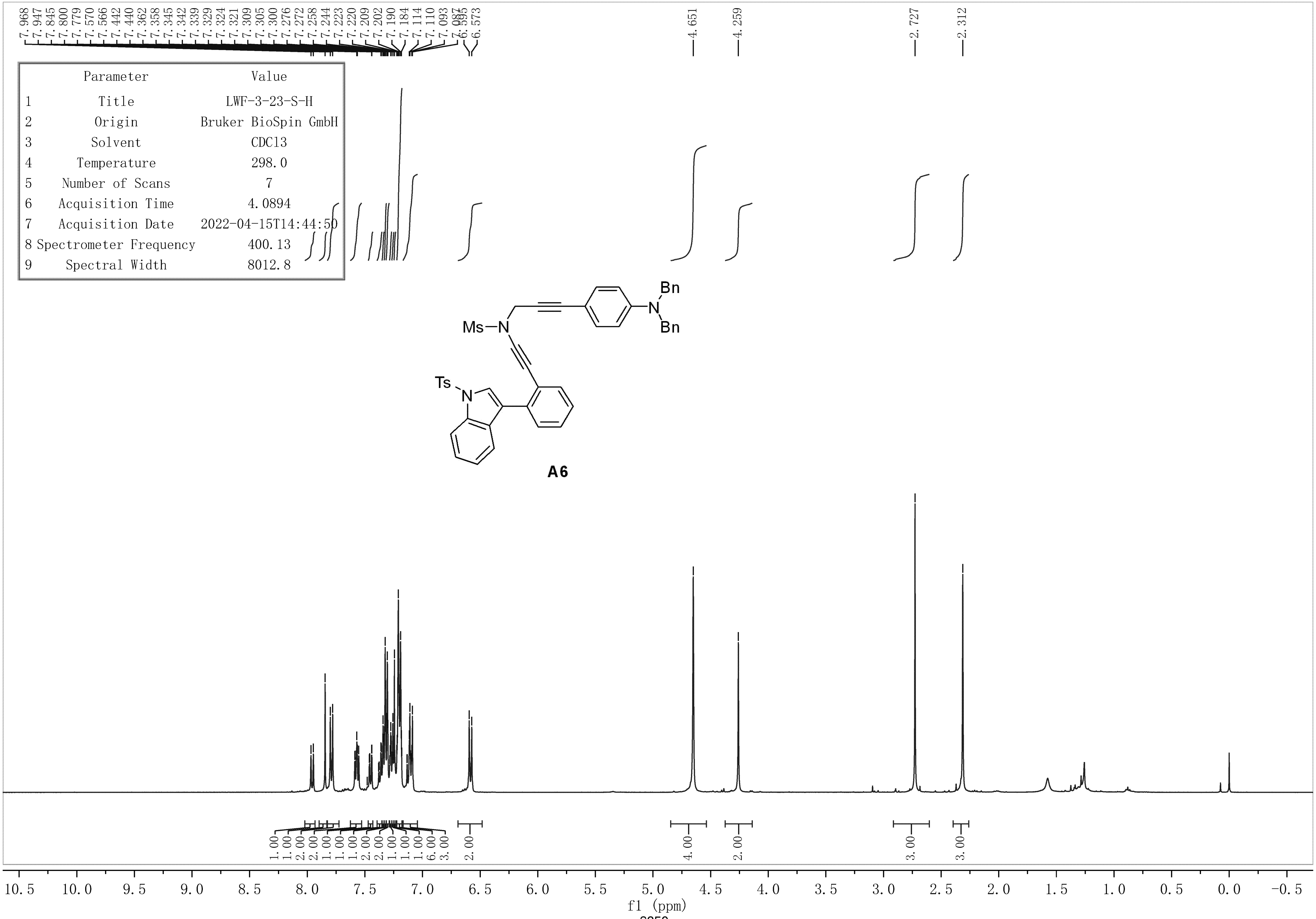

Parameter | Value
1 Title | LWF-3-23-S-H
2 Origin | Bruker BioSpin GmbH
3 Solvent | CDCl3
4 Temperature | 298.0
5 Number of Scans | 7
6 Acquisition Time | 4.0894
7 Acquisition Date | 2022-04-15T14:44:50
8 Spectrometer Frequency | 400.13
9 Spectral Width | 8012.8
7.968 7.947 7.845 7.800 7.779 7.570 7.566 7.442 7.440 7.362 7.358 7.345 7.342 7.339 7.329 7.324 7.321 7.309 7.305 7.300 7.276 7.272 7.258 7.244 7.223 7.220 7.209 7.202 7.190 7.184 7.114 7.110 7.093 6.595 6.573
4.651
4.259
2.727
2.312
Bn
N
Bn
Ms
N
Ts
N
A6
2.00
4.00
2.00
3.00
3.00
f1 (ppm)

	Parameter	Value
1	Title	LWF-3-23-S-C
2	Origin	Bruker BioSpin GmbH
3	Solvent	CDCl3
4	Temperature	300.0
5	Number of Scans	102
6	Acquisition Time	1.3631
7	Acquisition Date	2022-04-14T14:35:14
8	Spectrometer Frequency	100.61
9	Spectral Width	24038.5

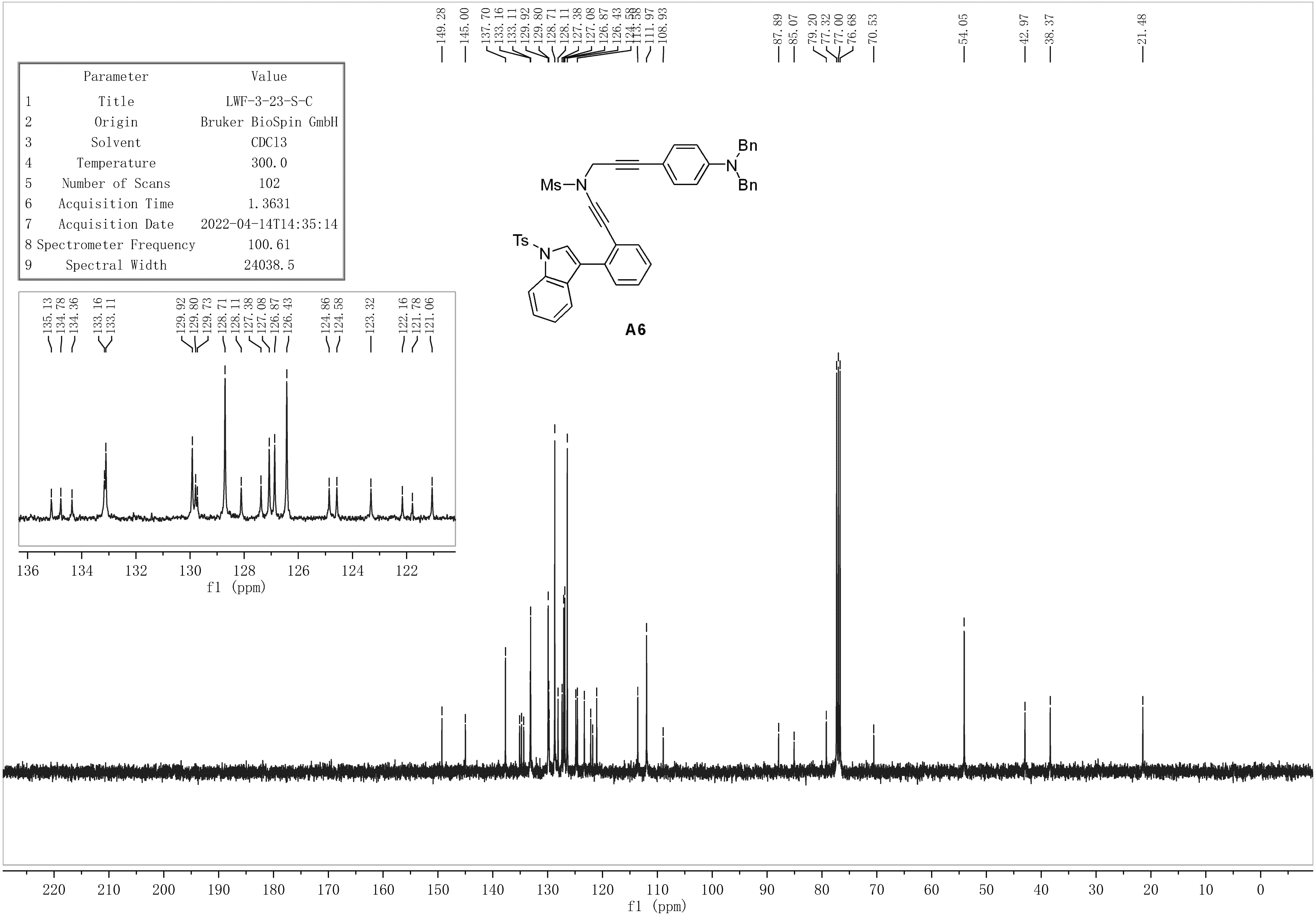

	Parameter	Value
1	Title	LWF-3-17-S-H
2	Origin	Bruker BioSpin GmbH
3	Solvent	CDCl3
4	Temperature	298.0
5	Number of Scans	9
6	Acquisition Time	4.0894
7	Acquisition Date	2022-04-15T14:29:17
8	Spectrometer Frequency	400.13
9	Spectral Width	8012.8

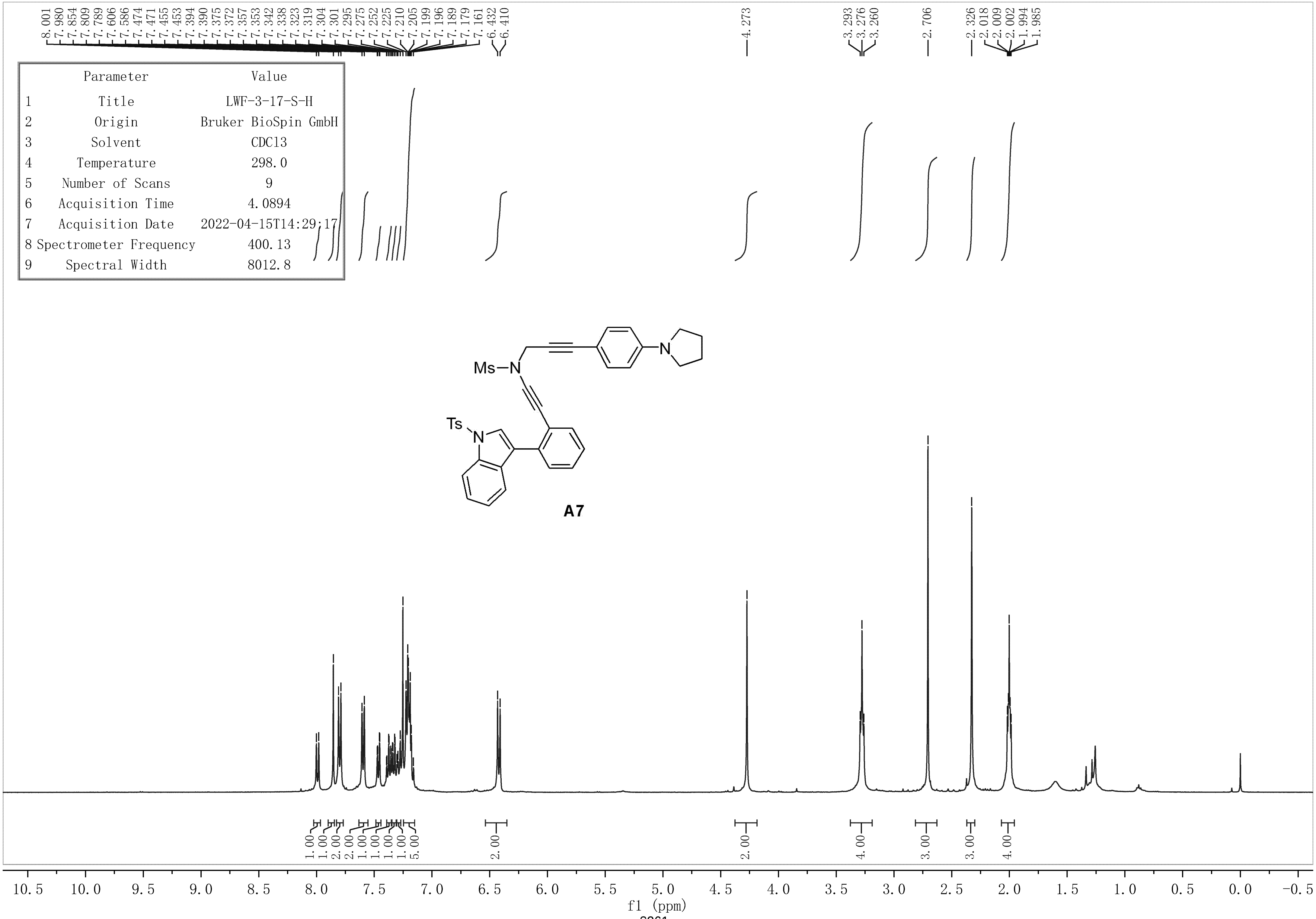

A7

	Parameter	Value
1	Title	LWF-3-17-S-C
2	Origin	Bruker BioSpin GmbH
3	Solvent	CDCl3
4	Temperature	300.0
5	Number of Scans	194
6	Acquisition Time	1.3631
7	Acquisition Date	2022-04-15T14:30:41
8	Spectrometer Frequency	100.61
9	Spectral Width	24038.5

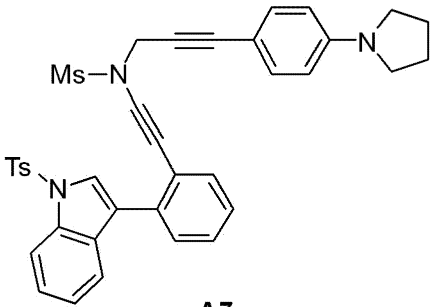

A7

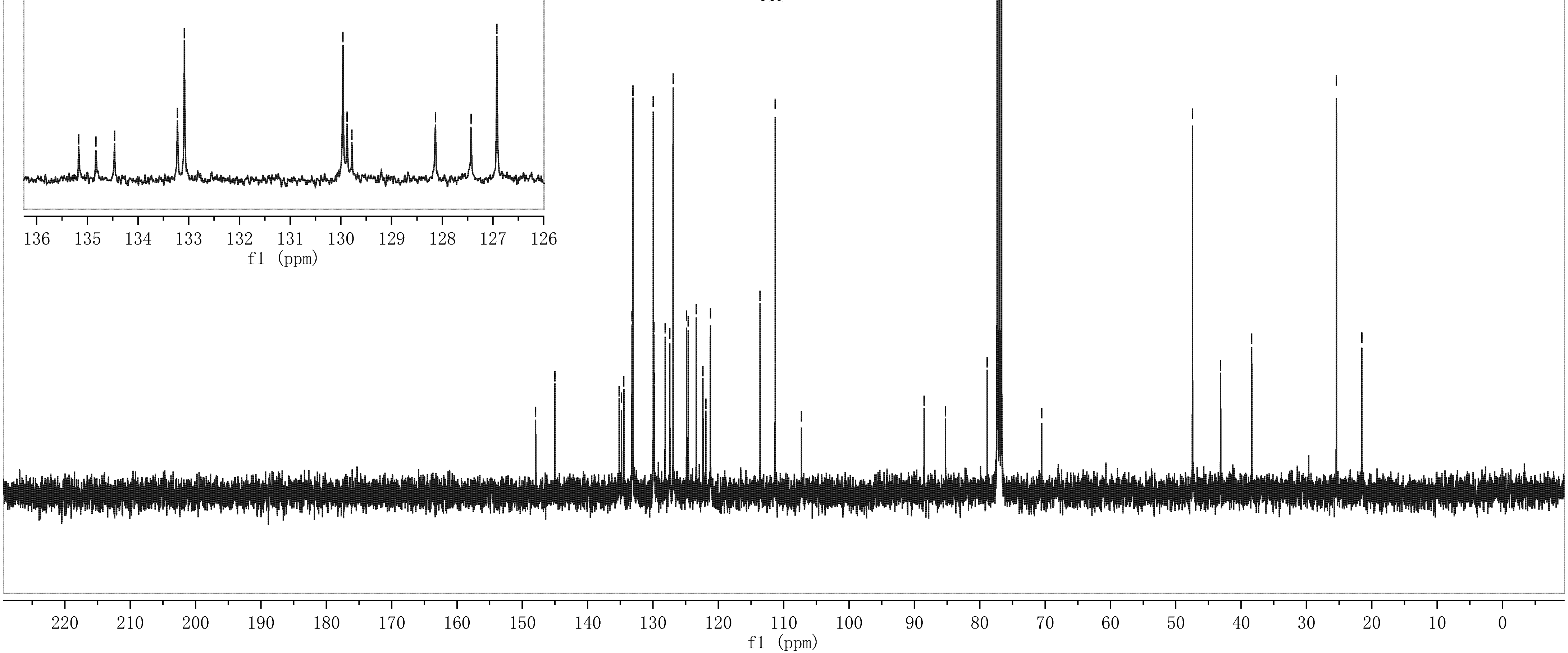

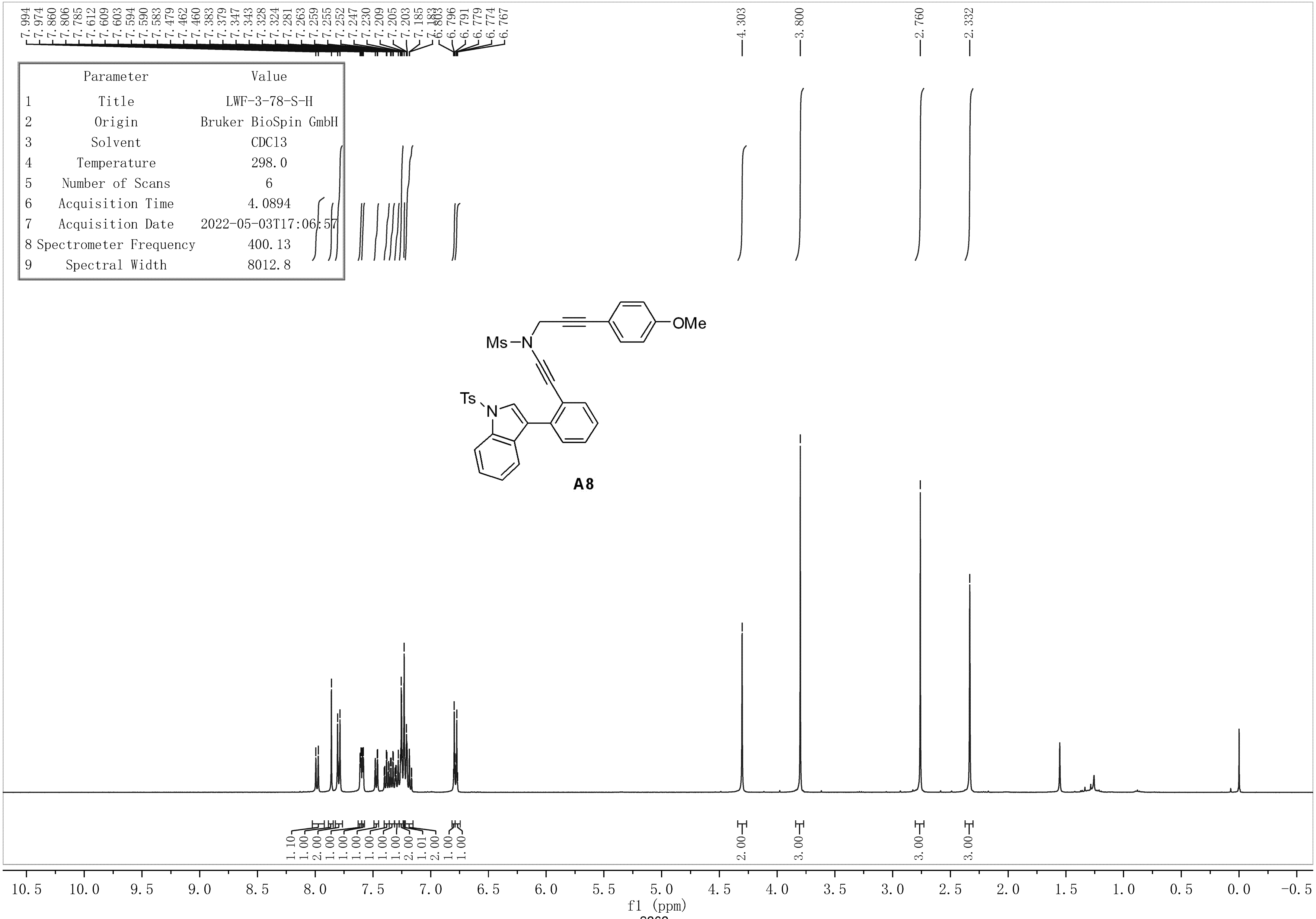
7.994 7.974 7.860 7.806 7.785 7.612 7.609 7.603 7.594 7.590 7.583 7.479 7.462 7.460 7.383 7.379 7.347 7.343 7.328 7.324 7.281 7.263 7.259 7.255 7.252 7.247 7.230 7.209 7.205 7.203 7.185 7.183 6.803 6.796 6.791 6.779 6.774 6.767
4.303
3.800
2.760
2.332
Parameter Value
1 Title LWF-3-78-S-H
2 Origin Bruker BioSpin GmbH
3 Solvent CDCl3
4 Temperature 298.0
5 Number of Scans 6
6 Acquisition Time 4.0894
7 Acquisition Date 2022-05-03T17:06:57
8 Spectrometer Frequency 400.13
9 Spectral Width 8012.8
Ms
N
OMe
Ts
N
A8
1.10 1.00 2.00 1.00 1.00 1.00 1.00 1.00 2.00 1.01 2.00 1.00 1.00
2.00
3.00
3.00
3.00
10.5 10.0 9.5 9.0 8.5 8.0 7.5 7.0 6.5 6.0 5.5 5.0 4.5 4.0 3.5 3.0 2.5 2.0 1.5 1.0 0.5 0.0 -0.5
f1 (ppm)

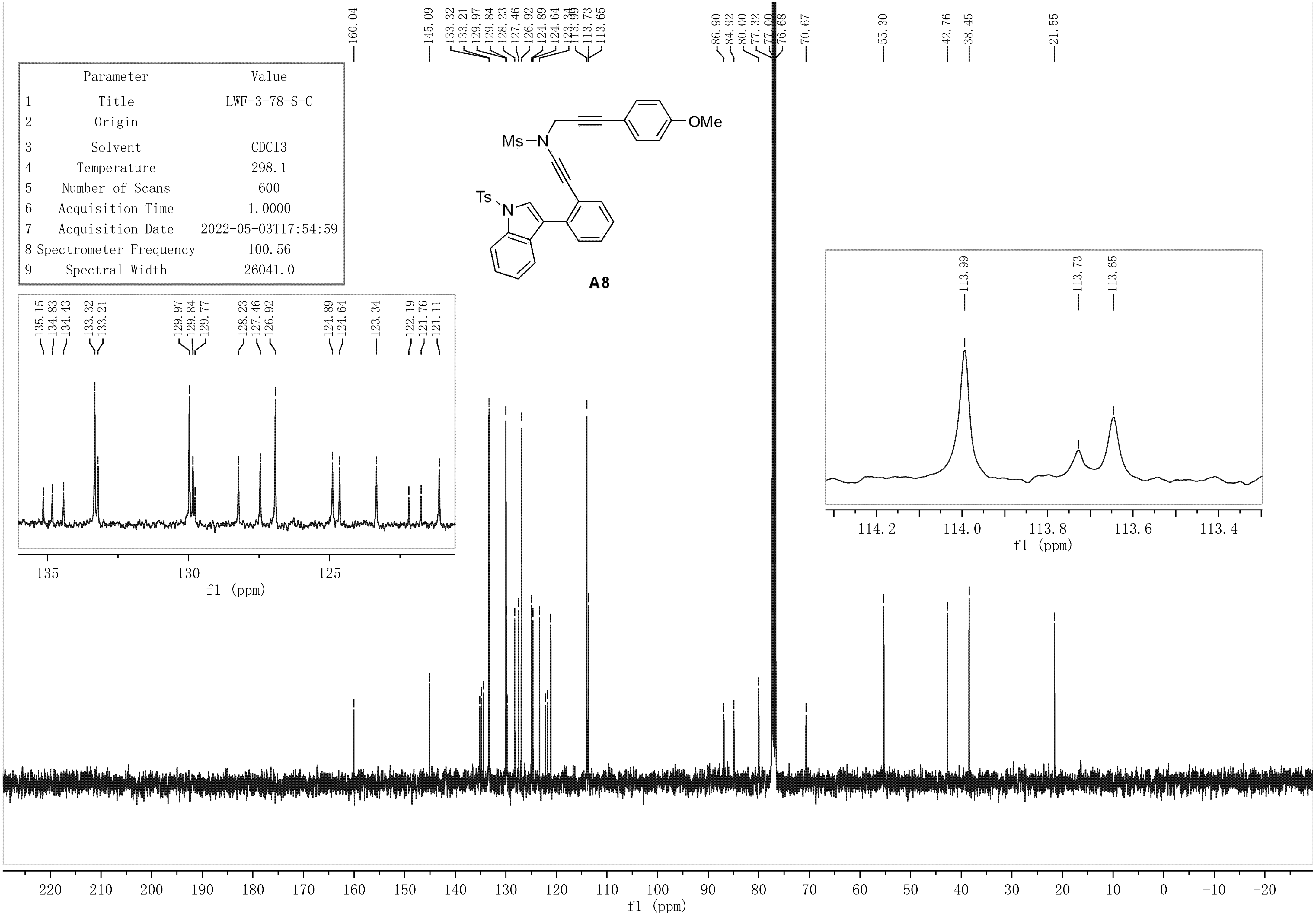

160.04
145.09
133.32
133.21
129.97
129.84
128.23
127.46
126.92
124.89
124.64
123.34
113.99
113.73
113.65
86.90
84.92
80.00
77.32
77.00
76.68
70.67
55.30
42.76
38.45
21.55
Parameter Value
1 Title LWF-3-78-S-C
2 Origin
3 Solvent CDCl3
4 Temperature 298.1
5 Number of Scans 600
6 Acquisition Time 1.0000
7 Acquisition Date 2022-05-03T17:54:59
8 Spectrometer Frequency 100.56
9 Spectral Width 26041.0
Ms
N
OMe
Ts
A8
135.15
134.83
134.43
133.32
133.21
129.97
129.84
129.77
128.23
127.46
126.92
124.89
124.64
123.34
122.19
121.76
121.11
f1 (ppm)
113.99
113.73
113.65
f1 (ppm)
f1 (ppm)

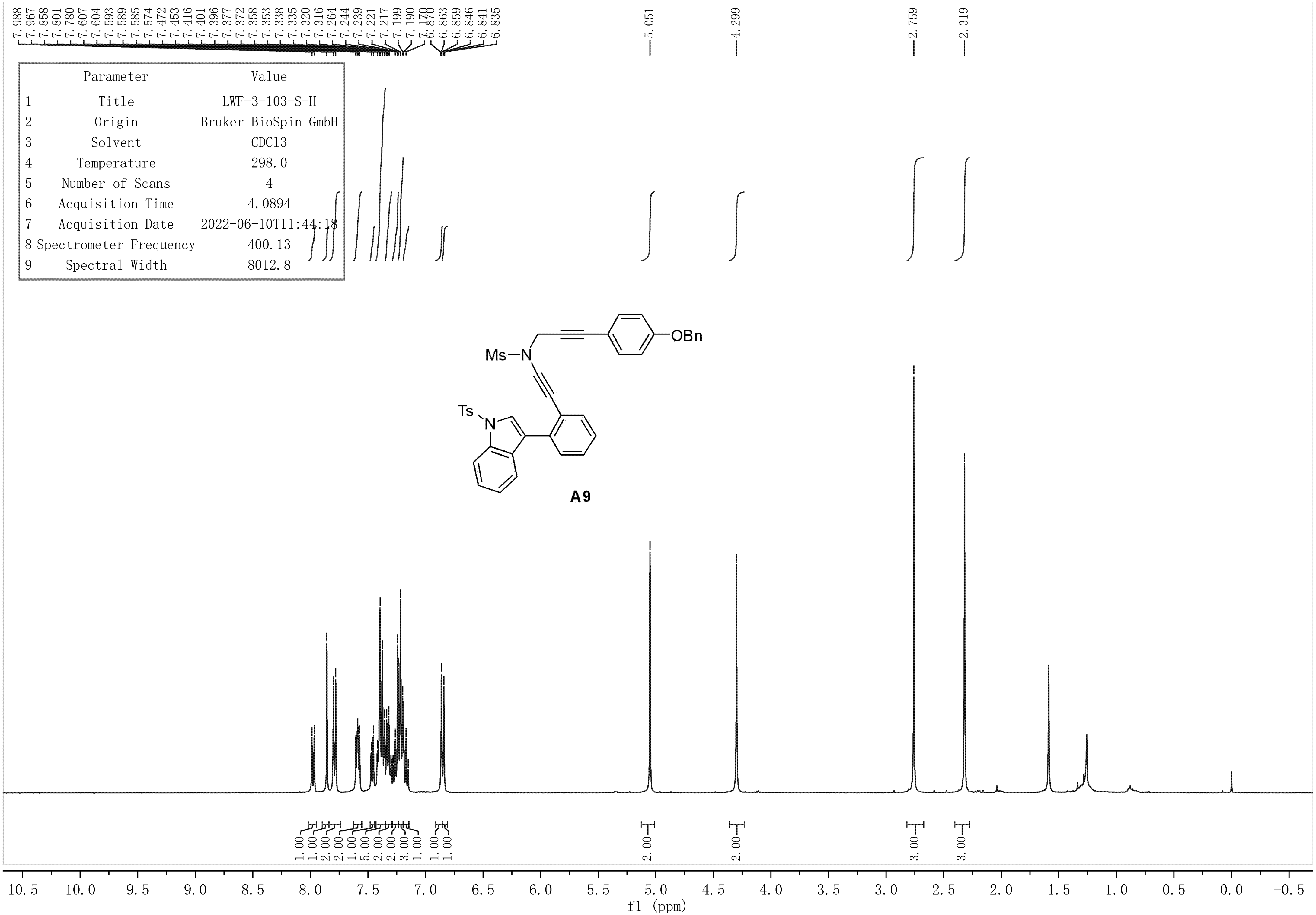

	Parameter	Value
1	Title	LWF-3-103-S-H
2	Origin	Bruker BioSpin GmbH
3	Solvent	CDCl3
4	Temperature	298.0
5	Number of Scans	4
6	Acquisition Time	4.0894
7	Acquisition Date	2022-06-10T11:44:18
8	Spectrometer Frequency	400.13
9	Spectral Width	8012.8

A9

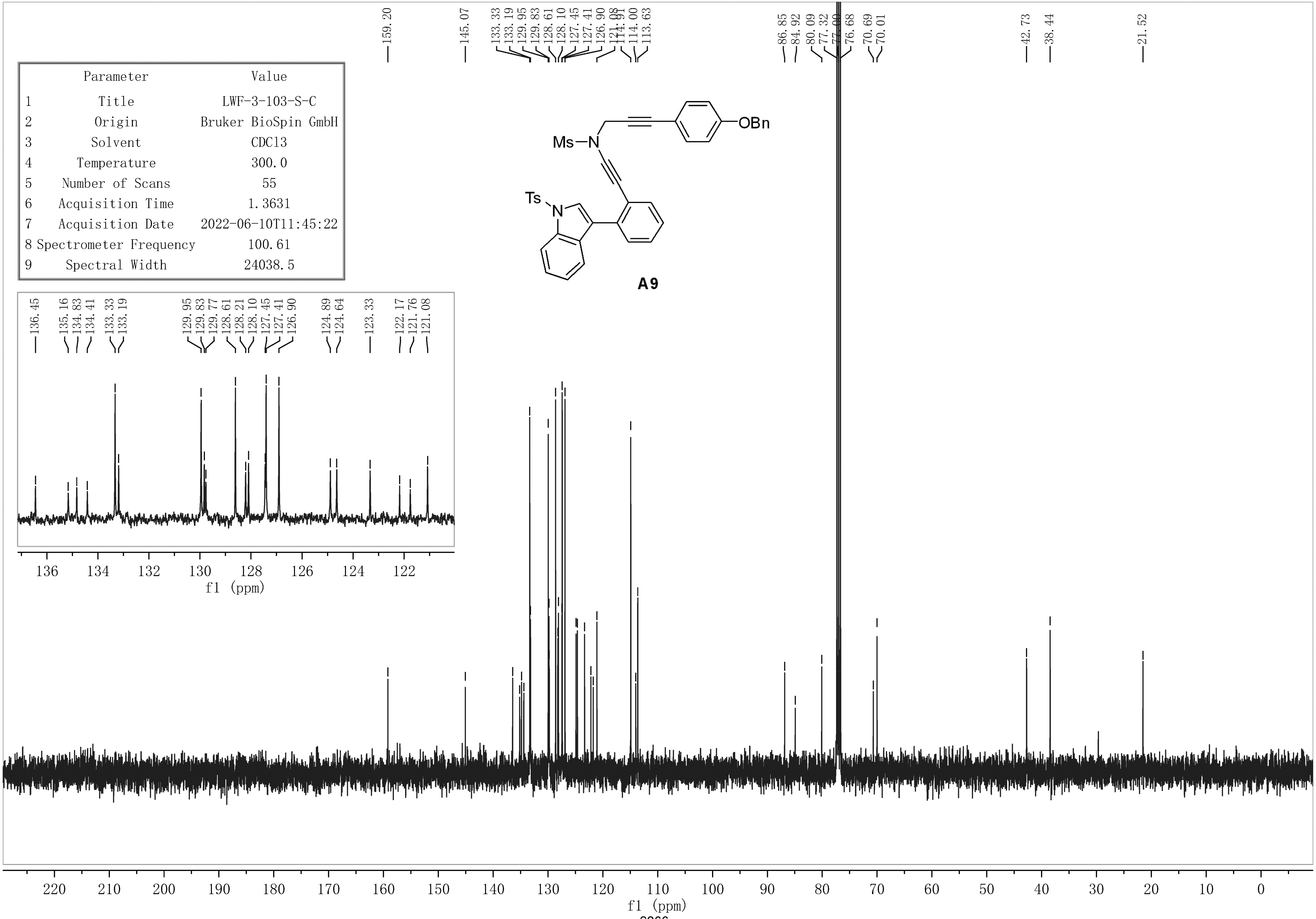

	Parameter	Value
1	Title	LWF-3-103-S-C
2	Origin	Bruker BioSpin GmbH
3	Solvent	CDCl3
4	Temperature	300.0
5	Number of Scans	55
6	Acquisition Time	1.3631
7	Acquisition Date	2022-06-10T11:45:22
8	Spectrometer Frequency	100.61
9	Spectral Width	24038.5

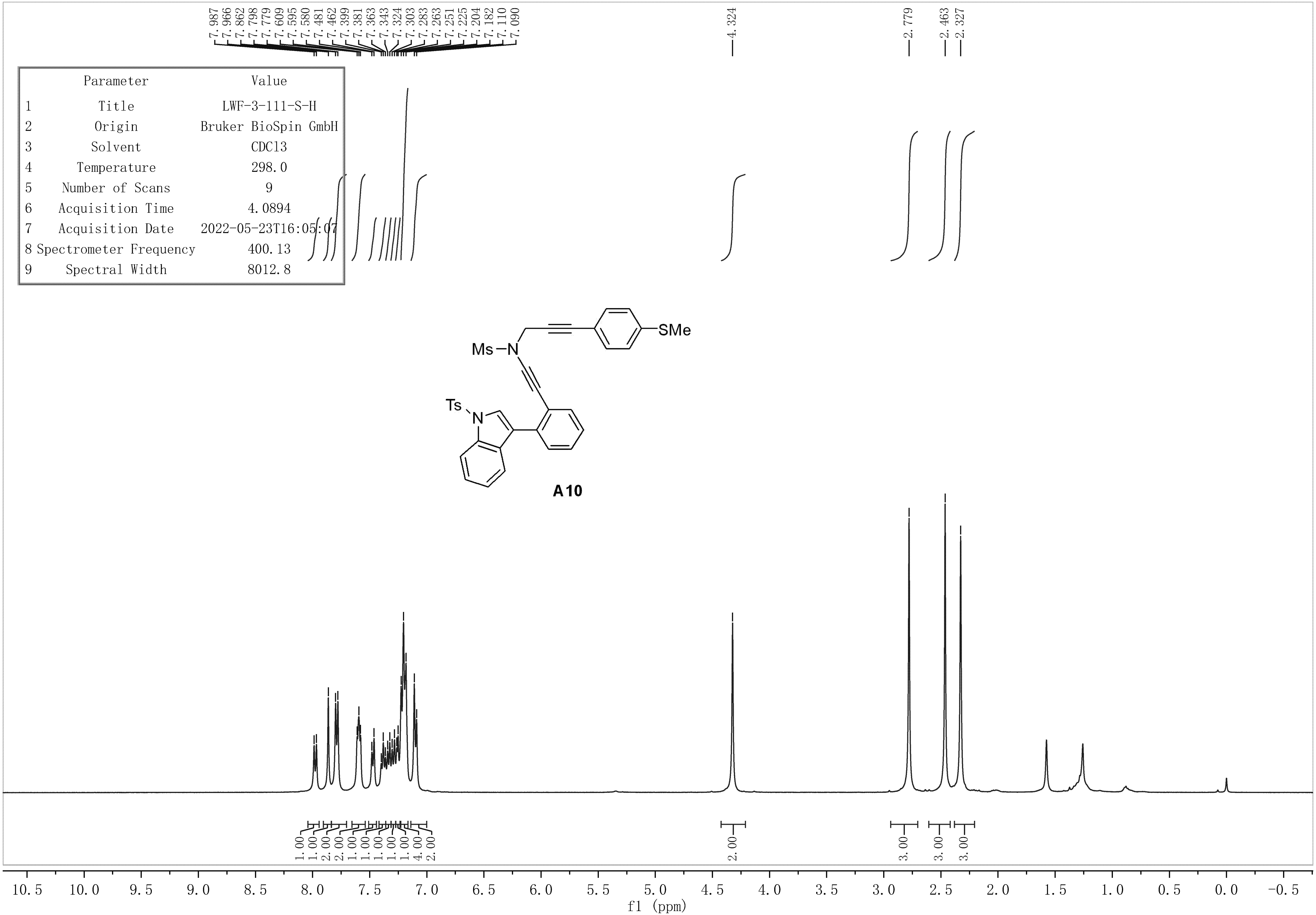
7.987 7.966 7.862 7.798 7.779 7.609 7.595 7.580 7.481 7.462 7.399 7.381 7.363 7.343 7.324 7.303 7.283 7.263 7.251 7.225 7.204 7.182 7.110 7.090
4.324
2.779 2.463 2.327
Parameter Value
1 Title LWF-3-111-S-H
2 Origin Bruker BioSpin GmbH
3 Solvent CDCl3
4 Temperature 298.0
5 Number of Scans 9
6 Acquisition Time 4.0894
7 Acquisition Date 2022-05-23T16:05:07
8 Spectrometer Frequency 400.13
9 Spectral Width 8012.8
Ms
N
SMe
Ts
N
A10
1.00 1.00 2.00 2.00 1.00 1.00 1.00 1.00 1.00 4.00 2.00
2.00
3.00 3.00 3.00
10.5 10.0 9.5 9.0 8.5 8.0 7.5 7.0 6.5 6.0 5.5 5.0 4.5 4.0 3.5 3.0 2.5 2.0 1.5 1.0 0.5 0.0 -0.5
f1 (ppm)

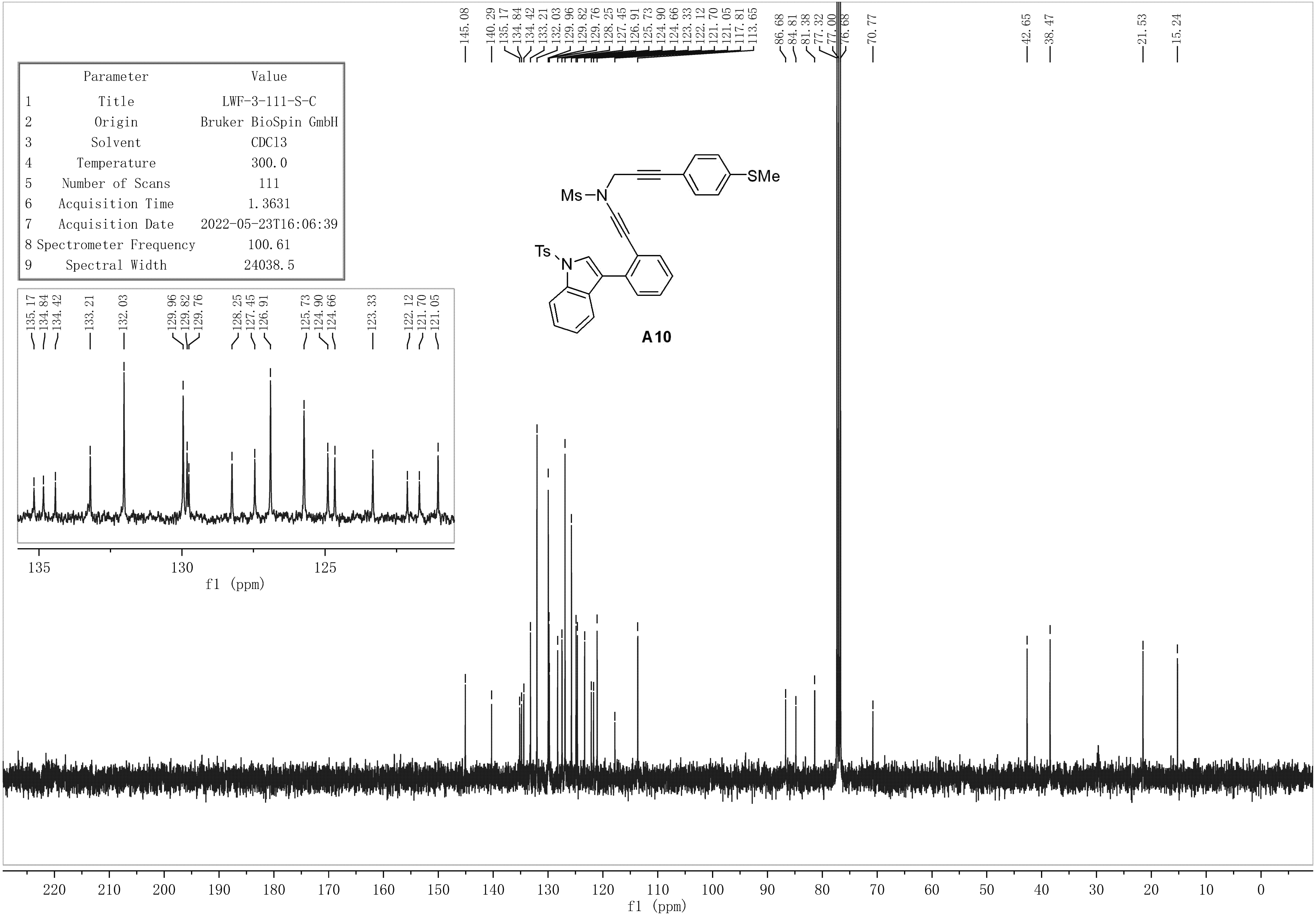

	Parameter	Value
1	Title	LWF-3-111-S-C
2	Origin	Bruker BioSpin GmbH
3	Solvent	CDCl3
4	Temperature	300.0
5	Number of Scans	111
6	Acquisition Time	1.3631
7	Acquisition Date	2022-05-23T16:06:39
8	Spectrometer Frequency	100.61
9	Spectral Width	24038.5

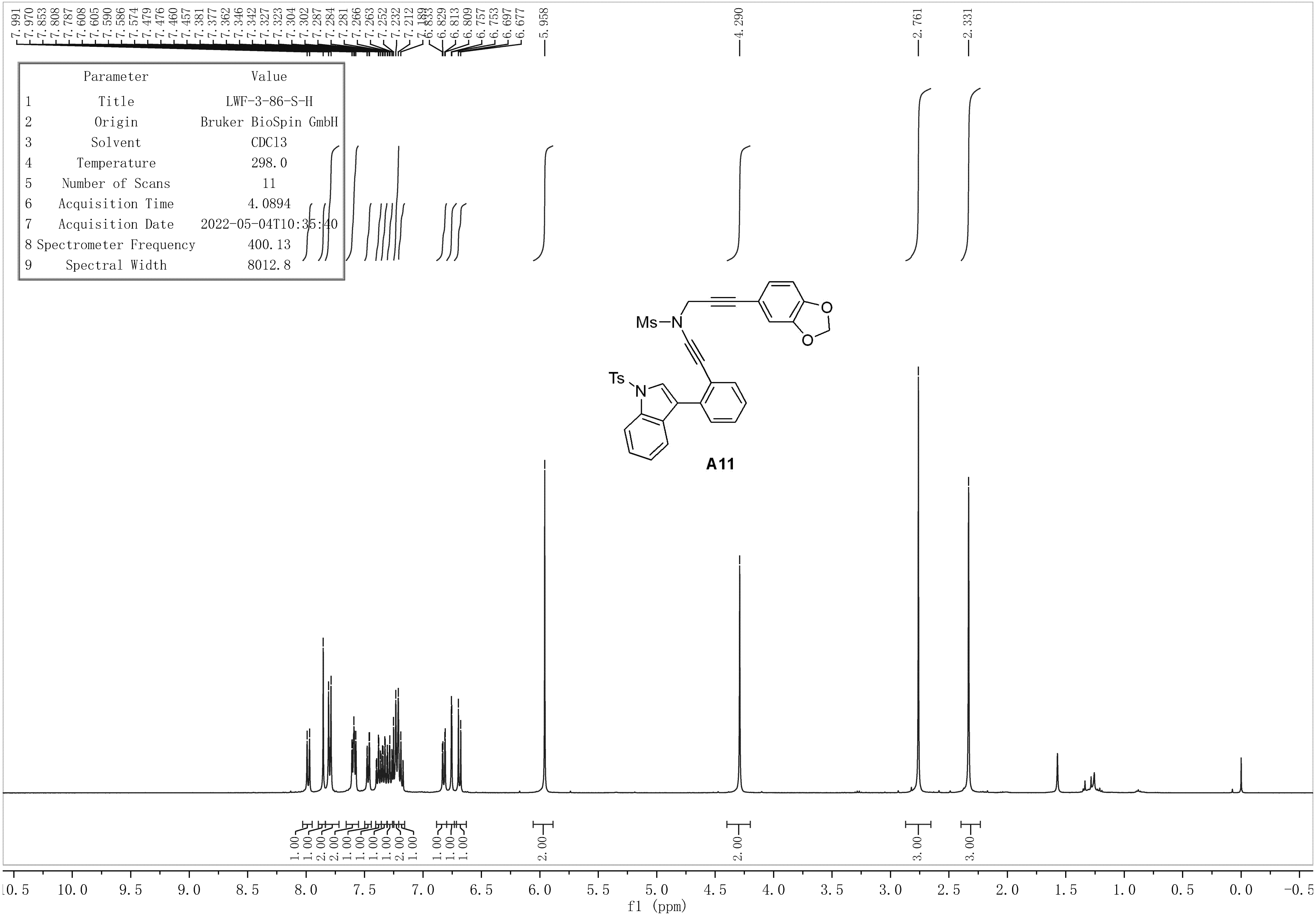
7.991 7.970 7.853 7.808 7.787 7.608 7.605 7.590 7.586 7.574 7.479 7.476 7.460 7.457 7.381 7.377 7.362 7.346 7.342 7.327 7.323 7.304 7.302 7.287 7.284 7.281 7.266 7.263 7.252 7.232 7.212 7.189 6.833 6.829 6.813 6.809 6.757 6.753 6.697 6.677
5.958
4.290
2.761
2.331
Parameter Value
1 Title LWF-3-86-S-H
2 Origin Bruker BioSpin GmbH
3 Solvent CDCl3
4 Temperature 298.0
5 Number of Scans 11
6 Acquisition Time 4.0894
7 Acquisition Date 2022-05-04T10:35:40
8 Spectrometer Frequency 400.13
9 Spectral Width 8012.8
Ms
N
O
Ts
A11
1.00 1.00 2.00 2.00 1.00 1.00 1.00 1.00 2.00 1.00 1.00 1.00 1.00
2.00
2.00
3.00
3.00
10.5 10.0 9.5 9.0 8.5 8.0 7.5 7.0 6.5 6.0 5.5 5.0 4.5 4.0 3.5 3.0 2.5 2.0 1.5 1.0 0.5 0.0 -0.5
f1 (ppm)

	Parameter	Value
1	Title	LWF-3-86-S-C
2	Origin	
3	Solvent	CDCl3
4	Temperature	298.0
5	Number of Scans	600
6	Acquisition Time	1.0000
7	Acquisition Date	2022-05-03T11:26:00
8	Spectrometer Frequency	100.56
9	Spectral Width	26041.0

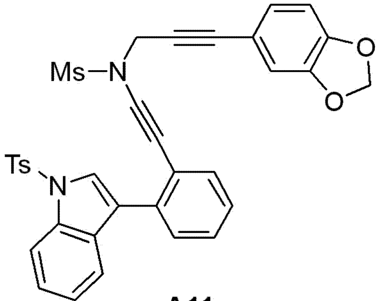

A11

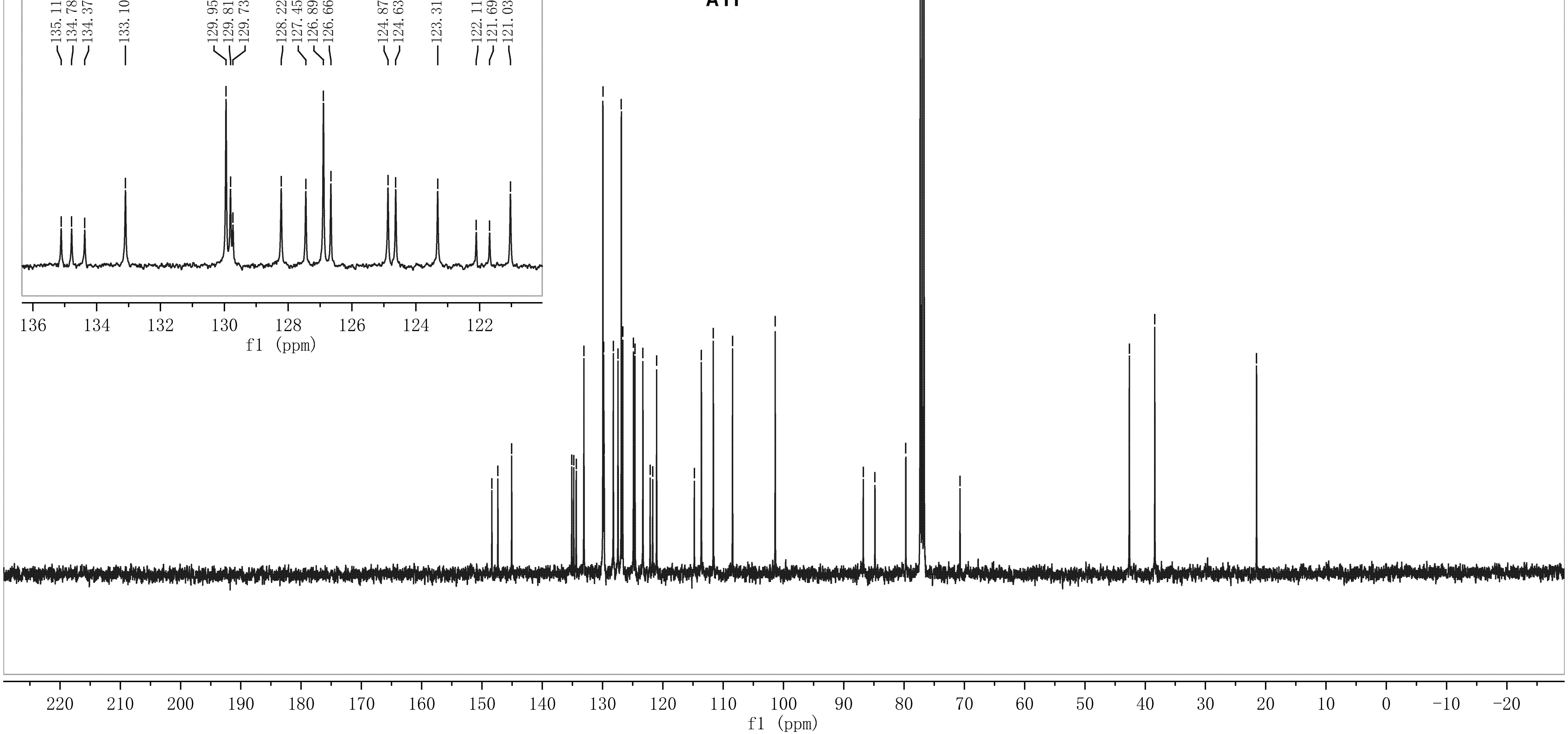

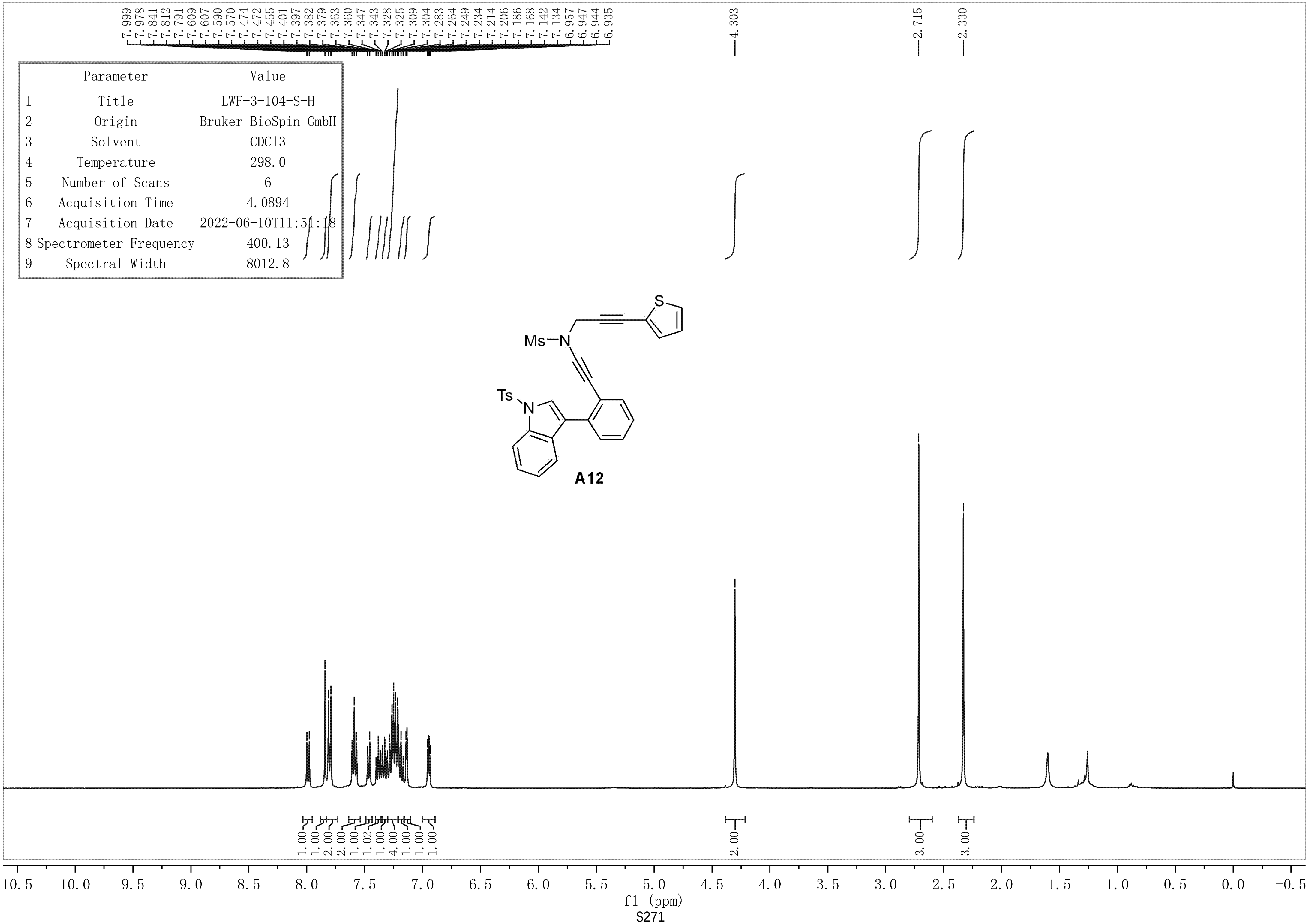
7.999 7.978 7.841 7.812 7.791 7.609 7.607 7.590 7.570 7.474 7.472 7.455 7.401 7.397 7.382 7.379 7.363 7.360 7.347 7.343 7.328 7.325 7.309 7.304 7.283 7.264 7.249 7.234 7.214 7.206 7.186 7.168 7.142 7.134 6.957 6.947 6.944 6.935
4.303
2.715
2.330
Parameter Value
1 Title LWF-3-104-S-H
2 Origin Bruker BioSpin GmbH
3 Solvent CDCl3
4 Temperature 298.0
5 Number of Scans 6
6 Acquisition Time 4.0894
7 Acquisition Date 2022-06-10T11:51:18
8 Spectrometer Frequency 400.13
9 Spectral Width 8012.8
Ms
N
S
Ts
N
A12
1.00 1.00 2.00 2.00 1.00 1.02 1.00 4.00 1.00 1.00 1.00
2.00
3.00
3.00
10.5 10.0 9.5 9.0 8.5 8.0 7.5 7.0 6.5 6.0 5.5 5.0 4.5 4.0 3.5 3.0 2.5 2.0 1.5 1.0 0.5 0.0 -0.5
f1 (ppm)

	Parameter	Value
1	Title	LWF-3-104-S-C
2	Origin	Bruker BioSpin GmbH
3	Solvent	CDCl3
4	Temperature	300.0
5	Number of Scans	46
6	Acquisition Time	1.3631
7	Acquisition Date	2022-06-10T11:52:49
8	Spectrometer Frequency	100.61
9	Spectral Width	24038.5

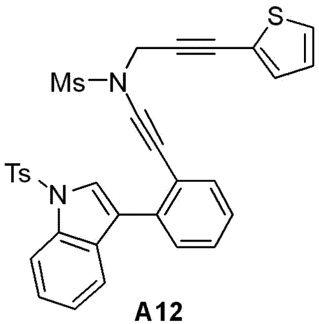

A12

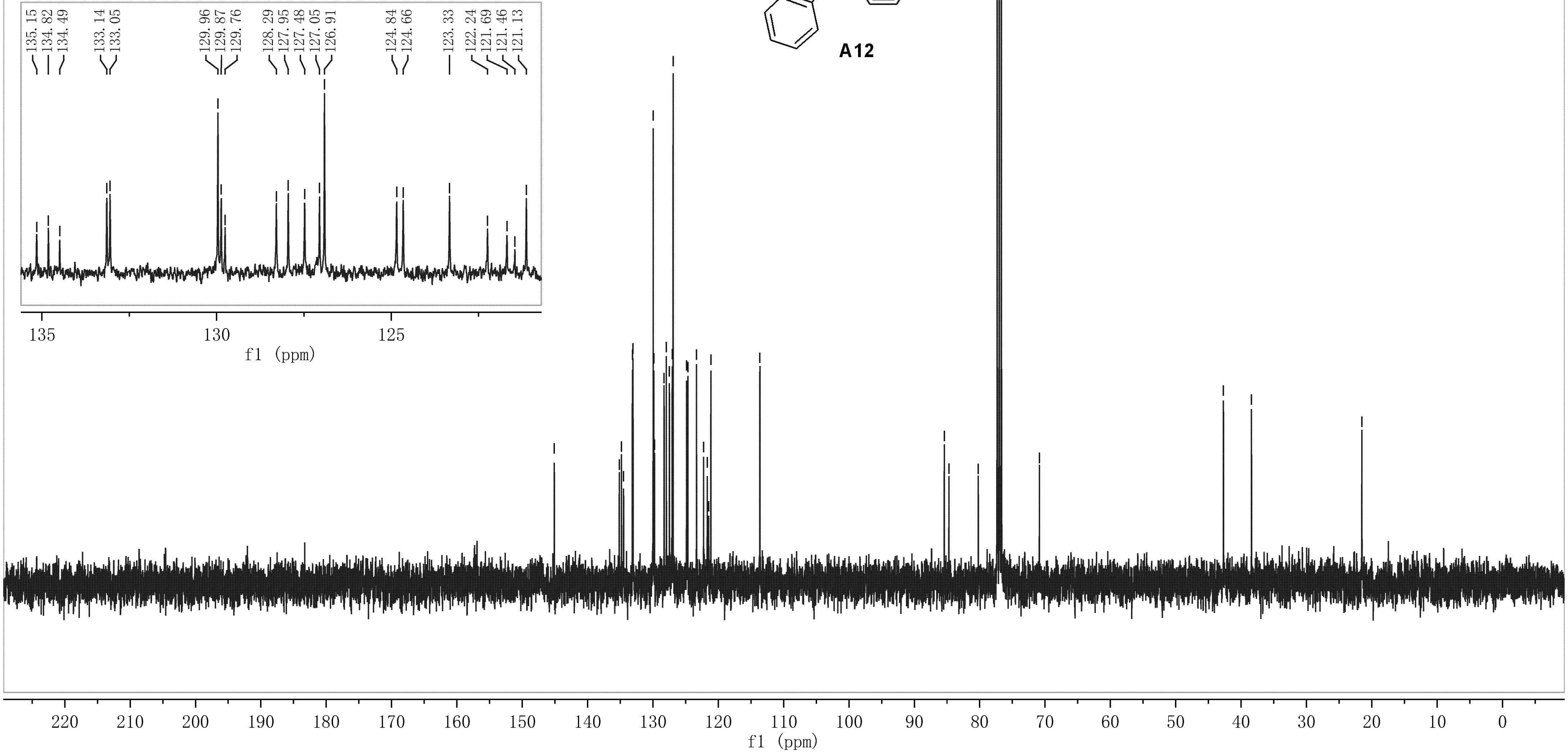

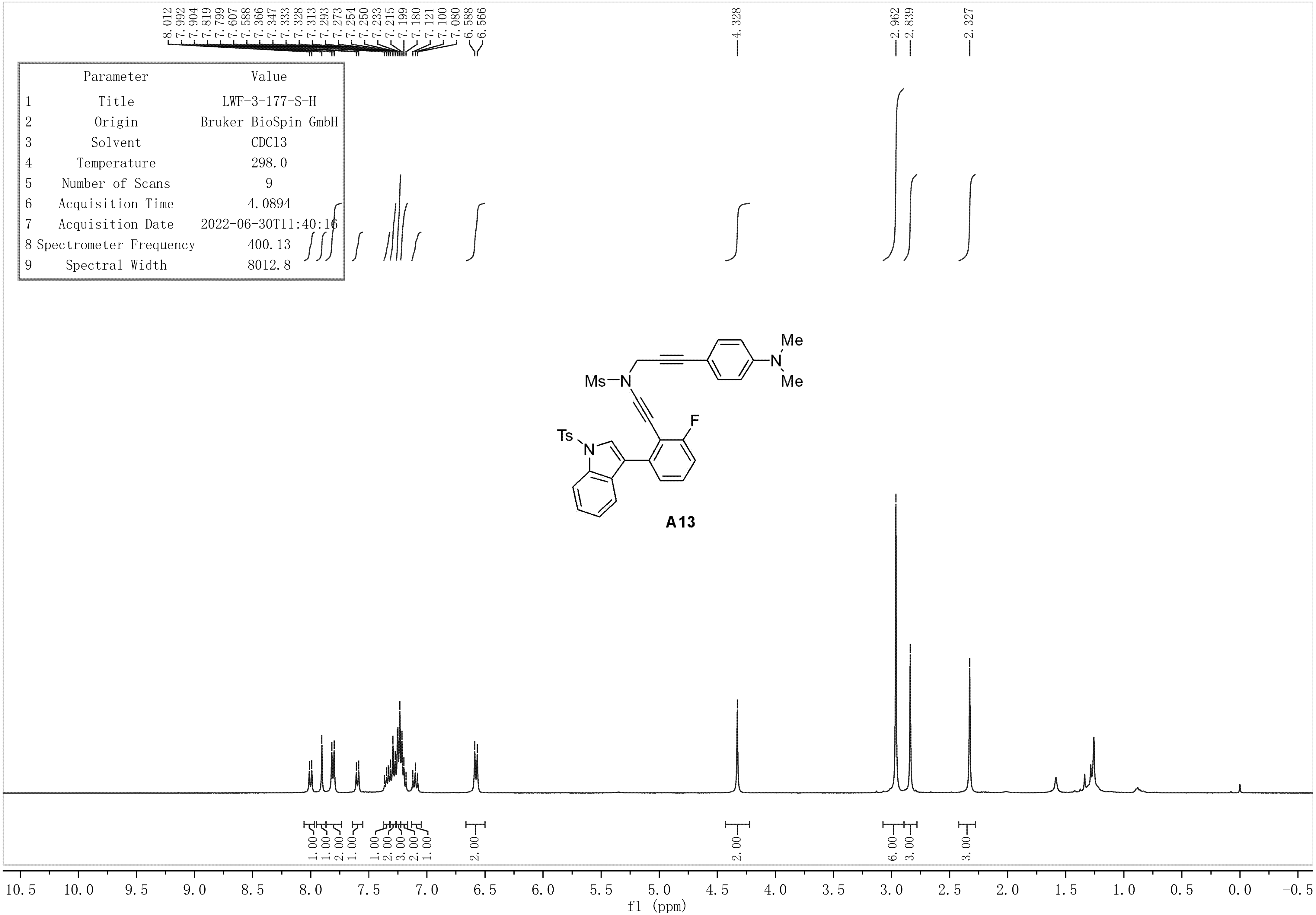
Parameter Value
1 Title LWF-3-177-S-H
2 Origin Bruker BioSpin GmbH
3 Solvent CDCl3
4 Temperature 298.0
5 Number of Scans 9
6 Acquisition Time 4.0894
7 Acquisition Date 2022-06-30T11:40:16
8 Spectrometer Frequency 400.13
9 Spectral Width 8012.8
8.012 7.992 7.904 7.819 7.799 7.607 7.588 7.366 7.347 7.333 7.328 7.313 7.293 7.273 7.254 7.250 7.233 7.215 7.199 7.180 7.121 7.100 7.080 6.588 6.566
4.328
2.962 2.839
2.327
Ms
N
Me
Me
F
Ts
A13
1.00 1.00 2.00 1.00 1.00 2.00 3.00 2.00 1.00 2.00
2.00
6.00 3.00
3.00
10.5 10.0 9.5 9.0 8.5 8.0 7.5 7.0 6.5 6.0 5.5 5.0 4.5 4.0 3.5 3.0 2.5 2.0 1.5 1.0 0.5 0.0 -0.5
f1 (ppm)

Parameter	Value
1 Title	LWF-3-177-S-C
2 Origin	Bruker BioSpin GmbH
3 Solvent	CDCl3
4 Temperature	300.0
5 Number of Scans	332
6 Acquisition Time	1.3631
7 Acquisition Date	2022-06-30T11:42:25
8 Spectrometer Frequency	100.61
9 Spectral Width	24038.5

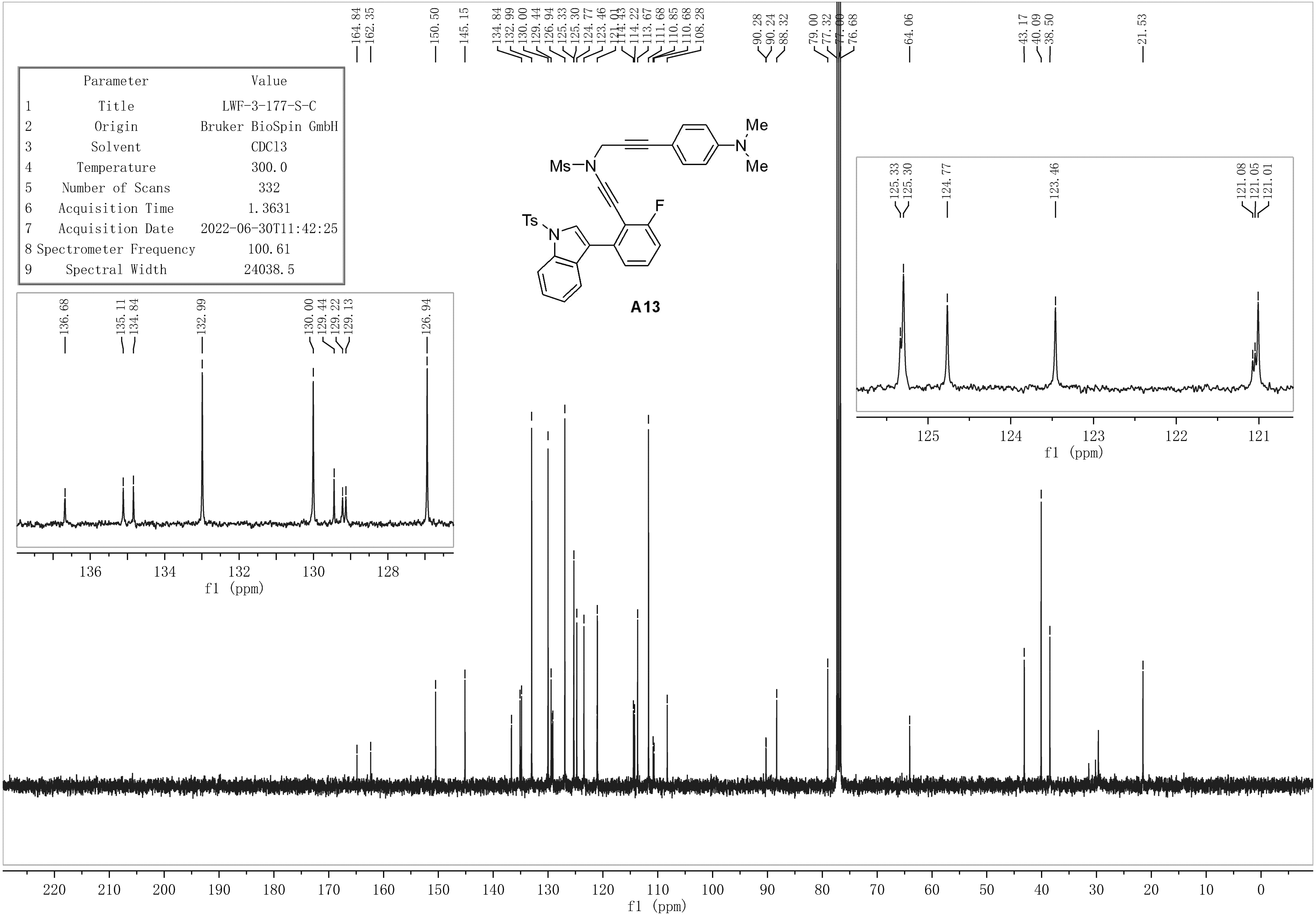

A13

	Parameter	Value
1	Title	LWF-3-177-S-F
2	Origin	
3	Solvent	CDCl3
4	Temperature	299.4
5	Number of Scans	100
6	Acquisition Time	1.0000
7	Acquisition Date	2022-06-30T14:15:06
8	Spectrometer Frequency	376.30
9	Spectral Width	96153.0

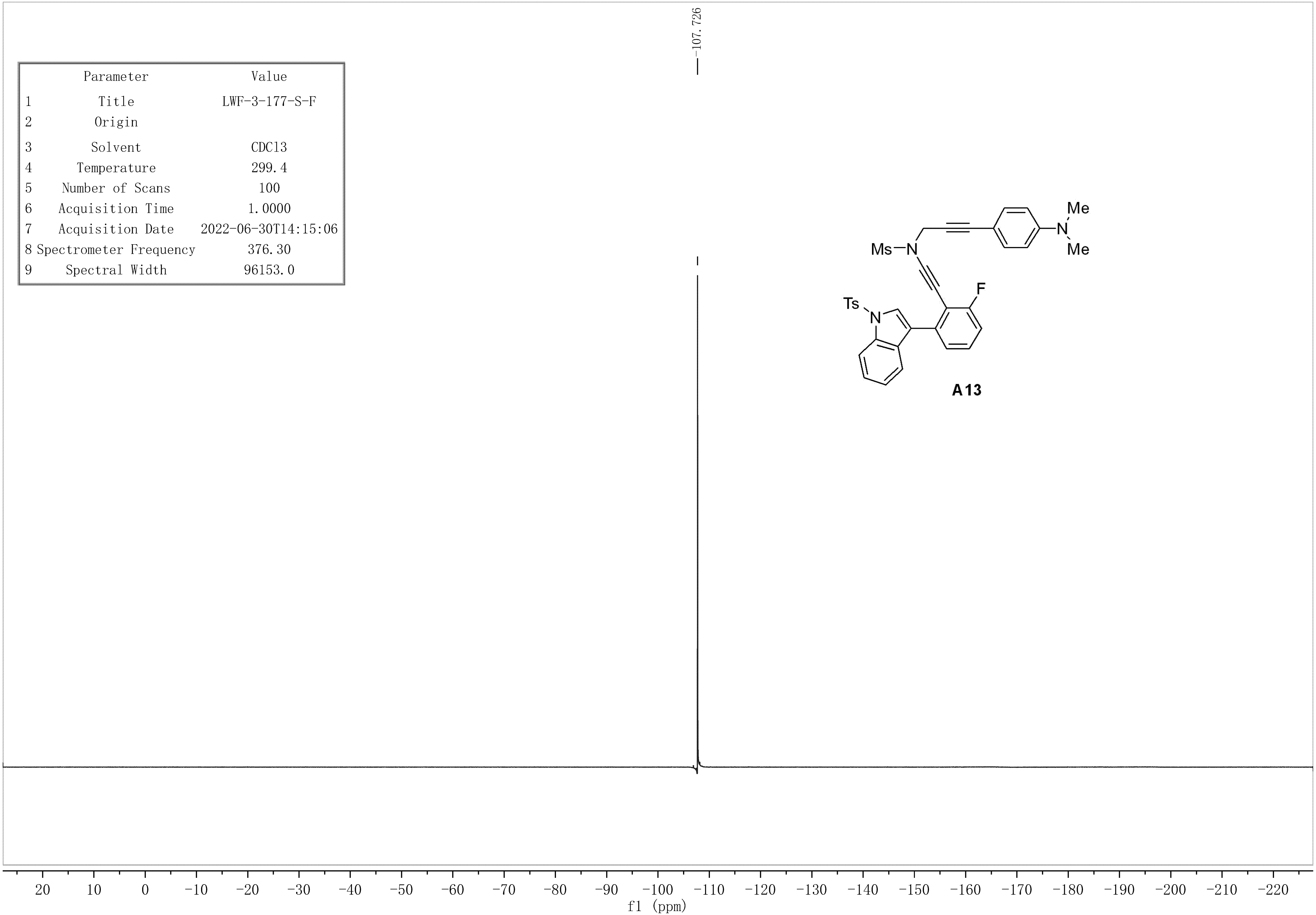

A13

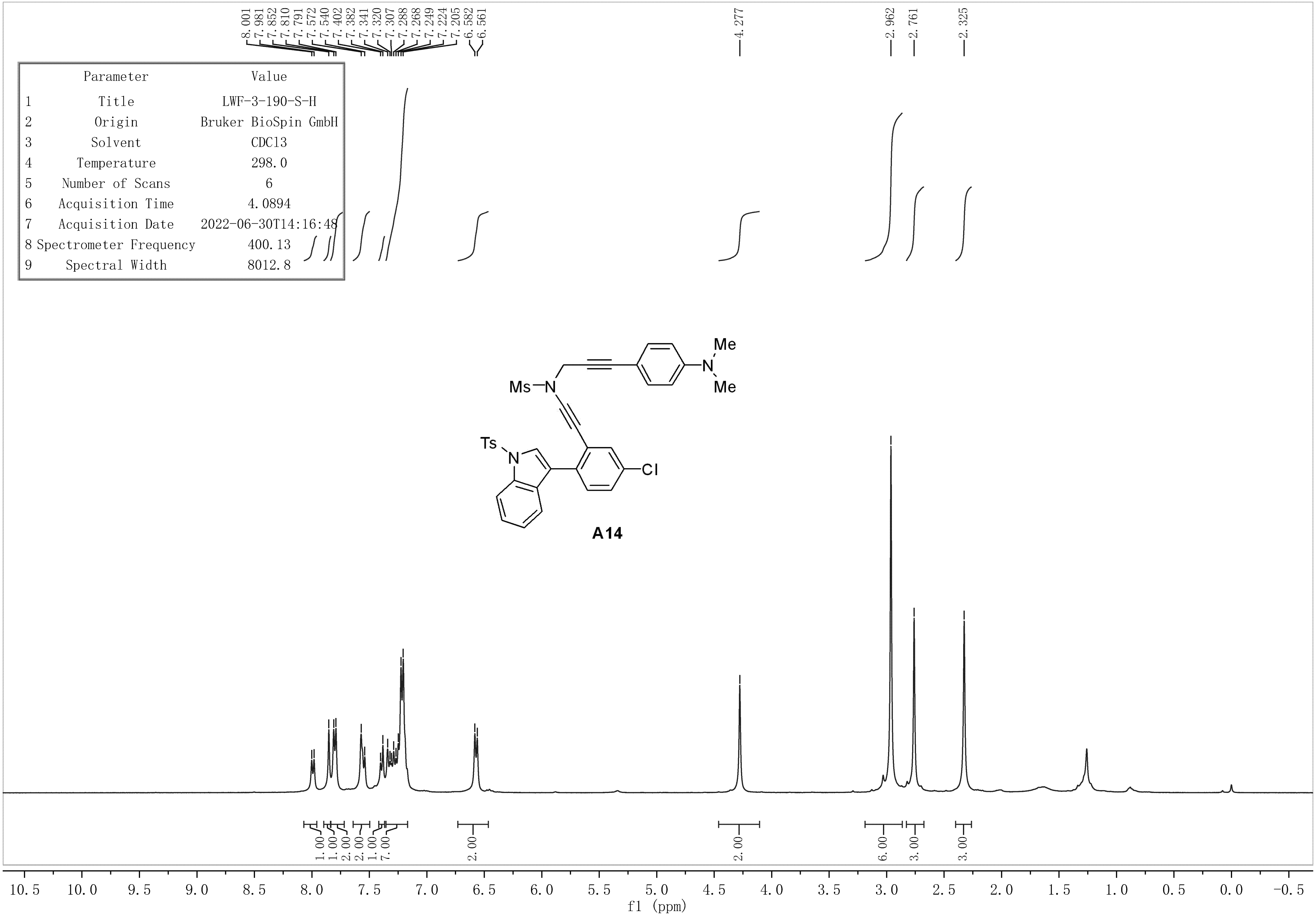
8.001
7.981
7.852
7.810
7.791
7.572
7.540
7.402
7.382
7.341
7.320
7.307
7.288
7.268
7.249
7.224
7.205
6.582
6.561
4.277
2.962
2.761
2.325
Parameter Value
1 Title LWF-3-190-S-H
2 Origin Bruker BioSpin GmbH
3 Solvent CDCl3
4 Temperature 298.0
5 Number of Scans 6
6 Acquisition Time 4.0894
7 Acquisition Date 2022-06-30T14:16:48
8 Spectrometer Frequency 400.13
9 Spectral Width 8012.8
Me
N
Me
Ms
N
Ts
N
Cl
A14
1.00
1.00
2.00
2.00
1.00
7.00
2.00
2.00
6.00
3.00
3.00
10.5 10.0 9.5 9.0 8.5 8.0 7.5 7.0 6.5 6.0 5.5 5.0 4.5 4.0 3.5 3.0 2.5 2.0 1.5 1.0 0.5 0.0 -0.5
f1 (ppm)

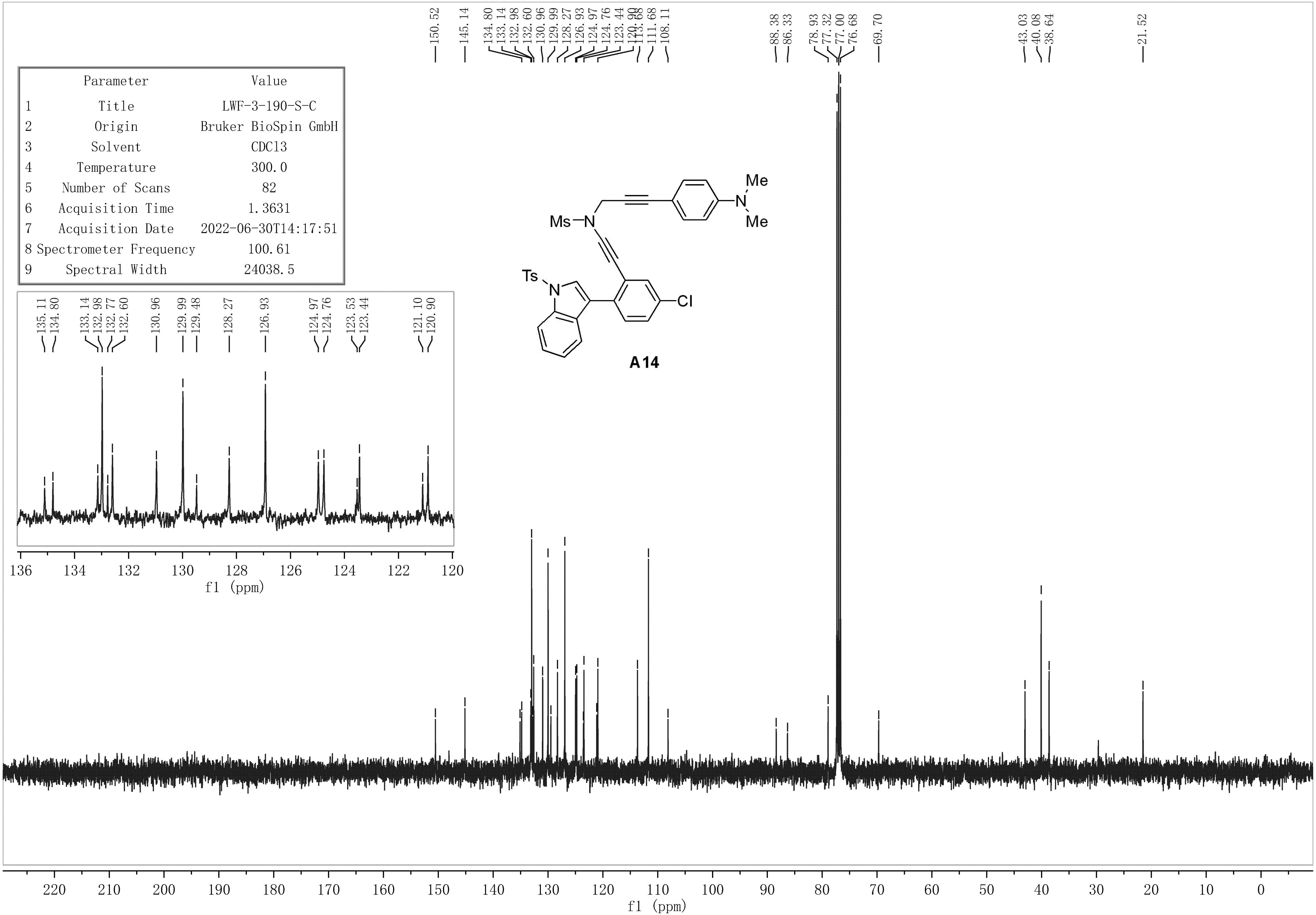

	Parameter	Value
1	Title	LWF-3-190-S-C
2	Origin	Bruker BioSpin GmbH
3	Solvent	CDCl3
4	Temperature	300.0
5	Number of Scans	82
6	Acquisition Time	1.3631
7	Acquisition Date	2022-06-30T14:17:51
8	Spectrometer Frequency	100.61
9	Spectral Width	24038.5

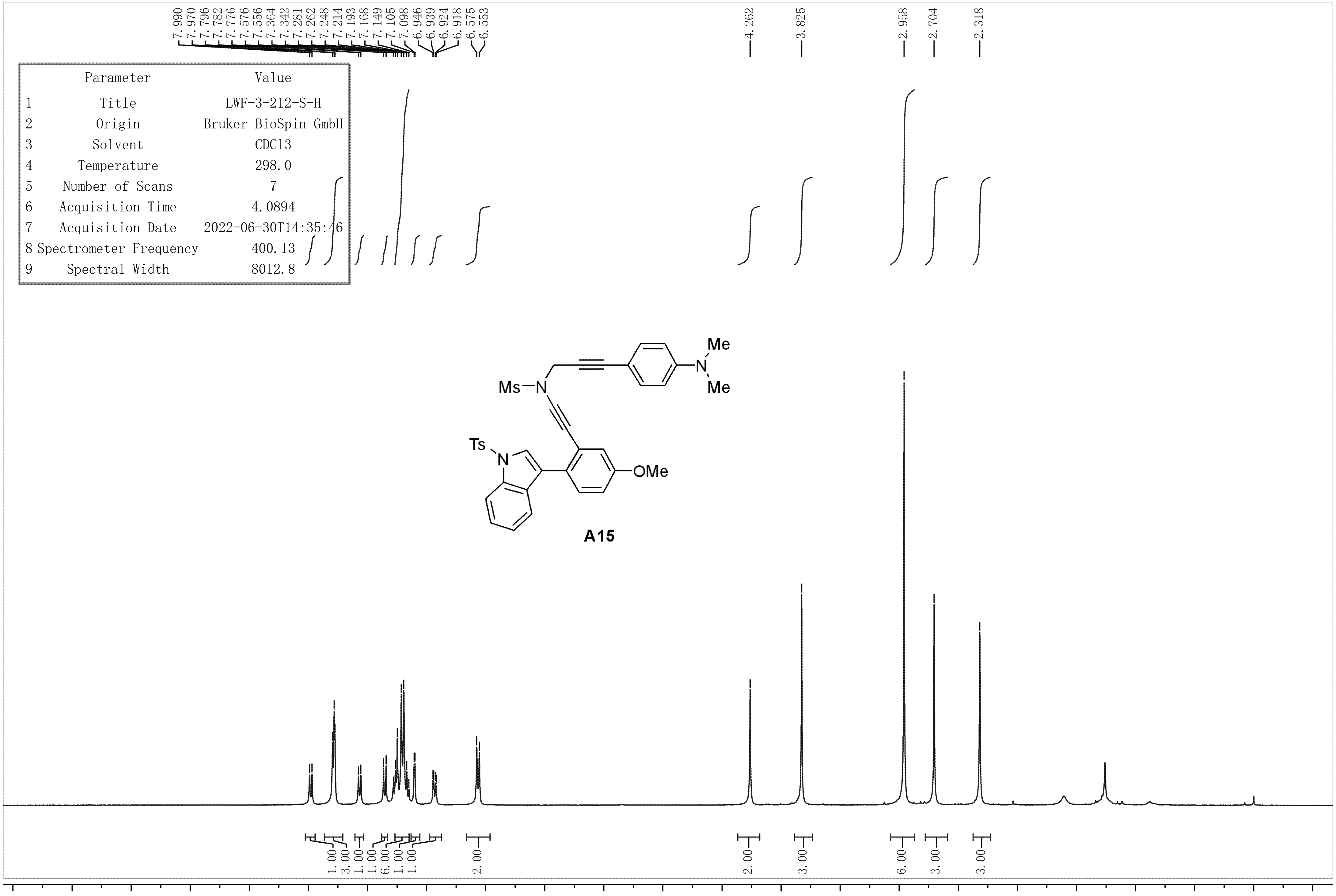
Parameter Value
1 Title LWF-3-212-S-H
2 Origin Bruker BioSpin GmbH
3 Solvent CDCl3
4 Temperature 298.0
5 Number of Scans 7
6 Acquisition Time 4.0894
7 Acquisition Date 2022-06-30T14:35:46
8 Spectrometer Frequency 400.13
9 Spectral Width 8012.8
7.990 7.970 7.796 7.782 7.776 7.576 7.556 7.364 7.342 7.281 7.262 7.248 7.214 7.193 7.168 7.149 7.105 7.098 6.946 6.939 6.924 6.918 6.575 6.553
4.262
3.825
2.958
2.704
2.318
Ms
N
Me
Me
Ts
OMe
A15
1.00 3.00 1.00 1.00 6.00 1.00 1.00 2.00
2.00 3.00 6.00 3.00 3.00
10.5 10.0 9.5 9.0 8.5 8.0 7.5 7.0 6.5 6.0 5.5 5.0 4.5 4.0 3.5 3.0 2.5 2.0 1.5 1.0 0.5 0.0 -0.5
f1 (ppm)

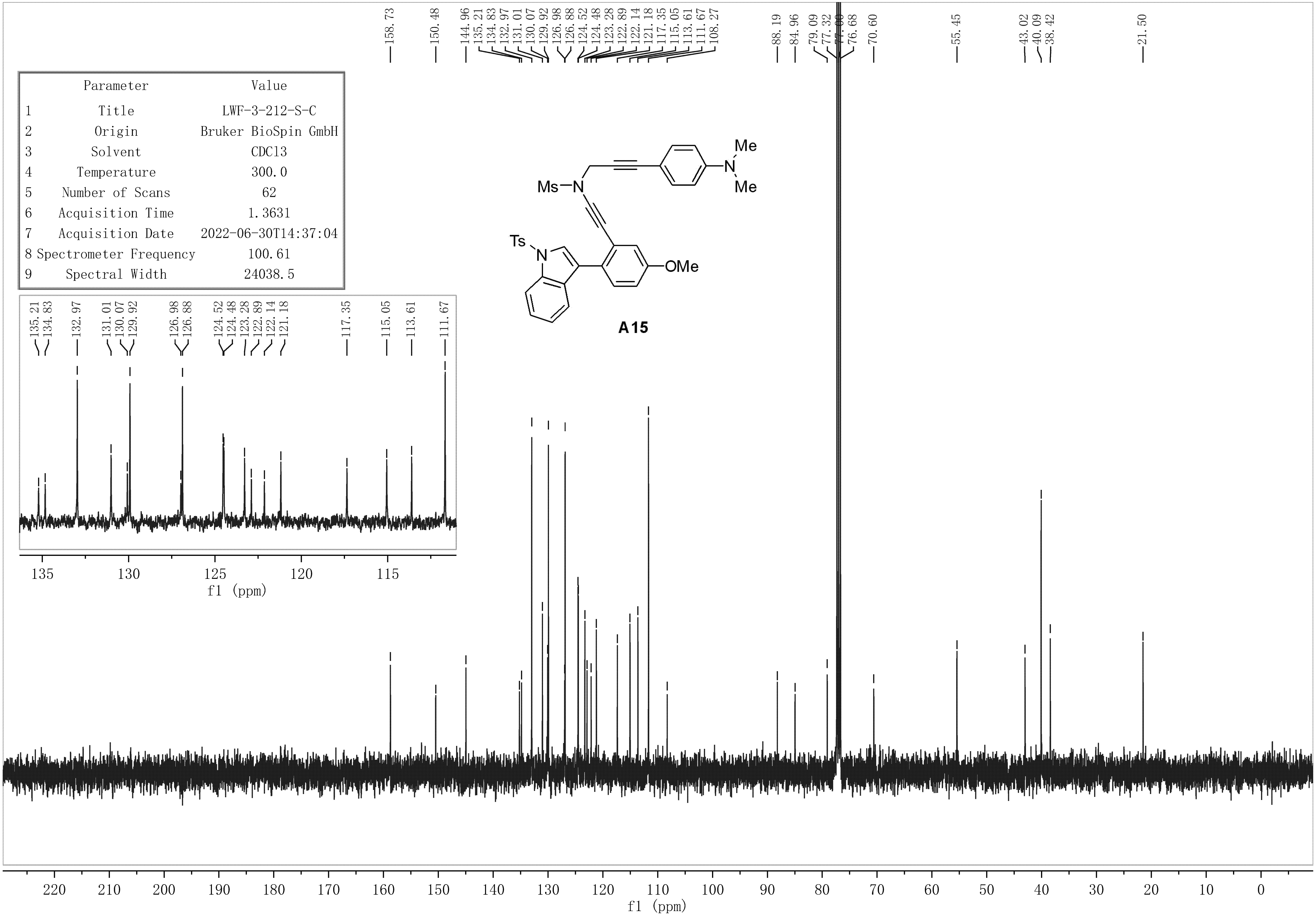

	Parameter	Value
1	Title	LWF-3-212-S-C
2	Origin	Bruker BioSpin GmbH
3	Solvent	CDCl3
4	Temperature	300.0
5	Number of Scans	62
6	Acquisition Time	1.3631
7	Acquisition Date	2022-06-30T14:37:04
8	Spectrometer Frequency	100.61
9	Spectral Width	24038.5

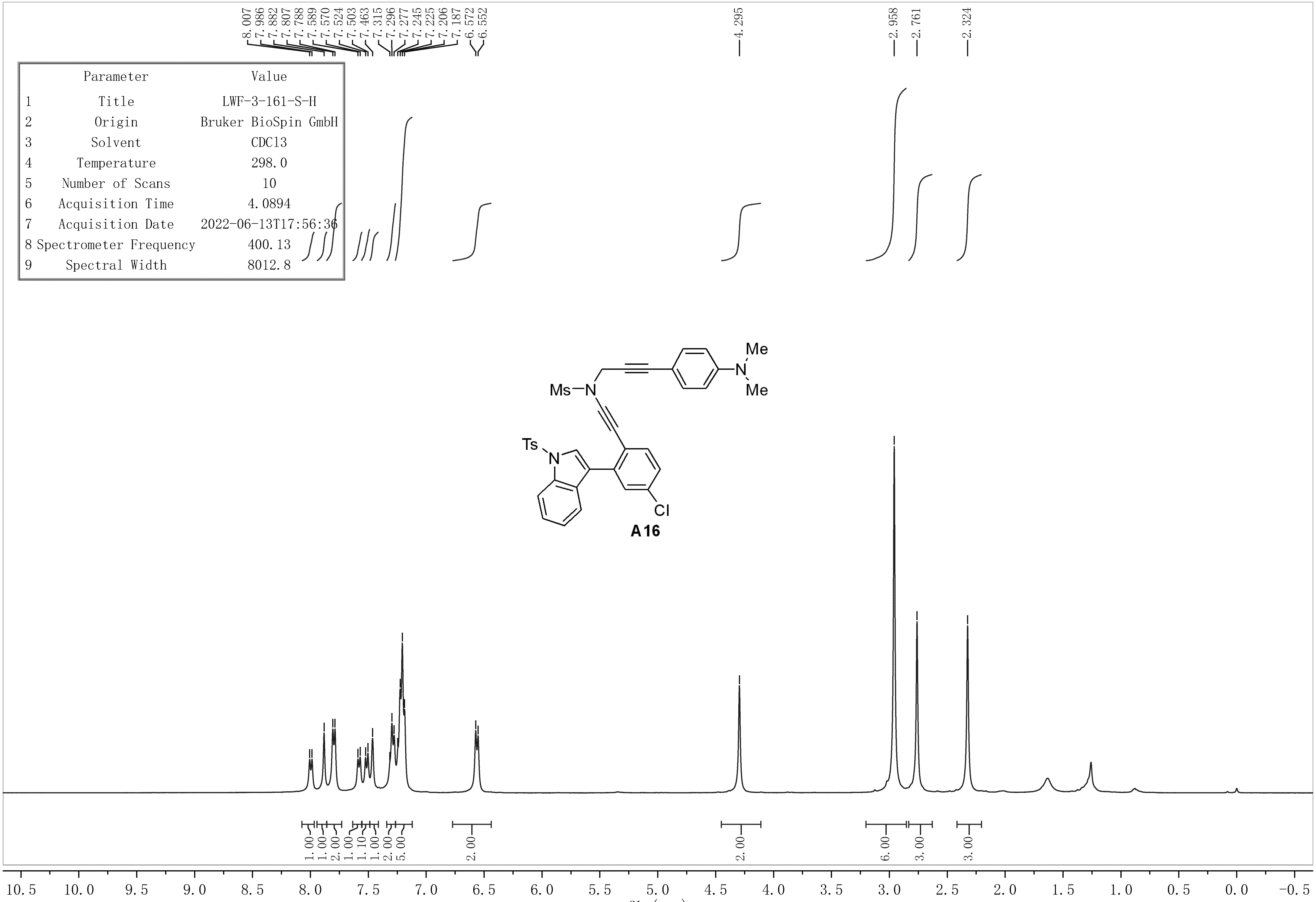

Parameter Value
1 Title LWF-3-161-S-H
2 Origin Bruker BioSpin GmbH
3 Solvent CDCl3
4 Temperature 298.0
5 Number of Scans 10
6 Acquisition Time 4.0894
7 Acquisition Date 2022-06-13T17:56:36
8 Spectrometer Frequency 400.13
9 Spectral Width 8012.8
8.007 7.986 7.882 7.807 7.788 7.589 7.570 7.524 7.503 7.463 7.315 7.296 7.277 7.245 7.225 7.206 7.187 6.572 6.552
4.295
2.958
2.761
2.324
1.00 1.00 2.00 1.00 1.10 1.00 2.00 5.00
2.00
2.00
6.00
3.00
3.00
Ms
N
Me
N
Me
Ts
N
Cl
f1 (ppm)

A16

	Parameter	Value
1	Title	LWF-3-161-S-C
2	Origin	Bruker BioSpin GmbH
3	Solvent	CDCl3
4	Temperature	300.0
5	Number of Scans	108
6	Acquisition Time	1.3631
7	Acquisition Date	2022-06-13T17:58:17
8	Spectrometer Frequency	100.61
9	Spectral Width	24038.5

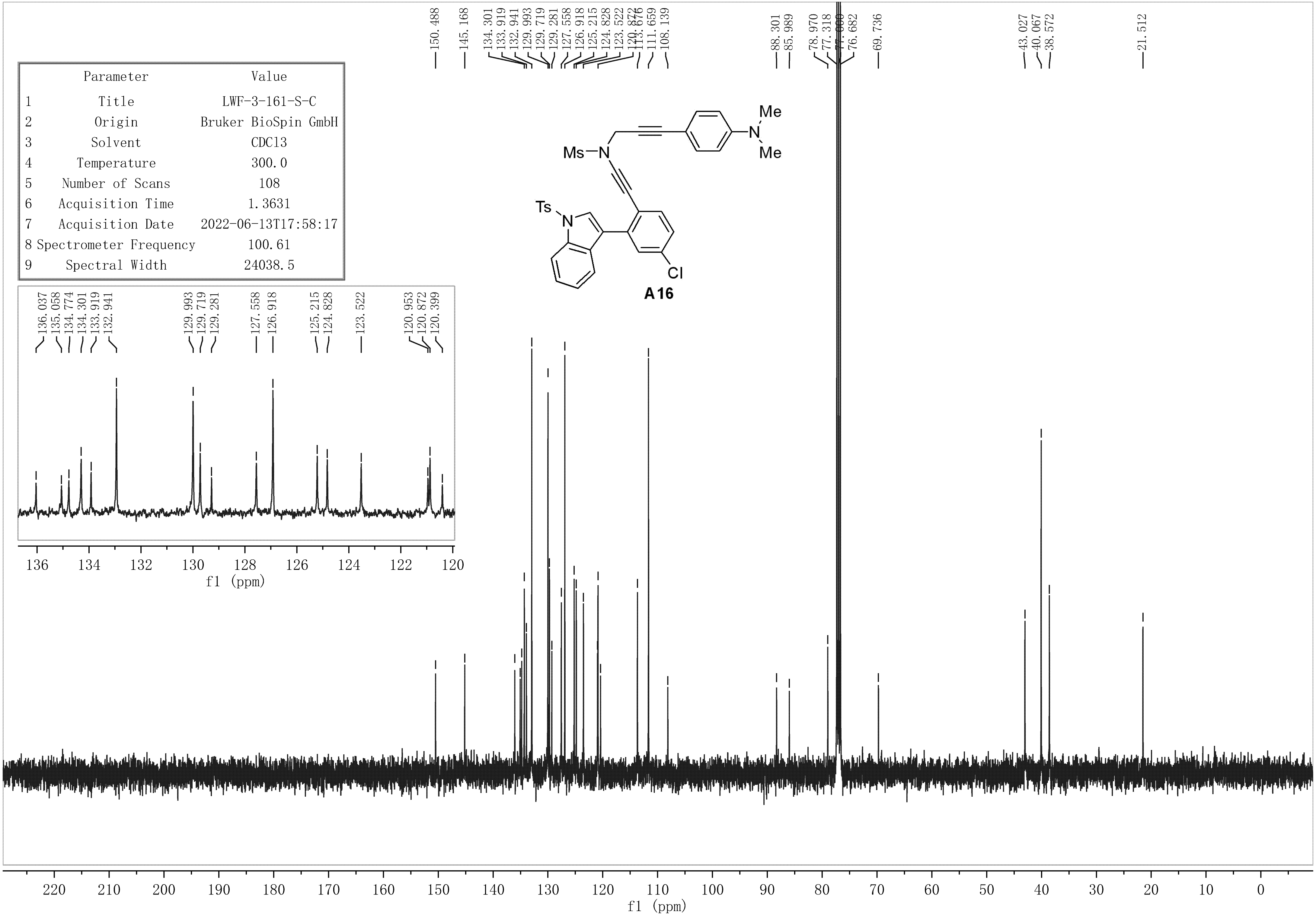

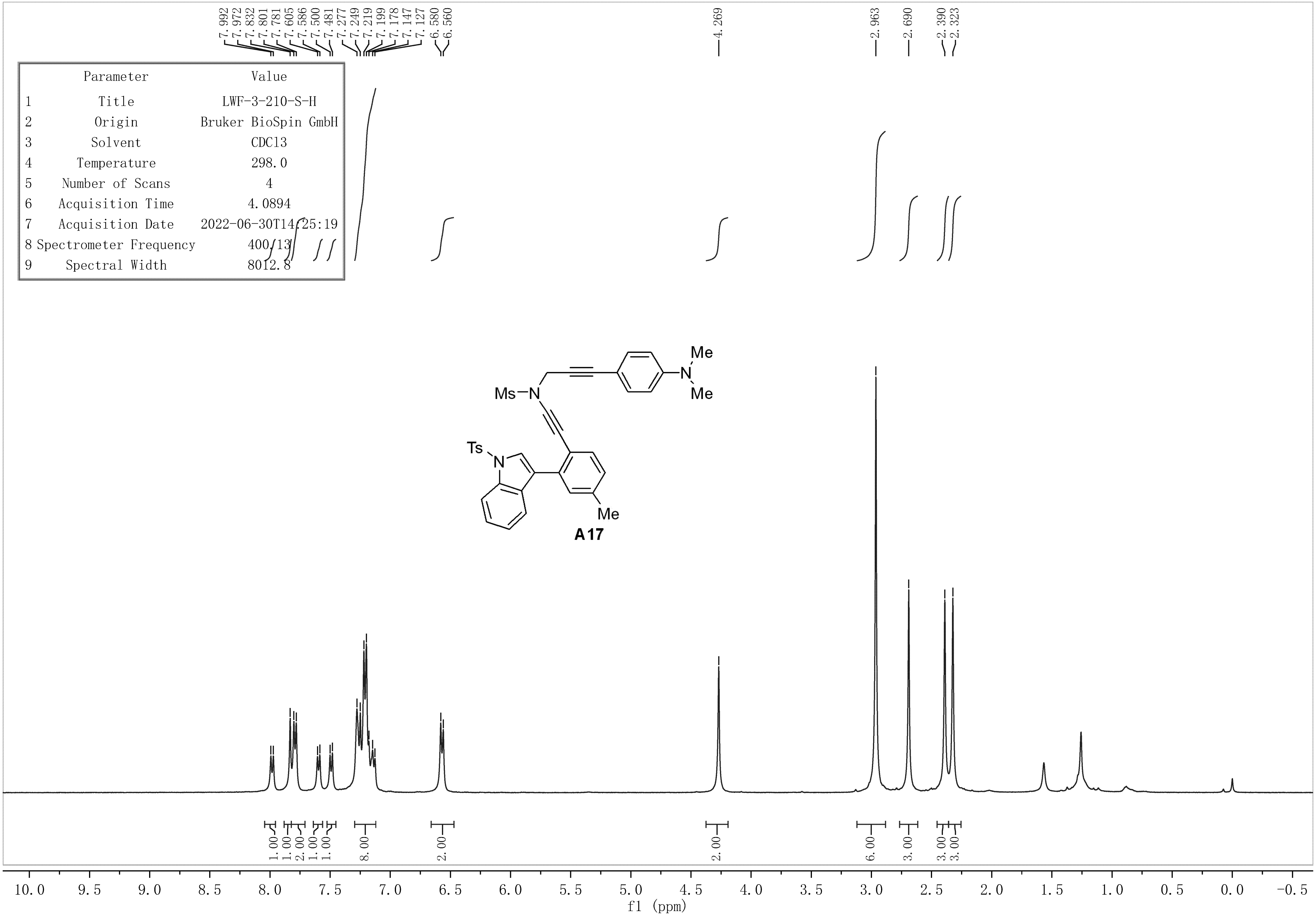

Parameter Value
1 Title LWF-3-210-S-H
2 Origin Bruker BioSpin GmbH
3 Solvent CDCl3
4 Temperature 298.0
5 Number of Scans 4
6 Acquisition Time 4.0894
7 Acquisition Date 2022-06-30T14:25:19
8 Spectrometer Frequency 400.13
9 Spectral Width 8012.8
7.992 7.972 7.832 7.801 7.781 7.605 7.586 7.500 7.481 7.277 7.249 7.219 7.199 7.178 7.147 7.127 6.580 6.560
4.269
2.963 2.690 2.390 2.323
Ms
N
Me
Ts
A17
1.00 1.00 2.00 1.00 1.00 8.00 2.00 2.00 6.00 3.00 3.00 3.00
10.0 9.5 9.0 8.5 8.0 7.5 7.0 6.5 6.0 5.5 5.0 4.5 4.0 3.5 3.0 2.5 2.0 1.5 1.0 0.5 0.0 -0.5
f1 (ppm)

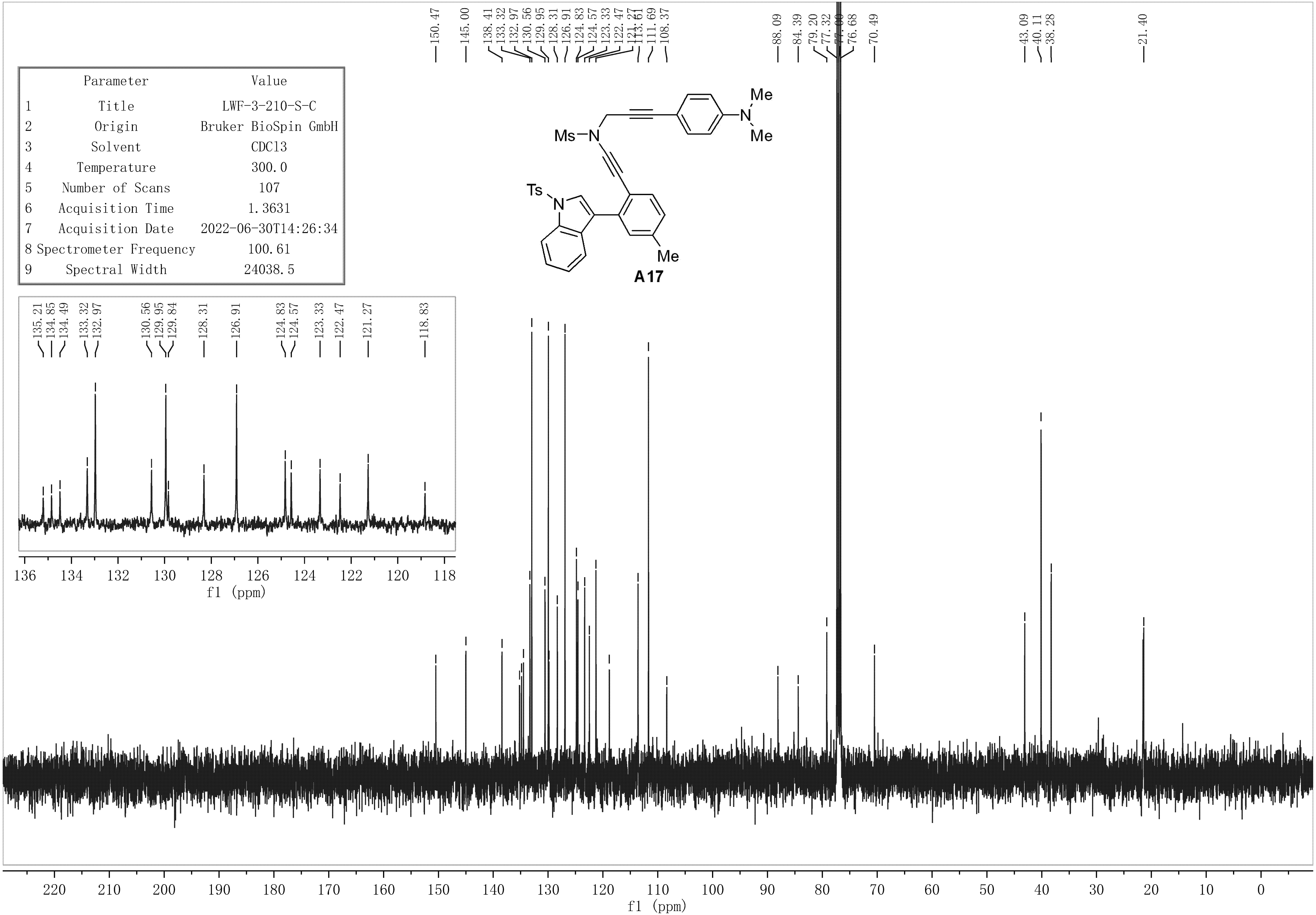

	Parameter	Value
1	Title	LWF-3-210-S-C
2	Origin	Bruker BioSpin GmbH
3	Solvent	CDCl3
4	Temperature	300.0
5	Number of Scans	107
6	Acquisition Time	1.3631
7	Acquisition Date	2022-06-30T14:26:34
8	Spectrometer Frequency	100.61
9	Spectral Width	24038.5

	Parameter	Value
1	Title	LWF-3-121-S-H
2	Origin	Bruker BioSpin GmbH
3	Solvent	CDCl3
4	Temperature	298.0
5	Number of Scans	11
6	Acquisition Time	4.0894
7	Acquisition Date	2022-05-25T15:58:11
8	Spectrometer Frequency	400.13
9	Spectral Width	8012.8

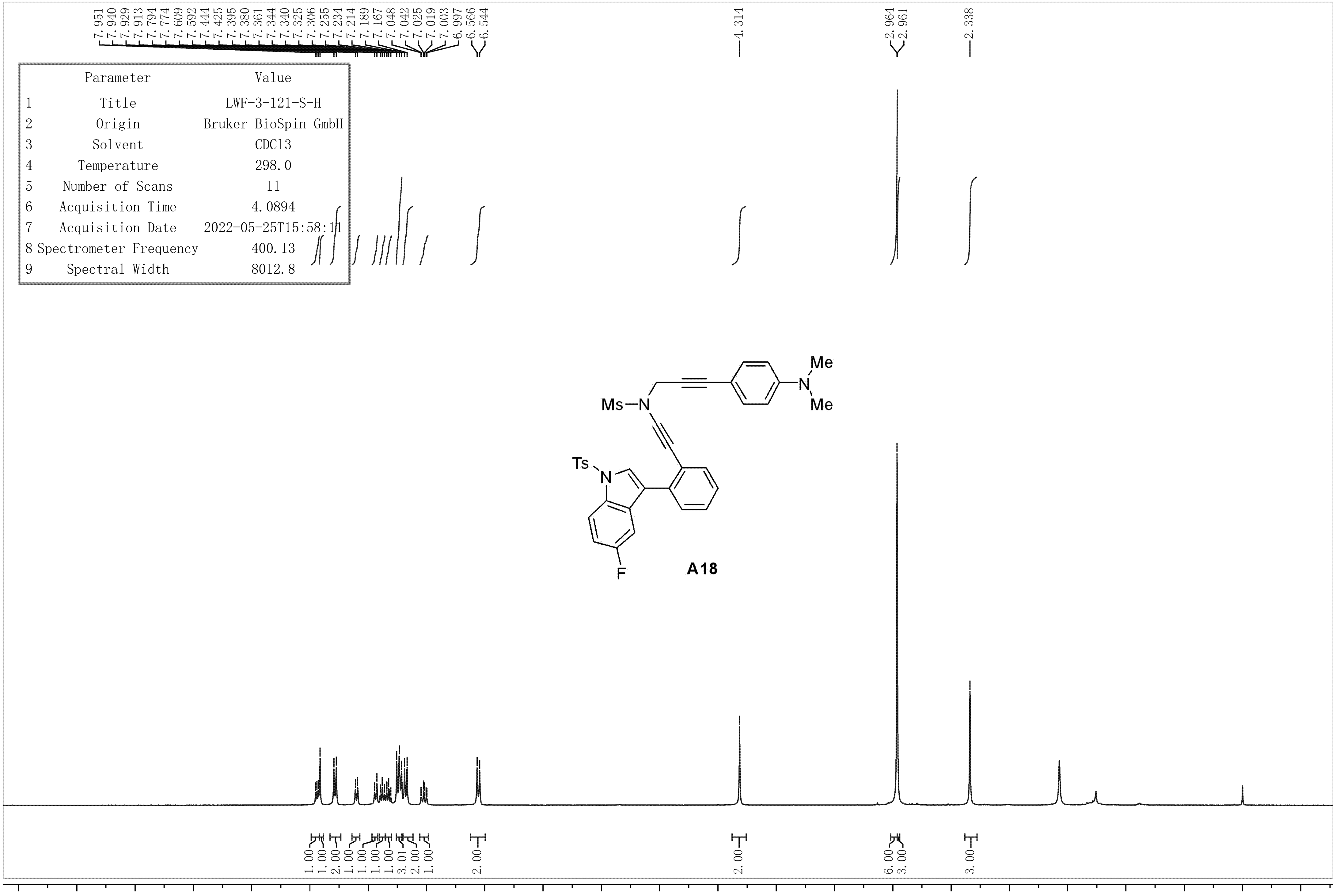

A18

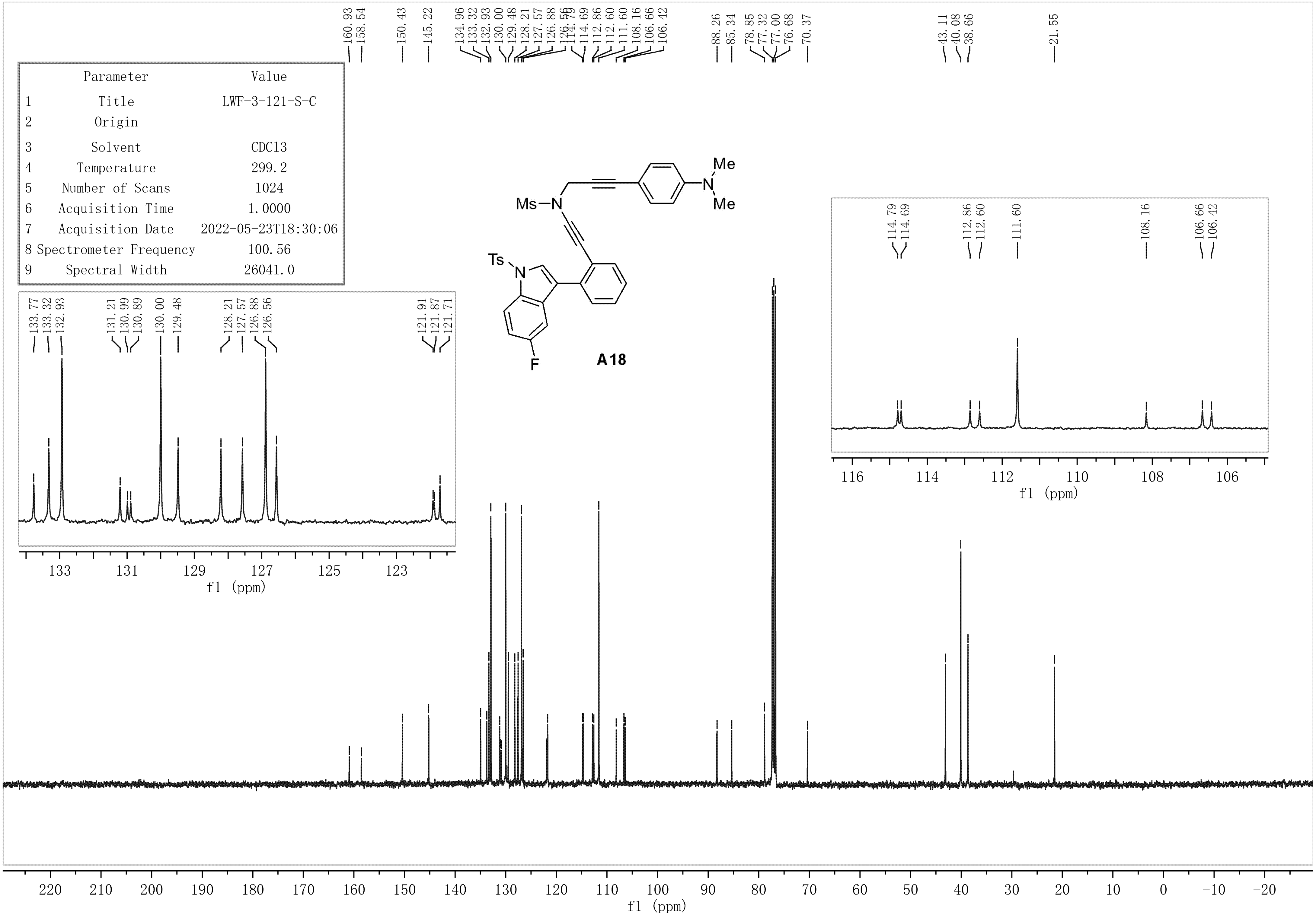

	Parameter	Value
1	Title	LWF-3-121-S-C
2	Origin	
3	Solvent	CDCl3
4	Temperature	299.2
5	Number of Scans	1024
6	Acquisition Time	1.0000
7	Acquisition Date	2022-05-23T18:30:06
8	Spectrometer Frequency	100.56
9	Spectral Width	26041.0

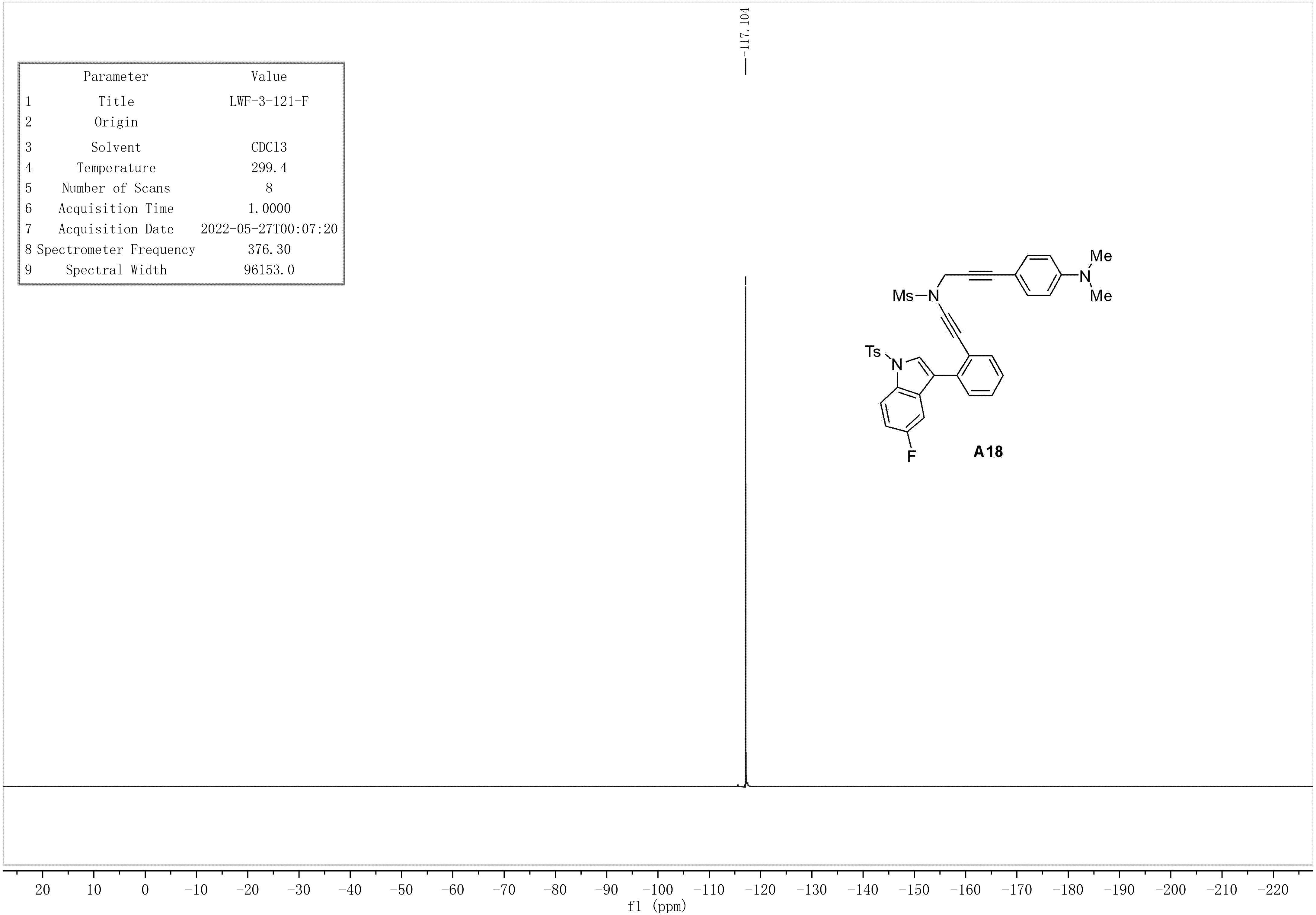

	Parameter	Value
1	Title	LWF-3-121-F
2	Origin	
3	Solvent	CDCl3
4	Temperature	299.4
5	Number of Scans	8
6	Acquisition Time	1.0000
7	Acquisition Date	2022-05-27T00:07:20
8	Spectrometer Frequency	376.30
9	Spectral Width	96153.0

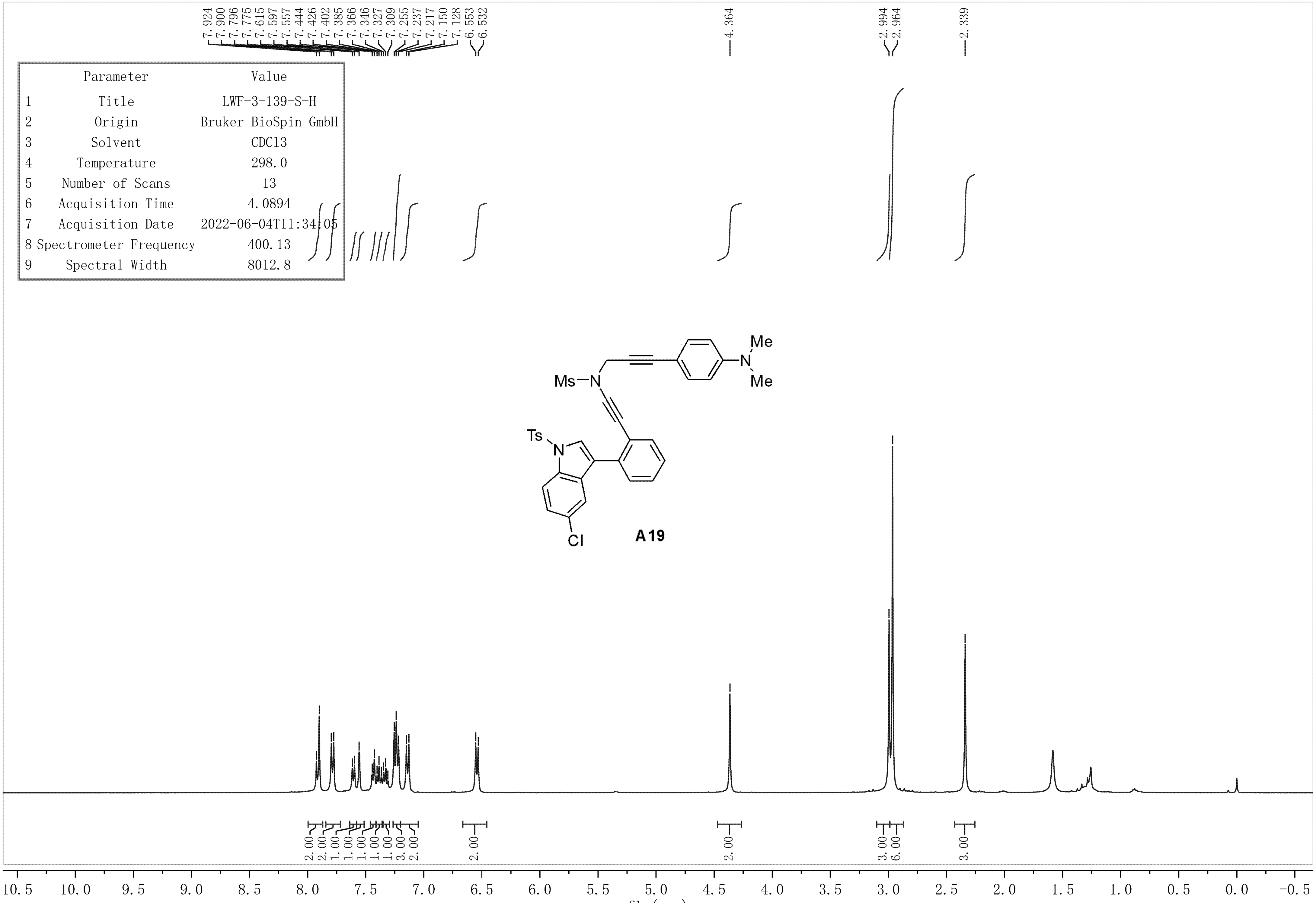
7.924
7.900
7.796
7.775
7.615
7.597
7.557
7.444
7.426
7.402
7.385
7.366
7.346
7.327
7.309
7.255
7.237
7.217
7.150
7.128
6.553
6.532
4.364
2.994
2.964
2.339
Parameter Value
1 Title LWF-3-139-S-H
2 Origin Bruker BioSpin GmbH
3 Solvent CDCl3
4 Temperature 298.0
5 Number of Scans 13
6 Acquisition Time 4.0894
7 Acquisition Date 2022-06-04T11:34:05
8 Spectrometer Frequency 400.13
9 Spectral Width 8012.8
Ms
N
Me
Ts
Cl
A19
2.00
2.00
1.00
1.00
1.00
1.00
3.00
2.00
2.00
2.00
3.00
6.00
3.00
10.5 10.0 9.5 9.0 8.5 8.0 7.5 7.0 6.5 6.0 5.5 5.0 4.5 4.0 3.5 3.0 2.5 2.0 1.5 1.0 0.5 0.0 -0.5
f1 (ppm)

	Parameter	Value
1	Title	LWF-3-139-S-C
2	Origin	Bruker BioSpin GmbH
3	Solvent	CDCl3
4	Temperature	300.0
5	Number of Scans	124
6	Acquisition Time	1.3631
7	Acquisition Date	2022-06-03T14:37:13
8	Spectrometer Frequency	100.61
9	Spectral Width	24038.5

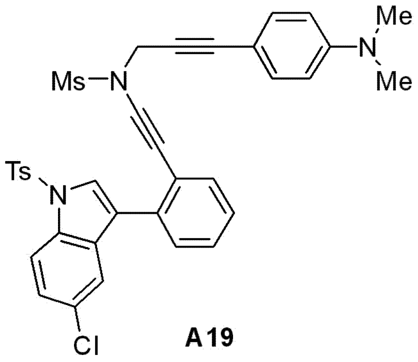

A19

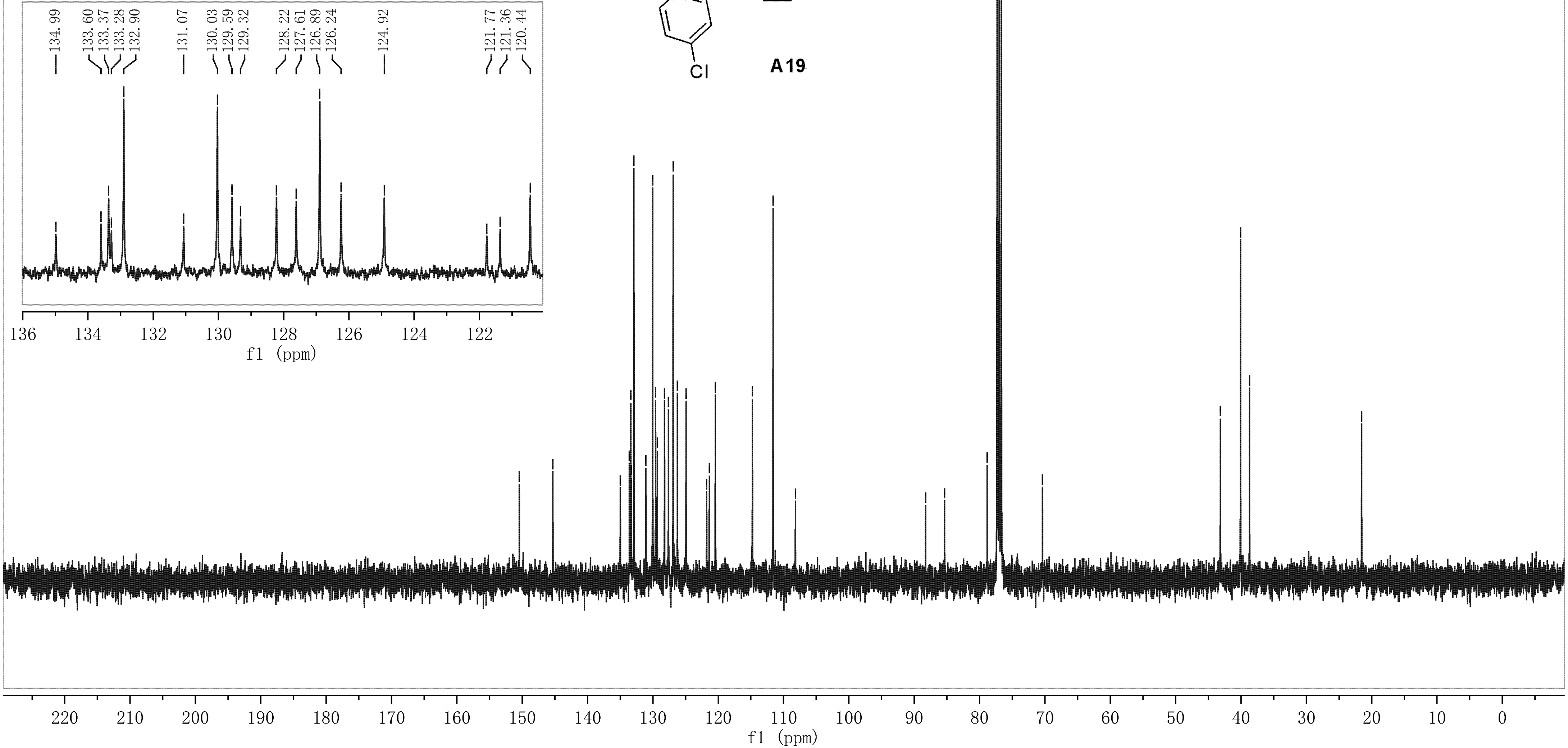

	Parameter	Value
1	Title	LWF-3-52-S-H
2	Origin	Bruker BioSpin GmbH
3	Solvent	CDCl3
4	Temperature	298.0
5	Number of Scans	7
6	Acquisition Time	4.0894
7	Acquisition Date	2022-05-03T10:32:41
8	Spectrometer Frequency	400.13
9	Spectral Width	8012.8

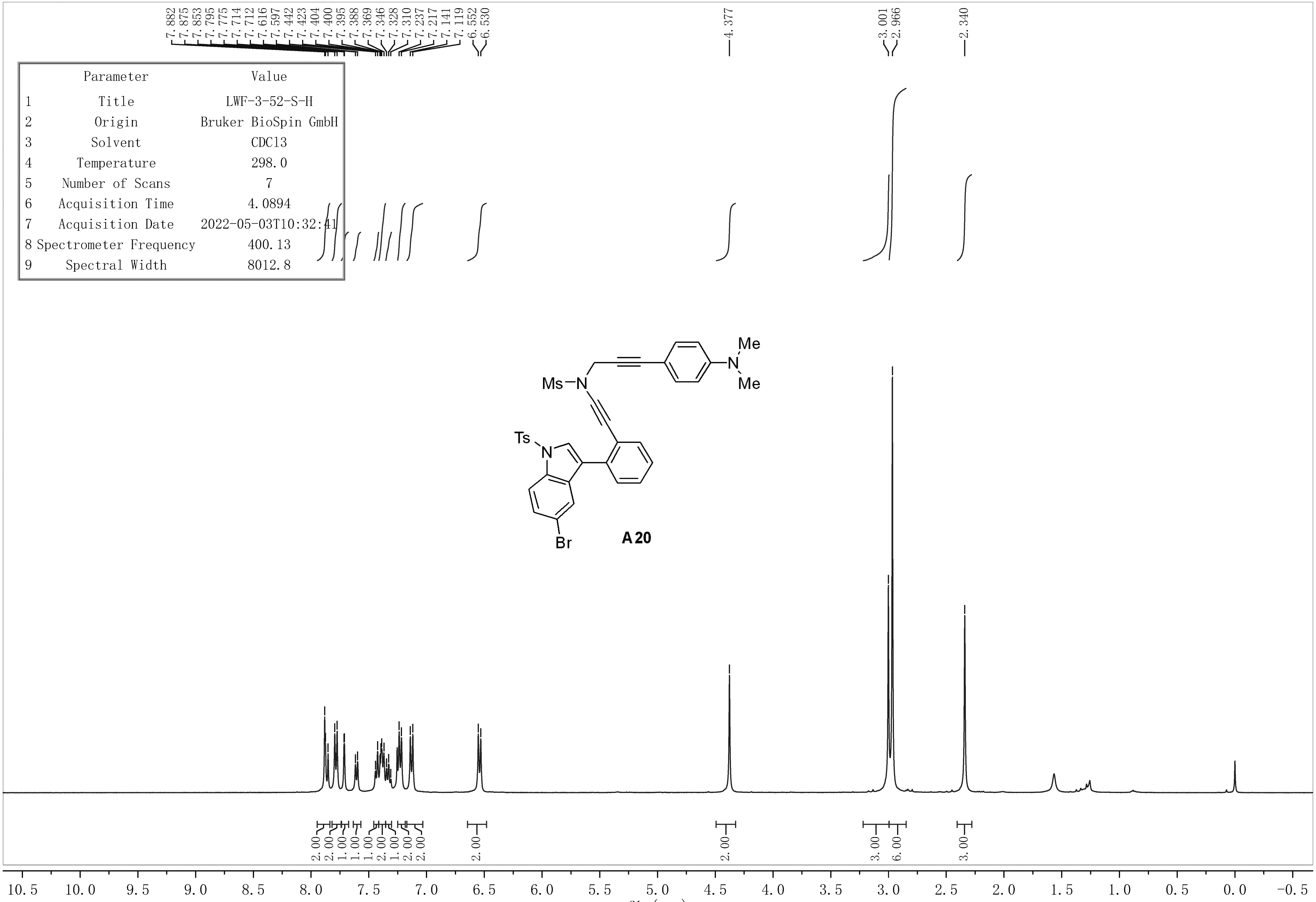

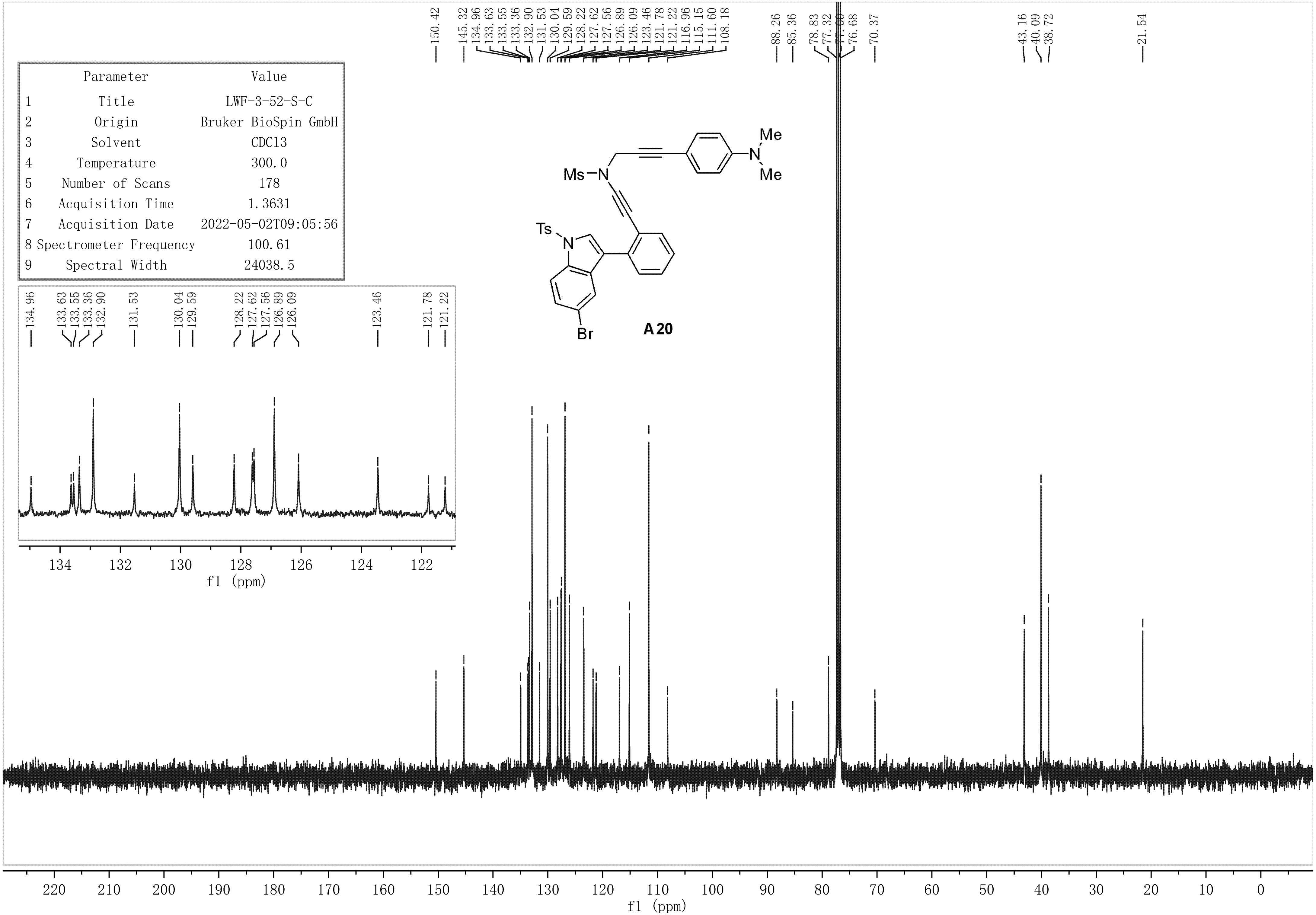

	Parameter	Value
1	Title	LWF-3-52-S-C
2	Origin	Bruker BioSpin GmbH
3	Solvent	CDCl3
4	Temperature	300.0
5	Number of Scans	178
6	Acquisition Time	1.3631
7	Acquisition Date	2022-05-02T09:05:56
8	Spectrometer Frequency	100.61
9	Spectral Width	24038.5

A 20

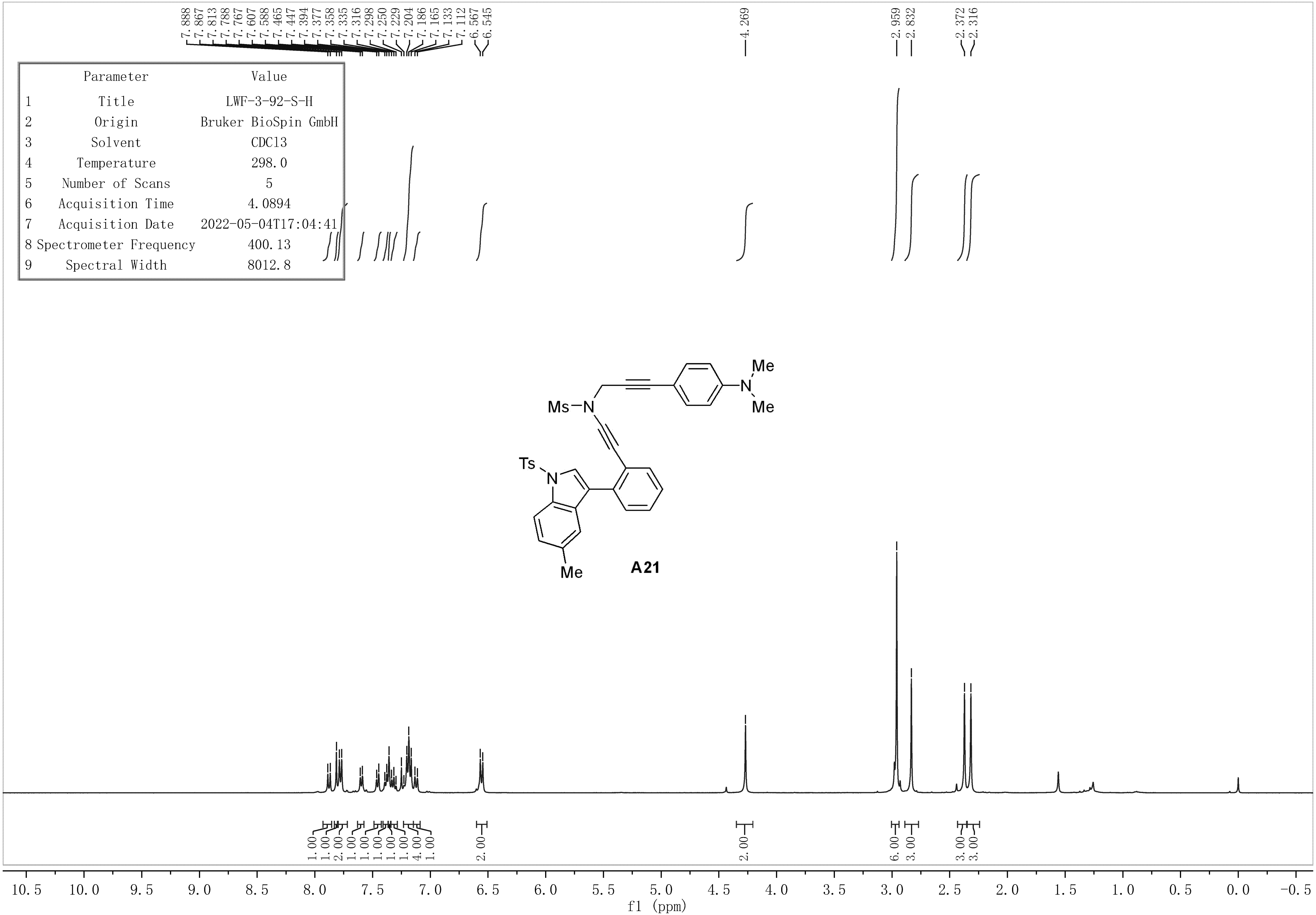
7.888 7.867 7.813 7.788 7.767 7.607 7.588 7.465 7.447 7.394 7.377 7.358 7.335 7.316 7.298 7.250 7.229 7.204 7.186 7.165 7.133 7.112 6.567 6.545
4.269
2.959 2.832
2.372 2.316
Parameter Value
1 Title LWF-3-92-S-H
2 Origin Bruker BioSpin GmbH
3 Solvent CDCl3
4 Temperature 298.0
5 Number of Scans 5
6 Acquisition Time 4.0894
7 Acquisition Date 2022-05-04T17:04:41
8 Spectrometer Frequency 400.13
9 Spectral Width 8012.8
Me
Ms−N
N
Me
Ts
N
Me
A21
1.00 1.00 2.00 1.00 1.00 1.00 1.00 4.00 1.00
2.00
2.00
6.00 3.00
3.00 3.00
10.5 10.0 9.5 9.0 8.5 8.0 7.5 7.0 6.5 6.0 5.5 5.0 4.5 4.0 3.5 3.0 2.5 2.0 1.5 1.0 0.5 0.0 −0.5
f1 (ppm)

	Parameter	Value
1	Title	LWF-3-92-S-C
2	Origin	
3	Solvent	CDCl3
4	Temperature	298.3
5	Number of Scans	600
6	Acquisition Time	1.0000
7	Acquisition Date	2022-05-04T17:39:25
8	Spectrometer Frequency	100.56
9	Spectral Width	26041.0

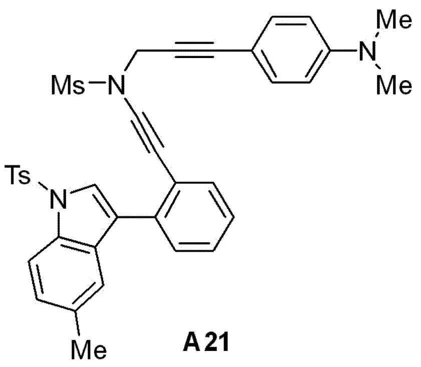

A21

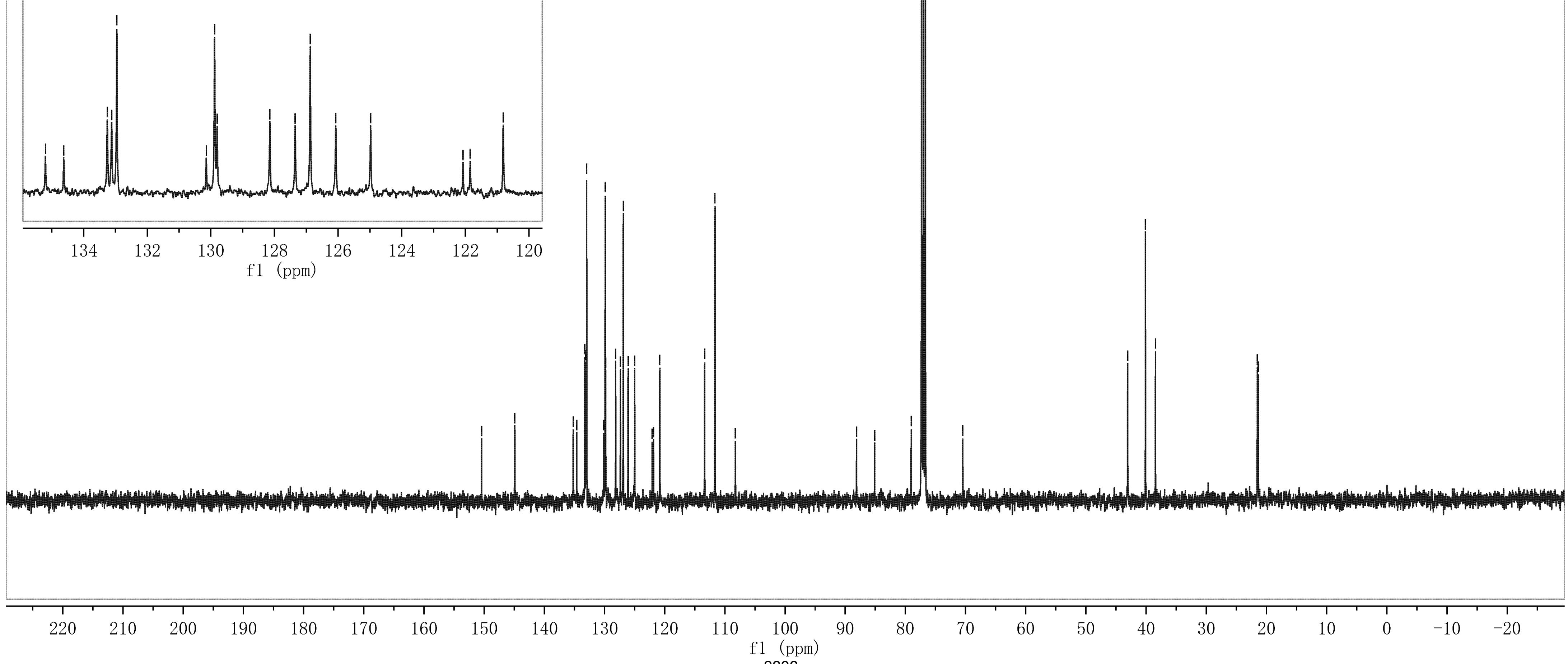

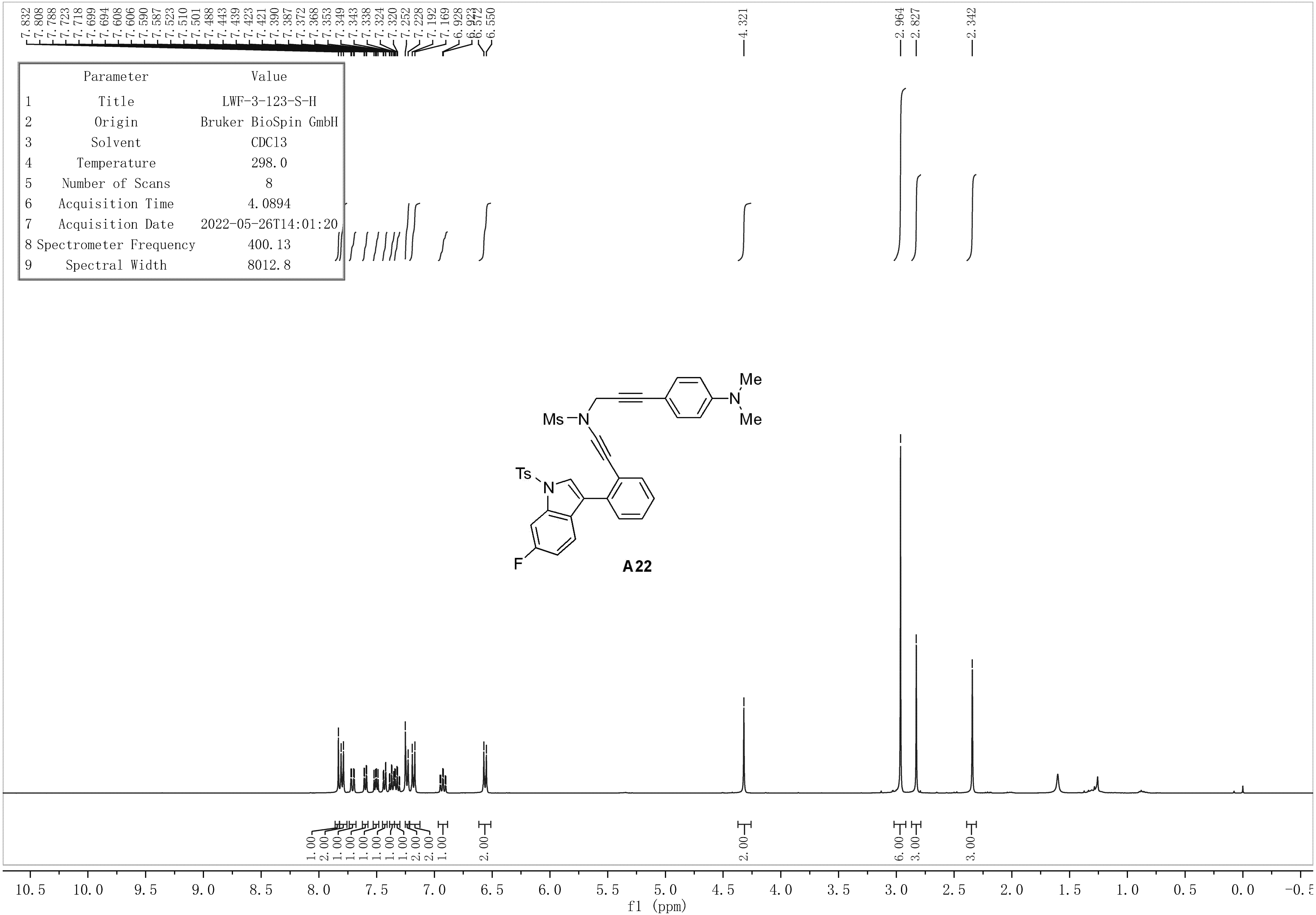
Parameter Value
1 Title LWF-3-123-S-H
2 Origin Bruker BioSpin GmbH
3 Solvent CDCl3
4 Temperature 298.0
5 Number of Scans 8
6 Acquisition Time 4.0894
7 Acquisition Date 2022-05-26T14:01:20
8 Spectrometer Frequency 400.13
9 Spectral Width 8012.8
7.832 7.808 7.788 7.723 7.718 7.699 7.694 7.608 7.606 7.590 7.587 7.523 7.510 7.501 7.488 7.443 7.439 7.423 7.421 7.390 7.387 7.372 7.368 7.353 7.349 7.343 7.338 7.324 7.320 7.252 7.228 7.192 7.169 6.928 6.923 6.572 6.550
4.321
2.964 2.827
2.342
Ms
N
Me
Ts
F
A22
1.00 2.00 1.00 1.00 1.00 1.00 1.00 1.00 2.00 2.00 1.00
2.00
2.00
6.00 3.00
3.00
10.5 10.0 9.5 9.0 8.5 8.0 7.5 7.0 6.5 6.0 5.5 5.0 4.5 4.0 3.5 3.0 2.5 2.0 1.5 1.0 0.5 0.0 -0.5
f1 (ppm)

	Parameter	Value
1	Title	LWF-3-123-S-C
2	Origin	
3	Solvent	CDCl3
4	Temperature	299.1
5	Number of Scans	2000
6	Acquisition Time	1.0000
7	Acquisition Date	2022-05-27T01:17:32
8	Spectrometer Frequency	100.56
9	Spectral Width	26041.0

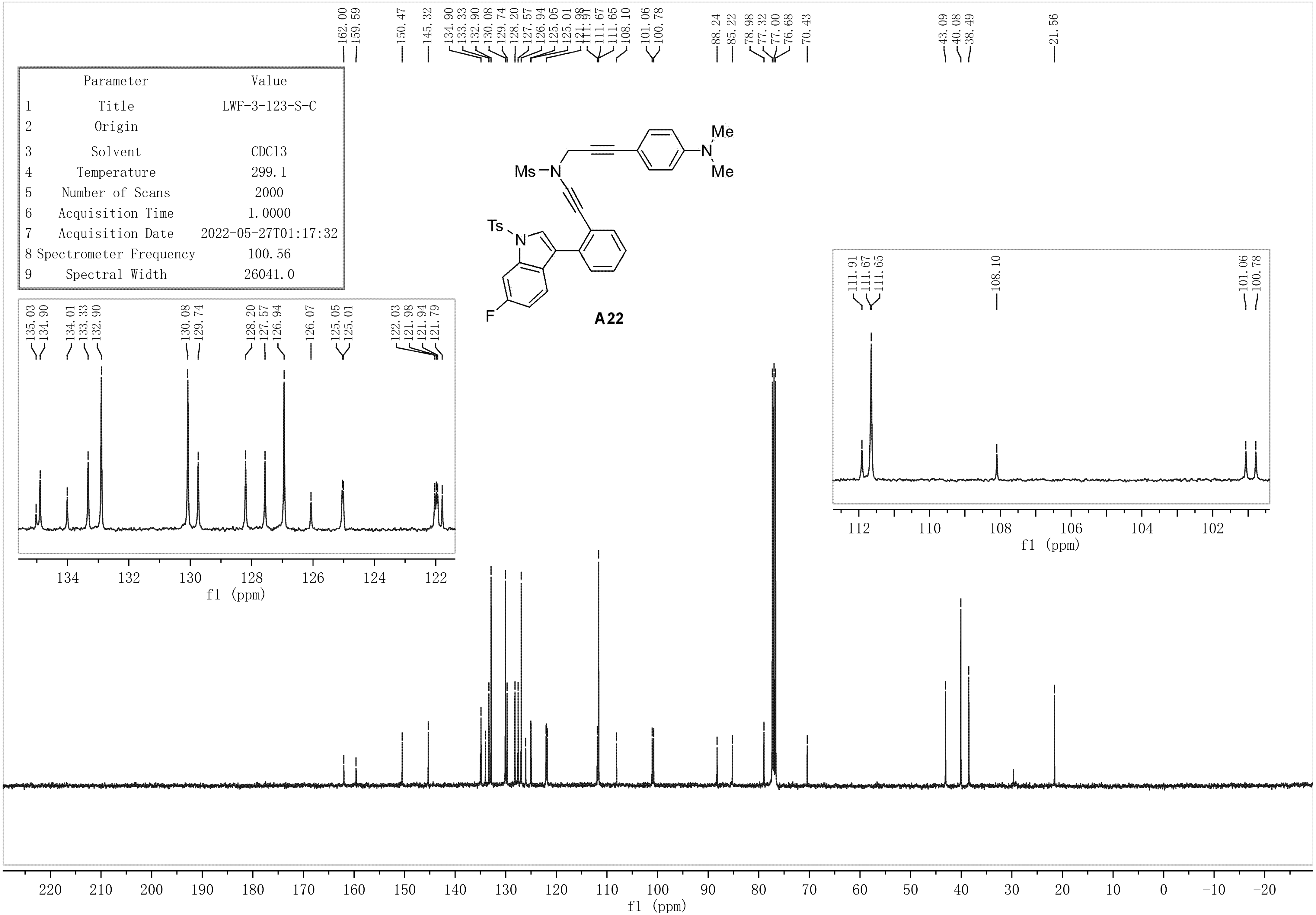

	Parameter	Value
1	Title	LWF-3-123-S-F
2	Origin	
3	Solvent	CDCl3
4	Temperature	299.2
5	Number of Scans	8
6	Acquisition Time	1.0000
7	Acquisition Date	2022-05-27T01:19:52
8	Spectrometer Frequency	376.30
9	Spectral Width	96153.0

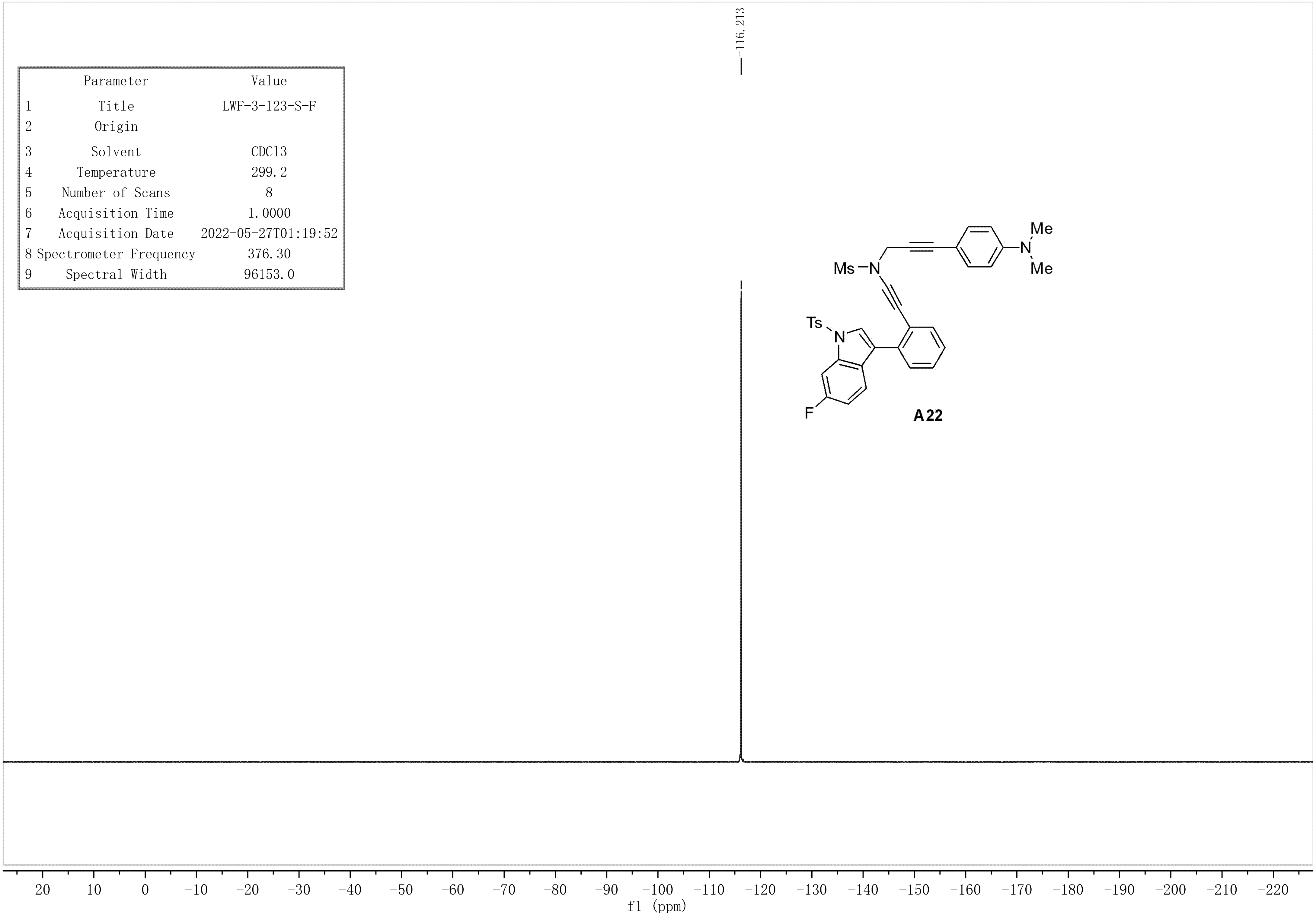

A22

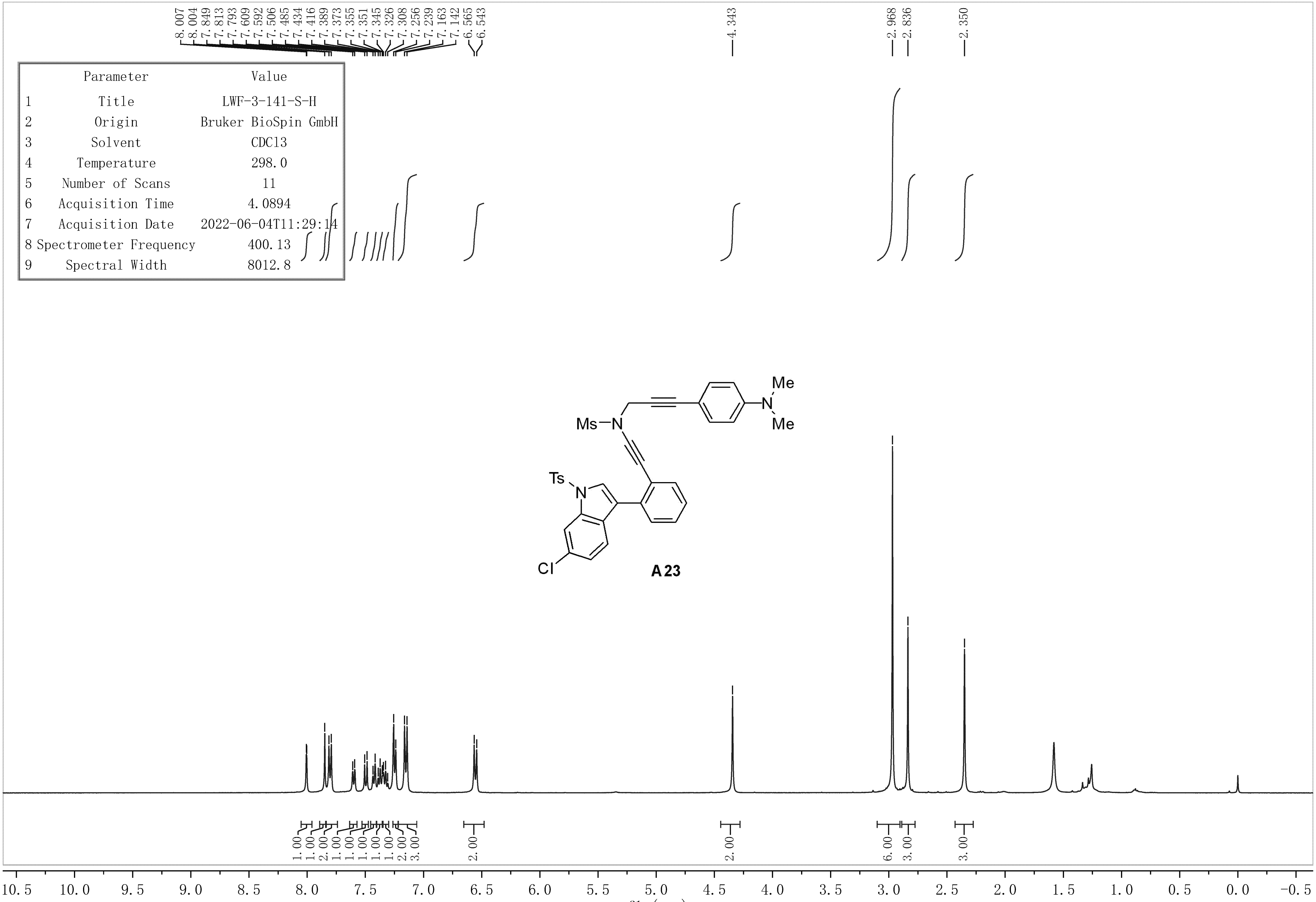
8.007
8.004
7.849
7.813
7.793
7.609
7.592
7.506
7.485
7.434
7.416
7.389
7.373
7.355
7.351
7.345
7.326
7.308
7.256
7.239
7.163
7.142
6.565
6.543
4.343
2.968
2.836
2.350
Parameter Value
1 Title LWF-3-141-S-H
2 Origin Bruker BioSpin GmbH
3 Solvent CDCl3
4 Temperature 298.0
5 Number of Scans 11
6 Acquisition Time 4.0894
7 Acquisition Date 2022-06-04T11:29:14
8 Spectrometer Frequency 400.13
9 Spectral Width 8012.8
Me
N
Me
Ms
N
Ts
N
Cl
A23
1.00
1.00
2.00
1.00
1.00
1.00
1.00
1.00
2.00
3.00
2.00
2.00
6.00
3.00
3.00
10.5 10.0 9.5 9.0 8.5 8.0 7.5 7.0 6.5 6.0 5.5 5.0 4.5 4.0 3.5 3.0 2.5 2.0 1.5 1.0 0.5 0.0 -0.5
f1 (ppm)

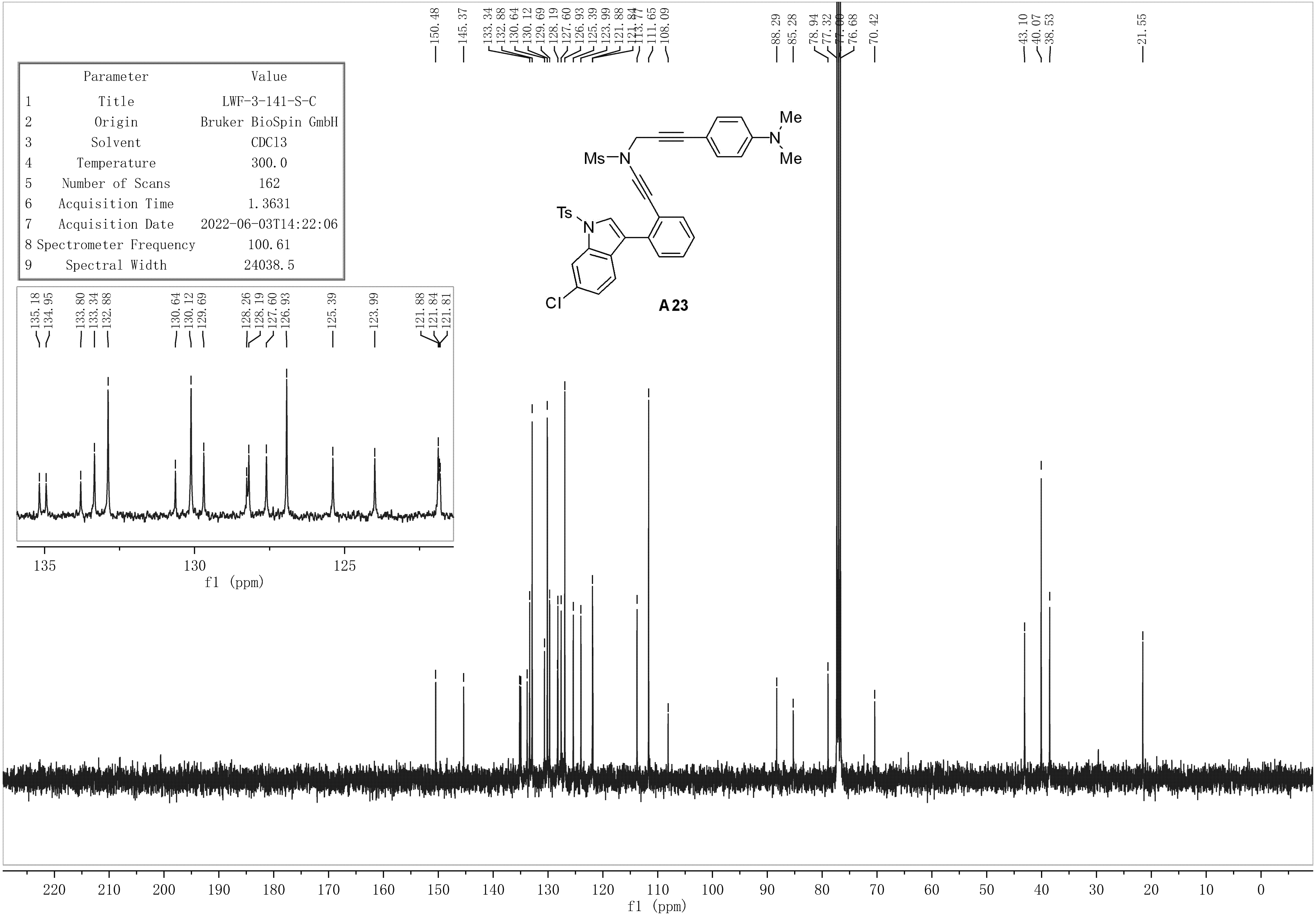

Parameter | Value
1 Title LWF-3-141-S-C
2 Origin Bruker BioSpin GmbH
3 Solvent CDCl3
4 Temperature 300.0
5 Number of Scans 162
6 Acquisition Time 1.3631
7 Acquisition Date 2022-06-03T14:22:06
8 Spectrometer Frequency 100.61
9 Spectral Width 24038.5
150.48
145.37
133.34
132.88
130.64
130.12
129.69
128.19
127.60
126.93
125.39
123.99
121.88
111.65
108.09
88.29
85.28
78.94
77.32
76.68
70.42
43.10
40.07
38.53
21.55
135.18
134.95
133.80
133.34
132.88
130.64
130.12
129.69
128.26
128.19
127.60
126.93
125.39
123.99
121.88
121.84
121.81
Me
Ms
N
Me
Ts
N
Cl
A 23
f1 (ppm)

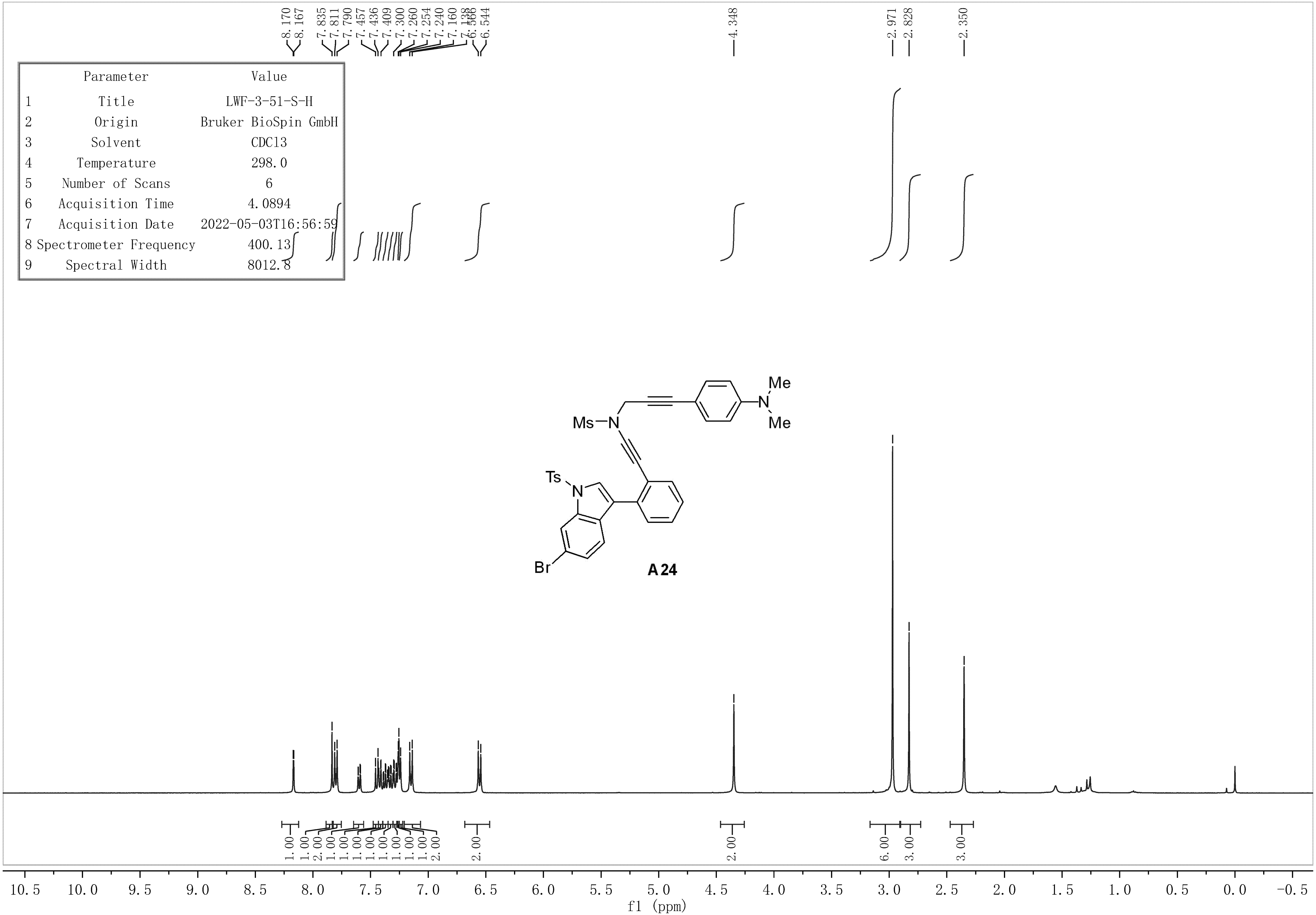

	Parameter	Value
1	Title	LWF-3-51-S-H
2	Origin	Bruker BioSpin GmbH
3	Solvent	CDCl3
4	Temperature	298.0
5	Number of Scans	6
6	Acquisition Time	4.0894
7	Acquisition Date	2022-05-03T16:56:59
8	Spectrometer Frequency	400.13
9	Spectral Width	8012.8

A24

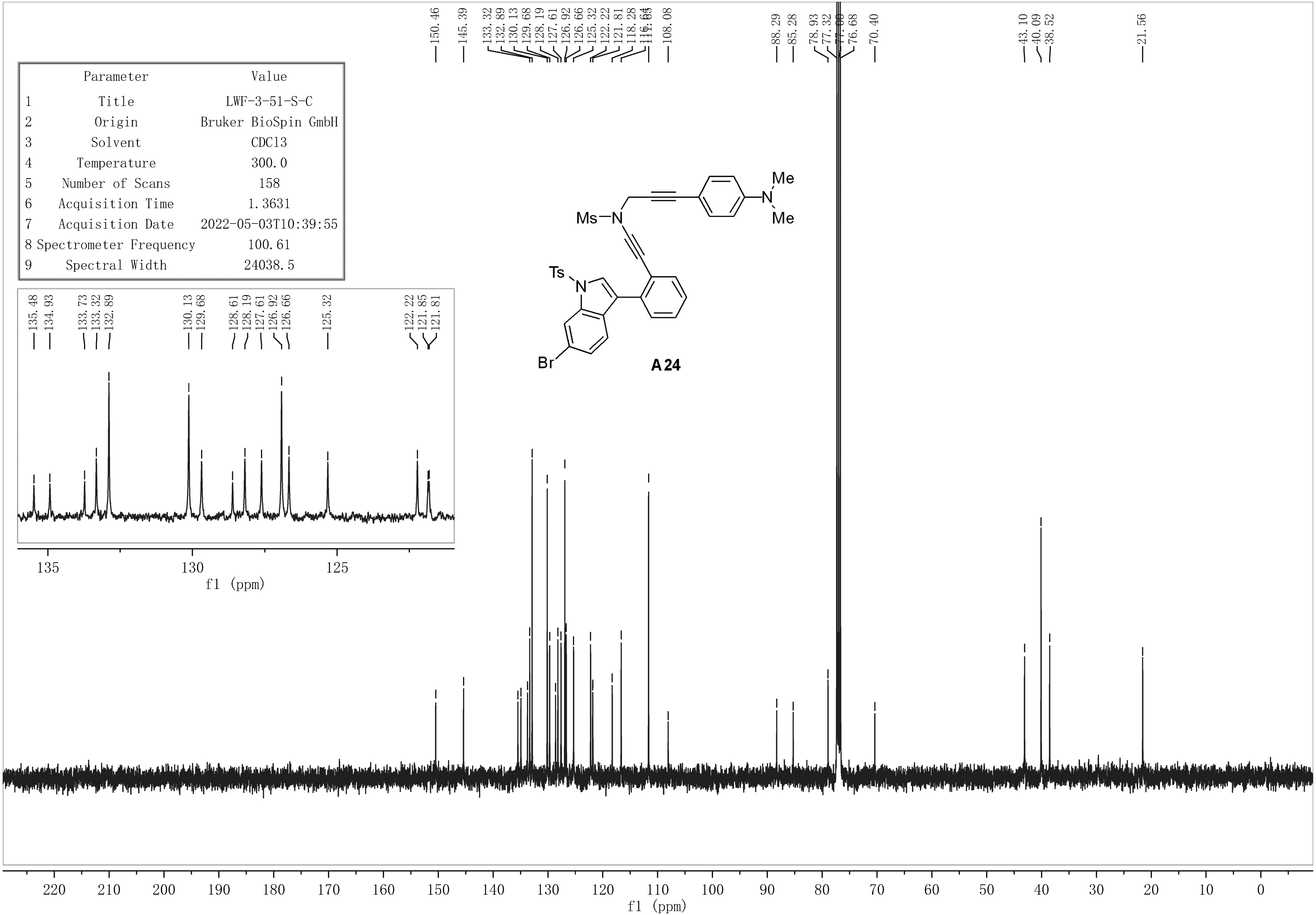

	Parameter	Value
1	Title	LWF-3-51-S-C
2	Origin	Bruker BioSpin GmbH
3	Solvent	CDCl3
4	Temperature	300.0
5	Number of Scans	158
6	Acquisition Time	1.3631
7	Acquisition Date	2022-05-03T10:39:55
8	Spectrometer Frequency	100.61
9	Spectral Width	24038.5

Parameter		Value
1	Title	LWF-3-94-S-H
2	Origin	Bruker BioSpin GmbH
3	Solvent	CDCl3
4	Temperature	298.0
5	Number of Scans	8
6	Acquisition Time	4.0894
7	Acquisition Date	2022-05-04T17:09:01
8	Spectrometer Frequency	400.13
9	Spectral Width	8012.8

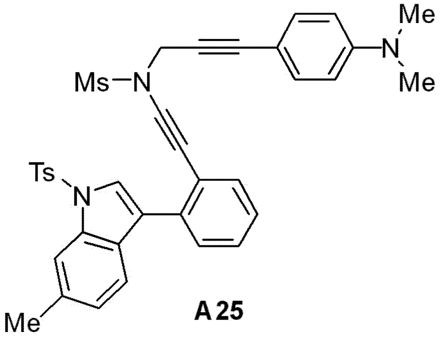

A25

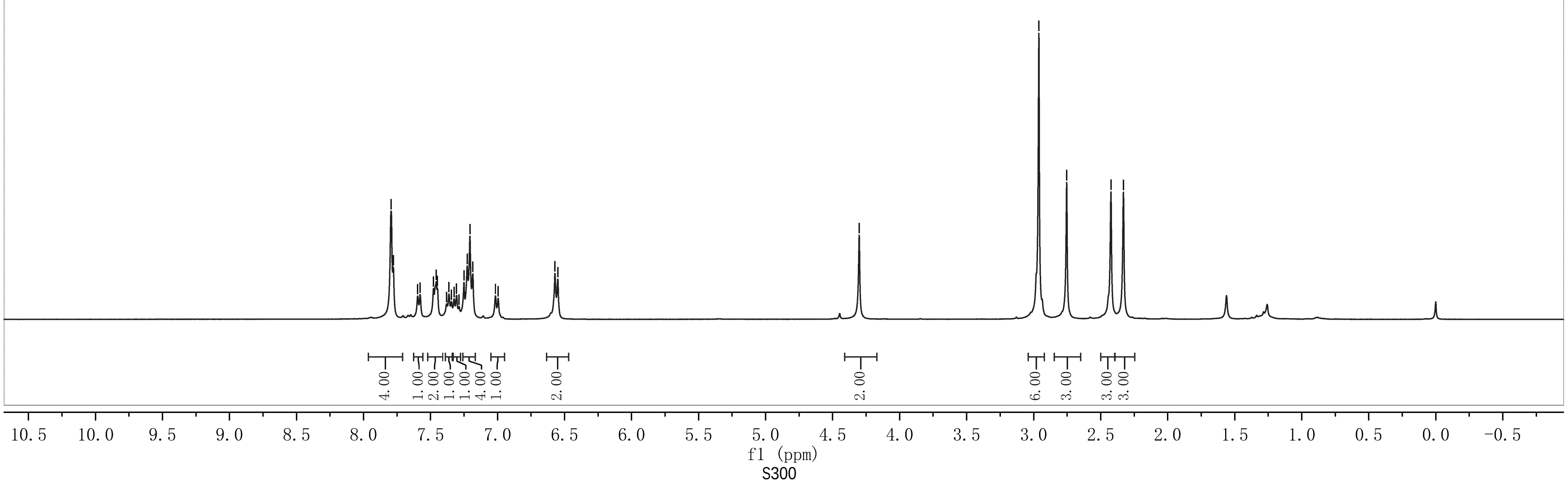

	Parameter	Value
1	Title	LWF-3-94-S-C
2	Origin	
3	Solvent	CDCl3
4	Temperature	298.6
5	Number of Scans	700
6	Acquisition Time	1.0000
7	Acquisition Date	2022-05-04T18:06:08
8	Spectrometer Frequency	100.56
9	Spectral Width	26041.0

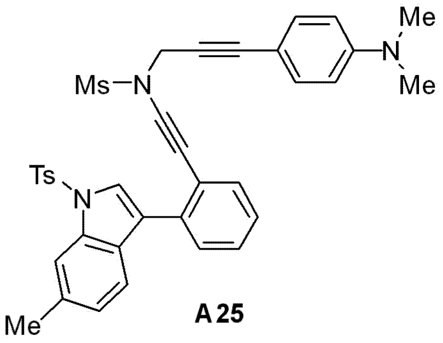

A 25

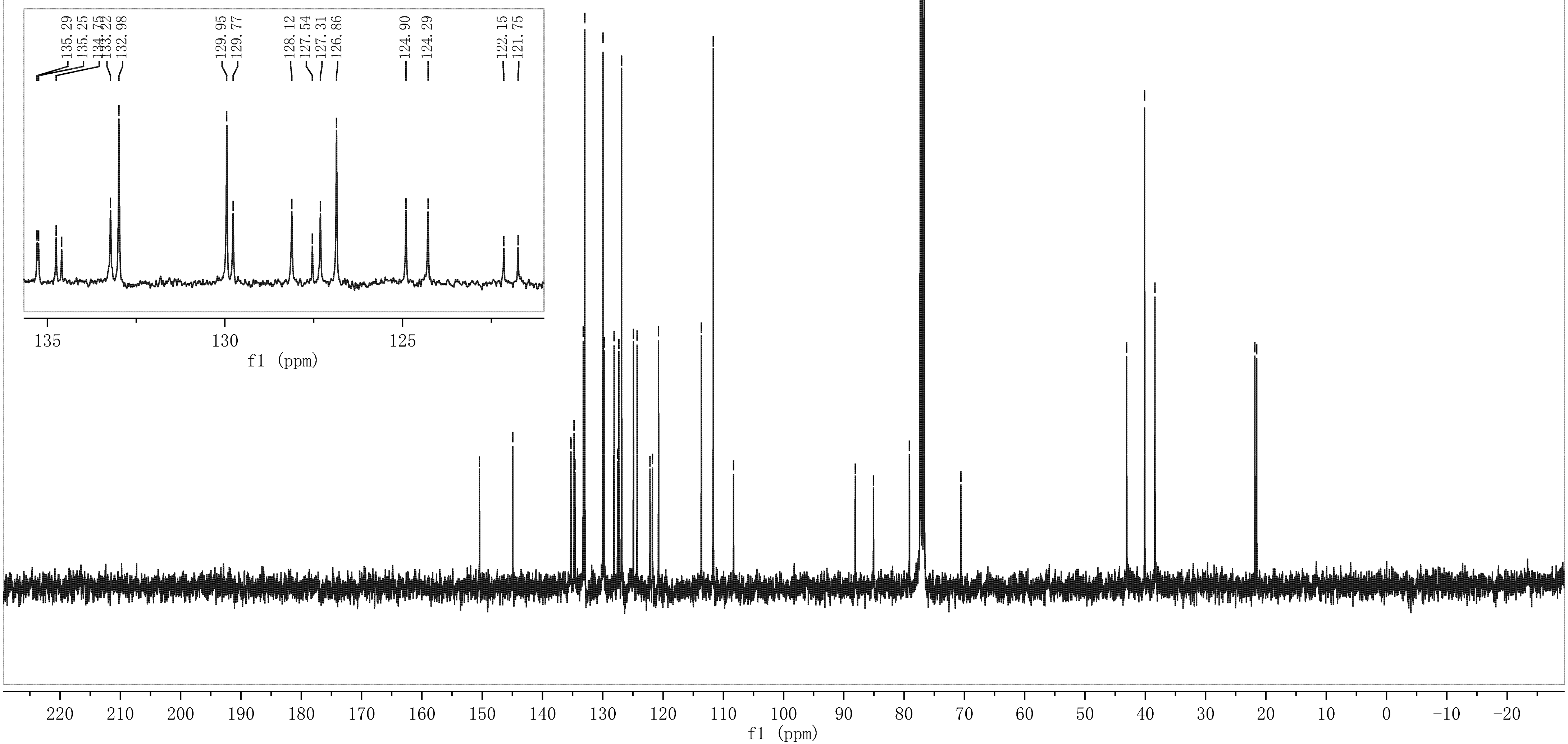

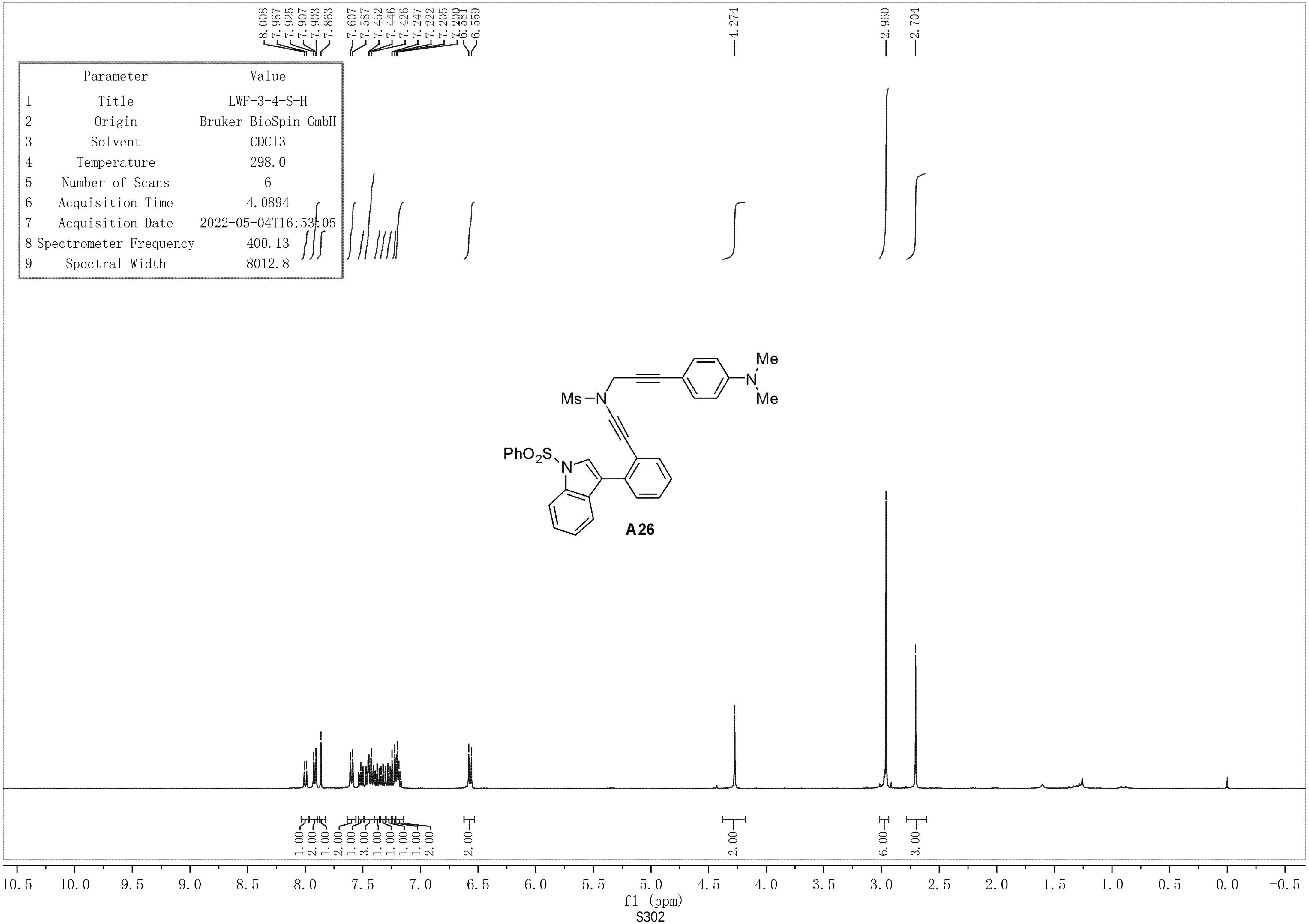
8.008
7.987
7.925
7.907
7.903
7.863
7.607
7.587
7.452
7.446
7.426
7.247
7.222
7.205
7.200
6.581
6.559
4.274
2.960
2.704
Parameter Value
1 Title LWF-3-4-S-H
2 Origin Bruker BioSpin GmbH
3 Solvent CDCl3
4 Temperature 298.0
5 Number of Scans 6
6 Acquisition Time 4.0894
7 Acquisition Date 2022-05-04T16:53:05
8 Spectrometer Frequency 400.13
9 Spectral Width 8012.8
Me
Ms−N
N
Me
PhO2S
N
A26
1.00
2.00
1.00
2.00
1.00
3.00
1.00
1.00
1.00
1.00
2.00
2.00
2.00
6.00
3.00
10.5 10.0 9.5 9.0 8.5 8.0 7.5 7.0 6.5 6.0 5.5 5.0 4.5 4.0 3.5 3.0 2.5 2.0 1.5 1.0 0.5 0.0 −0.5
f1 (ppm)

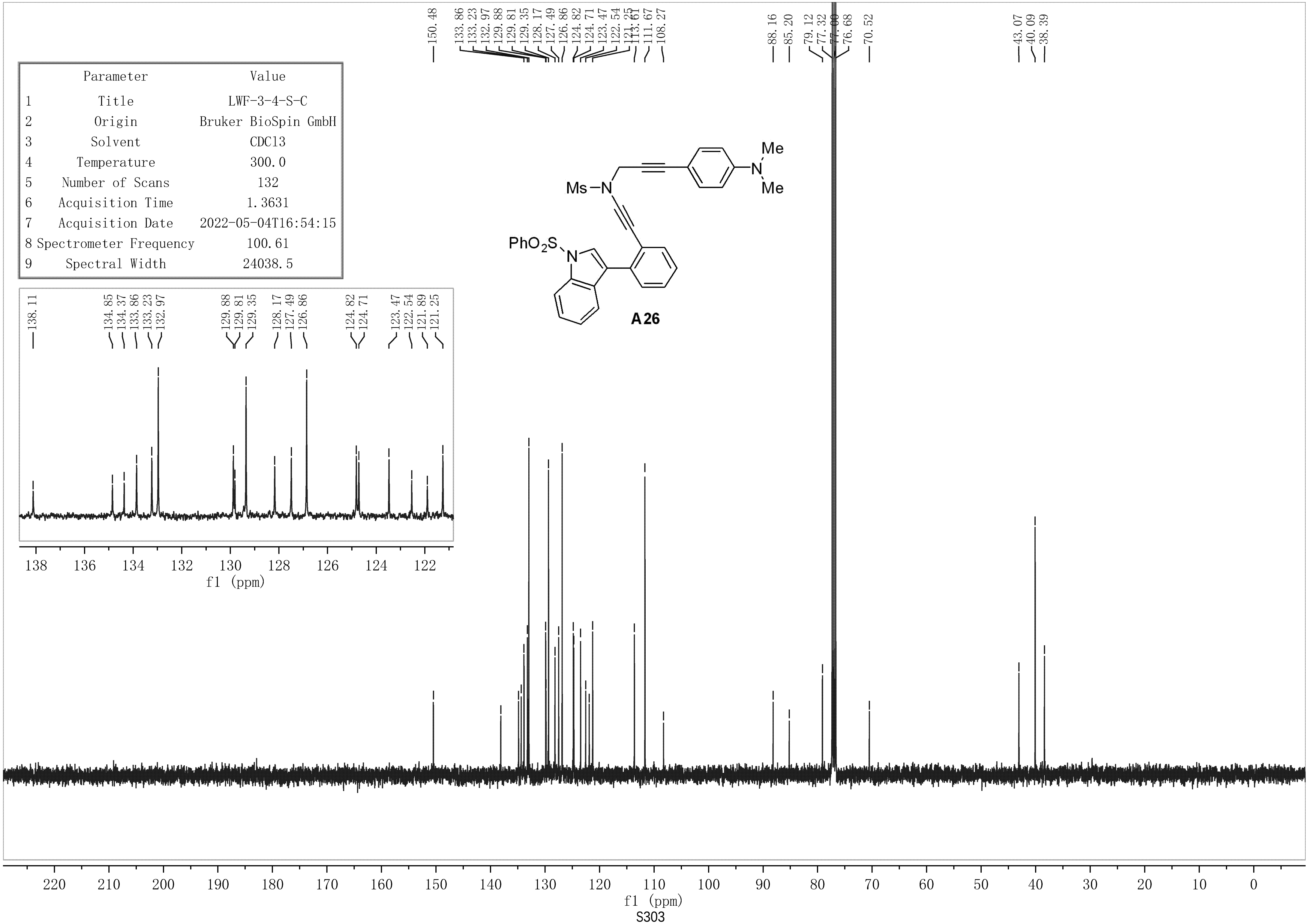

	Parameter	Value
1	Title	LWF-3-4-S-C
2	Origin	Bruker BioSpin GmbH
3	Solvent	CDCl3
4	Temperature	300.0
5	Number of Scans	132
6	Acquisition Time	1.3631
7	Acquisition Date	2022-05-04T16:54:15
8	Spectrometer Frequency	100.61
9	Spectral Width	24038.5

A26

	Parameter	Value
1	Title	LWF-3-3-S-1
2	Origin	Bruker BioSpin GmbH
3	Solvent	CDCl3
4	Temperature	298.0
5	Number of Scans	8
6	Acquisition Time	4.0894
7	Acquisition Date	2022-04-15T14:21:14
8	Spectrometer Frequency	400.13
9	Spectral Width	8012.8

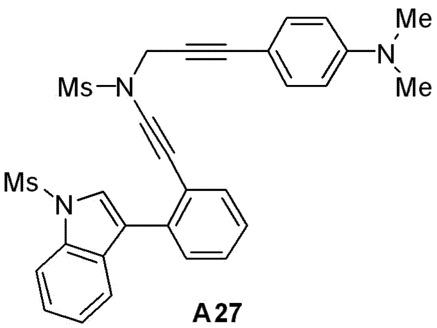

A27

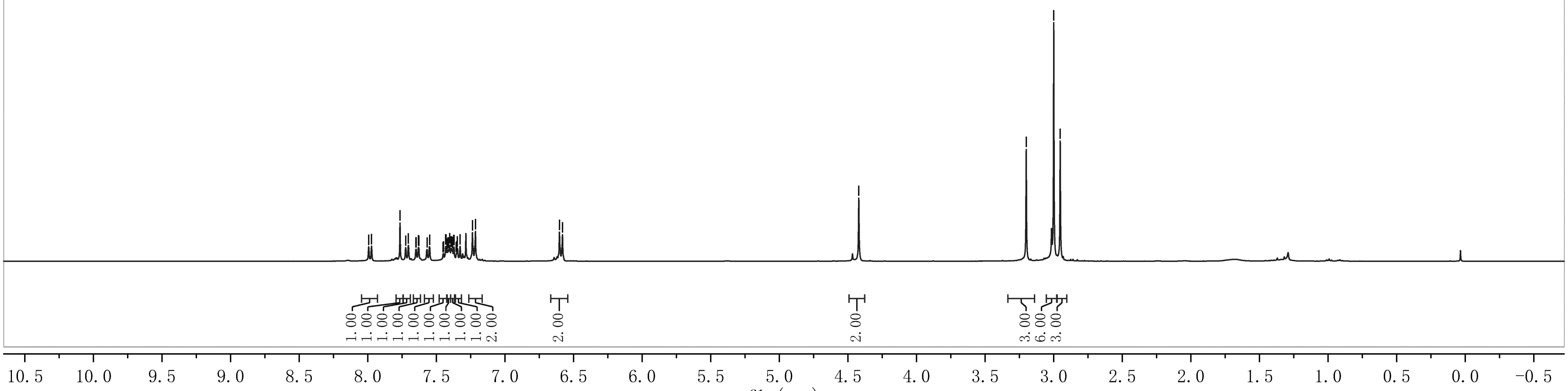

	Parameter	Value
1	Title	LWF-3-3-S-C
2	Origin	Bruker BioSpin GmbH
3	Solvent	CDCl3
4	Temperature	300.0
5	Number of Scans	60
6	Acquisition Time	1.3631
7	Acquisition Date	2022-04-12T14:37:53
8	Spectrometer Frequency	100.61
9	Spectral Width	24038.5

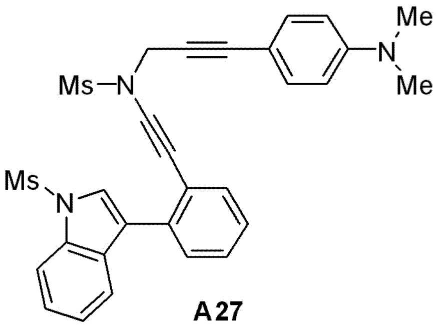

A27

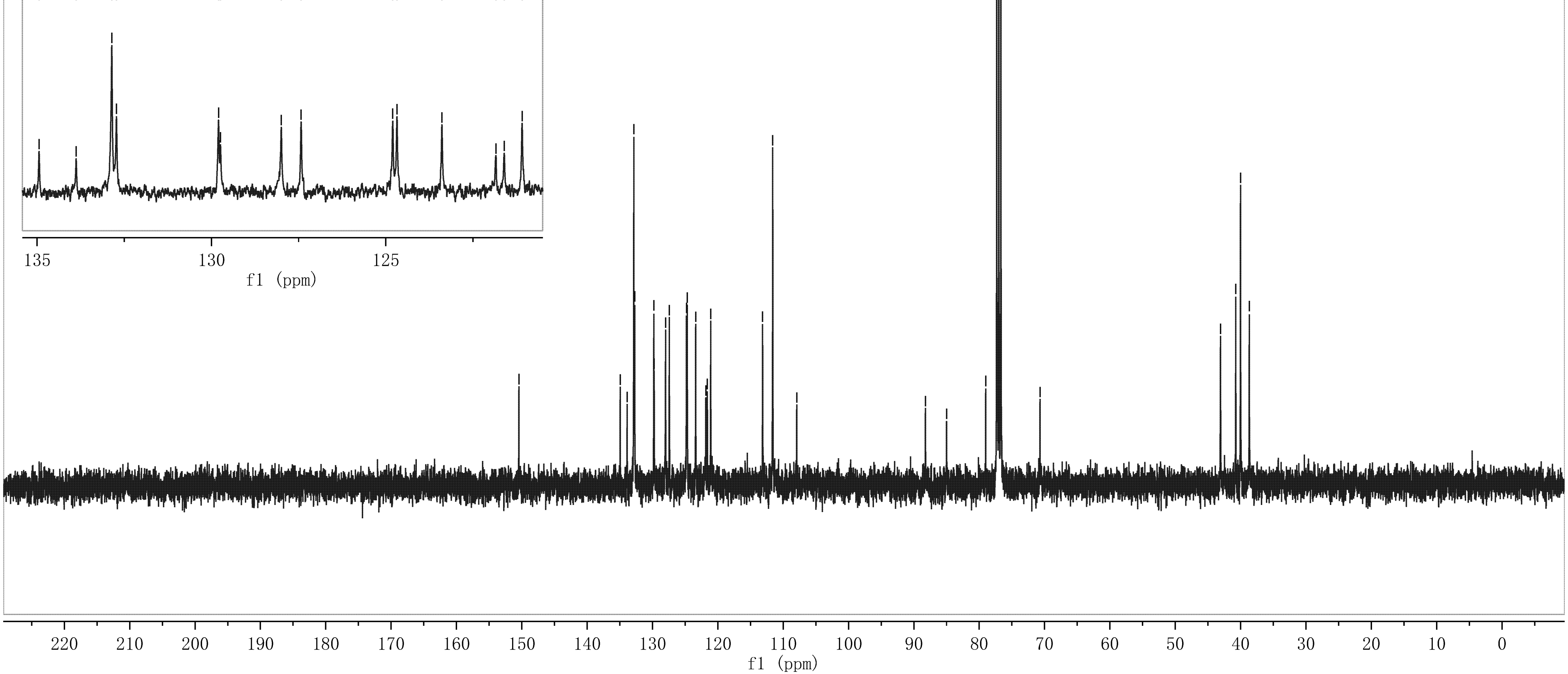

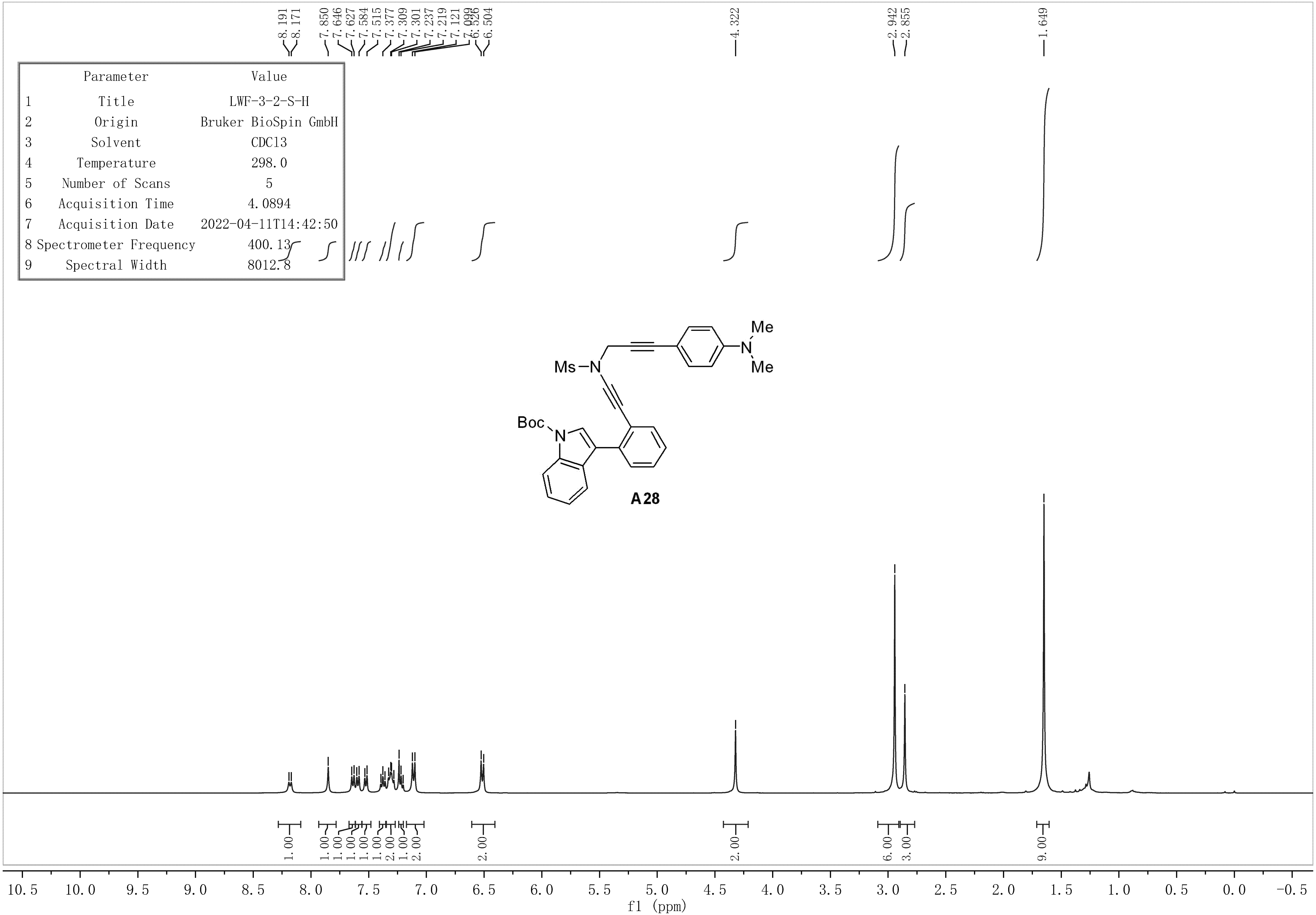

	Parameter	Value
1	Title	LWF-3-2-S-H
2	Origin	Bruker BioSpin GmbH
3	Solvent	CDCl3
4	Temperature	298.0
5	Number of Scans	5
6	Acquisition Time	4.0894
7	Acquisition Date	2022-04-11T14:42:50
8	Spectrometer Frequency	400.13
9	Spectral Width	8012.8

	Parameter	Value
1	Title	LWF-3-2-S-C
2	Origin	Bruker BioSpin GmbH
3	Solvent	CDCl3
4	Temperature	300.0
5	Number of Scans	30
6	Acquisition Time	1.3631
7	Acquisition Date	2022-04-11T14:44:43
8	Spectrometer Frequency	100.61
9	Spectral Width	24038.5

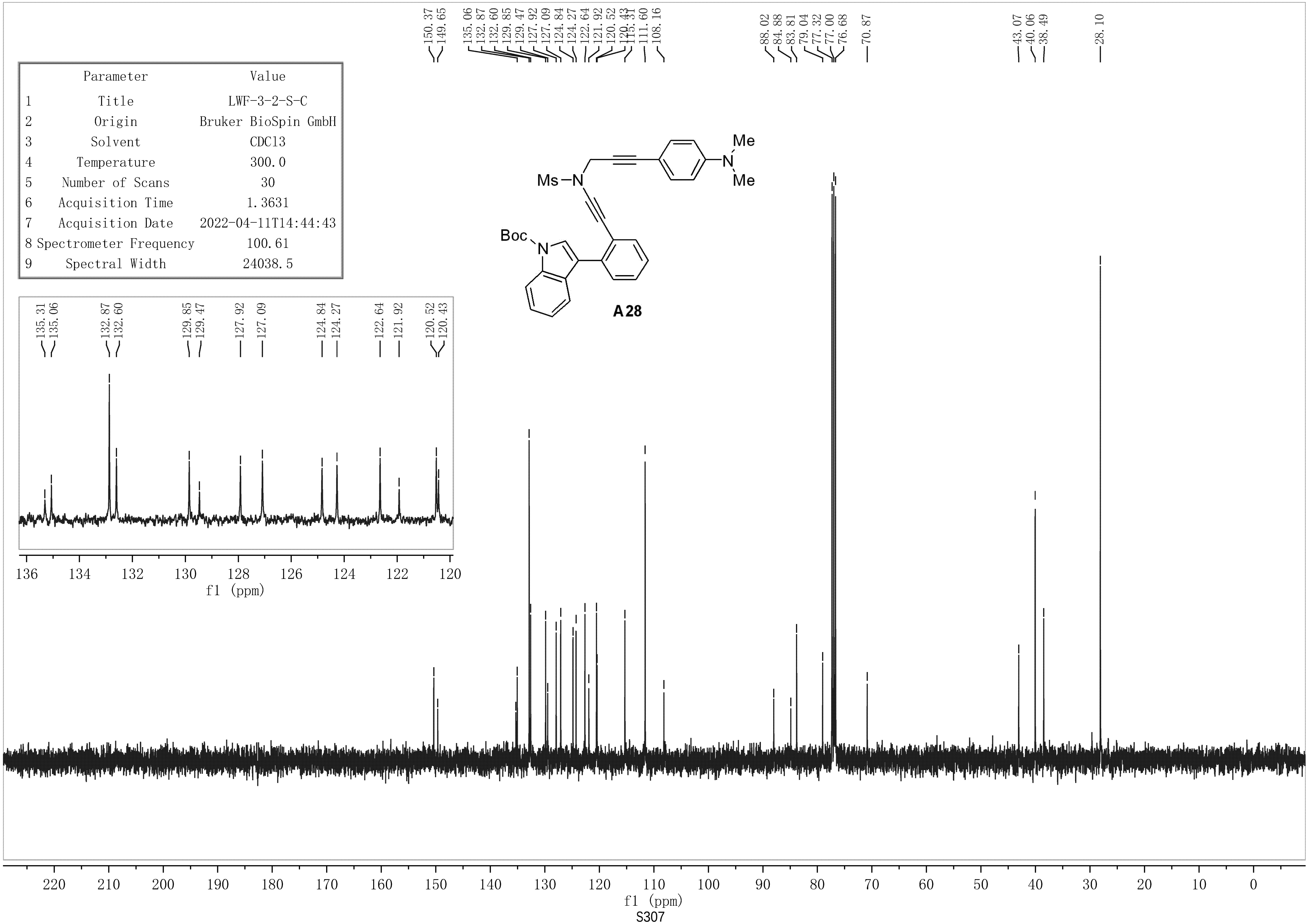

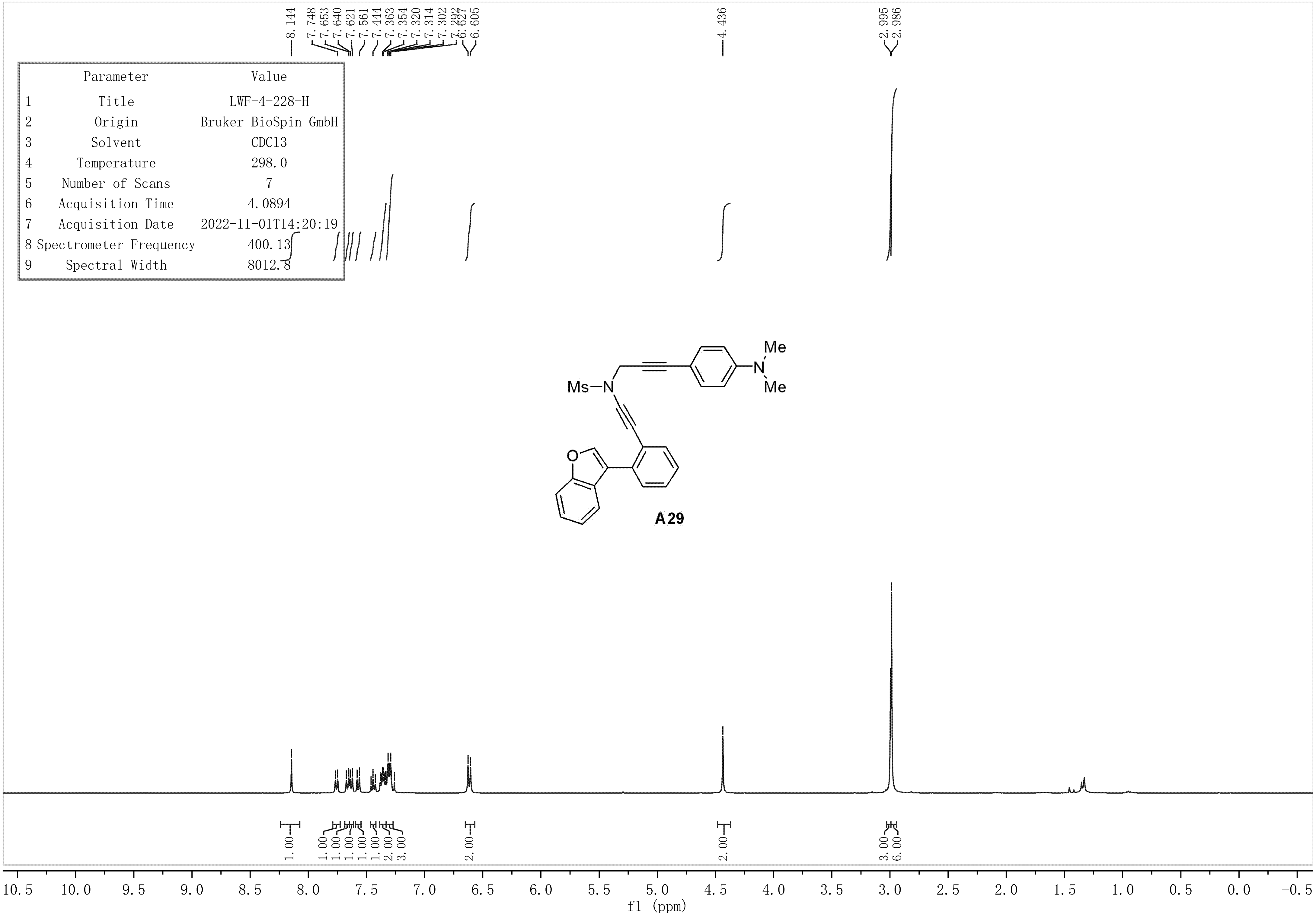

	Parameter	Value
1	Title	LWF-4-228-H
2	Origin	Bruker BioSpin GmbH
3	Solvent	CDCl3
4	Temperature	298.0
5	Number of Scans	7
6	Acquisition Time	4.0894
7	Acquisition Date	2022-11-01T14:20:19
8	Spectrometer Frequency	400.13
9	Spectral Width	8012.8

	Parameter	Value
1	Title	LWF-4-228-C
2	Origin	
3	Solvent	CDCl3
4	Temperature	298.1
5	Number of Scans	100
6	Acquisition Time	1.0000
7	Acquisition Date	2022-11-01T17:03:55
8	Spectrometer Frequency	100.56
9	Spectral Width	26041.0

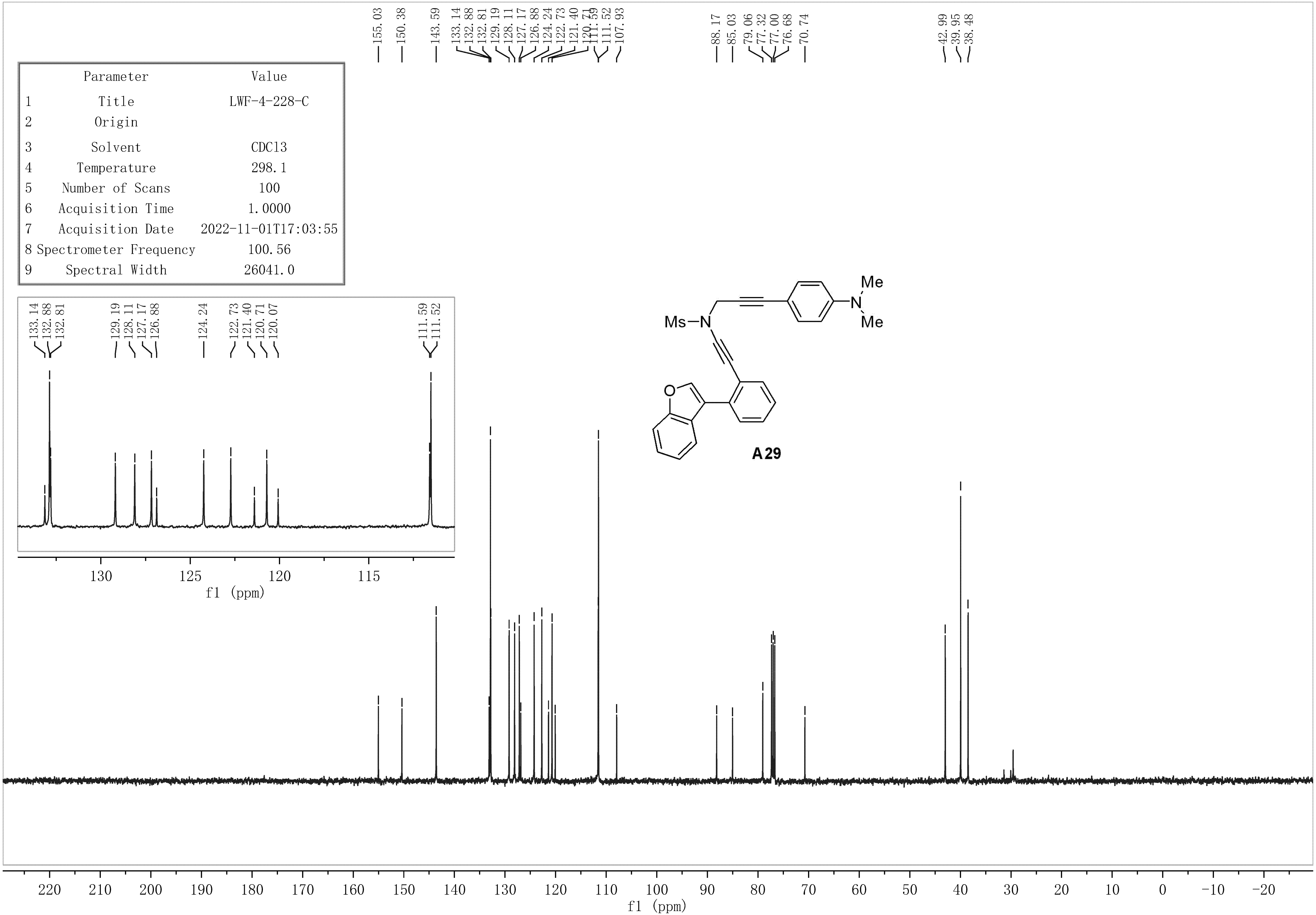

A29

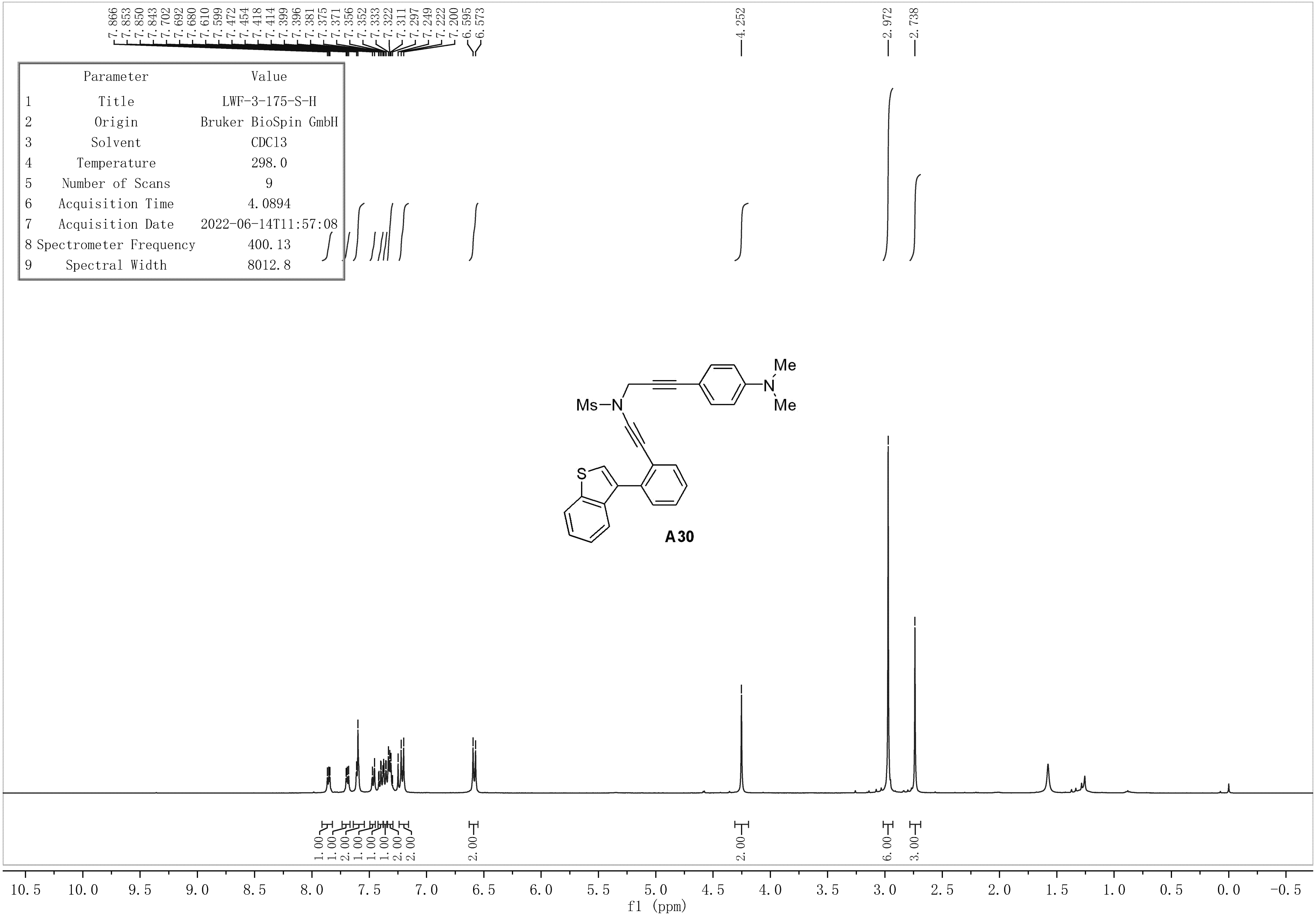

	Parameter	Value
1	Title	LWF-3-175-S-H
2	Origin	Bruker BioSpin GmbH
3	Solvent	CDCl3
4	Temperature	298.0
5	Number of Scans	9
6	Acquisition Time	4.0894
7	Acquisition Date	2022-06-14T11:57:08
8	Spectrometer Frequency	400.13
9	Spectral Width	8012.8

	Parameter	Value
1	Title	LWF-3-175-S-C
2	Origin	Bruker BioSpin GmbH
3	Solvent	CDCl3
4	Temperature	300.0
5	Number of Scans	116
6	Acquisition Time	1.3631
7	Acquisition Date	2022-06-13T18:09:05
8	Spectrometer Frequency	100.61
9	Spectral Width	24038.5

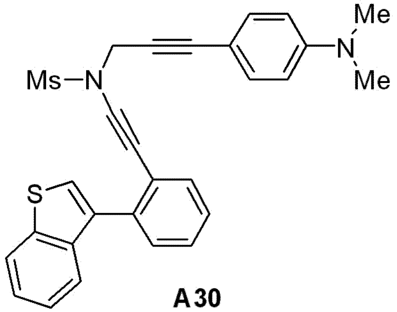

A30

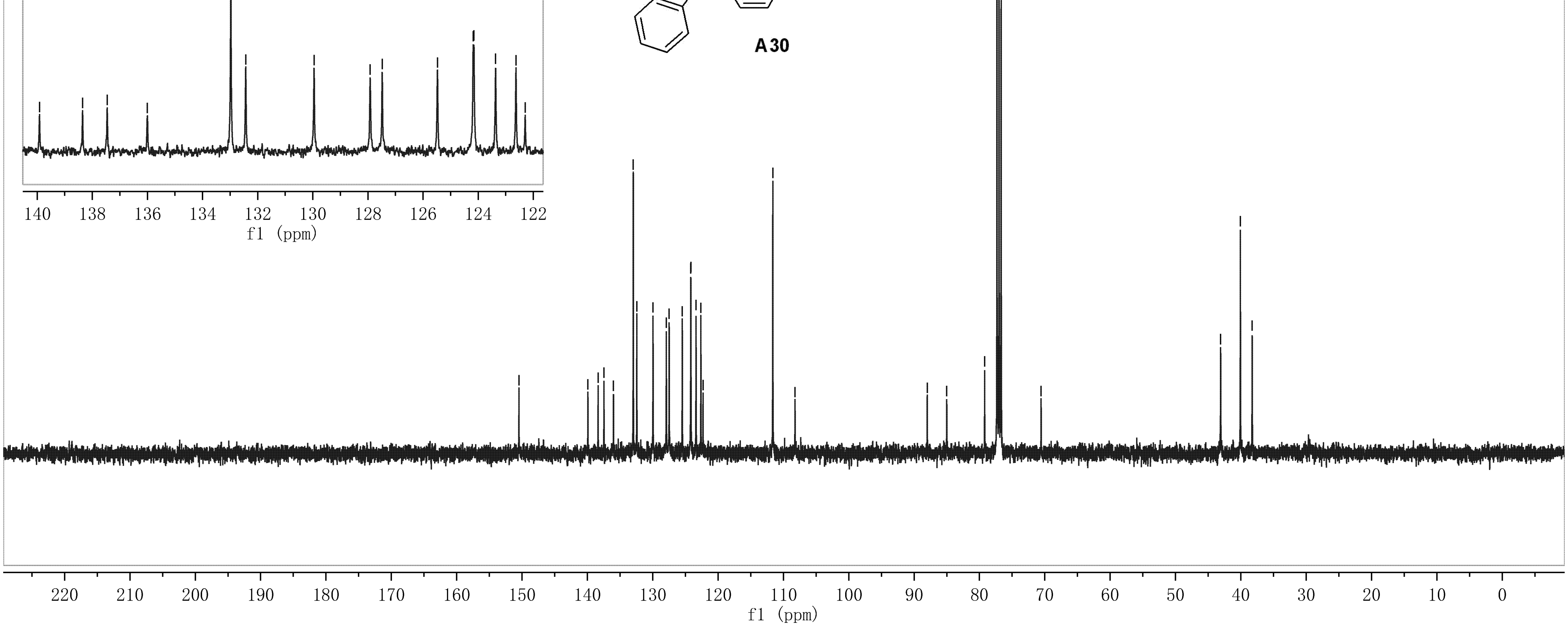

	Parameter	Value
1	Title	LWF-3-201-S-H
2	Origin	Bruker BioSpin GmbH
3	Solvent	CDCl3
4	Temperature	298.0
5	Number of Scans	11
6	Acquisition Time	4.0894
7	Acquisition Date	2022-06-17T11:57:35
8	Spectrometer Frequency	400.13
9	Spectral Width	8012.8

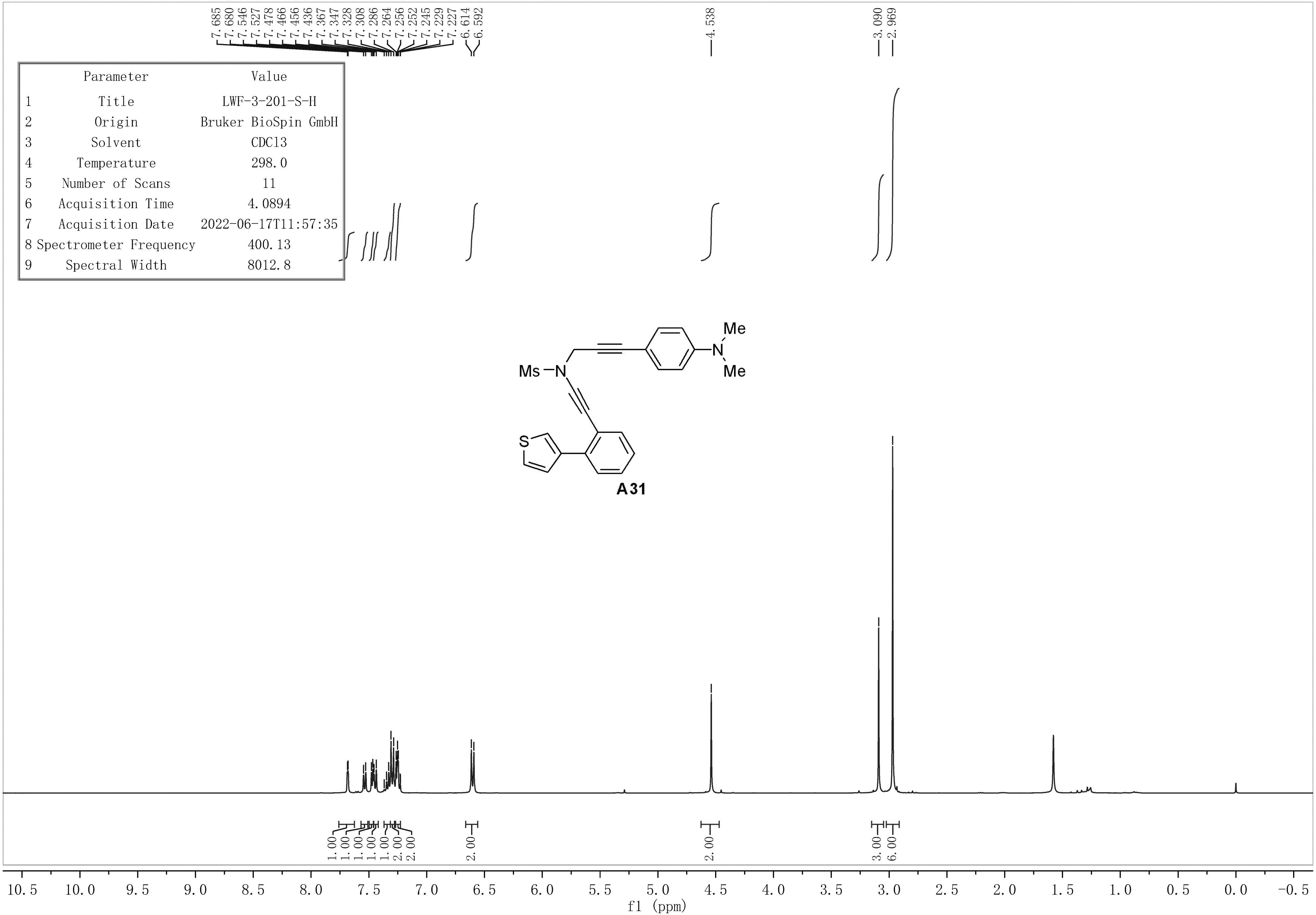

A31

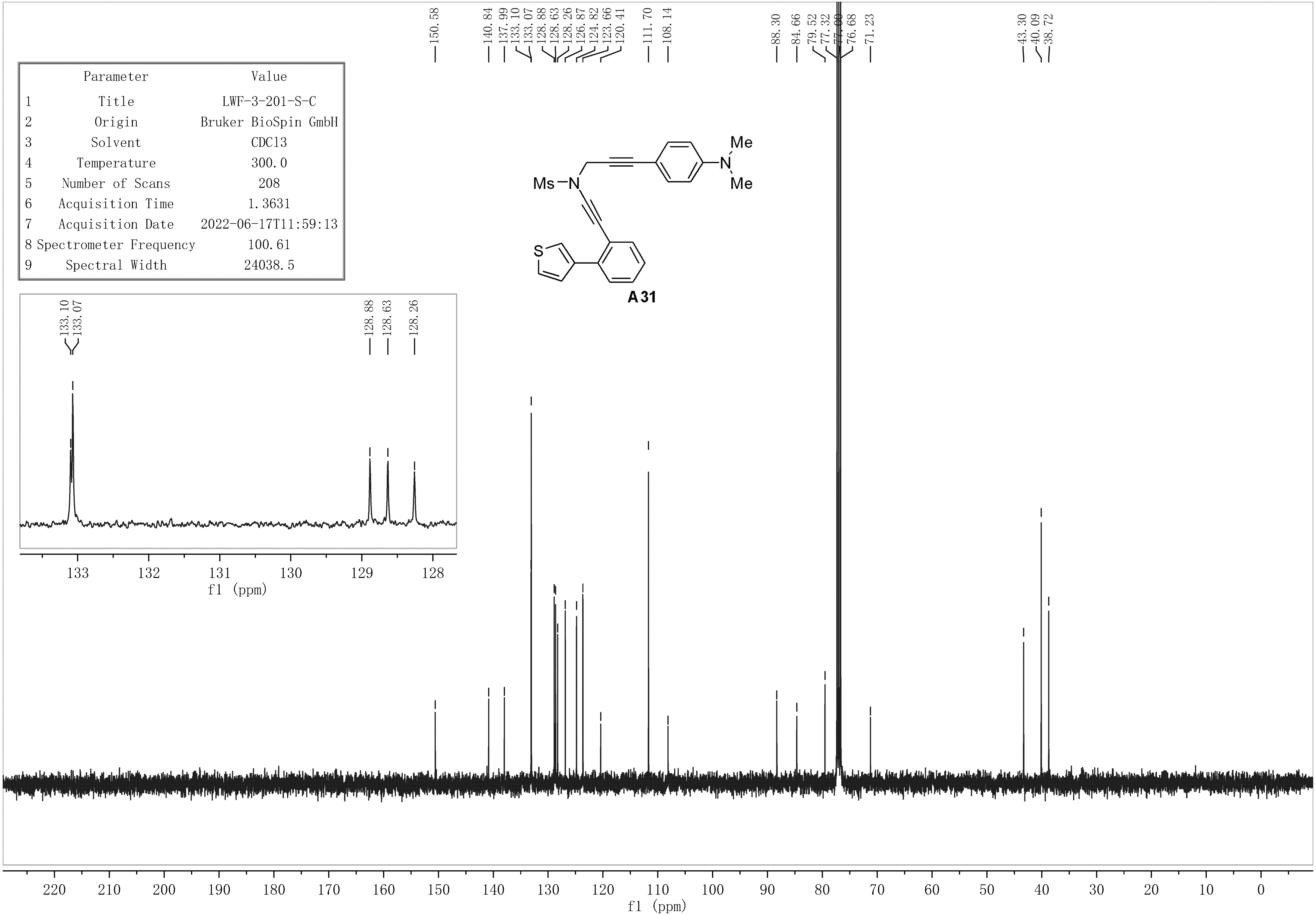

Parameter Value
1 Title LWF-3-201-S-C
2 Origin Bruker BioSpin GmbH
3 Solvent CDCl3
4 Temperature 300.0
5 Number of Scans 208
6 Acquisition Time 1.3631
7 Acquisition Date 2022-06-17T11:59:13
8 Spectrometer Frequency 100.61
9 Spectral Width 24038.5
150.58
140.84
137.99
133.10
133.07
128.88
128.63
128.26
126.87
124.82
123.66
120.41
111.70
108.14
88.30
84.66
79.52
77.32
76.68
71.23
43.30
40.09
38.72
Ms
N
Me
S
A 31
f1 (ppm)

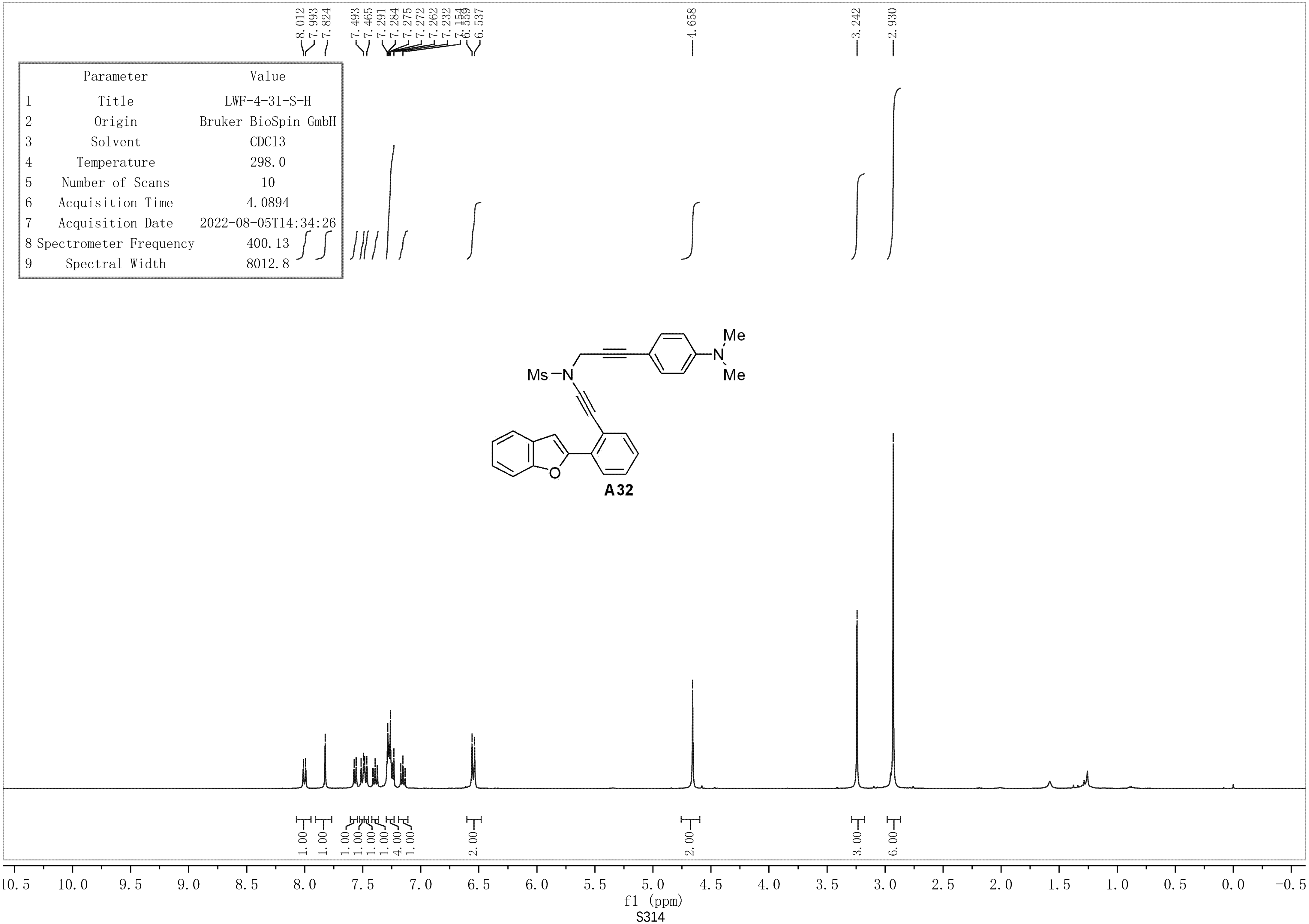

	Parameter	Value
1	Title	LWF-4-31-S-H
2	Origin	Bruker BioSpin GmbH
3	Solvent	CDCl3
4	Temperature	298.0
5	Number of Scans	10
6	Acquisition Time	4.0894
7	Acquisition Date	2022-08-05T14:34:26
8	Spectrometer Frequency	400.13
9	Spectral Width	8012.8

	Parameter	Value
1	Title	LWF-4-31-S-C
2	Origin	Bruker BioSpin GmbH
3	Solvent	CDCl3
4	Temperature	300.0
5	Number of Scans	51
6	Acquisition Time	1.3631
7	Acquisition Date	2022-08-05T14:35:58
8	Spectrometer Frequency	100.61
9	Spectral Width	24038.5

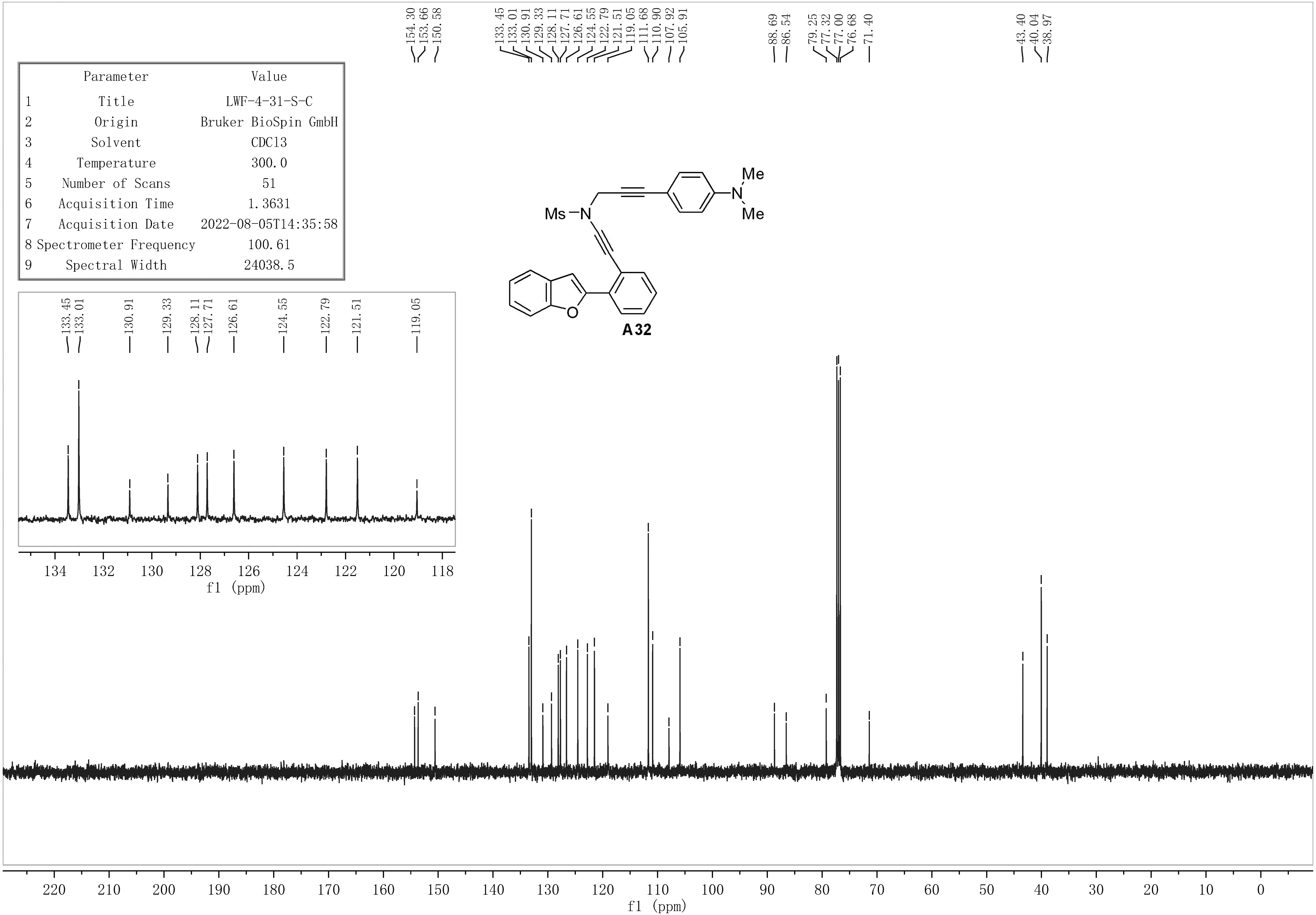

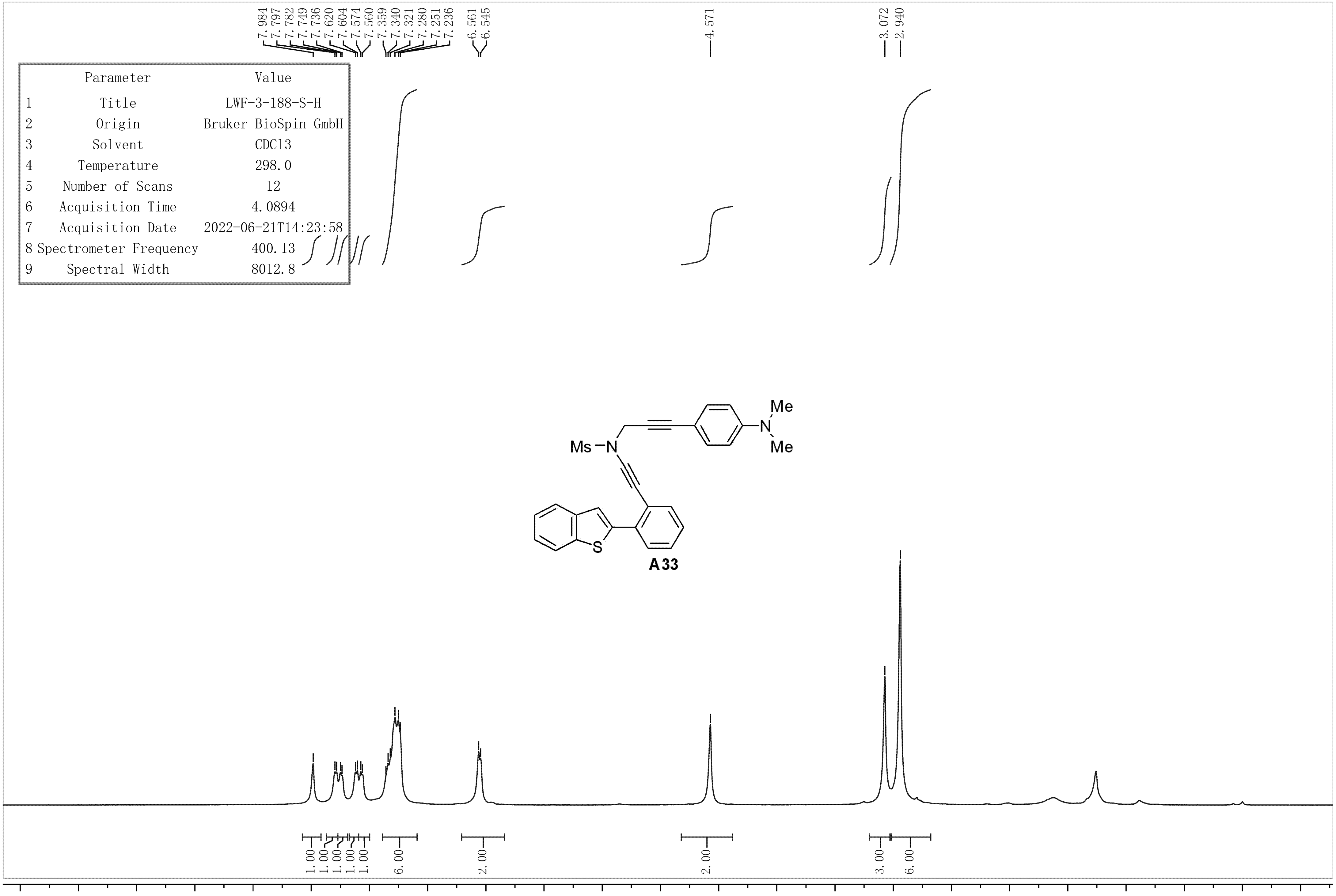

	Parameter	Value
1	Title	LWF-3-188-S-H
2	Origin	Bruker BioSpin GmbH
3	Solvent	CDCl3
4	Temperature	298.0
5	Number of Scans	12
6	Acquisition Time	4.0894
7	Acquisition Date	2022-06-21T14:23:58
8	Spectrometer Frequency	400.13
9	Spectral Width	8012.8

A33

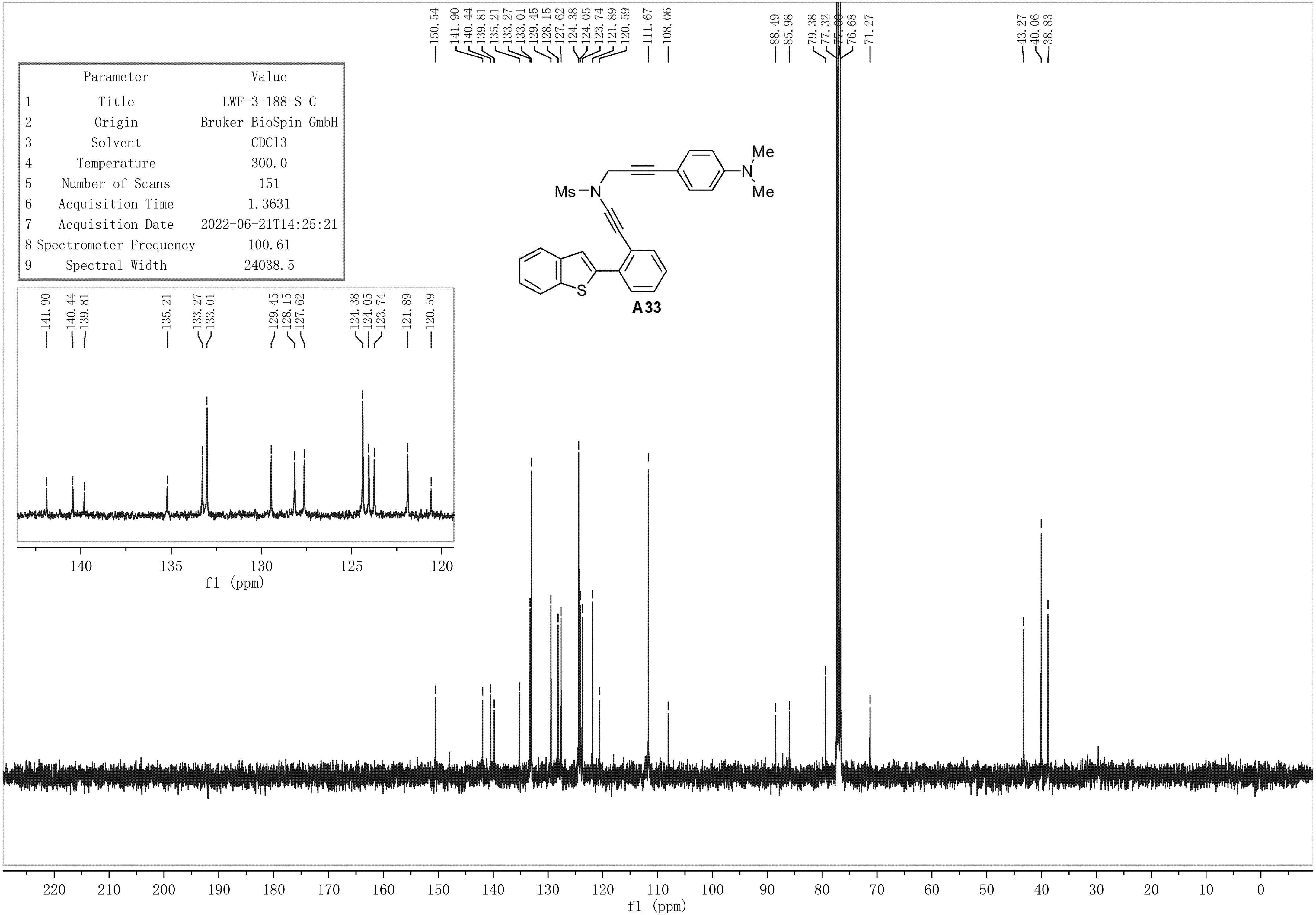

	Parameter	Value
1	Title	LWF-3-188-S-C
2	Origin	Bruker BioSpin GmbH
3	Solvent	CDCl3
4	Temperature	300.0
5	Number of Scans	151
6	Acquisition Time	1.3631
7	Acquisition Date	2022-06-21T14:25:21
8	Spectrometer Frequency	100.61
9	Spectral Width	24038.5

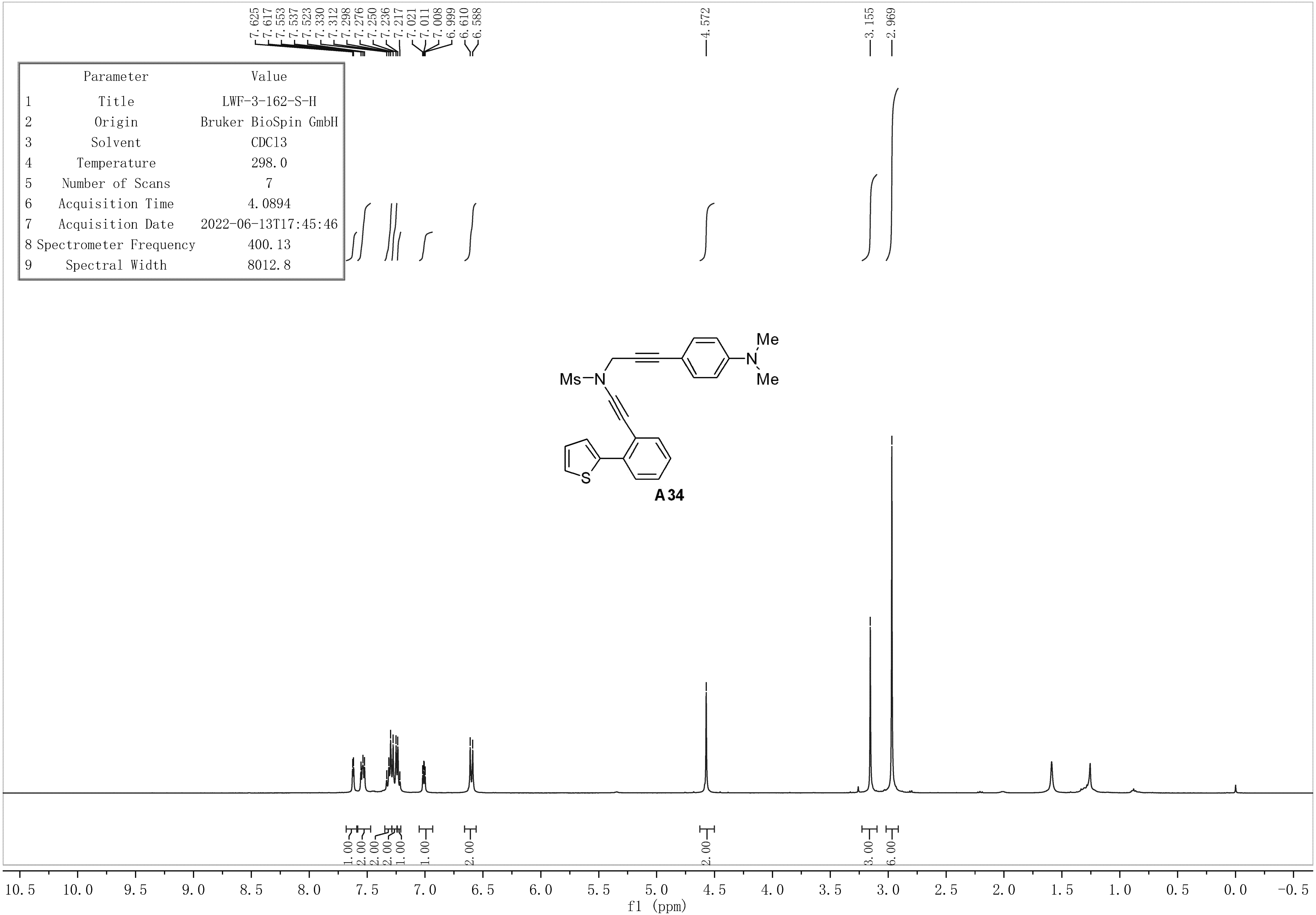
7.625 7.617 7.553 7.537 7.523 7.330 7.312 7.298 7.276 7.250 7.236 7.217 7.021 7.011 7.008 6.999 6.610 6.588
4.572
3.155 2.969
Parameter Value
1 Title LWF-3-162-S-H
2 Origin Bruker BioSpin GmbH
3 Solvent CDCl3
4 Temperature 298.0
5 Number of Scans 7
6 Acquisition Time 4.0894
7 Acquisition Date 2022-06-13T17:45:46
8 Spectrometer Frequency 400.13
9 Spectral Width 8012.8
Me
Ms
N
Me
S
A 34
1.00 2.00 2.00 2.00 1.00 1.00 2.00 2.00 3.00 6.00
10.5 10.0 9.5 9.0 8.5 8.0 7.5 7.0 6.5 6.0 5.5 5.0 4.5 4.0 3.5 3.0 2.5 2.0 1.5 1.0 0.5 0.0 −0.5
f1 (ppm)

	Parameter	Value
1	Title	LWF-3-162-S-C
2	Origin	Bruker BioSpin GmbH
3	Solvent	CDCl3
4	Temperature	300.0
5	Number of Scans	104
6	Acquisition Time	1.3631
7	Acquisition Date	2022-06-13T17:46:55
8	Spectrometer Frequency	100.61
9	Spectral Width	24038.5

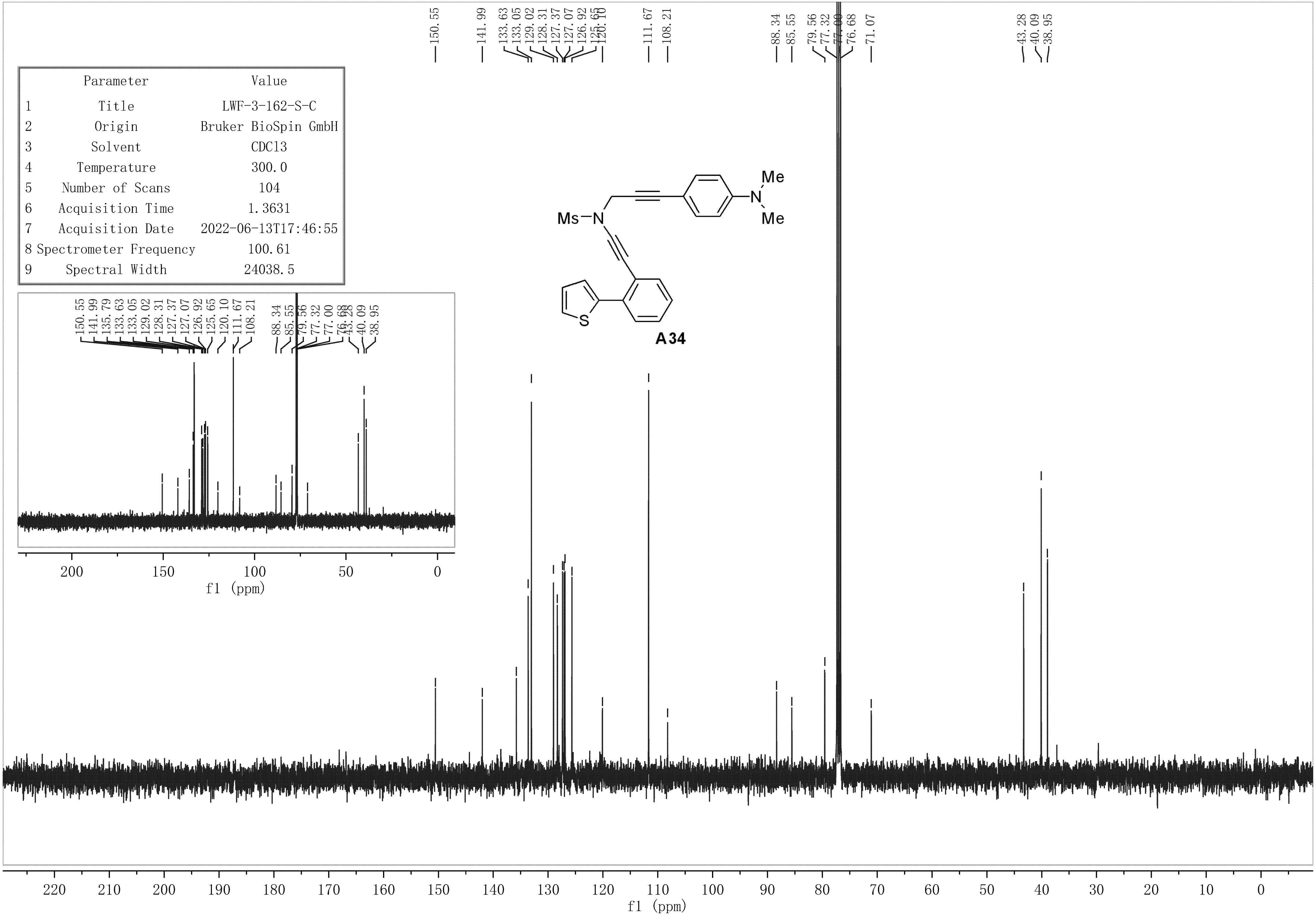

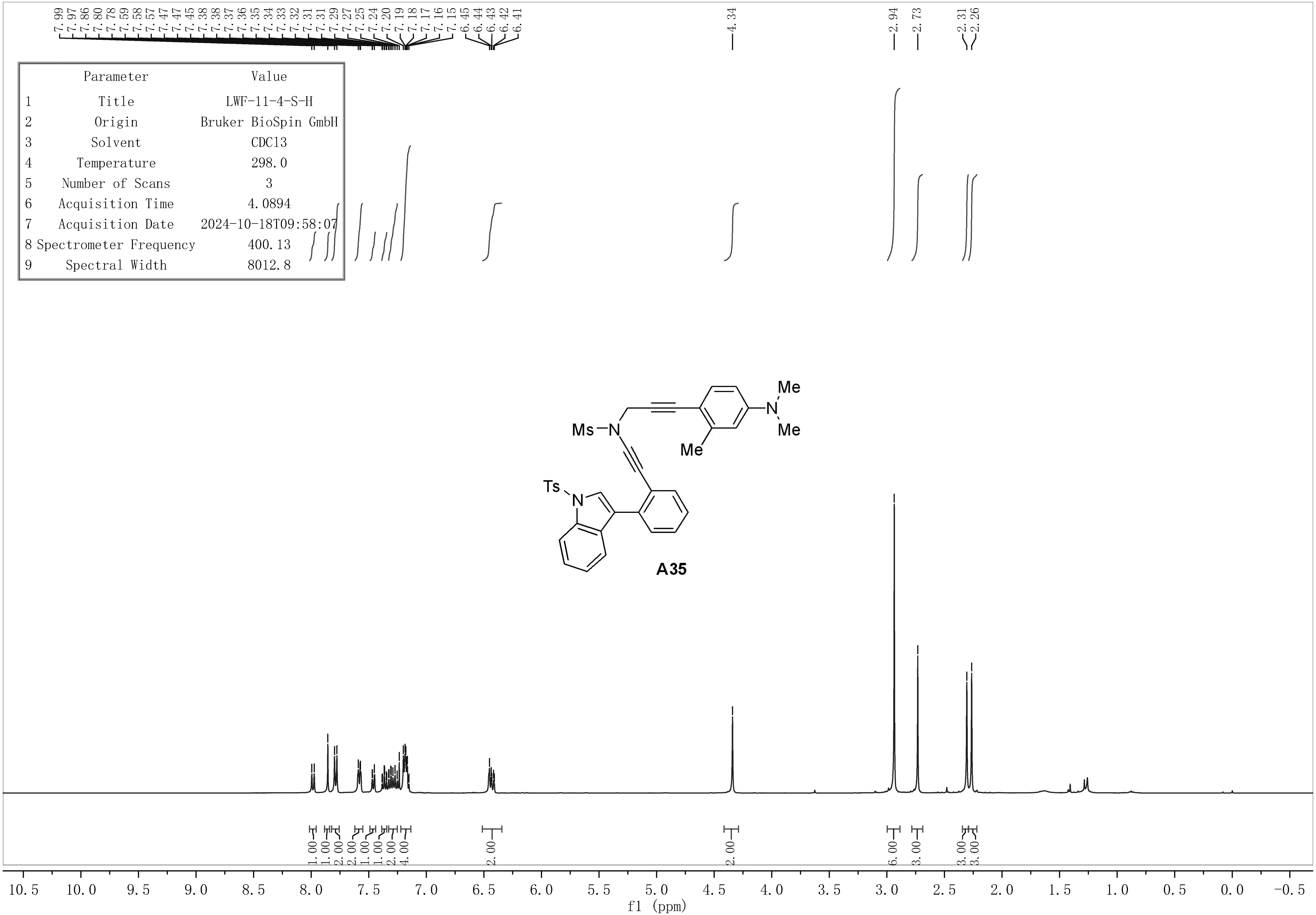

	Parameter	Value
1	Title	LWF-11-4-S-H
2	Origin	Bruker BioSpin GmbH
3	Solvent	CDCl3
4	Temperature	298.0
5	Number of Scans	3
6	Acquisition Time	4.0894
7	Acquisition Date	2024-10-18T09:58:07
8	Spectrometer Frequency	400.13
9	Spectral Width	8012.8

	Parameter	Value
1	Title	LWF-11-4-S-C
2	Origin	Bruker BioSpin GmbH
3	Solvent	CDCl3
4	Temperature	300.0
5	Number of Scans	105
6	Acquisition Time	1.3631
7	Acquisition Date	2024-10-18T10:25:21
8	Spectrometer Frequency	100.61
9	Spectral Width	24038.5

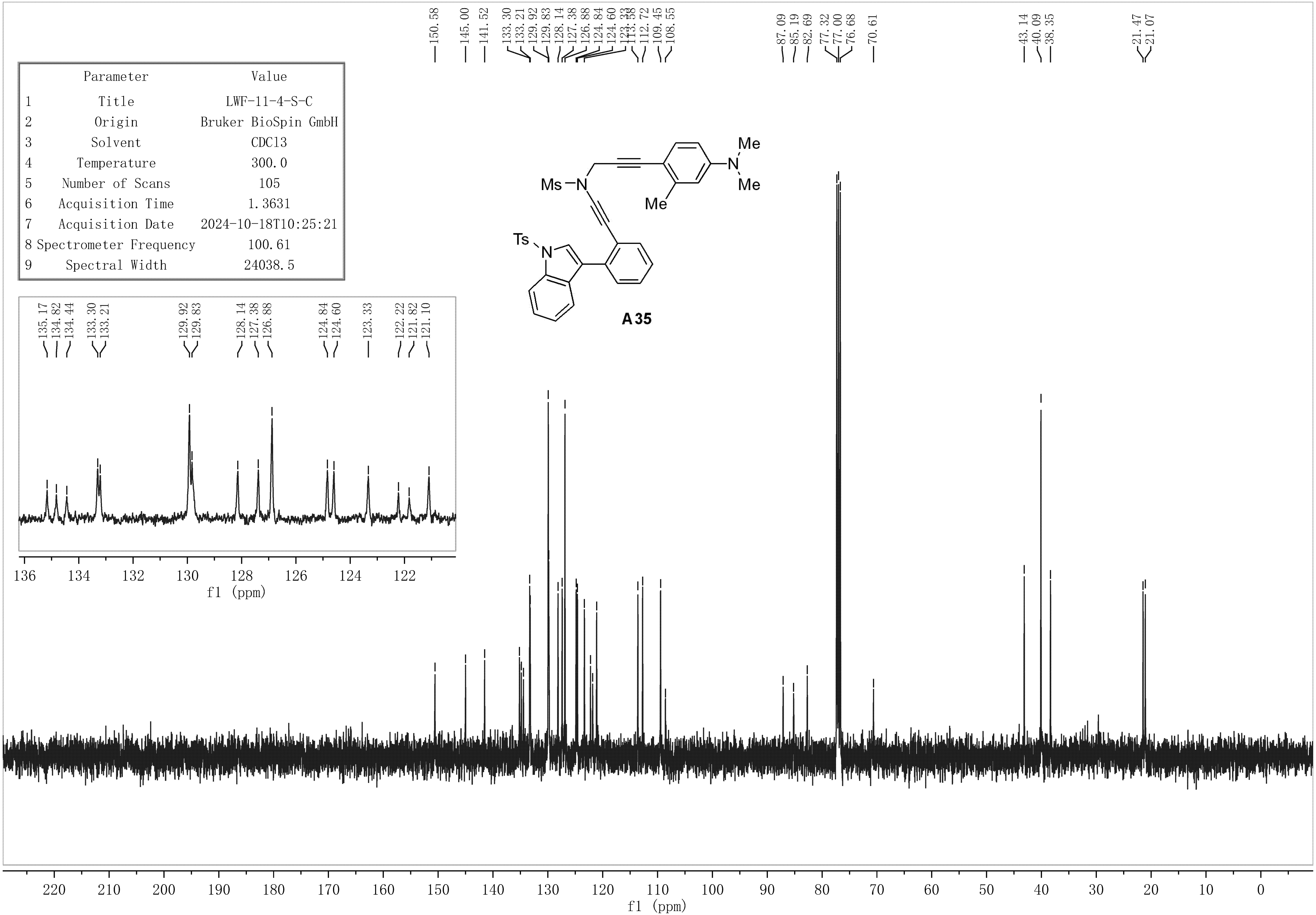

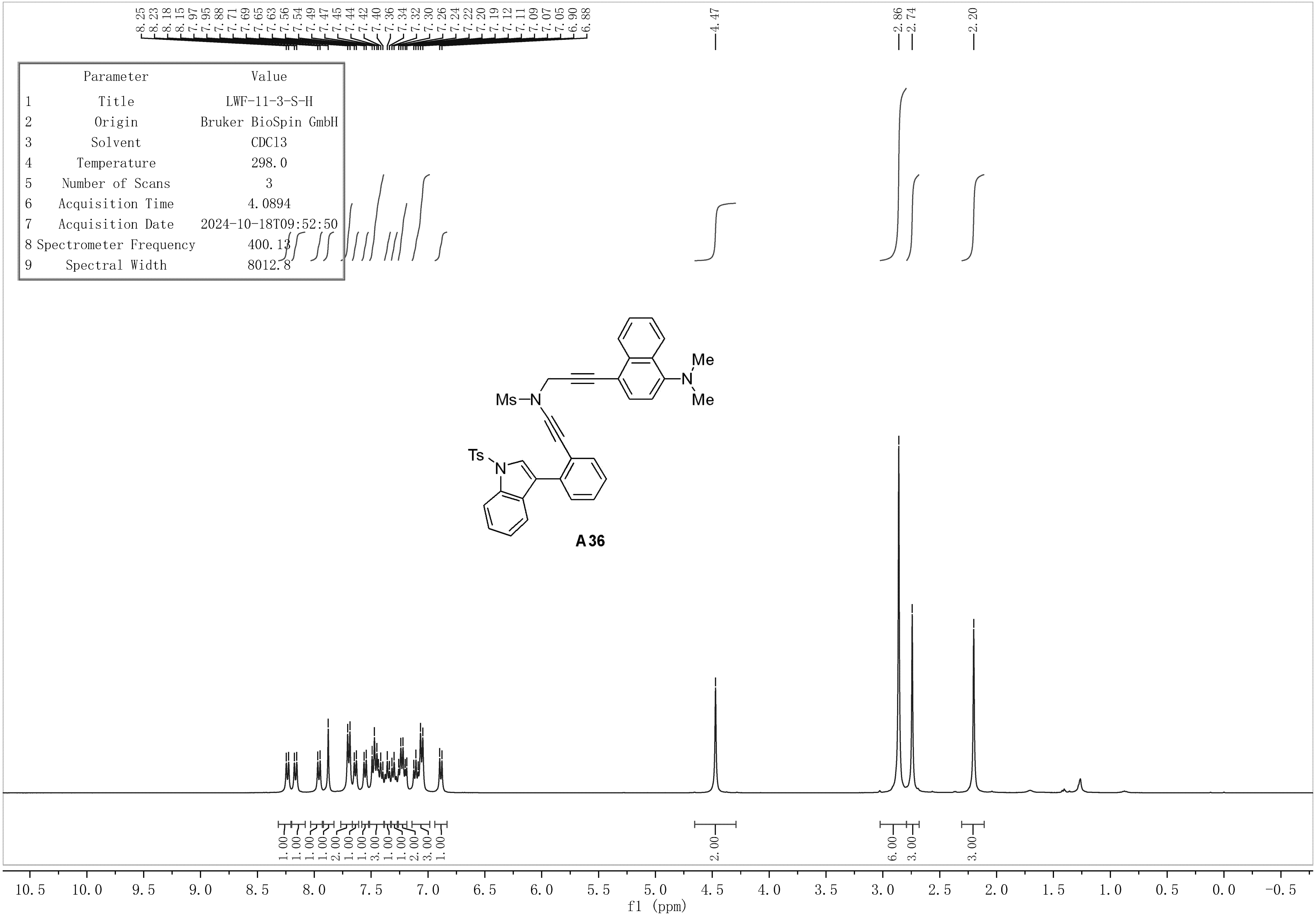
8.25 8.23 8.18 8.15 7.97 7.95 7.88 7.71 7.69 7.65 7.63 7.56 7.54 7.49 7.47 7.45 7.44 7.42 7.40 7.36 7.34 7.32 7.30 7.26 7.24 7.22 7.20 7.19 7.12 7.11 7.09 7.07 7.05 6.90 6.88
4.47
2.86 2.74
2.20
Parameter Value
1 Title LWF-11-3-S-H
2 Origin Bruker BioSpin GmbH
3 Solvent CDCl3
4 Temperature 298.0
5 Number of Scans 3
6 Acquisition Time 4.0894
7 Acquisition Date 2024-10-18T09:52:50
8 Spectrometer Frequency 400.13
9 Spectral Width 8012.8
Me
N
Me
Ms
N
Ts
N
A36
1.00 1.00 1.00 2.00 1.00 1.00 3.00 3.00 1.00 2.00 3.00 1.00
2.00
6.00 3.00
3.00
10.5 10.0 9.5 9.0 8.5 8.0 7.5 7.0 6.5 6.0 5.5 5.0 4.5 4.0 3.5 3.0 2.5 2.0 1.5 1.0 0.5 0.0 −0.5
f1 (ppm)

	Parameter	Value
1	Title	LWF-11-3-S-C
2	Origin	Bruker BioSpin GmbH
3	Solvent	CDCl3
4	Temperature	300.0
5	Number of Scans	17
6	Acquisition Time	1.3631
7	Acquisition Date	2024-10-18T09:53:57
8	Spectrometer Frequency	100.61
9	Spectral Width	24038.5

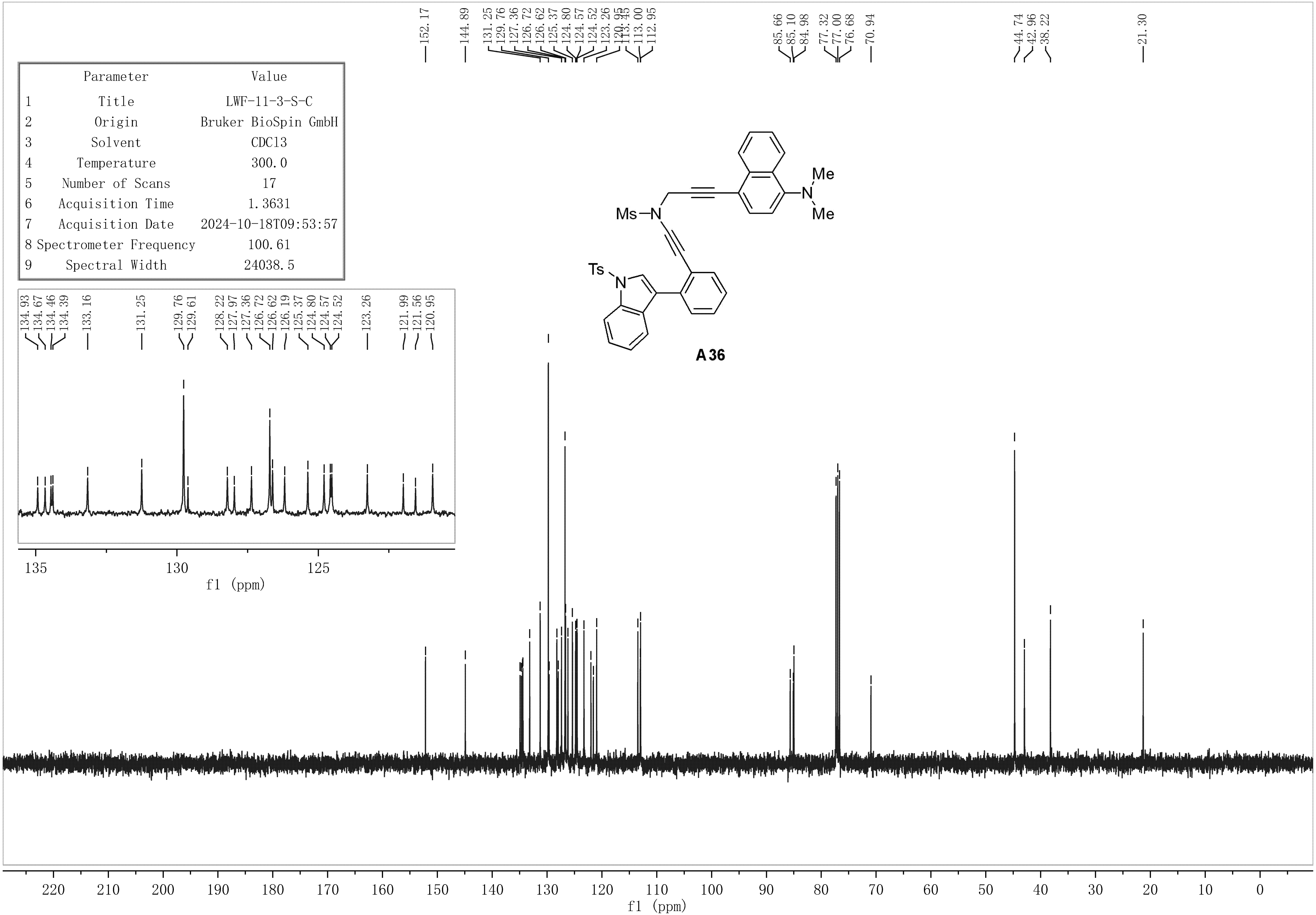

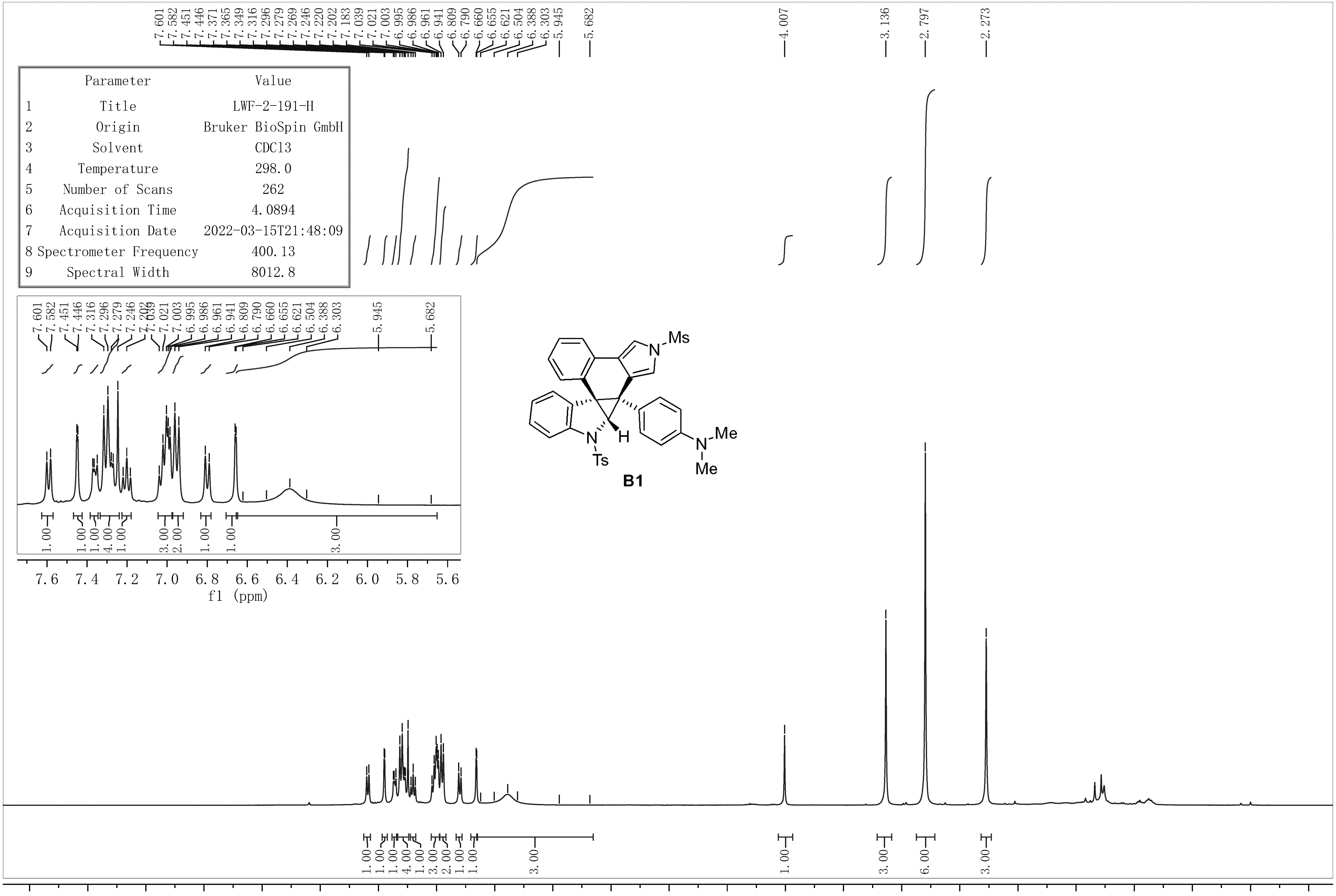
7.601 7.582 7.451 7.446 7.371 7.365 7.349 7.316 7.296 7.279 7.269 7.246 7.220 7.202 7.183 7.039 7.021 7.003 6.995 6.986 6.961 6.941 6.809 6.790 6.660 6.655 6.621 6.504 6.388 6.303 5.945 5.682
4.007
3.136
2.797
2.273
Parameter Value
1 Title LWF-2-191-H
2 Origin Bruker BioSpin GmbH
3 Solvent CDCl3
4 Temperature 298.0
5 Number of Scans 262
6 Acquisition Time 4.0894
7 Acquisition Date 2022-03-15T21:48:09
8 Spectrometer Frequency 400.13
9 Spectral Width 8012.8
Ms
N
N
H
Ts
N
Me
Me
B1
1.00 1.00 1.00 4.00 1.00 3.00 2.00 1.00 1.00 3.00
1.00 3.00 6.00 3.00
f1 (ppm)

	Parameter	Value
1	Title	LWF-2-191-C
2	Origin	Bruker BioSpin GmbH
3	Solvent	CDCl3
4	Temperature	300.0
5	Number of Scans	108
6	Acquisition Time	1.3631
7	Acquisition Date	2022-03-14T15:55:56
8	Spectrometer Frequency	100.61
9	Spectral Width	24038.5

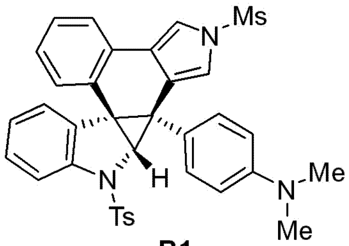

B1

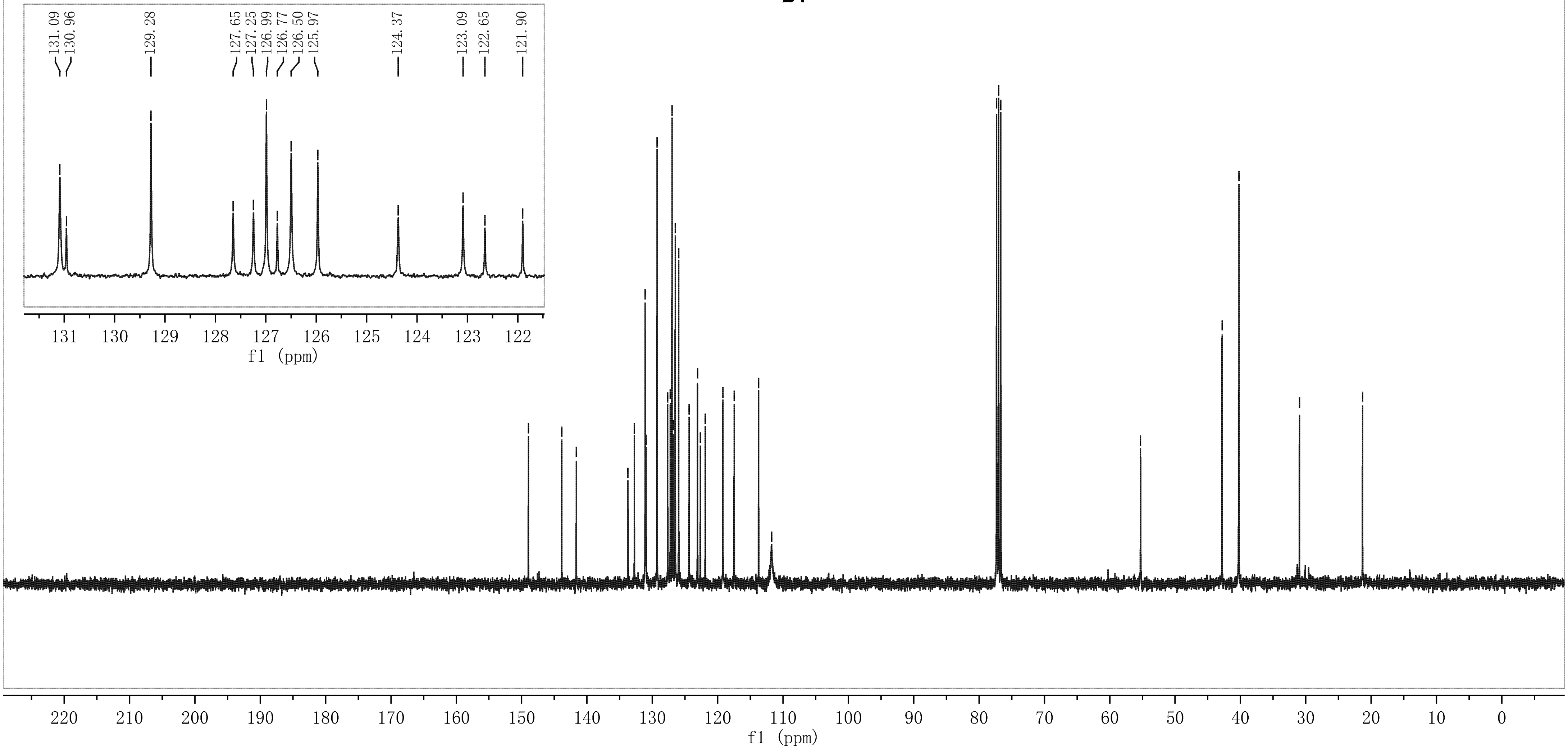

	Parameter	Value
1	Title	LWF-2-191-DEP
2	Origin	
3	Solvent	CDCl3
4	Temperature	297.4
5	Number of Scans	256
6	Acquisition Time	1.0000
7	Acquisition Date	2022-03-14T17:25:02
8	Spectrometer Frequency	100.56
9	Spectral Width	26041.0

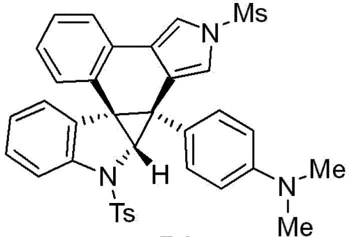

B1

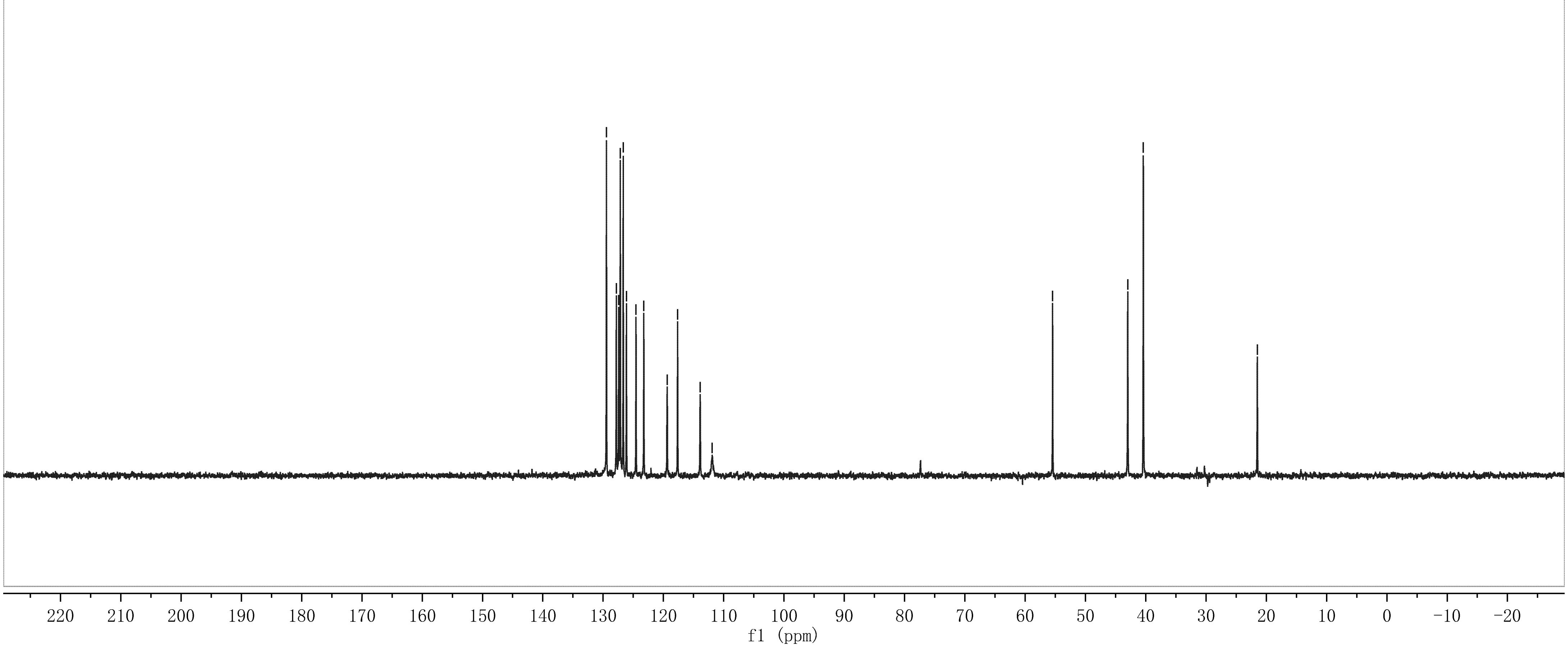

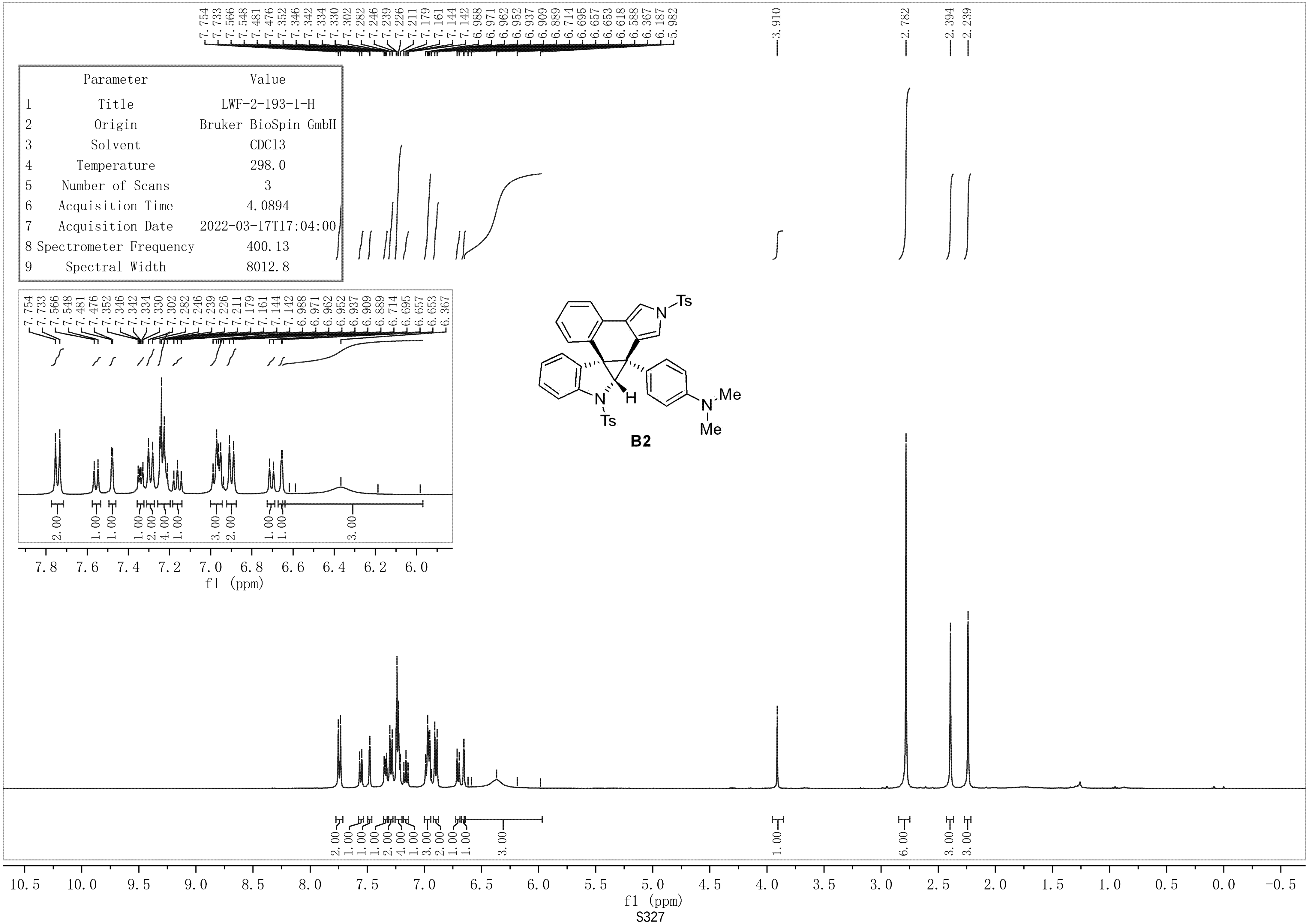

7.754 7.733 7.566 7.548 7.481 7.476 7.352 7.346 7.342 7.334 7.330 7.302 7.282 7.246 7.239 7.226 7.211 7.179 7.161 7.144 7.142 6.988 6.971 6.962 6.952 6.937 6.909 6.889 6.714 6.695 6.657 6.653 6.618 6.588 6.367 6.187 5.982
3.910
2.782
2.394
2.239
Parameter Value
1 Title LWF-2-193-1-H
2 Origin Bruker BioSpin GmbH
3 Solvent CDCl3
4 Temperature 298.0
5 Number of Scans 3
6 Acquisition Time 4.0894
7 Acquisition Date 2022-03-17T17:04:00
8 Spectrometer Frequency 400.13
9 Spectral Width 8012.8
N
Ts
H
N
Me
Me
B2
2.00 1.00 1.00 1.00 2.00 4.00 1.00 3.00 2.00 1.00 1.00 3.00
1.00
6.00
3.00
3.00
7.8 7.6 7.4 7.2 7.0 6.8 6.6 6.4 6.2 6.0
f1 (ppm)
10.5 10.0 9.5 9.0 8.5 8.0 7.5 7.0 6.5 6.0 5.5 5.0 4.5 4.0 3.5 3.0 2.5 2.0 1.5 1.0 0.5 0.0 -0.5
f1 (ppm)

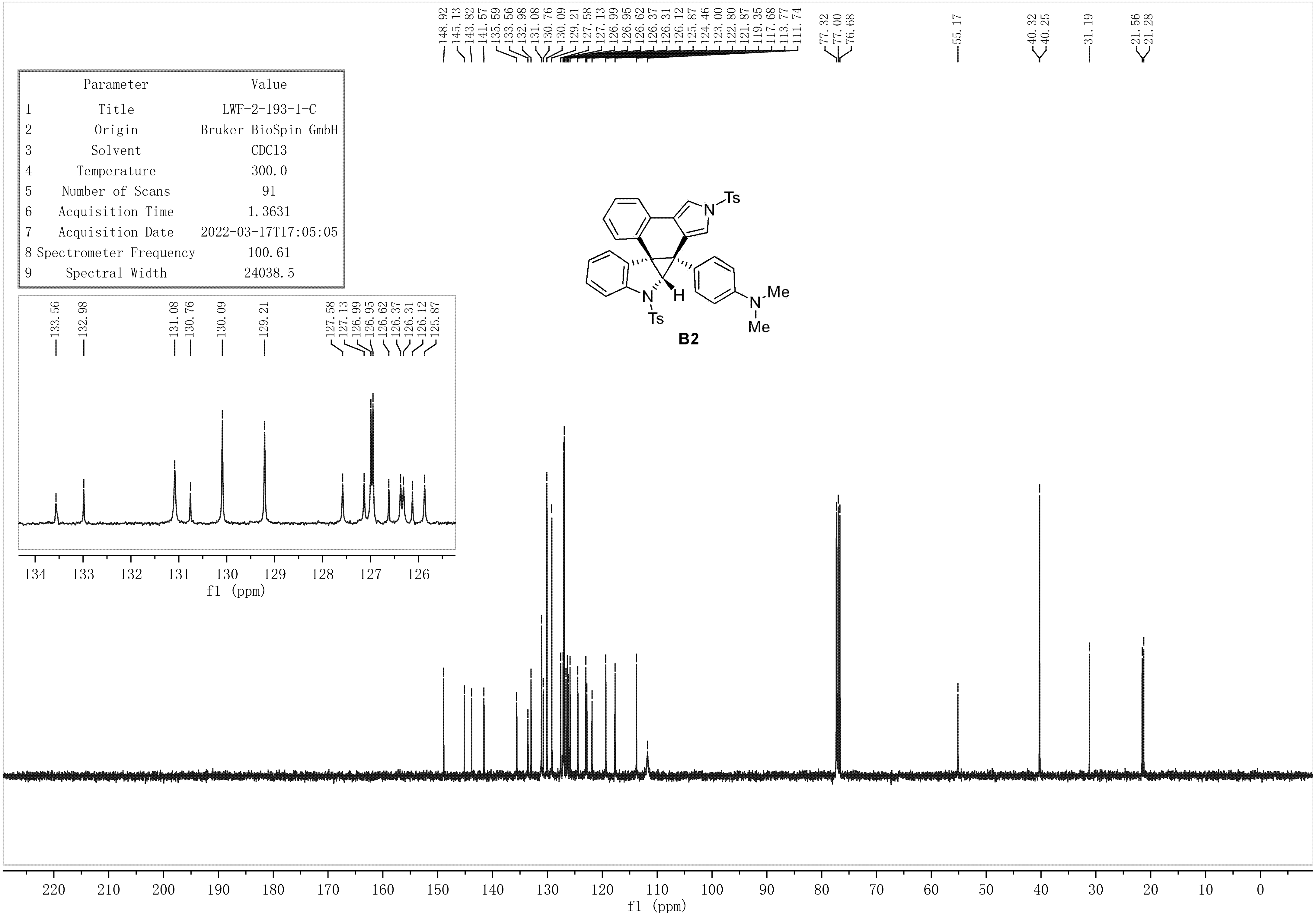

	Parameter	Value
1	Title	LWF-2-193-1-C
2	Origin	Bruker BioSpin GmbH
3	Solvent	CDCl3
4	Temperature	300.0
5	Number of Scans	91
6	Acquisition Time	1.3631
7	Acquisition Date	2022-03-17T17:05:05
8	Spectrometer Frequency	100.61
9	Spectral Width	24038.5

B2

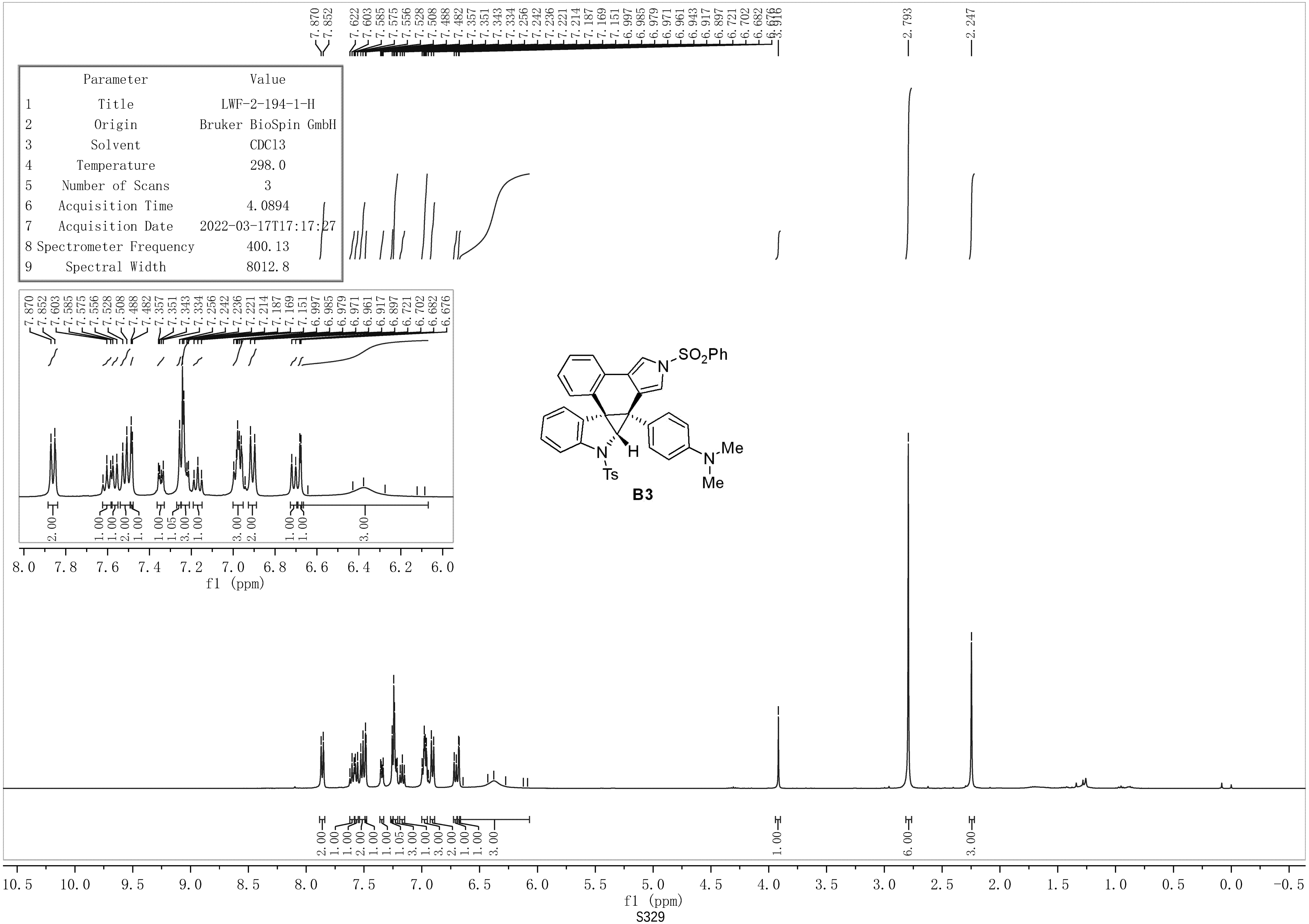

	Parameter	Value
1	Title	LWF-2-194-1-H
2	Origin	Bruker BioSpin GmbH
3	Solvent	CDCl3
4	Temperature	298.0
5	Number of Scans	3
6	Acquisition Time	4.0894
7	Acquisition Date	2022-03-17T17:17:27
8	Spectrometer Frequency	400.13
9	Spectral Width	8012.8

B3

	Parameter	Value
1	Title	LWF-2-194-1
2	Origin	
3	Solvent	CDCl3
4	Temperature	297.8
5	Number of Scans	500
6	Acquisition Time	1.0000
7	Acquisition Date	2022-03-18T01:34:42
8	Spectrometer Frequency	100.56
9	Spectral Width	26041.0

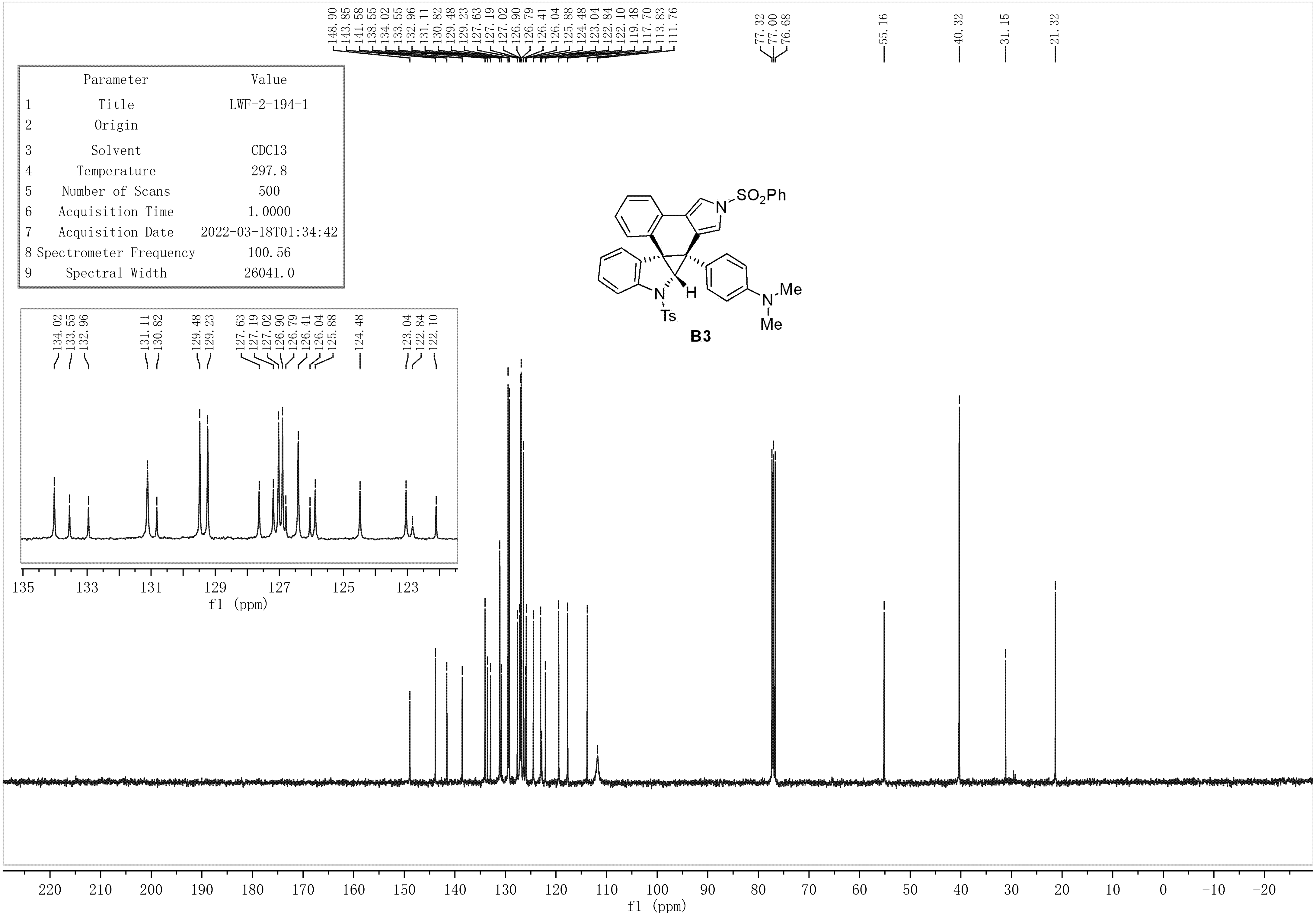

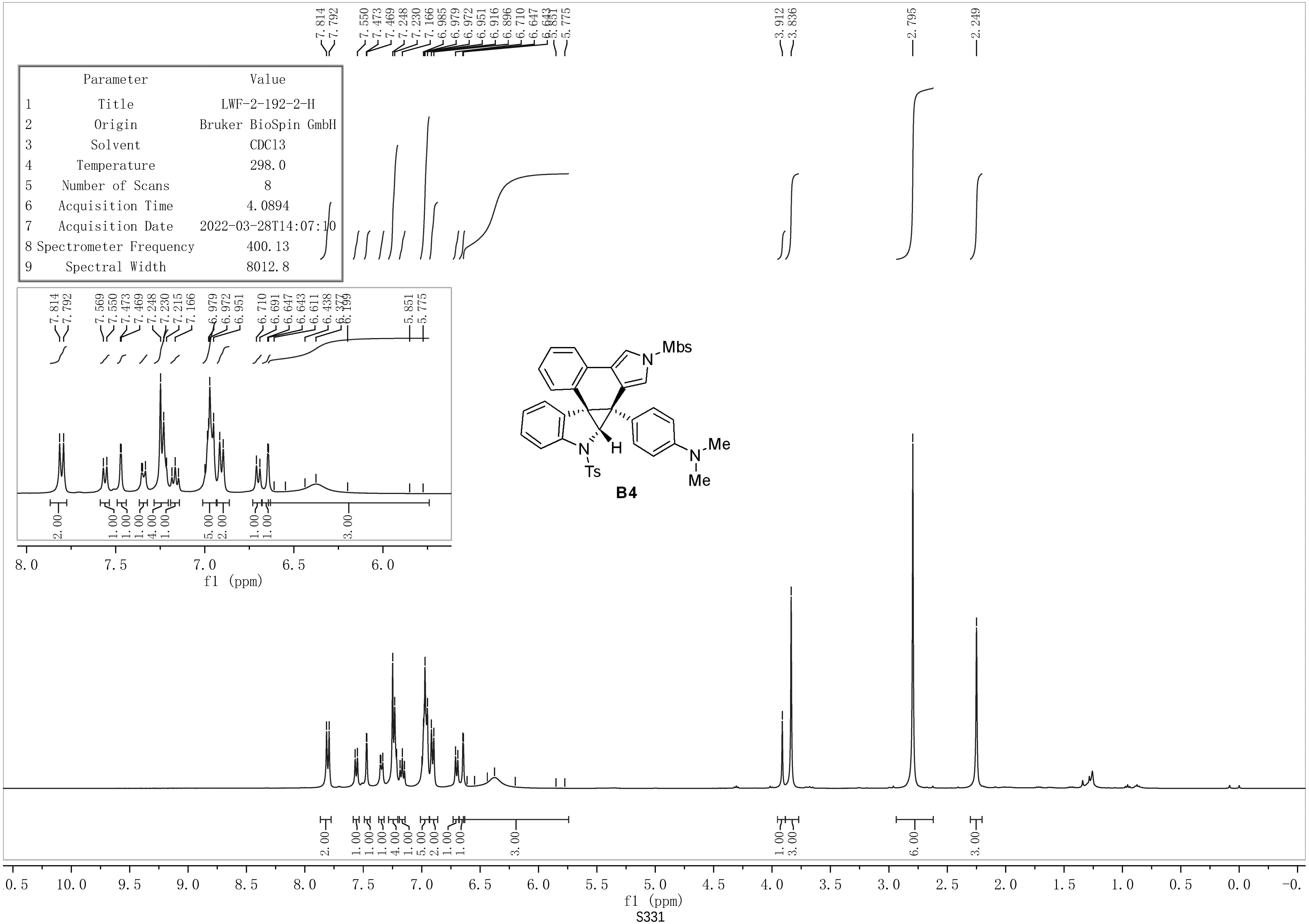

	Parameter	Value
1	Title	LWF-2-192-2-H
2	Origin	Bruker BioSpin GmbH
3	Solvent	CDCl3
4	Temperature	298.0
5	Number of Scans	8
6	Acquisition Time	4.0894
7	Acquisition Date	2022-03-28T14:07:10
8	Spectrometer Frequency	400.13
9	Spectral Width	8012.8

	Parameter	Value
1	Title	LWF-2-192-2
2	Origin	
3	Solvent	CDCl3
4	Temperature	297.1
5	Number of Scans	400
6	Acquisition Time	1.0000
7	Acquisition Date	2022-03-28T15:48:13
8	Spectrometer Frequency	100.56
9	Spectral Width	26041.0

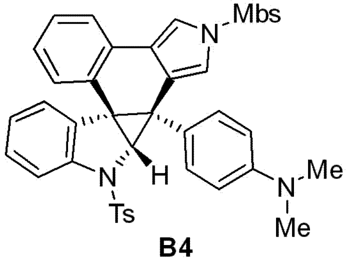

B4

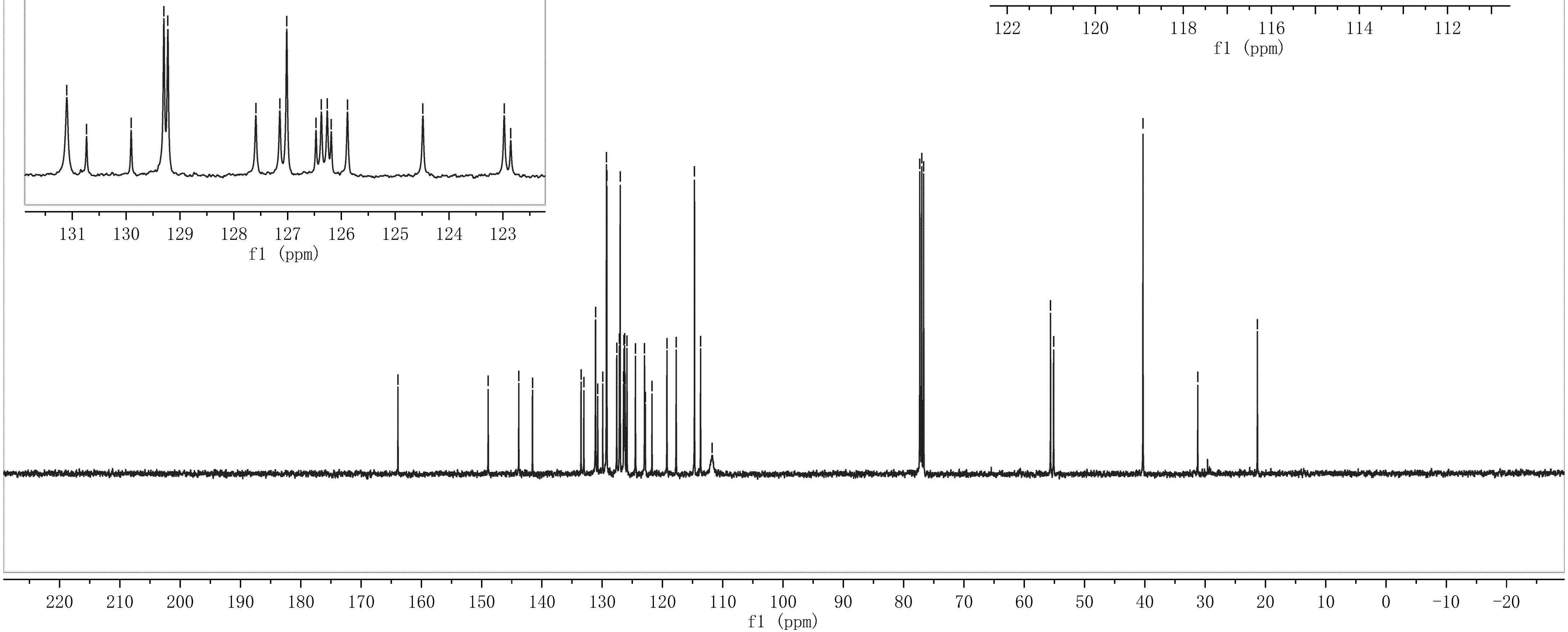

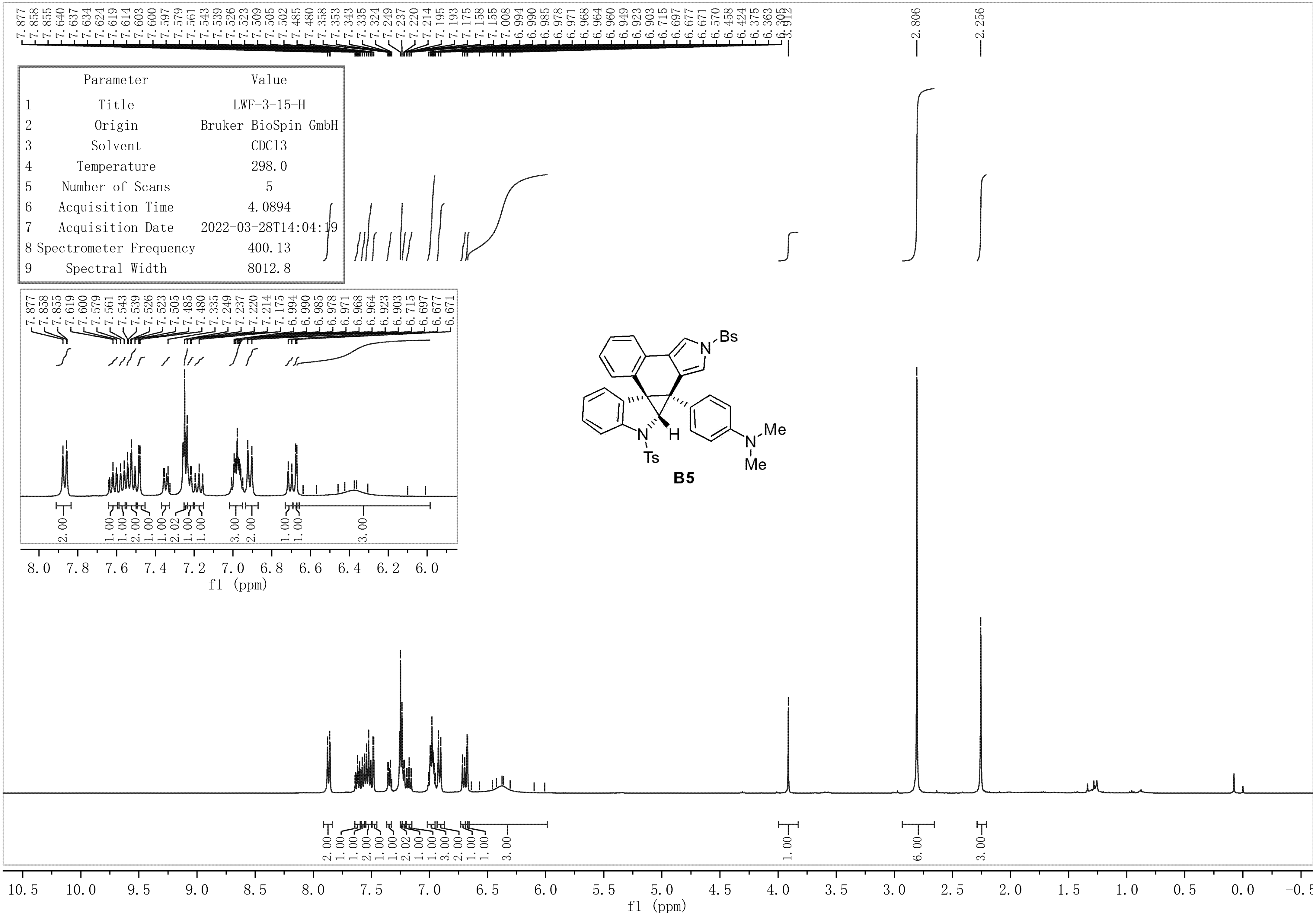

	Parameter	Value
1	Title	LWF-3-15-H
2	Origin	Bruker BioSpin GmbH
3	Solvent	CDCl3
4	Temperature	298.0
5	Number of Scans	5
6	Acquisition Time	4.0894
7	Acquisition Date	2022-03-28T14:04:19
8	Spectrometer Frequency	400.13
9	Spectral Width	8012.8

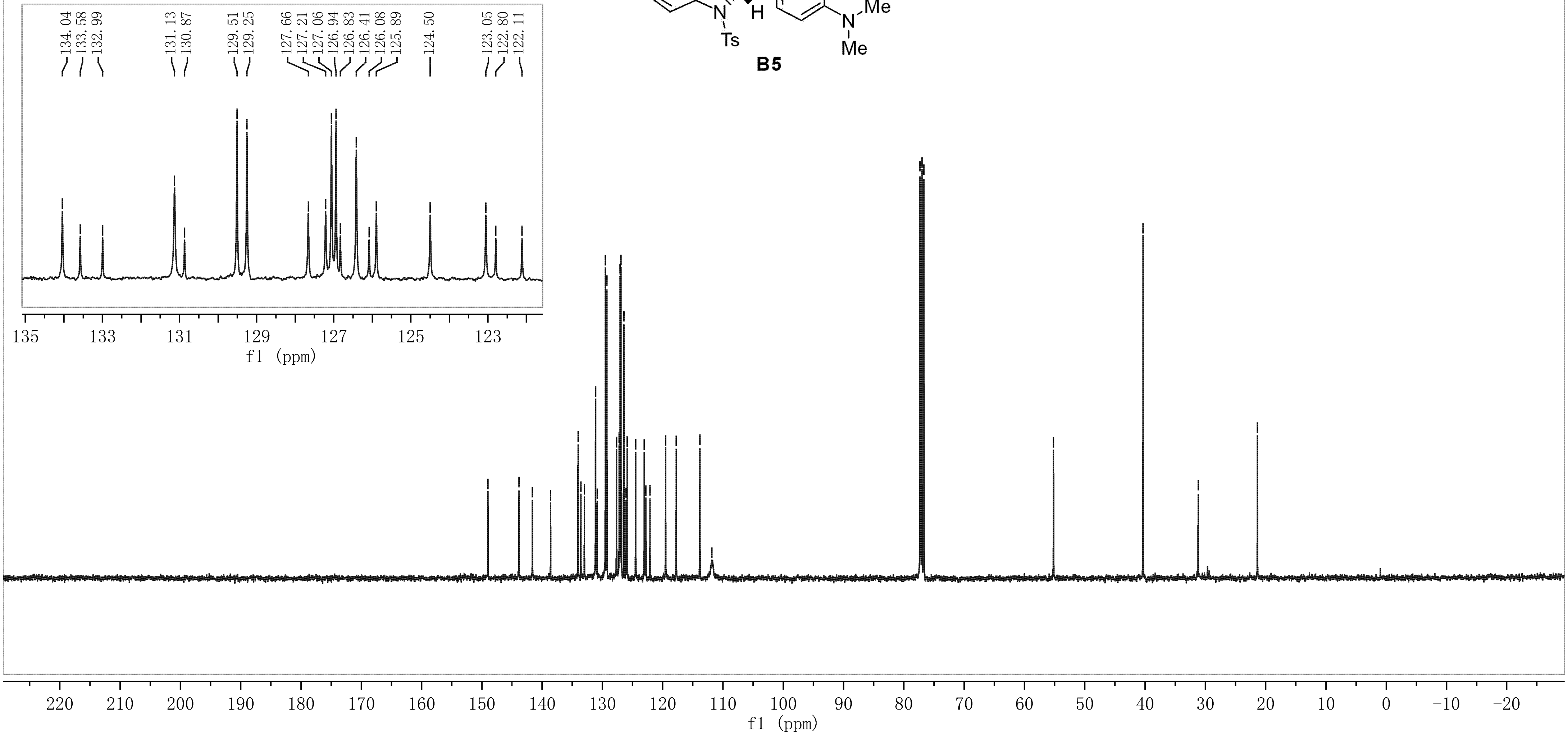

	Parameter	Value
1	Title	LWF-2-179-2
2	Origin	
3	Solvent	CDCl3
4	Temperature	297.3
5	Number of Scans	700
6	Acquisition Time	1.0000
7	Acquisition Date	2022-03-28T16:29:21
8	Spectrometer Frequency	100.56
9	Spectral Width	26041.0

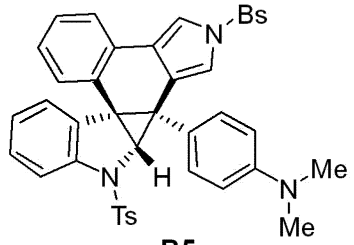

B5

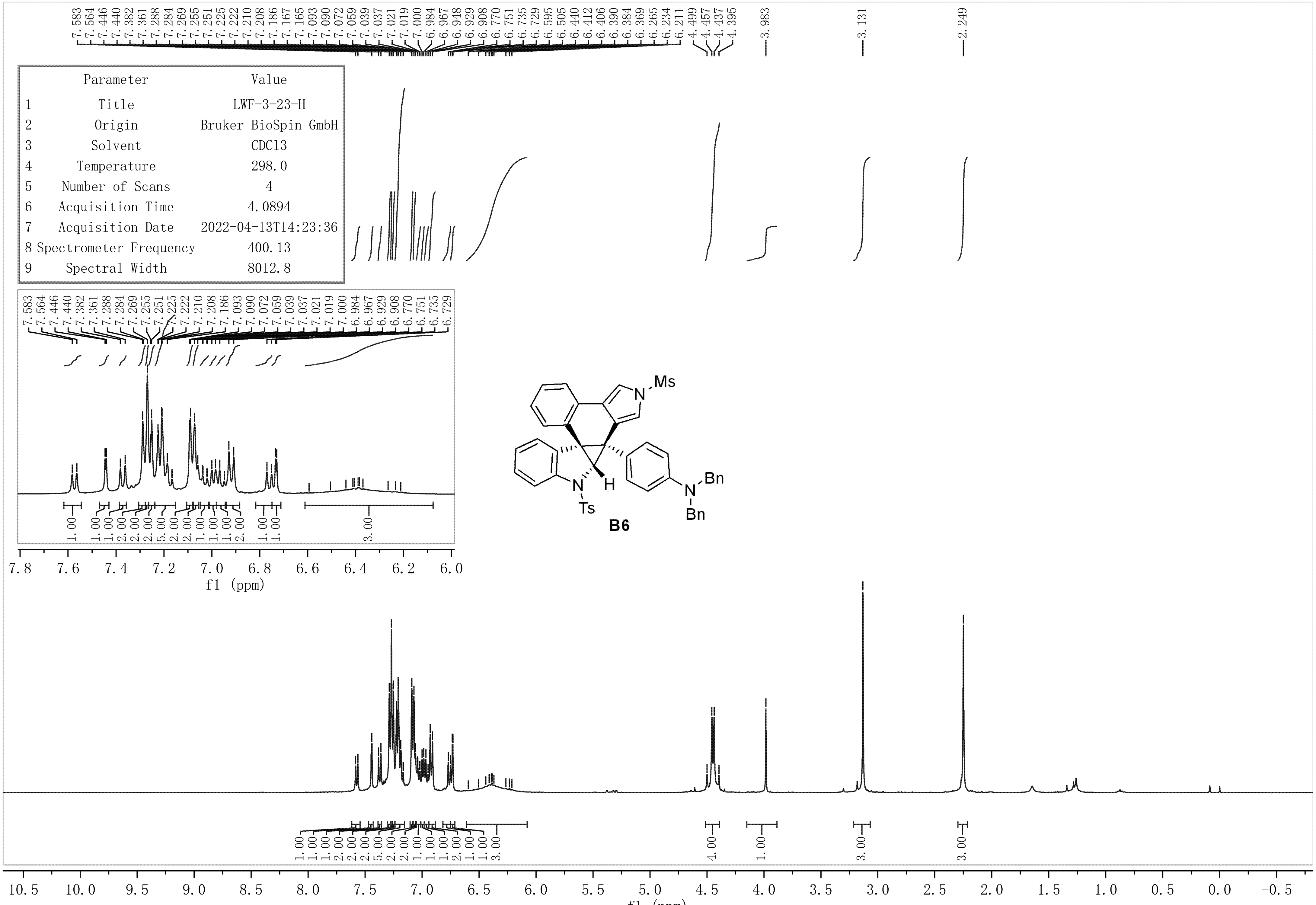
7.583 7.564 7.446 7.440 7.382 7.361 7.288 7.284 7.269 7.255 7.251 7.225 7.222 7.210 7.208 7.186 7.167 7.165 7.093 7.090 7.072 7.059 7.039 7.037 7.021 7.019 7.000 6.984 6.967 6.948 6.929 6.908 6.770 6.751 6.735 6.729 6.595 6.505 6.440 6.412 6.406 6.390 6.384 6.369 6.265 6.234 6.211 4.499 4.457 4.437 4.395 3.983 3.131 2.249
Parameter Value
1 Title LWF-3-23-H
2 Origin Bruker BioSpin GmbH
3 Solvent CDCl3
4 Temperature 298.0
5 Number of Scans 4
6 Acquisition Time 4.0894
7 Acquisition Date 2022-04-13T14:23:36
8 Spectrometer Frequency 400.13
9 Spectral Width 8012.8
Ms
N
N
Ts
H
N
Bn
Bn
B6
f1 (ppm)

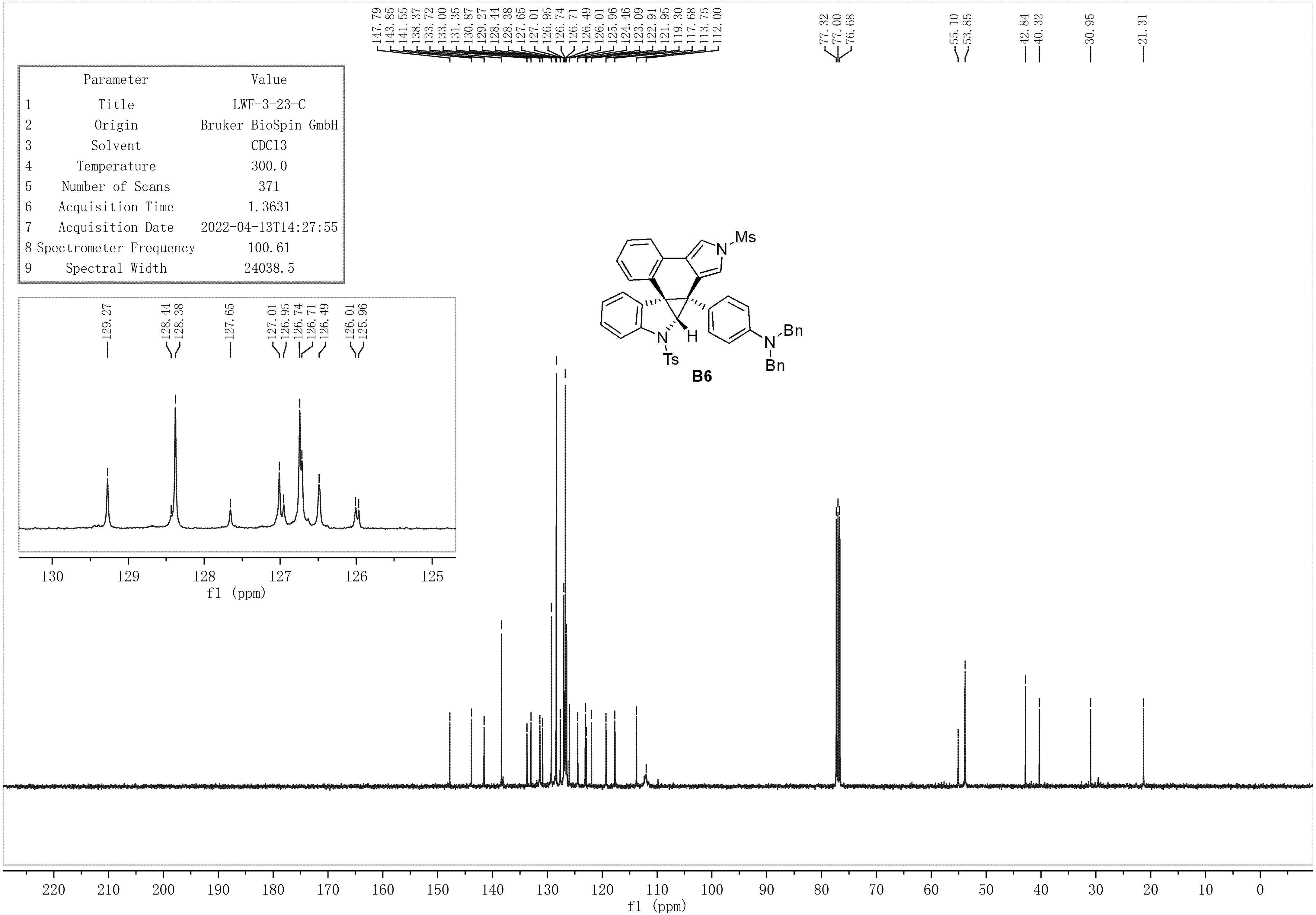
147.79 143.85 141.55 138.37 133.72 133.00 131.35 130.87 129.27 128.44 128.38 127.65 127.01 126.95 126.74 126.71 126.49 126.01 125.96 124.46 123.09 122.91 121.95 119.30 117.68 113.75 112.00
77.32 77.00 76.68
55.10 53.85
42.84 40.32
30.95
21.31
Parameter Value
1 Title LWF-3-23-C
2 Origin Bruker BioSpin GmbH
3 Solvent CDCl3
4 Temperature 300.0
5 Number of Scans 371
6 Acquisition Time 1.3631
7 Acquisition Date 2022-04-13T14:27:55
8 Spectrometer Frequency 100.61
9 Spectral Width 24038.5
129.27 128.44 128.38 127.65 127.01 126.95 126.74 126.71 126.49 126.01 125.96
130 129 128 127 126 125
f1 (ppm)
Ms
N
N
Ts
H
N
Bn
Bn
B6
220 210 200 190 180 170 160 150 140 130 120 110 100 90 80 70 60 50 40 30 20 10 0
f1 (ppm)

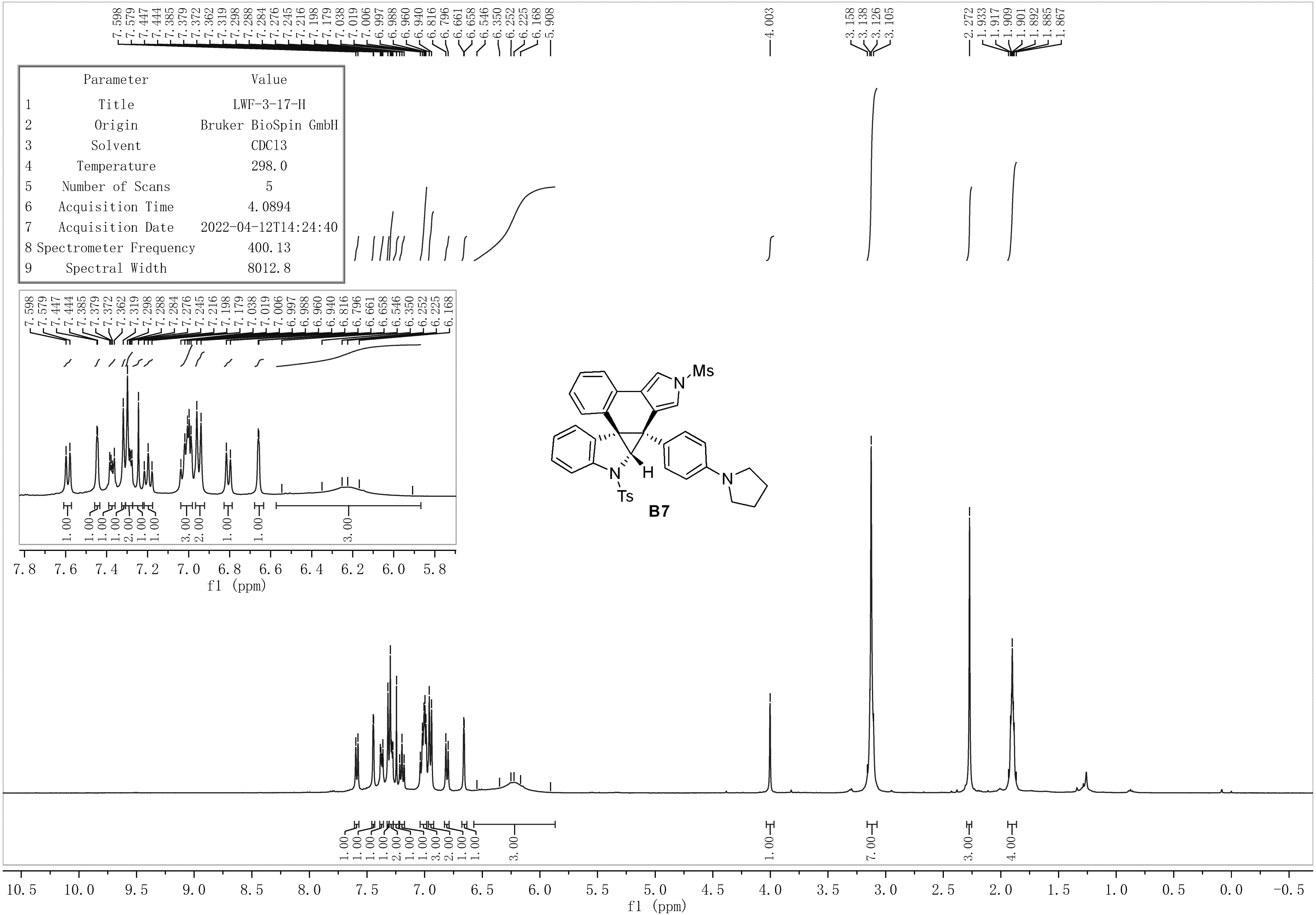

Parameter Value
1 Title LWF-3-17-H
2 Origin Bruker BioSpin GmbH
3 Solvent CDCl3
4 Temperature 298.0
5 Number of Scans 5
6 Acquisition Time 4.0894
7 Acquisition Date 2022-04-12T14:24:40
8 Spectrometer Frequency 400.13
9 Spectral Width 8012.8
7.598 7.579 7.447 7.444 7.385 7.379 7.372 7.362 7.319 7.298 7.288 7.284 7.276 7.245 7.216 7.198 7.179 7.038 7.019 7.006 6.997 6.988 6.960 6.940 6.816 6.796 6.661 6.658 6.546 6.350 6.252 6.225 6.168 5.908
4.003
3.158 3.138 3.126 3.105
2.272 1.933 1.917 1.909 1.901 1.892 1.885 1.867
Ms
N
N
H
Ts
N
B7
1.00 1.00 1.00 1.00 2.00 1.00 1.00 3.00 2.00 1.00 1.00 3.00
1.00
7.00
3.00
4.00
f1 (ppm)

	Parameter	Value
1	Title	LWF-3-17-C
2	Origin	Bruker BioSpin GmbH
3	Solvent	CDCl3
4	Temperature	300.0
5	Number of Scans	302
6	Acquisition Time	1.3631
7	Acquisition Date	2022-04-12T15:32:41
8	Spectrometer Frequency	100.61
9	Spectral Width	24038.5

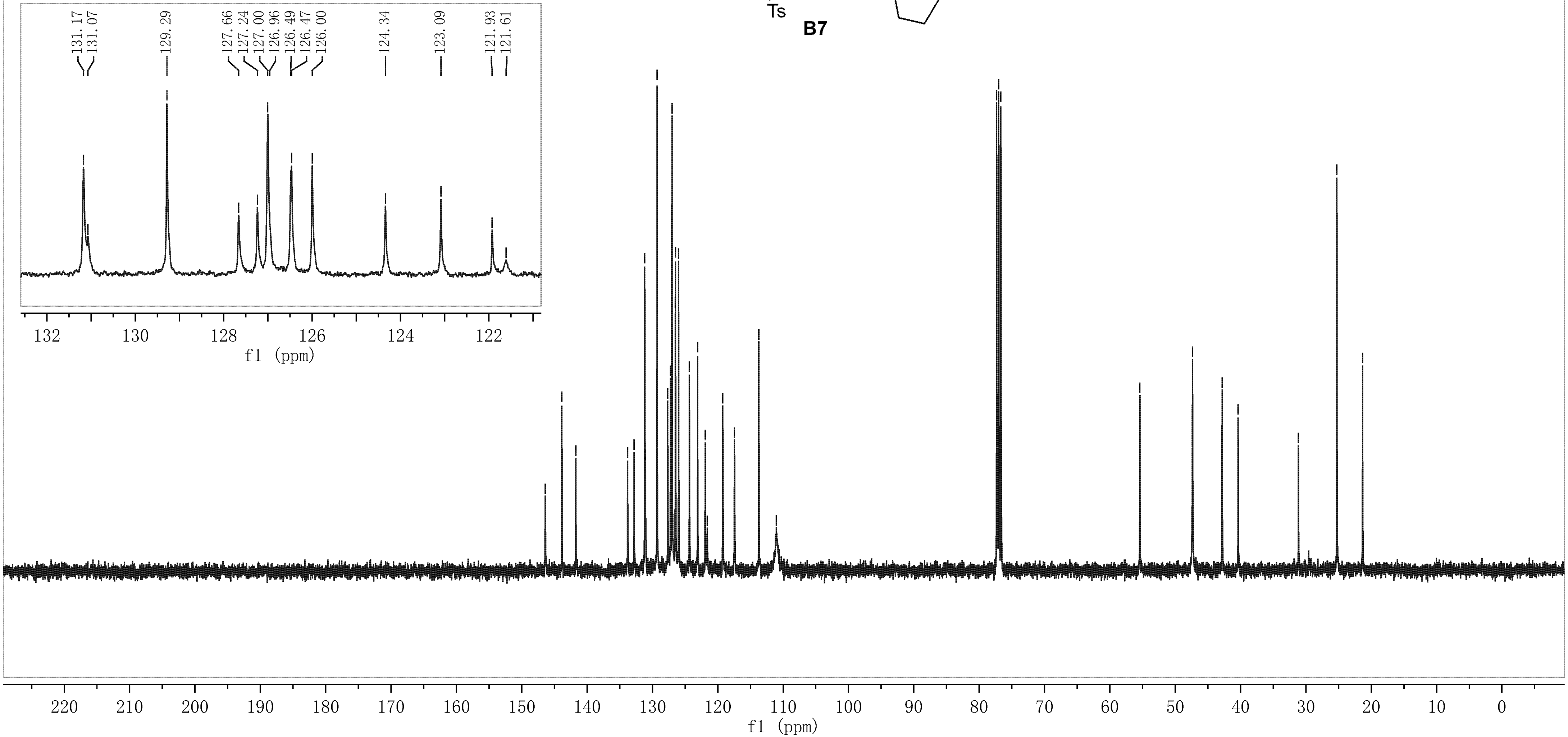

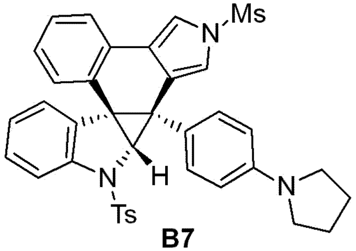

B7

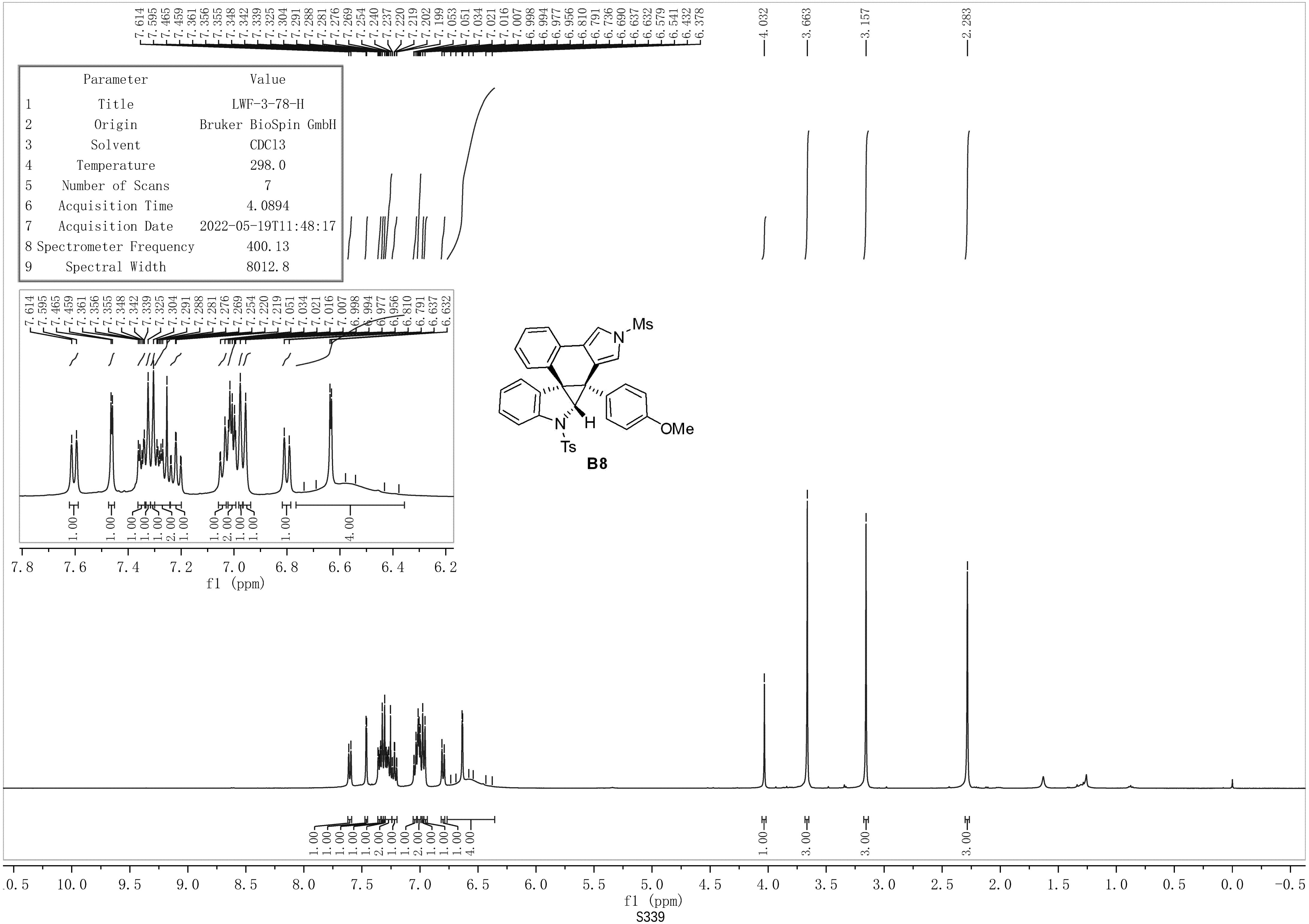

	Parameter	Value
1	Title	LWF-3-78-H
2	Origin	Bruker BioSpin GmbH
3	Solvent	CDCl3
4	Temperature	298.0
5	Number of Scans	7
6	Acquisition Time	4.0894
7	Acquisition Date	2022-05-19T11:48:17
8	Spectrometer Frequency	400.13
9	Spectral Width	8012.8

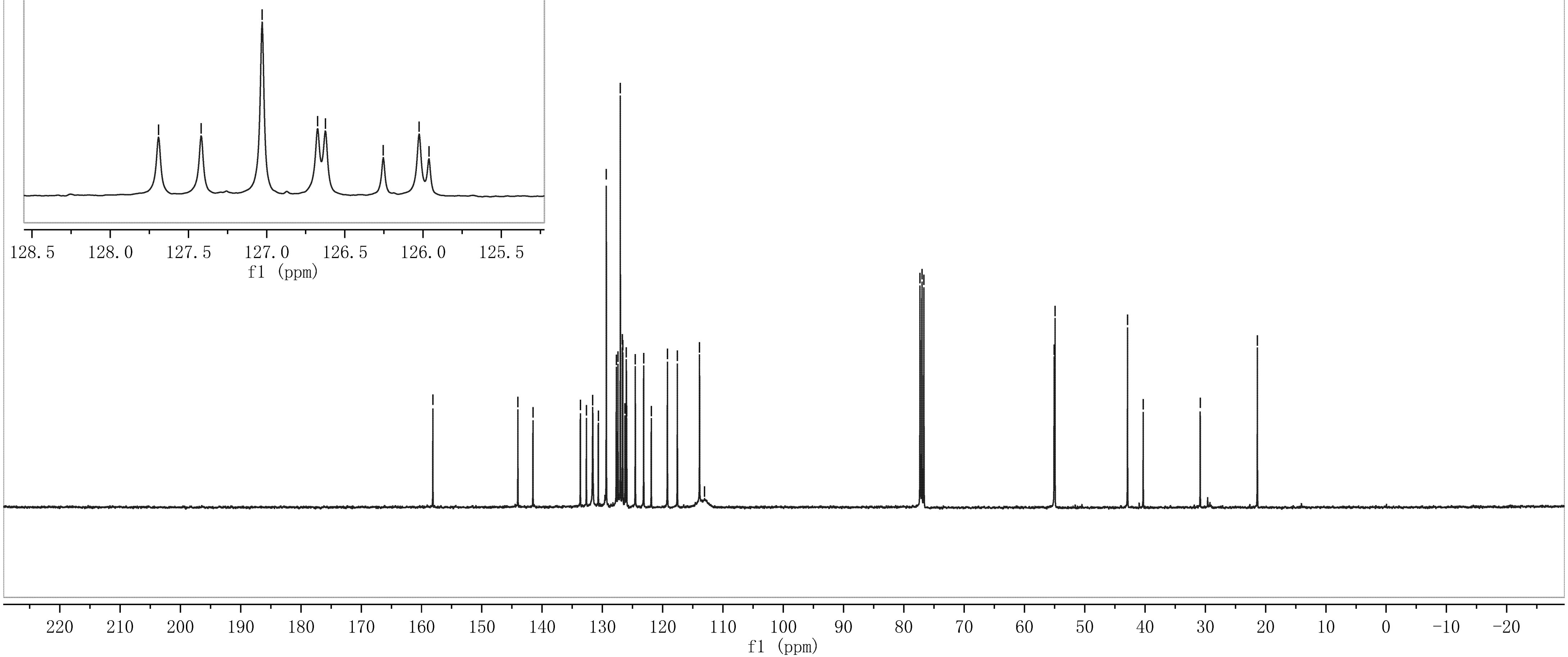

	Parameter	Value
1	Title	LWF-3-78-C
2	Origin	
3	Solvent	CDCl3
4	Temperature	299.5
5	Number of Scans	3000
6	Acquisition Time	1.0000
7	Acquisition Date	2022-05-20T04:23:56
8	Spectrometer Frequency	100.56
9	Spectral Width	26041.0

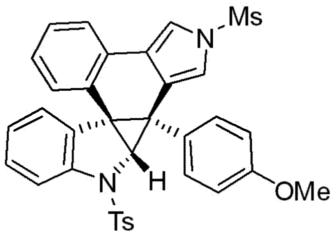

B8

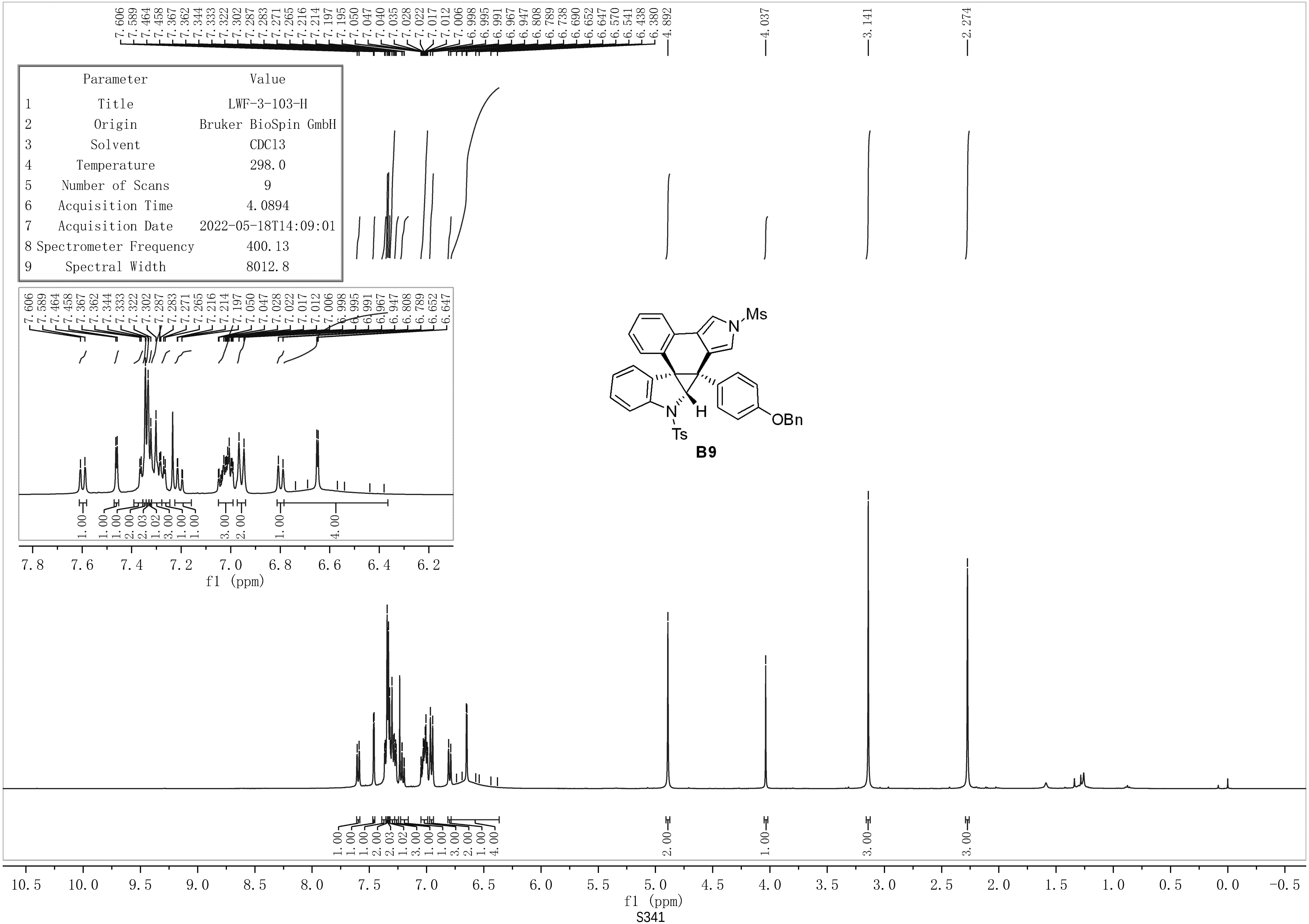

7.606 7.589 7.464 7.458 7.367 7.362 7.344 7.333 7.322 7.302 7.287 7.283 7.271 7.265 7.216 7.214 7.197 7.195 7.050 7.047 7.040 7.035 7.028 7.022 7.017 7.012 7.006 6.998 6.995 6.991 6.967 6.947 6.808 6.789 6.738 6.690 6.652 6.647 6.570 6.541 6.438 6.380
4.892
4.037
3.141
2.274
Parameter Value
1 Title LWF-3-103-H
2 Origin Bruker BioSpin GmbH
3 Solvent CDCl3
4 Temperature 298.0
5 Number of Scans 9
6 Acquisition Time 4.0894
7 Acquisition Date 2022-05-18T14:09:01
8 Spectrometer Frequency 400.13
9 Spectral Width 8012.8
7.606 7.589 7.464 7.458 7.367 7.362 7.344 7.333 7.322 7.302 7.287 7.283 7.271 7.265 7.216 7.214 7.197 7.050 7.047 7.028 7.022 7.017 7.012 7.006 6.998 6.995 6.991 6.967 6.947 6.808 6.789 6.652 6.647
1.00 1.00 1.00 2.00 2.03 1.02 3.00 1.00 1.00 3.00 2.00 1.00 4.00
7.8 7.6 7.4 7.2 7.0 6.8 6.6 6.4 6.2
f1 (ppm)
Ms
N
N
Ts
H
OBn
B9
1.00 1.00 1.00 2.00 2.03 1.02 3.00 1.00 1.00 3.00 2.00 1.00 4.00
2.00
1.00
3.00
3.00
10.5 10.0 9.5 9.0 8.5 8.0 7.5 7.0 6.5 6.0 5.5 5.0 4.5 4.0 3.5 3.0 2.5 2.0 1.5 1.0 0.5 0.0 -0.5
f1 (ppm)

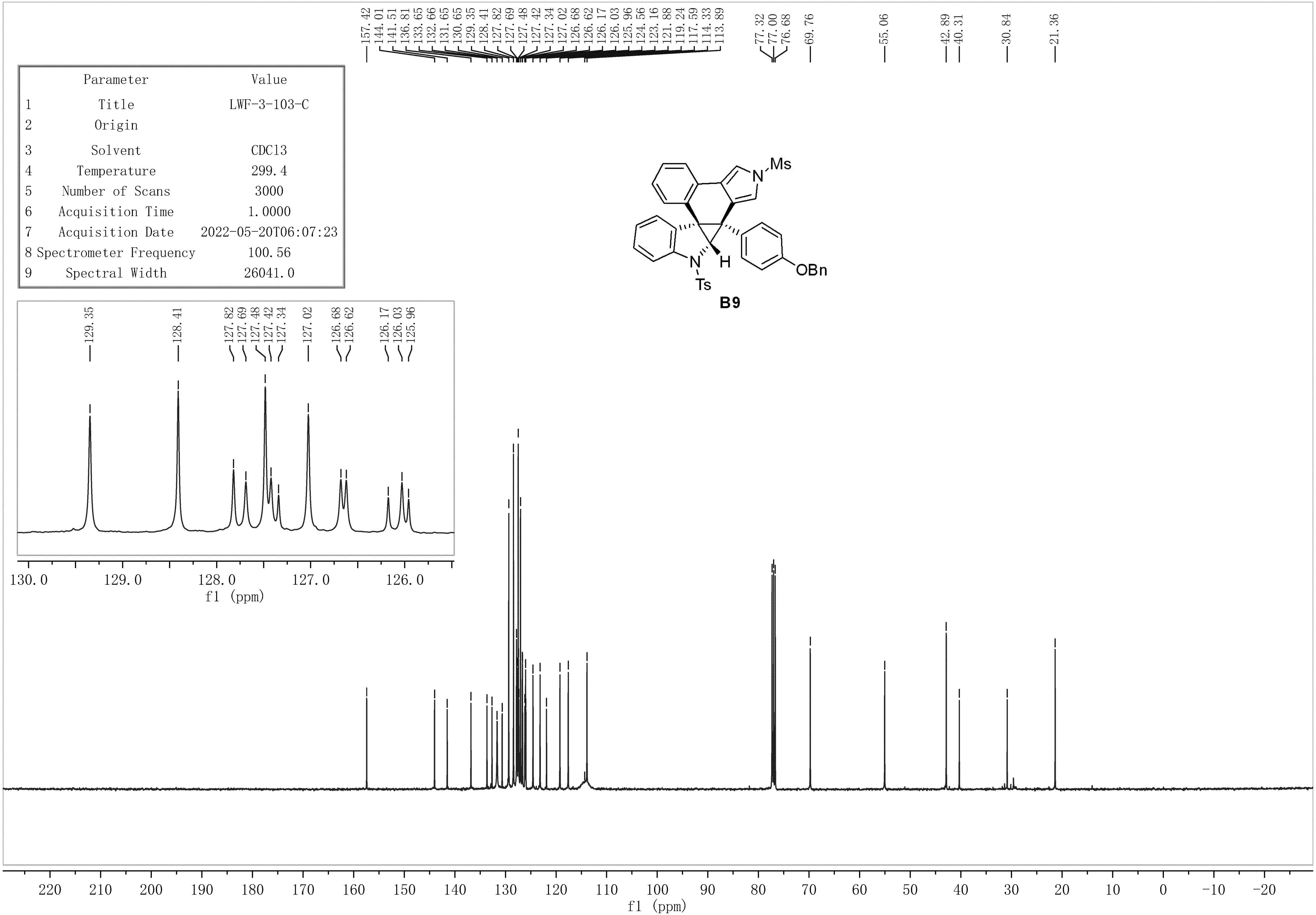

	Parameter	Value
1	Title	LWF-3-103-C
2	Origin	
3	Solvent	CDCl3
4	Temperature	299.4
5	Number of Scans	3000
6	Acquisition Time	1.0000
7	Acquisition Date	2022-05-20T06:07:23
8	Spectrometer Frequency	100.56
9	Spectral Width	26041.0

B9

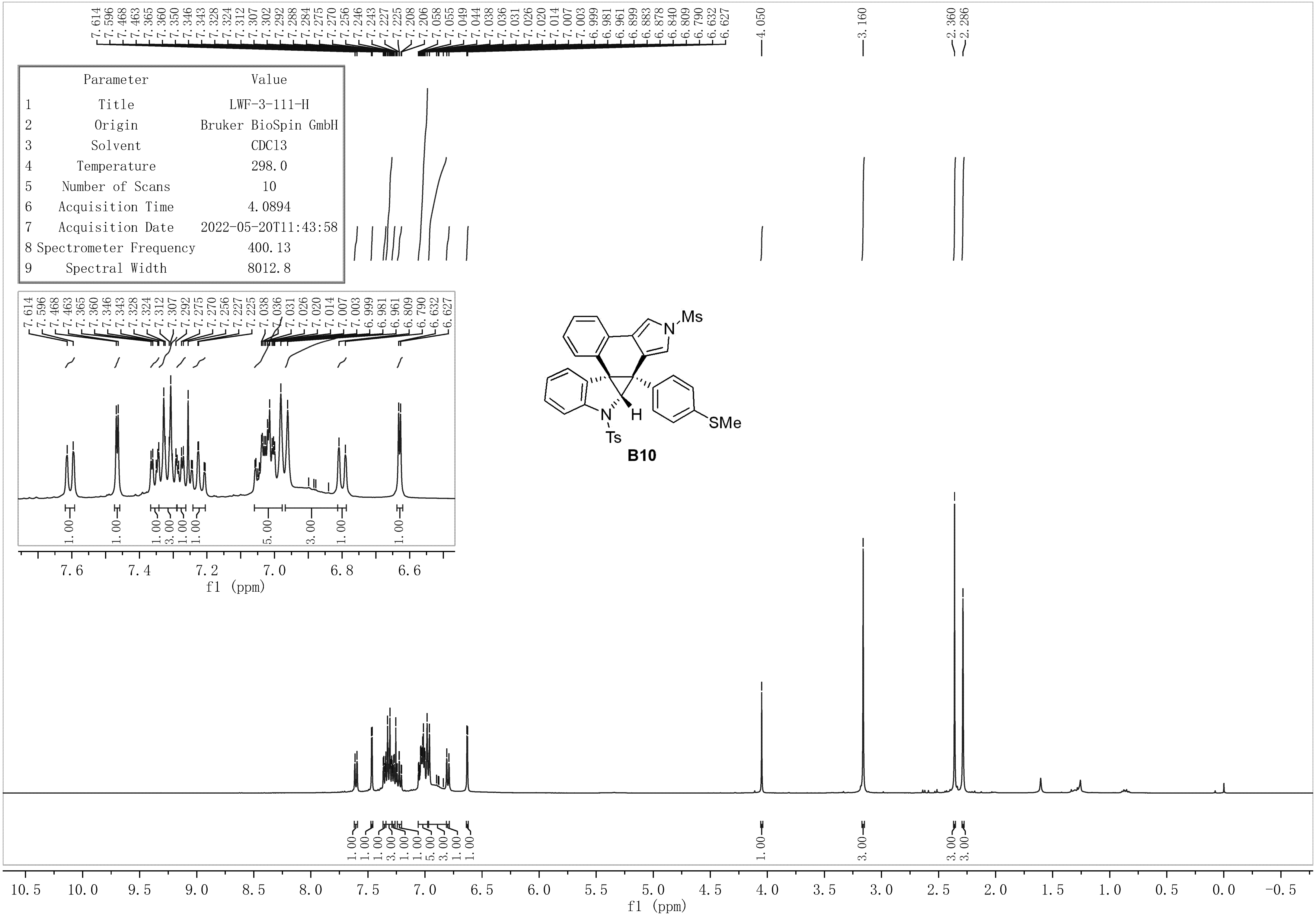

Parameter Value
1 Title LWF-3-111-H
2 Origin Bruker BioSpin GmbH
3 Solvent CDCl3
4 Temperature 298.0
5 Number of Scans 10
6 Acquisition Time 4.0894
7 Acquisition Date 2022-05-20T11:43:58
8 Spectrometer Frequency 400.13
9 Spectral Width 8012.8
7.614 7.596 7.468 7.463 7.365 7.360 7.350 7.346 7.343 7.328 7.324 7.312 7.307 7.302 7.292 7.288 7.284 7.275 7.270 7.256 7.246 7.243 7.227 7.225 7.208 7.206 7.058 7.055 7.049 7.044 7.038 7.036 7.031 7.026 7.020 7.014 7.007 7.003 6.999 6.981 6.961 6.899 6.883 6.878 6.840 6.809 6.790 6.632 6.627
4.050
3.160
2.360 2.286
Ms
N
N
Ts
H
SMe
B10
1.00 1.00 1.00 3.00 1.00 1.00 5.00 3.00 1.00 1.00
1.00
3.00
3.00 3.00
f1 (ppm)

	Parameter	Value
1	Title	LWF-3-111-C
2	Origin	Bruker BioSpin GmbH
3	Solvent	CDCl3
4	Temperature	300.0
5	Number of Scans	273
6	Acquisition Time	1.3631
7	Acquisition Date	2022-05-20T11:45:20
8	Spectrometer Frequency	100.61
9	Spectral Width	24038.5

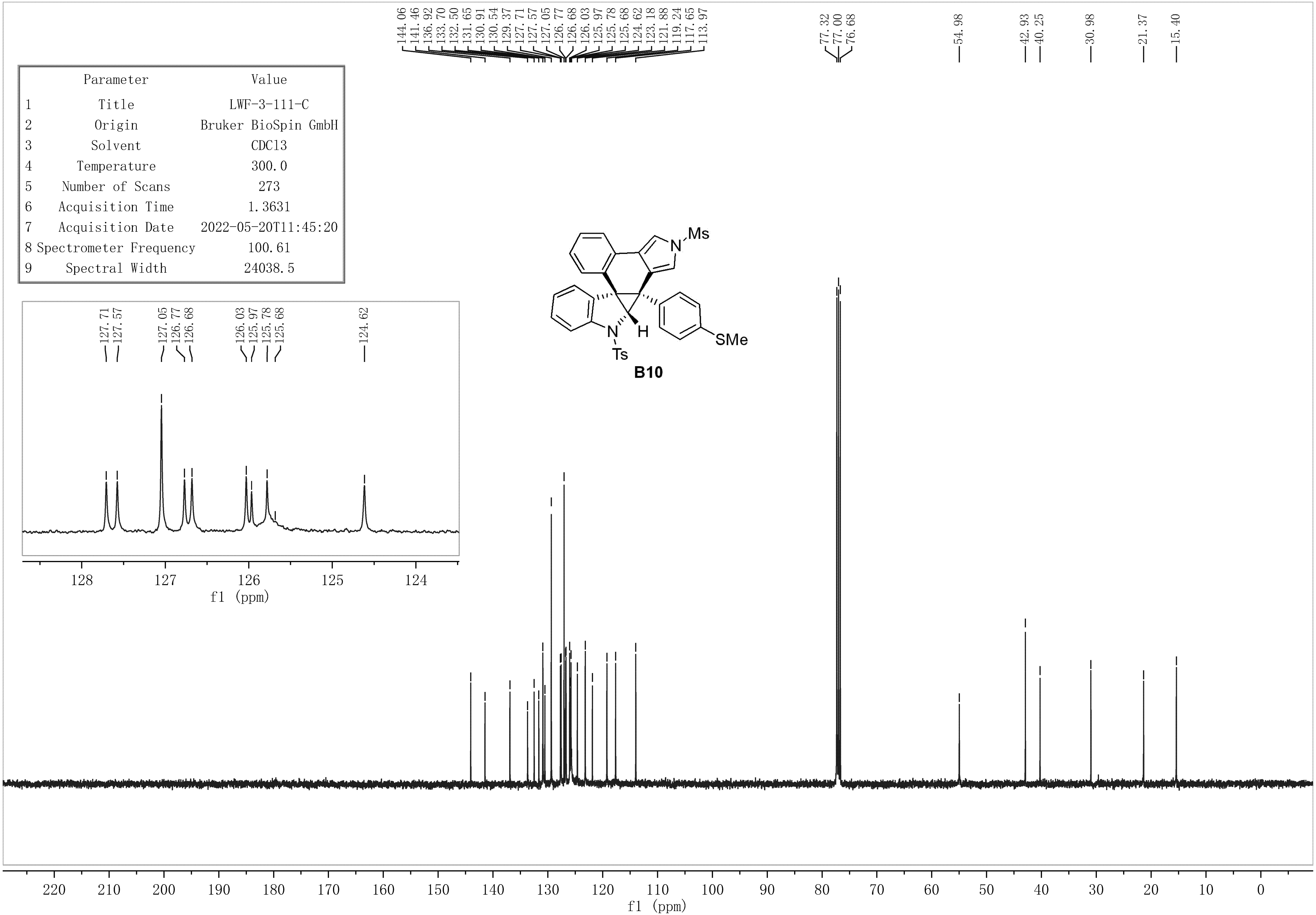

B10

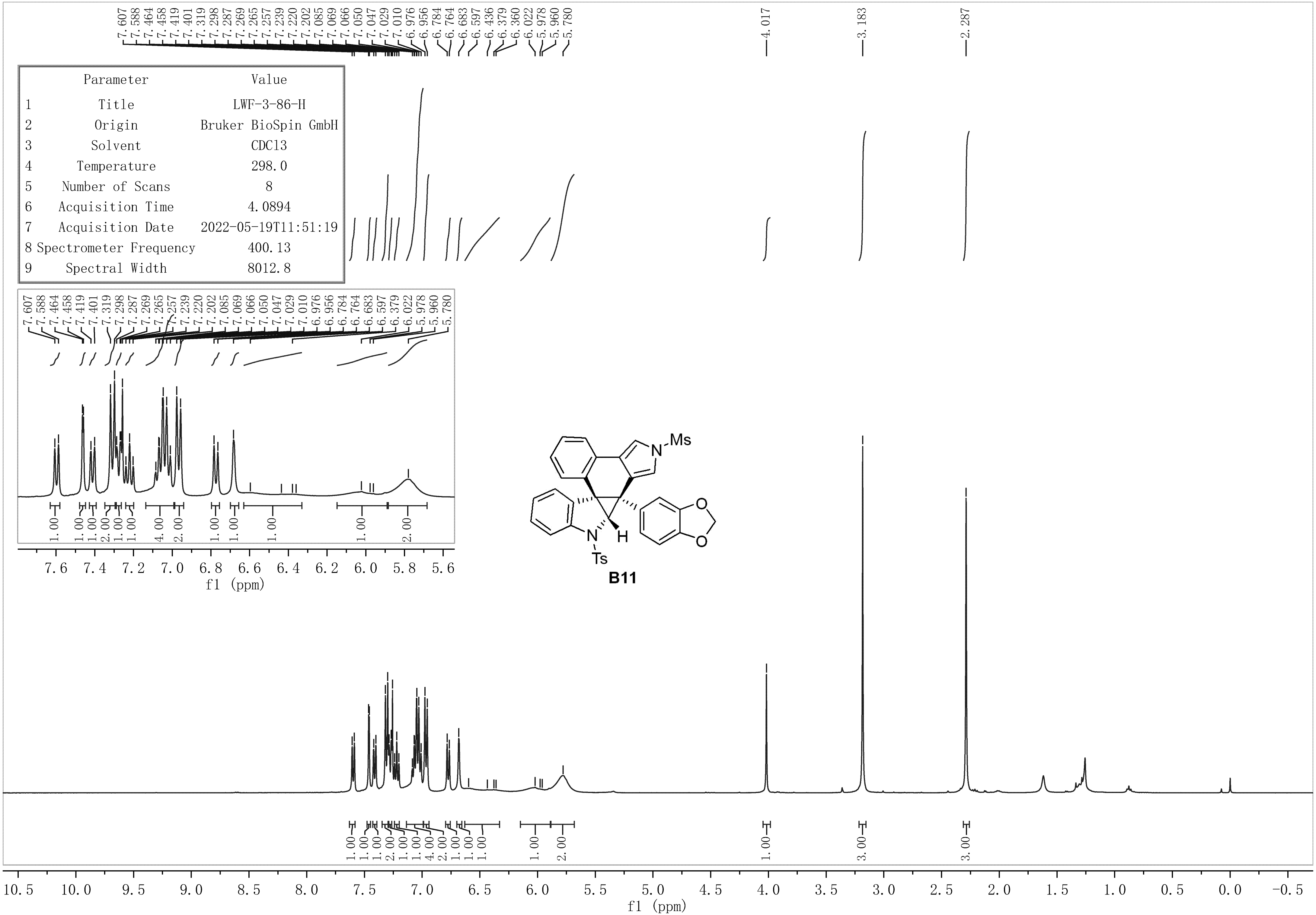

Parameter Value
1 Title LWF-3-86-H
2 Origin Bruker BioSpin GmbH
3 Solvent CDCl3
4 Temperature 298.0
5 Number of Scans 8
6 Acquisition Time 4.0894
7 Acquisition Date 2022-05-19T11:51:19
8 Spectrometer Frequency 400.13
9 Spectral Width 8012.8
7.607 7.588 7.464 7.458 7.419 7.401 7.319 7.298 7.287 7.269 7.265 7.257 7.239 7.220 7.202 7.085 7.069 7.066 7.050 7.047 7.029 7.010 6.976 6.956 6.784 6.764 6.683 6.597 6.436 6.379 6.360 6.022 5.978 5.960 5.780
4.017
3.183
2.287
N-Ms
N
Ts
H
O
O
B11
f1 (ppm)

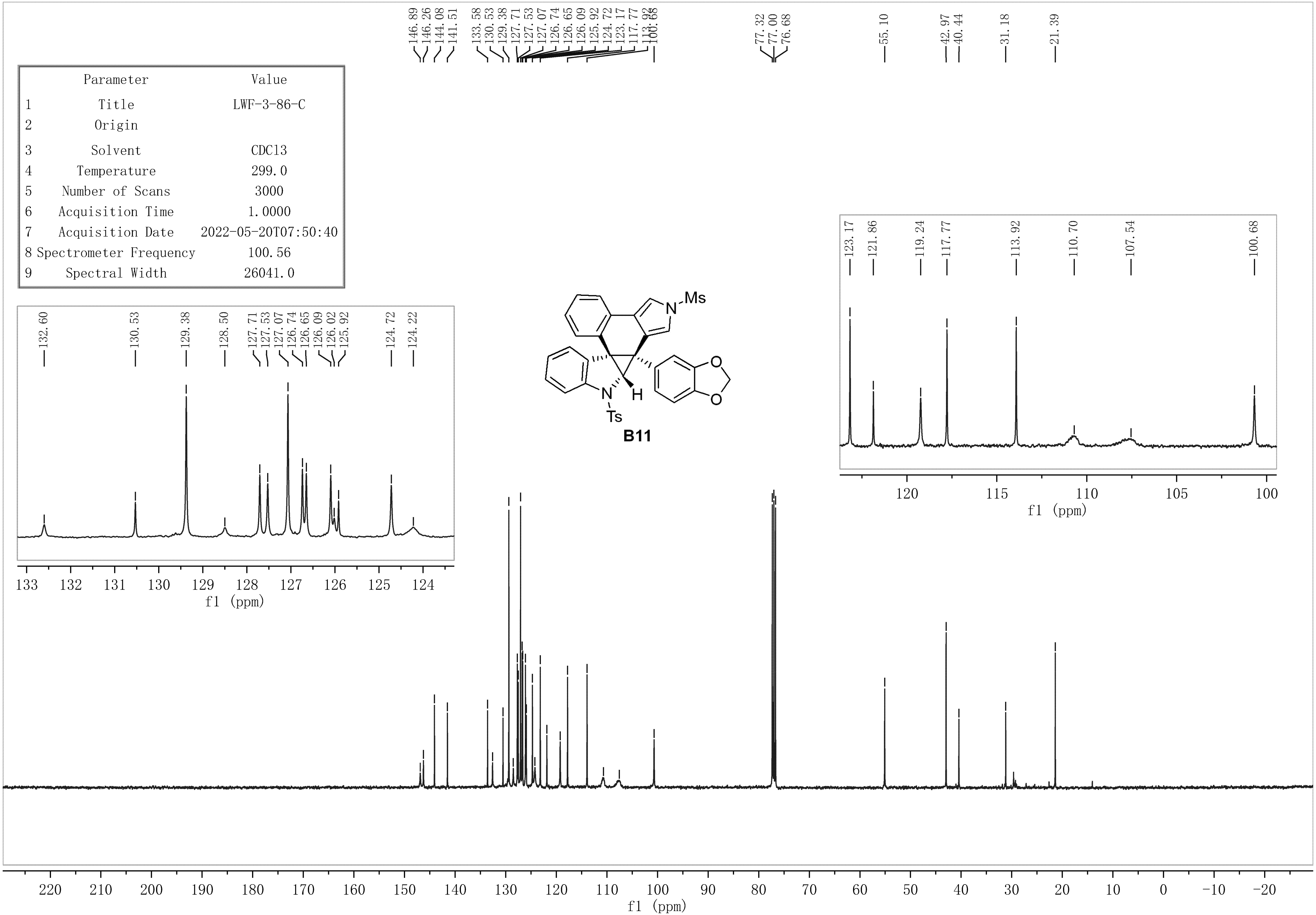

	Parameter	Value
1	Title	LWF-3-86-C
2	Origin	
3	Solvent	CDCl3
4	Temperature	299.0
5	Number of Scans	3000
6	Acquisition Time	1.0000
7	Acquisition Date	2022-05-20T07:50:40
8	Spectrometer Frequency	100.56
9	Spectral Width	26041.0

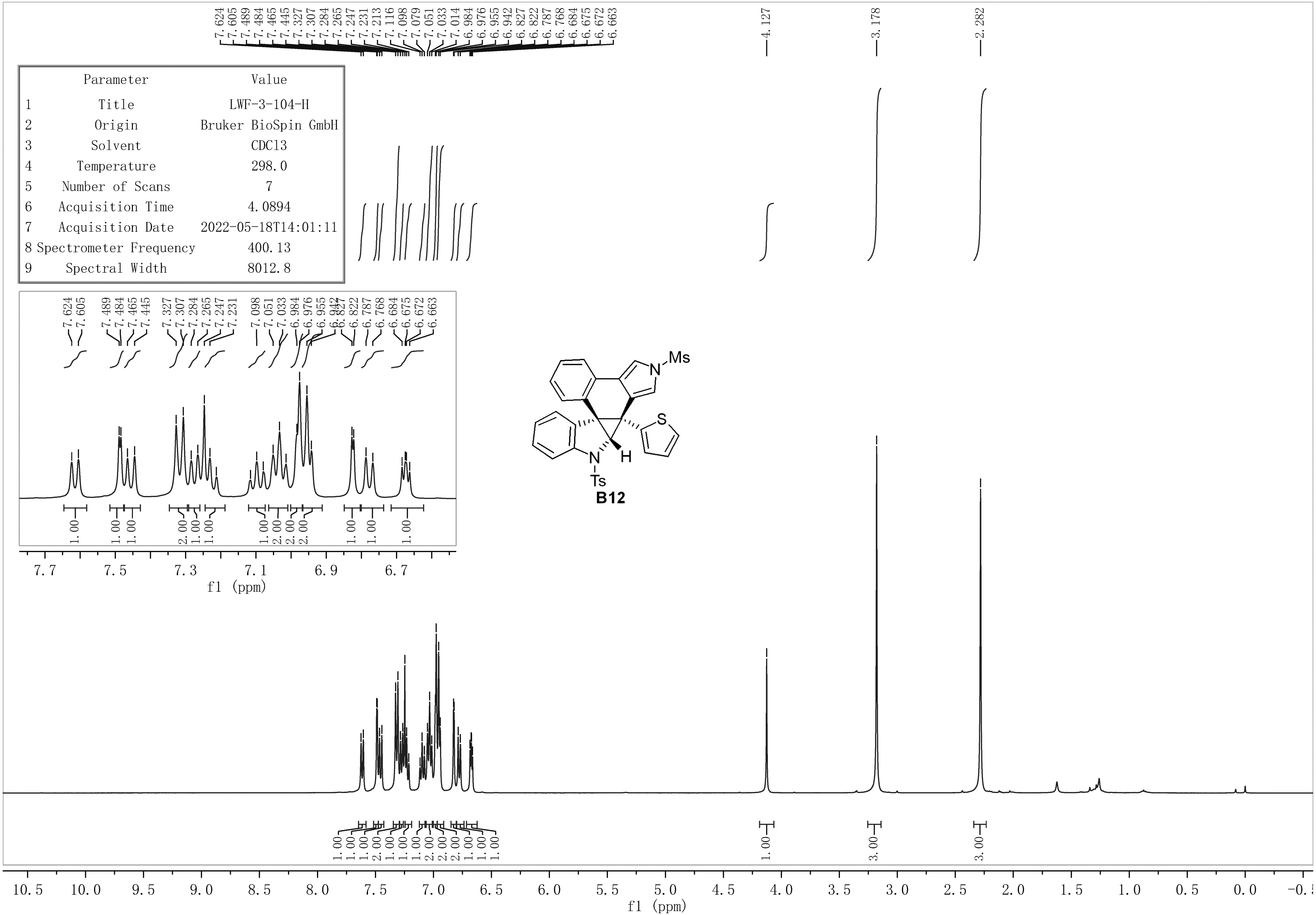

Parameter Value
1 Title LWF-3-104-H
2 Origin Bruker BioSpin GmbH
3 Solvent CDCl3
4 Temperature 298.0
5 Number of Scans 7
6 Acquisition Time 4.0894
7 Acquisition Date 2022-05-18T14:01:11
8 Spectrometer Frequency 400.13
9 Spectral Width 8012.8
7.624 7.605 7.489 7.484 7.465 7.445 7.327 7.307 7.284 7.265 7.247 7.231 7.213 7.116 7.098 7.079 7.051 7.033 7.014 6.984 6.976 6.955 6.942 6.827 6.822 6.787 6.768 6.684 6.675 6.672 6.663
4.127
3.178
2.282
N
Ms
S
H
N
Ts
B12
f1 (ppm)

	Parameter	Value
1	Title	LWF-3-104-C
2	Origin	Bruker BioSpin GmbH
3	Solvent	CDCl3
4	Temperature	300.0
5	Number of Scans	63
6	Acquisition Time	1.3631
7	Acquisition Date	2022-05-18T14:02:41
8	Spectrometer Frequency	100.61
9	Spectral Width	24038.5

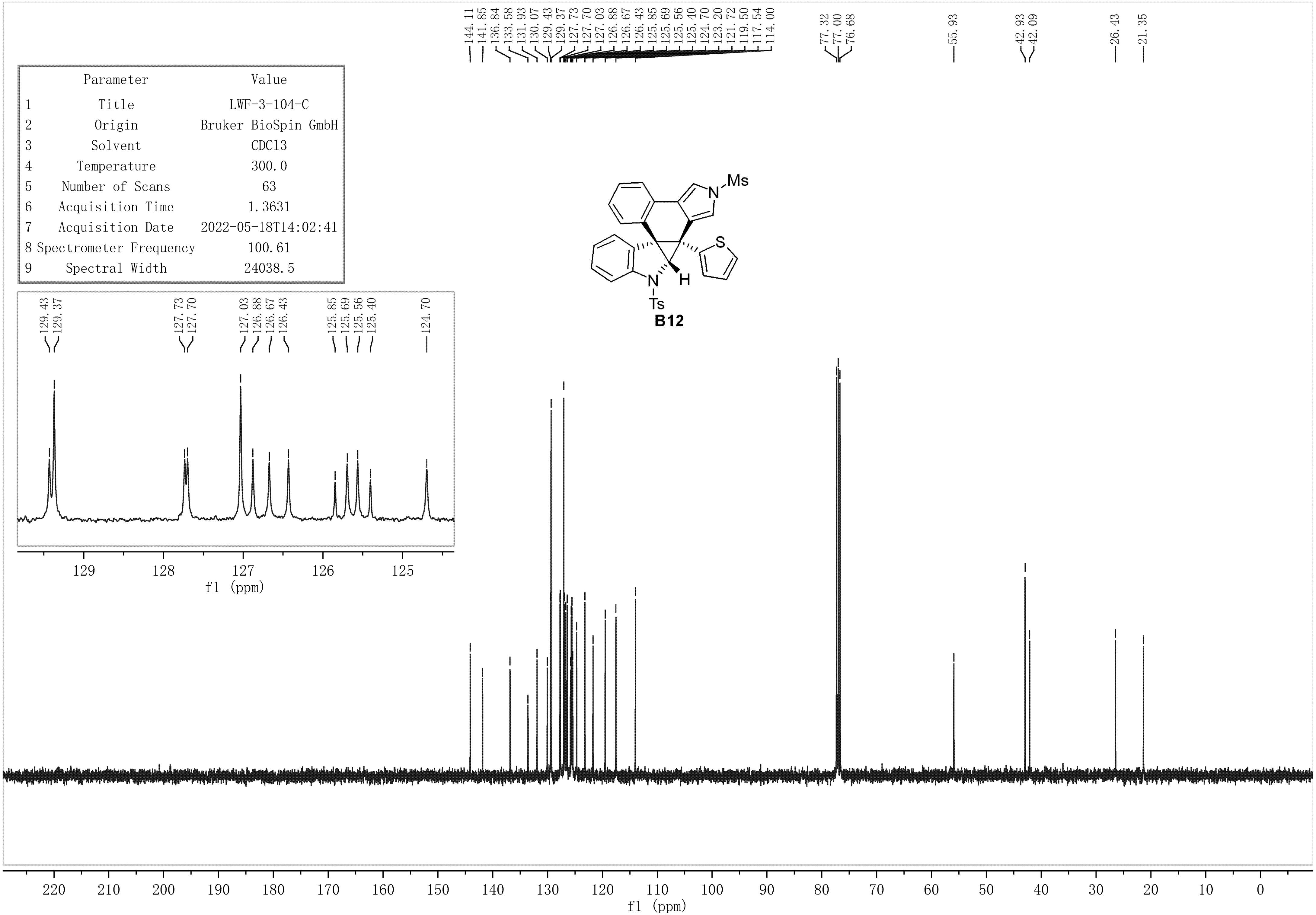

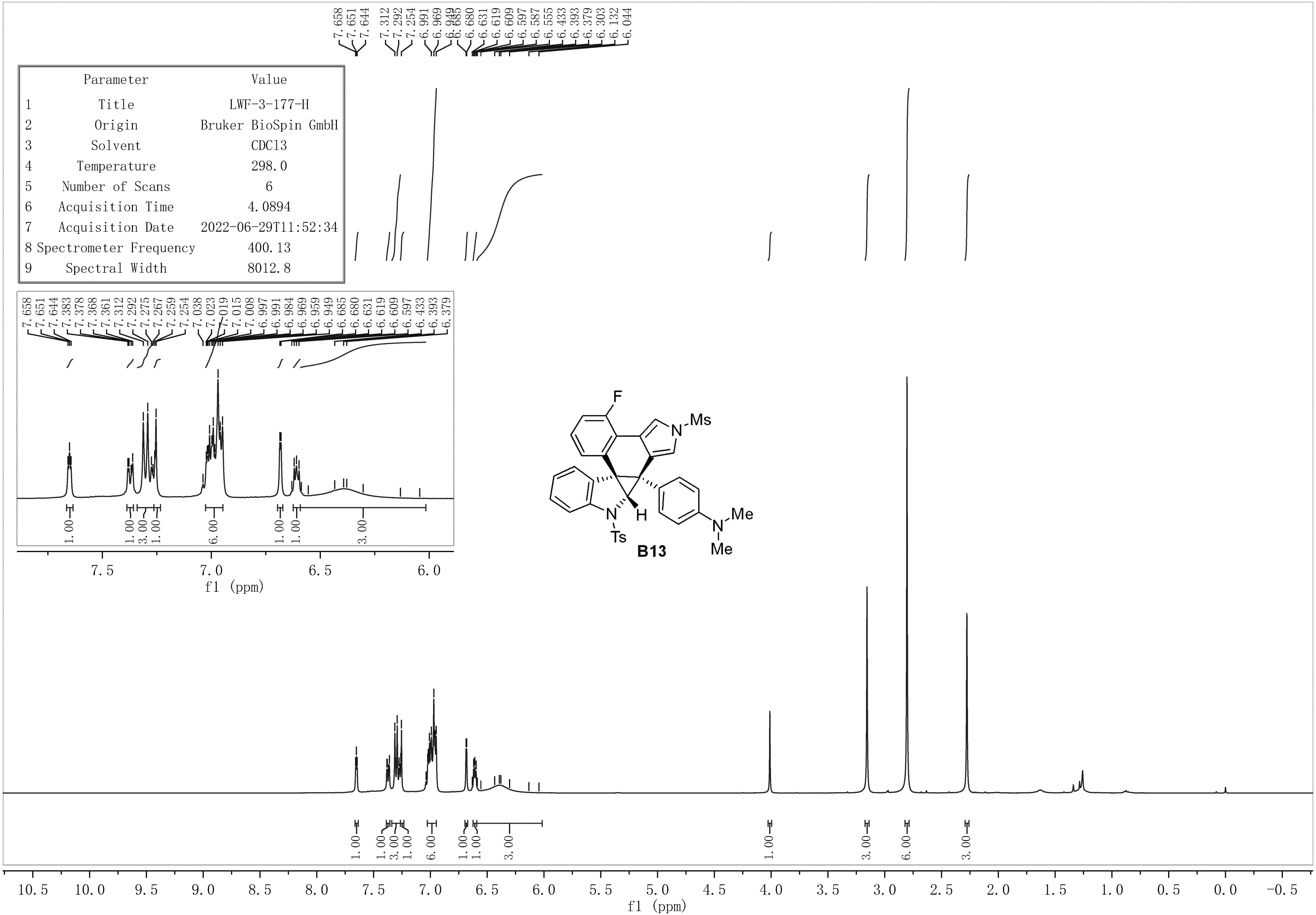

	Parameter	Value
1	Title	LWF-3-177-H
2	Origin	Bruker BioSpin GmbH
3	Solvent	CDCl3
4	Temperature	298.0
5	Number of Scans	6
6	Acquisition Time	4.0894
7	Acquisition Date	2022-06-29T11:52:34
8	Spectrometer Frequency	400.13
9	Spectral Width	8012.8

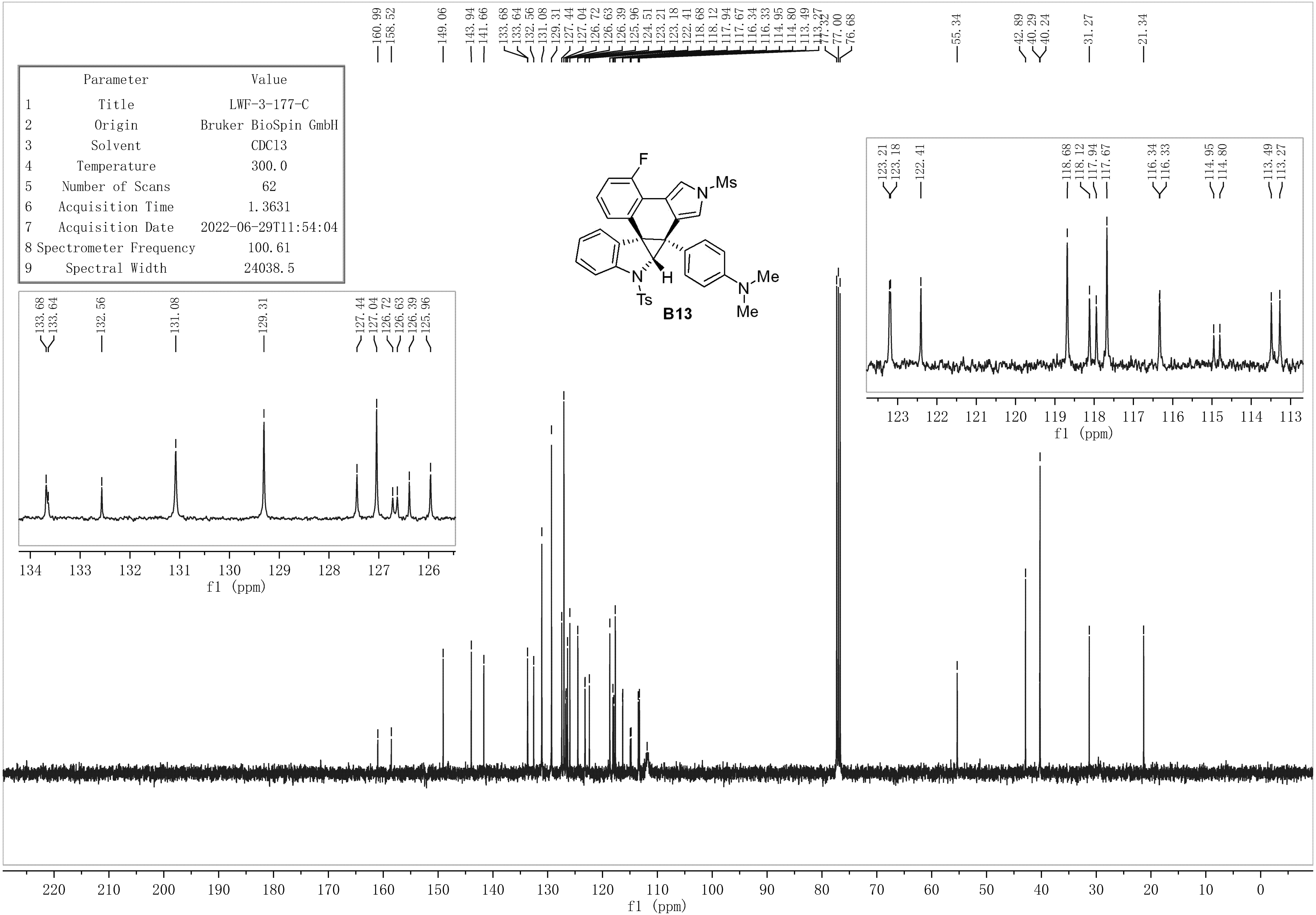

	Parameter	Value
1	Title	LWF-3-177-C
2	Origin	Bruker BioSpin GmbH
3	Solvent	CDCl3
4	Temperature	300.0
5	Number of Scans	62
6	Acquisition Time	1.3631
7	Acquisition Date	2022-06-29T11:54:04
8	Spectrometer Frequency	100.61
9	Spectral Width	24038.5

	Parameter	Value
1	Title	LWF-3-177-F
2	Origin	
3	Solvent	CDCl3
4	Temperature	299.2
5	Number of Scans	100
6	Acquisition Time	1.0000
7	Acquisition Date	2022-06-29T15:58:35
8	Spectrometer Frequency	376.30
9	Spectral Width	96153.0

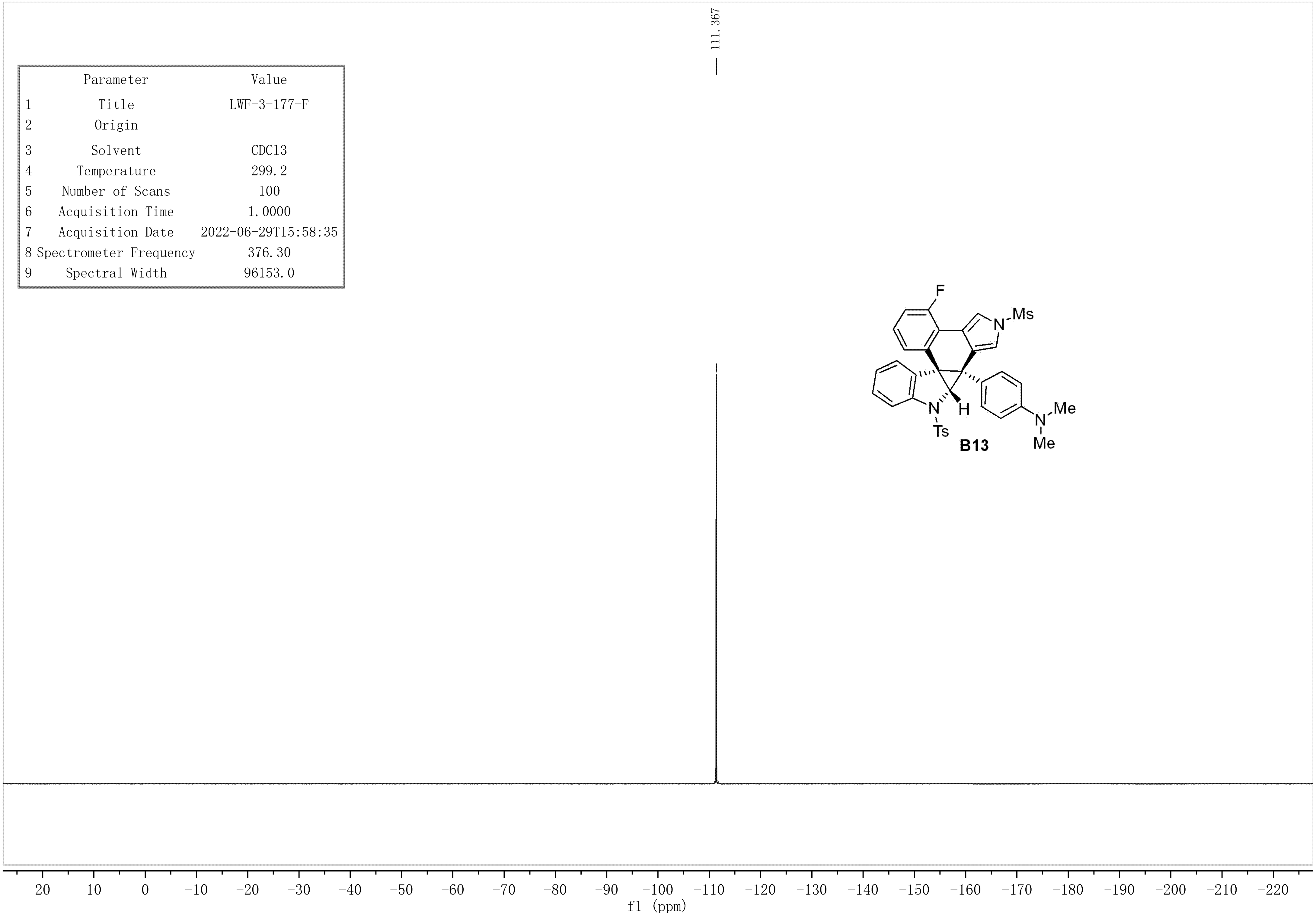

B13

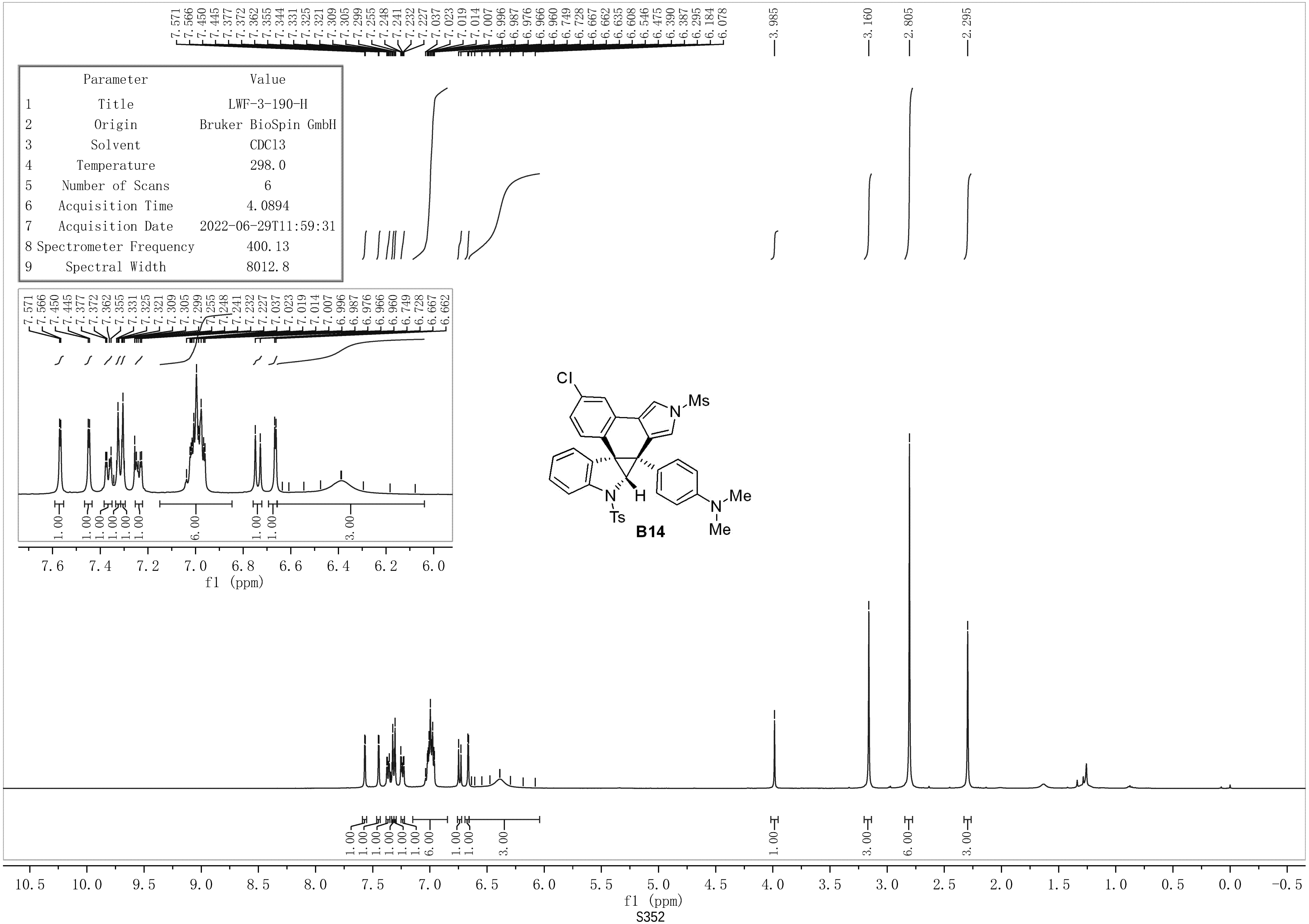

	Parameter	Value
1	Title	LWF-3-190-H
2	Origin	Bruker BioSpin GmbH
3	Solvent	CDCl3
4	Temperature	298.0
5	Number of Scans	6
6	Acquisition Time	4.0894
7	Acquisition Date	2022-06-29T11:59:31
8	Spectrometer Frequency	400.13
9	Spectral Width	8012.8

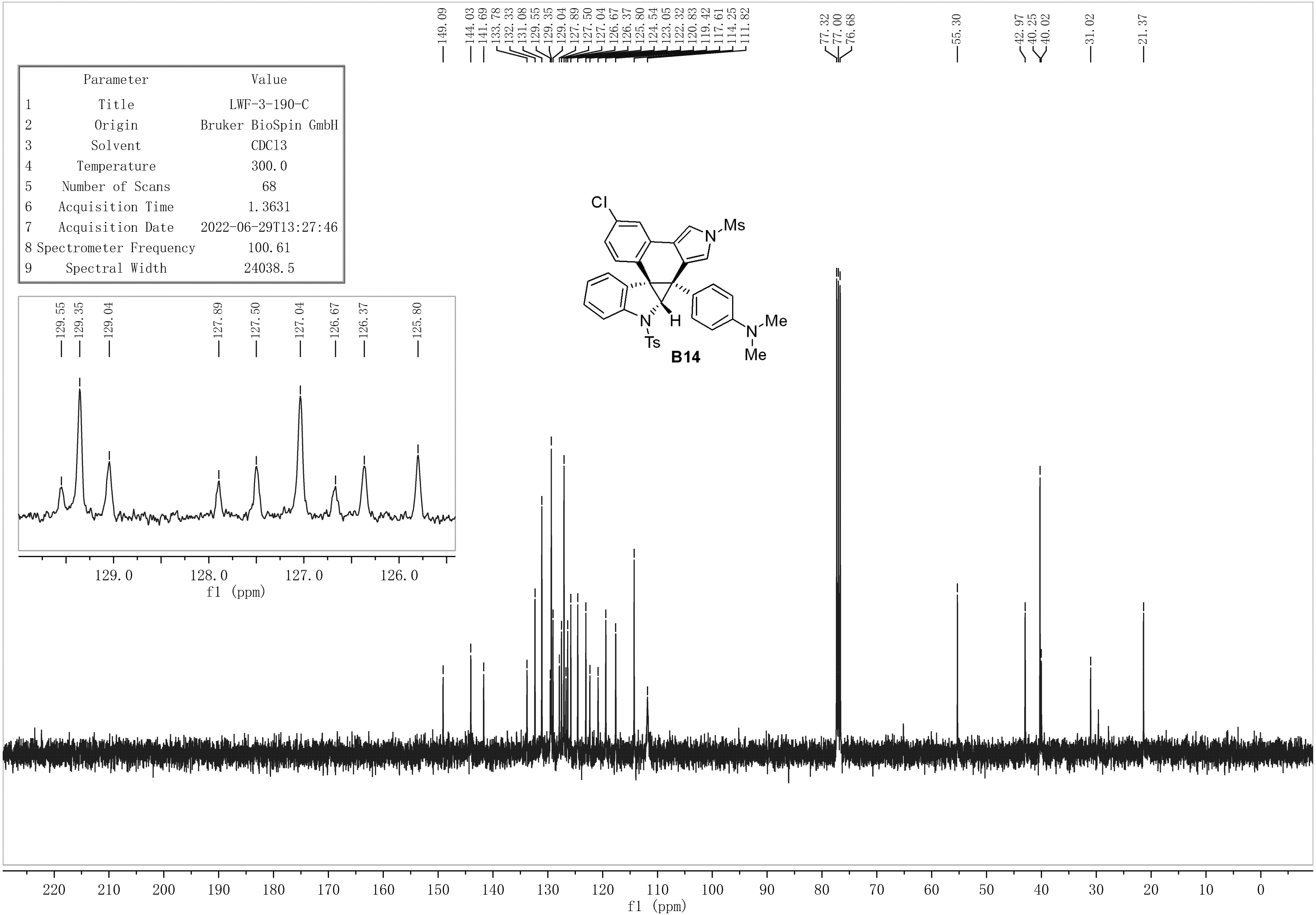

	Parameter	Value
1	Title	LWF-3-190-C
2	Origin	Bruker BioSpin GmbH
3	Solvent	CDCl3
4	Temperature	300.0
5	Number of Scans	68
6	Acquisition Time	1.3631
7	Acquisition Date	2022-06-29T13:27:46
8	Spectrometer Frequency	100.61
9	Spectral Width	24038.5

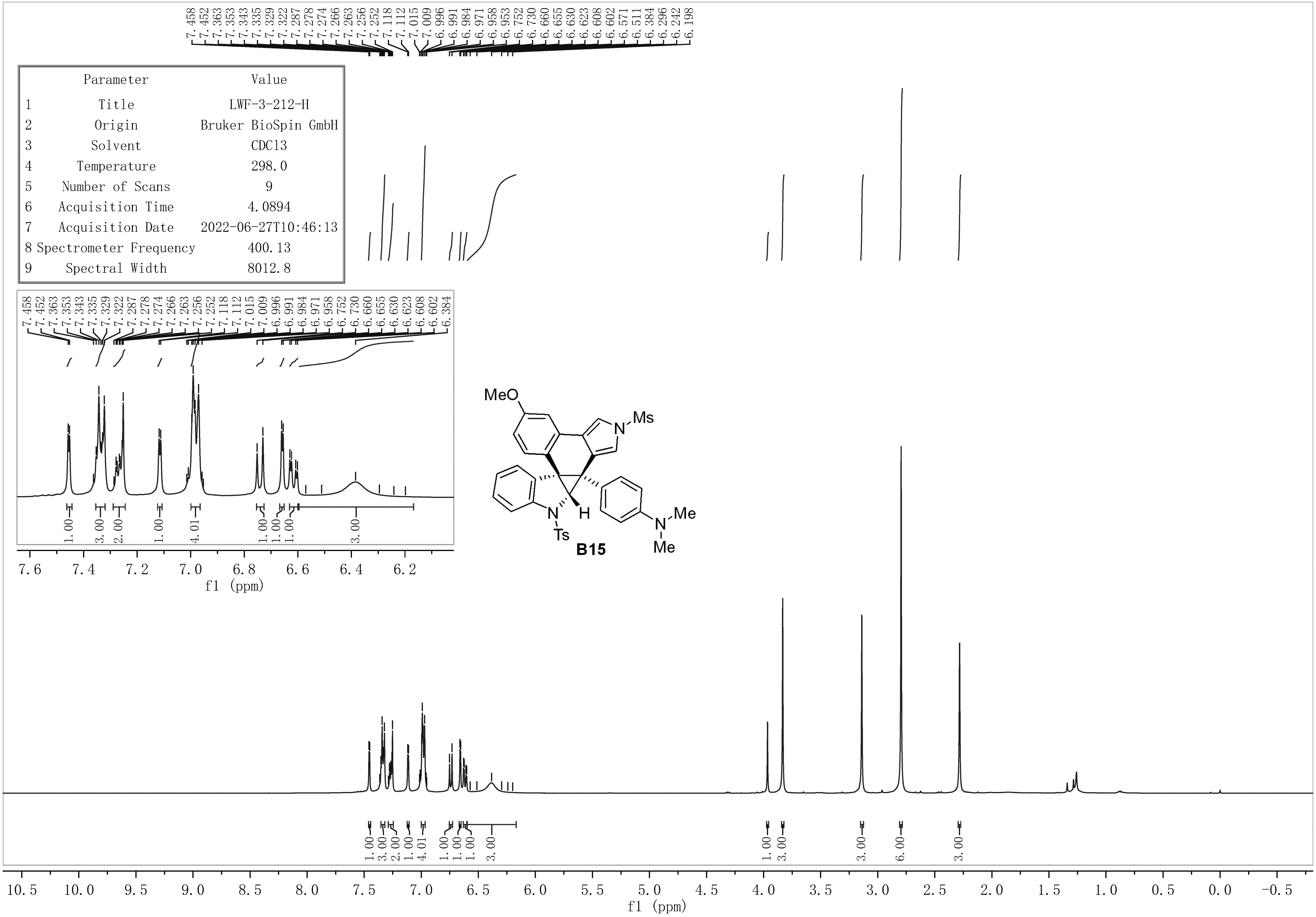
7.458 7.452 7.363 7.353 7.343 7.335 7.329 7.322 7.287 7.278 7.274 7.266 7.263 7.256 7.252 7.118 7.112 7.015 7.009 6.996 6.991 6.984 6.971 6.958 6.953 6.752 6.730 6.660 6.655 6.630 6.623 6.608 6.602 6.571 6.511 6.384 6.296 6.242 6.198
Parameter Value
1 Title LWF-3-212-H
2 Origin Bruker BioSpin GmbH
3 Solvent CDCl3
4 Temperature 298.0
5 Number of Scans 9
6 Acquisition Time 4.0894
7 Acquisition Date 2022-06-27T10:46:13
8 Spectrometer Frequency 400.13
9 Spectral Width 8012.8
1.00 3.00 2.00 1.00 4.01 1.00 1.00 1.00 3.00
f1 (ppm)
MeO
Ms
N
H
Ts
Me
B15
1.00 3.00 3.00 6.00 3.00
f1 (ppm)

	Parameter	Value
1	Title	LWF-3-212-C
2	Origin	Bruker BioSpin GmbH
3	Solvent	CDCl3
4	Temperature	300.0
5	Number of Scans	54
6	Acquisition Time	1.3631
7	Acquisition Date	2022-06-27T10:48:13
8	Spectrometer Frequency	100.61
9	Spectral Width	24038.5

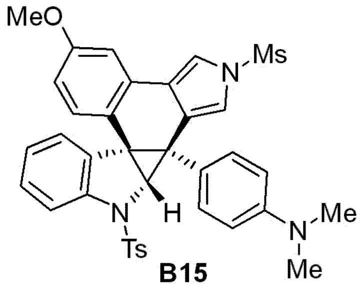

B15

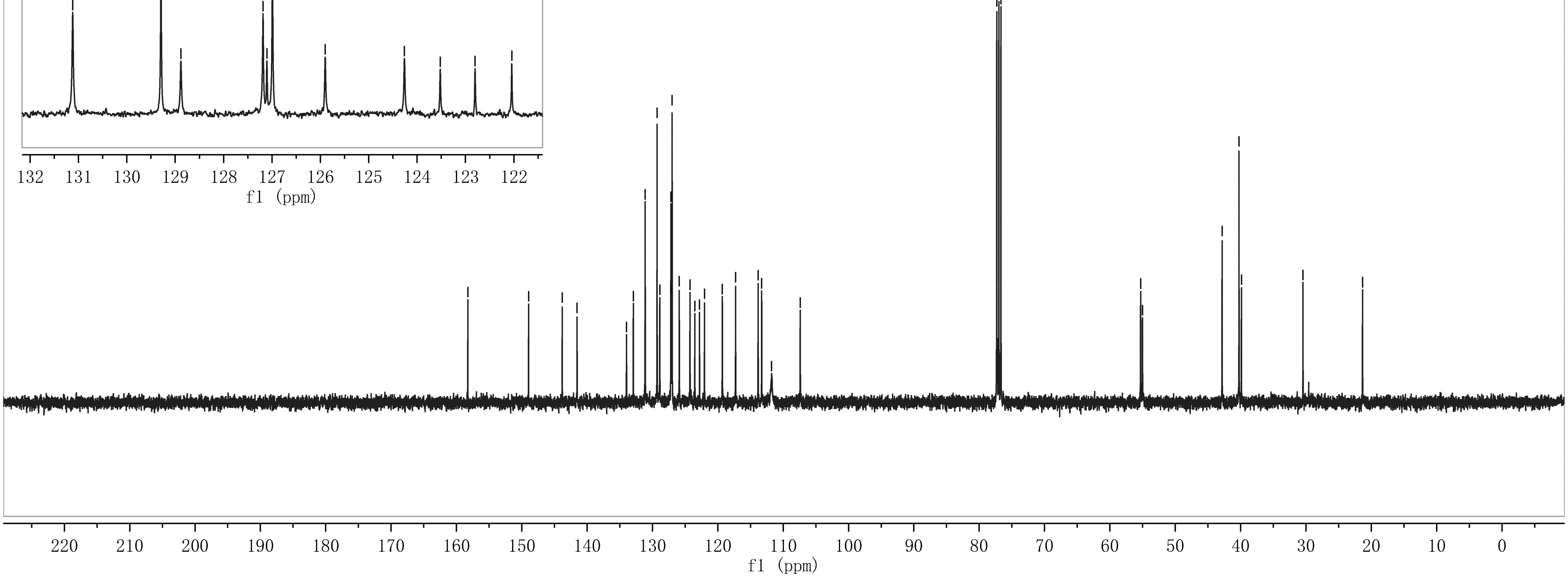

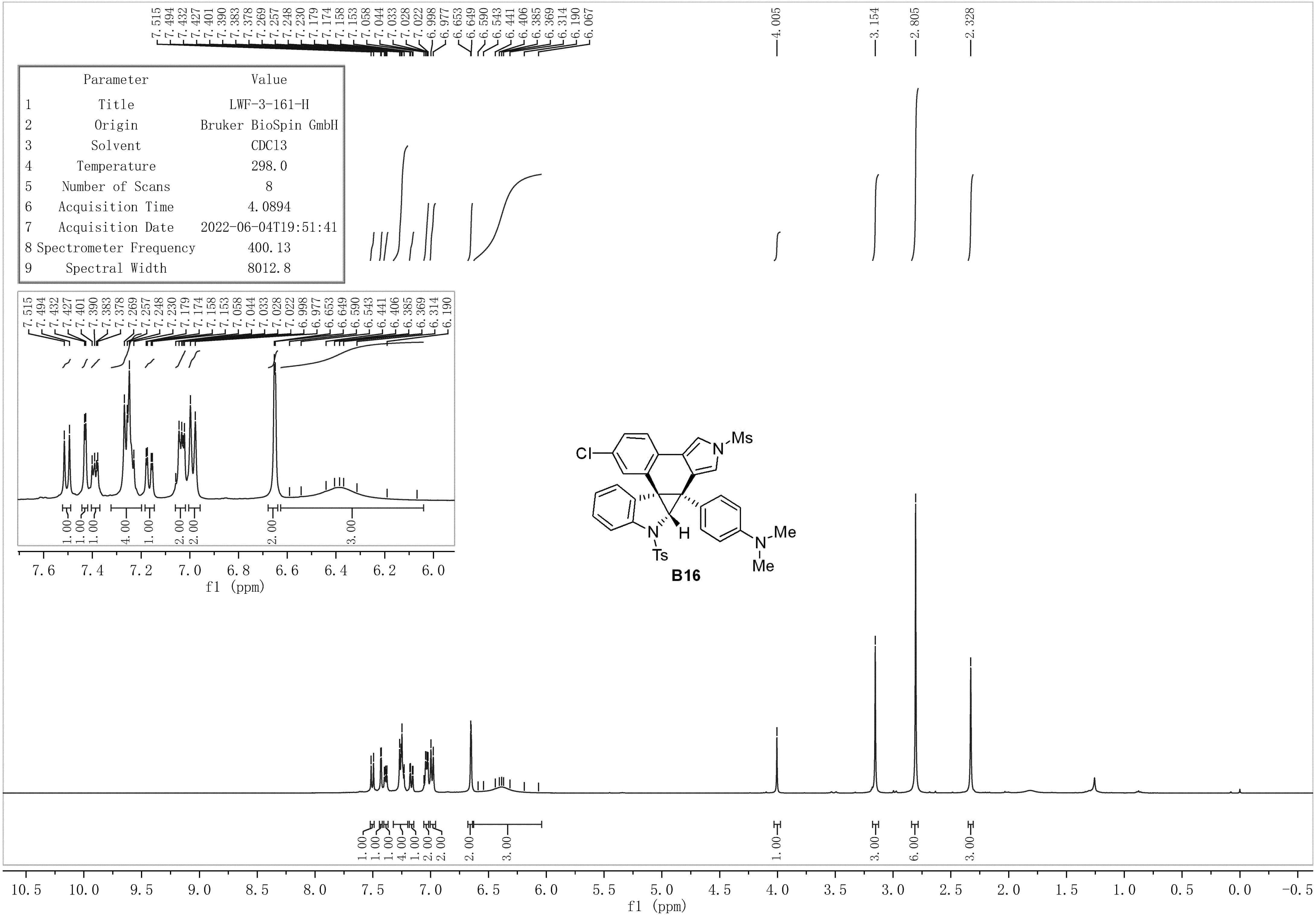

	Parameter	Value
1	Title	LWF-3-161-H
2	Origin	Bruker BioSpin GmbH
3	Solvent	CDCl3
4	Temperature	298.0
5	Number of Scans	8
6	Acquisition Time	4.0894
7	Acquisition Date	2022-06-04T19:51:41
8	Spectrometer Frequency	400.13
9	Spectral Width	8012.8

B16

	Parameter	Value
1	Title	LWF-3-161-C
2	Origin	Bruker BioSpin GmbH
3	Solvent	CDCl3
4	Temperature	300.0
5	Number of Scans	119
6	Acquisition Time	1.3631
7	Acquisition Date	2022-06-04T19:53:10
8	Spectrometer Frequency	100.61
9	Spectral Width	24038.5

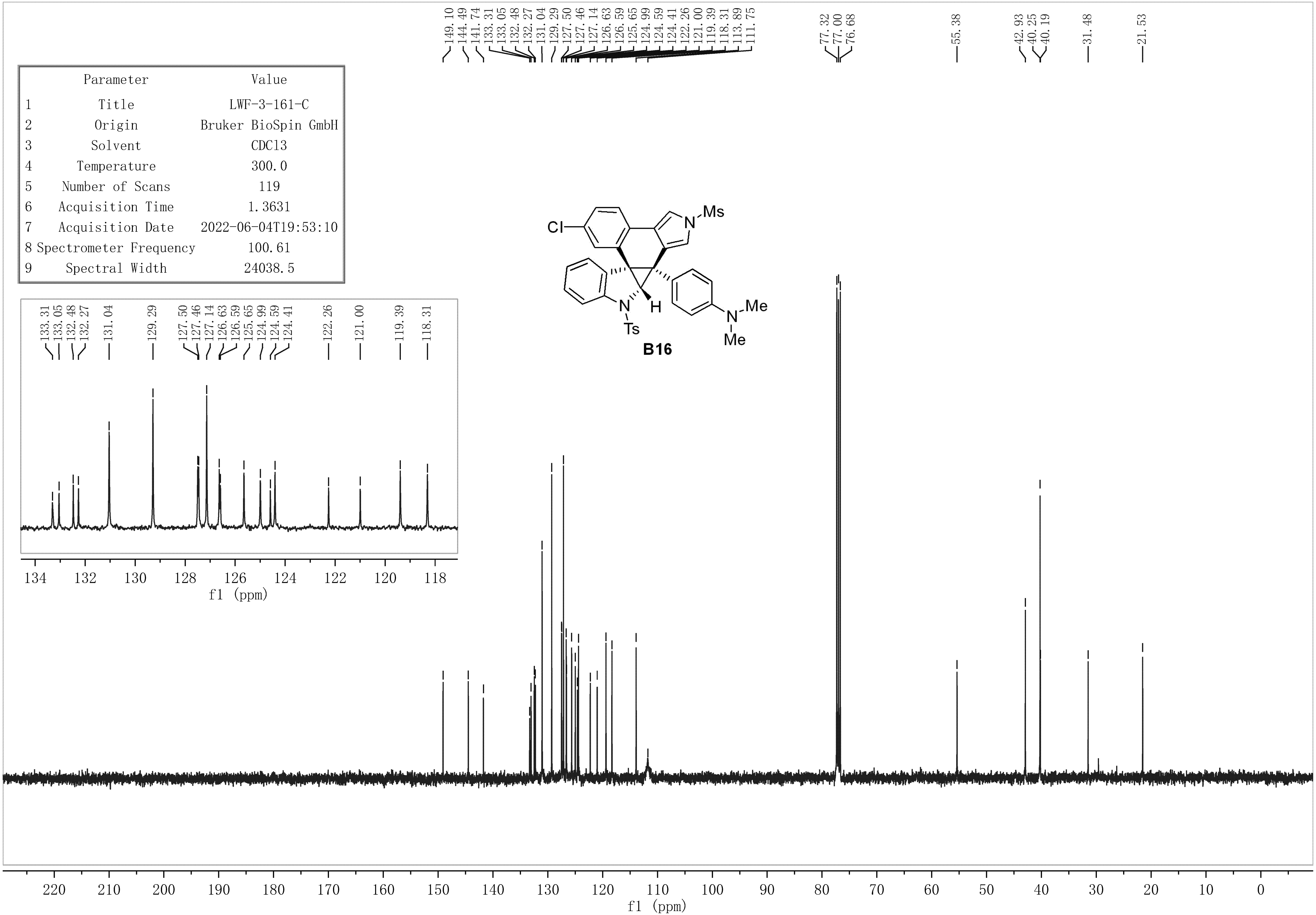

B16

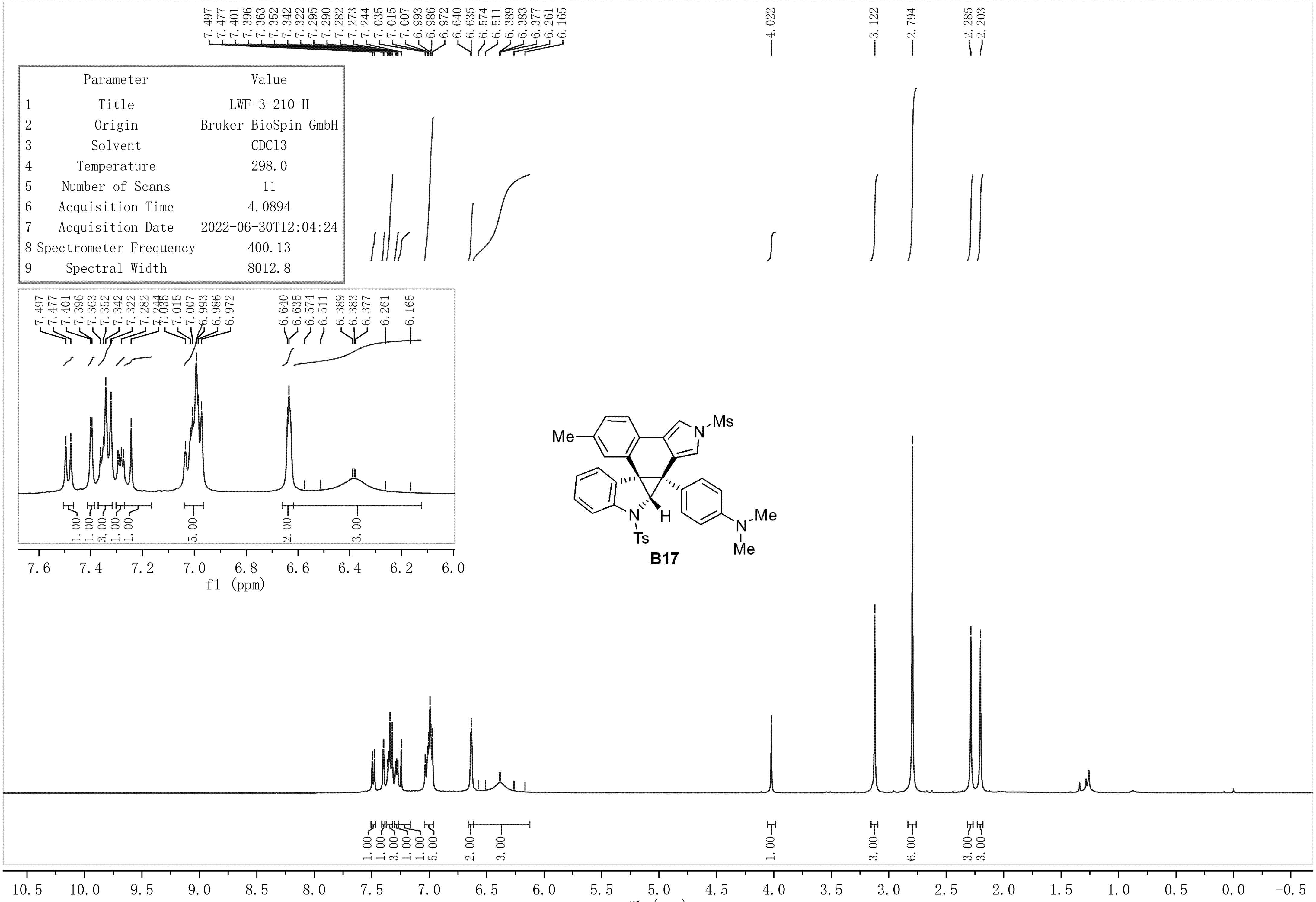

Parameter Value
1 Title LWF-3-210-H
2 Origin Bruker BioSpin GmbH
3 Solvent CDCl3
4 Temperature 298.0
5 Number of Scans 11
6 Acquisition Time 4.0894
7 Acquisition Date 2022-06-30T12:04:24
8 Spectrometer Frequency 400.13
9 Spectral Width 8012.8
7.497 7.477 7.401 7.396 7.363 7.352 7.342 7.322 7.295 7.290 7.282 7.273 7.244 7.035 7.015 7.007 6.993 6.986 6.972 6.640 6.635 6.574 6.511 6.389 6.383 6.377 6.261 6.165
4.022
3.122
2.794
2.285 2.203
1.00 1.00 3.00 1.00 1.00 5.00 2.00 3.00
1.00 3.00 6.00 3.00 3.00
f1 (ppm)
Me
N
Ms
N
Ts
H
N
Me
Me
B17

	Parameter	Value
1	Title	LWF-3-210-C
2	Origin	Bruker BioSpin GmbH
3	Solvent	CDCl3
4	Temperature	300.0
5	Number of Scans	48
6	Acquisition Time	1.3631
7	Acquisition Date	2022-06-30T12:06:13
8	Spectrometer Frequency	100.61
9	Spectral Width	24038.5

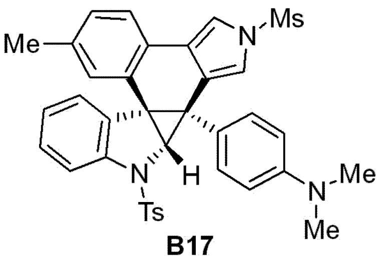

B17

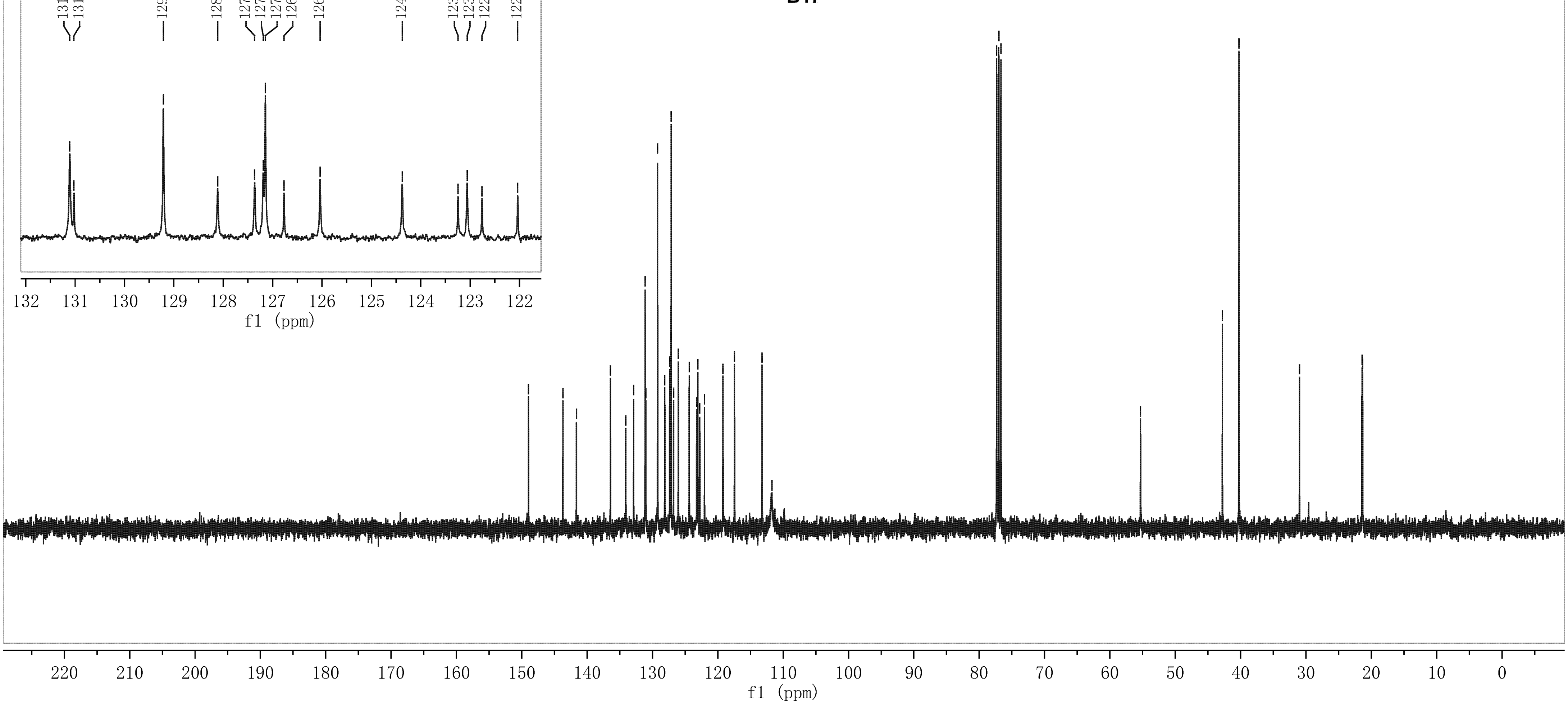

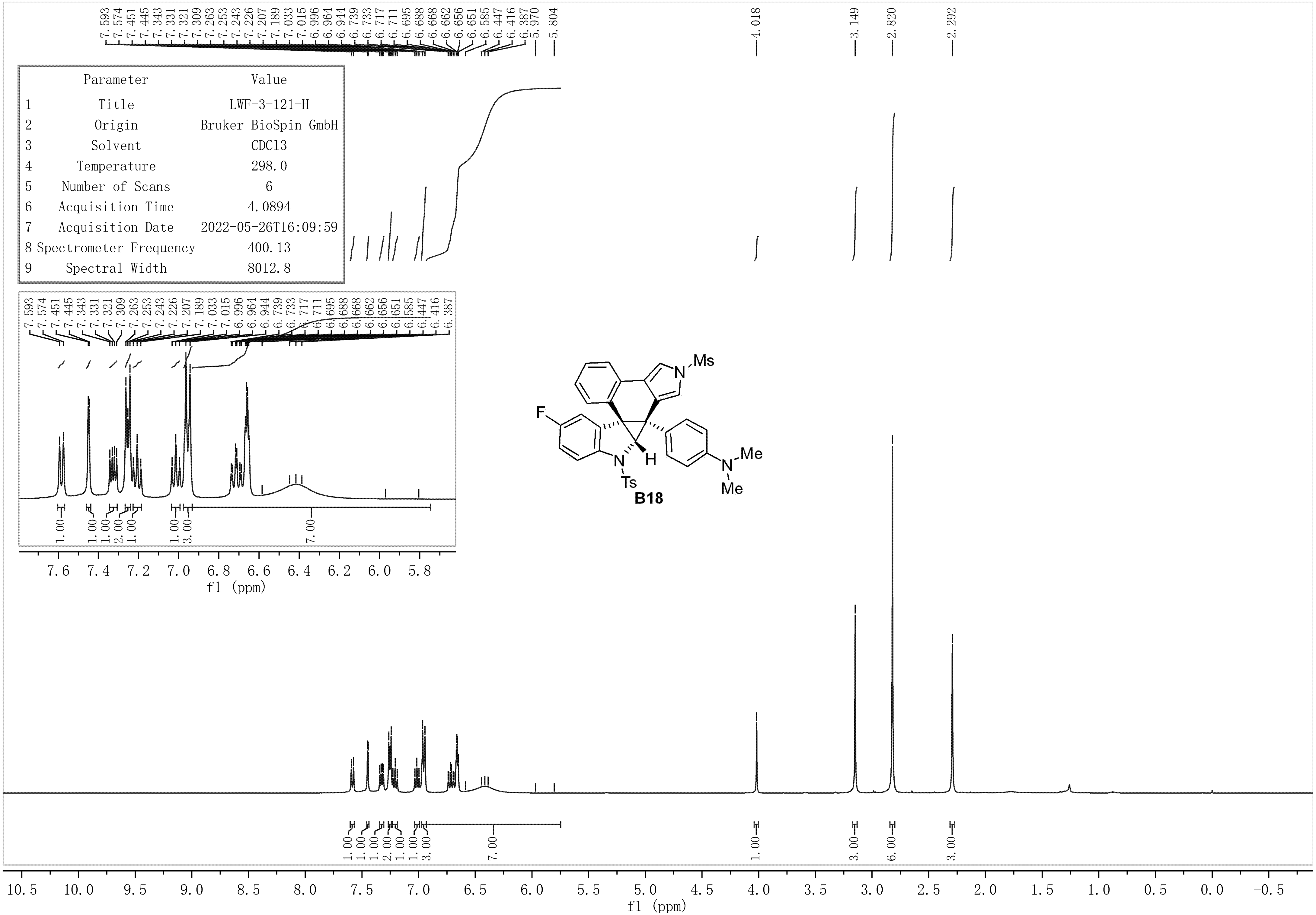

	Parameter	Value
1	Title	LWF-3-121-H
2	Origin	Bruker BioSpin GmbH
3	Solvent	CDCl3
4	Temperature	298.0
5	Number of Scans	6
6	Acquisition Time	4.0894
7	Acquisition Date	2022-05-26T16:09:59
8	Spectrometer Frequency	400.13
9	Spectral Width	8012.8

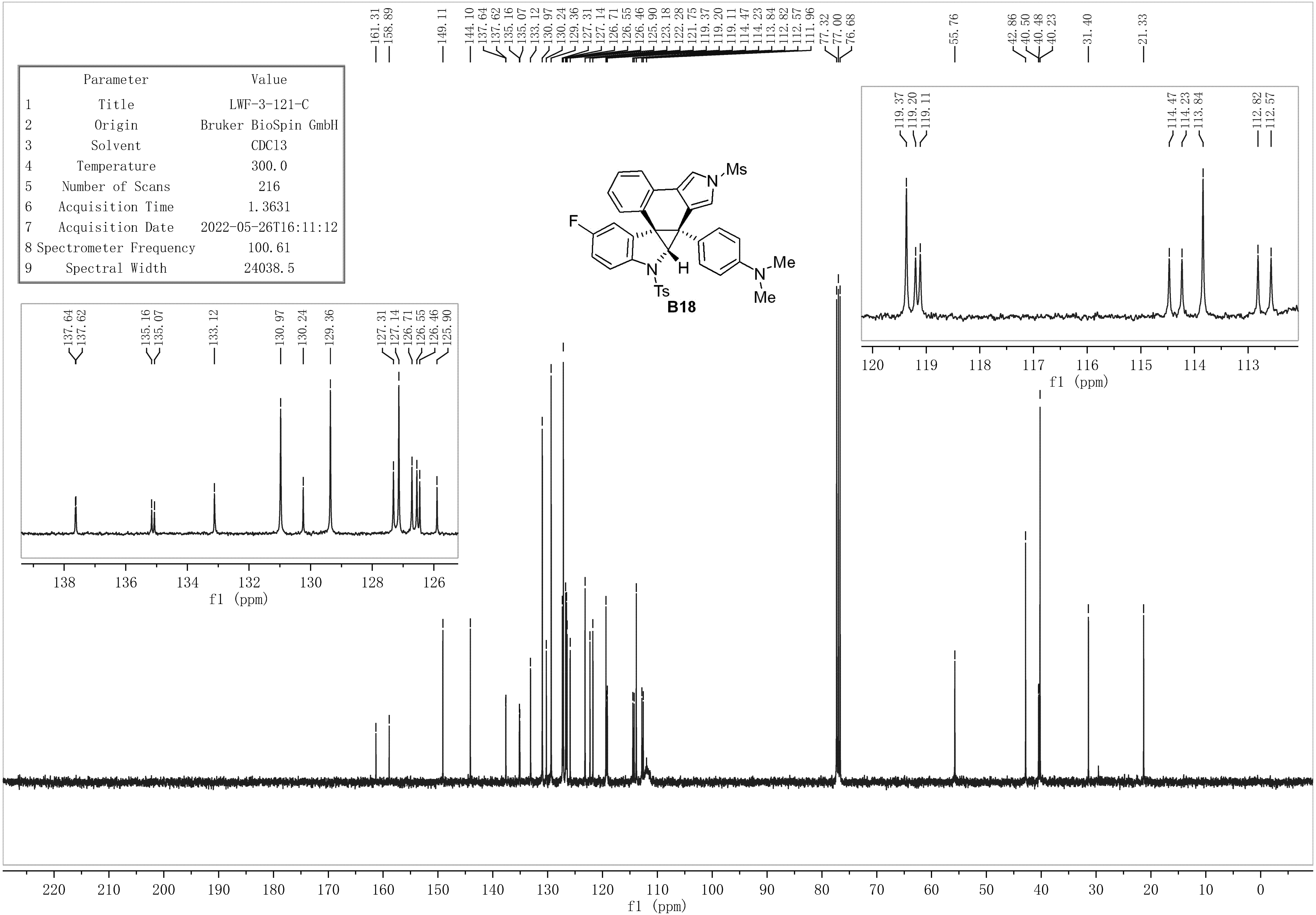

	Parameter	Value
1	Title	LWF-3-121-C
2	Origin	Bruker BioSpin GmbH
3	Solvent	CDCl3
4	Temperature	300.0
5	Number of Scans	216
6	Acquisition Time	1.3631
7	Acquisition Date	2022-05-26T16:11:12
8	Spectrometer Frequency	100.61
9	Spectral Width	24038.5

	Parameter	Value
1	Title	LWF-3-121-F
2	Origin	
3	Solvent	CDCl3
4	Temperature	299.4
5	Number of Scans	8
6	Acquisition Time	1.0000
7	Acquisition Date	2022-05-27T00:07:20
8	Spectrometer Frequency	376.30
9	Spectral Width	96153.0

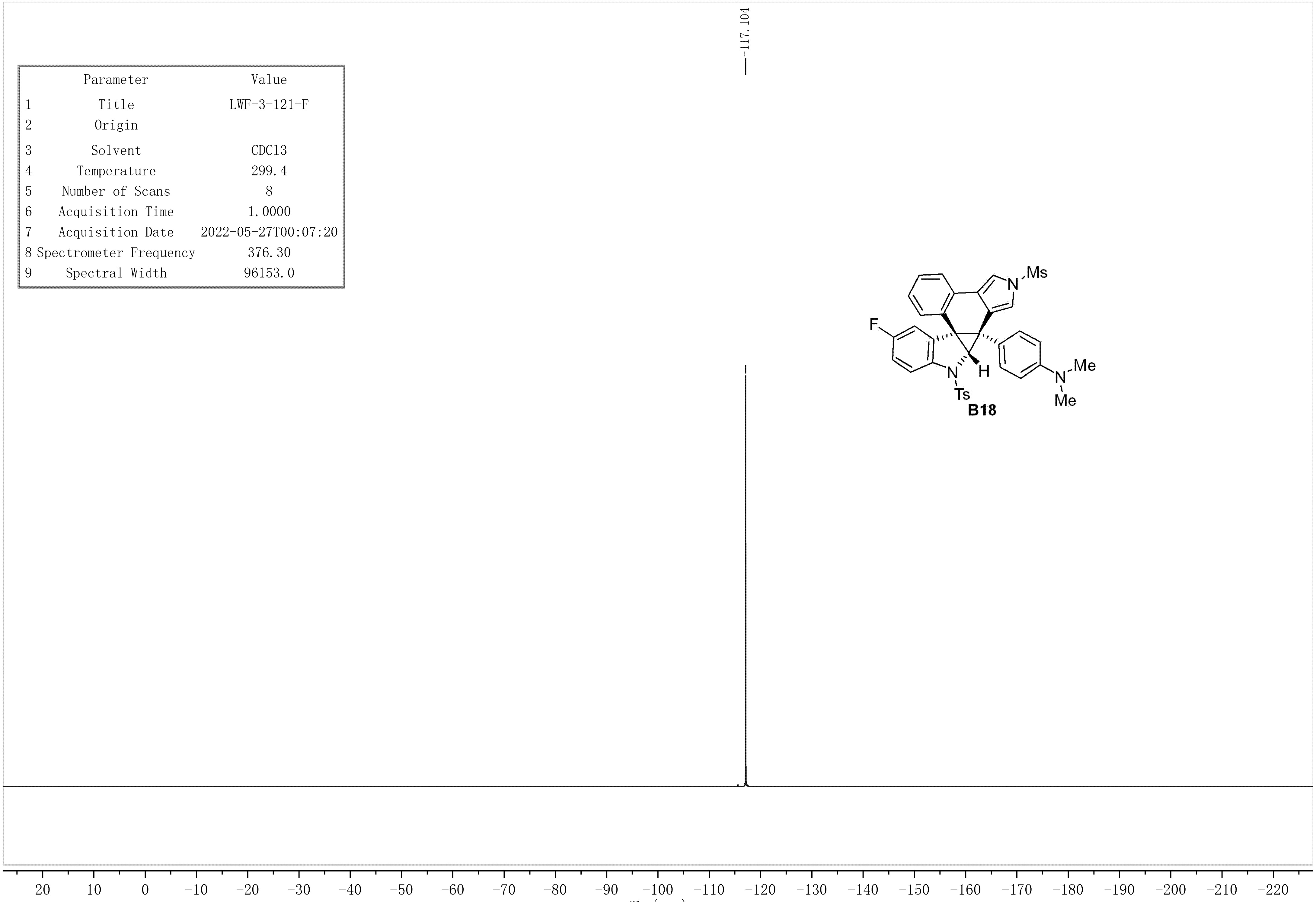

B18

	Parameter	Value
1	Title	LWF-3-139-H
2	Origin	Bruker BioSpin GmbH
3	Solvent	CDCl3
4	Temperature	298.0
5	Number of Scans	6
6	Acquisition Time	4.0894
7	Acquisition Date	2022-06-07T16:59:37
8	Spectrometer Frequency	400.13
9	Spectral Width	8012.8

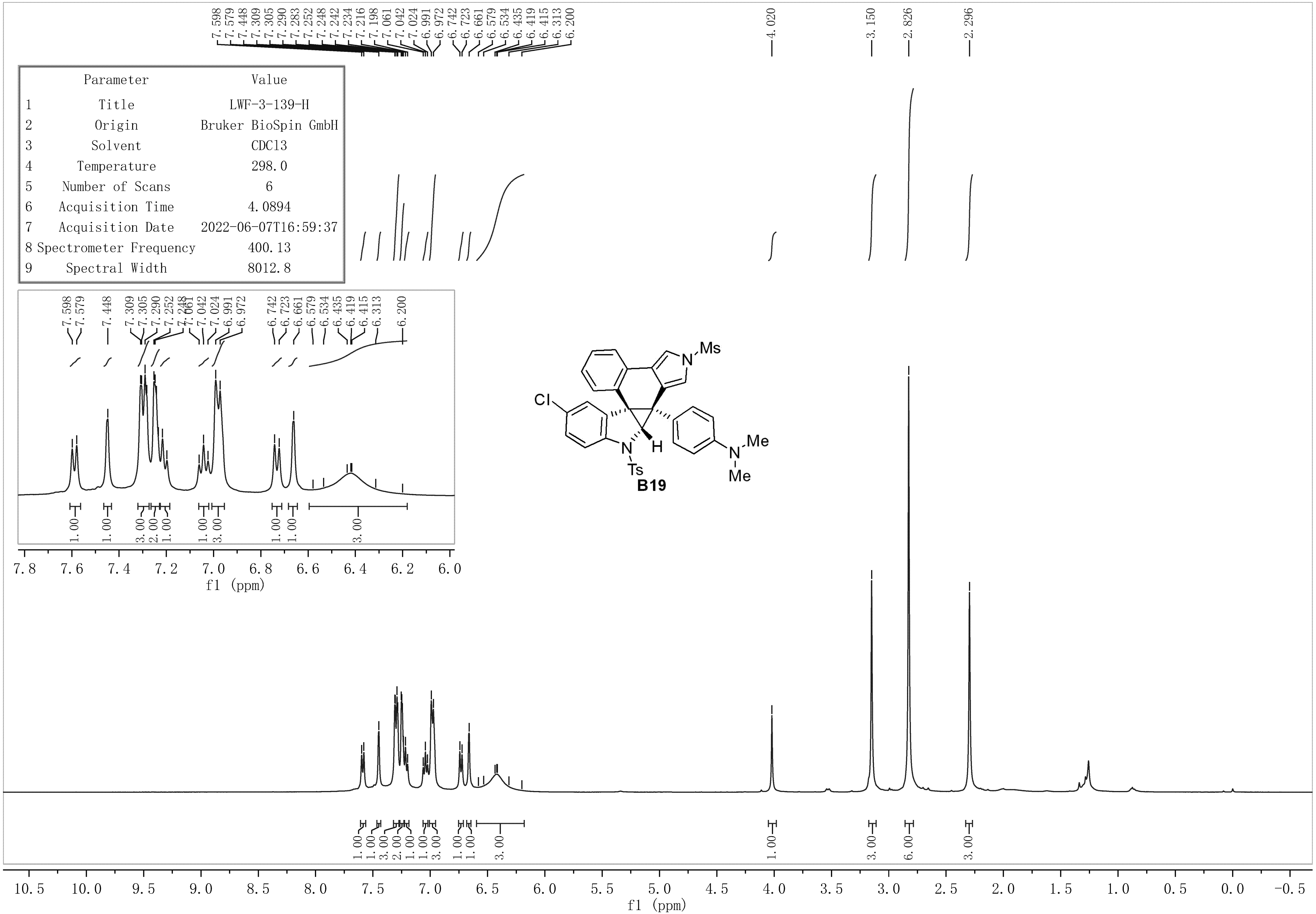

B19

	Parameter	Value
1	Title	LWF-3-139-C
2	Origin	Bruker BioSpin GmbH
3	Solvent	CDCl3
4	Temperature	300.0
5	Number of Scans	109
6	Acquisition Time	1.3631
7	Acquisition Date	2022-06-07T17:00:48
8	Spectrometer Frequency	100.61
9	Spectral Width	24038.5

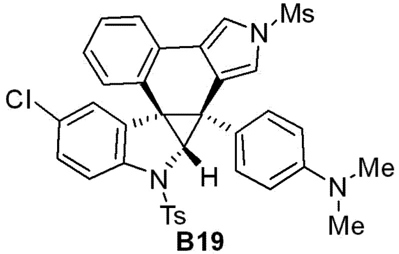

B19

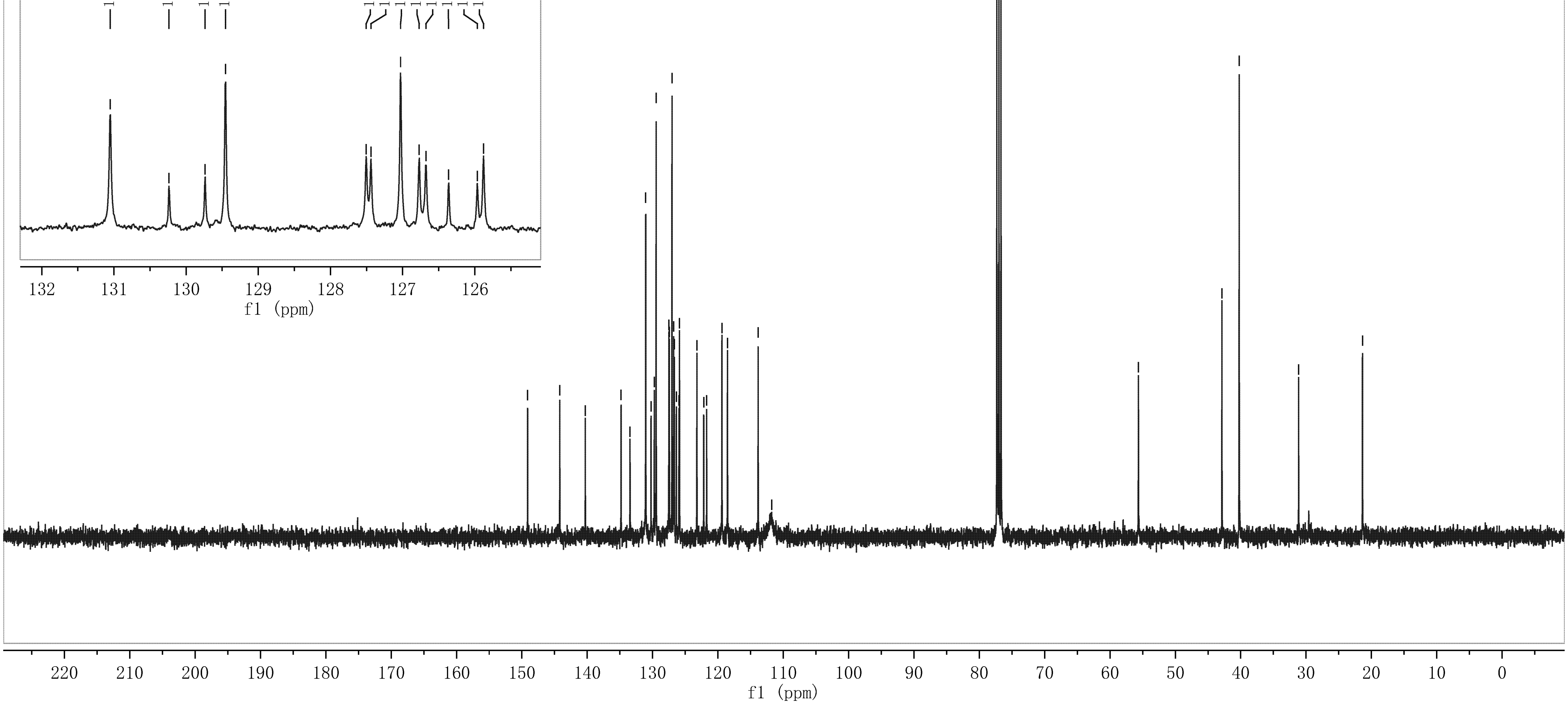

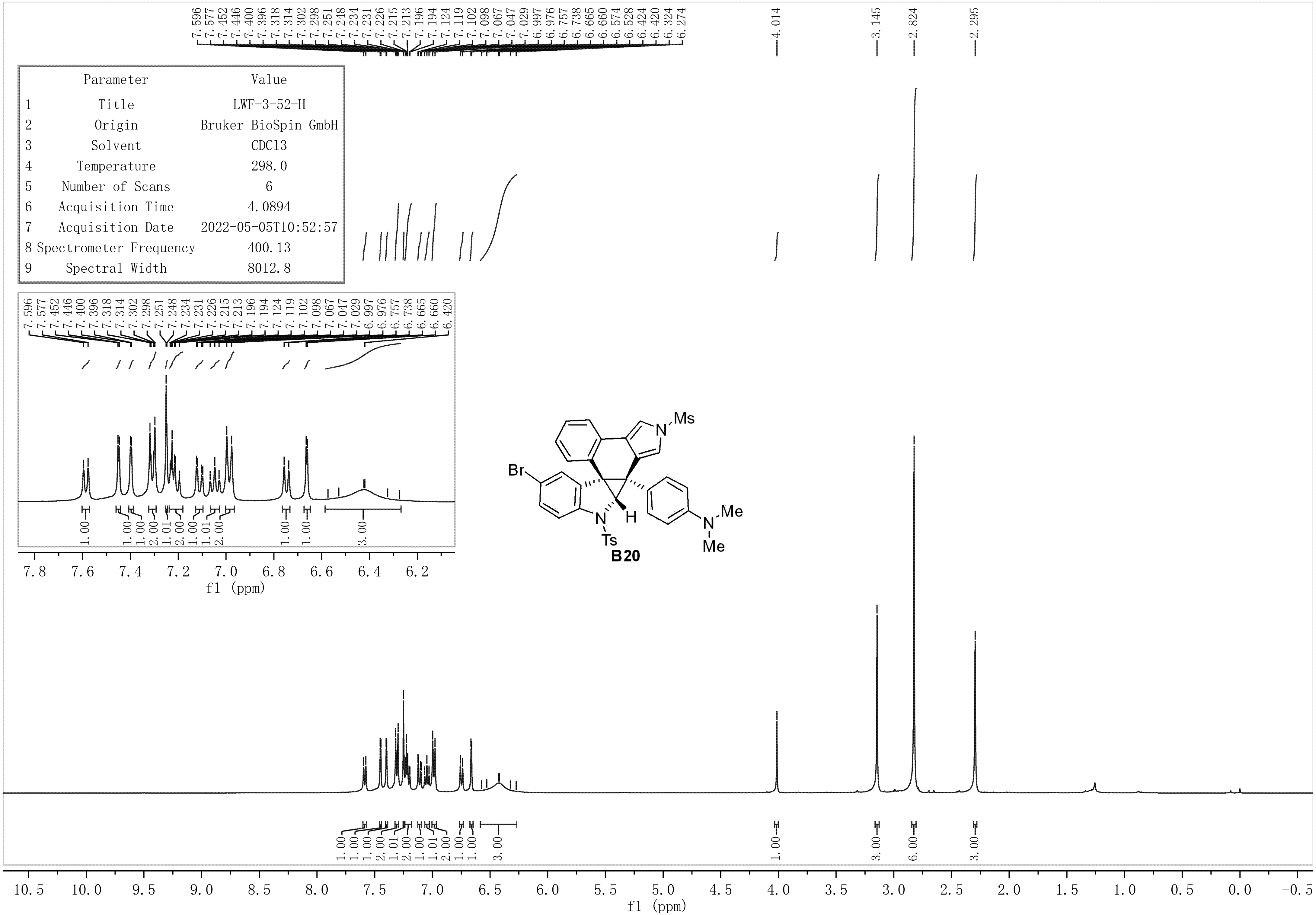
7.596 7.577 7.452 7.446 7.400 7.396 7.318 7.314 7.302 7.298 7.251 7.248 7.234 7.231 7.226 7.215 7.213 7.196 7.194 7.124 7.119 7.102 7.098 7.067 7.047 7.029 6.997 6.976 6.757 6.738 6.665 6.660 6.574 6.528 6.424 6.420 6.324 6.274
4.014
3.145
2.824
2.295
Parameter Value
1 Title LWF-3-52-H
2 Origin Bruker BioSpin GmbH
3 Solvent CDCl3
4 Temperature 298.0
5 Number of Scans 6
6 Acquisition Time 4.0894
7 Acquisition Date 2022-05-05T10:52:57
8 Spectrometer Frequency 400.13
9 Spectral Width 8012.8
Br
N
Ms
Ts
H
Me
B20
f1 (ppm)

	Parameter	Value
1	Title	LWF-3-52-C
2	Origin	Bruker BioSpin GmbH
3	Solvent	CDCl3
4	Temperature	300.0
5	Number of Scans	104
6	Acquisition Time	1.3631
7	Acquisition Date	2022-05-05T10:54:13
8	Spectrometer Frequency	100.61
9	Spectral Width	24038.5

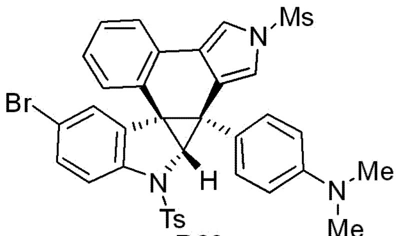

B20

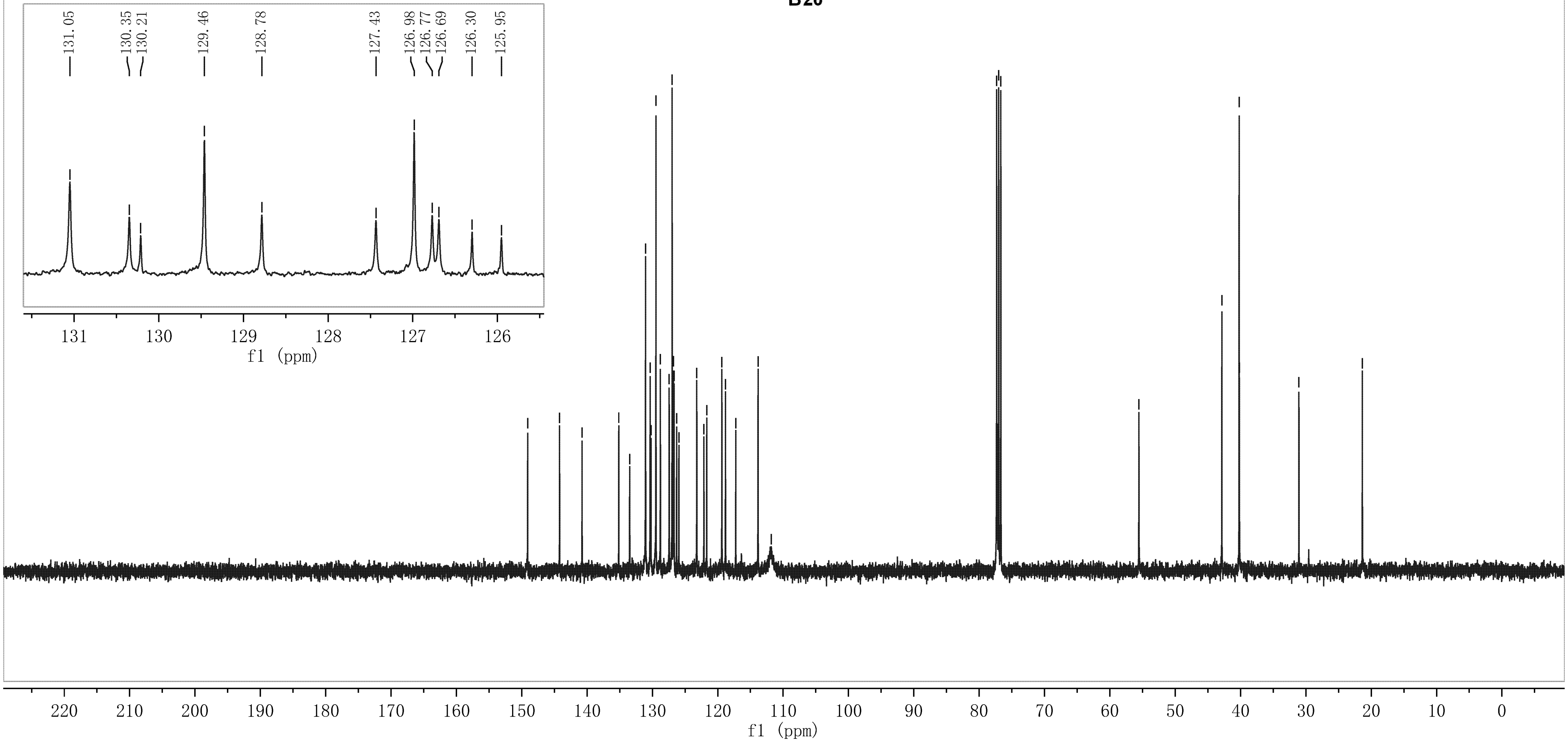

Parameter		Value
1	Title	LWF-3-92-H
2	Origin	Bruker BioSpin GmbH
3	Solvent	CDCl3
4	Temperature	298.0
5	Number of Scans	6
6	Acquisition Time	4.0894
7	Acquisition Date	2022-05-12T11:39:43
8	Spectrometer Frequency	400.13
9	Spectral Width	8012.8

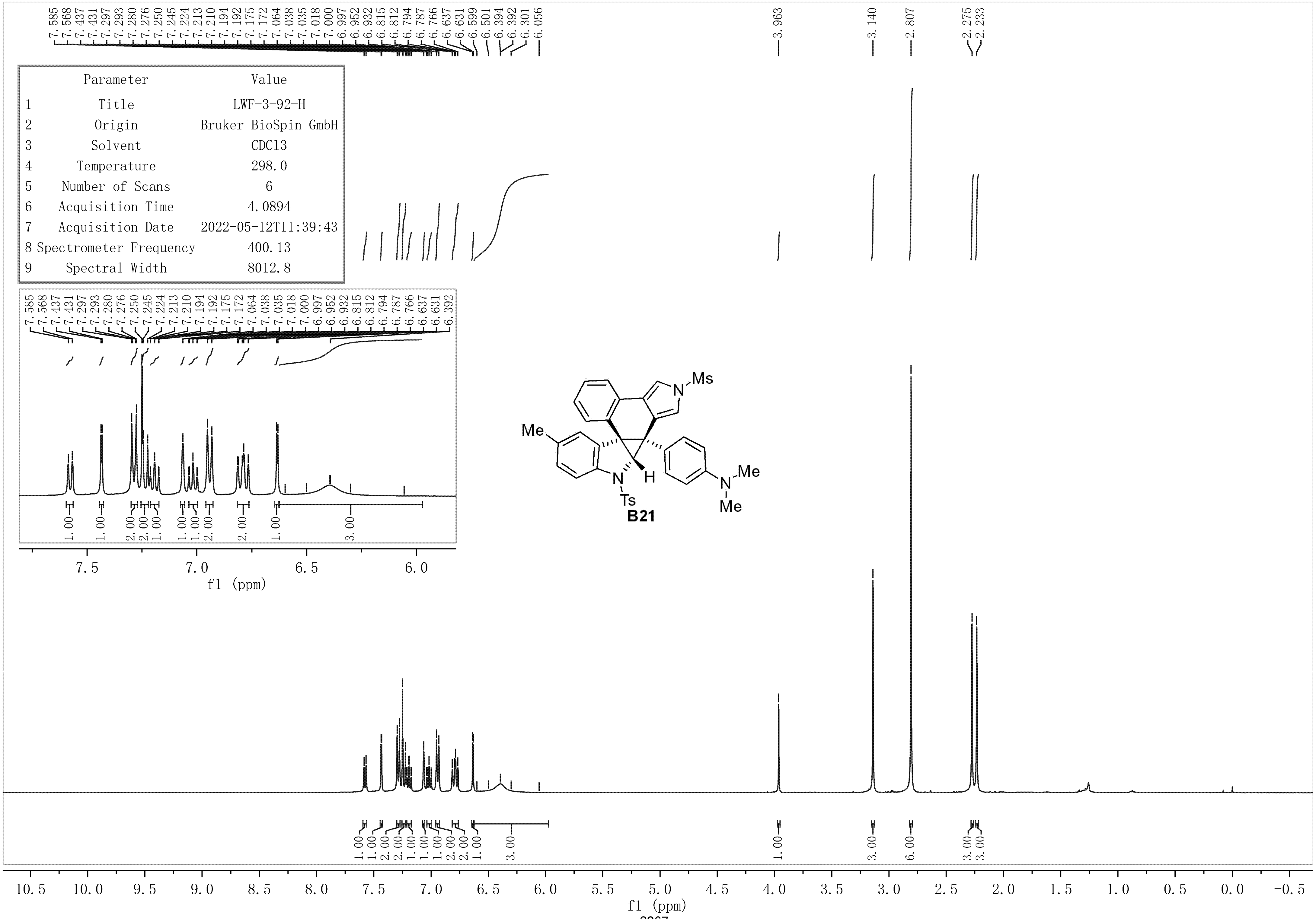

	Parameter	Value
1	Title	LWF-3-92-C
2	Origin	Bruker BioSpin GmbH
3	Solvent	CDCl3
4	Temperature	300.0
5	Number of Scans	132
6	Acquisition Time	1.3631
7	Acquisition Date	2022-05-12T11:41:25
8	Spectrometer Frequency	100.61
9	Spectral Width	24038.5

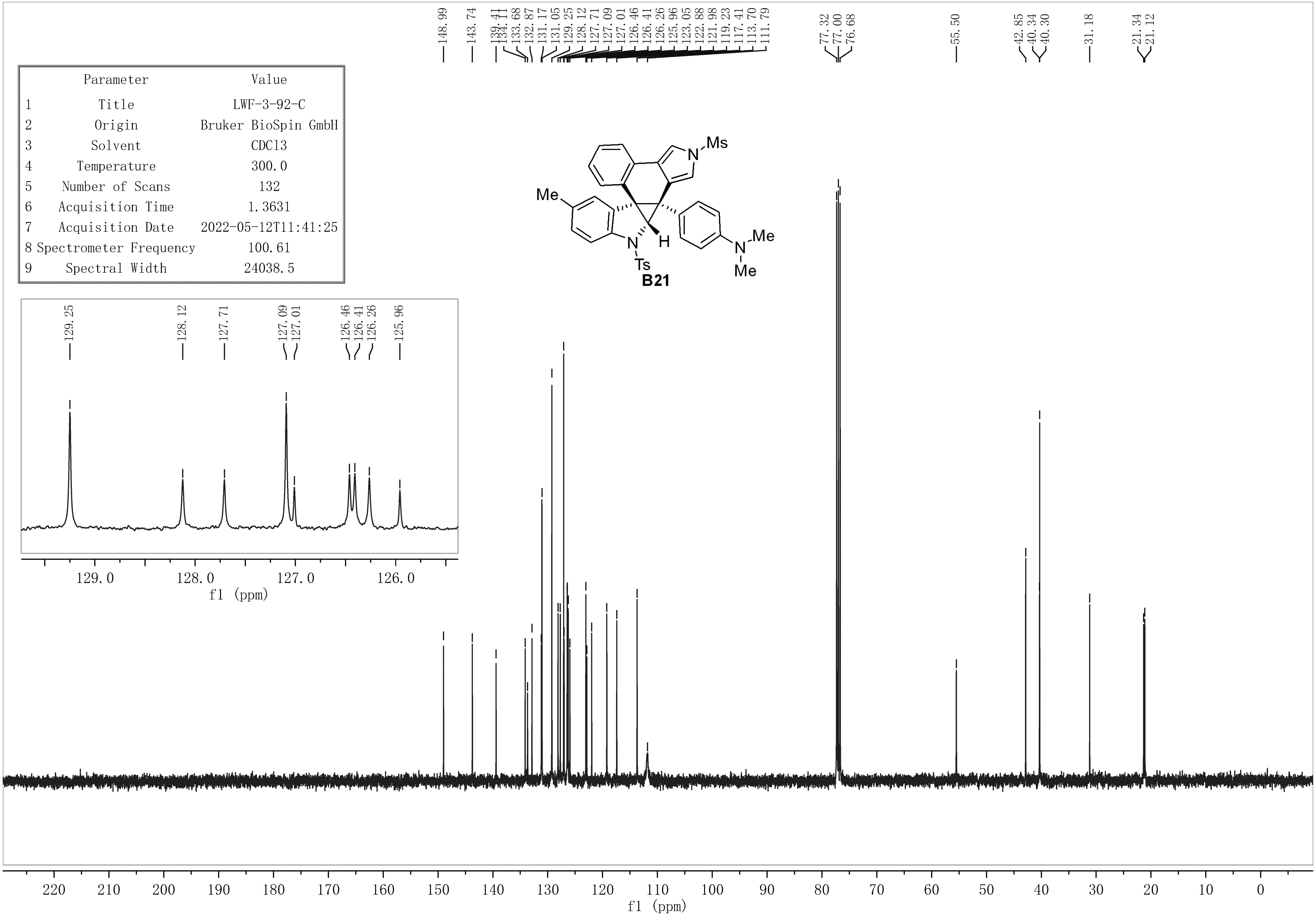

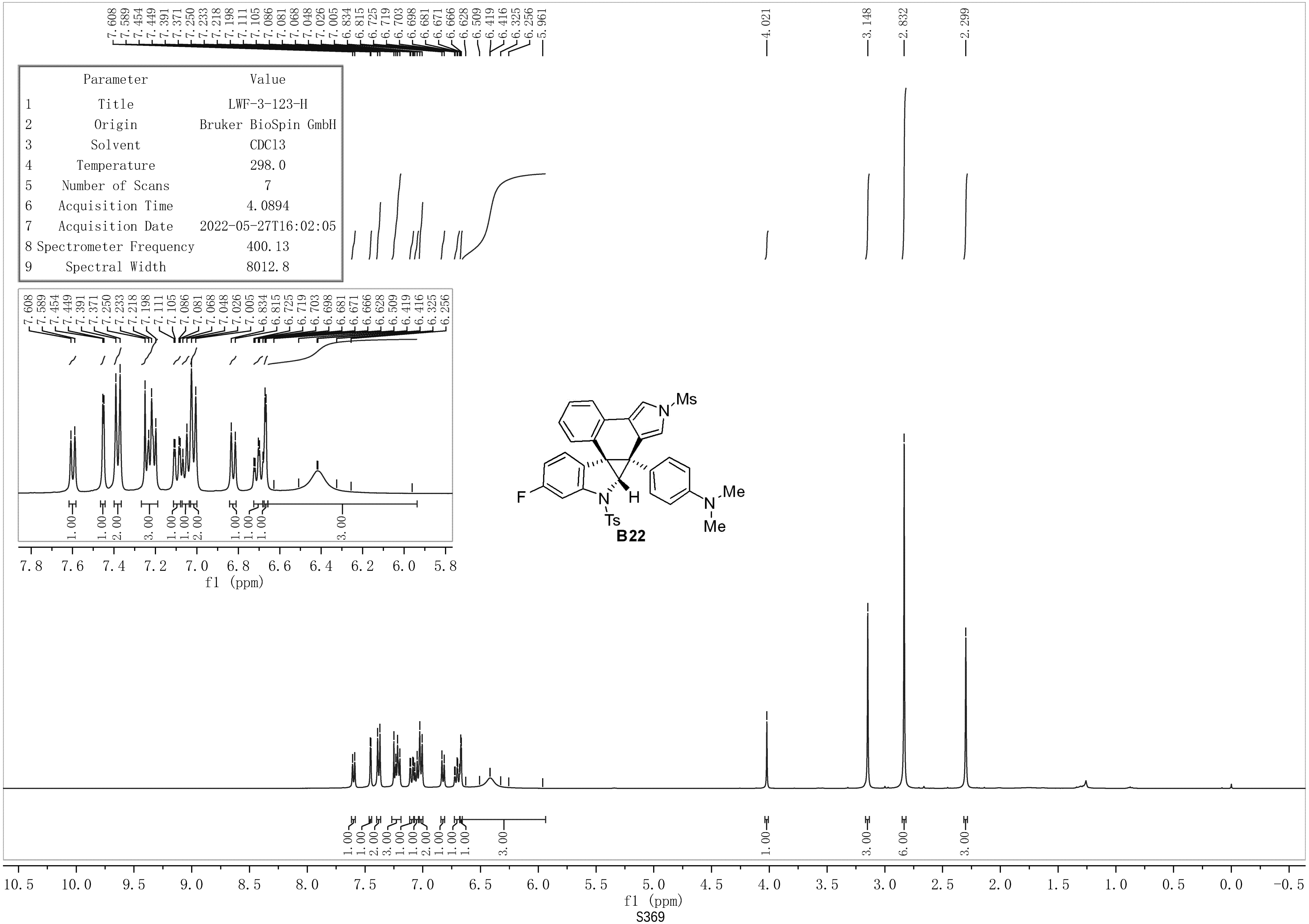

	Parameter	Value
1	Title	LWF-3-123-H
2	Origin	Bruker BioSpin GmbH
3	Solvent	CDCl3
4	Temperature	298.0
5	Number of Scans	7
6	Acquisition Time	4.0894
7	Acquisition Date	2022-05-27T16:02:05
8	Spectrometer Frequency	400.13
9	Spectral Width	8012.8

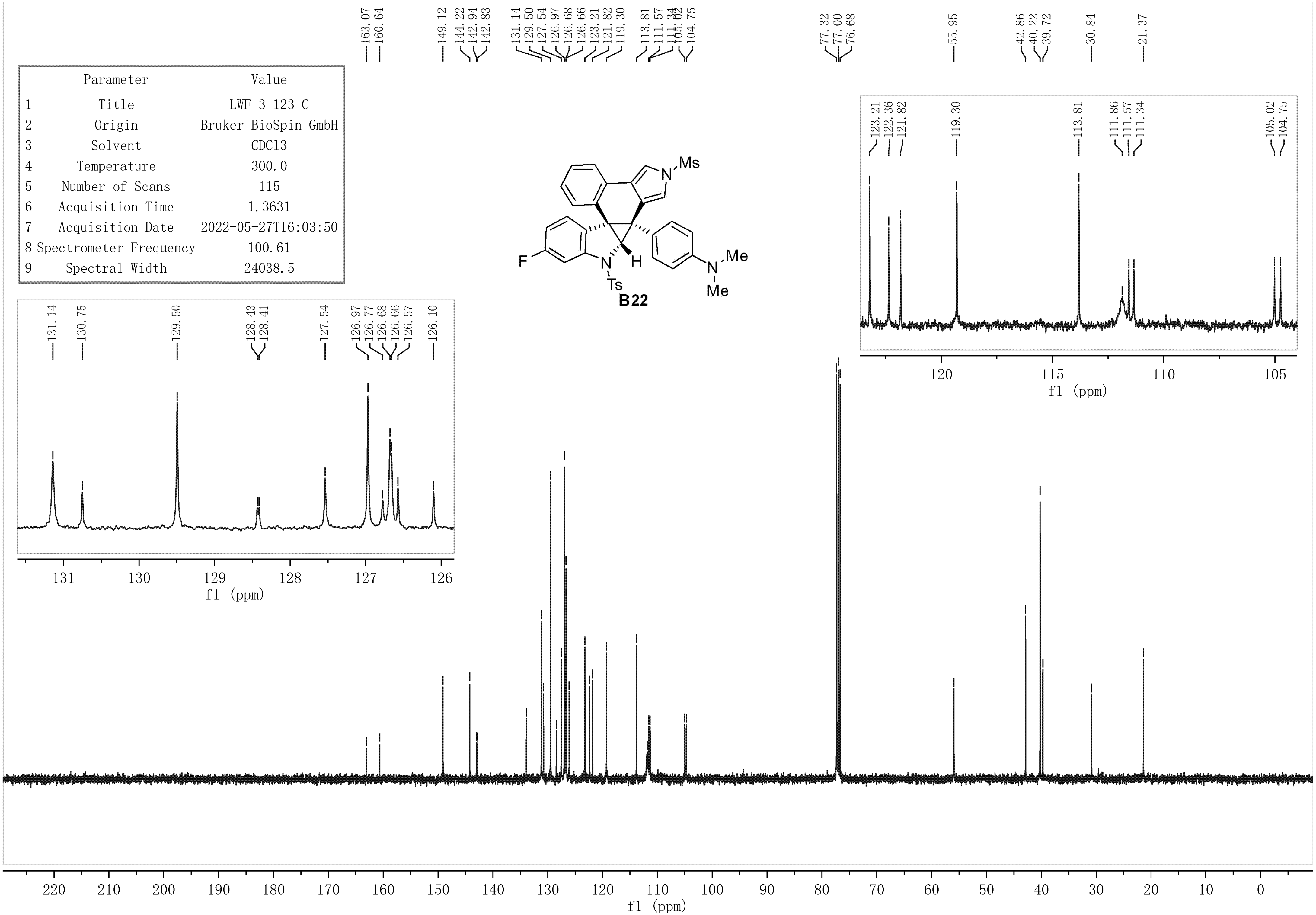

163.07
160.64
149.12
144.22
142.94
142.83
131.14
129.50
127.54
126.97
126.68
126.66
123.21
121.82
119.30
113.81
111.57
111.34
105.02
104.75
77.32
77.00
76.68
55.95
42.86
40.22
39.72
30.84
21.37
Parameter Value
1 Title LWF-3-123-C
2 Origin Bruker BioSpin GmbH
3 Solvent CDCl3
4 Temperature 300.0
5 Number of Scans 115
6 Acquisition Time 1.3631
7 Acquisition Date 2022-05-27T16:03:50
8 Spectrometer Frequency 100.61
9 Spectral Width 24038.5
Ms
N
F
N
Ts
H
N
Me
Me
B22
131.14
130.75
129.50
128.43
128.41
127.54
126.97
126.77
126.68
126.66
126.57
126.10
f1 (ppm)
123.21
122.36
121.82
119.30
113.81
111.86
111.57
111.34
105.02
104.75
f1 (ppm)
f1 (ppm)

	Parameter	Value
1	Title	LWF-3-123-F
2	Origin	
3	Solvent	CDCl3
4	Temperature	299.4
5	Number of Scans	16
6	Acquisition Time	1.0000
7	Acquisition Date	2022-05-28T00:33:47
8	Spectrometer Frequency	376.30
9	Spectral Width	96153.0

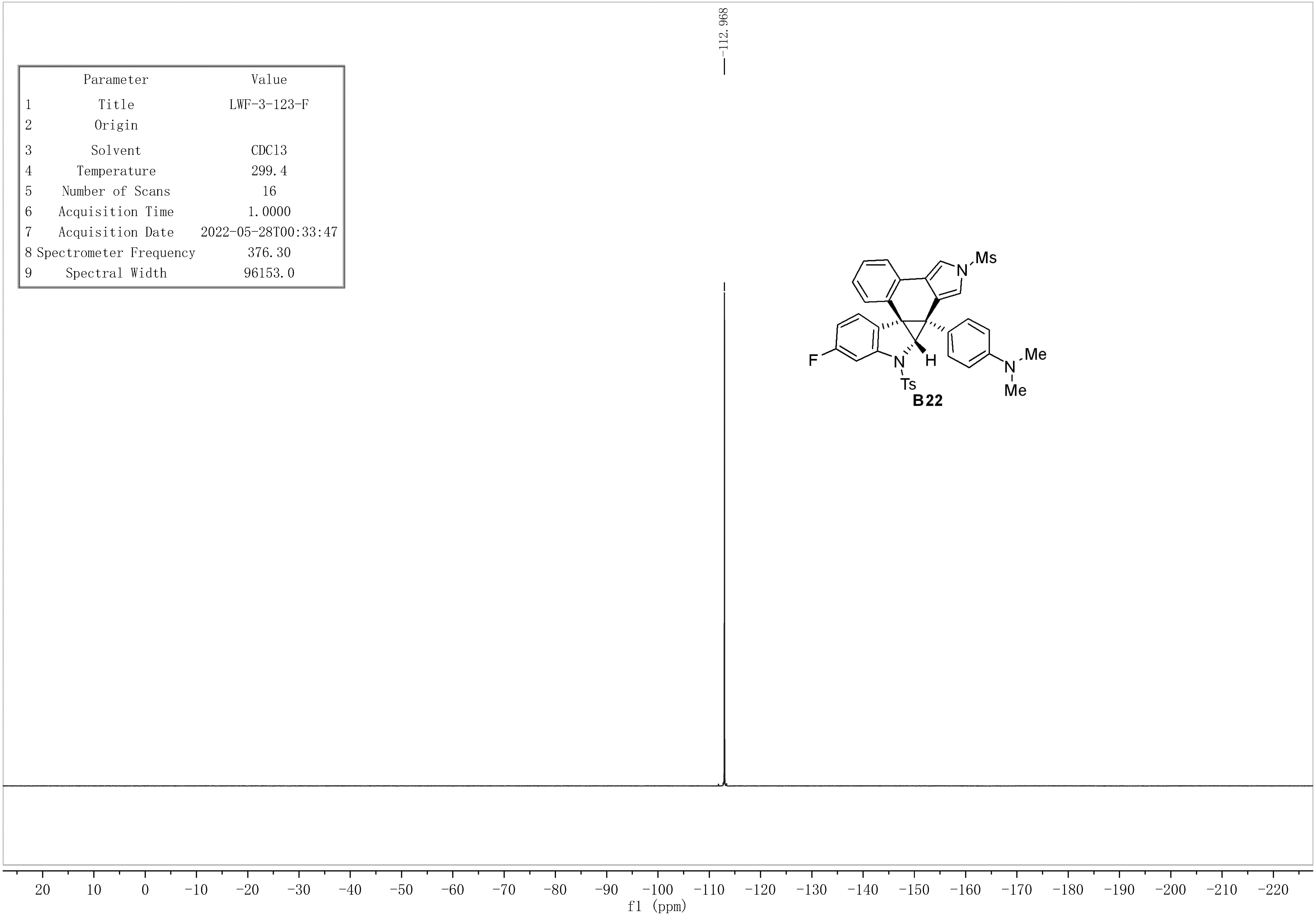

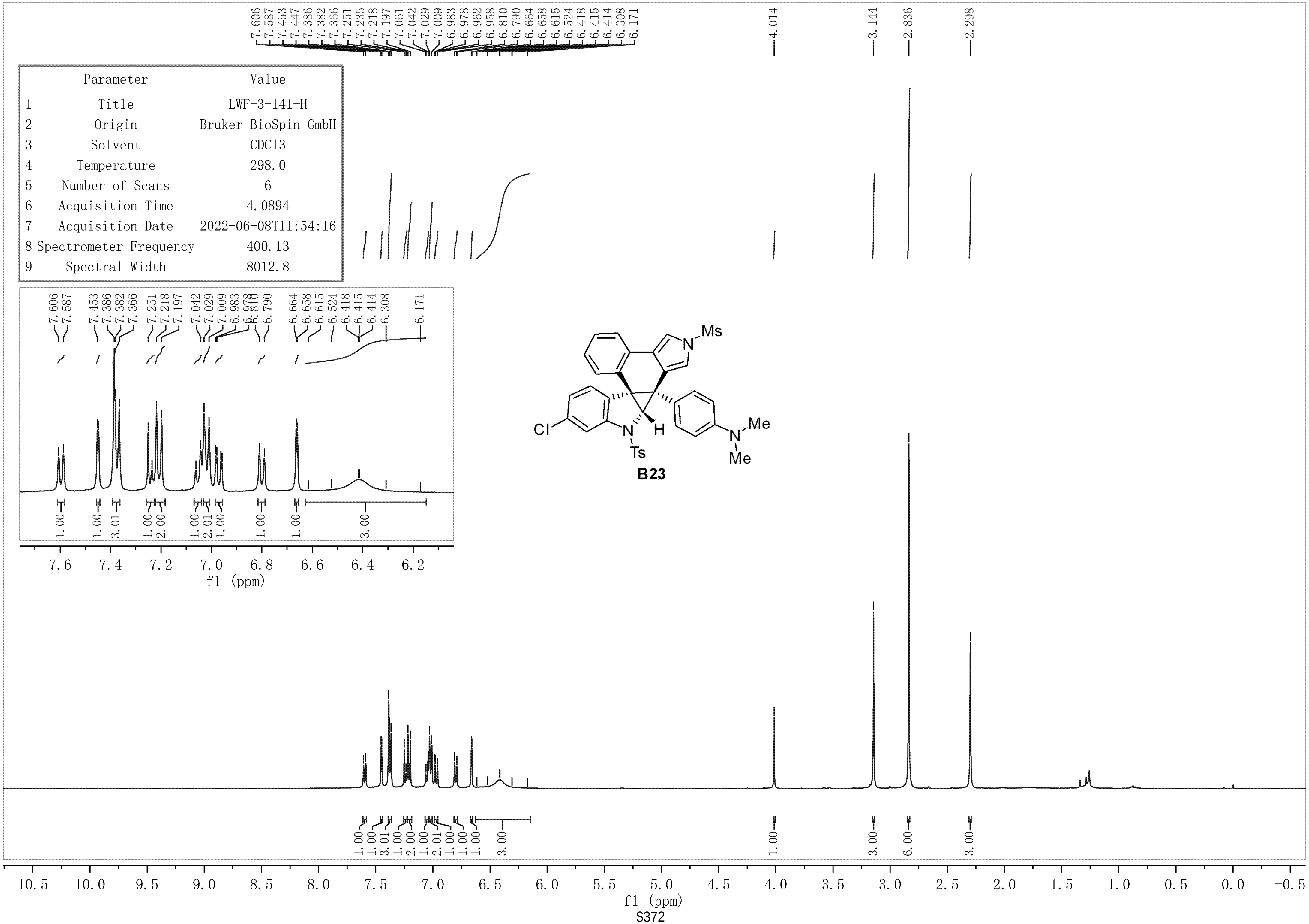

7.606 7.587 7.453 7.447 7.386 7.382 7.366 7.251 7.235 7.218 7.197 7.061 7.042 7.029 7.009 6.983 6.978 6.962 6.958 6.810 6.790 6.664 6.658 6.615 6.524 6.418 6.415 6.414 6.308 6.171
4.014
3.144
2.836
2.298
Parameter Value
1 Title LWF-3-141-H
2 Origin Bruker BioSpin GmbH
3 Solvent CDCl3
4 Temperature 298.0
5 Number of Scans 6
6 Acquisition Time 4.0894
7 Acquisition Date 2022-06-08T11:54:16
8 Spectrometer Frequency 400.13
9 Spectral Width 8012.8
1.00 1.00 3.01 1.00 2.00 1.00 2.01 1.00 1.00 1.00 3.00
1.00 3.00 6.00 3.00
f1 (ppm)
Ms
N
Cl
N
Ts
H
N
Me
Me
B23

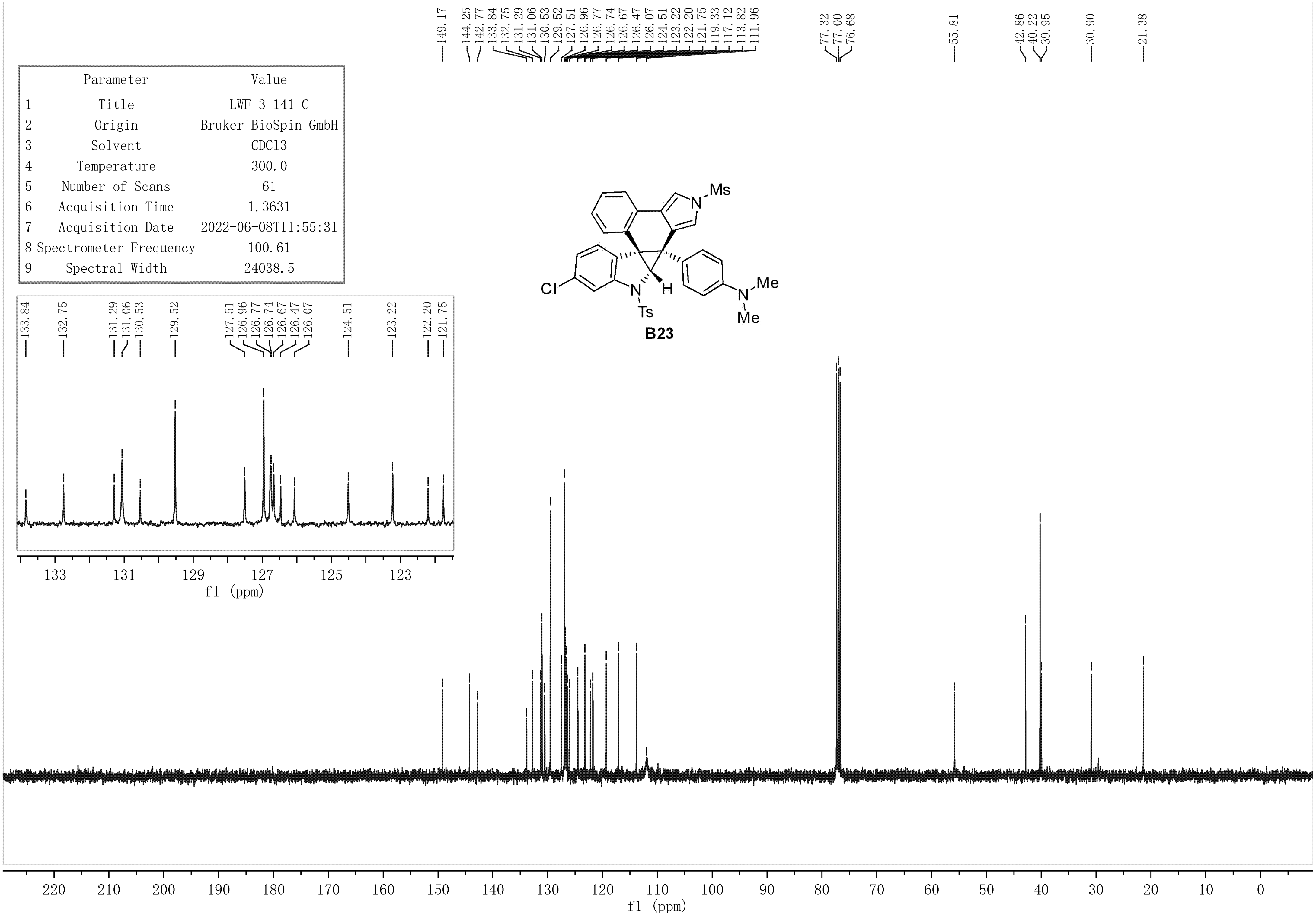

	Parameter	Value
1	Title	LWF-3-141-C
2	Origin	Bruker BioSpin GmbH
3	Solvent	CDCl3
4	Temperature	300.0
5	Number of Scans	61
6	Acquisition Time	1.3631
7	Acquisition Date	2022-06-08T11:55:31
8	Spectrometer Frequency	100.61
9	Spectral Width	24038.5

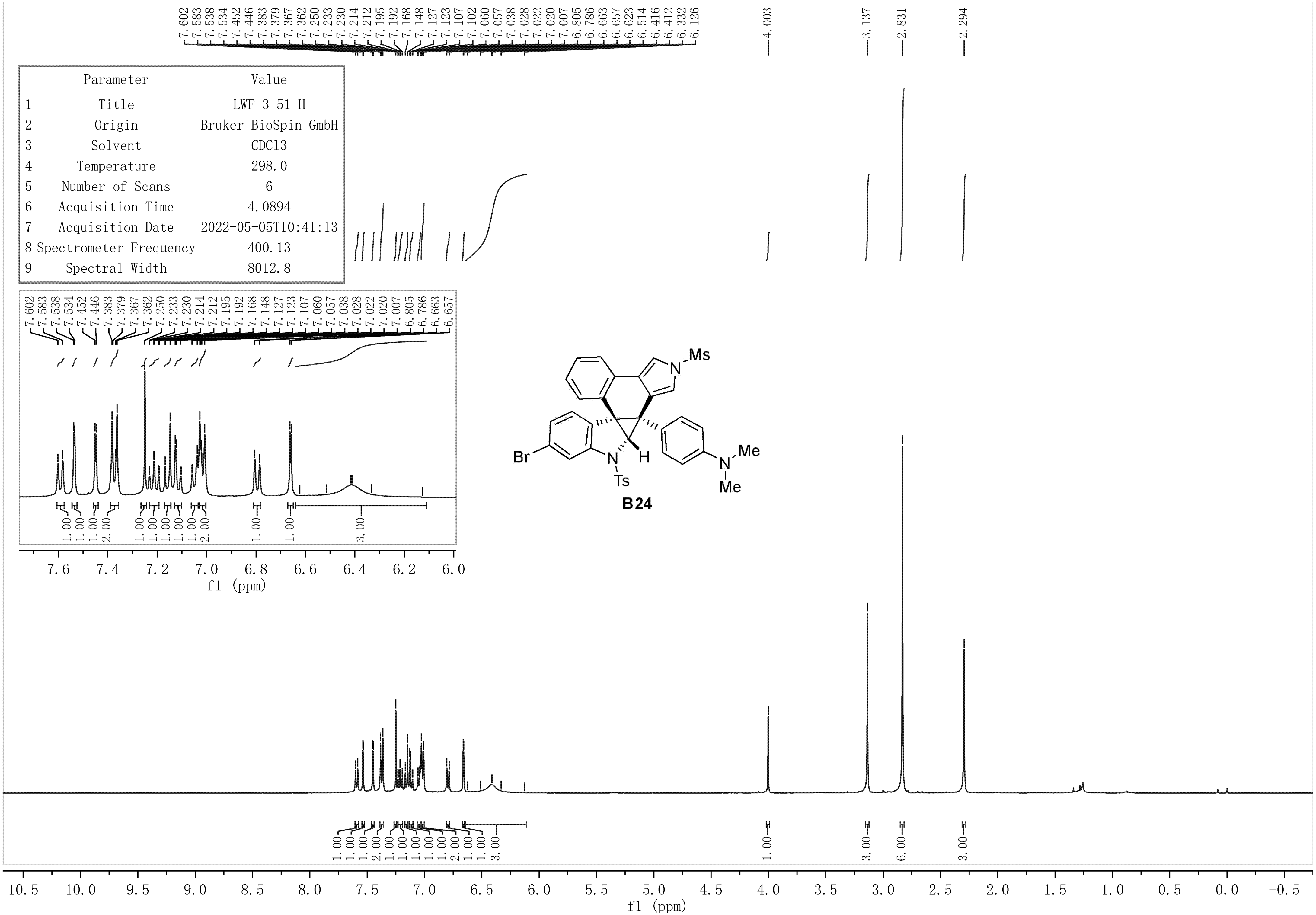

	Parameter	Value
1	Title	LWF-3-51-H
2	Origin	Bruker BioSpin GmbH
3	Solvent	CDCl3
4	Temperature	298.0
5	Number of Scans	6
6	Acquisition Time	4.0894
7	Acquisition Date	2022-05-05T10:41:13
8	Spectrometer Frequency	400.13
9	Spectral Width	8012.8

	Parameter	Value
1	Title	LWF-3-51-C
2	Origin	Bruker BioSpin GmbH
3	Solvent	CDCl3
4	Temperature	300.0
5	Number of Scans	102
6	Acquisition Time	1.3631
7	Acquisition Date	2022-05-05T10:43:39
8	Spectrometer Frequency	100.61
9	Spectral Width	24038.5

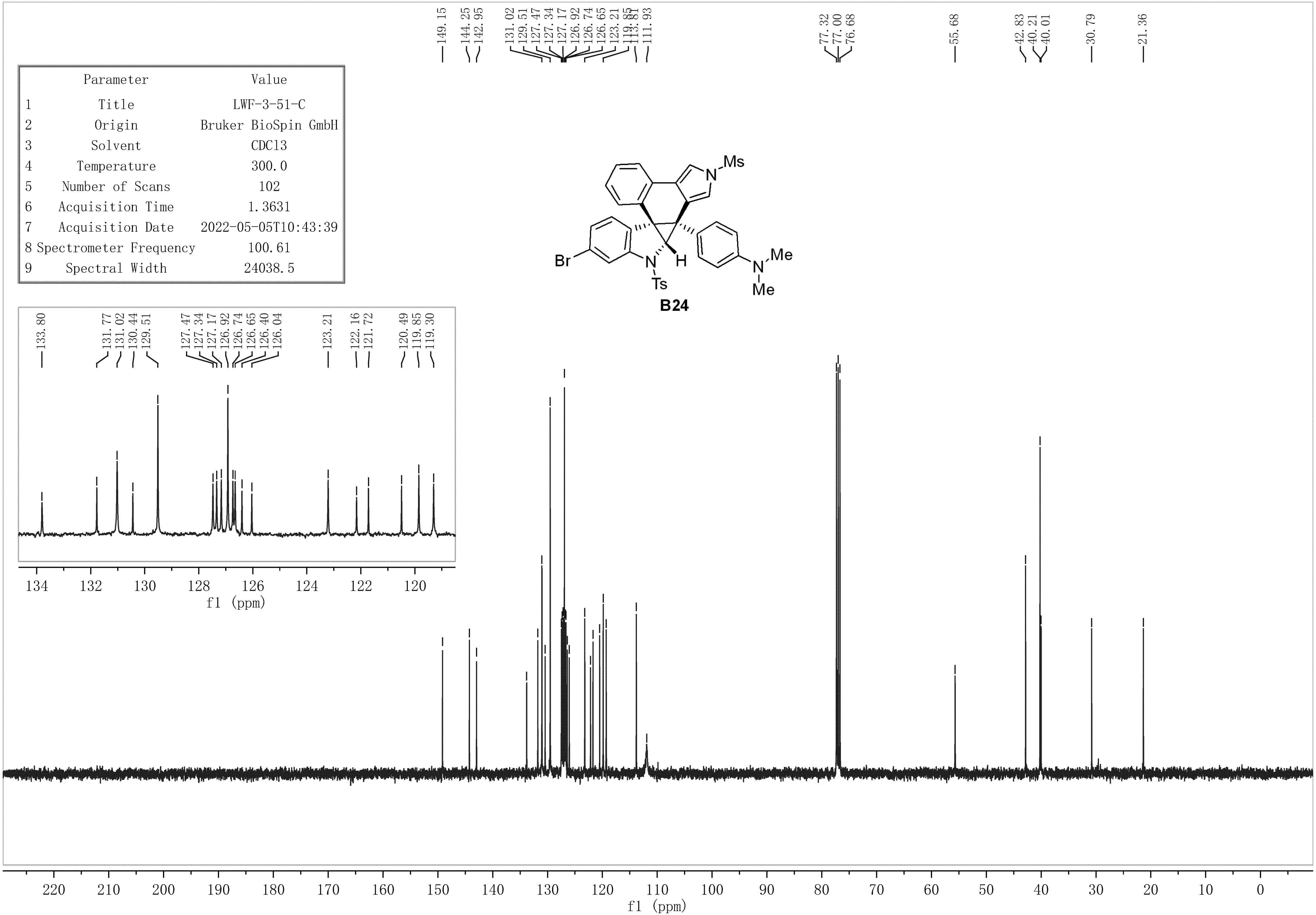

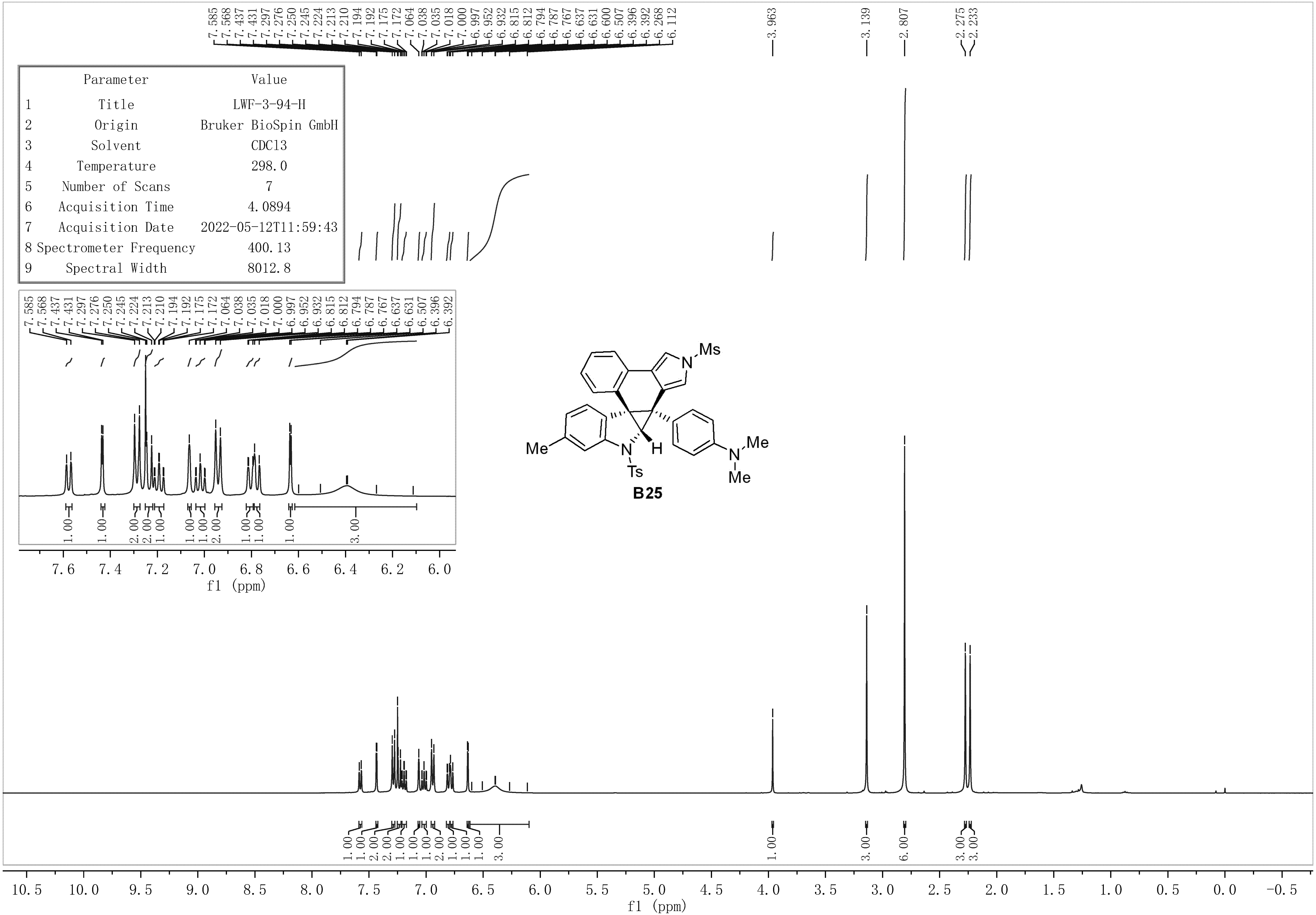
7.585 7.568 7.437 7.431 7.297 7.276 7.250 7.245 7.224 7.213 7.210 7.194 7.192 7.175 7.172 7.064 7.038 7.035 7.018 7.000 6.997 6.952 6.932 6.815 6.812 6.794 6.787 6.767 6.637 6.631 6.600 6.507 6.396 6.392 6.268 6.112
3.963
3.139
2.807
2.275 2.233
Parameter Value
1 Title LWF-3-94-H
2 Origin Bruker BioSpin GmbH
3 Solvent CDCl3
4 Temperature 298.0
5 Number of Scans 7
6 Acquisition Time 4.0894
7 Acquisition Date 2022-05-12T11:59:43
8 Spectrometer Frequency 400.13
9 Spectral Width 8012.8
Ms
N
Me
N
H
N
Me
Ts
Me
B25
1.00 1.00 2.00 2.00 1.00 1.00 1.00 2.00 1.00 1.00 1.00 3.00
1.00
3.00
6.00
3.00 3.00
f1 (ppm)

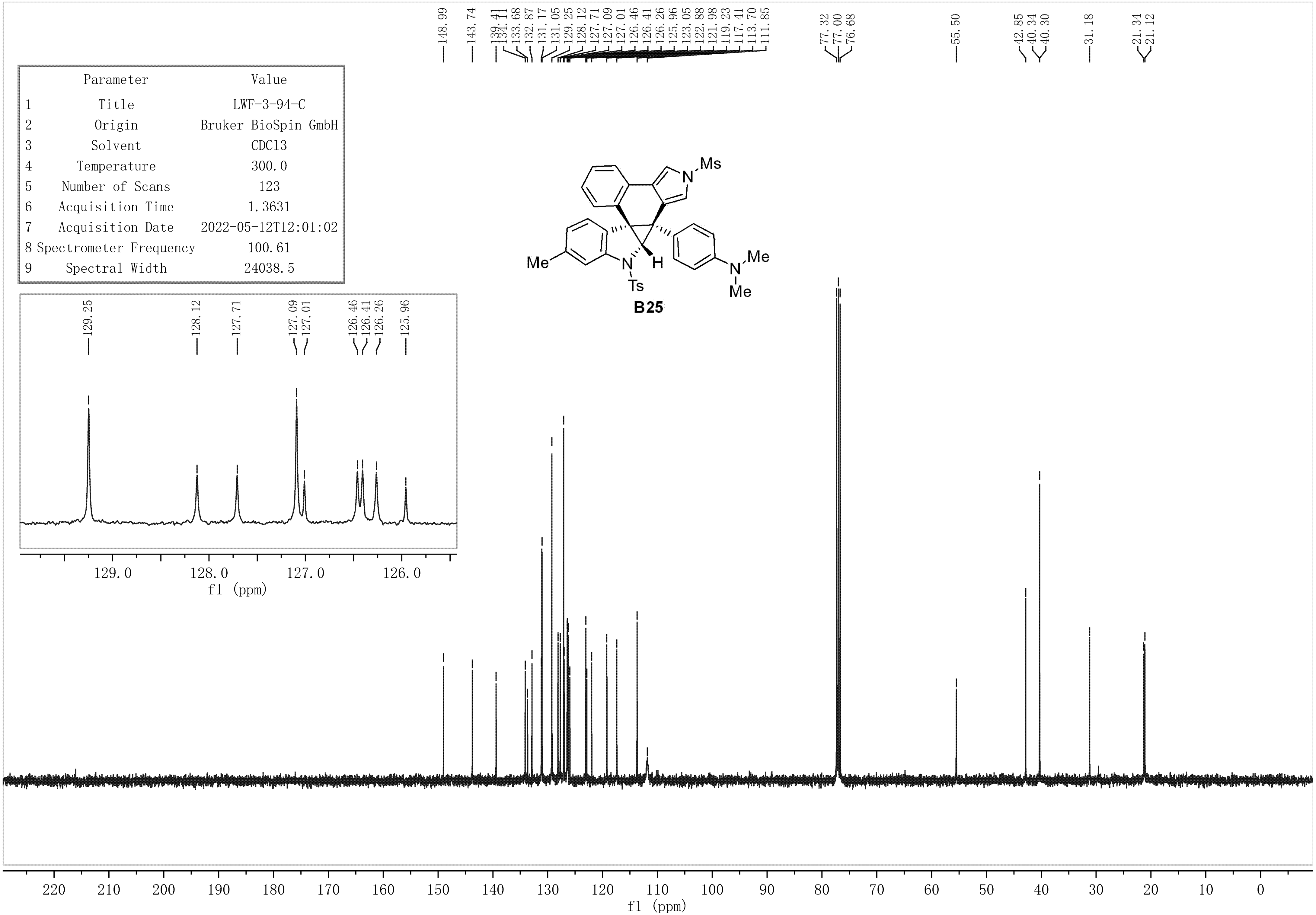
148.99
143.74
139.41
134.11
133.68
132.87
131.17
131.05
129.25
128.12
127.71
127.09
127.01
126.46
126.41
126.26
125.96
123.05
122.88
121.98
119.23
117.41
113.70
111.85
77.32
77.00
76.68
55.50
42.85
40.34
40.30
31.18
21.34
21.12
Parameter Value
1 Title LWF-3-94-C
2 Origin Bruker BioSpin GmbH
3 Solvent CDCl3
4 Temperature 300.0
5 Number of Scans 123
6 Acquisition Time 1.3631
7 Acquisition Date 2022-05-12T12:01:02
8 Spectrometer Frequency 100.61
9 Spectral Width 24038.5
Ms
N
Me
N
Ts
H
N
Me
Me
B25
f1 (ppm)
f1 (ppm)

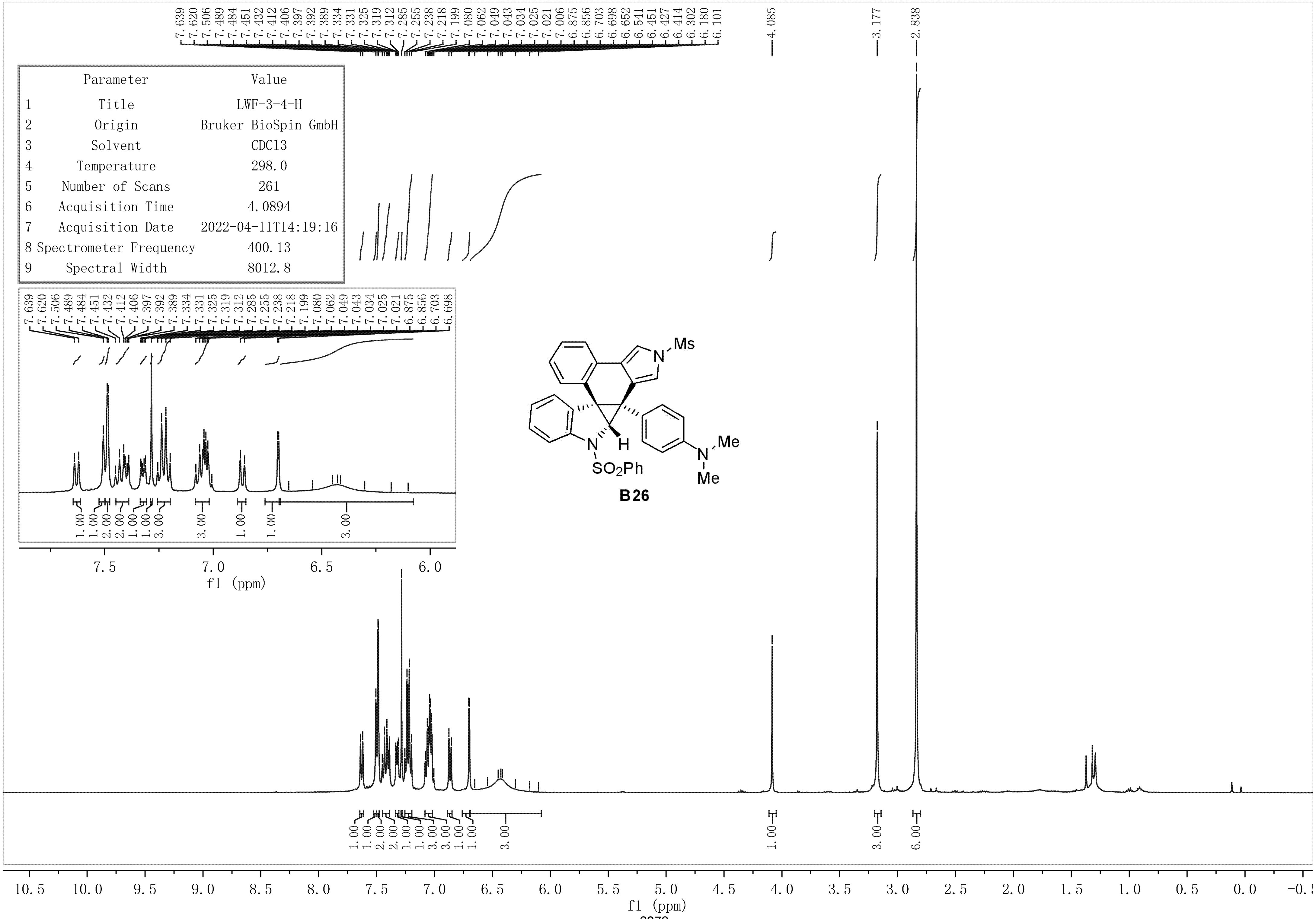
Parameter Value
1 Title LWF-3-4-H
2 Origin Bruker BioSpin GmbH
3 Solvent CDCl3
4 Temperature 298.0
5 Number of Scans 261
6 Acquisition Time 4.0894
7 Acquisition Date 2022-04-11T14:19:16
8 Spectrometer Frequency 400.13
9 Spectral Width 8012.8
7.639 7.620 7.506 7.489 7.484 7.451 7.432 7.412 7.406 7.397 7.392 7.389 7.334 7.331 7.325 7.319 7.312 7.285 7.255 7.238 7.218 7.199 7.080 7.062 7.049 7.043 7.034 7.025 7.021 7.006 6.875 6.856 6.703 6.698 6.652 6.541 6.451 6.427 6.414 6.302 6.180 6.101
4.085
3.177
2.838
1.00 1.00 2.00 2.00 1.00 3.00 3.00 1.00 1.00 3.00
1.00
3.00
6.00
Ms
N
Me
N
SO2Ph
H
Me
B26
f1 (ppm)

	Parameter	Value
1	Title	LWF-3-4-C
2	Origin	Bruker BioSpin GmbH
3	Solvent	CDCl3
4	Temperature	300.0
5	Number of Scans	191
6	Acquisition Time	1.3631
7	Acquisition Date	2022-05-05T10:27:16
8	Spectrometer Frequency	100.61
9	Spectral Width	24038.5

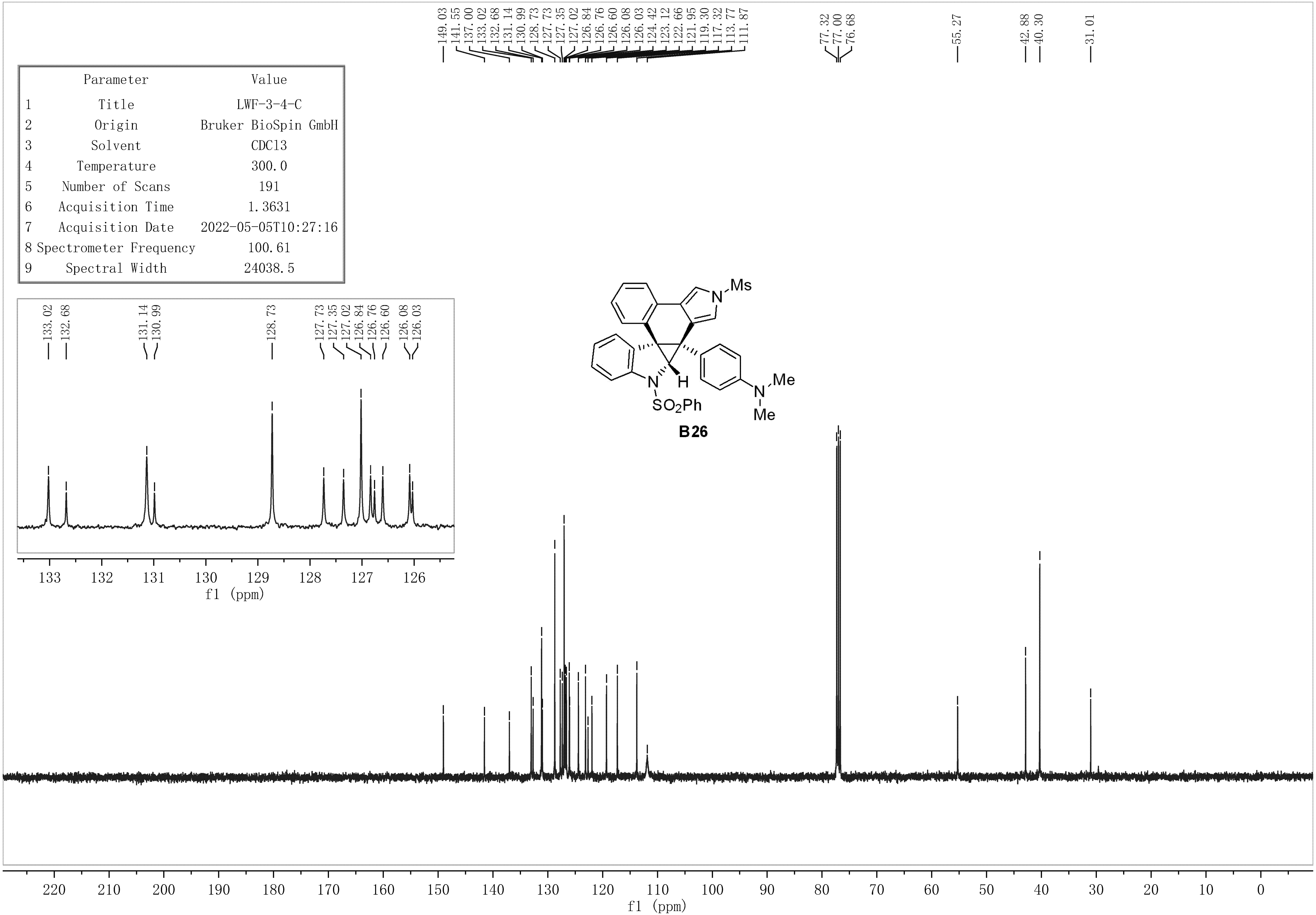

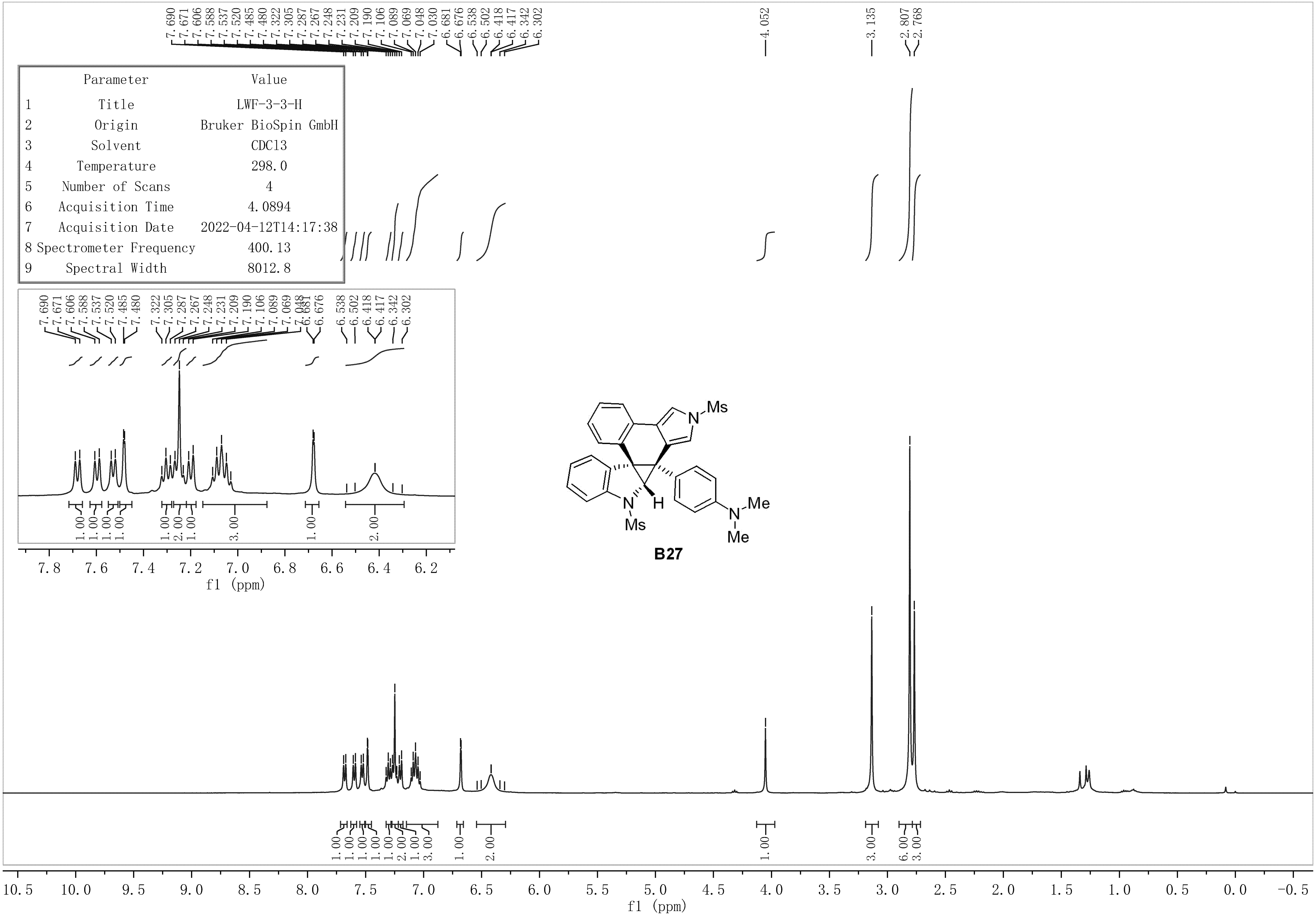
7.690 7.671 7.606 7.588 7.537 7.520 7.485 7.480 7.322 7.305 7.287 7.267 7.248 7.231 7.209 7.190 7.106 7.089 7.069 7.048 7.030 6.681 6.676 6.538 6.502 6.418 6.417 6.342 6.302
4.052
3.135
2.807 2.768
Parameter Value
1 Title LWF-3-3-H
2 Origin Bruker BioSpin GmbH
3 Solvent CDCl3
4 Temperature 298.0
5 Number of Scans 4
6 Acquisition Time 4.0894
7 Acquisition Date 2022-04-12T14:17:38
8 Spectrometer Frequency 400.13
9 Spectral Width 8012.8
N-Ms
N
Ms
H
N-Me
Me
B27
f1 (ppm)
1.00 1.00 1.00 1.00 1.00 2.00 1.00 3.00 1.00 2.00 1.00 3.00 6.00 3.00
10.5 10.0 9.5 9.0 8.5 8.0 7.5 7.0 6.5 6.0 5.5 5.0 4.5 4.0 3.5 3.0 2.5 2.0 1.5 1.0 0.5 0.0 -0.5

	Parameter	Value
1	Title	LWF-3-3-C
2	Origin	Bruker BioSpin GmbH
3	Solvent	CDCl3
4	Temperature	300.0
5	Number of Scans	49
6	Acquisition Time	1.3631
7	Acquisition Date	2022-04-12T14:18:41
8	Spectrometer Frequency	100.61
9	Spectral Width	24038.5

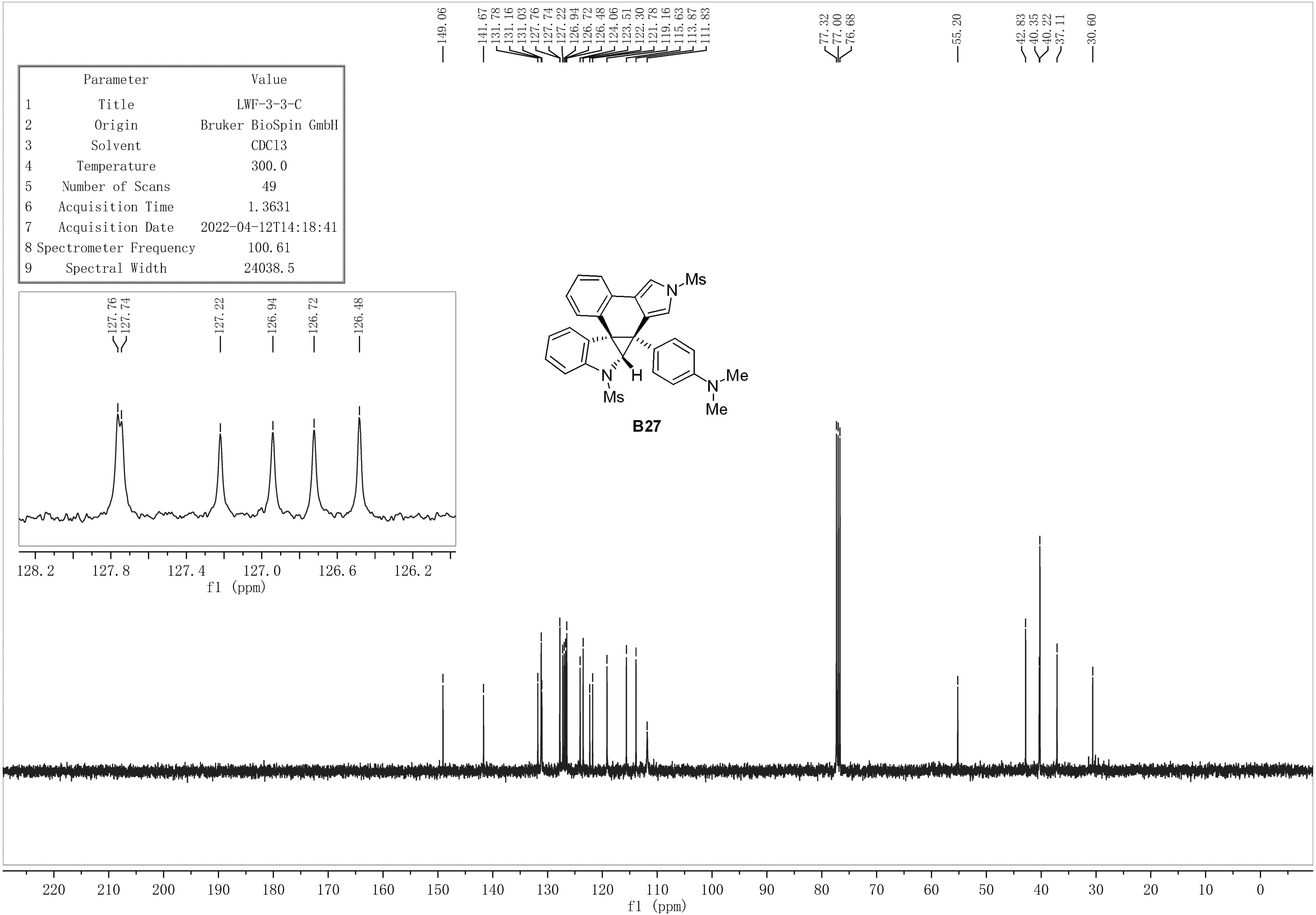

B27

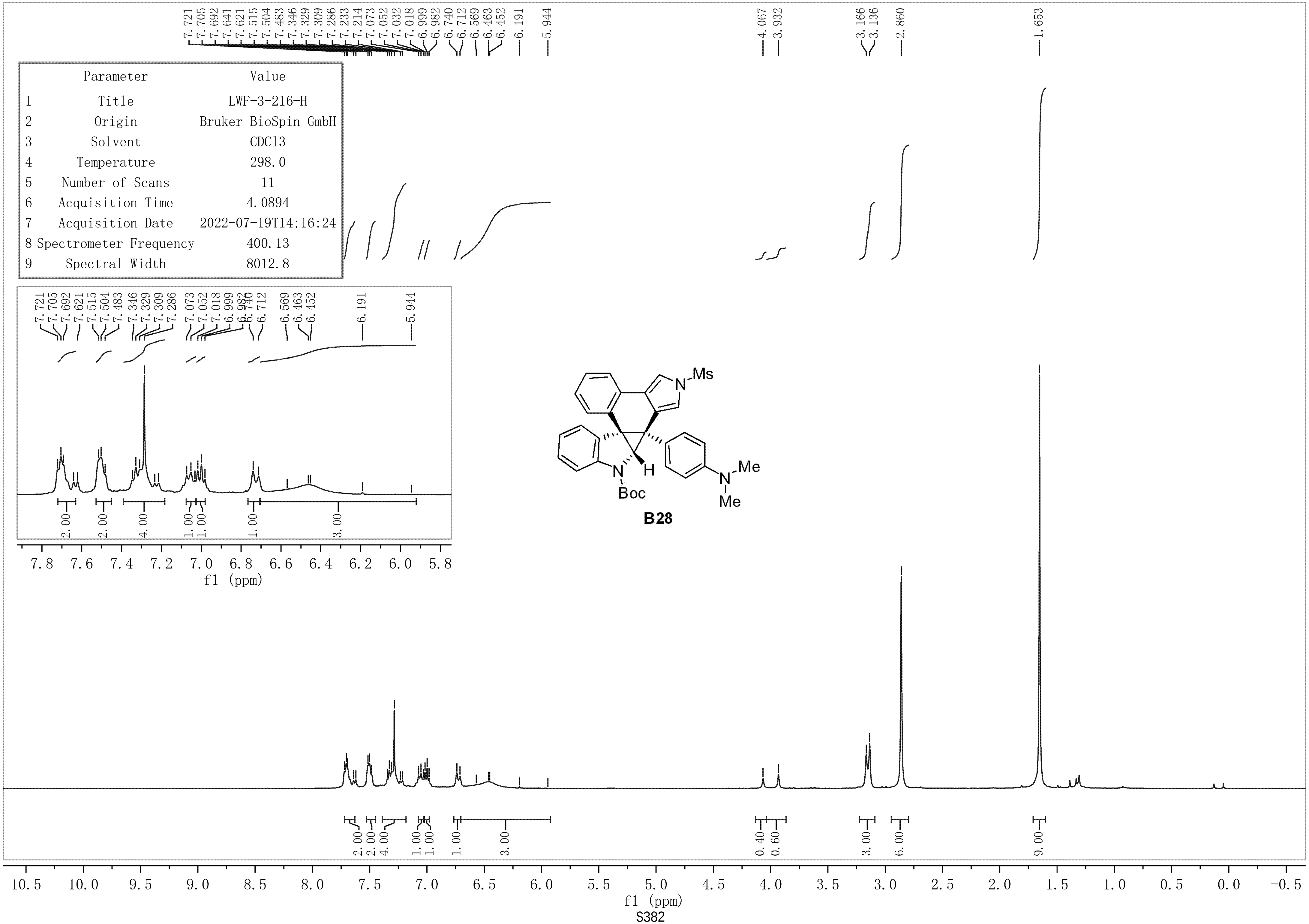

	Parameter	Value
1	Title	LWF-3-216-H
2	Origin	Bruker BioSpin GmbH
3	Solvent	CDCl3
4	Temperature	298.0
5	Number of Scans	11
6	Acquisition Time	4.0894
7	Acquisition Date	2022-07-19T14:16:24
8	Spectrometer Frequency	400.13
9	Spectral Width	8012.8

B28

	Parameter	Value
1	Title	LWF-Boc-C
2	Origin	Bruker BioSpin GmbH
3	Solvent	CDCl3
4	Temperature	298.2
5	Number of Scans	359
6	Acquisition Time	1.1010
7	Acquisition Date	2024-11-10T21:07:52
8	Spectrometer Frequency	125.77
9	Spectral Width	29761.9

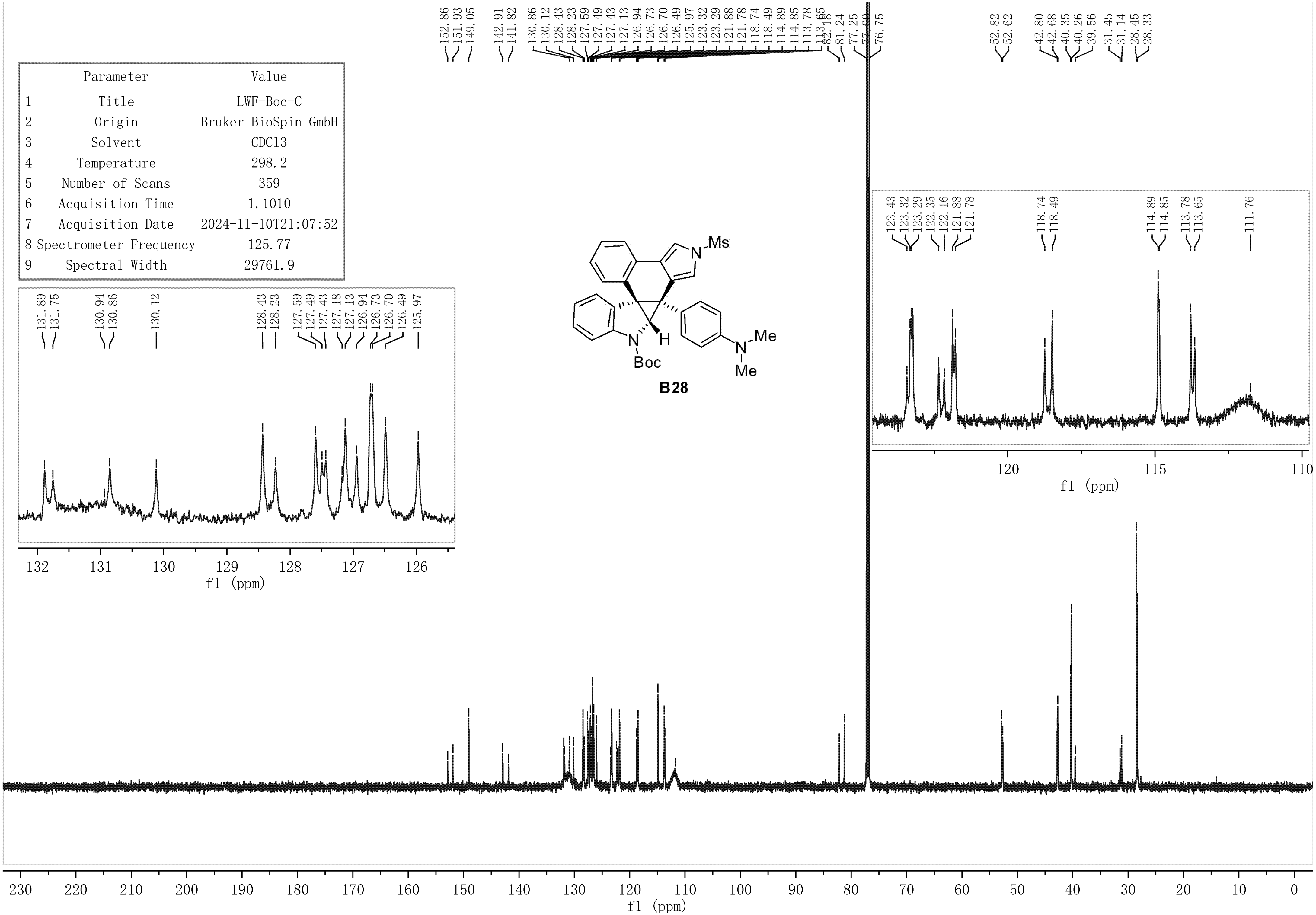

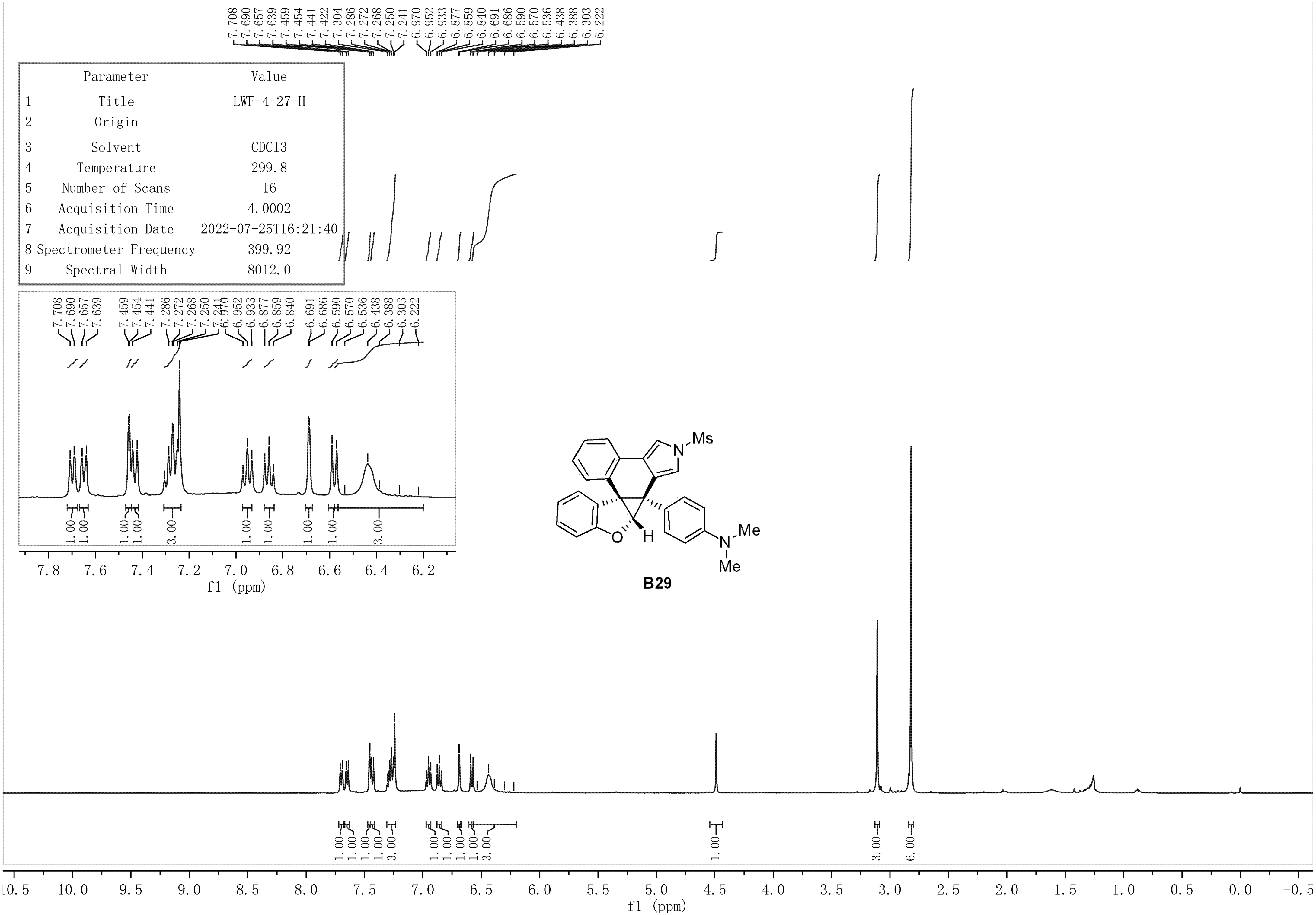
7.708 7.690 7.657 7.639 7.459 7.454 7.441 7.422 7.304 7.286 7.272 7.268 7.250 7.241 6.970 6.952 6.933 6.877 6.859 6.840 6.691 6.686 6.590 6.570 6.536 6.438 6.388 6.303 6.222
Parameter Value
1 Title LWF-4-27-H
2 Origin
3 Solvent CDCl3
4 Temperature 299.8
5 Number of Scans 16
6 Acquisition Time 4.0002
7 Acquisition Date 2022-07-25T16:21:40
8 Spectrometer Frequency 399.92
9 Spectral Width 8012.0
Ms
N
O
H
N
Me
Me
B29
1.00 1.00 1.00 1.00 3.00 1.00 1.00 1.00 1.00 3.00 1.00 3.00 6.00
f1 (ppm)

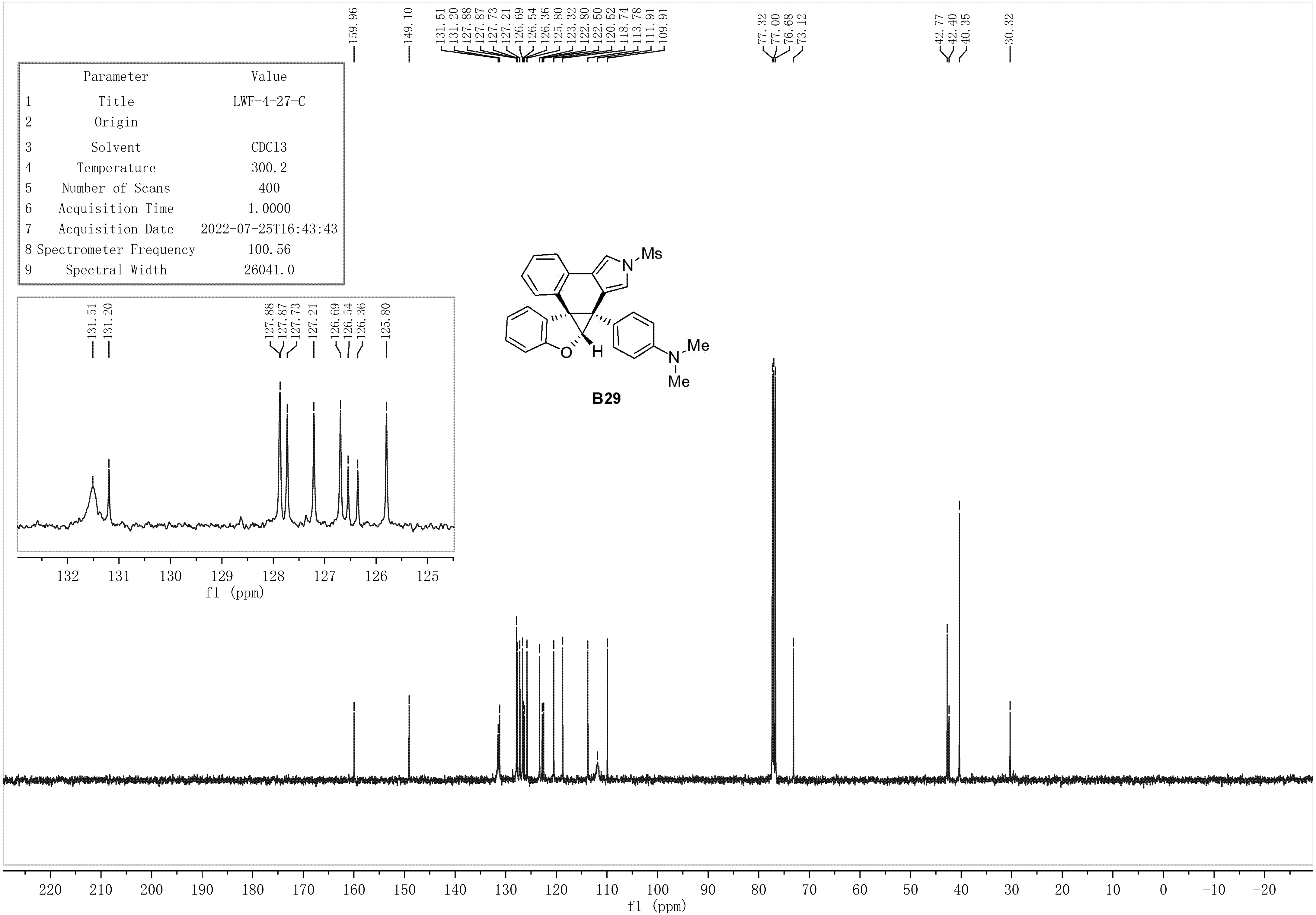
159.96
149.10
131.51
131.20
127.88
127.87
127.73
127.21
126.69
126.54
126.36
125.80
123.32
122.80
122.50
120.52
118.74
113.78
111.91
109.91
77.32
77.00
76.68
73.12
42.77
42.40
40.35
30.32
Parameter Value
1 Title LWF-4-27-C
2 Origin
3 Solvent CDCl3
4 Temperature 300.2
5 Number of Scans 400
6 Acquisition Time 1.0000
7 Acquisition Date 2022-07-25T16:43:43
8 Spectrometer Frequency 100.56
9 Spectral Width 26041.0
N
Ms
O
H
N
Me
Me
B29
f1 (ppm)
f1 (ppm)

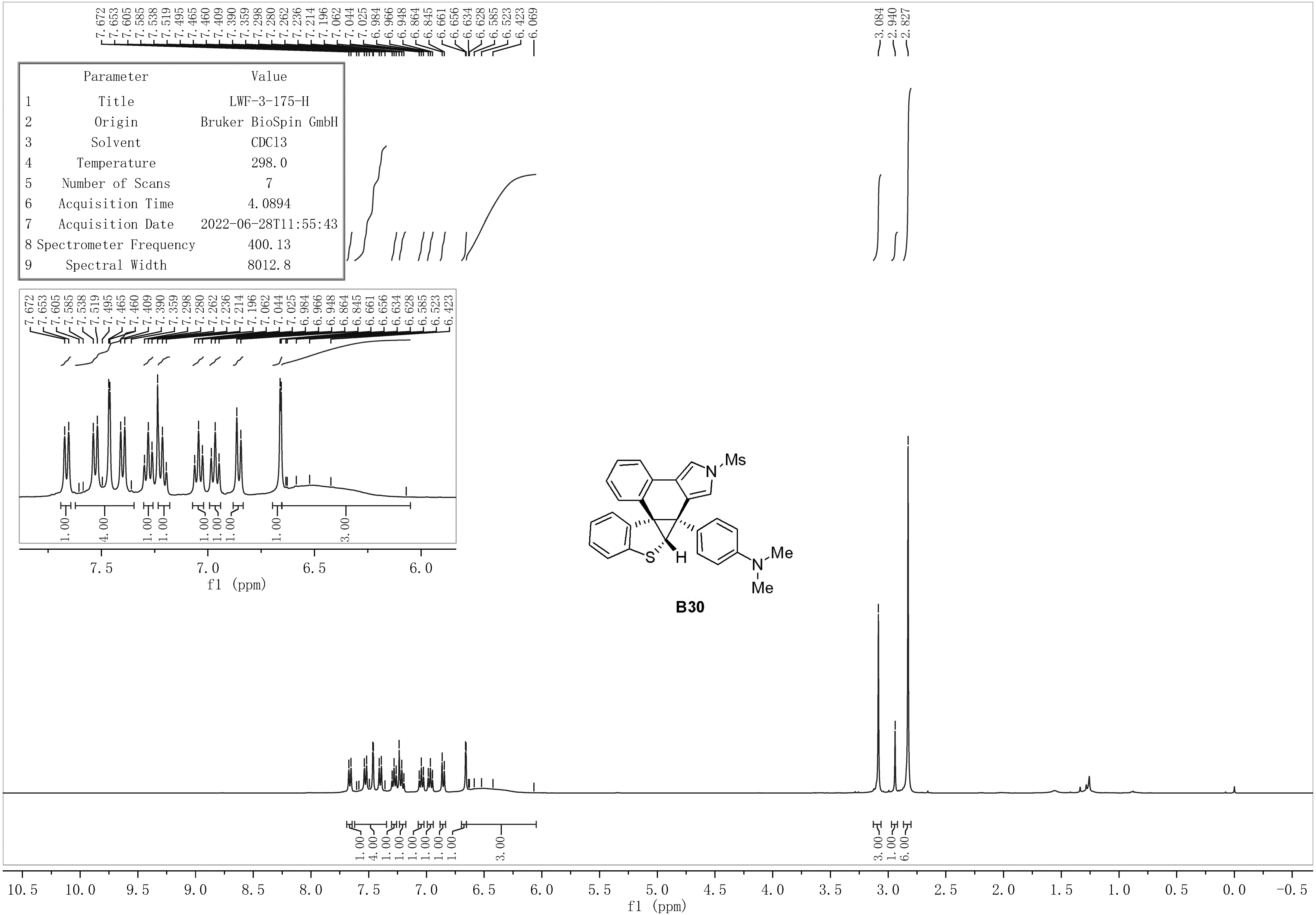
7.672 7.653 7.605 7.585 7.538 7.519 7.495 7.465 7.460 7.409 7.390 7.359 7.298 7.280 7.262 7.236 7.214 7.196 7.062 7.044 7.025 6.984 6.966 6.948 6.864 6.845 6.661 6.656 6.634 6.628 6.585 6.523 6.423 6.069
3.084 2.940 2.827
Parameter Value
1 Title LWF-3-175-H
2 Origin Bruker BioSpin GmbH
3 Solvent CDCl3
4 Temperature 298.0
5 Number of Scans 7
6 Acquisition Time 4.0894
7 Acquisition Date 2022-06-28T11:55:43
8 Spectrometer Frequency 400.13
9 Spectral Width 8012.8
1.00 4.00 1.00 1.00 1.00 1.00 1.00 1.00 3.00
7.5 7.0 6.5 6.0
f1 (ppm)
Ms
N
S
H
N
Me
Me
B30
3.00 1.00 6.00
10.5 10.0 9.5 9.0 8.5 8.0 7.5 7.0 6.5 6.0 5.5 5.0 4.5 4.0 3.5 3.0 2.5 2.0 1.5 1.0 0.5 0.0 -0.5
f1 (ppm)

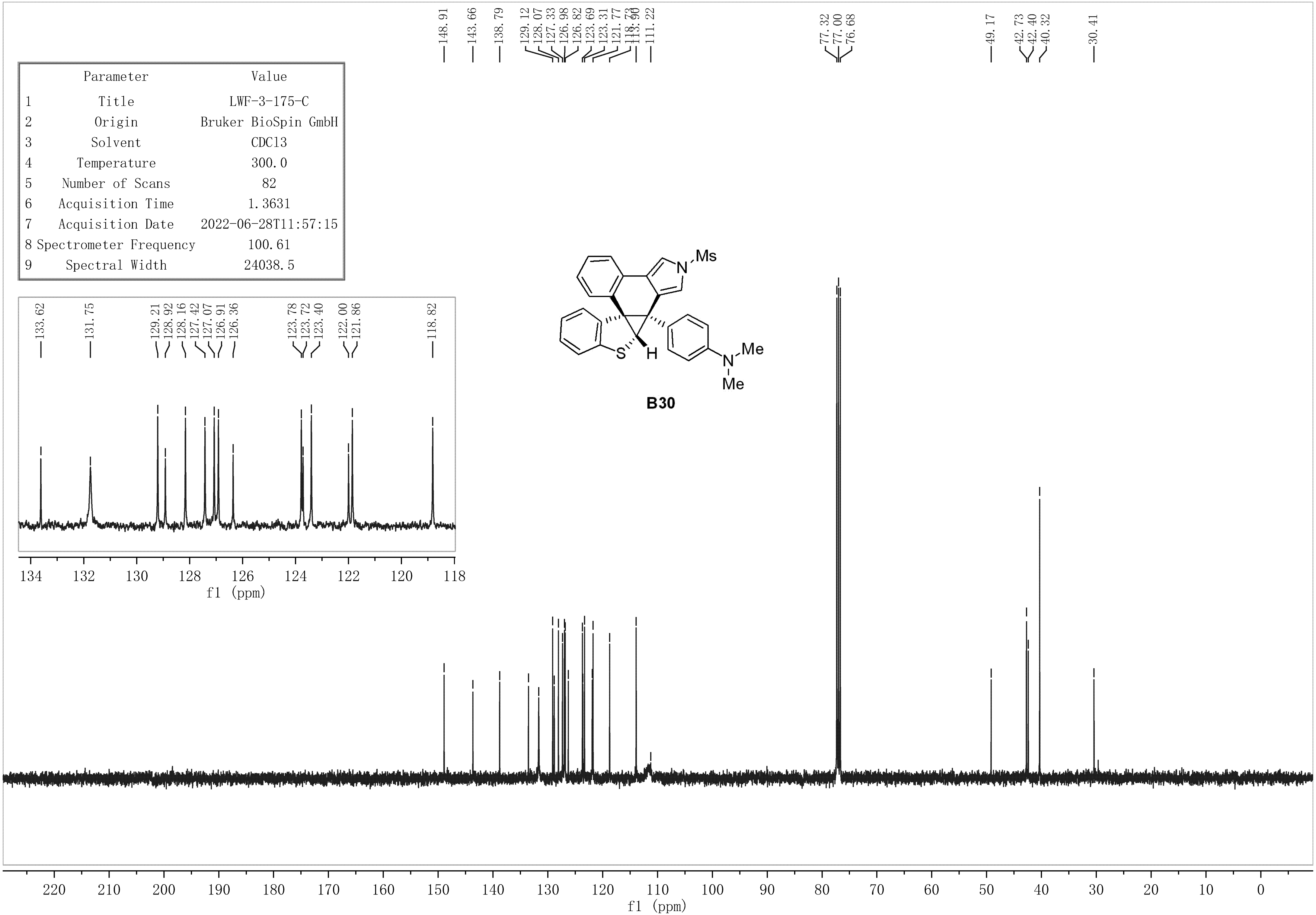

	Parameter	Value
1	Title	LWF-3-175-C
2	Origin	Bruker BioSpin GmbH
3	Solvent	CDCl3
4	Temperature	300.0
5	Number of Scans	82
6	Acquisition Time	1.3631
7	Acquisition Date	2022-06-28T11:57:15
8	Spectrometer Frequency	100.61
9	Spectral Width	24038.5

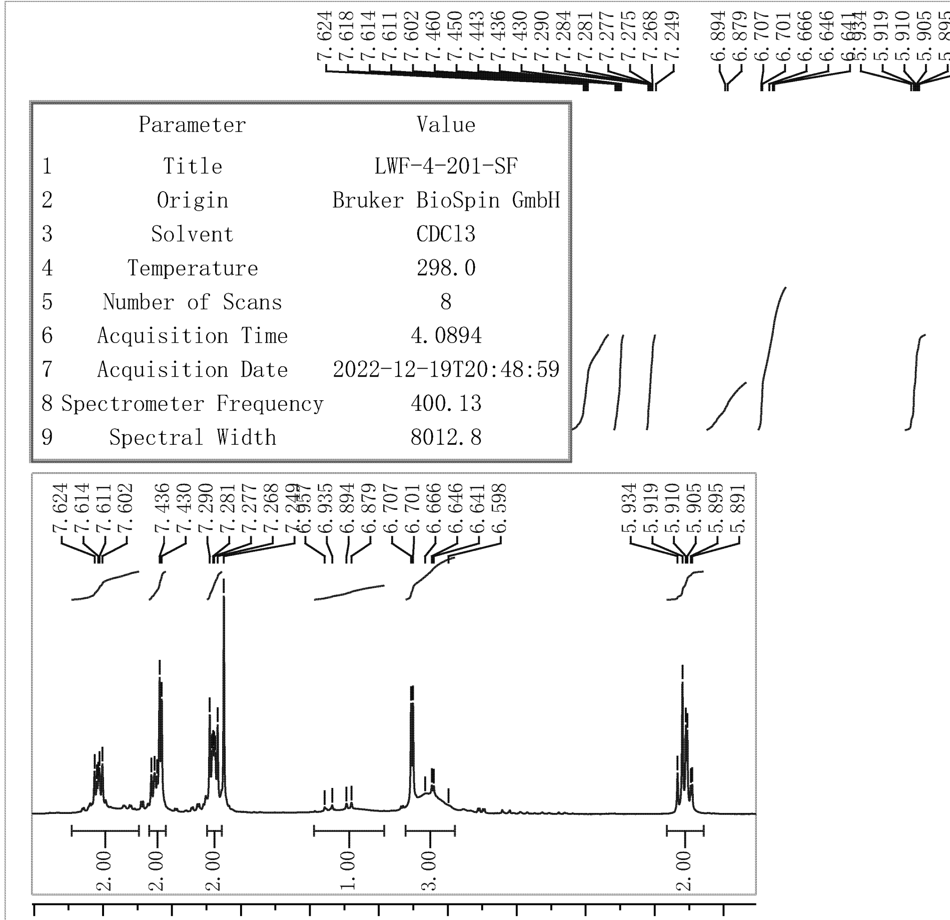
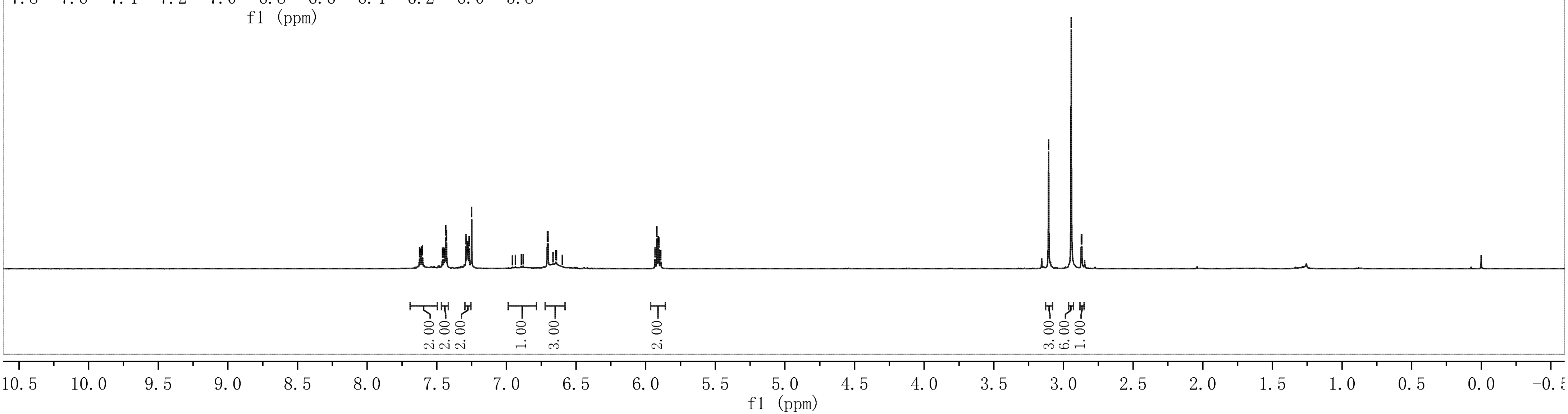

	Parameter	Value
1	Title	LWF-4-201-SF
2	Origin	Bruker BioSpin GmbH
3	Solvent	CDCl3
4	Temperature	298.0
5	Number of Scans	8
6	Acquisition Time	4.0894
7	Acquisition Date	2022-12-19T20:48:59
8	Spectrometer Frequency	400.13
9	Spectral Width	8012.8

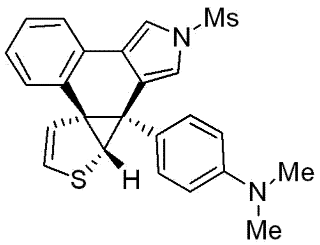

B31

	Parameter	Value
1	Title	LWF-4-201-SF-C
2	Origin	Bruker BioSpin GmbH
3	Solvent	CDCl3
4	Temperature	300.0
5	Number of Scans	409
6	Acquisition Time	1.3631
7	Acquisition Date	2022-12-19T20:50:29
8	Spectrometer Frequency	100.61
9	Spectral Width	24038.5

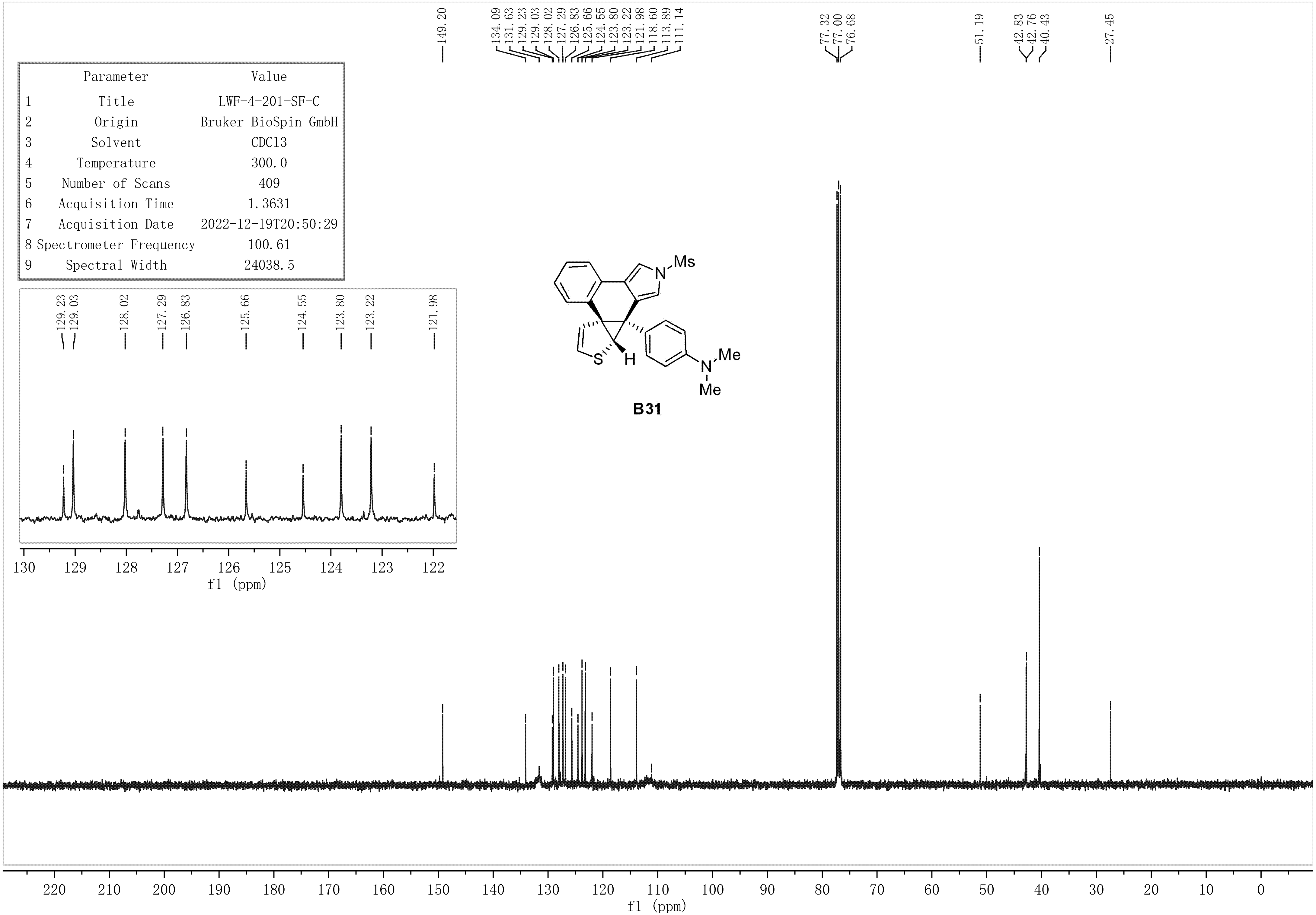

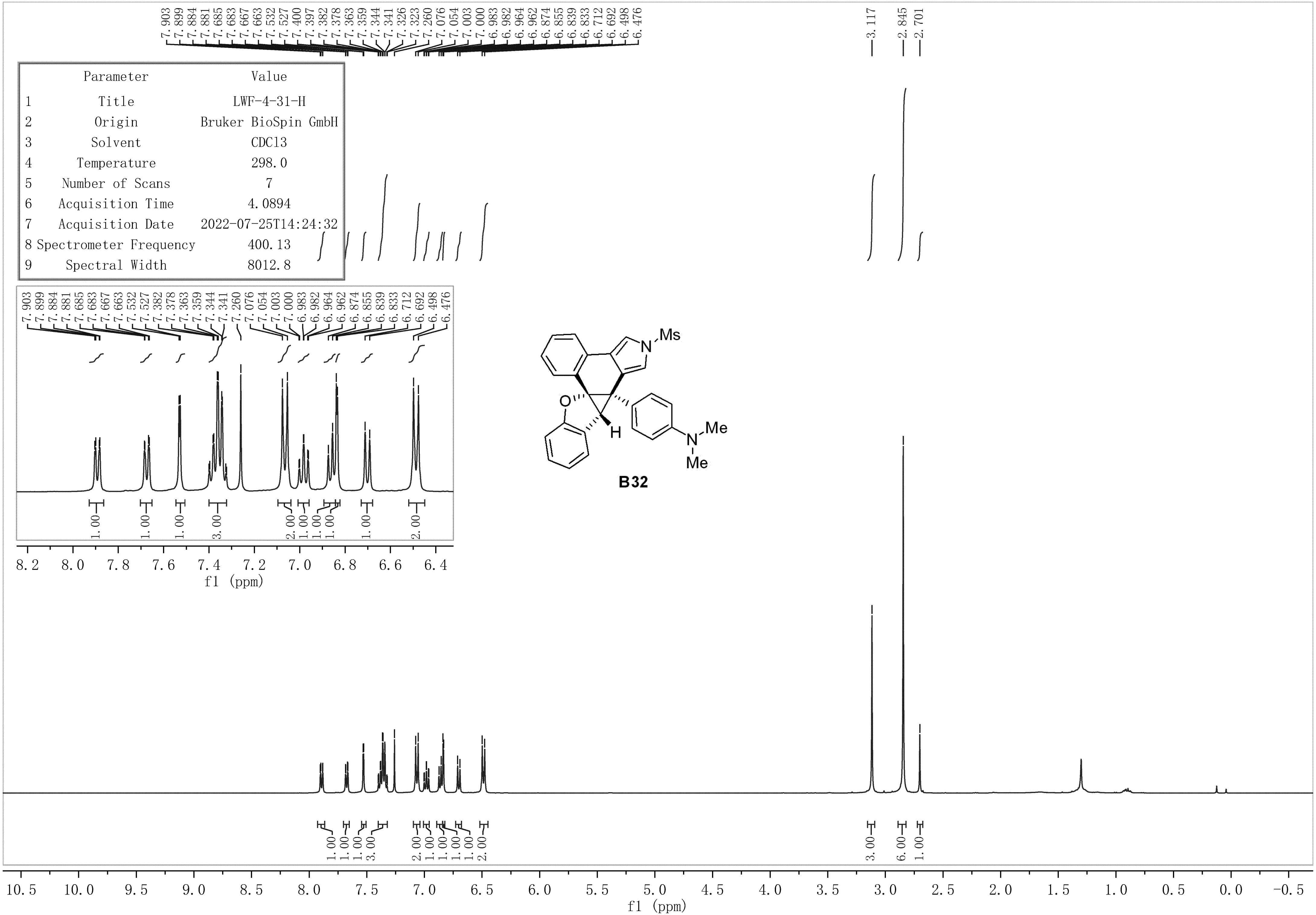
7.903 7.899 7.884 7.881 7.685 7.683 7.667 7.663 7.532 7.527 7.400 7.397 7.382 7.378 7.363 7.359 7.344 7.341 7.326 7.323 7.260 7.076 7.054 7.003 7.000 6.983 6.982 6.964 6.962 6.874 6.855 6.839 6.833 6.712 6.692 6.498 6.476
3.117 2.845 2.701
Parameter Value
1 Title LWF-4-31-H
2 Origin Bruker BioSpin GmbH
3 Solvent CDCl3
4 Temperature 298.0
5 Number of Scans 7
6 Acquisition Time 4.0894
7 Acquisition Date 2022-07-25T14:24:32
8 Spectrometer Frequency 400.13
9 Spectral Width 8012.8
Ms
N
O
H
N
Me
Me
B32
1.00 1.00 1.00 3.00 2.00 1.00 1.00 1.00 1.00 2.00
3.00 6.00 1.00
f1 (ppm)

	Parameter	Value
1	Title	LWF-4-31-C
2	Origin	Bruker BioSpin GmbH
3	Solvent	CDCl3
4	Temperature	300.0
5	Number of Scans	75
6	Acquisition Time	1.3631
7	Acquisition Date	2022-07-25T14:25:55
8	Spectrometer Frequency	100.61
9	Spectral Width	24038.5

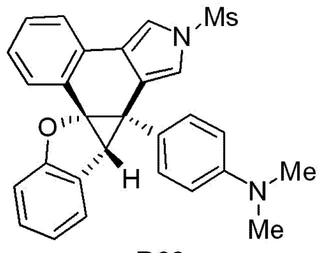

B32

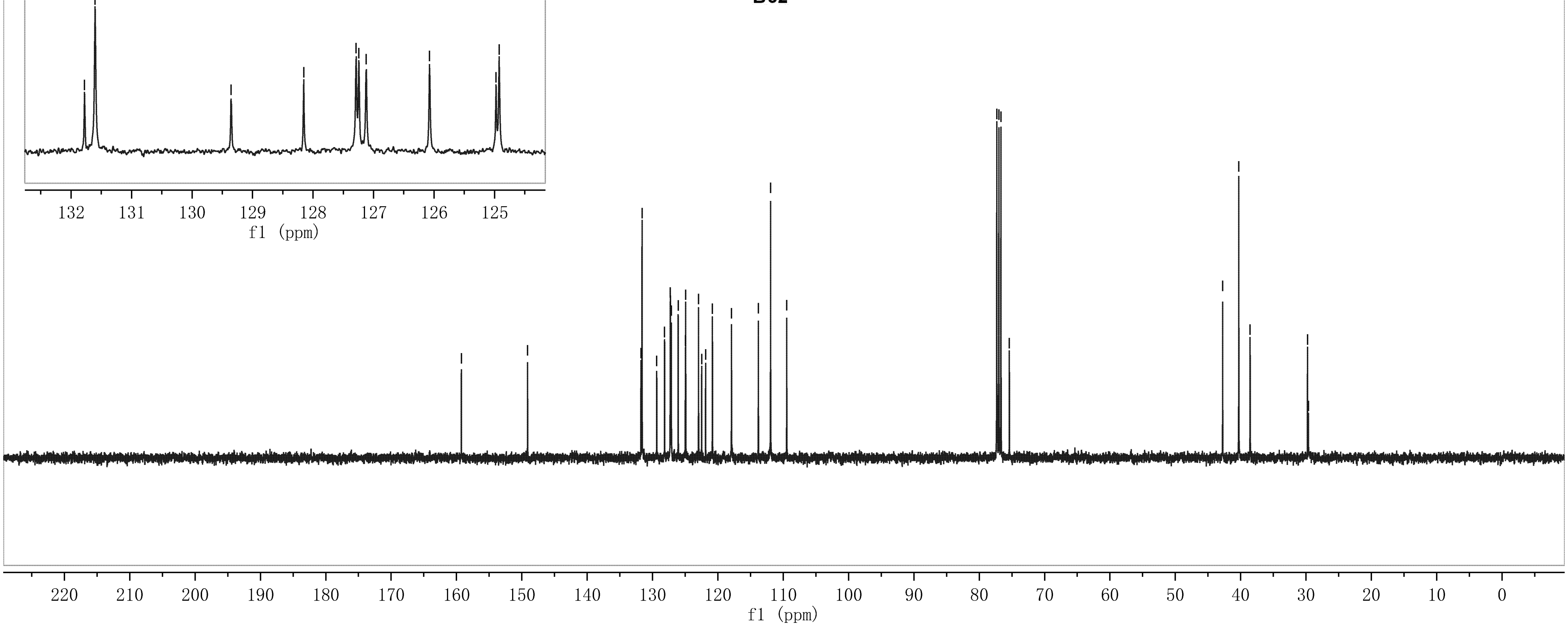

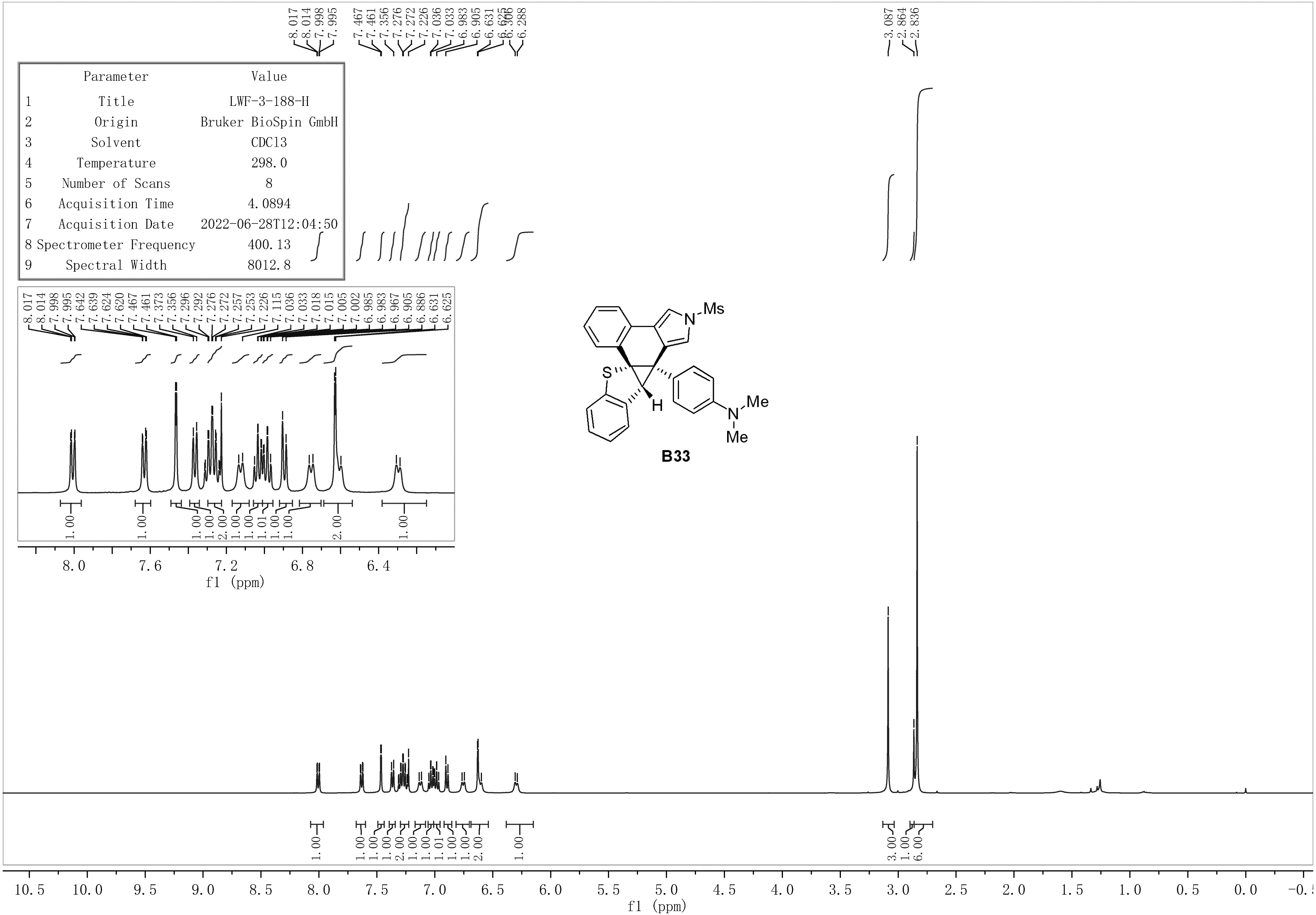
Parameter Value
1 Title LWF-3-188-H
2 Origin Bruker BioSpin GmbH
3 Solvent CDCl3
4 Temperature 298.0
5 Number of Scans 8
6 Acquisition Time 4.0894
7 Acquisition Date 2022-06-28T12:04:50
8 Spectrometer Frequency 400.13
9 Spectral Width 8012.8
8.017 8.014 7.998 7.995 7.467 7.461 7.356 7.276 7.272 7.226 7.036 7.033 6.983 6.905 6.631 6.625 6.306 6.288
3.087 2.864 2.836
8.017 8.014 7.998 7.995 7.642 7.639 7.624 7.620 7.467 7.461 7.373 7.356 7.296 7.292 7.276 7.272 7.257 7.253 7.226 7.115 7.036 7.033 7.018 7.015 7.005 7.002 6.985 6.983 6.967 6.905 6.886 6.631 6.625
1.00 1.00 1.00 1.00 2.00 1.00 1.00 1.01 1.00 1.00 2.00 1.00
1.00 1.00 1.00 1.00 2.00 1.00 1.00 1.01 1.00 1.00 2.00 1.00 3.00 1.00 6.00
f1 (ppm)
N-Ms
S
H
N
Me
Me
B33

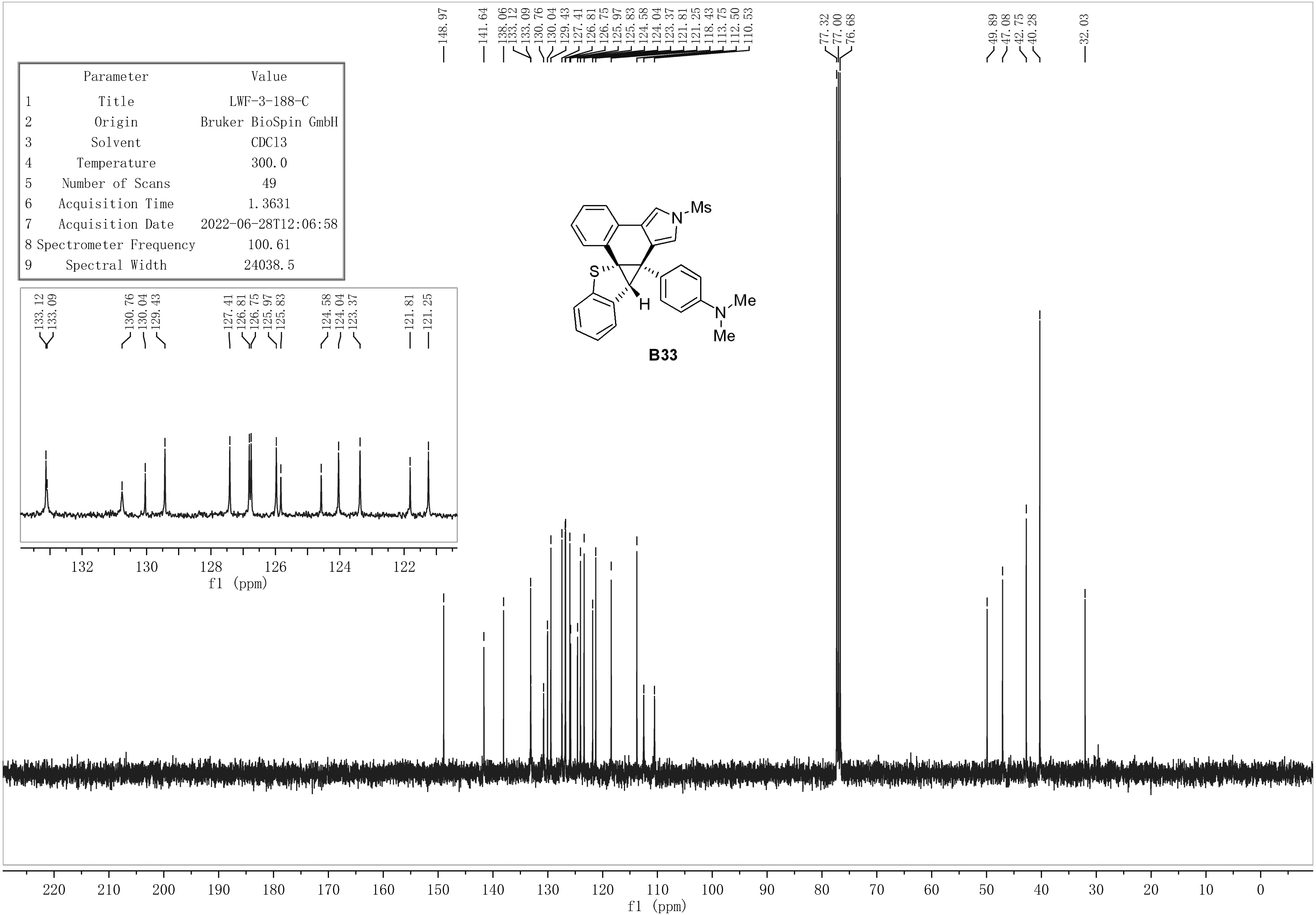

	Parameter	Value
1	Title	LWF-3-188-C
2	Origin	Bruker BioSpin GmbH
3	Solvent	CDCl3
4	Temperature	300.0
5	Number of Scans	49
6	Acquisition Time	1.3631
7	Acquisition Date	2022-06-28T12:06:58
8	Spectrometer Frequency	100.61
9	Spectral Width	24038.5

	Parameter	Value
1	Title	LWF-3-162-H
2	Origin	Bruker BioSpin GmbH
3	Solvent	CDCl3
4	Temperature	298.0
5	Number of Scans	9
6	Acquisition Time	4.0894
7	Acquisition Date	2022-06-04T20:03:29
8	Spectrometer Frequency	400.13
9	Spectral Width	8012.8

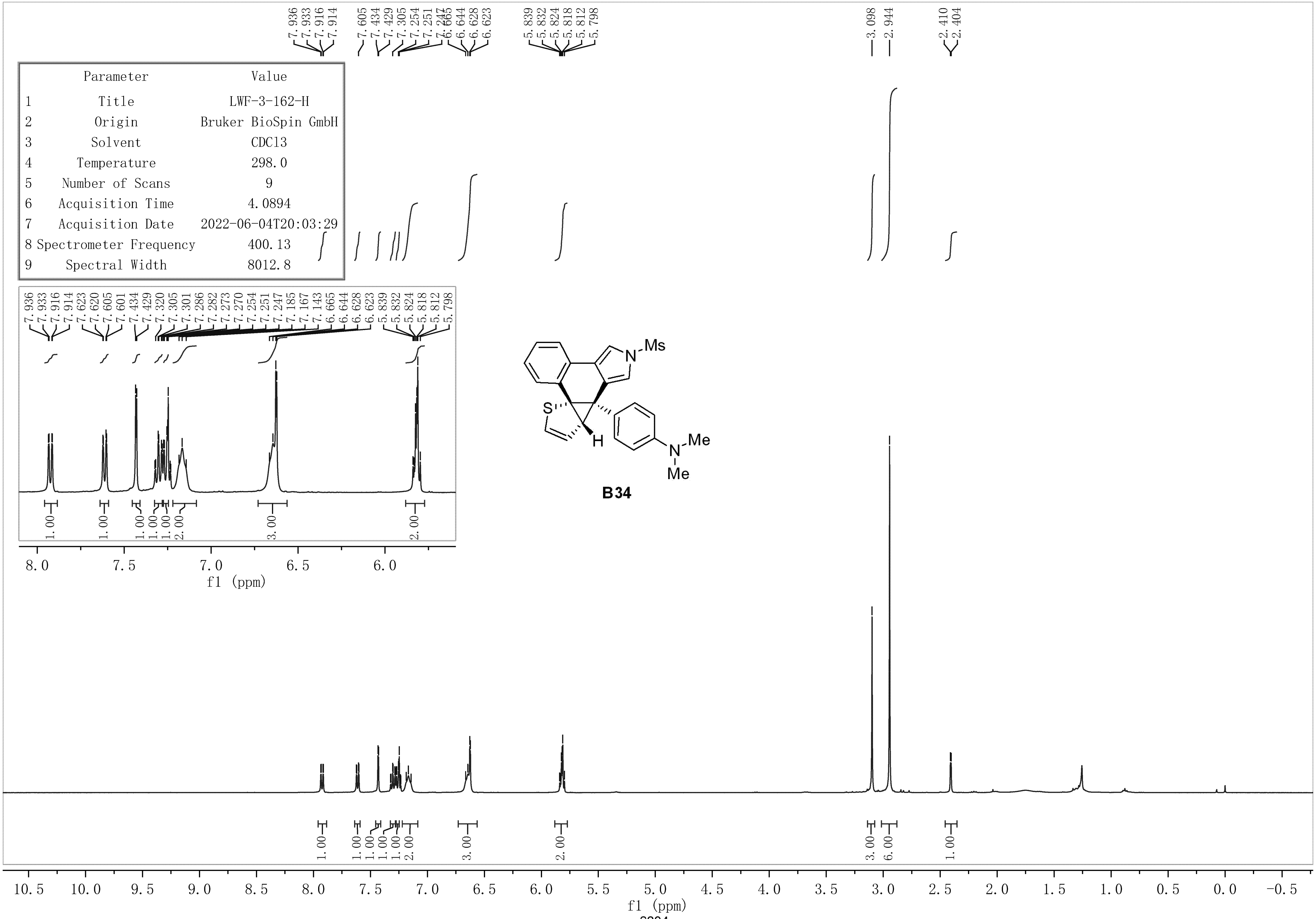

B34

	Parameter	Value
1	Title	LWF-3-162-C
2	Origin	Bruker BioSpin GmbH
3	Solvent	CDCl3
4	Temperature	300.0
5	Number of Scans	308
6	Acquisition Time	1.3631
7	Acquisition Date	2022-06-04T20:05:08
8	Spectrometer Frequency	100.61
9	Spectral Width	24038.5

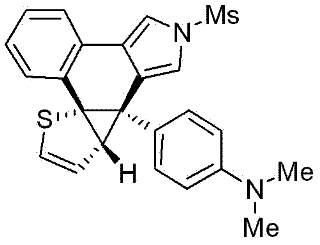

B34

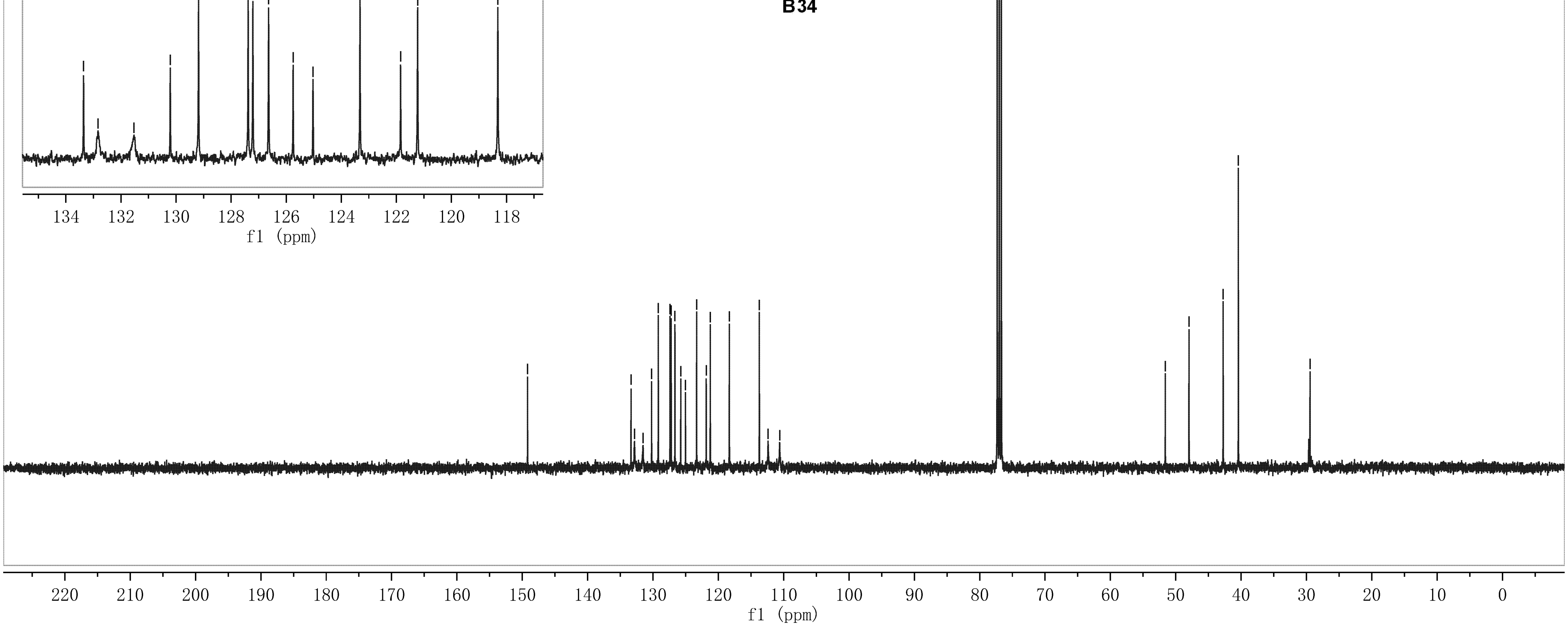

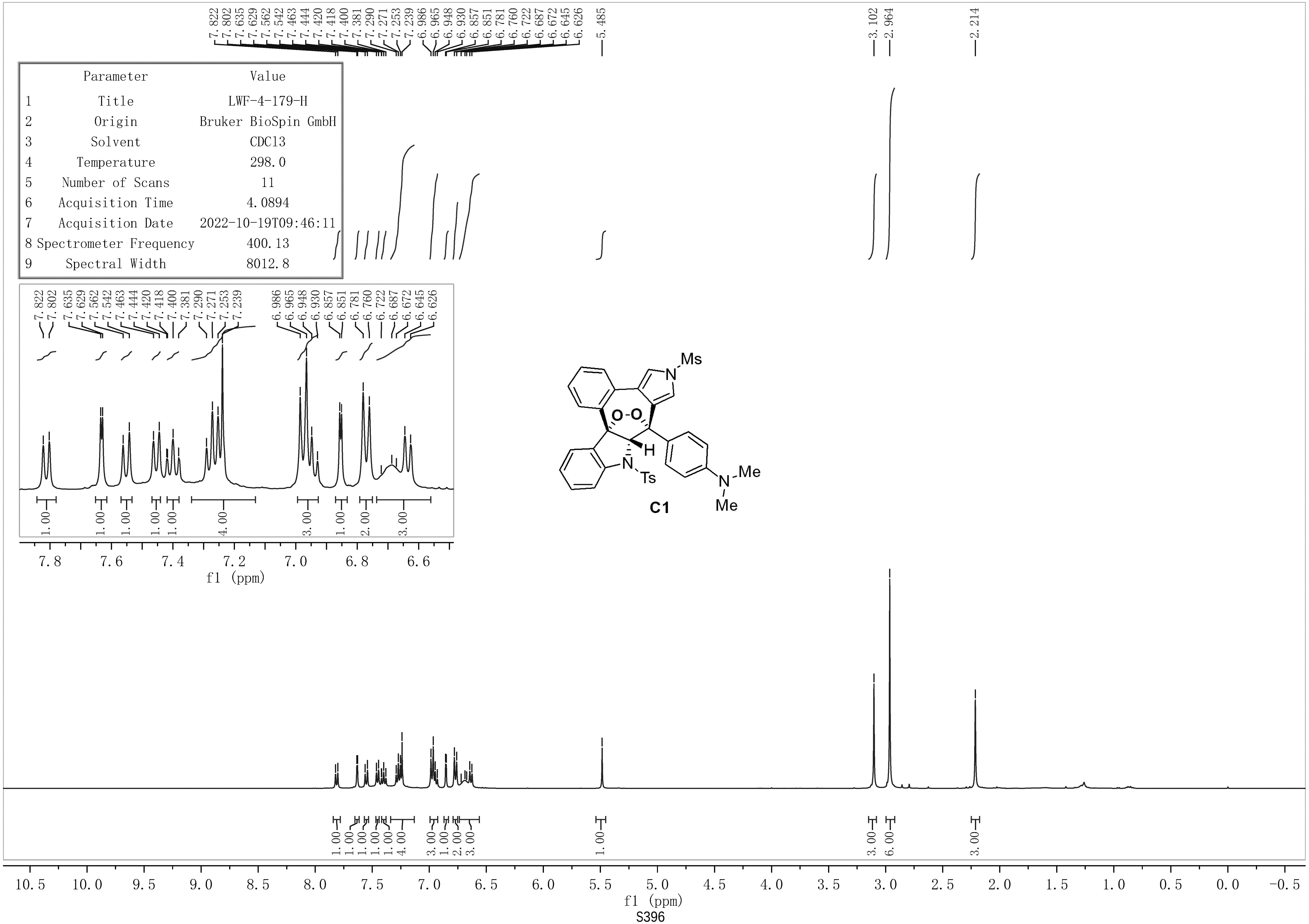
7.822 7.802 7.635 7.629 7.562 7.542 7.463 7.444 7.420 7.418 7.400 7.381 7.290 7.271 7.253 7.239 6.986 6.965 6.948 6.930 6.857 6.851 6.781 6.760 6.722 6.687 6.672 6.645 6.626
5.485
3.102 2.964
2.214
Parameter Value
1 Title LWF-4-179-H
2 Origin Bruker BioSpin GmbH
3 Solvent CDCl3
4 Temperature 298.0
5 Number of Scans 11
6 Acquisition Time 4.0894
7 Acquisition Date 2022-10-19T09:46:11
8 Spectrometer Frequency 400.13
9 Spectral Width 8012.8
1.00 1.00 1.00 1.00 1.00 4.00 3.00 1.00 2.00 3.00
7.8 7.6 7.4 7.2 7.0 6.8 6.6
f1 (ppm)
Ms
N
O-O
H
N
Ts
N
Me
Me
C1
1.00 1.00 1.00 1.00 1.00 4.00 3.00 1.00 2.00 3.00 1.00 3.00 6.00 3.00
10.5 10.0 9.5 9.0 8.5 8.0 7.5 7.0 6.5 6.0 5.5 5.0 4.5 4.0 3.5 3.0 2.5 2.0 1.5 1.0 0.5 0.0 -0.5
f1 (ppm)

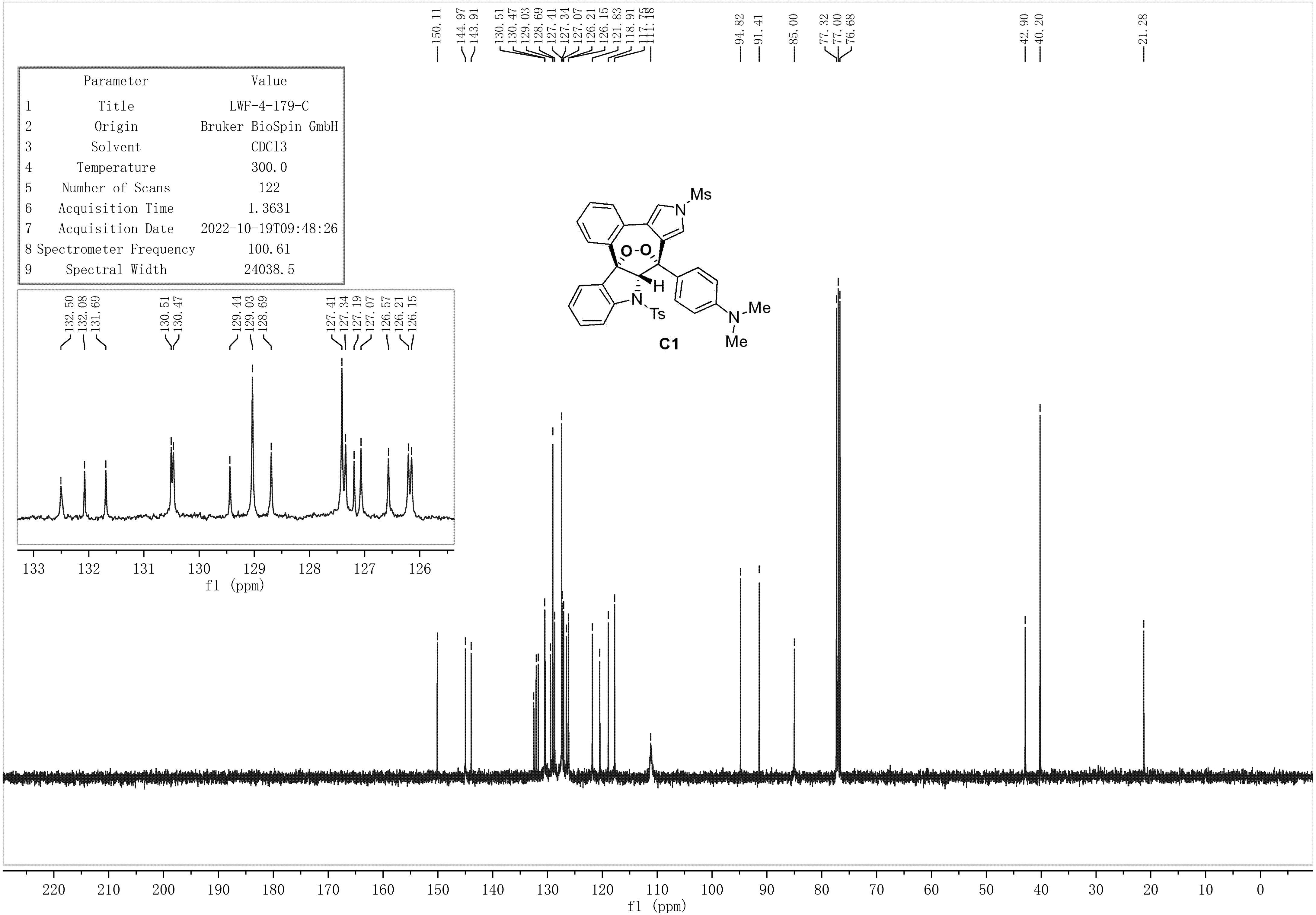

150.11
144.97
143.91
130.51
130.47
129.03
128.69
127.41
127.34
127.07
126.21
126.15
121.83
118.91
117.75
111.18
94.82
91.41
85.00
77.32
77.00
76.68
42.90
40.20
21.28
Parameter Value
1 Title LWF-4-179-C
2 Origin Bruker BioSpin GmbH
3 Solvent CDCl3
4 Temperature 300.0
5 Number of Scans 122
6 Acquisition Time 1.3631
7 Acquisition Date 2022-10-19T09:48:26
8 Spectrometer Frequency 100.61
9 Spectral Width 24038.5
132.50
132.08
131.69
130.51
130.47
129.44
129.03
128.69
127.41
127.34
127.19
127.07
126.57
126.21
126.15
f1 (ppm)
Ms
N
O-O
H
N
Ts
N
Me
Me
C1
f1 (ppm)

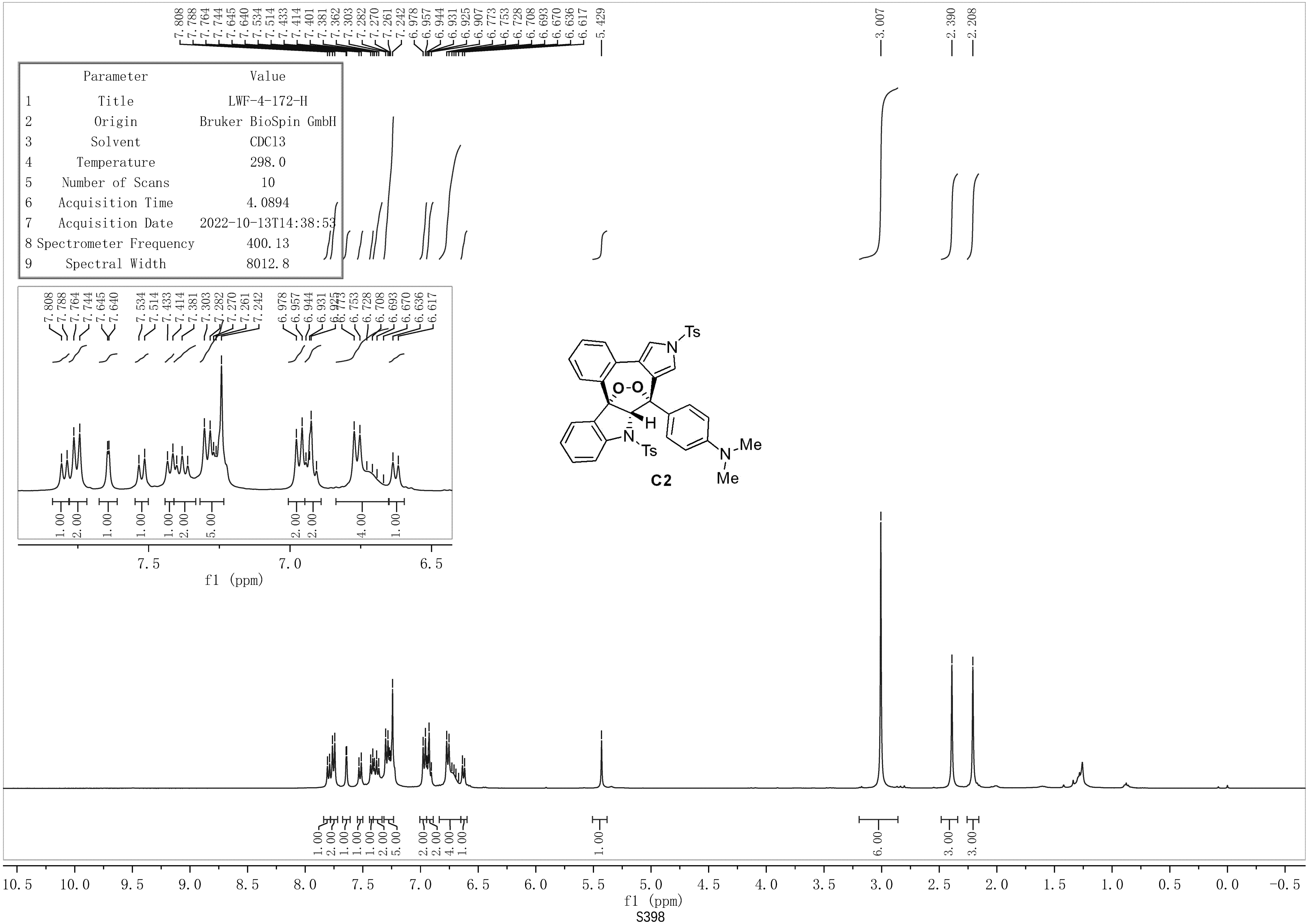
7.808 7.788 7.764 7.744 7.645 7.640 7.534 7.514 7.433 7.414 7.401 7.381 7.362 7.303 7.282 7.270 7.261 7.242 6.978 6.957 6.944 6.931 6.925 6.907 6.773 6.753 6.728 6.708 6.693 6.670 6.636 6.617 5.429 3.007 2.390 2.208
Parameter Value
1 Title LWF-4-172-H
2 Origin Bruker BioSpin GmbH
3 Solvent CDCl3
4 Temperature 298.0
5 Number of Scans 10
6 Acquisition Time 4.0894
7 Acquisition Date 2022-10-13T14:38:53
8 Spectrometer Frequency 400.13
9 Spectral Width 8012.8
1.00 2.00 1.00 1.00 1.00 2.00 5.00 2.00 2.00 4.00 1.00 1.00 6.00 3.00 3.00
Ts
N
O-O
H
N
Ts
N
Me
Me
C2
f1 (ppm)

	Parameter	Value
1	Title	LWF-4-172-C
2	Origin	Bruker BioSpin GmbH
3	Solvent	CDCl3
4	Temperature	300.0
5	Number of Scans	54
6	Acquisition Time	1.3631
7	Acquisition Date	2022-10-13T19:55:25
8	Spectrometer Frequency	100.61
9	Spectral Width	24038.5

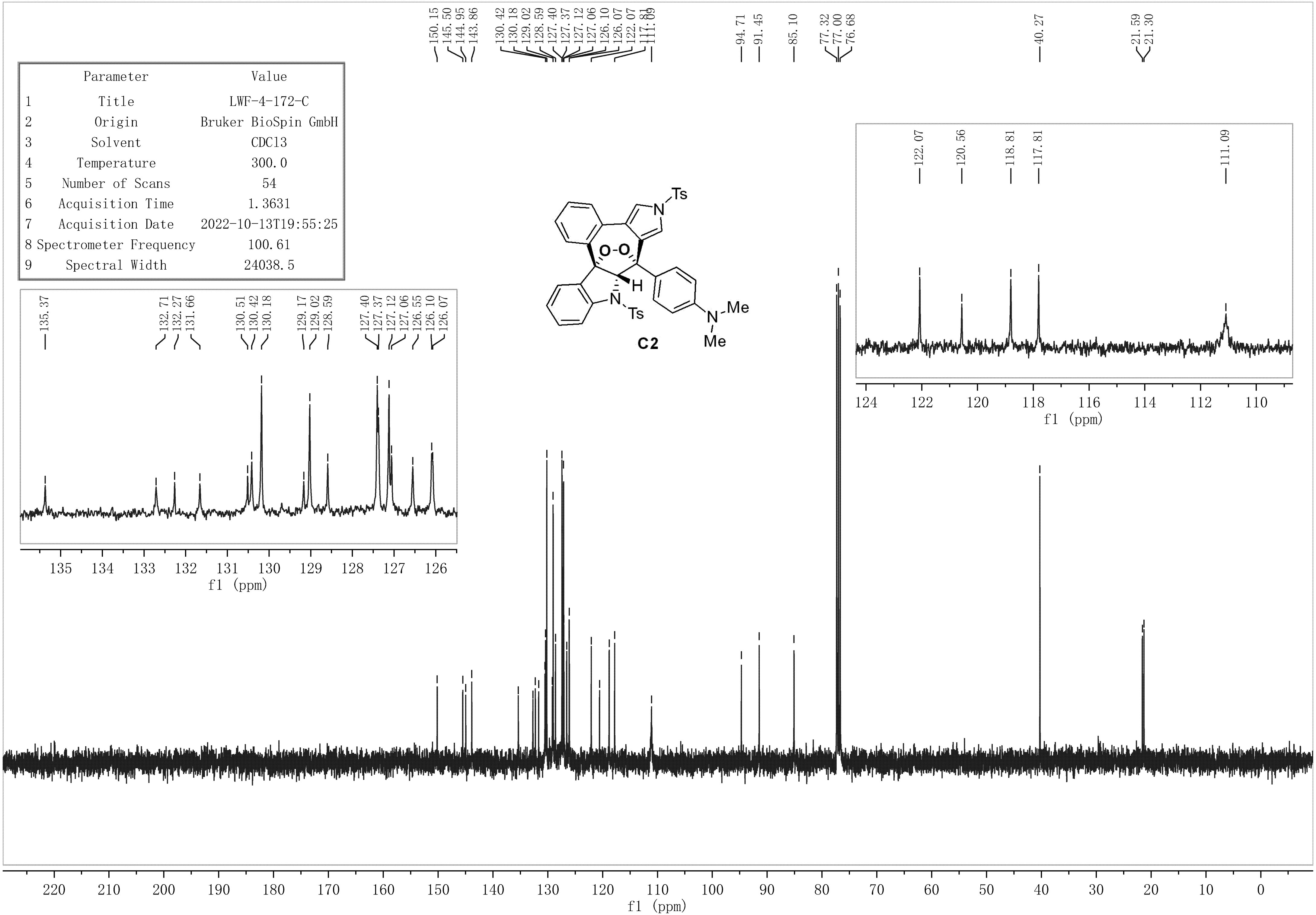

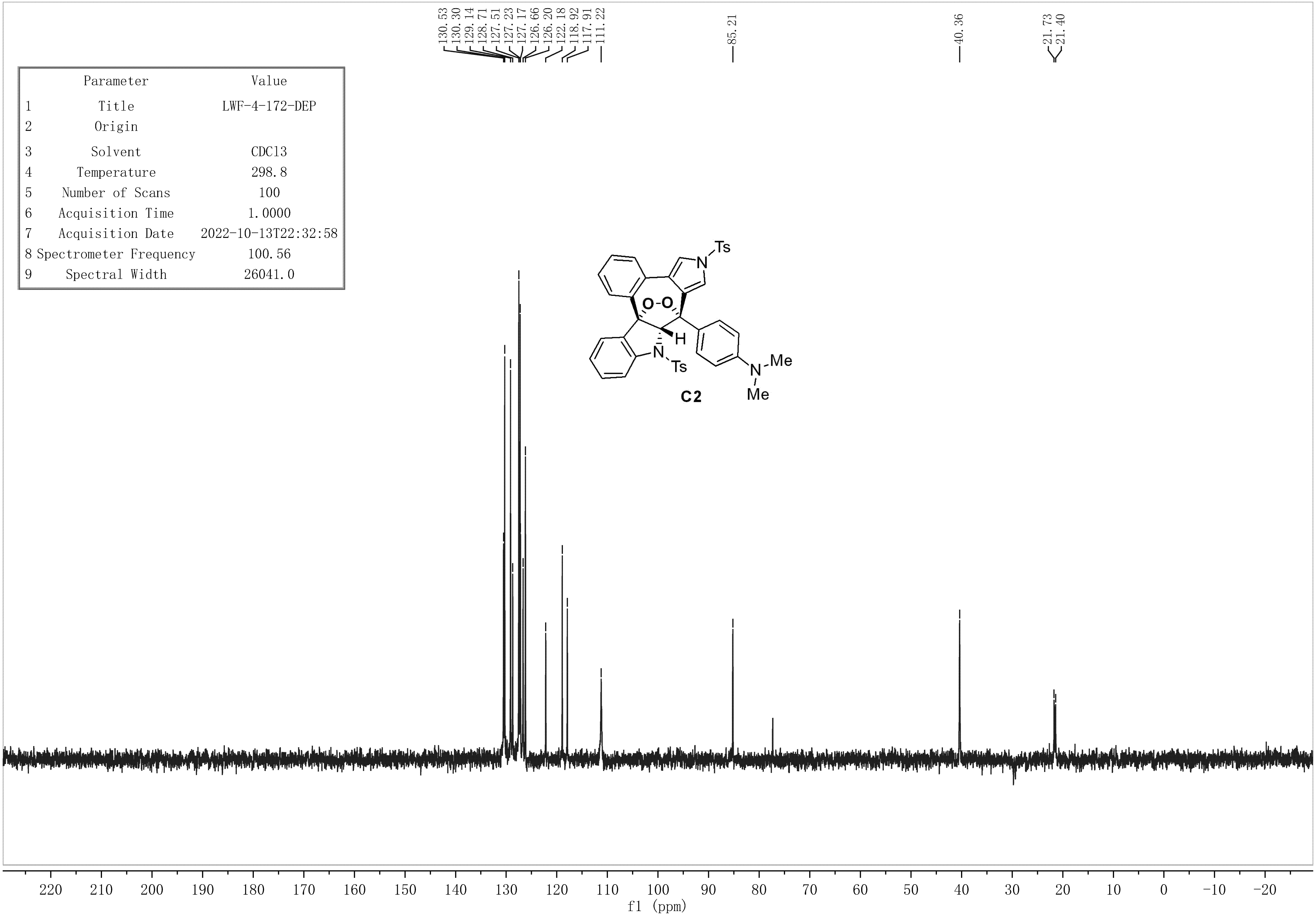

	Parameter	Value
1	Title	LWF-4-172-DEP
2	Origin	
3	Solvent	CDCl3
4	Temperature	298.8
5	Number of Scans	100
6	Acquisition Time	1.0000
7	Acquisition Date	2022-10-13T22:32:58
8	Spectrometer Frequency	100.56
9	Spectral Width	26041.0

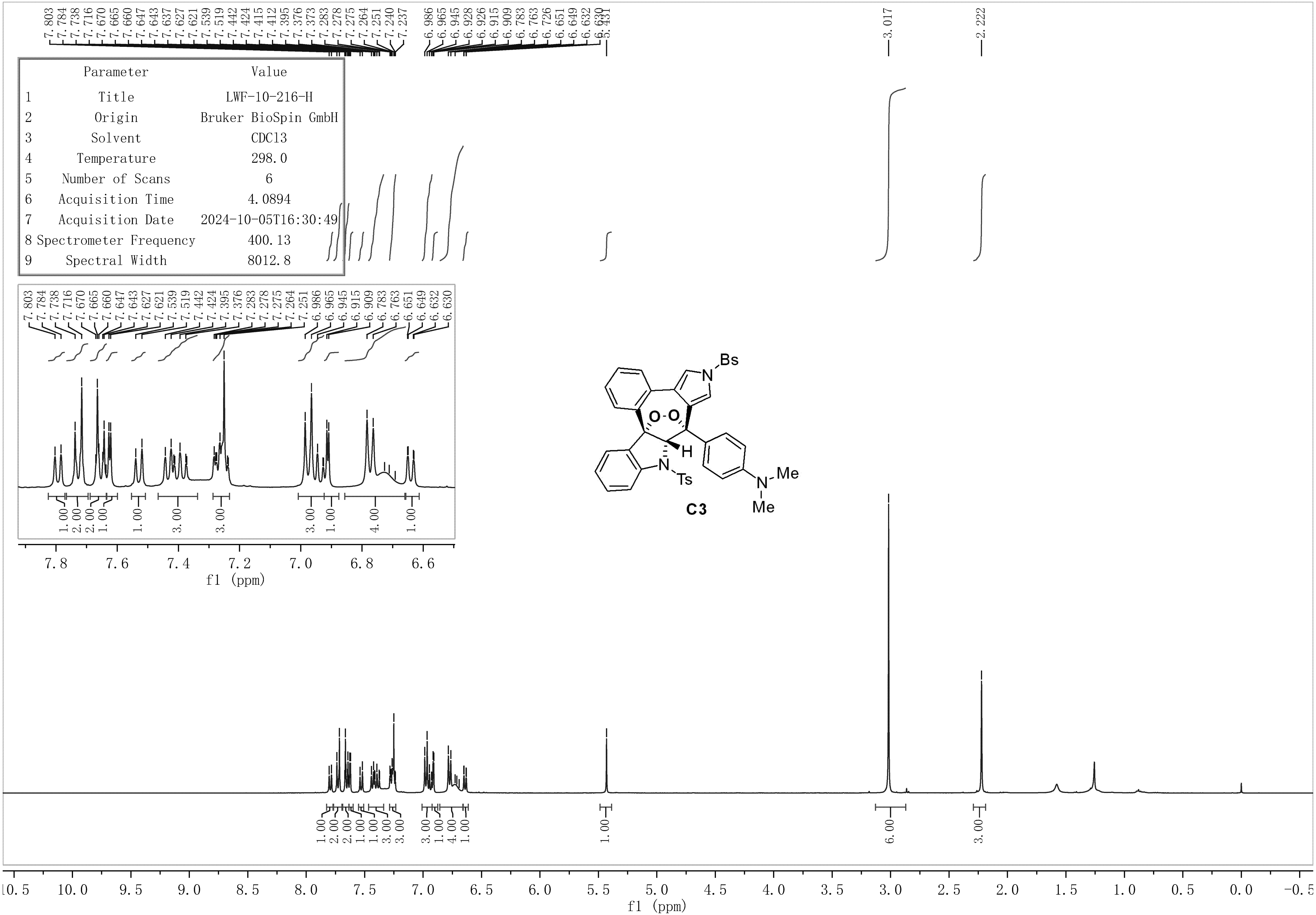
Parameter Value
1 Title LWF-10-216-H
2 Origin Bruker BioSpin GmbH
3 Solvent CDCl3
4 Temperature 298.0
5 Number of Scans 6
6 Acquisition Time 4.0894
7 Acquisition Date 2024-10-05T16:30:49
8 Spectrometer Frequency 400.13
9 Spectral Width 8012.8
7.803 7.784 7.738 7.716 7.670 7.665 7.660 7.647 7.643 7.637 7.627 7.621 7.539 7.519 7.442 7.424 7.415 7.412 7.395 7.376 7.373 7.283 7.278 7.275 7.264 7.251 7.240 7.237
6.986 6.965 6.945 6.928 6.926 6.915 6.909 6.783 6.763 6.726 6.651 6.649 6.632 6.630 5.431
3.017
2.222
Bs
N
O-O
H
N
Ts
N
Me
Me
C3
1.00 2.00 2.00 1.00 1.00 3.00 3.00 3.00 1.00 4.00 1.00 1.00 6.00 3.00
f1 (ppm)

	Parameter	Value
1	Title	LWF-10-216-C
2	Origin	Bruker BioSpin GmbH
3	Solvent	CDCl3
4	Temperature	300.0
5	Number of Scans	106
6	Acquisition Time	1.3631
7	Acquisition Date	2024-10-05T16:32:07
8	Spectrometer Frequency	100.61
9	Spectral Width	24038.5

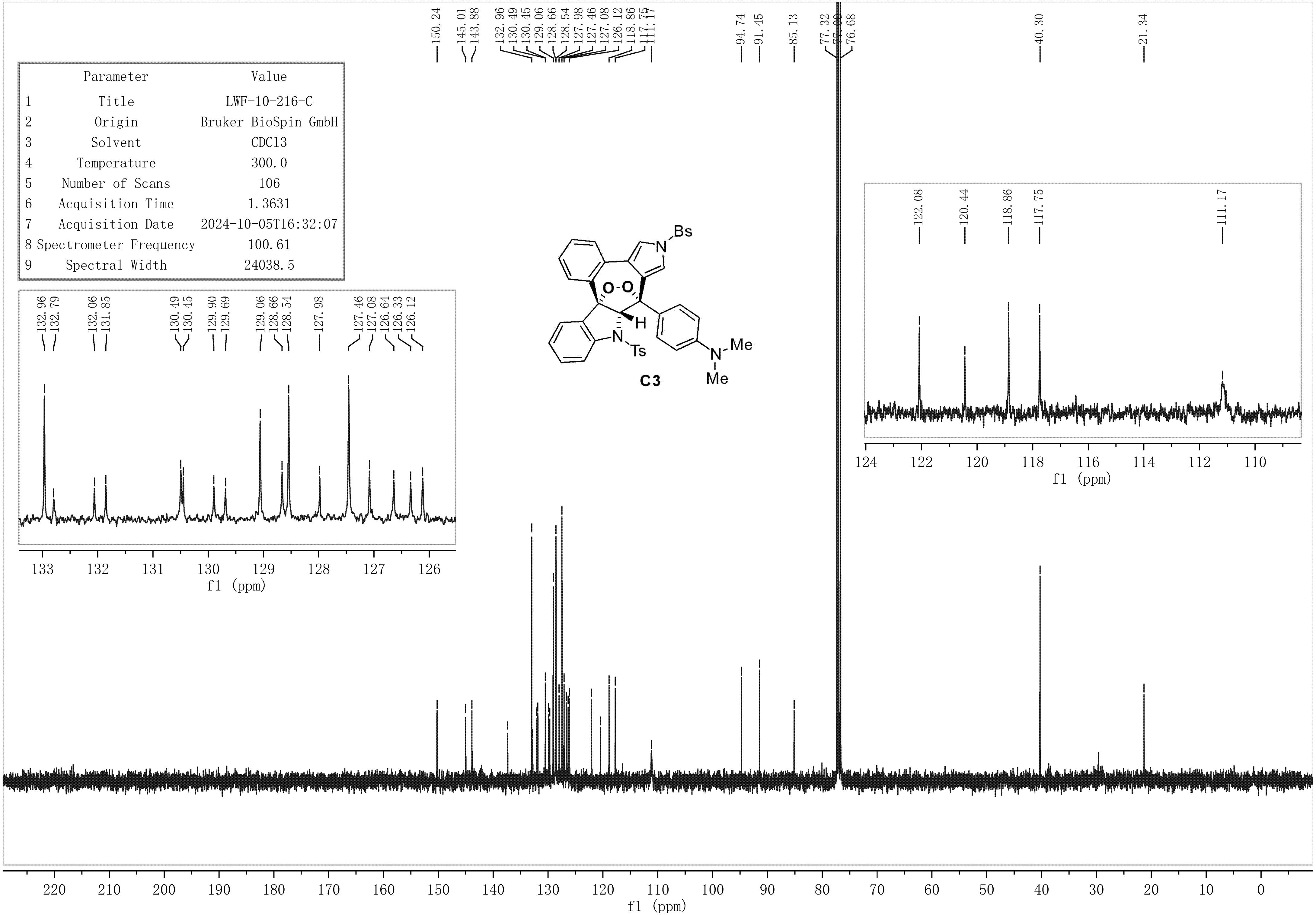

	Parameter	Value
1	Title	LWF-4-180-H
2	Origin	Bruker BioSpin GmbH
3	Solvent	CDCl3
4	Temperature	298.0
5	Number of Scans	9
6	Acquisition Time	4.0894
7	Acquisition Date	2022-10-09T16:58:40
8	Spectrometer Frequency	400.13
9	Spectral Width	8012.8

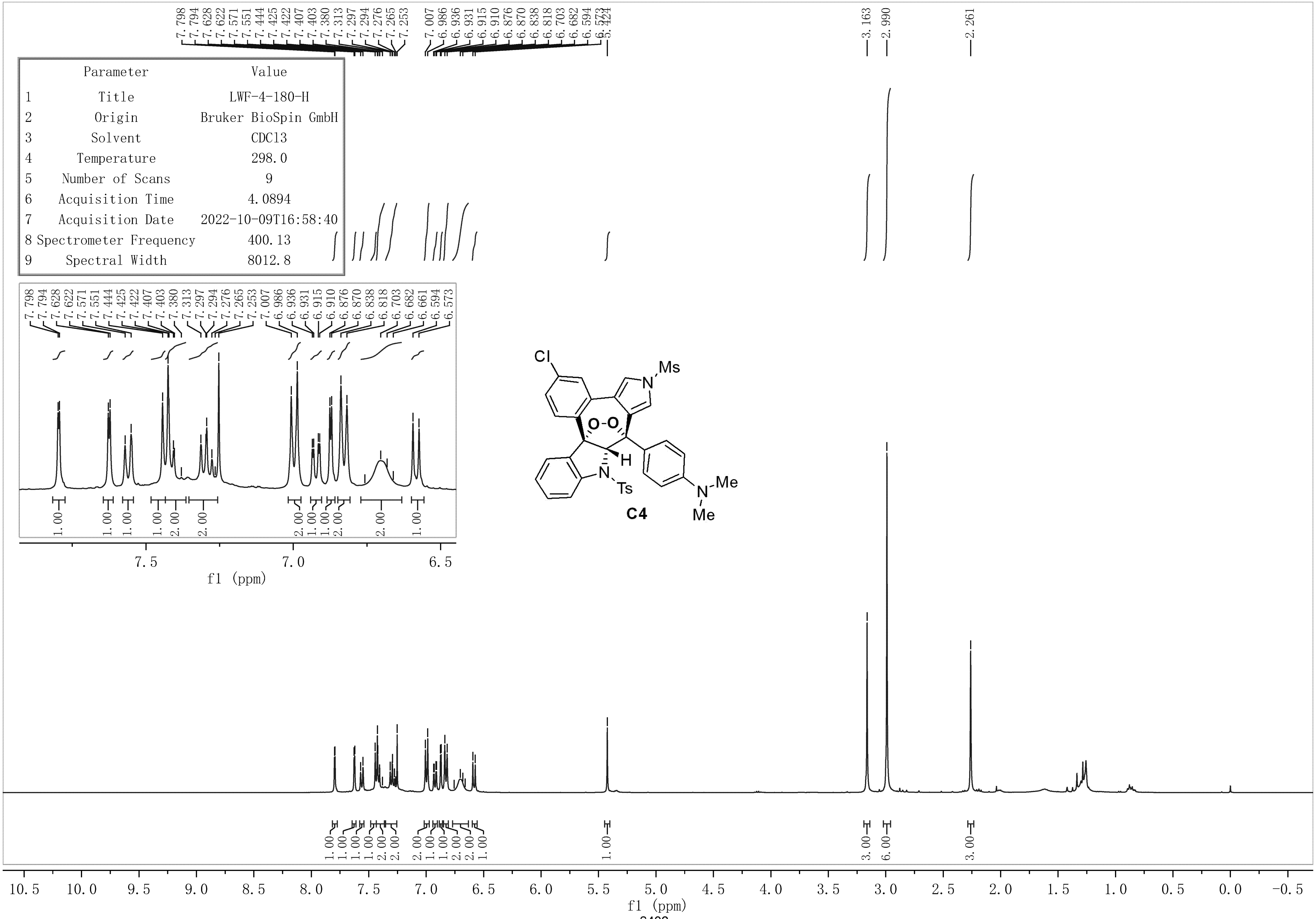

C4

Parameter	Value
1 Title	LWF-4-180-C
2 Origin	Bruker BioSpin GmbH
3 Solvent	CDCl3
4 Temperature	300.0
5 Number of Scans	63
6 Acquisition Time	1.3631
7 Acquisition Date	2022-10-09T17:00:26
8 Spectrometer Frequency	100.61
9 Spectral Width	24038.5

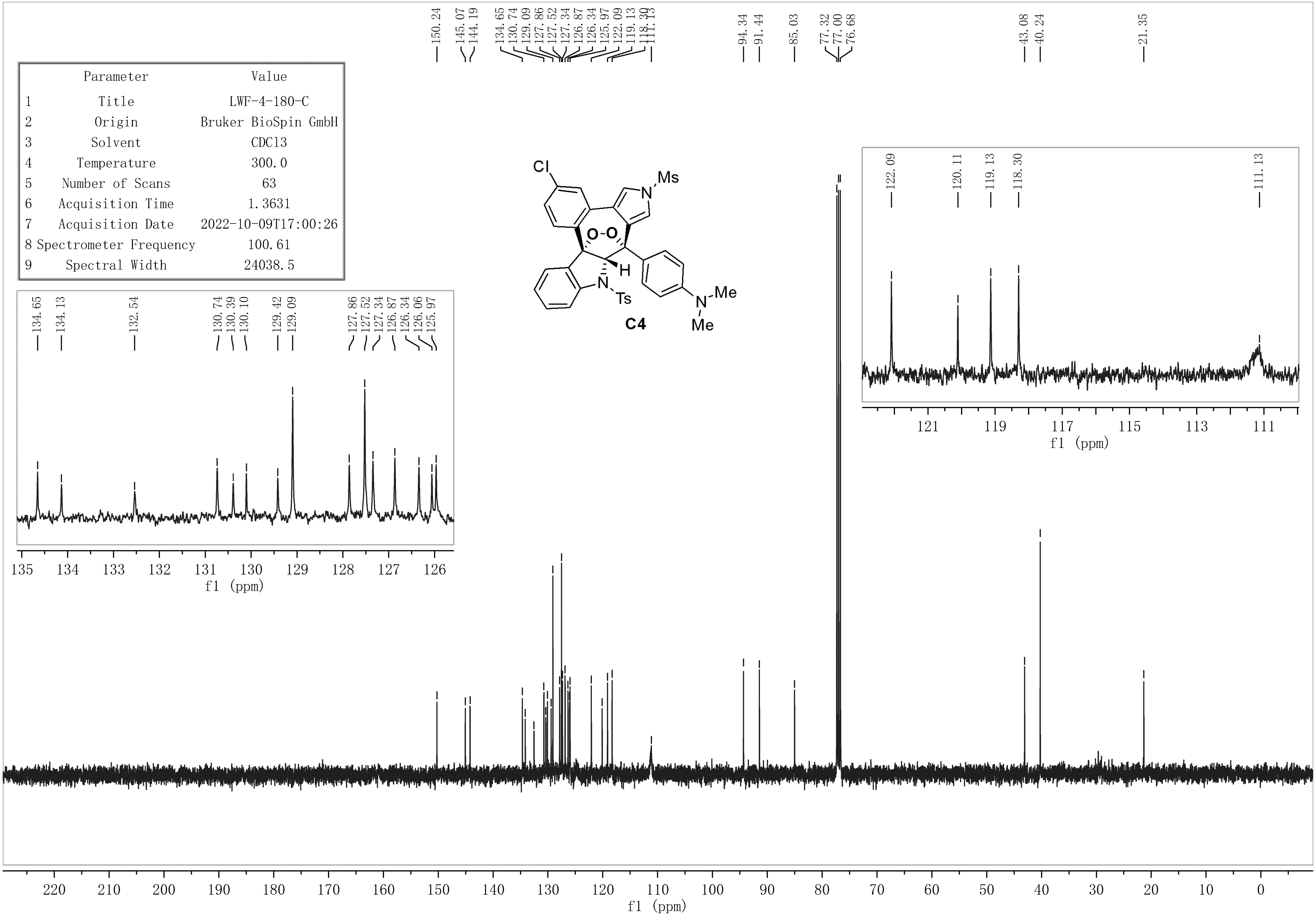

	Parameter	Value
1	Title	LWF-4-194-H
2	Origin	Bruker BioSpin GmbH
3	Solvent	CDCl3
4	Temperature	298.0
5	Number of Scans	10
6	Acquisition Time	4.0894
7	Acquisition Date	2022-10-17T21:43:18
8	Spectrometer Frequency	400.13
9	Spectral Width	8012.8

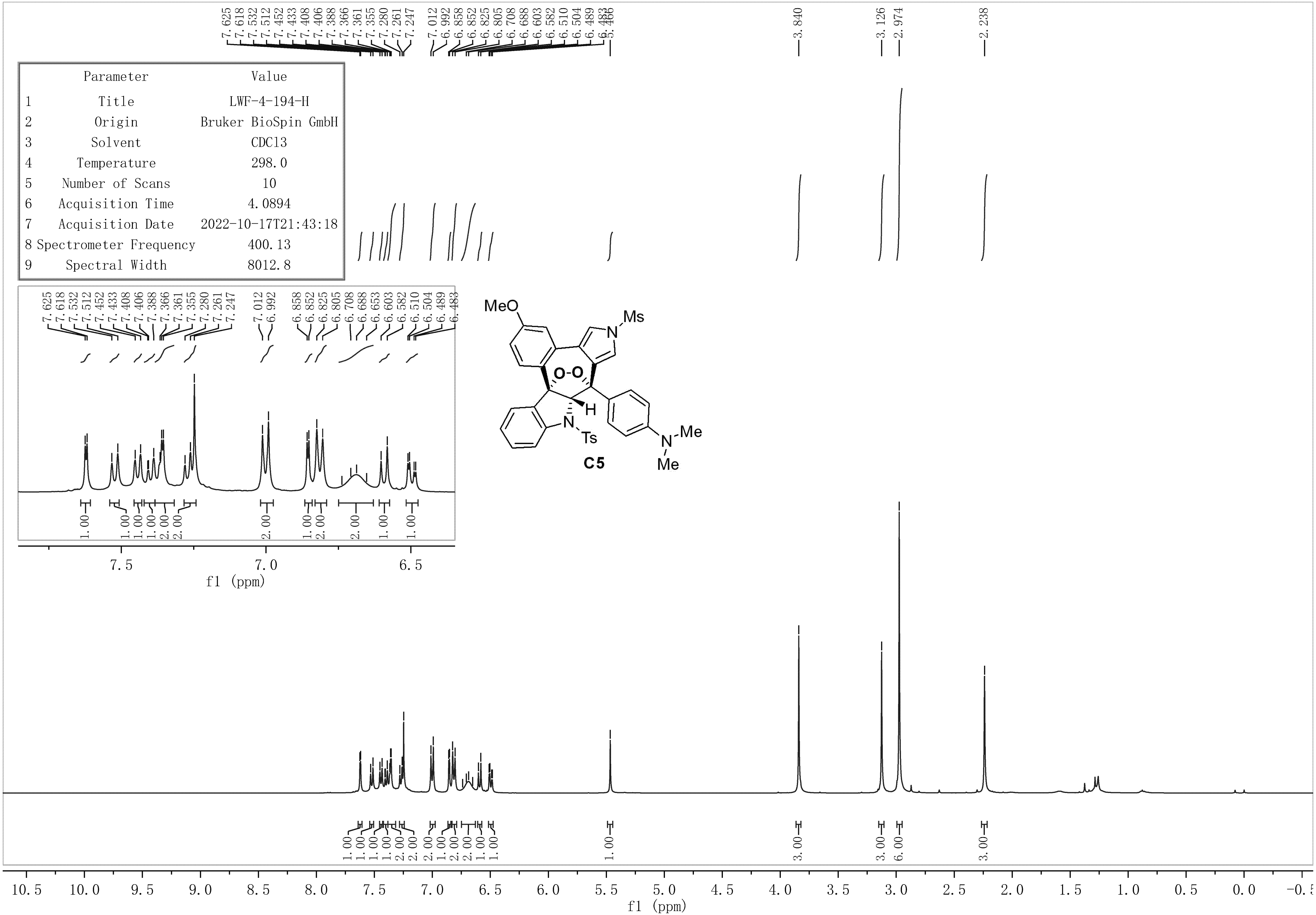

C5

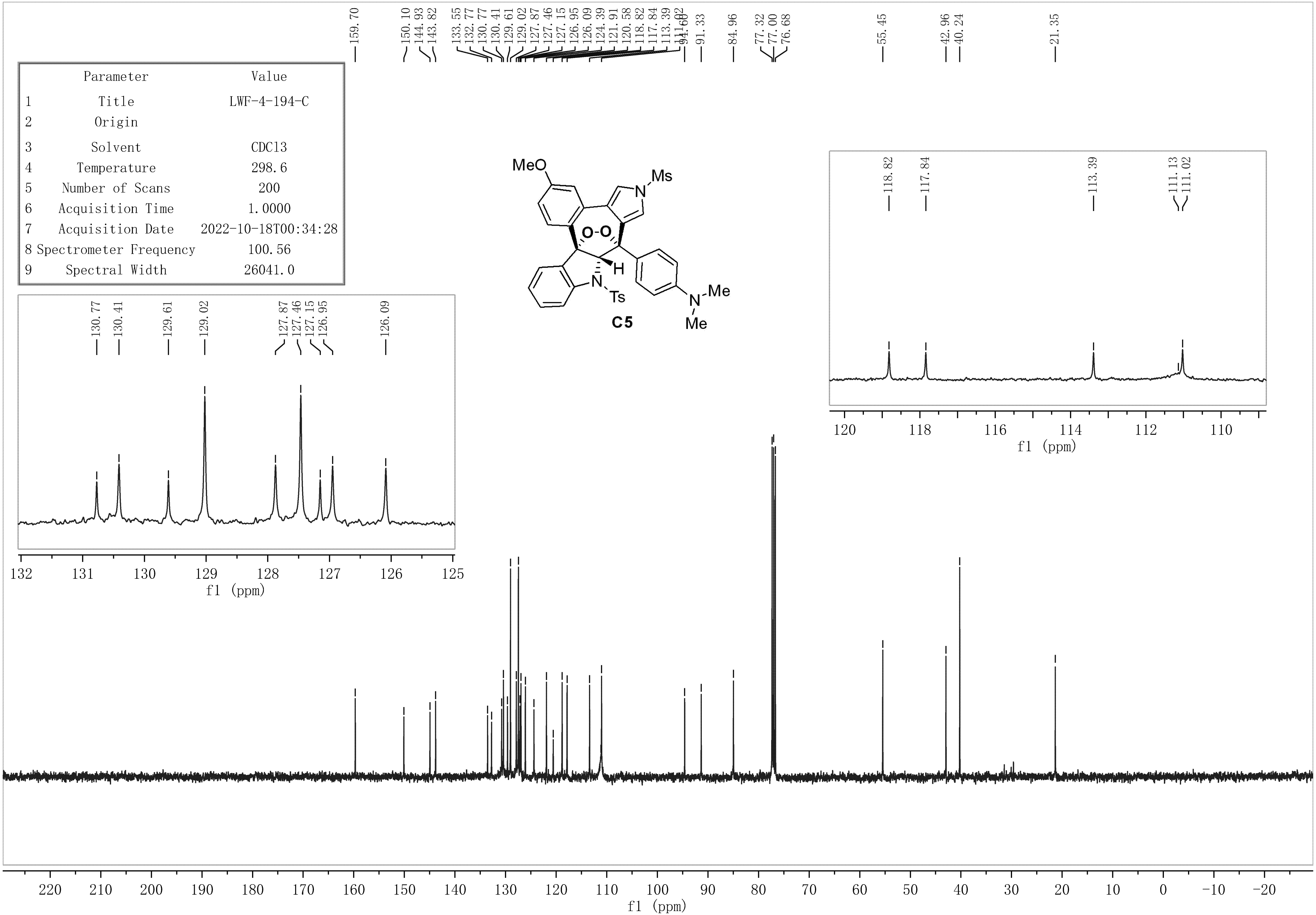

	Parameter	Value
1	Title	LWF-4-194-C
2	Origin	
3	Solvent	CDCl3
4	Temperature	298.6
5	Number of Scans	200
6	Acquisition Time	1.0000
7	Acquisition Date	2022-10-18T00:34:28
8	Spectrometer Frequency	100.56
9	Spectral Width	26041.0

C5

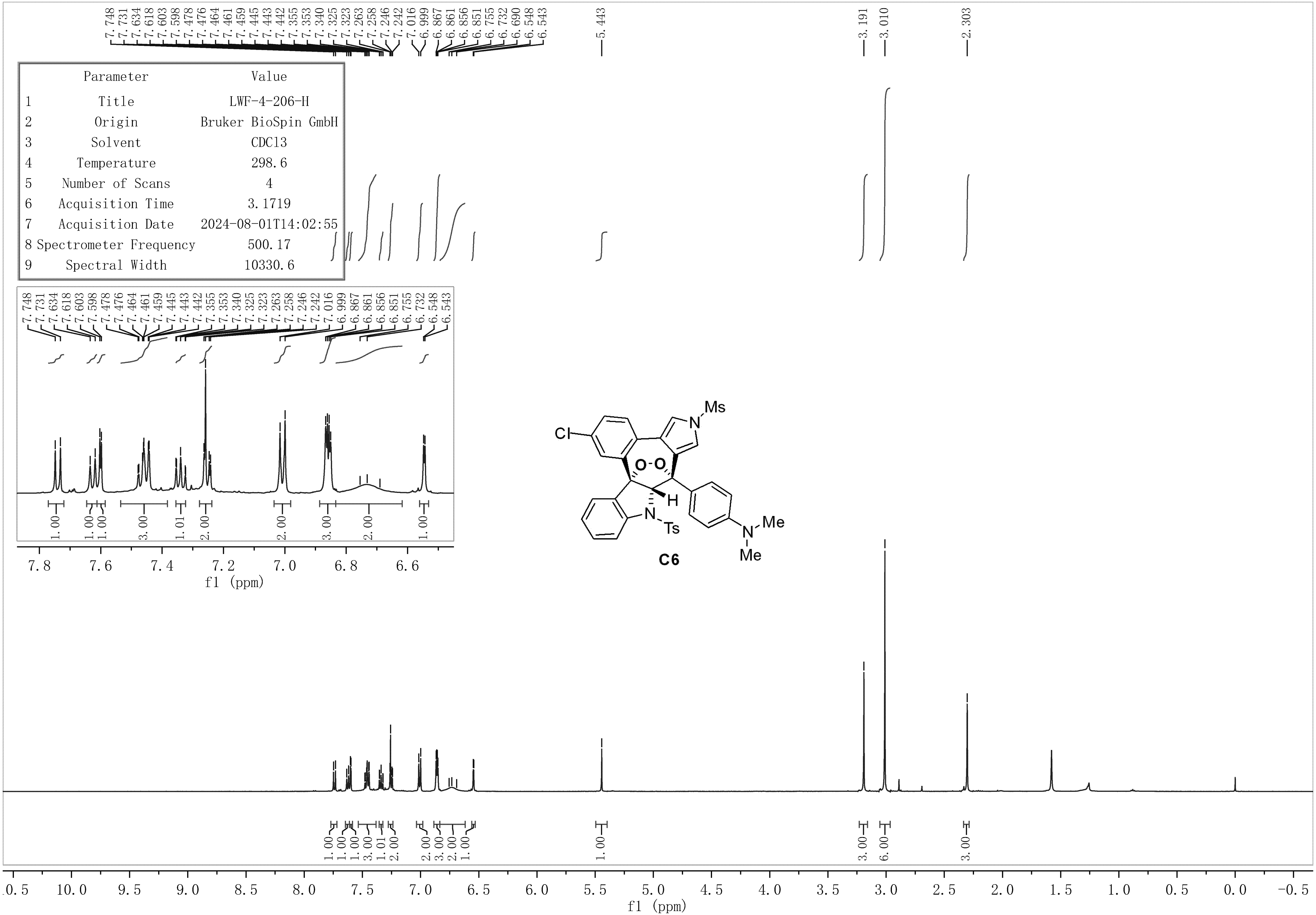
7.748 7.731 7.634 7.618 7.603 7.598 7.478 7.476 7.464 7.461 7.459 7.445 7.443 7.442 7.355 7.353 7.340 7.325 7.323 7.263 7.258 7.246 7.242 7.016 6.999 6.867 6.861 6.856 6.851 6.755 6.732 6.690 6.548 6.543
5.443
3.191
3.010
2.303
Parameter Value
1 Title LWF-4-206-H
2 Origin Bruker BioSpin GmbH
3 Solvent CDCl3
4 Temperature 298.6
5 Number of Scans 4
6 Acquisition Time 3.1719
7 Acquisition Date 2024-08-01T14:02:55
8 Spectrometer Frequency 500.17
9 Spectral Width 10330.6
1.00 1.00 1.00 3.00 1.01 2.00 2.00 3.00 2.00 1.00
1.00
3.00 6.00
3.00
f1 (ppm)
Cl
Ms
N
O-O
H
N
Ts
N
Me
Me
C6

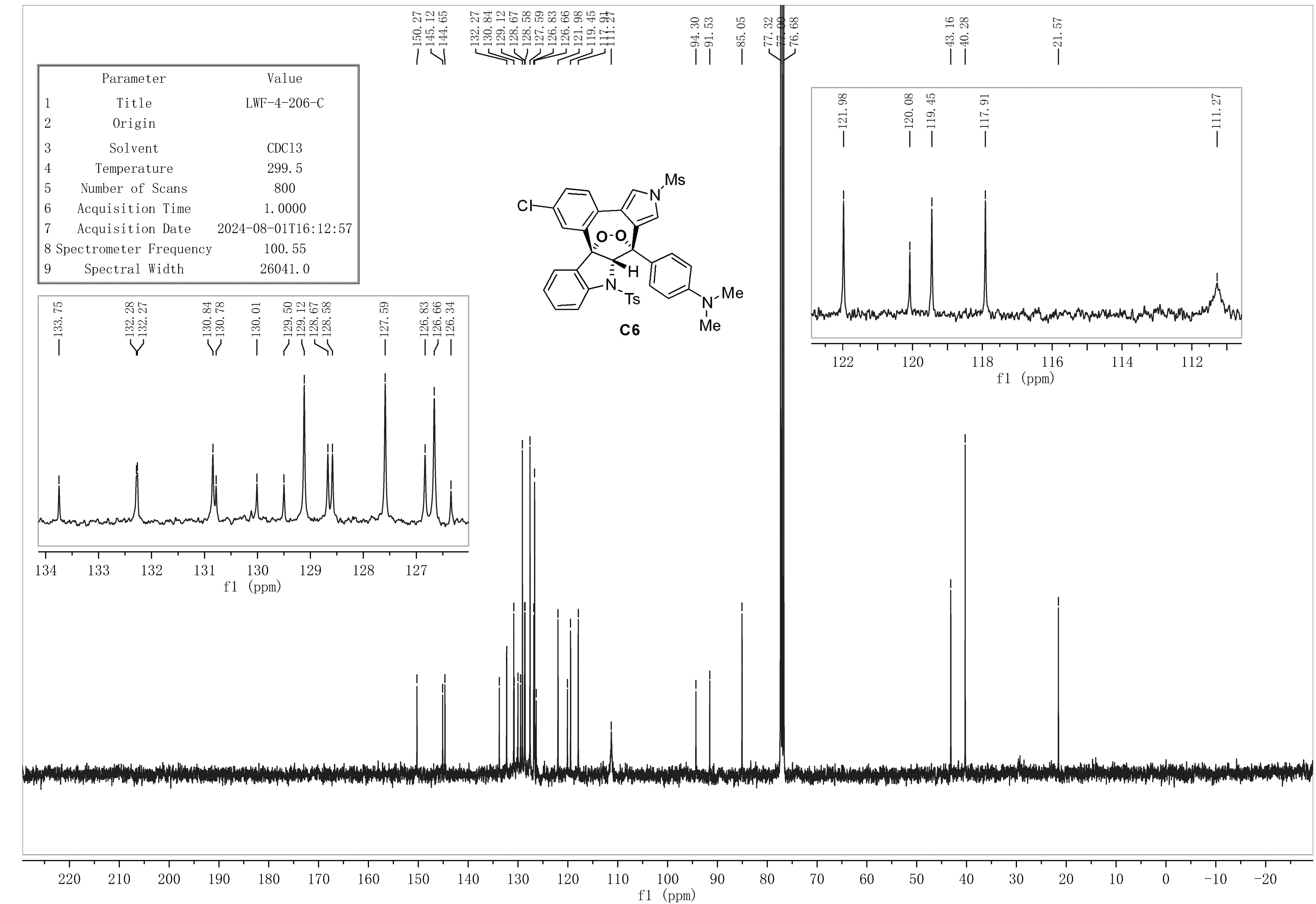
150.27
145.12
144.65
132.27
130.84
129.12
128.67
128.58
127.59
126.83
126.66
121.98
119.45
117.91
111.27
94.30
91.53
85.05
77.32
77.00
76.68
43.16
40.28
21.57
Parameter Value
1 Title LWF-4-206-C
2 Origin
3 Solvent CDCl3
4 Temperature 299.5
5 Number of Scans 800
6 Acquisition Time 1.0000
7 Acquisition Date 2024-08-01T16:12:57
8 Spectrometer Frequency 100.55
9 Spectral Width 26041.0
133.75
132.28
132.27
130.84
130.78
130.01
129.50
129.12
128.67
128.58
127.59
126.83
126.66
126.34
121.98
120.08
119.45
117.91
111.27
Cl
Ms
N
O-O
H
N
Ts
N
Me
Me
C6
f1 (ppm)
f1 (ppm)
f1 (ppm)

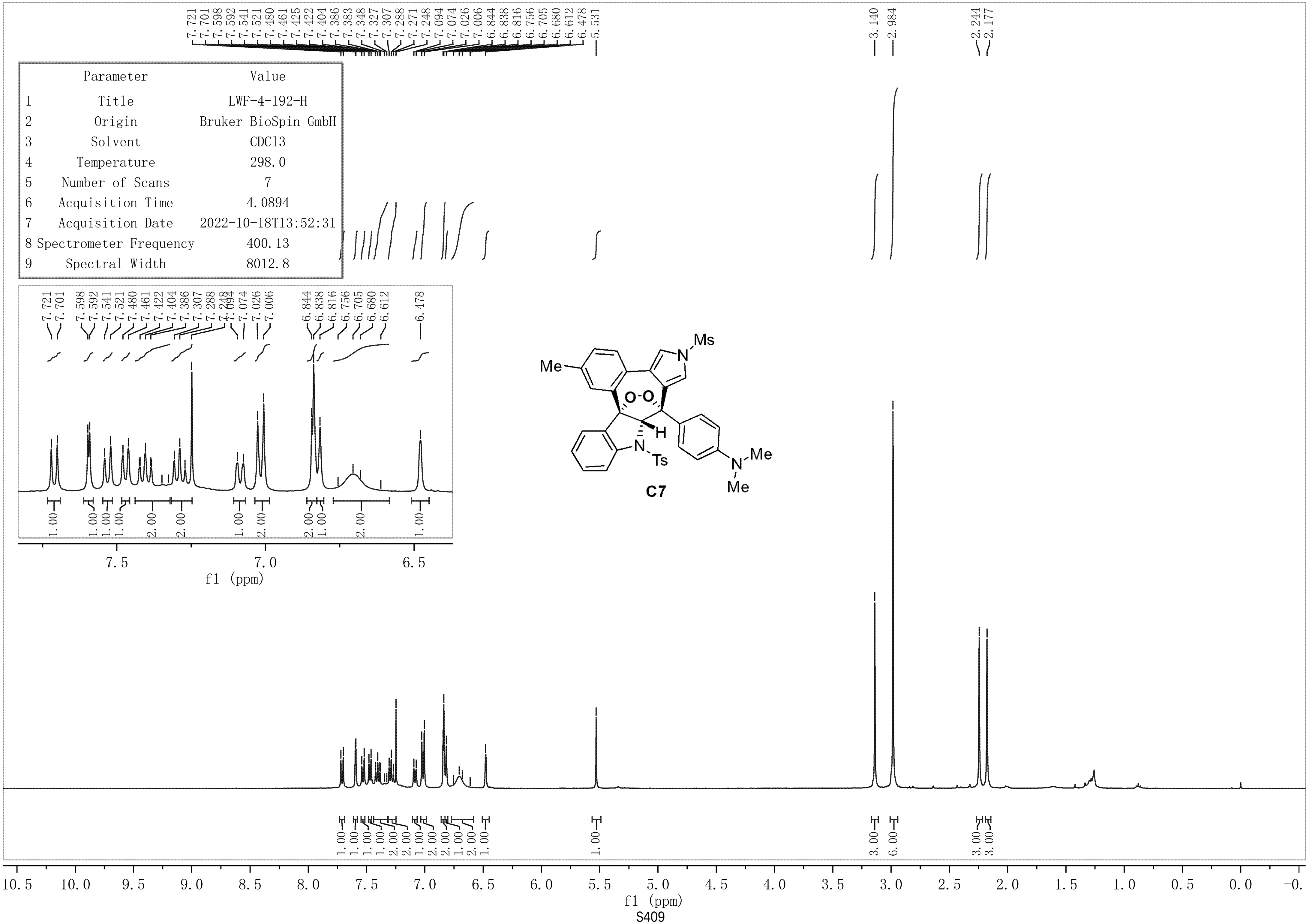

	Parameter	Value
1	Title	LWF-4-192-H
2	Origin	Bruker BioSpin GmbH
3	Solvent	CDCl3
4	Temperature	298.0
5	Number of Scans	7
6	Acquisition Time	4.0894
7	Acquisition Date	2022-10-18T13:52:31
8	Spectrometer Frequency	400.13
9	Spectral Width	8012.8

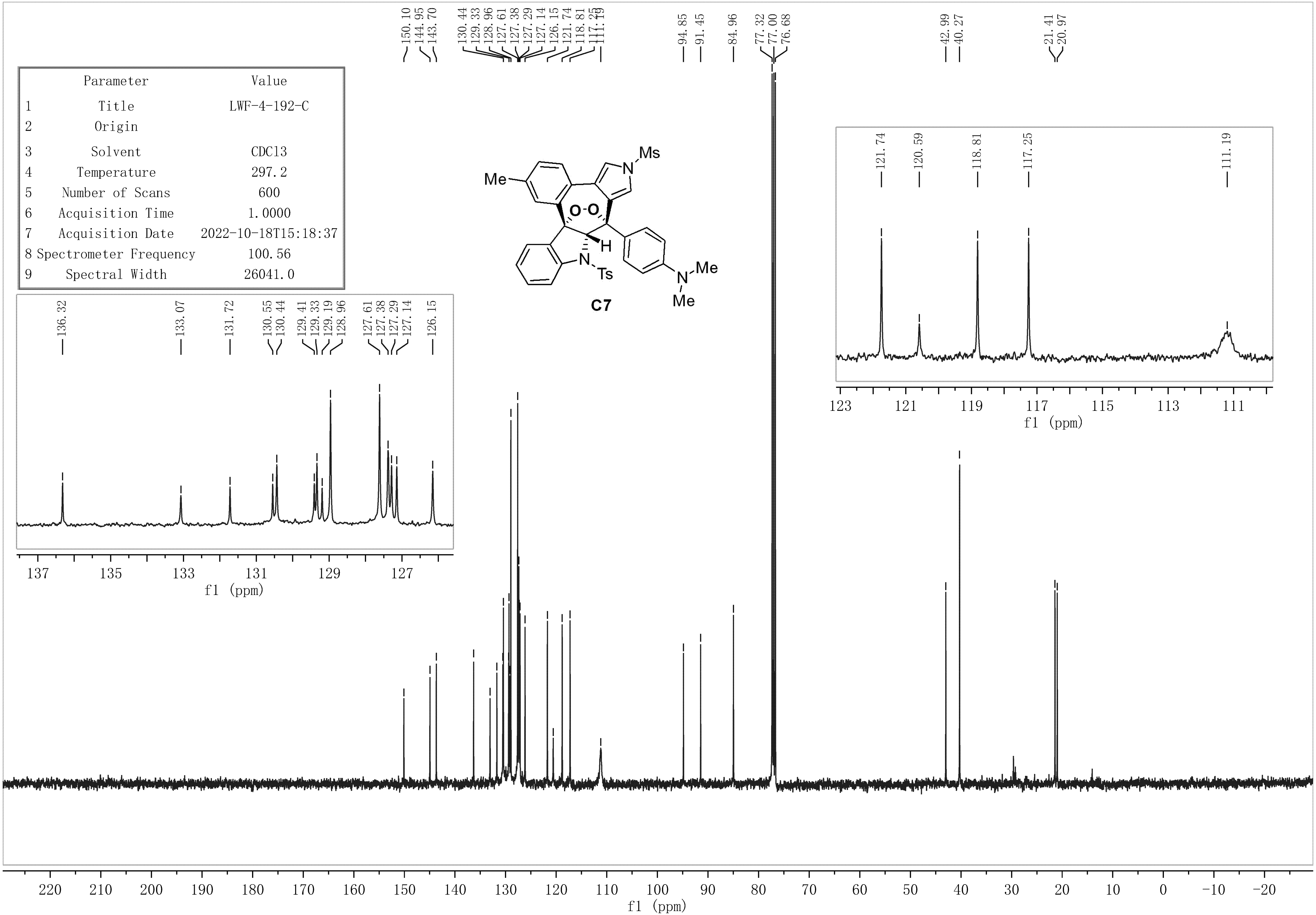

150.10
144.95
143.70
130.44
129.33
128.96
127.61
127.38
127.29
127.14
126.15
121.74
118.81
117.25
111.19
94.85
91.45
84.96
77.32
77.00
76.68
42.99
40.27
21.41
20.97
Parameter Value
1 Title LWF-4-192-C
2 Origin
3 Solvent CDCl3
4 Temperature 297.2
5 Number of Scans 600
6 Acquisition Time 1.0000
7 Acquisition Date 2022-10-18T15:18:37
8 Spectrometer Frequency 100.56
9 Spectral Width 26041.0
Me
Ms
N
O-O
H
N
Ts
N
Me
Me
C7
136.32
133.07
131.72
130.55
130.44
129.41
129.33
129.19
128.96
127.61
127.38
127.29
127.14
126.15
137 135 133 131 129 127
f1 (ppm)
121.74
120.59
118.81
117.25
111.19
123 121 119 117 115 113 111
f1 (ppm)
220 210 200 190 180 170 160 150 140 130 120 110 100 90 80 70 60 50 40 30 20 10 0 -10 -20
f1 (ppm)

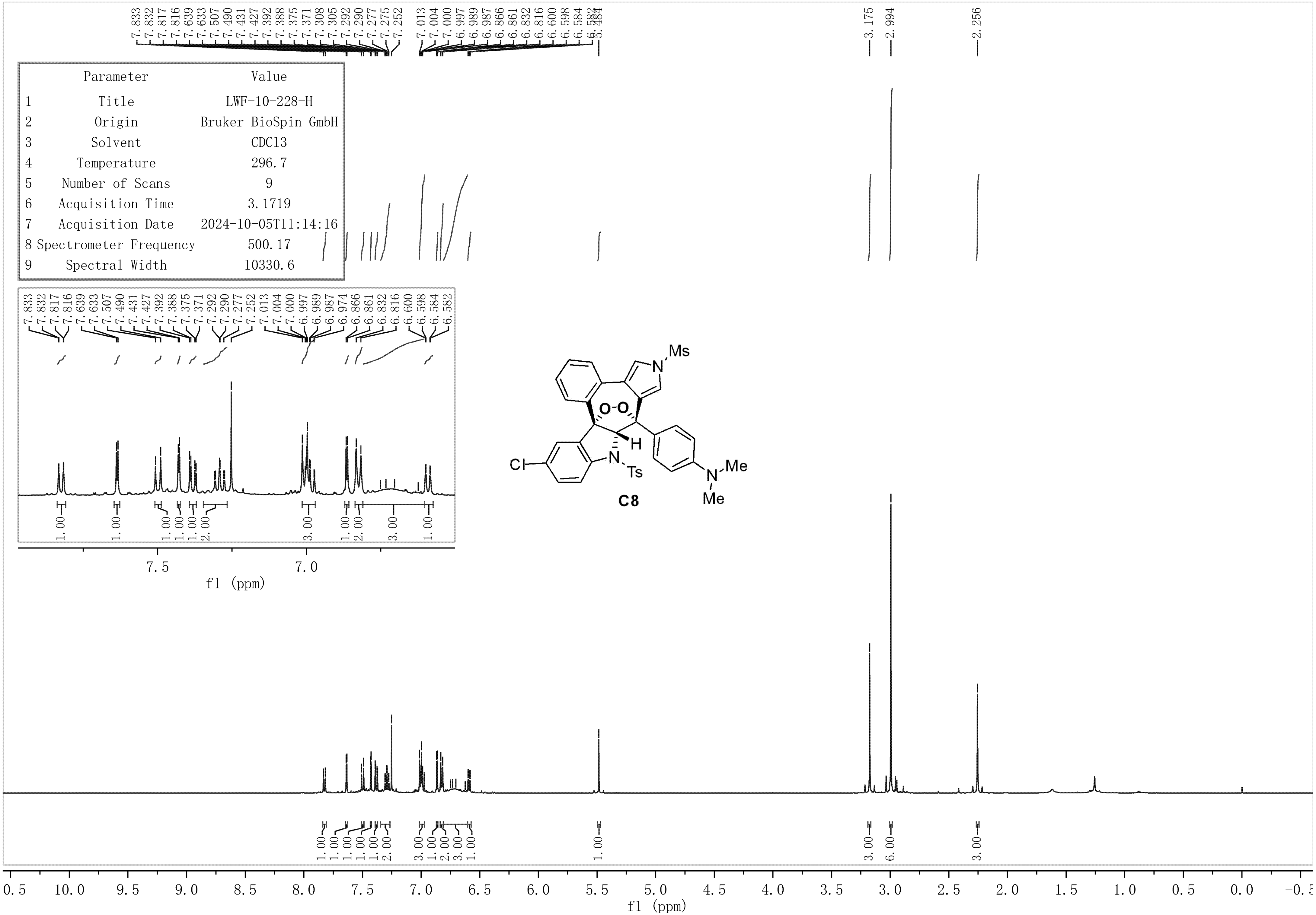
7.833 7.832 7.817 7.816 7.639 7.633 7.507 7.490 7.431 7.427 7.392 7.388 7.375 7.371 7.308 7.305 7.292 7.290 7.277 7.275 7.252 7.013 7.004 7.000 6.997 6.989 6.987 6.866 6.861 6.832 6.816 6.600 6.598 6.584 6.582 5.484
3.175 2.994 2.256
Parameter Value
1 Title LWF-10-228-H
2 Origin Bruker BioSpin GmbH
3 Solvent CDCl3
4 Temperature 296.7
5 Number of Scans 9
6 Acquisition Time 3.1719
7 Acquisition Date 2024-10-05T11:14:16
8 Spectrometer Frequency 500.17
9 Spectral Width 10330.6
Ms
N
O-O
H
Cl
N
Ts
N
Me
Me
C8
f1 (ppm)

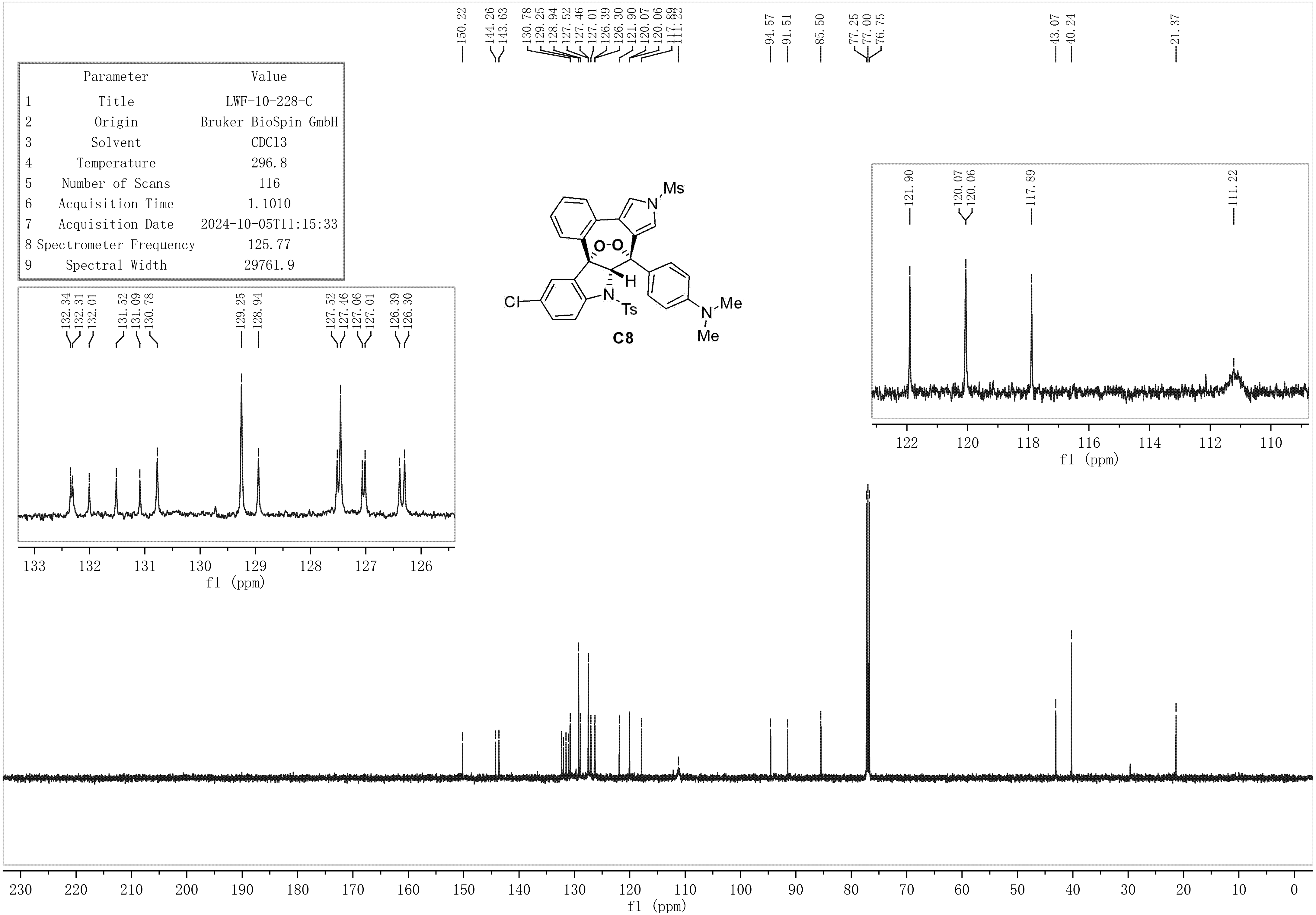

	Parameter	Value
1	Title	LWF-10-228-C
2	Origin	Bruker BioSpin GmbH
3	Solvent	CDCl3
4	Temperature	296.8
5	Number of Scans	116
6	Acquisition Time	1.1010
7	Acquisition Date	2024-10-05T11:15:33
8	Spectrometer Frequency	125.77
9	Spectral Width	29761.9

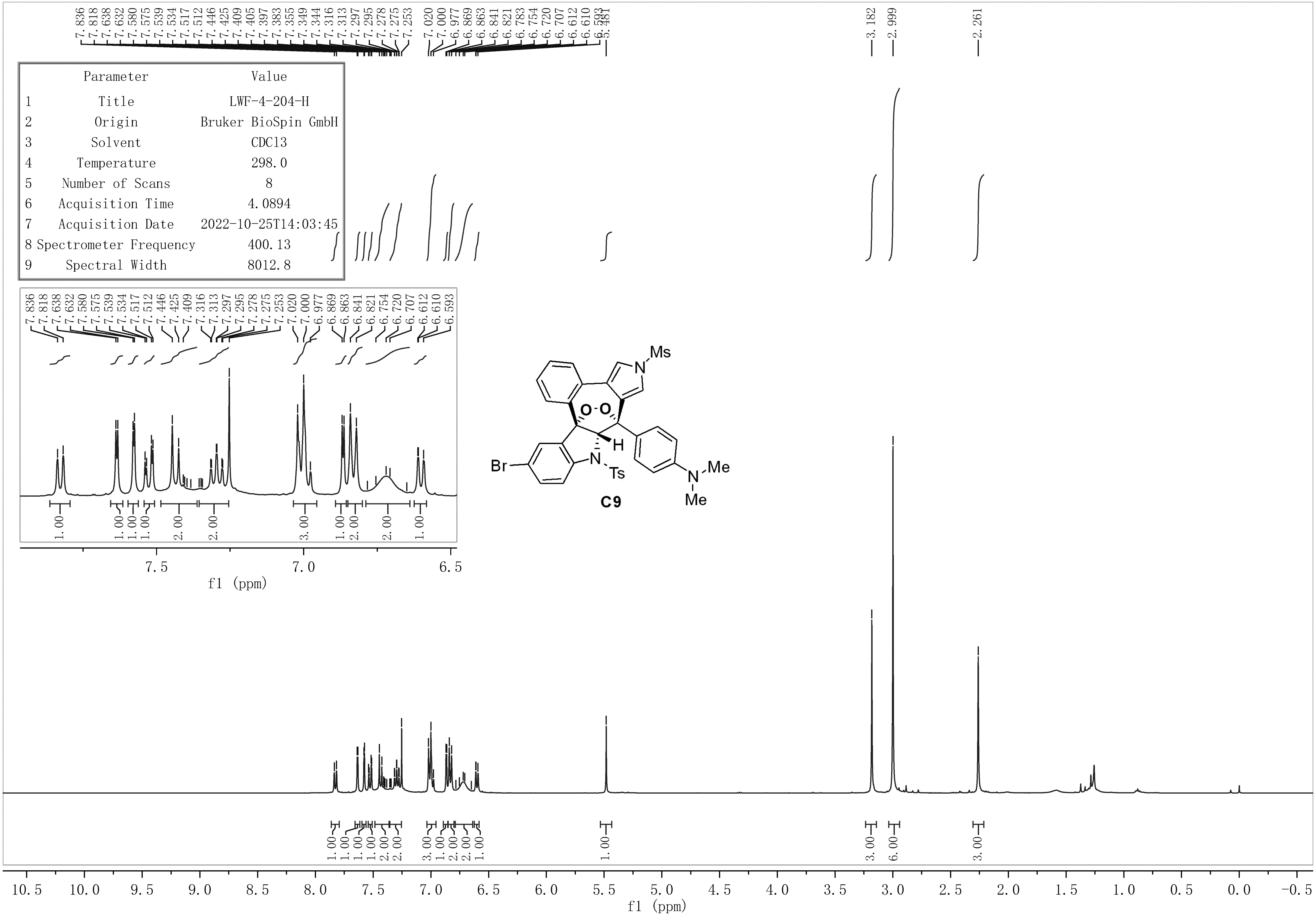

	Parameter	Value
1	Title	LWF-4-204-H
2	Origin	Bruker BioSpin GmbH
3	Solvent	CDCl3
4	Temperature	298.0
5	Number of Scans	8
6	Acquisition Time	4.0894
7	Acquisition Date	2022-10-25T14:03:45
8	Spectrometer Frequency	400.13
9	Spectral Width	8012.8

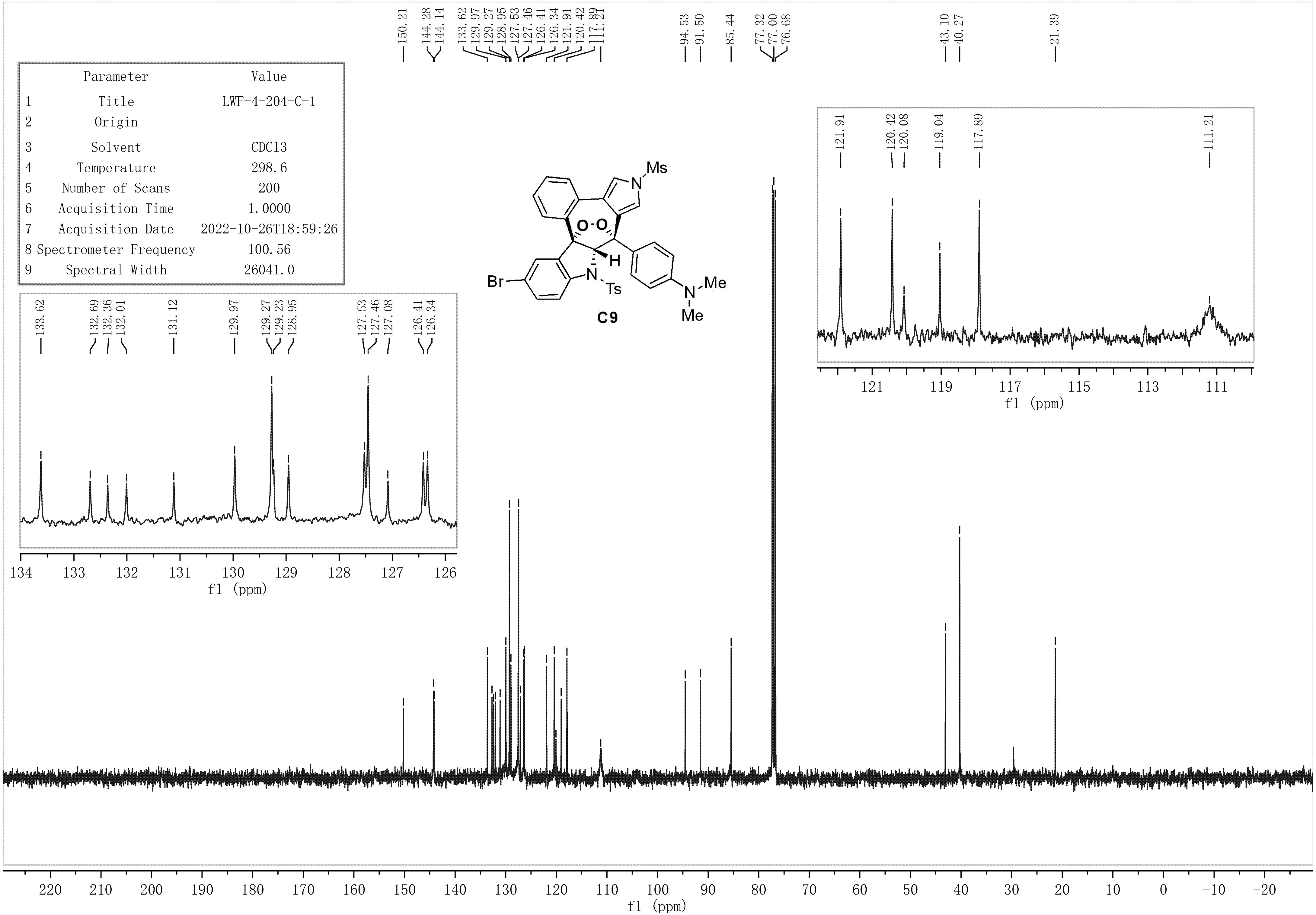
150.21
144.28
144.14
133.62
129.97
129.27
128.95
127.53
127.46
126.41
126.34
121.91
120.42
117.89
111.21
94.53
91.50
85.44
77.32
77.00
76.68
43.10
40.27
21.39
Parameter Value
1 Title LWF-4-204-C-1
2 Origin
3 Solvent CDCl3
4 Temperature 298.6
5 Number of Scans 200
6 Acquisition Time 1.0000
7 Acquisition Date 2022-10-26T18:59:26
8 Spectrometer Frequency 100.56
9 Spectral Width 26041.0
133.62
132.69
132.36
132.01
131.12
129.97
129.27
129.23
128.95
127.53
127.46
127.08
126.41
126.34
134 133 132 131 130 129 128 127 126
f1 (ppm)
Ms
N
O-O
H
Br
N
Ts
N
Me
Me
C9
121.91
120.42
120.08
119.04
117.89
111.21
121 119 117 115 113 111
f1 (ppm)
220 210 200 190 180 170 160 150 140 130 120 110 100 90 80 70 60 50 40 30 20 10 0 -10 -20
f1 (ppm)

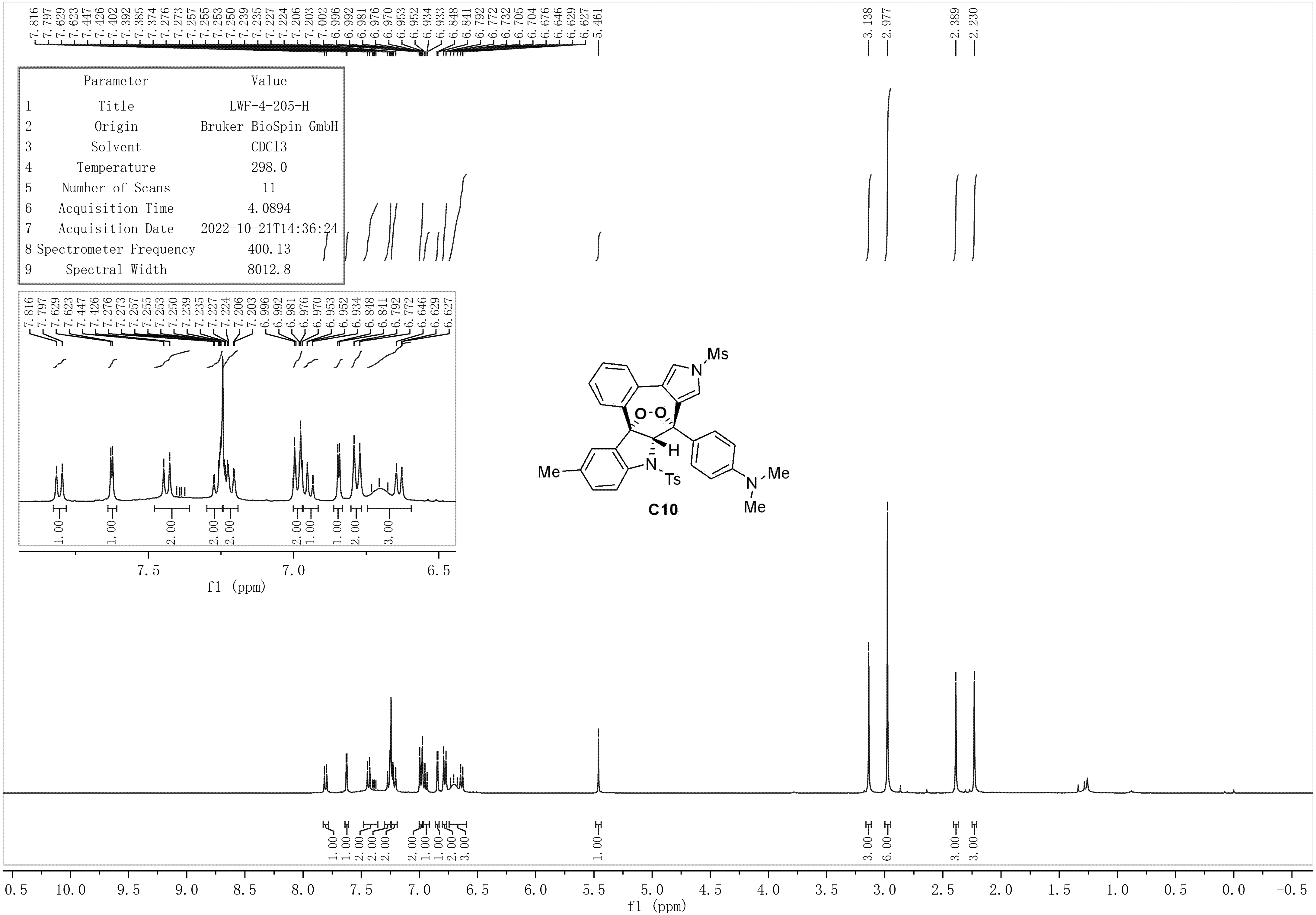
7.816 7.797 7.629 7.623 7.447 7.426 7.402 7.392 7.385 7.374 7.276 7.273 7.257 7.255 7.253 7.250 7.239 7.235 7.227 7.224 7.206 7.203 7.002 6.996 6.992 6.981 6.976 6.970 6.953 6.952 6.934 6.933 6.848 6.841 6.792 6.772 6.732 6.705 6.704 6.676 6.646 6.629 6.627 5.461
3.138 2.977
2.389 2.230
Parameter Value
1 Title LWF-4-205-H
2 Origin Bruker BioSpin GmbH
3 Solvent CDCl3
4 Temperature 298.0
5 Number of Scans 11
6 Acquisition Time 4.0894
7 Acquisition Date 2022-10-21T14:36:24
8 Spectrometer Frequency 400.13
9 Spectral Width 8012.8
Ms
N
O-O
H
Me
N
Ts
N
Me
Me
C10
f1 (ppm)

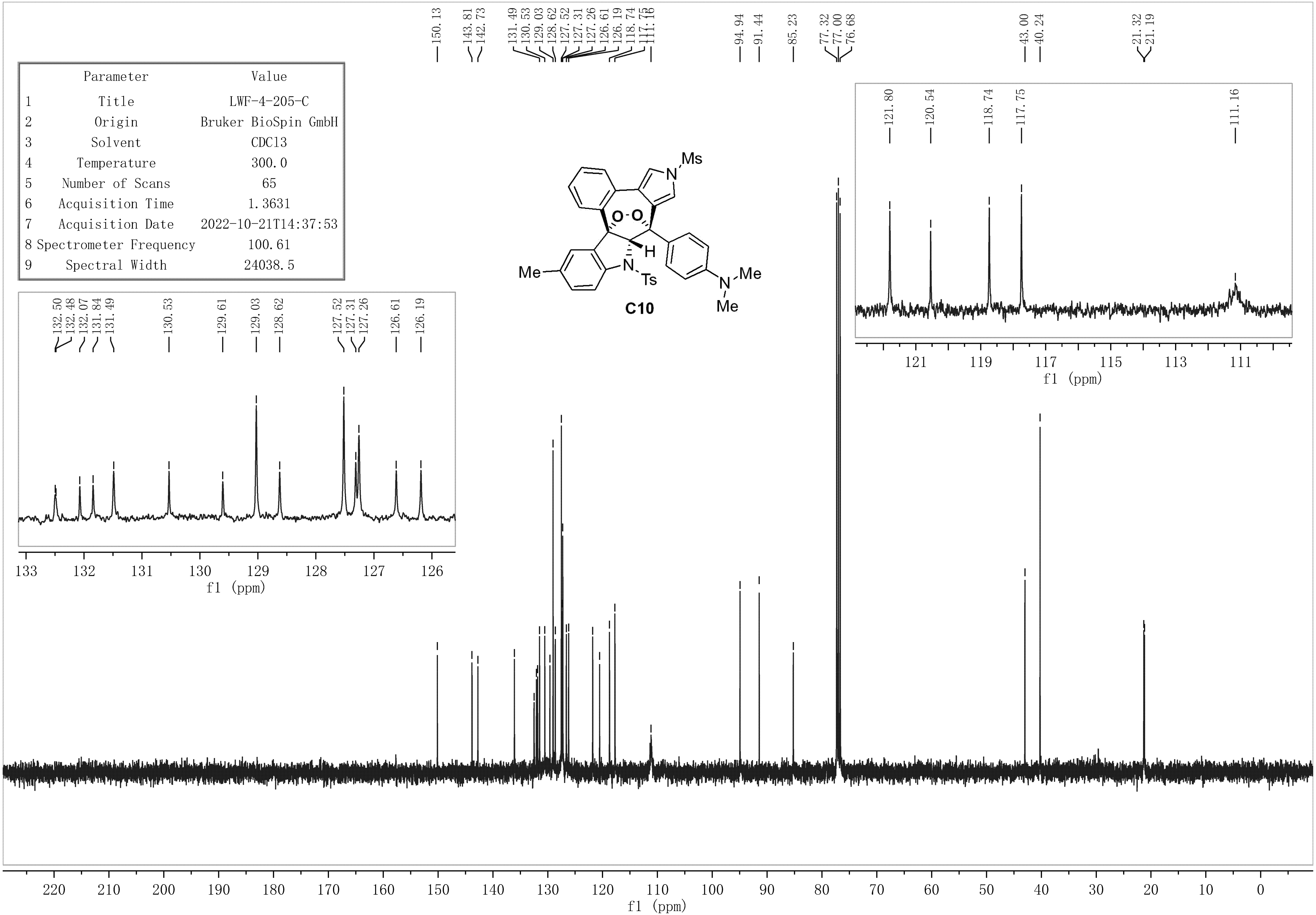

	Parameter	Value
1	Title	LWF-4-205-C
2	Origin	Bruker BioSpin GmbH
3	Solvent	CDCl3
4	Temperature	300.0
5	Number of Scans	65
6	Acquisition Time	1.3631
7	Acquisition Date	2022-10-21T14:37:53
8	Spectrometer Frequency	100.61
9	Spectral Width	24038.5

C10

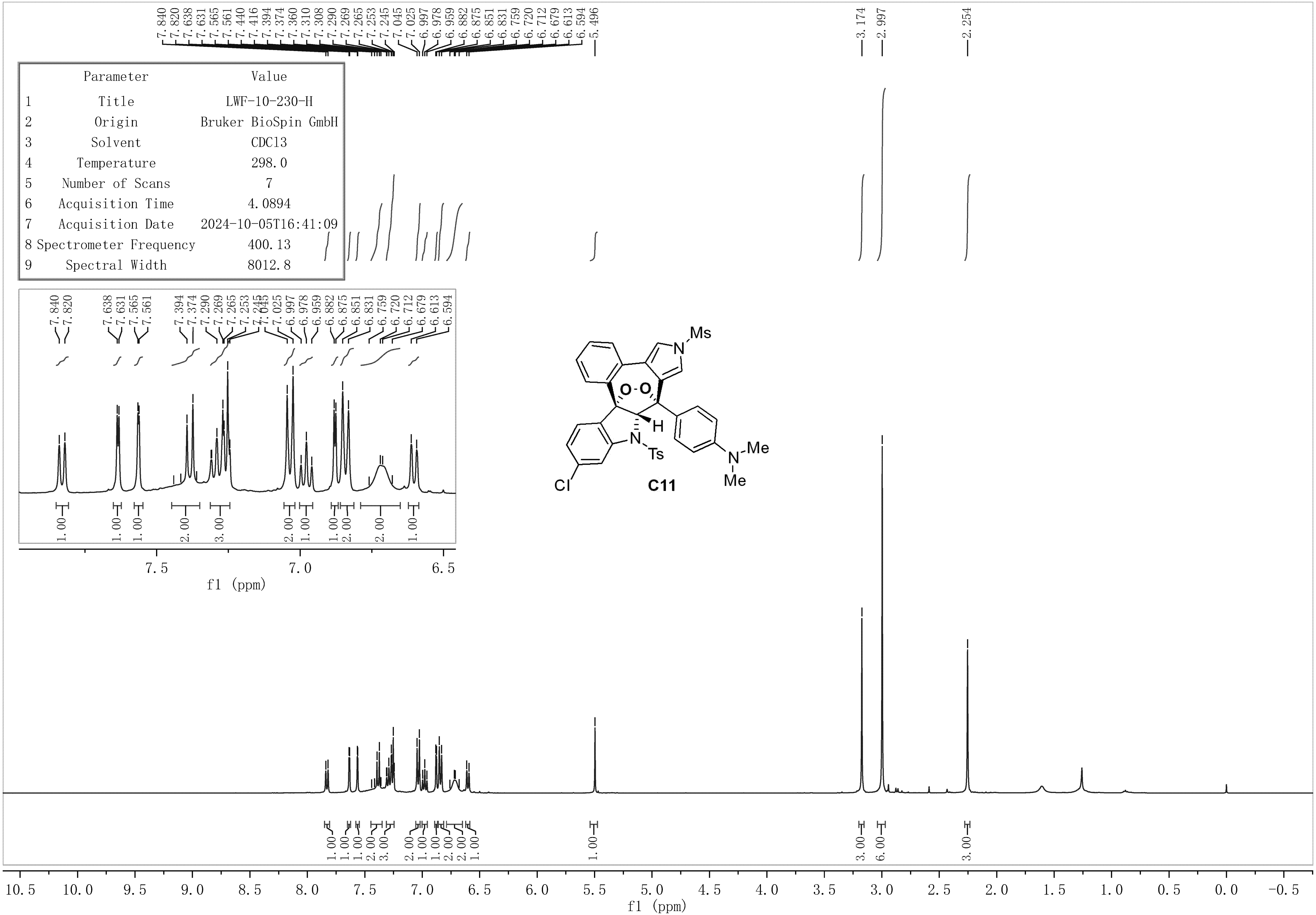

	Parameter	Value
1	Title	LWF-10-230-H
2	Origin	Bruker BioSpin GmbH
3	Solvent	CDCl3
4	Temperature	298.0
5	Number of Scans	7
6	Acquisition Time	4.0894
7	Acquisition Date	2024-10-05T16:41:09
8	Spectrometer Frequency	400.13
9	Spectral Width	8012.8

C11

	Parameter	Value
1	Title	LWF-10-230-C
2	Origin	Bruker BioSpin GmbH
3	Solvent	CDCl3
4	Temperature	300.0
5	Number of Scans	52
6	Acquisition Time	1.3631
7	Acquisition Date	2024-10-05T16:42:35
8	Spectrometer Frequency	100.61
9	Spectral Width	24038.5

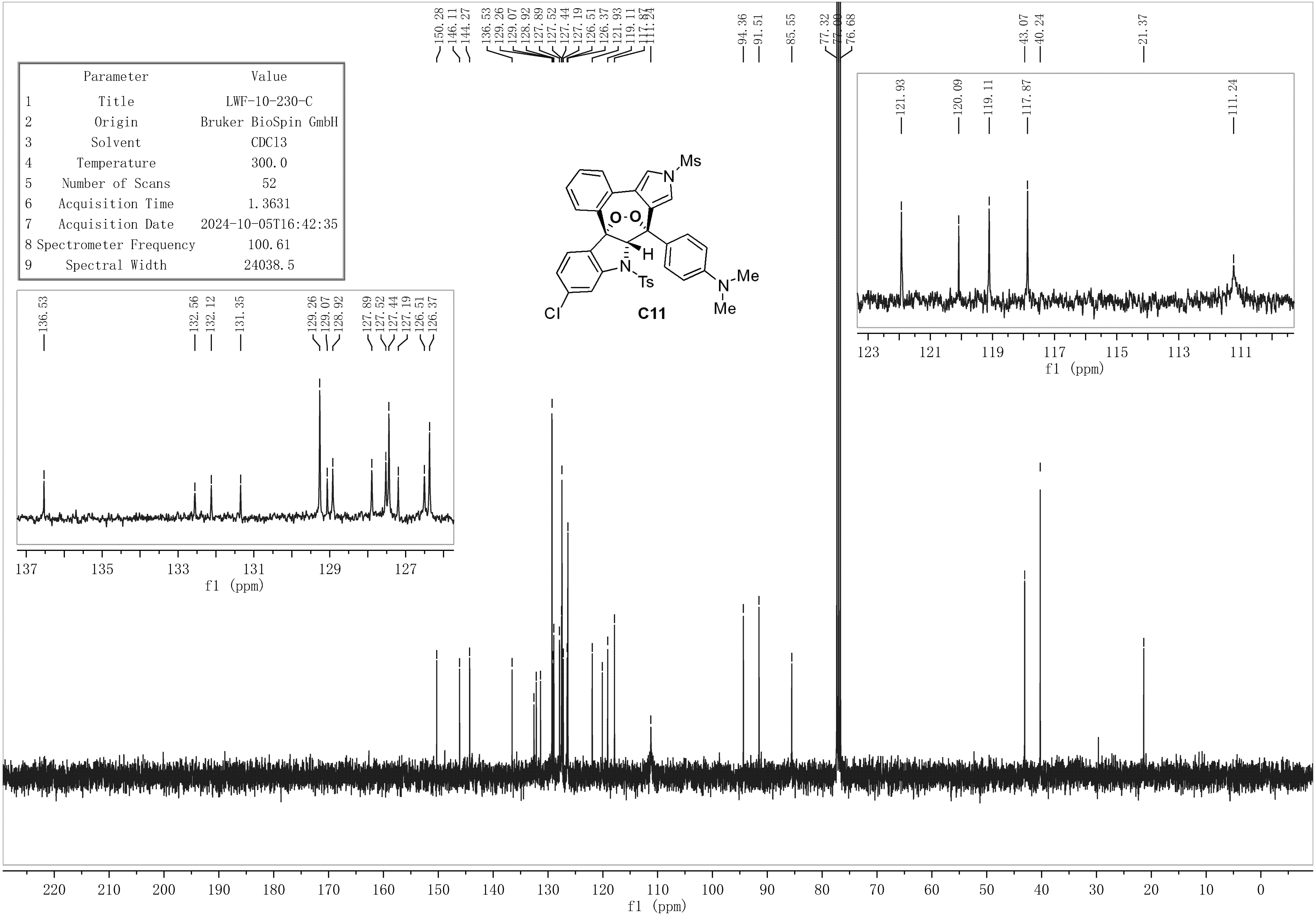

	Parameter	Value
1	Title	LWF-4-207-H
2	Origin	Bruker BioSpin GmbH
3	Solvent	CDCl3
4	Temperature	298.0
5	Number of Scans	6
6	Acquisition Time	4.0894
7	Acquisition Date	2022-10-26T14:19:37
8	Spectrometer Frequency	400.13
9	Spectral Width	8012.8

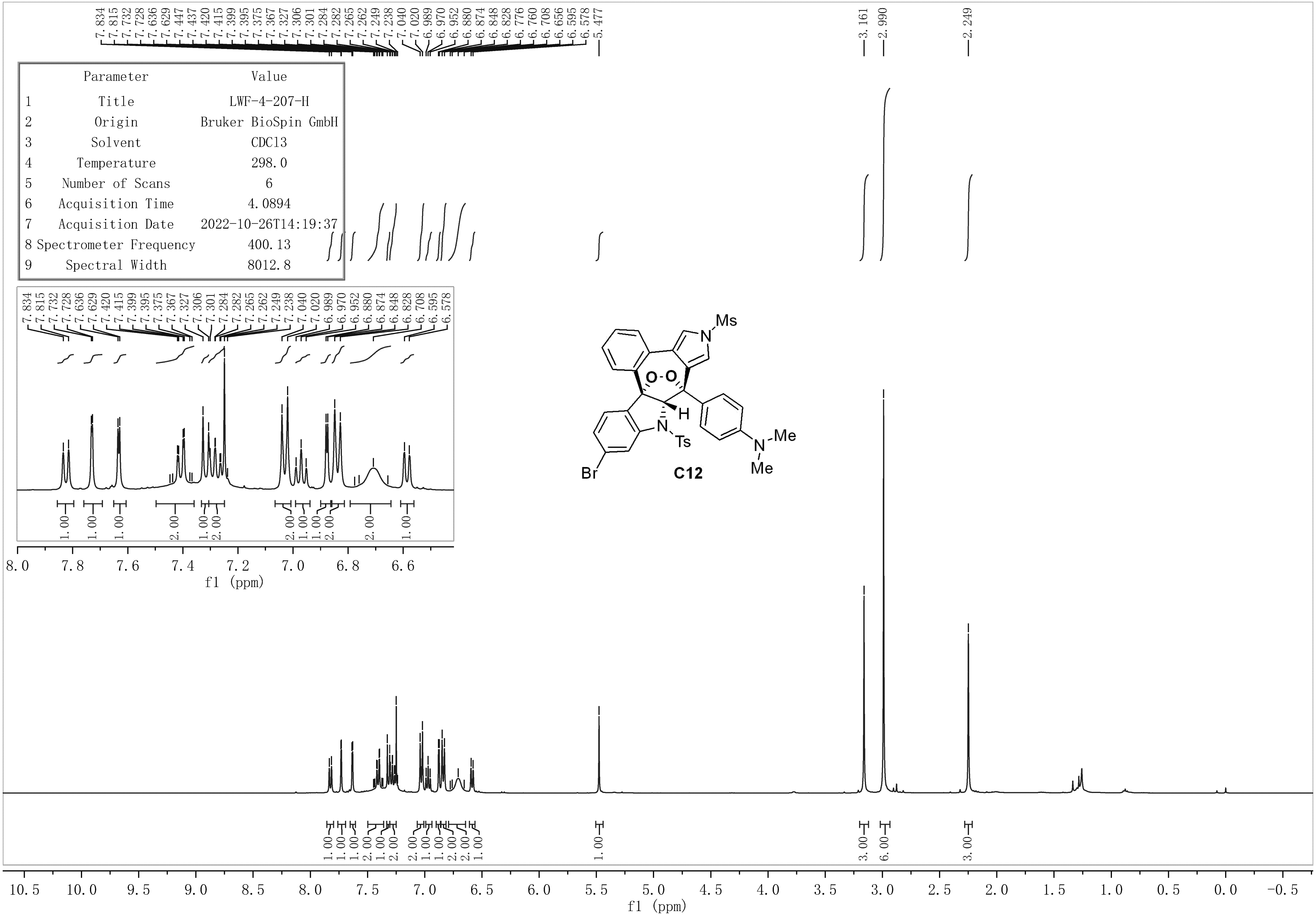

	Parameter	Value
1	Title	LWF-4-207-C
2	Origin	Bruker BioSpin GmbH
3	Solvent	CDCl3
4	Temperature	300.0
5	Number of Scans	61
6	Acquisition Time	1.3631
7	Acquisition Date	2022-10-26T14:20:55
8	Spectrometer Frequency	100.61
9	Spectral Width	24038.5

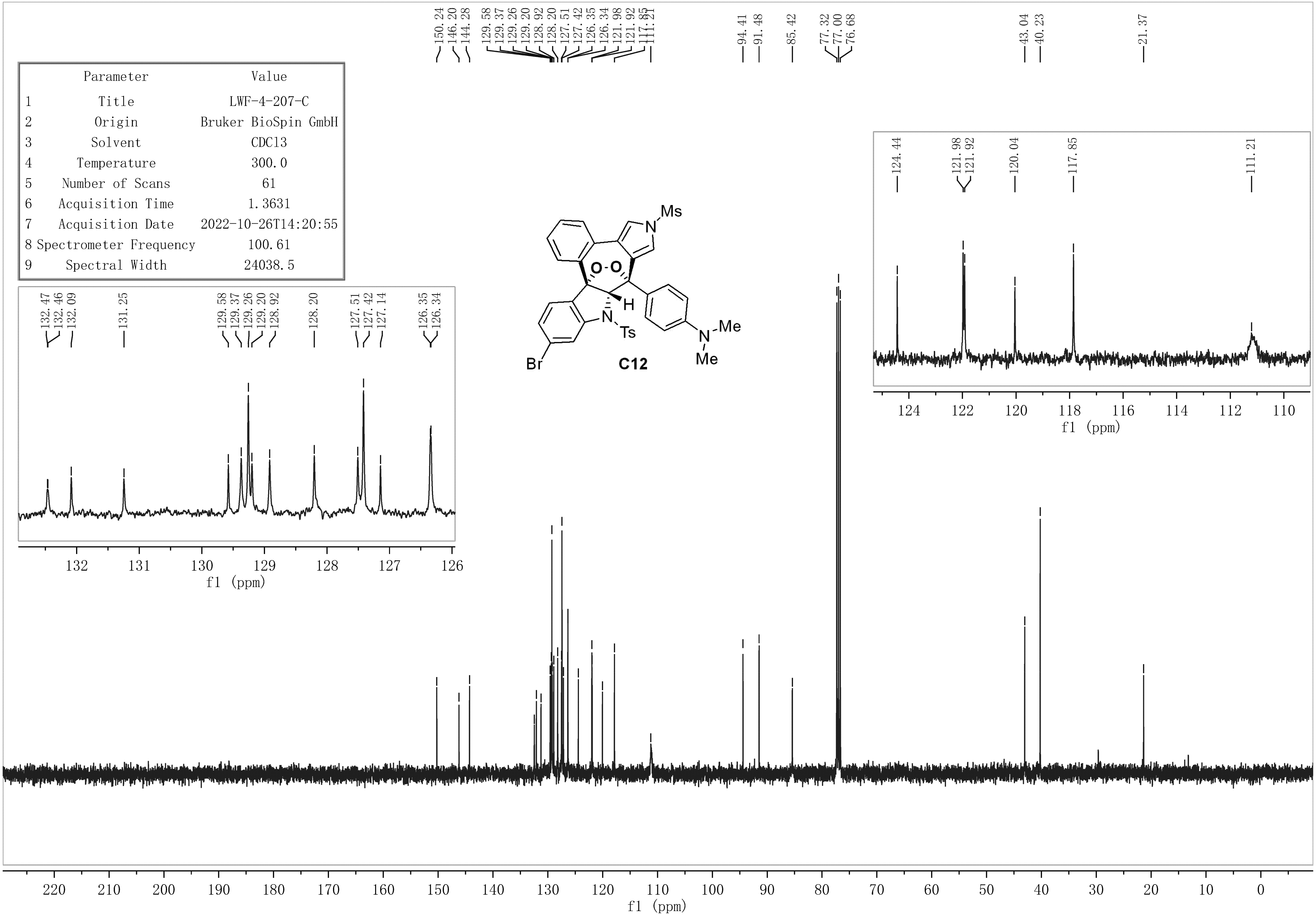

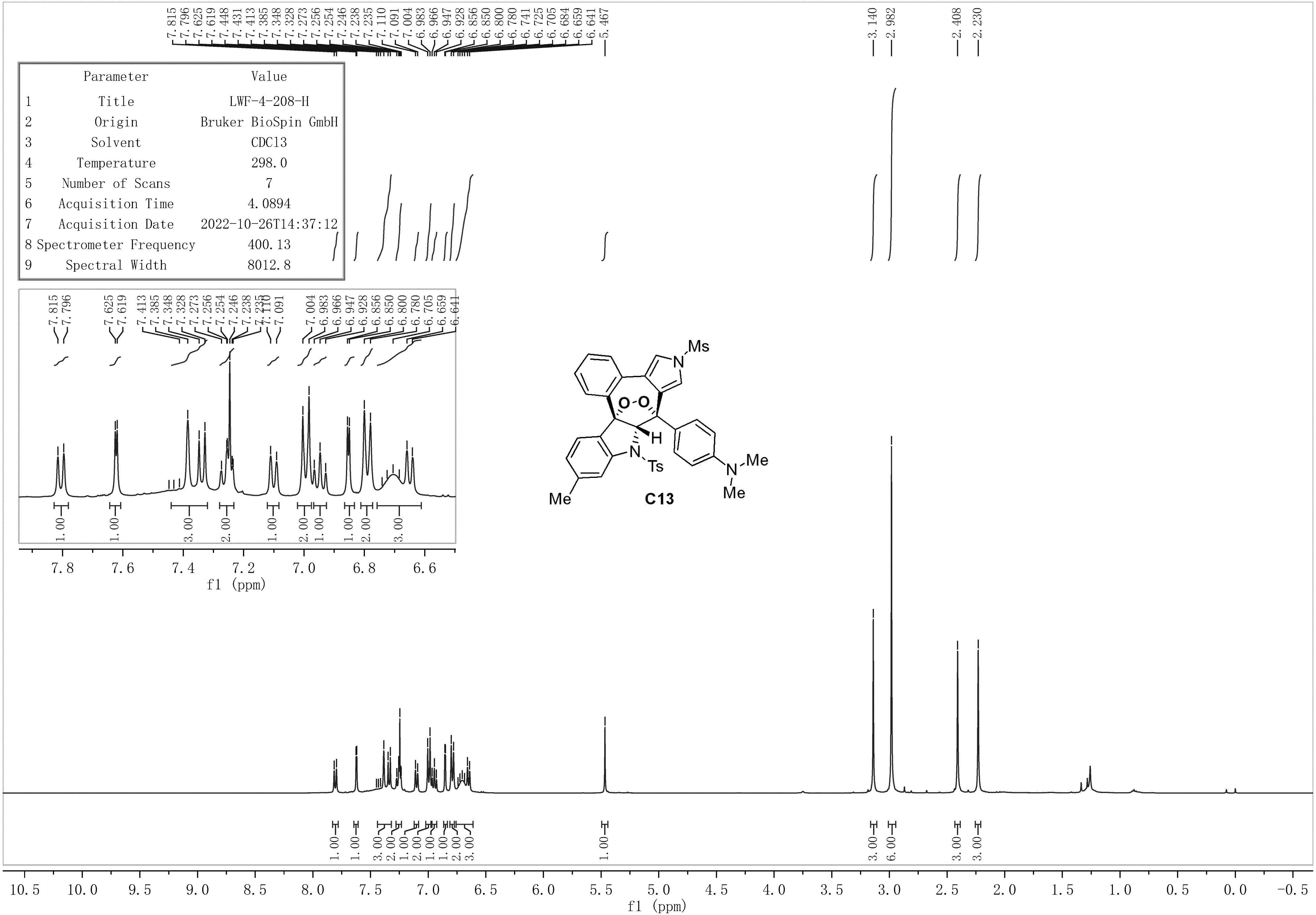

	Parameter	Value
1	Title	LWF-4-208-H
2	Origin	Bruker BioSpin GmbH
3	Solvent	CDCl3
4	Temperature	298.0
5	Number of Scans	7
6	Acquisition Time	4.0894
7	Acquisition Date	2022-10-26T14:37:12
8	Spectrometer Frequency	400.13
9	Spectral Width	8012.8

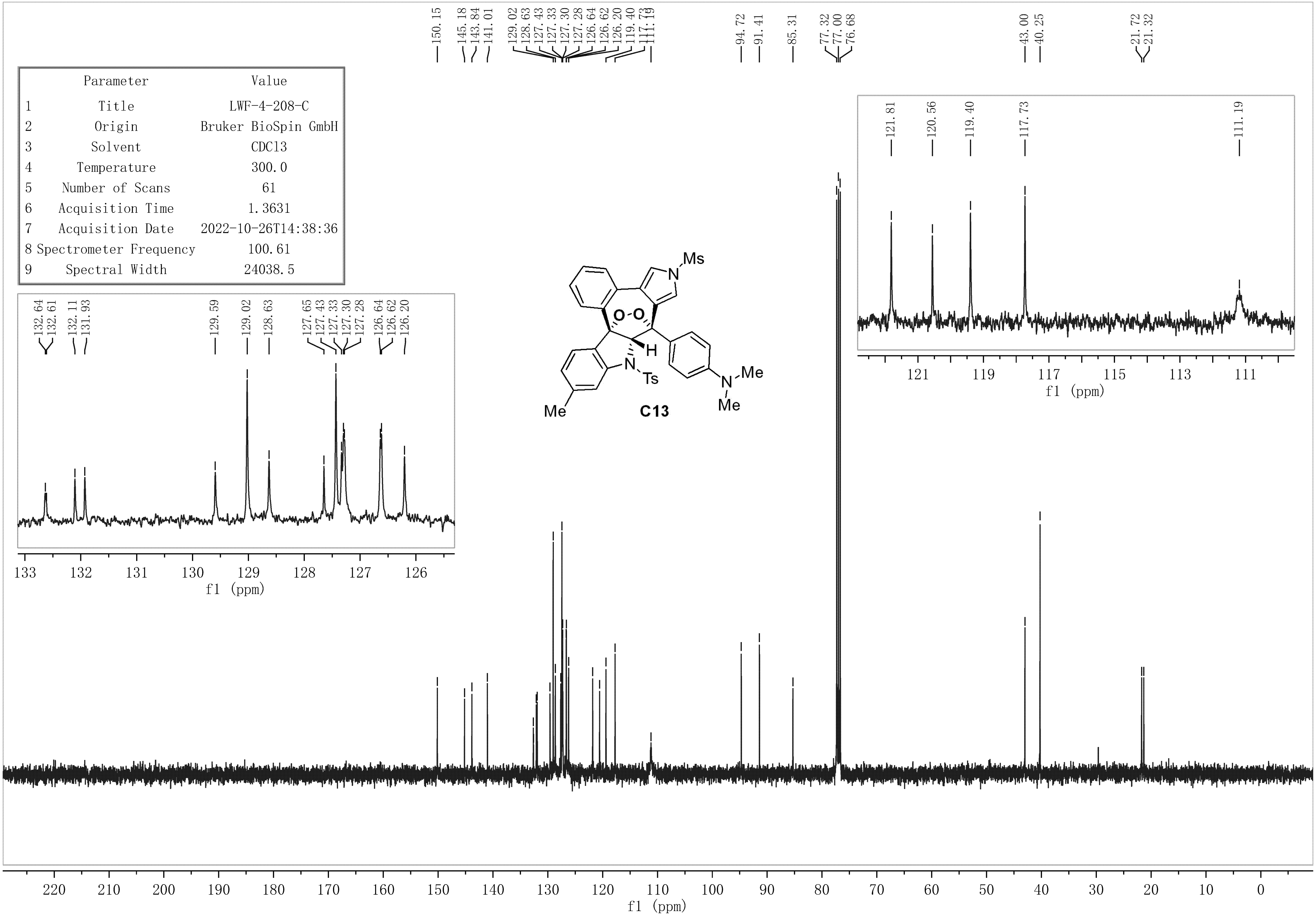
150.15
145.18
143.84
141.01
129.02
128.63
127.43
127.33
127.30
127.28
126.64
126.62
126.20
119.40
117.73
111.19
94.72
91.41
85.31
77.32
77.00
76.68
43.00
40.25
21.72
21.32
Parameter Value
1 Title LWF-4-208-C
2 Origin Bruker BioSpin GmbH
3 Solvent CDCl3
4 Temperature 300.0
5 Number of Scans 61
6 Acquisition Time 1.3631
7 Acquisition Date 2022-10-26T14:38:36
8 Spectrometer Frequency 100.61
9 Spectral Width 24038.5
132.64
132.61
132.11
131.93
129.59
129.02
128.63
127.65
127.43
127.33
127.30
127.28
126.64
126.62
126.20
133 132 131 130 129 128 127 126
f1 (ppm)
121.81
120.56
119.40
117.73
111.19
121 119 117 115 113 111
f1 (ppm)
Ms
N
O
O
H
N
Ts
N
Me
Me
Me
C13
220 210 200 190 180 170 160 150 140 130 120 110 100 90 80 70 60 50 40 30 20 10 0
f1 (ppm)

	Parameter	Value
1	Title	LWF-10-212-H
2	Origin	Bruker BioSpin GmbH
3	Solvent	CDCl3
4	Temperature	298.0
5	Number of Scans	5
6	Acquisition Time	4.0894
7	Acquisition Date	2024-10-01T17:54:03
8	Spectrometer Frequency	400.13
9	Spectral Width	8012.8

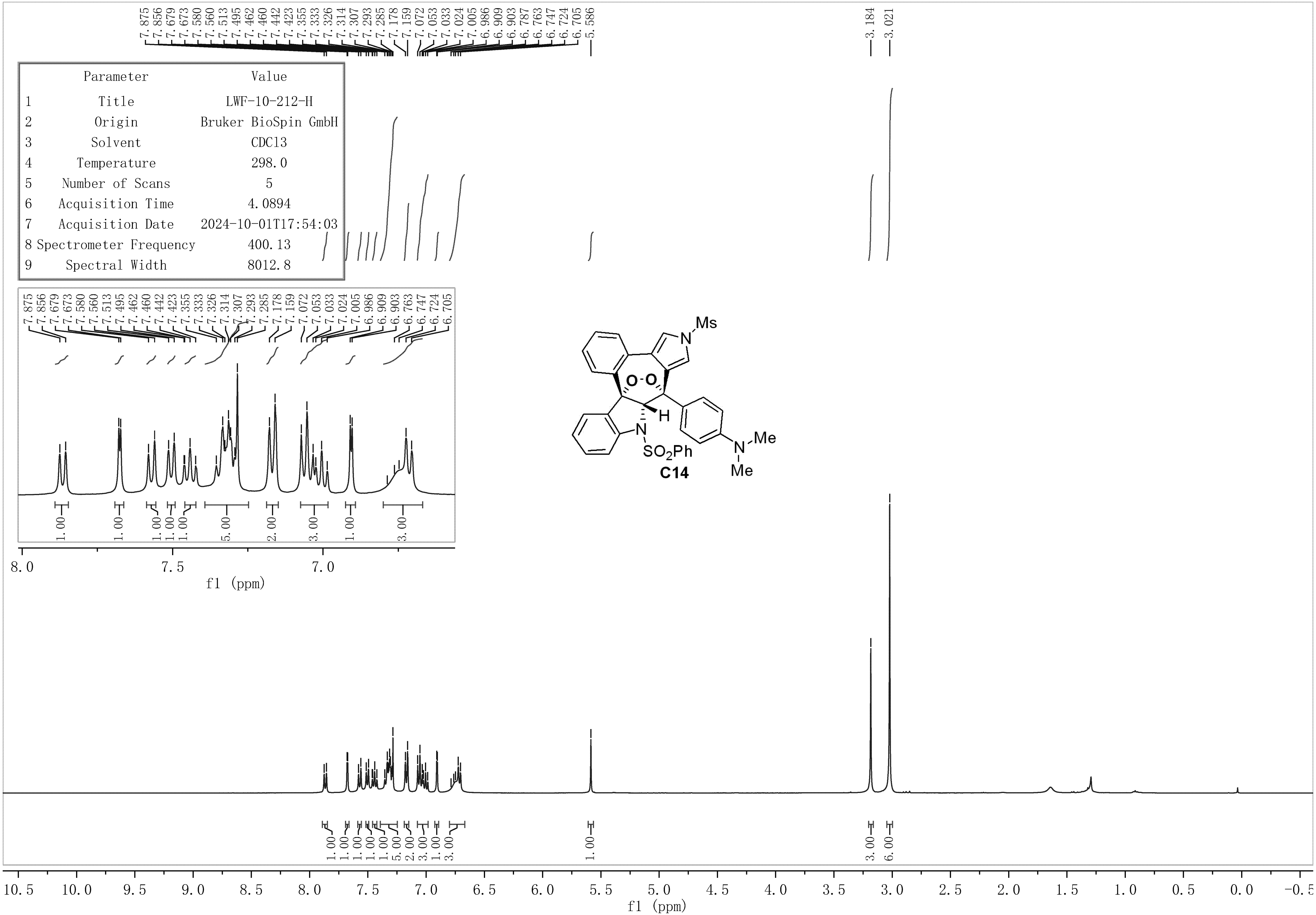

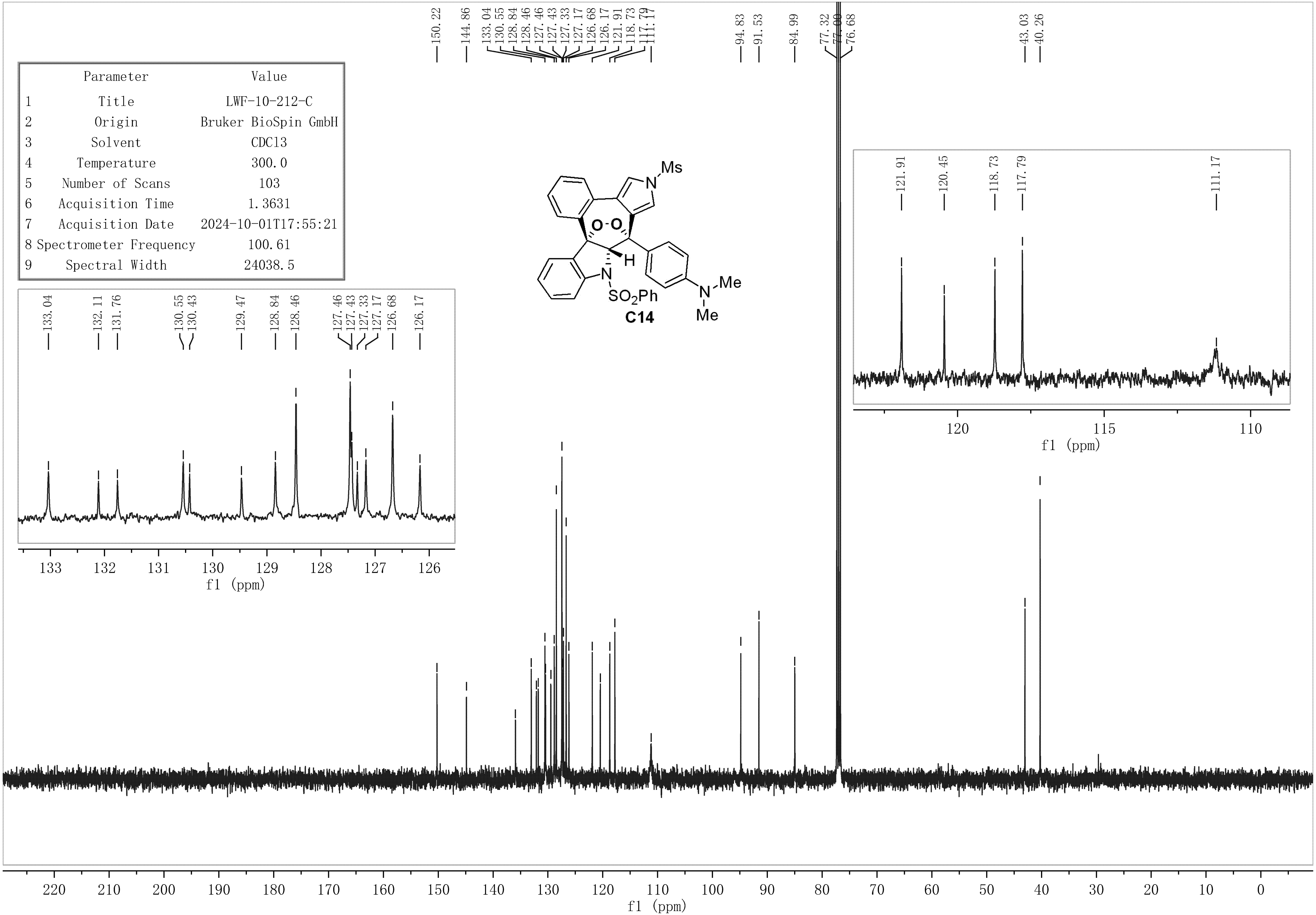

	Parameter	Value
1	Title	LWF-10-212-C
2	Origin	Bruker BioSpin GmbH
3	Solvent	CDCl3
4	Temperature	300.0
5	Number of Scans	103
6	Acquisition Time	1.3631
7	Acquisition Date	2024-10-01T17:55:21
8	Spectrometer Frequency	100.61
9	Spectral Width	24038.5

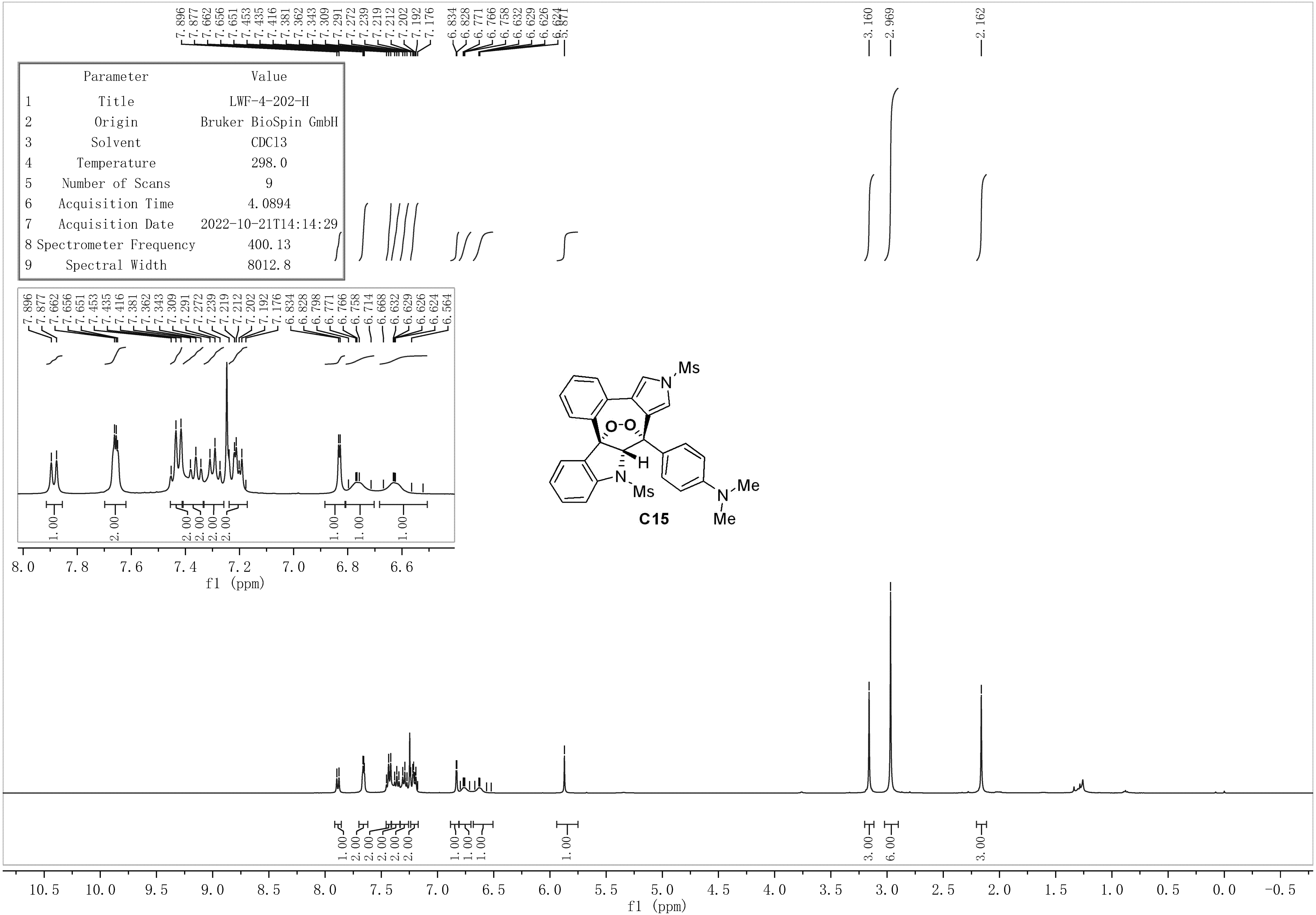

	Parameter	Value
1	Title	LWF-4-202-H
2	Origin	Bruker BioSpin GmbH
3	Solvent	CDCl3
4	Temperature	298.0
5	Number of Scans	9
6	Acquisition Time	4.0894
7	Acquisition Date	2022-10-21T14:14:29
8	Spectrometer Frequency	400.13
9	Spectral Width	8012.8

C15

	Parameter	Value
1	Title	LWF-4-202-C
2	Origin	Bruker BioSpin GmbH
3	Solvent	CDCl3
4	Temperature	300.0
5	Number of Scans	121
6	Acquisition Time	1.3631
7	Acquisition Date	2022-10-21T14:16:20
8	Spectrometer Frequency	100.61
9	Spectral Width	24038.5

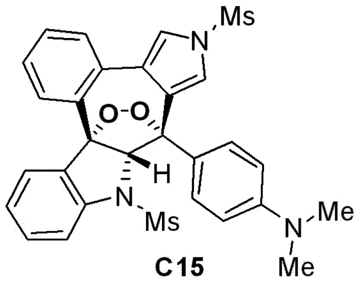

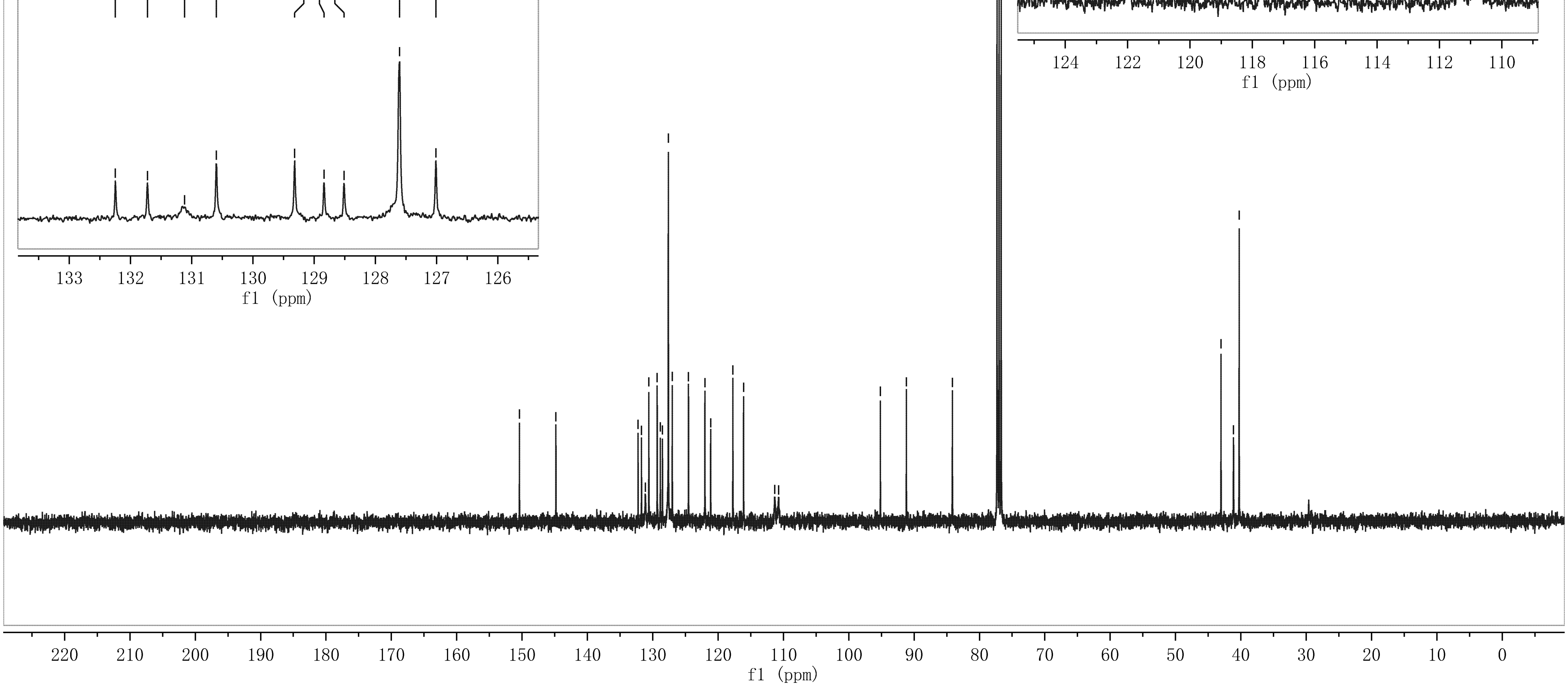

	Parameter	Value
1	Title	LWF-4-235-H
2	Origin	
3	Solvent	DMSO
4	Temperature	297.8
5	Number of Scans	4
6	Acquisition Time	4.0002
7	Acquisition Date	2022-11-08T09:05:30
8	Spectrometer Frequency	399.90
9	Spectral Width	8012.0

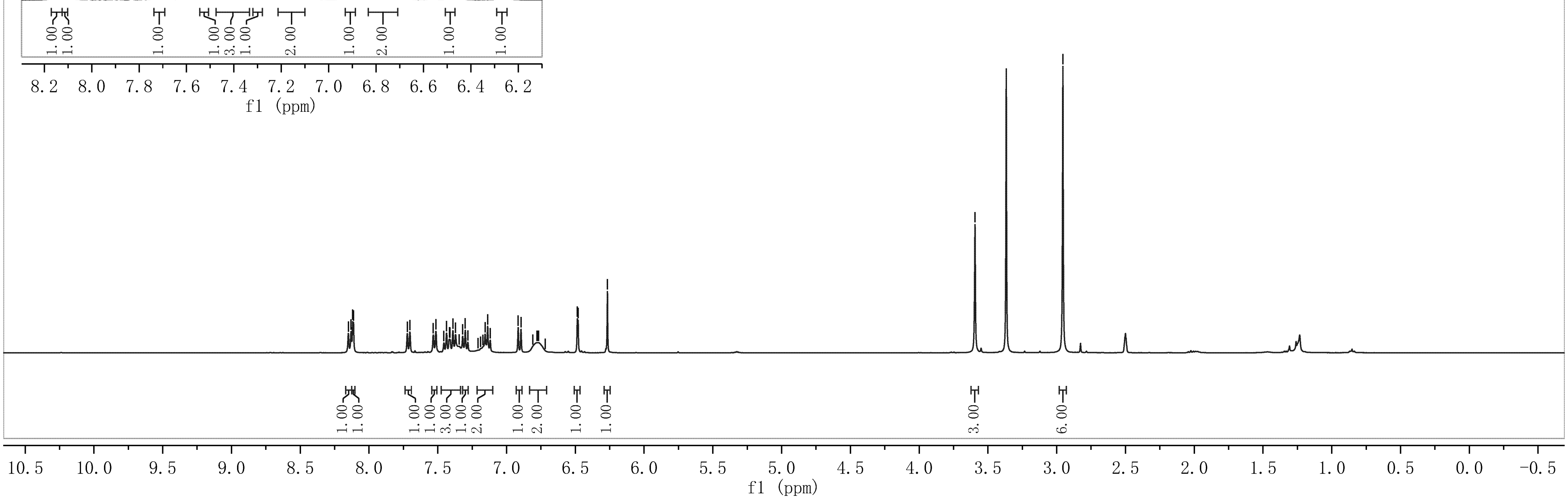

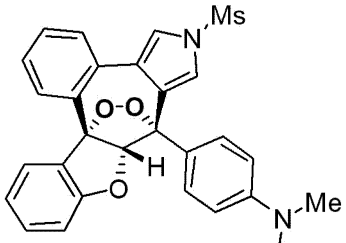

C16

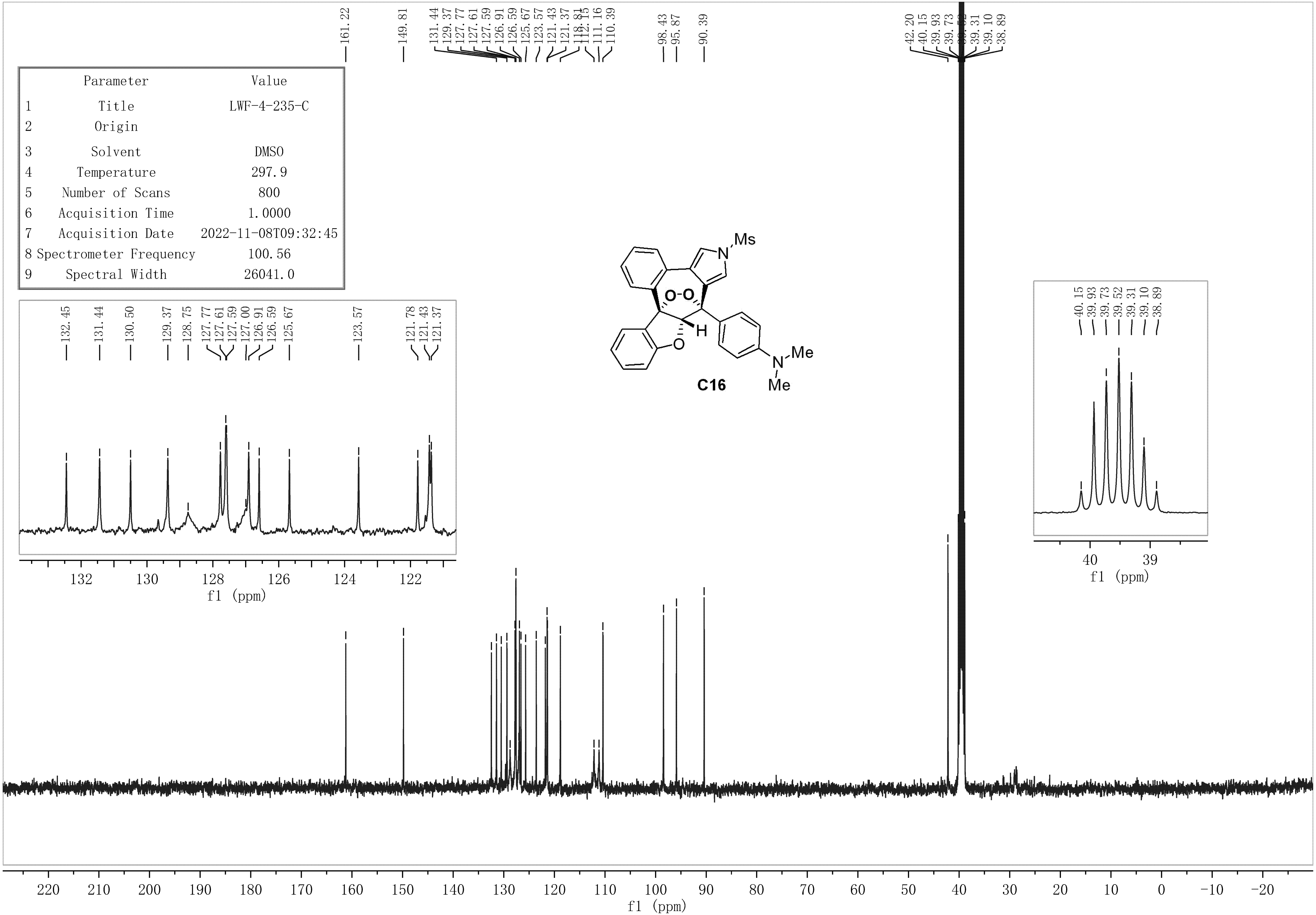

161.22
149.81
131.44
129.37
127.77
127.61
127.59
126.91
126.59
125.67
123.57
121.43
121.37
111.16
110.39
98.43
95.87
90.39
42.20
40.15
39.93
39.73
39.31
39.10
38.89
Parameter Value
1 Title LWF-4-235-C
2 Origin
3 Solvent DMSO
4 Temperature 297.9
5 Number of Scans 800
6 Acquisition Time 1.0000
7 Acquisition Date 2022-11-08T09:32:45
8 Spectrometer Frequency 100.56
9 Spectral Width 26041.0
132.45
131.44
130.50
129.37
128.75
127.77
127.61
127.59
127.00
126.91
126.59
125.67
123.57
121.78
121.43
121.37
132 130 128 126 124 122
f1 (ppm)
Ms
N
O-O
H
O
N
Me
Me
C16
40.15
39.93
39.73
39.52
39.31
39.10
38.89
40 39
f1 (ppm)
220 210 200 190 180 170 160 150 140 130 120 110 100 90 80 70 60 50 40 30 20 10 0 -10 -20
f1 (ppm)

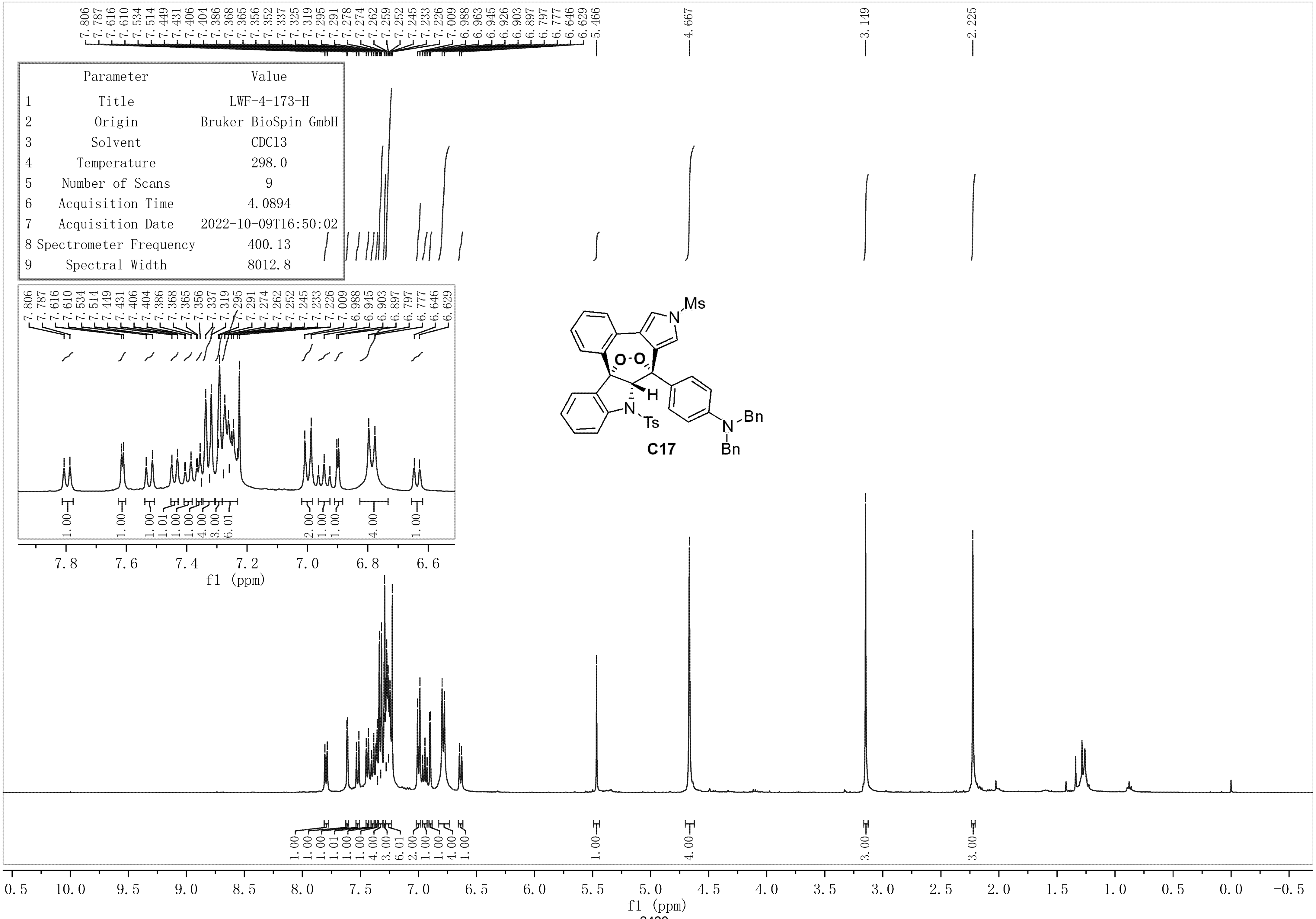
7.806 7.787 7.616 7.610 7.534 7.514 7.449 7.431 7.406 7.404 7.386 7.368 7.365 7.356 7.352 7.337 7.325 7.319 7.295 7.291 7.278 7.274 7.262 7.259 7.252 7.245 7.233 7.226 7.009 6.988 6.963 6.945 6.926 6.903 6.897 6.797 6.777 6.646 6.629 5.466
4.667
3.149
2.225
Parameter Value
1 Title LWF-4-173-H
2 Origin Bruker BioSpin GmbH
3 Solvent CDCl3
4 Temperature 298.0
5 Number of Scans 9
6 Acquisition Time 4.0894
7 Acquisition Date 2022-10-09T16:50:02
8 Spectrometer Frequency 400.13
9 Spectral Width 8012.8
Ms
N
O-O
H
N
Ts
N
Bn
Bn
C17
1.00 1.00 1.00 1.01 1.00 1.00 4.00 3.00 6.01 2.00 1.00 1.00 4.00 1.00
1.00
4.00
3.00
3.00
f1 (ppm)

	Parameter	Value
1	Title	LWF-4-173-C
2	Origin	Bruker BioSpin GmbH
3	Solvent	CDCl3
4	Temperature	300.0
5	Number of Scans	62
6	Acquisition Time	1.3631
7	Acquisition Date	2022-10-09T16:51:42
8	Spectrometer Frequency	100.61
9	Spectral Width	24038.5

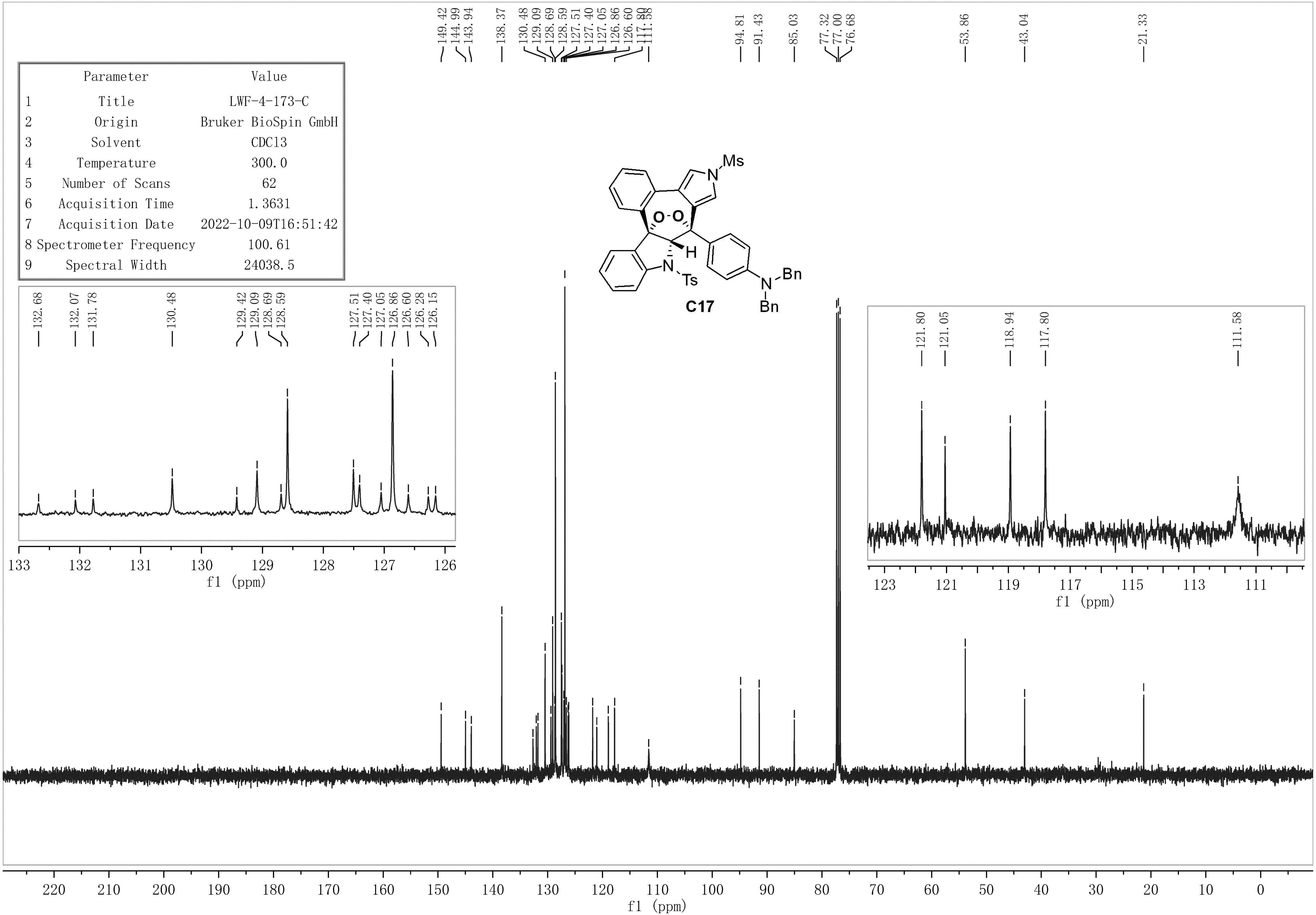

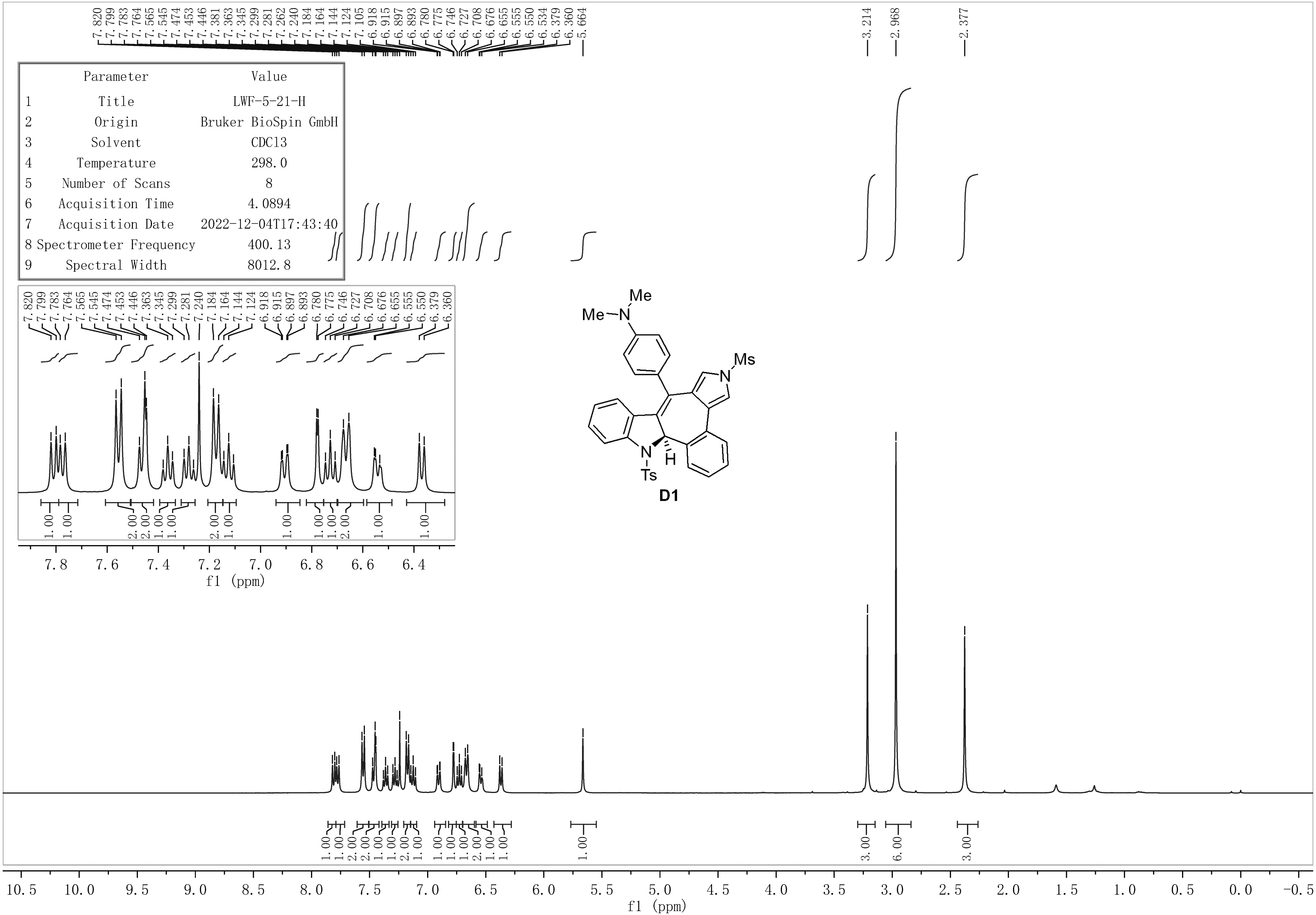

	Parameter	Value
1	Title	LWF-5-21-H
2	Origin	Bruker BioSpin GmbH
3	Solvent	CDCl3
4	Temperature	298.0
5	Number of Scans	8
6	Acquisition Time	4.0894
7	Acquisition Date	2022-12-04T17:43:40
8	Spectrometer Frequency	400.13
9	Spectral Width	8012.8

	Parameter	Value
1	Title	LWF-5-21-C
2	Origin	Bruker BioSpin GmbH
3	Solvent	CDCl3
4	Temperature	300.0
5	Number of Scans	71
6	Acquisition Time	1.3631
7	Acquisition Date	2022-12-04T17:45:29
8	Spectrometer Frequency	100.61
9	Spectral Width	24038.5

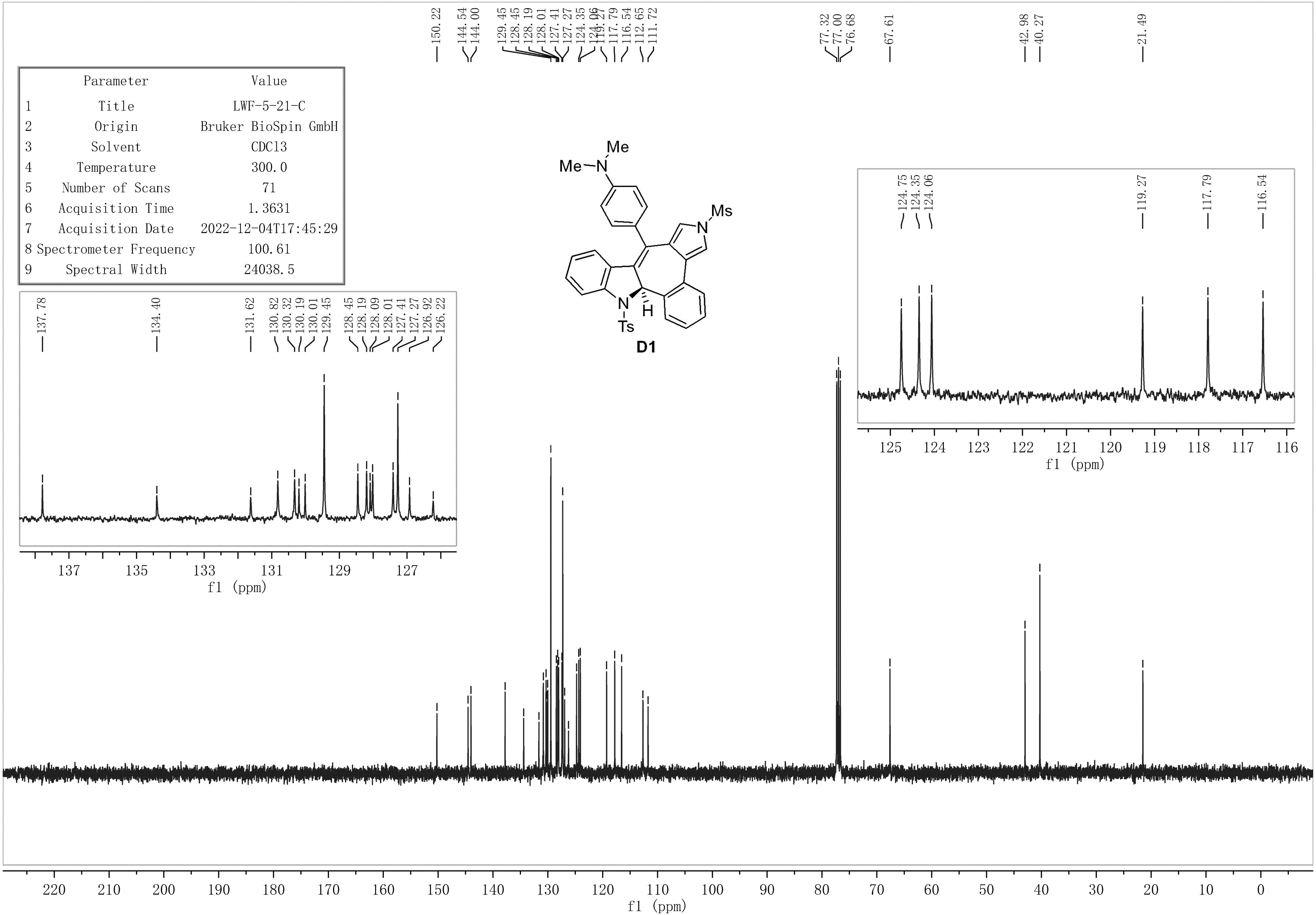

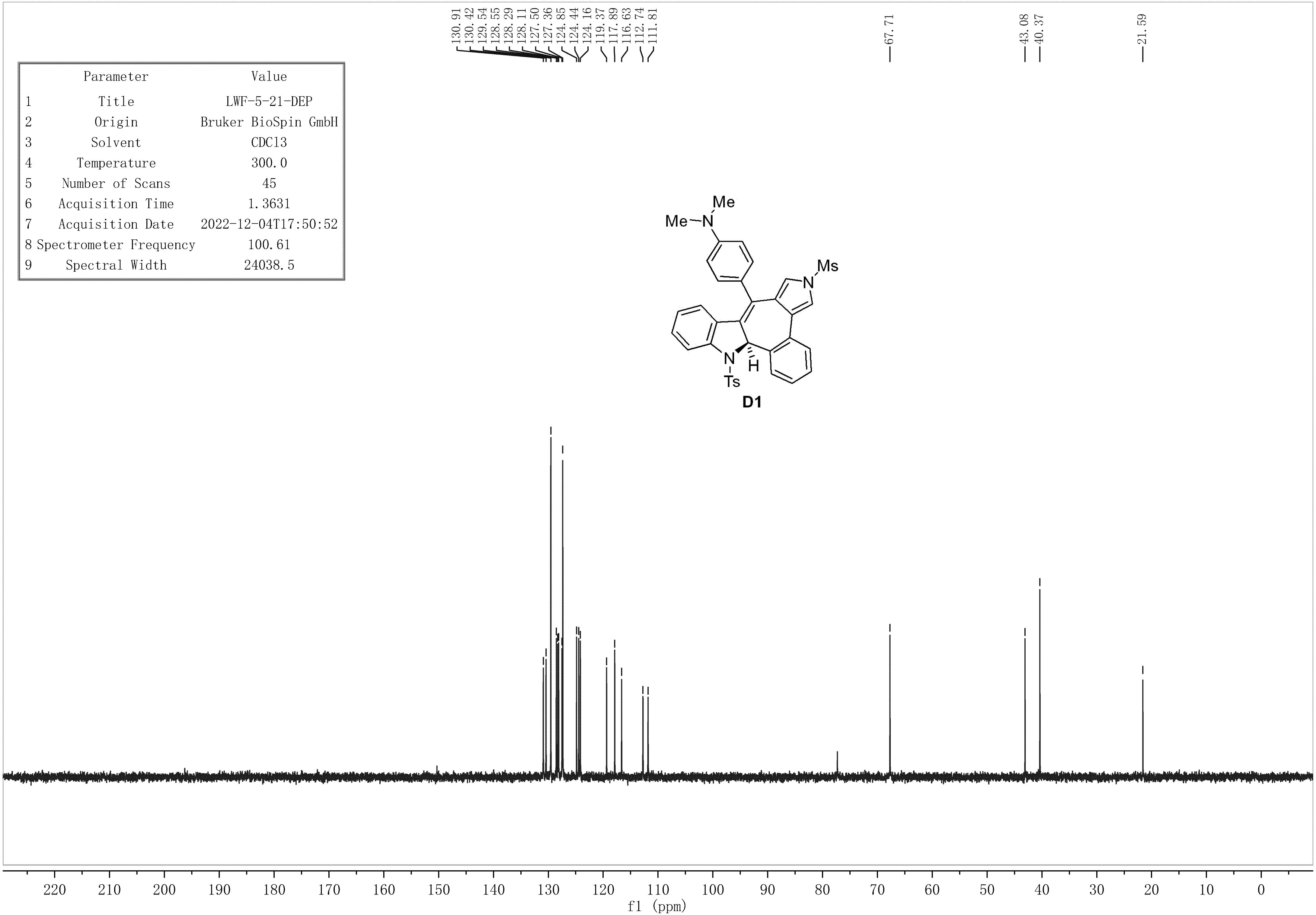

	Parameter	Value
1	Title	LWF-5-21-DEP
2	Origin	Bruker BioSpin GmbH
3	Solvent	CDCl3
4	Temperature	300.0
5	Number of Scans	45
6	Acquisition Time	1.3631
7	Acquisition Date	2022-12-04T17:50:52
8	Spectrometer Frequency	100.61
9	Spectral Width	24038.5

Parameter		Value
1	Title	LWF-5-96-H
2	Origin	Bruker BioSpin GmbH
3	Solvent	CDCl3
4	Temperature	298.0
5	Number of Scans	8
6	Acquisition Time	4.0894
7	Acquisition Date	2022-12-12T17:38:31
8	Spectrometer Frequency	400.13
9	Spectral Width	8012.8

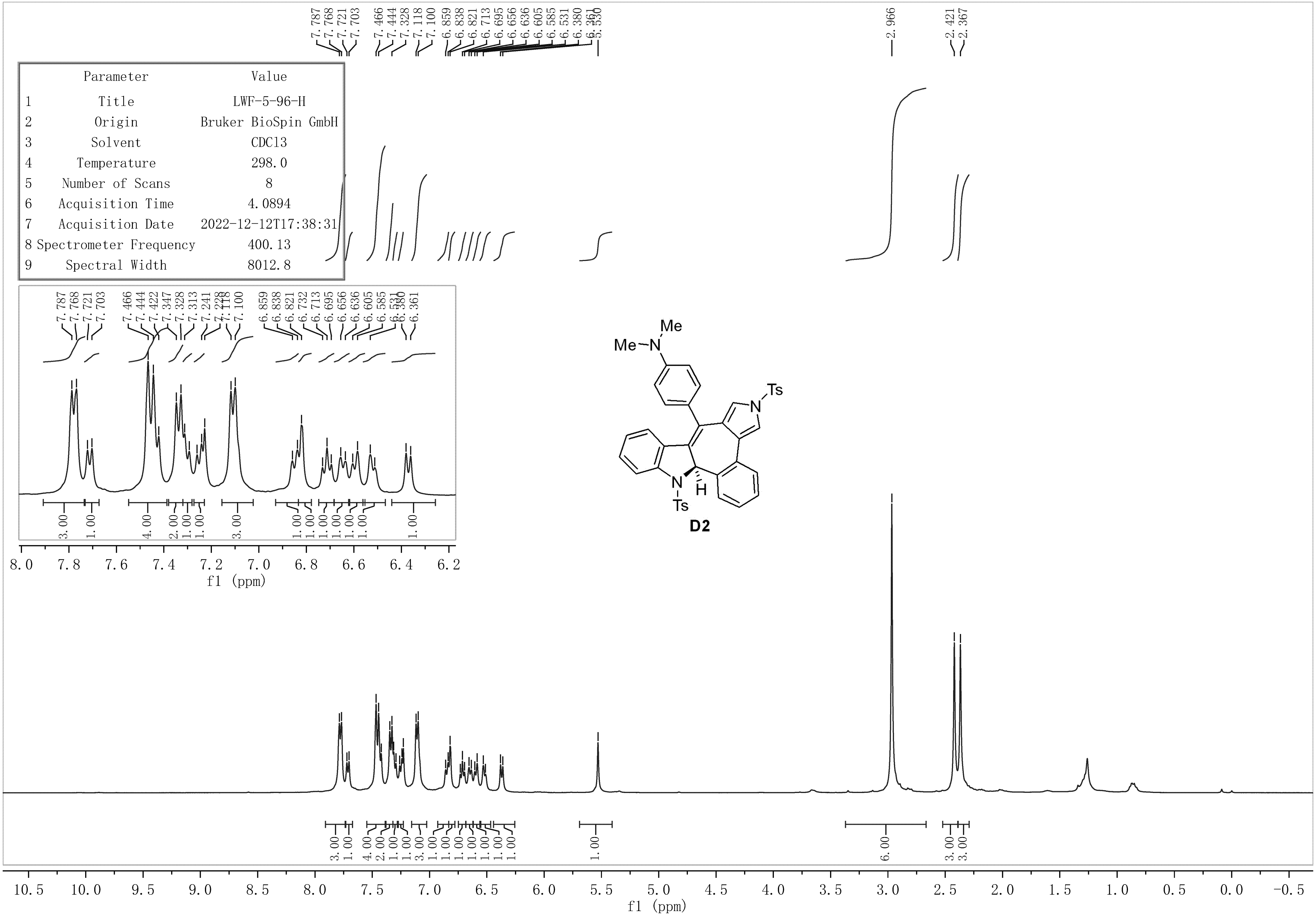

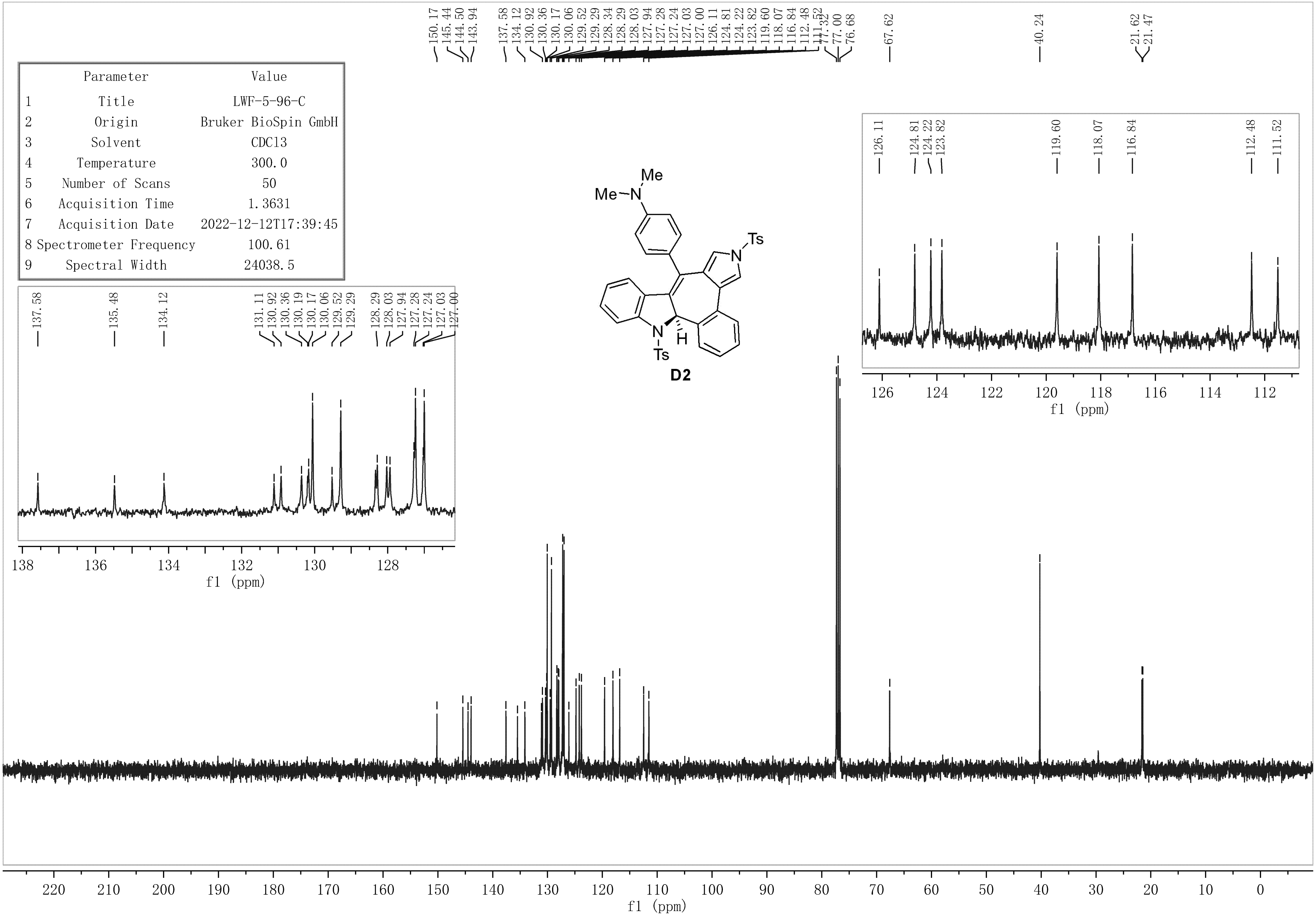

	Parameter	Value
1	Title	LWF-5-96-C
2	Origin	Bruker BioSpin GmbH
3	Solvent	CDCl3
4	Temperature	300.0
5	Number of Scans	50
6	Acquisition Time	1.3631
7	Acquisition Date	2022-12-12T17:39:45
8	Spectrometer Frequency	100.61
9	Spectral Width	24038.5

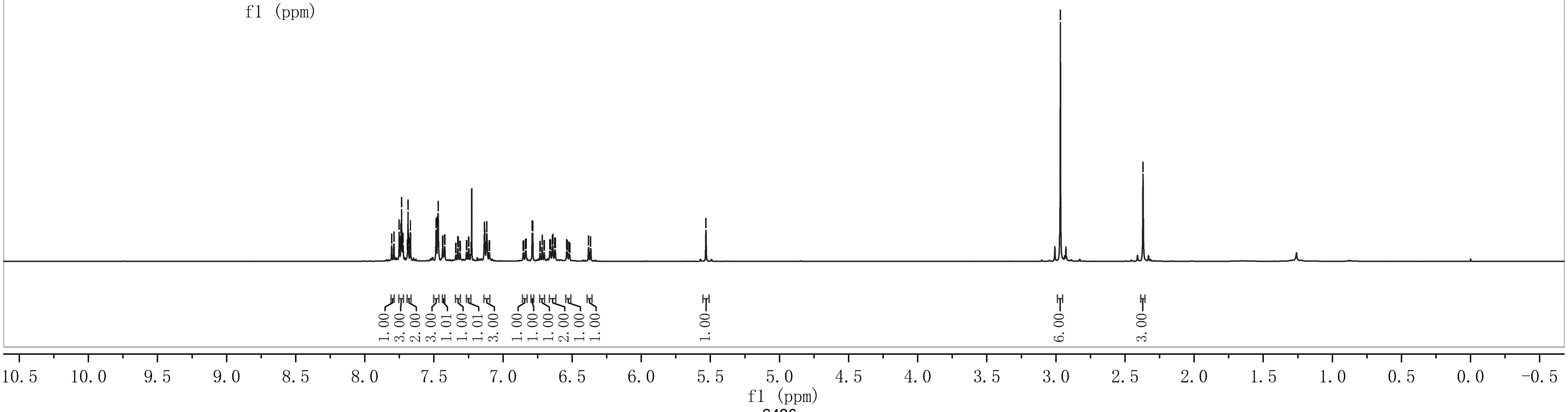

	Parameter	Value
1	Title	LWF-10-218-H
2	Origin	Bruker BioSpin GmbH
3	Solvent	CDCl3
4	Temperature	296.6
5	Number of Scans	7
6	Acquisition Time	3.1719
7	Acquisition Date	2024-10-05T11:05:07
8	Spectrometer Frequency	500.17
9	Spectral Width	10330.6

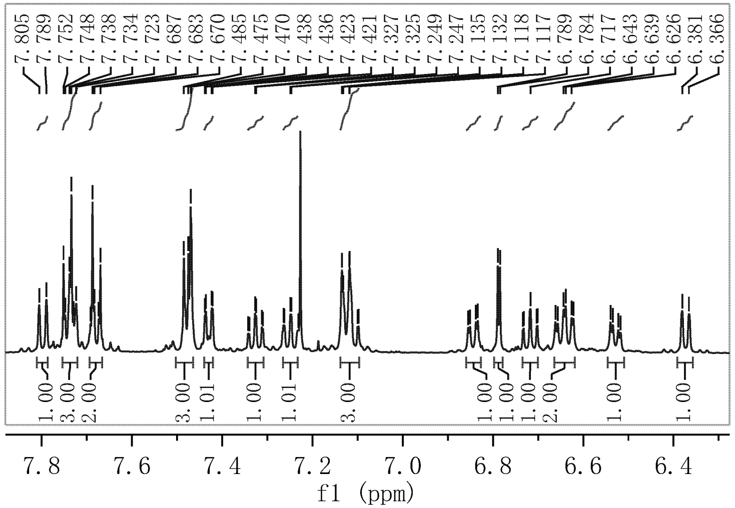

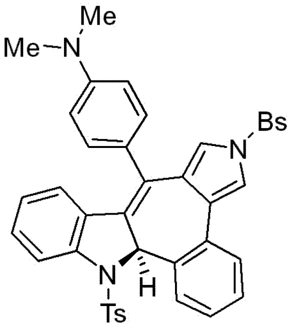

D3

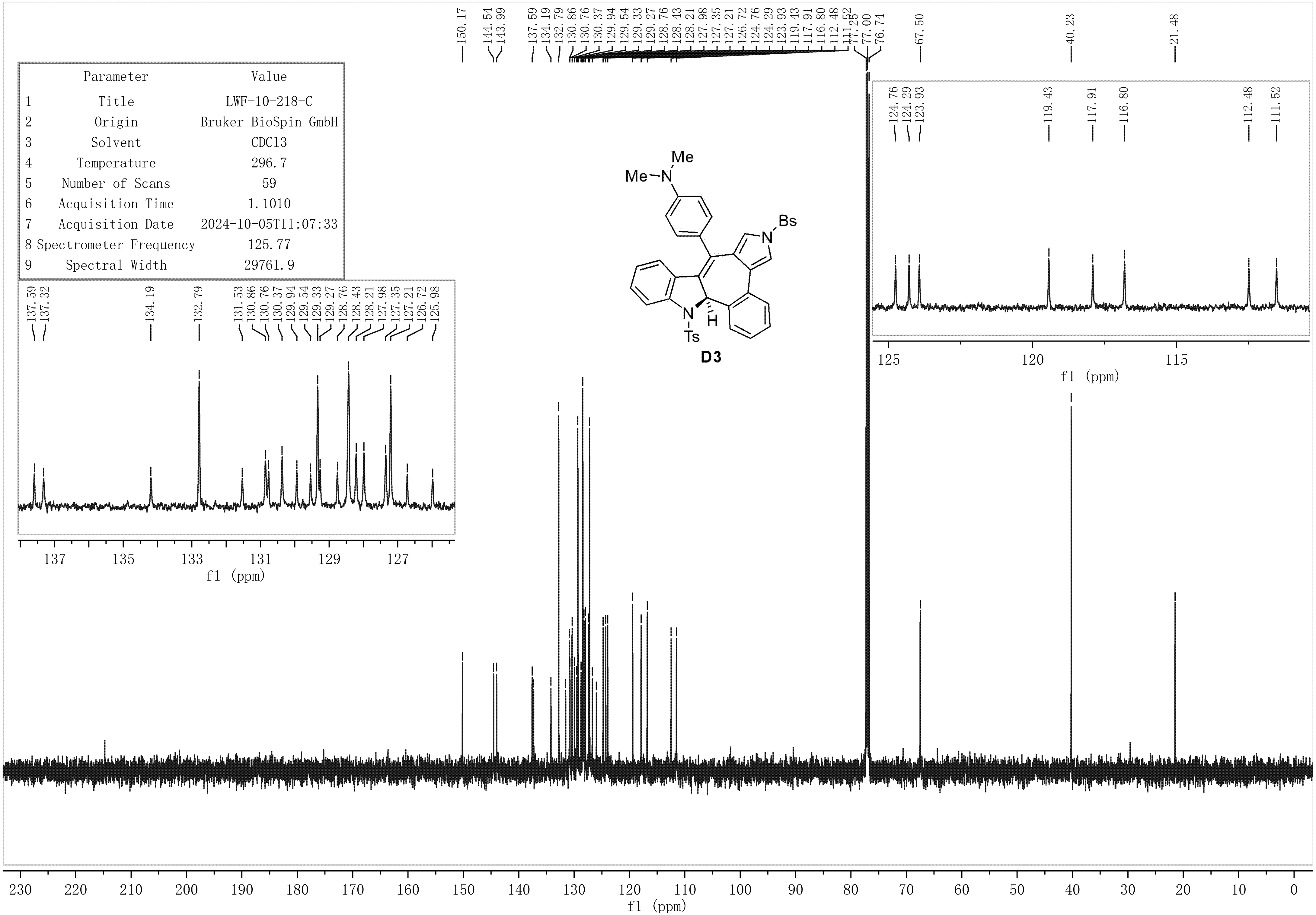
150.17
144.54
143.99
137.59
134.19
132.79
130.86
130.76
130.37
129.94
129.54
129.33
129.27
128.76
128.43
128.21
127.98
127.35
127.21
126.72
124.76
124.29
123.93
119.43
117.91
116.80
112.48
111.52
77.25
77.00
76.74
67.50
40.23
21.48
Parameter Value
1 Title LWF-10-218-C
2 Origin Bruker BioSpin GmbH
3 Solvent CDCl3
4 Temperature 296.7
5 Number of Scans 59
6 Acquisition Time 1.1010
7 Acquisition Date 2024-10-05T11:07:33
8 Spectrometer Frequency 125.77
9 Spectral Width 29761.9
Me
Me
N
Bs
N
N
H
Ts
D3
137.59
137.32
134.19
132.79
131.53
130.86
130.76
130.37
129.94
129.54
129.33
129.27
128.76
128.43
128.21
127.98
127.35
127.21
126.72
125.98
137
135
133
131
129
127
f1 (ppm)
124.76
124.29
123.93
119.43
117.91
116.80
112.48
111.52
125
120
115
f1 (ppm)
230
220
210
200
190
180
170
160
150
140
130
120
110
100
90
80
70
60
50
40
30
20
10
0
f1 (ppm)

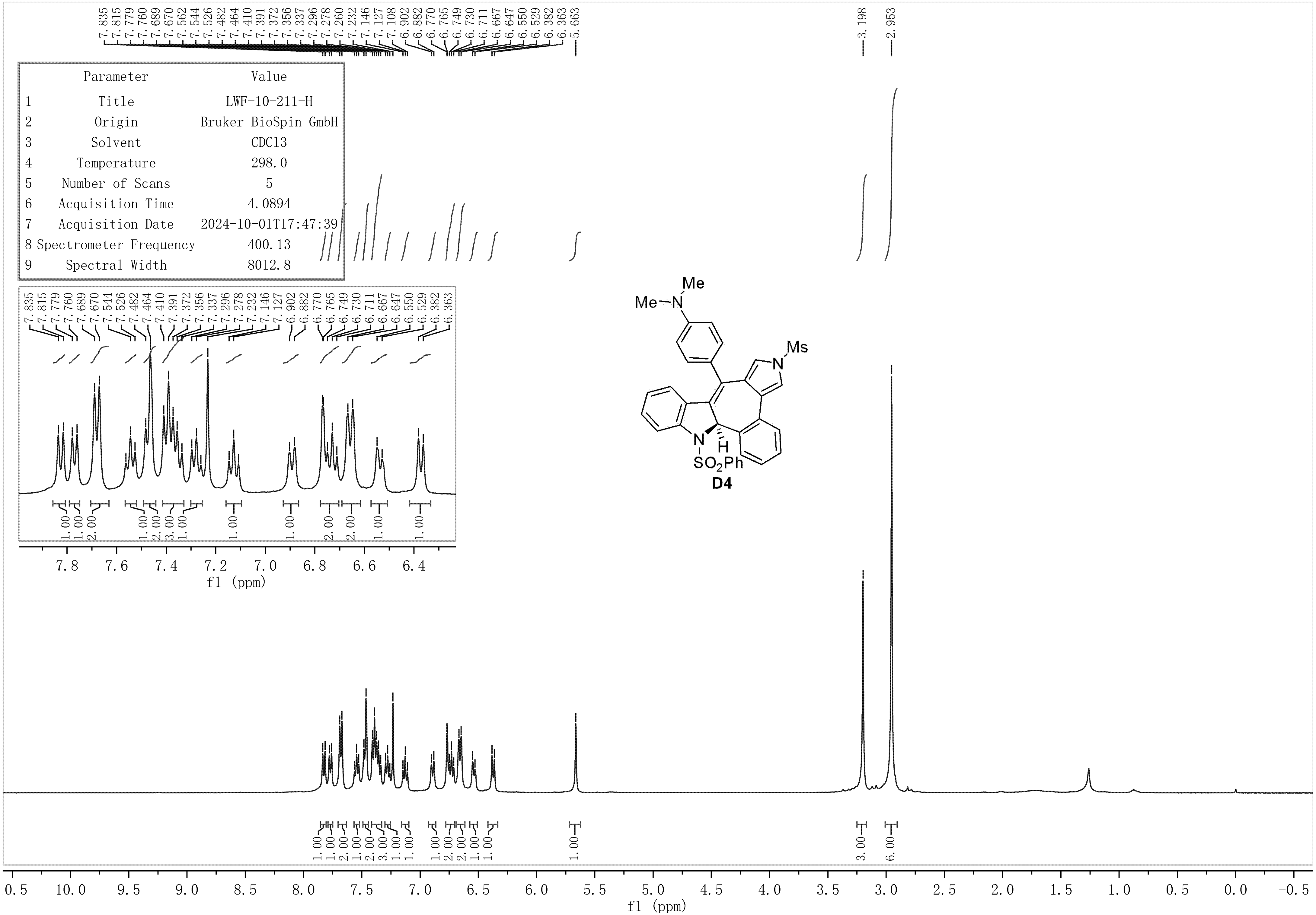
7.835 7.815 7.779 7.760 7.689 7.670 7.562 7.544 7.526 7.482 7.464 7.410 7.391 7.372 7.356 7.337 7.296 7.278 7.260 7.232 7.146 7.127 7.108 6.902 6.882 6.770 6.765 6.749 6.730 6.711 6.667 6.647 6.550 6.529 6.382 6.363 5.663
3.198 2.953
Parameter Value
1 Title LWF-10-211-H
2 Origin Bruker BioSpin GmbH
3 Solvent CDCl3
4 Temperature 298.0
5 Number of Scans 5
6 Acquisition Time 4.0894
7 Acquisition Date 2024-10-01T17:47:39
8 Spectrometer Frequency 400.13
9 Spectral Width 8012.8
Me
Me
N
Ms
N
N
H
SO2Ph
D4
f1 (ppm)
1.00 1.00 2.00 1.00 2.00 3.00 1.00 1.00 1.00 2.00 2.00 1.00 1.00 1.00 3.00 6.00

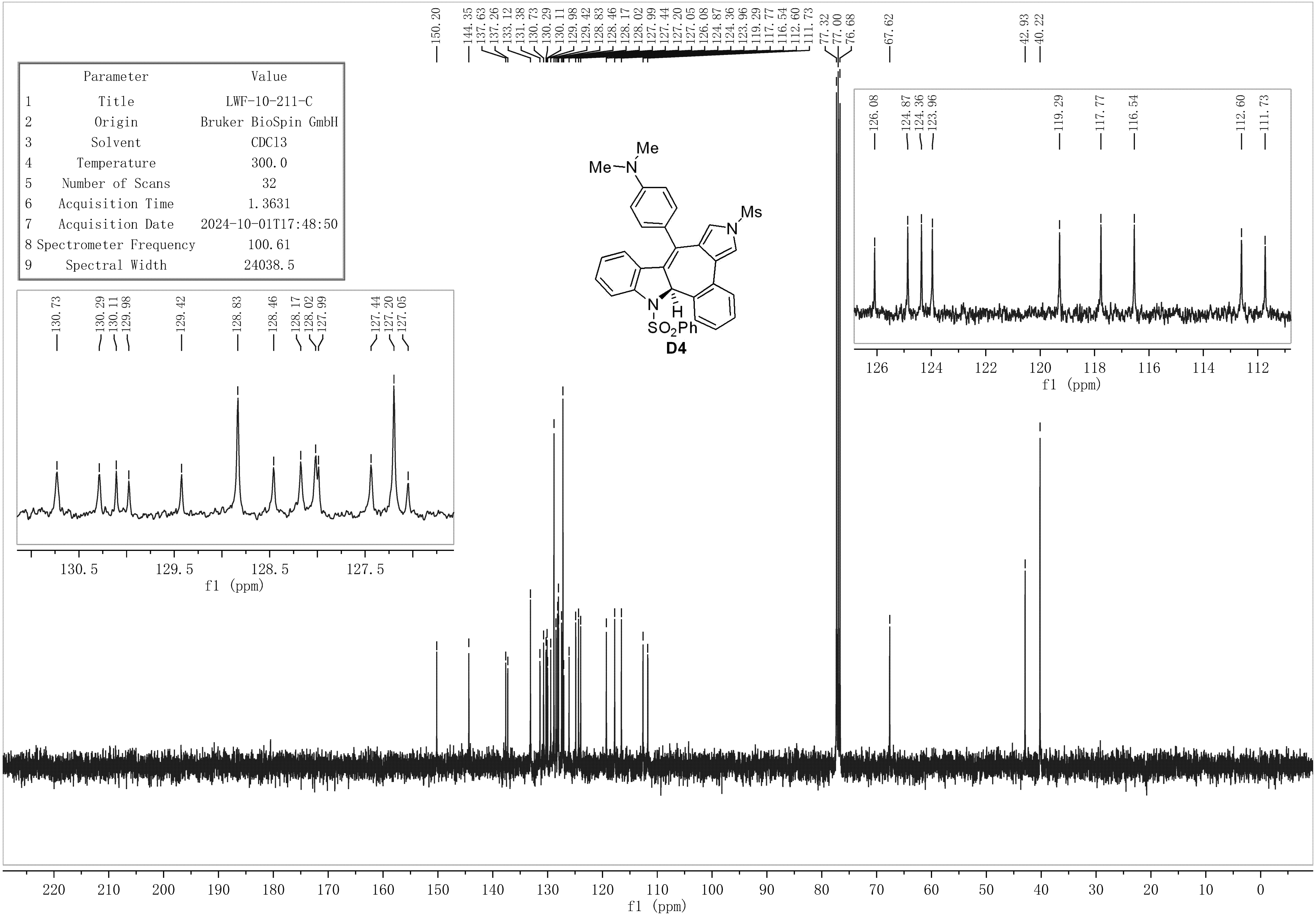
Parameter Value
1 Title LWF-10-211-C
2 Origin Bruker BioSpin GmbH
3 Solvent CDCl3
4 Temperature 300.0
5 Number of Scans 32
6 Acquisition Time 1.3631
7 Acquisition Date 2024-10-01T17:48:50
8 Spectrometer Frequency 100.61
9 Spectral Width 24038.5
150.20 144.35 137.63 137.26 133.12 131.38 130.73 130.29 130.11 129.98 129.42 128.83 128.46 128.17 128.02 127.99 127.44 127.20 127.05 126.08 124.87 124.36 123.96 119.29 117.77 116.54 112.60 111.73 77.32 77.00 76.68 67.62 42.93 40.22
Me
Me
N
Ms
N
N
H
SO2Ph
D4
130.73 130.29 130.11 129.98 129.42 128.83 128.46 128.17 128.02 127.99 127.44 127.20 127.05
130.5 129.5 128.5 127.5
f1 (ppm)
126.08 124.87 124.36 123.96 119.29 117.77 116.54 112.60 111.73
126 124 122 120 118 116 114 112
f1 (ppm)
220 210 200 190 180 170 160 150 140 130 120 110 100 90 80 70 60 50 40 30 20 10 0
f1 (ppm)

	Parameter	Value
1	Title	LWF-5-133-H
2	Origin	Bruker BioSpin GmbH
3	Solvent	CDCl3
4	Temperature	298.0
5	Number of Scans	6
6	Acquisition Time	4.0894
7	Acquisition Date	2022-12-21T17:04:58
8	Spectrometer Frequency	400.13
9	Spectral Width	8012.8

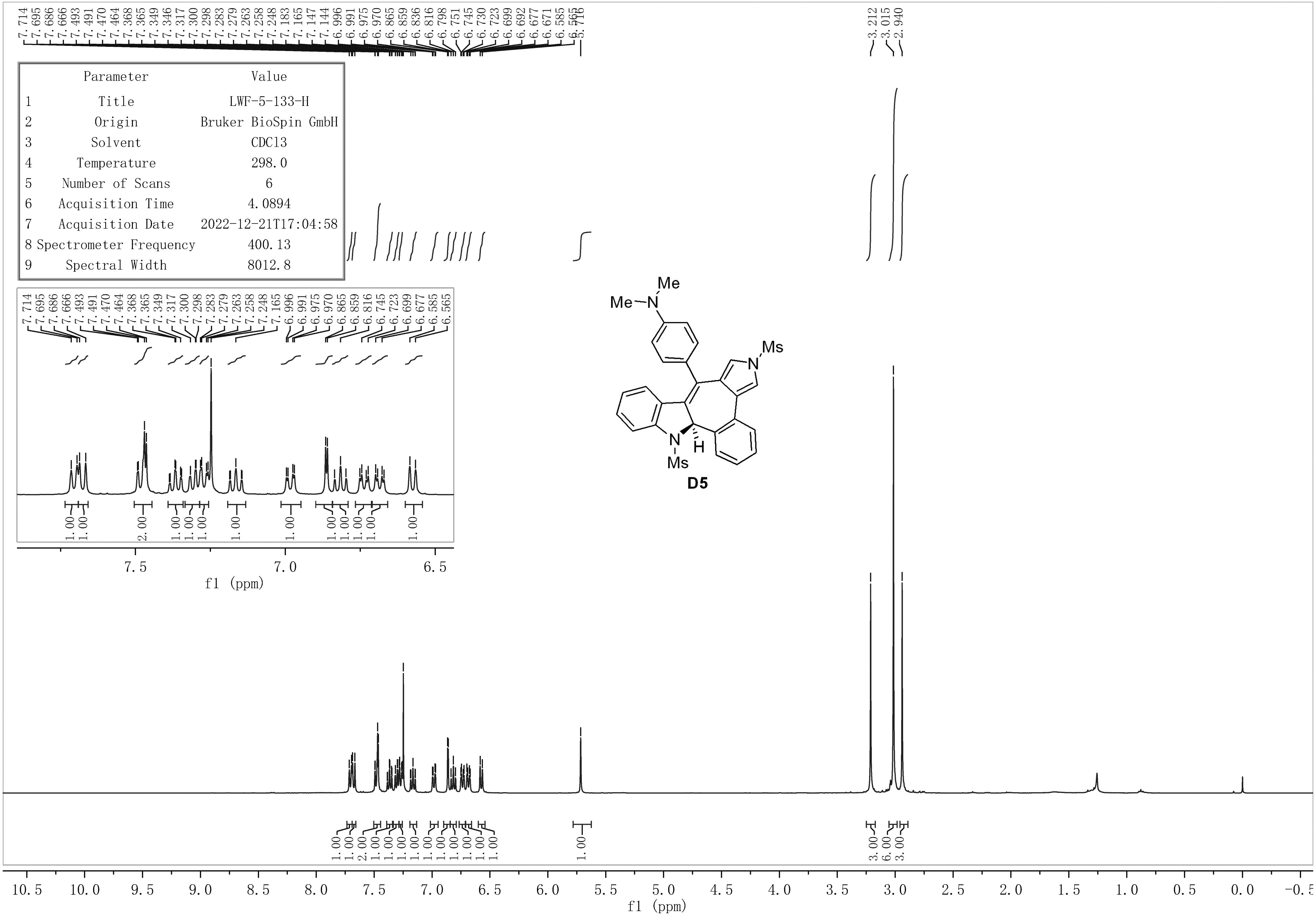

D5

	Parameter	Value
1	Title	LWF-5-133-C
2	Origin	Bruker BioSpin GmbH
3	Solvent	CDCl3
4	Temperature	300.0
5	Number of Scans	70
6	Acquisition Time	1.3631
7	Acquisition Date	2022-12-21T17:06:20
8	Spectrometer Frequency	100.61
9	Spectral Width	24038.5

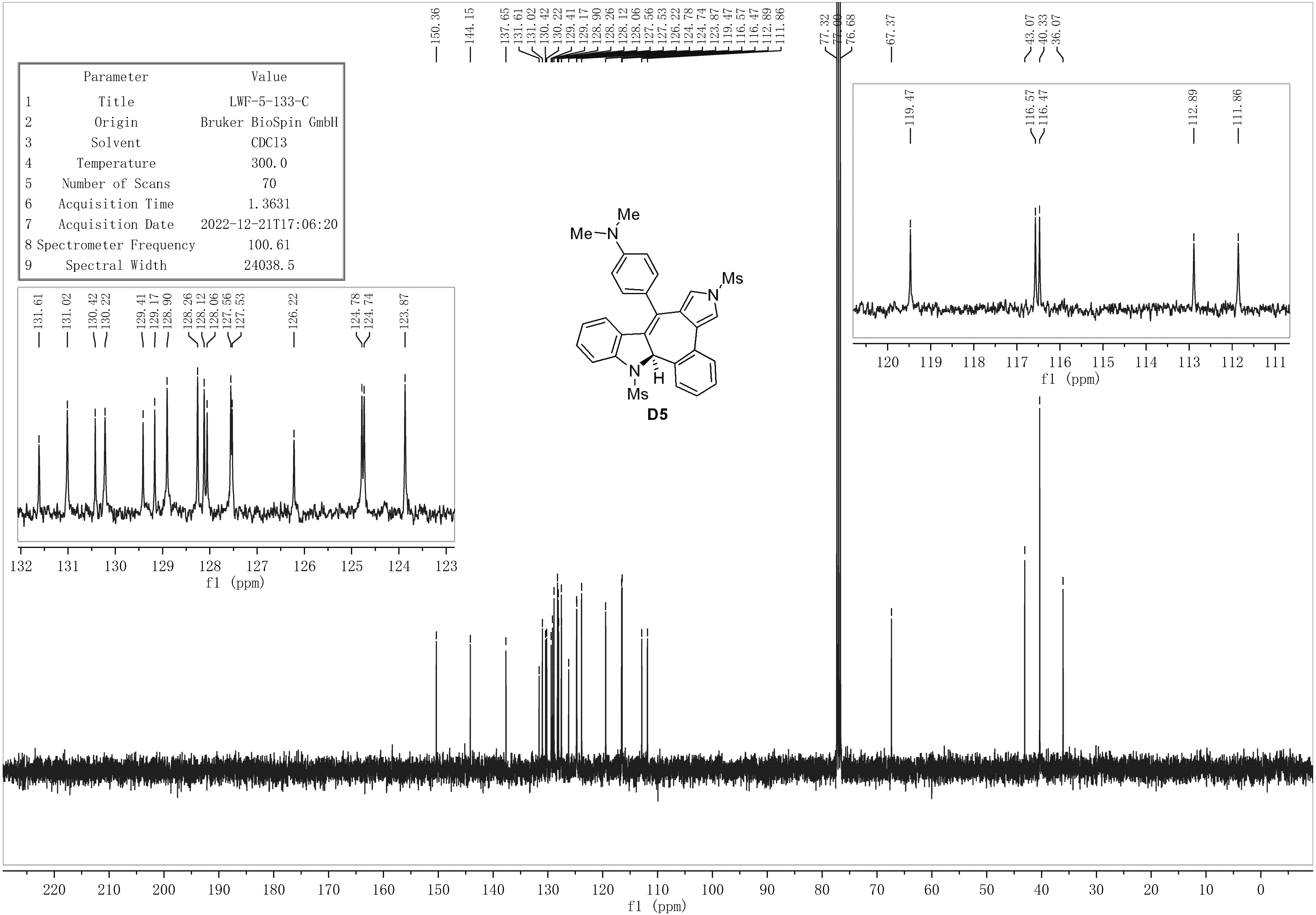

	Parameter	Value
1	Title	LWF-5-134-H
2	Origin	Bruker BioSpin GmbH
3	Solvent	CDCl3
4	Temperature	298.0
5	Number of Scans	6
6	Acquisition Time	4.0894
7	Acquisition Date	2022-12-20T13:52:11
8	Spectrometer Frequency	400.13
9	Spectral Width	8012.8

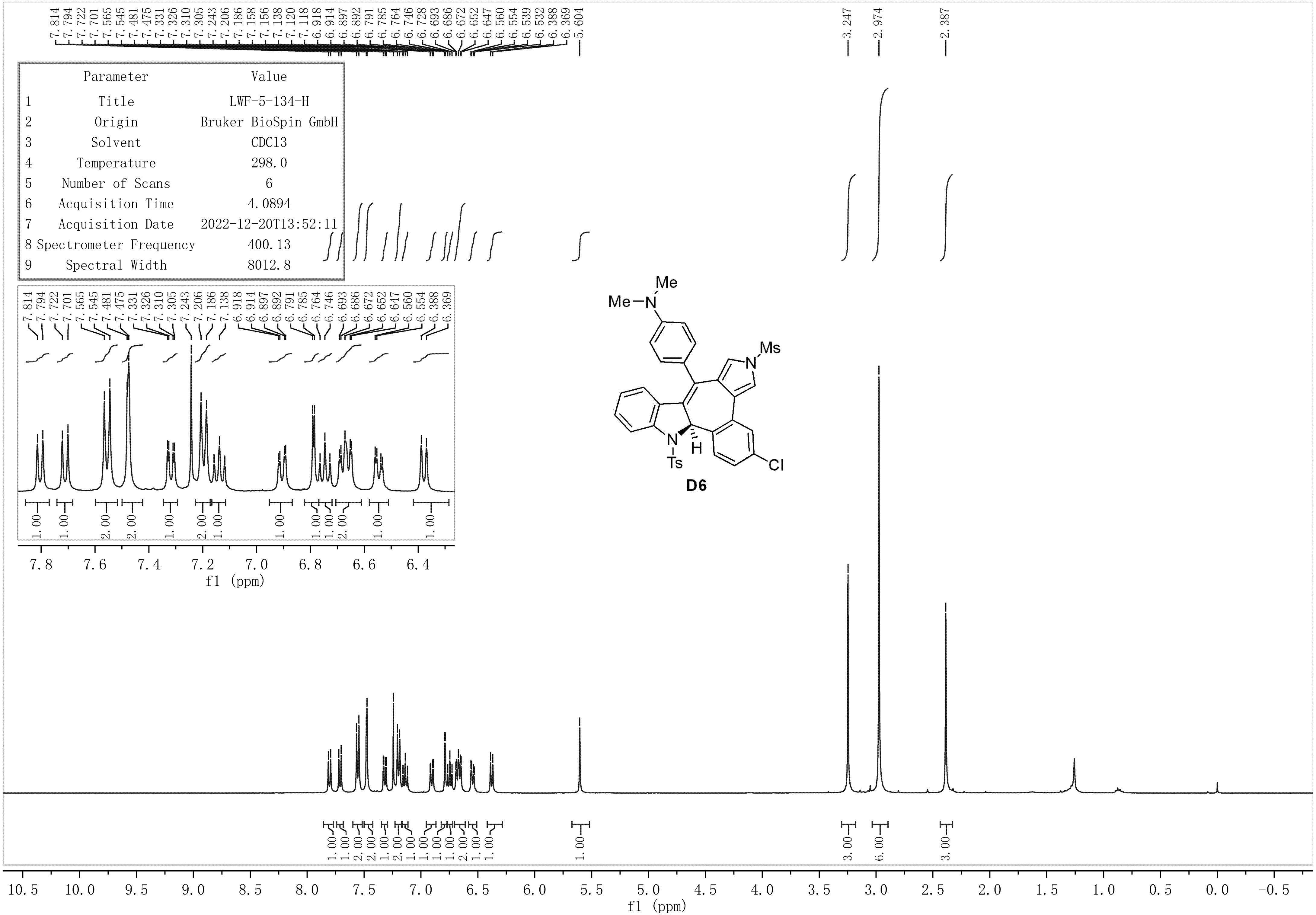

D6

	Parameter	Value
1	Title	LWF-5-134-C
2	Origin	Bruker BioSpin GmbH
3	Solvent	CDCl3
4	Temperature	300.0
5	Number of Scans	31
6	Acquisition Time	1.3631
7	Acquisition Date	2022-12-20T13:55:04
8	Spectrometer Frequency	100.61
9	Spectral Width	24038.5

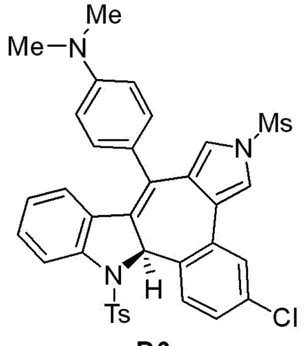

D6

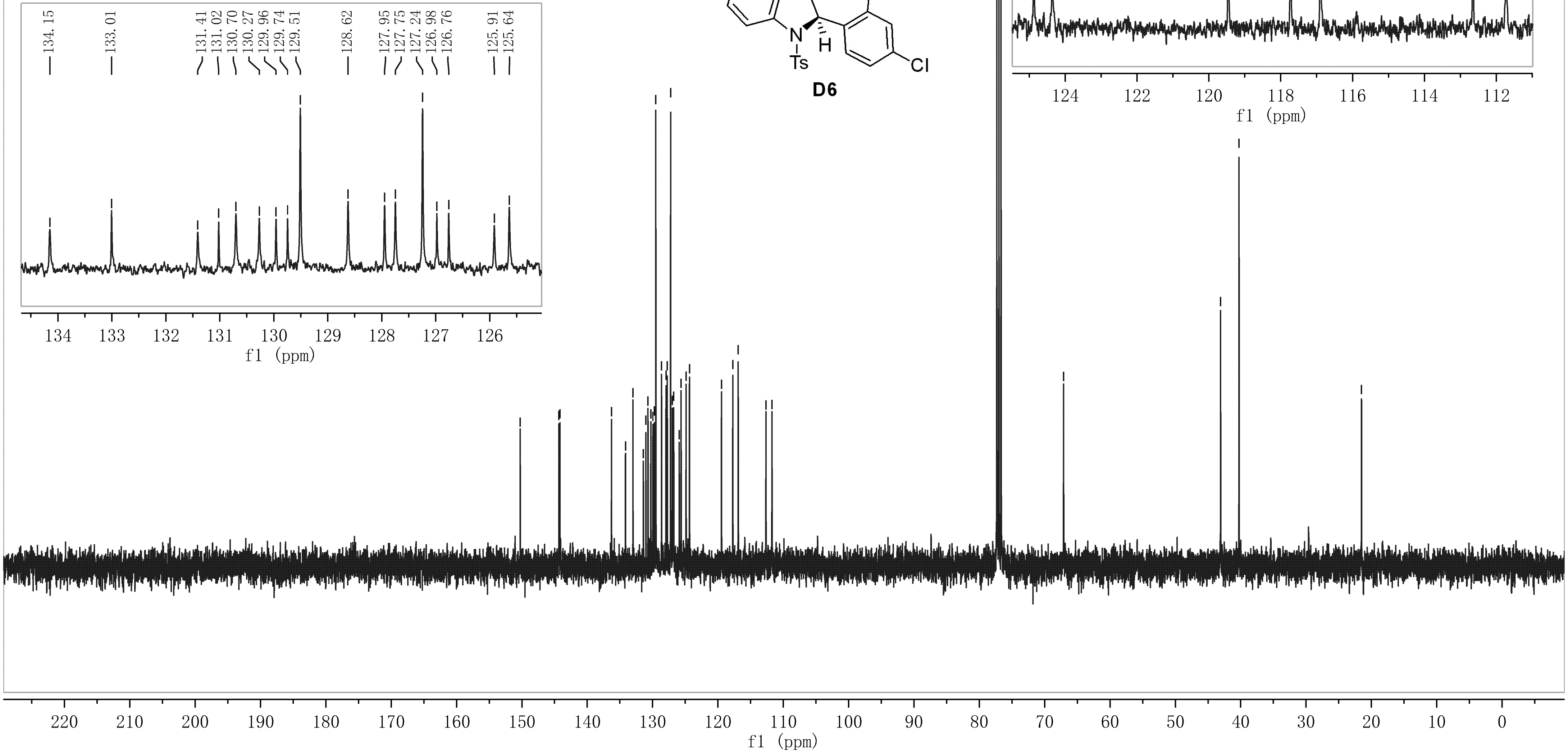

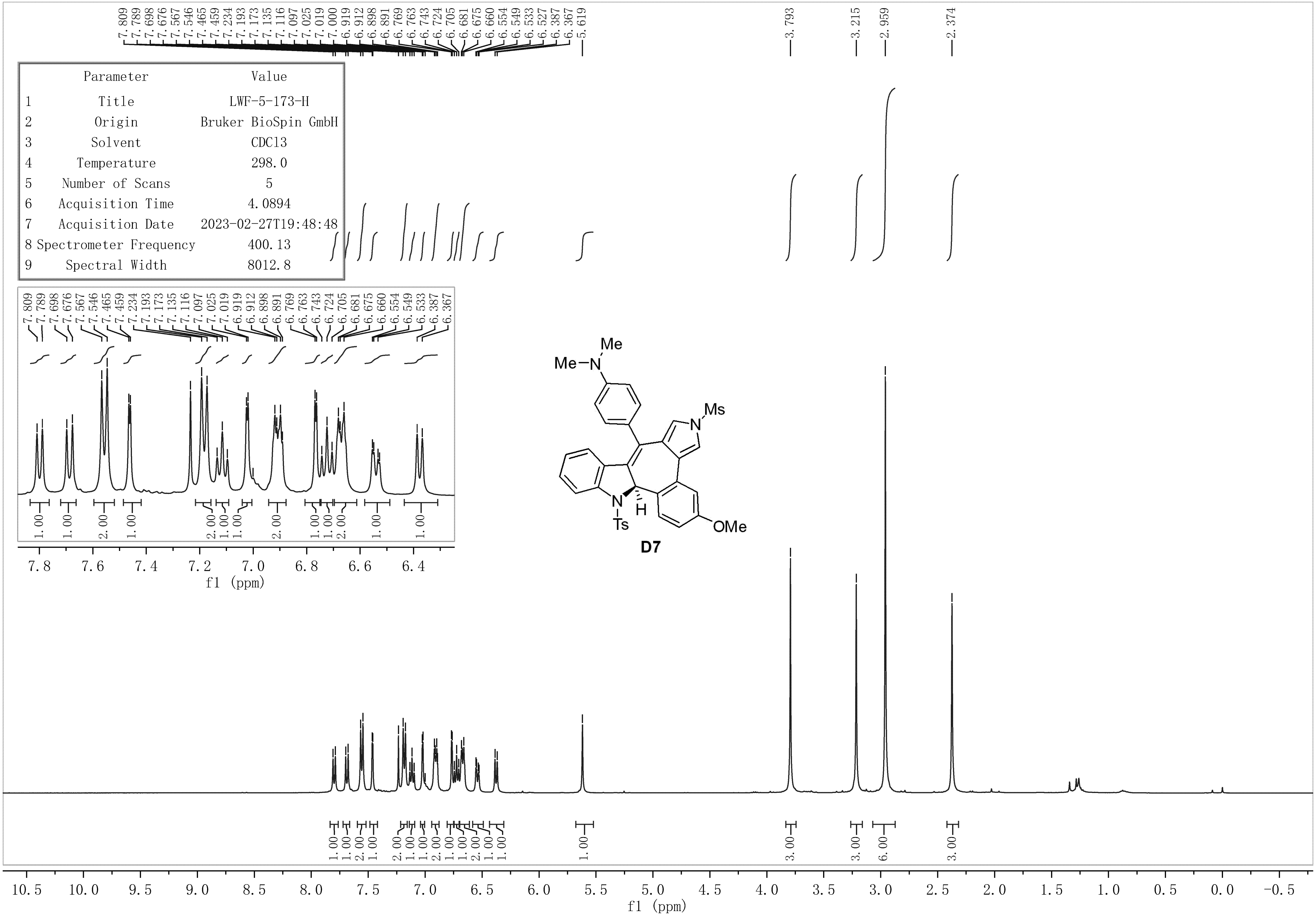

Parameter Value
1 Title LWF-5-173-H
2 Origin Bruker BioSpin GmbH
3 Solvent CDCl3
4 Temperature 298.0
5 Number of Scans 5
6 Acquisition Time 4.0894
7 Acquisition Date 2023-02-27T19:48:48
8 Spectrometer Frequency 400.13
9 Spectral Width 8012.8
Me
Me−N
Ms
N
N
H
Ts
OMe
D7
7.809 7.789 7.698 7.676 7.567 7.546 7.465 7.459 7.234 7.193 7.173 7.135 7.116 7.097 7.025 7.019 7.000 6.919 6.912 6.898 6.891 6.769 6.763 6.743 6.724 6.705 6.681 6.675 6.660 6.554 6.549 6.533 6.527 6.387 6.367 5.619
3.793
3.215
2.959
2.374
f1 (ppm)

	Parameter	Value
1	Title	LWF-5-173-C
2	Origin	Bruker BioSpin GmbH
3	Solvent	CDCl3
4	Temperature	300.0
5	Number of Scans	20
6	Acquisition Time	1.3631
7	Acquisition Date	2023-02-27T19:52:57
8	Spectrometer Frequency	100.61
9	Spectral Width	24038.5

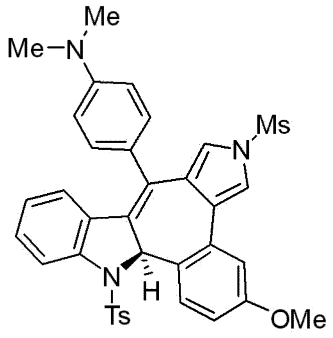

D7

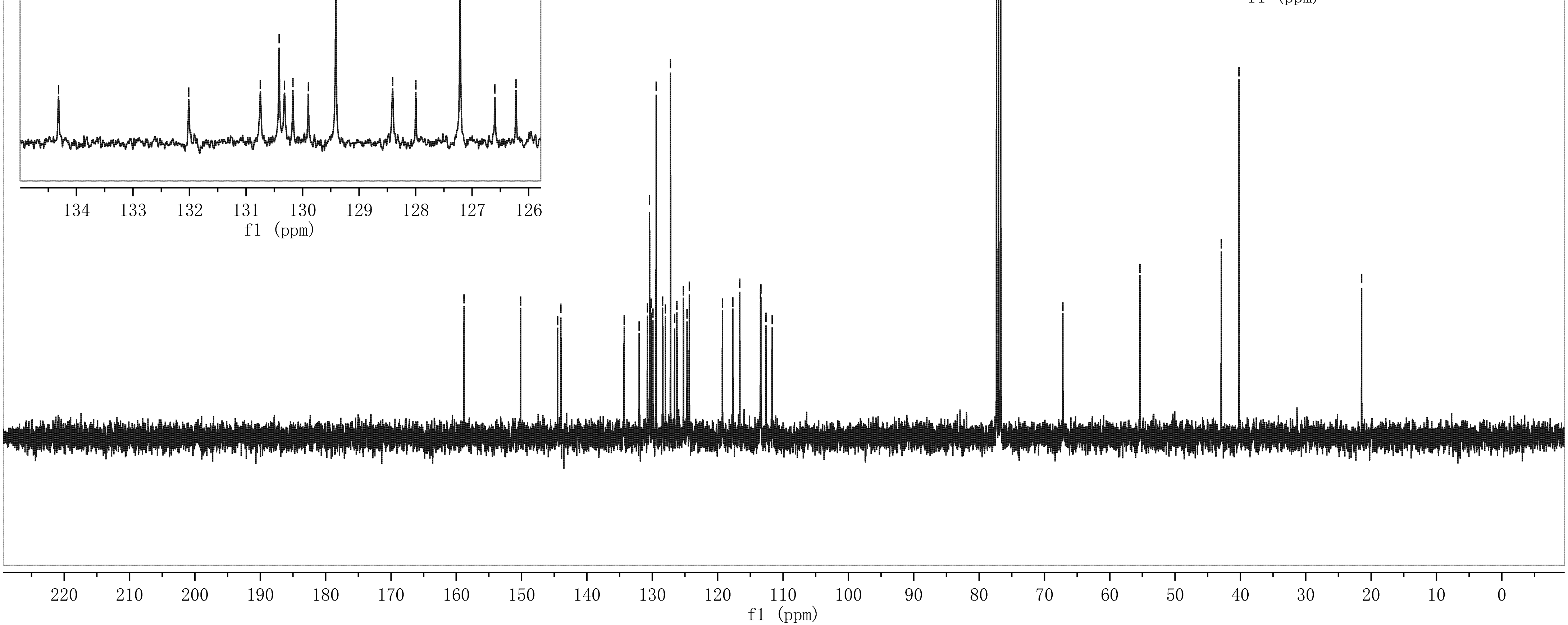

	Parameter	Value
1	Title	LWF-5-174-H
2	Origin	Bruker BioSpin GmbH
3	Solvent	CDCl3
4	Temperature	298.0
5	Number of Scans	6
6	Acquisition Time	4.0894
7	Acquisition Date	2023-02-27T19:56:28
8	Spectrometer Frequency	400.13
9	Spectral Width	8012.8

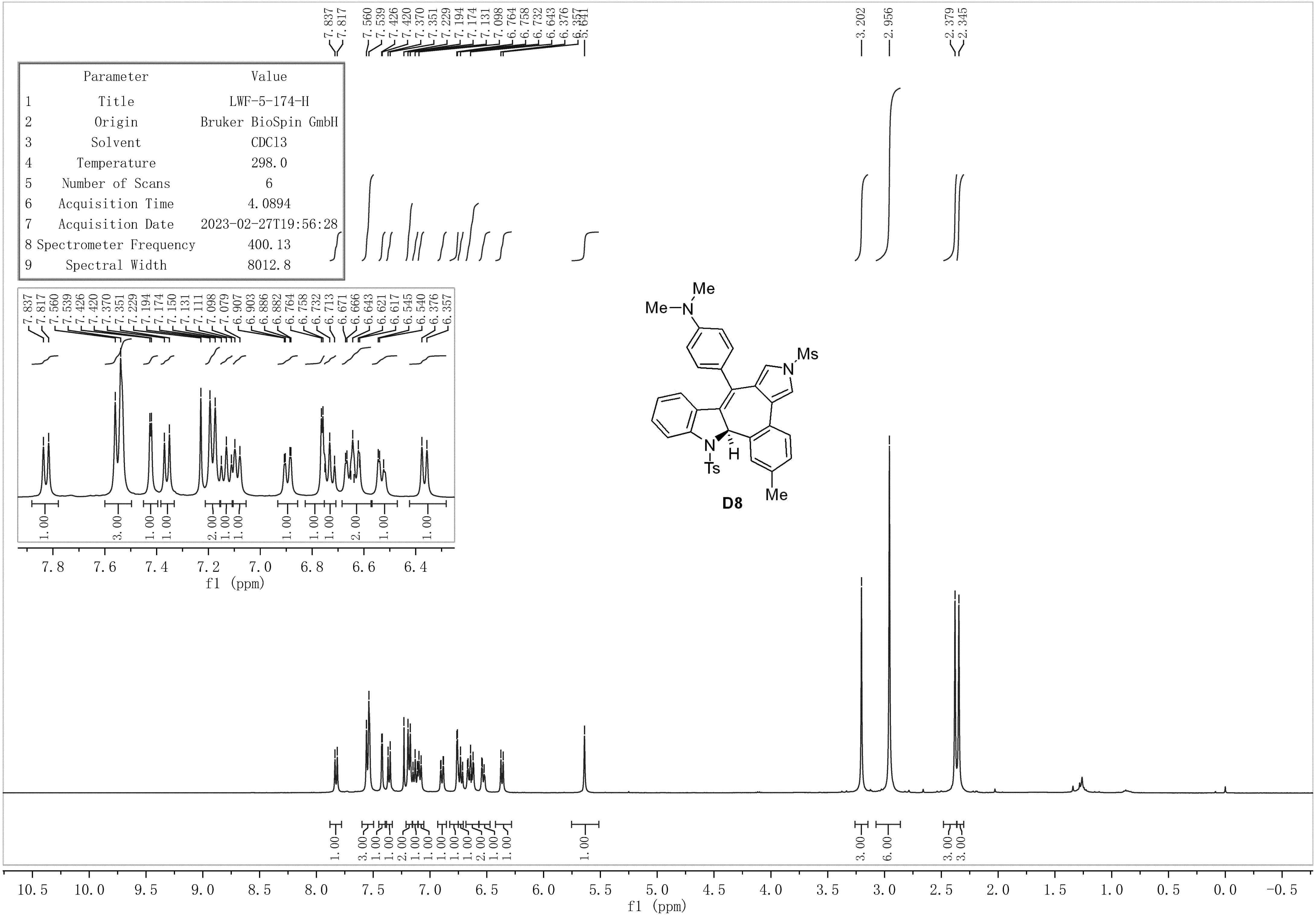

D8

	Parameter	Value
1	Title	LWF-5-174-C
2	Origin	Bruker BioSpin GmbH
3	Solvent	CDCl3
4	Temperature	300.0
5	Number of Scans	23
6	Acquisition Time	1.3631
7	Acquisition Date	2023-02-27T19:57:39
8	Spectrometer Frequency	100.61
9	Spectral Width	24038.5

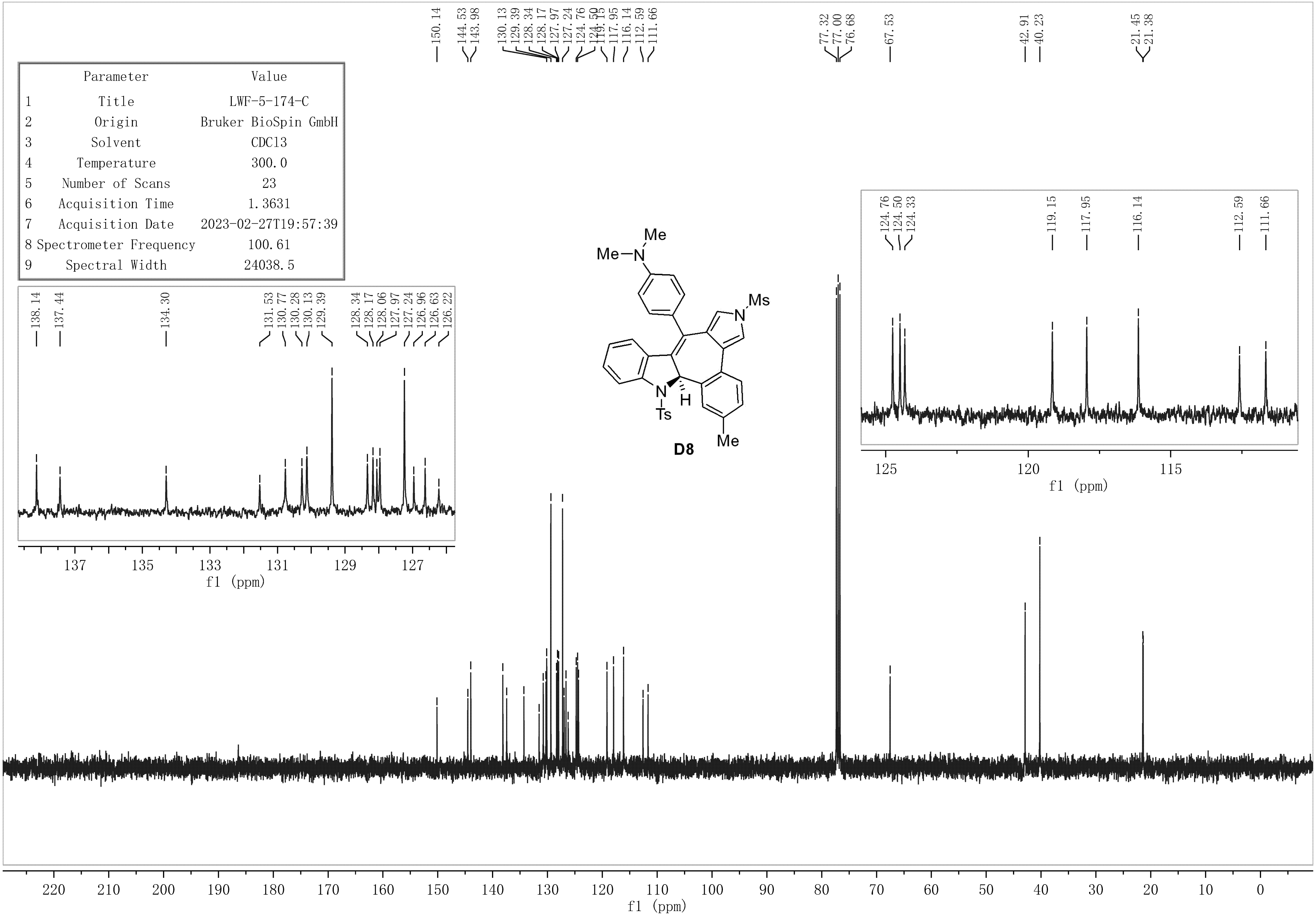

	Parameter	Value
1	Title	LWF-5-128-H
2	Origin	Bruker BioSpin GmbH
3	Solvent	CDCl3
4	Temperature	298.0
5	Number of Scans	7
6	Acquisition Time	4.0894
7	Acquisition Date	2022-12-18T17:52:39
8	Spectrometer Frequency	400.13
9	Spectral Width	8012.8

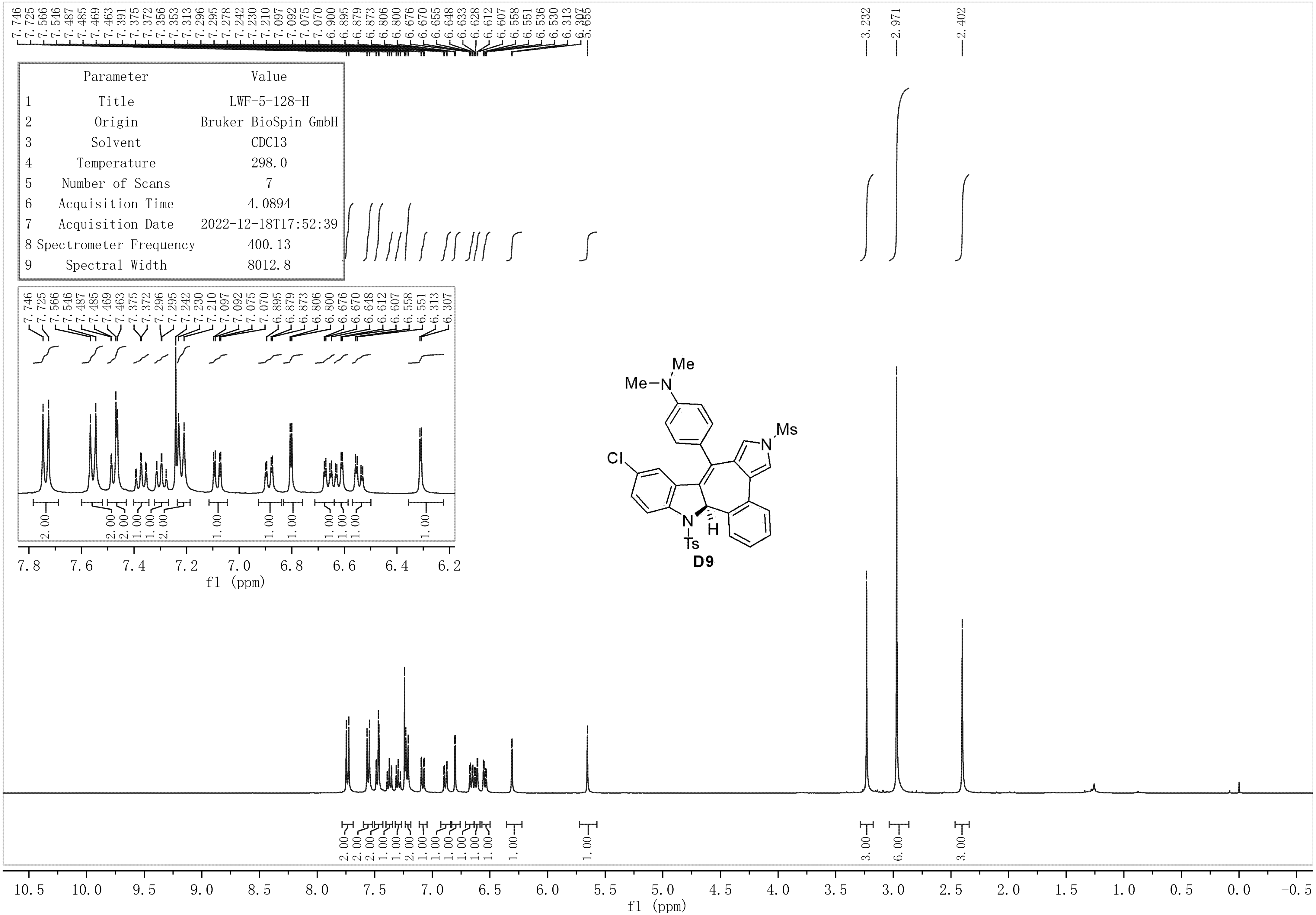

D9

	Parameter	Value
1	Title	LWF-5-128-C
2	Origin	Bruker BioSpin GmbH
3	Solvent	CDCl3
4	Temperature	300.0
5	Number of Scans	35
6	Acquisition Time	1.3631
7	Acquisition Date	2022-12-18T17:54:13
8	Spectrometer Frequency	100.61
9	Spectral Width	24038.5

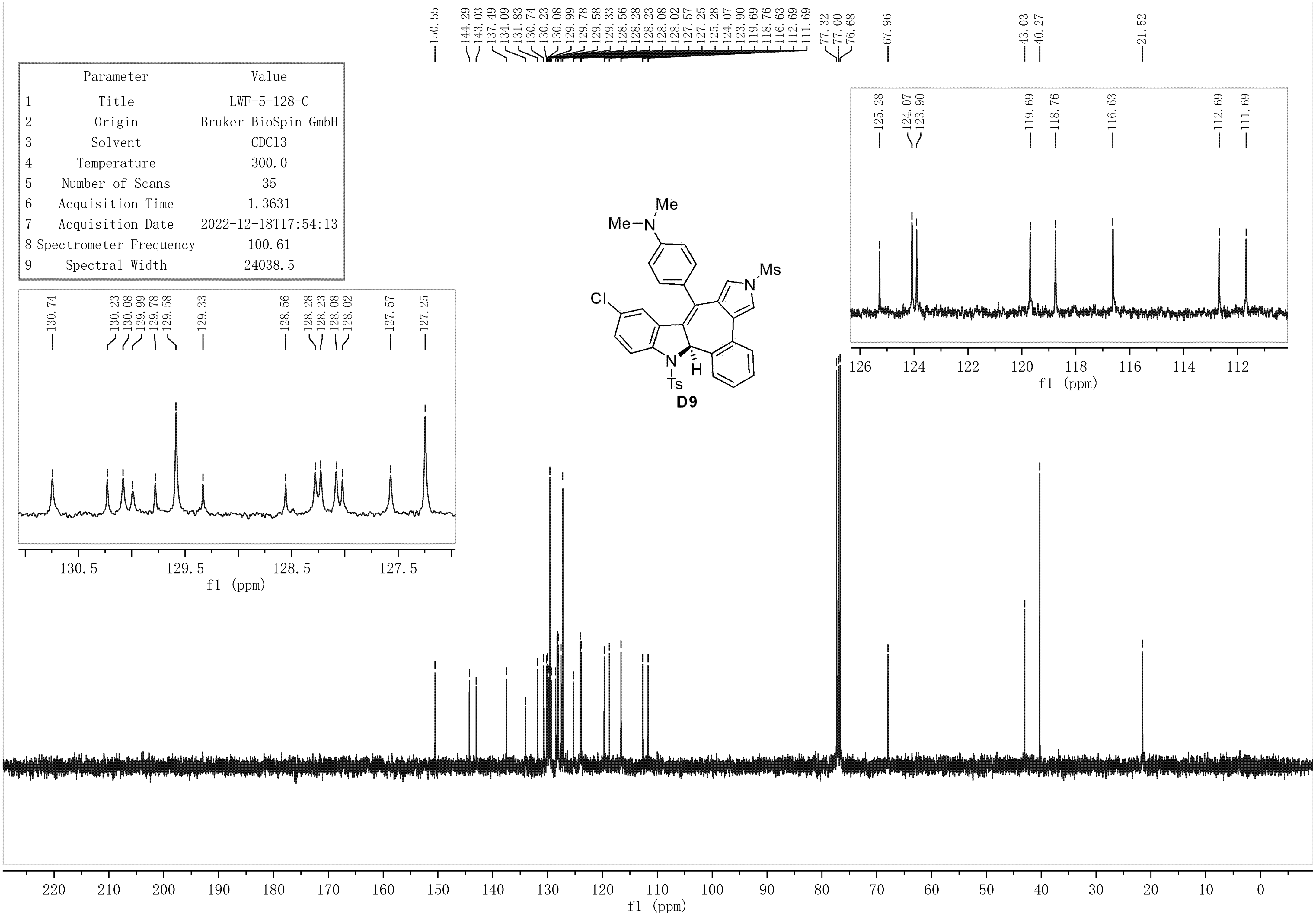

D9

	Parameter	Value
1	Title	LWF-11-42-H
2	Origin	Bruker BioSpin GmbH
3	Solvent	CDCl3
4	Temperature	298.0
5	Number of Scans	7
6	Acquisition Time	4.0894
7	Acquisition Date	2024-11-06T17:31:56
8	Spectrometer Frequency	400.13
9	Spectral Width	8012.8

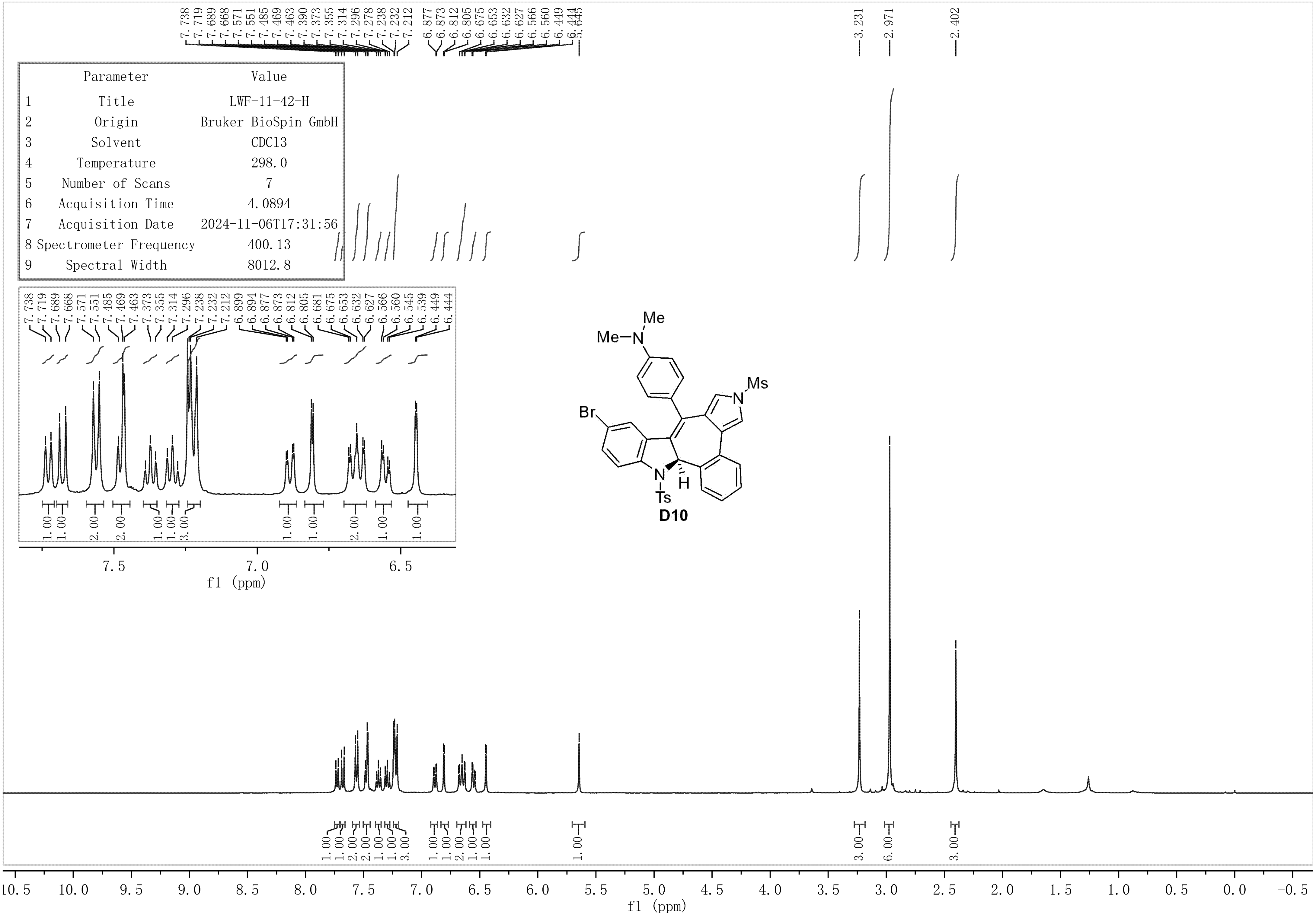

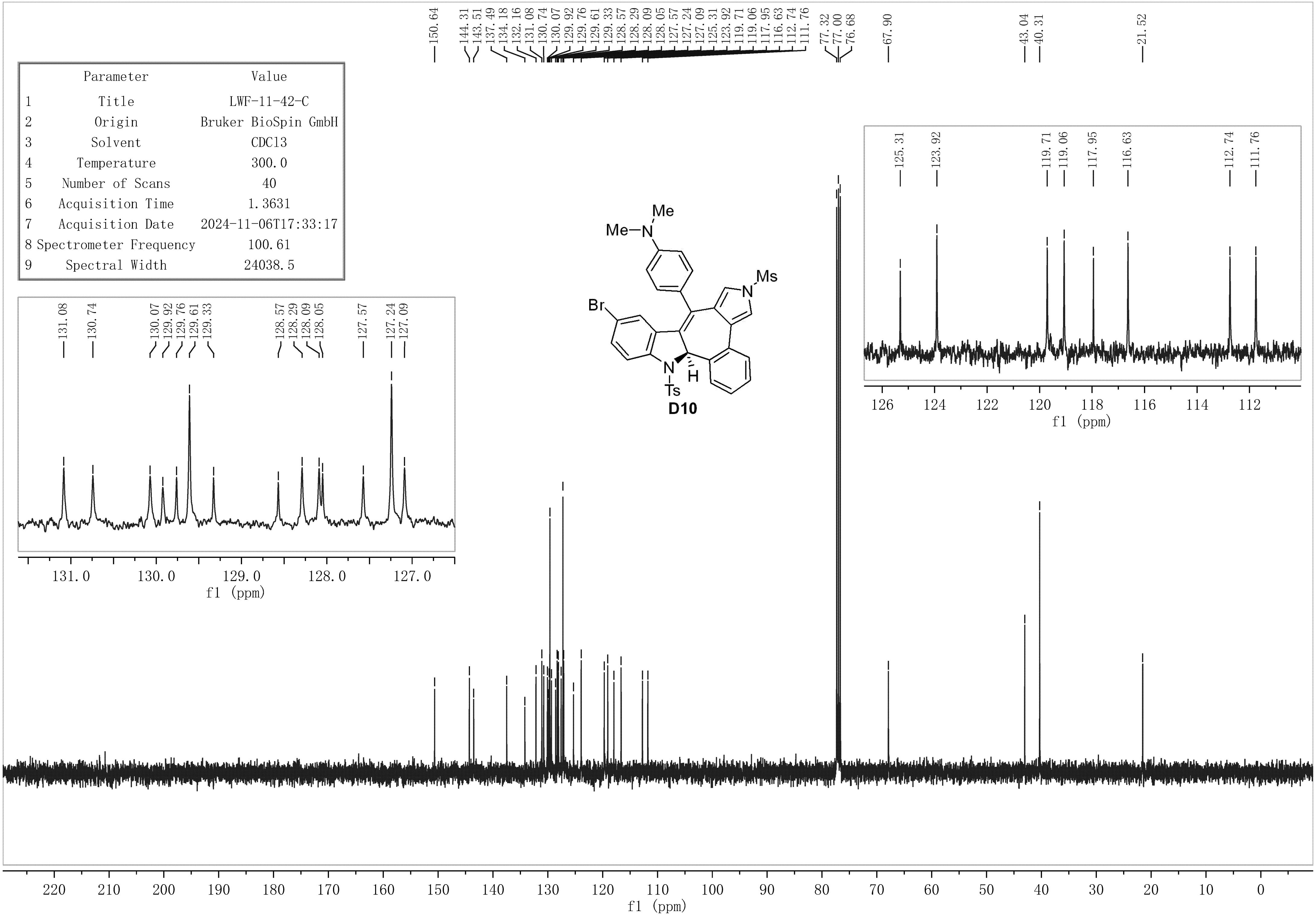

Parameter Value
1 Title LWF-11-42-C
2 Origin Bruker BioSpin GmbH
3 Solvent CDCl3
4 Temperature 300.0
5 Number of Scans 40
6 Acquisition Time 1.3631
7 Acquisition Date 2024-11-06T17:33:17
8 Spectrometer Frequency 100.61
9 Spectral Width 24038.5
150.64 144.31 143.51 137.49 134.18 132.16 131.08 130.74 130.07 129.92 129.76 129.61 129.33 128.57 128.29 128.09 128.05 127.57 127.24 127.09 125.31 123.92 119.71 119.06 117.95 116.63 112.74 111.76
77.32 77.00 76.68
67.90
43.04 40.31
21.52
Me
Me–N
Br
N
Ms
N
Ts
H
D10
f1 (ppm)

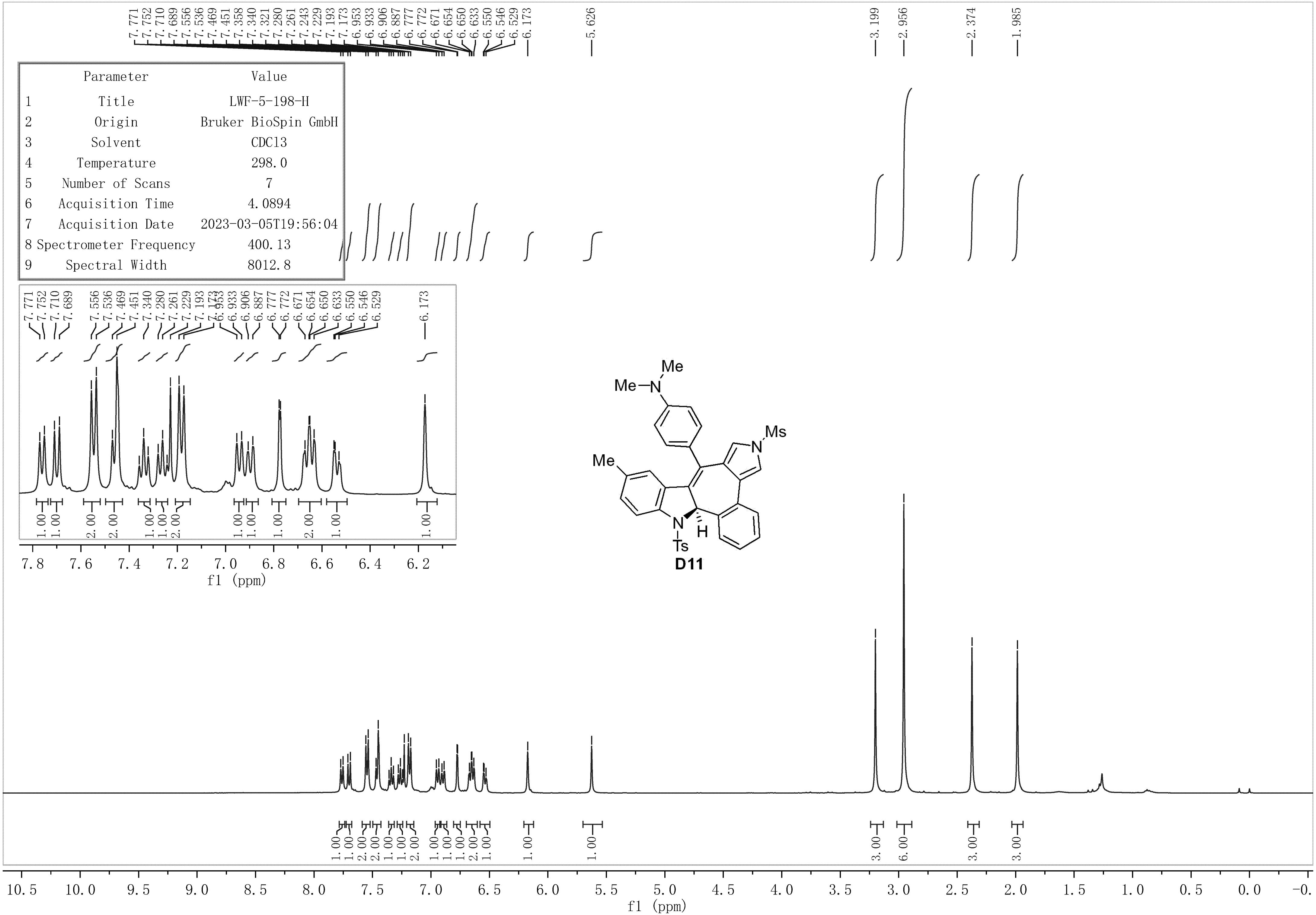

Parameter Value
1 Title LWF-5-198-H
2 Origin Bruker BioSpin GmbH
3 Solvent CDCl3
4 Temperature 298.0
5 Number of Scans 7
6 Acquisition Time 4.0894
7 Acquisition Date 2023-03-05T19:56:04
8 Spectrometer Frequency 400.13
9 Spectral Width 8012.8
7.771 7.752 7.710 7.689 7.556 7.536 7.469 7.451 7.358 7.340 7.321 7.280 7.261 7.243 7.229 7.193 7.173 6.953 6.933 6.906 6.887 6.777 6.772 6.671 6.654 6.650 6.633 6.550 6.546 6.529 6.173
5.626
3.199 2.956 2.374 1.985
1.00 1.00 2.00 2.00 1.00 1.00 2.00 1.00 1.00 1.00 2.00 1.00 1.00 1.00 3.00 6.00 3.00 3.00
Me
Me–N
Ms
N
Me
N
H
Ts
D11
f1 (ppm)

	Parameter	Value
1	Title	LWF-5-198-C
2	Origin	Bruker BioSpin GmbH
3	Solvent	CDCl3
4	Temperature	300.0
5	Number of Scans	52
6	Acquisition Time	1.3631
7	Acquisition Date	2023-03-05T19:59:07
8	Spectrometer Frequency	100.61
9	Spectral Width	24038.5

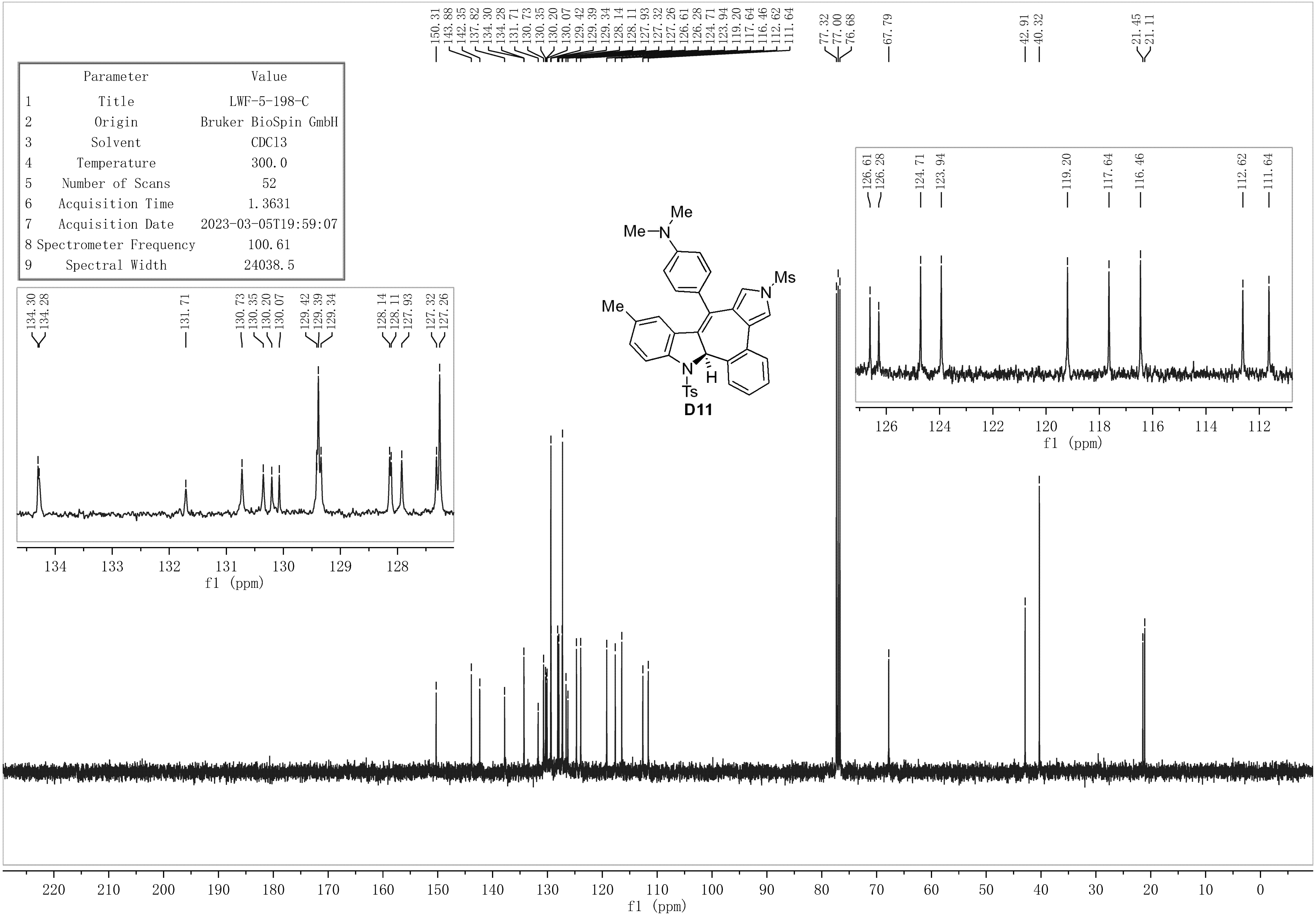

	Parameter	Value
1	Title	LWF-5-184-H
2	Origin	Bruker BioSpin GmbH
3	Solvent	CDCl3
4	Temperature	298.0
5	Number of Scans	8
6	Acquisition Time	4.0894
7	Acquisition Date	2023-03-05T19:41:12
8	Spectrometer Frequency	400.13
9	Spectral Width	8012.8

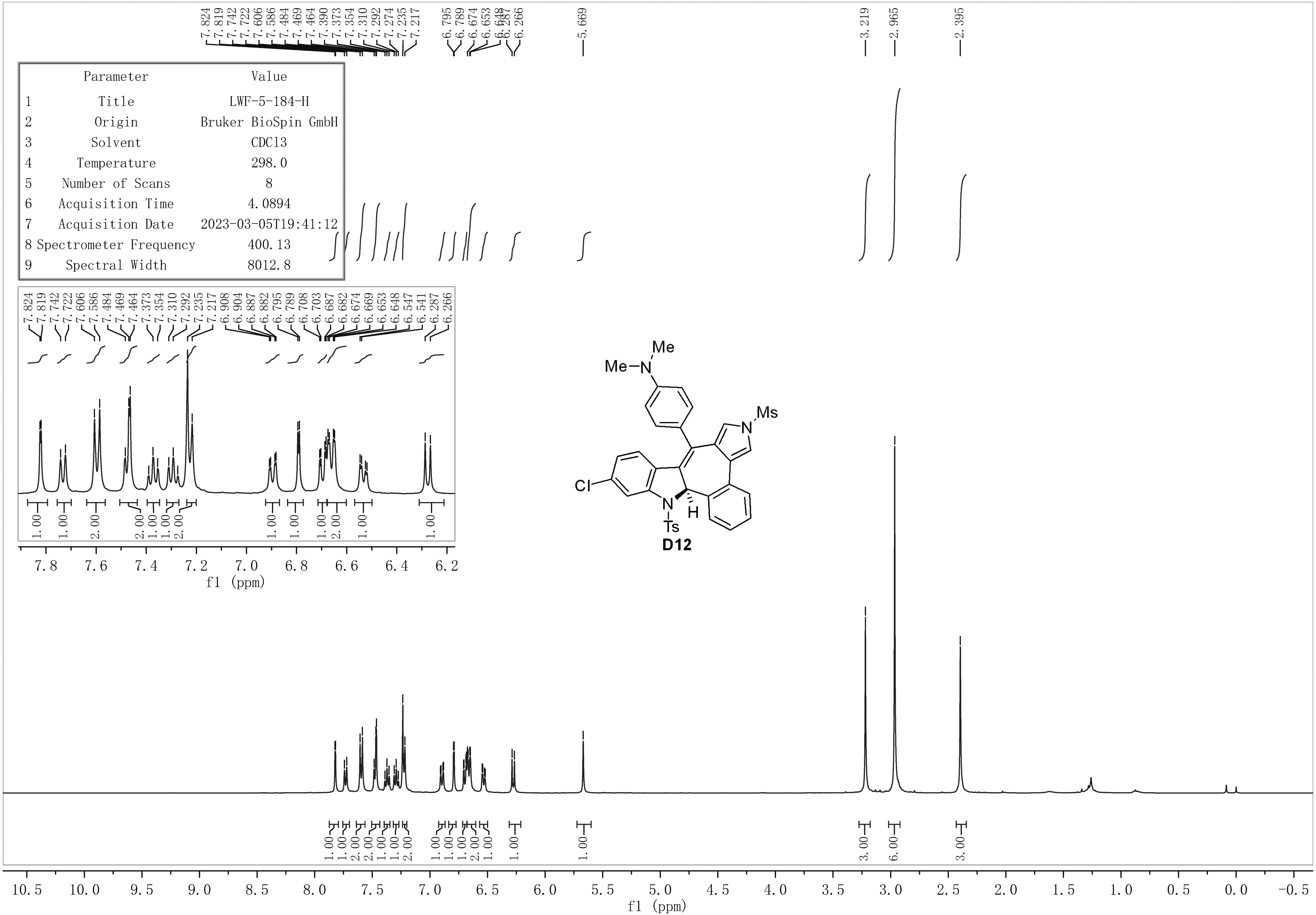

D12

	Parameter	Value
1	Title	LWF-5-184-C
2	Origin	Bruker BioSpin GmbH
3	Solvent	CDCl3
4	Temperature	300.0
5	Number of Scans	50
6	Acquisition Time	1.3631
7	Acquisition Date	2023-03-05T19:42:49
8	Spectrometer Frequency	100.61
9	Spectral Width	24038.5

D12

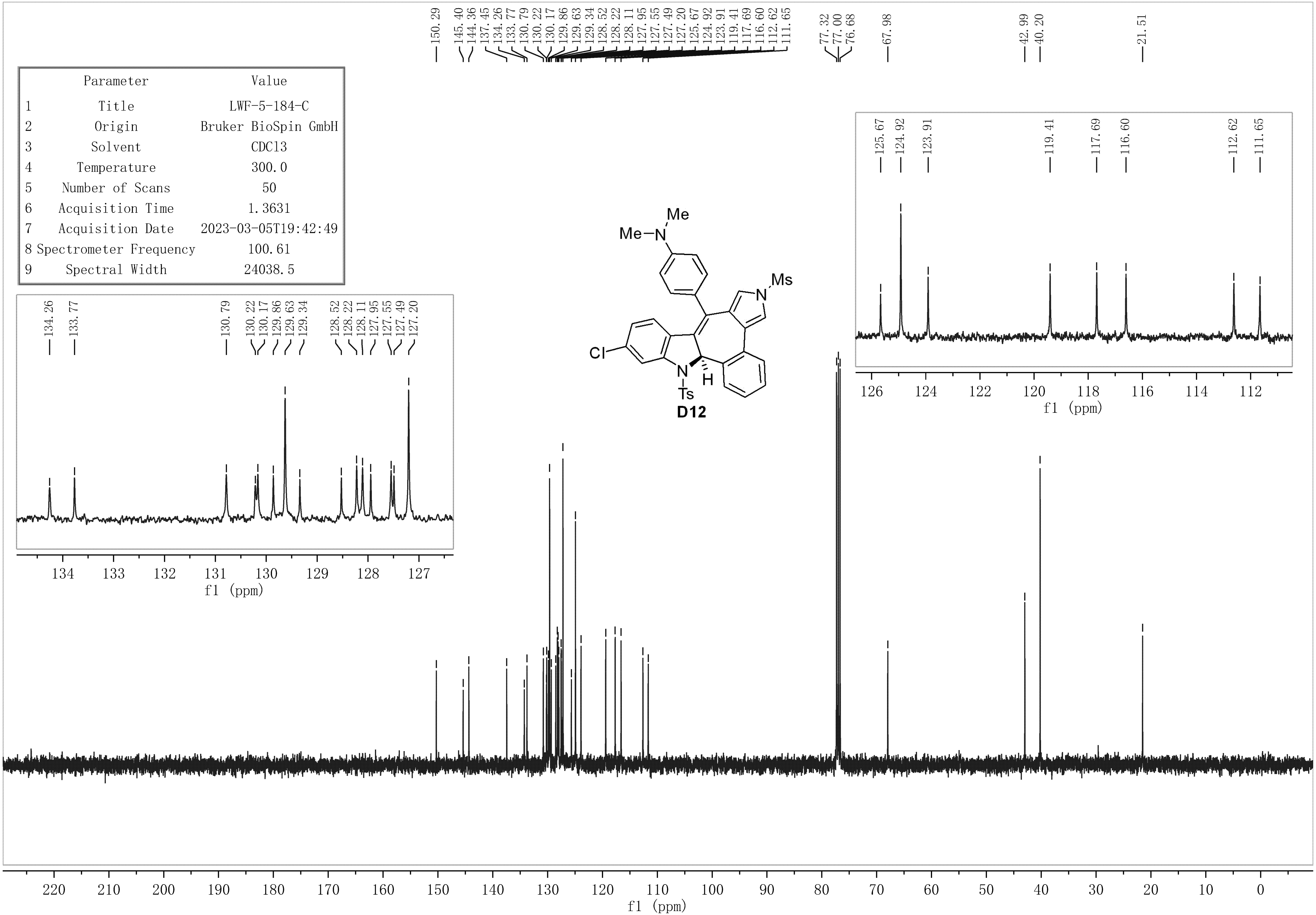

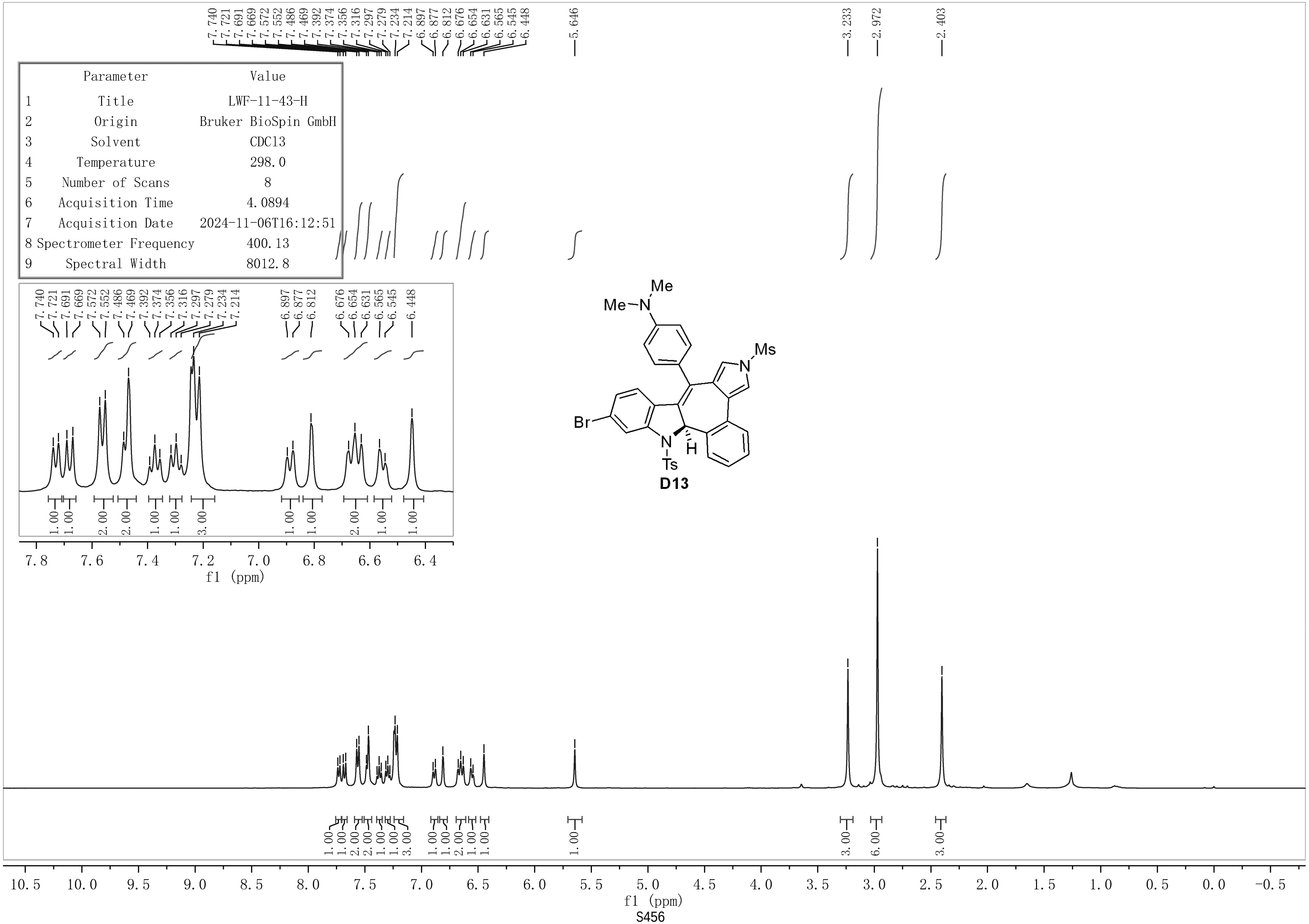

	Parameter	Value
1	Title	LWF-11-43-H
2	Origin	Bruker BioSpin GmbH
3	Solvent	CDCl3
4	Temperature	298.0
5	Number of Scans	8
6	Acquisition Time	4.0894
7	Acquisition Date	2024-11-06T16:12:51
8	Spectrometer Frequency	400.13
9	Spectral Width	8012.8

D13

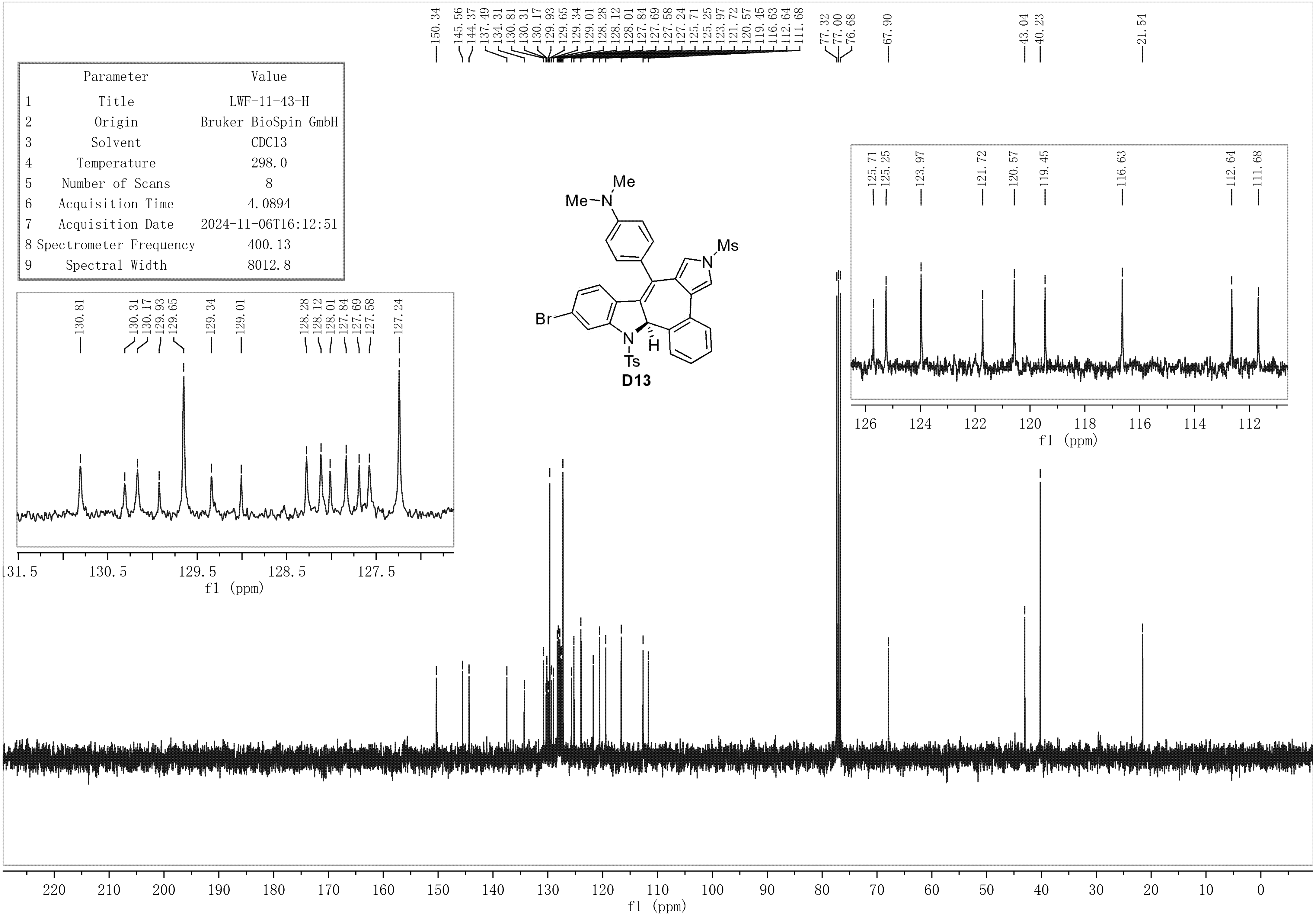

150.34
145.56
144.37
137.49
134.31
130.81
130.31
130.17
129.93
129.65
129.34
129.01
128.28
128.12
128.01
127.84
127.69
127.58
127.24
125.71
125.25
123.97
121.72
120.57
119.45
116.63
112.64
111.68
77.32
77.00
76.68
67.90
43.04
40.23
21.54
Parameter | Value
1 Title | LWF-11-43-H
2 Origin | Bruker BioSpin GmbH
3 Solvent | CDCl3
4 Temperature | 298.0
5 Number of Scans | 8
6 Acquisition Time | 4.0894
7 Acquisition Date | 2024-11-06T16:12:51
8 Spectrometer Frequency | 400.13
9 Spectral Width | 8012.8
Me
Me–N
Ms
N
Br
N
H
Ts
D13
f1 (ppm)

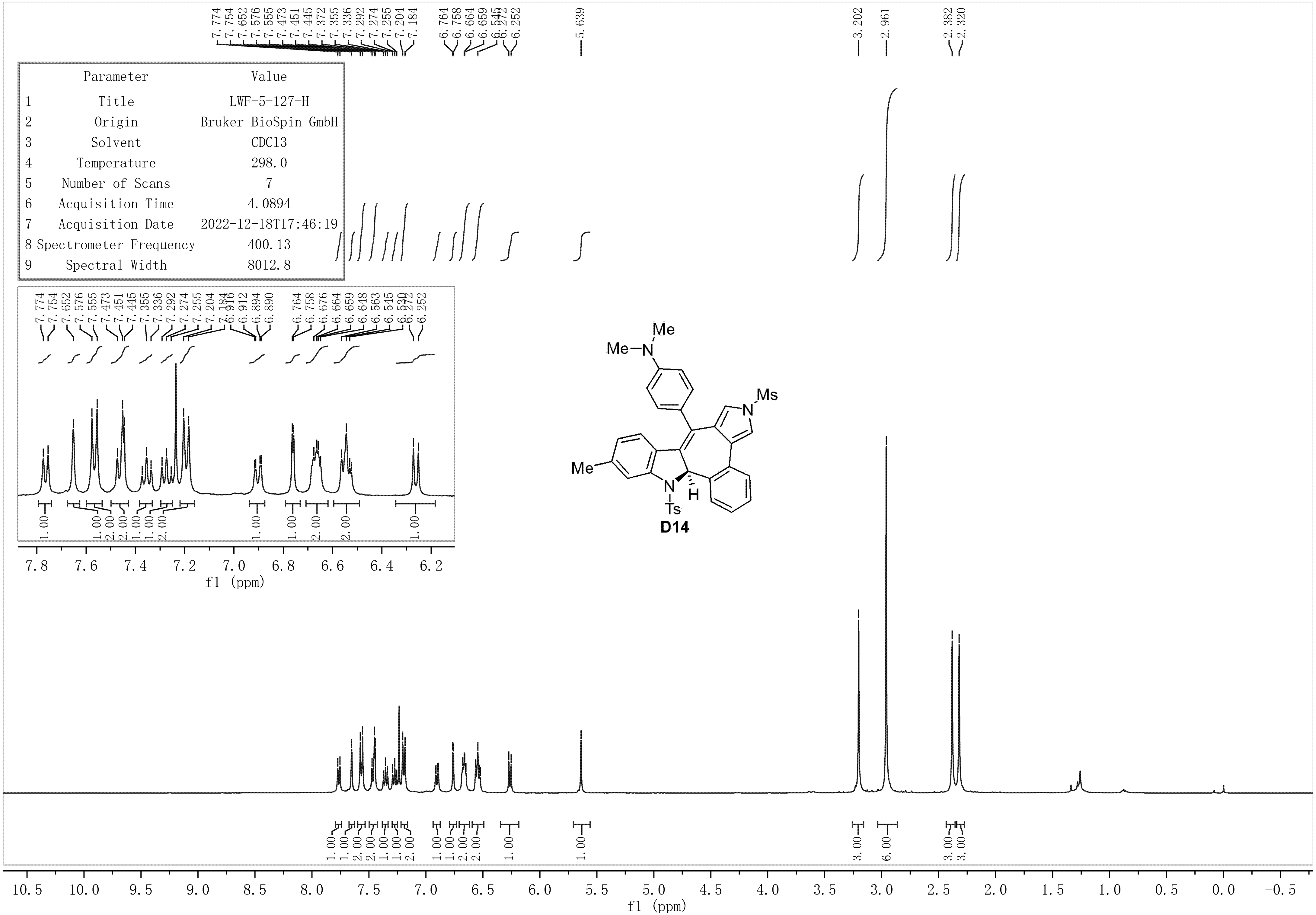

	Parameter	Value
1	Title	LWF-5-127-H
2	Origin	Bruker BioSpin GmbH
3	Solvent	CDCl3
4	Temperature	298.0
5	Number of Scans	7
6	Acquisition Time	4.0894
7	Acquisition Date	2022-12-18T17:46:19
8	Spectrometer Frequency	400.13
9	Spectral Width	8012.8

	Parameter	Value
1	Title	LWF-5-127-C
2	Origin	Bruker BioSpin GmbH
3	Solvent	CDCl3
4	Temperature	300.0
5	Number of Scans	35
6	Acquisition Time	1.3631
7	Acquisition Date	2022-12-18T17:47:28
8	Spectrometer Frequency	100.61
9	Spectral Width	24038.5

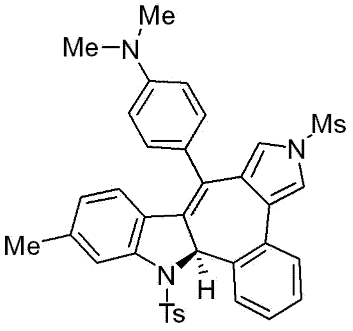

D14

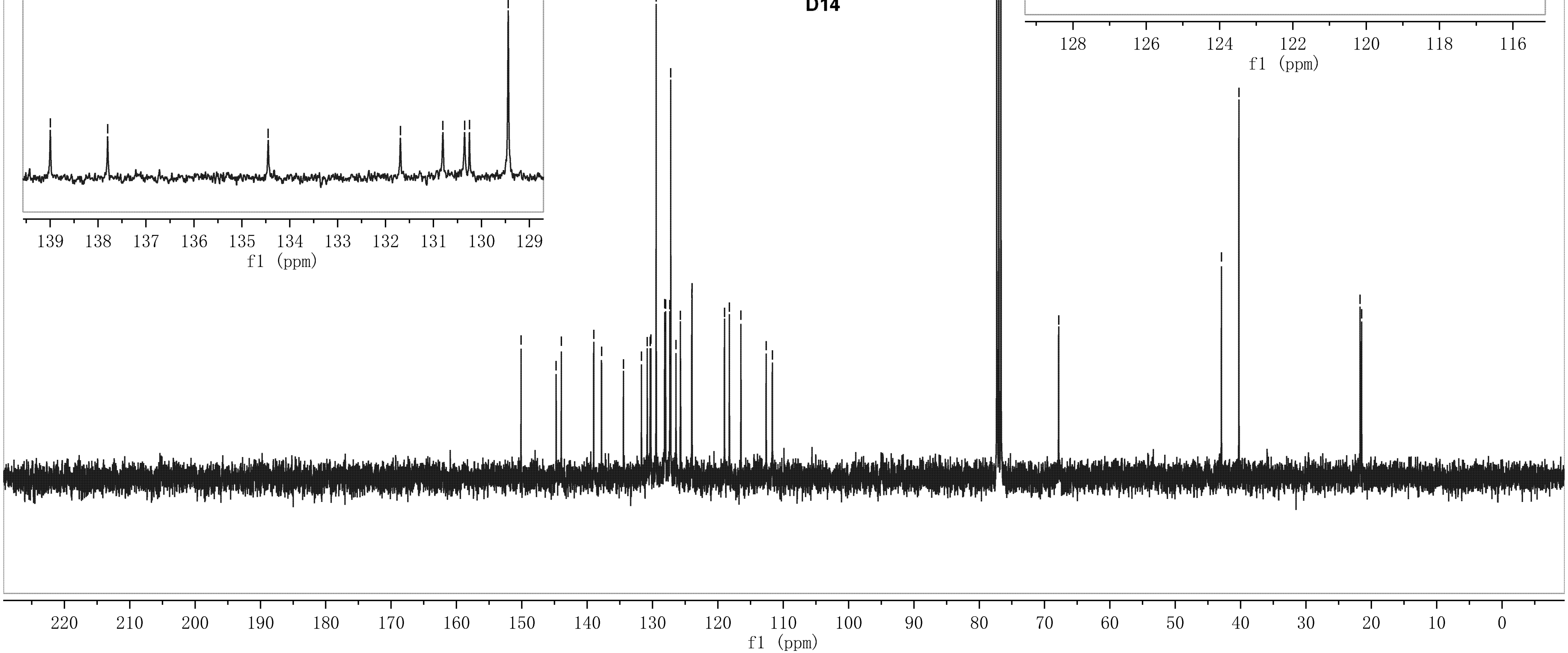

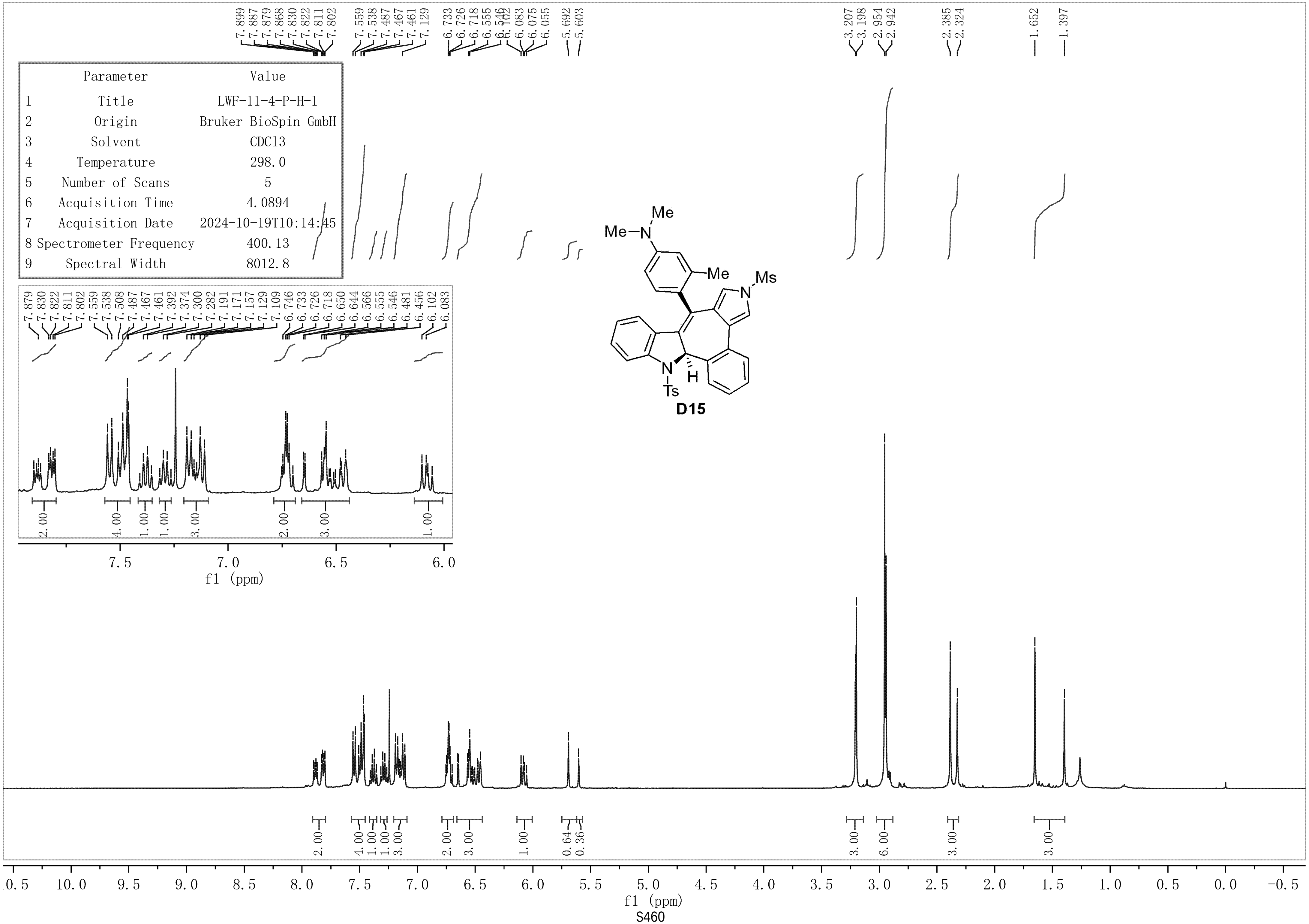

D15

	Parameter	Value
1	Title	LWF-11-4-P-H-1
2	Origin	Bruker BioSpin GmbH
3	Solvent	CDCl3
4	Temperature	298.0
5	Number of Scans	5
6	Acquisition Time	4.0894
7	Acquisition Date	2024-10-19T10:14:45
8	Spectrometer Frequency	400.13
9	Spectral Width	8012.8

	Parameter	Value
1	Title	LWF-11-4-P-C
2	Origin	Bruker BioSpin GmbH
3	Solvent	CDCl3
4	Temperature	300.0
5	Number of Scans	62
6	Acquisition Time	1.3631
7	Acquisition Date	2024-10-19T10:15:58
8	Spectrometer Frequency	100.61
9	Spectral Width	24038.5

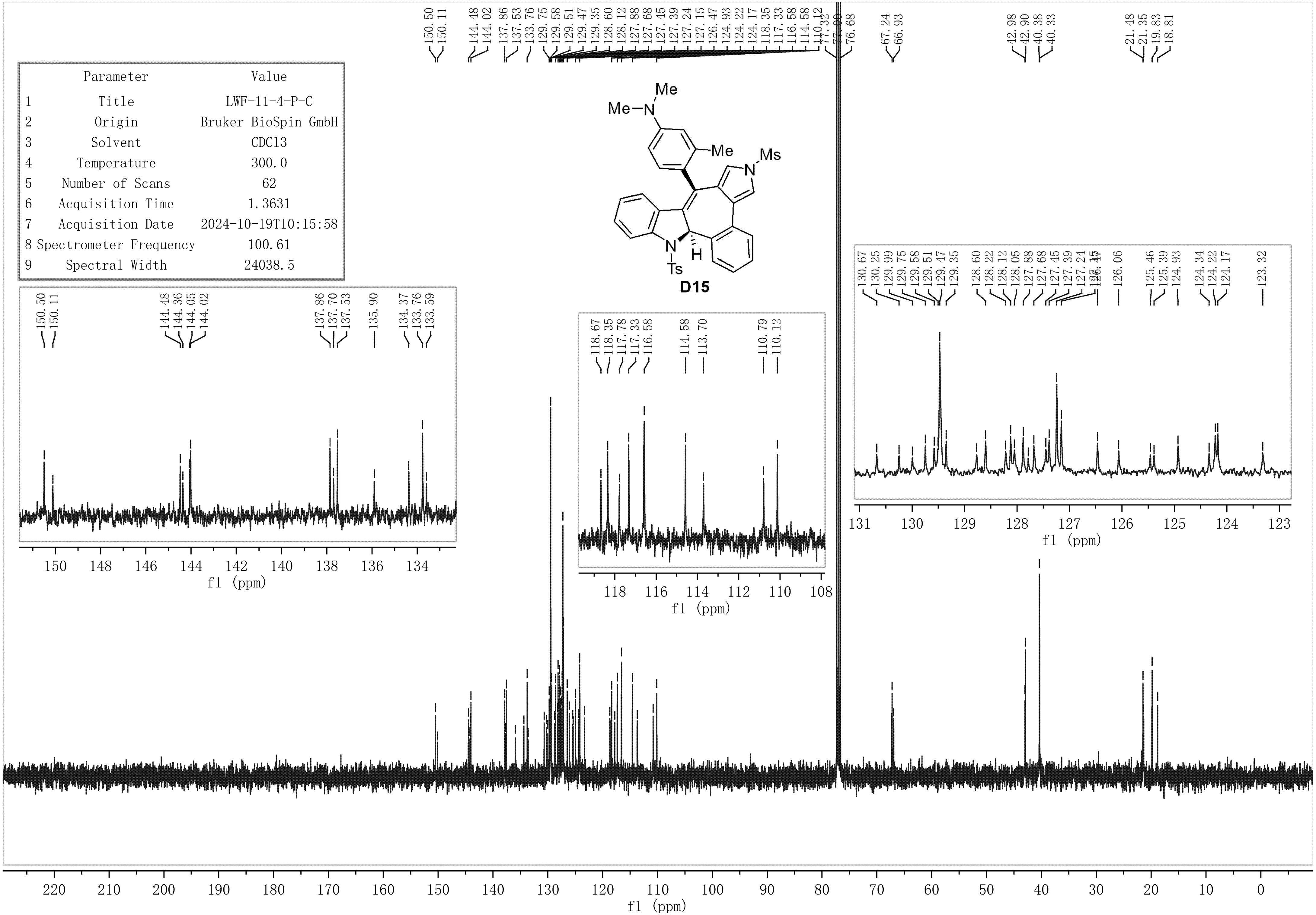

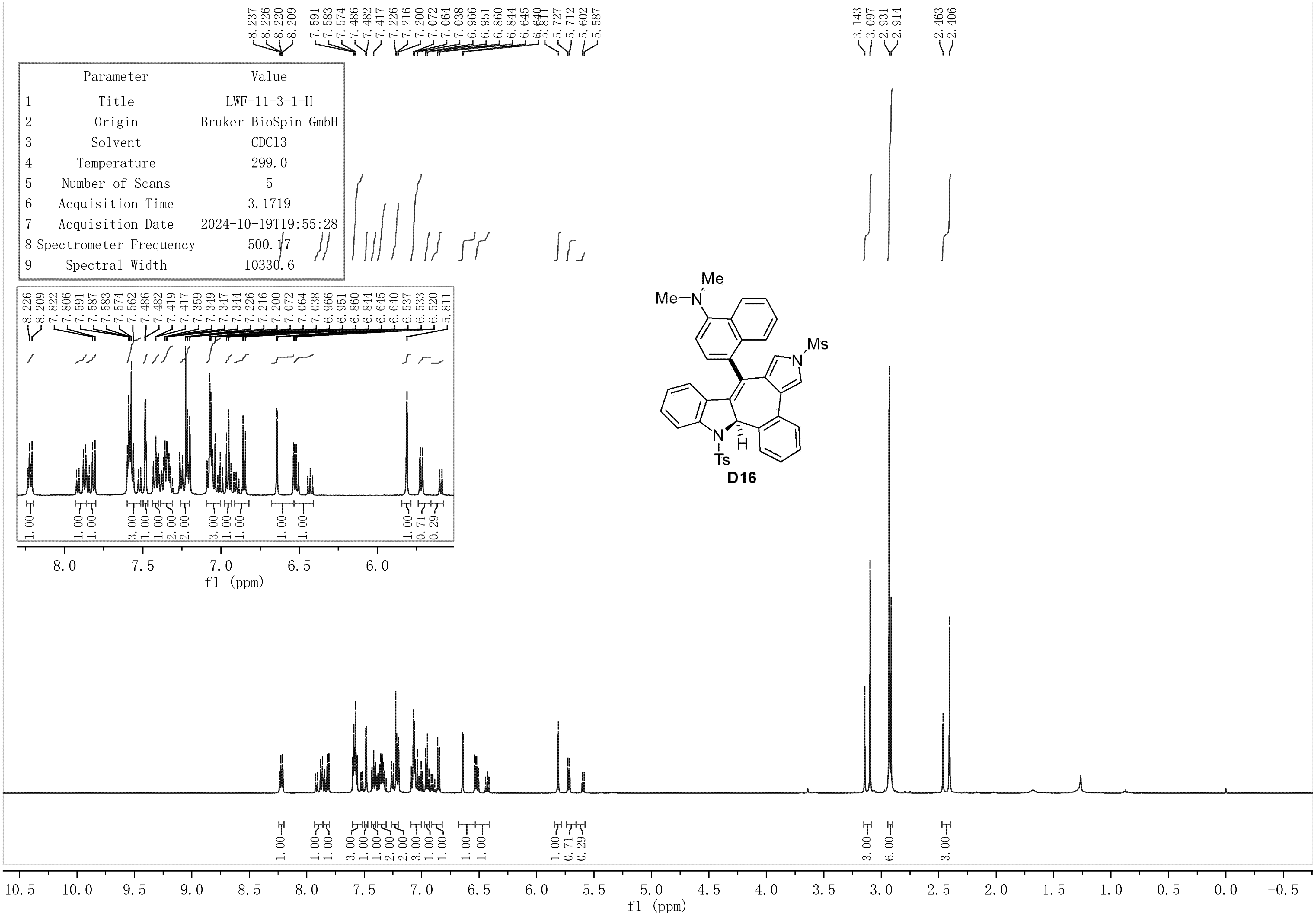

Parameter		Value
1	Title	LWF-11-3-1-H
2	Origin	Bruker BioSpin GmbH
3	Solvent	CDCl3
4	Temperature	299.0
5	Number of Scans	5
6	Acquisition Time	3.1719
7	Acquisition Date	2024-10-19T19:55:28
8	Spectrometer Frequency	500.17
9	Spectral Width	10330.6

D16

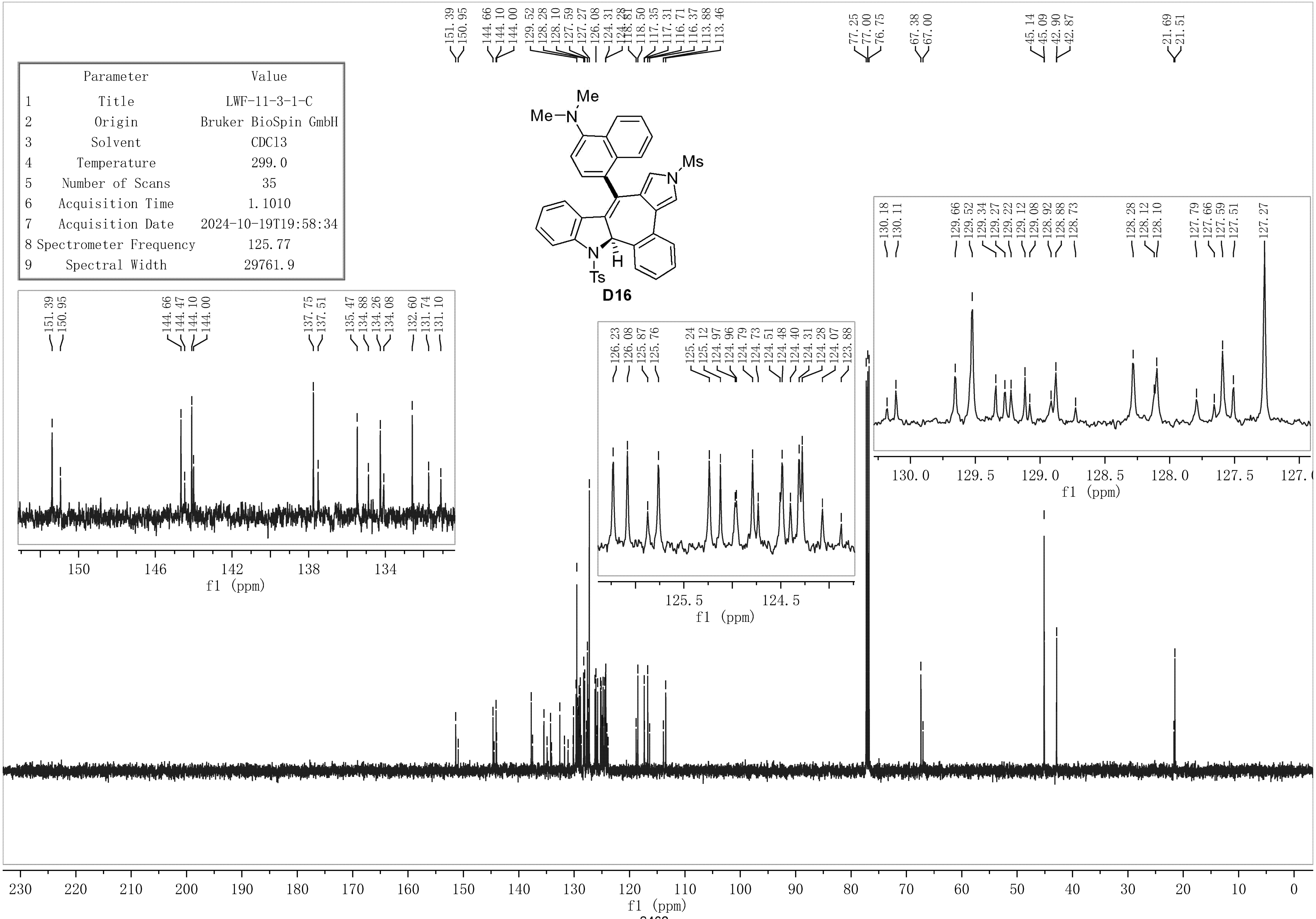

	Parameter	Value
1	Title	LWF-11-3-1-C
2	Origin	Bruker BioSpin GmbH
3	Solvent	CDCl3
4	Temperature	299.0
5	Number of Scans	35
6	Acquisition Time	1.1010
7	Acquisition Date	2024-10-19T19:58:34
8	Spectrometer Frequency	125.77
9	Spectral Width	29761.9

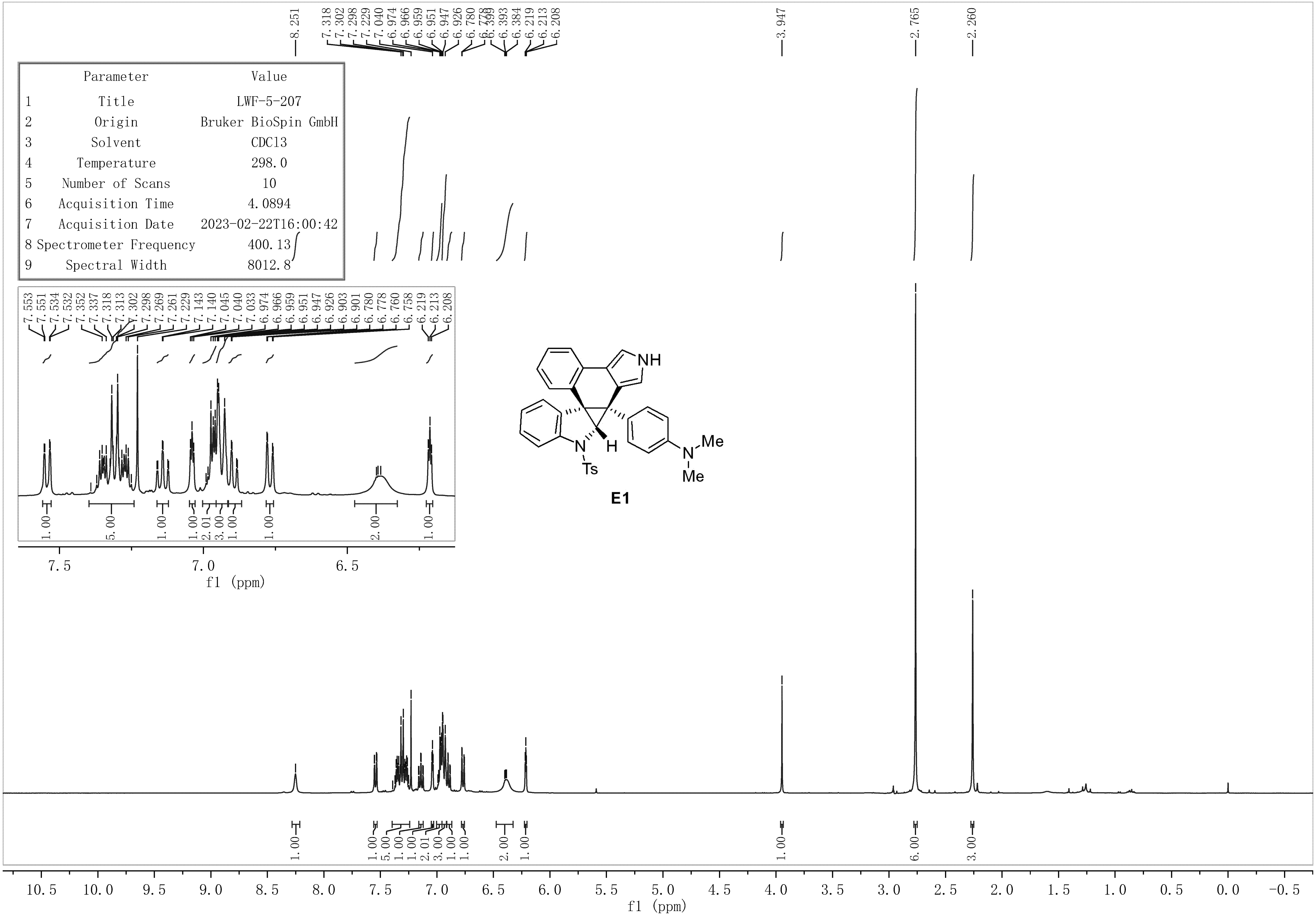
Parameter Value
1 Title LWF-5-207
2 Origin Bruker BioSpin GmbH
3 Solvent CDCl3
4 Temperature 298.0
5 Number of Scans 10
6 Acquisition Time 4.0894
7 Acquisition Date 2023-02-22T16:00:42
8 Spectrometer Frequency 400.13
9 Spectral Width 8012.8
8.251
7.318 7.302 7.298 7.229 7.040 6.974 6.966 6.959 6.951 6.947 6.926 6.780
6.393 6.384 6.219 6.213 6.208
3.947
2.765
2.260
7.553 7.551 7.534 7.532 7.352 7.337 7.318 7.313 7.302 7.298 7.269 7.261 7.229 7.143 7.140 7.045 7.040 7.033 6.974 6.966 6.959 6.951 6.947 6.926 6.903 6.901 6.780 6.778 6.760 6.758 6.219 6.213 6.208
NH
N
Ts
H
N
Me
Me
E1
f1 (ppm)

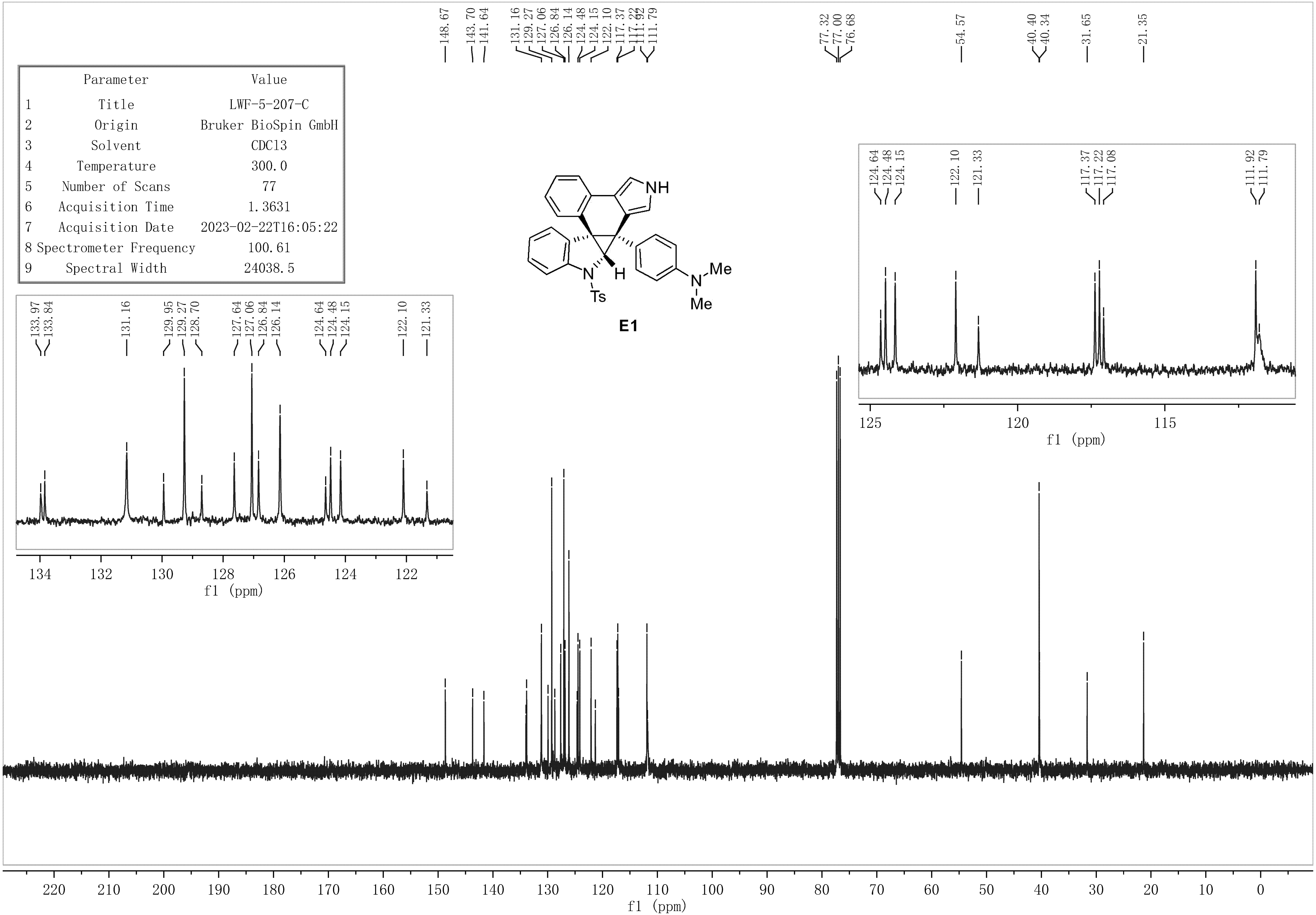

	Parameter	Value
1	Title	LWF-5-207-C
2	Origin	Bruker BioSpin GmbH
3	Solvent	CDCl3
4	Temperature	300.0
5	Number of Scans	77
6	Acquisition Time	1.3631
7	Acquisition Date	2023-02-22T16:05:22
8	Spectrometer Frequency	100.61
9	Spectral Width	24038.5

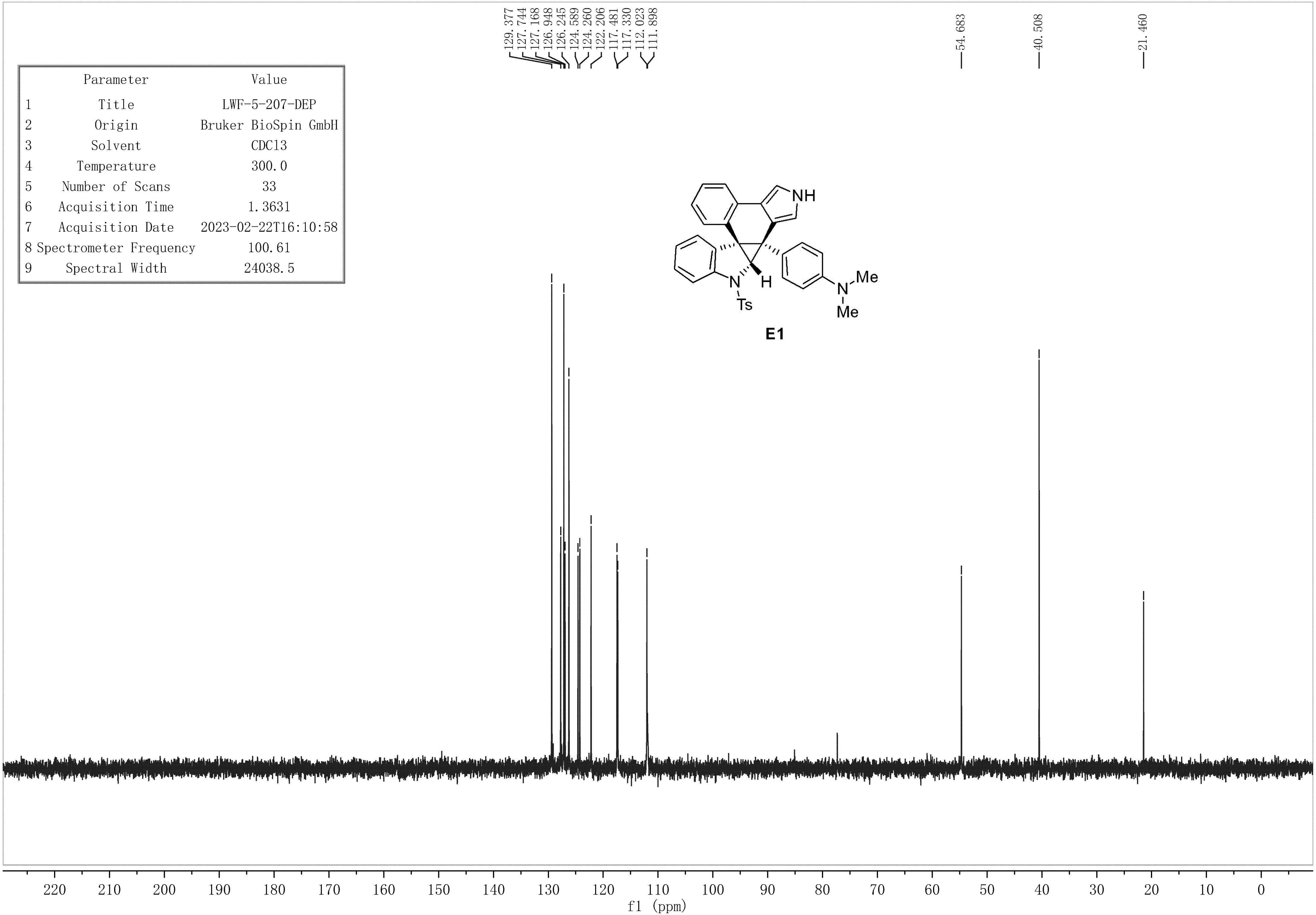

	Parameter	Value
1	Title	LWF-5-207-DEP
2	Origin	Bruker BioSpin GmbH
3	Solvent	CDCl3
4	Temperature	300.0
5	Number of Scans	33
6	Acquisition Time	1.3631
7	Acquisition Date	2023-02-22T16:10:58
8	Spectrometer Frequency	100.61
9	Spectral Width	24038.5

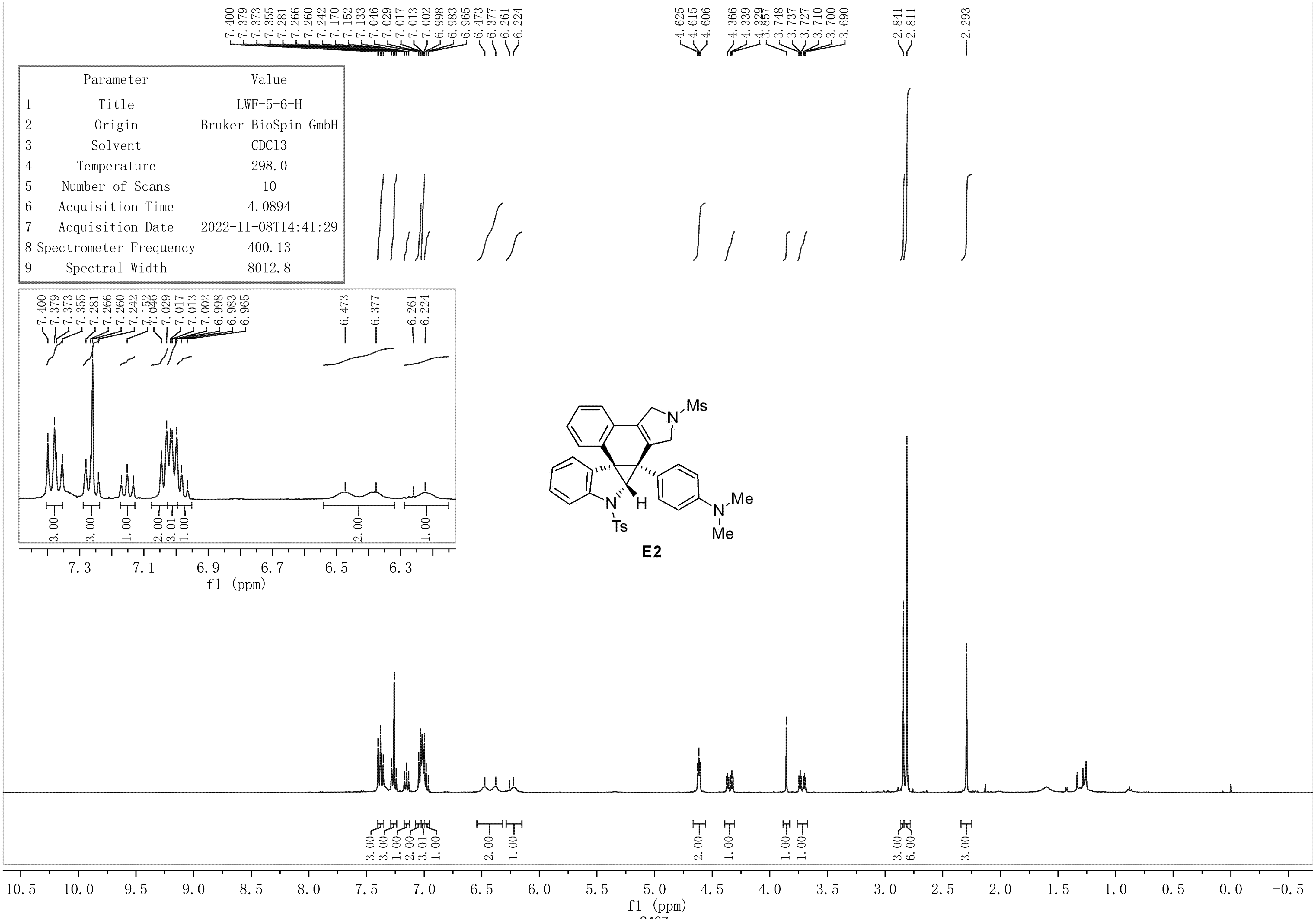
Parameter Value
1 Title LWF-5-6-H
2 Origin Bruker BioSpin GmbH
3 Solvent CDCl3
4 Temperature 298.0
5 Number of Scans 10
6 Acquisition Time 4.0894
7 Acquisition Date 2022-11-08T14:41:29
8 Spectrometer Frequency 400.13
9 Spectral Width 8012.8
7.400 7.379 7.373 7.355 7.281 7.266 7.260 7.242 7.170 7.152 7.133 7.046 7.029 7.017 7.013 7.002 6.998 6.983 6.965 6.473 6.377 6.261 6.224
4.625 4.615 4.606 4.366 4.339 4.329 3.857 3.748 3.737 3.727 3.710 3.700 3.690
2.841 2.811
2.293
3.00 3.00 1.00 2.00 3.01 1.00 2.00 1.00 2.00 1.00 1.00 1.00 3.00 6.00 3.00
Ms
N
Me
Ts
H
E2
f1 (ppm)

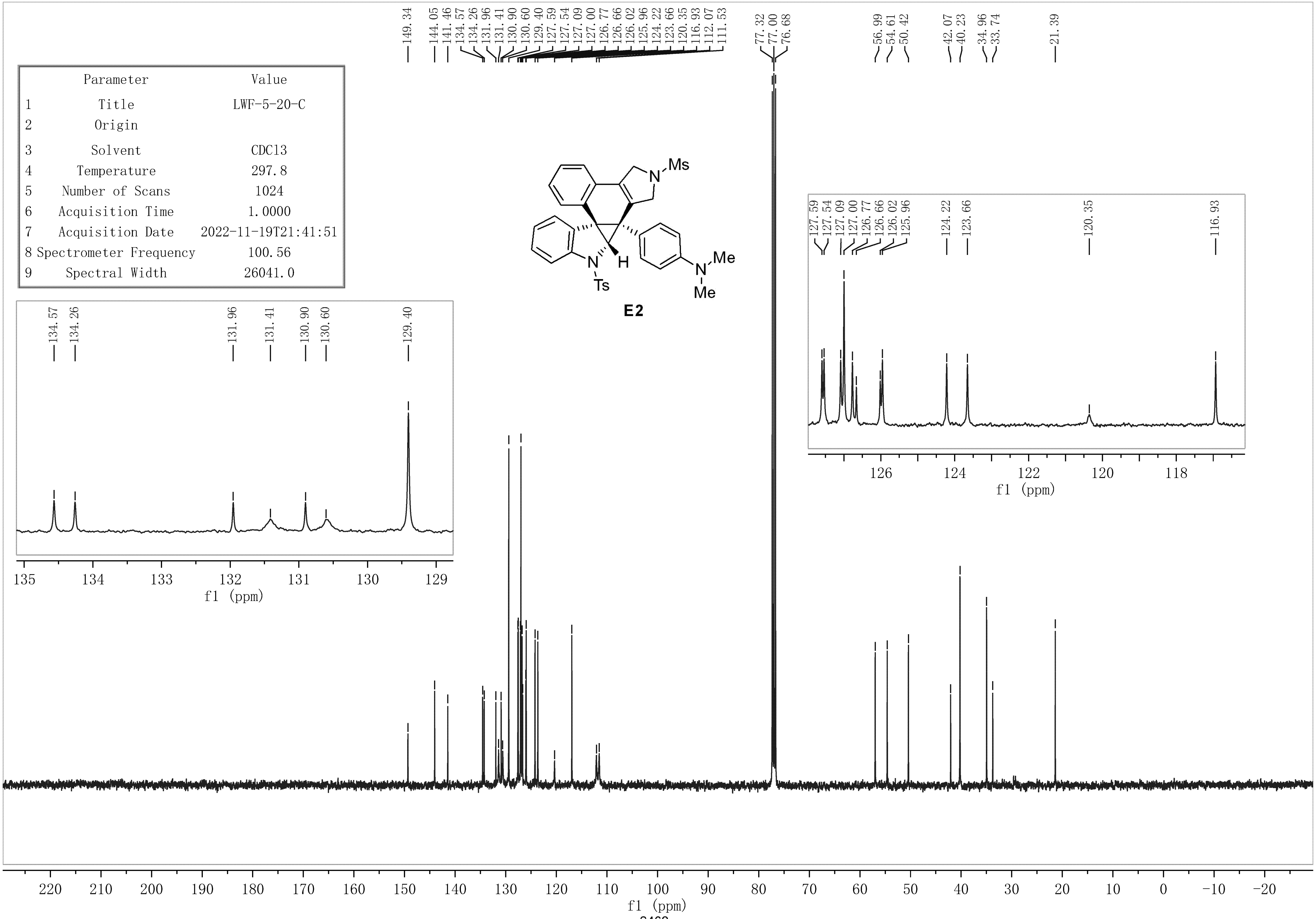
149.34
144.05
141.46
134.57
134.26
131.96
131.41
130.90
130.60
129.40
127.59
127.54
127.09
127.00
126.77
126.66
126.02
125.96
124.22
123.66
120.35
116.93
112.07
111.53
77.32
77.00
76.68
56.99
54.61
50.42
42.07
40.23
34.96
33.74
21.39
Parameter Value
1 Title LWF-5-20-C
2 Origin
3 Solvent CDCl3
4 Temperature 297.8
5 Number of Scans 1024
6 Acquisition Time 1.0000
7 Acquisition Date 2022-11-19T21:41:51
8 Spectrometer Frequency 100.56
9 Spectral Width 26041.0
Ms
N
Me
N
Me
H
N
Ts
E2
f1 (ppm)

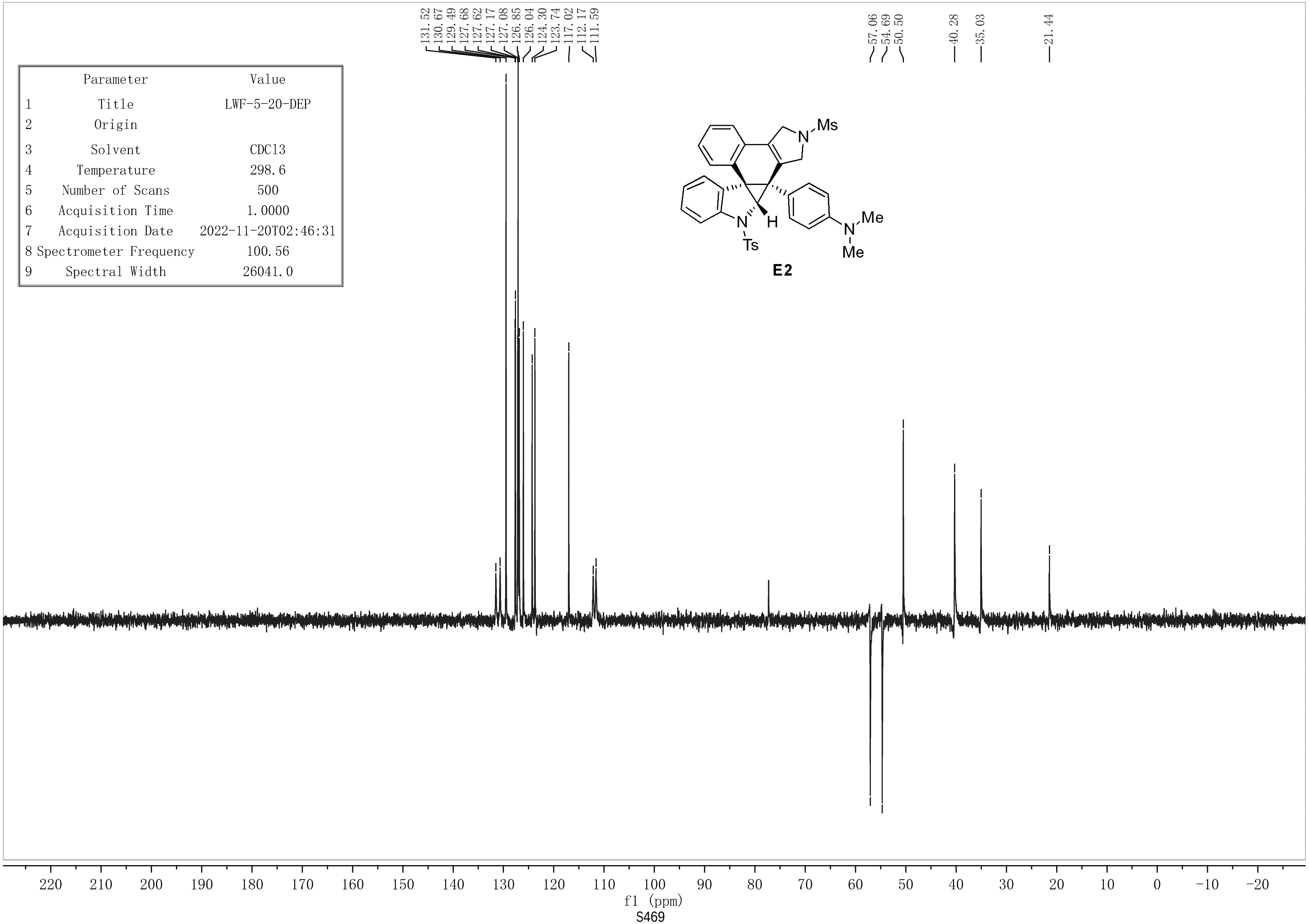

	Parameter	Value
1	Title	LWF-5-20-DEP
2	Origin	
3	Solvent	CDCl3
4	Temperature	298.6
5	Number of Scans	500
6	Acquisition Time	1.0000
7	Acquisition Date	2022-11-20T02:46:31
8	Spectrometer Frequency	100.56
9	Spectral Width	26041.0

E2

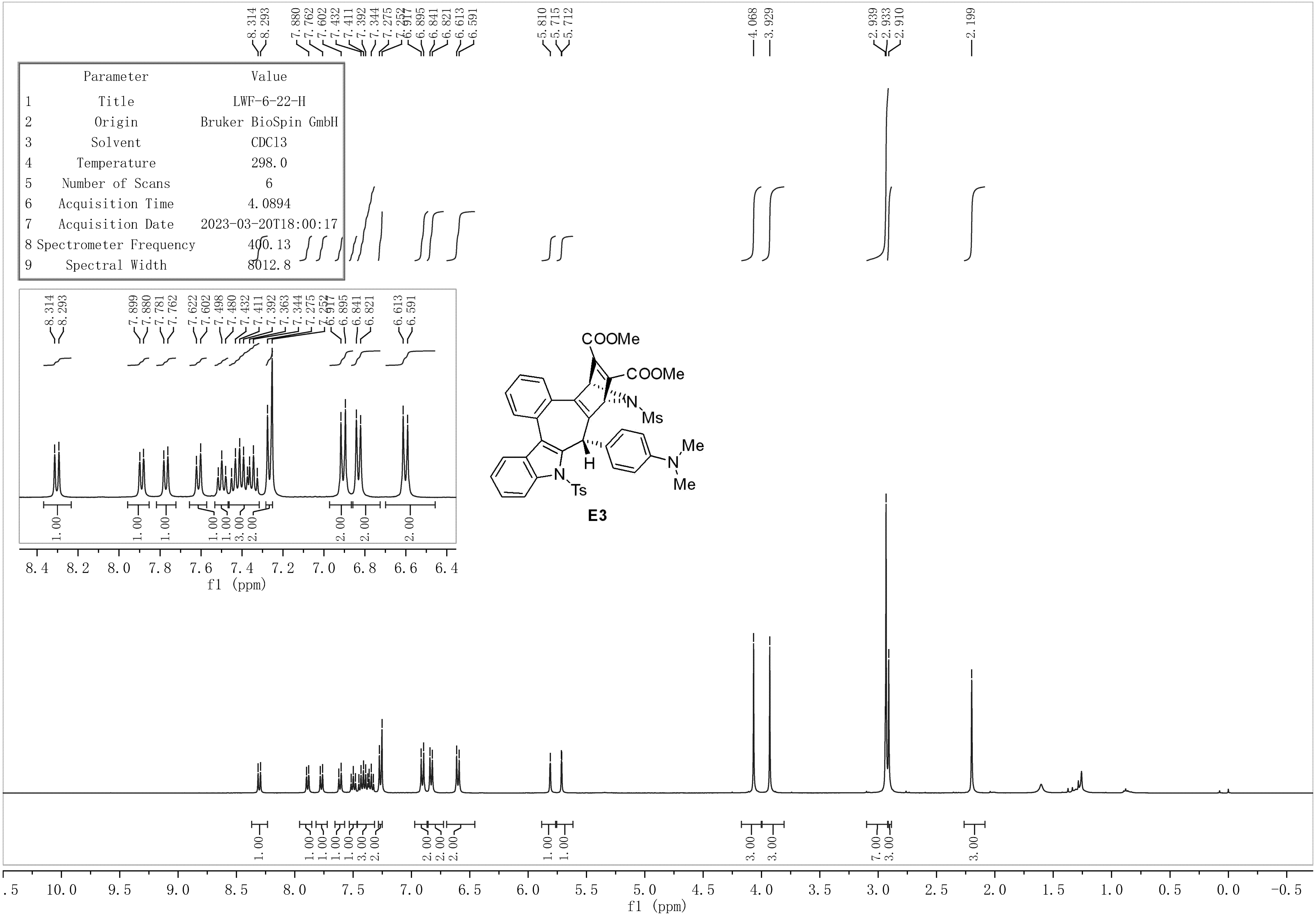

	Parameter	Value
1	Title	LWF-6-22-H
2	Origin	Bruker BioSpin GmbH
3	Solvent	CDCl3
4	Temperature	298.0
5	Number of Scans	6
6	Acquisition Time	4.0894
7	Acquisition Date	2023-03-20T18:00:17
8	Spectrometer Frequency	400.13
9	Spectral Width	8012.8

E3

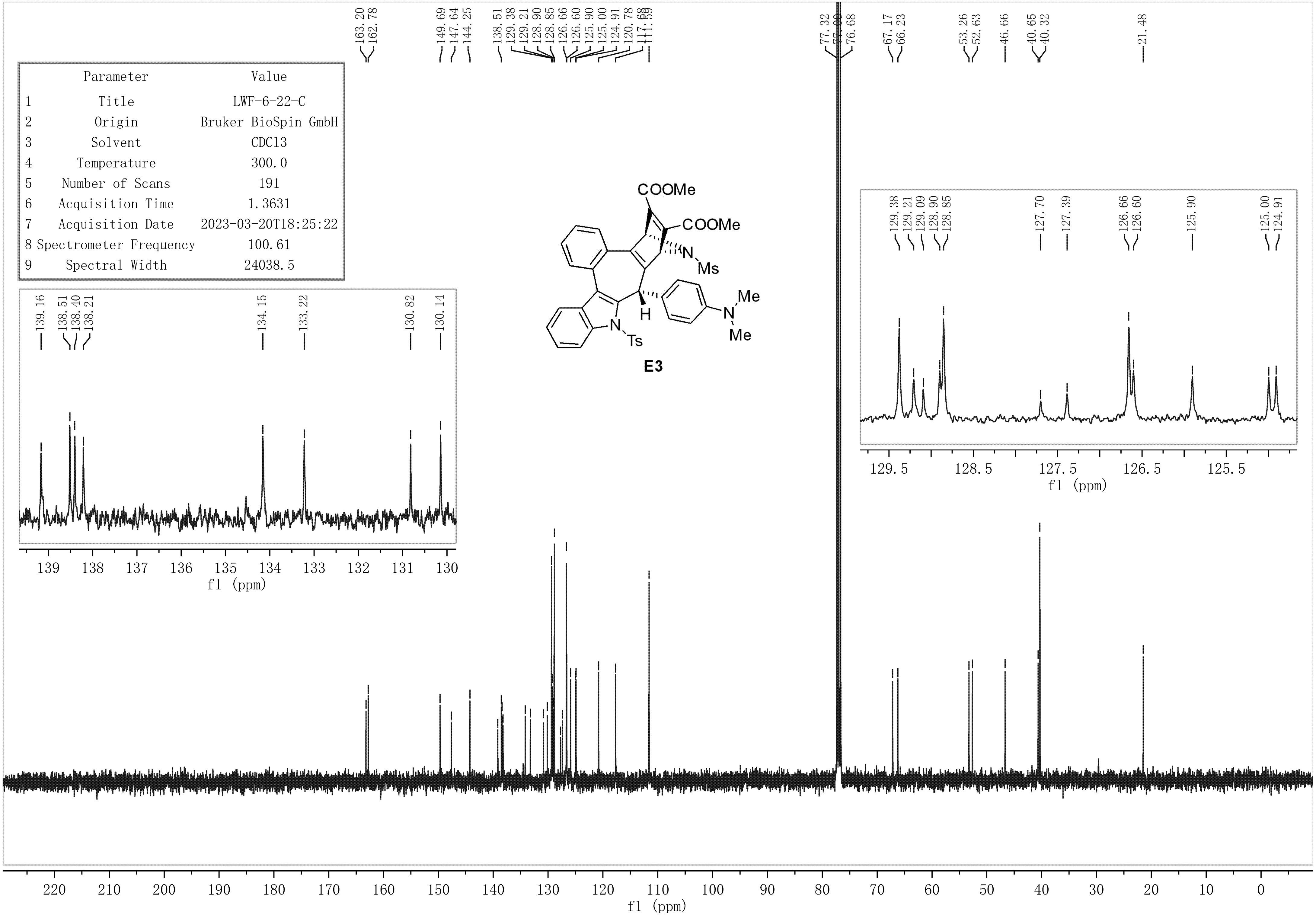
163.20
162.78
149.69
147.64
144.25
138.51
129.38
129.21
128.90
128.85
126.66
126.60
125.90
125.00
124.91
120.78
77.32
77.00
76.68
67.17
66.23
53.26
52.63
46.66
40.65
40.32
21.48
Parameter Value
1 Title LWF-6-22-C
2 Origin Bruker BioSpin GmbH
3 Solvent CDCl3
4 Temperature 300.0
5 Number of Scans 191
6 Acquisition Time 1.3631
7 Acquisition Date 2023-03-20T18:25:22
8 Spectrometer Frequency 100.61
9 Spectral Width 24038.5
COOMe
COOMe
N
Ms
H
Me
N
Me
N
Ts
E3
139.16
138.51
138.40
138.21
134.15
133.22
130.82
130.14
f1 (ppm)
129.38
129.21
129.09
128.90
128.85
127.70
127.39
126.66
126.60
125.90
125.00
124.91
f1 (ppm)
f1 (ppm)

	Parameter	Value
1	Title	LWF-6-22-DEP
2	Origin	Bruker BioSpin GmbH
3	Solvent	CDCl3
4	Temperature	300.0
5	Number of Scans	41
6	Acquisition Time	1.3631
7	Acquisition Date	2023-03-20T18:22:31
8	Spectrometer Frequency	100.61
9	Spectral Width	24038.5

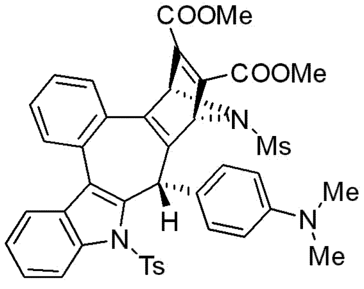

E3

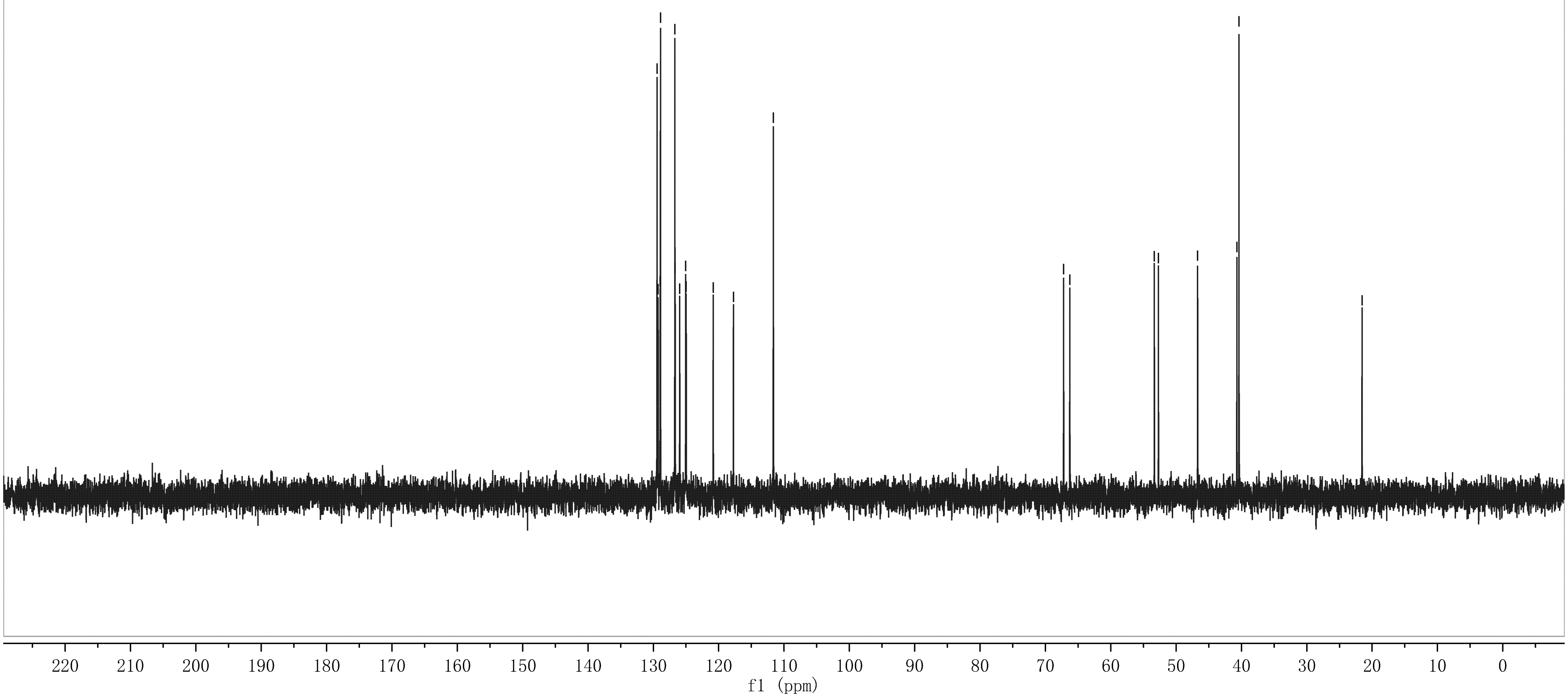

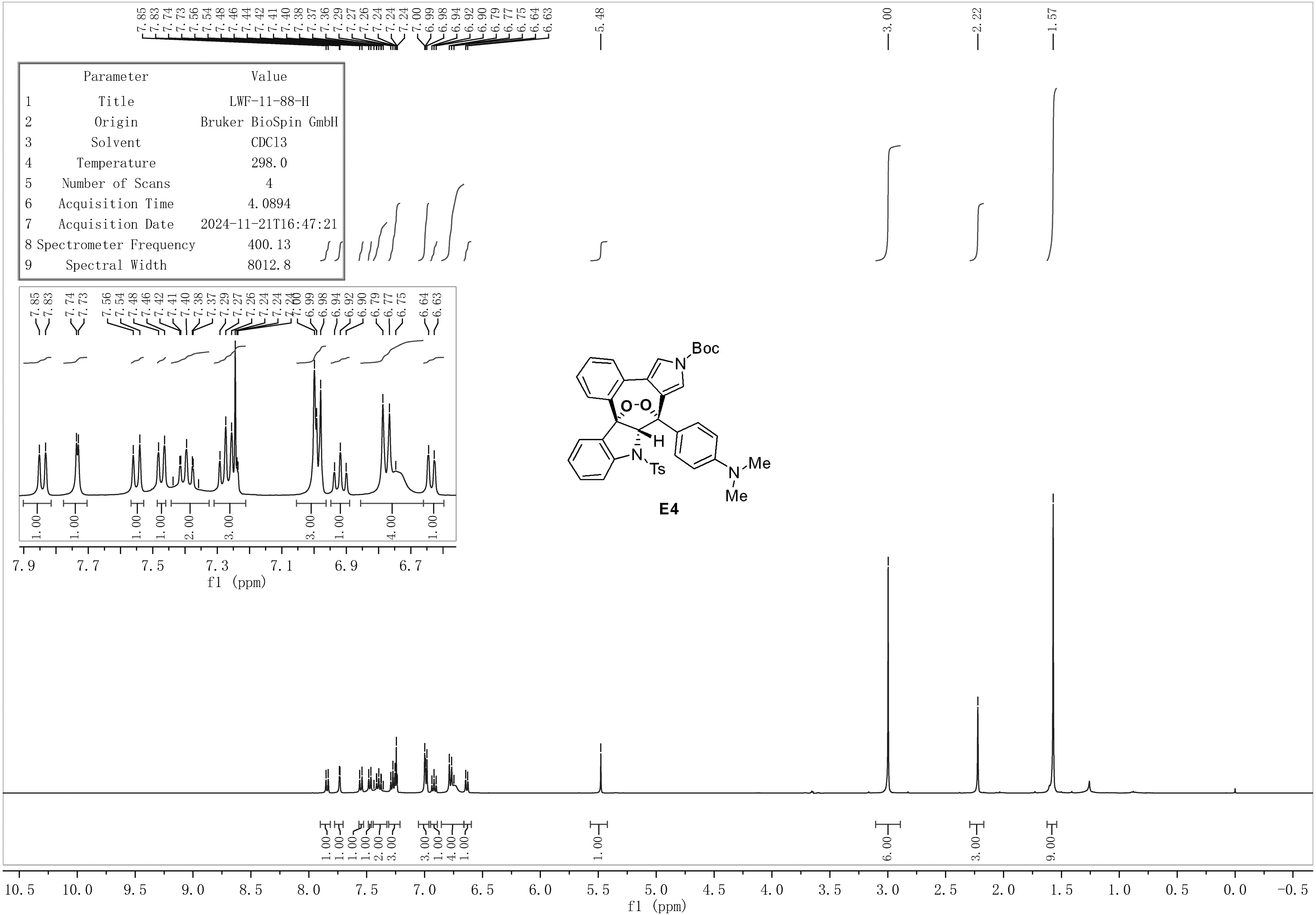

	Parameter	Value
1	Title	LWF-11-88-H
2	Origin	Bruker BioSpin GmbH
3	Solvent	CDCl3
4	Temperature	298.0
5	Number of Scans	4
6	Acquisition Time	4.0894
7	Acquisition Date	2024-11-21T16:47:21
8	Spectrometer Frequency	400.13
9	Spectral Width	8012.8

	Parameter	Value
1	Title	LWF-11-88-C
2	Origin	Bruker BioSpin GmbH
3	Solvent	CDCl3
4	Temperature	300.0
5	Number of Scans	43
6	Acquisition Time	1.3631
7	Acquisition Date	2024-11-21T16:48:22
8	Spectrometer Frequency	100.61
9	Spectral Width	24038.5

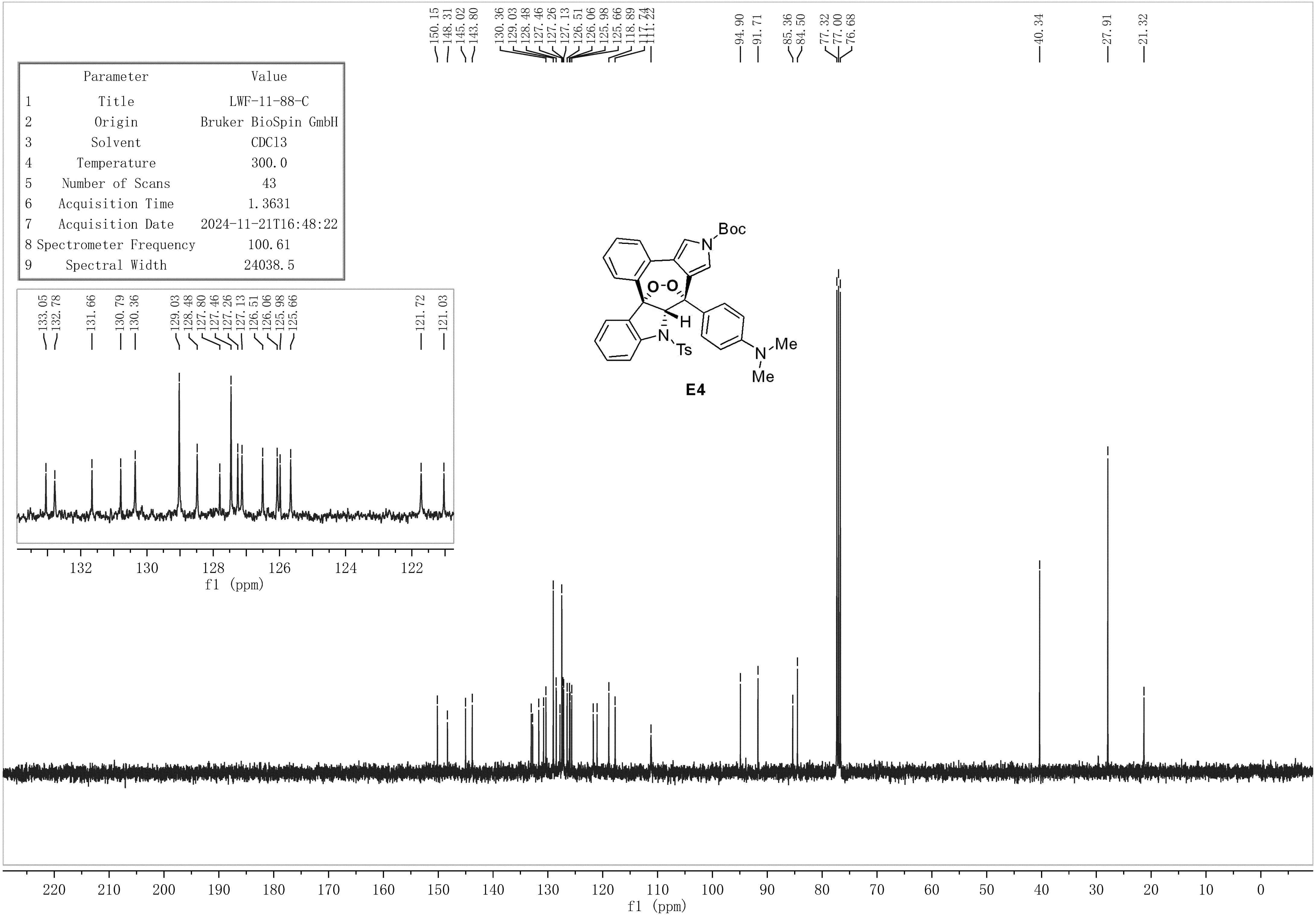

E4

	Parameter	Value
1	Title	LWF-11-88-DEP
2	Origin	Bruker BioSpin GmbH
3	Solvent	CDCl3
4	Temperature	300.0
5	Number of Scans	21
6	Acquisition Time	1.3631
7	Acquisition Date	2024-11-21T16:51:27
8	Spectrometer Frequency	100.61
9	Spectral Width	24038.5

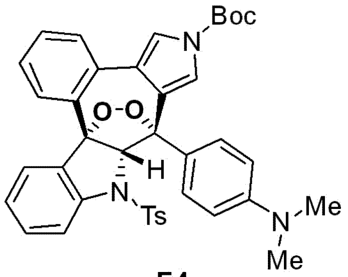

E4

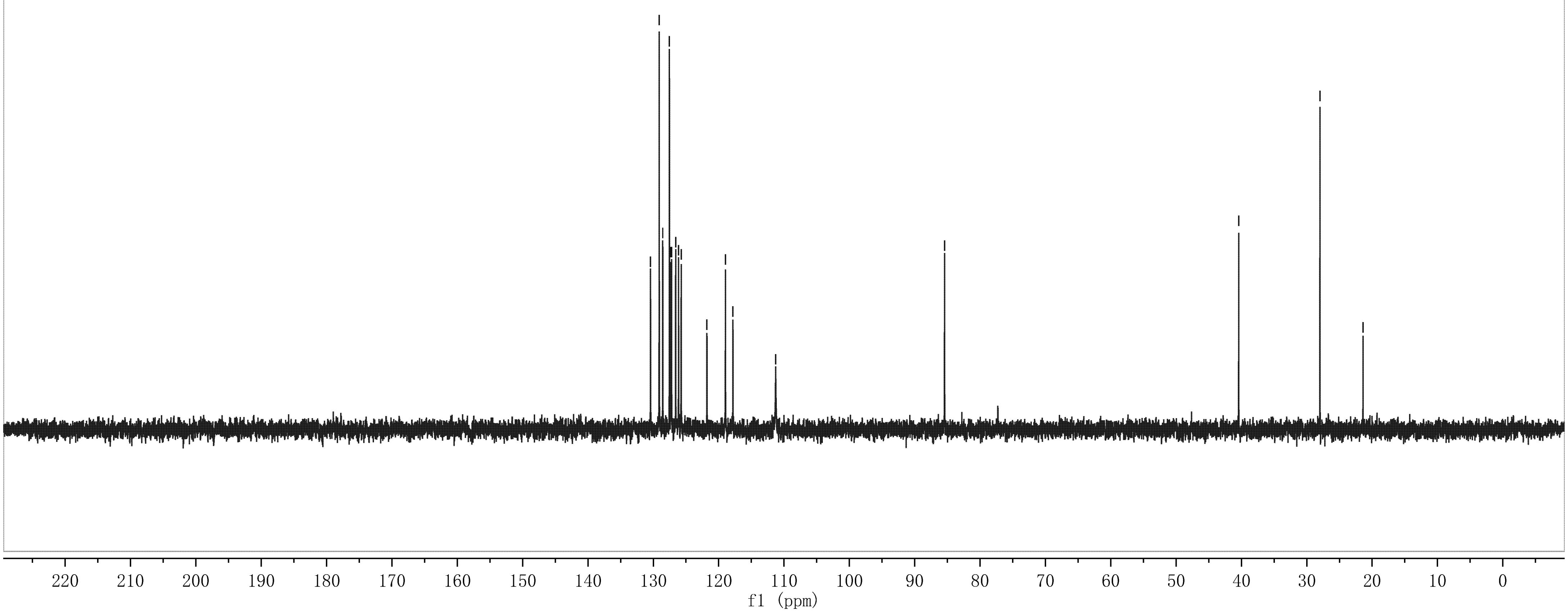

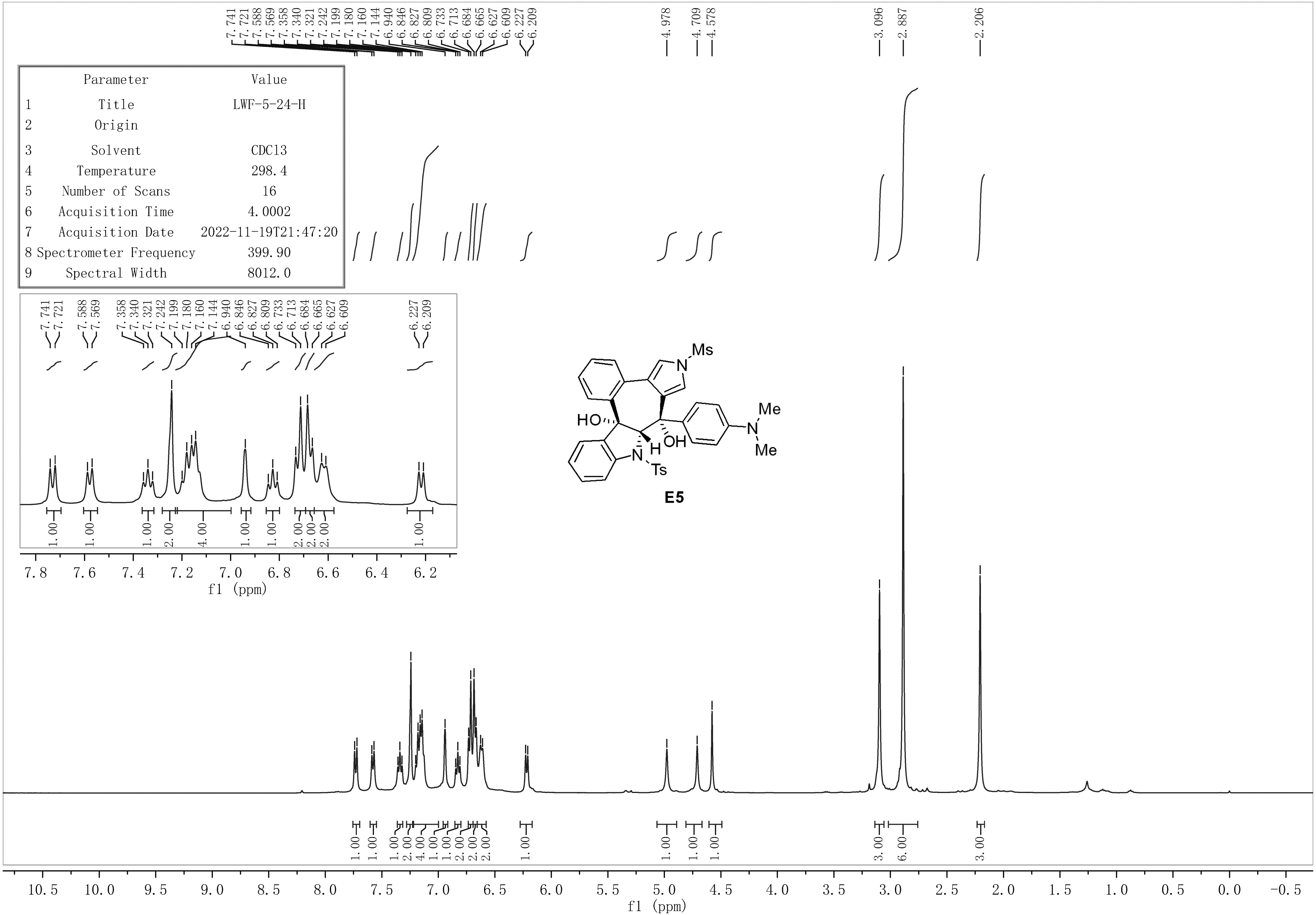

Parameter Value
1 Title LWF-5-24-H
2 Origin
3 Solvent CDCl3
4 Temperature 298.4
5 Number of Scans 16
6 Acquisition Time 4.0002
7 Acquisition Date 2022-11-19T21:47:20
8 Spectrometer Frequency 399.90
9 Spectral Width 8012.0
Ms
HO
OH
H
N
Ts
Me
Me
E5
f1 (ppm)

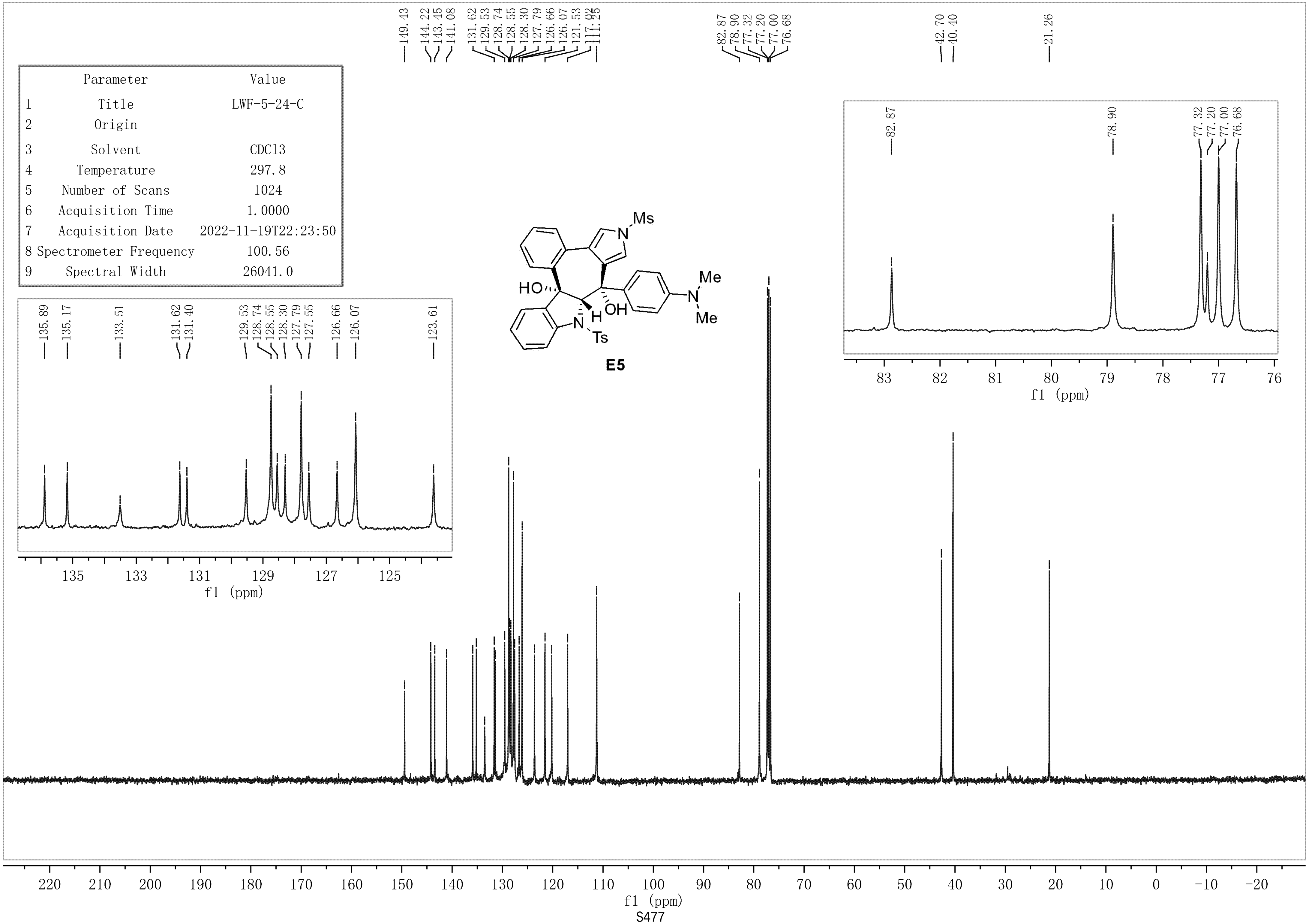
149.43
144.22
143.45
141.08
131.62
129.53
128.74
128.55
128.30
127.79
126.66
126.07
121.53
117.02
111.25
82.87
78.90
77.32
77.20
77.00
76.68
42.70
40.40
21.26
Parameter Value
1 Title LWF-5-24-C
2 Origin
3 Solvent CDCl3
4 Temperature 297.8
5 Number of Scans 1024
6 Acquisition Time 1.0000
7 Acquisition Date 2022-11-19T22:23:50
8 Spectrometer Frequency 100.56
9 Spectral Width 26041.0
135.89
135.17
133.51
131.62
131.40
129.53
128.74
128.55
128.30
127.79
127.55
126.66
126.07
123.61
135 133 131 129 127 125
f1 (ppm)
Ms
N
HO
OH
H
N
Ts
N
Me
Me
E5
82.87
78.90
77.32
77.20
77.00
76.68
83 82 81 80 79 78 77 76
f1 (ppm)
220 210 200 190 180 170 160 150 140 130 120 110 100 90 80 70 60 50 40 30 20 10 0 -10 -20
f1 (ppm)

	Parameter	Value
1	Title	LWF-5-24-DEP
2	Origin	Bruker BioSpin GmbH
3	Solvent	CDCl3
4	Temperature	300.0
5	Number of Scans	62
6	Acquisition Time	1.3631
7	Acquisition Date	2022-11-20T18:04:23
8	Spectrometer Frequency	100.61
9	Spectral Width	24038.5

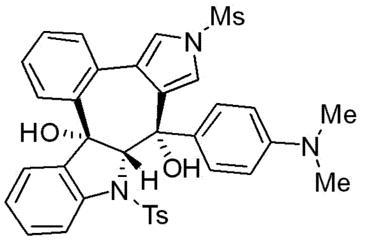

E5

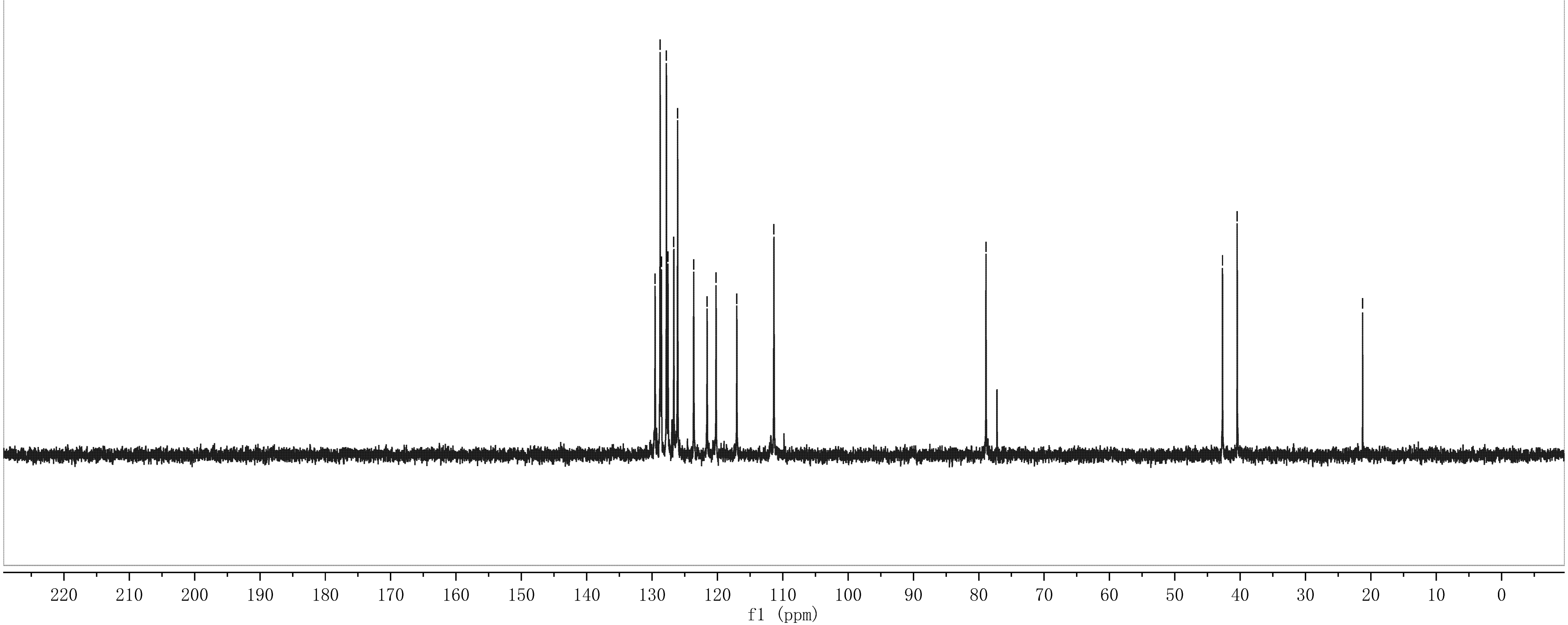

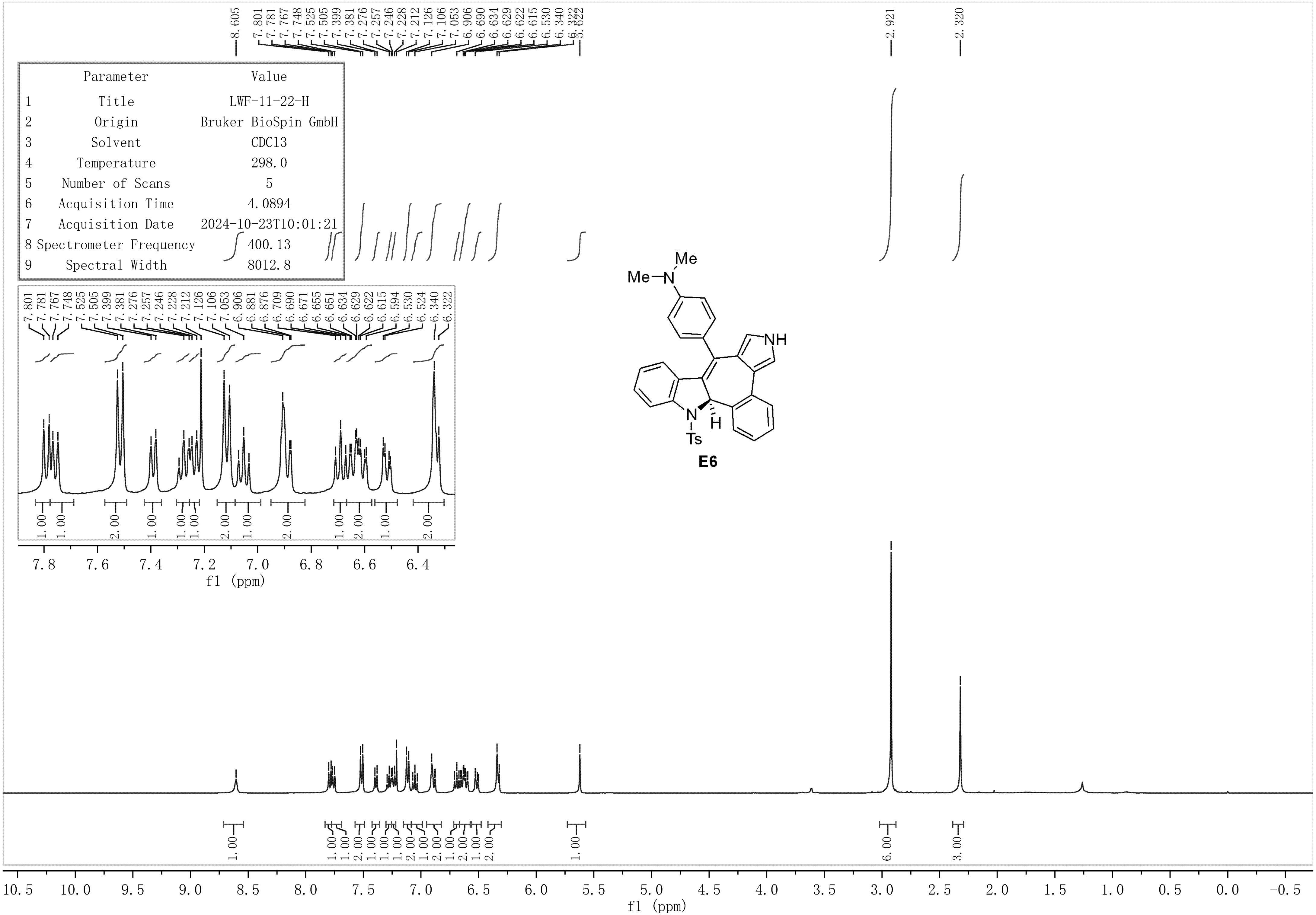

8.605
7.801 7.781 7.767 7.748 7.525 7.505 7.399 7.381 7.276 7.257 7.246 7.228 7.212 7.126 7.106 7.053 6.906 6.690 6.634 6.629 6.622 6.615 6.530 6.340 6.322 5.622
2.921
2.320
Parameter Value
1 Title LWF-11-22-H
2 Origin Bruker BioSpin GmbH
3 Solvent CDCl3
4 Temperature 298.0
5 Number of Scans 5
6 Acquisition Time 4.0894
7 Acquisition Date 2024-10-23T10:01:21
8 Spectrometer Frequency 400.13
9 Spectral Width 8012.8
7.801 7.781 7.767 7.748 7.525 7.505 7.399 7.381 7.276 7.257 7.246 7.228 7.212 7.126 7.106 7.053 6.906 6.881 6.876 6.709 6.690 6.671 6.655 6.651 6.634 6.629 6.622 6.615 6.594 6.530 6.524 6.340 6.322
1.00 1.00 2.00 1.00 1.00 1.00 2.00 1.00 2.00 1.00 2.00 1.00 2.00
7.8 7.6 7.4 7.2 7.0 6.8 6.6 6.4
f1 (ppm)
Me
Me—N
NH
N
H
Ts
E6
1.00 1.00 1.00 2.00 1.00 1.00 1.00 2.00 1.00 2.00 1.00 2.00 1.00 2.00 1.00 6.00 3.00
10.5 10.0 9.5 9.0 8.5 8.0 7.5 7.0 6.5 6.0 5.5 5.0 4.5 4.0 3.5 3.0 2.5 2.0 1.5 1.0 0.5 0.0 -0.5
f1 (ppm)

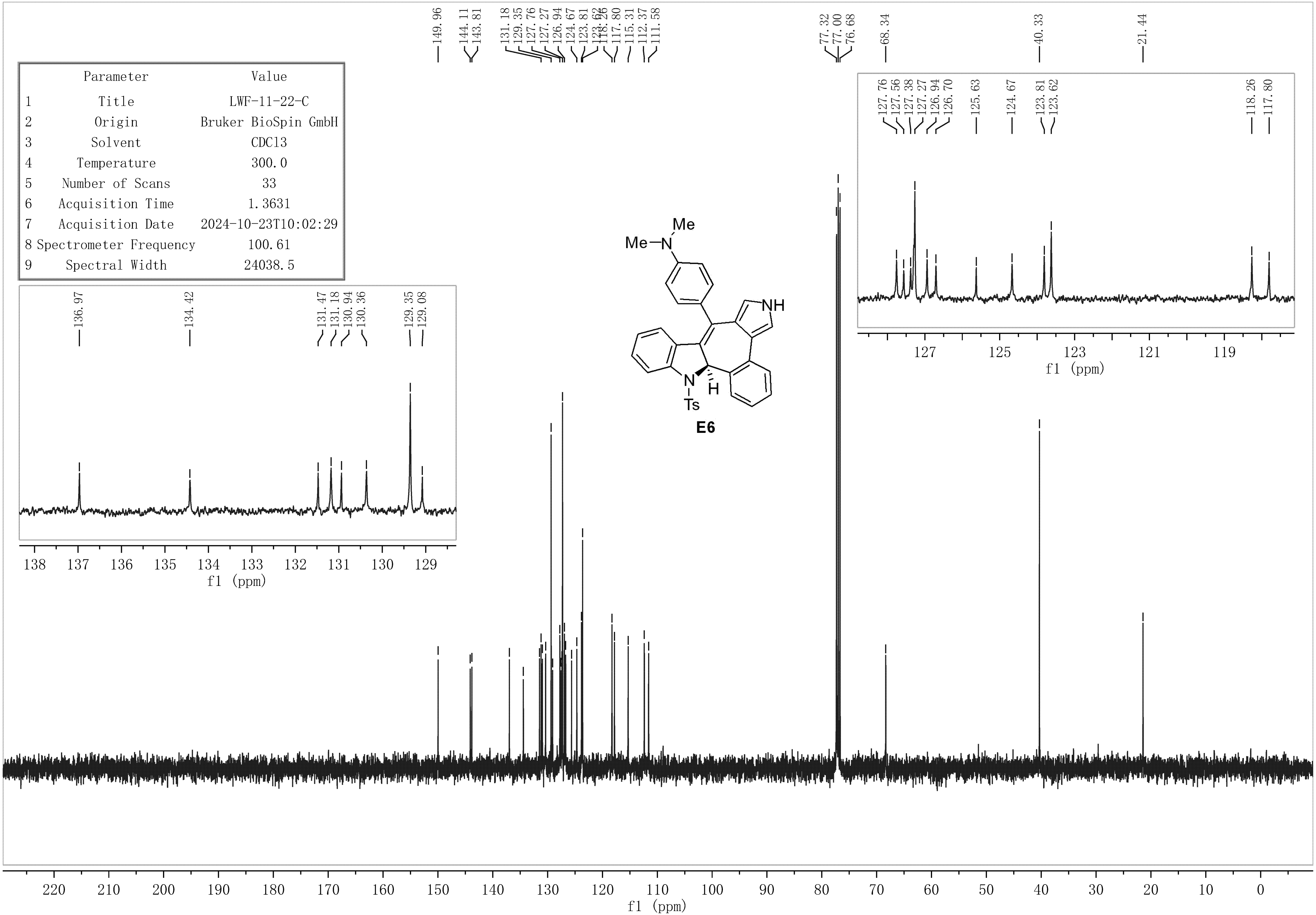

	Parameter	Value
1	Title	LWF-11-22-C
2	Origin	Bruker BioSpin GmbH
3	Solvent	CDCl3
4	Temperature	300.0
5	Number of Scans	33
6	Acquisition Time	1.3631
7	Acquisition Date	2024-10-23T10:02:29
8	Spectrometer Frequency	100.61
9	Spectral Width	24038.5

	Parameter	Value
1	Title	LWF-11-22-DEP
2	Origin	Bruker BioSpin GmbH
3	Solvent	CDCl3
4	Temperature	300.0
5	Number of Scans	17
6	Acquisition Time	1.3631
7	Acquisition Date	2024-10-23T10:05:15
8	Spectrometer Frequency	100.61
9	Spectral Width	24038.5

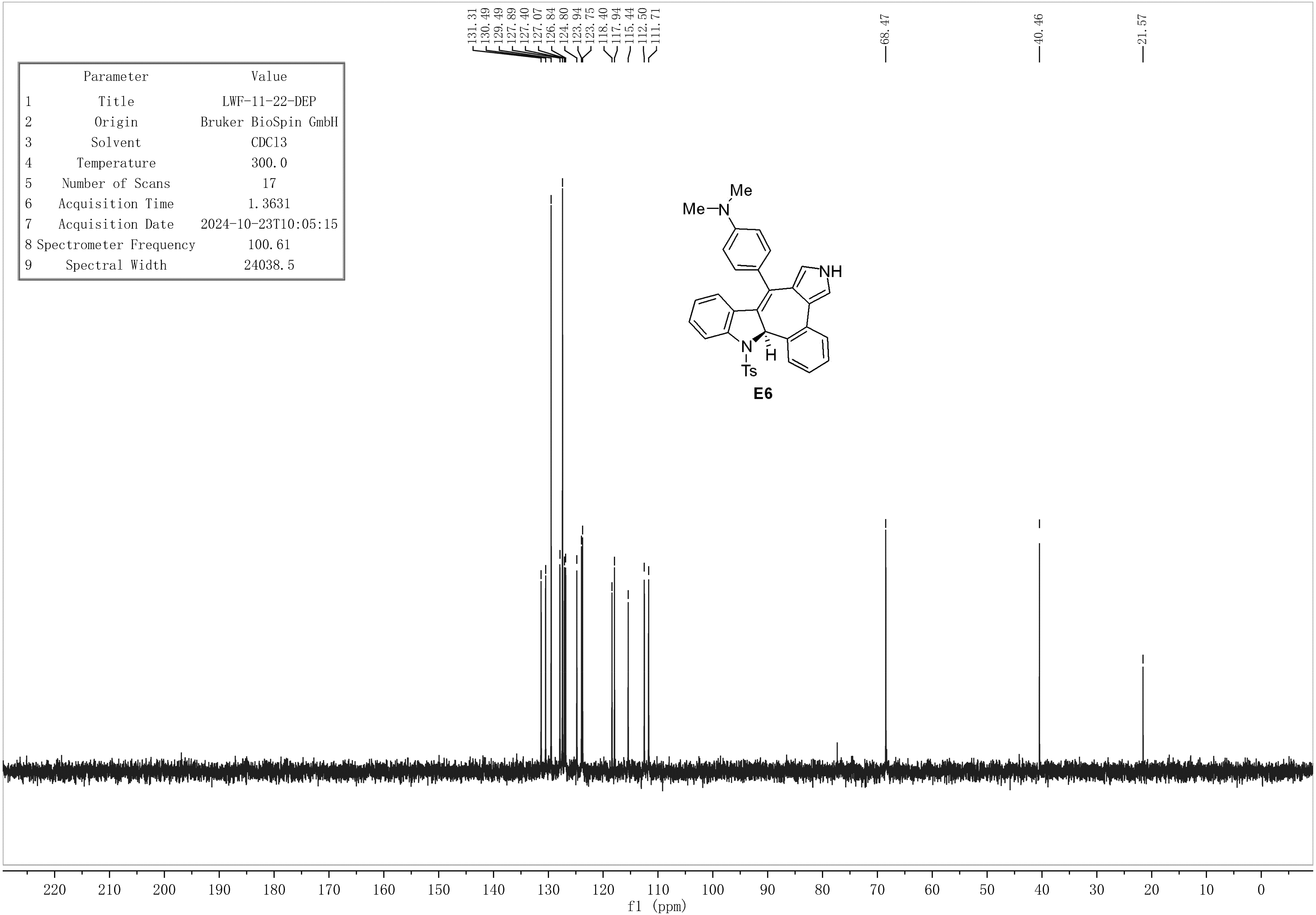

	Parameter	Value
1	Title	LWF-11-81-H
2	Origin	Bruker BioSpin GmbH
3	Solvent	CDCl3
4	Temperature	298.0
5	Number of Scans	4
6	Acquisition Time	4.0894
7	Acquisition Date	2024-11-19T12:10:53
8	Spectrometer Frequency	400.13
9	Spectral Width	8012.8

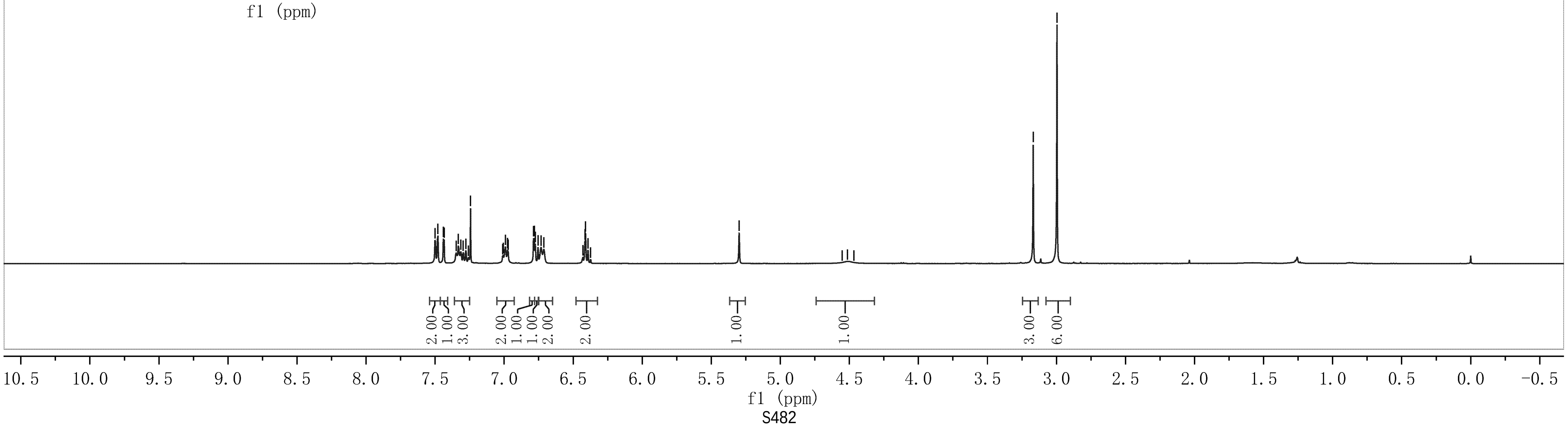

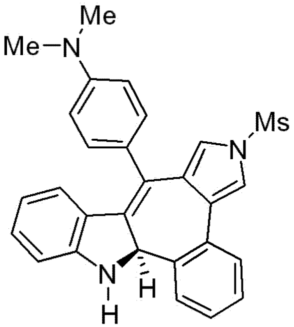

E7

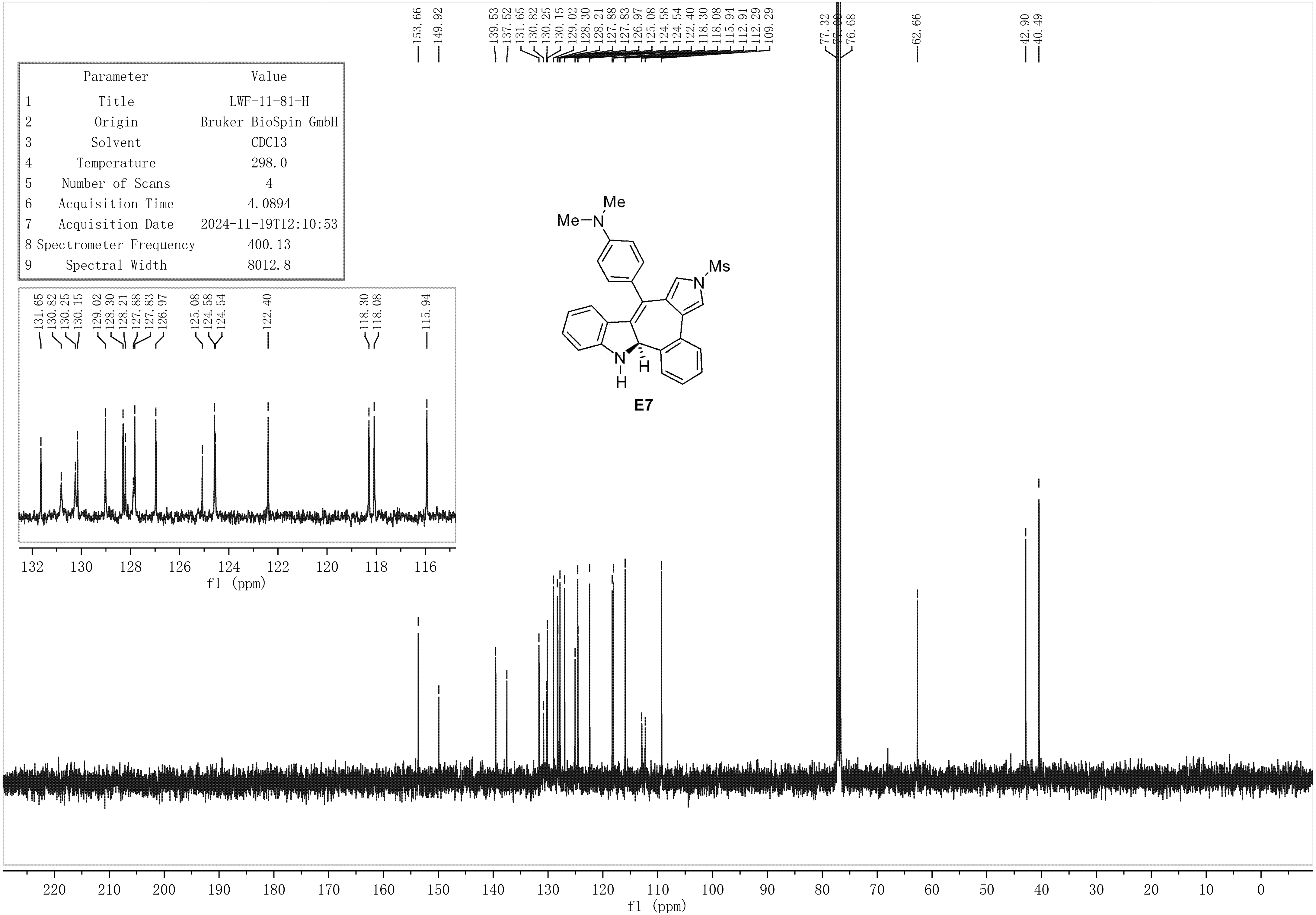

153.66
149.92
139.53
137.52
131.65
130.82
130.25
130.15
129.02
128.30
128.21
127.88
127.83
126.97
125.08
124.58
124.54
122.40
118.30
118.08
115.94
112.91
112.29
109.29
77.32
77.00
76.68
62.66
42.90
40.49
Parameter Value
1 Title LWF-11-81-H
2 Origin Bruker BioSpin GmbH
3 Solvent CDCl3
4 Temperature 298.0
5 Number of Scans 4
6 Acquisition Time 4.0894
7 Acquisition Date 2024-11-19T12:10:53
8 Spectrometer Frequency 400.13
9 Spectral Width 8012.8
Me
Me−N
Ms
N
N
H
H
E7
f1 (ppm)
f1 (ppm)

	Parameter	Value
1	Title	LWF-11-81-H
2	Origin	Bruker BioSpin GmbH
3	Solvent	CDCl3
4	Temperature	298.0
5	Number of Scans	4
6	Acquisition Time	4.0894
7	Acquisition Date	2024-11-19T12:10:53
8	Spectrometer Frequency	400.13
9	Spectral Width	8012.8

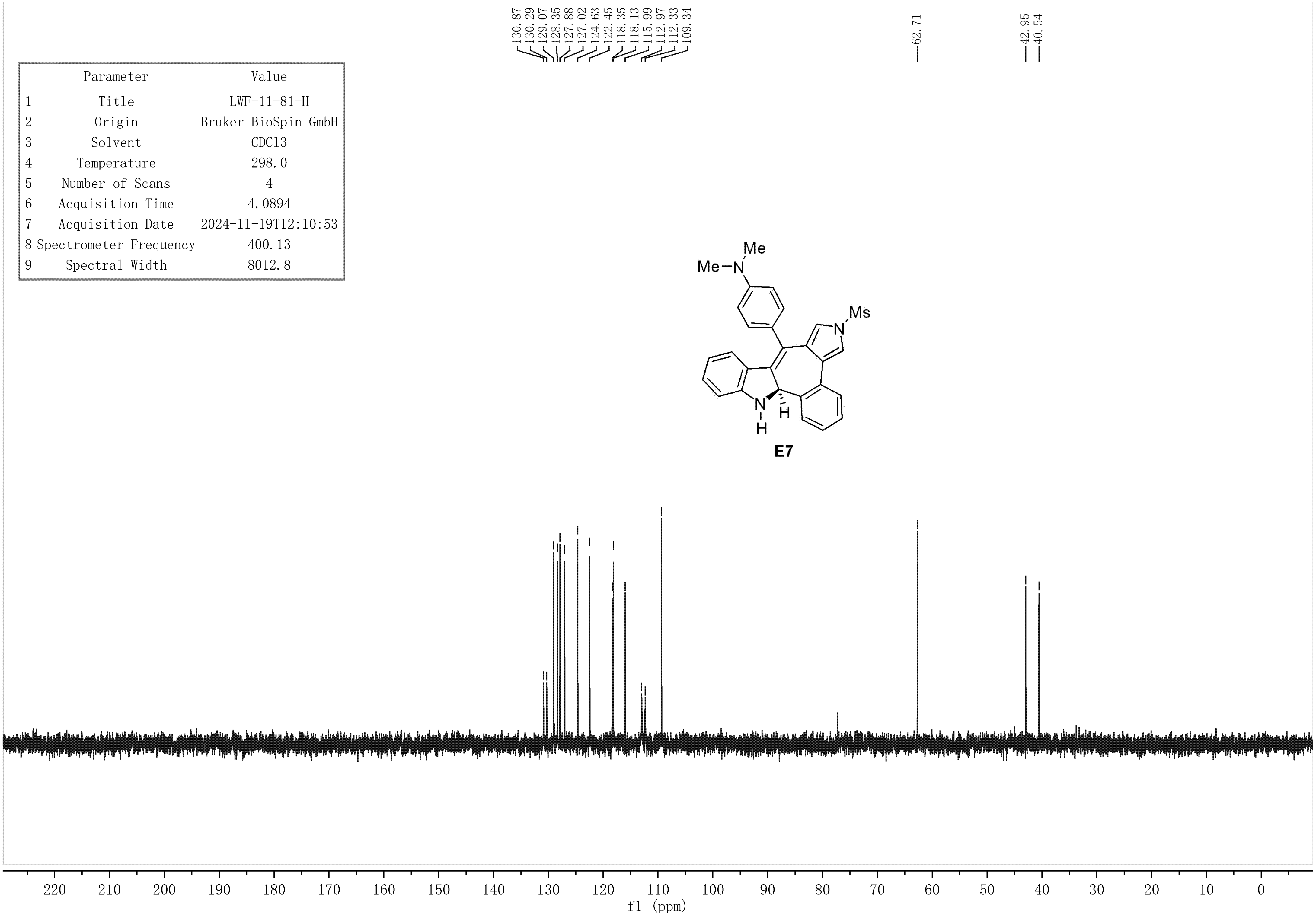

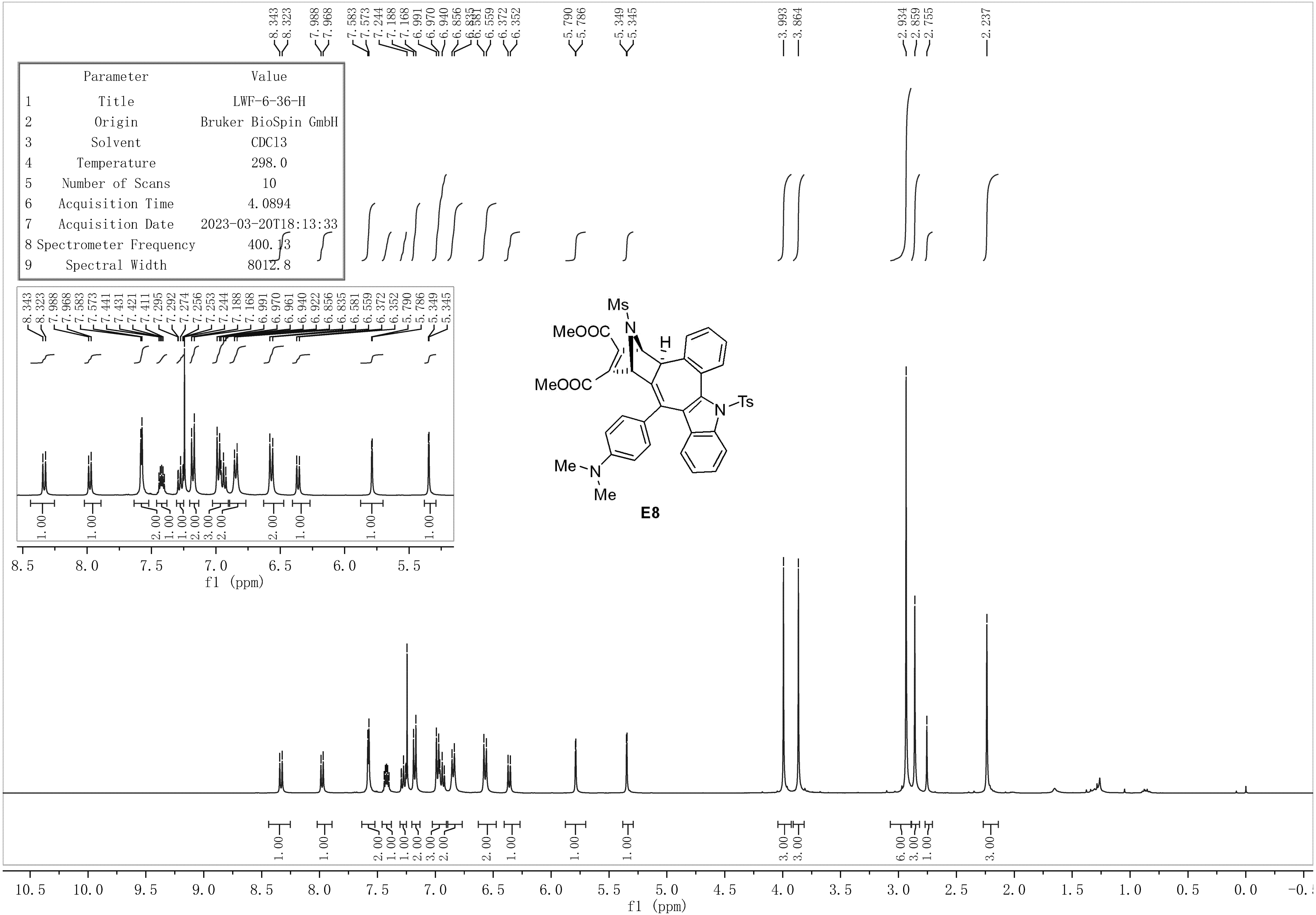
Parameter Value
1 Title LWF-6-36-H
2 Origin Bruker BioSpin GmbH
3 Solvent CDCl3
4 Temperature 298.0
5 Number of Scans 10
6 Acquisition Time 4.0894
7 Acquisition Date 2023-03-20T18:13:33
8 Spectrometer Frequency 400.13
9 Spectral Width 8012.8
8.343 8.323 7.988 7.968 7.583 7.573 7.244 7.188 7.168 6.991 6.970 6.940 6.856 6.835 6.581 6.559 6.372 6.352 5.790 5.786 5.349 5.345 3.993 3.864 2.934 2.859 2.755 2.237
1.00 1.00 2.00 1.00 1.00 2.00 3.00 2.00 2.00 1.00 1.00 1.00 3.00 3.00 6.00 3.00 1.00 3.00
8.343 8.323 7.988 7.968 7.583 7.573 7.441 7.431 7.421 7.411 7.295 7.292 7.274 7.256 7.253 7.244 7.188 7.168 6.991 6.970 6.961 6.940 6.922 6.856 6.835 6.581 6.559 6.372 6.352 5.790 5.786 5.349 5.345
f1 (ppm)
Ms
N
MeOOC
H
MeOOC
N
Ts
Me
N
Me
E8
10.5 10.0 9.5 9.0 8.5 8.0 7.5 7.0 6.5 6.0 5.5 5.0 4.5 4.0 3.5 3.0 2.5 2.0 1.5 1.0 0.5 0.0
f1 (ppm)

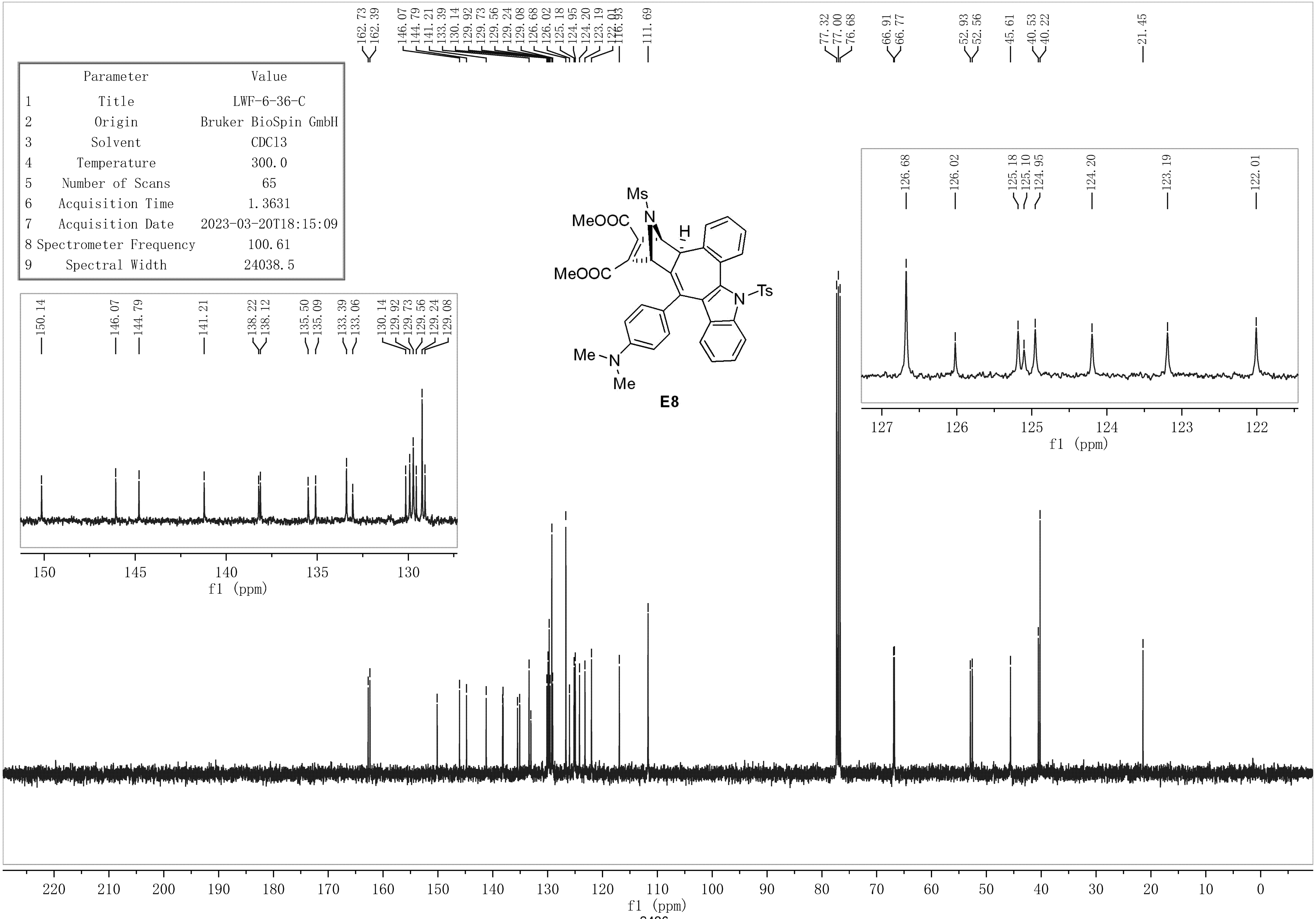

	Parameter	Value
1	Title	LWF-6-36-C
2	Origin	Bruker BioSpin GmbH
3	Solvent	CDCl3
4	Temperature	300.0
5	Number of Scans	65
6	Acquisition Time	1.3631
7	Acquisition Date	2023-03-20T18:15:09
8	Spectrometer Frequency	100.61
9	Spectral Width	24038.5

	Parameter	Value
1	Title	LWF-6-36-DEP
2	Origin	Bruker BioSpin GmbH
3	Solvent	CDCl3
4	Temperature	300.0
5	Number of Scans	25
6	Acquisition Time	1.3631
7	Acquisition Date	2023-03-20T18:19:24
8	Spectrometer Frequency	100.61
9	Spectral Width	24038.5

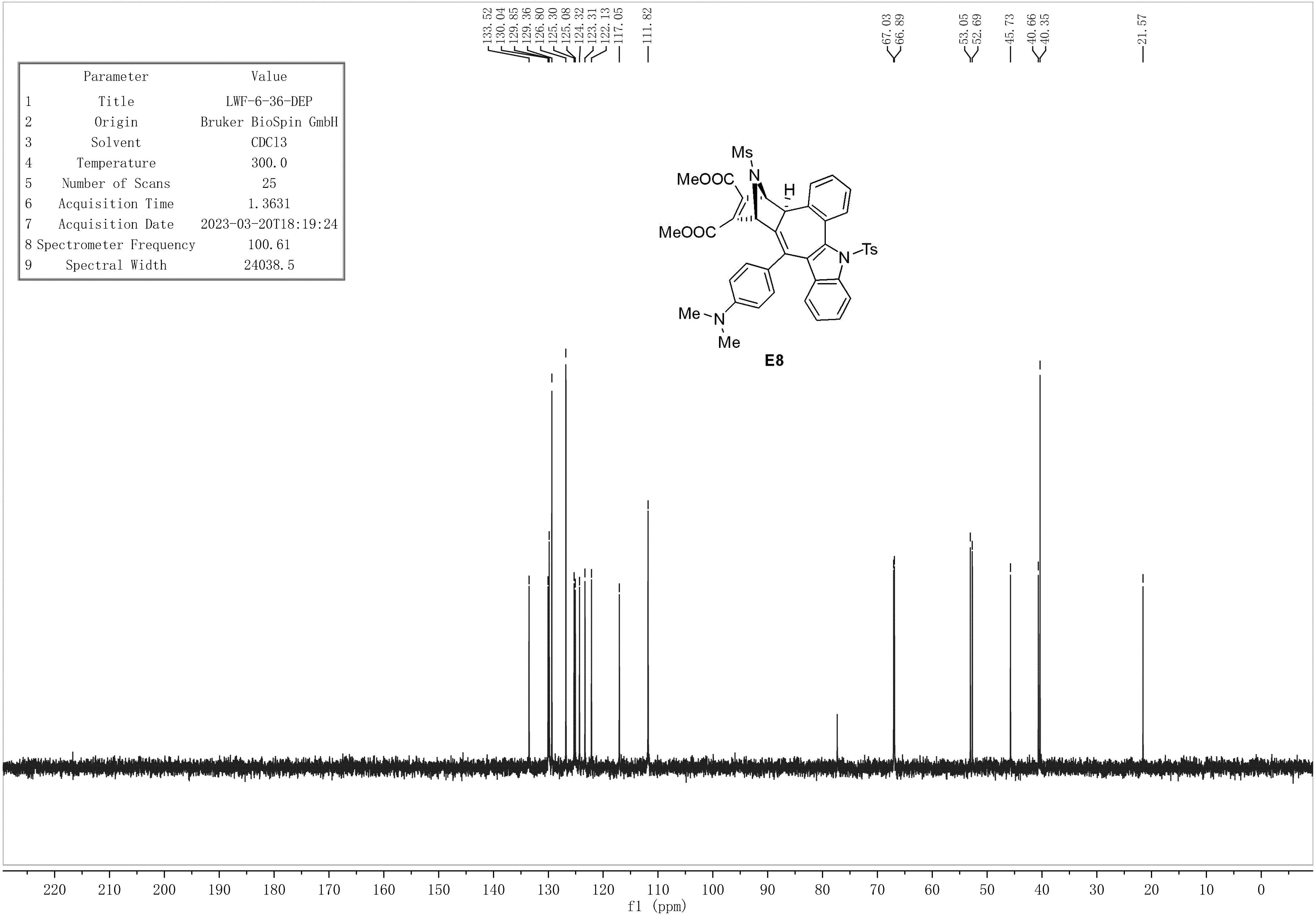

Supplement: Supplementary file 1 [file oc5c00248_si_001.pdf]
