# Supplementary material for: Divergent and Enantioselective Synthesis of Three Types of Chiral Polycyclic N‑Heterocycles via Copper-Catalyzed Dearomative Cyclization
Source: ACS Cent Sci. 2025 May 2;11(5):805–15. doi: 10.1021/acscentsci.5c00248 (PMC12123464; doi:10.1021/acscentsci.5c00248)
Supplement: Supplementary file 2 [file oc5c00248_si_002.pdf]

oc-2025-002489.R1

Name: Peer Review Information for "Divergent and Enantioselective Synthesis of Three Types of Chiral Poly-cyclic N-Heterocycles via Copper-Catalyzed Dearomative Cyclization"

First Round of Reviewer Comments

Reviewer: 1

Comments to the Author

Ye, Li and coworkers present a Cu-catalyzed, highly enantioselective dearomative cyclopropanation of indole-diynes, enabling the stereoselective synthesis of chiral cyclopropanes. The cyclopropane products can be further transformed into 1,2-dioxolane-fused indolines via a [3 + 2] cycloaddition with oxygen. Additionally, Brønsted acid-promoted ring-opening and rearrangement of cyclopropane-fused indolines provide access to chiral cyclohepta[b]indoles. The study is further supported by mechanistic insights from control experiments and theoretical calculations, reinforcing its significance. The Ye group is expertise in copper-catalyzed diyne cyclization and applied similar methodologies to catalytic asymmetric dearomative annulation reactions. The results presented are intriguing and have the potential for publication in ACS Central Science after addressing the following comments.

Comments:

1. All indolyl diyne substrates examined contain an aryl linkage (Ar^1 in Table 3). The reviewer suggests exploring other linkages, such as alkyl chains or alkenes, to see whether these substrates are compatible with the reaction. Additionally, evaluating their impact on enantioselectivity would provide further insights into the scope and limitations of this methodology.

2. One limitation that the authors should comment on is that the aryl substitution at one end of the alkyne group contains electron-donating groups, and only aniline derivatives achieve enantioselectivities above 90% ee. The reviewer suggests conducting DFT calculations to investigate how these electronic effects influence both enantioselectivity and yield.

3. The authors cite an excessive number of their previously published works, particularly references 12a–12l, which all focus on transformations of vinyl groups developed by their group. The reviewer discourages excessive self-citation and recommends citing only the most relevant initial reports wherever possible.

4. The enantioselective dearomative cyclopropanation demonstrates the broadest substrate scope, including various protecting groups on the N-atom of propargyl ynamides. Additionally, this transformation can be successfully extended to other heteroarenes containing oxygen and sulfur. However, not all of these substrates are compatible with the subsequent [3 + 2] cycloaddition with oxygen or the rearrangement of cyclopropane reactions. Therefore, the reviewer encourages the inclusion of more examples of unsuccessful substrates, as these can provide valuable insights and may inspire readers to explore potential solutions.

Reviewer: 2

Comments to the Author

This manuscript describes a copper-catalyzed enantioselective dearomative cyclization of indolyl diynes, yielding cyclopropane-fused indolines and their transformations. While the results are excellent, the substrate scope is limited to a single core structure. Representative natural products containing cyclopropane-fused indoline structures are shown in Figure 1. However, these natural products cannot be synthesized using this new reaction. Consequently, the synthetic utility of the method is not demonstrated in this manuscript. Although the results are interesting, their impact and utility are insufficient for publication in ACS Central Science.

Reviewer: 3

Comments to the Author

In this manuscript, Li and coworkers present a chiral copper-catalyzed asymmetric dearomative cyclopropanation of indole diynes, yielding a diverse array of chiral cyclopropane-fused polycyclic N-heterocycles. The reactions proceed under mild

conditions, delivering products in good to excellent yields with outstanding enantio- and diastereoselectivity. This work represents the first asymmetric dearomative cyclopropanation of indoles using alkynes as carbene precursors and represents the inaugural catalytic asymmetric synthesis of chiral 1,2-dioxolanes with high stereoselectivity. Furthermore, Brønsted acid-promoted ring-opening and rearrangement of cyclopropane-fused indolines exhibit remarkable chemoselectivity, enabling the efficient and enantiocontrolled synthesis of chiral cyclohepta indoles. Control experiments and theoretical calculations provide insights into the reaction pathways and the origin of enantiocontrol.

This review considers the work highly suitable for publication in ACS Central Science after addressing the following points:

1. Stability of Compound B: Can compound B remain stable under ambient conditions? If not, what methods are employed for its separation and purification?

2. Substrate Scope: In Table 2, the R groups on substrate A are exclusively electron-donating. How does the reaction perform with electron-withdrawing groups, such as halogens, trifluoromethyl, or nitro groups?

3. Selectivity in Bond Cleavage: In the formation of products C and D, different C–C bonds in the cyclopropane moiety are cleaved under varying conditions. What factors govern this selectivity?

4. HRMS Analysis: For halogen-containing compounds (Cl or Br), the supporting information should include identification of the corresponding high-resolution mass spectrometry (HRMS) isotopic peaks.

5. Catalyst Loading: How do the reactivity and enantioselectivity of the reaction change when the catalyst loading is reduced?

Reviewer: 4

Comments to the Author

Li and co-workers report a tandem reaction of indole diynes under Cu catalysis that results in the generation of cyclopropanated indoles with loss of aromaticity. Because the lower

aromaticity of the indole, several studies have employed indoles in catalytic asymmetric dearomatization procedures.

Table 1 shows catalyst optimization, which finds ligand L3 the most enantioselective. Can the authors comment on the role of the sodium tetrakis[3,5-bis(trifluoromethyl)phenyl]borate? Why is it needed and how important is it for catalyst enantioselectivity? The scope of the cyclopropanes is good, with most examples shown giving high enantioselectivities. The reactions with substrates other than indoles is a very nice addition to the manuscript. In table 3, they explore the reaction with dioxygen to make 1,2-dioxolane-fused indolines. This transformation of the cyclopropanes is proposed to be catalyzed by the Cu catalyst that promotes the cyclopropanation. The compounds are made with very high ee and dr. Further, the reaction is interesting. There is an R group in the substrate, but not in the products. Next, in Table 4 is a protonation of the cyclopropane followed by ring expansion/aryl migration. This reaction can be done in tandem with the cyclopropanation reaction, making it convenient. Here again the products are formed with high enantioselectivities.

Since there have been several examples of catalytic asymmetric dearomatization of indoles, is this sentence really true? “ However, the asymmetric dearomative cyclopropanation of indoles continues to pose a formidable challenge”. I think not.

Minor points. Don’t rotate the structures. Pick an orientation and use that orientation all the way through the scheme. Look, for example, at Table 4, where the structure is turned or flipped every time it is drawn. Please fix all these issues. Also, it might be easier for the reader if the key intermediate in the box in Scheme 1c is shown in the optimization table as a possible intermediate. It didn’t really make sense to me in its current position.

Scheme 1 is crowded and convoluted. I do not understand the significance of the second arrow pointing down in Scheme 1B. What is the starting material?

The authors state: “we took the initiative to achieve the catalytic asymmetric synthesis of 1,2-dioxolanes through the strain release of chiral cyclopropanes.” And “…first catalytic asymmetric synthesis of chiral 1,2-dioxolanes with high diastereoselectivity and enantiopurity.” This is a nice transformation. Is the formation of the dioxolane really catalytic? This is not discussed until several pages later, leaving the reader wondering.

This sentence does not make sense to me: Significantly, this reaction was also successfully conducted in DCE with O2 atmosphere without necessitating a one-pot operation to produce chiral C1 in 65% yield and 97% ee.

Something to think about: Racemic compounds are chiral. Enantioenriched compounds possess more of one enantiomer than the other. Chiral is use frequently in this manuscript, where enantioenriched should be used. Also, if a compound has an ee, you don't need to say it is chiral because it must be.

"via a remote stereocontrol strategy". What is this? Just normal asymmetric catalysis.

"Inspired by our recent studies on the chiral N-heterocycle synthesis from ynamides13

and…". Is this the correct reference? Doesn't seem like it. I am not sure what their past work is due to lack of clarity.

SI: The procedures are written clearly. Some of the NMR spectra need inserts with expansions so that the peaks are tall enough so one can actually see what they are and determine if there are impurities. Please check the NMRs again. The worst on is B28 proton, B29 proton, B30, etc. There are a lot, so pleased check all the compounds and add expansions to the aromatic region of the 1H NMR to make the spectra publication quality. This problem starts on page S5, where the spectra is too small to read and there is not figure number. In the scheme on page 8 of the SI, it looks like the compounds are 2 or 3 substituted because of the way the R3 substituent is drawn. Please clarify why this is done. Are the starting materials mixtures? Consider using schemes that are specific for the compounds used (indole vs. benzofuran).

Overall, the strengths of this paper is novel chemistry with several new types of indoline based compounds prepared with high enantioselectivity. This will be of broad interest to the chemical community. The chemistry is mechanistically interesting, building on the theme of vinyl cations in cyclopropanation processes. The weaknesses are the writing needs some work, as indicated, and the synthesis of the starting materials is laborious.

The SI needs a lot of minor edits. I recommend that this work could be acceptable after the changes noted are made.

Author's Response to Peer Review Comments:

WENZHOU UNIVERSITY

Wenzhou, Zhejiang, China

March 10, 2025

Dear Editor-in-Chief, Dr. **Carolyn R. Bertozzi**

Thanks very much for offering us an opportunity to revise our manuscript (oc-2025-002489). We also thank the reviewers for their kind comments and recommendation. In particular, we thank the reviewers for pointing out several important issues for us to examine, which greatly benefits our research work and makes our work more convincing. Here are my responses to those comments by the reviewer **point-by-point** and the changes made to the manuscript (revised portions are marked with a yellow background in the paper).

Responses to editor's comments are detailed:

Formatting Needs:

1：Response to comment: References: Number references individually, with only one citation per reference. Do not group references.

- We thank the editor very much for the valuable suggestions and nice comments. We have made these corrections in the manuscript.

2：Response to comment: Synopsis: ACS Central Science requires a brief synopsis. The synopsis should be no more than 200 characters (including spaces) and should reasonably correlate with the Table of Contents (TOC) graphic. The synopsis is intended to explain the importance of the article to a broader readership across the sciences. Please place your synopsis in the manuscript file after the TOC graphic.

- The synopsis has been added in the manuscript.

Responses to reviewers' comments are detailed:

Reviewer(s)' Comments to Author:

Reviewer: 1

Recommendation: Publish in ACS Central Science after minor revisions noted.

Comments:

Ye, Li and coworkers present a Cu-catalyzed, highly enantioselective dearomative cyclopropanation of indole-diynes, enabling the stereoselective synthesis of chiral cyclopropanes. The cyclopropane products can be further transformed into 1,2-dioxolane-fused indolines via a [3 + 2] cycloaddition with oxygen. Additionally, Brønsted acid-promoted ring-opening and rearrangement of cyclopropane-fused indolines provide access to chiral cyclohepta[b]indoles. The study is further supported by mechanistic insights from control experiments and theoretical calculations, reinforcing its significance. The Ye group is expertise in copper-catalyzed diyne cyclization and applied similar methodologies to catalytic asymmetric dearomative annulation reactions. The results presented are intriguing and have the potential for publication in ACS Central Science after addressing the following comments.

1： Response to comment: All indolyl diyne substrates examined contain an aryl linkage (Ar^1 in Table 3). The reviewer suggests exploring other linkages, such as alkyl chains or alkenes, to see whether these substrates are compatible with the reaction. Additionally, evaluating their impact on enantioselectivity would provide further insights into the scope and limitations of this methodology.

- We first thank the reviewer very much for the valuable suggestions and nice comments. Actually, we had tried many methods to prepare other substrates with different linkages such as **SA1**, **SA2**, and **SA3**, but unfortunately, all our efforts failed (Scheme S1).

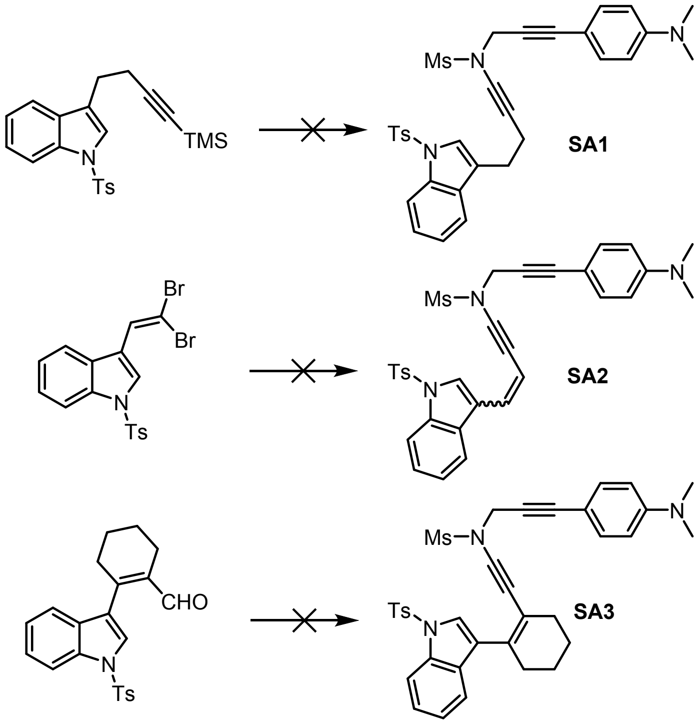

Scheme S1

2： Response to comment: One limitation that the authors should comment on is that the aryl substitution at one end of the alkyne group contains electron-donating groups, and only aniline derivatives achieve enantioselectivities above 90% ee. The reviewer suggests conducting DFT calculations to investigate how these electronic effects influence both enantioselectivity and yield.

- Indeed, the electron-donating groups on the aryl ring play a key role to initiate this reaction. As illustrated in Scheme S2A, chiral vinyl cation intermediate is generated through intramolecular attack of the alkyne on the copper-activated ynamide. The alkyne, functioning as a nucleophile, needs to be nucleophilic enough to effectively initiate this reaction. Conversely, when the alkyne is associated with an electron-withdrawing group, the reaction becomes significantly less favorable and requires elevated temperatures for initiation. As a result of the thermal conditions required, the enantioselectivity of the reaction is lower.
- Substrates **SA4** and **SA5** were subjected to this reaction, however, the reaction was not triggered even under heating conditions (Scheme S2B).

- Finally, similar results have been observed in the related previous studies (see Refs 43-47). Based on the aforementioned results, it seems that additional elucidation via Density Functional Theory (DFT) calculations may not be necessary.

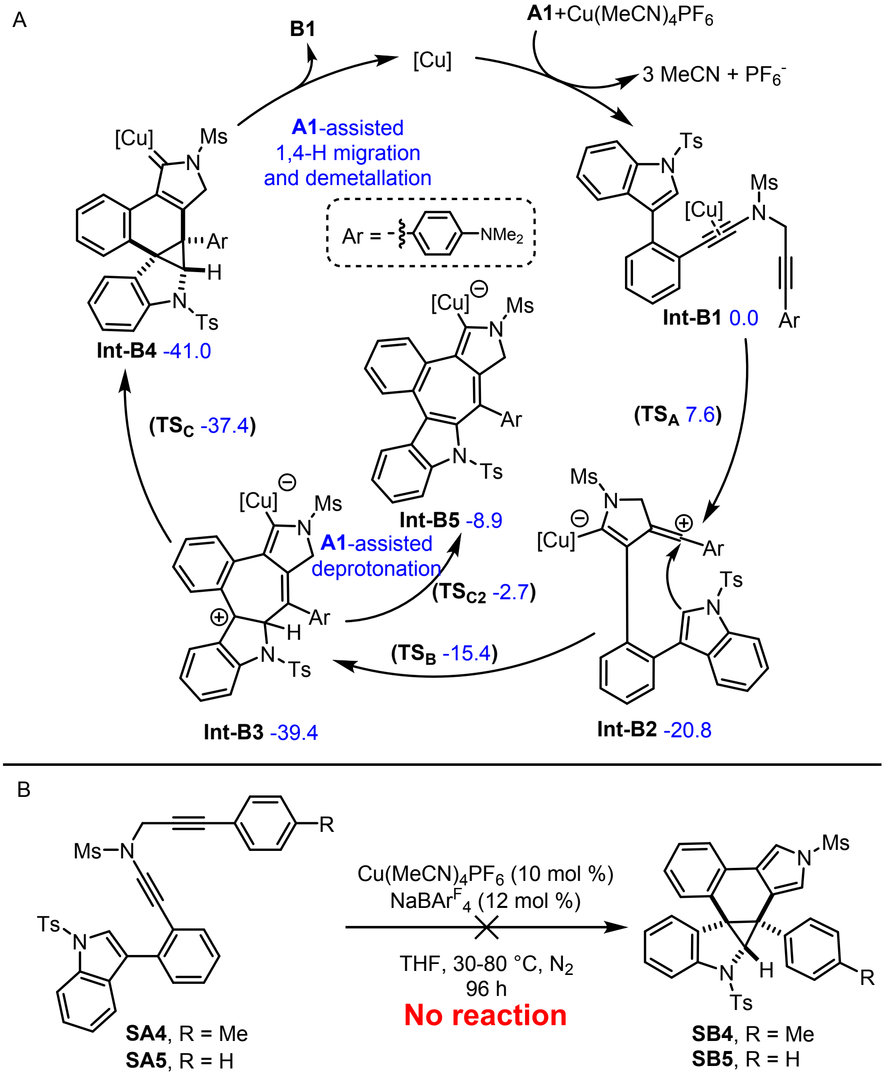

Scheme S2

3： Response to comment: The authors cite an excessive number of their previously published works, particularly references 12a–12l, which all focus on transformations of vinyl groups developed by their group. The reviewer discourages excessive self-citation and recommends citing only the most relevant initial reports wherever possible.

- As suggested, we have deleted more than half of these literatures and only cited the most relevant reports in the manuscript.

4： Response to comment: The enantioselective dearomative cyclopropanation demonstrates the broadest substrate scope, including various protecting groups on the N-atom of propargyl ynamides. Additionally, this transformation can be successfully extended to other heteroarenes containing oxygen and sulfur. However, not all of these substrates are compatible with the

subsequent [3 + 2] cycloaddition with oxygen or the rearrangement of cyclopropane reactions. Therefore, the reviewer encourages the inclusion of more examples of unsuccessful substrates, as these can provide valuable insights and may inspire readers to explore potential solutions.

- As suggested, some examples of the unsuccessful substrates have been added in the revised manuscript.

Additional Questions:
Quality of experimental data, technical rigor: Top 5%
Significance to chemistry researchers in this and related fields: High
Broad interest to other researchers: Top 5%
Novelty: High
Is this research study suitable for media coverage or a First Reactions (a News & Views piece in the journal)?: No

Reviewer: 2

Recommendation: Does not meet the requirements of publishing in ACS Central Science.

Comments:
This manuscript describes a copper-catalyzed enantioselective dearomative cyclization of indolyl diynes, yielding cyclopropane-fused indolines and their transformations. While the results are excellent, the substrate scope is limited to a single core structure. Representative natural products containing cyclopropane-fused indoline structures are shown in Figure 1. However, these natural products cannot be synthesized using this new reaction. Consequently, the synthetic utility of the method is not demonstrated in this manuscript. Although the results are interesting, their impact and utility are insufficient for publication in ACS Central Science.

Additional Questions:
Quality of experimental data, technical rigor: High
Significance to chemistry researchers in this and related fields: Moderate
Broad interest to other researchers: Moderate
Novelty: Moderate
Is this research study suitable for media coverage or a First Reactions (a News & Views piece in the journal)?: No

- We totally disagree with the reviewer’s above comments. Indeed, we have not been able to synthesize natural products through this asymmetric Cu-catalyzed indole-diynes cyclization, but this method is very meaningful and provides an important platform for the synthesis of a variety pf valuable chiral N-teterocycles. In this manuscript, we disclose a Cu-catalyzed asymmetric dearomative cyclopropanation of indole-diynes and subsequent [3 + 2] cycloaddition with oxygen, facilitating the divergent and atom-economical synthesis of chiral cyclopropane- and 1,2-dioxolane-fused indolines with moderate to excellent yields and

generally outstanding diastereo-, and enantioselectivities with broad substrate scope. Importantly, this protocol not only represents the first asymmetric dearomative cyclopropanation of indoles utilizing alkynes as carbene precursors, but also constitutes the first catalytic asymmetric construction of chiral 1,2-dioxolanes with high stereoselectivity. Interestingly, Brønsted acid-promoted ring-opening and rearrangement of cyclopropane-fused indolines display distinctive chemoselectivity to afford chiral cyclohepta[*b*]indoles in good to excellent efficiency and enantiocontrol. **As highly recommended by other reviewers (reviewers 1, 3, and 4), this work's novelty is enough to publish in *ACS Central Science* after minor revisions.**

Reviewer: 3
Recommendation: Publish in ACS Central Science after minor revisions noted.

Comments:
In this manuscript, Li and coworkers present a chiral copper-catalyzed asymmetric dearomative cyclopropanation of indole diynes, yielding a diverse array of chiral cyclopropane-fused polycyclic N-heterocycles. The reactions proceed under mild conditions, delivering products in good to excellent yields with outstanding enantio- and diastereoselectivity. This work represents the first asymmetric dearomative cyclopropanation of indoles using alkynes as carbene precursors and represents the inaugural catalytic asymmetric synthesis of chiral 1,2-dioxolanes with high stereoselectivity. Furthermore, Brønsted acid-promoted ring-opening and rearrangement of cyclopropane-fused indolines exhibit remarkable chemoselectivity, enabling the efficient and enantiocontrolled synthesis of chiral cyclohepta indoles. Control experiments and theoretical calculations provide insights into the reaction pathways and the origin of enantiocontrol.
This review considers the work highly suitable for publication in ACS Central Science after addressing the following points:

5： Response to comment: Stability of Compound B: Can compound B remain stable under ambient conditions? If not, what methods are employed for its separation and purification?

- We acknowledge the constructive comments from reviewer 3. Compound **B** is easily isolated and purified by conventional column chromatography (see Supporting Information) and very stable under ambient conditions.

6： Response to comment: Substrate Scope: In Table 2, the R groups on substrate A are exclusively electron-donating. How does the reaction perform with electron-withdrawing groups, such as halogens, trifluoromethyl, or nitro groups?

- Actually, substrates **SA4** and **SA5** were introduced to performed this reaction, however, this reaction was not triggered under heating condition (Scheme S2B). As shown in Scheme S2A, the alkyne, functioning as a nucleophile, needs to be nucleophilic enough to effectively initiate this reaction. Conversely, when the alkyne is associated with an electron-withdrawing

group, the reaction becomes significantly less favorable. Please also see entry 2 (Response to reviewer 1) for details.

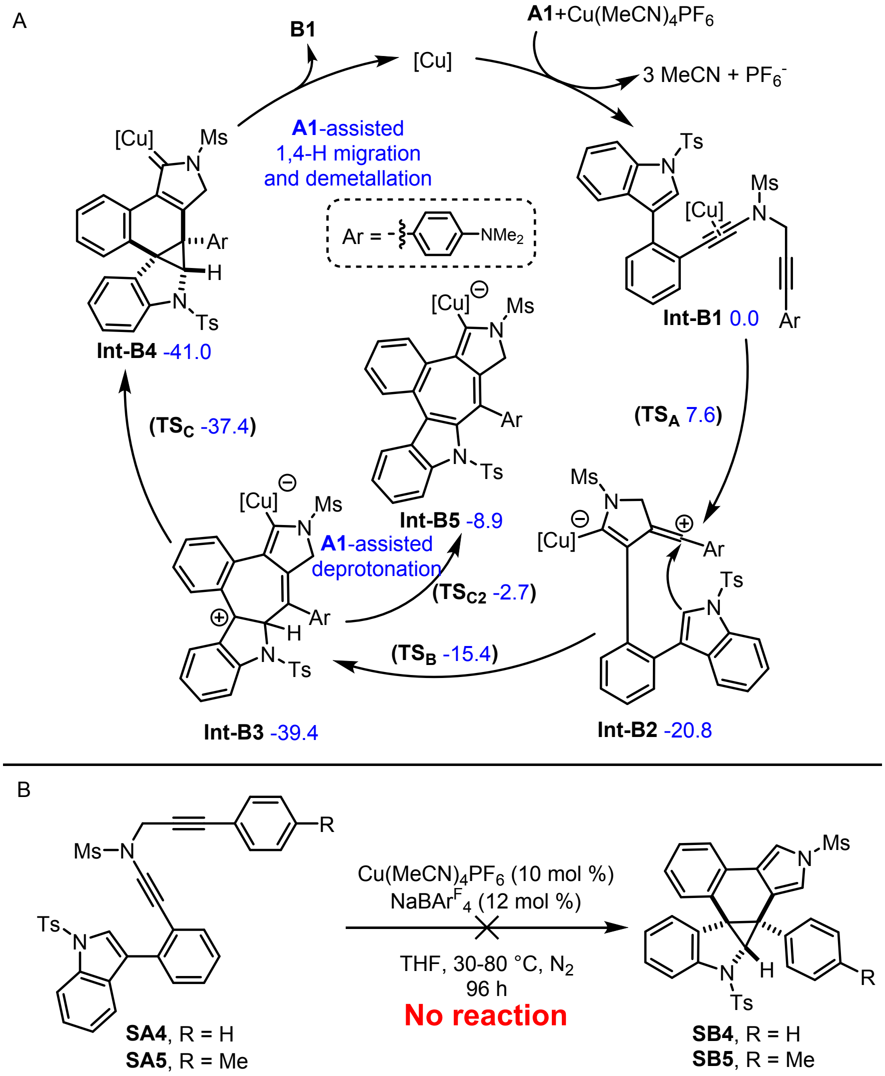

Scheme S2

7： Response to comment: Selectivity in Bond Cleavage: In the formation of products C and D, different C–C bonds in the cyclopropane moiety are cleaved under varying conditions. What factors govern this selectivity?

- As depicted in Scheme S3A, we suspect that intermediate **Int-C1′** was unfavorable to react with O_2 due to the effect of coordination from SO_2 group. In the acidic system, selective C-C bond cleavage is achieved, likely due to the driving force for subsequent migration steps (Scheme S3A). In fact, the mechanism of selective C–C bond cleavage remains unclear, and further studies in this direction are still ongoing.

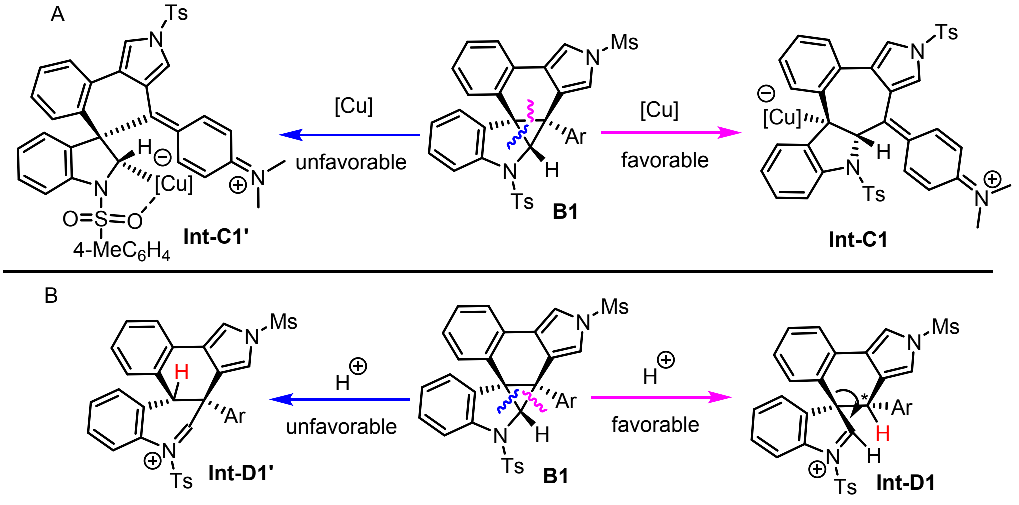

Scheme S3

8：Response to comment: HRMS Analysis: For halogen-containing compounds (Cl or Br), the supporting information should include identification of the corresponding high-resolution mass spectrometry (HRMS) isotopic peaks.

- As suggested, the corresponding high-resolution mass spectrometry (HRMS) isotopic peaks have been added in the Supporting Information.

9：Response to comment: Catalyst Loading: How do the reactivity and enantioselectivity of the reaction change when the catalyst loading is reduced?

- In the presence of 5 mol % catalyst, the reaction time was greatly extended from the original 24 hours to 80 hours, but the yield and enantioselectivity of the reaction remained unchanged (Scheme S4).

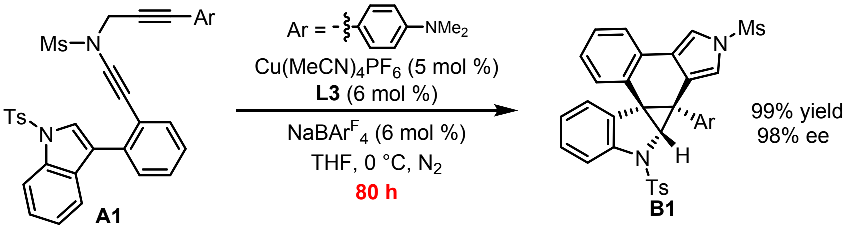

Scheme S4

Additional Questions:
Quality of experimental data, technical rigor: High
Significance to chemistry researchers in this and related fields: Top 5%
Broad interest to other researchers: Top 5%
Novelty: High
Is this research study suitable for media coverage or a First Reactions (a News & Views piece in the journal)?: Yes

Reviewer: 4

Recommendation: Publish in ACS Central Science after minor revisions noted.

Comments:
Li and co-workers report a tandem reaction of indole diynes under Cu catalysis that results in the generation of cyclopropanated indoles with loss of aromaticity. Because the lower aromaticity of the indole, several studies have employed indoles in catalytic asymmetric dearomatization procedures. Table 1 shows catalyst optimization, which finds ligand L3 the most enantioselective. Can the authors comment on the role of the sodium tetrakis[3,5-bis(trifluoromethyl)phenyl]borate? Why is it needed and how important is it for catalyst enantioselectivity? The scope of the cyclopropanes is good, with most examples shown giving high enantioselectivities. The reactions with substrates other than indoles is a very nice addition to the manuscript. In table 3, they explore the reaction with dioxygen to make 1,2-dioxolane-fused indolines. This transformation of the cyclopropanes is proposed to be catalyzed by the Cu catalyst that promotes the cyclopropanation. The compounds are made with very high ee and dr. Further, the reaction is interesting. There is an R group in the substrate, but not in the products. Next, in Table 4 is a protonation of the cyclopropane followed by ring expansion/aryl migration. This reaction can be done in tandem with the cyclopropanation reaction, making it convenient. Here again the products are formed with high enantioselectivities.

10：Response to comment: Table 1 shows catalyst optimization, which finds ligand L3 the most enantioselective. Can the authors comment on the role of the sodium tetrakis[3,5-bis(trifluoromethyl)phenyl]borate? Why is it needed and how important is it for catalyst enantioselectivity?

- We thank the reviewer very much for the valuable suggestions and nice comments. When the reaction was conducted in the absence of sodium tetrakis[3,5-bis(trifluoromethyl)phenyl]borate, the yield remained satisfactory but the enantioselectivity decreased slightly (Scheme S5). Actually, $NaBAr^{F}_{4}$ has been widely used as additive in asymmetric transition metal catalysis to enhance the acidity and/or solubility of metal catalysts, and thus leading to significantly improved reactivity and selectivity. Sodium salt here may increase the steric effect of chiral copper catalysis, thus improving the enantioselectivity of the reaction.

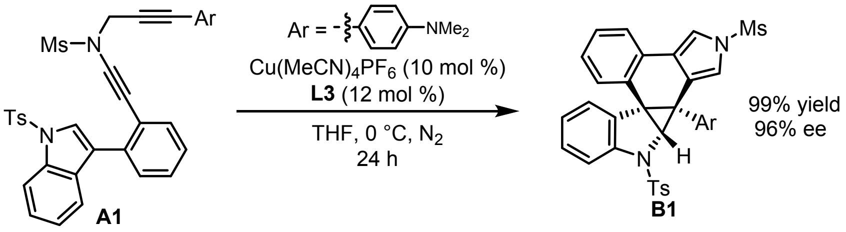

Scheme S5

11： Response to comment: Since there have been several examples of catalytic asymmetric dearomatization of indoles, is this sentence really true? “ However, the asymmetric dearomative cyclopropanation of indoles continues to pose a formidable challenge”. I think not.

- In fact, successful examples of asymmetric dearomative cyclopropanation of indoles are still very scarce (**only 4 literatures**), and most have been constrained by reliance on the use of the high-energy, potentially explosive diazo compounds. Moreover, integrating safety with atom economy in catalytic asymmetric dearomative cyclopropanation of indoles with high efficacy and enantiopurity, especially based on the alkynes as precursors, is still under-explored and extremely challenging. So, I think the sentence “the asymmetric dearomative cyclopropanation of indoles continues to pose a formidable challenge” is reasonable.

12： Response to comment: Minor points. Don’t rotate the structures. Pick an orientation and use that orientation all the way through the scheme. Look, for example, at Table 4, where the structure is turned or flipped every time it is drawn. Please fix all these issues. Also, it might be easier for the reader if the key intermediate in the box in Scheme 1c is shown in the optimization table as a possible intermediate. It didn’t really make sense to me in its current position.

- As suggested, we have made these corrections.

13： Response to comment: Scheme 1 is crowded and convoluted. I do not understand the significance of the second arrow pointing down in Scheme 1B. What is the starting material?

- As suggested, the second arrow pointing down in Scheme 1B has been removed. We also tried to further optimize Scheme 1, unfortunately we didn't find a better solution.

14： Response to comment: The authors state: “we took the initiative to achieve the catalytic asymmetric synthesis of 1,2-dioxolanes through the strain release of chiral cyclopropanes.” And “…first catalytic asymmetric synthesis of chiral 1,2-dioxolanes with high diastereoselectivity and enantiopurity.” This is a nice transformation. Is the formation of the dioxolane really catalytic? This is not discussed until several pages later, leaving the reader wondering.

- Actually, the formation of the dioxolane is catalytic by employing Cu-catalyst. We have improved the sentence “Encouraged by López’ and Vicente’s findings (Scheme 1B left bottom),[38] oxygen was directly introduced to perform [3 + 2] cycloaddition with **B1** by replacing nitrogen into oxygen atmosphere for the one-pot synthesis of chiral 1,2-dioxolane **C1** by employing Cu-catalyst (see Supporting Information).”

15： Response to comment: This sentence does not make sense to me: Significantly, this reaction was also successfully conducted in DCE with O2 atmosphere without necessitating a one-pot operation to produce chiral C1 in 65% yield and 97% ee.

- This sentence has been corrected into "Significantly, this reaction was also directly conducted in DCE with O_2 atmosphere to produce chiral **C1** in 65% yield and 97% ee without a complex one-pot operation."

16： Response to comment: Something to think about: Racemic compounds are chiral. Enantioenriched compounds possess more of one enantiomer than the other. Chiral is use frequently in this manuscript, where enantioenriched should be used. Also, if a compound has an ee, you don't need to say it is chiral because it must be.

- As suggested, we have made these corrections.

16： Response to comment: "via a remote stereocontrol strategy". What is this? Just normal asymmetric catalysis.

- As shown Scheme S6, the chiral production site is located four atoms away from the chiral copper center. I think the statement "via a remote stereocontrol strategy" is reasonable.

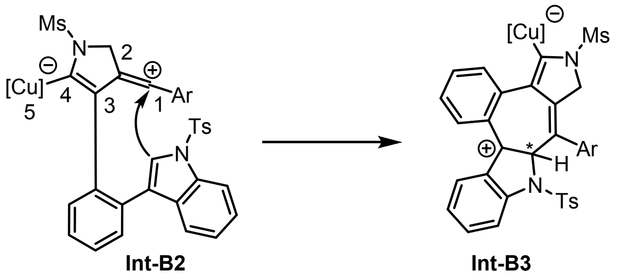

Scheme S6

16： Response to comment: "Inspired by our recent studies on the chiral N-heterocycle synthesis from ynamides13 and…". Is this the correct reference? Doesn't seem like it. I am not sure what their past work is due to lack of clarity.

- Actually, these references (48-51) are recent review papers on the ynamide chemistry.

17：Response to comment: SI: The procedures are written clearly. Some of the NMR spectra need inserts with expansions so that the peaks are tall enough so one can actually see what they are and determine if there are impurities. Please check the NMRs again. The worst on is B28 proton, B29 proton, B30, etc. There are a lot, so pleased check all the compounds and add expansions to the aromatic region of the 1H NMR to make the spectra publication quality. This problem starts on page S5, where the spectra is too small to read and there is not figure number. In the scheme on page 8 of the SI, it looks like the compounds are 2 or 3 substituted because of the way the R3 substituent is drawn. Please clarify why this is done. Are the starting materials mixtures? Consider using schemes that are specific for the compounds used (indole vs. benzofuran).

- As suggested, we have made the related modifications in the Supporting Information. It should be emphasized that all of our product spectra are very clean and error-free. The broad peaks of some compounds in the H NMR spectra may be caused by the steric resistance of the compound structure.

17：Response to comment: Overall, the strengths of this paper is novel chemistry with several new types of indoline based compounds prepared with high enantioselectivity. This will be of broad interest to the chemical community. The chemistry is mechanistically interesting, building on the theme of vinyl cations in cyclopropanation processes. The weaknesses are the writing needs some work, as indicated, and the synthesis of the starting materials is laborious. The SI needs a lot of minor edits. I recommend that this work could be acceptable after the changes noted are made.

- Again, we thank the reviewer very much for the kind recommendation and valuable suggestions. We have made all the above corrections in the manuscript and Supporting Information.

I hope my responses to the reviewers' comments as well as all the changes made to the manuscript are satisfactory to your office. My students have worked tirelessly on this chemistry and put so many late hours to finish the original manuscript and later to significantly improve the manuscript to satisfy the high level of *ACS Central Sci.* article. They truly deserve to have the chance.

Thank you very much.
Sincerely yours,

Professor Long Li
College of Chemistry and Materials Engineering, Wenzhou University
Telephone: 18790763089
Email: longwzu1990@wzu.edu.cn

Updated NMR of substates including SA4 and SA5, see as follow:

***N*-(3-phenylprop-2-yn-1-yl)-*N*-((2-(1-tosyl-1*H*-indol-3-yl)phenyl)ethynyl)methanesulfonamide (SA4)**

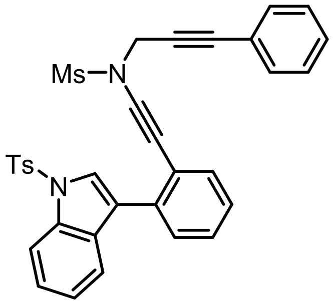

SA4

Compound **SA4** was prepared in 62% yield (370.1 mg) with four steps according to the general procedure as a white solid (mp 102–105 °C). ^{1}H NMR (400 MHz, $CDCl_3$) δ 7.98 (d, *J* = 8.4 Hz, 1H), 7.86 (s, 1H), 7.80 (d, *J* = 8.4 Hz, 2H), 7.65 – 7.56 (m, 2H), 7.47 (d, *J* = 6.8 Hz, 1H), 7.40 – 7.36 (m, 1H), 7.35 – 7.25 (m, 7H), 7.24 – 7.15 (m, 3H), 4.32 (s, 2H), 2.77 (s, 3H), 2.33 (s, 3H); ^{13}C NMR (100 MHz, $CDCl_3$) δ 145.1, 135.1, 134.8, 134.4, 133.2, 131.8, 130.0, 129.84, 129.76, 128.9, 128.4, 128.3, 127.5, 126.9, 124.9, 124.7, 123.3, 122.2, 121.7, 121.1, 113.6, 86.9, 84.8, 81.4, 70.8, 42.6, 38.5, 21.6; HRESIMS Calcd for $[C_{33}H_{26}N_2NaO_4S_2]^+$ (M + Na^+) 601.1226, found 601.1225.

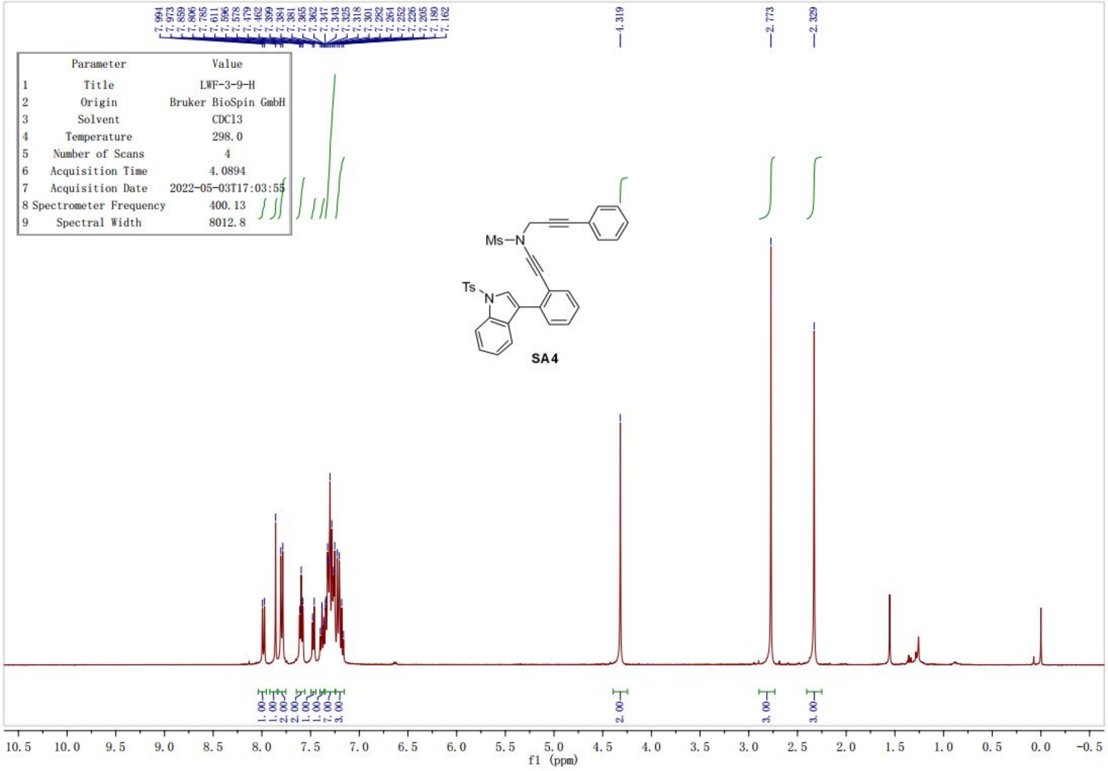
Parameter | Value
1 Title LWF-3-9-H
2 Origin Bruker BioSpin GmbH
3 Solvent CDCl3
4 Temperature 298.0
5 Number of Scans 4
6 Acquisition Time 4.0894
7 Acquisition Date 2022-05-03T17:03:55
8 Spectrometer Frequency 400.13
9 Spectral Width 8012.8
Ms
N
Ts
SA4
4.319
2.773
2.329
f1 (ppm)

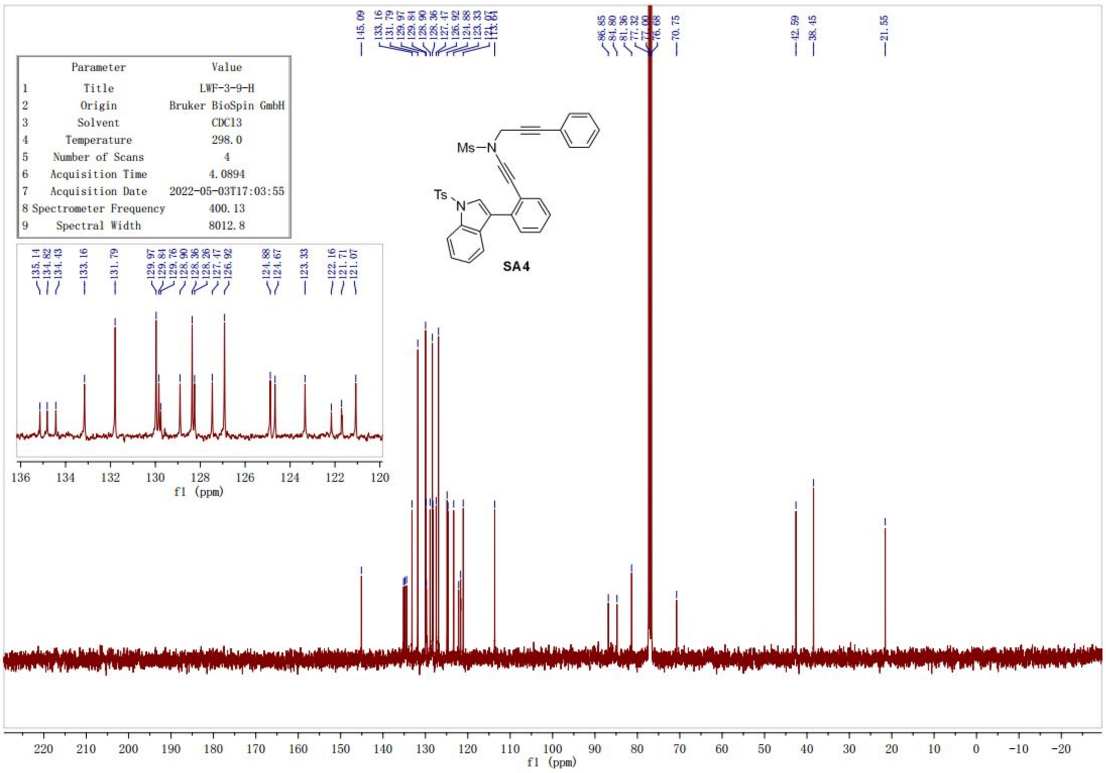
Parameter | Value
1 Title LWF-3-9-H
2 Origin Bruker BioSpin GmbH
3 Solvent CDCl3
4 Temperature 298.0
5 Number of Scans 4
6 Acquisition Time 4.0894
7 Acquisition Date 2022-05-03T17:03:55
8 Spectrometer Frequency 400.13
9 Spectral Width 8012.8
Ms
N
Ts
SA4
145.09
70.75
42.59
38.45
21.55
f1 (ppm)

_N_-(3-(p-tolyl)prop-2-yn-1-yl)-_N_-((2-(1-tosyl-1_H_-indol-3-yl)phenyl)ethynyl)methanesulfonamide (SA5)

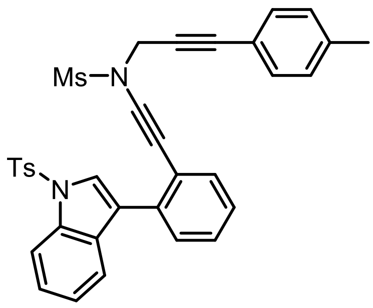

SA5

Compound **SA5** was prepared in 43% yield (498.0 mg) with four steps according to the general procedure as a white solid (mp 109–111 °C). ^{1}H NMR (400 MHz, $CDCl_3$) δ 7.98 (d, J = 8.4 Hz, 1H), 7.85 (s, 1H), 7.79 (d, J = 8.4 Hz, 2H), 7.64 – 7.55 (m, 2H), 7.47 (d, J = 6.8 Hz, 1H), 7.40 – 7.35 (m, 1H), 7.34 – 7.30 (m, 1H), 7.29 – 7.16 (m, 6H), 7.07 (d, J = 8.0 Hz, 2H), 4.30 (s, 2H), 2.75 (s, 3H), 2.33 (s, 3H), 2.32 (s, 3H); ^{13}C NMR (100 MHz, $CDCl_3$) δ 145.1, 139.1, 135.2, 134.8, 134.5, 133.2, 131.7, 130.0, 129.9, 129.8, 129.1, 128.2, 127.5, 126.9, 124.9, 124.6, 123.3, 122.2, 121.8, 121.1, 118.6, 113.6, 87.1, 84.9, 80.7, 70.7, 42.7, 38.4, 21.5, 21.5; HRESIMS Calcd for $[C_{34}H_{28}N_2NaO_4S_2]^+$ (M + Na^+) 615.1383, found 615.1380.

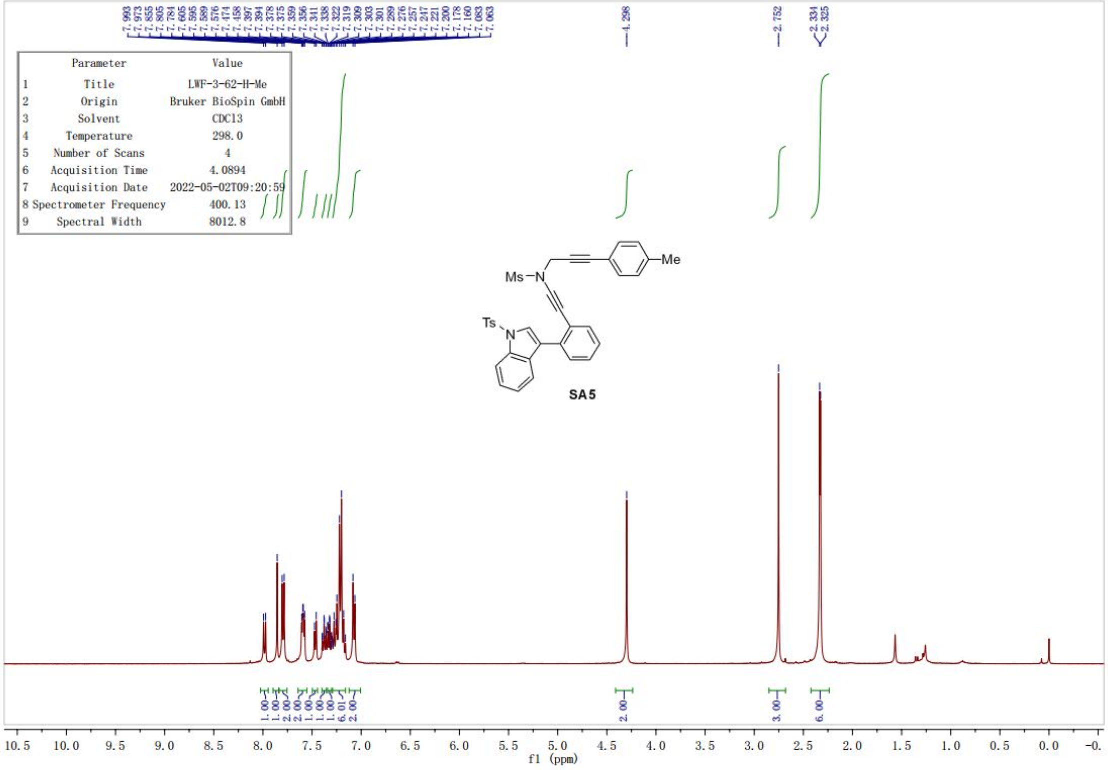
Parameter | Value
1 Title | LWF-3-62-H-Me
2 Origin | Bruker BioSpin GmbH
3 Solvent | CDCl3
4 Temperature | 298.0
5 Number of Scans | 4
6 Acquisition Time | 4.0894
7 Acquisition Date | 2022-05-02T09:20:59
8 Spectrometer Frequency | 400.13
9 Spectral Width | 8012.8
Ms
N
Me
Ts
SA5
4.298
2.752
2.334
2.325
f1 (ppm)

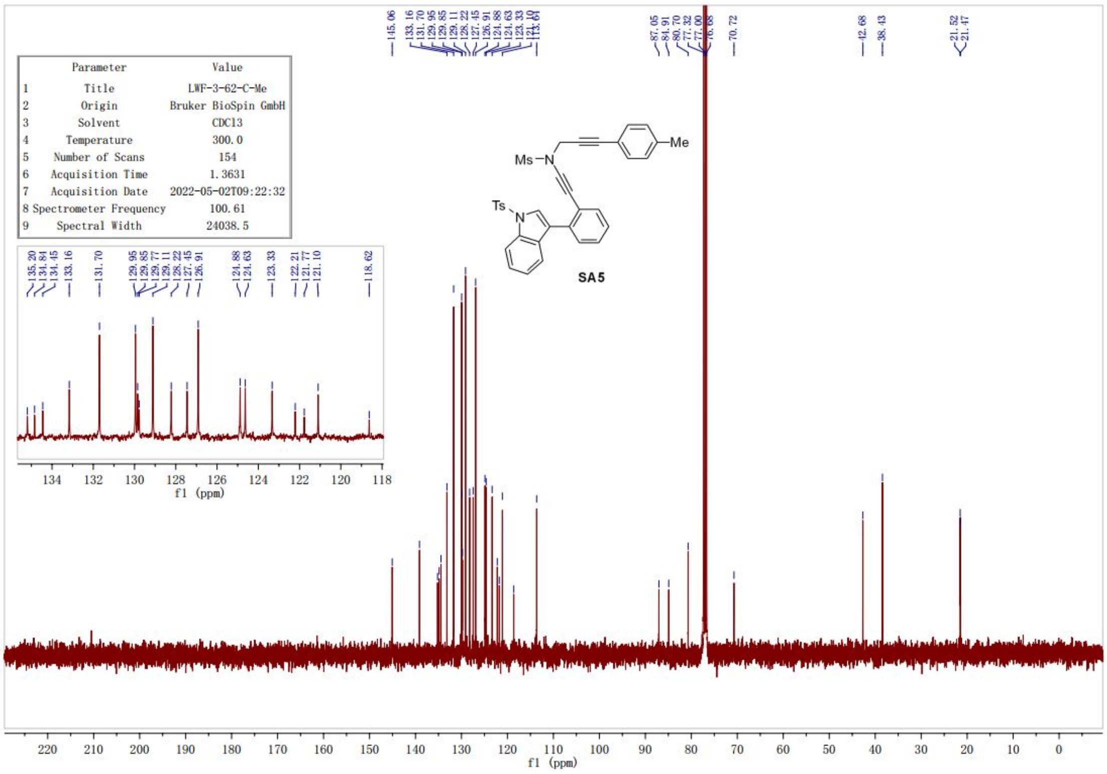
Parameter | Value
1 Title | LWF-3-62-C-Me
2 Origin | Bruker BioSpin GmbH
3 Solvent | CDCl3
4 Temperature | 300.0
5 Number of Scans | 154
6 Acquisition Time | 1.3631
7 Acquisition Date | 2022-05-02T09:22:32
8 Spectrometer Frequency | 100.61
9 Spectral Width | 24038.5
Ms
N
Me
Ts
SA5
145.06
87.05
84.91
80.70
77.32
77.00
76.68
70.72
42.68
38.43
21.52
21.47
f1 (ppm)

oc-2025-002489.R2

Name: Peer Review Information for "Divergent and Enantioselective Synthesis of Three Types of Chiral Polycyclic N-Heterocycles via Copper-Catalyzed Dearomative Cyclization"

Second Round of Reviewer Comments

Reviewer: 3

Comments to the Author

I have reviewed the original submission of this manuscript. The authors carefully revised it according to comments and suggestions by the reviewers. Now, the revised version is suitable for publication in ACS Central Science. Additional revision and review process are not necessary.

Reviewer: 1

Comments to the Author

The authors have adequately addressed all comments from the reviewer. I recommend publishing the manuscript in its current form.

Reviewer: 2

Comments to the Author

This reviewer completely disagrees with the authors' comments. Since the authors acknowledge that they have not been able to synthesize natural products through the asymmetric Cu-catalyzed cyclization of indole-diynes, Figure 1 should be omitted to avoid misleading readers. Consequently, the synthetic utility of the method is not sufficiently

demonstrated in this manuscript. Although the results are interesting, their impact and practical utility are inadequate for publication in ACS Central Science.

Reviewer: 4

Comments to the Author

The authors have addressed my concerns and modified the manuscript. I recommend that it be published and have no further changes.

Author's Response to Peer Review Comments:

(There are no comments.)

April 4, 2025

Dear Editor-in-Chief, Dr. **Carolyn R. Bertozzi**

Thanks very much for offering us an opportunity to revise our manuscript (oc-2025-002489.R1). We also thank the reviewers for their kind comments and recommendation. In particular, we thank the reviewers for pointing out several important issues for us to examine, which greatly benefits our research work and makes our work more convincing. Here are my responses to those comments by the reviewer **point-by-point** and the changes made to the manuscript (revised portions are marked with a yellow background in the paper).

Responses to editor's comments are detailed:

Formatting Needs:

1：Response to comment: Uniform structure sizing:

a) Ensure that all structures in Figures and Schemes have a visually consistent size, matching the size used in Figure 1.

b) Avoid variations in figure scaling that lead to different structure sizes (even if the Object Setting in ChemDraw is the same).

- As suggested, these corrections have been made.

2：Response to comment: Color adjustments for Scheme 1 and TOC

a) Avoid using bright red and blue colors and also light blue background colors.

b) Use pastel colors instead.

c) Limit the number of colors in Scheme 1 (and also in TOC) to 3-4 for clarity (currently, it looks overloaded). Please check out recently published papers as examples:

- As suggested, these corrections have been made.

3：Response to comment: Table 1 adjustments: Ensure that the size of all compounds in Table 1 is uniform.

- As suggested, we have made this correction.

4：Response to comment: Supporting Information: Please label the Table 1 in the following format: "Table S1…" instead of "Supplementary Table 1."

- As suggested, we have made this correction.

5：Response to comment: Author List: Please include the email address(es) of the corresponding author(s) on the first page of the manuscript.

- As suggested, the email address of the corresponding author has been added on the first page of the manuscript.

6：Response to comment: Supporting Information: A Supporting Information for Publication file must be submitted if the manuscript references Supporting Information. If multiple files are

mentioned in the manuscript, all must be present and of the correct file type. Review-only files should be designated as such, and there should be no duplicate files. All PDF Supporting Information files should have the title and author names and affiliations present on the first page, matching the manuscript. There should be no highlighting or tracked changes present anywhere in the Supporting Information for Publication file(s).

- As suggested, these mistakes have been corrected.

7： Response to comment: Supporting Information: Please number all pages in the following format: S1, S2, S3, etc.

- As suggested, we have made this correction.

8： Response to comment: Synopsis: The synopsis should be no more than 200 characters (including spaces).

- As suggested, a better synopsis has been provided.

Responses to reviewers' comments are detailed:

Reviewer(s)' Comments to Author:

9：Response to comment:

Reviewer: 3

Recommendation: Publish in ACS Central Science without change.

Comments:

I have reviewed the original submission of this manuscript. The authors carefully revised it according to comments and suggestions by the reviewers. Now, the revised version is suitable for publication in ACS Central Science. Additional revision and review process are not necessary.

Additional Questions:

Quality of experimental data, technical rigor: High

Significance to chemistry researchers in this and related fields: Top 5%

Broad interest to other researchers: High

Novelty: Top 5%

Is this research study suitable for media coverage or a First Reactions (a News & Views piece in the journal)?: Yes

- We thank the reviewer very much for the nice comments.

10：Response to comment:

Reviewer: 1

Recommendation: Publish in ACS Central Science without change.

Comments:

The authors have adequately addressed all comments from the reviewer. I recommend publishing the manuscript in its current form.

Additional Questions:

Quality of experimental data, technical rigor: Top 5%

Significance to chemistry researchers in this and related fields: Top 5%
Broad interest to other researchers: Top 5%
Novelty: Top 5%
Is this research study suitable for media coverage or a First Reactions (a News & Views piece in the journal)?: No

- We thank the reviewer very much for the nice comments.

11：Response to comment:
Reviewer: 2
Recommendation: Does not meet the requirements of publishing in ACS Central Science.
Comments:
This reviewer completely disagrees with the authors' comments. Since the authors acknowledge that they have not been able to synthesize natural products through the asymmetric Cu-catalyzed cyclization of indole-diynes, Figure 1 should be omitted to avoid misleading readers. Consequently, the synthetic utility of the method is not sufficiently demonstrated in this manuscript. Although the results are interesting, their impact and practical utility are inadequate for publication in ACS Central Science.
Additional Questions:
Quality of experimental data, technical rigor: High
Significance to chemistry researchers in this and related fields: High
Broad interest to other researchers: High
Novelty: High
Is this research study suitable for media coverage or a First Reactions (a News & Views piece in the journal)?: No

- **As highly recommended by other reviewers (reviewers 1, 3, and 4), this work's novelty is enough to publish in *ACS Central Science* without any changes.**

12：Response to comment:
Reviewer: 4
Recommendation: Publish in ACS Central Science without change.
Comments:
The authors have addressed my concerns and modified the manuscript. I recommend that it be published and have no further changes.
Additional Questions:
Quality of experimental data, technical rigor: High
Significance to chemistry researchers in this and related fields: High
Broad interest to other researchers: High
Novelty: High
Is this research study suitable for media coverage or a First Reactions (a News & Views piece in the journal)?: No

- We thank the reviewer very much for the nice comments.

WENZHOU UNIVERSITY

Wenzhou, Zhejiang, China

I hope my responses to the reviewers' comments as well as all the changes made to the manuscript are satisfactory to your office. My students have worked tirelessly on this chemistry and put so many late hours to finish the original manuscript and later to significantly improve the manuscript to satisfy the high level of *ACS Central Sci.* article. They truly deserve to have the chance.

Thank you very much.
Sincerely yours,

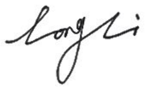

Professor Long Li
College of Chemistry and Materials Engineering, Wenzhou University
Telephone: 18790763089
Email: longwzu1990@wzu.edu.cn
